# Structural revisions of small molecules reported to cross-link G-quadruplex DNA *in vivo* reveal a repetitive assignment error in the literature

**DOI:** 10.1038/srep23499

**Published:** 2016-03-23

**Authors:** Paul E. Reyes-Gutiérrez, Tomáš Kapal, Blanka Klepetářová, David Šaman, Radek Pohl, Zbigniew Zawada, Erika Kužmová, Miroslav Hájek, Filip Teplý

**Affiliations:** 1 Institute of Organic Chemistry and Biochemistry, Academy of Sciences of the Czech Republic, v.v.i., Flemingovo n. 2, 166 10 Prague 6, Czech Republic

## Abstract

Two molecules of mistaken identity are addressed. Uncovering these assignment errors led us to formulate more general guidelines about additional misassignments in cases of published bis-imines derived from 1,2-phenylenediamine and hydroxybenzaldehydes having no substituent in *ortho*-positions. The main purpose of this article is to highlight this repetitive assignment error in the literature and thus increase the likelihood of correct assignments in future papers.

Yuan *et al.*^1^ recently published a paper entitled *Existence of G-quadruplex structures in promoter region of oncogenes confirmed by G-quadruplex DNA cross-linking strategy*. Their report is based on the synthesis and use of bis-imine **1** (Fig. 1). We found that this key compound is misassigned. From the body of evidence gathered in this letter it is apparent that in their study Yuan *et al.* used benzimidazole **1**_**revised**_ and not the isomeric bis-imine **1**. Furthermore, to prove the existence of G-quadruplex structures in promoter region of oncogenes *in vivo*, the authors reported a carboxy-substituted derivative of bis-imine **1** which they used in a pull-down study (see compound **5**, Fig. 2). We found that structural revision is also necessary in this case. As the targeting of G-quadruplex DNA holds considerable promise for anti-cancer therapy, the structural revisions presented herein are of importance for future research in this area^2^,^3^,^4^,^5^.

In addition, the assignment errors in the report by Yuan *et al.*^1^ led us to more general concerns about additional misassignments in cases of published bis-imines. This concerns bis-imines produced from 1,2-phenylenediamine and hydroxybenzaldehydes having no substituent in *ortho*-positions. As highlighted in this paper, any such published bis-imines lacking substantial NMR and/or X-ray crystal structure evidence should be taken with caution as they may actually be benzimidazoles and not bis-imines as documented for two further misassignments in the literature. As the chemistry of bis-imines with *ortho*-hydroxy group (salenes) and related compounds remains influential for development of molecular science (*e.g.* catalysis^6^,^7^,^8^,^9^, supramolecular^10^ and polymer science^11^,^12^, chemical biology^13^,^14^,^15^), the danger of propagation of errors in this and related fields is greater than in other branches of chemistry. It is therefore crucial to uncover examples of repetitive assignment errors and prevent their perpetuation, which is the main objective of this paper.

## Results

### Misassignment 1

**Figure 1:**
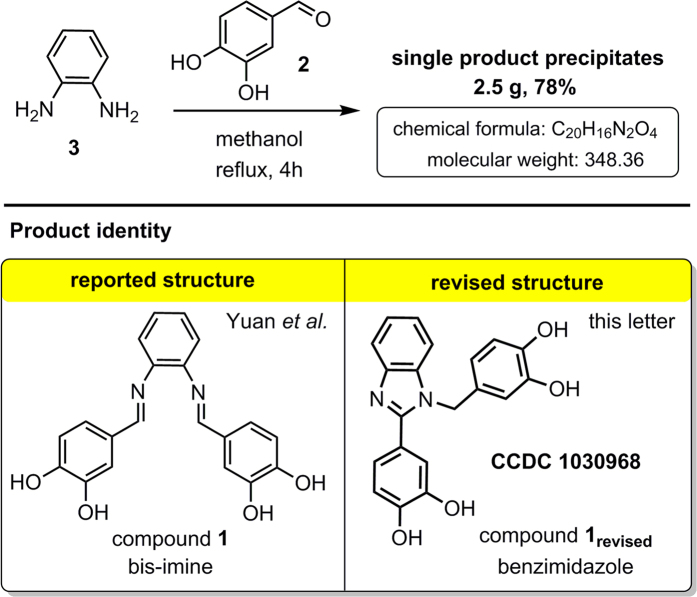
Reaction leading to compound 1_revised_ erroneously assigned as compound 1.

In their paper Yuan *et al.* described bis-imine **1** as a pro-drug that, after oxidative activation, significantly stabilizes G-quadruplex DNA structures *via* covalent cross-linking. However, our results show that the synthetic protocol described by the authors yields the benzimidazole product **1**_**revised**_ and not bis-imine **1**. Specifically, the reaction of 3,4-dihydroxybenzaldehyde (**2**) with 1,2-phenylenediamine (**3**) in methanol using the conditions reported by Yuan *et al.* consistently produced compound **1**_**revised**_ (Fig. 1). Furthermore, several variants of the reported conditions were tested with compound **1**_**revised**_ being the sole isolated product in each of these experiments (Supplementary Table S1).

Initial evidence pointing to an erroneous assignment of the reaction product as bis-imine structure **1** was based on NMR analysis. Structure **1** is expected to give a symmetric ^1^H NMR spectrum featuring 5 aromatic ^1^H signals accompanied by two OH signals and one imine −N = CH− signal. However, the ^1^H NMR spectrum of the reaction product turned out to be more complex, and did not show the symmetry anticipated for structure **1** (Fig. 3 and Supplementary Table S2). After detailed NMR analysis using H, C-HSQC, H, C-HMBC, and H, H-COSY experiments, the identity of the product was shown to be consistent with the non-symmetric benzimidazole structure **1**_**revised**_.

Additional evidence for structure **1**_**revised**_ having the benzimidazole moiety and two symmetrically non-equivalent dihydroxyphenyl units was obtained from single crystal X-ray structure analysis (Fig. 4). This evidence provided unambiguous support for structure **1**_**revised**_ which is the single product isolated from the uncatalyzed reaction of dihydroxybenzaldehyde **2** with phenylenediamine **3** in methanol.

### Misassignment 2

**Figure 2:**
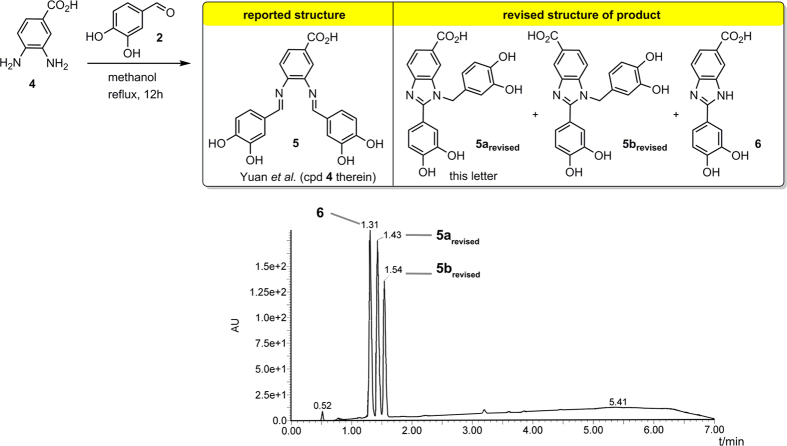
Revised structure of the product generated in the catalyst-free reaction of diamine **4** with aldehyde 2. UPLC analysis (bottom) shows three peaks corresponding to products **5a**_**revised**_, **5b**_**revised**_, and **6**. See the SI for synthetic and characterization details.

**Figure 3:**
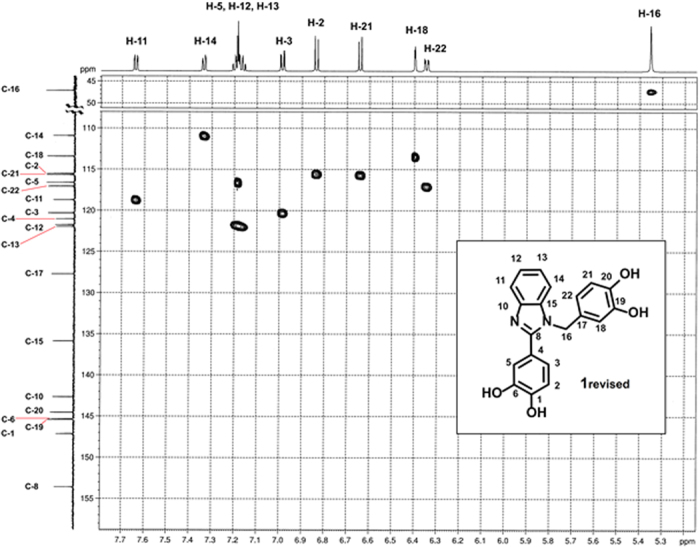
^1^H, ^13^C and H, C-HSQC NMR spectra consistent with non-symmetric structure 1_revised_.

**Figure 4:**
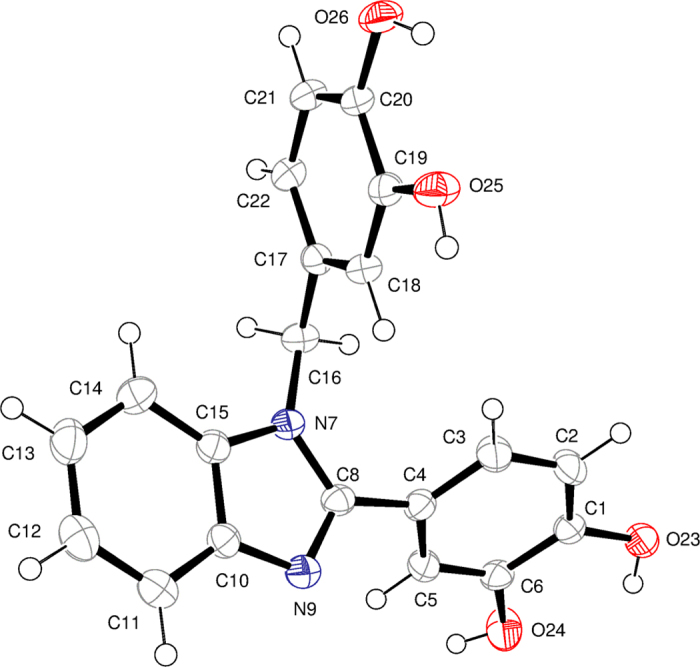
Single crystal X-ray structure of compound 1_revised_ (CCDC 1030968). An ORTEP view with displacement ellipsoids shown at 50% probability.

Yuan *et al.*^1^ also reported that carboxy-substituted diamine **4** reacts with aldehyde **2** to afford bis-imine **5** (Fig. 2). They described the use of bis-imine **5** in a pull-down study to prove the existence of G-quadruplex structures in promoter region of oncogenes *in vivo*. We found that structural revision is necessary in this case as well. As revealed by UPLC-MS (Fig. 2 bottom) followed by comprehensive NMR analysis, the reported reaction leads to three products, none of which is the bis-imine **5** reported by Yuan *et al.* Two isomeric carboxy-benzimidazoles **5a**_**revised**_ and **5b**_**revised**_ are formed besides species **6**, which is the third reaction product (see the SI for details).

### Bis-imines or benzimidazoles – experimental survey

**Figure 5:**
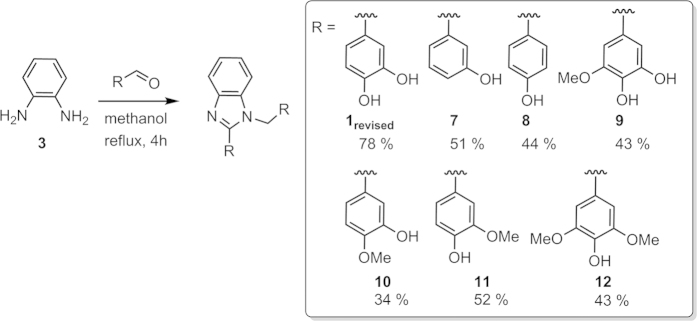
Catalyst-free synthesis of hydroxybenzimidazoles from the corresponding hydroxybenzaldehydes lacking substituents in the *ortho*-position and the isolated product yields after simple precipitation. See the SI for synthetic and characterization details.

**Figure 6:**
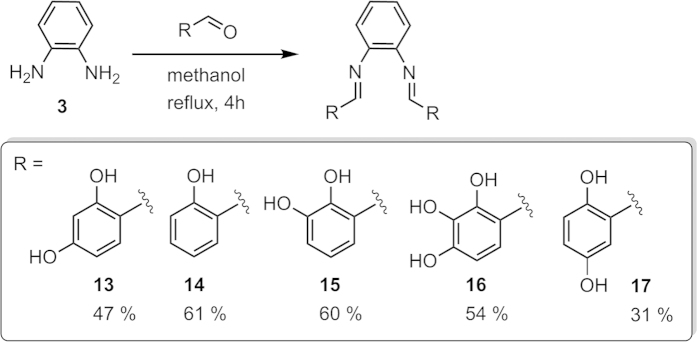
Synthesis of salenes 13–17 from the corresponding salicylaldehydes and the isolated product yields after simple precipitation. See the SI for synthetic and characterization details.

Our experimental survey provided evidence that catalyst-free reactions of phenylenediamine **3** with hydroxybenzaldehydes having no substitution in *ortho*-positions typically produce benzimidazole derivatives (*e.g.* **1**_**revised**_, **7**–**12**, Fig. 5, see the SI for synthetic and characterization details). This is in stark contrast to salicylaldehydes, *i.e.* arylaldehydes that contain an *ortho*-hydroxy group, which react with **3** to form bis-imines (salenes) such as **13**–**17** (Fig. 6).

Synthesis of benzimidazoles **1**_**revised**_, **7**, **8**, **10**, and **11** in the presence of various catalysts has been previously reported (ref. 16 and Supplementary references 3–6). Notably, even catalyst-free syntheses of benzimidazoles **7**, **8**, and **11** starting from phenylenediamine **3** and the corresponding hydroxybenzaldehydes have been described^17^,^18^,^19^,^20^,^21^,^22^,^23^,^24^,^25^,^26^. Salenes **13**–**17** have all been previously described (refs 27, 28, 29 and Supplementary references 8–11).

### Concerns about additional potential misassignments

**Figure 7:**
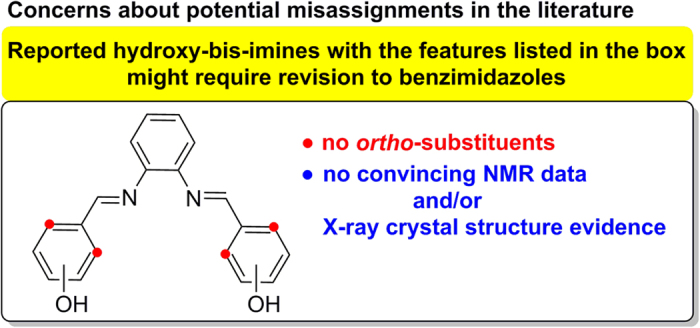
Concerns about potential misassignments of some hydroxy-bis-imines.

**Figure 8:**
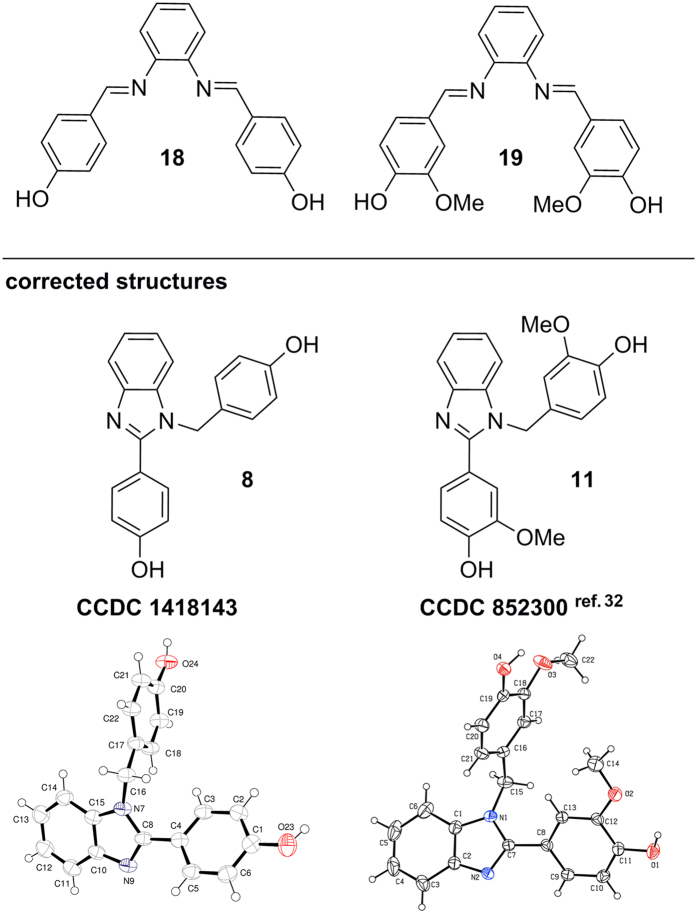
Further hydroxy-bis-imines 18 and 19 reported in the literature for which structural revision is necessary based on the evidence in this report. X-ray data of compound **11** were taken from refs 32 (Reproduced with permission of the International Union of Crystallography, http://journals.iucr.org/, permission was granted by John Wiley & Sons, Inc.).

In the context of the misassignments in the report by Yuan *et al.*^1^, we realized that additional published hydroxy-bis-imines in other reports might also be misassigned. This concerns hydroxy-bis-imines produced from 1,2-phenylenediamine and hydroxybenzaldehydes having no substitution in *ortho*-positions. As summarized in Fig. 7, such hydroxy-bis-imines published in the absence of convincing NMR and/or X-ray crystal structure evidence should be taken with caution as they may actually be benzimidazoles. This is documented in Fig. 8 giving two specific examples of salenes (**18**, **19**) reported in the literature^30^,^31^, for which structural revision is necessary as shown in this report. To this end, we reproduced synthetic protocols described in the original reports where the bis-imine products **18** and **19** were reported^30^,^31^. ^1^H and ^13^C NMR spectra gave a clear indication that the respective products isolated are benzimidazoles **8**, **11** and not the symmetric bis-imines **18** and **19**, respectively. Important diagnostic feature in the NMR spectra of the benzimidazoles **8** and **11** is their non-symmetry and the presence of a signature for a “N-**CH**_**2**_-“ fragment near δ 5.5 ppm in ^1^H NMR and near δ 47 ppm in ^13^C NMR (see S60 and S63). In addition, the identity of compound **8** has been confirmed by our data from X-ray crystallography (**8**: CCDC 1418143, see S42). The X-ray crystal structure of benzimidazole **11** (CCDC 852300) along with its synthesis has been reported previously in ref. 32 providing independent evidence in support of the benzimidazole product structure. Indeed, when we synthesized compound **11** according to the procedure described in ref. 32 (see S31) we found that this sample of benzimidazole **11** had identical ^1^H and ^13^C NMR spectra to the compound we prepared following the protocol in ref. 31 (see S30). This unambiguously proves that the compound synthesized according to ref. 31 is benzimidazole **11** and not bis-imine **19**.

**Figure 9:**
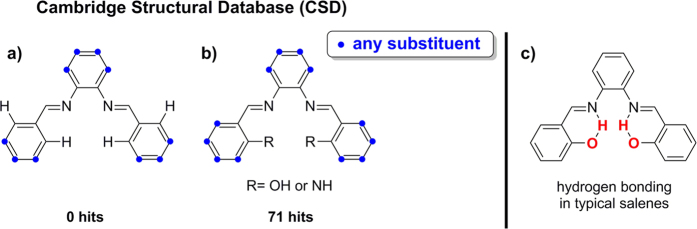
(**a**) No salenes derived from 1,2-phenylenediamine and benzaldehydes lacking substituents in the *ortho*-positions were found in the CSD. (**b**) 71 Salenes *having* −OH or −NH in the *ortho*-positions were found in the CSD. (**c**) Intramolecular hydrogen-bonding motif stabilizing the typical salen structure.

To support our concerns about other potential misassignments in the literature (Fig. 7) our searches of the Cambridge Structural Database (CSD) are summarized here. We found that the CSD does not contain any salenes derived from 1,2-phenylenediamine and benzaldehydes lacking substituents in the *ortho*-positions (Fig. 9a, see the Supplementary CSD search overview 1). By contrast, the CSD contains 71 salenes *having* −OH or −NH in the *ortho*-positions (Fig. 9b) and as many as 772 crystal structures of metal complexes derived from such salen ligands (see the Supplementary CSD search overviews 2 and 3, respectively). The CSD search results thus clearly show that salenes that are well documented in the literature typically possess an *ortho*−OH or *ortho*−NH group. The presence of such groups allows intramolecular hydrogen bonding (Fig. 9c) which appears to be required to stabilize the imine moiety and thus prevent cyclization to benzimidazoles.

However, it should be emphasized that the particular misassignment cases documented in this report as well as the results of our CSD searches cannot completely rule out the existence of a potentially isolable bis-imine derived from 1,2-phenylenediamine lacking hydroxy groups in the *ortho*-position.

### Error propagation

**Figure 10:**
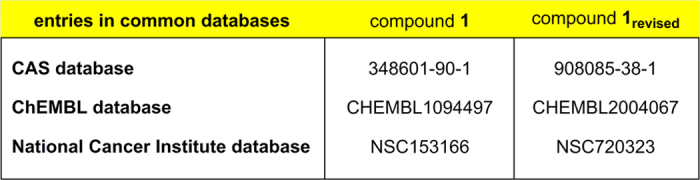
Compound registry numbers for benzimidazole 1_revised_ and misassigned bis-imine 1 in the CAS, ChEMBL, and NCI databases.

Misassigned structures^33^,^34^ occur in widely utilized chemistry databases such as CAS, ChEMBL, and the National Cancer Institute database (Fig. 10), which can contribute to error propagation. We believe that such error propagation can be common to all of the misassignment cases uncovered in this letter (Figs 1,2 and 8). Interestingly, focusing at bis-imine **1** our Sci-Finder search revealed six papers dealing with its synthesis and/or use^35^,^36^,^37^,^38^,^39^,^40^. Three further reports were found in which bis-imine **1** appeared as a hit after screening compounds from the National Cancer Institute database^41^,^42^,^43^. This widespread occurrence of the misassigned bis-imine **1** contrasts with the fact that the reactivity pattern leading to the related isomeric benzimidazoles (summarized in Fig. 5) is well documented in a series of papers^17^,^18^,^19^,^20^,^21^,^22^,^23^,^24^,^25^,^26^,^32^,^44^.

## Conclusions

In summary, from the body of evidence gathered in this paper it is apparent that in their study Yuan *et al.* used benzimidazole **1**_**revised**_ and not the isomeric bis-imine **1**. Furthermore, the reaction of carboxy-substituted diamine **4** and aldehyde **2** leads to a mixture of three products **5a**_**revised**_, **5b**_**revised**_, and **6** and not the single bis-imine product **5** reported by Yuan *et al.* The oxidizable 3,4-dihydroxyphenyl motif was reported to serve as a warhead for the crosslinking strategy described by Yuan *et al.* and it is therefore key for the biological conclusions of this paper. As this structural moiety is present also in the benzimidazoles **1**_**revised**_, **5a**_**revised**_, **5b**_**revised**_, and **6** we expect that the conclusions of Yuan *et al.* with respect to the biological properties of the compounds studied are correct. As the targeting of G-quadruplexes holds considerable promise for anti-cancer therapy, the structural revisions presented herein are significant for future research in this area.

A more general concern about further similar misassignments of published hydroxy-bis-imines is also formulated in this letter. Specifically, bis-imines **18** and **19** produced reportedly from 1,2-phenylenediamine and hydroxybenzaldehydes that lack an *ortho*-hydroxy group were found to be benzimidazoles **8** and **11**, respectively. As the chemistry of salenes and related compounds remains influential for development of molecular science (*e.g.* chemical biology, catalysis, polymer science), the potential for propagation of errors in this field is greater than in other areas of chemistry. To this end, the main goal of this article is to highlight this specific type of repetitive misassignment and increase the likelihood of correct assignments in future papers.

## Additional Information

**How to cite this article**: Reyes-Gutiérrez, P. E. *et al.* Structural revisions of small molecules reported to cross-link G-quadruplex DNA *in vivo* reveal a repetitive assignment error in the literature. *Sci. Rep.* **6**, 23499; doi: 10.1038/srep23499 (2016).

## Supplementary Material

Supplementary Information

Supplementary Information
