# Erratum: Structural revisions of small molecules reported to cross-link G-quadruplex DNA *in vivo* reveal a repetitive assignment error in the literature

**DOI:** 10.1038/srep24891

**Published:** 2016-04-29

**Authors:** Paul E. Reyes-Gutiérrez, Tomáš Kapal, Blanka Klepetářová, David Šaman, Radek Pohl, Zbigniew Zawada, Erika Kužmová, Miroslav Hájek, Filip Teplý

Scientific Reports 6: Article number: 23499; 10.1038/srep23499 published online: 03232016; updated: 04292016.

In the Supplementary Information file originally published with this Article, references 31 and 32 were incorrectly given as references 44 and 45 respectively. These errors have been corrected in the Supplementary Information that now accompanies the Article.

