# Corrigendum: Structural revisions of small molecules reported to cross-link G-quadruplex DNA *in vivo* reveal a repetitive assignment error in the literature

**DOI:** 10.1038/srep40471

**Published:** 2017-02-09

**Authors:** Paul E. Reyes-Gutiérrez, Tomáš Kapal, Blanka Klepetářová, David Šaman, Radek Pohl, Zbigniew Zawada, Erika Kužmová, Miroslav Hájek, Filip Teplý

Scientific Reports 6: Article number: 2349910.1038/srep23499; published online: 03 23 2016; updated: 02 09 2017

This article contains inaccuracies in its description of the work presented in reference 1.

In the Introduction,

“the authors reported a carboxy-substituted derivative of bis-imine **1** which they used in a pull-down study”.

should read:

“the authors reported a carboxy-substituted derivative of bis-imine **1** as a synthetic intermediate for their further pull-down study”.

In the ‘Misassignment 2’ section,

‘They described the use of bis-imine **5** in a pull-down study to prove the existence of G-quadruplex structures in promoter region of oncogenes *in vivo*’.

should read:

‘They described the use of bis-imine **5** as a synthetic intermediate for their further pull-down study to prove the existence of G-quadruplex structures in promoter region of oncogenes *in vivo*.’

In the Conclusions,

‘As this structural moiety is present also in the benzimidazoles 1_revised_ 5a_revised_, 5b_revised_, and 6 we expect that the conclusions of Yuan *et al*. with respect to the biological properties of the compounds studied are correct’.

Should read

‘As this structural moiety is present also in the benzimidazole 1_revised_ and biotinylated analogues of 5a_revised_, 5b_revised_, and 6, we expect that the conclusions of Yuan *et al*. with respect to the biological properties of the compounds studied are correct’.

