# Supplementary material for: Structural revisions of small molecules reported to cross-link G-quadruplex DNA in vivo reveal a repetitive assignment error in the literature
Source: Sci Rep. 2016 Mar 23;6:23499. doi: 10.1038/srep23499 (PMC4804300; doi:10.1038/srep23499)

Supplementary Information

Structural revisions of small molecules reported to cross-link G-quadruplex DNA *in vivo* reveal a repetitive assignment error in the literature

Paul E. Reyes-Gutiérrez, Tomáš Kapal, Blanka Klepetářová, David Šaman, Radek Pohl, Zbigniew Zawada, Erika Kužmová, Miroslav Hájek, Filip Teplý*

Table of contents

1) General information ………………………………………………. S3

2) Materials ………………………………………………. S4

3) Procedures and analytical data ………………………………………………. S5

Part A: Synthesis and characterization of $\mathbf{1}_{\mathbf{revised}}$ ……………………..…..…. S5

Part B: Synthesis and characterization of $\mathbf{5a}_{\mathbf{revised}}$, $\mathbf{5b}_{\mathbf{revised}}$, and **6** ....…........... S17

Part C: General procedure for the synthesis of 1,2-disubstituted benzimidazoles **7**, **8**, **9**, **10**, **11**, and **12** and their characterization ....…… S23

Part D: General procedure for the synthesis of salenes **13**, **14**, **15**, **16**, and **17** and their characterization ....… S33

4) X-ray analysis of $\mathbf{1}_{\mathbf{revised}}$, **7**, **8** and **14** ………………………………………….. S39

5) References ………………..…………….……… S44

6) NMR spectra scans ………………..…………….……… S50

1) General information

Melting points were determined on a Wagner & Munz PolyTherm A micro melting point apparatus and on a Stuart melting point SMP30 apparatus, and are uncorrected. Thin-layer chromatography (TLC) analysis was performed on silica gel plates (Silica gel 60 F_{254}-coated aluminium sheets, Merck, cat. no. 1.05554.0001) and visualized by UV (UV lamp 254/365 nm, Spectroline® Model ENF – 240C/FE) and/or chemical staining with $KMnO_4$ staining solution [$KMnO_4$ (1% aq.), Na_2CO_3 (2% aq.)]. Sonication was conducted with a BANDELIN SONOREX sonicator. NMR spectra were measured on a Bruker Avance 600 (600 MHz for ^{1}H, 151 MHz for ^{13}C) or Bruker Avance 400 (400 MHz for ^{1}H, 101 MHz for ^{13}C) NMR spectrometer. In ^{1}H and ^{13}C NMR spectra, chemical shifts are referenced in DMSO-d_6 δ_H = 2.50 ppm and δ_C = 39.50 ppm. Chemical shifts are given in δ-scale as parts per million (ppm); coupling constants (*J*) are given in Hertz. Where indicated, the signal assignments in the NMR spectra are unambiguous; the numbering scheme is arbitrary and is shown in the inserts. Where assigned, all ^{1}H and ^{13}C resonance assignments are based on analysis of H,H-COSY; H,C-HSQC; and H,C-HMBC spectra. IR spectra were recorded on a Bruker EQUINOX55 (IFS55) spectrometer in KBr pellets. Mass spectra were obtained at the Mass Spectrometry Facility operated by the Institute of Organic Chemistry and Biochemistry, Academy of Sciences of the Czech Republic, v.v.i. (IOCB ASCR). Electrospray ionization (ESI) mass spectra were recorded using a Thermo Scientific LCQ Fleet mass spectrometer equipped with an electrospray ion source and controlled by Xcalibur software. The sample was dissolved, diluted with MeOH : water (9 : 1) and injected using a 5 μL loop (flow rate of 200 μL.min^{-1}). Spray voltage, capillary voltage, tube lens voltage and capillary temperature were 5.5 kV, 5 V, 80 V and 275 °C, respectively. High-resolution mass spectra (HR MS) were obtained with the ESI instrument. Analytical UPLC separation was performed using an ACQUITY UPLC system (Waters Corp.), with ACQUITY UPLC® BEH C18 (1.7 μm, 2.1 x 50 mm) column, flow of 0.3 mL/min and gradient elution of 5-90% MeCN/H_2O. Preparative and analytical HPLC separations were both performed using an Agilent 1200 series HPLC equipped with a DAD monitoring absorbance at 301 nm. Preparative HPLC was performed with Vydac Protein & Peptide C18 218TP510 (10 μm, 250 x 10 mm) column. The mobile phase was MeCN in 0.05% aqueous TFA (3 mL/min). Analytical HPLC was run with a Poroshell 120 EC-C18 (2.7 μm, 3.0 x 50 mm) column. The eluent was MeCN in 0.05% aqueous TFA (1 mL/min). General information on X-ray measurements is given in Section 4.

2) Materials

EtOH, CH_3CN and EtOAc were purified via distillation under argon over CaH_2 and were used directly after distillation. MeOH was obtained from J.T.Baker (HPLC gradient grade). For anhydrous conditions, MeOH was distilled from Mg/I_2 as follows. MeOH (100 mL) was charged into a 1 L round-bottomed flask. Then, 5 g of Mg was added followed by addition of I_2 (500 mg). The mixture was heated to reflux under Ar atmosphere for 15 min. Then, more I_2 (500 mg) and MeOH (500 mL) were added and the mixture was refluxed under Ar atmosphere for 2h. Degassed MeOH was obtained via the freeze-pump-thaw method. The solvent was frozen under argon, and then thawed under vacuum. This process was repeated (3×). Finally the thawed solvent was purged with argon. DMSO-d_6 was dried over 4 Å molecular sieves. Unless otherwise stated, all other starting materials and reagents were obtained from commercial suppliers and used without further purification.

3) Procedures and analytical data

Part A: Synthesis and characterization of **1**$_{\mathbf{revised}}$

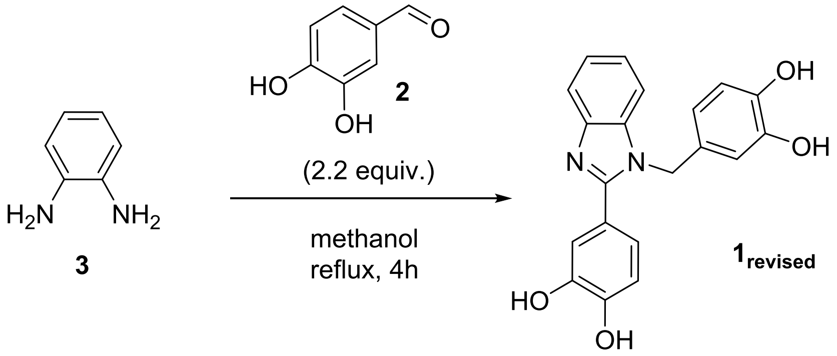

$C_{20}H_{16}N_2O_4$
[348.36]

A solution of 3,4-dihydroxybenzaldehyde (2.81 g, 20.34 mmol, 2.2 equiv.) in MeOH (10 mL) was added dropwise to a MeOH solution (10 mL) of 1,2-phenylenediamine (1.0 g, 9.25 mmol) in a two-neck round-bottom flask equipped with a water-cooled condenser. The resulting solution was refluxed 4 hours (bath temperature 70 ^{o}C). After complete consumption of the starting 1,2-phenylenediamine (by TLC, 1:1, EtOAc : hexane, R_f = 0.52), the reaction mixture was cooled down to RT. Then, the reaction mixture was transferred into a 100 mL round-bottom flask and MeOH was removed using rotary evaporator. Resulting oil was treated with MeOH (1-2 mL) and the mixture was sonicated to obtain suspension of a yellowish solid. The solids were filtered and washed with diethyl ether (3 x 25 mL) to give 2.5 g of an off-white powder (78% yield). Alternative reaction conditions that uniformly lead to **1**$_{\mathbf{revised}}$ as a single isolated product are shown in Table S1.

Characterization data for **1**$_{\mathbf{revised}}$ (4-(1-(3,4-dihydroxybenzyl)-1*H*-benzo[d]imidazol-2-yl)benzene-1,2-diol)

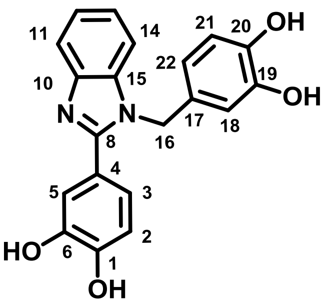

Mp 225-229 ^{o}C (236-238 ^{o}C ref. [1]). ^{1}H NMR (600 MHz, DMSO-d_6): 5.35 (s, 2H, H-16); 6.35 (ddt, J = 0.7, 2.2, 8.0 Hz, 1H, H-22); 6.40 (d, J = 2.2 Hz, 1H, H-18); 6.64 (d, J = 8.0 Hz, 1H, H-21); 6.84 (d, J = 8.2 Hz, 1H, H-2); 6.99 (dd, J = 2.3, 8.2 Hz, 1H, H-3); 7.15-7.18 (m, 1H, H-13); 7.18-7.21 (m, 1H, H-12); 7.19 (d, J =

2.3 Hz, 1H, H-5); 7.33-7.35 (m, 1H, H-14); 7.63-7.65 (m, 1H, H-11); 9.08 (br, 4H, OH). ^{13}C NMR (151 MHz, DMSO-d_6): δ = 47.17 (C-16); 110.92 (C-14); 113.43 (C-18); 115.54 (C-2); 115.68 (C-21), 116.60 (C-5); 117.07 (C-22); 118.72 (C-11); 120.32 (C-3); 121.05 (C-4); 121.75 (C-12); 121.97 (C-13); 127.73 (C-17); 135.89 (C-15); 142.67 (C-10); 144.57 (C-20); 145.38 (C-6); 145.43 (C-19); 147.16 (C-1); 153.63 (C-8). IR (KBr): ν (cm^{-1}) 3381; 1606; 1530; 1483; 1468; 1451; 1424; 1333; 1283; 1248; 1225; 1200; 1126; 1116; 878; 810; 764; 759; 736. MS (EI) m/z (%): 227 (35), 226 (100), 169 (70), 122 (28). MS(ESI) m/z (%): 349 (100), 227 (37). HRMS (ESI) m/z: ($C_{20}H_{17}N_2O_4^+$) calc.: 349.11828, found: 349.11839. Elem. Anal. Calcd. for $C_{20}H_{16}N_2O_4$: C, 68.96; H, 4.63; N, 8.04. Found C, 68.50; H, 4.55; N, 8.24. ^{1}H NMR, ^{13}C NMR, H,C-HSQC, and H,C-HMBC spectra are shown in Figures S1-S3. ^{1}H NMR and ^{13}C NMR data for compound **1**$_{\mathbf{revised}}$ obtained in this work have been compared with the data reported by Yuan *et al.*[2] for compound **1** (see Table S2). For X-ray crystal structure data of **1**$_{\mathbf{revised}}$, see Section 4 (Figure S11).

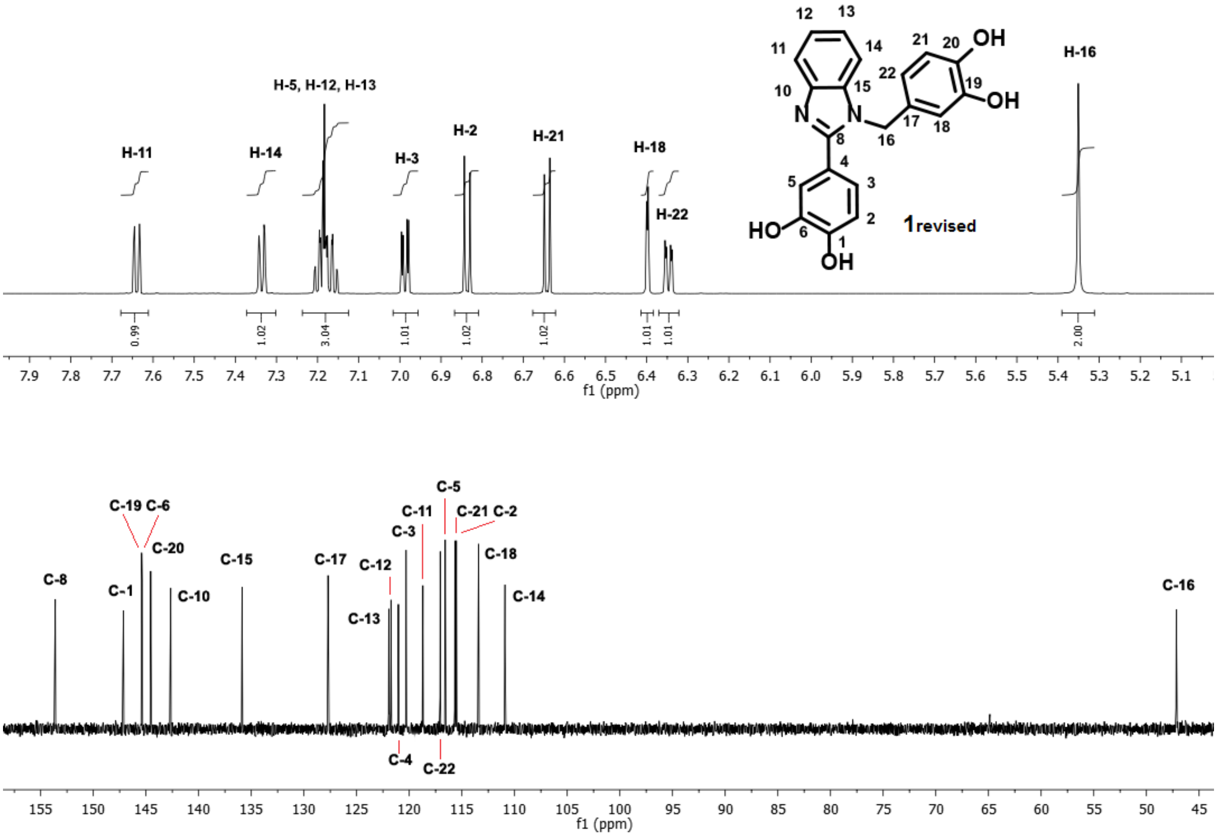

Figure S1. ^{1}H NMR (600 MHz) and ^{13}C NMR (151 MHz) spectra of isolated **1**$_{\mathbf{revised}}$ in DMSO-d_6.

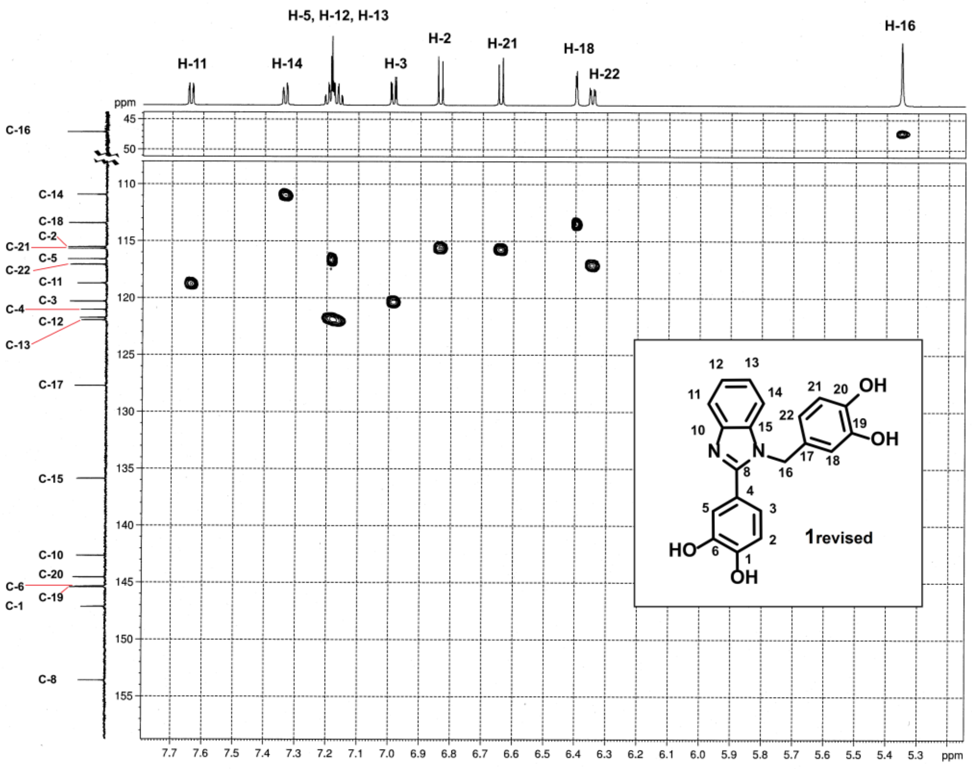

Figure S2. H,C-HSQC spectrum of isolated $\mathbf{1}_{\mathbf{revised}}$ (DMSO-d_6).

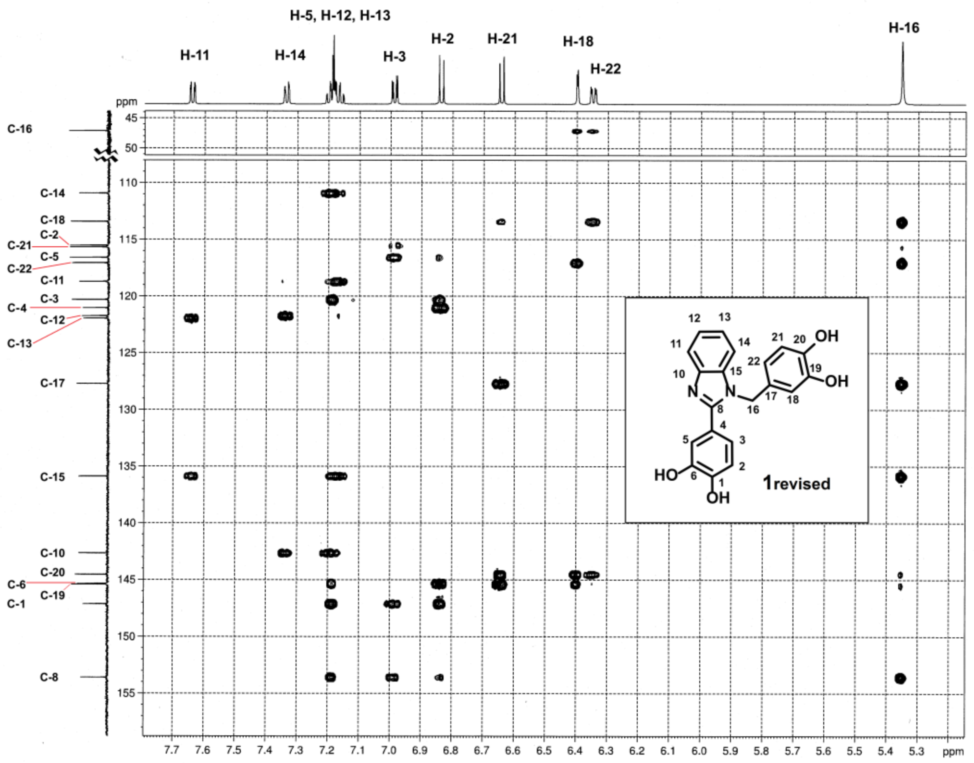

Figure S3. H,C-HMBC spectrum of isolated $\mathbf{1}_{\mathbf{revised}}$ (DMSO-d_6).

Table S1. Condition screen for reaction of **3** and **2** leading to **1**$_{\textbf{revised}}$.

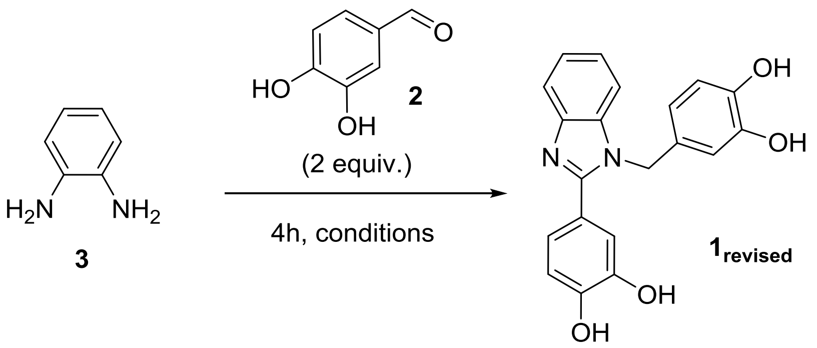

Entry	Conditions	Yield of 1$_{revised}$ (%)[6]
1	MeOH, air, reflux[1]	54
2	MeOH, air, reflux[2]	78
3	MeOH, air, RT[1]	51
4	MeOH, argon, -10 ^{o}C[1,3]	65
5	MeOH, argon, reflux[1,3]	50
6	EtOH, argon, reflux[4]	43
7	CH_3CN, argon, reflux[4]	44
8	AcOEt, argon, reflux[4]	39
9	water, argon, reflux[5]	74

[1] Aldehyde **2** (0.25 g, 1.84 mmol, 2 equiv.) in MeOH (2.5 mL) was added dropwise to a solution of **3** (0.10 g, 0.92 mmol) in MeOH (2.5 mL). [2] Reaction was scaled up; **3 (**1.0 g, 9.2 mmol). [3] Dry and degassed MeOH was used. [4] Solution of **2** (0.25 g, 1.84 mmol, 2 equiv.) in dry solvent (2.5 mL) was added dropwise to a solution of **3** (0.10 g, 0.92 mmol) in dry solvent (2.5 mL). [5]Water (5 mL) was added to a mixture of **2** (0.25 g, 1.84 mmol, 2 equiv.) and **3** (0.10 g). [6]Isolated product yields after simple filtration.

Table S2. ^{1}H NMR and ^{13}C NMR data for compound $\mathbf{1}_{\mathbf{revised}}$ obtained in this work compared with the data reported by Yuan *et al.*[2] for compound **1**.

**^{1}H NMR$_{\mathbf{reported}}$ (ppm)[a]**	**^{1}H NMR$_{\mathbf{this\ work}}$ (ppm)[b]**
**1**	$\mathbf{1}_{\mathbf{revised}}$
9.37 (br,2H)	9.08 (br,4H,OH)
8.90 (br,2H)	
7.65 (m,1H)	7.63-7.65 (m,1H,H-11)
7.33 (m,1H)	7.33-7.35 (m,1H,H-14)
7.18 (m,3H)	7.19 (d,1H,H-5)<br>7.18-7.21 (m,1H,H-12)<br>7.15-7.18 (m,1H,H-13)
6.99 (dd,1H)	6.99 (dd,1H,H-3)
6.85 (d,1H)	6.84 (d,1H,H-2)
6.65 (d,1H)	6.64 (d,1H,H-21)
6.39 (s,1H)	6.40 (d,1H,H-18)
6.36 (d,1H)	6.35 (ddt,1H,H-22)
5.35 (s,2H)	5.35 (s,2H,H-16)

**^{13}C NMR$_{\mathbf{reported}}$ (ppm)[a]**	**^{13}C NMR$_{\mathbf{this\ work}}$ (ppm)[b]**
**1**	$\mathbf{1}_{\mathbf{revised}}$
153.71	153.63 (C-8)
147.24	147.16 (C-1)
145.51	145.43 (C-19)
145.47	145.38 (C-6)
144.65	144.57 (C-20)
142.72	142.67 (C-10)
135.96	135.89 (C-15)
127.81	127.73 (C-17)
122.09	121.97 (C-13)
121.89	121.75 (C-12)
121.11	121.05 (C-4)
120.43	120.32 (C-3)
118.81	118.72 (C-11)
117.18	117.07 (C-22)
116.69	116.60 (C-5)
115.77	115.68 (C-21)
115.65	115.54 (C-2)
113.50	113.43 (C-18)
111.04	110.92 (C-14)
--------	47.17 (C-16)

[a] ^{1}H NMR (300 MHz, DMSO-d_6). ^{13}C NMR (75 MHz, DMSO-d_6). [b] ^{1}H NMR (600 MHz, DMSO-d_6). ^{13}C NMR (151 MHz, DMSO-d_6).

Reaction monitoring by ^{1}H NMR: Reaction of aldehyde 2 with phenylenediamine 3

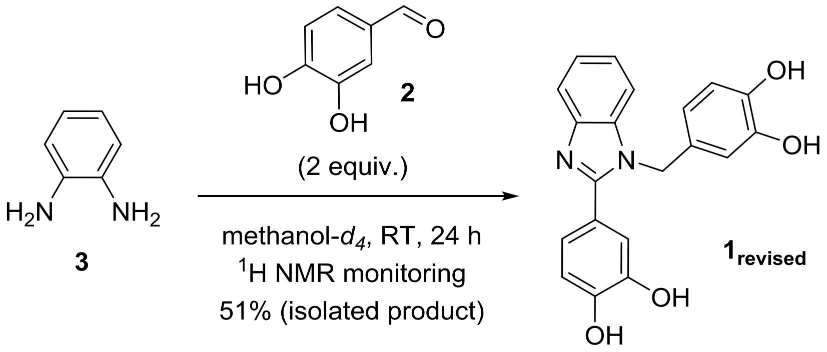

An NMR cuvette was loaded with 3,4-dihydroxybenzaldehyde (**2**, 0.025 g, 0.185 mmol, 2 equiv.). The aldehyde **2** was dissolved in methanol-d_4 (0.4 mL). Then, a solution of 1,2-phenylenediamine (**3**, 0.010 g, 0.092 mmol) in methanol-d_4 (0.1 mL) was added to the solution of aldehyde **2**. ^{1}H NMR (400 MHz) was run immediately after the solutions were mixed (mixing point, Figure S4). After this initial measurement, ^{1}H NMR was measured at several timepoints (up to 24 hours), as it is shown in Figure S4. Reaction mixture was stirred manually during short intervals of time and by attaching the NMR cuvette to a rotary evaporator during long intervals of time. After the last ^{1}H NMR measurement, the reaction mixture was transferred into a 10 mL vial and MeOH was removed using rotary evaporator. Resulting oil was treated with MeOH (0.5 mL) and the mixture was sonicated to obtain suspension of a yellowish solid. The solids were filtered and washed with diethyl ether (3 x 10 mL). This procedure led to isolation of compound $\mathbf{1_{revised}}$ as an off-white powder (0.016 g, 0.047 mmol, 51% yield).

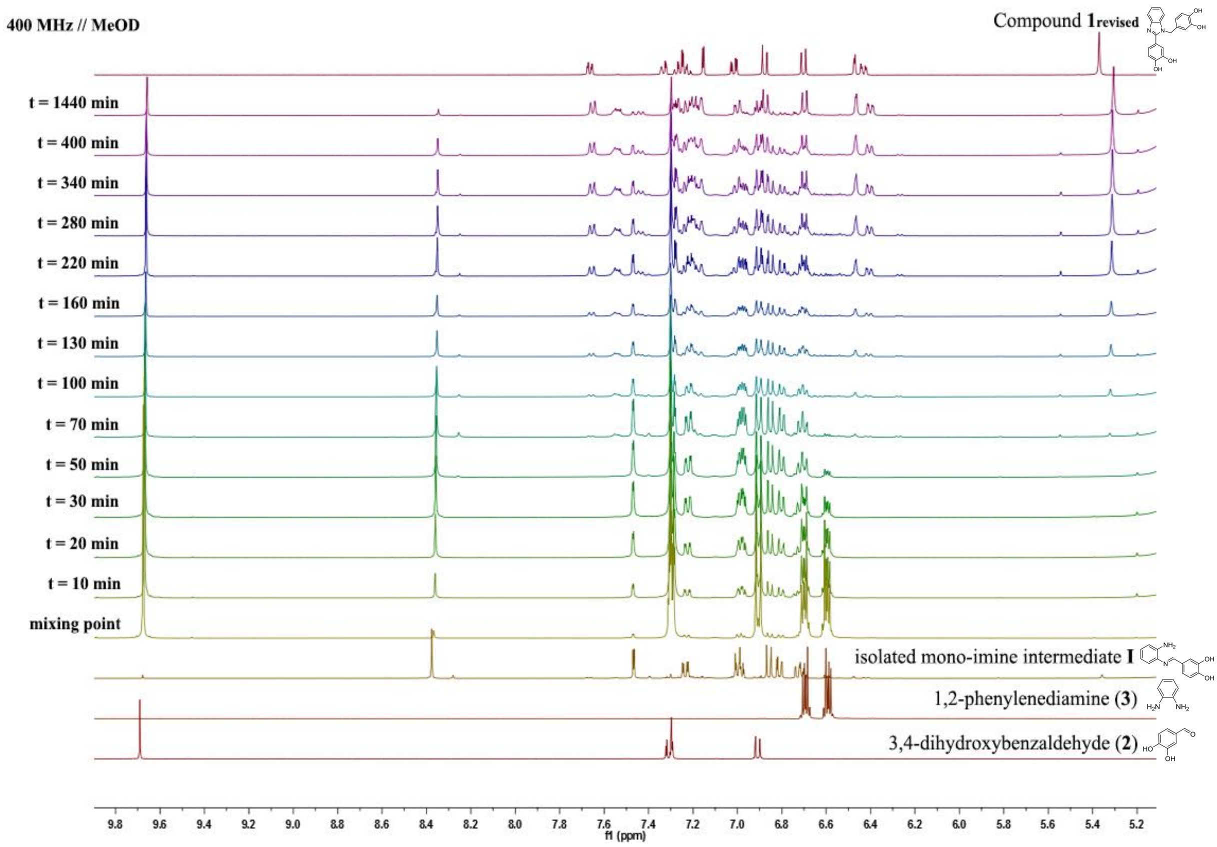

Figure S4. ^{1}H NMR (methanol-d_4, 400 MHz) monitoring of reaction of aldehyde **2** with phenylenediamine **3**.

Isolation and characterization of mono-imine intermediate I

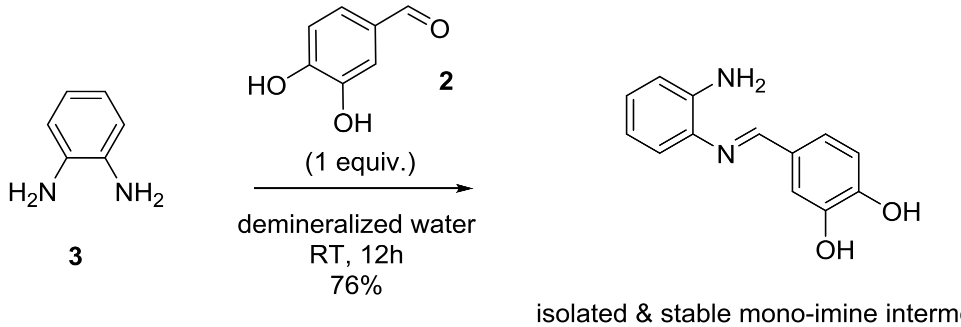

isolated & stable mono-imine intermediate **I**

$C_{13}H_{12}N_2O_2$
[228.25]

Demineralized water (10 mL) was added to a mixture of 3,4-dihydroxybenzaldehyde (0.127 g, 0.92 mmol, 1 equiv.) and 1,2-phenylenediamine (0.10 g, 0.92 mmol). Reaction mixture was stirred for 12 hours at RT. Then, the crude solid was collected by filtration and washed with demineralized water (50 mL) to obtain mono-imine intermediate **I** as a yellowish solid (0.16 g, 0.70 mmol, 76% yield).

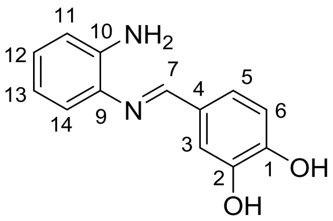

Mp 124-126 ^{o}C. ^{1}H NMR (600 MHz, DMSO-*d$_6$*): 5.01 (bs, 2H, NH_2), 6.54 (ddd, *J* = 1.4, 7.2, 7.9 Hz, 1H, H-13), 6.69 (dd, *J* = 1.3, 7.9 Hz, 1H, H-11); 6.83 (d, *J* = 8.1 Hz, 1H, H-6); 6.92 (ddd, *J* = 1.4, 7.2, 7.9 Hz, 1H, H-12); 7.01 (dd, *J* = 1.3, 7.9 Hz, 1H, H-14); 7.22 (dd, *J* = 1.9, 8.2 Hz, 1H, H-5); 7.42 (d, *J* = 1.9 Hz, 1H, H-3); 8.40 (s, 1H, H-7). ^{13}C NMR (101 MHz, DMSO-*d$_6$*): δ = 114.36 (C-3); 114.36 (C-11); 116.30 (C-6); 116.88 (C-13); 117.05 (C-14); 122.04 (C-5); 126.62 (C-12); 128.49 (C-4); 136.18 (C-9); 143.25 (C10); 145.56 (C-2); 148.85 (C-1); 156.68 (C-7). IR (KBr): ν (cm^{-1}) 3370, 3303, 3071, 3028, 2864, 1627, 1604, 1593, 1585, 1513, 1493, 1459, 1143, 1407, 1369, 1327, 1294, 1249, 1197, 1159, 1119, 1039, 1011, 975, 920, 876, 852, 817, 787, 759, 744, 680, 611, 599, 483. MS (EI) m/z (%): 227 (8), 108 (100), 80 (75). MS (ESI) m/z (%): 229.1 (100). HRMS (ESI) m/z: ($C_{13}H_{13}N_2O_2^{+}$) calc.: 229.09715, found: 229.09679.

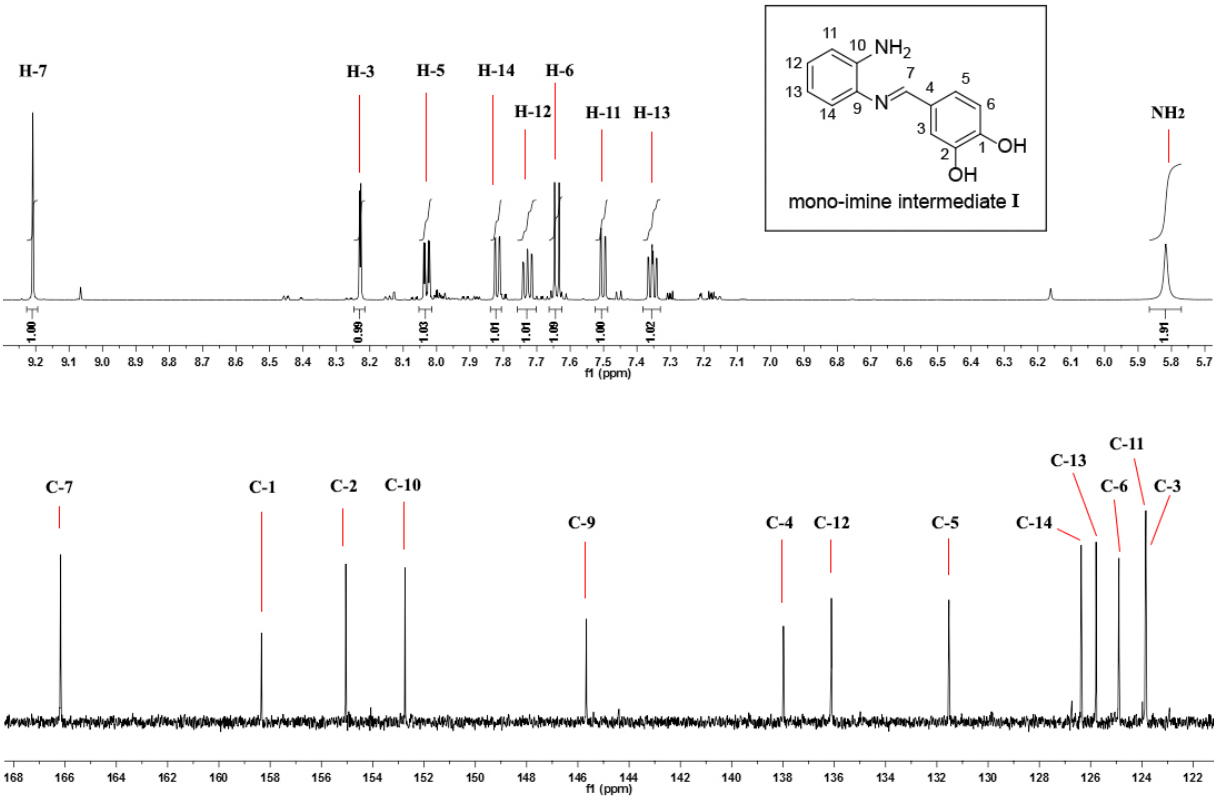

Figure S5. ^{1}H NMR (400 MHz) and ^{13}C NMR (100 MHz) spectra of isolated mono-imine intermediate **I** in DMSO-d_6.

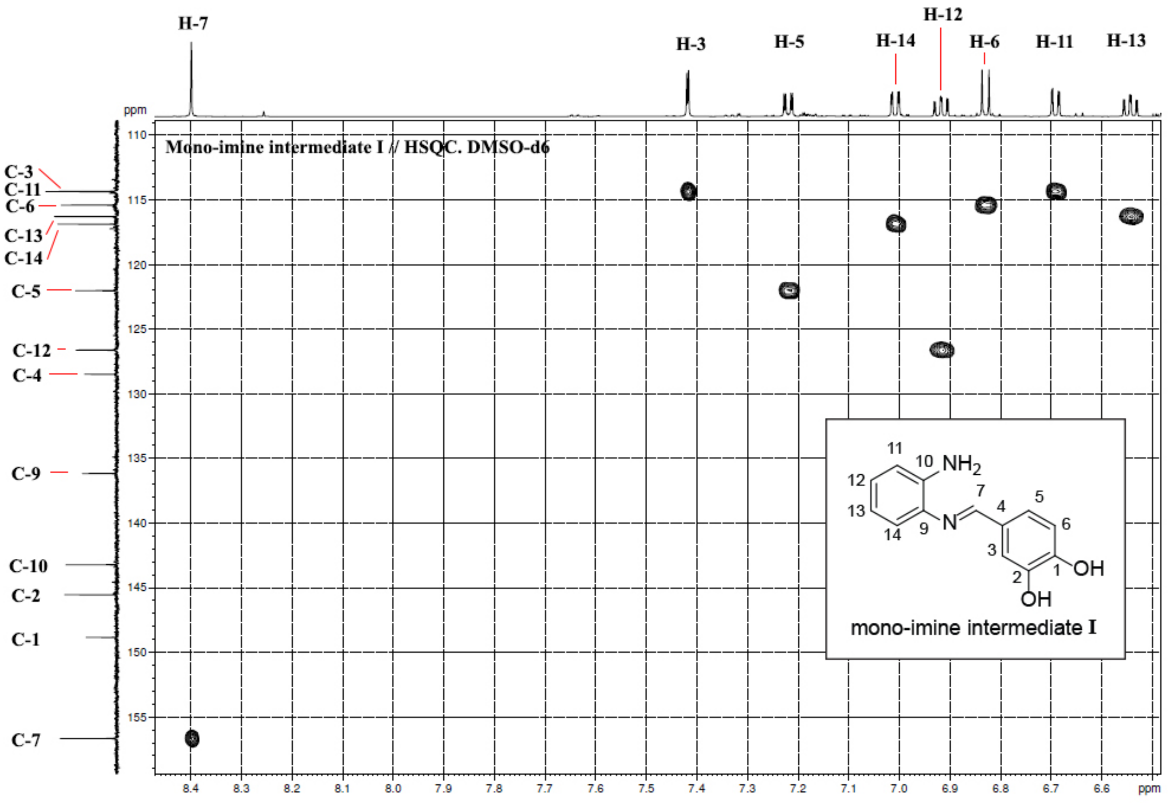

Figure S6. H,C-HSQC spectrum of isolated mono-imine intermediate **I** (DMSO-d_6).

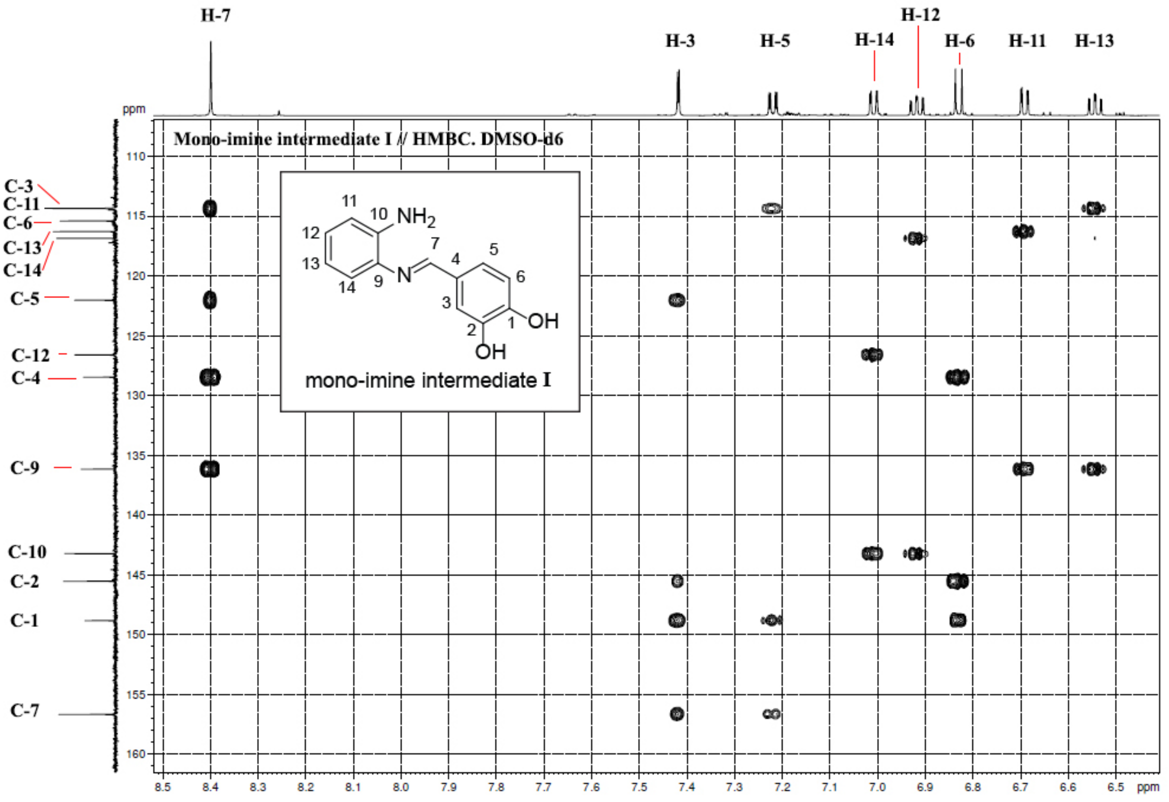

Figure S7. H,C-HMBC spectrum of isolated mono-imine intermediate **I** (DMSO-d_6).

Reaction monitoring by ^{1}H NMR: Reaction of mono-imine intermediate I with aldehyde 2

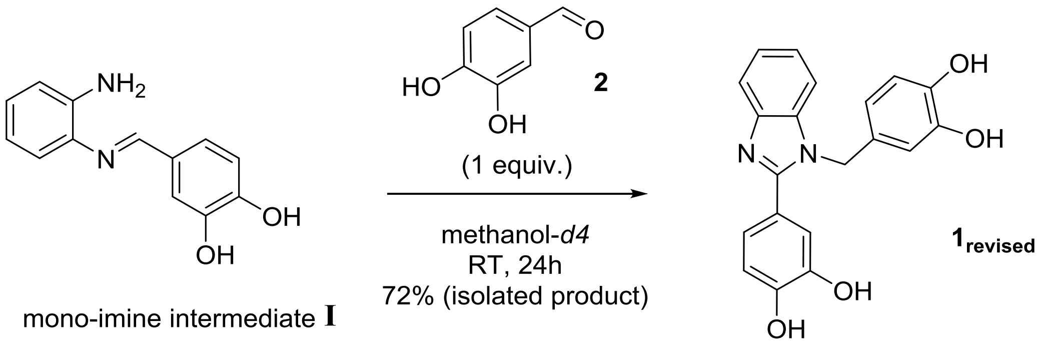

An NMR cuvette was loaded with mono-imine intermediate **I** (0.021 g, 0.092 mmol) and then methanol-d_4 was added (0.4 mL). A solution of 3,4-dihydroxybenzaldehyde (**2**, 0.013 g, 0.092 mmol, 1 equiv.) in methanol-d_4 (0.1 mL) was added to the solution of mono-imine intermediate **I**. ^{1}H NMR (400 MHz) was run immediately after the solutions were mixed (mixing point, Figure S8). After this initial measurement, ^{1}H NMR was measured at several timepoints (up to 24 hours), as it is shown in Figure S8. Reaction mixture was stirred manually during short intervals of time and by attaching the NMR cuvette to a rotary evaporator during long intervals of time. After the last ^{1}H NMR measurement, the reaction mixture was transferred into a 10 mL vial and MeOH was removed using rotary evaporator. Resulting oil was treated with MeOH (0.5 mL) and the mixture was sonicated to obtain suspension of a yellowish solid. The solids were filtered and washed with diethyl ether (3 x 10 mL). This procedure led to isolation of compound $\mathbf{1_{revised}}$ as an off-white powder (0.023 g, 0.066 mmol, 72% yield).

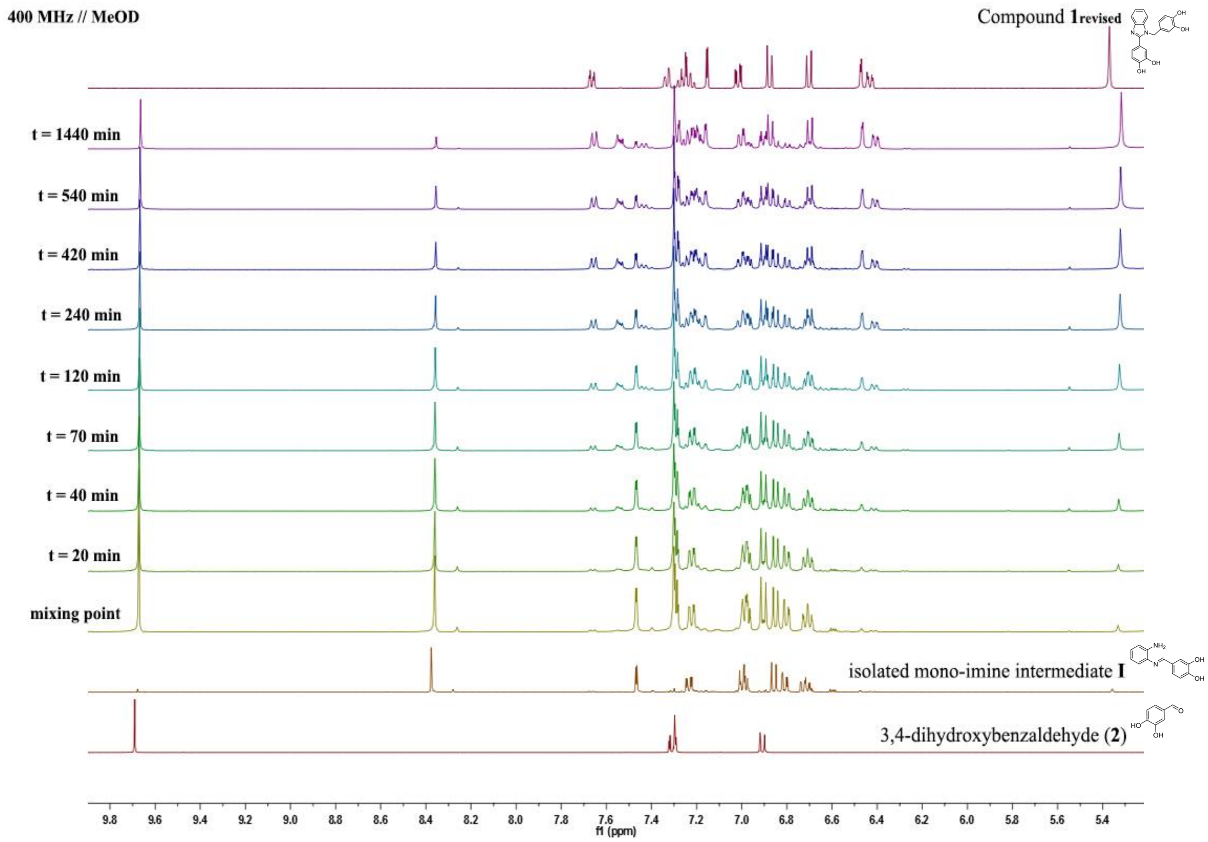

Figure S8. ^{1}H NMR (methanol-d_4, 400 MHz) monitoring of reaction of isolated mono-imine intermediate **I** with aldehyde **2**.

Part B: Synthesis and characterization $\mathbf{5a_{revised}}$, $\mathbf{5b_{revised}}$, and 6

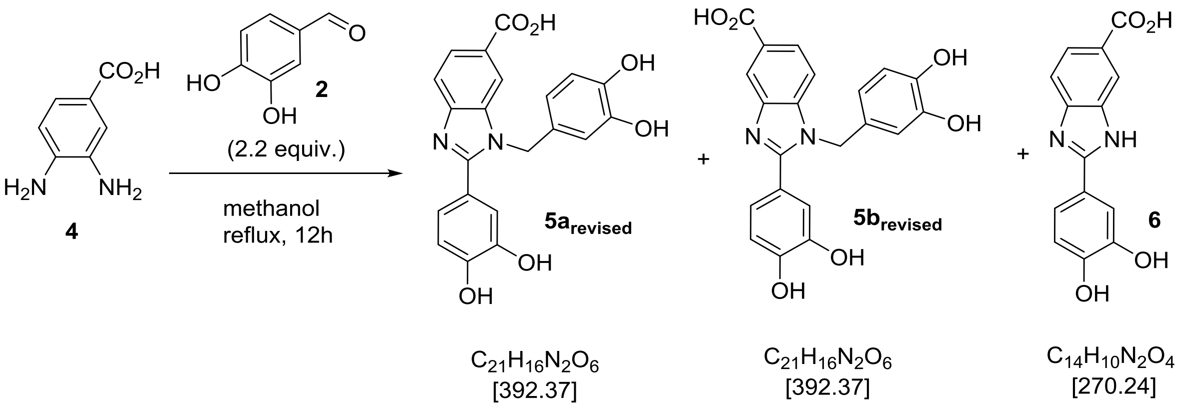

A solution 3,4-dihydroxybenzaldehyde (0.2 g, 1.45 mmol, 2.2 equiv.) in MeOH (2.5 mL) was added dropwise to a solution of 3,4-diaminobenzoic acid (0.1 g, 0.657 mmol) in MeOH (2.5 mL) in a two-neck round-bottom flask equipped with a water-cooled condenser. The resulting solution was refluxed 12 hours (bath temperature 70 ^{o}C). After complete consumption of the starting 3,4-diaminobenzoic acid (by TLC, 1:1, EtOAc : hexane, R_f = 0.43), the reaction mixture was cooled down to RT. Then, the reaction mixture was transferred into a 100 mL round-bottom flask and MeOH was removed using rotary evaporator. Resulting oil was treated with MeOH (1-2 mL), followed by addition of 50 mL of diethyl ether. The resulting mixture was then sonicated to obtain suspension of beige solid. The resulting solids were filtered and washed with diethyl ether (10 mL) to give 156 mg of beige solid. This solid was found to be a mixture of three compounds by UPLC-MS and NMR analysis (ratio determined by ^{1}H NMR: 1.0 : 0.7 : 1.0). The mixture consisted of two 1,2-disubstituted benzimidazole isomers $\mathbf{5a_{revised}}$ and $\mathbf{5b_{revised}}$ accompanied by 2-substituted benzimidazole **6**. Results from UPLC-MS analysis are shown in Figure S9 and S10.

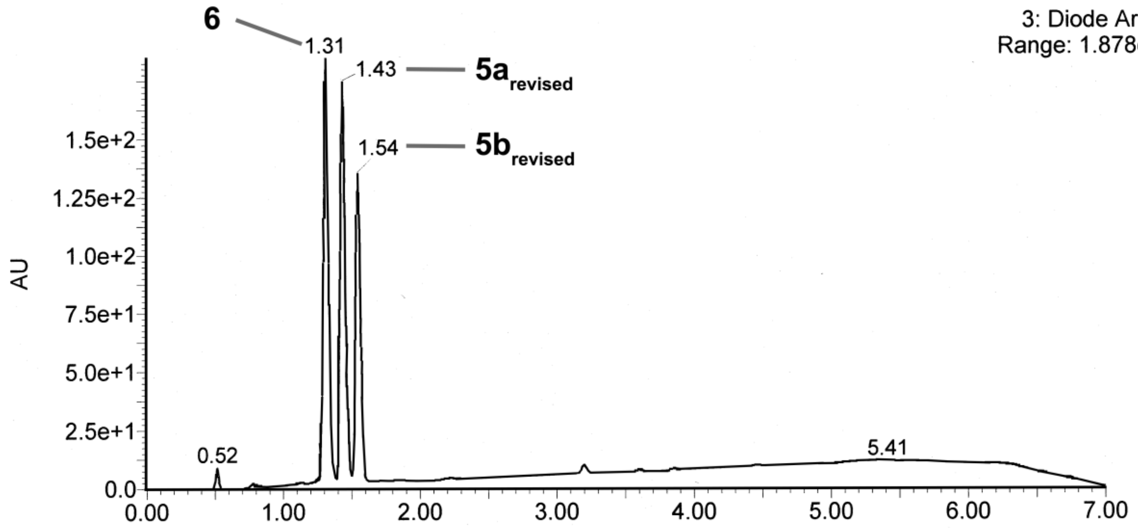

Figure S9. Result of UPLC analysis of product mixture obtained by reaction of **2** and **4**, 1 mg of sample was dissolved in 1 mL of H_2O:MeOH:*i*-PrOH:MeCN (1:1:1:1) solution containing 0.1% of HCO_2H. Sample was measured in an ACQUITY UPLC system (Waters Corp.), with a flow of 0.3 mL/min and gradient elution of 5-90% MeCN/H_2O reverse phase. ACQUITY UPLC® BEH C18 1.7 µm, 2.1 x 50 mm column.

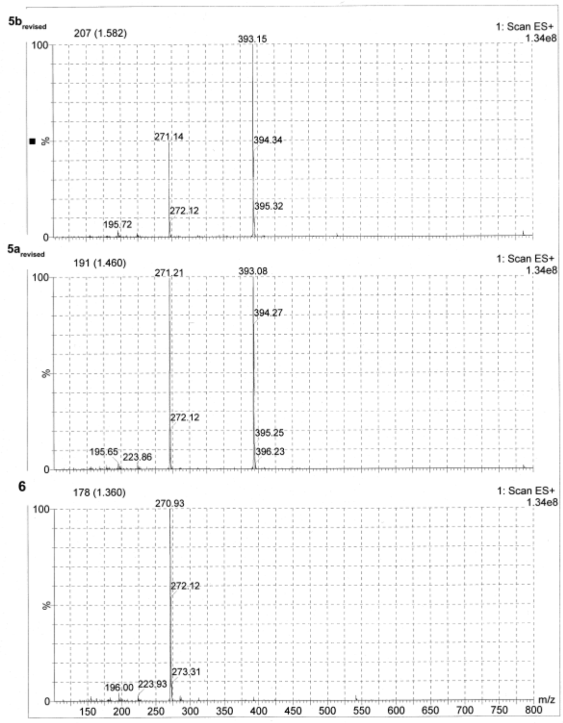

Figure S10. MS ESI pos. data corresponding to the three UPLC peaks (Figure S4). The data are consistent with structures $\mathbf{5a}_{\mathbf{revised}}$, $\mathbf{5b}_{\mathbf{revised}}$, and **6**.

Preparative HPLC separation of **5a**$_{\mathbf{revised}}$, **5b**$_{\mathbf{revised}}$, and **6** was performed using a Vydac Protein & Peptide C18 218TP510 (10 µm, 250 x 10 mm) column. The eluent was MeCN in 0.05% aqueous TFA. The flow rate was 3 mL/min at 25 ^{o}C and the gradient elution was as follows:

**Time (min)**	**MeCN (%)**
0	9
20	20

Purity of the collected samples was analyzed by analytical HPLC with DAD monitoring absorbance (301 nm) and a Poroshell 120 EC-C18 (2.7 µm, 3.0 x 50 mm) column. The eluent was MeCN in 0.05% aqueous TFA. The flow rate was 1 mL/min at 25 ^{o}C and the gradient elution was as follows:

**Time (min)**	**MeCN (%)**
0	13
3	25

The retention times for **5a**$_{\mathbf{revised}}$, **5b**$_{\mathbf{revised}}$, and **6** are shown in Table S3.

Table S3.

	**Time (min)**	**Compound[1]**
$t_{(1)}$	1.19	**6**
$t_{(2)}$	1.40	**5a**$_{\mathbf{revised}}$
$t_{(3)}$	1.68	**5b**$_{\mathbf{revised}}$

[1] Chemical structure was determined by ^{1}H NMR, ^{13}C NMR, H,C-HSQC and H,C-HMBC.

Characterization data for **5a**$_{\mathbf{revised}}$ (1-(3,4-dihydroxybenzyl)-2-(3,4-dihydroxyphenyl)-1*H*-benzo[d]imidazole-6-carboxylic acid)

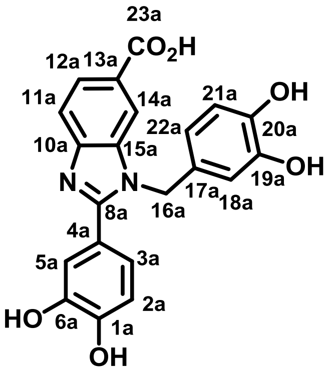

^{1}H NMR (600 MHz, DMSO-d_6): 5.51 (s, 2H, H-16a); 6.39 (dd, *J* = 2.2 Hz, 8.1, 1H, H-22a); 6.47 (d, *J* = 2.2 Hz, 1H, H-18a); 6.67 (d, *J* = 8.1 Hz, 1H, H-21a); 6.92 (d, *J* = 8.2 Hz, 1H, H-2a); 7.10 (dd, *J* = 2.2, 8.2 Hz, 1H, H-3a); 7.25 (d, *J* = 2.2 Hz, 1H, H-5a); 7.78 (d, *J* = 8.5 Hz, 1H, H-11a); 7.94 (dd, *J* = 1.5, 8.5 Hz, 1H, H-12a); 8.06 (d, *J* = 1.5 Hz, 1H, H-14a), 8.97 (bs, 2H, OH); 9.49 (bs, 1H, OH); 9.82 (bs, 1H, OH), 12.96 (bs, 1H, OH). ^{13}C NMR (151 MHz, DMSO-d_6): 47.95 (C-16a); 113.66 (C-18a); 113.80 (C-14a); 116.02 (C-21a); 116.08 (C-2a); 116.83 (C-5a); 117.06 (C-11a); 117.29 (C-22a); 117.70 (C-4a); 121.30 (C-3a); 124.87 (C-12a); 125.82 (C-13a); 126.74 (C-17a); 134.71 (C-10a, C-15a); 145.09 (C-20a); 145.78 (C-19a); 145.90 (C-6a); 148.91 (C-1a); 155.28 (C-8a); 167.39 (C-23a). MS (ES+) m/z (%): 393 (100), 271 (100). HRMS (ESI) m/z: ($C_{21}H_{16}N_2O_6^+$) calc.: 393.10811, found: 393.10818.

Characterization data for **5b**$_{\mathbf{revised}}$ (1-(3,4-dihydroxybenzyl)-2-(3,4-dihydroxyphenyl)-1*H*-benzo[d]imidazole-5-carboxylic acid)

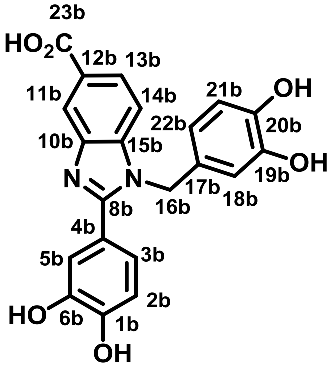

^{1}H NMR (600 MHz, DMSO-d_6): 5.45 (s, 2H, H-16b); 6.37 (dd, *J* = 2.2, 8.1 Hz, 1H, H-22b); 6.43 (d, *J* = 2.2 Hz, 1H, H-18b); 6.65 (d, *J* = 8.1 Hz, 1H, H-21b); 6.90 (d, *J* = 8.2 Hz, 1H, H-2b); 7.07 (dd, *J* = 2.2, 8.2 Hz, 1H, H-3b); 7.23 (d, *J* = 2.2 Hz, 1H, H-5b); 7.55 (d, *J* = 8.5 Hz, 1H, H-14b); 7.89 (dd, *J* = 1.5, 8.5 Hz, 1H, H-13b); 8.24 (d, *J* = 1.5 Hz, 1H, H-11b); 8.93 (bs, 2H, OH); 9.44 (bs, 1H, OH); 9.74 (bs, 1H, OH); 12.90 (bs, 1H, OH). ^{13}C NMR (151 MHz, DMSO-d_6): 47.93 (C-16b); 111.79 (C-14b); 113.75 (C-18b); 115.94 (C-21b); 115.99 (C-2b); 116.83 (C-5b); 117.42 (C-22b); 118.42 (C-4b); 119.31 (C-11b); 121.09 (C-3b); 124.42 (C-13b); 125.89 (C-12b); 126.87 (C-17b); 138.35 (C-15b); 139.31 (C-10b); 145.04 (C-20b); 145.72 (C-19b); 145.83 (C-6b); 148.52 (C-1b); 154.95 (C-8b); 167.60 (C-23b). MS (ES+) m/z (%): 393 (100), 271 (50). HRMS (ESI) m/z: ($C_{21}H_{16}N_2O_6^+$) calc.: 393.10811, found: 393.10818.

Characterization data for **6** (2-(3,4-dihydroxyphenyl)-1*H*-benzo[d]imidazole-6-carboxylic acid)

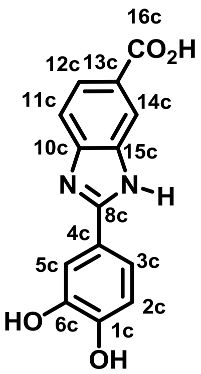

^{1}H NMR (600 MHz, DMSO-d_6): 6.97 (d, J = 8.3, 1H, H-2c); 7.54 (dd, J = 2.2, 8.3 Hz, 1H, H-3c); 7.61 (d, J = 2.2 Hz, 1H, H-5c); 7.70 (d, J = 8.4 Hz, 1H, H-11c); 7.93 (dd, J = 1.5, 8.4 Hz, 1H, H-12c); 8.17 (dd, J = 0.4, 1.5 Hz, 1H, H-14c), 9.51 (bs, 1H, OH); 10.02 (bs, 1H, OH), 12.98 (bs, 1H, OH). ^{13}C NMR (151 MHz, DMSO-d_6): 113.71 (C-11c); 114.85 (C-5c); 115.77 (C-14c); 116.26 (C-2c); 117.46 (C-4c); 119.92 (C-3c); 124.88 (C-12c); 126.01 (C-13c); 135.77 (C-15c); 139.18 (C-10c); 146.12 (C-6c); 149.85 (C-1c); 153.18 (C-8c); 167.46 (C-16c). MS (ES+) m/z (%): 271 (100). HRMS (ESI) m/z: ($C_{14}H_{10}N_2O_4^+$) calc.: 271.07133, found: 271.07138.

^{1}H NMR and ^{13}C NMR data for **5a$_{\mathbf{revised}}$**, **5b$_{\mathbf{revised}}$**, and **6** obtained in this work have been compared with the data reported by Yuan *et al.*[2] for compound **5** (see Table S4).

Table S4. ^{1}H NMR and ^{13}C NMR data for **5a$_{revised}$**, **5b$_{revised}$**, and **6** obtained in this work compared with the data reported by Yuan *et al.*[2] for compound **5**.

^{1}H NMR $_{reported}$ (ppm)[a]	^{1}H NMR$_{this\ work}$ (ppm)[b]		
**5**	**5a$_{revised}$**	**5b$_{revised}$**	**6**
12.88 (s,1H)	12.96 (bs,1H,COOH)	12.90 (bs,1H,COOH)	12.98 (bs,1H,COOH)
--------	--------	--------	10.02 (bs,1H,OH)
	9.82 (bs,1H,OH)	9.74 (bs,1H,OH)	--------
9.37 (bs,2H)	9.49 (bs,1H,OH)	9.44 (bs,1H,OH)	9.51 (bs,1H,OH)
8.95 (bs,2H)	8.97 (bs,2H,OH)	8.93 (bs,2H,OH)	--------
--------	8.06 (d,1H,H-14a)	8.24 (d,1H,H-11b)	8.17 (dd,1H,H-14c)
--------	7.94 (dd,1H,H-12a)	--------	7.93 (dd,1H,H-12c)
7.80 (m,1H)	--------	7.89 (dd,1H,H-13b)	
7.72 (m,1H)	7.78 (d,1H,H-11a)	--------	7.70 (d,1H,H-11c)
--------	--------	--------	7.61 (d,1H,H-5c)
7.47 (m,1H)	--------	7.55 (d,1H,H-14b)	7.54 (dd,1H,H-3c)
7.22 (s,1H)	7.25 (d,1H,H-5a)	7.23 (d,1H,H-5b)	--------
7.04 (t,1H)	7.10 (dd,1H,H-3a)	7.07 (dd,1H,H-3b)	--------
6.87 (t,1H)	6.92 (d,1H,H-2a)	6.90 (d,1H,H-2b)	6.97 (d,1H,H-2c)
6.63 (t,1H)	6.67 (d,1H,H-21a)	6.65 (d,1H,H-21b)	--------
6.37 (t,2H)	6.47 (d,1H,H-18a)	6.43 (d,1H,H-18b)	--------
--------	6.39 (dd,1H,H-22a)	6.37 (dd,1H,H-22b)	--------
5.44 (s,1H)	5.51 (s,2H,H-16a)	--------	--------
5.40 (s,1H)	--------	5.45 (s,2H,H-16b)	--------

^{13}C NMR $_{reported}$ (ppm)[a]	^{13}C NMR$_{this\ work}$ (ppm)[b]		
**5**	**5a$_{revised}$**	**5b$_{revised}$**	**6**
168.69	--------	--------	--------
--------	167.39 (C-23a)	167.60 (C-23b)	167.46 (C-16c)
157.06	--------	--------	--------
--------	155.28 (C-8a)	--------	--------
--------	--------	154.95 (C-8b)	--------
--------	--------	--------	153.18 (C-8c)
--------	--------	--------	149.85 (C-1c)
148.70	148.91 (C-1a)	148.52 (C-1b)	
148.36	--------	--------	--------
146.73	--------	--------	--------
146.21	--------	--------	146.12 (C-6c)
--------	145.90 (C-6a)	145.83 (C-6b)	--------
--------	145.78 (C19a)	145.72 (C-19b)	--------
145.41	145.09 (C-20a)	145.04 (C-20b)	--------
--------	--------	139.31 (C-10b)	139.18 (C-10c)
--------	--------	138.35 (C-15b)	--------
--------	--------	--------	135.77 (C-15c)
--------	134.71 (C-10a,C15a)	--------	--------
128.14	--------	--------	--------
--------	126.74 (C-17a)	126.87 (C-17b)	126.01 (C-13c)
125.17	125.82 (C-13a)	125.89 (C-12b)	--------
124.80	124.87 (C-12a)	--------	124.88 (C-12c)
124.05	--------	124.42 (C-13b)	--------
121.62	--------	--------	--------
121.22	121.30 (C-3a)	121.09 (C-3b)	--------
119.33	--------	119.31 (C-11b)	119.92 (C-3c)
119.10	--------	--------	--------
--------	--------	118.42 (C-4b)	--------
--------	117.70 (C-4a)	--------	--------
117.64	117.29 (C-22a)	--------	--------
117.35	117.06 (C-11a)	117.42 (C-22b)	117.46 (C-4c)
--------	116.83 (C-5a)	116.83 (C-5b)	
116.55	116.08 (C-2a)	--------	116.26 (C-2c)
--------	116.02 (C-21a)	115.99 (C-2b)	--------
115.00	--------	115.94 (C-21b)	115.77 (C-14c)
--------	--------	--------	114.85 (C-5c)
113.97	113.80 (C-14a)	--------	--------
113.51	113.66 (C-18a)	113.75 (C-18b)	113.71 (C-11c)
--------	--------	111.79 (C-14b)	--------
--------	47.95 (C-16a)	47.93 (C-16b)	--------

[a] ^{1}H NMR (300 MHz, DMSO-d_6). ^{13}C NMR (75 MHz, DMSO-d_6). [b] ^{1}H NMR (600 MHz, DMSO-d_6). ^{13}C NMR (151 MHz, DMSO-d_6).

Part C: General procedure for the synthesis of 1,2-disubstituted benzimidazoles 7, 8, 9, 10, 11, and 12 and their characterization

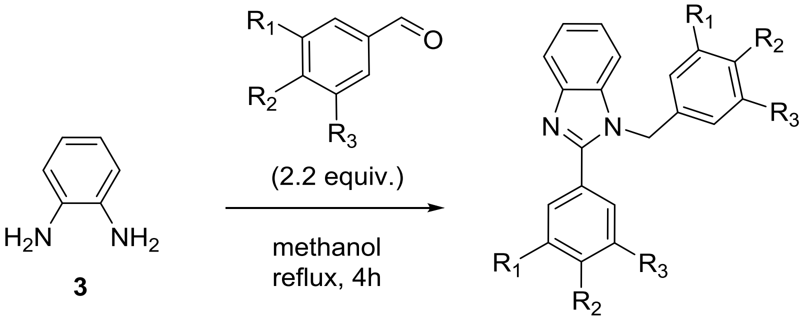

7, R_1=R_2=H, R_3=OH
8, R_1=R_3=H, R_2=OH
9, R_1=OMe, R_2=R_3=OH
10, R_1=H, R_2=OMe, R_3=OH
11, R_1=H, R_2=OH, R_3=OMe
12, R_1=R_3=OMe, R_2=OH

A solution of a respective benzaldehyde (2.04 mmol, 2.2 equiv.) in MeOH (2.5 mL) was added dropwise to a solution of 1,2-phenylenediamine (0.1 g, 0.925 mmol) in a two-neck round-bottom flask equipped with a water-cooled condenser. The resulting solutions were refluxed 4 hours (bath temperature 70 ^{o}C). After complete consumption of the starting 1,2-phenylenediamine (by TLC, 1:1, EtOAc : hexane), the mixture was cooled down to RT. Then, the mixture was transferred into a 100 mL round-bottom flask and MeOH was removed using rotary evaporator. Then, MeOH was added (1.5 mL) to obtain a suspension. In case, complete dissolution took place after the addition of MeOH, diethyl ether was added (30 mL) to trigger precipitation (typically after sonication for 1 min). Solids were then separated by filtration and washed with diethyl ether (30 mL).

Benzimidazole 7 (3-(1-(3-hydroxybenzyl)-1*H*-benzo[d]imidazol-2-yl)phenol)[3a-k]

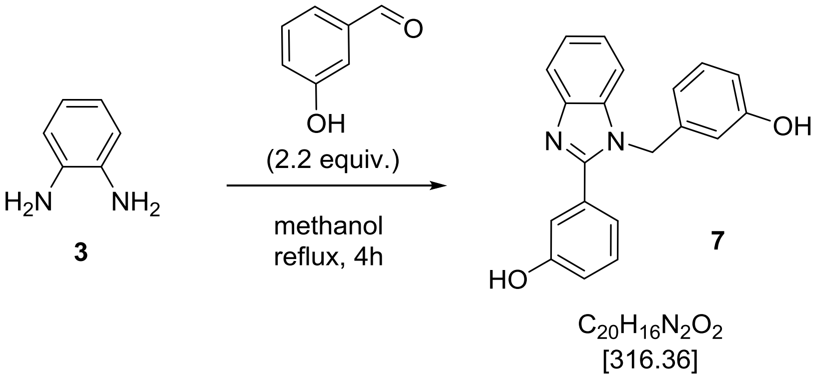

1,2-Disubstituted benzimidazole **7** was prepared according to the general procedure given above using 3-hydroxybenzaldehyde (0.25 g, 2.05 mmol, 2.2 equiv.). Compound **7** was isolated as an off-white powder (150 mg, 51% yield). For X-ray crystal structure data of **7**, see Section 4 (Figure S12).

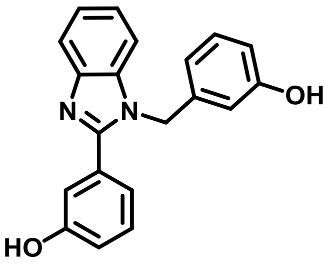

Mp 253-255 °C (253 °C [ref. 3k]). ^{1}H NMR (400 MHz, DMSO-d_6): 5.48 (s, 2H); 6.35-6.38 (m, 1H); 6.47 (d, J = 7.7 Hz, 1H); 6.62 (dd, J = 1.7, 8.1 Hz, 1H); 6.87-6.96 (m, 1H); 7.10 (t, J = 7.8 Hz, 2H); 7.13-7.16 (m, 1H); 7.18-7.23 (2H); 7.31 (t, J = 7.9 Hz, 1H); 7.39 (dd, J = 1.4, 7.3 Hz, 1H); 7.71 (dd, J = 1.3, 7.3 Hz, 1H); 9.41 (br, 1H); 9.78 (br, 1H). ^{13}C NMR (101 MHz, DMSO-d_6): δ = 47.43; 111.13; 112.68; 114.44; 115.99; 116.62; 116.91; 119.21; 119.49; 122.18; 122.63; 129.86; 129.86; 131.22; 135.90; 138.40; 142.62; 153.31; 157.55; 157.72. IR (KBr): ν (cm^{-1}) 3210, 3091, 3060, 2813, 2683, 2570, 2491, 1613, 1599, 1584, 1529, 1480, 1455, 1402, 1332, 1309, 1286, 1275, 1261, 1210, 1167, 1157, 997, 798, 791, 766, 753. MS (ESI) m/z (%): 317 (100). HRMS (ESI) m/z: ($C_{20}H_{17}N_2O_2^{+}$) calc.: 317.12845, found: 317.12850.

Benzimidazole 8 (4-(1-(4-hydroxybenzyl)-1*H*-benzo[d]imidazol-2-yl)phenol)[3b, 3e, 3h, 3i, 3j, 3k, 4a-q]

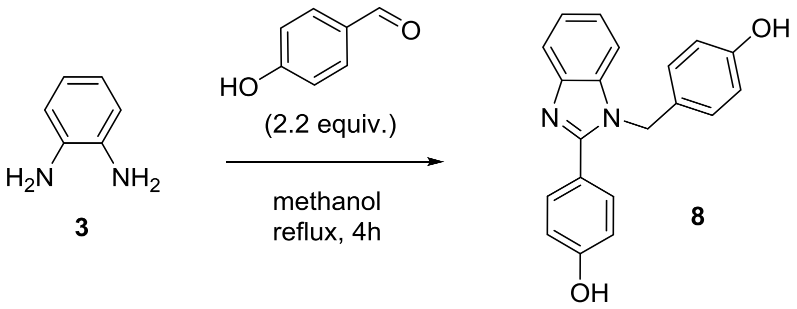

$C_{20}H_{16}N_2O_2$
[316.36]

1,2-Disubstituted benzimidazole **8** was prepared according to the general procedure given above using 4-hydroxybenzaldehyde (0.25 g, 2.04 mmol, 2.2 equiv.). Compound **8** was isolated as an off-white powder (130 mg, 44% yield). For X-ray crystal structure data of **8**, see Section 4 (Figure S13).

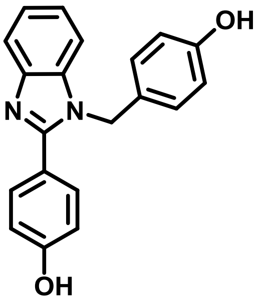

Mp 224-226 ^{o}C (222 ^{o}C [ref. 3k]). ^{1}H NMR (400 MHz, DMSO-d_6): 5.41 (s, 2H); 6.59-6.68 (m, 2H); 6.83 (d, J = 8.6 Hz, 2H); 6.86-6.92 (m, 2H); 7.14-7.24 (m, 2H); 7.41 (dd, J = 1.6, 7.1 Hz, 1H); 7.52-7.58 (m, 2H); 7.65 (dd, 1.5, 7.1 Hz, 1H); 9.39 (bs, 1H); 9.94 (bs, 1H). ^{13}C NMR (101 MHz, DMSO-d_6): δ = 47.07; 110.97; 115.48; 115.54; 118.84; 120.84; 121.88; 122.13; 127.14; 127.51; 130.61; 135.86; 142.72; 153.59; 156.67; 158.83. IR (KBr): ν (cm^{-1}) 3425, 3060, 2927, 2802, 2678, 2595, 1612, 1540, 1516, 1482, 1461, 1448, 1393, 1372; 1351; 1282, 1270, 1251; 1172; 1104; 1013; 987; 906; 840; 747. MS (ESI) m/z (%): 317 (100); 211 (65). HRMS (ESI) m/z: ($C_{20}H_{17}N_2O_2^{+}$) calc.: 317.12845, found: 317.12853.

Synthesis of compound 8 following the methodology reported by Kamaci and Kaya.[4r]

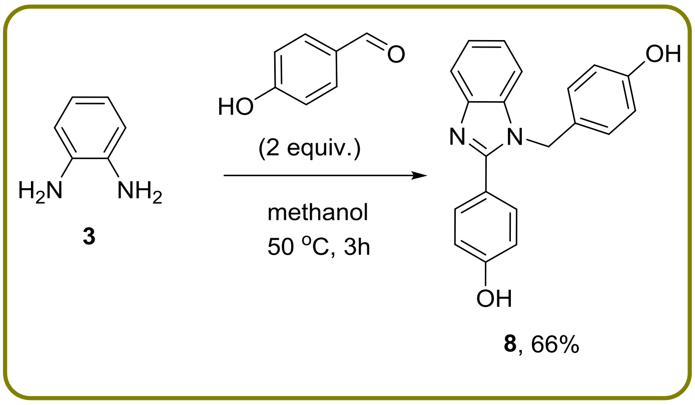

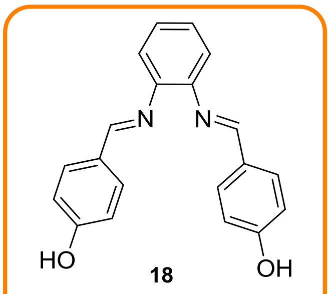

Structure of the reaction product reported by Kamaci and Kaya[4r]

4-Hydroxybenzaldehyde (1.0 g, 8.2 mmol, 2 equiv.) was added into a 100 mL three-necked round-bottom flask, which was fitted with condenser, and the aldehyde was dissolved in MeOH (8 mL). Aldehyde solution was heated up to 50 °C and then 1,2-phenylenediamine (0.443 g, 4.1 mmol) in MeOH (5 mL) was added into the flask. Reaction was maintained for 3 hours under reflux, and then cooled to room temperature. The compound **8** obtained as precipitate was washed with MeCN (2 × 5 mL) and water (2 × 10 mL). The product was dried in a vacuum oven at 75 °C for 24 hours. Compound **8** was obtained as a white solid (0.85 g, 2.7 mmol) in 66% yield. We confirmed the structure of benzimidazole **8** by X-ray crystallography (see below CCDC 1418143 and Section 4, Figure S13).

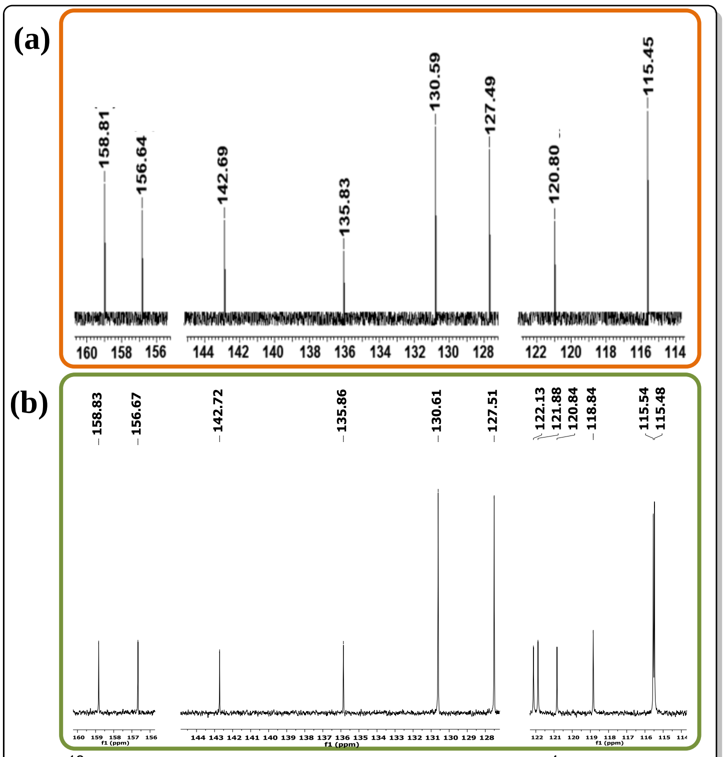

(a) ^{13}C NMR spectrum reported by Kamaci and Kaya.[4r]

(b) ^{13}C NMR spectrum of compound **8** obtained by us after we have followed the procedure described by Kamaci and Kaya.[4r]

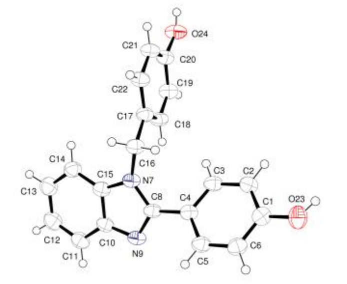

ORTEP view of compound **8** (see Section 4, CCDC 1418143)

Benzimidazole 9 (5-(1-(3,4-dihydroxy-5-methoxybenzyl)-1*H*-benzo[d]imidazol-2-yl)-3-methoxybenzene-1,2-diol)

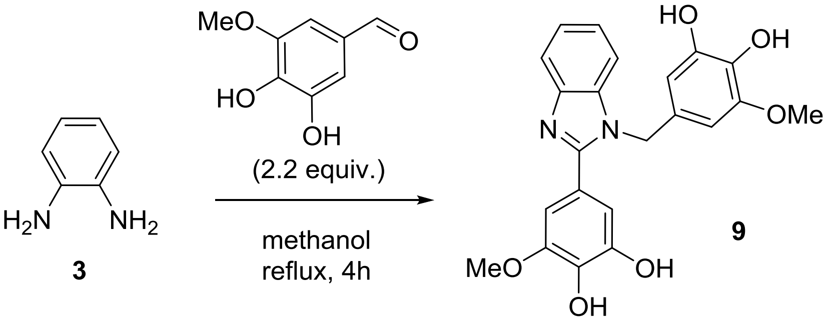

$C_{22}H_{20}N_2O_6$
[408.41]

1,2-Disubstituted benzimidazole **9** was prepared according to the general procedure given above using 3,4-dihydroxy-5-methoxybenzaldehyde (0.34 g, 2.02 mmol, 2.2 equiv.). Compound **9** was isolated as an off-white powder (160 mg, 43% yield).

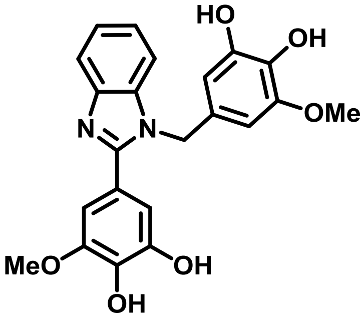

Mp 188-190 ^{o}C. ^{1}H NMR (400 MHz, DMSO-d_6): 3.65 (s, 3H); 3.68 (s, 3H); 5.38 (s, 2H); 6.04 (s, 1H); 6.28-6.33 (m, 1H); 6.78 (d, J = 1.6 Hz, 1H); 6.88 (d, J = 1.7 Hz, 1H); 7.17-7.25 (m, 2H); 7.42 (d, J = 4.0 Hz, 1H); 7.62-7.69 (m, 1H); 8.20 (s, 1H); 8.79 (s, 1H); 8.87 (s, 1H); 9.26 (s, 1H). ^{13}C NMR (101 MHz, DMSO-d_6): δ = 47.51; 55.73; 55.73; 101.91; 104.63; 106.68; 110.15; 110.89; 118.79; 119.99; 121.90; 122.16; 127.17; 133.27; 136.00; 136.13; 142.56; 145.82; 145.98; 148.20; 148.55; 153.71. IR (KBr): ν (cm^{-1}) 3431, 3010, 2964, 2339, 2844, 1609, 1518, 1489, 1457, 1397, 1341, 1208, 1095, 1014, 1001, 937, 911, 852, 811, 747. MS (ESI) m/z (%): 409 (100), 257 (52). HRMS (ESI) m/z: ($C_{22}H_{21}N_2O_6^{+}$) calc.: 409.13941, found: 409.13964.

Benzimidazole 10 (5-(1-(3-hydroxy-4-methoxybenzyl)-1*H*-benzo[d]imidazol-2-yl)-2-methoxyphenol)[3j, 3k, 5]

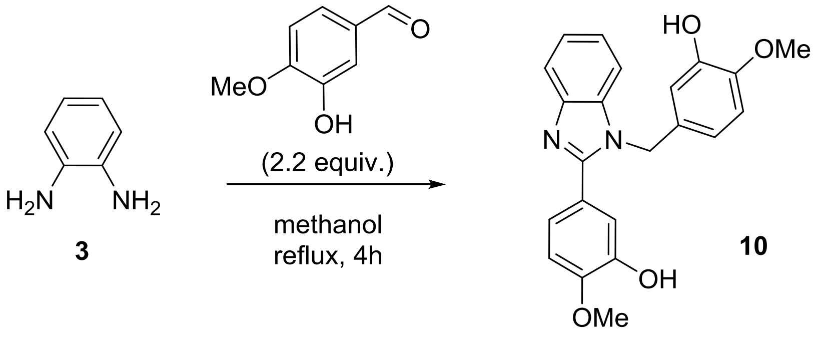

$C_{22}H_{20}N_2O_4$
[376.41]

1,2-Disubstituted benzimidazole **10** was prepared according to the general procedure given above using 3-hydroxy-4-methoxybenzaldehyde (0.31 g, 2.04 mmol, 2.2 equiv.). Compound **10** was isolated as a yellowish powder (120 mg, 34% yield).

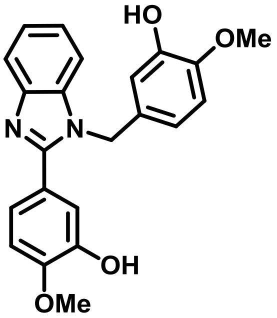

Mp 234-236 °C (229-231 °C [ref. 3k]). ^{1}H NMR (400 MHz, DMSO-d_6): 3.70 (s, 3H); 3.82 (s, 3H); 5.41 (s, 2H); 6.44 (m, 2H); 6.83 (d, J = 8.0 Hz, 1H); 7.04 (d, J = 8.4 Hz, 1H); 7.11 (dd, J = 2.1, 8.3 Hz, 1H); 7.15-7.21 (m, 3H); 7.37 (dd, J = 1.2, 7.4 Hz, 1H); 7.65-7.69 (m, 1H); 9.00 (bs, 1H); 9.35 (bs, 1H). ^{13}C NMR (101 MHz, DMSO-d_6): δ = 47.08, 55.56, 55.61, 110.98, 111.97, 112.38, 113.27, 116.28, 116.74, 118.90, 120.09, 121.95, 122.23, 122.54, 129.41, 135.92, 142.66, 146.55, 146.73, 146.94, 149.11, 153.30. IR (KBr): ν (cm^{-1}) 3438, 3056, 2973, 2939, 2839, 2730, 1620, 1593, 1535, 1513, 1486, 1459, 1140, 1393, 1350, 1277, 1242, 1230, 1220, 1166, 1132, 1023, 1015, 803, 763, 755. MS (ESI) m/z (%): 377 (100), 241 (10). HRMS (ESI) m/z: ($C_{22}H_{21}N_2O_4^+$) calc.: 377.14958, found: 377.14938.

Benzimidazole 11 (4-(1-(4-hydroxy-3-methoxybenzyl)-1*H*-benzo[d]imidazol-2-yl)-2-methoxyphenol)[3h, 3j, 3k, 4i, 4n, 4o, 6a-c, 6e]

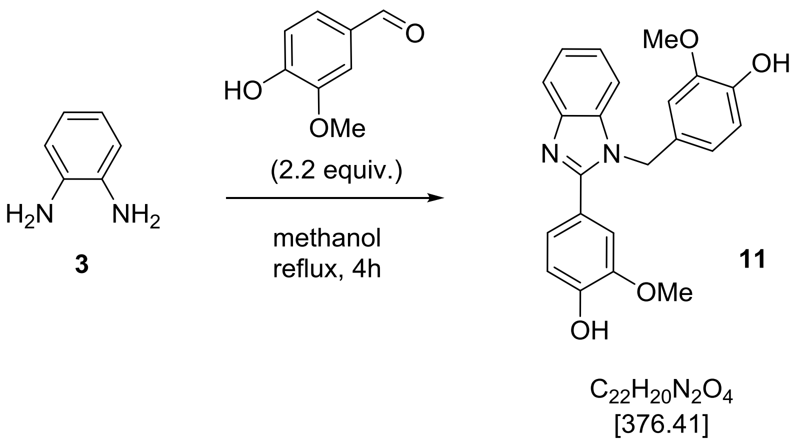

1,2-Disubstituted benzimidazole **11** was prepared according to the general procedure given above using 4-hydroxy-3-methoxybenzaldehyde (0.31 g, 2.04 mmol, 2.2 equiv.). Compound **11** was isolated as a yellowish powder (180 mg, 52% yield).

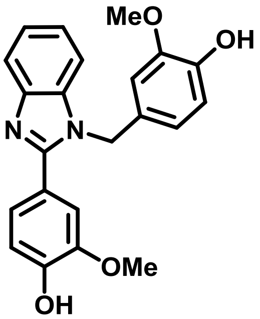

Mp 194-196 °C (184-186 °C [ref. 3k]). ^{1}H NMR (400 MHz, DMSO-d_6): 3.63 (s, 3H); 3.71 (s, 3H); 5.44 (s, 2H); 6.37 (dd, *J* = 1.7, 8.1 Hz, 1H); 6.65 (d, *J* = 8.1 Hz, 1H); 6.69 (d, *J* = 1.7 Hz, 1H); 6.91 (d, *J* = 8.2 Hz, 1H); 7.15-7.28 (m, 4H); 7.44-7.51 (m, 1H); 7.63-7.69 (m, 1H); 8.96 (s, 1H); 9.54 (s, 1H). ^{13}C NMR (101 MHz, DMSO-d_6): δ = 47.32; 55.46; 55.48; 110.70; 110.88; 113.00; 115.55; 115.57; 118.52; 118.84; 121.13; 121.90; 122.03; 122.18; 127.86; 136.04; 142.63; 145.85; 147.58; 147.69; 148.19; 153.55. IR (KBr): ν (cm^{-1}) 3431, 3062, 2999, 2935, 2836, 1604, 1527, 1516, 1489, 1461, 1442, 1427, 1389, 1334, 1278, 1251, 1220, 1180, 1164, 1150, 1123, 1032, 904, 808, 747. MS (ESI) m/z (%): 377 (35), 241 (100). HRMS (ESI) m/z: ($C_{22}H_{21}N_2O_4^+$) calc.: 377.14958, found: 377.14981.

Synthesis of compound 11 following the methodologies reported by Liu *et al.*[6d] (ref. 44 in the manuscript) and Xiao, Z.-A. *et al.*[6e] (ref. 45 in the manuscript)

Liu *et al.*[6d]

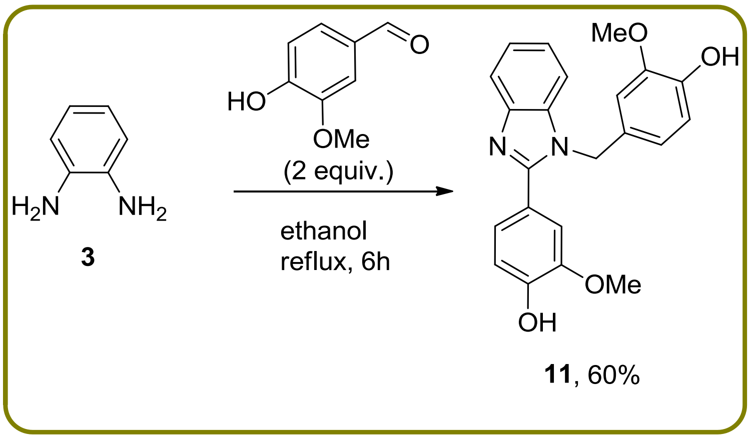

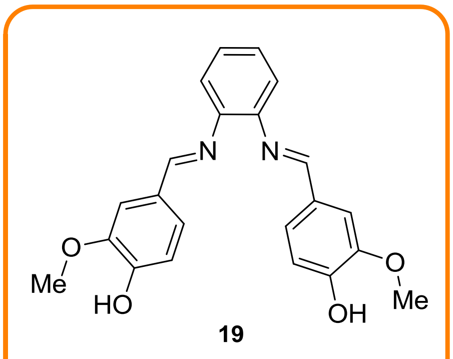

Structure of the reaction product reported by Liu *et al.*[6d]

Procedure following Liu *et al.*[6d] (ref. 44 in the manuscript)

4-Hydroxy-3-methoxybenzaldehyde (1.0 g, 6.6 mmol, 2 equiv.) and 1,2-phenylenediamine (0.355 g, 3.3 mmol) were put into a two-neck round-bottom flask equipped with a water-cooled condenser. Then anhydrous ethanol (5 mL) was added and the resulting solution was stirred for 6 hours at 80 ^{o}C. The reaction mixture was cooled to RT. Yellow precipitate formed. The solid was purified by recrystallization from ethanol/DMF (1 : 1). Compound **11** was isolated as a white solid (0.74 g, 1.96 mmol) in 60% yield.

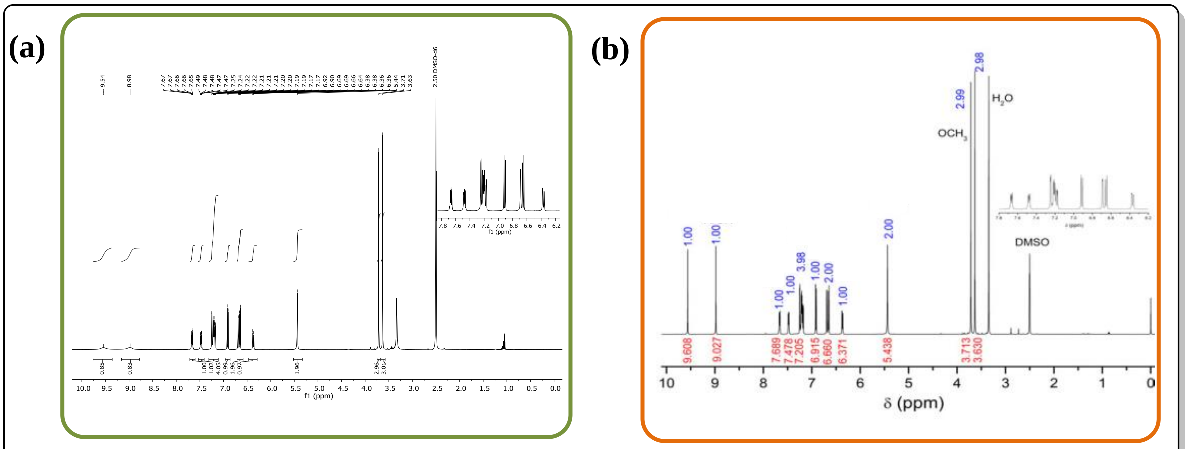

(a) ^{1}H NMR spectrum of product **8** after we have repeated procedure described by Liu *et al.*[6d] (ref. 44)
(b) ^{1}H NMR spectrum reported by Liu *et al.* in ref. 6d.

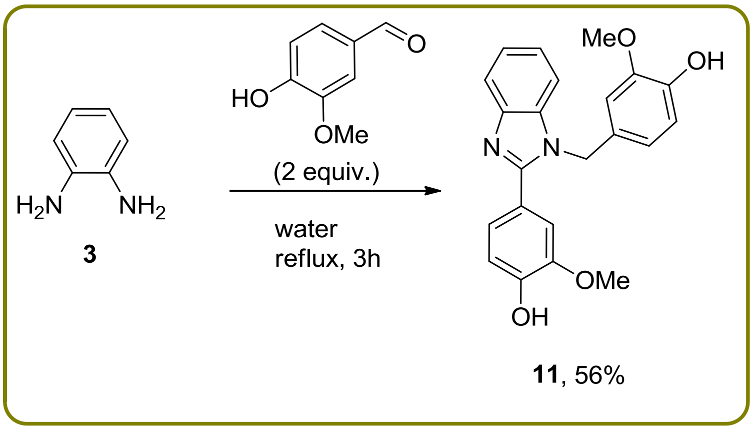

Xiao, Z.-A. *et al.*[6e]

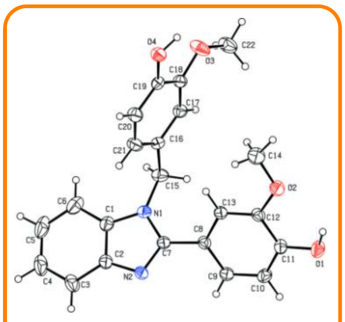

ORTEP view of compound **11** reported by Xiao, Z.-A. *et al.*[6e] (CCDC 852300)

Procedure following Xiao, Z.-A. *et al.*[6e] (ref. 45 in the manuscript)

4-Hydroxy-3-methoxybenzaldehyde (1.0 g, 6.6 mmol, 2 equiv.) and of 1,2-phenylenediamine (0.36 g, 3.3 mmol) were mixed in hot water (20 mL, 60 ^{o}C), the resulting mixture was stirred and refluxed for 3 hours. The solution was filtered, and the resulting yellow precipitate was recrystallized from methanol to obtain compound **11** as a white solid (0.7 g, 1.85 mmol) in 56% yield.

Benzimidazole 12 (4-(1-(4-hydroxy-3,5-dimethoxybenzyl)-1*H*-benzo[d]imidazol-2-yl)-2,6-dimethoxyphenol)

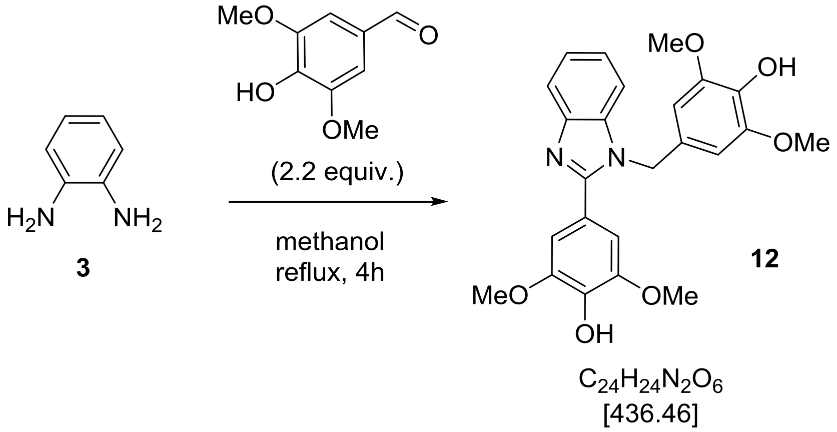

1,2-Disubstituted benzimidazole **12** was prepared according to the general procedure given above using 3,5-dimethoxy-4-hydroxybenzaldehyde (0.372 g, 2.04 mmol, 2.2 equiv.). Compound **12** was isolated as a yellowish powder (176 mg, 43% yield).

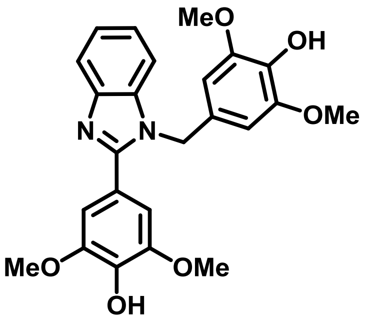

Mp 190-192 °C. ^{1}H NMR (400 MHz, DMSO-d_6): 3.58 (s, 6H); 3.72 (s, 6H); 5.46 (s, 2H); 6.34 (s, 2H); 6.99 (s, 2H); 7.21-7.26 (m, 2H); 7.56 (d, *J* = 5.4 Hz, 1H); 7.66-7.70 (m, 1H); 8.37 (s, 1H); 8.92 (s, 1H). ^{13}C NMR (101 MHz, DMSO-d_6): δ = 47.68; 55.93; 104.02; 104.08; 106.66; 110.84; 118.89; 120.04; 122.00; 122.31; 127.22; 134.89; 136.22; 137.21; 142.55; 147.97; 148.15; 153.68. IR (KBr): ν (cm^{-1}) 3431, 3063, 2989, 2938, 2838, 1608, 1518, 1489, 1464, 1441, 1425, 1319, 1245, 1216, 1115, 1038, 1011, 994, 911. MS (ESI) m/z (%): 437 (100), 271 (45). HRMS (ESI) m/z: ($C_{24}H_{25}N_2O_6^{+}$) calc.: 437.17071, found: 437.17066.

Part D: General procedure for the synthesis of salenes 13, 14, 15, 16, and 17 and their characterization

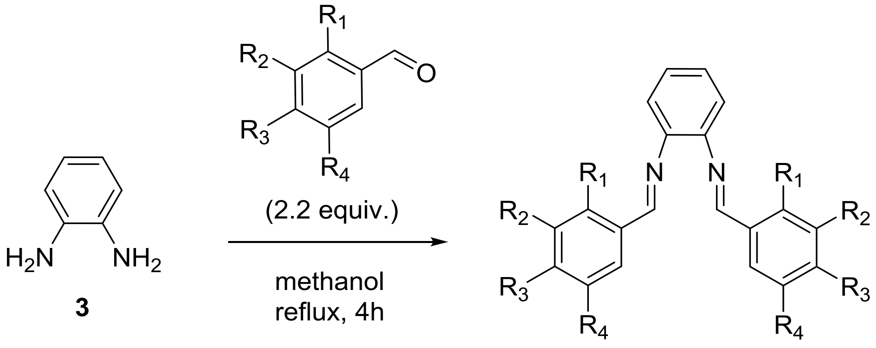

13, R_1=R_3=OH, R_2=R_4=H
14, R_1=OH, R_2=R_3=R_4=H
15, R_1=R_2=OH, R_3=R_4=H
16, R_1=R_2=R_3=OH, R_4=H
17, R_1=R_4=OH, R_2=R_3=H

A solution of a respective salicylaldehyde (2.04 mmol, 2.2 equiv.) in MeOH (2.5 mL) was added dropwise to a solution of 1,2-phenylenediamine (0.1 g, 0.925 mmol) in MeOH (2.5 mL) in a two-neck round-bottom flask equipped with a water-cooled condenser. The resulting solutions were refluxed 4 hours (bath temperature 70 °C). After complete consumption of the starting 1,2-phenylenediamine (by TLC, 1:1, EtOAc : hexane), the mixture was cooled down to RT. Then, the mixture was transferred into a 100 mL round-bottom flask and MeOH was removed using rotary evaporator. Then, MeOH was added (1.5 mL) followed by addition of diethyl ether (50 mL) to trigger precipitation. Suspension was then sonicated for 1 min. Solids were separated by filtration and washed with diethyl ether (30 mL).

Salen 13 (4,4'-((1*E*,1'*E*)-(1,2-phenylenebis(azanylylidene))bis(methanylylidene)) bis(benzene-1,3-diol)[7]

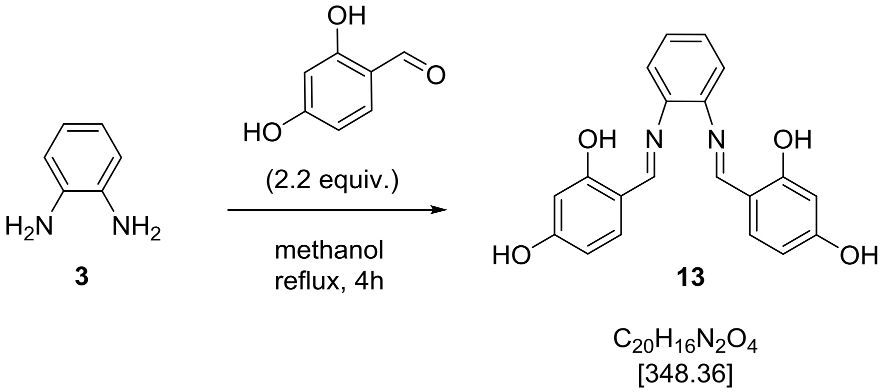

Salen **13** was prepared according to the general procedure given above using 2,4-dihydroxybenzaldehyde (0.28 g, 2.03 mmol, 2.2 equiv.). Compound **13** was isolated as an orange-yellow powder (151 mg, 47% yield). For previous syntheses and characterization of salen **13**, see also ref. 9 in the manuscript.

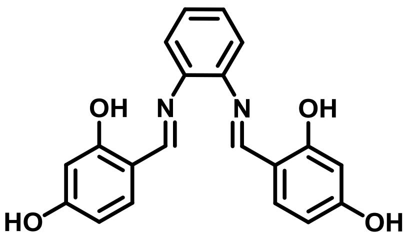

Mp 206-208 ^{o}C (224 ^{o}C [ref. 7]). ^{1}H NMR (400 MHz, DMSO-*d$_6$*): 6.28 (d, *J* = 2.3 Hz, 2H); 6.39 (dd, *J* = 2.3, 8.5 Hz, 2H); 7.32 (dd, *J* = 3.6, 5.8 Hz, 2H); 7.38 (dd, *J* = 3.3, 6.1 Hz, 2H); 7.43 (d, *J* = 8.6 Hz, 2H); 8.75 (s, 2H); 10.26 (bs, 2H); 13.38 (s, 2H). ^{13}C NMR (101 MHz, DMSO-*d$_6$*): δ = 102.41; 107.83; 112.30; 119.50; 127.05; 134.45; 142.03; 162.63; 162.92; 163.33. IR (KBr): ν (cm^{-1}) 3421, 3064, 2921, 2823, 2756, 1625, 1611, 1573, 1546, 1498, 1476, 1456, 1388, 1362, 1325, 1310, 1249, 1210, 1189, 1160, 1123, 1035, 1020, 977, 886, 855, 797, 747. MS (ESI) m/z (%): 348 (12), 347 (100), 238 (3), 237 (15), 225 (7). HRMS (ESI) m/z: ($C_{20}H_{15}N_2O_4^-$) calc.: 347.10373, found: 347.10309.

Salen 14 (2,2'-((1*E*,1'*E*)-(1,2-phenylenebis(azanylylidene))bis(methanylylidene)) diphenol)[8]

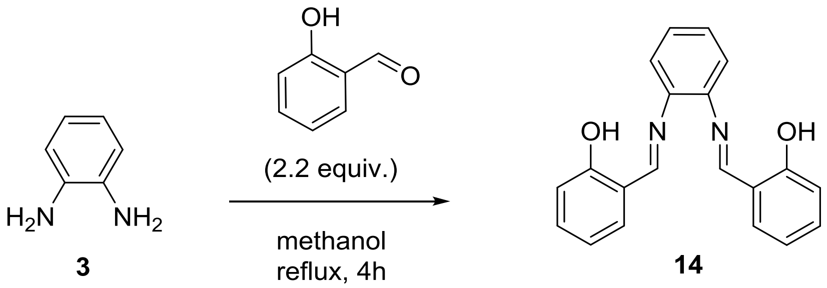

$C_{20}H_{16}N_2O_2$
[316.36]

Salen **14** was prepared according to the general procedure given above using salicylaldehyde (0.25 g, 2.04 mmol, 2.2 equiv.). Compound **14** was isolated as a yellow powder (180 mg, 61% yield). For X-ray crystal structure data of **14**, see Section 4 (Figure S14).

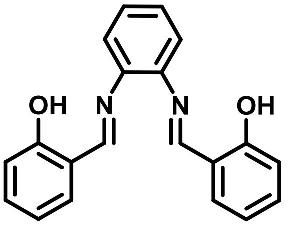

Mp 165 ^{o}C. ^{1}H NMR (400 MHz, DMSO-d_6): 6.95-6.99 (m, 4H); 7.38-7.49 (m, 6H); 7.67 (d, J = 7.6 Hz, 2H); 8.94 (s, 2H); 12.93 (s, 2H). ^{13}C NMR (101 MHz, DMSO-d_6): δ = 116.68; 119.09; 119.49; 119.75; 127.82; 132.46; 133.45; 142.26; 160.38; 164.04. IR (KBr): ν (cm^{-1}) 3433, 3054, 2989, 2926, 2854, 1616, 1586, 1563, 1495, 1481, 1458, 1449, 1404, 1277, 1236, 1193, 1151, 1116, 1104, 1045, 1030, 999, 944, 910, 760. MS (ESI) m/z (%): 317 (100). HRMS (ESI) m/z: ($C_{20}H_{17}N_2O_2^+$) calc.: 317.12845, found: 317.12855.

Salen 15 (3,3'-((1*E*,1'*E*)-(1,2-phenylenebis(azanylylidene))bis(methanylylidene)) bis(benzene-1,2-diol))[9]

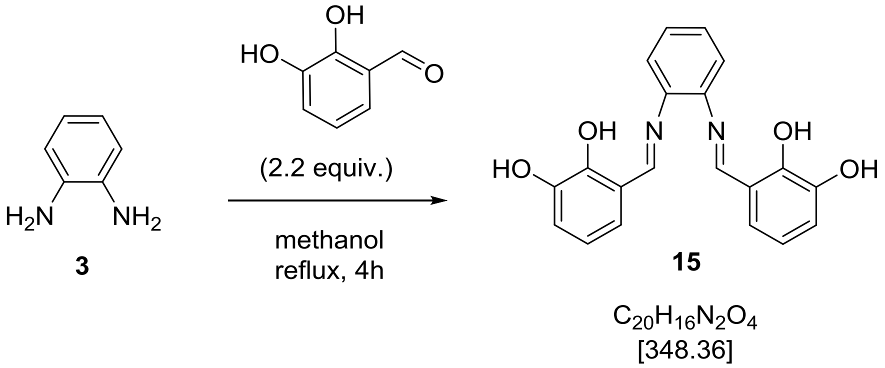

Salen **15** was prepared according to the general procedure given above prepared using 2,3-dihydroxybenzaldehyde (0.281 g, 2.04 mmol, 2.2 equiv.). Compound **15** was isolated as a red powder (192 mg, 60% yield).

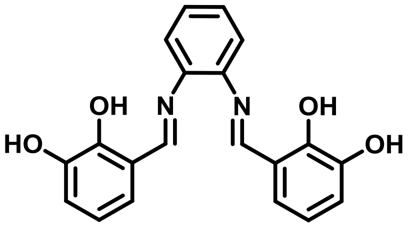

Mp 194-196 °C (186 °C ref. 9d). ^{1}H NMR (400 MHz, DMSO-*d$_6$*): 6.79 (t, *J* = 7.8 Hz, 2H); 6.94 (dd, *J* = 1.6, 7.8 Hz, 2H); 7.12 (dd, *J* = 1.5, 7.8 Hz, 2H); 7.39-745 (m, 4H), 8.88 (s, 2H); 9.26 (s, 2H); 12.91 (s, 2H). ^{13}C NMR (101 MHz, DMSO-*d$_6$*): δ = 118.77; 119.13; 119.60; 119.99; 122.80; 127.74; 142.18; 145.63; 149.46; 164.77. IR (KBr): ν (cm^{-1}) 3437, 3053, 2889, 1623, 1582, 1464, 1366, 1273, 1207, 1179, 1167, 1104, 1075, 1030, 982, 878, 863, 845, 780, 771, 733. MS (ESI) m/z (%): 347 (35), 225 (100). HRMS (ESI) m/z: ($C_{20}H_{15}N_2O_4^-$) calc.: 347.10373, found: 347.10367.

Salen 16 (4,4'-((1*E*,1'*E*)-(1,2-phenylenebis(azanylylidene))bis(methanylylidene)) bis(benzene-1,2,3-triol))[10]

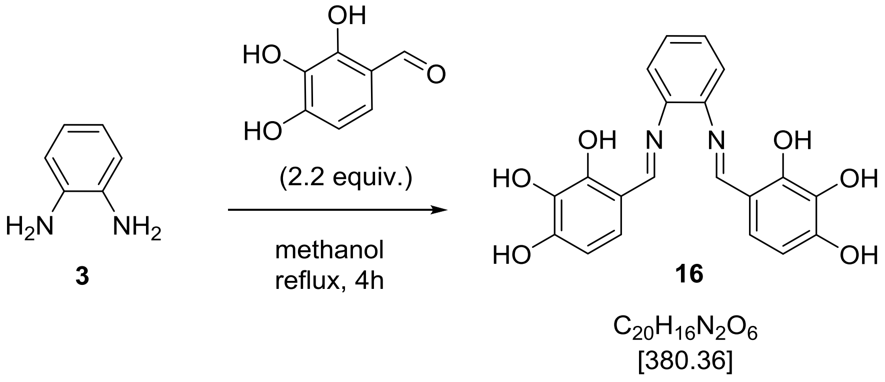

Salen **16** was prepared according to the general procedure given above using 2,3,4-trihydroxybenzaldehyde (0.314 g, 2.04 mmol, 2.2 equiv.). Compound **16** was isolated as an orange powder (190 mg, 54% yield).

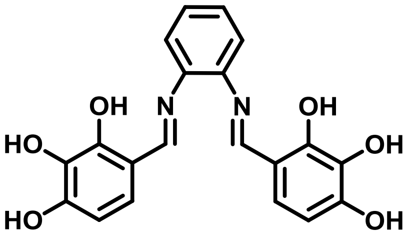

Mp 220-225 ^{o}C. ^{1}H NMR (400 MHz, DMSO-d_6): 6.41 (d, J = 8.5 Hz, 2H); 6.97 (d, J = 8.6 Hz, 2H); 7.21-7.43 (m, 4H); 8.59 (bs, 2H); 8.72 (s, 2H); 9.67 (bs, 2H); 13.35 (bs, 2H). ^{13}C NMR (101 MHz, DMSO-d_6): δ = 107.80; 112.62; 119.77; 124.20; 127.04; 132.37; 141.81; 150.48; 151.77; 163.74. IR (KBr): ν (cm^{-1}) 3427, 1621, 1580, 1156, 1513, 1460, 1365, 1303, 1275, 1207, 1162, 1078, 984, 870, 794, 752. MS (ESI) m/z (%): 381 (12), 403 (100, +Na), 404 (25, +Na). HRMS (ESI) m/z: ($C_{20}H_{17}N_2O_6^+$) calc.: 381.10811, found: 381.10822.

Salen 17 (2,2'-((1*E*,1'*E*)-(1,2-phenylenebis(azanylylidene))bis(methanylylidene)) bis(benzene-1,4-diol))[11]

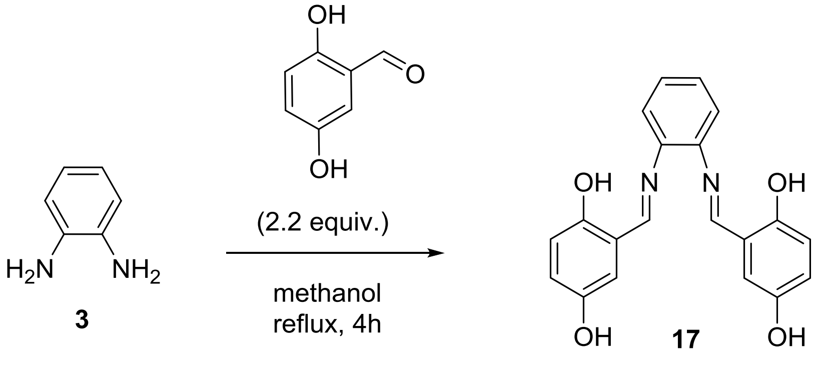

$C_{20}H_{16}N_2O_4$
[348.36]

Salen **17** was prepared according to the general procedure given above using 2,5-dihydroxybenzaldehyde (0.28 g, 2.04 mmol, 2.2 equiv.). Compound **17** was isolated as an orange powder (100 mg, 31% yield).

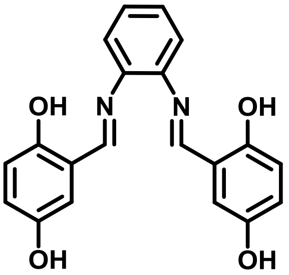

Mp 206-208 ^{o}C (140 ^{o}C decomp., MeOH [ref. 11d]). ^{1}H NMR (400 MHz, DMSO-d_6): 6.78 (d, J = 8.8 Hz, 2H); 6.86 (dd, J = 2.9, 8.8 Hz, 2H); 7.03 (d, J = 2.3 Hz, 2H); 7.33-7.45 (m, 4H); 8.80 (s, 2H); 9.10 (bs, 2H); 12.12 (bs, 2H). ^{13}C NMR (101 MHz, DMSO-d_6): δ = 116.84; 117.21; 119.42; 119.82; 121.28; 127.58; 142.54; 149.55; 153.20; 163.79. IR (KBr): ν (cm^{-1}) 3471, 3061, 1681, 1592, 1573, 1488, 1456, 1371, 1337, 1285, 1212, 1199, 1156, 1106, 1047, 1019, 976, 961, 886, 868, 822, 786, 753, 726. MS (ESI) m/z (%): 372 (25, +Na), 371 (100, +Na), 349 (20), 227 (2.5). HRMS (ESI) m/z: ($C_{20}H_{17}N_2O_4^{+}$) calc.: 349.11828, found: 349.11838.

4) X-ray crystallography

The single crystal data were collected at 180K on Xcalibur PX diffractometer with the graphite monochromatized $Cu_{K\alpha}$ radiation (λ=1.54180 Å). CrysAlisProCCD[12] was used for data collection, cell refinement and data reduction. The structure was solved by charge flipping using SUPERFLIP[13] and refined by full-matrix least-squares on F with CRYSTALS.[14] All non-hydrogen atoms were refined anisotropically. Hydrogen atoms were located in a Fourier difference map, but those attached to carbon atoms were repositioned geometrically and then refined with riding constraints.

Crystal data for $\mathbf{1_{revised}}$ (0.02 x 0.06 x 0.21 mm)**:**

Single crystals of $\mathbf{1_{revised}}$ suitable for X-ray analysis were grown *via* a slow diffusion of EtOAc into a MeOH solution of $\mathbf{1_{revised}}$.

$C_{24}H_{24}N_2O_6$, triclinic, space group *P*-1, a = 9.1566(5) Å, b = 9.3244(6) Å, c = 12.9830(6) Å, α = 91.302(4)°, β = 91.301(4)°, γ = 108.164(6)°, V = 1052.45(11) Å^3, Z = 2, M = 436.46, 18508 reflections measured, 4298 independent reflections. Final R = 0.047, wR = 0.019, *GoF* = 0.875 for 3251 reflections with $I > 2\sigma(I)$ and 307 parameters. CCDC 1030968.

The asymmetric unit contains one molecule of $\mathbf{1_{revised}}$ and one molecule of ethyl acetate, which was found to be disordered over two sites with occupancies of 0.6 and 0.4.

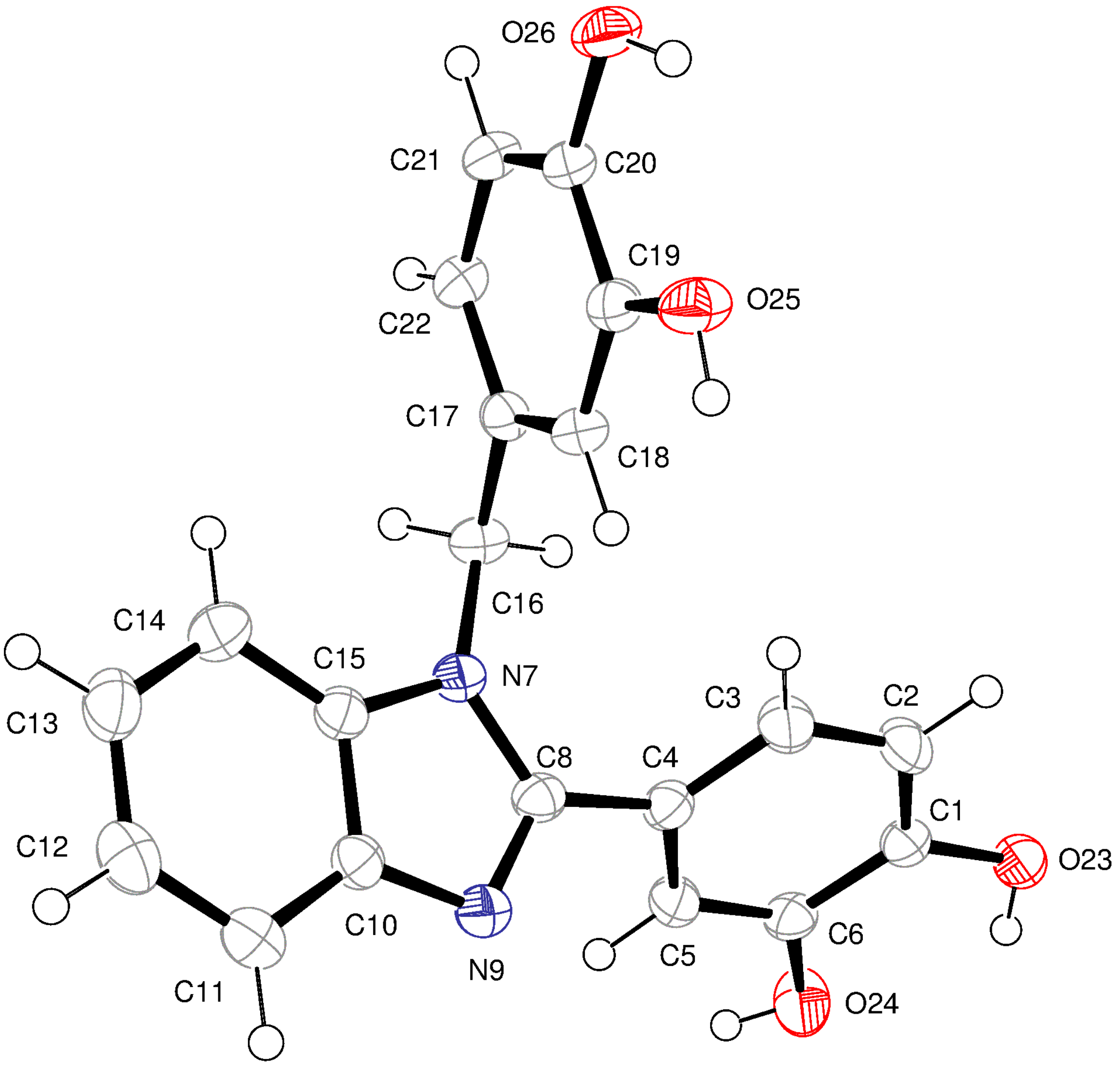

Figure 11. An ORTEP[15] view of $\mathbf{1}_{\mathbf{revised}}$, displacement ellipsoids shown with 50 % probability.

Single-crystal diffraction data of **7**, **8** and **14** were collected on Xcalibur X-ray diffractometer with $Cu_{K\alpha}$ (λ=1.54180 Å) at 180K. CrysAlisProCCD[12] was used for data collection, cell refinement and data reduction. The structures were solved by direct methods with SIR92[16] and refined by full-matrix least-squares on F with CRYSTALS.[14] The hydrogen atoms were all located in a difference Fourier map, but those attached to carbon atoms were recalculated into idealized positions and refined with riding constraints. All non-hydrogen atoms were refined with anisotropic displacement parameters.

Crystal data for 7 (0.10 x 0.22 x 0.41 mm)**:**

Single crystals of **7** suitable for X-ray analysis were grown *via* a slow diffusion of EtOAc into a MeOH solution of **7**.

$C_{20}H_{16}N_2O_2$, monoclinic, space group $P2_1/n$, a = 10.5990(5) Å, b = 12.0928(5) Å, c = 12.6438(6) Å, β = 96.933(4)°, V = 1608.72(14) $Å^3$, Z = 4, M = 316.36, 14378 reflections measured, 3280 independent reflections. Final R = 0.048, wR = 0.039, GoF = 1.292 for 2896 reflections with $I > 2\sigma(I)$ and 218 parameters. CCDC 1418142.

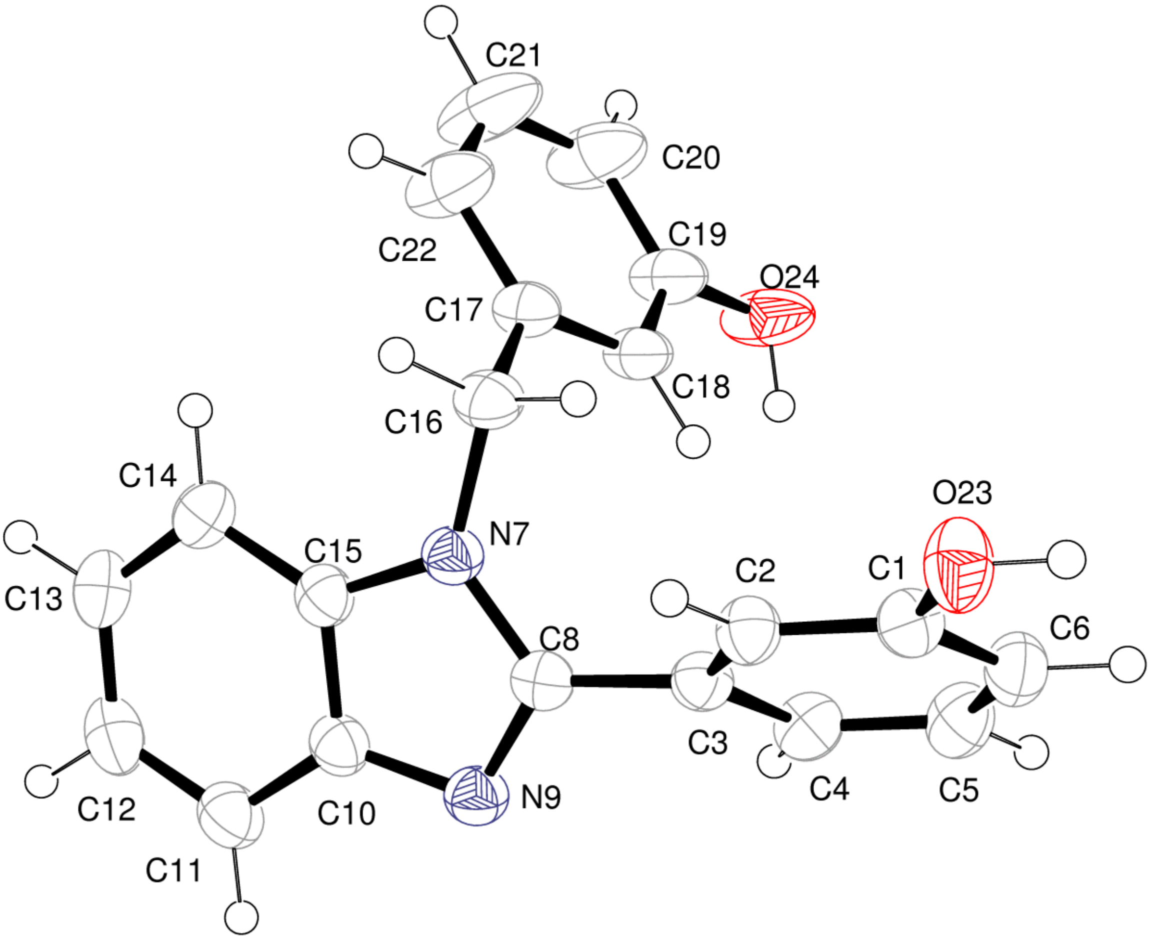

Figure S12. An ORTEP[15] view of **7**, displacement ellipsoids shown with 50 % probability.

Crystal data for 8 (0.08 x 0.37 x 0.76 mm)**:**

Single crystals of **8** suitable for X-ray analysis were grown *via* a slow evaporation of a CH_3CN solution of **8**.

$C_{20}H_{16}N_2O_2.0.5(CH_3CN).0.5(H_2O)$, triclinic, space group *P*-1, a = 5.2966(3) Å, b = 10.3809(8) Å, c = 16.8834(15) Å, α = 106.687(7)°, β = 92.736(6)°, γ = 100.603(6)°, V = 869.02(12) $Å^3$, Z = 2, M = 345.89, 8304 reflections measured, 3502 independent reflections. Final R = 0.081, wR = 0.081, GoF = 1.089 for 3022 reflections with $I > 2\sigma(I)$ and 253 parameters. CCDC 1418143. Besides one molecule of **8** the asymmetric unit also contains partially occupied molecules of acetonitrile and water (with occupancy factors being 0.5 in both cases).

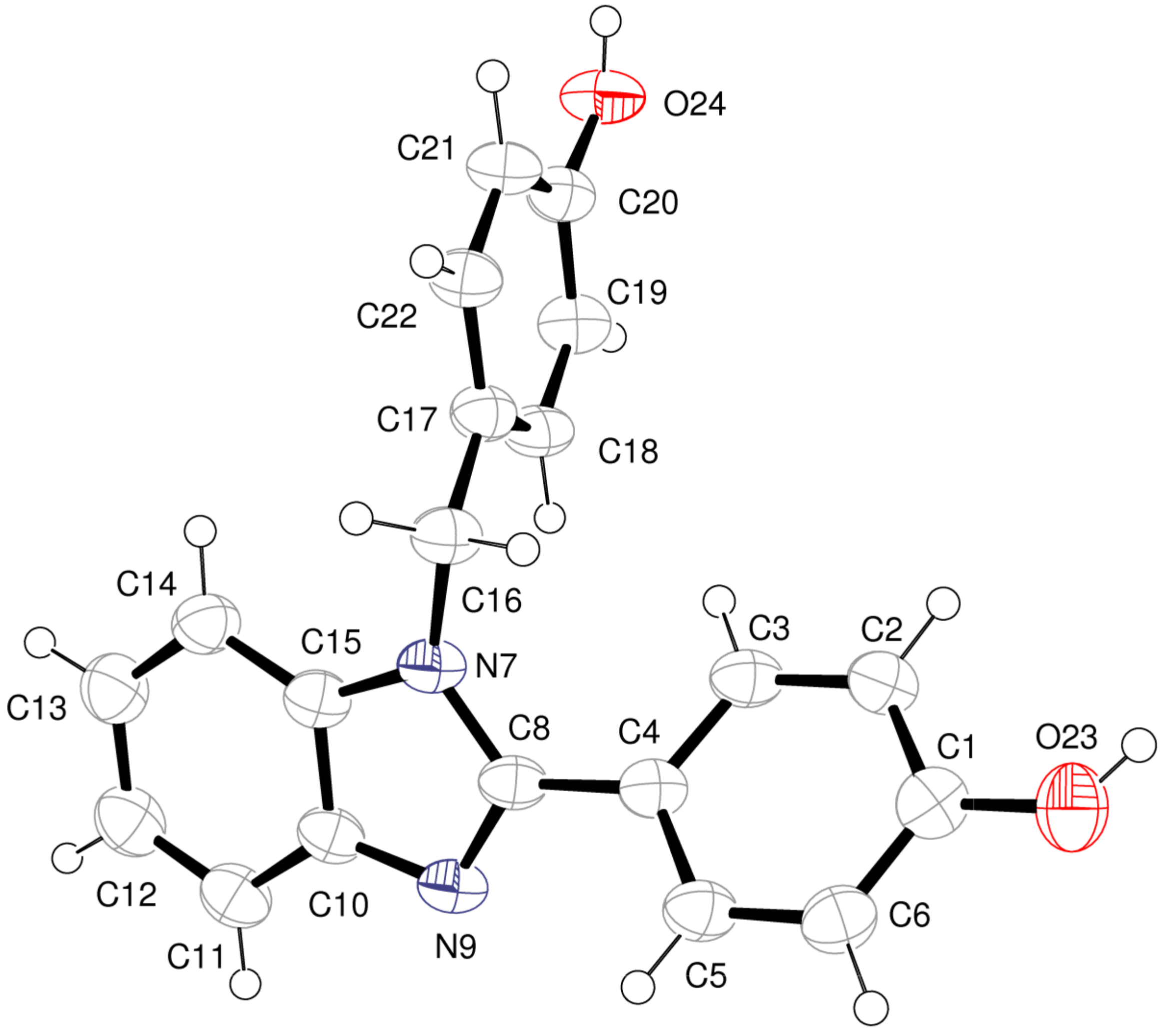

Figure S13. An ORTEP[15] view of **8**, displacement ellipsoids shown with 50 % probability. The solvent molecules were omitted for clarity.

Crystal data for 14 (0.03 x 0.25 x 0.65 mm)**:**

Single crystals of **14** suitable for X-ray analysis were grown *via* a slow evaporation of a MeOH solution of **14**.

$C_{20}H_{16}N_2O_2$, monoclinic, space group $P2_1/c$, a = 5.9538(2) Å, b = 16.4375(5) Å, c = 16.2392(5) Å, β = 91.785(3)°, V = 1588.49(9) Å^3, Z = 4, M = 316.36, 13421 reflections measured, 3246 independent reflections. Final R = 0.055, wR = 0.063, GoF = 1.114 for 2329 reflections with $I > 2\sigma(I)$ and 217 parameters. CCDC 1423721.

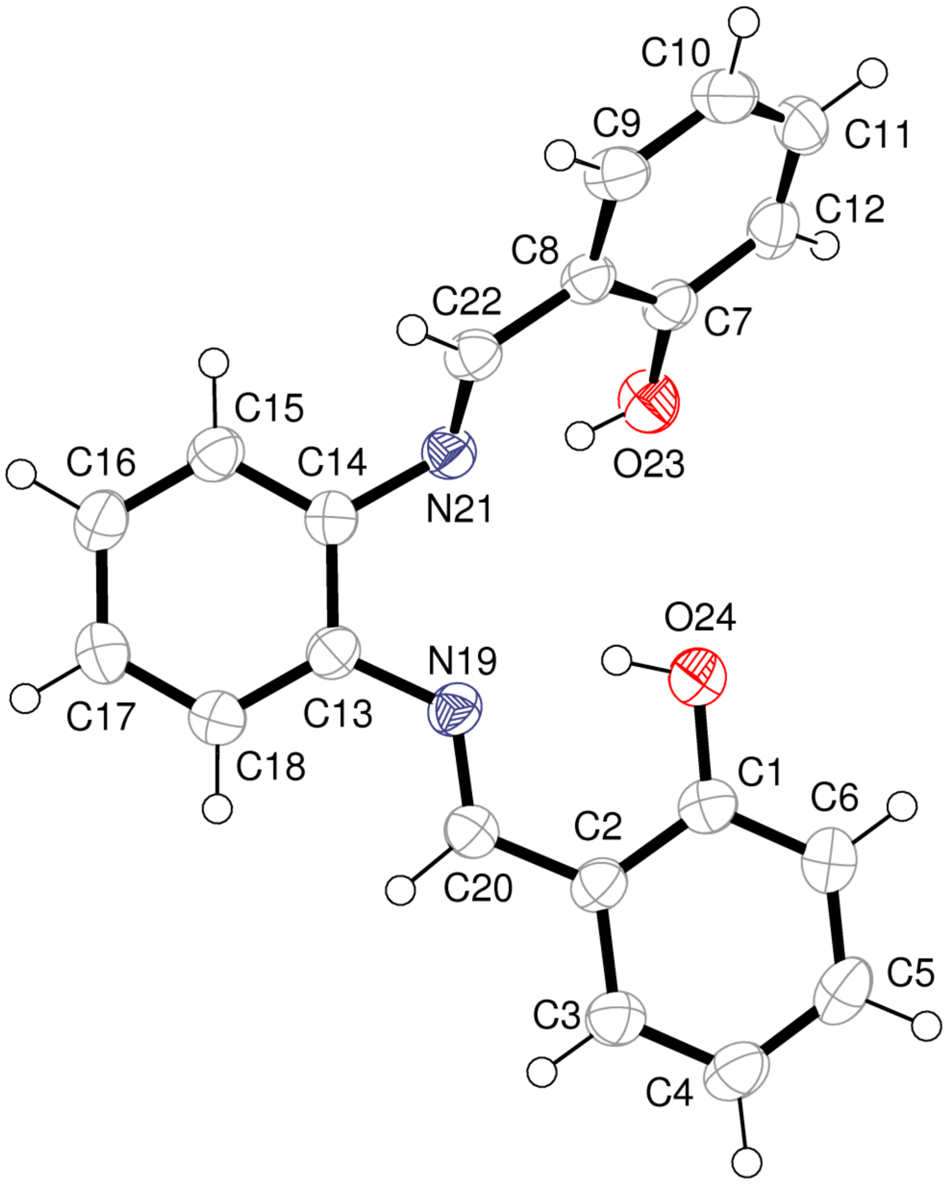

Figure S14. An ORTEP[15] view of **14**, displacement ellipsoids shown with 50 % probability.

5) References

1. Kumar, T. B., Sumanth, Ch., Rao, A. V. D., Kalita, D., Rao, M. S., Sekhar, K. B. C., Kumar, K. S. & Pal, M. Catalysis by FeF_3 in water: a green synthesis of 2-substituted 1,3-benzazoles and 1,2-disubstituted benzimidazoles. *RCS Advances* **2**, 11510-11519 (2012).
2. Yuan, L.B., Tian, T., Chen, Y., Yan, Sh., Xing, X., Zhang, Zh., Zhai, Q., Xu, L., Wang, Sh., Weng, X., Yuan, B., Feng, Y. & Zhou, X. Existence of G-quadruplex structures in promoter region of oncogenes confirmed by G-quadruplex DNA cross-linking strategy. *Sci. Rep.* **3**, 1811; DOI:10.1038/srep01811 (2013).
3. (a) Ghosh, P., Mandal, A. & Subba, R. γ-Maghemite-silica nanocomposite: A green catalyst for diverse aromatic N-heterocycles. *Catal. Commun.* **41**, 146-152 (2013); (b) Shelkar, R., Sarode, S. & Nagarkar, J. Nano ceria catalyzed synthesis of substituted benzimidazole, benzothiazole, and benzoxazole in aqueous media. *Tetrahedron Lett.* **54**, 6986-6990 (2013); (c) Paul, S. & Basu, B. Highly selective synthesis of libraries of 1,2-disubstituted benzimidazoles using silica gel soaked with ferric sulfate. *Tetrahedron Lett.* **53**, 4130-4133 (2012); (d) Ghosh, P. & Mandal, A. Synthesis of functionalized benzimidazoles and quinoxalines catalyzed by sodium hexafluorophosphate bound Amberlite resin in aqueous medium. *Tetrahedron Lett.* **53**, 6483-6488 (2012); (e) Behbahani, F. K. & Ziaei, P. A green route for the one-pot synthesis of 1,2-disubstituted benzimidazoles using iron(III) phosphate under solvent-less conditions. *Chin. J. Chem.* **30**, 65-70 (2012); (f) Ghosh, P. & Mandal, A. Catalytic role of sodium dodecyl sulfate: Selective synthesis of 1, 2-disubstituted benzimidazoles in water. *Catal. Commun.* **12**, 744-747 (2011); (g) Mohammadizadeh, M. R. & Zeinakhatoun-Taghavi, S. Trifluoroacetic acid as an efficient catalyst for the room temperature synthesis of 2-aryl-1-arylmethyl-1H-1,3-benzimidazoles in aqueous media. *E-J. Chem.* **8**, 101-106 (2011); (h) Mukhopadhyay, Ch., Datta, A., Butcher, R. J., Paul, B. K., Guchhait, N. & Singha, R. Water mediated expeditious and highly selective synthesis of 2-aryl-1-arylmethyl-1H-benzimidazoles by Dowex 50W: fluorescence properties of some representative compounds. *ARKIVOC* **13**, 1-22 (2009); (i) Wan, J.-P.; Gan, Sh.-F.; Wu, J.-M. & Pan, Y. Water mediated chemoselective synthesis of 1,2-disubstituted benzimidazoles using o-phenylenediamine and the extended synthesis of quinoxalines. *Green Chem.* **11**, 1633-

1637 (2009); (j) Shaterian, H. R., Fahimi, N. & Azizi, K. Selective synthesis of 2-aryl-1-benzylated-1H-benzimidazoles. *Chin. J. Chem.* **29**, 2389-2393 (2011); (k) Das-Sharma, S. & Konwar, D. Practical, ecofriendly, and chemoselective method for the synthesis of 2-aryl-1-arylmethyl-1H-benzimidazoles using amberlite IR-120 as a reusable heterogeneous catalyst in aqueous media. *Synth. Commun.* **39**, 980-991 (2009).

4. (a) Bahrami, K. Khodaei, M. M. & Nejati, A. Synthesis of 1,2-disubstituted benzimidazoles, 2-substituted benzimidazoles and 2-substituted benzothiazoles in SDS micelles. *Green Chem.* **12**, 1237-1241 (2010); (b) Chebolu, R., Kommi, D. N., Kumar, D., Bollineni, N. & Chakraborti, A. K. Hydrogen-Bond-Driven Electrophilic Activation for Selectivity Control: Scope and Limitations of Fluorous Alcohol-Promoted Selective Formation of 1,2-Disubstituted Benzimidazoles and Mechanistic Insight for Rationale of Selectivity. *J. Org. Chem.* **77**, 10158-10167 (2012); (c) Lei, M., Ma, L. & Hu, L. One-pot synthesis of 1H-benzimidazole derivatives using thiamine hydrochloride as a reusable organocatalyst. *Synth. Commun.* **42**, 2981-2993 (2012); (d) Veisi, H., Sedrpoushan, A., Zolfigol, M. A. & Mohanazadeh, F. Synthesis and application of silica phenyl sulfonic acid as a solid acid heterogeneous catalyst for one-pot synthesis of 2-aryl-1-arylmethyl-1H-1,3-benzimidazoles and bis(indolyl)methanes in water. *J. Heterocycl. Chem.* **48**, 1448-1454 (2011); (e) Azarifar, D., Khosravi, K., Najminejad, Z. & Soleimani, K. Synthesis of 1,2-disubstituted benzimidazoles and 2-substituted benzothiazoles catalyzed by HCl-treated trans-3,5-dihydroperoxy-3,5-dimethyl-1,2-dioxolane. *Heterocycles* **81**, 2855-2863 (2010); (f) Patil, P. P., Deshmukh, M. B., Mulik, A. G., Chandam, D. R., Patil, D. R., Jagdale, S. D., Anbhule, P. V., Salunkhe, D. K. & Sankpal, S. A. An efficient, greener synthesis of 2-aryl-1-arylmethyl-1H-benzimidazoles using polystyrene sulfonic acid as a catalyst. *Pharm. Chem.* **3**, 599-605 (2011); (g) Yadav, J. S., Reddy, B. V. S., Premalatha, K. & Shankar, K. S. Bismuth(III)-catalyzed rapid and highly efficient synthesis of 2-aryl-1-arylmethyl-1H-benzimidazoles in water. *Can. J. Chem.* **86**, 124-128 (2008); (h) Kumar, A. & Kapoor, K. K. Antimony chloride immobilized on neutral alumina: an efficient catalyst for the solvent-free selective synthesis of 1,2-disubstituted benzimidazoles. *J. Chem. Pharm. Res.* **3**, 369-374 (2011); (i) Santra, S., Majee, A. & Hajra, A. Nano indium oxide: an efficient catalyst for the synthesis of 1,2-disubstituted benzimidazoles in aqueous media. *Tetrahedron Lett.* **53**, 1974-1977 (2012); (j) Azarifar, D., Pirhayati, M., Maleki, B., Sanginabadi, M. & Yami, R. N.

Acetic acid-promoted condensation of *o*-phenylenediamine with aldehydes into 2-aryl-1-(arylmethyl)-1H-benzimidazoles under microwave irradiation. *J. Serb. Chem. Soc.* **75**, 1181-1189, (2010); (k) Bandyopadhyay, P., Sathe, M., Ponmariappan, S., Sharma, A., Sharma, P., Srivastava, A. K. & Kaushik, M. P. Exploration of in vitro time point quantitative evaluation of newly synthesized benzimidazole and benzothiazole derivatives as potential antibacterial agents. *Bioorg. Med. Chem. Lett.* **21**, 7306-7309 (2011); (l) Khosravi, K., Mobinikhaledi, A., Kazemi, S., Azarifar, D. & Rahmani, P. Trans-3,5-dihydroperoxy-3,5-dimethyl-1,2-dioxolane/HOAc/KI system as a new and mild catalyst for efficient synthesis of 1H-benzimidazoles and 1H-benzothiazoles in water. *Iran. J. Catal.* **4**, 25-31 (2014); (m) Khosravi, K. & Kazemi, S. Trans-3,5-dihydroperoxy-3,5-dimethyl-1,2-dioxolane/HBr system as new, effective, mild and non-toxic reagent for synthesis of 2-aryl-1H-benzothiazoles and 2-aryl-1-arylmethyl-1H-benzimidazoles. *J. Chin. Chem. Soc.* **59**, 557-560 (2012); (n) Durgareddy, G. A. N. K., Ravikumar, R., Ravi, S. & Adapa, S. R. A $Cu(NO_3)_2.3H_2O$ catalysed facile synthesis of substituted 4(3H)-quinazolinones and benzimidazoles. *J. Chem. Sci.* **125**, 175-182 (2013); (o) Rani, O. S., Devi, M. A., Srinivas-Rao, A., Reddy, E. R. & Mastan, S. K. Synthesis and antioxidant activity of some new [1-benzyl-2-phenyl-Substituted]-1H-5,6-substituted-benzo(d)imidazole derivatives. *PharmacologyOnLine* **1**, 373-380 (2009); (p) Ravi, V., Ramu, E., Vijay, K. & Srinivas-Rao, A. Zn-proline catalyzed selective synthesis of 1,2-disubstituted benzimidazoles in water. *Chem. Pharm. Bull.* **55**, 1254-1257 (2007); (q) Chakrabarty, M., Karmakar, S., Mukherji, A., Arima, S. & Harigaya, Y. Application of sulfamic acid as an eco-friendly catalyst in an expedient synthesis of benzimidazoles. *Heterocycles* **68**, 967-974 (2006); (r) Kamaci, M. & Kaya, I. Synthesis, thermal and morphological properties of polyurethanes containing azomethine linkage. *J. Inorg. Organomet. Polym.* **24**, 803-818 (2014).

5. Chaturvedi, A. K., Negi, A. S. & Khare, P. A simple and straightforward synthesis of substituted 2-arylbenzimidazoles over silica gel. *RSC Advances* **3**, 4500-4504 (2013).
6. (a) Jin, M., Xia, H., Kan, S. & Li, Z. Solvent-free synthesis of 1,2-disubstituted benzimidazoles in ball mill. *Jingxi Huagong* **28**, 1032-1035 (2011); (b) Pramanik, A., Roy, R., Khan, S., Ghatak, A. & Bhar, S. Eco-friendly synthesis of 2-aryl-1-arylmethyl-1H-benzimidazoles using alumina-sulfuric acid as a heterogeneous reusable catalyst. *Tetrahedron Lett.* **55**, 1771-1777 (2014); (c) Srinivasula-Reddy, L., Gangi-Reddy, N. C., Ram-Reddy, T., Lingappa, Y. & Mohan, R. B. Chemoselective

synthesis of 2-aryl-1-arylmethyl-1H-benzo[d]imidazoles using Indion 190 resin as a heterogeneous recyclable catalyst. *J. Korean Chem. Soc.* **55**, 304-307 (2011); (d) Liu, C. P., Wang, M. K. & Xiao, Q. Preparation, property characterization and UV-converting application of poly(conjugated azomethine-urethane)/hydroxyl polyacrylate resin. *J. Appl. Polym. Sci.*, 3629-3639 (2013); (e) Xiao, Z.-A., Gao, T., Huang, F.-J. & Jiang, T.-T. 4-[1-(4-Hydroxy-3-methoxybenzyl)-1Hbenzimidazol-2-yl]-2-methoxyphenol. Acta Cryst. **E67**, o3087 (2011).

7. Muñoz-Flores, B. M., Santillán, R., Farfán, N., Álvarez-Venicio, V., Jiménez-Pérez, V. M., Rodríguez, M., Morales-Saavedra, O. G., Lacroix, P. G., Lepetit, Ch. & Nakatani, K. Synthesis, X-ray diffraction analysis and nonlinear optical properties of hexacoordinated organotin compounds derived from Schiff bases. *J. Organomet. Chrem.* **769**, 64-71 (2014).
8. (a) Mirza-Aghayan, M., Ghassemzadeh, M., Hoseini, M. & Bolourtchian, M. Microwave-assisted synthesis of the tetradentate Schiff-bases under solvent-free and catalyst-free condition. *Synth. Commun.* **33**, 521-525 (2003); (b) Conte, V., Fabbianesi, F., Floris, B., Galloni, P., Sordi, D., Arends, I. W. C. E., Bonchio, M., Rehder, D. & Bogdal, D. Vanadium-catalyzed, microwave-assisted oxidations with H_2O_2 in ionic liquids. *Pure Appl. Chem.* **81**, 1265-127 (2009); (c) Reich, B. J. E., Greenwald, E. E., Justice, A. K., Beckstead, B. T., Reibenspies, J. H., North, S. W. & Miller, S. A. Ene-diamine versus Imine-amine Isomeric Preferences. *J. Org. Chem.* **70**, 8409-8416 (2005); (d) Doctrow, S. R., Huffman, K., Bucay-Marcus, C., Tocco, G., Malfroy, E., Adinolfi, C. A., Kruk, H., Baker, K., Lazarowych, N., Mascarenhas, J. & Bernard, M. Salen-Manganese Complexes as Catalytic Scavengers of Hydrogen Peroxide and Cytoprotective Agents: Structure-Activity Relationship Studies. *J. Med. Chem.* **45**, 4549-4558 (2002); (e) Carradori, S., De Monte, C., D'Ascenzio, M., Secci, D., Celik, G., Ceruso, M., Vullo, D., Scozzafava, A. & Supuran, C. T. Salen and tetrahydrosalen derivatives act as effective inhibitors of the tumor-associated carbonic anhydrase XII-A new scaffold for designing isoform-selective inhibitors. *Bioorg. Med. Chem. Lett.* **23**, 6759-6763 (2013); (f) Ansari, K. I., Grant, J. D., Woldemariam, G. A., Kasiri, S. & Mandal, S. S. Iron(III)-salen complexes with less DNA cleavage activity exhibit more efficient apoptosis in MCF7 cells. *Org. Biomol. Chem.* **7**, 926-932 (2009); (g) Reich, B. J. E., Justice, A. K., Beckstead, B. T., Reibenspies, J. H. & Miller, S. A. Cyanide-catalyzed cyclizations via aldimine coupling. *J. Org. Chem.* **69**, 1357-1359 (2004); (h) Singh, M. S., Singh, A. K., Singh, P. & Jain, R. An improved

and facile synthesis of Schiff bases in aqueous medium. *Org. Prep. Proced. Int.* **37**, 173-177 (2005); (i) Ganguly, M., Pal, A., Negishi, Y. & Pal, T. Diiminic Schiff Bases: An Intriguing Class of Compounds for a Copper-Nanoparticle-Induced Fluorescence Study. *Chem. – Eur. J.* **18**, 15845-15855 (2012); (j) Misra, S., Pandeya, K. B., Tiwari, A. K., Ali, Amtul Z., Saradamani, T., Agawane, S. B. & Madhusudana, K. Antihyperglycemic, α-glucosidase inhibitory and DPPH free radical scavenging activity of 5-bromosalicylaldehyde and schiff bases. *Med. Chem. Res.* **20**, 1431-1437 (2011); (k) Lee, S. H., Xu, L., Park, B. K., Mironov, Y. V., Kim, S. H., Song, Y. J., Kim, C., Kim, Y. & Kim, Sung-Jin. Efficient Olefin Epoxidation by Robust Re4 Cluster-Supported MnIII Complexes with Peracids: Evidence of Simultaneous Operation of Multiple Active Oxidant Species, MnV=O, MnIV=O, and MnIII-OOC(O)R. *Chem. – Eur. J.* **16**, 4678-4685, (2010).

9. (a) Bhattacharya, K., Maity, M., Abtab, Sk Md T., Majee, M. Ch. & Chaudhury, M. Homo- and Heterometal Complexes of Oxido-Metal Ions with a Triangular [V(V)O-MO-V(V)O] [M = V(IV) and Re(V)] Core: Reporting Mixed-Oxidation Oxido-Vanadium(V/IV/V) Compounds with Valence Trapped Structures. *Inorg. Chem.* **52**, 9597-9605 (2013); (b) Haak, R. M., Decortes, A., Escudero-Adan, E. C., Belmonte, M. M., Martin, E., Benet-Buchholz, J. & Kleij, A. W. Shape-Persistent Octanuclear Zinc Salen Clusters: Synthesis, Characterization, and Catalysis. *Inorg. Chem.* **50**, 7934-7936 (2011); (c) Martinez Belmonte, M., Escudero-Adan, E. C., Martin, E. & Kleij, A. W. Isolation and characterization of unusual multinuclear Schiff base complexes: rearrangements reactions and octanuclear cluster formation. *Dalton Trans.* **41**, 5193-5200 (2012); (d) Rodríguez-Dautón, M. J., Fernández, M. I., González-Noya, A. M. & Maneiro, M. Novel Manganese (III) Complexes with the Schiff Base *N,N'*-(1,2-Phenylene)-bis(3-Hydroxysalicylidenimine). *Synth. React. Inorg., Met.-Org. & Nano-Met. Chem.* **36**, 655-662 (2006).
10. Koikawa, M., Matsuda, Y. & Tokii, T. Synthesis and crystal structure of a mononuclear copper(II) complex of N,N'-bis-2,3,4-trihydroxybenzylidene-1,2-phenylendiamine. *Anal. Sci.: X-Ray Structure Analysis Online* **24**, 283-284 (2008).
11. (a) Naeimi, H. & Karshenas, A. Highly regioselective conversion of epoxides to β-hydroxy nitriles using metal(II) Schiff base complexes as new catalysts under mild conditions. *Polyhedron* **49**, 234-238 (2013); (b) Kocak, N., Sahin, M., Arslan, G. & Ucan, H. Synthesis of Crosslinked Chitosan Possessing Schiff Base and Its Use in Metal Removal. *J. Inorg. Organomet. Polym. Mat.* **22**, 166-177 (2012); (c) Mandal, S.

S., Ansari, K. I. & Grant, J. D., III. Apoptotic and antitumor activities of metallo-salenes. U.S. Pat. Appl. Publ. US 20090326061 (2009); (d) Charles, E. H., Chia, L. M. L.; Rothery, J., Watson, E. L., McInnes, E. J. L., Farley, R.D., Bridgeman, A. J., Mabbs, F. E., Rowlands, C. C. & Halcrow, M. A. Electronic structures of copper (II) colplexes of tetradentate hydroquinone-containing Schiff bases. *J. Chem. Soc., Dalton Trans.*, 2087-2095 (1999).

12. CrysAlisPro, Oxford Diffraction (2002).
13. Palatinus, L. & Chapuis, G. *J. Appl. Cryst.* **40**, 786-790 (2007).
14. Betteridge, P. W., Carruthers, J. R., Cooper, R. I., Prout, K. & Watkin, D. J. *J. Appl. Cryst.* **36**, 1487 (2003).
15. Farrugia, L. J. *J. Appl. Cryst.* **45**, 849-854 (2001).
16. Altomare, A., Cascarano, G., Giacovazzo G., Guagliardi A., Burla M. C., Polidori, G., Camalli, M. *J. Appl. Cryst.* **27**, 435 (1994).

6) NMR spectra scans

TK039 // 600 MHz, DMSO-d6

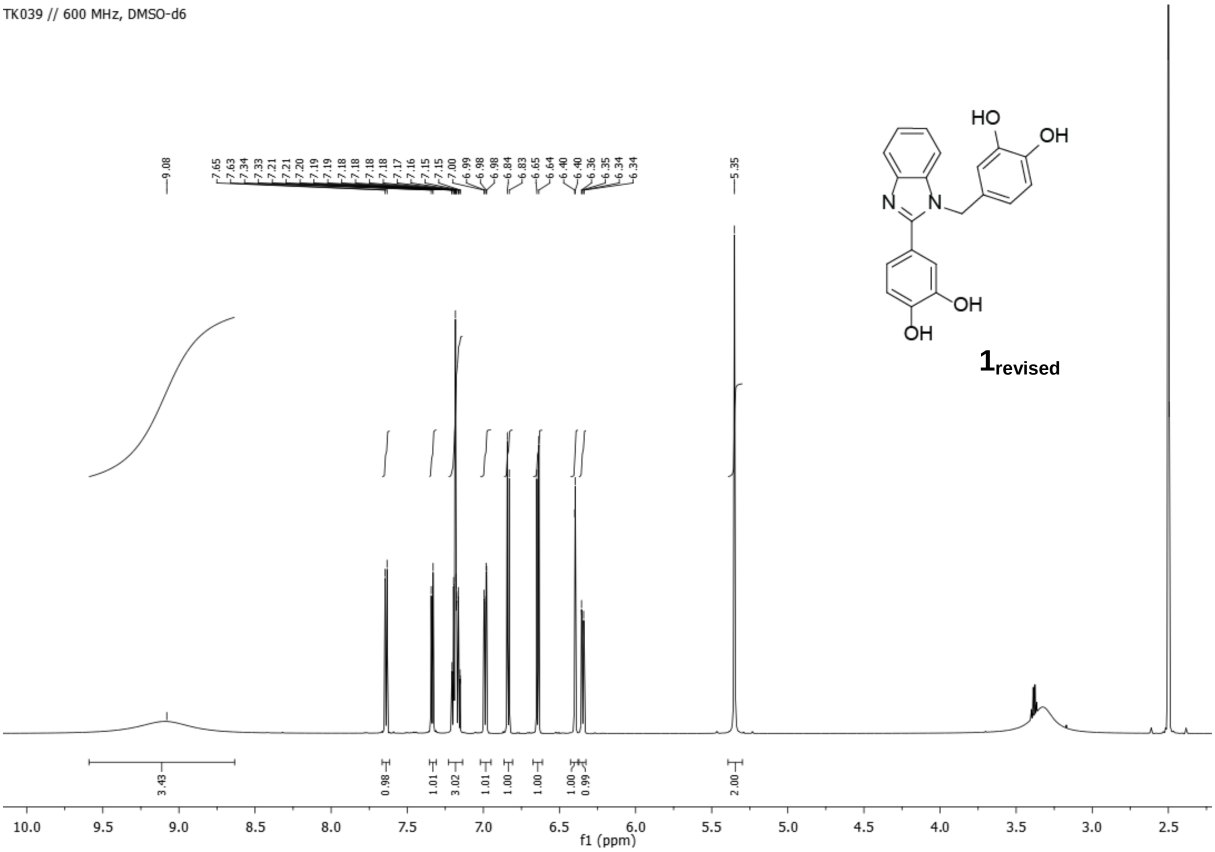

TK039// 150 MHz, DMSO- d6

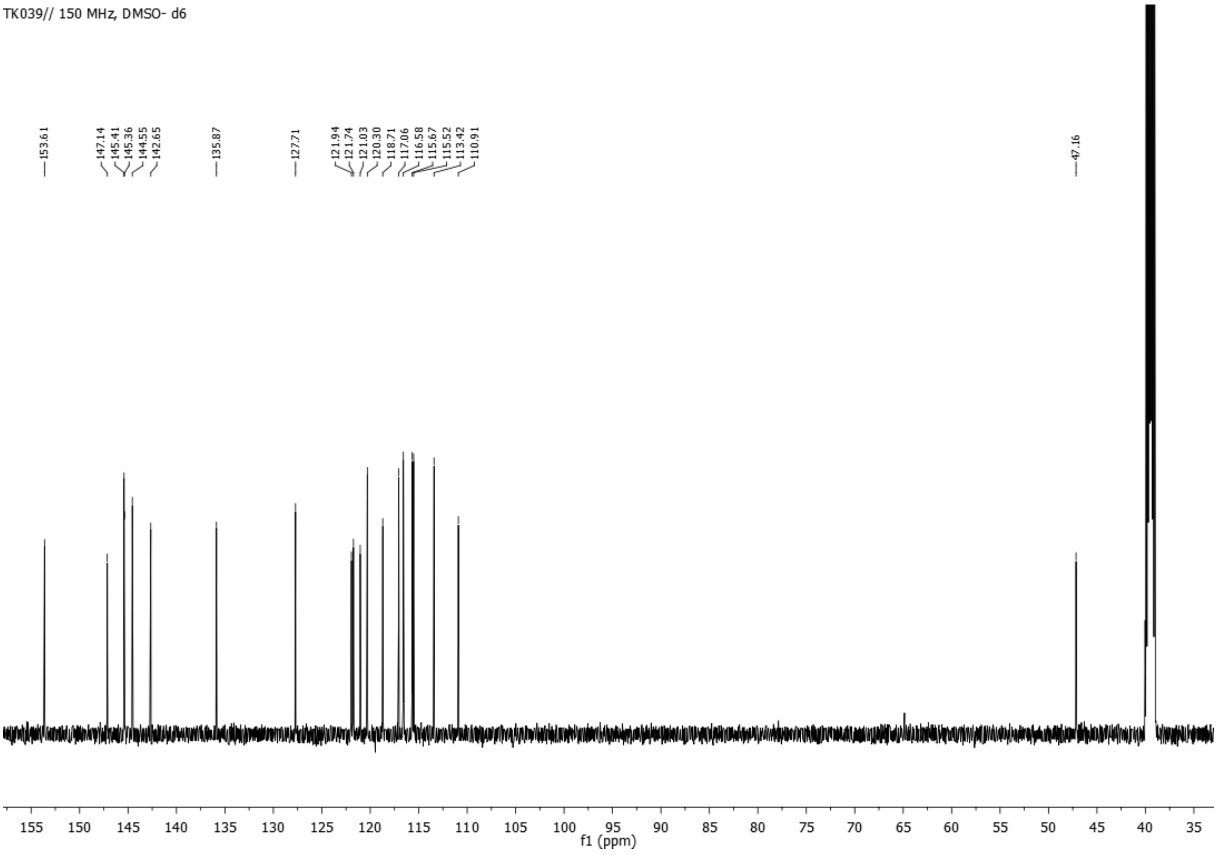

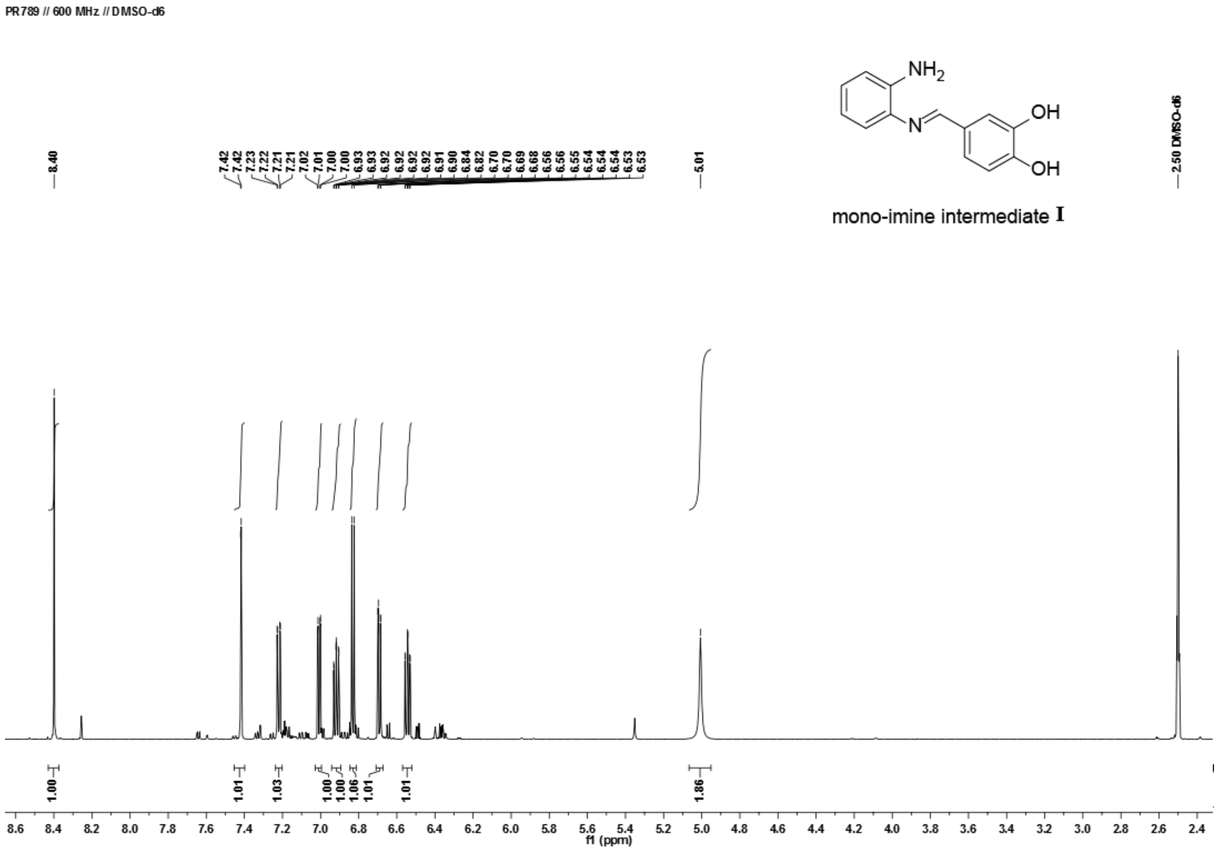
PR789 // 600 MHz // DMSO-d6
NH2
OH
N
OH
mono-imine intermediate I
8.40
7.42 7.42 7.23 7.22 7.21 7.21 7.02 7.01 7.00 7.00 6.93 6.93 6.92 6.92 6.92 6.92 6.91 6.90 6.84 6.82 6.70 6.69 6.68 6.56 6.55 6.54 6.54 6.54 6.53 6.53
5.01
2.50 DMSO-d6
1.00 1.01 1.03 1.00 1.00 1.06 1.01 1.01 1.86
f1 (ppm)

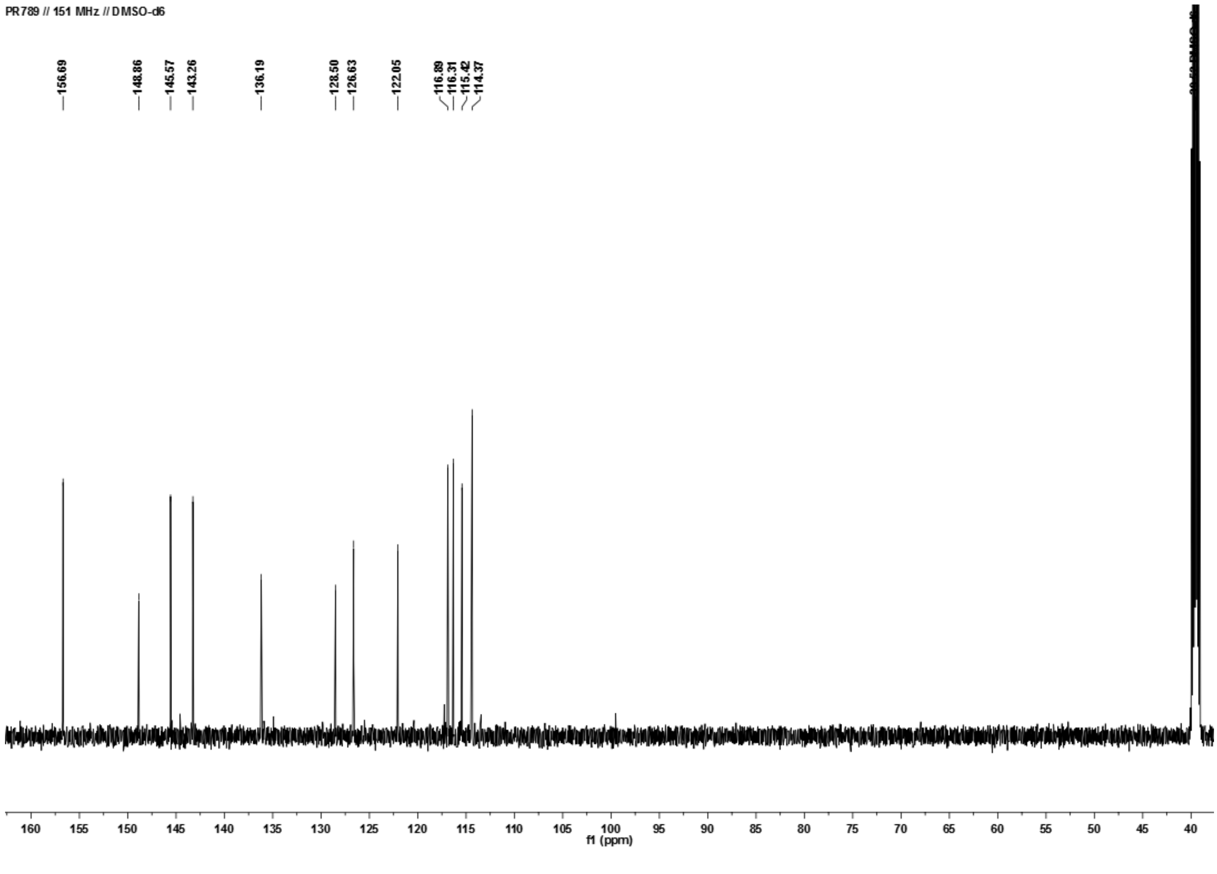
PR789 // 151 MHz // DMSO-d6
156.69
148.86
145.57
143.26
136.19
128.50
126.63
122.05
116.89 116.31 115.42 114.37
f1 (ppm)

TK060-2// 400 MHz, DMSO-d6

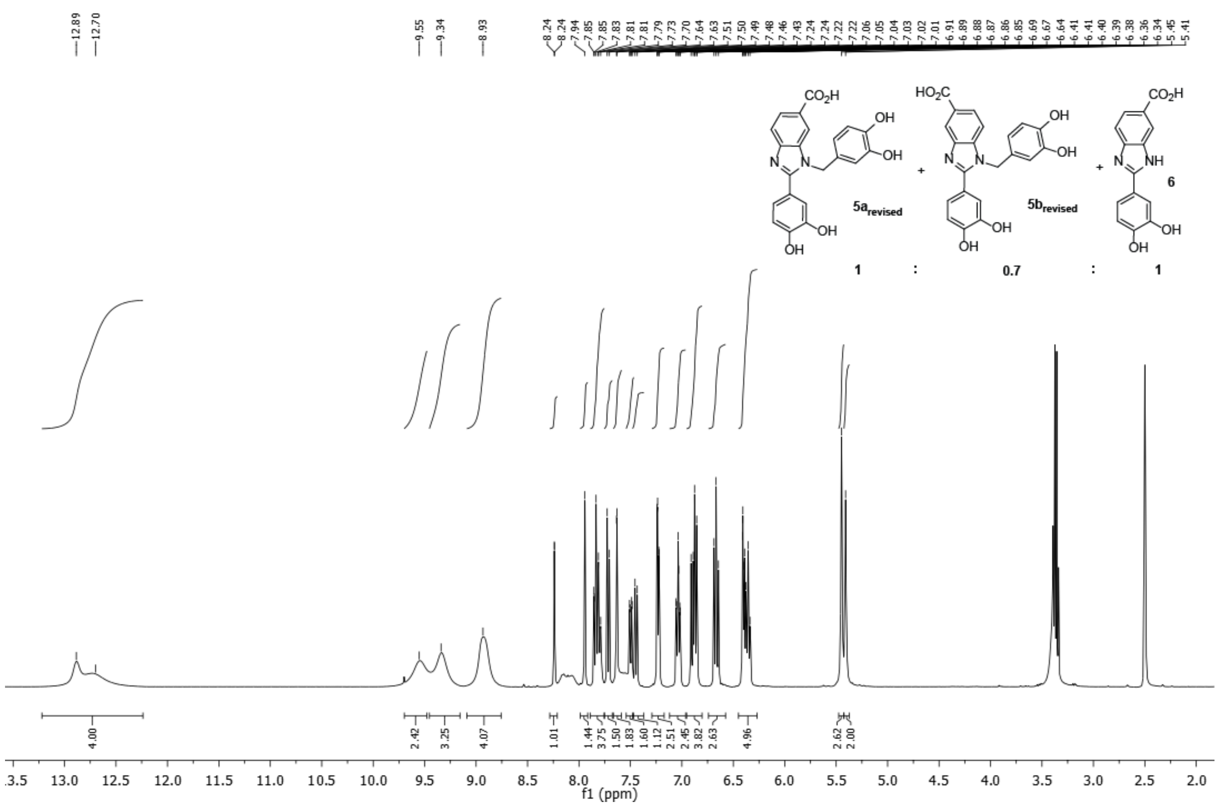

TK060-2// 100 MHz, DMSO-d6

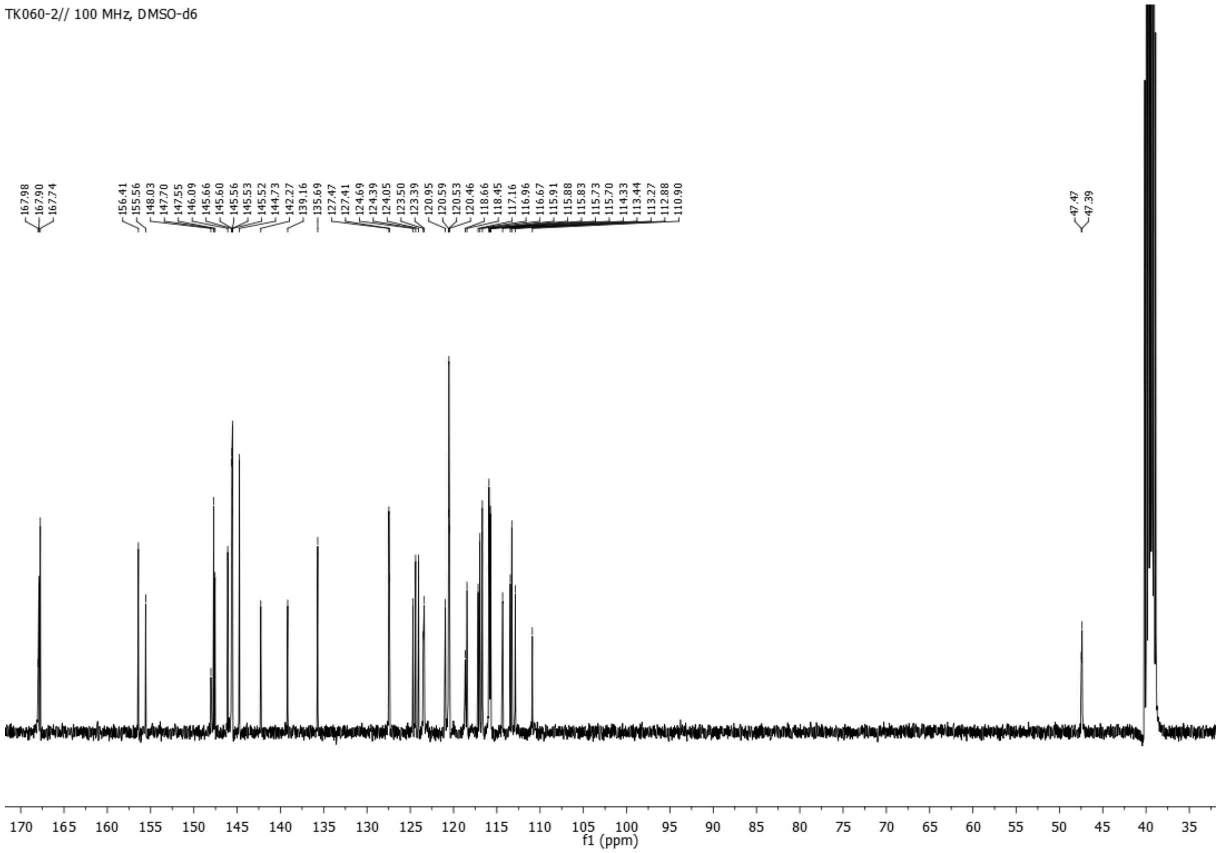

TK060-2 - HPLC fraction 1 // 600 MHz, DMSO-d6

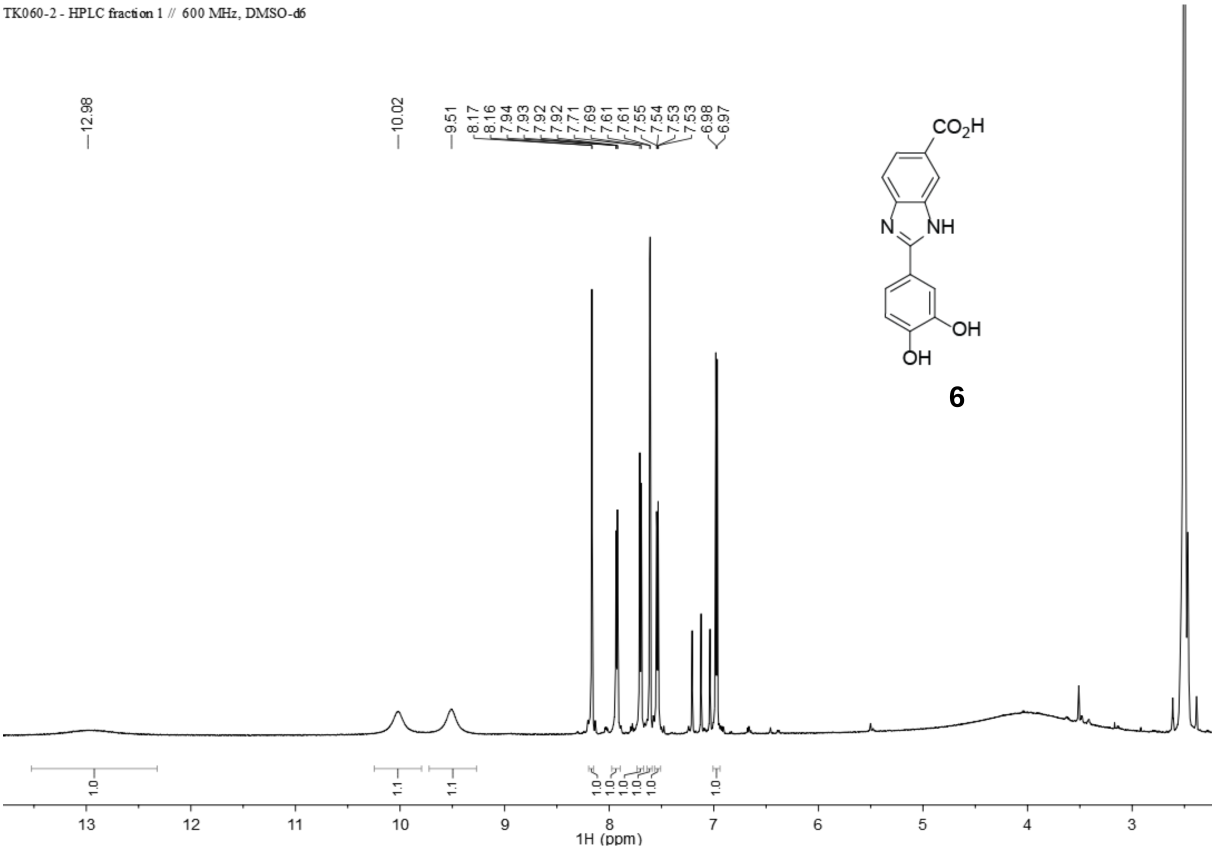

TK060-2, HPLC fraction 1 // 150 MHz, DMSO-d6

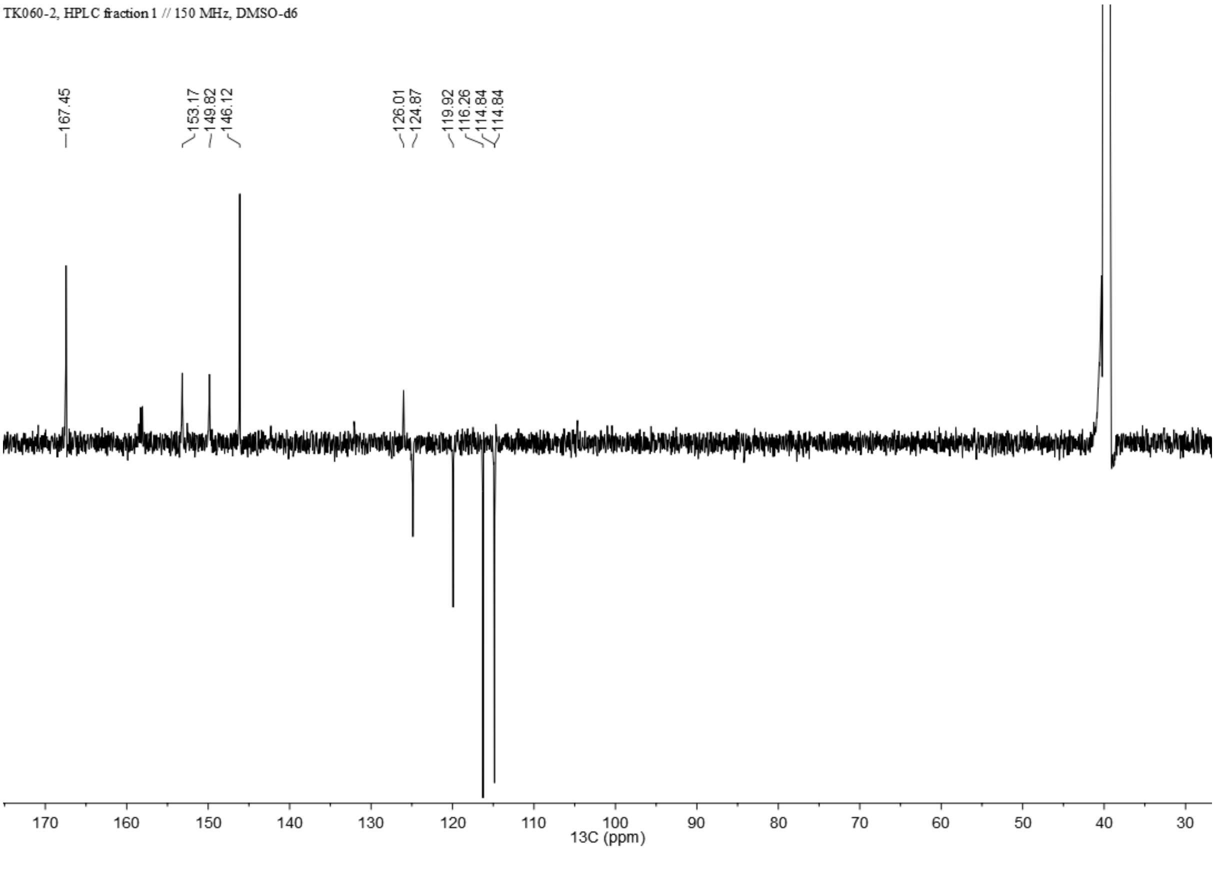

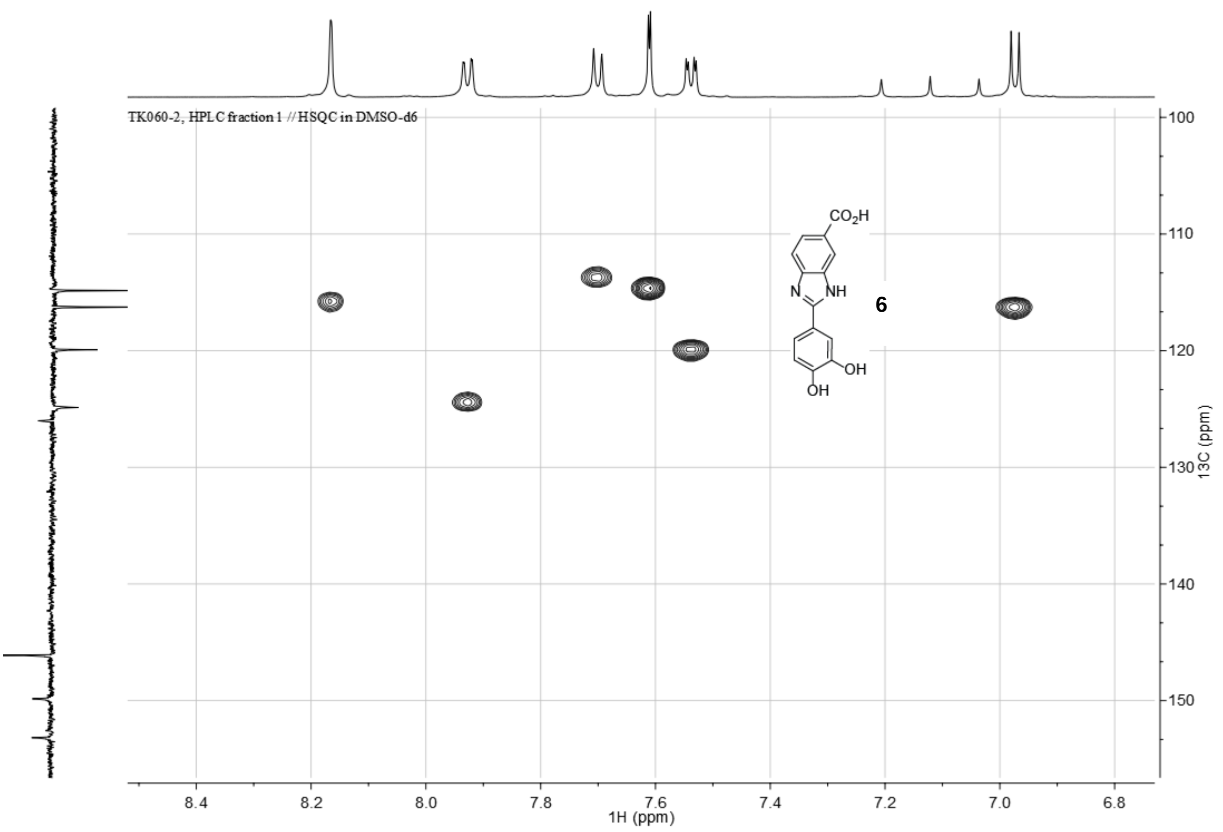
TK060-2, HPLC fraction 1 // HSQC in DMSO-d6
CO2H
N
NH
OH
OH
6
1H (ppm)
13C (ppm)

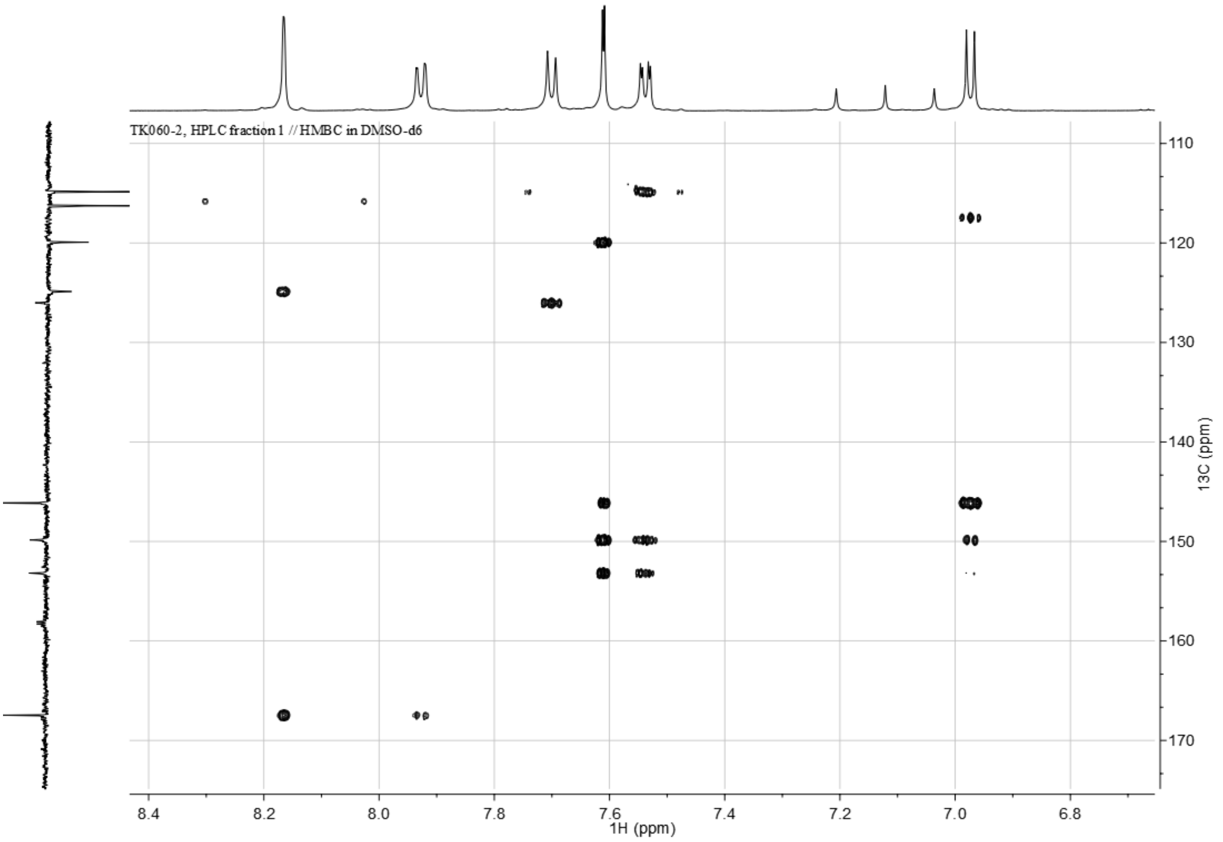
TK060-2, HPLC fraction 1 // HMBC in DMSO-d6
1H (ppm)
13C (ppm)

TK060-2, HPLC fraction 4 // 600 MHz, DMSO-d6

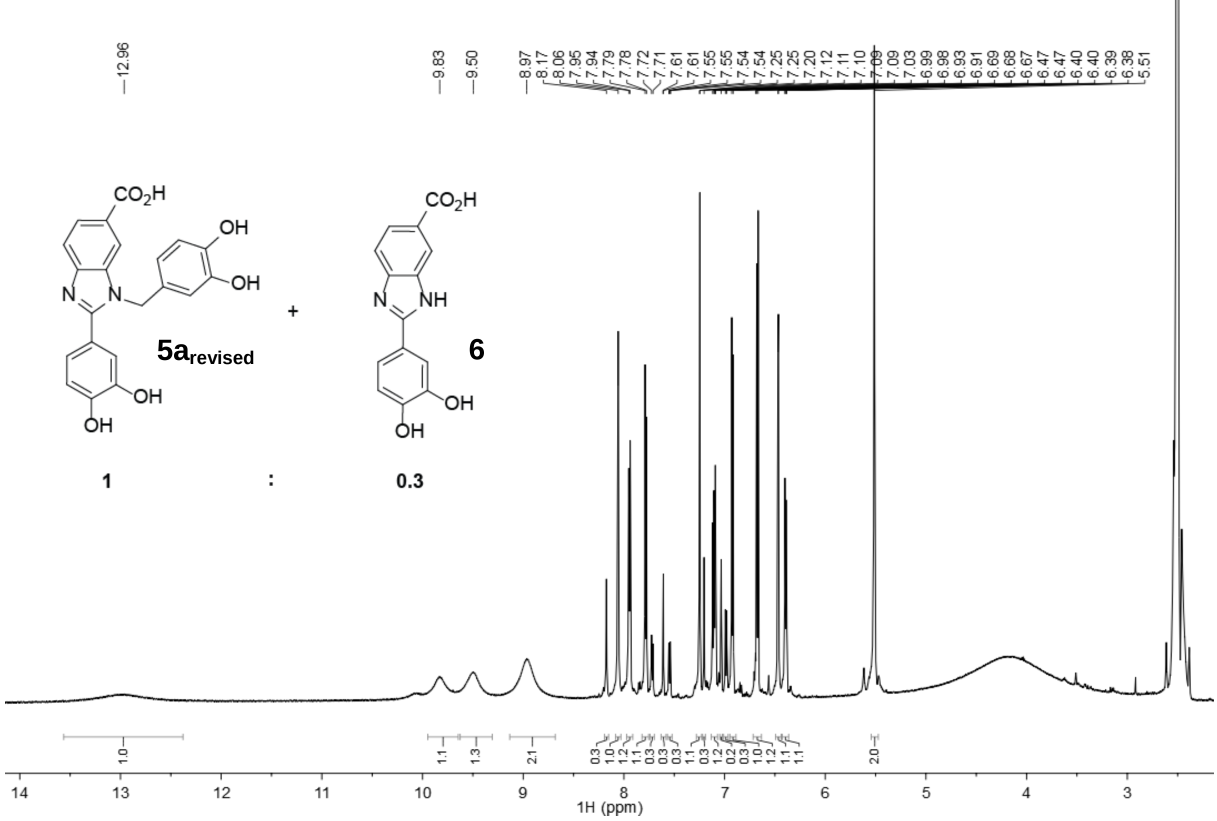

TK060-2, HPLC fraction 4 // 151 MHz, DMSO-d6

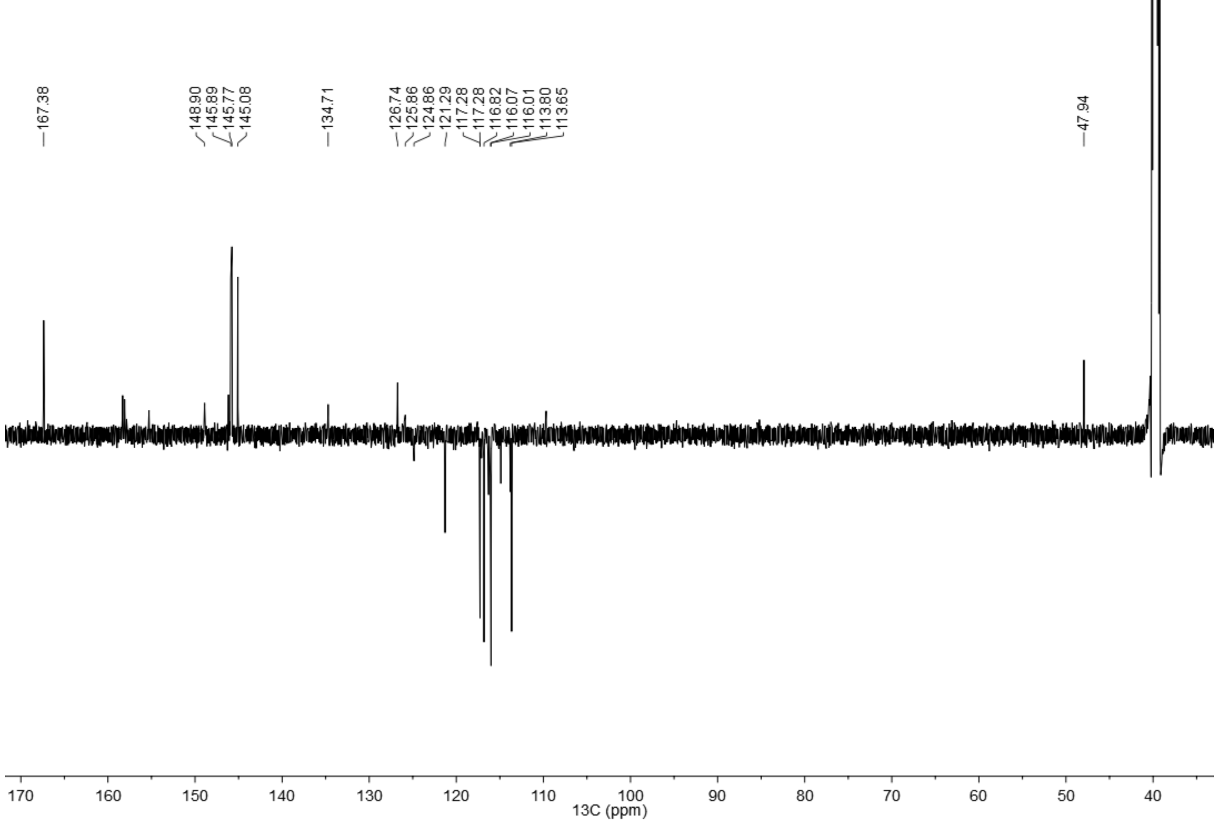

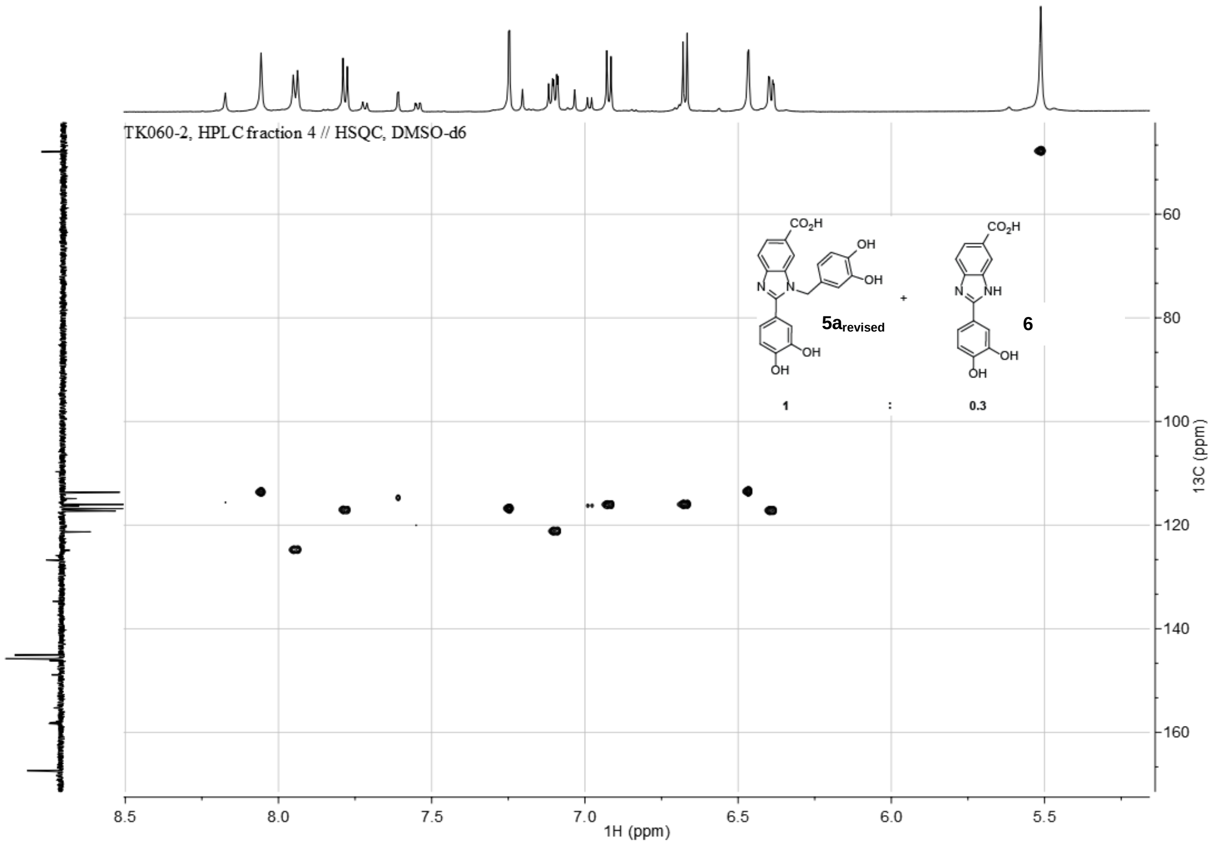
TK060-2, HPLC fraction 4 // HSQC, DMSO-d6
CO2H
OH
OH
N
N
5arevised
OH
OH
+
CO2H
N
NH
6
OH
OH
1
:
0.3
13C (ppm)
1H (ppm)

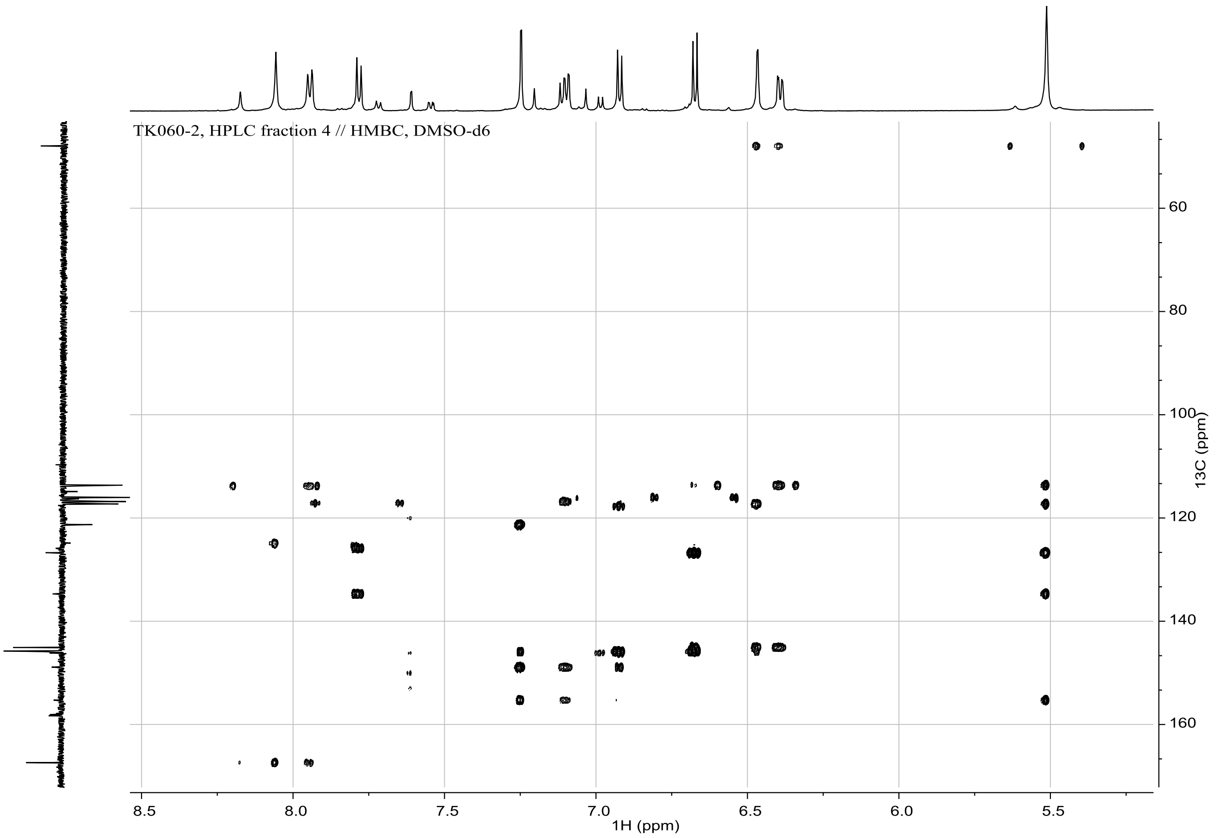
TK060-2, HPLC fraction 4 // HMBC, DMSO-d6
13C (ppm)
1H (ppm)

TK060-2, HPLC fraction 6 // 600 MHz, DMSO-d6

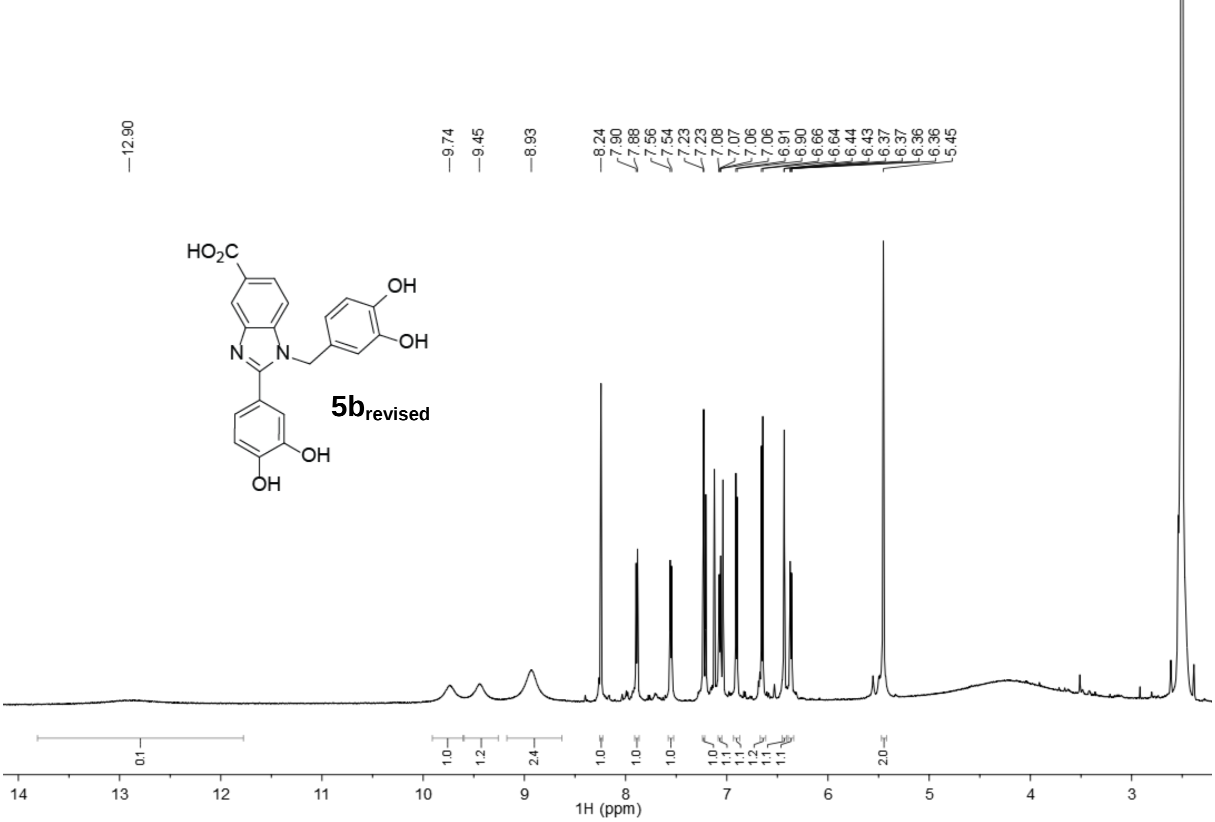

TK060-2, HPLC fraction 6 // 151 MHz, DMSO-d6

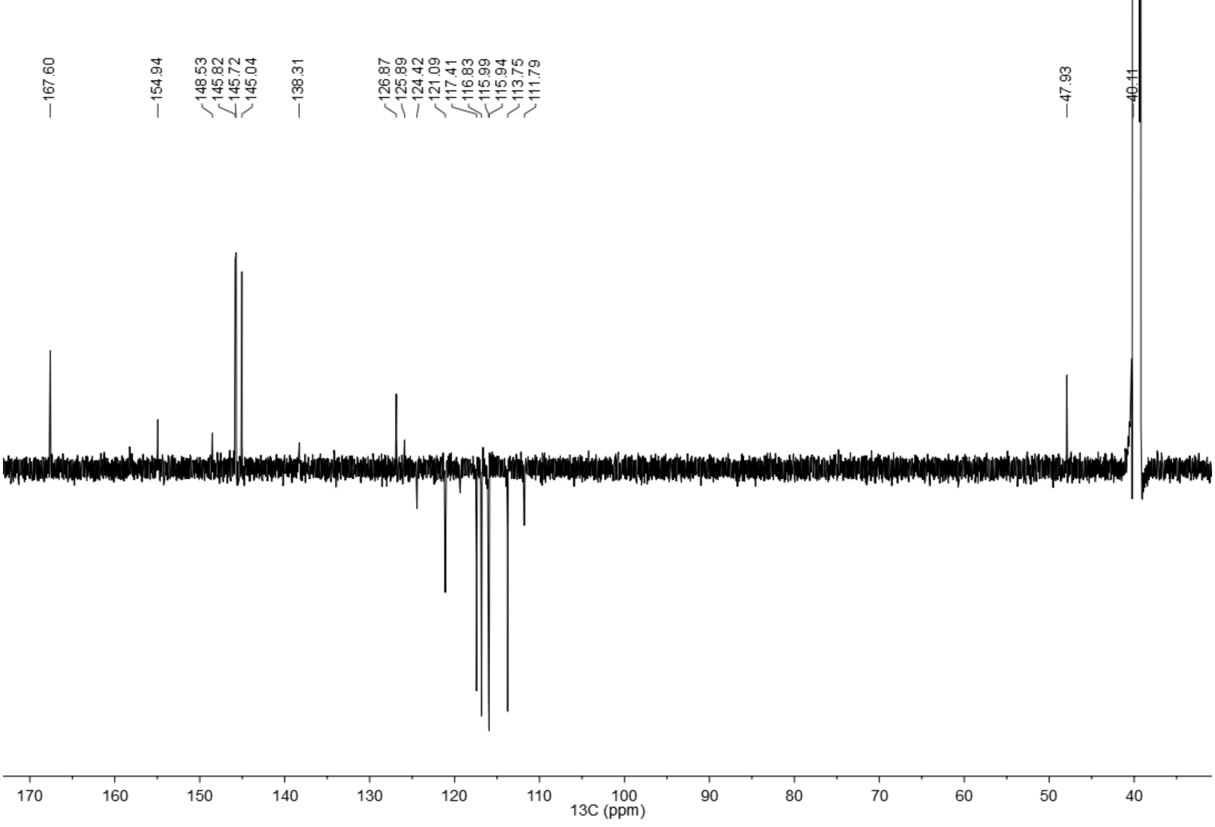

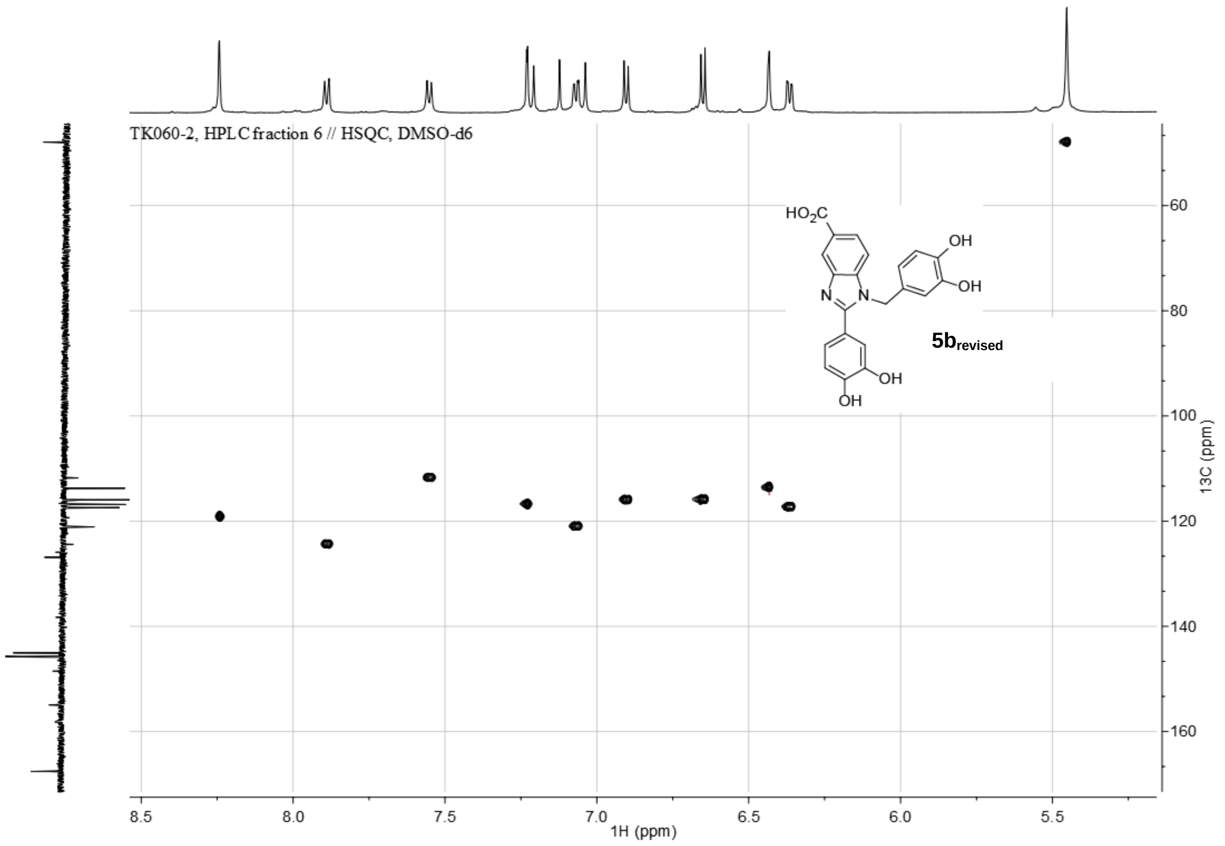
TK060-2, HPLC fraction 6 // HSQC, DMSO-d6
HO2C
OH
OH
OH
OH
N
N
5brevised
60
80
100
120
140
160
13C (ppm)
8.5
8.0
7.5
7.0
6.5
6.0
5.5
1H (ppm)

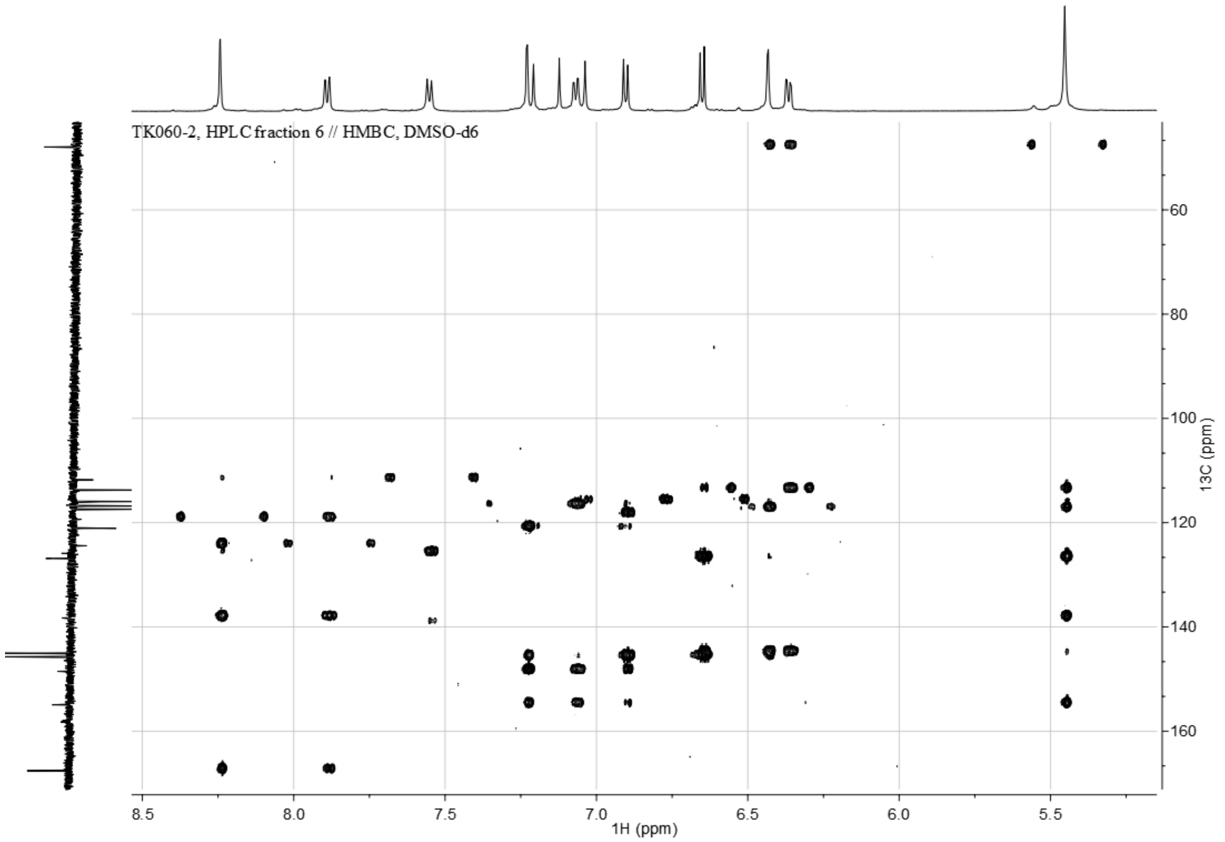
TK060-2, HPLC fraction 6 // HMBC, DMSO-d6
60
80
100
120
140
160
13C (ppm)
8.5
8.0
7.5
7.0
6.5
6.0
5.5
1H (ppm)

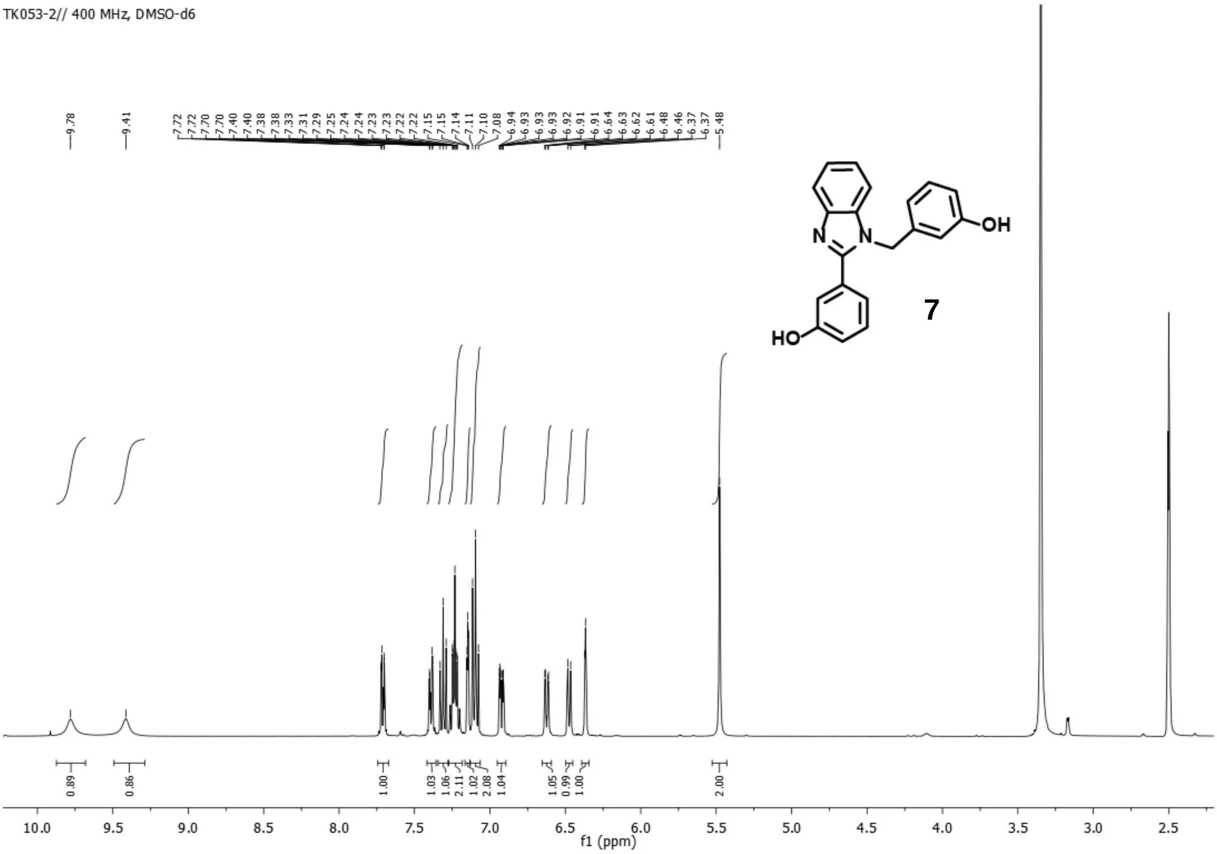
TK053-2// 400 MHz, DMSO-d6
9.78
9.41
7.72
7.72
7.70
7.70
7.40
7.40
7.38
7.38
7.33
7.31
7.29
7.25
7.24
7.24
7.23
7.23
7.23
7.22
7.22
7.22
7.15
7.15
7.14
7.11
7.10
7.08
6.94
6.93
6.93
6.93
6.92
6.91
6.91
6.64
6.63
6.62
6.61
6.48
6.46
6.37
6.37
5.48
N
N
OH
HO
7
0.89
0.86
1.00
1.03
1.06
2.11
1.02
2.08
1.04
1.05
0.99
1.00
2.00
f1 (ppm)

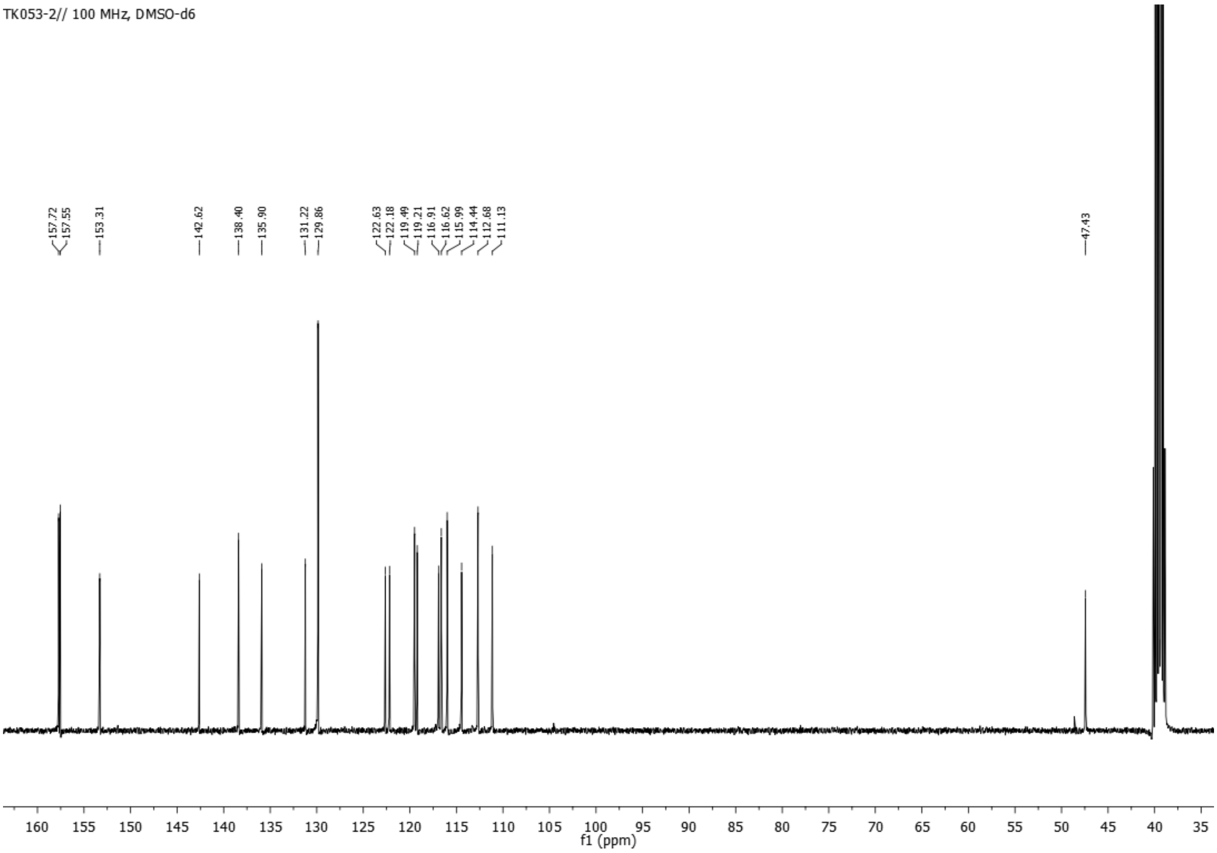
TK053-2// 100 MHz, DMSO-d6
157.72
157.55
153.31
142.62
138.40
135.90
131.22
129.86
122.63
122.18
119.49
119.21
116.91
116.62
115.99
114.44
112.68
111.13
47.43
f1 (ppm)

TK053-3// 400 MHz, DMSO-d6

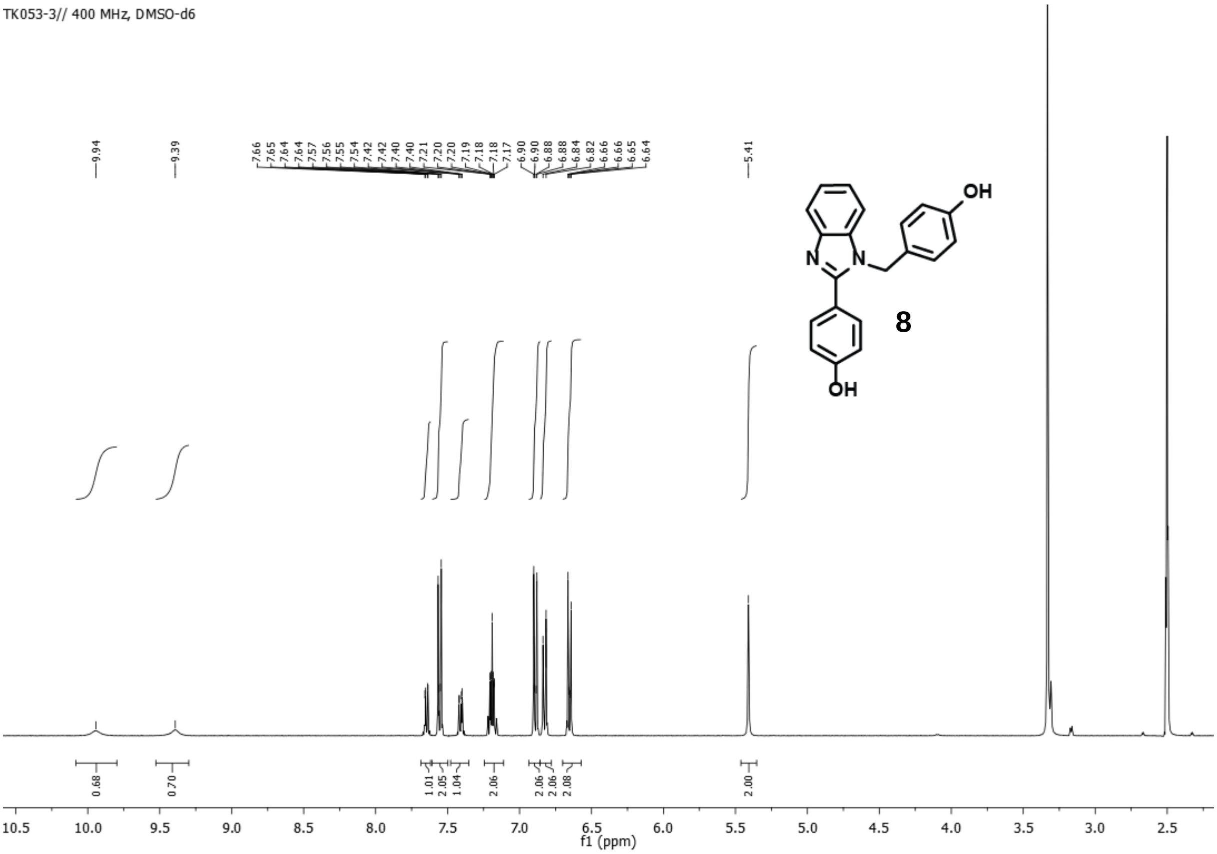

8

TK053-3// 100 MHz, DMSO-d6

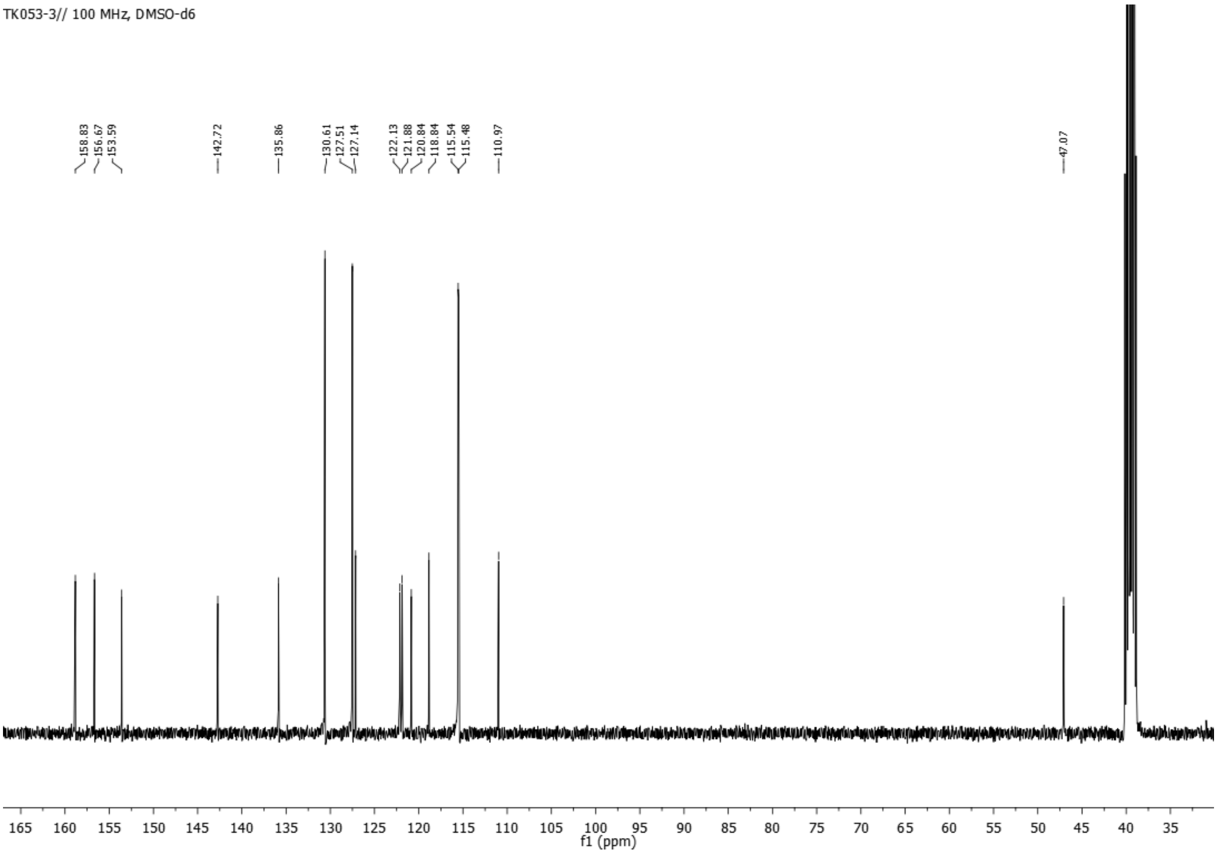

TK058-1// 400 MHz, DMSO-d6

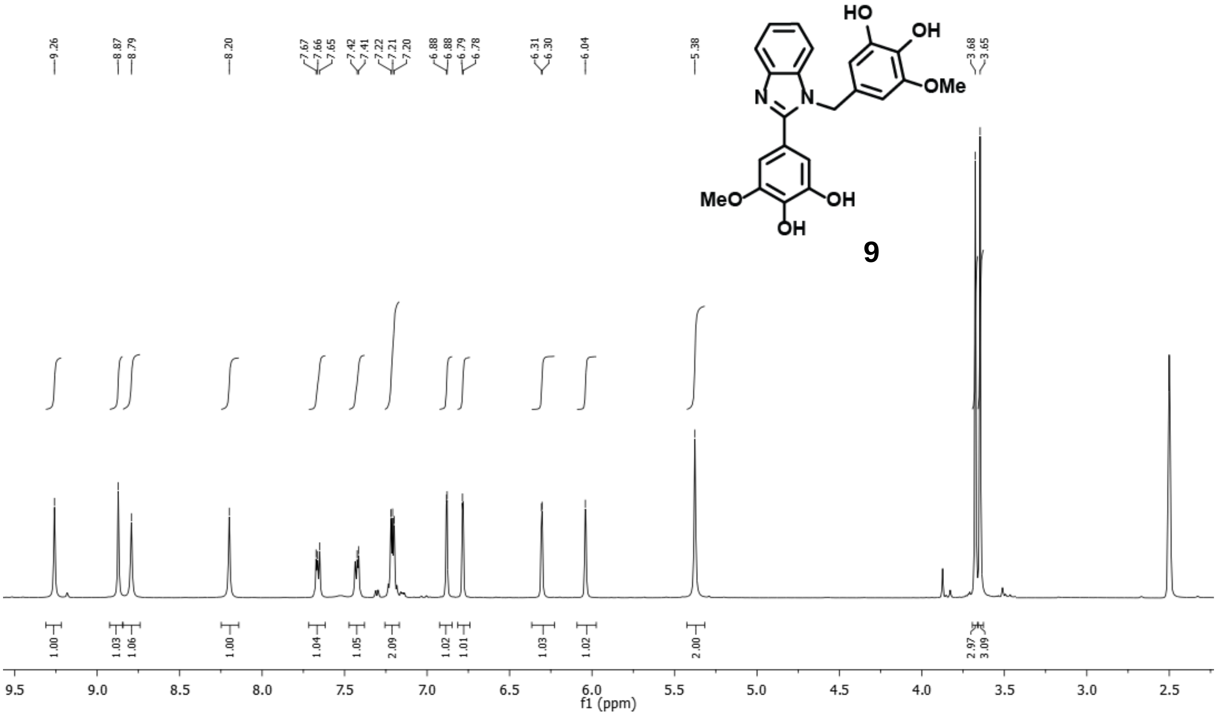

TK058-1// 100 MHz, DMSO-d6

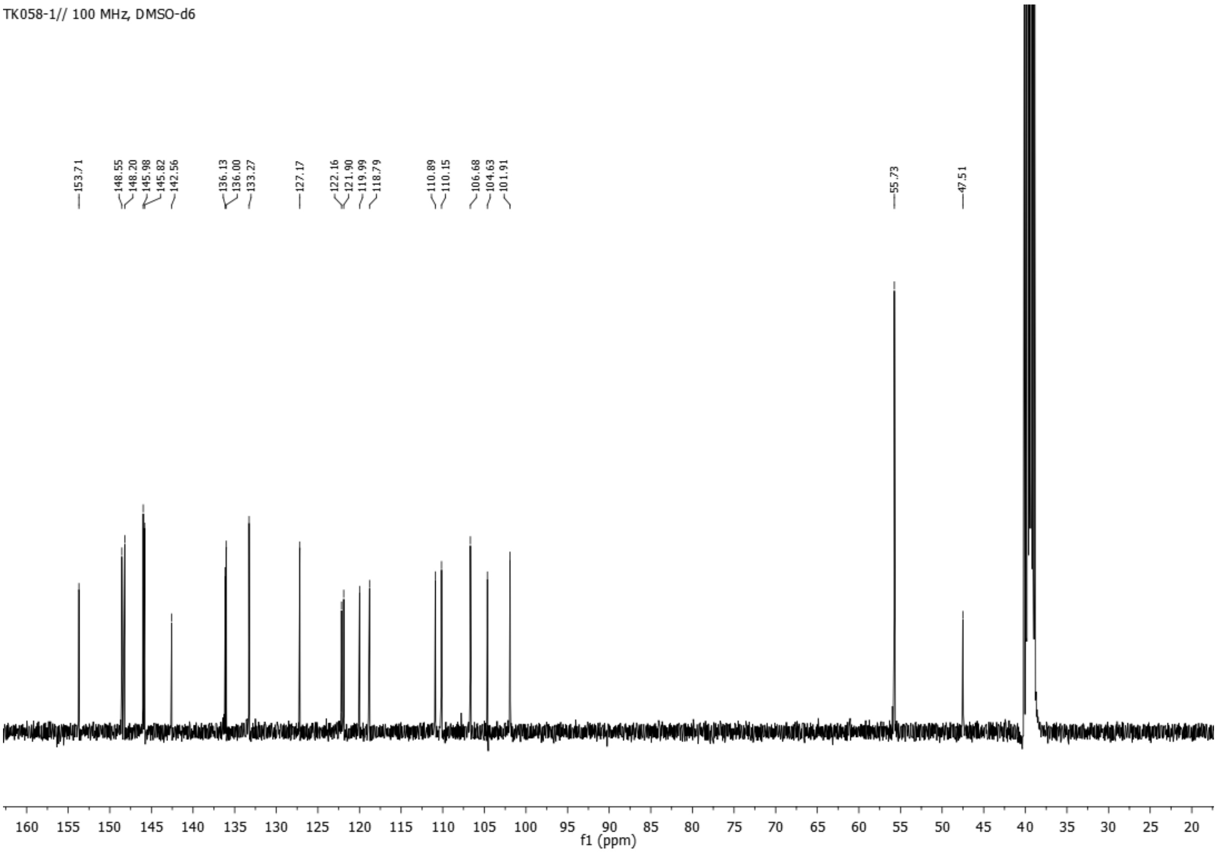

PR563-2// 400 MHz, DMSO-d6

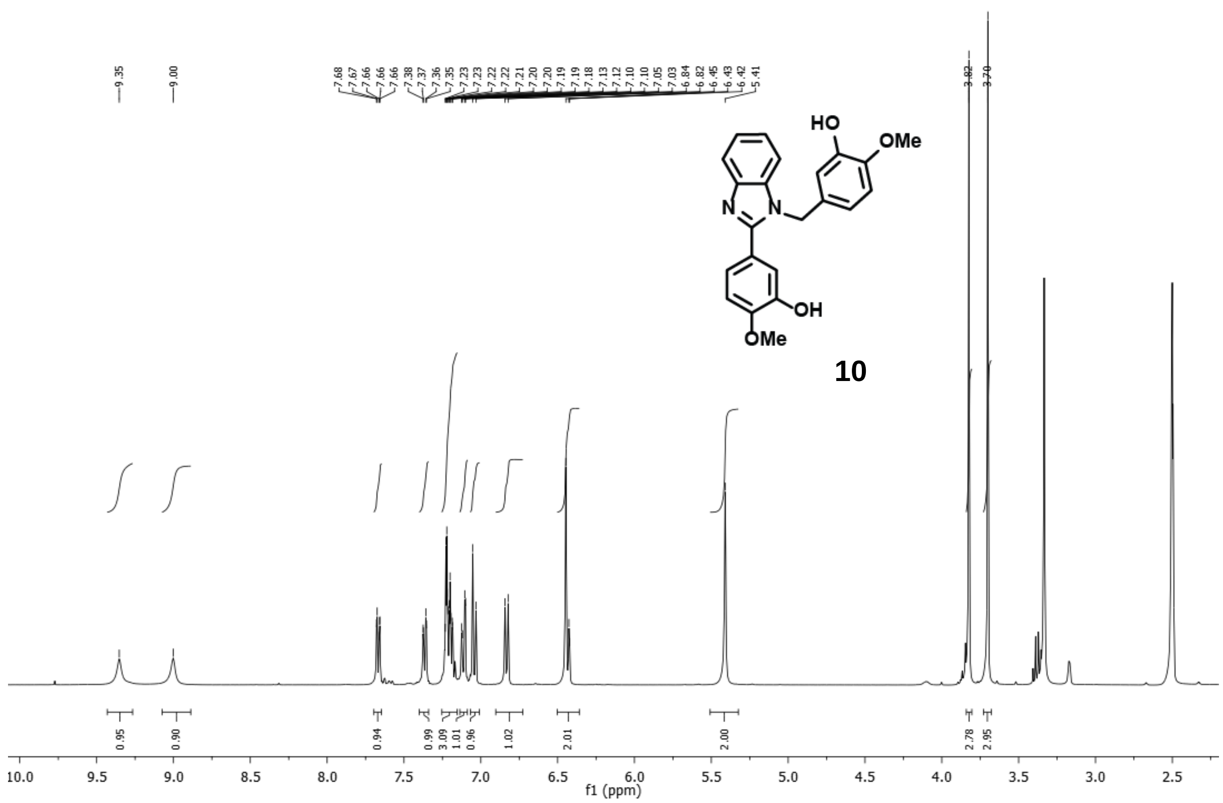

PR563-2// 100 MHz, DMSO-d6

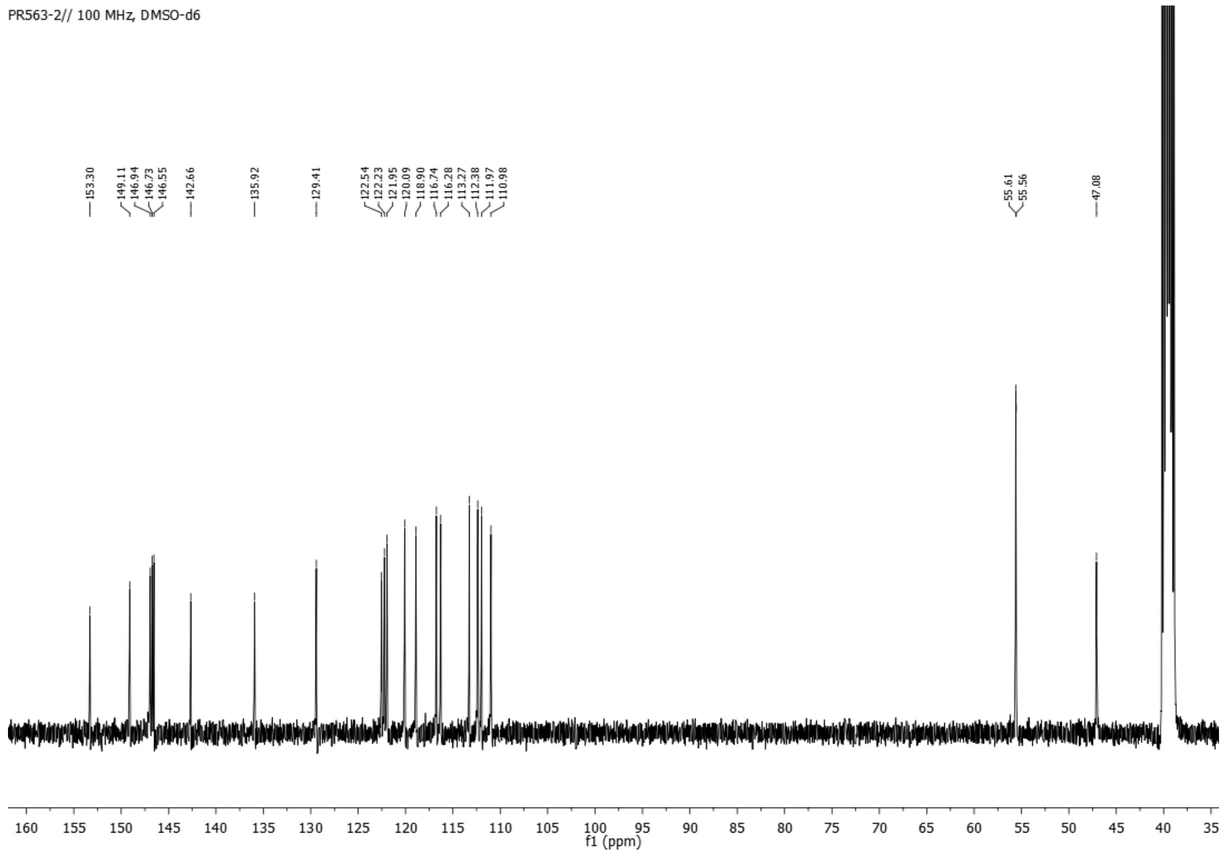

PR563-1// 400 MHz, DMSO-d6

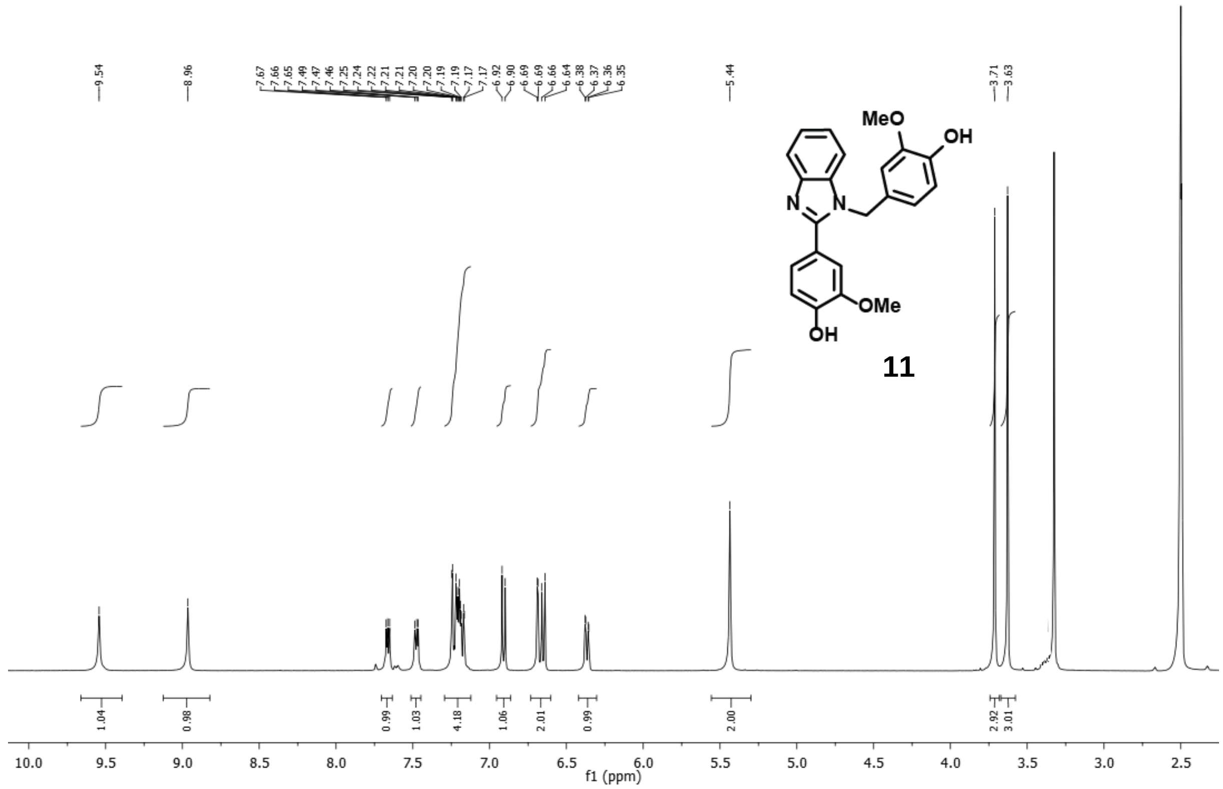

PR563-1// 100 MHz, DMSO-d6

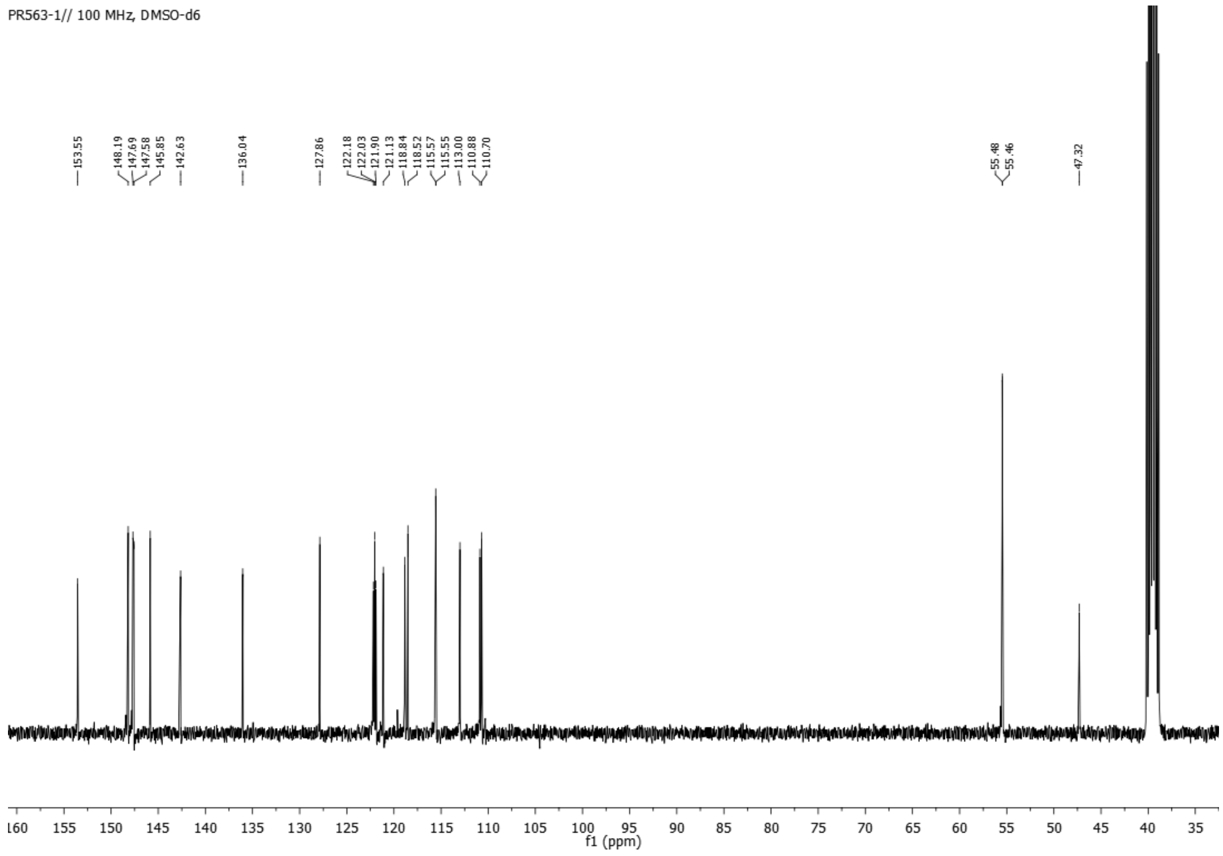

TK054-2// 400 MHz, DMSO-d6

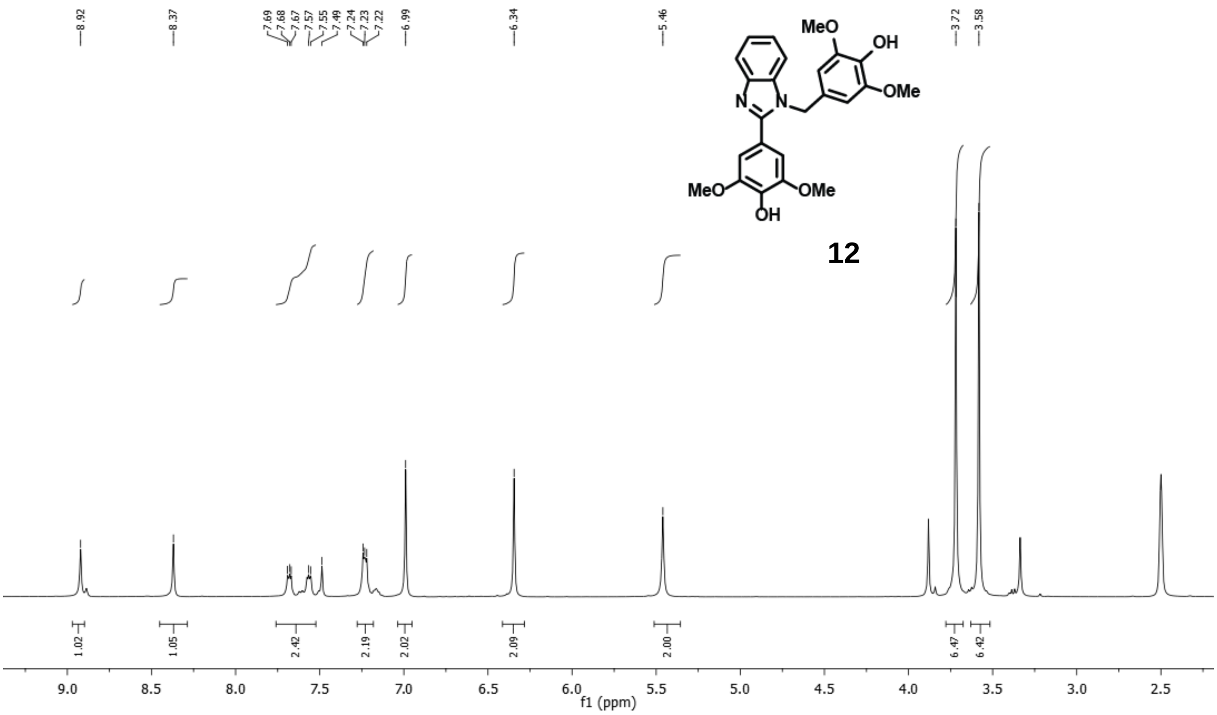

12

TK054-2// 100 MHz, DMS0-d6

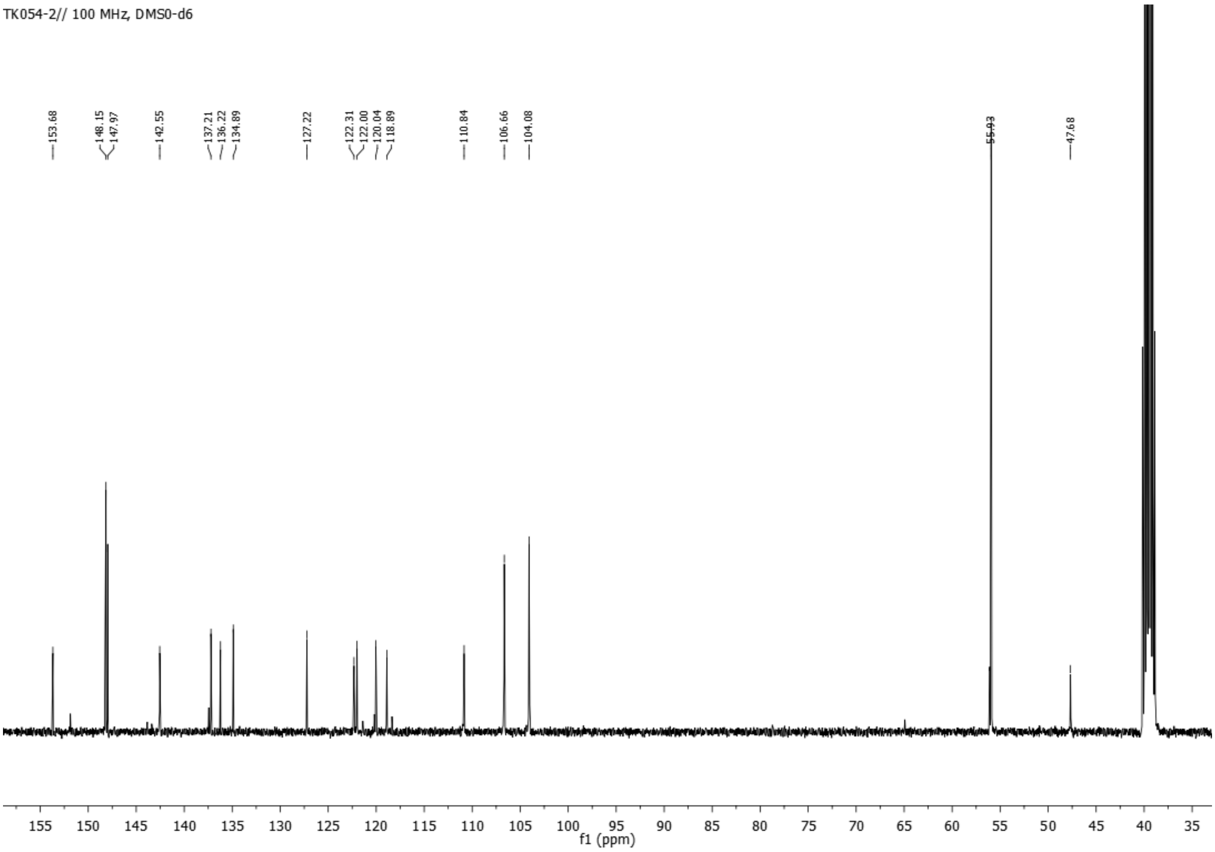

PR562-2// 400 MHz, DMS0-d6

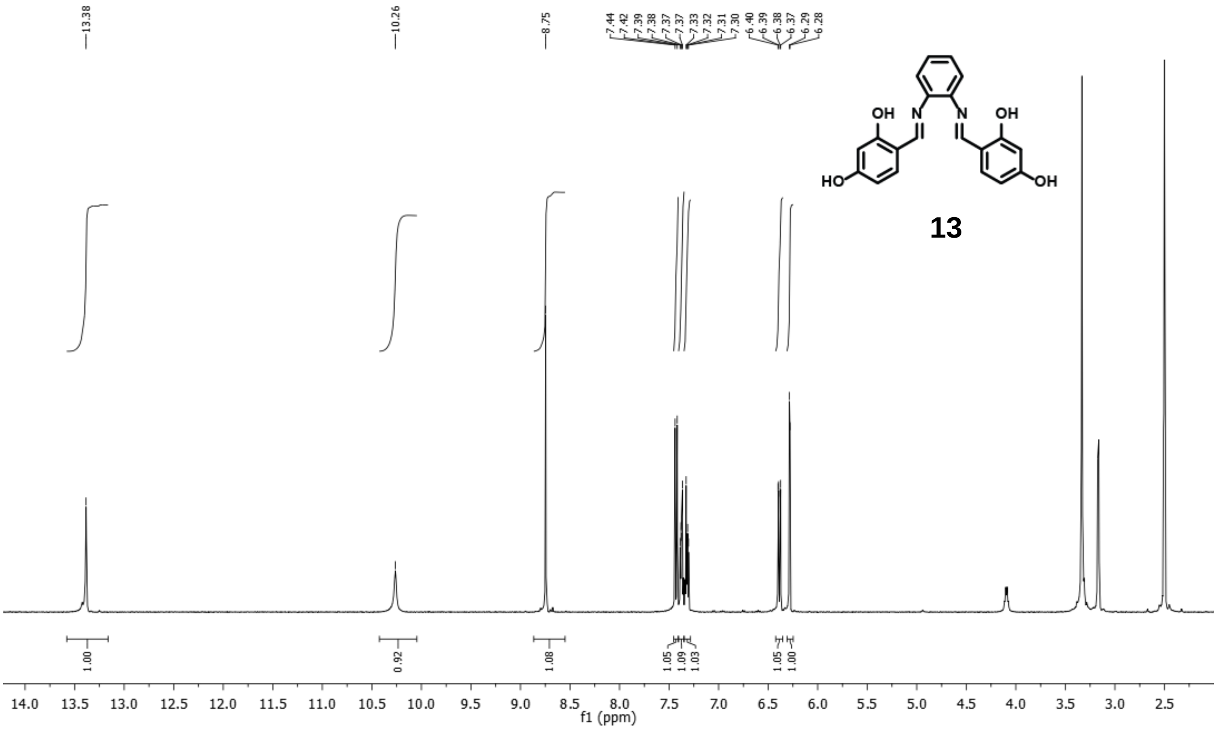

PR562-2// 100 MHz, DMSO-d6

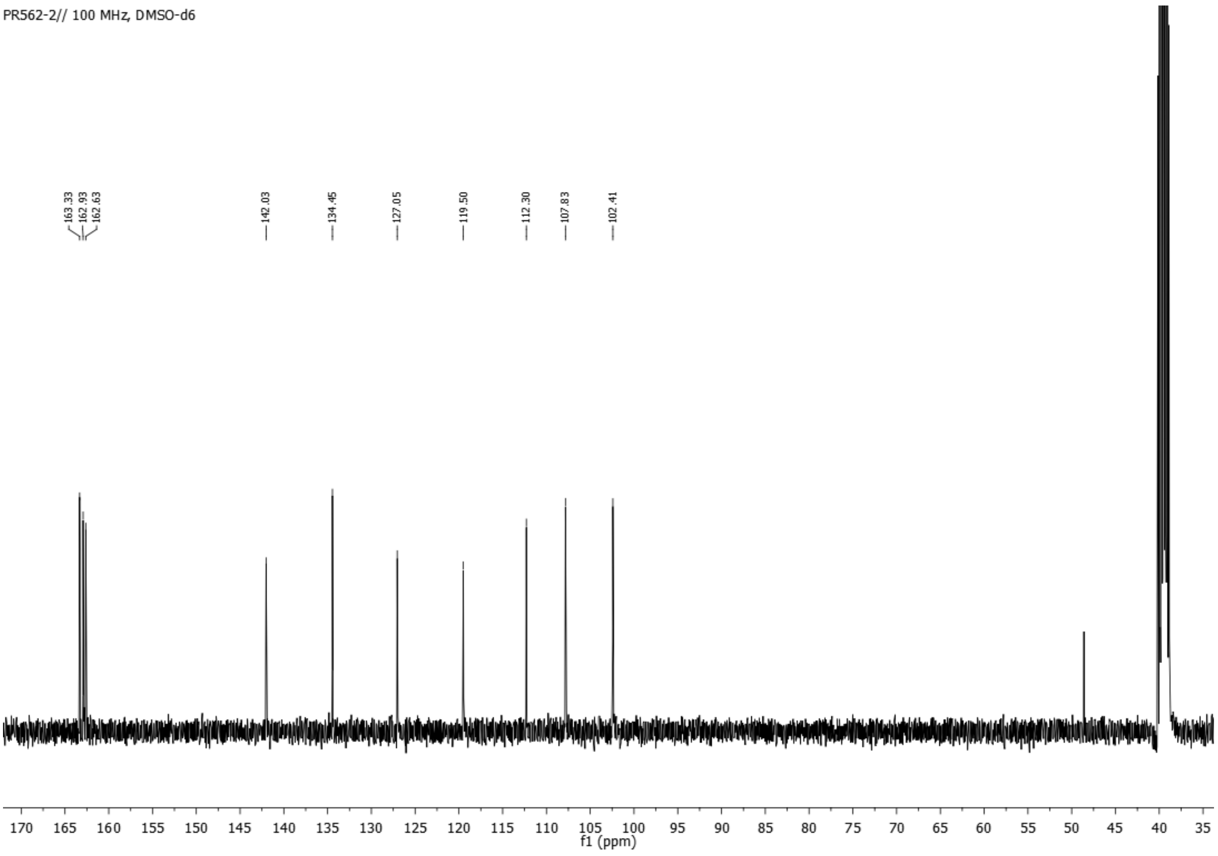

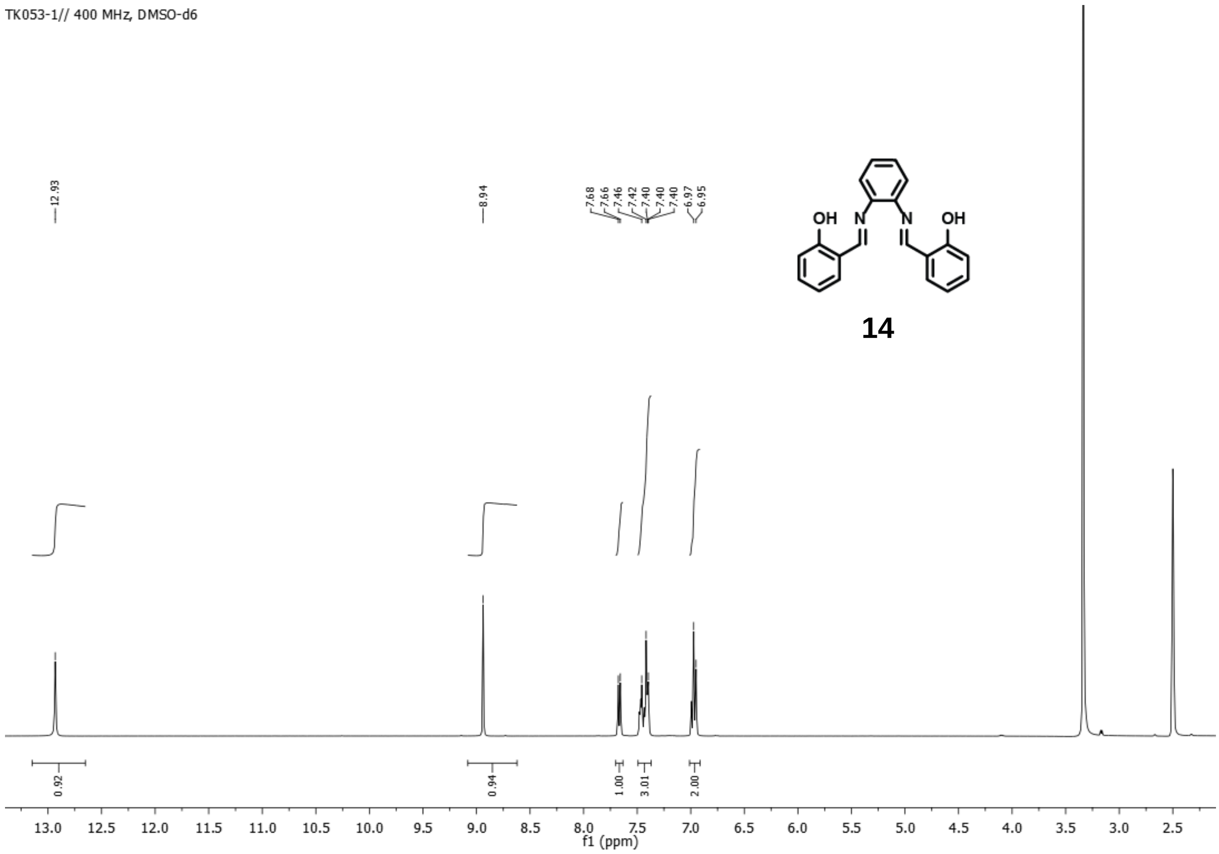
TK053-1// 400 MHz, DMSO-d6
12.93
8.94
7.68
7.66
7.46
7.42
7.40
7.40
7.40
6.97
6.95
OH
N
N
OH
14
0.92
0.94
1.00
3.01
2.00
f1 (ppm)

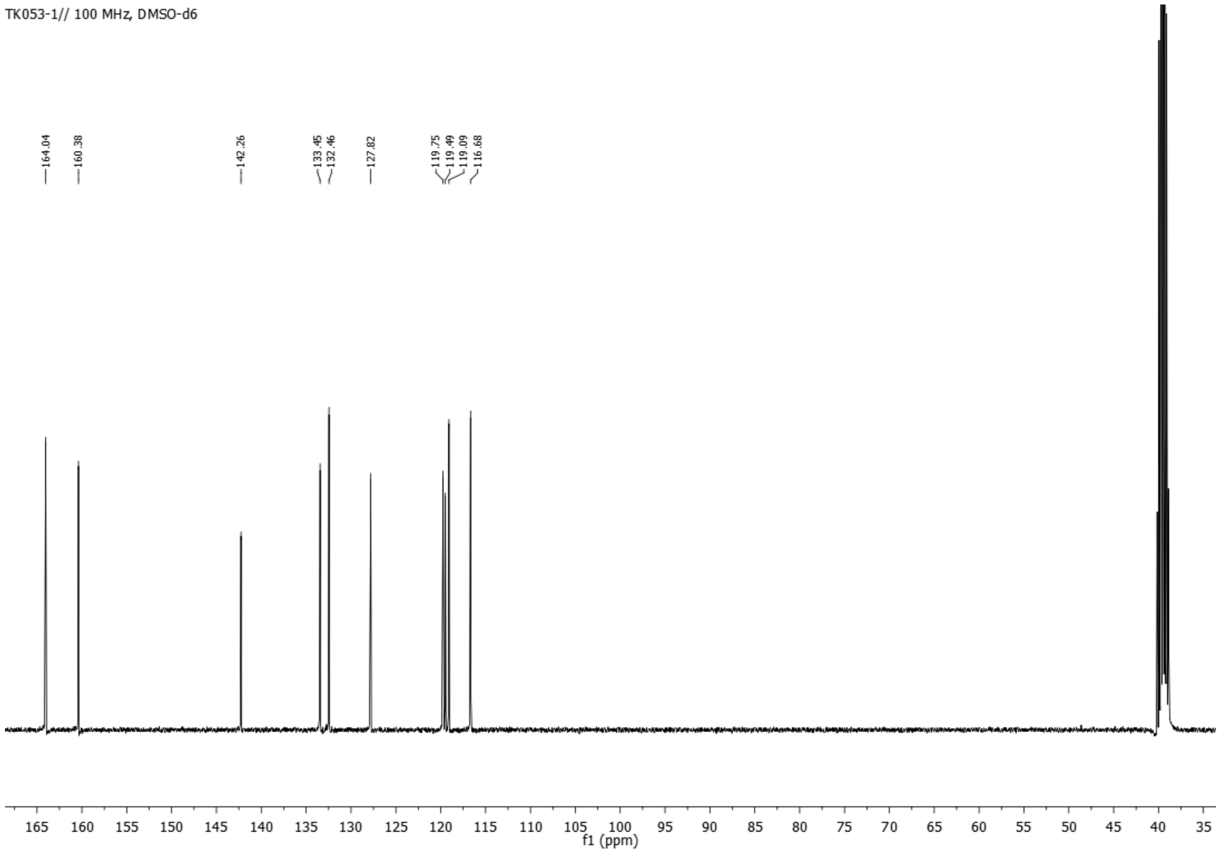
TK053-1// 100 MHz, DMSO-d6
164.04
160.38
142.26
133.45
132.46
127.82
119.75
119.49
119.09
116.68
f1 (ppm)

PR562-3// 400 MHz, DMSO-d6

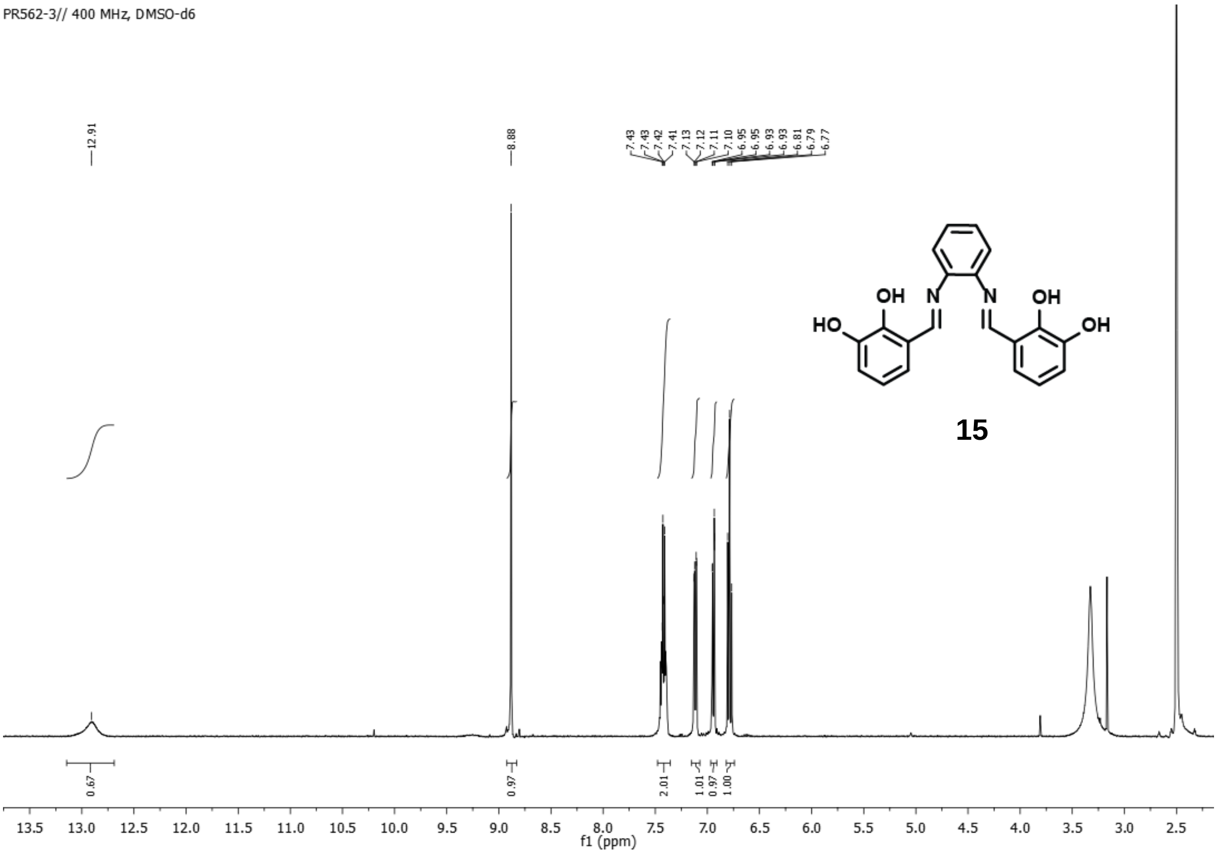

PR562-3// 100 MHz, DMSO-d6

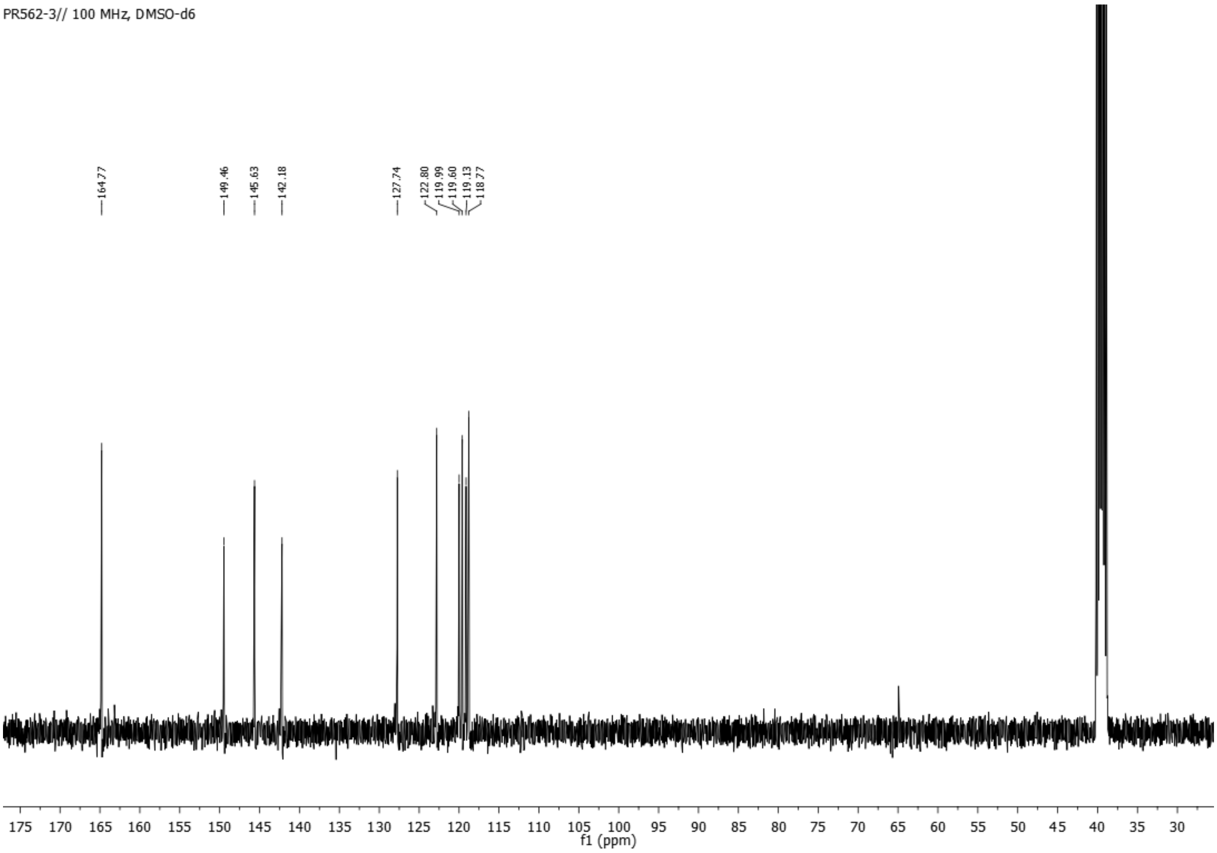

TK054-1// 400 MHz, DMSO-d6

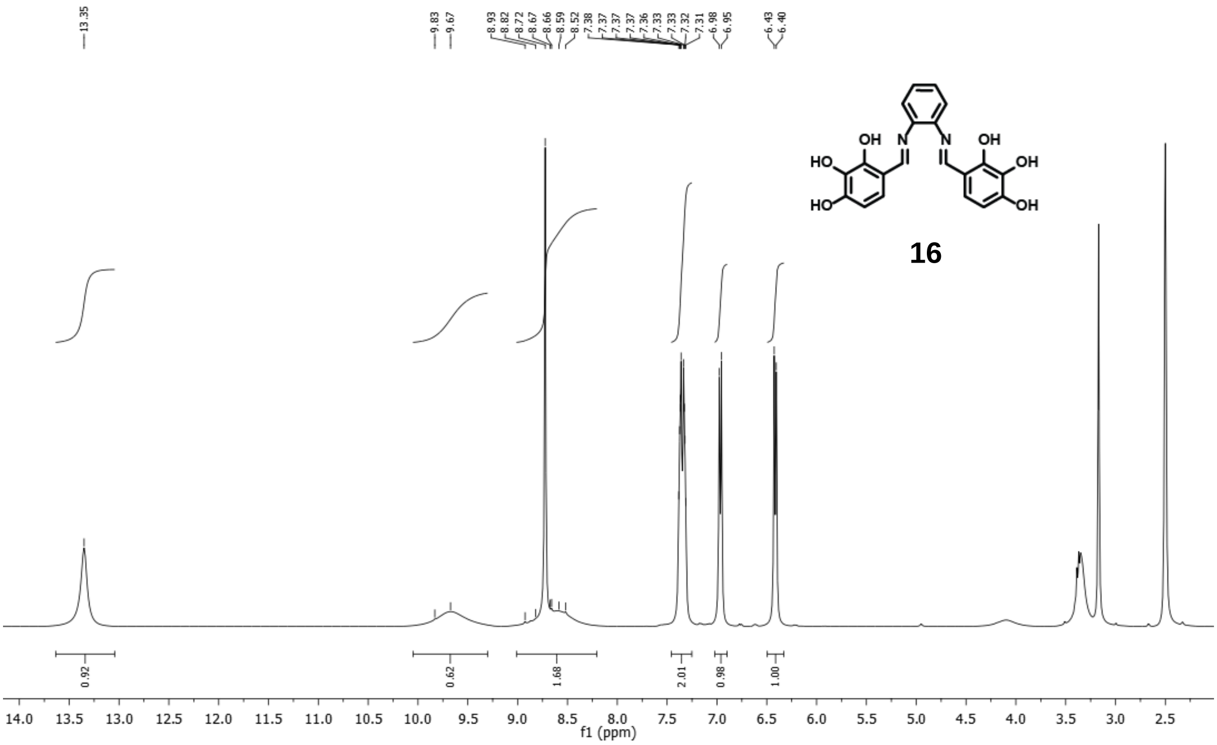

TK054-1// 100 MHz, DMSO-d6

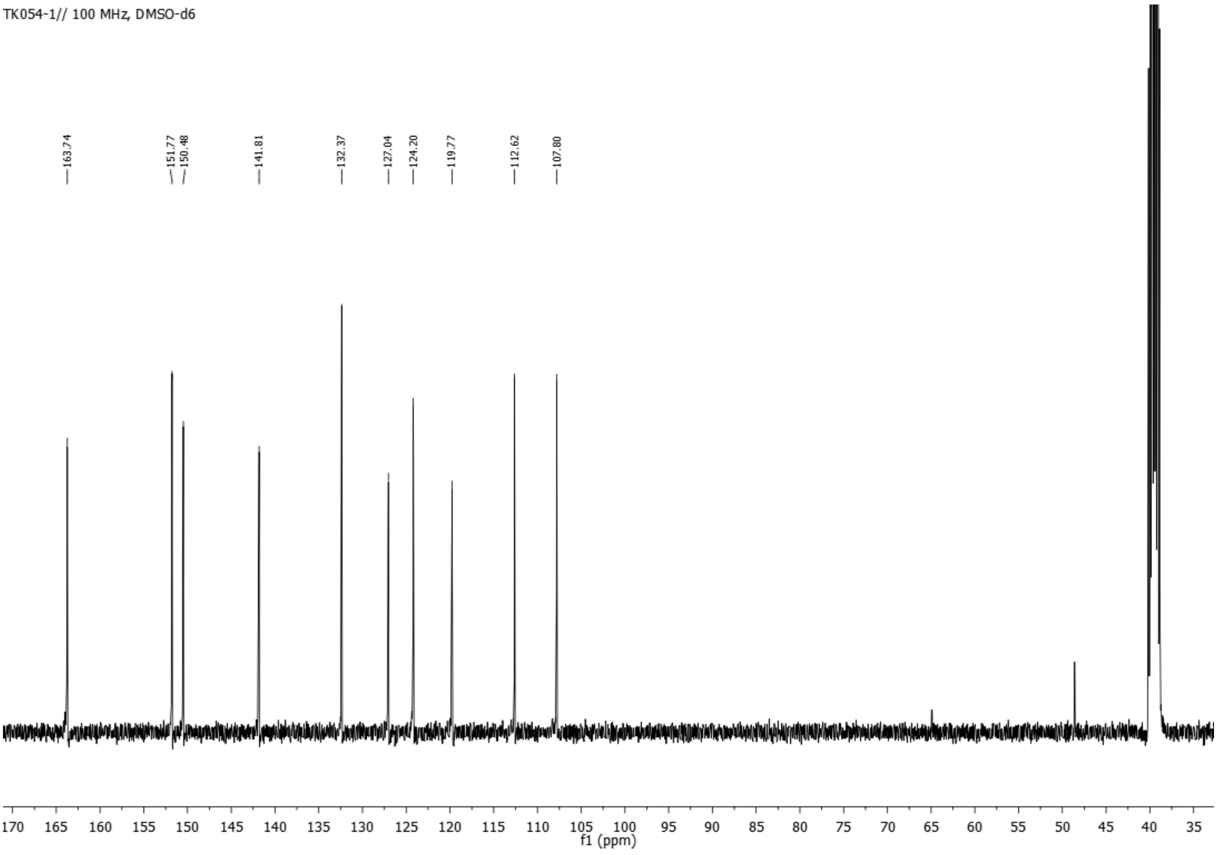

PR569-1// 400MHz, DMSO-d6

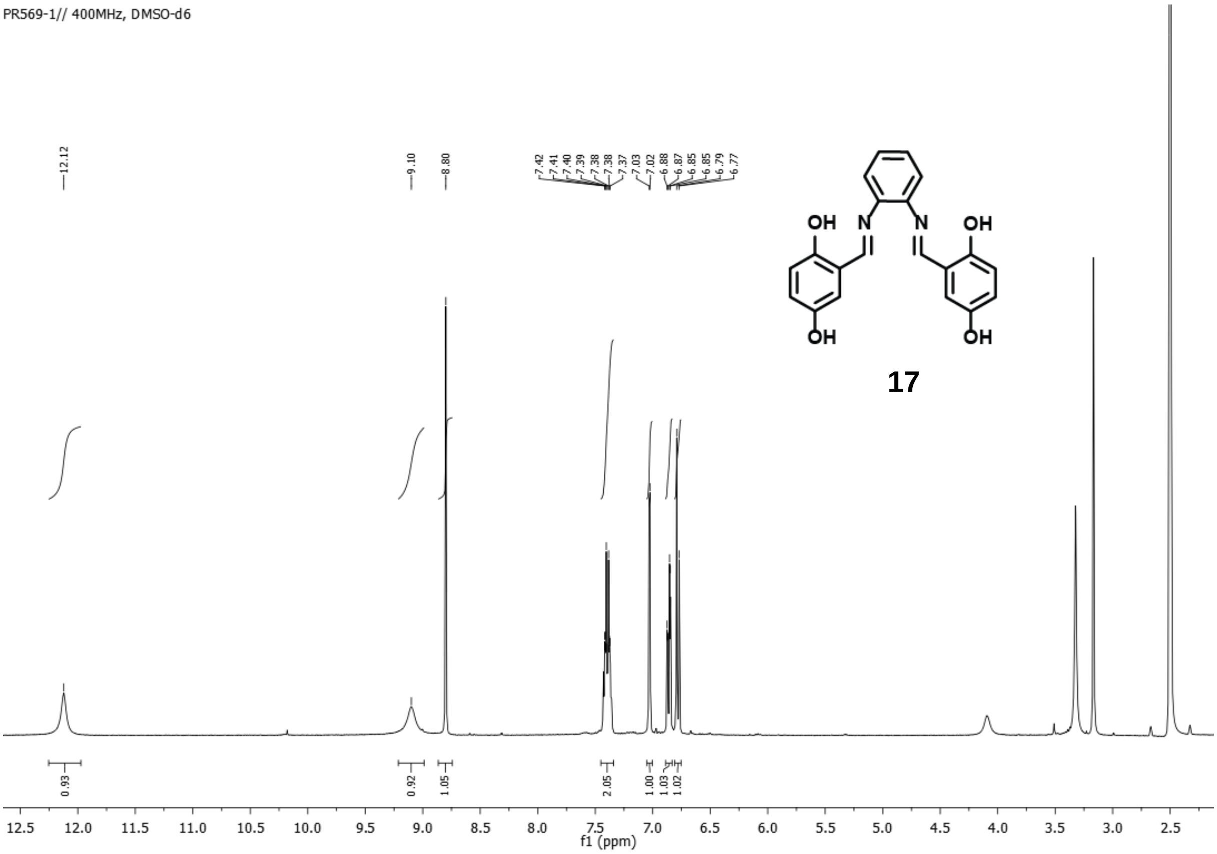

PR569-1// 100 MHz, DMSO-d6

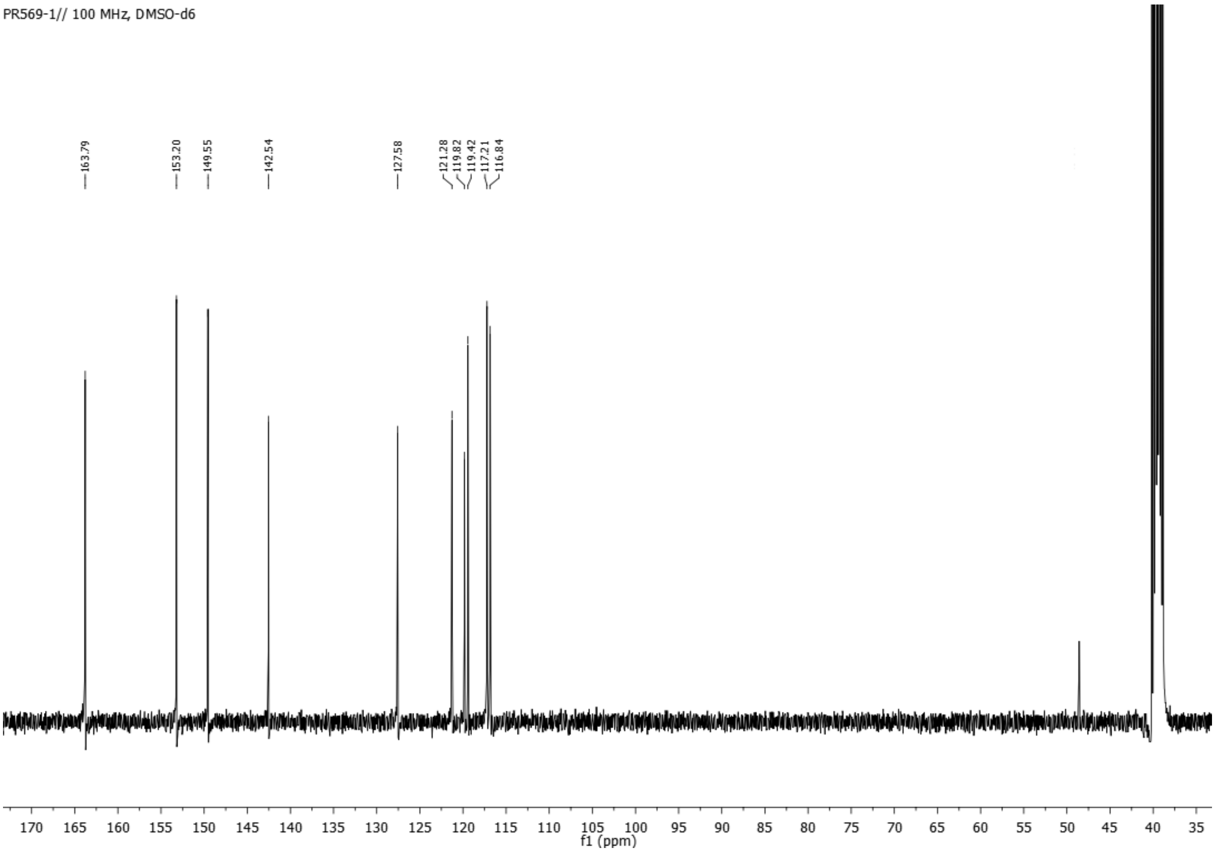

Supplement: Supplementary Information [file srep23499-s1.pdf]
