# Supplementary material for: Structural revisions of small molecules reported to cross-link G-quadruplex DNA in vivo reveal a repetitive assignment error in the literature
Source: Sci Rep. 2016 Mar 23;6:23499. doi: 10.1038/srep23499 (PMC4804300; doi:10.1038/srep23499)

Supplementary Information

Structural revisions of small molecules reported to cross-link G-quadruplex DNA *in vivo* reveal a repetitive assignment error in the literature

Paul E. Reyes-Gutiérrez, Tomáš Kapal, Blanka Klepetářová, David Šaman, Radek Pohl, Zbigniew Zawada, Erika Kužmová, Miroslav Hájek, Filip Teplý*

Search Overview

Search:	search2
Date/Time done:	Thu Dec 11 13:15:48 2014
Database(s):	CSD version 5.35 updates (Nov 2013)<br>CSD version 5.35 (November 2013)<br>CSD version 5.35 updates (Feb 2014)<br>CSD version 5.35 updates (May 2014)
Restriction Info:	No refcode restrictions applied
Filters:	None
Percentage Completed:	100%
Number of Hits:	0

Single query used. Search found structures that:

match

Query 1

Query 1

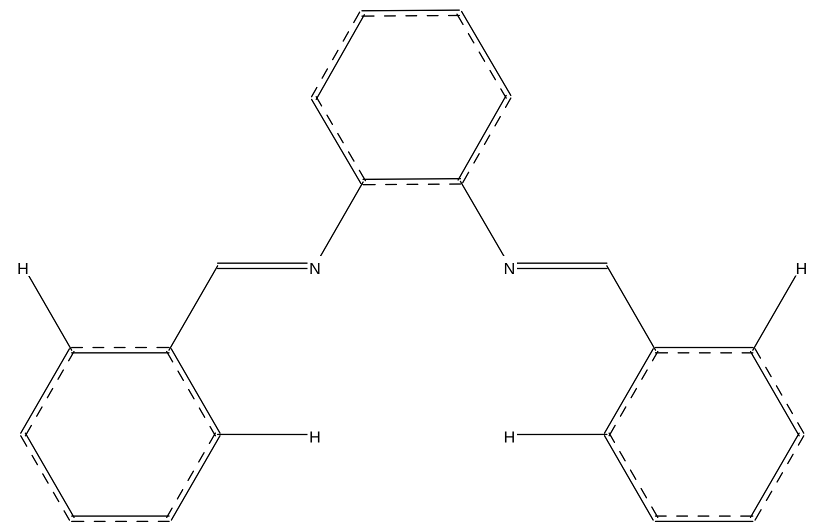

Search Overview

Search:	search1
Date/Time done:	Tue Dec 2 16:18:38 2014
Database(s):	CSD version 5.35 updates (Nov 2013)<br>CSD version 5.35 (November 2013)<br>CSD version 5.35 updates (Feb 2014)<br>CSD version 5.35 updates (May 2014)
Restriction Info:	No refcode restrictions applied
Filters:	None
Percentage Completed:	100%
Number of Hits:	71

Summary of queries used. Search found structures that:

do not match
Query 1

match at least one of
Query 2 **Query 3**

Query 1

4M

Query 2

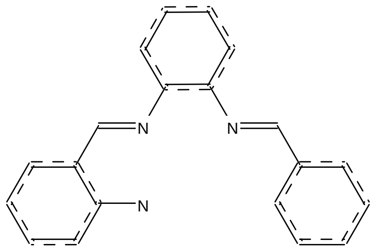

Query 3

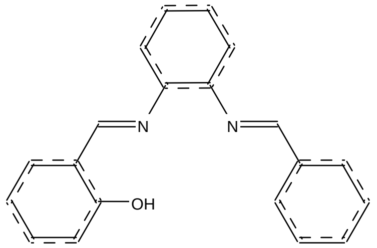

Search: search1 (Tue Dec 2 16:18:38 2014): Hits 1-4

OPHSAL11

Reference: V.Z.Mota, G.S.G.de Carvalho, P.P.Corbi, F.R.G.Bergamini, A.L.B.Formiga, R.Diniz, M.C.R.Freitas, A.D.da Silva, A.Cuin (2012) *Spectrochim.Acta,Part A* ,**99,**110

Formula: $C_{20}\ H_{16}\ N_2\ O_2$

Compound Name: 2,2'-(1,2-Phenylenebis(nitrilomethylylidene))diphenol

Synonym: (E)-3-(3-hydroxyphenyl)acrylic acid

Space Group:	P21/c	***Cell:***	***a*** 5.964(0)	***b*** 16.521(0)	***c*** 16.312(0)
Space Group No.:	14	***(Å,°)***	α 90.00	β 91.57(0)	γ 90.00

R-Factor (%): 3.65 ***Temperature(K):*** 293 ***Density(g/cm^3):*** 1.308

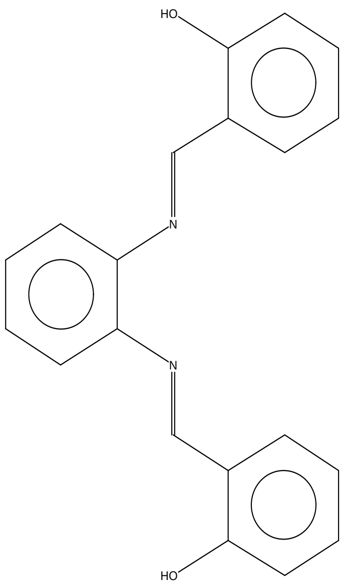

AFETOV

Reference: Xi-Quan Che (2007) *Acta Crystallogr.,Sect.E:Struct.Rep.Online* ,**63,**o3885

Formula: $C_{40}\ H_{30}\ N_4\ O_4,C_3\ H_7\ N_1\ O_1$

Compound Name: N,N',N'',N'''-tetrakis(2-hydroxybenzylidene)biphenyl-3,3',4,4'-tetramine dimethylformamide solvate

Space Group:	P21/n	***Cell:***	***a*** 15.211(1)	***b*** 6.361(0)	***c*** 20.119(3)
Space Group No.:	14	***(Å,°)***	α 90.00	β 93.88(0)	γ 90.00

R-Factor (%): 6.63 ***Temperature(K):*** 187 ***Density(g/cm^3):*** 1.204

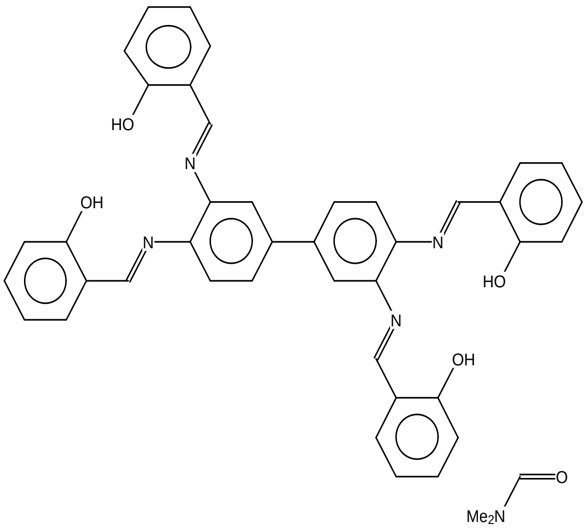

CACYAH

Reference: S.Akine, T.Taniguchi, T.Nabeshima (2001) *Tetrahedron Lett.* ,**42,**8861

Formula: $C_{42}\ H_{30}\ N_6\ O_6,3(C_2\ H_3\ N_1)$

Compound Name: 2,6,8,12,14,18-Hexa-aza-4^2,4^3,10^2,10^3,16^2,16^3-hexahydroxy-1,7,13(1,2),4,10,16(1,4)-hexabenzenaoctadecaphane-2,5,8,12,14,17-hexaene tris(acetonitrile) clathrate

Space Group:	P3121	***Cell:***	***a*** 18.201(0)	***b*** 18.201(0)	***c*** 10.781(0)
Space Group No.:	152	***(Å,°)***	α 90.00	β 90.00	γ 120.00

R-Factor (%): 5.27 ***Temperature(K):*** 120 ***Density(g/cm^3):*** 1.349

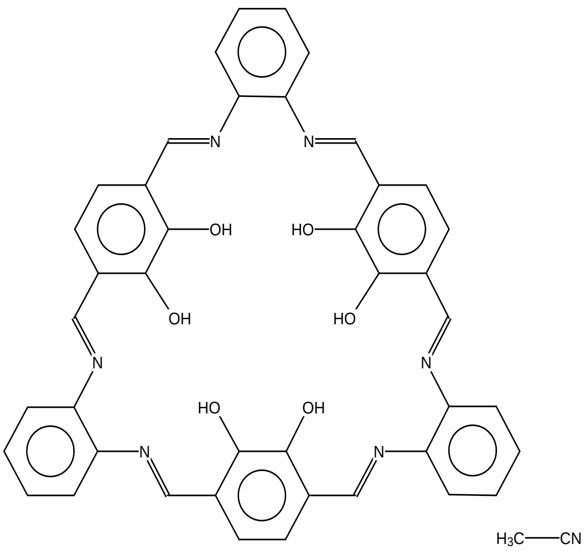

CACYEL

Reference: S.Akine, T.Taniguchi, T.Nabeshima (2001) *Tetrahedron Lett.* ,**42,**8861

Formula: $C_{42}\ H_{30}\ N_6\ O_6,C_2\ H_3\ N_1,H_2\ O_1$

Compound Name: 2,6,8,12,14,18-Hexa-aza-4^2,4^3,10^2,10^3,16^2,16^3-hexahydroxy-1,7,13(1,2),4,10,16(1,4)-hexabenzenaoctadecaphane-2,5,8,12,14,17-hexaene monohydrate clathrate acetonitrile solvate

Space Group:	P21/c	***Cell:***	***a*** 9.646(3)	***b*** 18.910(1)	***c*** 21.032(9)
Space Group No.:	14	***(Å,°)***	α 90.00	β 95.52(0)	γ 90.00

R-Factor (%): 9.35 ***Temperature(K):*** 120 ***Density(g/cm^3):*** 1.346

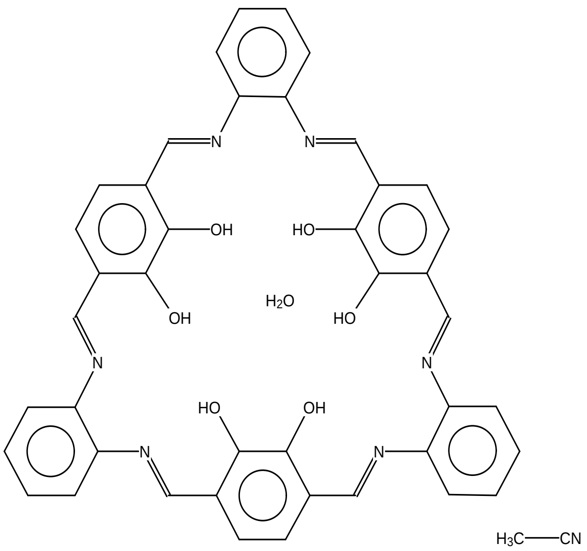

Search: search1 (Tue Dec 2 16:18:38 2014): Hits 5-8

CAJKOO

Reference: H.Houjou, Y.Nagawa, K.Hiratani (2001) *Tetrahedron Lett.* , **42,**3861

Formula: $C_{48}H_{40}N_4O_4$

Compound Name: 47,48,49,50-Tetrahydroxy-3,26-dimethylene-11,18,34,41-tetra-azaheptacyclopentaconta[41.3.1.$1^{5,9}$.$1^{20,24}$.$1^{28,32}$.$0^{12,17}$.$0^{35,40}$]-1(47),5(48),6,8,10,12,14,16,18,20(49),21,23,28(50),29,31,33,35,37,39,41,43,45-docosaene

Space Group: P21/n ***Cell:*** ***a*** 7.337(0) ***b*** 20.777(0) ***c*** 12.031(0)
Space Group No.: 14 ***(Å,°)*** α 90.00 β 95.20(0) γ 90.00

R-Factor (%): 7.50 ***Temperature(K):*** 193 ***Density(g/cm³):*** 1.340

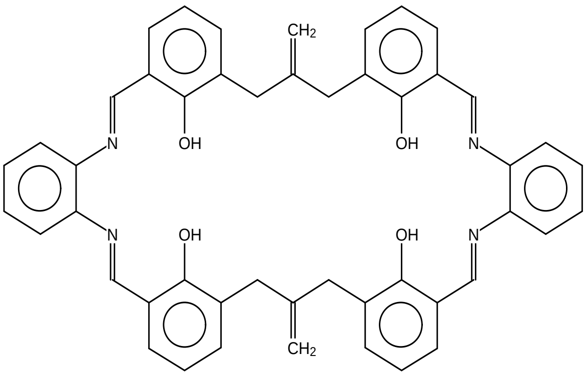

DAWREZ

Reference: A.J.Gallant, J.K.-H.Hui, F.E.Zahariev, Y.A.Wang, M.J.MacLachlan (2005) *J.Org.Chem.* ,**70,**7936

Formula: $C_{54}H_{54}N_6O_{12}$,6($C_3H_7N_1O_1$)

Compound Name: 4^4,4^5,10^4,10^5,16^4,16^5-Hexaethoxy-1^2,1^3,7^2,7^3,13^2,13^3-hexahydroxy-3,5,9,11,15,17-hexa-aza-1,7,13(1,4)4,10,16(1,2)-hexabenzenacyclo-octadecaphane-2,5,8,11,14,17-hexaene dimethylformamide solvate

Space Group: P-1 ***Cell:*** ***a*** 10.314(0) ***b*** 16.569(2) ***c*** 22.171(3)
Space Group No.: 2 ***(Å,°)*** α 83.10(0) β 79.19(0) γ 77.88(0)

R-Factor (%): 7.54 ***Temperature(K):*** 173 ***Density(g/cm³):*** 1.299

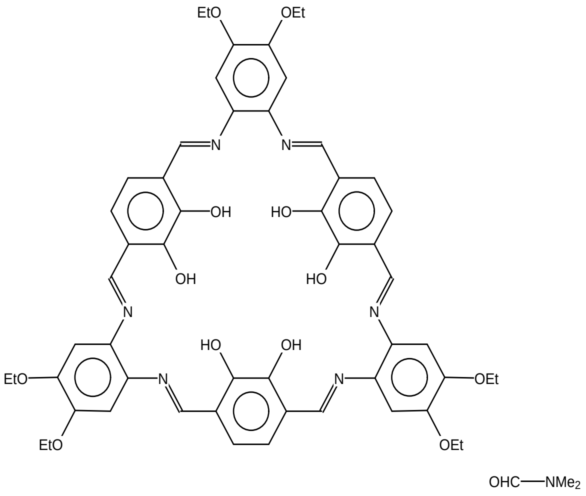

DEDPEI

Reference: Xiaoping Yang, R.A.Jones, Qiaoyin Wu, M.M.Oye, Wing-Kit Lo, Wai-Kwok Wong, A.L.Holmes (2006) *Polyhedron* ,**25,**271

Formula: $C_{22}H_{18}Br_2N_2O_4$,$C_2H_3N_1$

Compound Name: (N,N'-o-Phenylene-bis(5-bromo-3-methoxysalicylidineamine)) acetonitrile solvate

Space Group: P-1 ***Cell:*** ***a*** 7.931(1) ***b*** 12.429(3) ***c*** 13.345(3)
Space Group No.: 2 ***(Å,°)*** α 64.83(3) β 76.76(3) γ 83.18(3)

R-Factor (%): 5.97 ***Temperature(K):*** 153 ***Density(g/cm³):*** 1.649

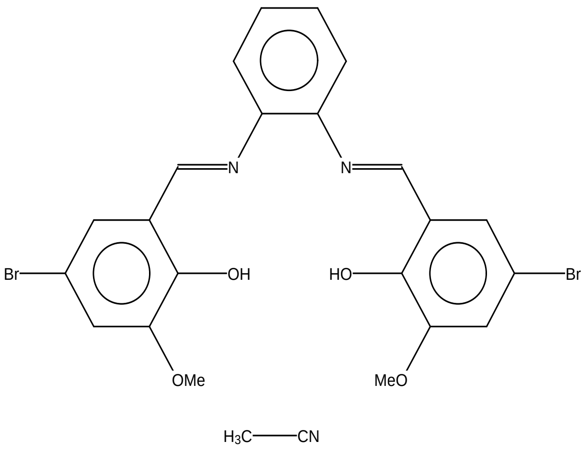

DUJGUL

Reference: N.E.Eltayeb, S.G.Teoh, C.S.Yeap, H.-K.Fun, R.Adnan (2009) *Acta Crystallogr.,Sect.E:Struct.Rep.Online* ,**65,**o3142

Formula: $C_{28}H_{32}N_2O_2$

Compound Name: 6,6'-Di-t-butyl-2,2'-[1,2-phenylenebis(nitrilomethylidyne)]diphenol

Space Group: P-1 ***Cell:*** ***a*** 6.831(0) ***b*** 13.963(1) ***c*** 14.069(1)
Space Group No.: 2 ***(Å,°)*** α 116.62(0) β 99.07(0) γ 98.21(0)

R-Factor (%): 5.93 ***Temperature(K):*** 100 ***Density(g/cm³):*** 1.238

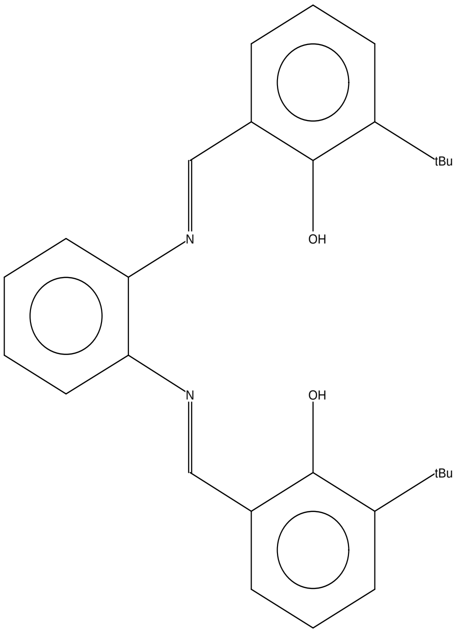

Search: search1 (Tue Dec 2 16:18:38 2014): Hits 9-12

EZIMAB

Reference: Shih-Sheng Sun, C.L.Stern, S.T.Nguyen, J.T.Hupp (2004) *J.Am.Chem.Soc.* ,**126,**6314

Formula: $C_{42} H_{38} N_4 O_2, C_1 H_2 Cl_2$

Compound Name: 1,2-Diaminobenzene-N,N'-bis(3-t-butyl-5-(2-(4-pyridyl)ethynyl) salicylidene) dichloromethane solvate

Space Group:	C2/c	***Cell:***	***a*** 18.020(4)	***b*** 22.933(5)	***c*** 9.780(1)
Space Group No.:	15	***(Å,°)***	α 90.00	β 108.51(1)	γ 90.00

R-Factor (%): 5.42 ***Temperature(K):*** 153 ***Density(g/cm³):*** 1.240

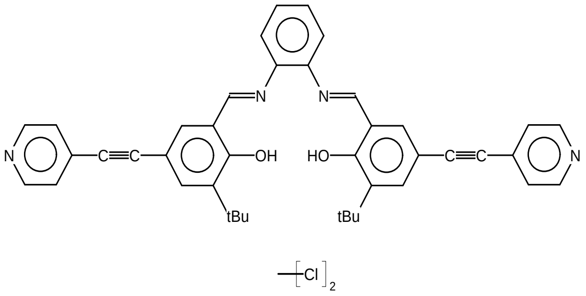

FANTEU

Reference: A.J.Gallant, B.O.Patrick, M.J.MacLachlan (2004) *J.Org.Chem.* ,**69,**8739

Formula: $C_{78} H_{104} N_6 O_{12}, H_2 O_1$

Compound Name: 6,7,20,21,34,35-hexakis(Hexyloxy)-13,14,27,28,41,42-hexahydroxy-3,10,17,24,31,38-hexaaza-heptacyclo(38.2.2.2^{12,15}.2^{26,29}.0^{4,9}.0^{18,23}.0^{32,37})octatetraconta-1(42),2,4,6,8,10,12,14,16,18,20,22,24,26,28,30,32,34,36,40,43,45,47-tricosaene monohydrate

Space Group:	C2/c	***Cell:***	***a*** 18.170(3)	***b*** 28.681(3)	***c*** 14.745(3)
Space Group No.:	15	***(Å,°)***	α 90.00	β 104.00(1)	γ 90.00

R-Factor (%): 11.15 ***Temperature(K):*** 173 ***Density(g/cm³):*** 1.190

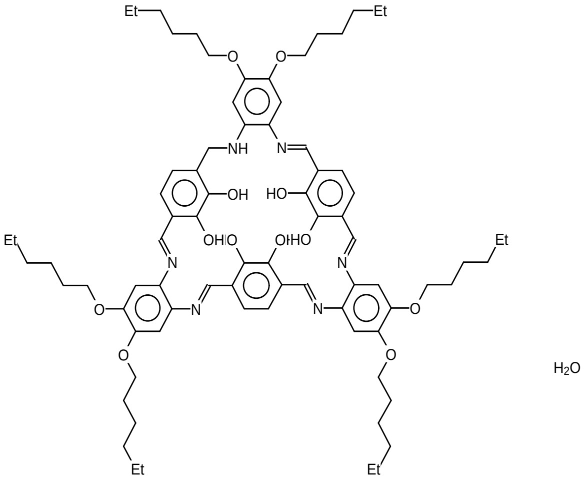

FIZYOC

Reference: A.Mederos, A.Medina, P.Gili, F.G.Manrique, P.Nunez (1986) *Anales de Quimica,Ser.B* ,**82,**338

Formula: $C_{20} H_{15} Cl_1 N_2 O_2$

Compound Name: N,N'-3,4-Chlorophenylene-bis(salicylaldimine)

Space Group:		***Cell:***	***a*** 11.417(7)	***b*** 12.370(10)	***c*** 13.220(10)
Space Group No.:	0	***(Å,°)***	α 90.00	β 67.45(8)	γ 90.00

R-Factor (%): 0.00 ***Temperature(K):*** 295 ***Density(g/cm³):*** 1.351

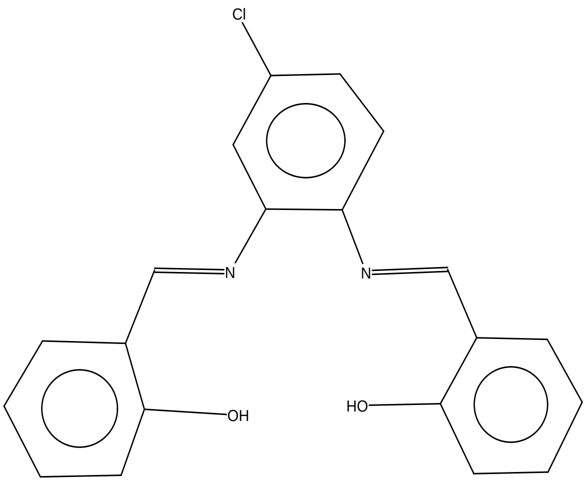

FIZYUI

Reference: A.Mederos, A.Medina, P.Gili, F.G.Manrique, P.Nunez (1986) *Anales de Quimica,Ser.B* ,**82,**338

Formula: $C_{21} H_{18} N_2 O_2$

Compound Name: N,N'-3,4-Toluene-bis(salicylaldimine)

Space Group:		***Cell:***	***a*** 11.220(4)	***b*** 13.379(3)	***c*** 12.316(4)
Space Group No.:	0	***(Å,°)***	α 90.00	β 65.08(4)	γ 90.00

R-Factor (%): 0.00 ***Temperature(K):*** 295 ***Density(g/cm³):*** 1.309

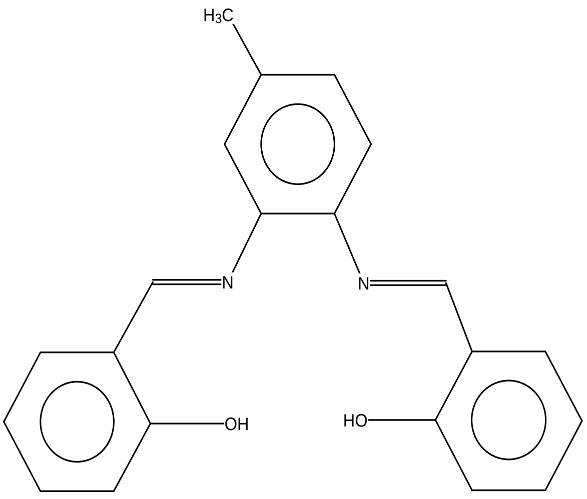

Search: search1 (Tue Dec 2 16:18:38 2014): Hits 13-16

GETXEJ

Reference: N.E.Eltayeb, Siang Guan Teoh, J.Bee-Jan Teh, Hoong-Kun Fun, K.Ibrahim (2007)
Acta Crystallogr.,Sect.E:Struct.Rep.Online ,**63,**o117

Formula: $C_{28} H_{20} N_2 O_2$,0.5($C_1 H_4 O_1$)

Compound Name: N,N'-bis(2-Hydroxy-1-naphthaldimine)-o-phenylenediamine methanol solvate

Space Group:	Pna21	***Cell:***	***a*** 19.361(0)	***b*** 12.510(0)	***c*** 17.713(0)
Space Group No.:	33	***(Å,°)***	α 90.00	β 90.00	γ 90.00

R-Factor (%): 4.38 ***Temperature(K):*** 295 ***Density(g/cm^3):*** 1.339

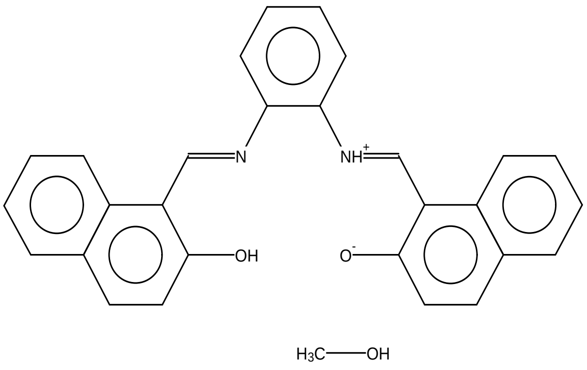

GISFOE

Reference: K.-W.Lei, H.-M.Feng, T.-H.Zhou (2008)
Acta Crystallogr.,Sect.E:Struct.Rep.Online ,**64,**o161

Formula: $C_{22} H_{20} N_2 O_2$

Compound Name: 4,4'-Dimethyl-2,2'-(1,2-phenylenebis(nitrilomethylidene))diphenol

Space Group:	P21/c	***Cell:***	***a*** 6.083(1)	***b*** 16.207(3)	***c*** 18.607(4)
Space Group No.:	14	***(Å,°)***	α 90.00	β 98.28(3)	γ 90.00

R-Factor (%): 4.87 ***Temperature(K):*** 296 ***Density(g/cm^3):*** 1.260

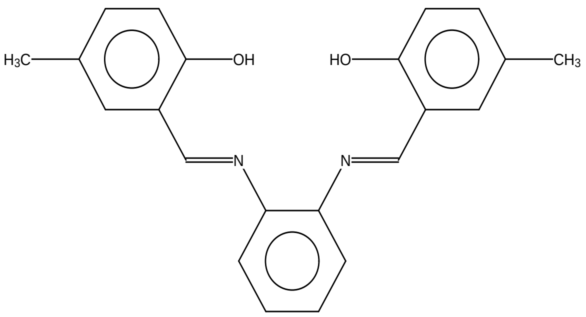

HIVXUG

Reference: I.Yoon, T.Shimizu, M.Asakawa (2008)
Anal.Sci.:X-Ray Struct.Anal.Online ,**24,**x11

Formula: $C_{34} H_{34} N_2 O_8$,$C_2 H_3 N_1$

Compound Name: 4^2,20^2-dihydroxy-1,12(1,2)-dibenzena-4,20(1,3)-dibenzena-2,22-diaza-5,8,11,13,16,19-hexaoxacyclodocosaphane-2,21-diene acetonitrile solvate

Synonym: N,N'-o-phenylenebis(salicylideneimine)-o-phenylenebis(2-(2-oxyethoxy)ethoxy) acetonitrile solvate; (salophen)-o-phenylenebis(2-(2-oxyethoxy)ethoxy) acetonitrile solvate

Space Group:	P21/c	***Cell:***	***a*** 14.008(0)	***b*** 18.358(1)	***c*** 12.670(0)
Space Group No.:	14	***(Å,°)***	α 90.00	β 97.10(0)	γ 90.00

R-Factor (%): 4.57 ***Temperature(K):*** 153 ***Density(g/cm^3):*** 1.314

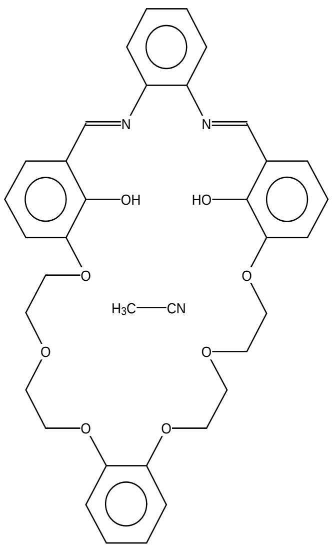

HUCQEC

Reference: N.E.Eltayeb, S.G.Teoh, C.S.Yeap, H.-K.Fun, R.Adnan (2009) *Acta Crystallogr.,Sect.E:Struct.Rep.Online* ,**65,**o2065

Formula: $C_{21} H_{18} N_2 O_4$

Compound Name: 2-((E)-(2-[((E)-2,3-dihydroxybenzylidene)amino]-5-methylphenyl)iminiomethyl)-6-hydroxyphenolate

Space Group:	P21/c	***Cell:***	***a*** 17.063(0)	***b*** 21.346(0)	***c*** 20.029(0)
Space Group No.:	14	***(Å,°)***	α 90.00	β 99.98(0)	γ 90.00

R-Factor (%): 4.80 ***Temperature(K):*** 100 ***Density(g/cm^3):*** 1.340

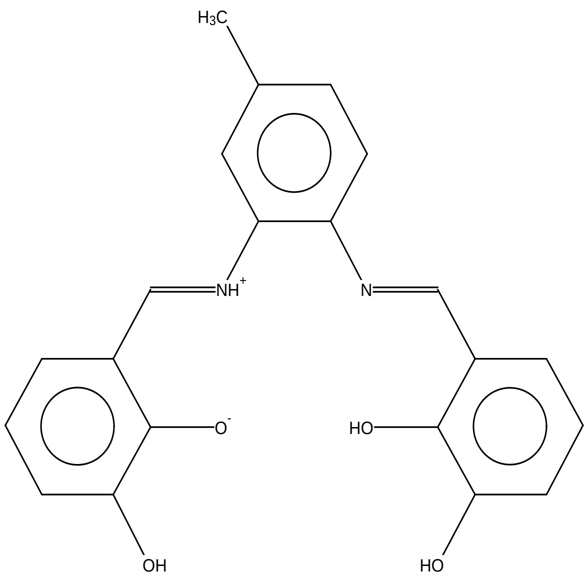

HYCTDC

Reference: P.G.Owston, R.Peters, E.Ramsammy, P.A.Tasker, J.Trotter (1980) *Chem.Commun.* ,1218

Formula: $C_{22} H_{20} N_4$

Compound Name: 17,18,19,20-Tetrahydrotribenzo(e,i,m)(1,4,8,11)tetra-azacyclotetradecine

Space Group:	P21/n	***Cell:***	***a*** 20.924(11)	***b*** 4.772(1)	***c*** 18.302(11)
Space Group No.:	14	***(Å,°)***	α 90.00	β 106.32(1)	γ 90.00

R-Factor (%): 6.40 ***Temperature(K):*** 295 ***Density(g/cm³):*** 1.289

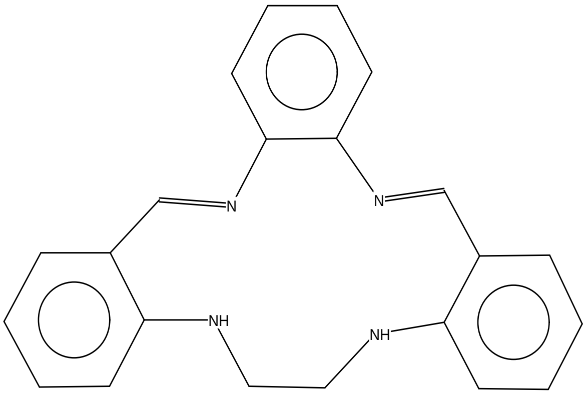

IZUZUY

Reference: S.Akine, D.Hashimoto, T.Saiki, T.Nabeshima (2004) *Tetrahedron Lett.* ,**45,**4225

Formula: $C_{60} H_{42} N_6 O_{12}$,8.5($C_2 H_6 O_1 S_1$),2.75($H_2 O_1$)

Compound Name: $4^2,4^3,5^2,5^3,11^2,11^3,12^2,12^3,18^2,18^3,19^2,19^3$-Dodecahydroxy-2,7,9,14,16,21-hexa-aza-1,8,15(1,2),4,5,11,12,18,19(1,4)-nonabenzenacyclohenicosaphane-2,6,9,13,16,20-hexaene dimethylsulfoxide solvate hydrate

Space Group:	P321	***Cell:***	***a*** 26.052(5)	***b*** 26.052(5)	***c*** 29.249(7)
Space Group No.:	150	***(Å,°)***	α 90.00	β 90.00	γ 120.00

R-Factor (%): 9.64 ***Temperature(K):*** 120 ***Density(g/cm³):*** 1.354

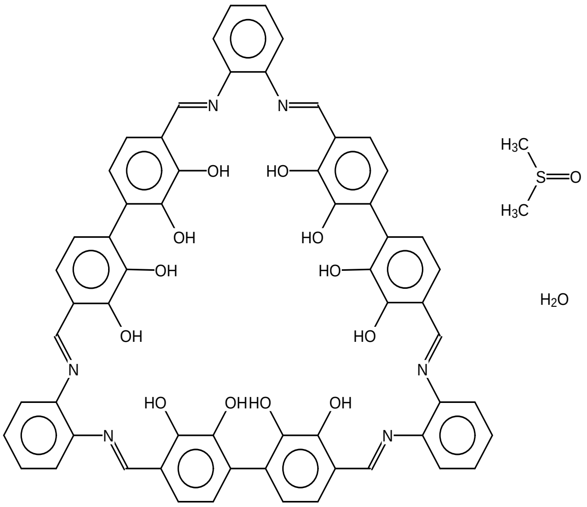

JEVREI

Reference: Naser Eltaher Eltayeb, Siang Guan Teoh, J.Bee-Jan Teh, H.-K.Fun, Kamarulazizi Ibrahim (2007) *Acta Crystallogr.,Sect.E:Struct.Rep.Online* ,**63,o**766

Formula: $C_{22} H_{20} N_2 O_2$

Compound Name: 6,6'-Dimethyl-2,2'-(1,2-phenylenebis(nitrilomethylidyne))diphenol

Space Group:	P21/c	***Cell:***	***a*** 10.792(0)	***b*** 7.765(0)	***c*** 23.344(0)
Space Group No.:	14	***(Å,°)***	α 90.00	β 116.79(0)	γ 90.00

R-Factor (%): 5.72 ***Temperature(K):*** 295 ***Density(g/cm³):*** 1.310

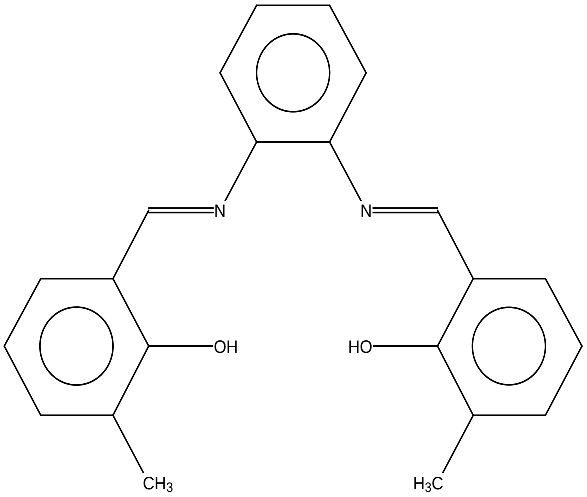

KIYDOL

Reference: J.Mahia, M.A.Maestro, M.Vazquez, M.R.Bermejo, A.M.Gonzalez, M.Maneiro (2000) *Acta Crystallogr.,Sect.C:Cryst.Struct.Commun.* ,**56,**492

Formula: $C_{34} H_{30} N_4 O_4 S_2$

Compound Name: N,N'-bis(2-(p-Tosylamino)benzylidene)benzene-1,2-diamine

Space Group:	P21/c	***Cell:***	***a*** 9.704(2)	***b*** 19.102(4)	***c*** 17.407(4)
Space Group No.:	14	***(Å,°)***	α 90.00	β 103.85(2)	γ 90.00

R-Factor (%): 5.70 ***Temperature(K):*** 295 ***Density(g/cm³):*** 1.320

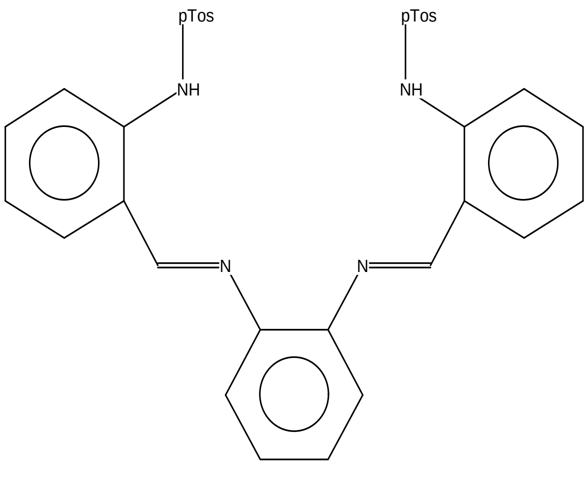

Search: search1 (Tue Dec 2 16:18:38 2014): Hits 21-24

KUSJIS

Reference: H.Kargar, R.Kia, I.U.Khan, A.Sahraei, P.A.Azar (2010)
Acta Crystallogr.,Sect.E:Struct.Rep.Online ,**66,**o728

Formula: $C_{24} H_{24} N_2 O_4$

Compound Name: 5,5'-Dimethoxy-2,2'-[4,5-dimethyl-o-phenylenebis(nitrilomethylidyne)]-diphenol

Space Group:	P21/n	***Cell:***	***a*** 6.672(0)	***b*** 14.319(0)	***c*** 21.740(0)		
Space Group No.:	14	***(Å,°)***	α 90.00	β 95.18(0)	γ 90.00		

R-Factor (%): 4.61 ***Temperature(K):*** 296 ***Density(g/cm^3):*** 1.299

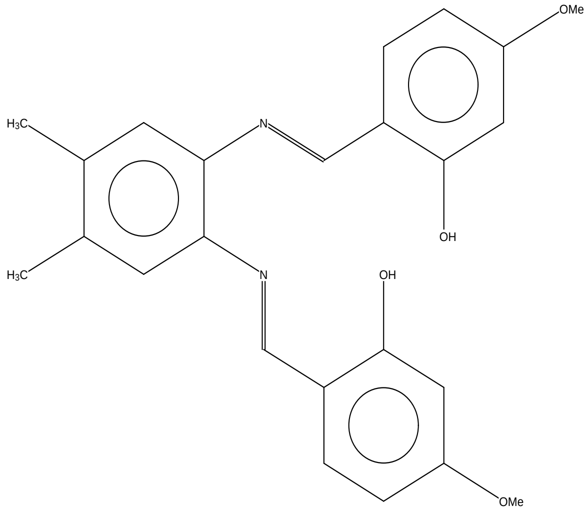

LIVSOZ

Reference: N.E.Eltayeb, S.G.Teoh, S.Chantrapromma, Hoong-Kun Fun, R.Adnan (2008)
Acta Crystallogr.,Sect.E:Struct.Rep.Online ,**64,**o576

Formula: $C_{36} H_{48} N_2 O_2, C_3 H_6 O_1$

Compound Name: 4,4',6,6'-Tetra-t-butyl-2,2'-(1,2-phenylenebis(nitrilomethylidyne))diphenol acetone solvate

Synonym: 2,2'-(1,2-phenylenebis(nitrilomethylidyne))bis(4,6-di-t-butylphenol) acetone solvate

Space Group:	P-1	***Cell:***	***a*** 10.001(0)	***b*** 12.002(0)	***c*** 17.037(0)		
Space Group No.:	2	***(Å,°)***	α 82.07(0)	β 86.32(0)	γ 65.76(0)		

R-Factor (%): 5.99 ***Temperature(K):*** 296 ***Density(g/cm^3):*** 1.077

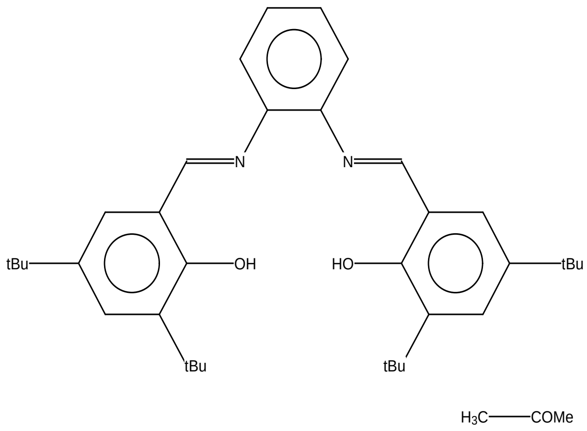

LORPEO

Reference: H.Kargar, R.Kia, A.Jamshidvand, H.-K.Fun (2009)
Acta Crystallogr.,Sect.E:Struct.Rep.Online ,**65,**o776

Formula: $C_{26} H_{28} N_2 O_4, C_2 H_6 O_1, H_2 O_1$

Compound Name: 6,6'-Diethoxy-2,2'-(4,5-dimethyl-o-phenylenebis(nitrilomethylidyne)) diphenol ethanol solvate monohydrate

Synonym: 2,2'-((4,5-Dimethyl-1,2-phenylene)bis(nitrilomethylylidene))bis(6-ethoxyphenol) ethanol solvate monohydrate

Space Group:	P21/n	***Cell:***	***a*** 9.510(0)	***b*** 25.663(1)	***c*** 10.777(0)		
Space Group No.:	14	***(Å,°)***	α 90.00	β 99.18(0)	γ 90.00		

R-Factor (%): 6.59 ***Temperature(K):*** 100 ***Density(g/cm^3):*** 1.270

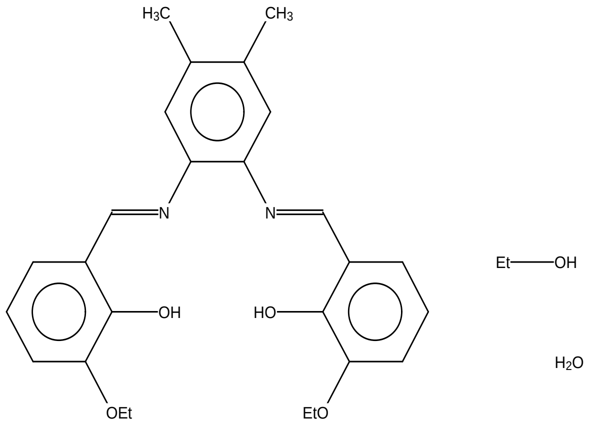

MANMIX

Reference: Yunqi Tian, Jian Tong, G.Frenzen, Jin-Yu Sun (1999)
J.Org.Chem. ,**64,**1442

Formula: $C_{30} H_{26} N_4 O_2{}^{2+}, 2(Cl_1 O_4{}^{1-})$

Compound Name: (N,N-Dihydrobis(benzene-1,2-diamino)-N,N'-bis(4-methyl-2,6-diformylphenol)) diperchlorate

Space Group:	P-1	***Cell:***	***a*** 8.671(5)	***b*** 9.163(4)	***c*** 10.872(3)		
Space Group No.:	2	***(Å,°)***	α 69.62(2)	β 87.57(3)	γ 63.72(4)		

R-Factor (%): 4.19 ***Temperature(K):*** 193 ***Density(g/cm^3):*** 1.554

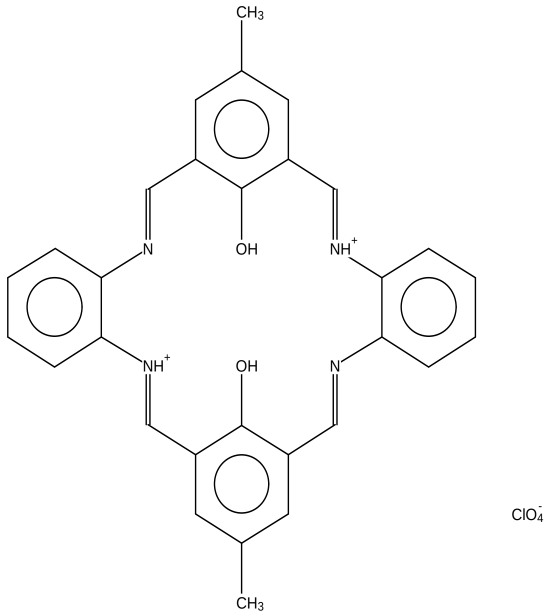

Search: search1 (Tue Dec 2 16:18:38 2014): Hits 25-28

MANMOD

Reference: Yunqi Tian, Jian Tong, G.Frenzen, Jin-Yu Sun (1999) *J.Org.Chem.* ,**64,**1442

Formula: $C_{36}\ H_{38}\ N_4\ O_2\ ^{2+}$,2($Cl_1\ O_4\ ^{1-}$)

Compound Name: (N,N-Dihydrobis(benzene-1,2-diamino)-N,N'-bis(4-t-butyl-2,6-diformylphenol)) diperchlorate

Space Group:	P21/n	***Cell:***	***a***	6.320(1)	***b***	16.615(3)	***c***	16.580(3)
Space Group No.:	14	***(Å,°)***	α	90.00	β	99.87(3)	γ	90.00

R-Factor (%): 5.30 ***Temperature(K):*** 133 ***Density(g/cm³):*** 1.467

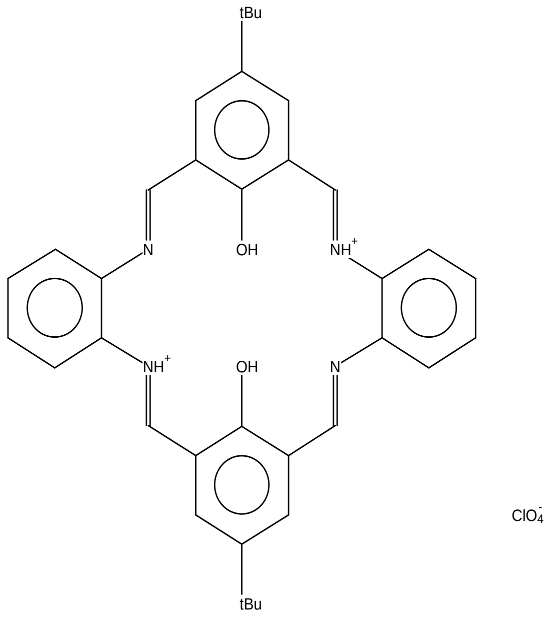

MATFET

Reference: Fan Zhang, Shi Bai, G.P.A.Yap, V.Tarwade, J.M.Fox (2005) *J.Am.Chem.Soc.* ,**127,**10590

Formula: $C_{48}\ H_{54}\ N_4\ O_4$

Compound Name: 4,5-Dihexyl-1,2-bis(2'-hydroxy-3'-(N-p-tolylcarbamoyl)benzylideneamino) benzene

Space Group:	P21	***Cell:***	***a***	12.373(1)	***b***	10.381(1)	***c***	16.608(2)
Space Group No.:	4	***(Å,°)***	α	90.00	β	93.25(0)	γ	90.00

R-Factor (%): 8.28 ***Temperature(K):*** 298 ***Density(g/cm³):*** 1.171

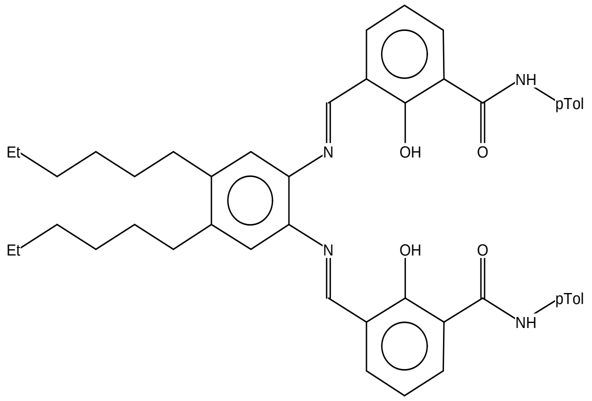

MEPWUA

Reference: Wing-Kit Lo, Wai-Kwok Wong, Wai-Yeung Wong, Jianping Guo, Kai-Tai Yeung, Yuen-Kit Cheng, Xiaoping Yang, R.A.Jones (2006) *Inorg.Chem.* ,**45,**9315

Formula: $C_{22}\ H_{20}\ N_2\ O_4$

Compound Name: N,N'-bis(3-Methoxysalicylidene)phenylene-1,2-diamine

Space Group:	P21/c	***Cell:***	***a***	6.652(0)	***b***	16.879(2)	***c***	17.184(2)
Space Group No.:	14	***(Å,°)***	α	90.00	β	97.91(0)	γ	90.00

R-Factor (%): 3.93 ***Temperature(K):*** 293 ***Density(g/cm³):*** 1.308

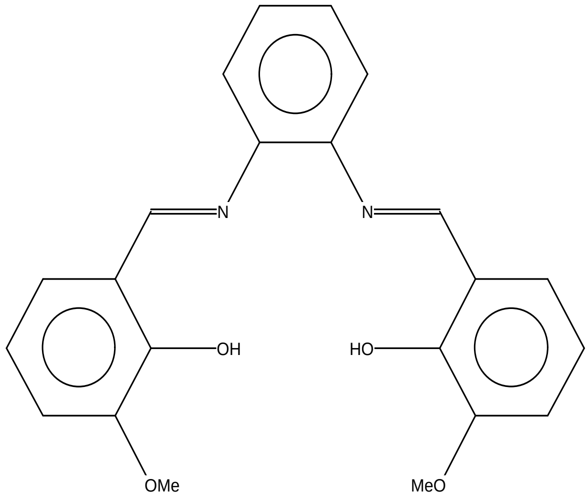

MEPWUA01

Reference: Y.-F.Liu, H.-T.Xia, S.-P.Yang, D.-Q.Wang (2006) *Acta Crystallogr.,Sect.E:Struct.Rep.Online* ,**62,**o5908

Formula: $C_{22}\ H_{20}\ N_2\ O_4$

Compound Name: (E,E)-6,6'-Dimethoxy-2,2'-(o-phenylenebis(nitrilomethylidyne))diphenol

Space Group:	P21/c	***Cell:***	***a***	6.638(2)	***b***	16.838(3)	***c***	17.137(3)
Space Group No.:	14	***(Å,°)***	α	90.00	β	97.86(0)	γ	90.00

R-Factor (%): 6.33 ***Temperature(K):*** 298 ***Density(g/cm³):*** 1.318

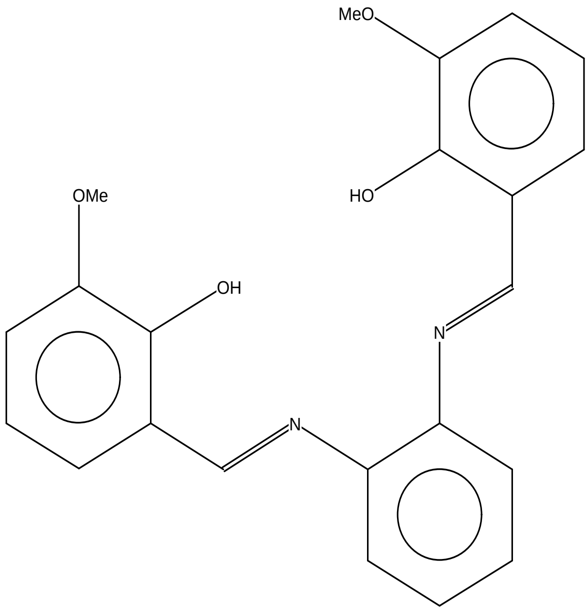

Search: search1 (Tue Dec 2 16:18:38 2014): Hits 29-32

MEPWUA02

Reference: Yong Wang, Hong-Gang Li, Handong Yin, Guo-Dong Wei, Xiao Wang (2009) *Acta Crystallogr.,Sect.E:Struct.Rep.Online* ,**65,**o754

Formula: $C_{22}\,H_{20}\,N_2\,O_4$

Compound Name: (E,E)-6,6'-Dimethoxy-2,2'-[o-phenylenebis(nitrilomethylidyne)]diphenol

Synonym: 2,2'-(1,2-Phenylenebis(nitrilomethylylidene))bis(6-methoxyphenol)

Space Group:	P21/c	***Cell:***	***a*** 6.586(0)	***b*** 16.726(2)	***c*** 17.023(3)		
Space Group No.:	14	***(Å,°)***	α 90.00	β 97.93(0)	γ 90.00		
R-Factor (%):	5.03	***Temperature(K):***	298	***Density(g/cm³):***	1.346		

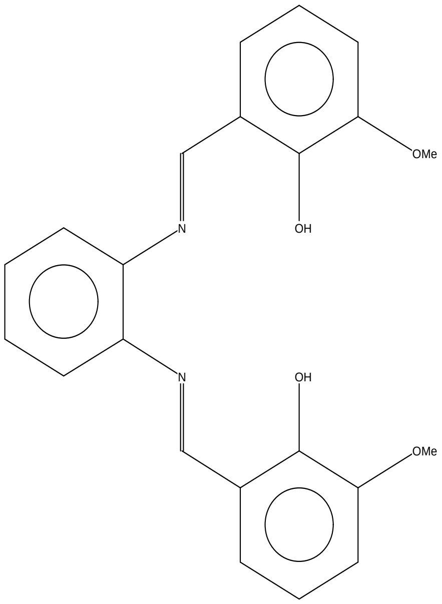

MEPXAH

Reference: Wing-Kit Lo, Wai-Kwok Wong, Wai-Yeung Wong, Jianping Guo, Kai-Tai Yeung, Yuen-Kit Cheng, Xiaoping Yang, R.A.Jones (2006) *Inorg.Chem.* ,**45,**9315

Formula: $C_{36}\,H_{32}\,N_2\,O_4$

Compound Name: N,N'-bis(3-Methoxy-5-(p-tolyl)salicylidene)phenylene-1,2-diamine

Space Group:	P21/n	***Cell:***	***a*** 16.761(1)	***b*** 7.422(0)	***c*** 23.799(3)
Space Group No.:	14	***(Å,°)***	α 90.00	β 105.98(0)	γ 90.00
R-Factor (%):	5.17	***Temperature(K):***	293	***Density(g/cm³):***	1.299

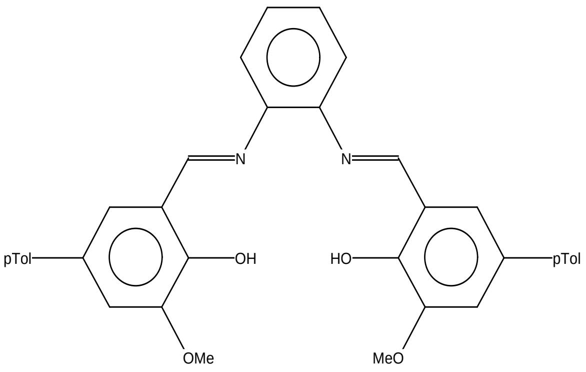

MIJWUY

Reference: M.J.Rodriguez-Douton, M.I.Fernandez, A.M.Gonzalez-Noya, M.Maneiro, R.Pedrido, M.J.Romero (2006) *Synth.React.Inorg.,Met.-Org.,Nano-Met.Chem.* ,**36,**655

Formula: $C_{20}\,H_{16}\,N_2\,O_4$

Compound Name: N,N'-(1,2-Phenylene)-bis(3-hydroxysalicylidenimine)

Synonym: (N,N'-bis(2,3-Dihydroxybenzylidene))-benzene-1,2-diamine

Space Group:	P21/n	***Cell:***	***a*** 15.475(5)	***b*** 6.430(5)	***c*** 16.796(5)
Space Group No.:	14	***(Å,°)***	α 90.00	β 96.08(0)	γ 90.00
R-Factor (%):	5.98	***Temperature(K):***	293	***Density(g/cm³):***	1.392

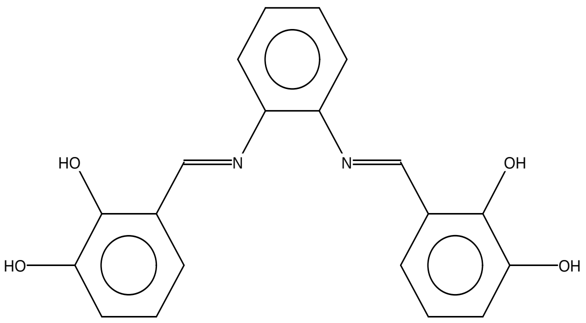

MISWIV

Reference: T.Yamato, S.Miyamoto, M.Takimoto, P.Thuery (2007) *J.Chem.Res.* ,649

Formula: $C_{62}\,H_{72}\,N_4\,O_4,2(C_1\,H_2\,Cl_2)$

Compound Name: $1^5,7^5,11^5,17^5$-Tetra-t-butyl-$1^2,7^2,11^2,17^2$-tetrahydroxy-3,5,13,15-tetra-aza-4,14(1,2)dibenzena-1,7,11,17(1,3)tetrabenzenacycloicosaphane-2,5,12,15-tetraene dichloromethane solvate

Space Group:	P-1	***Cell:***	***a*** 9.608(1)	***b*** 10.945(1)	***c*** 14.349(2)
Space Group No.:	2	***(Å,°)***	α 91.97(0)	β 101.05(0)	γ 103.51(0)
R-Factor (%):	4.37	***Temperature(K):***	100	***Density(g/cm³):***	1.281

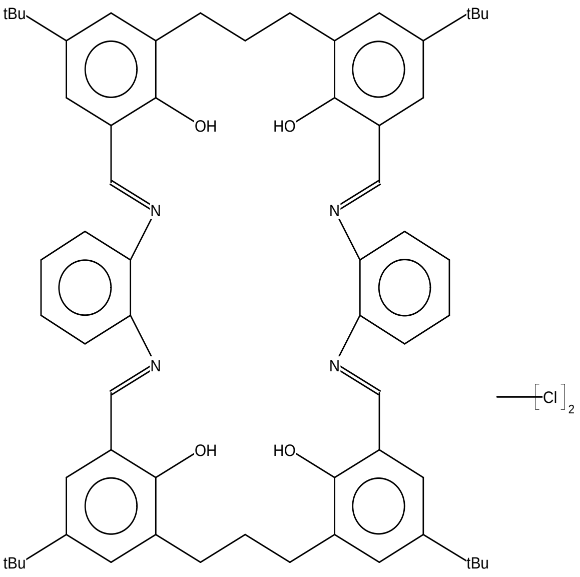

Search: search1 (Tue Dec 2 16:18:38 2014): Hits 33-36

MOQCIE

Reference: M.Kabak, A.Elmali, Y.Elerman, T.N.Durlu (2000) *J.Mol.Struct.* ,**553,**187

Formula: C_{20} H_{14} Br_2 N_2 O_2

Compound Name: N,N'-bis(p-Bromosalicylidene)benzene-1,2-diamine

Space Group:	Pbca	***Cell:***	***a***	18.805(3)	***b***	25.394(4)	***c***	7.549(2)
Space Group No.:	61	***(Å,°)***	α	90.00	β	90.00	γ	90.00

R-Factor (%): 4.77 ***Temperature(K):*** 293 ***Density(g/cm^3):*** 1.747

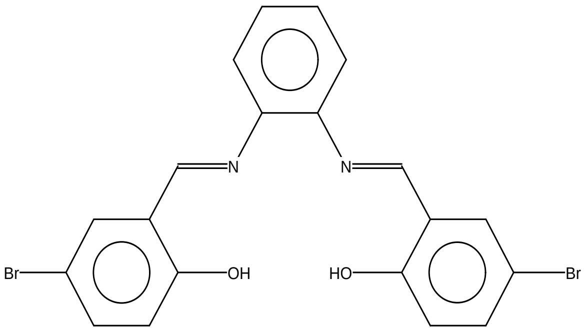

MOSPOA

Reference: Jing Gao, Yan Cheng (2009) *Acta Crystallogr.,Sect.E:Struct.Rep.Online* ,**65,**o979

Formula: C_{20} H_{15} Br_1 N_2 O_2

Compound Name: 2,2'-[4-Bromo-o-phenylenebis(nitrilomethylidyne)]diphenol

Space Group:	P21/c	***Cell:***	***a***	12.874(1)	***b***	5.997(1)	***c***	22.106(2)
Space Group No.:	14	***(Å,°)***	α	90.00	β	91.22(0)	γ	90.00

R-Factor (%): 4.51 ***Temperature(K):*** 297 ***Density(g/cm^3):*** 1.539

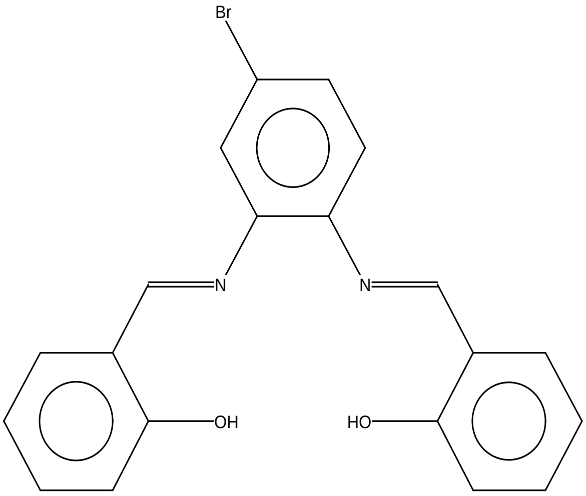

MURQEW

Reference: Wei You, Cheng Yao, Wei Huang (2010) *Wuji Huaxue Xuebao(Chin.)(Chin.J.Inorg.Chem.)* ,**26,**867

Formula: C_{20} H_{12} Br_4 N_2 O_2

Compound Name: 2,2'-(1,2-Phenylenebis(nitrilomethylylidene))bis(4,6-dibromophenol)

Space Group:	P21/n	***Cell:***	***a***	12.714(1)	***b***	7.743(0)	***c***	20.849(3)
Space Group No.:	14	***(Å,°)***	α	90.00	β	98.80(0)	γ	90.00

R-Factor (%): 4.19 ***Temperature(K):*** 291 ***Density(g/cm^3):*** 2.069

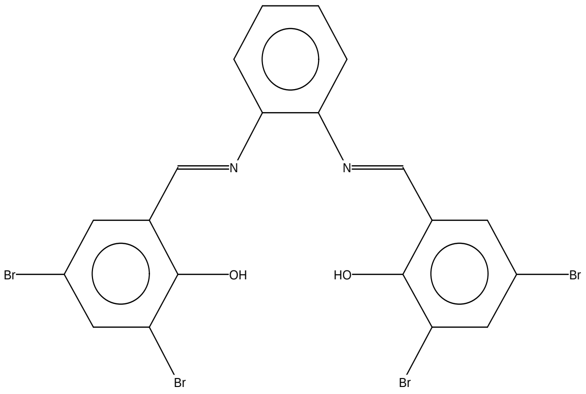

NENHIY

Reference: T.Nabeshima, H.Miyazaki, A.Iwasaki, S.Akine, T.Saiki, C.Ikeda, S.Sato (2006) *Chem.Lett.* ,**35,**1070

Formula: C_{66} H_{78} N_6 O_{12},2(C_1 H_2 Cl_2)

Compound Name: $1^4,1^5,7^4,7^5,13^4,13^5$-Hexa-n-butoxy-$4^2,4^3,10^2,10^3,16^2,16^3$-hexahydroxy-2,6,8,12,14,18-hexa-aza-1,7,13(1,2)-tribenzena-4,10,16(1,4)tribenzena-octadecacyclophane-2,5,8,11,14,17-hexaene dichloromethane solvate

Space Group:	Pbcn	***Cell:***	***a***	26.427(12)	***b***	24.883(1)	***c***	10.406(1)
Space Group No.:	60	***(Å,°)***	α	90.00	β	90.00	γ	90.00

R-Factor (%): 9.93 ***Temperature(K):*** 180 ***Density(g/cm^3):*** 1.279

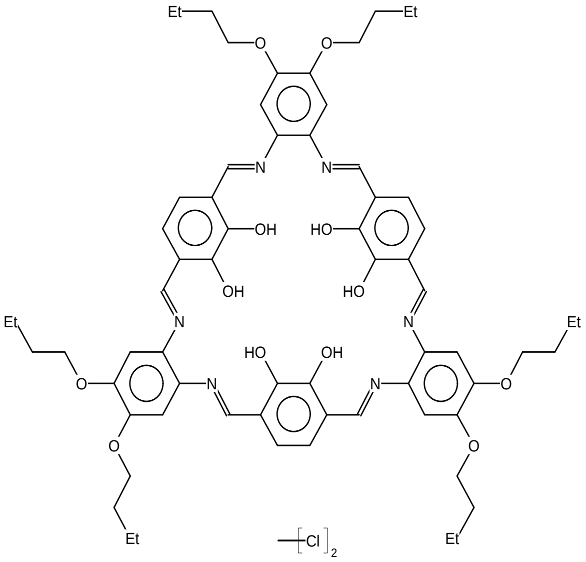

Search: search1 (Tue Dec 2 16:18:38 2014): Hits 37-40

NUGWES

Reference: Zhen-Xiang Xu, Zhi-Tang Huang, Chuan-Feng Chen (2009) *Tetrahedron Lett.* ,**50,**5430

Formula: $C_{74}\,H_{98}\,N_2\,O_6, C_1\,H_4\,O_1, 0.5(C_1\,H_2\,Cl_2)$

Compound Name: (cS)-4,5-bis(2-Hydroxy-5-t-butylbenzylideneamino)-11,17,23-tri-t-butyl-25,26,27,28-tetrakis(n-propoxy)calix(4)arene dichloromethane methanol solvate

Space Group:	P21	***Cell:***	***a*** 13.418(8)	***b*** 14.397(9)	***c*** 18.553(11)
Space Group No.:	4	***(Å,°)***	α 90.00	β 94.64(0)	γ 90.00

R-Factor (%): 9.70 ***Temperature(K):*** 113 ***Density(g/cm³):*** 1.103

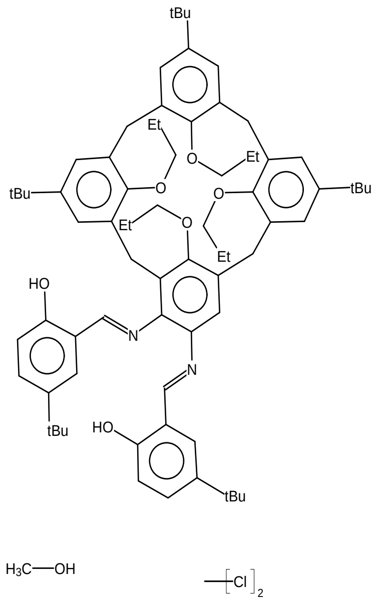

NUGWIW

Reference: Zhen-Xiang Xu, Zhi-Tang Huang, Chuan-Feng Chen (2009) *Tetrahedron Lett.* ,**50,**5430

Formula: $C_{82}\,H_{114}\,N_2\,O_6$

Compound Name: (cS)-4,5-bis(2-Hydroxy-3,5-di-t-butylbenzylideneamino)-11,17,23-tri-t-butyl-25,26,27,28-tetrakis(n-propoxy)calix(4)arene

Space Group:	P212121	***Cell:***	***a*** 10.086(2)	***b*** 22.698(5)	***c*** 33.244(7)
Space Group No.:	19	***(Å,°)***	α 90.00	β 90.00	γ 90.00

R-Factor (%): 7.08 ***Temperature(K):*** 113 ***Density(g/cm³):*** 1.068

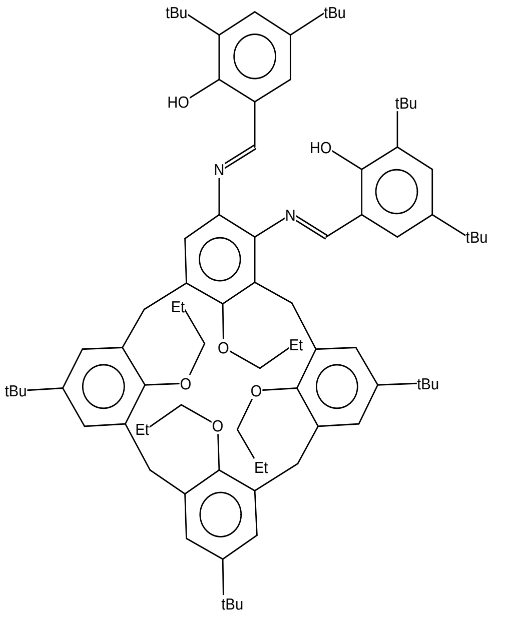

NUGWOC

Reference: Zhen-Xiang Xu, Zhi-Tang Huang, Chuan-Feng Chen (2009) *Tetrahedron Lett.* ,**50,**5430

Formula: $C_{82}\,H_{114}\,N_2\,O_6$

Compound Name: (cR)-4,5-bis(2-Hydroxy-3,5-di-t-butylbenzylideneamino)-11,17,23-tri-t-butyl-25,26,27,28-tetrakis(n-propoxy)calix(4)arene

Space Group:	P212121	***Cell:***	***a*** 10.084(0)	***b*** 22.679(0)	***c*** 33.227(1)
Space Group No.:	19	***(Å,°)***	α 90.00	β 90.00	γ 90.00

R-Factor (%): 6.08 ***Temperature(K):*** 113 ***Density(g/cm³):*** 1.070

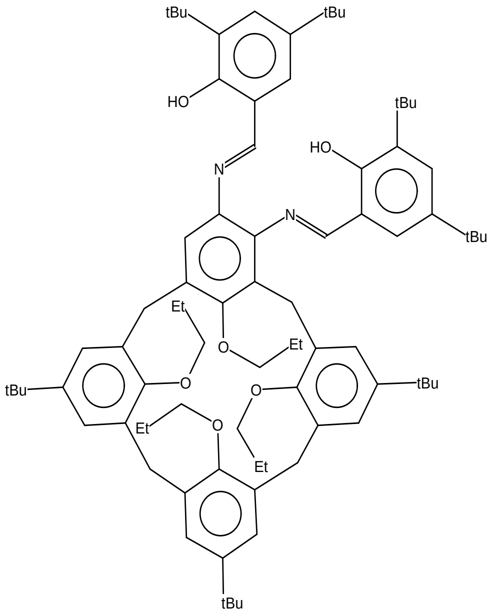

OPHSAL01

Reference: C.Subrahmanyam, M.Seshasayee, G.Aravamudan (1982) *Cryst.Struct.Commun.* ,**11,**1719

Formula: $C_{20}\,H_{16}\,N_2\,O_2$

Compound Name: bis(Salicylidene)-o-phenylenedi-imine

Space Group:	P21/c	***Cell:***	***a*** 5.981(4)	***b*** 16.579(3)	***c*** 16.347(5)
Space Group No.:	14	***(Å,°)***	α 90.00	β 91.28(0)	γ 90.00

R-Factor (%): 7.00 ***Temperature(K):*** 295 ***Density(g/cm³):*** 1.297

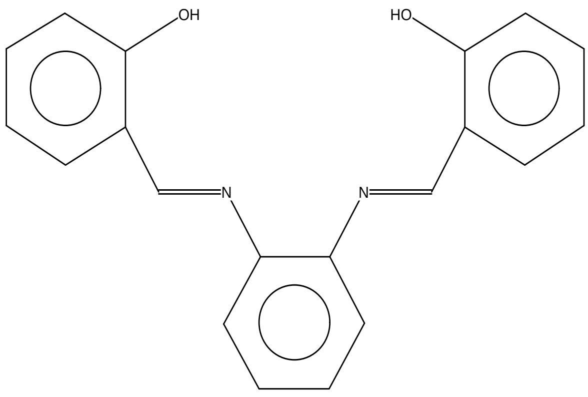

Search: search1 (Tue Dec 2 16:18:38 2014): Hits 41-44

OPHSAL10

Reference: N.B.Pahor, M.Calligaris, P.Delise, G.Dodic, G.Nardin, L.Randaccio (1976) *J.Chem.Soc.,Dalton Trans.* ,2478

Formula: $C_{20}\ H_{16}\ N_2\ O_2$

Compound Name: N,N'-(o-Phenylene)-bis(salicylideneamine)

Space Group:	P21/c	***Cell:***	***a***	6.064(3)	***b***	16.541(7)	***c***	16.306(7)
Space Group No.:	14	***(Å,°)***	α	90.00	β	91.50(10)	γ	90.00

R-Factor (%): 5.60 ***Temperature(K):*** 295 ***Density(g/cm3):*** 1.285

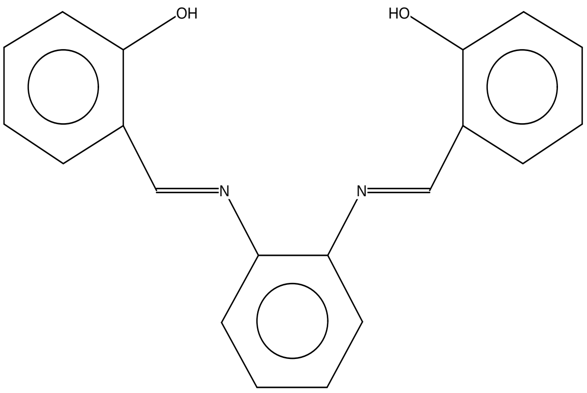

PIXWUP

Reference: Tianzhi Yu, Kai Zhang, Yuling Zhao, Changhui Yang, Hui Zhang, Long Qian, Duowang Fan, Wenkui Dong, Lili Chen, Yongqing Qiu (2008) *Inorg.Chim.Acta* ,**361,**233

Formula: $C_{28}\ H_{34}\ N_4\ O_2$

Compound Name: N,N'-bis(4-(Diethylamino)salicylidene)-1,2-phenylenediamine

Space Group:	P21/c	***Cell:***	***a***	19.487(3)	***b***	9.119(2)	***c***	14.246(3)
Space Group No.:	14	***(Å,°)***	α	90.00	β	97.36(0)	γ	90.00

R-Factor (%): 6.07 ***Temperature(K):*** 293 ***Density(g/cm3):*** 1.213

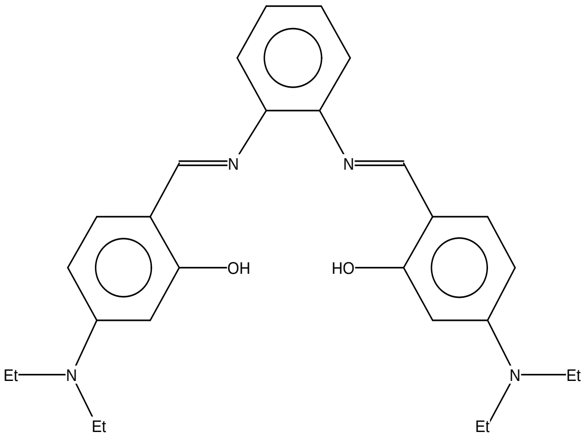

PIYDEH

Reference: E.C.Escudero-Adan, J.Benet-Buchholz, A.W.Kleij (2008) *Dalton Trans.* ,734

Formula: $C_{36}\ H_{48}\ N_2\ O_2,1.5(C_2\ H_3\ N_1)$

Compound Name: N,N'-bis(3,5-Di-t-butylsalicylidene)benzene-1,2-diamine acetonitrile solvate

Space Group:	P-1	***Cell:***	***a***	9.967(0)	***b***	11.721(0)	***c***	33.572(1)
Space Group No.:	2	***(Å,°)***	α	81.39(0)	β	82.28(0)	γ	69.16(0)

R-Factor (%): 7.81 ***Temperature(K):*** 100 ***Density(g/cm3):*** 1.108

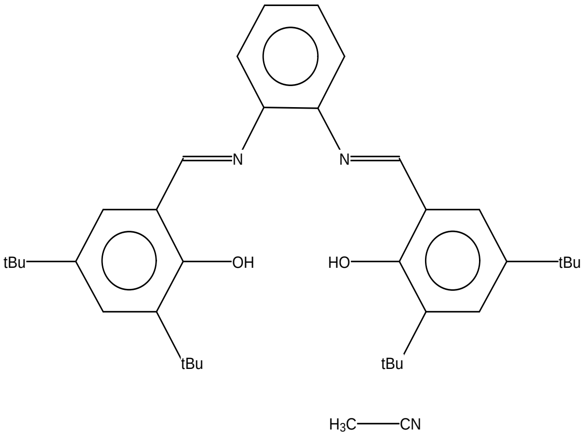

PIYXIF

Reference: Tuan-Jie Meng, Xiao-Qiang Qin, Wen-Xian Zhao, Xian-Qiang Huang, Guo-Dong Wei (2008) *Acta Crystallogr.,Sect.E:Struct.Rep.Online* ,**64,**o1520

Formula: $C_{28}\ H_{20}\ N_2\ O_2,0.5(C_2\ H_6\ O_1)$

Compound Name: 1,1'-(o-Phenylenebis(nitrilomethylidyne))di-2-naphthol ethanol solvate

Space Group:	Pna21	***Cell:***	***a***	19.956(2)	***b***	12.474(1)	***c***	18.189(2)
Space Group No.:	33	***(Å,°)***	α	90.00	β	90.00	γ	90.00

R-Factor (%): 11.54 ***Temperature(K):*** 298 ***Density(g/cm3):*** 1.289

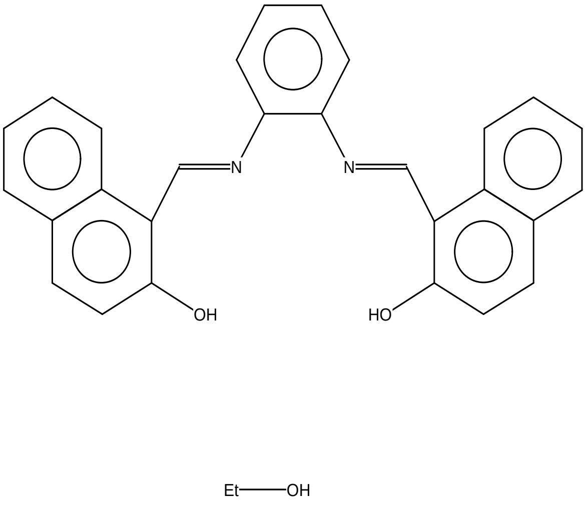

Search: search1 (Tue Dec 2 16:18:38 2014): Hits 45-48

PUJGIL

Reference: H.Kargar, R.Kia, I.U.Khan, A.Sahraei (2010) *Acta Crystallogr.,Sect.E:Struct.Rep.Online* ,**66,**o539

Formula: $C_{24} H_{24} N_2 O_4,H_2 O_1$

Compound Name: 6,6'-Dimethoxy-2,2'-[4,5-dimethyl-o-phenylenebis(nitrilomethylidyne)]diphenol monohydrate

Space Group:	P-1	***Cell:***	***a*** 8.743(0)	***b*** 10.305(0)	***c*** 13.661(0)
Space Group No.:	2	***(Å,°)***	α 69.56(0)	β 83.85(0)	γ 70.28(0)

R-Factor (%): 4.62 ***Temperature(K):*** 296 ***Density(g/cm^3):*** 1.292

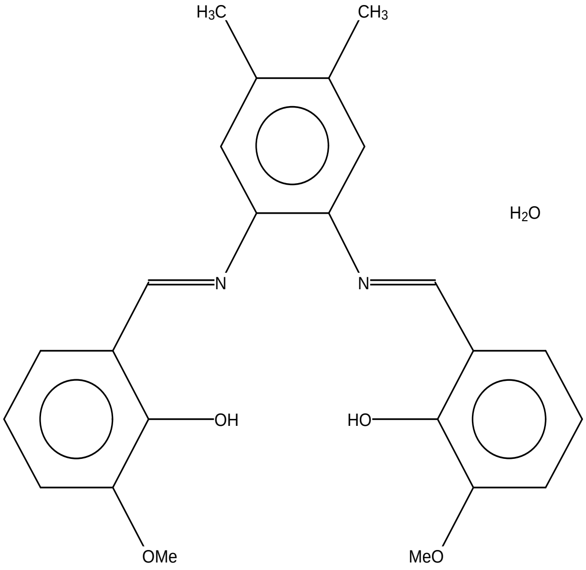

PUSWEG

Reference: S.Akine, F.Utsuno, T.Nabeshima (2010) *Chem.Commun.* , **46,**1029

Formula: $C_{40} H_{28} N_4 O_6,2(C_2 H_3 N_1)$

Compound Name: 2,23-Dioxa-9,16,30,37-tetraazaheptacyclo[37.3.1.1^{3,7}.1^{18,22}.1^{24,28}.0^{10,15}.0^{31,36}]hexatetraconta-1(43),3(46),4,6,8,10,12,14,16,18(45), 19,21,24(44),25,27,29,31,33,35,37,39,41-docosaene-43,44,45,46-tetrol acetonitrile solvate

Space Group:	Pbcn	***Cell:***	***a*** 21.767(0)	***b*** 8.396(0)	***c*** 20.408(0)
Space Group No.:	60	***(Å,°)***	α 90.00	β 90.00	γ 90.00

R-Factor (%): 4.88 ***Temperature(K):*** 120 ***Density(g/cm^3):*** 1.323

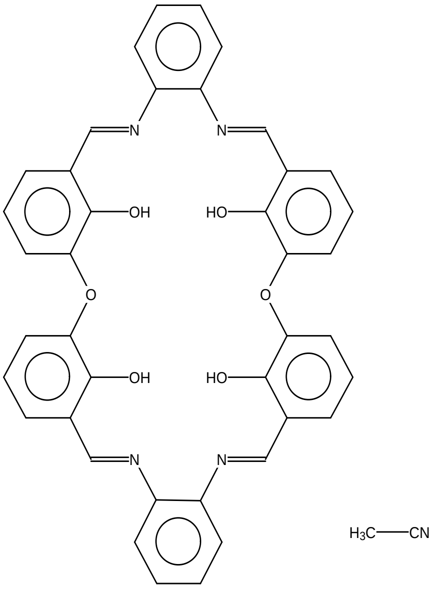

QAYYAS

Reference: M.Mariappan, M.Suenaga, A.Mukhopadhyay, B.G.Maiya (2012) *Inorg.Chim.Acta* ,**390,**95

Formula: $C_{33} H_{23} N_3 O_2,C_3 H_7 N_1 O_1$

Compound Name: 2,2'-((4-(acridin-9-yl)-1,2-phenylene)bis(nitrilomethylylidene))diphenol N, N-dimethylformamide solvate

Space Group:	P-1	***Cell:***	***a*** 9.021(0)	***b*** 13.024(1)	***c*** 13.776(1)
Space Group No.:	2	***(Å,°)***	α 102.86(0)	β 102.41(0)	γ 103.83(0)

R-Factor (%): 7.34 ***Temperature(K):*** 298 ***Density(g/cm^3):*** 1.281

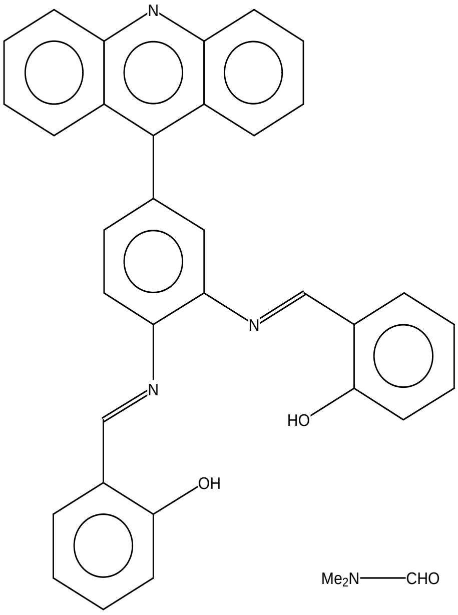

QEXLOV

Reference: N.E.Eltayeb, S.G.Teoh, J.B.-J.Teh, H.-K.Fun, K.Ibrahim (2007) *Acta Crystallogr.,Sect.E:Struct.Rep.Online* ,**63,**o695

Formula: $C_{22} H_{20} N_2 O_2$

Compound Name: 2,2'-(1,2-Phenylenebis(nitrilomethylidene))bis(5-methylphenol)

Space Group:	P21/c	***Cell:***	***a*** 6.078(0)	***b*** 16.557(0)	***c*** 17.499(0)
Space Group No.:	14	***(Å,°)***	α 90.00	β 90.35(0)	γ 90.00

R-Factor (%): 4.93 ***Temperature(K):*** 100 ***Density(g/cm^3):*** 1.299

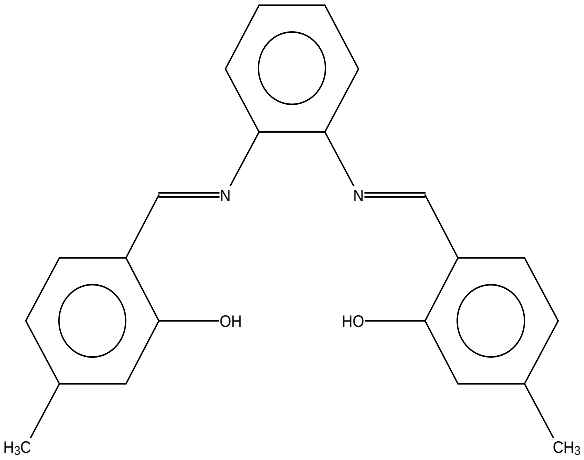

Search: search1 (Tue Dec 2 16:18:38 2014): Hits 49-52

RIHDUI

Reference: N.E.Eltayeb, S.G.Teoh, S.Chantrapromma, H.-K.Fun, K.Ibrahim (2007) *Acta Crystallogr.,Sect.E:Struct.Rep.Online* ,**63,**o3234

Formula: $C_{22} H_{20} N_2 O_4$

Compound Name: 4,4'-Dimethoxy-2,2'-(1,2-phenylenebis(nitrilomethylidyne))diphenol

Space Group:	P21/c	***Cell:***	***a*** 11.677(0) ***b*** 13.872(0) ***c*** 13.118(0)
Space Group No.:	14	***(Å,°)***	α 90.00 β 119.88(0) γ 90.00
R-Factor (%):	5.33	***Temperature(K):*** 100	***Density(g/cm³):*** 1.357

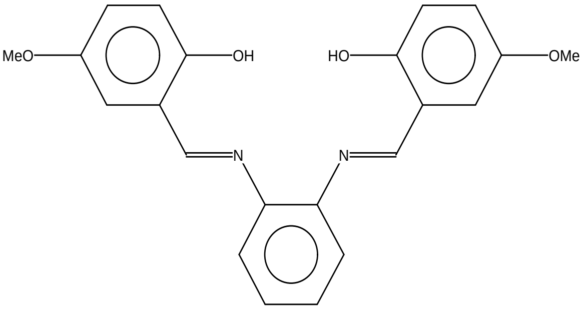

SAFTEA

Reference: Rui-Fang Ding, Qi-Bao Wang, Xin-Min Wen (2012) *Acta Crystallogr.,Sect.E:Struct.Rep.Online* ,**68,**o40

Formula: $C_{30} H_{36} N_2 O_2$

Compound Name: 2,2'-(1,2-Phenylenebis(nitrilomethylylidene))bis(6-t-butyl-4-methylphenol)

Space Group:	P-1	***Cell:***	***a*** 10.578(7) ***b*** 11.394(7) ***c*** 12.217(7)
Space Group No.:	2	***(Å,°)***	α 72.20(0) β 73.53(0) γ 72.98(0)
R-Factor (%):	7.52	***Temperature(K):*** 296	***Density(g/cm³):*** 1.158

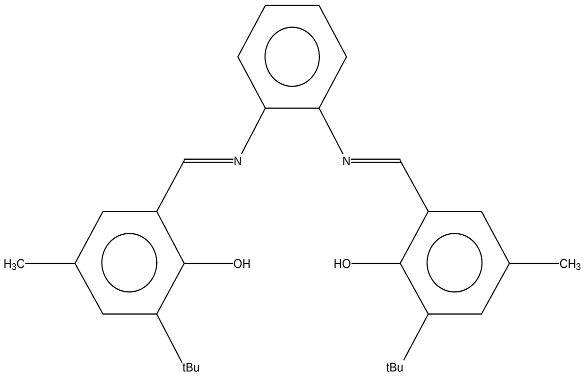

SAJMIA

Reference: R.Kannappan, S.Tanase, D.M.Tooke, A.L.Spek, I.Mutikainen, U.Turpeinen, J.Reedijk (2004) *Polyhedron* ,**23,**2285

Formula: $C_{38} H_{52} N_2 O_2,C_3 H_6 O_1$

Compound Name: N,N'-bis(3,5-Di-t-butylsalicylidene)-4,5-dimethyl-1,2-phenylenediamine acetone solvate

Space Group:	P21/c	***Cell:***	***a*** 9.908(1) ***b*** 35.084(1) ***c*** 11.912(1)
Space Group No.:	14	***(Å,°)***	α 90.00 β 113.93(0) γ 90.00
R-Factor (%):	6.35	***Temperature(K):*** 150	***Density(g/cm³):*** 1.100

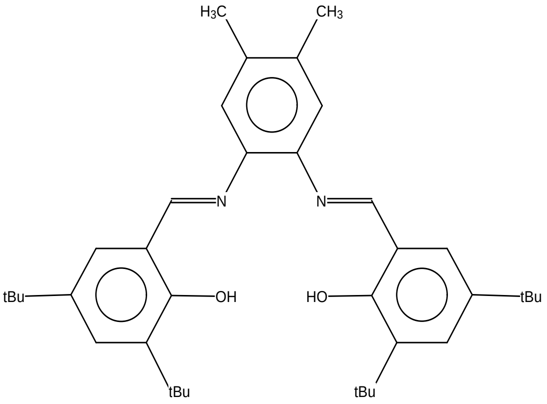

H_3C——COMe

TUGJEL

Reference: K.E.Shopsowitz, D.Edwards, A.J.Gallant, M.J.MacLachlan (2009) *Tetrahedron* ,**65,**8113

Formula: $C_{60} H_{66} N_6 O_{12},5(C_3 H_7 N_1 O_1),H_2 O_1$

Compound Name: 6,7,20,21,34,35-hexaethoxy-43,44,45,46,47,48-hexamethyl-3,10,17,24,31,38-hexaazaheptacyclo[38.2.2.2^{12,15}.2^{26,29}.0^{4,9}.0^{18,23}.0^{32,37}]octatetraconta-1(42),2,4,6,8,10,12,14,16,18,20,22,24,26,28,30,32,34,36,38,40,43,45,47-tetracosaene-13,14,27,28,41,42-hexol N,N-dimethylformamide solvate monohydrate

Space Group:	P-1	***Cell:***	***a*** 12.350(1) ***b*** 17.709(3) ***c*** 19.450(3)
Space Group No.:	2	***(Å,°)***	α 64.83(0) β 84.17(0) γ 80.87(0)
R-Factor (%):	6.92	***Temperature(K):*** 173	***Density(g/cm³):*** 1.265

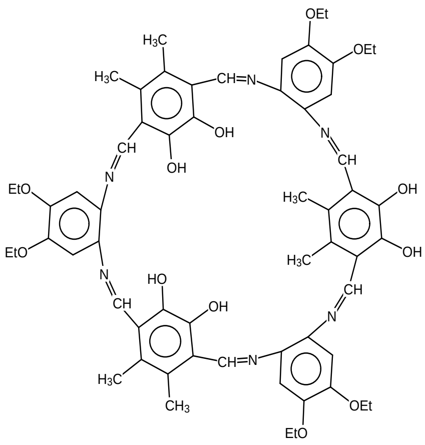

Me_2N——CHO

H_2O

Search: search1 (Tue Dec 2 16:18:38 2014): Hits 53-56

TUGJIP

Reference: K.E.Shopsowitz, D.Edwards, A.J.Gallant, M.J.MacLachlan (2009) *Tetrahedron* ,**65,**8113

Formula: C_{66} H_{78} N_6 O_6,C_2 H_3 N_1,H_2 O_1

Compound Name: 43,44,45,46,47,48-hexabutyl-3,10,17,24,31,38-hexaazaheptacyclo[38.2.2.2^{12,15}.2^{26,29}.0^{4,9}.0^{18,23}.0^{32,37}]octatetraconta-1(42),2,4,6,8,10,12,14,16,18,20,22,24,26,28,30,32,34,36,38,40,43,45,47-tetracosaene-13,14,27,28,41,42-hexol acetonitrile solvate monohydrate

Space Group:	P-1	***Cell:***	***a***	13.284(0)	***b***	16.492(0)	***c***	16.775(0)
Space Group No.:	2	***(Å,°)***	α	61.51(0)	β	68.97(0)	γ	80.20(0)

R-Factor (%): 7.88 ***Temperature(K):*** 173 ***Density(g/cm^3):*** 1.223

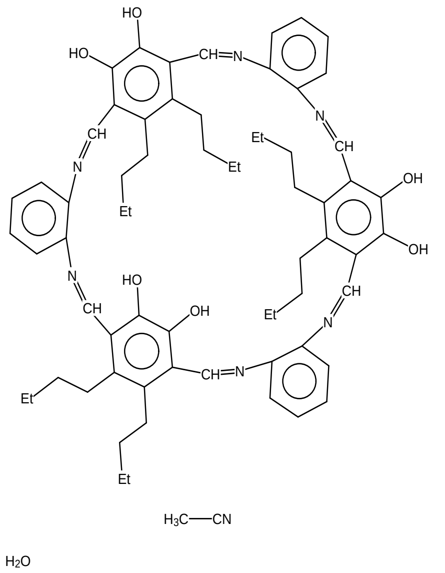

UFUYUP

Reference: H.Houjou, S.Tsuzuki, Y.Nagawa, K.Hiratani (2002) *Bull.Chem.Soc.Jpn.* ,**75,**831

Formula: C_{64} H_{72} N_4 O_4,C_6 H_6

Compound Name: 1^5,7^5,11^5,17^5-Tetra-t-butyl-9,19-dimethylene-1^2,7^2,11^2,17^2-tetrahydroxy-3,5,13,15-tetra-aza-1,7,11,17(1,3)-tetrabenzena-4,14(1,2)-dibenzenacycloicosaphane-2,5,12,15-tetraene benzene solvate

Space Group:	P-1	***Cell:***	***a***	12.475(0)	***b***	14.420(2)	***c***	9.065(0)
Space Group No.:	2	***(Å,°)***	α	101.08(0)	β	92.13(0)	γ	114.85(0)

R-Factor (%): 10.26 ***Temperature(K):*** 193 ***Density(g/cm^3):*** 1.199

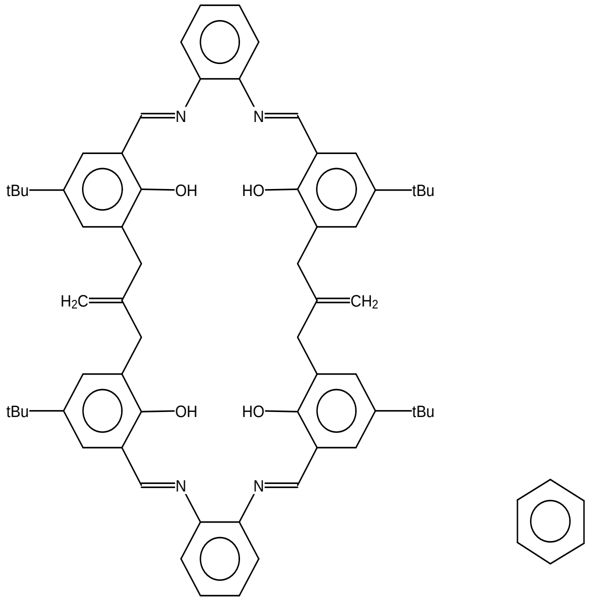

UFUZAW

Reference: H.Houjou, S.Tsuzuki, Y.Nagawa, K.Hiratani (2002) *Bull.Chem.Soc.Jpn.* ,**75,**831

Formula: C_{52} H_{48} N_4 O_4,2(C_3 H_7 N_1 O_1)

Compound Name: 4^4,4^5,14^4,14^5-Tetramethyl-9,19-dimethylene-1^2,7^2,11^2,17^2-tetrahydroxy-3,5,13,15-tetra-aza-1,7,11,17(1,3)-tetrabenzena-4,14(1,2)-dibenzenacycloicosaphane-2,5,12,15-tetraene dimethylformamide solvate

Space Group:	P-1	***Cell:***	***a***	11.787(1)	***b***	15.860(1)	***c***	7.178(0)
Space Group No.:	2	***(Å,°)***	α	97.60(0)	β	98.19(0)	γ	70.05(0)

R-Factor (%): 9.37 ***Temperature(K):*** 193 ***Density(g/cm^3):*** 1.254

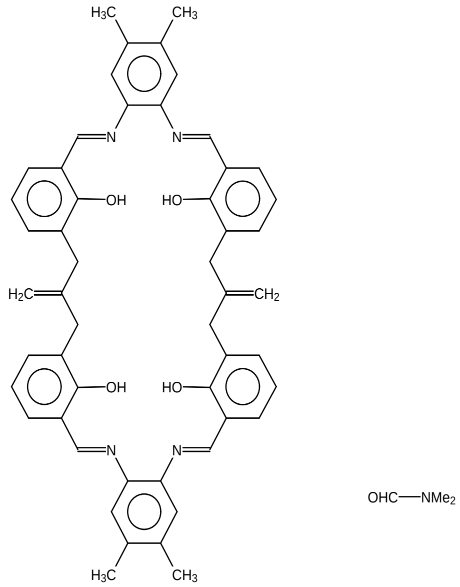

UFUZEA

Reference: H.Houjou, S.Tsuzuki, Y.Nagawa, K.Hiratani (2002) *Bull.Chem.Soc.Jpn.* ,**75,**831

Formula: C_{56} H_{56} N_4 O_4,2(C_1 H_1 Cl_3)

Compound Name: 1^5,4^4,4^5,7^5,11^5,14^4,14^5,17^5-Octamethyl-9,19-dimethylene-1^2,7^2,11^2,17^2-tetrahydroxy-3,5,13,15-tetra-aza-1,7,11,17(1,3)-tetrabenzena-4,14(1,2)-dibenzenacycloicosaphane-2,5,12,15-tetraene chloroform solvate

Space Group:	C2/c	***Cell:***	***a***	13.792(0)	***b***	15.932(0)	***c***	24.967(0)
Space Group No.:	15	***(Å,°)***	α	90.00	β	101.48(0)	γ	90.00

R-Factor (%): 5.79 ***Temperature(K):*** 193 ***Density(g/cm^3):*** 1.344

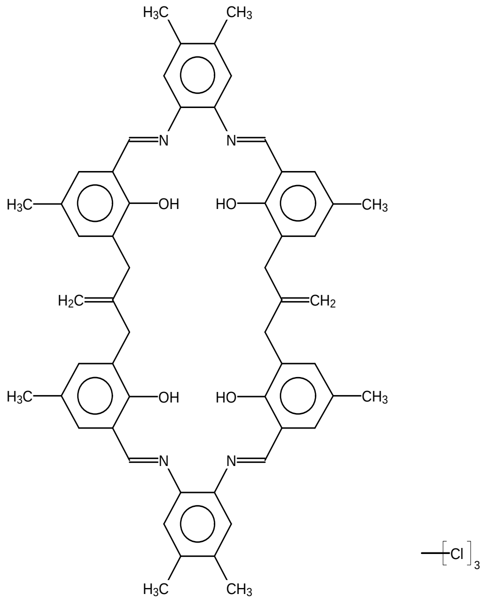

Search: search1 (Tue Dec 2 16:18:38 2014): Hits 57-60

ULETAH

Reference: A.Sahraei, H.Kargar, R.Kia, I.U.Khan (2011)
Acta Crystallogr.,Sect.E:Struct.Rep.Online ,**67,**o636

Formula: C_{24} H_{24} N_2 O_4

Compound Name: 2,2'-((4,5-Dimethyl-1,2-phenylene)bis(nitrilomethylylidene))bis(3-methoxyphenol)

Space Group:	P-1	***Cell:***	***a*** 8.031(0)	***b*** 12.584(0)	***c*** 20.617(0)
Space Group No.:	2	***(Å,°)***	α 86.90(0)	β 82.55(0)	γ 81.81(0)

R-Factor (%): 5.29 ***Temperature(K):*** 296 ***Density(g/cm3):*** 1.315

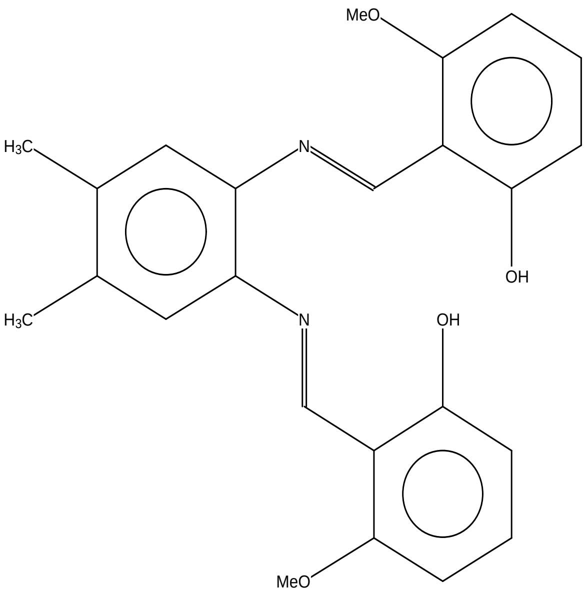

VEFTEG

Reference: P.M.Dominiak, A.Makal, P.R.Mallinson, K.Trzcinska, J.Eilmes, E.Grech, M.Chruszcz, W.Minor, K.Wozniak (2006)
Chem.-Eur.J. ,**12,**1941

Formula: C_{20} H_{16} N_2 O_4

Compound Name: N,N'-bis(2,5-Dihydroxybenzylidene)benzene-1,2-amine

Space Group:	P-1	***Cell:***	***a*** 8.676(0)	***b*** 8.957(0)	***c*** 11.306(0)
Space Group No.:	2	***(Å,°)***	α 101.97(0)	β 108.54(0)	γ 97.17(0)

R-Factor (%): 3.13 ***Temperature(K):*** 100 ***Density(g/cm3):*** 1.451

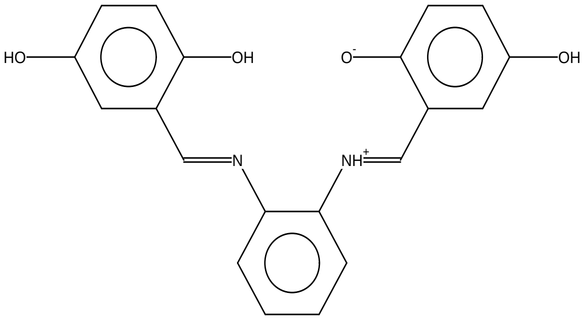

WACSAW

Reference: H.Kargar, R.Kia, M.N.Tahir (2010)
Acta Crystallogr.,Sect.E:Struct.Rep.Online ,**66,**o3110

Formula: C_{30} H_{38} N_4 O_2,C_{30} H_{38} N_4 O_2

Compound Name: 5,5'-bis(Diethylamino)-2,2'-(4,5-dimethyl-o-phenylenebis(nitrilomethylidyne))diphenol (Z)-3-diethylamino-6-(((2-((E)-4-(diethylamino)-2-hydroxybenzylideneamino)-4,5-dimethylphenyl)amino) methylidene)cyclohexa-2,4-dienone

Space Group:	P-1	***Cell:***	***a*** 11.443(1)	***b*** 12.025(1)	***c*** 22.171(2)
Space Group No.:	2	***(Å,°)***	α 88.24(0)	β 89.37(0)	γ 65.21(0)

R-Factor (%): 10.61 ***Temperature(K):*** 296 ***Density(g/cm3):*** 1.168

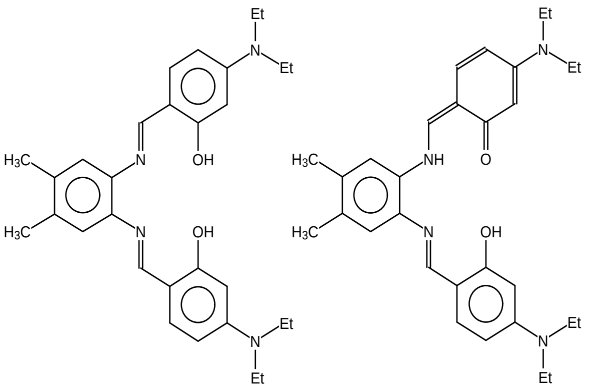

WINPAL

Reference: M.H.Habibi, M.Montazerozohori, R.Mokhtari, R.W.Harrington, W.Clegg (2007)
Acta Crystallogr.,Sect.E:Struct.Rep.Online ,**63,**o4462

Formula: C_{22} H_{20} N_2 O_4

Compound Name: 3,3'-Dimethoxy-2,2'-(1,2-phenylenebis(nitrilomethylidyne))diphenol

Synonym: N,N'-bis(6-methoxysalicylidene)-1,2-diaminobenzene

Space Group:	P-1	***Cell:***	***a*** 8.377(0)	***b*** 11.152(1)	***c*** 11.258(1)
Space Group No.:	2	***(Å,°)***	α 101.85(0)	β 108.36(0)	γ 107.08(0)

R-Factor (%): 3.86 ***Temperature(K):*** 150 ***Density(g/cm3):*** 1.387

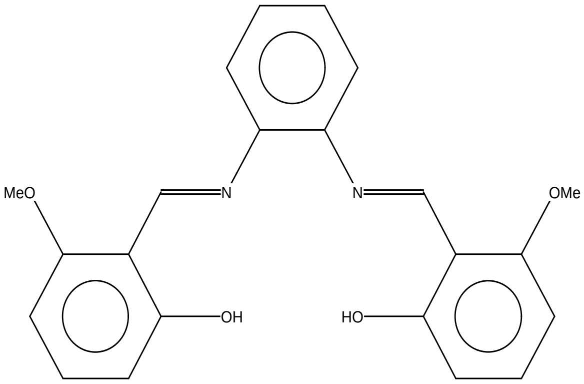

Search: search1 (Tue Dec 2 16:18:38 2014): Hits 61-64

WOFDOL

Reference: D.Homden, C.Redshaw, J.A.Wright, D.L.Hughes, M.R.J.Elsegood (2008) *Inorg.Chem.* ,**47**,5799

Formula: $C_{86}\ H_{116}\ N_2\ O_6$,1.5($C_2\ H_3\ N_1$)

Compound Name: 1,2-bis(2,2'-methylenebis(4,6-di-t-butylphenol)-5-t-butylsalicylidene) benzyldiimine acetonitrile solvate

Space Group:	P-1	***Cell:***	***a*** 11.312(0)	***b*** 17.262(0)	***c*** 23.147(0)		
Space Group No.:	2	***(Å,°)***	α 108.02(0)	β 90.48(0)	γ 107.75(0)		
R-Factor (%):	5.13	***Temperature(K):***	140	***Density(g/cm³):***	1.090		

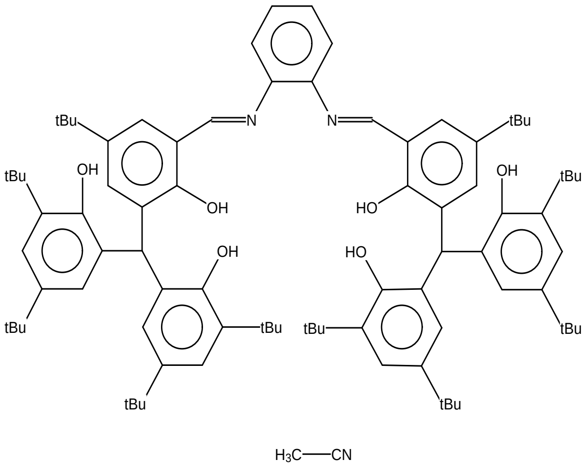

WUKYAC01

Reference: N.E.Eltayeb, S.G.Teoh, S.Chantrapromma, H.-K.Fun, R.Adnan (2008) *Acta Crystallogr.,Sect.E:Struct.Rep.Online* ,**64,**o1246

Formula: $C_{20}\ H_{16}\ N_2\ O_4$,$C_1\ H_4\ O_1$

Compound Name: 2-((E)-((2-((1E)-(2,4-Dihydroxybenzylidene)amino)phenyl))iminiomethyl)-5-hydroxyphenolate methanol solvate

Space Group:	P-1	***Cell:***	***a*** 8.367(0)	***b*** 11.081(0)	***c*** 20.322(0)		
Space Group No.:	2	***(Å,°)***	α 89.31(0)	β 80.31(0)	γ 79.64(0)		
R-Factor (%):	5.61	***Temperature(K):***	100	***Density(g/cm³):***	1.383		

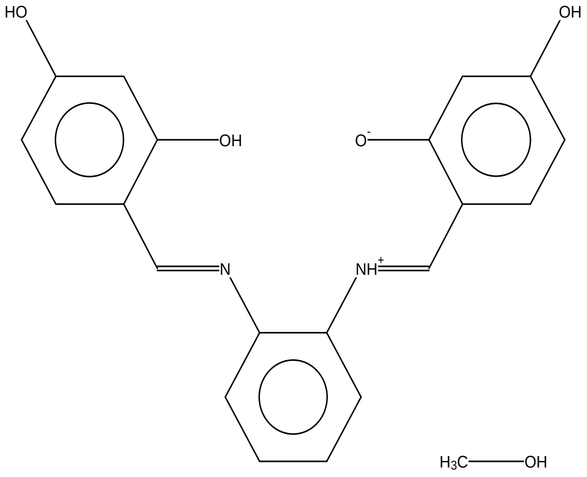

XIFREK

Reference: N.E.Eltayeb, S.G.Teoh, S.Chantrapromma, Hoong-Kun Fun, K.Ibrahim (2007) *Acta Crystallogr.,Sect.E:Struct.Rep.Online* ,**63,**o3094

Formula: $C_{22}\ H_{20}\ N_2\ O_4$

Compound Name: 5,5'-Dimethoxy-2,2'-(1,2-phenylenebis(nitrilomethylidyne))diphenol

Space Group:	P21/c	***Cell:***	***a*** 6.204(0)	***b*** 17.851(0)	***c*** 16.622(0)		
Space Group No.:	14	***(Å,°)***	α 90.00	β 91.07(0)	γ 90.00		
R-Factor (%):	5.28	***Temperature(K):***	100	***Density(g/cm³):***	1.358		

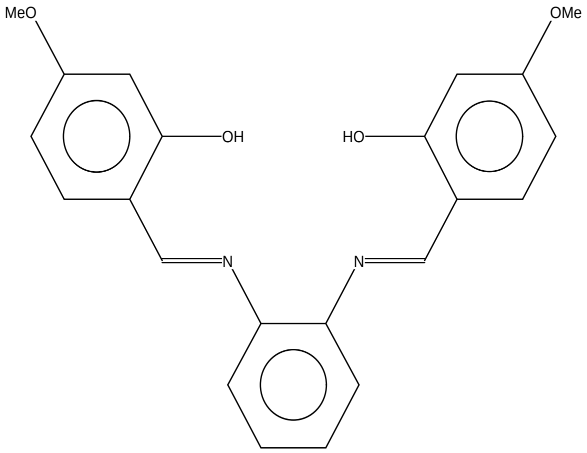

XOFSOA

Reference: H.Shimakoshi, H.Takemoto, I.Aritome, Y.Hisaeda (2002) *Tetrahedron Lett.* ,**43,**4809

Formula: $C_{58}\ H_{64}\ N_4\ O_4$,$H_2\ O_1$

Compound Name: N,N:N',N'-bis(3,3'-methylenebis(5-t-butylsalicylidene))benzene-1,2-diamine monohydrate

Space Group:	P-1	***Cell:***	***a*** 10.117(0)	***b*** 11.875(0)	***c*** 12.994(0)		
Space Group No.:	2	***(Å,°)***	α 114.86(0)	β 92.66(0)	γ 113.78(0)		
R-Factor (%):	5.81	***Temperature(K):***	173	***Density(g/cm³):***	1.193		

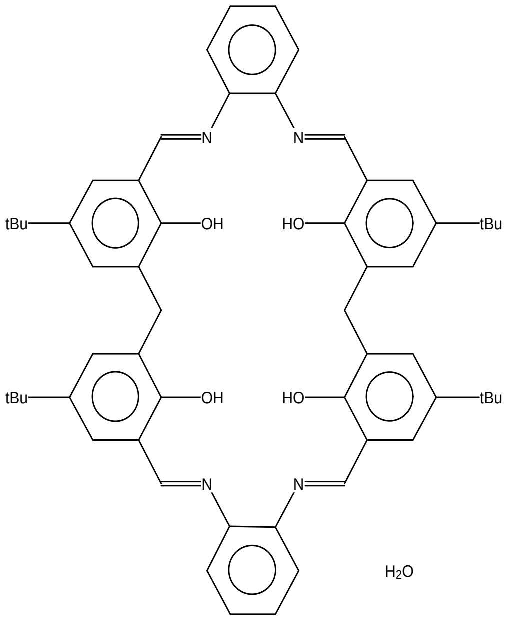

Search: search1 (Tue Dec 2 16:18:38 2014): Hits 65-68

YASWAR

Reference: P.Plitt, F.Rominger, R.Kramer (2005)
Acta Crystallogr.,Sect.C:Cryst.Struct.Commun. ,**61,**o396

Formula: $C_{40} H_{28} N_4 O_4$,4($C_2 H_6 O_1 S_1$)

Compound Name: N,N:N',N'-bis(2,2'-Dihydroxybiphenyl-3,3'-dimethylidene)benzene-1,2-diamine dimethyl sulfoxide solvate

Synonym: 8,15,28,35-Tetra-azaheptacyclo(35.3.1.$1^{2,6}$.$1^{17,21}$.$1^{22,26}$.$0^{9,14}$.$O^{29,34}$)tetraconta-41,42,43,44-tetrol dimethyl sulfoxide solvate

Space Group:	P-1	***Cell:***	***a*** 10.488(0)	***b*** 11.257(0)	***c*** 12.500(0)		
Space Group No.:	2	***(Å,°)***	α 99.60(0)	β 110.87(0)	γ 112.70(0)		
R-Factor (%):	4.91	***Temperature(K):***	200	***Density(g/cm³):***	1.313		

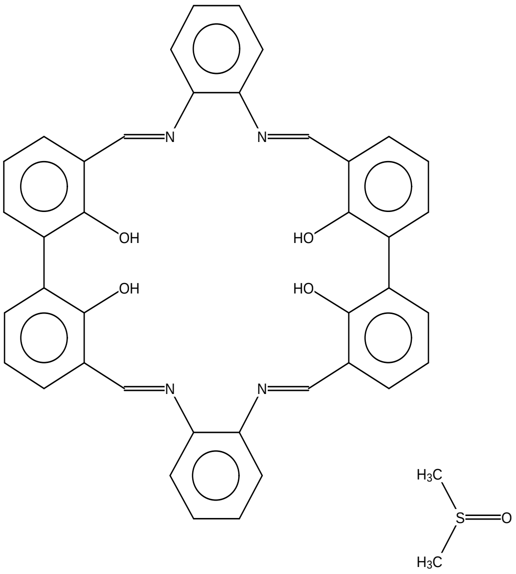

YICHAT

Reference: Y.Elerman, A.Elmali, M.Kabak, M.Aydin, M.Peder (1994)
J.Chem.Cryst. ,**24,**603

Formula: $C_{20} H_{14} Cl_2 N_2 O_2$

Compound Name: N,N'-bis(4-Chlorosalicylideneamine)-1,2-diaminobenzene

Space Group:	Pbca	***Cell:***	***a*** 7.532(3)	***b*** 18.563(2)	***c*** 25.089(3)		
Space Group No.:	61	***(Å,°)***	α 90.00	β 90.00	γ 90.00		
R-Factor (%):	4.90	***Temperature(K):***	295	***Density(g/cm³):***	1.459		

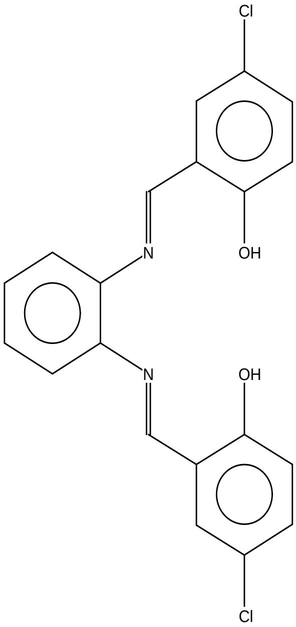

DIDRAL

Reference: D.Durak, Ani Delikanl, C.Demetgul, I.Kani, S.Serin (2013)
Transition Met.Chem. ,**38,**199

Formula: $C_{21} H_{18} N_2 O_3$

Compound Name: 2-(((2-((2-Hydroxybenzylidene)amino)phenyl)imino)methyl)-6-methoxyphenol

Space Group:	Pbca	***Cell:***	***a*** 36.989(1)	***b*** 14.129(0)	***c*** 6.294(0)		
Space Group No.:	61	***(Å,°)***	α 90.00	β 90.00	γ 90.00		
R-Factor (%):	4.74	***Temperature(K):***	100	***Density(g/cm³):***	1.399		

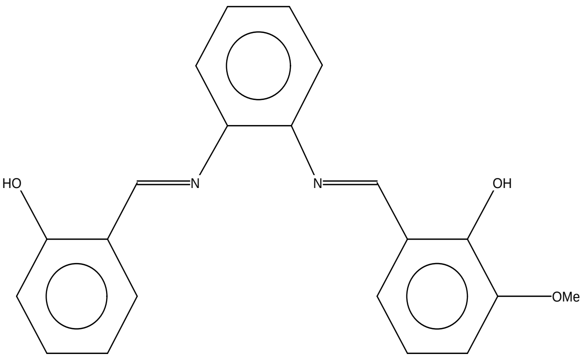

QILHIE

Reference: L.Li, S.Zeng (2013)
Acta Crystallogr.,Sect.E:Struct.Rep.Online ,**69,**o1714

Formula: $C_{25} H_{26} N_2 O_4$,$C_2 H_3 N_1$

Compound Name: 2,2'-((4-Methyl-1,2-phenylene)bis(nitrilomethylylidene))bis(6-ethoxyphenol) acetonitrile solvate

Space Group:	P21/c	***Cell:***	***a*** 11.580(3)	***b*** 24.999(7)	***c*** 8.995(3)		
Space Group No.:	14	***(Å,°)***	α 90.00	β 106.89(0)	γ 90.00		
R-Factor (%):	6.09	***Temperature(K):***	293	***Density(g/cm³):***	1.225		

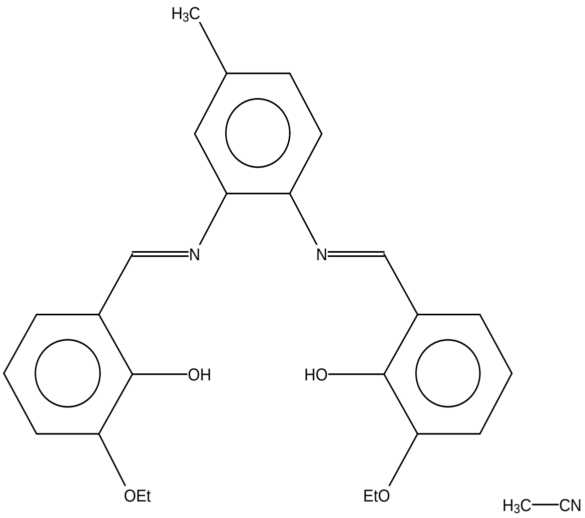

Search: search1 (Tue Dec 2 16:18:38 2014): Hits 69-71

BOFTAT

Reference: P.Biswas, P.Bag, A.K.Dutta, U.Florke, K.Nag (2014)
Polyhedron ,**75,**118

Formula: $C_{58} H_{64} N_4 O_4$

Compound Name: 5,20,26,41-tetra-t-butyl-9,16,30,37-tetraazaheptacyclo[37.3.1.1^{3,7}.1^{18,22}.1^{24,28}.0^{10,15}.0^{31,36}]hexatetraconta-1(43),3(46),4,6,8,10,12,14,16,18(45),19,21,24(44),25,27,29,31,33,35,37,39,41-docosaene-43,44,45,46-tetrol

Space Group:	P-1	***Cell:***	***a***	10.065(1)	***b***	11.837(1)	***c***	12.955(1)
Space Group No.:	2	***(Å,°)***	α	114.76(0)	β	92.67(0)	γ	113.69(0)

R-Factor (%): 5.55 ***Temperature(K):*** 120 ***Density(g/cm^3):*** 1.180

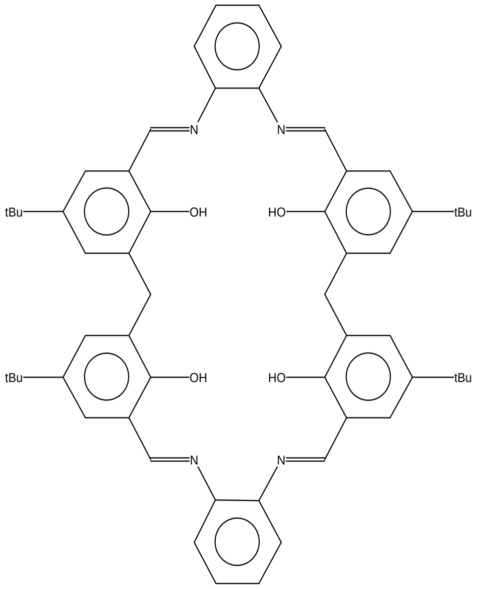

DIJNAN

Reference: S.Akine, S.Piao, M.Miyashita, T.Nabeshima (2013)
Tetrahedron Lett. ,**54,**6541

Formula: $C_{62} H_{44} N_6 O_6$,6.5($C_2 H_3 N_1$),2($C_1 H_1 Cl_3$)

Compound Name: 8,15,29,36,49,56-hexaazaundecacyclo[20.20.20.1^{2,6}.1^{17,21}.1^{23,27}.1^{38,42}.1^{43,47}.1^{58,62}.0^{9,14}.0^{30,35}.0^{50,55}]octahexaconta-2(68),3,5,7,9,11,13,15,17(67),18,20,23(66),24,26,28,30,32,34,36,38(65),39,41,43(64),44,46,48,50,52,54,56,58,60,62-tritriacontaene-63,64,65,66,67,68-hexol acetonitrile chloroform solvate

Space Group:	P21/c	***Cell:***	***a***	24.266(0)	***b***	27.445(1)	***c***	25.147(0)
Space Group No.:	14	***(Å,°)***	α	90.00	β	112.98(0)	γ	90.00

R-Factor (%): 11.50 ***Temperature(K):*** 120 ***Density(g/cm^3):*** 1.271

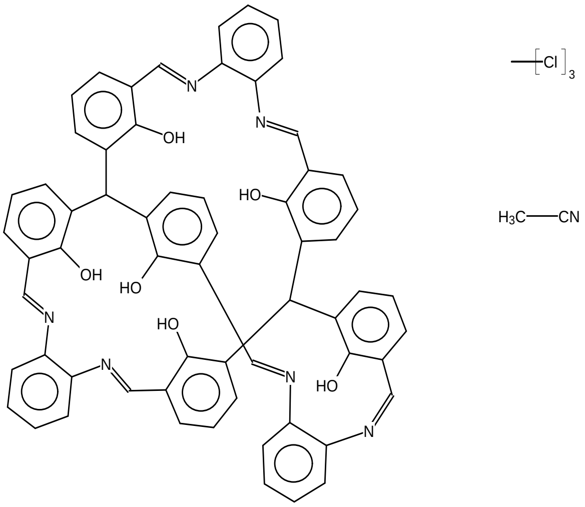

SIWLIV

Reference: S.Akine, M.Miyashita, Shunjin Piao, T.Nabeshima (2014)
Inorg.Chem.Front. ,

Formula: $C_{102} H_{108} N_6 O_{12}$,11($C_2 H_6 O_1 S_1$),3($H_2 O_1$)

Compound Name: 6,21,31,46,53,68-Hexa-t-butyl-13,14,38,39,60,61-hexamethoxy-10,17,35,42,57,64-hexa-azatridecacyclo[24.24.20.1^{3,49}.1^{4,8}.1^{19,23}.1^{24,28}.1^{29,33}.1^{44,48}.1^{51,55}.1^{66,70}.0^{11,16}.0^{36,41}.0^{58,63}]octaheptaconta-1,3(73),4(78),5,7,9,11,13,15,17,19(77),20,22,24(76),25,27,29(75),30,32,34,36,38,40,42,44(74),45,47,49,51(72),52,54,56,58,60,62,64,66,68,70-nonatriacontaene-71,72,74,75,77,78-hexol dimethylsulfoxide solvate trihydrate

Space Group:	R-3	***Cell:***	***a***	24.132(1)	***b***	24.132(1)	***c***	43.595(1)
Space Group No.:	148	***(Å,°)***	α	90.00	β	90.00	γ	120.00

R-Factor (%): 9.62 ***Temperature(K):*** 120 ***Density(g/cm^3):*** 1.144

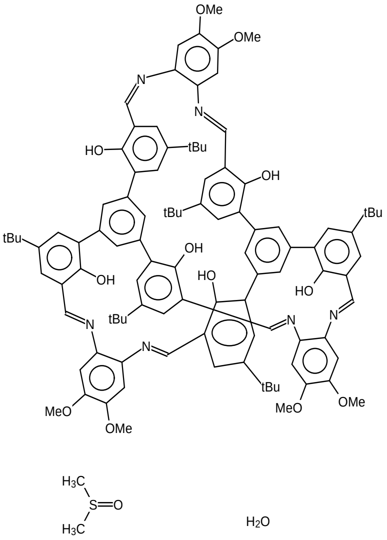

Search Overview

Search:	search26
Date/Time done:	Tue Dec 2 15:48:50 2014
Database(s):	CSD version 5.35 updates (Nov 2013)<br>CSD version 5.35 (November 2013)<br>CSD version 5.35 updates (Feb 2014)<br>CSD version 5.35 updates (May 2014)
Restriction Info:	No refcode restrictions applied
Filters:	None
Percentage Completed:	100%
Number of Hits:	772

Summary of queries used. Search found structures that:

match
Query 4

match at least one of
Query 3   Query 5

Query 3

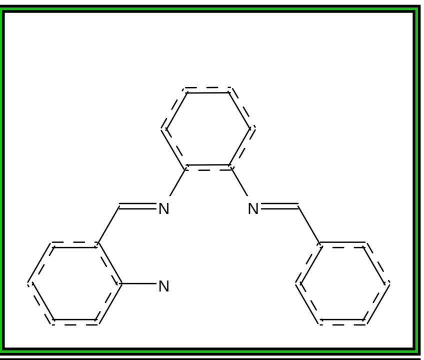

Query 4

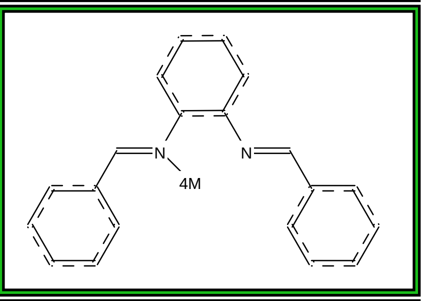

Query 5

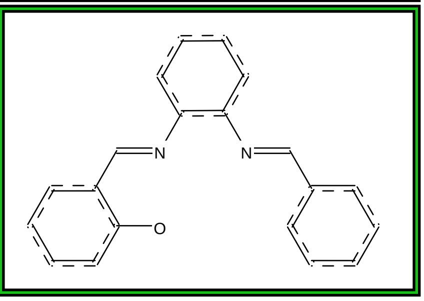

Search: search26 (Tue Dec 2 15:48:50 2014): Hits 1-4

AFATAE

Reference: H.Lin, J.-G.Wang, H.-T.Shi, Q.Chen, Q.-F.Zhang (2013) *Acta Crystallogr.,Sect.E:Struct.Rep.Online* ,**69,**m404

Formula: $C_{20}\ H_{14}\ Cl_1\ Mn_1\ N_2\ O_2, C_1\ H_4\ O_1$

Compound Name: Chloro-(2,2'-(1,2-phenylenebis((nitrilo)methylylidene))diphenolato)-manganese(iii) methanol solvate

Space Group:	P-1	***Cell:***	***a*** 7.425(0)	***b*** 9.834(0)	***c*** 13.304(0)		
Space Group No.:	2	***(Å,°)***	α 78.80(0)	β 83.31(0)	γ 86.34(0)		
R-Factor (%):	3.45	***Temperature(K):***	296	***Density(g/cm³):***	1.534		

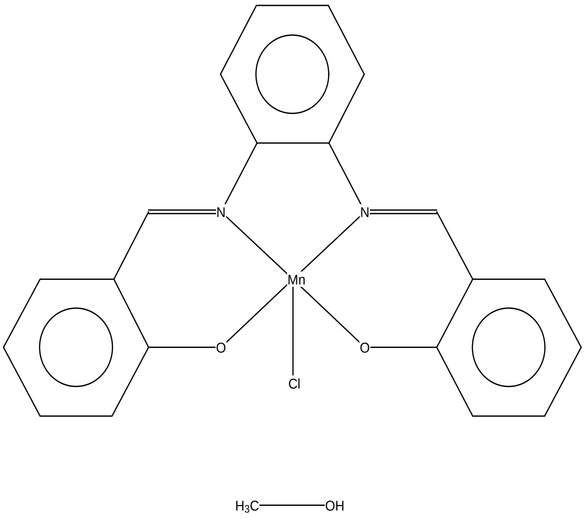

BEVZOT

Reference: A.Jana, T.Weyhermuller, S.Mohanta (2013) *CrystEngComm* ,**15,**4099

Formula: $2(C_{24}\ H_{22}\ Cu_1\ N_2\ O_4), Cl_1\ O_4\ ^{1-}, H_3\ O_1\ ^{1+}, H_2\ O_1$

Compound Name: (Oxonium monohydrate) perchlorate bis[(2,2'-(1,2-phenylenebis((nitrilo) methylylidene))bis(6-ethoxyphenolato))-copper]

Space Group:	P-1	***Cell:***	***a*** 10.487(1)	***b*** 13.908(2)	***c*** 17.158(3)		
Space Group No.:	2	***(Å,°)***	α 67.38(0)	β 89.53(0)	γ 81.19(0)		
R-Factor (%):	4.44	***Temperature(K):***	296	***Density(g/cm³):***	1.557		

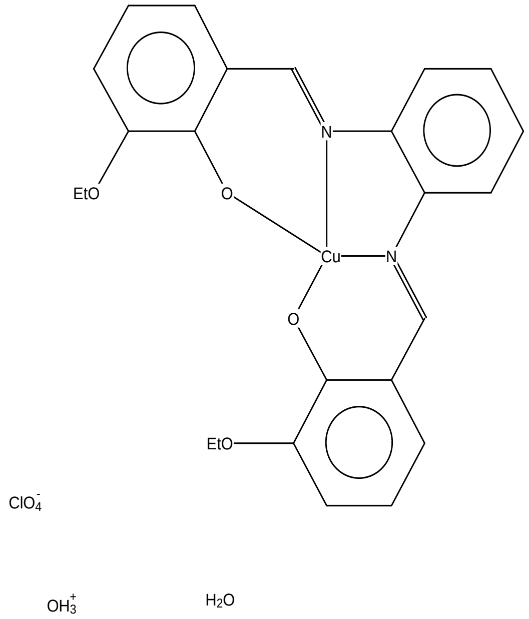

FEVDOB

Reference: Hailong Wang, Wei Cao, Tao Liu, Chunying Duan, Jianzhuang Jiang (2013) *Chem.-Eur.J.* ,**19,**2266

Formula: $C_{110}\ H_{76}\ Ca_1\ Dy_2\ N_{20}\ O_{10}, 7(C_1\ H_1\ Cl_3), C_1\ H_4\ O_1$

Compound Name: bis(μ_2-N,N'-bis(3-Methoxysalicylidene)benzene-1,2-diamine)-bis(methanol)-bis(phthalocyaninato)-calcium-di-dysprosium chloroform methanol solvate

Space Group:	P21/n	***Cell:***	***a*** 19.995(0)	***b*** 22.148(0)	***c*** 27.319(0)		
Space Group No.:	14	***(Å,°)***	α 90.00	β 90.59(0)	γ 90.00		
R-Factor (%):	8.85	***Temperature(K):***	120	***Density(g/cm³):***	1.686		

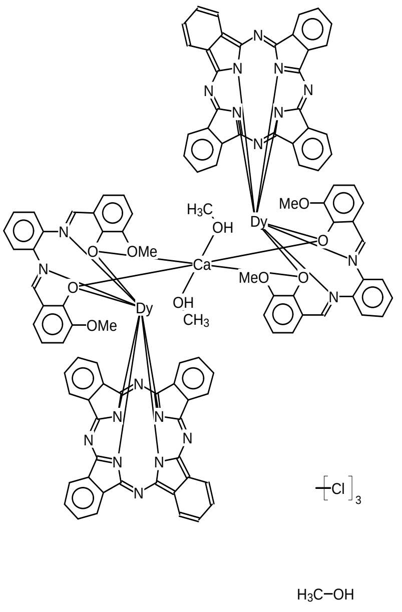

FEVDUH

Reference: Hailong Wang, Wei Cao, Tao Liu, Chunying Duan, Jianzhuang Jiang (2013) *Chem.-Eur.J.* ,**19,**2266

Formula: $C_{55}\ H_{38}\ Dy_1\ K_1\ N_{10}\ O_5$

Compound Name: bis(μ_2-N,N'-bis(3-Methoxysalicylidene)benzene-1,2-diamine)-methanol-(phthalocyaninato)-dysprosium-potassium

Space Group:	Pbcm	***Cell:***	***a*** 8.423(0)	***b*** 28.029(0)	***c*** 18.902(0)		
Space Group No.:	57	***(Å,°)***	α 90.00	β 90.00	γ 90.00		
R-Factor (%):	3.25	***Temperature(K):***	150	***Density(g/cm³):***	1.668		

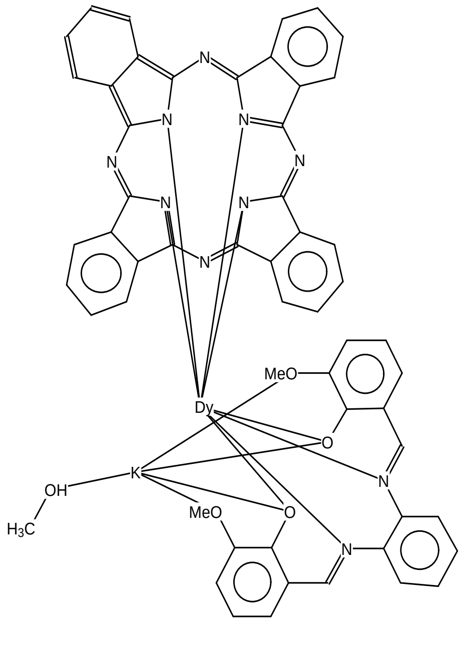

FIJHAJ

Reference: Chuan Chen, Han Chen, Pengfei Yan, Guangfeng Hou, Guangming Li (2013) *Inorg.Chim.Acta* ,**405,**182

Formula: C_{44} H_{36} Ce_{1} N_{4} O_{8},2(C_{1} H_{2} Cl_{2})

Compound Name: bis(2,2'-(1,2-phenylenebis((nitrilo)methylylidene))bis(6-methoxyphenolato))-cerium dichloromethane solvate

Space Group: C2/c ***Cell:*** ***a*** 15.487(0) ***b*** 20.214(0) ***c*** 17.226(0)
Space Group No.: 15 ***(Å,°)*** α 90.00 β 109.01(0) γ 90.00

R-Factor (%): 3.17 ***Temperature(K):*** 296 ***Density(g/cm3):*** 1.379

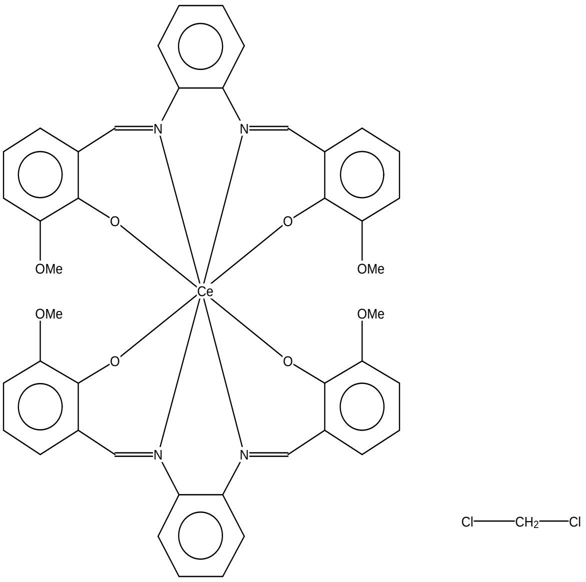

FIJHEN

Reference: Chuan Chen, Han Chen, Pengfei Yan, Guangfeng Hou, Guangming Li (2013) *Inorg.Chim.Acta* ,**405,**182

Formula: C_{44} H_{36} Ce_{1} N_{4} O_{8},C_{2} H_{6} O_{1}

Compound Name: Bis(2,2'-(1,2-phenylenebis((nitrilo)methylylidene))bis(6-methoxyphenolato))-cerium ethanol solvate

Space Group: C2/c ***Cell:*** ***a*** 21.786(4) ***b*** 13.855(3) ***c*** 14.914(3)
Space Group No.: 15 ***(Å,°)*** α 90.00 β 109.64(3) γ 90.00

R-Factor (%): 2.39 ***Temperature(K):*** 291 ***Density(g/cm3):*** 1.465

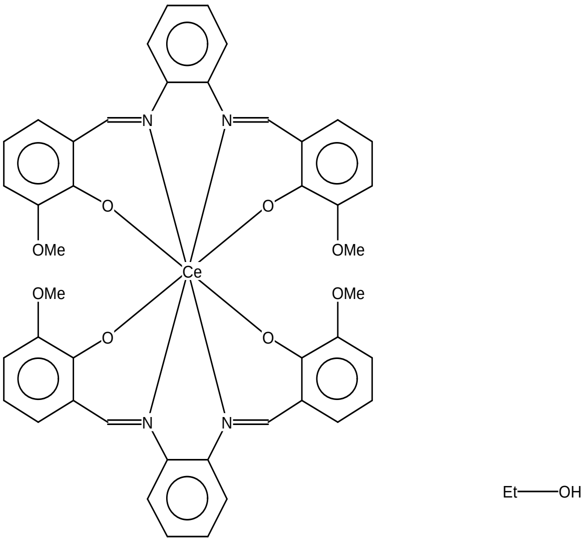

FIJVAX

Reference: Chuan Chen, Han Chen, Pengfei Yan, Guangfeng Hou, Guangming Li (2013) *Inorg.Chim.Acta* ,**405,**182

Formula: C_{44} H_{36} Ce_{1} N_{4} O_{8},2(H_{4} N_{1} $^{1+}$),2(F_{6} P_{1} $^{1-}$)

Compound Name: diammonium bis(hexafluorophosphate) bis(2,2'-(1,2-phenylenebis((nitrilo) methylylidene))bis(6-methoxyphenolato))-cerium

Space Group: P2/c ***Cell:*** ***a*** 6.907(1) ***b*** 14.395(3) ***c*** 24.221(5)
Space Group No.: 13 ***(Å,°)*** α 90.00 β 98.10(3) γ 90.00

R-Factor (%): 4.73 ***Temperature(K):*** 293 ***Density(g/cm3):*** 1.692

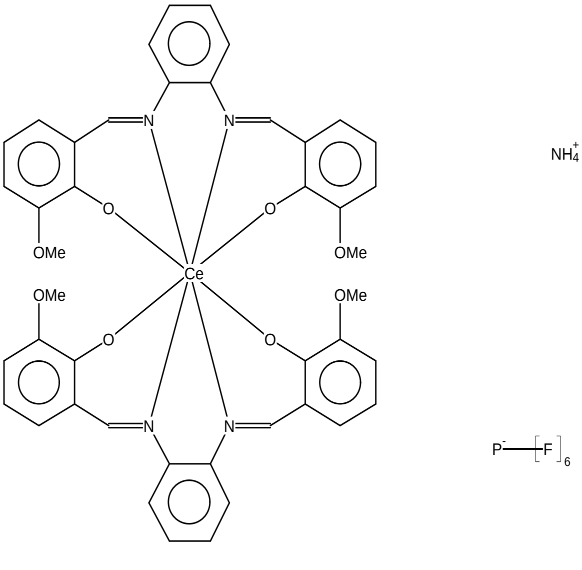

LEXXET

Reference: I.Jacobs, A.C.T.van Duin, A.W.Kleij, M.Kuil, D.M.Tooke, A.L.Spek, J.N.H.Reek (2013) *Cat.Sci.Tech.* ,**3,**1955

Formula: C_{99} H_{102} N_{9} O_{6} P_{1} Zn_{3}

Compound Name: (3,3',3''-(tris(μ_3-2,2'-(1,2-Phenylenebis(nitrilomethylylidene))bis(6-t-butylphenol)-zinc)pyridine))phosphine unknown solvate

Space Group: P-3c1 ***Cell:*** ***a*** 20.864(0) ***b*** 20.864(0) ***c*** 52.949(1)
Space Group No.: 165 ***(Å,°)*** α 90.00 β 90.00 γ 120.00

R-Factor (%): 5.38 ***Temperature(K):*** 150 ***Density(g/cm3):*** 1.159

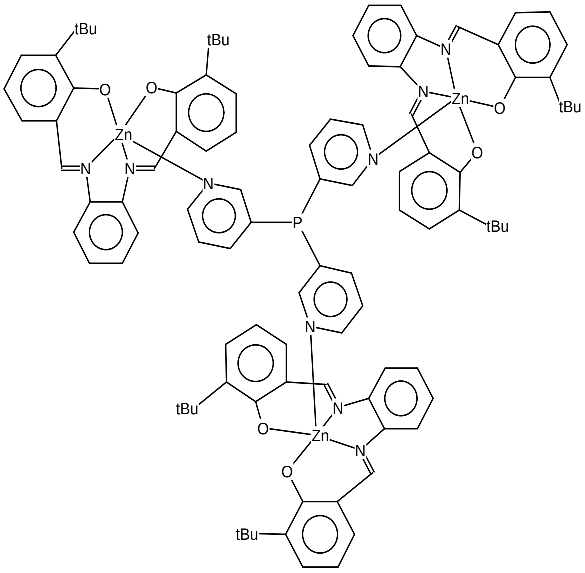

Search: search26 (Tue Dec 2 15:48:50 2014): Hits 9-12

REPPEJ

Reference: C.Bejger, Yong-Hui Tian, B.J.Barker, K.S.Boland, B.L.Scott, E.R.Batista, S.A.Kozimor, J.L.Sessler (2013) *Dalton Trans.* , **42,**6716

Formula: $C_{31} H_{30} N_2 O_5 S_6 U_1,0.5(C_1 H_2 Cl_2)$

Compound Name: (2,2'-((2-(4,5-Bis(propylsulfanyl)-1,3-dithiol-2-ylidene)-1,3-benzodithiole-5,6-diyl)bis((nitrilo)methylylidene))diphenolato)-methanol-dioxo-uranium dichloromethane solvate

Space Group:	P-1	***Cell:***	***a*** 9.714(0)	***b*** 12.038(0)	***c*** 16.561(0)
Space Group No.:	2	***(Å,°)***	α 82.01(0)	β 75.10(0)	γ 68.09(0)

R-Factor (%): 2.44 ***Temperature(K):*** 120 ***Density(g/cm³):*** 1.883

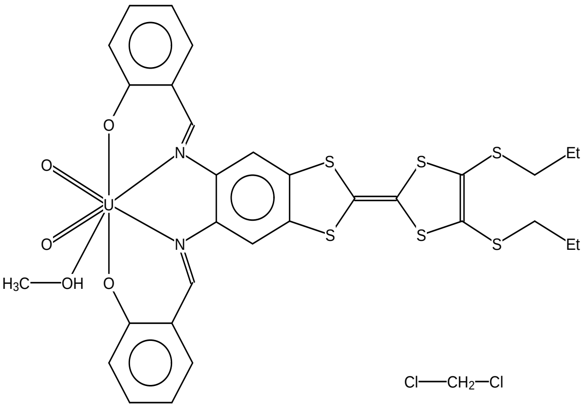

REQTUE

Reference: D.Anselmo, G.Salassa, E.C.Escudero-Adan, E.Martin, A.W.Kleij (2013) *Dalton Trans.* ,**42,**7962

Formula: $C_{62} H_{50} N_4 O_6 S_2 Zn_2,2(C_2 H_6 O_1 S_1)$

Compound Name: (μ_2-1,2,4,5-tetrakis(((2-oxybiphenyl-3-yl)methylene)amino)benzene)-bis(dimethyl sulfoxide)-di-zinc dimethyl sulfoxide solvate

Space Group:	P-1	***Cell:***	***a*** 9.148(0)	***b*** 10.469(0)	***c*** 17.615(1)
Space Group No.:	2	***(Å,°)***	α 98.41(0)	β 97.46(0)	γ 114.97(0)

R-Factor (%): 5.89 ***Temperature(K):*** 100 ***Density(g/cm³):*** 1.459

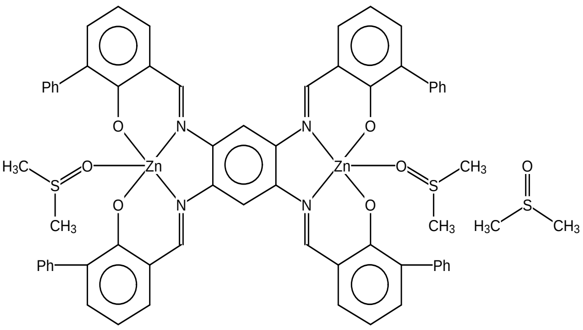

SEWHOT

Reference: Zhengqing Guo, Shek-Man Yiu, M.C.W.Chan (2013) *Chem.-Eur.J.* ,**19,**8937

Formula: $C_{60} H_{48} N_4 O_4 Pt_2,C_4 H_8 O_2,H_2 O_1$

Compound Name: (μ_2-2-(((4-(8-(3,4-bis((2-Oxybenzylidene)amino)phenyl)-3,6-di-t-butylbiphenylen-1-yl)-2-((2-oxybenzylidene)amino)phenyl)imino)methyl)phenolato)-di-platinum ethyl acetate solvate monohydrate

Space Group:	P21/n	***Cell:***	***a*** 18.154(0)	***b*** 13.877(0)	***c*** 22.422(0)
Space Group No.:	14	***(Å,°)***	α 90.00	β 109.54(0)	γ 90.00

R-Factor (%): 2.37 ***Temperature(K):*** 133 ***Density(g/cm³):*** 1.728

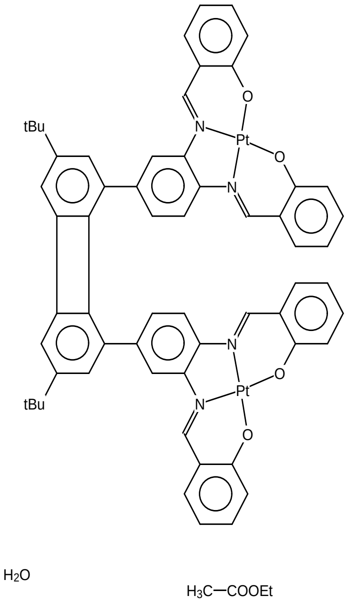

SEWHUZ

Reference: Zhengqing Guo, Shek-Man Yiu, M.C.W.Chan (2013) *Chem.-Eur.J.* ,**19,**8937

Formula: $C_{63} H_{54} N_4 O_5 Pt_2$

Compound Name: (μ_2-2-(((4-(5-(3,4-bis((2-Oxybenzylidene)amino)phenyl)-2,7-di-t-butyl-9,9-dimethyl-9H-xanthen-4-yl)-2-((2-oxybenzylidene)amino)phenyl)imino)methyl)phenolato)-di-platinum

Space Group:	P21/c	***Cell:***	***a*** 15.834(0)	***b*** 27.230(0)	***c*** 11.582(0)
Space Group No.:	14	***(Å,°)***	α 90.00	β 101.98(0)	γ 90.00

R-Factor (%): 3.00 ***Temperature(K):*** 133 ***Density(g/cm³):*** 1.818

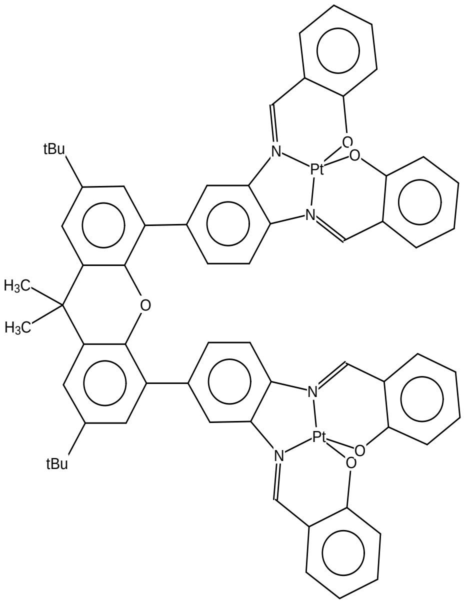

ADEKEB

Reference: D.Anselmo, E.C.Escudero-Adan, M.M.Belmonte, A.W.Kleij (2012) *Eur.J.Inorg.Chem.* ,4694

Formula: $C_{30} H_{34} N_4 O_7 Zn_1,C_2 H_3 N_1$

Compound Name: (Ethanol)-(2,2'-(1,2-phenylenebis((nitrilo)methylylidene))bis(6-t-butyl-4-nitrophenolato))-zinc(ii) acetonitrile solvate

Space Group: P21/c	***Cell:***	***a*** 10.686(0)	***b*** 16.687(0)	***c*** 17.984(0)
Space Group No.: 14	***(Å,°)***	α 90.00	β 103.53(0)	γ 90.00

R-Factor (%): 2.40 ***Temperature(K):*** 100 ***Density(g/cm^3):*** 1.425

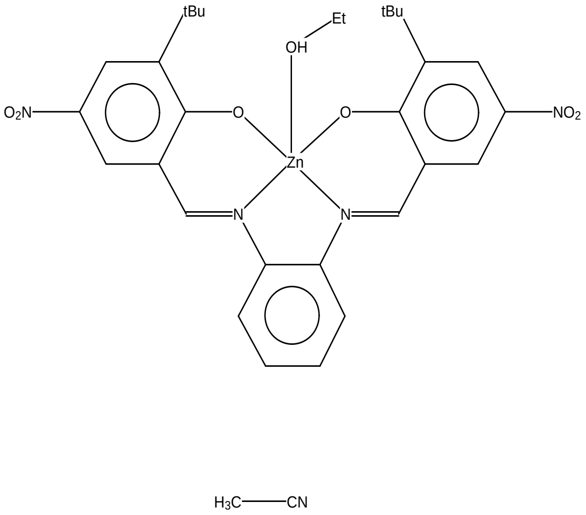

ADIBEU

Reference: Y.Wang, S.Parkin, D.Atwood (2002) *Inorg.Chem.* ,**41,**558

Formula: $C_{39} H_{53} Al_1 N_2 O_2,2(C_4 H_8 O_1)$

Compound Name: (N,N'-(4,5-Dimethyl-1,2-phenylene)-bis(3,5-di-t-butylsalicylaldiminato))-methyl-aluminium tetrahydrofuran solvate

Space Group: C2/c	***Cell:***	***a*** 35.539(6)	***b*** 10.287(1)	***c*** 27.986(5)
Space Group No.: 15	***(Å,°)***	α 90.00	β 119.97(0)	γ 90.00

R-Factor (%): 9.08 ***Temperature(K):*** 173 ***Density(g/cm^3):*** 1.129

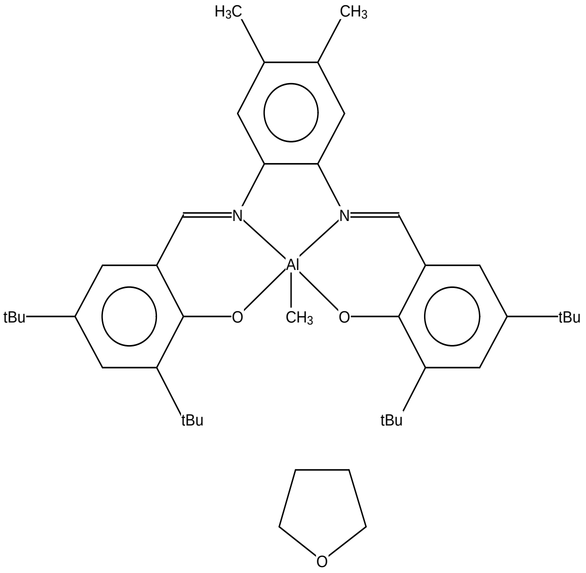

ADIZUK

Reference: M.Taherimehr, A.Decortes, S.M.Al-Amsyar, W.Lueangchaichaweng, C.J.Whiteoak, E.C.Escudero-Adan, A.W.Kleij, P.P.Pescarmona (2012) *Cat.Sci.Tech.* ,**2,**2231

Formula: $C_{40} H_{54} N_2 O_3 Zn_1$

Compound Name: (2,3-Dimethyloxirane)-(2,2'-(1,2-phenylenebis((nitrilo)methylylidene)) bis(4,6-di-t-butylphenolato))-zinc

Space Group: P-1	***Cell:***	***a*** 10.970(0)	***b*** 12.972(0)	***c*** 13.343(0)
Space Group No.: 2	***(Å,°)***	α 91.83(0)	β 104.28(0)	γ 92.66(0)

R-Factor (%): 4.31 ***Temperature(K):*** 100 ***Density(g/cm^3):*** 1.223

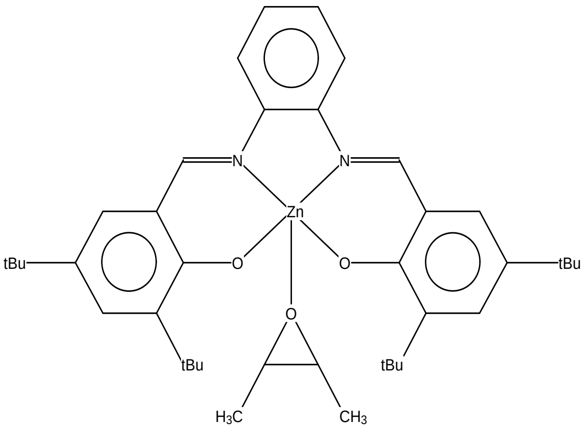

ADOBAY

Reference: M.Taherimehr, A.Decortes, S.M.Al-Amsyar, W.Lueangchaichaweng, C.J.Whiteoak, E.C.Escudero-Adan, A.W.Kleij, P.P.Pescarmona (2012) *Cat.Sci.Tech.* ,**2,**2231

Formula: $C_{33} H_{40} N_2 O_4 Zn_1,0.25(C_5 H_{10} O_2)$

Compound Name: ((3-Methyloxetan-3-yl)methanol)-(2,2'-(1,2-phenylenebis((nitrilo) methylylidene))bis(6-t-butylphenolato))-zinc (3-methyloxetan-3-yl) methanol

Space Group: C2/c	***Cell:***	***a*** 42.190	***b*** 9.719	***c*** 31.932
Space Group No.: 15	***(Å,°)***	α 90.00	β 110.08	γ 90.00

R-Factor (%): 3.93 ***Temperature(K):*** 100 ***Density(g/cm^3):*** 1.339

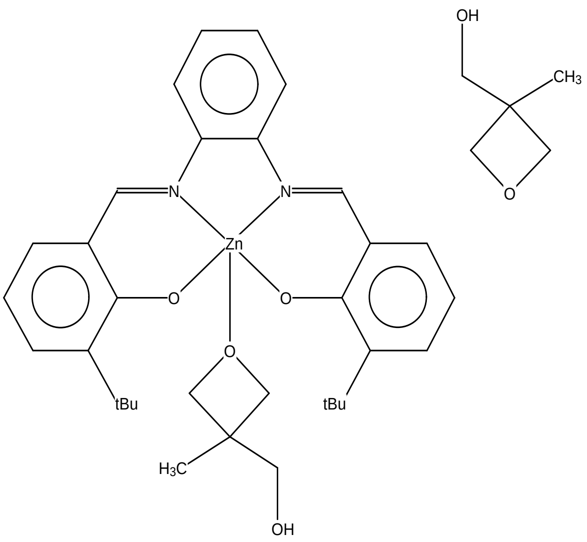

Search: search26 (Tue Dec 2 15:48:50 2014): Hits 17-20

AFEJAX

Reference: N.E.Eltayeb, S.G.Teoh, S.Chantrapromma, H.-K.Fun, K.Ibrahim (2007) *Acta Crystallogr.,Sect.E:Struct.Rep.Online* ,**63,**m2294

Formula: $C_{22} H_{20} N_2 O_3 Zn_1,C_1 H_1 Cl_3$

Compound Name: Aqua-(6,6'-dimethyl-2,2'-(o-phenylenebis(nitrilomethylidyne))diphenolato-κ4O,N,N',O')-zinc(ii) chloroform solvate

Space Group: P-1 ***Cell:*** ***a*** 9.012(0) ***b*** 10.777(0) ***c*** 12.902(0)
Space Group No.: 2 ***(Å,°)*** α 94.34(0) β 103.96(0) γ 109.85(0)

R-Factor (%): 3.66 ***Temperature(K):*** 100 ***Density(g/cm3):*** 1.607

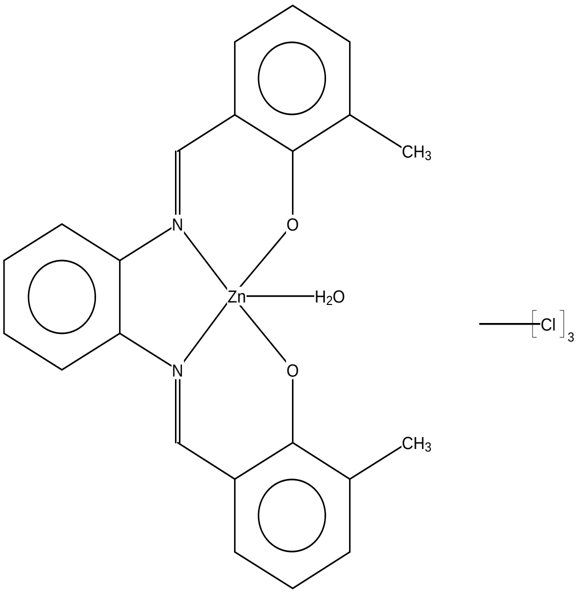

AFUBUZ

Reference: D.J.Darensbourg, A.I.Moncada, Wonsook Choi, J.H.Reibenspies (2008) *J.Am.Chem.Soc.* ,**130,**6523

Formula: $C_{75} H_{98} Cl_1 Cr_2 N_4 O_6,C_5 H_{12}$

Compound Name: (μ_2-Oxo)-chloro-bis(N,N'-bis(3,5-di-t-butylsalicylidene)-1,2-phenylenediamine-N,N',O,O')-(oxetane-O)-di-chromium(iii) n-pentane solvate

Space Group: P-1 ***Cell:*** ***a*** 12.266(5) ***b*** 14.702(5) ***c*** 21.630(5)
Space Group No.: 2 ***(Å,°)*** α 102.84(0) β 95.78(0) γ 100.25(0)

R-Factor (%): 7.55 ***Temperature(K):*** 120 ***Density(g/cm3):*** 1.223

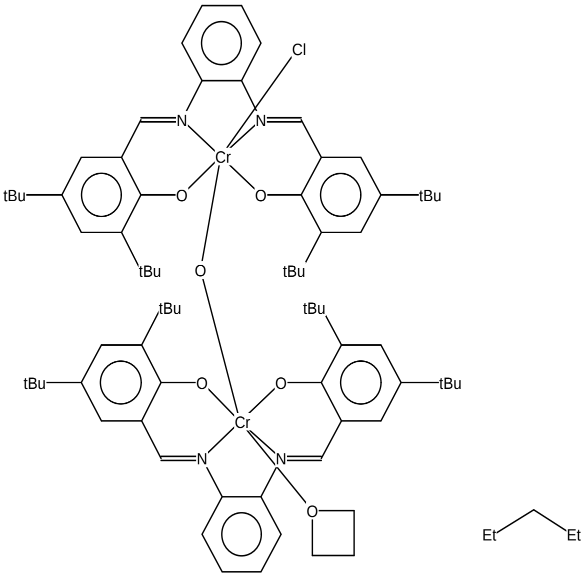

AFUCAG

Reference: D.J.Darensbourg, A.I.Moncada, Wonsook Choi, J.H.Reibenspies (2008) *J.Am.Chem.Soc.* ,**130,**6523

Formula: $C_{39} H_{52} Cl_1 Cr_1 N_2 O_3,1.5(C_1 H_2 Cl_2),0.5(C_5 H_{12})$

Compound Name: Chloro-(N,N'-bis(3,5-di-t-butylsalicylidene)-1,2-phenylenediamine-N,N',O,O')-(oxetane-O)-chromium(iii) dichloromethane n-pentane solvate

Space Group: P-1 ***Cell:*** ***a*** 16.175(5) ***b*** 16.394(5) ***c*** 17.126(6)
Space Group No.: 2 ***(Å,°)*** α 89.78(0) β 88.05(0) γ 79.25(0)

R-Factor (%): 5.79 ***Temperature(K):*** 110 ***Density(g/cm3):*** 1.263

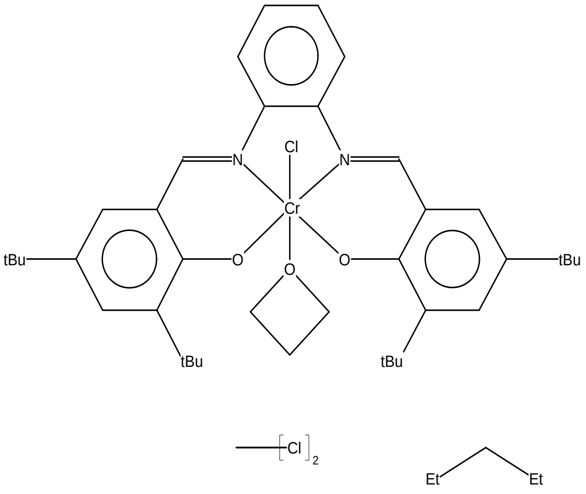

AGASOR

Reference: A.Nabei, T.Kuroda-Sowa, T.Okubo, M.Maekawa, M.Munakata (2008) *Inorg.Chim.Acta* ,**361,**3489

Formula: $(C_{52} H_{44} Fe_2 N_8 O_8)n,2n(C_1 H_2 Cl_2)$

Compound Name: catena-(bis(μ_2-Pyrazine-N,N')-bis(bis(o-vanillinato)-o-phenylenedi-imine-N,N',O,O')-di-iron(ii) dichloromethane solvate)

Space Group: P-1 ***Cell:*** ***a*** 14.310(10) ***b*** 13.700(20) ***c*** 15.180(20)
Space Group No.: 2 ***(Å,°)*** α 91.91(3) β 114.56(2) γ 89.46(4)

R-Factor (%): 18.05 ***Temperature(K):*** 280 ***Density(g/cm3):*** 1.462

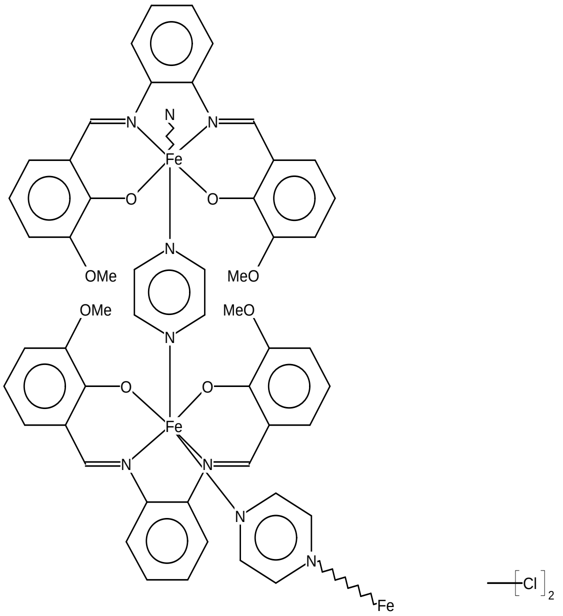

Search: search26 (Tue Dec 2 15:48:50 2014): Hits 21-24

AGASOR01

Reference: A.Nabei, T.Kuroda-Sowa, T.Okubo, M.Maekawa, M.Munakata (2008) *Inorg.Chim.Acta* ,**361,**3489

Formula: $(C_{52}\ H_{44}\ Fe_2\ N_8\ O_8)n,2n(C_1\ H_2\ Cl_2)$

Compound Name: catena-(bis(μ_2-Pyrazine-N,N')-bis(bis(o-vanillinato)-o-phenylenedi-imine-N,N',O,O')-di-iron(ii) dichloromethane solvate)

Space Group:	P-1	***Cell:***	***a***	13.430(10)	***b***	13.710(10)	***c***	15.460(10)
Space Group No.:	2	***(Å,°)***	α	91.54(1)	β	115.20(1)	γ	91.12(1)

R-Factor (%): 11.85 ***Temperature(K):*** 120 ***Density(g/cm^3):*** 1.537

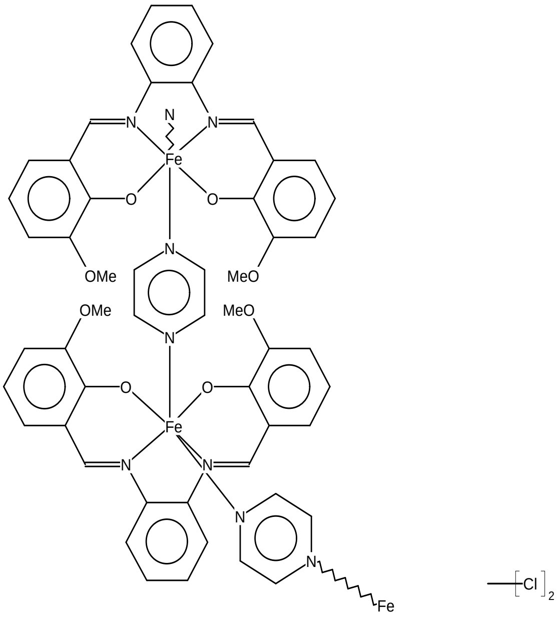

AGASUX

Reference: A.Nabei, T.Kuroda-Sowa, T.Okubo, M.Maekawa, M.Munakata (2008) *Inorg.Chim.Acta* ,**361,**3489

Formula: $(C_{30}\ H_{24}\ Fe_1\ N_4\ O_4)n,2n(C_1\ H_2\ Cl_2)$

Compound Name: catena-((μ_2-Pyrazine-N,N')-(bis(o-vanillinato)-2,3-naphthalenedi-imine-N,N',O,O')-iron(ii) dichloromethane solvate)

Space Group:	P-1	***Cell:***	***a***	7.359(6)	***b***	13.740(10)	***c***	16.130(10)
Space Group No.:	2	***(Å,°)***	α	81.44(2)	β	84.13(2)	γ	86.24(2)

R-Factor (%): 7.26 ***Temperature(K):*** 150 ***Density(g/cm^3):*** 1.514

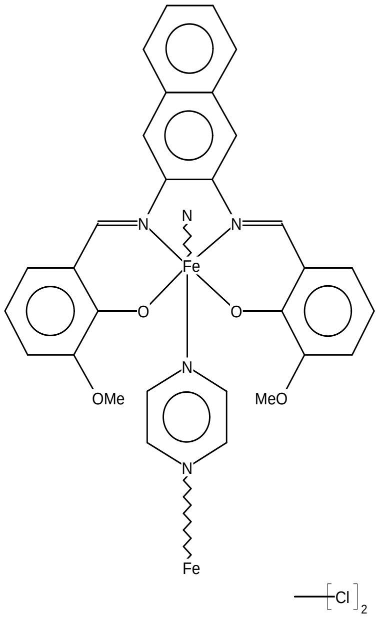

AHOFUZ

Reference: A.M.Castilla, S.Curreli, N.M.Carretero, E.Escudero-Adan, J.Benet-Buchholz, A.W.Kleij (2009) *Eur.J.Inorg.Chem.* ,2467

Formula: $C_{54}\ H_{54}\ N_6\ O_{11}\ Zn_2,0.5(C_4\ H_8\ O_1)$

Compound Name: (μ_2-4,4'-bis(3-t-Butylsalicylideneamino)-3-(5-allyl-3-methoxysalicylideneamino)-3'-(3,5-dinitrosalicylideneamino)biphenyl)-bis(methanol)-di-zinc tetrahydrofuran solvate

Space Group:	P-1	***Cell:***	***a***	13.620(0)	***b***	14.323(0)	***c***	15.181(1)
Space Group No.:	2	***(Å,°)***	α	78.44(0)	β	64.90(0)	γ	80.90(0)

R-Factor (%): 6.08 ***Temperature(K):*** 100 ***Density(g/cm^3):*** 1.433

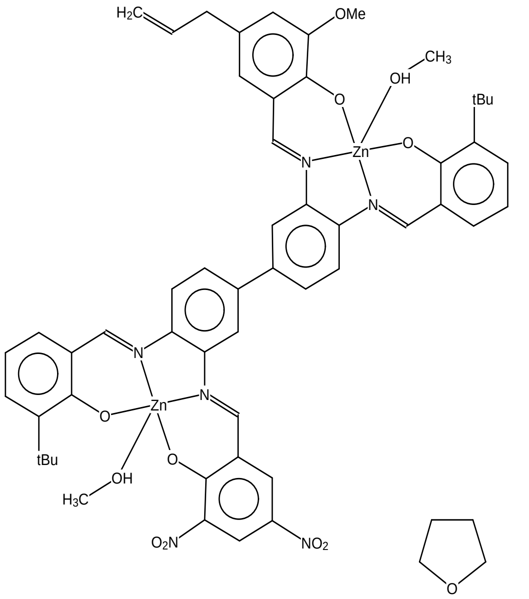

AHOKUE

Reference: Long Chen, Jangbae Kim, T.Ishizuka, Y.Honsho, A.Saeki, S.Seki, H.Ihee, Donglin Jiang (2009) *J.Am.Chem.Soc.* ,**131,**7287

Formula: $C_{66}\ H_{54}\ N_6\ O_9\ S_3\ Zn_3,8(C_2\ H_6\ O_1\ S_1)$

Compound Name: (μ_3-2,3,6,7,10,11-hexakis(Salicylideneamino)triphenylene)-tris(dimethylsulfoxide)-tri-zinc dimethylsulfoxide solvate

Space Group:	R-3	***Cell:***	***a***	24.940(30)	***b***	24.940(30)	***c***	26.910(40)
Space Group No.:	148	***(Å,°)***	α	90.00	β	90.00	γ	120.00

R-Factor (%): 13.70 ***Temperature(K):*** 153 ***Density(g/cm^3):*** 1.370

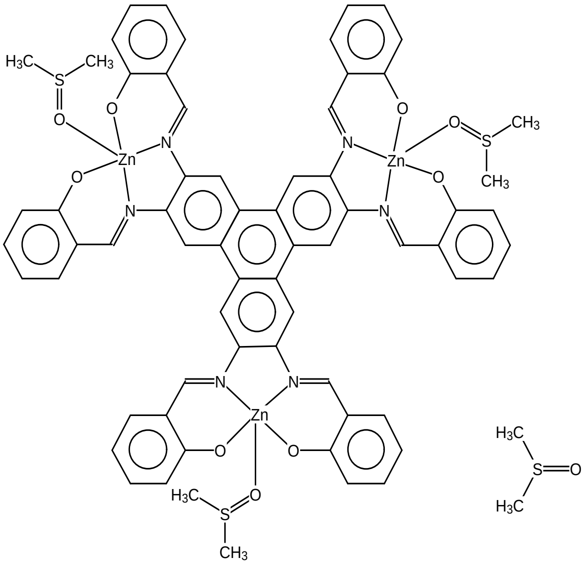

AJANUV

Reference: S.J.Wezenberg, E.C.Escudero-Adan, J.Benet-Buchholz, A.W.Kleij (2009) *Chem.-Eur.J.* ,**15**,5695

Formula: $C_{88} H_{112} N_6 O_{10} Zn_3,2(C_2 H_3 N_1)$

Compound Name: bis(μ_2-Acetato-O,O')-bis(N,N'-bis(3,5-di-t-butylsalicylidene)-1,2-phenylenediamine-N,N',O,O')-bis(2-hydroxymethylpyridine-N,O)-tri-zinc(ii) acetonitrile solvate

Space Group:	P-1	***Cell:***	***a*** 12.463(0)	***b*** 12.981(0)	***c*** 14.197(0)
Space Group No.:	2	***(Å,°)***	α 103.45(0)	β 98.84(0)	γ 95.34(0)

R-Factor (%): 3.63 ***Temperature(K):*** 100 ***Density(g/cm^3):*** 1.285

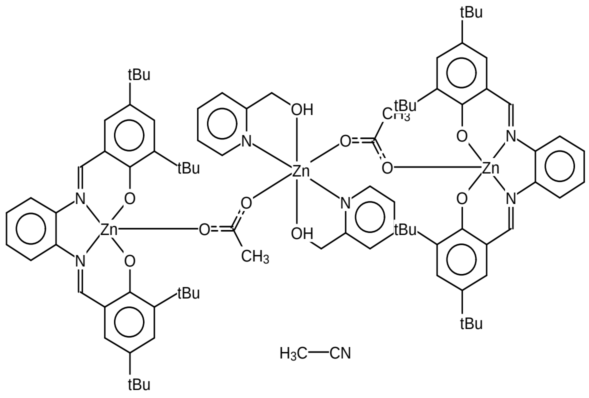

AJAPAD

Reference: S.J.Wezenberg, E.C.Escudero-Adan, J.Benet-Buchholz, A.W.Kleij (2009) *Chem.-Eur.J.* ,**15**,5695

Formula: $C_{16} H_{36} N_1{}^{1+},C_{58} H_{63} N_4 O_6 Zn_2{}^{1-},2(C_2 H_3 N_1)$

Compound Name: Tetra-n-butylammonium (μ_2-acetato-O,O')-bis(N,N'-bis(3-t-butylsalicylidene)-1,2-phenylenediamine-N,N',O,O')-di-zinc(ii) acetonitrile solvate

Space Group:	I-42d	***Cell:***	***a*** 21.611(0)	***b*** 21.611(0)	***c*** 31.249(1)
Space Group No.:	122	***(Å,°)***	α 90.00	β 90.00	γ 90.00

R-Factor (%): 3.13 ***Temperature(K):*** 100 ***Density(g/cm^3):*** 1.245

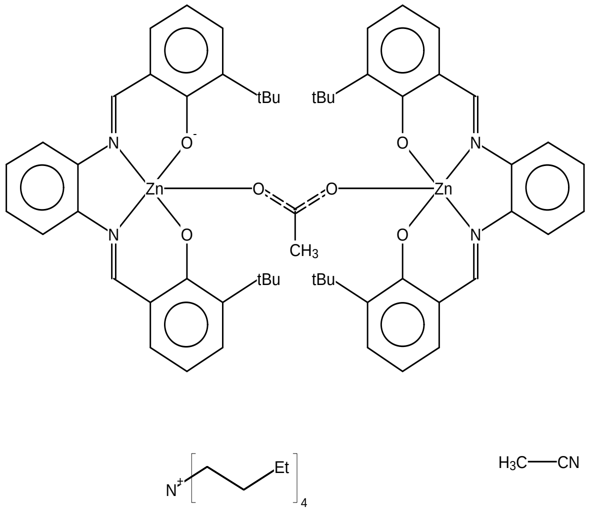

AJAPEH

Reference: S.J.Wezenberg, E.C.Escudero-Adan, J.Benet-Buchholz, A.W.Kleij (2009) *Chem.-Eur.J.* ,**15**,5695

Formula: $C_{16} H_{36} N_1{}^{1+},C_{36} H_{48} N_2 O_3 Zn_1,C_{36} H_{46} I_1 N_2 O_2 Zn_1{}^{1-},1.5(C_2 H_3 N_1)$

Compound Name: Tetra-n-butylammonium aqua-(N,N'-bis(3,5-di-t-butylsalicylidene)-1,2-phenylenediamine-N,N',O,O')-zinc(ii) iodo-(N,N'-bis(3,5-di-t-butylsalicylidene)-1,2-phenylenediamine-N,N',O,O')-zinc(ii) acetonitrile solvate

Space Group:	P-1	***Cell:***	***a*** 19.049(3)	***b*** 22.508(4)	***c*** 23.398(4)
Space Group No.:	2	***(Å,°)***	α 71.59(0)	β 71.59(0)	γ 89.23(0)

R-Factor (%): 7.07 ***Temperature(K):*** 100 ***Density(g/cm^3):*** 1.224

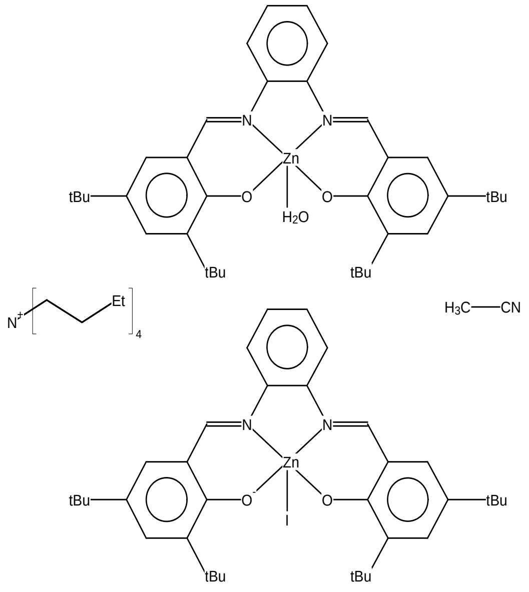

AJIYEY

Reference: Chi-Ming Che, Chi-Chung Kwok, Siu-Wai Lai, A.F.Rausch, W.J.Finkenzeller, Nianyong Zhu, H.Yersin (2010) *Chem.-Eur.J.* ,**16**,233

Formula: $C_{20} H_{14} N_2 O_2 Pt_1,C_3 H_7 N_1 O_1$

Compound Name: (N,N'-bis(2-Oxybenzylidene)phenylene-1,2-diamine)-platinum(ii) dimethylformamide solvate

Space Group:	P21/c	***Cell:***	***a*** 11.900(2)	***b*** 14.180(3)	***c*** 12.078(2)
Space Group No.:	14	***(Å,°)***	α 90.00	β 95.41(3)	γ 90.00

R-Factor (%): 4.40 ***Temperature(K):*** 295 ***Density(g/cm^3):*** 1.907

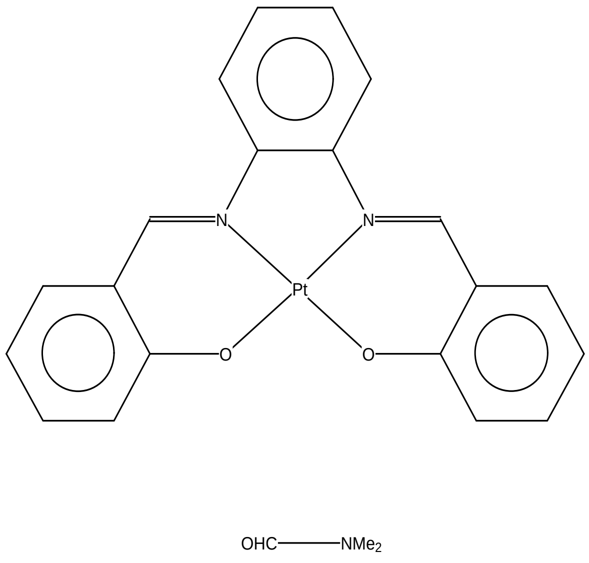

Search: search26 (Tue Dec 2 15:48:50 2014): Hits 29-32

AJIYOI

Reference: Chi-Ming Che, Chi-Chung Kwok, Siu-Wai Lai, A.F.Rausch, W.J.Finkenzeller, Nianyong Zhu, H.Yersin (2010) *Chem.-Eur.J.* ,**16,**233

Formula: $C_{24}\,H_{22}\,N_2\,O_2\,Pt_1,C_1\,H_2\,Cl_2$

Compound Name: (N,N'-bis(2-Oxybenzylidene)-4-t-butyl-phenylene-1,2-diamine)-platinum(ii) dichloromethane solvate

Space Group: P21/c ***Cell:*** ***a*** 14.946(3) ***b*** 14.046(3) ***c*** 12.410(3)
Space Group No.: 14 ***(Å,°)*** α 90.00 β 106.71(3) γ 90.00

R-Factor (%): 6.30 ***Temperature(K):*** 295 ***Density(g/cm³):*** 1.732

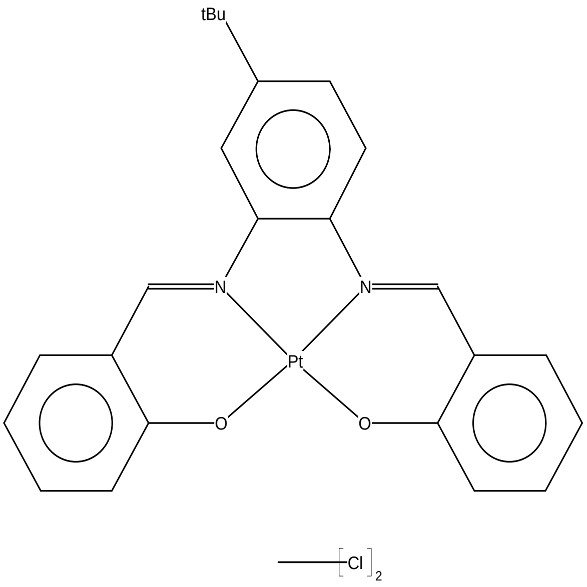

ALIBIH

Reference: S.J.Wezenberg, G.Salassa, E.C.Escudero-Adan, J.Benet-Buchholz, A.W.Kleij (2011) *Angew.Chem.,Int.Ed.* ,**50,**713

Formula: $C_{50}\,H_{46}\,N_4\,O_6\,Zn_2,2(C_2\,H_3\,N_1),C_7\,H_8$

Compound Name: (μ_2-Acetic acid-O,O')-(μ_2-3,3'-bis(((2-(((3-t-butyl-2-oxidophenyl) methylene)amino)phenyl)imino)methyl)-1,1'-biphenyl-2,2'-diolato)-di-zinc(ii) acetonitrile toluene solvate

Space Group: P21/c ***Cell:*** ***a*** 12.695 ***b*** 32.852 ***c*** 13.299
Space Group No.: 14 ***(Å,°)*** α 90.00 β 110.20 γ 90.00

R-Factor (%): 7.21 ***Temperature(K):*** 100 ***Density(g/cm³):*** 1.409

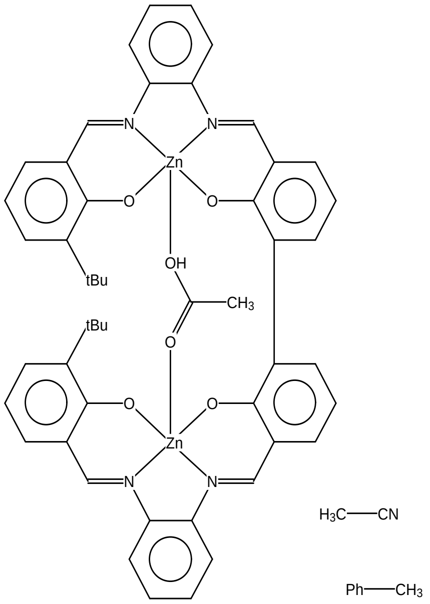

ALIBON

Reference: S.J.Wezenberg, G.Salassa, E.C.Escudero-Adan, J.Benet-Buchholz, A.W.Kleij (2011) *Angew.Chem.,Int.Ed.* ,**50,**713

Formula: $C_{57}\,H_{52}\,N_4\,O_6\,Zn_2,C_2\,H_3\,N_1$

Compound Name: (μ_2-rac-3,3'-bis(((2-(((3-t-Butyl-2-oxidophenyl)methylene)amino)phenyl) imino)methyl)-1,1'-biphenyl-2,2'-diolato)-(μ_2-(S)-2-phenylpropanoic acid-O,O')-di-zinc(ii) acetonitrile solvate

Space Group: P21 ***Cell:*** ***a*** 13.131(0) ***b*** 29.830(1) ***c*** 13.593(0)
Space Group No.: 4 ***(Å,°)*** α 90.00 β 110.19(0) γ 90.00

R-Factor (%): 5.63 ***Temperature(K):*** 100 ***Density(g/cm³):*** 1.410

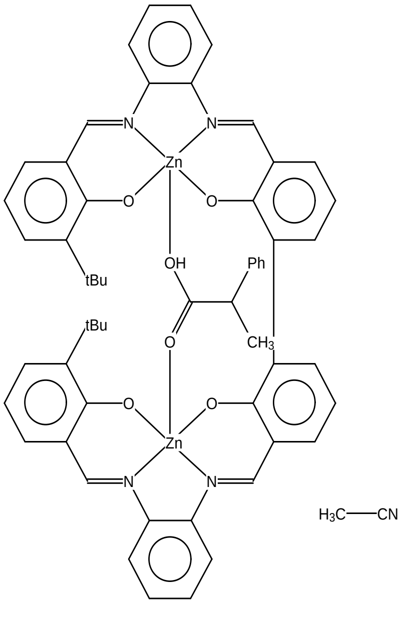

ALUSOQ

Reference: Gui Chen, Wai-Lun Man, Shek-Man Yiu, Tsz-Wing Wong, L.Szeto, Wing-Tak Wong, Tai-Chu Lau (2011) *Dalton Trans.* ,**40,**1938

Formula: $C_{74}\,H_{56}\,Cl_2\,Fe_1\,N_6\,O_4\,Os_2\,P_2$

Compound Name: (μ_2-1,1'-bis((imido)diphenylphosphanyl)ferrocene)-dichloro-bis(2,2'-(1,2-phenylenebis(nitrilomethylylidene))diphenolato)-di-osmium(iv)

Space Group: P-1 ***Cell:*** ***a*** 11.698(4) ***b*** 12.230(4) ***c*** 13.216(4)
Space Group No.: 2 ***(Å,°)*** α 85.21(0) β 69.35(0) γ 64.35(0)

R-Factor (%): 3.67 ***Temperature(K):*** 298 ***Density(g/cm³):*** 1.737

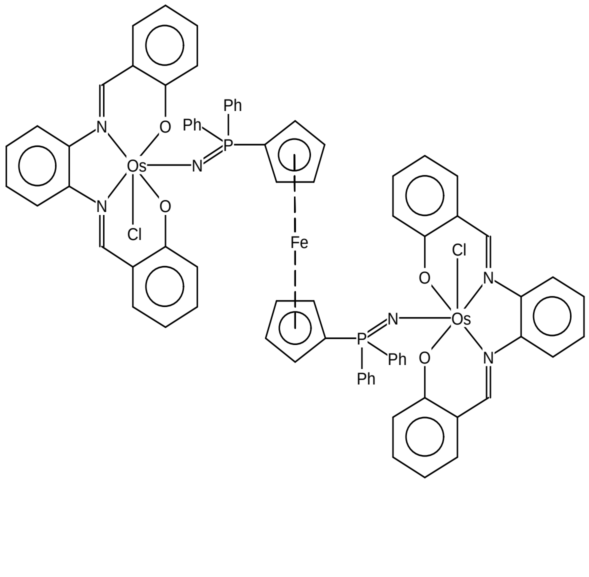

Search: search26 (Tue Dec 2 15:48:50 2014): Hits 33-36

APDSAL

Reference: G.D.Fallon, B.M.Gatehouse (1976)
Acta Crystallogr.,Sect.B:Struct.Crystallogr.Cryst.Chem. ,**32,**2591

Formula: $C_{36}\ H_{30}\ N_4\ O_4\ Pd_2$

Compound Name: (μ_2-N,N'-o-Phenylenebis(salicylaldiminato)-N,O)-bis((2-acetophenone oxime-C,N)-palladium(ii))

Space Group: P21/c | ***Cell:*** ***a*** 7.109(4) ***b*** 25.321(13) ***c*** 17.979(8)
Space Group No.: 14 | ***(Å,°)*** α 90.00 β 107.80(40) γ 90.00

R-Factor (%): 6.90 ***Temperature(K):*** 295 ***Density(g/cm³):*** 1.715

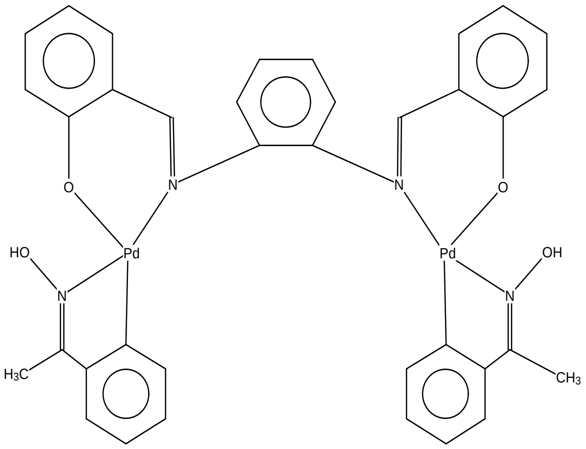

ASEROG

Reference: Chun Yang, Qing-Lun Wang, Yue Ma, Guo-Tao Tang, Dai-Zheng Liao, Shi-Ping Yan, Guang-Ming Yang, Peng Cheng (2010)
Inorg.Chem. ,**49,**2047

Formula: $C_{45}\ H_{32}\ Fe_1\ Mn_2\ N_{10}\ O_7,2(C_1\ H_4\ O_1)$

Compound Name: bis(μ_2-cyano)-nitrosyl-tricyano-diaqua-bis(2,2'-(1,2-phenylenebis(nitrilomethylylidene))diphenolato)-iron-di-manganese methanol solvate

Space Group: P-1 | ***Cell:*** ***a*** 10.854(2) ***b*** 11.239(2) ***c*** 11.240(2)
Space Group No.: 2 | ***(Å,°)*** α 101.51(3) β 105.41(3) γ 114.93(3)

R-Factor (%): 7.20 ***Temperature(K):*** 293 ***Density(g/cm³):*** 1.564

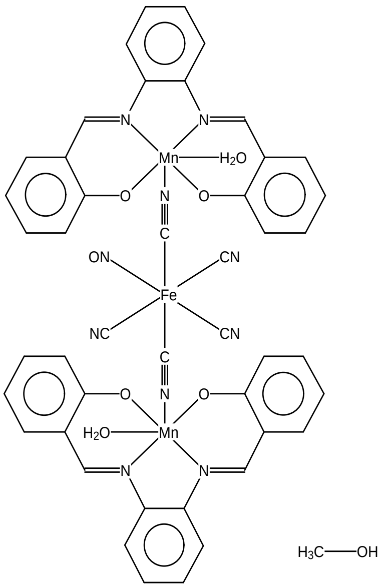

AVAVOJ

Reference: Liqin Ding, Zeng Chu, Leilei Chen, Xingqiang Lu, Biao Yan, Jirong Song, Daidi Fan, Feng Bao (2011)
Inorg.Chem.Commun. ,**14,**573

Formula: $C_{22}\ H_{18}\ N_2\ O_4\ Pd_1,3(H_2\ O_1)$

Compound Name: (2,2'-(1,2-Phenylenebis((nitrilo)methylylidene))bis(6-methoxyphenolato))-palladium trihydrate

Space Group: P-3c1 | ***Cell:*** ***a*** 21.219(0) ***b*** 21.219(0) ***c*** 8.471(0)
Space Group No.: 165 | ***(Å,°)*** α 90.00 β 90.00 γ 120.00

R-Factor (%): 4.78 ***Temperature(K):*** 293 ***Density(g/cm³):*** 1.613

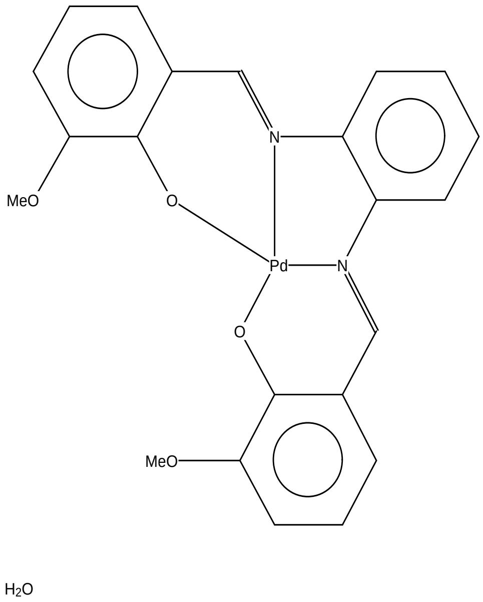

AVAVUP

Reference: Liqin Ding, Zeng Chu, Leilei Chen, Xingqiang Lu, Biao Yan, Jirong Song, Daidi Fan, Feng Bao (2011)
Inorg.Chem.Commun. ,**14,**573

Formula: $C_{22}\ H_{18}\ N_2\ O_4\ Pd_1,4(H_2\ O_1)$

Compound Name: (2,2'-(1,2-Phenylenebis((nitrilo)methylylidene))bis(6-methoxyphenolato))-palladium tetrahydrate

Space Group: P-3c1 | ***Cell:*** ***a*** 21.182(1) ***b*** 21.182(1) ***c*** 8.454(1)
Space Group No.: 165 | ***(Å,°)*** α 90.00 β 90.00 γ 120.00

R-Factor (%): 4.74 ***Temperature(K):*** 293 ***Density(g/cm³):*** 1.677

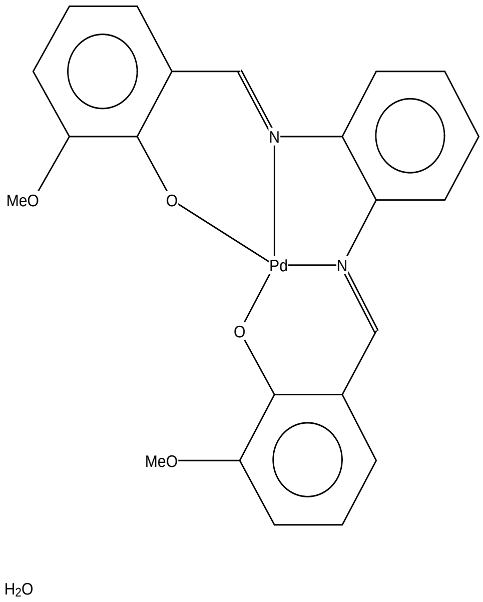

Search: search26 (Tue Dec 2 15:48:50 2014): Hits 37-40

AWEWEE

Reference:	L.Salmon, P.Thuery, M.Ephritikhine (2004) *Polyhedron* ,**23,** 623
Formula:	$2(C_5 H_6 N_1{}^{1+}),C_{80} H_{48} Cl_{10} N_8 O_{20} U_8{}^{2-},10(C_5 H_5 N_1)$
Compound Name:	bis(Pyridinium) tetrakis(μ_4-N,N'-bis(3-oxysalicylidene)-1,2-phenylenediamine)-tetrakis(μ_4-oxo)-bis(μ_2-chloro)-octachloro-octa-uranium(iv) pyridine solvate

Space Group:	C2/c	***Cell:***	***a*** 34.943(1)	***b*** 14.493(0)	***c*** 31.814(1)
Space Group No.:	15	***(Å,°)***	α 90.00	β 120.34(0)	γ 90.00
R-Factor (%):	5.48	***Temperature(K):***	100	***Density(g/cm³):***	2.222

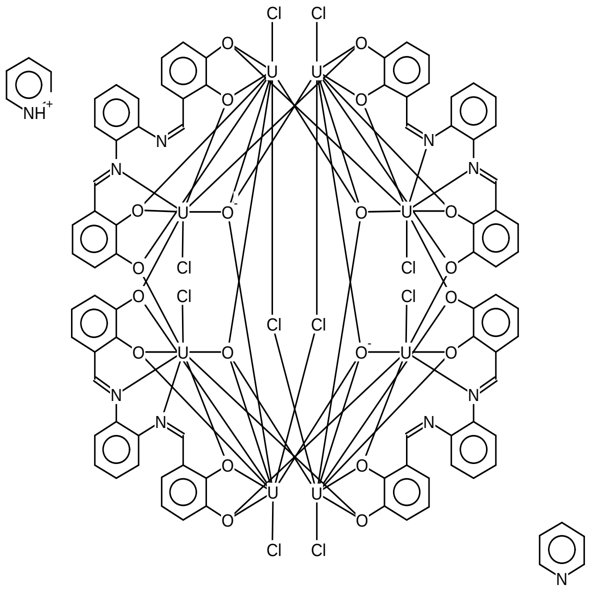

AWOZES

Reference:	Yang Lin, Guang-Ming Li, Peng Chen, Peng-Fei Yan, Guang-Feng Hou (2011) *Acta Crystallogr.,Sect.E:Struct.Rep.Online* , **67,**m1162
Formula:	$C_{23} H_{20} Co_1 N_3 O_5,C_2 H_3 N_1$
Compound Name:	Aqua-(cyano)-(6,6'-dimethoxy-2,2'-[o-phenylenebis(nitrilomethanylylidene)]diphenolato)-cobalt(iii) acetonitrile solvate

Space Group:	P21/c	***Cell:***	***a*** 10.829(2)	***b*** 13.209(3)	***c*** 18.906(6)
Space Group No.:	14	***(Å,°)***	α 90.00	β 118.30(2)	γ 90.00
R-Factor (%):	3.42	***Temperature(K):***	293	***Density(g/cm³):***	1.446

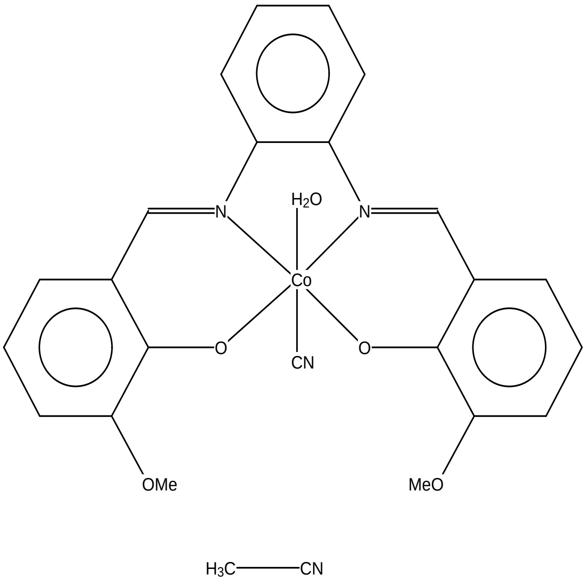

AYOKIJ

Reference:	Fan Yang, Guang-Ming Li, Peng Chen, Peng-Fei Yan, Guang-Feng Hou (2011) *Acta Crystallogr.,Sect.E:Struct.Rep.Online* , **67,**m1185
Formula:	$C_{28} H_{27} Gd_1 N_2 O_{10} Zn_1$
Compound Name:	(μ_2-2,2'-(1,2-phenylenebis((nitrilo)methylylidene))bis(6-(methoxy) phenolato))-(μ_2-acetato)-bis(acetato)-gadolinium(iii)-zinc

Space Group:	P21/c	***Cell:***	***a*** 14.012(3)	***b*** 13.581(3)	***c*** 15.426(3)
Space Group No.:	14	***(Å,°)***	α 90.00	β 103.65(3)	γ 90.00
R-Factor (%):	4.09	***Temperature(K):***	293	***Density(g/cm³):***	1.803

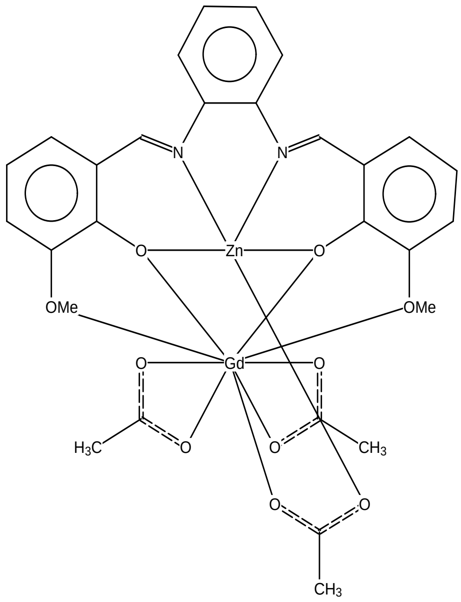

AYOYOC

Reference:	Il Yoon, M.Goto, T.Shimizu, Shim Sung Lee, M.Asakawa (2004) *Dalton Trans.* ,1513
Formula:	$C_{68} H_{68} N_4 Na_2 O_{18} Pd_2{}^{2+},2(Cl_1 O_4{}^{1-}),3(C_1 H_2 Cl_2)$
Compound Name:	bis(μ_2-Aqua)-bis(μ_2-28,35-diaza-2,5,8,15,18,21,41,42-octaoxapentacyclo(35.3.1.1^{22,26}.0^{9,14}.0^{29,34})dotetraconta-1(41),9,11,13,22,24,26(42),27,29,31,33,35,37,39-tetradecaene)-di-palladium(ii)-di-sodium(i) diperchlorate dichloromethane solvate

Space Group:	P21/n	***Cell:***	***a*** 13.695(0)	***b*** 24.817(1)	***c*** 23.027(1)
Space Group No.:	14	***(Å,°)***	α 90.00	β 92.32(0)	γ 90.00
R-Factor (%):	8.65	***Temperature(K):***	183	***Density(g/cm³):***	1.649

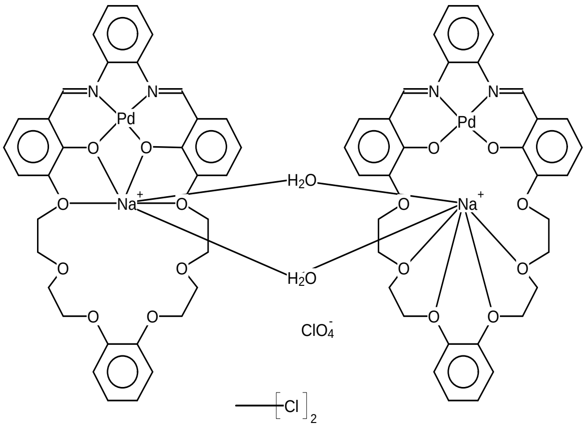

Search: search26 (Tue Dec 2 15:48:50 2014): Hits 41-44

AYOYUI

Reference: Il Yoon, M.Goto, T.Shimizu, Shim Sung Lee, M.Asakawa (2004) *Dalton Trans.* ,1513

Formula: C_{34} H_{32} N_2 Na_1 O_8 Pd_1,C_2 H_3 N_1,C_3 H_6 O_1

Compound Name: (μ_2-28,35-Diaza-2,5,8,15,18,21,41,42-octaoxapentacyclo(35.3.1.1^{22,26}.0^{9,14}.0^{29,34})dotetraconta-1(41),9,11,13,22,24,26(42),27,29,31,33,35,37,39-tetradecaene)-palladium(ii)-sodium(i) acetonitrile acetone solvate

Space Group:	P-1	***Cell:***	***a*** 11.304(0)	***b*** 12.188(1)	***c*** 15.396(1)
Space Group No.:	2	***(Å,°)***	α 73.04(0)	β 80.72(0)	γ 67.28(0)

R-Factor (%): 3.63 ***Temperature(K):*** 183 ***Density(g/cm^3):*** 1.467

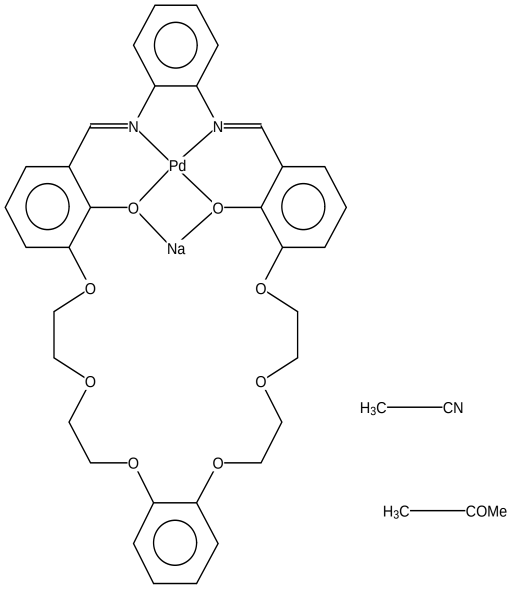

BANFOM

Reference: Kou-Lin Zhang, Yan Xu, You Song, Yang Zhang, Zheng Wang, Xiao-Zeng You (2001) *J.Mol.Struct.* ,**570,**137

Formula: C_{21} H_{18} Mn_1 N_5 O_3

Compound Name: Azido-(methanol)-(N,N'-o-phenylenebis(salicylidenealdiminato))-manganese(iii)

Space Group:	P-1	***Cell:***	***a*** 7.979(1)	***b*** 10.057(2)	***c*** 12.070(1)
Space Group No.:	2	***(Å,°)***	α 97.63(1)	β 91.39(1)	γ 91.93(1)

R-Factor (%): 6.19 ***Temperature(K):*** 293 ***Density(g/cm^3):*** 1.535

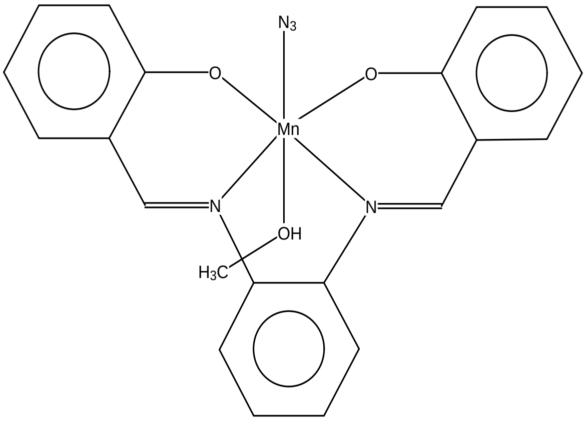

BANFOM01

Reference: Mei Yuan, Fei Zhao, Wen Zhang, Zhe-Ming Wang, Song Gao (2007) *Inorg.Chem.* ,**46,**11235

Formula: C_{21} H_{18} Mn_1 N_5 O_3

Compound Name: azido-(N,N'-bis(salicylidene)-o-phenylenediamine)-methanol-manganese(iii)

Space Group:	P-1	***Cell:***	***a*** 7.971(1)	***b*** 10.064(2)	***c*** 12.057(2)
Space Group No.:	2	***(Å,°)***	α 97.65(3)	β 91.31(3)	γ 91.85(3)

R-Factor (%): 4.17 ***Temperature(K):*** 293 ***Density(g/cm^3):*** 1.537

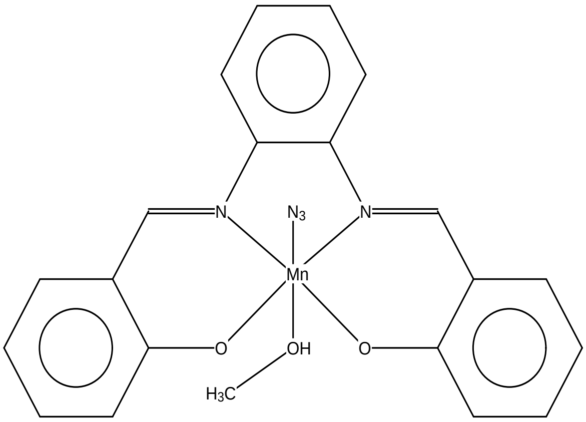

BAQNEO

Reference: P.D.Frischmann, S.H.M.Mehr, B.O.Patrick, F.Lelj, M.J.MacLachlan (2012) *Inorg.Chem.* ,**51,**3443

Formula: C_{66} H_{66} N_6 O_{25} Zn_7,10(C_3 H_7 N_1 O_1)

Compound Name: (μ_6-6,7,20,21,34,35-hexaethoxy-3,10,17,24,31,38-hexaazaheptacyclo[38.2.2.2^{12,15}.2^{26,29}.0^{4,9}.0^{18,23}.0^{32,37}]octatetraconta-1(42),2,4,6,8,10,12,14,16,18,20,22,24,26,28,30,32,34,36,38,40,43,45,47-tetracosaene-13,14,27,28,41,42-hexolato)-(μ_4-oxo)-tris(μ_2-acetato)-tris(acetato)-hepta-zinc(ii) N,N-dimethylformamide solvate

Space Group:	P-1	***Cell:***	***a*** 16.669(3)	***b*** 19.244(4)	***c*** 20.811(6)
Space Group No.:	2	***(Å,°)***	α 97.61(0)	β 106.64(0)	γ 115.01(0)

R-Factor (%): 6.40 ***Temperature(K):*** 90 ***Density(g/cm^3):*** 1.517

$Zn_7[\mu_6\text{-}C_{42}H_{18}N_6O_6(OEt)_6][\mu_4\text{-}O]$--
--$[\mu_2\text{-}CO_2Me\text{-}\kappa^2O,O']_3[OCOMe]_3$

OHC——NMe_2

Search: search26 (Tue Dec 2 15:48:50 2014): Hits 45-48

BATLIT

Reference:	Y.Tamaki, K.Tomono, Y.Hata, N.Saita, T.Yamamoto, K.Miyamura (2012) *Bull.Chem.Soc.Jpn.* ,**85,**592
Formula:	C_{36} H_{46} N_2 Ni_1 O_2
Compound Name:	(2,2'-(1,2-Phenylenebis((nitrilo)methylylidene))bis(4-octylphenolato))-nickel(ii)

Space Group:	P21/c	***Cell:***	***a***	18.867(2)	***b***	9.091(1)	***c***	18.433(2)
Space Group No.:	14	***(Å,°)***	α	90.00	β	94.51(0)	γ	90.00

R-Factor (%): 8.13 ***Temperature(K):*** 300 ***Density(g/cm³):*** 1.259

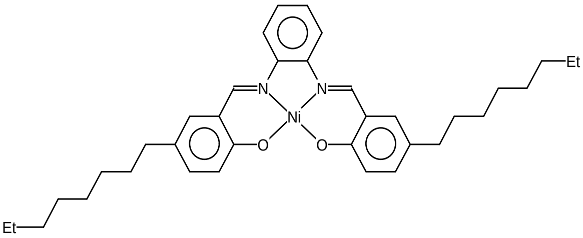

BEDGAU

Reference:	A.Coletti, C.J.Whiteoak, V.Conte, A.W.Kleij (2012) *ChemCatChem* ,**4,**1190
Formula:	C_{28} H_{30} N_2 O_3 V_1,C_1 H_4 O_1
Compound Name:	Oxo-(2,2'-(1,2-phenylenebis((nitrilo)methylylidene))bis(4-t-butylphenolato))-vanadium methanol solvate

Space Group:	P21/c	***Cell:***	***a***	12.021(0)	***b***	10.546(0)	***c***	21.287(1)
Space Group No.:	14	***(Å,°)***	α	90.00	β	101.77(0)	γ	90.00

R-Factor (%): 3.20 ***Temperature(K):*** 100 ***Density(g/cm³):*** 1.321

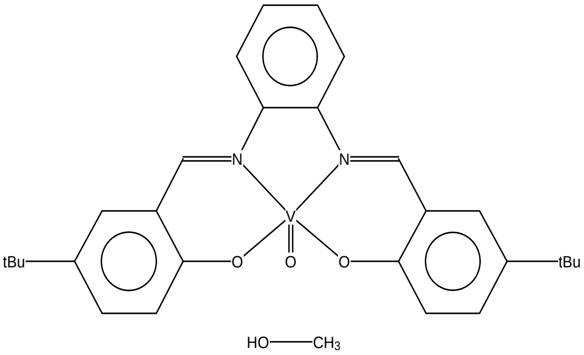

BEHHUT

Reference:	S.Jana, S.Chatterjee, S.Chattopadhyay (2012) *Polyhedron* ,**48,**189
Formula:	C_{21} H_{18} Fe_1 N_5 O_3
Compound Name:	Azido-methanol-(2,2'-(1,2-phenylenebis((nitrilo)methylylidene)) diphenolato)-iron(iii)

Space Group:	P-1	***Cell:***	***a***	7.924(0)	***b***	9.960(0)	***c***	12.256(0)
Space Group No.:	2	***(Å,°)***	α	97.17(0)	β	91.27(0)	γ	92.60(0)

R-Factor (%): 3.31 ***Temperature(K):*** 293 ***Density(g/cm³):*** 1.540

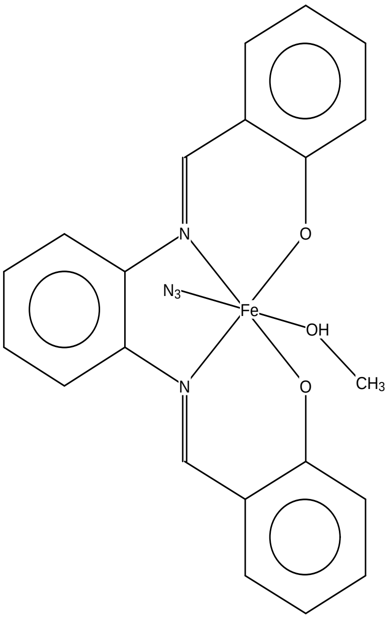

BEHJAB

Reference:	S.Jana, S.Chatterjee, S.Chattopadhyay (2012) *Polyhedron* ,**48,**189
Formula:	C_{22} H_{18} Fe_1 N_3 O_3 S_1
Compound Name:	(Isothiocyanato)-(methanol)-(2,2'-(1,2-phenylenebis((nitrilo)methylylidene))diphenolato)-iron(iii)

Space Group:	C2/c	***Cell:***	***a***	15.123(0)	***b***	9.877(0)	***c***	27.548(0)
Space Group No.:	15	***(Å,°)***	α	90.00	β	92.52(0)	γ	90.00

R-Factor (%): 3.86 ***Temperature(K):*** 293 ***Density(g/cm³):*** 1.488

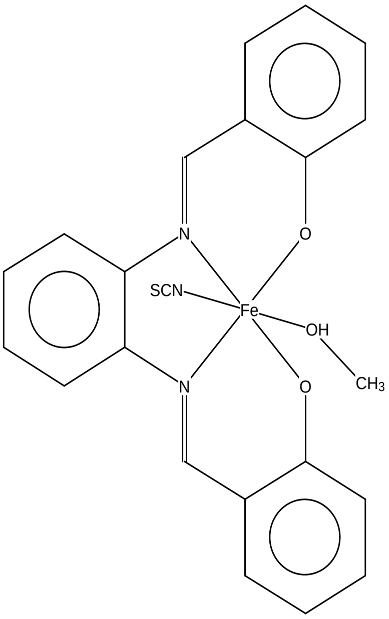

Search: search26 (Tue Dec 2 15:48:50 2014): Hits 49-52

BEHJEF

Reference: S.Jana, S.Chatterjee, S.Chattopadhyay (2012) *Polyhedron* ,**48,**189

Formula: $C_{28}\ H_{26}\ Fe_1\ N_6\ O_4\ ^{1+}, Cl_1\ O_4\ ^{1-}$

Compound Name: bis(1H-imidazole)-(2,2'-(1,2-phenylenebis((nitrilo)methylylidene))bis(6-methoxyphenolato))-iron(iii) perchlorate

Space Group:	P-1	***Cell:***	***a*** 10.126(0)	***b*** 10.550(0)	***c*** 15.546(0)
Space Group No.:	2	***(Å,°)***	α 101.47(0)	β 104.27(0)	γ 105.98(0)
R-Factor (%):	8.41	***Temperature(K):***	293	***Density(g/cm³):***	1.491

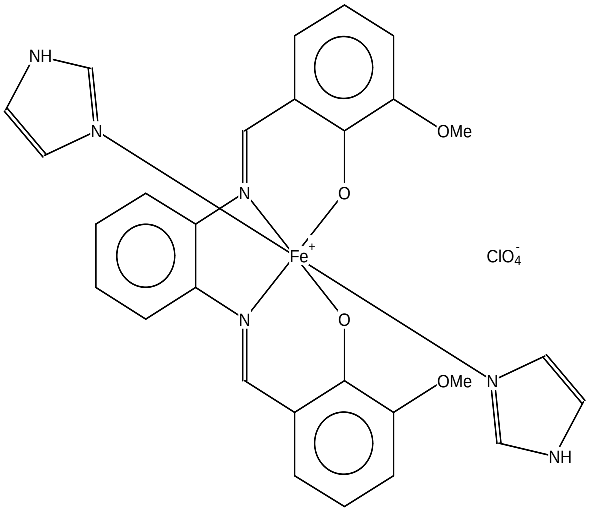

BEHJIJ

Reference: S.Jana, S.Chatterjee, S.Chattopadhyay (2012) *Polyhedron* ,**48,**189

Formula: $C_{44}\ H_{36}\ Fe_2\ N_4\ O_9, 2(H_2\ O_1)$

Compound Name: (μ_2-Oxo)-bis(2,2'-(1,2-phenylenebis(nitrilomethylylidene))bis(6-methoxyphenolato))-di-iron(iii) dihydrate

Space Group:	P-1	***Cell:***	***a*** 12.708(1)	***b*** 13.582(1)	***c*** 14.502(1)
Space Group No.:	2	***(Å,°)***	α 72.66(0)	β 78.00(0)	γ 65.44(0)
R-Factor (%):	7.91	***Temperature(K):***	293	***Density(g/cm³):***	1.401

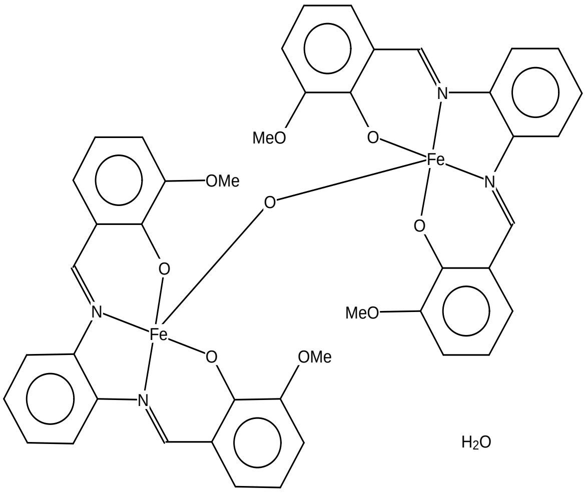

BEHJOP

Reference: S.Jana, S.Chatterjee, S.Chattopadhyay (2012) *Polyhedron* ,**48,**189

Formula: $C_{56}\ H_{36}\ Fe_2\ N_4\ O_5, 2(C_1\ H_2\ Cl_2)$

Compound Name: (μ_2-Oxo)-bis(1,1'-(1,2-phenylenebis(nitrilomethylylidene))bis(2-naphtholato))-di-iron(iii) dichloromethane solvate

Space Group:	P-1	***Cell:***	***a*** 12.328(5)	***b*** 13.821(5)	***c*** 14.695(5)
Space Group No.:	2	***(Å,°)***	α 92.42(0)	β 97.56(0)	γ 91.34(0)
R-Factor (%):	6.10	***Temperature(K):***	293	***Density(g/cm³):***	1.509

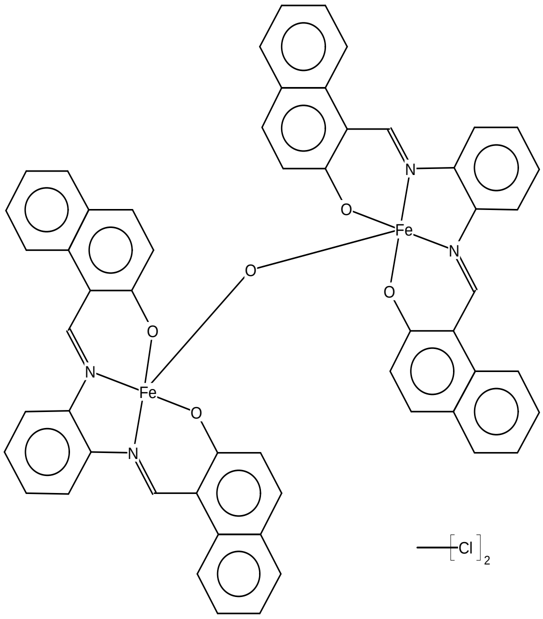

BEKZIA

Reference: R.H.Heistand, A.L.Roe, L.Que Junior (1982) *Inorg.Chem.* , **21,**676

Formula: $C_{26}\ H_{19}\ Fe_1\ N_2\ O_4$

Compound Name: (N,N'-(1,2-Phenylene)-bis(salicylideniminato))-(catecholato-O)-iron(iii)

Space Group:	P-1	***Cell:***	***a*** 9.960(6)	***b*** 8.108(3)	***c*** 13.849(8)
Space Group No.:	2	***(Å,°)***	α 95.73(4)	β 91.93(5)	γ 72.13(4)
R-Factor (%):	7.60	***Temperature(K):***	295	***Density(g/cm³):***	1.503

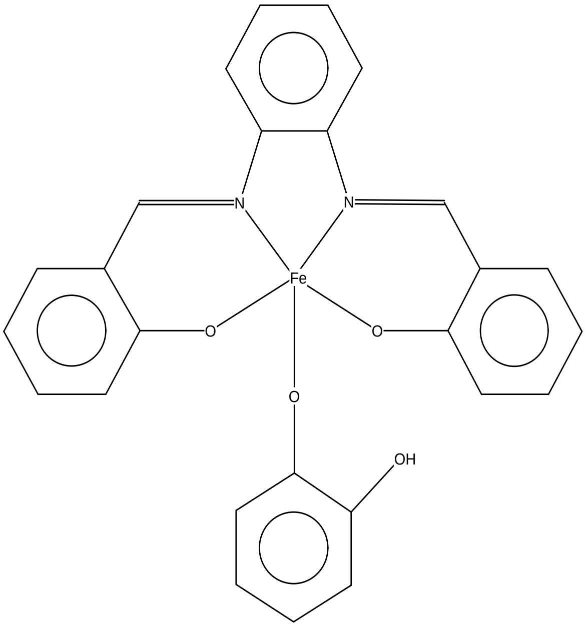

BENGEG

Reference: S.Gambarotta, M.L.Fiallo, C.Floriani, A.C.Villa, C.Guastini (1982) *Chem.Commun.* ,503

Formula: $C_{48} H_{44} Co_2 N_4 Na_1 O_6 S_2$ $^{1+}$,$C_{24} H_{20} B_1$ $^{1-}$

Compound Name: (μ_2-Persulfido)-(tetrahydrofuran-bis(N,N'-o-phenylene-bis(salicylideneaminato)-cobalt(iii)))-tetrahydrofuran-sodium tetraphenylborate

Space Group: P-1 ***Cell:*** ***a*** 11.816(1) ***b*** 15.549(2) ***c*** 17.437(2)
Space Group No.: 2 ***(Å,°)*** α 93.70(1) β 93.90(1) γ 94.86(1)

R-Factor (%): 5.90 ***Temperature(K):*** 295 ***Density(g/cm³):*** 1.356

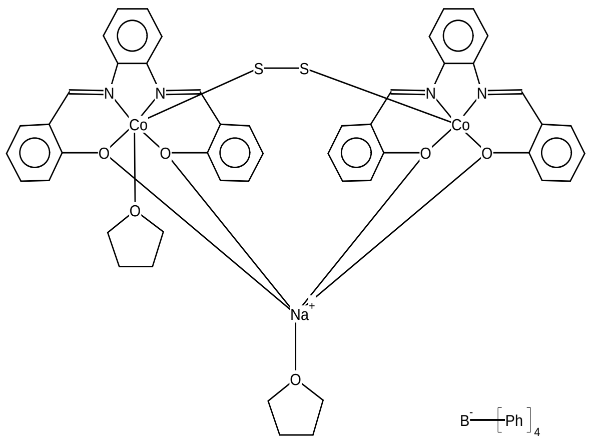

BENGEG10

Reference: C.Floriani, M.Fiallo, A.Chiesi-Villa, C.Guastini (1987) *J.Chem.Soc.,Dalton Trans.* ,1367

Formula: $C_{48} H_{44} Co_2 N_4 Na_1 O_6 S_2$ $^{1+}$,$C_{24} H_{20} B_1$ $^{1-}$

Compound Name: ((μ_2-Disulfido-S,S')-tetrahydrofuran-bis(N,N'-o-phenylene-bis(salicylideneiminato-N,N',O,O')-cobalt(iii))-O,O',O'',O'''-tetrahydrofuran-sodium) tetraphenylborate

Space Group: P-1 ***Cell:*** ***a*** 11.816(1) ***b*** 15.549(2) ***c*** 17.437(2)
Space Group No.: 2 ***(Å,°)*** α 93.70(1) β 93.90(1) γ 94.86(1)

R-Factor (%): 5.80 ***Temperature(K):*** 295 ***Density(g/cm³):*** 1.356

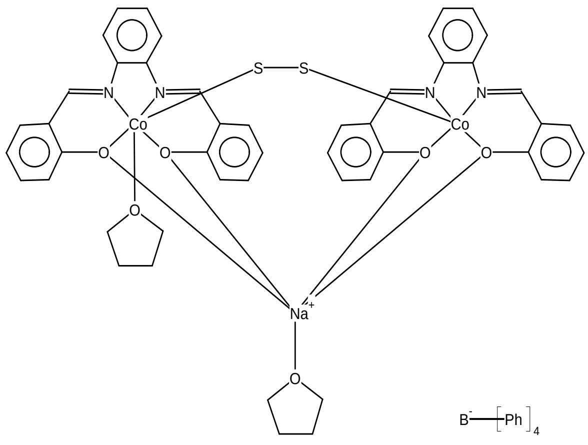

BEVWAB

Reference: R.Kitaura, G.Onoyama, H.Sakamoto, R.Matsuda, S.Noro, S.Kitagawa (2004) *Angew.Chem.,Int.Ed.* ,**43,**2684

Formula: $(C_{44} H_{26} Cu_2 N_4 O_{14} Zn_3)n,2n(C_3 H_7 N_1 O_1)$

Compound Name: catena-(bis((μ_5-N,N'-o-Phenylene-bis(salicylideneimino-5-carboxylato))-(μ_2-hydroxo))-di-copper(ii)-tri-zinc(ii) bis(dimethylformamide) clathrate)

Space Group: C2/m ***Cell:*** ***a*** 27.960(20) ***b*** 26.620(20) ***c*** 6.101(4)
Space Group No.: 12 ***(Å,°)*** α 90.00 β 94.96(1) γ 90.00

R-Factor (%): 9.96 ***Temperature(K):*** 293 ***Density(g/cm³):*** 0.957

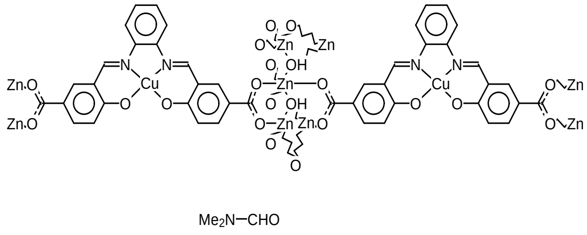

BEVWEF

Reference: R.Kitaura, G.Onoyama, H.Sakamoto, R.Matsuda, S.Noro, S.Kitagawa (2004) *Angew.Chem.,Int.Ed.* ,**43,**2684

Formula: $C_{22} H_{14} Cu_1 N_2 O_6$,$H_2 O_1$

Compound Name: (N,N'-o-Phenylene-bis(5-carboxysalicylideneamino))-copper(ii) monohydrate

Space Group: P21/c ***Cell:*** ***a*** 16.050(20) ***b*** 7.286(7) ***c*** 18.860(20)
Space Group No.: 14 ***(Å,°)*** α 90.00 β 93.39(1) γ 90.00

R-Factor (%): 7.99 ***Temperature(K):*** 223 ***Density(g/cm³):*** 1.460

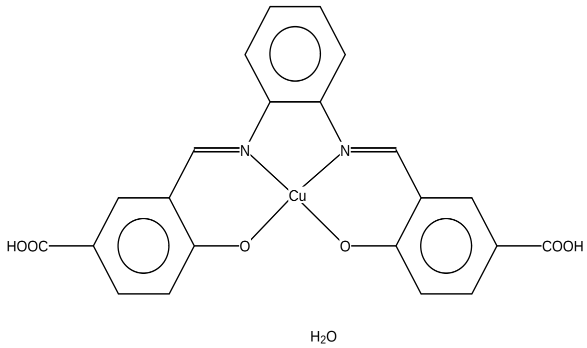

BILQAP

Reference: S.Naskar, S.Biswas, D.Mishra, B.Adhikary, L.R.Falvello, T.Soler, C.H.Schwalbe, S.K.Chattopadhyay (2004) *Inorg.Chim.Acta* , **357,**4257

Formula: $C_{26}\ H_{22}\ Mn_1\ N_6\ O_2\ ^{1+}, Cl_1\ O_4\ ^{1-}$

Compound Name: (N,N'-o-phenylenebis(salicylideneiminato))-bis(3-imidazole)-manganese(iii) perchlorate

Space Group:	R-3	***Cell:***	***a***	38.390(5)	***b***	38.390(5)	***c***	10.118(2)
Space Group No.:	148	***(Å,°)***	α	90.00	β	90.00	γ	120.00

R-Factor (%): 4.76 ***Temperature(K):*** 293 ***Density(g/cm³):*** 1.400

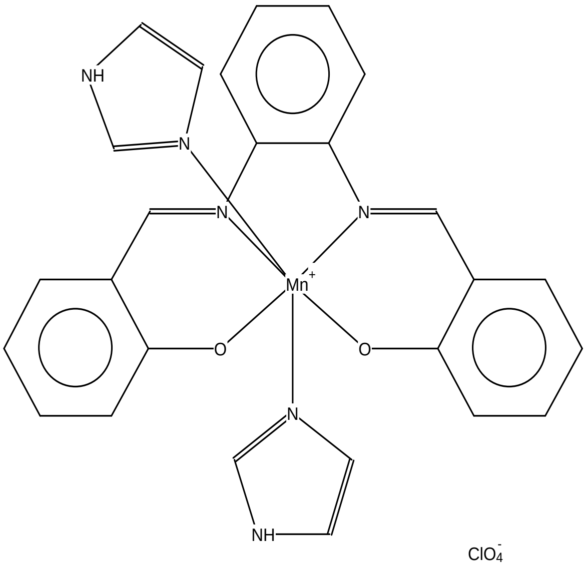

BIZBAO

Reference: Jiang Bian (2008) *Acta Crystallogr.,Sect.E:Struct.Rep.Online* ,**64,**m625

Formula: $C_{23}\ H_{22}\ Cl_1\ Cu_1\ N_2\ Na_1\ O_5$

Compound Name: (μ_2-N,N'-(1,2-Phenylene)-bis(3-methoxysalicylideneamine))-(methanol)-chloro-copper(ii)-sodium(i)

Synonym: (μ_2-6,6'-Dimethoxy-2,2'-(1,2-phenylenebis(nitrilomethylidyne)) diphenolato)-(methanol)-chloro-copper(ii)-sodium(i)

Space Group:	P21/n	***Cell:***	***a***	11.799(0)	***b***	7.966(0)	***c***	23.829(0)
Space Group No.:	14	***(Å,°)***	α	90.00	β	93.28(0)	γ	90.00

R-Factor (%): 2.78 ***Temperature(K):*** 295 ***Density(g/cm³):*** 1.570

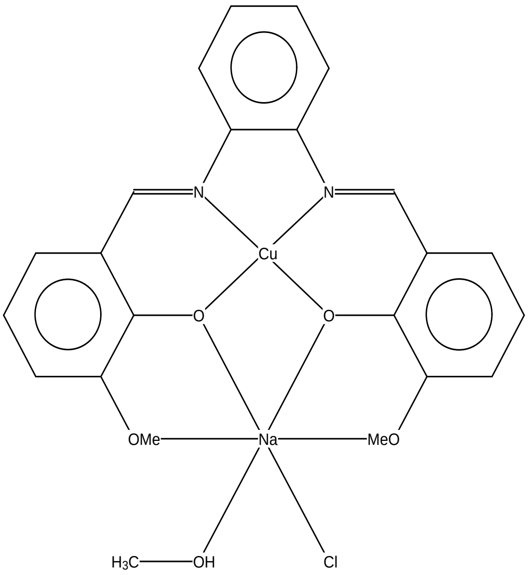

BIZBES

Reference: N.E.Eltayeb, S.G.Teoh, S.Chantrapromma, H.-K.Fun, R.Adnan (2008) *Acta Crystallogr.,Sect.E:Struct.Rep.Online* ,**64,**m626

Formula: $C_{29}\ H_{34}\ Cl_1\ Mn_1\ N_2\ O_3, C_{28}\ H_{32}\ Cl_1\ Mn_1\ N_2\ O_3$

Compound Name: Aqua-chloro-(N,N'-(1,2-phenylene)-bis(3-t-butylsalicylideneamine))-manganese(iii) chloro-(N,N'-(1,2-phenylene)-bis(3-t-butylsalicylideneamine))-(methanol)-manganese(iii)

Synonym: Aqua-chloro-(6,6'-di-t-butyl-2,2'-(1,2-phenylenebis(nitrilomethylidyne)) diphenolato)-manganese(iii) chloro-(6,6'-di-t-butyl-2,2'-(1,2-phenylenebis(nitrilomethylidyne))diphenolato)-(methanol)-manganese(iii)

Space Group:	P-1	***Cell:***	***a***	13.108(0)	***b***	13.879(0)	***c***	14.609(0)
Space Group No.:	2	***(Å,°)***	α	95.18(0)	β	100.00(0)	γ	95.64(0)

R-Factor (%): 5.65 ***Temperature(K):*** 100 ***Density(g/cm³):*** 1.390

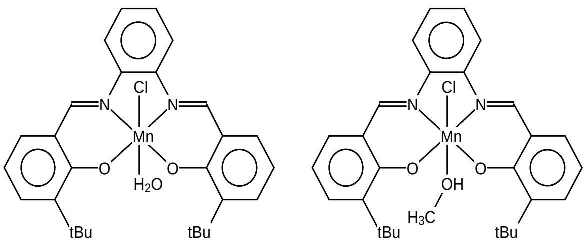

BOLJIW

Reference: D.J.Darensbourg, A.I.Moncada (2008) *Inorg.Chem.* ,**47,** 10000

Formula: $C_{36}\ H_{30}\ N_1\ P_2\ ^{1+}, C_{32}\ H_{34}\ Cr_1\ N_4\ O_4\ ^{1-}, 3(C_1\ H_2\ Cl_2)$

Compound Name: bis(Triphenylphosphoranylidene)ammonium trans-dicyano-(N,N'-bis(3-t-butyl-5-methoxysalicylidene)-1,2-phenylenediamine)-chromium(iii) dichloromethane solvate

Space Group:	P-1	***Cell:***	***a***	12.104(3)	***b***	16.019(4)	***c***	19.970(7)
Space Group No.:	2	***(Å,°)***	α	66.38(1)	β	80.31(2)	γ	79.03(1)

R-Factor (%): 8.43 ***Temperature(K):*** 110 ***Density(g/cm³):*** 1.327

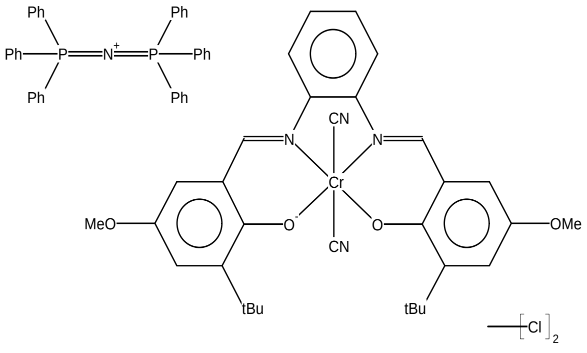

BOLJOC

Reference: D.J.Darensbourg, A.I.Moncada (2008) *Inorg.Chem.* ,**47**, 10000

Formula: $C_{36} H_{30} N_1 P_2{}^{1+}$,$C_{30} H_{34} Cl_1 Cr_1 N_5 O_4{}^{1-}$,$C_1 H_2 Cl_2$

Compound Name: bis(Triphenylphosphoranylidene)ammonium trans-(azido)-chloro-(N,N'-bis(3-t-butyl-5-methoxysalicylidene)-1,2-phenylenediamine)-chromium(iii) dichloromethane solvate

Space Group:	P-1	***Cell:***	***a***	12.447(2)	***b***	15.076(2)	***c***	18.509(3)
Space Group No.:	2	***(Å,°)***	α	70.03(0)	β	82.56(0)	γ	79.98(0)

R-Factor (%): 8.94 ***Temperature(K):*** 110 ***Density(g/cm³):*** 1.284

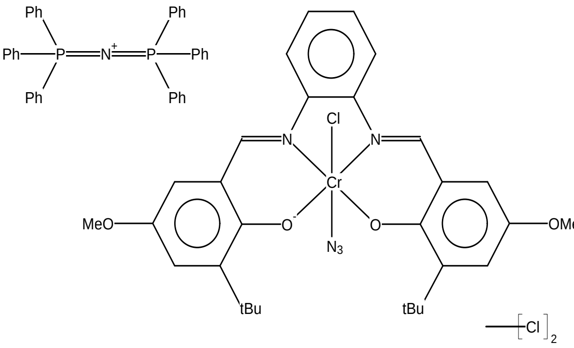

BOLJUI

Reference: D.J.Darensbourg, A.I.Moncada (2008) *Inorg.Chem.* ,**47**, 10000

Formula: $C_{16} H_{36} N_1{}^{1+}$,$C_{30} H_{34} Cr_1 N_8 O_4{}^{1-}$

Compound Name: Tetra-n-butylammonium trans-bis(azido)-(N,N'-bis(3-t-butyl-5-methoxysalicylidene)-1,2-phenylenediamine)-chromium(iii)

Space Group:	Pnna	***Cell:***	***a***	10.623(1)	***b***	25.105(2)	***c***	17.257(1)
Space Group No.:	52	***(Å,°)***	α	90.00	β	90.00	γ	90.00

R-Factor (%): 8.22 ***Temperature(K):*** 110 ***Density(g/cm³):*** 1.249

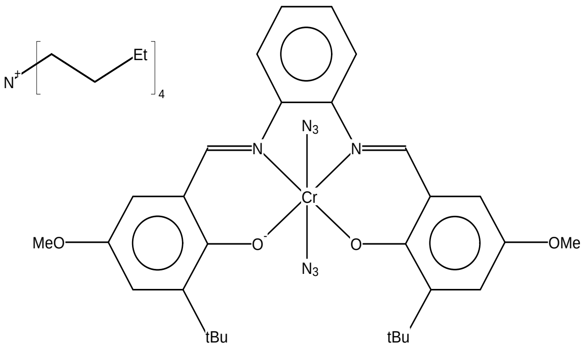

BOLKAP

Reference: D.J.Darensbourg, A.I.Moncada (2008) *Inorg.Chem.* ,**47**, 10000

Formula: $C_{36} H_{30} N_1 P_2{}^{1+}$,$C_{32} H_{34} Cr_1 N_4 O_6{}^{1-}$,$2(C_1 H_2 Cl_2)$

Compound Name: bis(Triphenylphosphoranylidene)ammonium trans-bis(isocyanato)-(N,N'-bis(3-t-butyl-5-methoxysalicylidene)-1,2-phenylenediamine)-chromium(iii) dichloromethane solvate

Space Group:	P-1	***Cell:***	***a***	11.961(2)	***b***	15.876(3)	***c***	18.842(4)
Space Group No.:	2	***(Å,°)***	α	68.18(1)	β	79.35(1)	γ	88.64(1)

R-Factor (%): 5.04 ***Temperature(K):*** 110 ***Density(g/cm³):*** 1.356

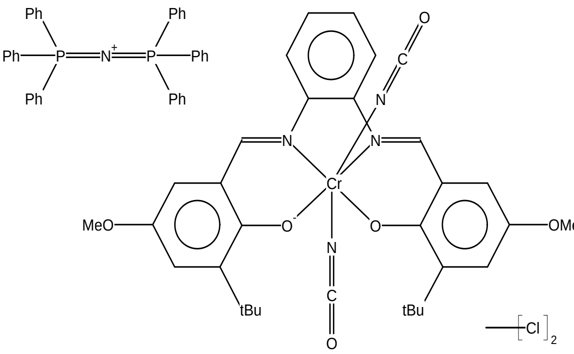

BOLKET

Reference: D.J.Darensbourg, A.I.Moncada (2008) *Inorg.Chem.* ,**47**, 10000

Formula: $C_{16} H_{36} N_1{}^{1+}$,$C_{30} H_{34} Cl_2 Cr_1 N_2 O_4{}^{1-}$,$C_1 H_2 Cl_2$

Compound Name: Tetra-n-butylammonium trans-dichloro-(N,N'-bis(3-t-butyl-5-methoxysalicylidene)-1,2-phenylenediamine)-chromium(iii) dichloromethane solvate

Space Group:	P-1	***Cell:***	***a***	13.039(3)	***b***	13.372(3)	***c***	14.703(3)
Space Group No.:	2	***(Å,°)***	α	93.05(0)	β	101.59(0)	γ	104.70(0)

R-Factor (%): 4.52 ***Temperature(K):*** 110 ***Density(g/cm³):*** 1.289

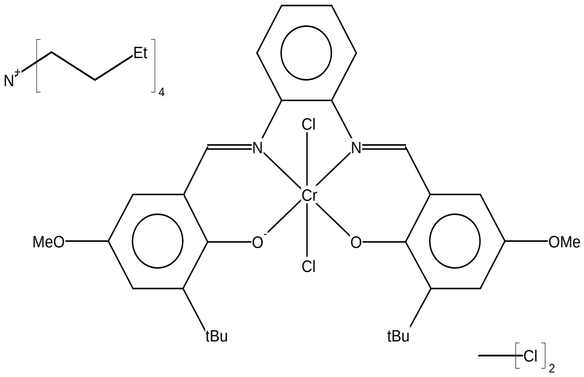

Search: search26 (Tue Dec 2 15:48:50 2014): Hits 65-68

BOQNUR

Reference: O.Rotthaus, O.Jarjayes, C.Philouze, C.P.Del Valle, F.Thomas (2009) *Dalton Trans.* ,1792

Formula: $C_{38}\ H_{50}\ N_2\ Ni_1\ O_4$

Compound Name: (N,N'-(4,5-dimethoxy-1,2-phenylene)-bis(3,5-di-t-butylsalicylaldiminato))-nickel(ii)

Space Group: C2/c ***Cell:*** ***a*** 29.792(4) ***b*** 19.255(2) ***c*** 13.806(0)
Space Group No.: 15 ***(Å,°)*** α 90.00 β 117.44(1) γ 90.00

R-Factor (%): 6.13 ***Temperature(K):*** 150 ***Density(g/cm³):*** 1.243

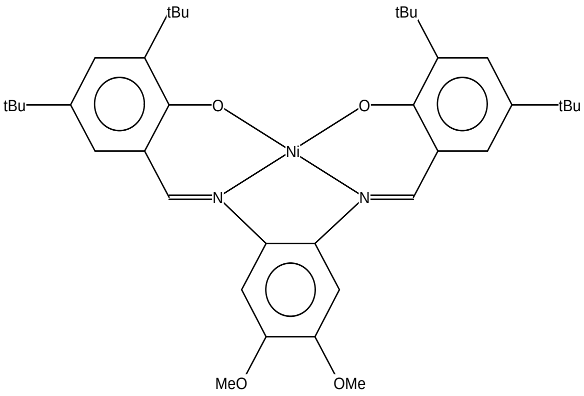

BOQPAZ

Reference: O.Rotthaus, O.Jarjayes, C.Philouze, C.P.Del Valle, F.Thomas (2009) *Dalton Trans.* ,1792

Formula: $C_{36}\ H_{44}\ N_4\ Ni_1\ O_6,C_1\ H_4\ O_1$

Compound Name: (N,N'-(4,5-dinitro-1,2-phenylene)-bis(3,5-di-t-butylsalicylaldiminato))-nickel(ii) methanol solvate

Space Group: P-1 ***Cell:*** ***a*** 12.029(6) ***b*** 12.317(4) ***c*** 13.160(10)
Space Group No.: 2 ***(Å,°)*** α 99.11(4) β 102.79(4) γ 100.68(3)

R-Factor (%): 6.68 ***Temperature(K):*** 293 ***Density(g/cm³):*** 1.307

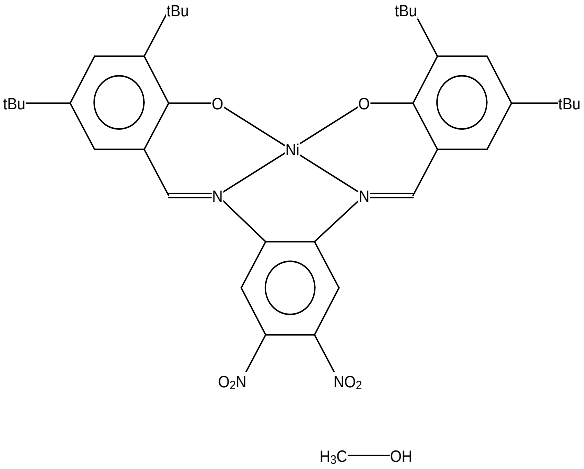

BOVGID

Reference: X.Zhang, P.Wei, B.Li, C.Wu, B.Hu (2009) *Acta Crystallogr.,Sect.E:Struct.Rep.Online* ,**65,**m707

Formula: $C_{20}\ H_{16}\ Mn_1\ N_5\ O_3,0.5(H_2\ O_1)$

Compound Name: Aqua-azido-(2,2'-(o-phenylenebis(nitrilomethylidene))diphenolato)-manganese(ii) hemihydrate

Space Group: C2/c ***Cell:*** ***a*** 25.100(10) ***b*** 11.478(5) ***c*** 12.599(5)
Space Group No.: 15 ***(Å,°)*** α 90.00 β 94.17(0) γ 90.00

R-Factor (%): 3.71 ***Temperature(K):*** 293 ***Density(g/cm³):*** 1.608

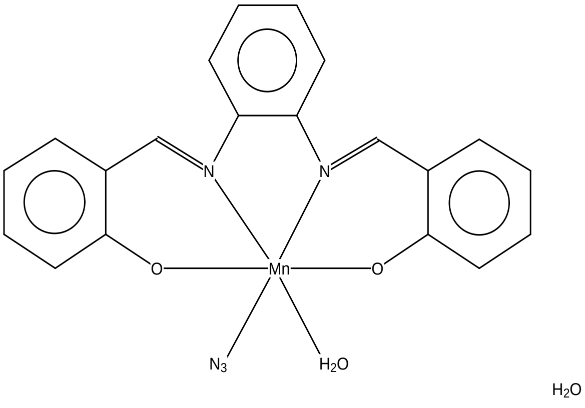

BZMPSC10

Reference: D.Cummins, E.D.McKenzie, H.Milburn (1976) *J.Chem.Soc.,Dalton Trans.* ,130

Formula: $C_{35}\ H_{25}\ Co_1\ N_2\ O_4$

Compound Name: Dibenzoylmethanato-(N,N'-o-phenylene-bis(salicylaldiminato)) cobalt(iii)

Space Group: P21/n ***Cell:*** ***a*** 16.890(20) ***b*** 16.470(20) ***c*** 9.840(10)
Space Group No.: 14 ***(Å,°)*** α 90.00 β 90.86(5) γ 90.00

R-Factor (%): 5.90 ***Temperature(K):*** 295 ***Density(g/cm³):*** 1.448

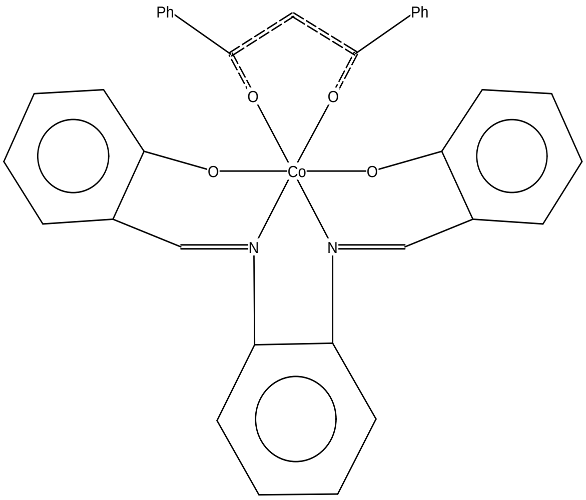

Search: search26 (Tue Dec 2 15:48:50 2014): Hits 69-72

CAKPEL

Reference: Xiao-Song Zhang, Guo-Hua Ding, Qiao-Zhen Qi (2010) *Yingyong Huaxue(Chin.)(Chin.J.Appl.Chem.)* ,**27,**1334

Formula: $C_{20}\,H_{10}\,Cl_4\,Cu_1\,N_2\,O_2{,}C_3\,H_7\,N_1\,O_1$

Compound Name: (2,2'-(1,2-Phenylenebis((nitrilo)methylylidene))bis(4,6-dichlorophenolato))-copper(ii) N,N-dimethylformamide solvate

Space Group: P21/n ***Cell:*** ***a*** 8.132(0) ***b*** 15.310(1) ***c*** 18.782(1)
Space Group No.: 14 ***(Å,°)*** α 90.00 β 92.45(0) γ 90.00

R-Factor (%): 5.84 ***Temperature(K):*** 298 ***Density(g/cm^3):*** 1.674

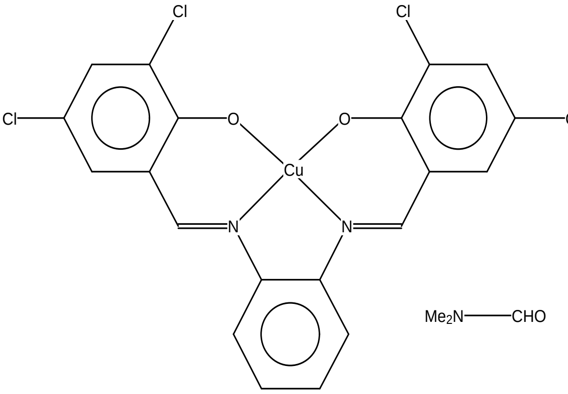

CAKYET

Reference: G.A.Morris, Hongying Zhou, C.L.Stern, SonBinh T.Nguyen (2001) *Inorg.Chem.* ,**40,**3222

Formula: $C_{43}\,H_{46}\,N_5\,O_2\,Zn_1{,}C_5\,H_{11}\,N_1$

Compound Name: (Benzene-1,2-diamine-N,N'-bis(3-t-butyl-5-(4-pyridyl)salicylidene))-(piperidine)-zinc(ii) piperidine solvate

Space Group: P-1 ***Cell:*** ***a*** 10.588(2) ***b*** 13.021(3) ***c*** 16.951(3)
Space Group No.: 2 ***(Å,°)*** α 85.16(0) β 74.48(0) γ 79.69(0)

R-Factor (%): 6.40 ***Temperature(K):*** 153 ***Density(g/cm^3):*** 1.223

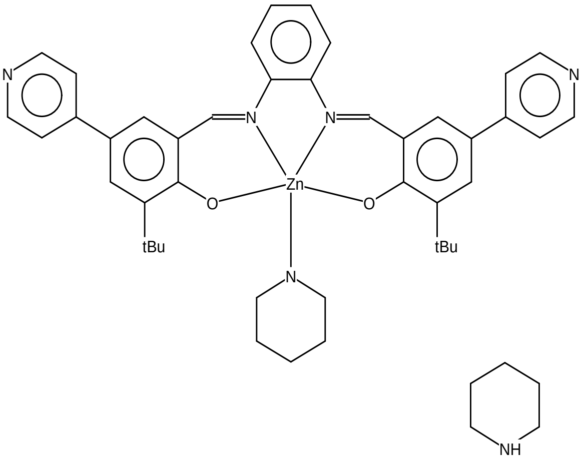

CAKZEU

Reference: M.-A.Munoz-Hernandez, T.S.Keizer, Pingrong Wei, S.Parkin, D.A.Atwood (2001) *Inorg.Chem.* ,**40,**6782

Formula: $C_{38}\,H_{50}\,Al_1\,Cl_1\,N_2\,O_2{,}C_7\,H_8$

Compound Name: Chloro-((4,5-dimethylbenzene-1,2-diamine)-N,N'-bis(3,5-di-t-butylsalicylidene))-aluminium toluene solvate

Space Group: P21/n ***Cell:*** ***a*** 19.256(3) ***b*** 10.208(2) ***c*** 21.640(3)
Space Group No.: 14 ***(Å,°)*** α 90.00 β 94.78(1) γ 90.00

R-Factor (%): 6.82 ***Temperature(K):*** 173 ***Density(g/cm^3):*** 1.130

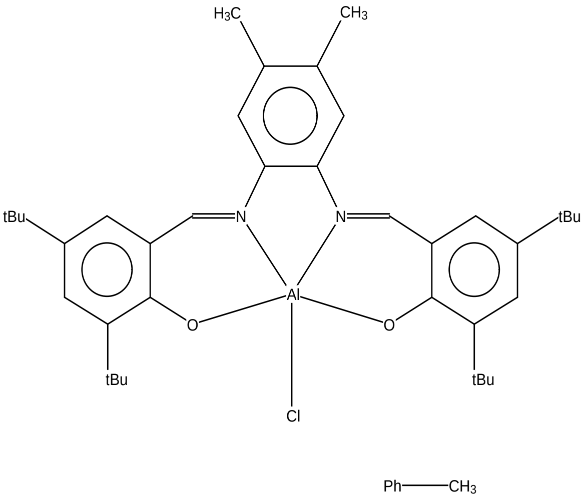

CAKZOE

Reference: M.-A.Munoz-Hernandez, T.S.Keizer, Pingrong Wei, S.Parkin, D.A.Atwood (2001) *Inorg.Chem.* ,**40,**6782

Formula: $C_{41}\,H_{59}\,Al_1\,N_2\,O_3\,Si_1$

Compound Name: ((4,5-Dimethylbenzene-1,2-diamine)-N,N'-bis(3,5-di-t-butylsalicylidene))-(trimethylsiloxy)-aluminium

Space Group: P21/c ***Cell:*** ***a*** 14.395(2) ***b*** 15.209(2) ***c*** 18.297(2)
Space Group No.: 14 ***(Å,°)*** α 90.00 β 93.97(1) γ 90.00

R-Factor (%): 5.44 ***Temperature(K):*** 173 ***Density(g/cm^3):*** 1.135

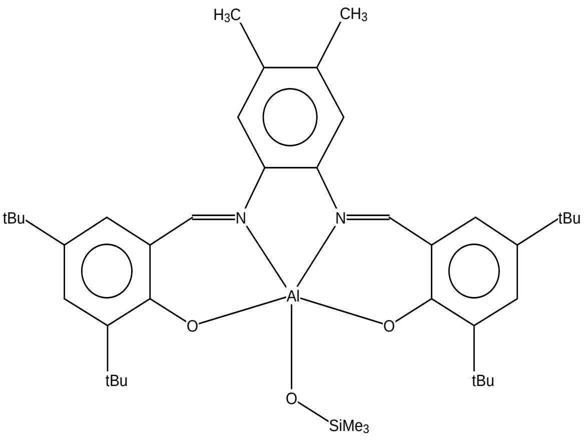

Search: search26 (Tue Dec 2 15:48:50 2014): Hits 73-76

CAKZUK

Reference: M.-A.Munoz-Hernandez, T.S.Keizer, Pingrong Wei, S.Parkin, D.A.Atwood (2001) *Inorg.Chem.* ,**40,**6782

Formula: $C_{56}\ H_{65}\ Al_1\ N_2\ O_3\ Si_1$

Compound Name: ((4,5-Dimethylbenzene-1,2-diamine)-N,N'-bis(3,5-di-t-butylsalicylidene))-(triphenylsiloxy)-aluminium

Space Group:	P21/c	***Cell:***	***a***	19.233(1)	***b***	14.227(0)	***c***	19.378(1)
Space Group No.:	14	***(Å,°)***	α	90.00	β	109.53(0)	γ	90.00

R-Factor (%): 4.31 ***Temperature(K):*** 173 ***Density(g/cm^3):*** 1.155

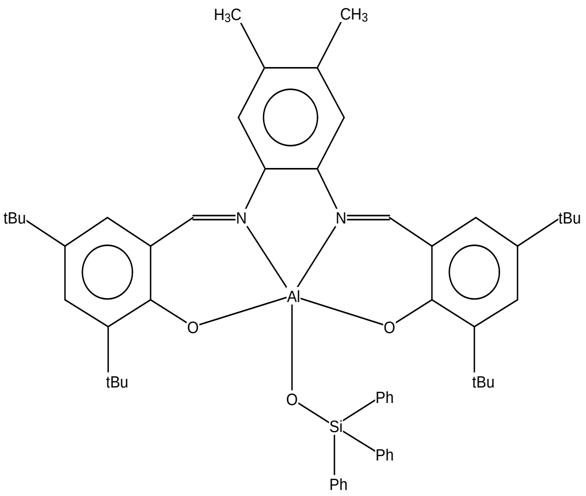

CAQHAF

Reference: M.M.Belmonte, E.C.Escudero-Adan, E.Martin, A.W.Kleij (2012) *Dalton Trans.* ,**41,**5193

Formula: $C_{84}\ H_{72}\ N_6\ O_{22}\ Zn_8{,}8(C_1\ H_4\ O_1)$

Compound Name: tris(μ_4-3-[((2-[(2,3-dioxidobenzylidene)amino]-4,5-dimethylphenyl)imino) methyl]benzene-1,2-diolato)-(μ_3-3-formylbenzene-1,2-diolato)-(μ_2-3-formylbenzene-1,2-diolato)-tetramethanol-octa-zinc(ii) methanol solvate

Space Group:	P21	***Cell:***	***a***	23.496(1)	***b***	13.726(1)	***c***	31.517(1)
Space Group No.:	4	***(Å,°)***	α	90.00	β	98.31(0)	γ	90.00

R-Factor (%): 5.61 ***Temperature(K):*** 100 ***Density(g/cm^3):*** 1.517

Zn_8--
--$[\mu_4\text{-}C_{20}H_{10}N_2O_4Me_2\text{-}\kappa^2N,N',\kappa^7O,O',O'',O''']_3[\mu_3\text{-}C_7H_4O_3\text{-}\kappa^5O,O',O'']$--
--$[\mu_2\text{-}C_7H_4O_3\text{-}\kappa^4O,O',O''][HOMe]_4$

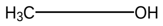

CAQHEJ

Reference: M.M.Belmonte, E.C.Escudero-Adan, E.Martin, A.W.Kleij (2012) *Dalton Trans.* ,**41,**5193

Formula: $C_{76}\ H_{74}\ N_8\ O_{14}\ Zn_6{,}6(C_7\ H_9\ N_1)$

Compound Name: bis(μ_4-3-[((2-[(2,3-dioxidobenzylidene)amino]-4,5-dimethylphenyl)imino) methyl]benzene-1,2-diolato)-bis(μ_3-oxo)-tetrakis(3,5-dimethylpyridine)-diacetato-hexa-zinc(ii) 3,5-dimethylpyridine solvate

Space Group:	P-1	***Cell:***	***a***	14.148(0)	***b***	14.798(0)	***c***	15.525(0)
Space Group No.:	2	***(Å,°)***	α	81.36(0)	β	80.48(0)	γ	65.42(0)

R-Factor (%): 3.68 ***Temperature(K):*** 100 ***Density(g/cm^3):*** 1.349

Zn_6--
--$[\mu_4\text{-}C_{20}H_{10}N_2O_4Me_2\text{-}\kappa^2N,N',\kappa^7O,O',O'',O''']_2[\mu_3\text{-}O]_2[C_5H_3NMe_2]_4[OCOMe]_2$

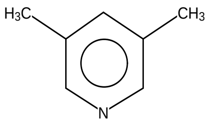

CAQHIN

Reference: M.M.Belmonte, E.C.Escudero-Adan, E.Martin, A.W.Kleij (2012) *Dalton Trans.* ,**41,**5193

Formula: $C_{134}\ H_{98}\ N_{14}\ O_{24}\ Zn_{10}{,}3(C_5\ H_5\ N_1)$

Compound Name: bis(μ_5-3-[((3-[(2,3-dioxidobenzylidene)amino]naphthalen-2-yl)imino) methyl]benzene-1,2-diolato)-bis(μ_4-3-[((3-[(2,3-dioxidobenzylidene) amino]naphthalen-2-yl)imino)methyl]benzene-1,2-diolato)-tetrakis(μ_2-acetato)-hexapyridine-deca-zinc(ii) pyridine solvate

Space Group:	P-1	***Cell:***	***a***	12.938(1)	***b***	13.978(1)	***c***	18.344(1)
Space Group No.:	2	***(Å,°)***	α	83.27(0)	β	87.46(0)	γ	88.39(0)

R-Factor (%): 4.64 ***Temperature(K):*** 100 ***Density(g/cm^3):*** 1.604

$Zn_{10}[\mu_5\text{-}C_{24}H_{14}N_2O_4]_2$--
--$[\mu_4\text{-}C_{24}H_{14}N_2O_4\text{-}\kappa^2N,N',\kappa^7O,O',O'',O''']_2[\mu_2\text{-}CO_2Me\text{-}\kappa^2O,O']_4[Py]_6$

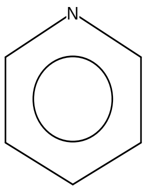

CAQHOT

Reference: M.M.Belmonte, E.C.Escudero-Adan, E.Martin, A.W.Kleij (2012) *Dalton Trans.* ,**41,**5193

Formula: $C_{96}H_{64}N_8O_{20}Zn_8$,7.8($C_3H_6O_1$),2($C_2H_6O_1S_1$),2.78($H_2O_1$)

Compound Name: tetrakis(μ_4-3-[((3-[(2,3-dioxidobenzylidene)amino]naphthalen-2-yl)imino)methyl]benzene-1,2-diolato)-tetra-aqua-octa-zinc(ii) acetone dimethyl sulfoxide solvate hydrate

Space Group:	P-1	***Cell:***	***a*** 17.741(1)	***b*** 18.440(1)	***c*** 24.546(1)		
Space Group No.:	2	***(Å,°)***	α 88.70(0)	β 75.45(0)	γ 62.68(0)		
R-Factor (%):	7.05	***Temperature(K):***	100	***Density(g/cm³):***	1.370		

$Zn_8[\mu_4\text{-}C_{24}H_{14}N_2O_4\text{-}\kappa^2N,N',\kappa^7O,O',O'',O''']_4[H_2O]_4$

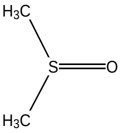

H_2O

H_3C—COMe

CAVMIX

Reference: Chengfeng Zhu, Weimin Xuan, Yong Cui (2012) *Dalton Trans.* ,**41,**3928

Formula: ($C_{100}H_{114}N_6O_{24}S_5Zn_7$)n

Compound Name: catena-(tris(μ_5-N,N'-bis(3-t-Butyl-5-carboxylato-2-oxybenzylidene)benzene-1,2-diamine)-(μ_4-oxo)-pentakis(dimethylsulfoxide)-hepta-zinc)

Space Group:	P21/n	***Cell:***	***a*** 22.286(2)	***b*** 19.845(2)	***c*** 34.290(4)
Space Group No.:	14	***(Å,°)***	α 90.00	β 90.97(0)	γ 90.00
R-Factor (%):	7.52	***Temperature(K):***	123	***Density(g/cm³):***	1.052

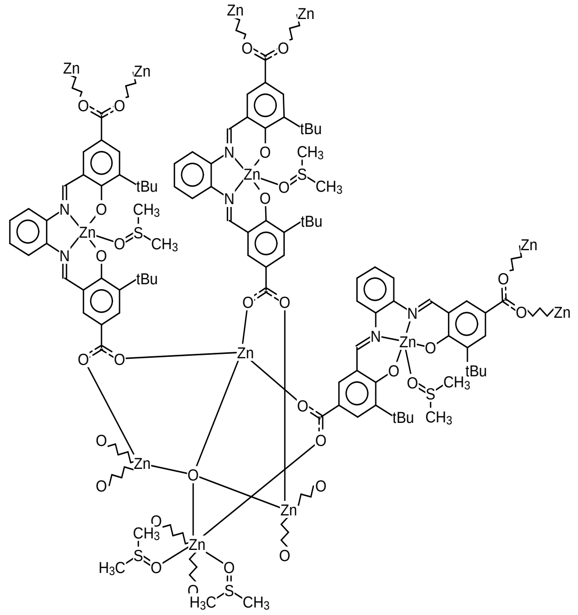

CAVMOD

Reference: Chengfeng Zhu, Weimin Xuan, Yong Cui (2012) *Dalton Trans.* ,**41,**3928

Formula: ($C_{74}H_{75}N_7O_{15}Zn_4$)n,4n($C_4H_9N_1O_1$),2n($H_2O_1$)

Compound Name: catena-(bis(μ_4-N,N'-bis(3-t-Butyl-5-carboxylato-2-oxybenzylidene)benzene-1,2-diamine)-(μ_2-4,4'-bipyridine)-(μ_2-oxo)-aqua-dimethylacetamide-tetra-zinc dimethylacetamide solvate dihydrate)

Space Group:	P21/n	***Cell:***	***a*** 23.001(0)	***b*** 14.058(0)	***c*** 31.238(0)
Space Group No.:	14	***(Å,°)***	α 90.00	β 90.88(0)	γ 90.00
R-Factor (%):	10.60	***Temperature(K):***	123	***Density(g/cm³):***	1.281

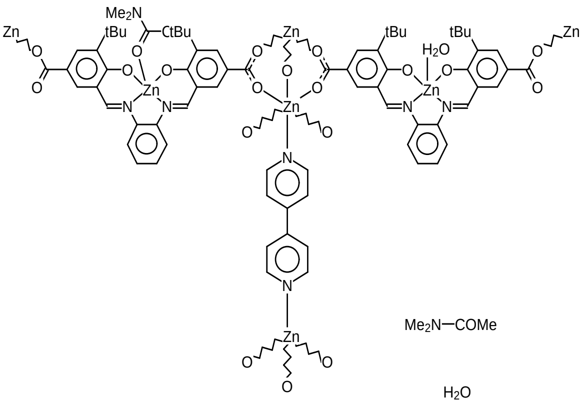

Me_2N—COMe

H_2O

CAVNUJ

Reference: K.L.Peretti, H.Ajiro, C.T.Cohen, E.B.Lobkovsky, G.W.Coates (2005) *J.Am.Chem.Soc.* ,**127,**11566

Formula: $C_{36}H_{46}Co_1N_2O_2$

Compound Name: N,N'-bis(3,5-di-t-butylsalicylidene-1,2-phenylenediamine)-cobalt(ii)

Space Group:	P-1	***Cell:***	***a*** 9.215(0)	***b*** 12.949(0)	***c*** 14.380(0)
Space Group No.:	2	***(Å,°)***	α 107.64(0)	β 93.62(0)	γ 95.85(0)
R-Factor (%):	3.26	***Temperature(K):***	173	***Density(g/cm³):***	1.226

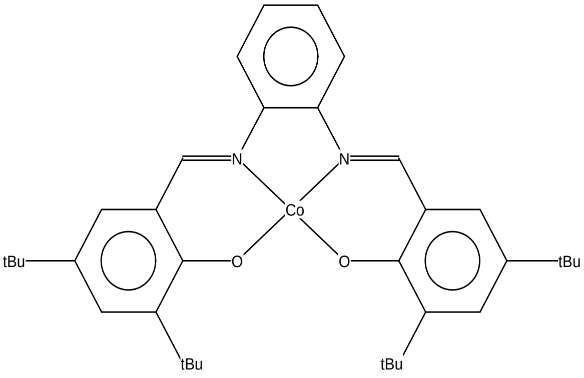

Search: search26 (Tue Dec 2 15:48:50 2014): Hits 81-84

CAZSUT

Reference: Po-Heng Lin, Wen-Bin Sun, Yong-Mei Tian, Peng-Fei Yan, L.Ungur, L.F.Chibotaru, M.Murugesu (2012) *Dalton Trans.* ,**41,**12349

Formula: $C_{50} H_{44} N_4 O_9 Yb_2, 2(C_1 H_2 Cl_2)$

Compound Name: (μ_2-2,2'-(1,2-Phenylenebis(nitrilomethylylidene))diphenoxo)-bis(acetylacetonato)-(2,2'-(1,2-phenylenebis(nitrilomethylylidene))-diphenolato)-aqua-di-ytterbium dichloromethane solvate

Space Group:	P21/n	***Cell:***	***a*** 15.397(0)	***b*** 19.228(0)	***c*** 17.805(0)
Space Group No.:	14	***(Å,°)***	α 90.00	β 91.77(0)	γ 90.00

R-Factor (%): 3.37 ***Temperature(K):*** 296 ***Density(g/cm^3):*** 1.716

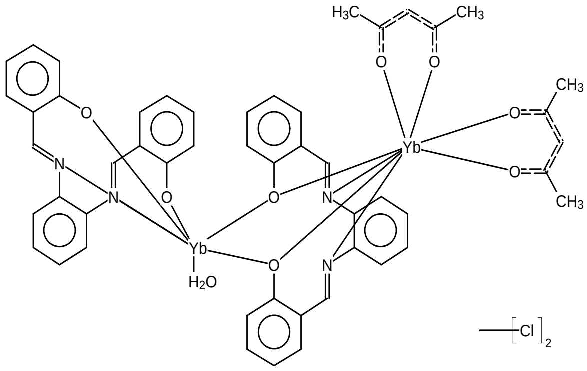

CEJQEO

Reference: Chen Wang, Yong-Qian Hou, Jun Li, Feng-Xing Zhang (2006) *Z.Kristallogr.-New Cryst.Struct.* ,**221,**51

Formula: $C_{28} H_{23} Mn_1 N_2 O_5$

Compound Name: Methanol-(N,N'-o-phenylenebis(salicylaldiminato)-N,N',O,O')-(salicylaldehydato-O)-manganese(iii)

Space Group:	P21/c	***Cell:***	***a*** 10.321(1)	***b*** 13.503(1)	***c*** 17.390(2)
Space Group No.:	14	***(Å,°)***	α 90.00	β 93.95(0)	γ 90.00

R-Factor (%): 3.83 ***Temperature(K):*** 295 ***Density(g/cm^3):*** 1.435

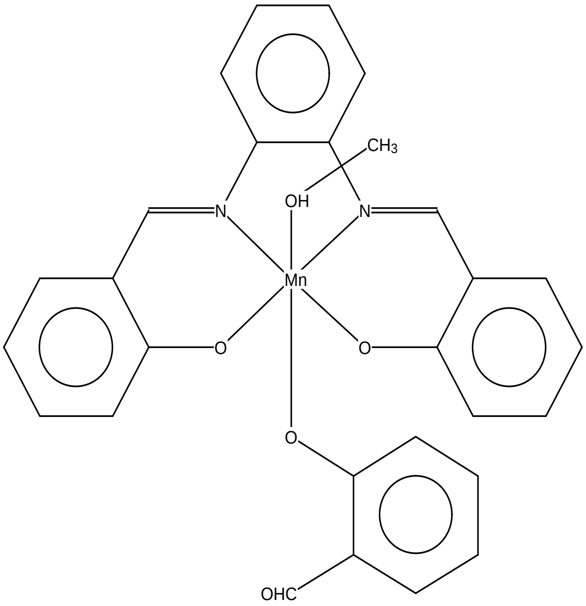

CEJVOE

Reference: Fan Yang, Pengfei Yan, Quan Li, Peng Chen, Guangming Li (2012) *Eur.J.Inorg.Chem.* ,4287

Formula: $C_{72} H_{63} Dy_3 N_6 O_{18}, 3(C_1 H_4 O_1), H_2 O_1$

Compound Name: bis(μ_2-N,N'-bis(3-methoxysalicylidene)benzene-1,2-diamine)-(μ_2-acetato)-bis(acetato)-(N,N'-bis(3-methoxysalicylidene)benzene-1,2-diamine)-tri-dysprosium methanol solvate monohydrate

Space Group:	P-1	***Cell:***	***a*** 13.960(3)	***b*** 14.342(3)	***c*** 19.529(4)
Space Group No.:	2	***(Å,°)***	α 90.05(3)	β 104.17(3)	γ 98.91(3)

R-Factor (%): 5.59 ***Temperature(K):*** 293 ***Density(g/cm^3):*** 1.688

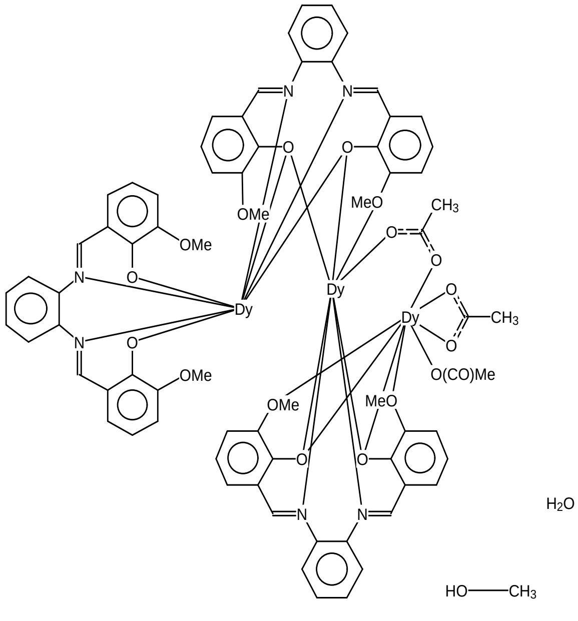

CEJVUK

Reference: Fan Yang, Pengfei Yan, Quan Li, Peng Chen, Guangming Li (2012) *Eur.J.Inorg.Chem.* ,4287

Formula: $C_{62} H_{50} Dy_3 N_9 O_{17}, C_1 H_4 O_1, 2(H_2 O_1), C_1 H_2 Cl_2$

Compound Name: bis(μ_2-N,N'-bis(salicylidene)benzene-1,2-diamine)-tris(nitrato)-(methanol)-(N,N'-bis(salicylidene)benzene-1,2-diamine)-tri-dysprosium dichloromethane methanol solvate dihydrate

Space Group:	P-1	***Cell:***	***a*** 13.785(3)	***b*** 15.175(3)	***c*** 19.161(4)
Space Group No.:	2	***(Å,°)***	α 86.08(3)	β 71.73(3)	γ 73.39(3)

R-Factor (%): 6.81 ***Temperature(K):*** 293 ***Density(g/cm^3):*** 1.670

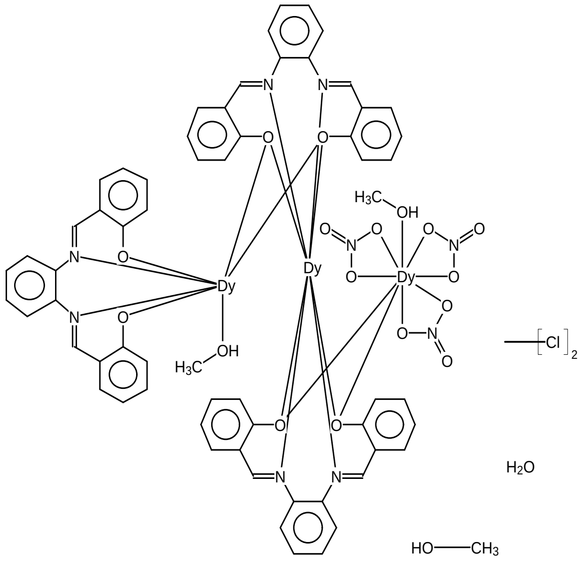

Search: search26 (Tue Dec 2 15:48:50 2014): Hits 85-88

CERDOT

Reference: Shi Wang, P.Day, J.D.Wallis, P.N.Horton, M.B.Hursthouse (2006) *Polyhedron* ,**25,**2583

Formula: $C_6 H_4 S_4{}^{1+}, C_{30} H_{18} Cr_1 N_4 O_2 S_2{}^{1-}$

Compound Name: Tetrathiafulvalenium (N,N'-bis(2-oxy-1-naphthylmethylidene)-1,2-phenylenediamine)-bis(isothiocyanato)-chromium(iii)

Space Group:	P21/c	***Cell:***	***a***	11.503(1)	***b***	17.988(3)	***c***	16.285(1)
Space Group No.:	14	***(Å,°)***	α	90.00	β	101.96(1)	γ	90.00

R-Factor (%): 5.33 ***Temperature(K):*** 120 ***Density(g/cm³):*** 1.586

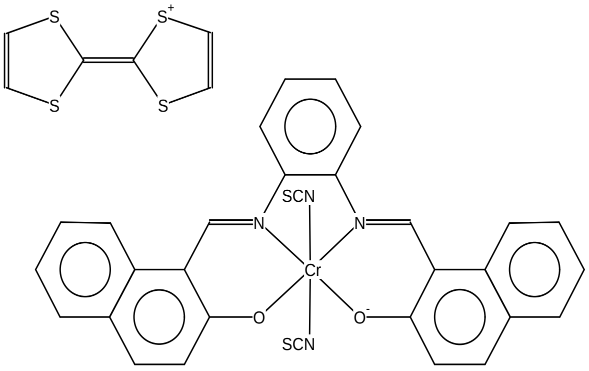

CERDUZ

Reference: Shi Wang, P.Day, J.D.Wallis, P.N.Horton, M.B.Hursthouse (2006) *Polyhedron* ,**25,**2583

Formula: $C_{10} H_8 S_8{}^{1+}, C_{30} H_{18} Cr_1 N_4 O_2 S_2{}^{1-}, 1.5(C_1 H_2 Cl_2)$

Compound Name: bis(Ethylenedithiolato)tetrathiafulvalenium (N,N'-bis(2-oxy-1-naphthylmethylidene)-1,2-phenylenediamine)-bis(isothiocyanato)-chromium(iii) dichloromethane solvate

Space Group:	Pbca	***Cell:***	***a***	15.451(1)	***b***	21.546(4)	***c***	27.380(4)
Space Group No.:	61	***(Å,°)***	α	90.00	β	90.00	γ	90.00

R-Factor (%): 7.74 ***Temperature(K):*** 120 ***Density(g/cm³):*** 1.595

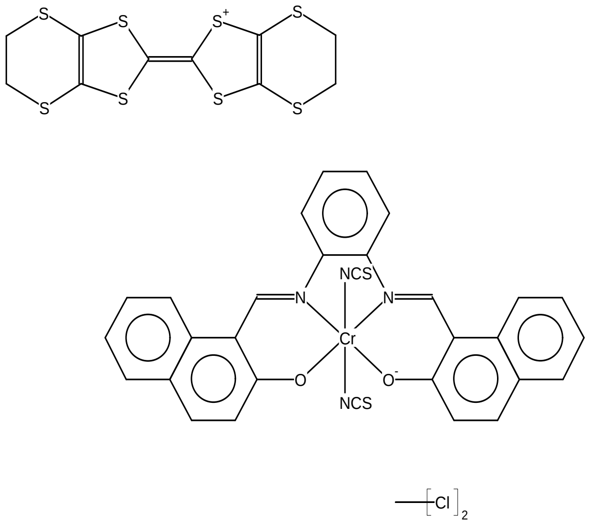

CERFAH

Reference: Shi Wang, P.Day, J.D.Wallis, P.N.Horton, M.B.Hursthouse (2006) *Polyhedron* ,**25,**2583

Formula: $C_{11} H_{10} O_1 S_8{}^{1+}, C_{30} H_{18} Cr_1 N_4 O_2 S_2{}^{1-}, 2.5(C_1 H_2 Cl_2)$

Compound Name: (1-Hydroxypropylene-2,3-dithiolato)-(ethylenedithiolato) tetrathiafulvalenium (N,N'-bis(2-oxy-1-naphthylmethylidene)-1,2-phenylenediamine)-bis(isothiocyanato)-chromium(iii) dichloromethane solvate

Space Group:	Pbca	***Cell:***	***a***	15.355(1)	***b***	21.699(1)	***c***	28.252(2)
Space Group No.:	61	***(Å,°)***	α	90.00	β	90.00	γ	90.00

R-Factor (%): 16.00 ***Temperature(K):*** 120 ***Density(g/cm³):*** 1.707

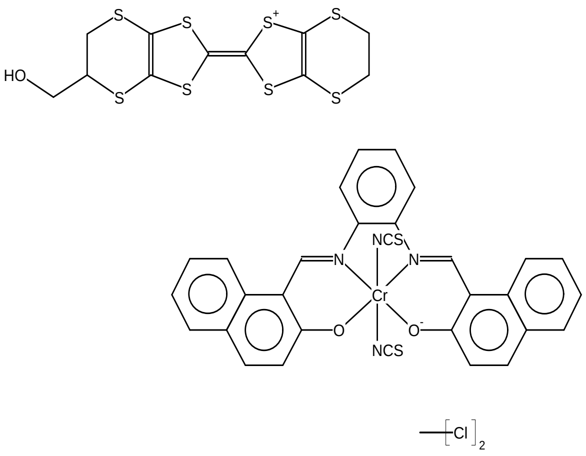

CEVDIR

Reference: V.Madhu, S.K.Das (2006) *J.Chem.Sci.(Bangalore,India)* , **118,**611

Formula: $C_{48} H_{32} Cu_1 Mn_2 N_8 O_6 S_4, 4(C_3 H_7 N_1 O_1)$

Compound Name: bis(μ_2-1,2-dicyanoethylenedithiolato)-diaqua-bis(N,N'-o-phenylenebis(salicylideneiminato))-copper(ii)-di-manganese(iii) dimethylformamide solvate

Space Group:	P21/c	***Cell:***	***a***	13.433(4)	***b***	16.283(5)	***c***	15.072(4)
Space Group No.:	14	***(Å,°)***	α	90.00	β	107.78(0)	γ	90.00

R-Factor (%): 6.18 ***Temperature(K):*** 298 ***Density(g/cm³):*** 1.493

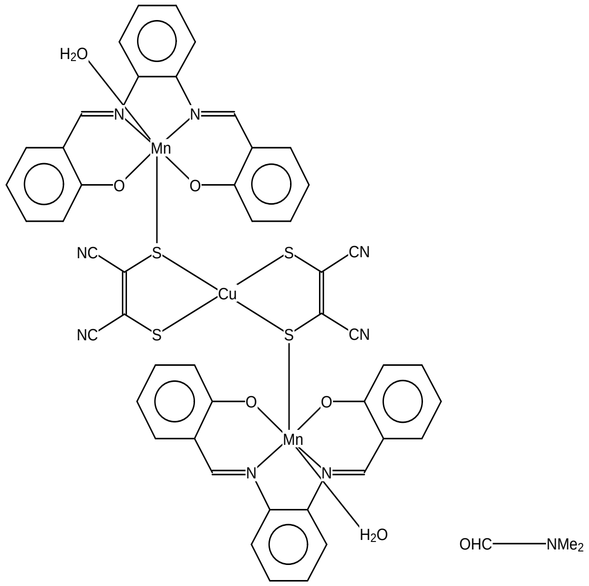

Search: search26 (Tue Dec 2 15:48:50 2014): Hits 89-92

CEYZUB

Reference: B.J.Kennedy, G.D.Fallon, B.M.K.C.Gatehouse, K.S.Murray (1984) *Inorg.Chem.* ,**23**,580

Formula: $C_{24}\ H_{20}\ Co_1\ N_4\ O_2$

Compound Name: 2-Methylimidazole-(N,N'-o-phenylene-bis(salicylaldiminato))-cobalt(ii)

Space Group: P21/n ***Cell:*** ***a*** 14.380(8) ***b*** 14.355(8) ***c*** 9.971(5)
Space Group No.: 14 ***(Å,°)*** α 90.00 β 98.33(5) γ 90.00

R-Factor (%): 6.20 ***Temperature(K):*** 295 ***Density(g/cm³):*** 1.485

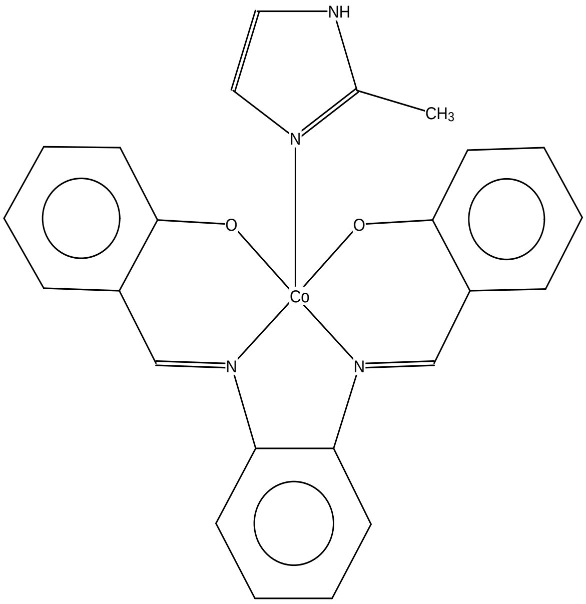

CEZYUB

Reference: A.Terzis, D.Mentzafos, H.A.Tajmir-Riahi (1984) *Inorg.Chim.Acta* ,**84,**187

Formula: $C_{40}\ H_{28}\ Ce_1\ N_4\ O_4$

Compound Name: bis(N,N'-Disalicylidene-1,2-phenylenediamino)-cerium(iv)

Space Group: P21/c ***Cell:*** ***a*** 11.090(2) ***b*** 16.332(3) ***c*** 18.916(3)
Space Group No.: 14 ***(Å,°)*** α 90.00 β 70.10(2) γ 90.00

R-Factor (%): 3.80 ***Temperature(K):*** 295 ***Density(g/cm³):*** 1.585

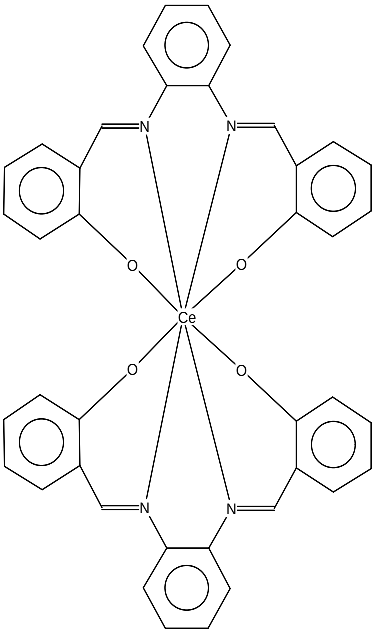

CIBPEJ

Reference: Tianzhi Yu, Kai Zhang, Yuling Zhao, Changhui Yang, Hui Zhang, Duowang Fan, Wenkui Dong (2007) *Inorg.Chem.Commun.* ,**10**,401

Formula: $C_{60}\ H_{70}\ N_8\ O_8\ Zn_3,4(C_1\ H_1\ Cl_3)$

Compound Name: bis(μ_2-Acetato)-bis(μ_2-N,N'-bis(4-(diethylamino)salicylidene)-1,2-phenylenediaminato)-tri-zinc(ii) chloroform solvate

Space Group: P-1 ***Cell:*** ***a*** 12.173(1) ***b*** 12.328(1) ***c*** 14.624(1)
Space Group No.: 2 ***(Å,°)*** α 69.66(0) β 70.16(0) γ 74.25(0)

R-Factor (%): 4.97 ***Temperature(K):*** 293 ***Density(g/cm³):*** 1.485

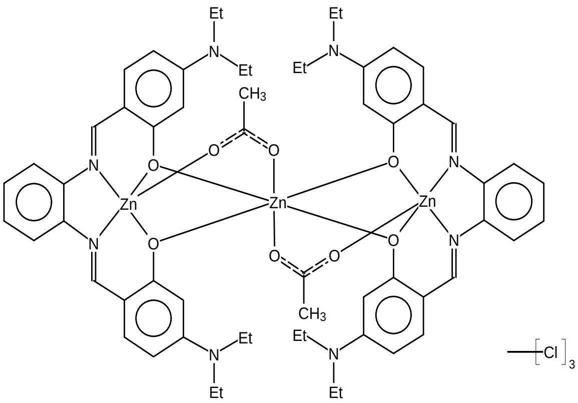

CIBPEJ01

Reference: Tianzhi Yu, Kai Zhang, Yuling Zhao, Changhui Yang, Hui Zhang, Long Qian, Duowang Fan, Wenkui Dong, Lili Chen, Yongqing Qiu (2008) *Inorg.Chim.Acta* ,**361,**233

Formula: $C_{60}\ H_{70}\ N_8\ O_8\ Zn_3,4(C_1\ H_1\ Cl_3)$

Compound Name: bis(μ_2-Acetato)-bis(μ_2-N,N'-bis(4-(diethylamino)salicylidene)-1,2-phenylenediaminato)-tri-zinc(ii) chloroform solvate

Space Group: P-1 ***Cell:*** ***a*** 12.173(1) ***b*** 12.328(1) ***c*** 14.624(1)
Space Group No.: 2 ***(Å,°)*** α 69.66(0) β 70.16(0) γ 74.25(0)

R-Factor (%): 4.97 ***Temperature(K):*** 293 ***Density(g/cm³):*** 1.485

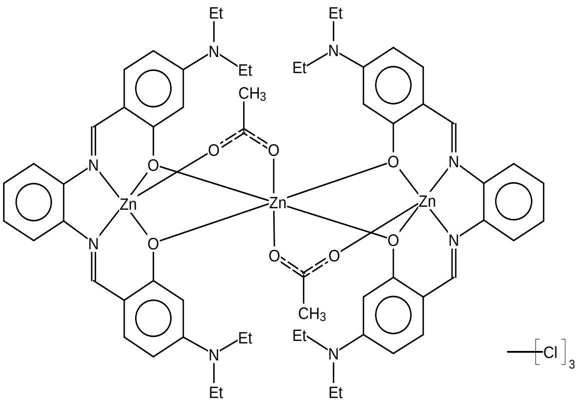

Search: search26 (Tue Dec 2 15:48:50 2014): Hits 93-96

CINQIZ

Reference: D.K.Dey, M.K.Saha, M.K.Das, N.Bhartiya, R.K.Bansal, G.Rosair, S.Mitra (1999) *Polyhedron* ,**18,**2687

Formula: $C_{30}\ H_{36}\ N_2\ O_4\ Sn_1$

Compound Name: Di-n-butyl-(N,N'-bis(3-methoxysalicylaldehyde)-1,12-phenylenedi-iminato)-tin(iv)

Space Group: P-1 ***Cell:*** ***a*** 13.428(2) ***b*** 14.013(2) ***c*** 16.498(3)
Space Group No.: 2 ***(Å,°)*** α 71.59(1) β 69.61(1) γ 88.13(1)

R-Factor (%): 2.67 ***Temperature(K):*** 160 ***Density(g/cm³):*** 1.467

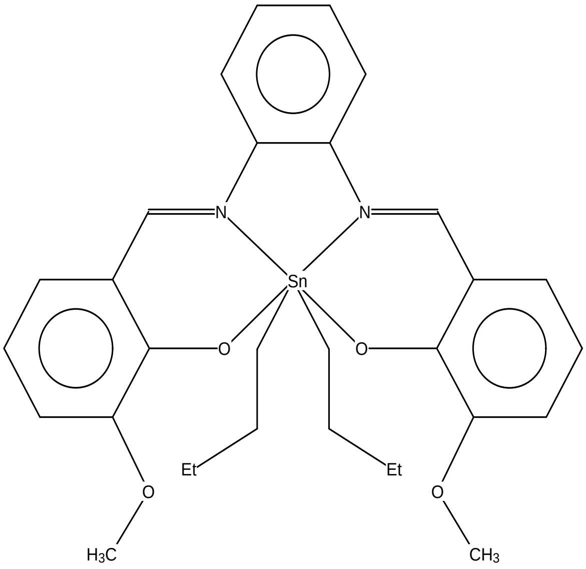

CINSAU

Reference: S.Chakraborty, S.Dinda, R.Bhattacharyya, A.K.Mukherjee (2006) *Z.Kristallogr.* ,**221,**606

Formula: $C_{20}\ H_{16}\ N_2\ O_5\ U_1$

Compound Name: aqua-(N,N'-bis(salicylidene)-o-phenylenediaminato-N,N',O,O')-dioxo-uranium(vi)

Space Group: Pbca ***Cell:*** ***a*** 21.178(1) ***b*** 7.728(1) ***c*** 22.016(1)
Space Group No.: 61 ***(Å,°)*** α 90.00 β 90.00 γ 90.00

R-Factor (%): 2.42 ***Temperature(K):*** 293 ***Density(g/cm³):*** 2.221

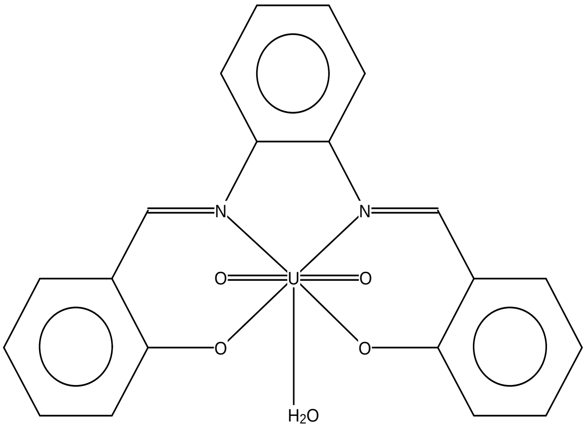

CIWXEM

Reference: Guofeng Yu, Yu Ding, Li Wang, Zhengbing Fu, Xinliang Hu (2008) *Acta Crystallogr.,Sect.E:Struct.Rep.Online* ,**64,** m504

Formula: $C_{40}\ H_{28}\ Cu_2\ N_4\ O_4,2(C_3\ H_7\ N_1\ O_1)$

Compound Name: bis(μ_2-2,2'-(o-Phenylenebis(nitrilomethylidyne))diphenolato)-di-copper(ii) N,N'-dimethylformamide solvate

Space Group: P21/n ***Cell:*** ***a*** 8.186(0) ***b*** 14.792(1) ***c*** 16.958(1)
Space Group No.: 14 ***(Å,°)*** α 90.00 β 93.25(0) γ 90.00

R-Factor (%): 5.09 ***Temperature(K):*** 294 ***Density(g/cm³):*** 1.461

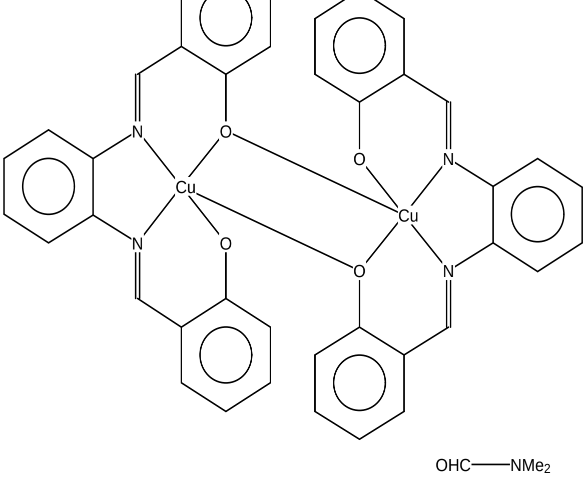

COJBIN

Reference: Mieock Kim, T.J.Taylor, F.P.Gabbai (2008) *J.Am.Chem.Soc.* ,**130,**6332

Formula: $0.5(C_{12}\ F_{10}\ Hg_1),C_{20}\ H_{14}\ N_2\ O_2\ Pd_1,C_2\ H_4\ Cl_2$

Compound Name: hemikis(bis(pentafluorophenyl)-mercury) (N,N'-o-phenylene-bis(salicylidenealdiminato))-palladium(ii) 1,2-dichloroethane solvate

Space Group: P21/n ***Cell:*** ***a*** 13.794(0) ***b*** 9.940(0) ***c*** 18.774(1)
Space Group No.: 14 ***(Å,°)*** α 90.00 β 92.15(0) γ 90.00

R-Factor (%): 3.47 ***Temperature(K):*** 295 ***Density(g/cm³):*** 2.032

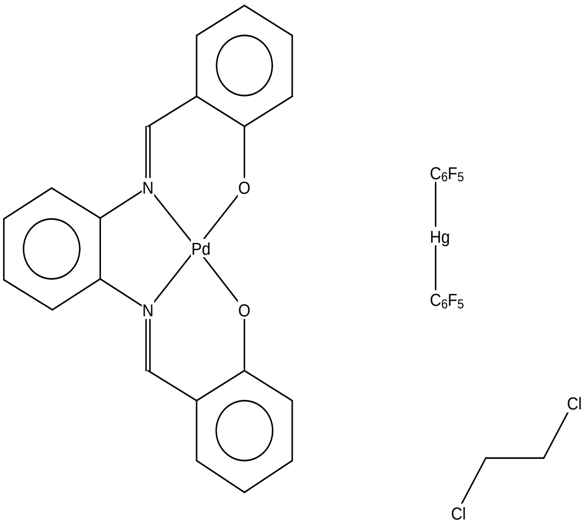

Search: search26 (Tue Dec 2 15:48:50 2014): Hits 97-100

CORHOG

Reference: M.F.Summers, L.G.Marzilli, N.Bresciani-Pahor, L.Randaccio (1984) *J.Am.Chem.Soc.* ,**106,**4478

Formula: C_{27} H_{24} Co_1 N_3 O_2,H_2 O_1

Compound Name: Ethyl-pyridine-(bis(salicylidene)-o-phenylenediamine-N,N',O,O')-cobalt monohydrate

Space Group:	P21/c	***Cell:***	***a***	12.140(7)	***b***	12.736(8)	***c***	15.822(9)
Space Group No.:	14	***(Å,°)***	α	90.00	β	100.20(10)	γ	90.00

R-Factor (%): 3.50 ***Temperature(K):*** 295 ***Density(g/cm3):*** 1.378

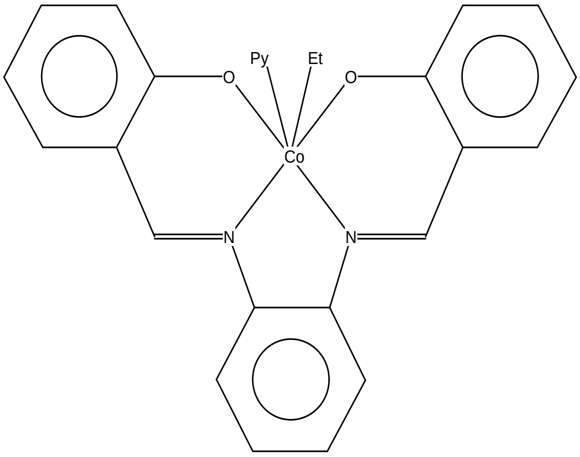

H_2O

CORHUM

Reference: M.F.Summers, L.G.Marzilli, N.Bresciani-Pahor, L.Randaccio (1984) *J.Am.Chem.Soc.* ,**106,**4478

Formula: C_{27} H_{21} Co_1 N_4 O_2,H_2 O_1

Compound Name: Cyanomethyl-pyridine-(bis(salicylidene)-o-phenylenediamine-N,N',O,O')-cobalt monohydrate

Space Group:	P21/n	***Cell:***	***a***	13.964(7)	***b***	12.219(7)	***c***	14.903(8)
Space Group No.:	14	***(Å,°)***	α	90.00	β	110.60(10)	γ	90.00

R-Factor (%): 4.90 ***Temperature(K):*** 295 ***Density(g/cm3):*** 1.424

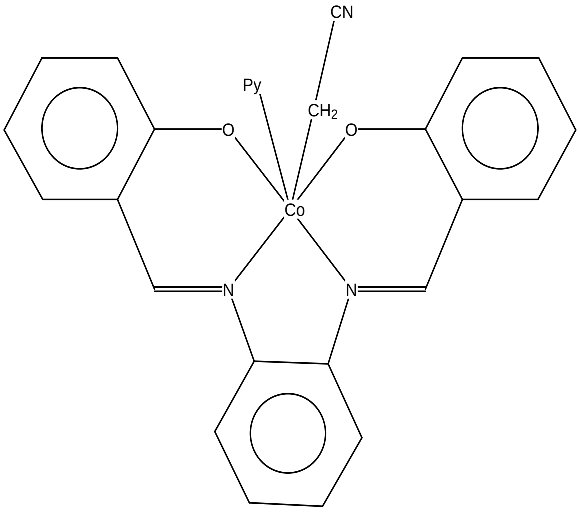

H_2O

COSALP10

Reference: N.B.Pahor, M.Calligaris, P.Delise, G.Dodic, G.Nardin, L.Randaccio (1976) *J.Chem.Soc.,Dalton Trans.* ,2478

Formula: C_{20} H_{14} Co_1 N_2 O_2

Compound Name: (N,N'-(o-Phenylene)-bis(salicylideneaminato))-cobalt(ii)

Space Group:	P21/n	***Cell:***	***a***	10.681(5)	***b***	8.354(4)	***c***	18.185(8)
Space Group No.:	14	***(Å,°)***	α	90.00	β	105.30(10)	γ	90.00

R-Factor (%): 4.10 ***Temperature(K):*** 295 ***Density(g/cm3):*** 1.584

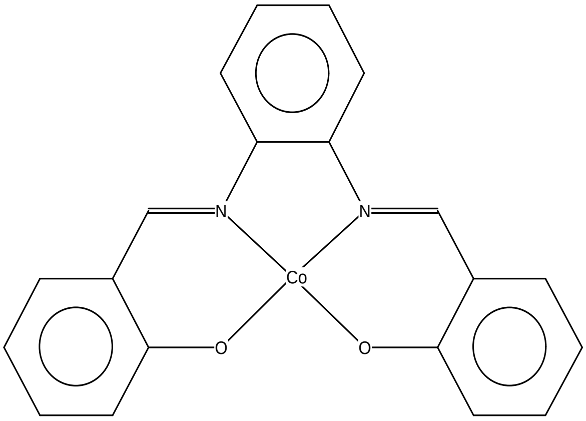

COSALP11

Reference: N.B.Pahor, M.Calligaris, P.Delise, G.Dodic, G.Nardin, L.Randaccio (1976) *J.Chem.Soc.,Dalton Trans.* ,2478

Formula: C_{20} H_{14} Co_1 N_2 O_2

Compound Name: (N,N'-(o-Phenylene)-bis(salicylideneaminato))-cobalt(ii)

Space Group:	P212121	***Cell:***	***a***	16.755(7)	***b***	17.532(8)	***c***	5.362(3)
Space Group No.:	19	***(Å,°)***	α	90.00	β	90.00	γ	90.00

R-Factor (%): 4.60 ***Temperature(K):*** 295 ***Density(g/cm3):*** 1.574

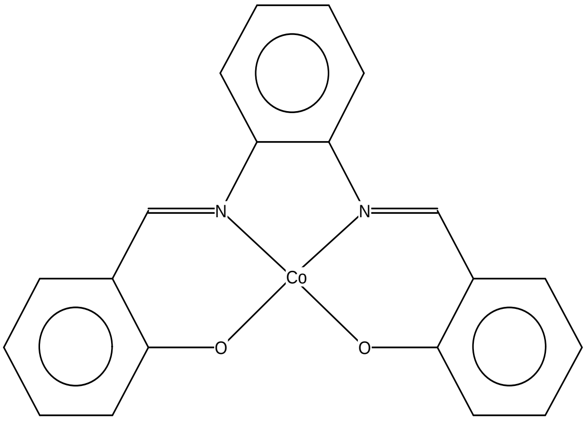

Search: search26 (Tue Dec 2 15:48:50 2014): Hits 101-104

COSZEQ

Reference: A.M.Kluwer, R.Kapre, F.Hartl, M.Lutz, A.L.Spek, A.M.Brouwer, P.W.N.M.van Leeuwen, J.N.H.Reek (2009) *Proc.Nat.Acad.Sci.USA* ,**106,**10460

Formula: $C_{131} H_{156} Fe_2 N_9 O_{11} P_1 S_2 Zn_3$

Compound Name: (μ_4-3,3',3''-(phosphinetriyl-P)tripyridine)-(μ_2-1,3-propanedithiolato-S,S,S',S')-tris(N,N'-bis(3,5-di-t-butylsalicylidene)-1,2-phenylenediamine-N,N',O,O')-pentacarbonyl-di-iron-tri-zinc(ii) unknown solvate

Space Group: P-1 ***Cell:*** ***a*** 17.780(0) ***b*** 19.358(0) ***c*** 24.253(0)
Space Group No.: 2 ***(Å,°)*** α 87.15(0) β 88.65(0) γ 64.00(0)

R-Factor (%): 4.67 ***Temperature(K):*** 150 ***Density(g/cm3):*** 1.079

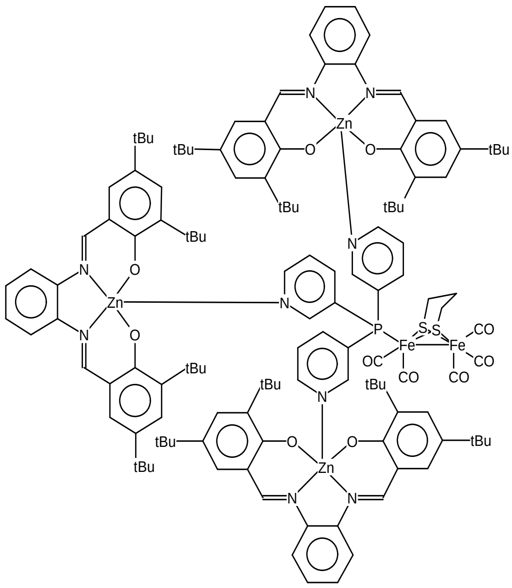

COXGIF

Reference: A.F.Marinovich, R.S.O'Mahony, J.M.Waters, T.N.M.Waters (1999) *Croat.Chem.Acta* ,**72,**685

Formula: $C_{20} H_{14} Cu_1 N_2 O_2,C_1 H_1 Cl_3$

Compound Name: N,N'-1,2-Phenylene-bis(salicylideneiminato)-copper(ii) chloroform solvate

Space Group: Pna21 ***Cell:*** ***a*** 20.159(2) ***b*** 14.918(1) ***c*** 13.329(1)
Space Group No.: 33 ***(Å,°)*** α 90.00 β 90.00 γ 90.00

R-Factor (%): 6.70 ***Temperature(K):*** 273 ***Density(g/cm3):*** 1.648

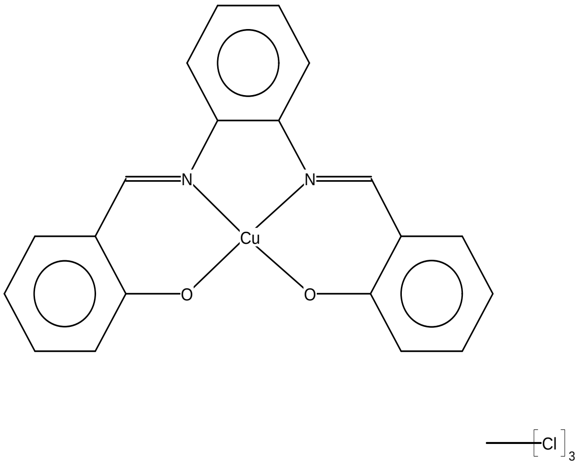

COXGOL

Reference: A.F.Marinovich, R.S.O'Mahony, J.M.Waters, T.N.M.Waters (1999) *Croat.Chem.Acta* ,**72,**685

Formula: $C_{20} H_{14} Cu_1 N_2 O_2,C_{25} H_{19} Cu_1 N_3 O_2,1.5(C_5 H_5 N_1),H_2 O_1$

Compound Name: N,N'-1,2-Phenylene-bis(salicylideneiminato)-copper(ii) N,N'-1,2-phenylene-bis(salicylideneiminato)-pyridyl-copper(ii) pyridine solvate hydrate

Space Group: P-1 ***Cell:*** ***a*** 8.748(4) ***b*** 14.499(4) ***c*** 18.725(4)
Space Group No.: 2 ***(Å,°)*** α 109.93(3) β 91.99(1) γ 101.64(2)

R-Factor (%): 4.60 ***Temperature(K):*** 273 ***Density(g/cm3):*** 1.485

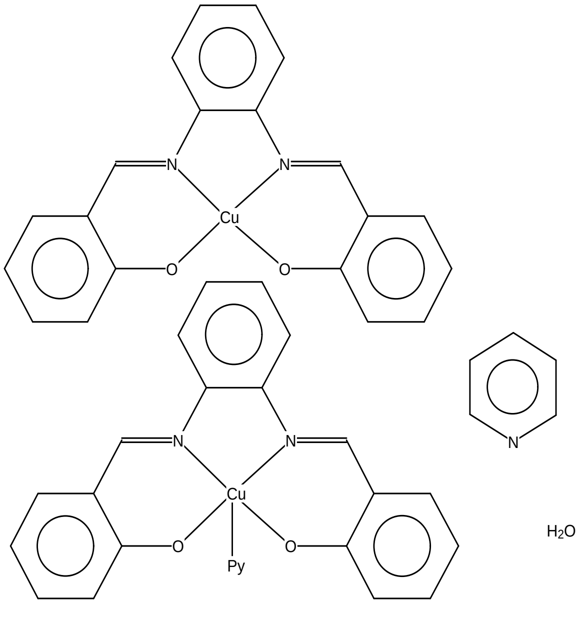

CUDFOX

Reference: M.Hirotsu, N.Ohno, T.Nakajima, C.Kushibe, K.Ueno, I.Kinoshita (2010) *Dalton Trans.* ,**39,**139

Formula: $C_{78} H_{72} Mn_2 N_4 O_{10}$ $^{2+}$,2($F_6 P_1$ $^{1-}$),2($C_2 H_6 O_1$)

Compound Name: (μ_2-4,4':5,5'-bis(9H-9,9-Dimethylxanthene)-bis(cyclohexan-1,2-diylbis(iminomethyl-2-oxyphenyl-4-yl)))-tetraethanol-di-manganese(iii) bis(hexafluorophosphate) ethanol solvate

Space Group: P21/n ***Cell:*** ***a*** 12.762(1) ***b*** 20.677(2) ***c*** 15.362(1)
Space Group No.: 14 ***(Å,°)*** α 90.00 β 102.81(0) γ 90.00

R-Factor (%): 5.49 ***Temperature(K):*** 173 ***Density(g/cm3):*** 1.443

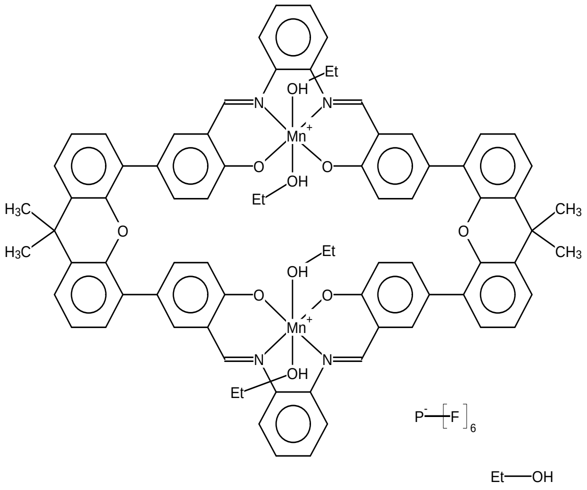

Search: search26 (Tue Dec 2 15:48:50 2014): Hits 105-108

CUJXIP

Reference: A.Blagus, B.Kaitner (2009) *Acta Crystallogr.,Sect.C:Cryst.Struct.Commun.* ,**65,**m455

Formula: $C_{28}\ H_{18}\ N_2\ Ni_1\ O_2$

Compound Name: (1,1'-(o-Phenylenebis(nitrilomethylidyne))di-2-naphtholato-κ^{4}O,N,N',O')-nickel(ii)

Synonym: (1,1'-(1,2-Phenylenebis(nitrilomethylylidene))bis(2-naphtholato)-N,N',O,O')-nickel(ii)

**Space Group:**	P21/n	**Cell:**	***a*** 15.867(0)	***b*** 7.577(0)	***c*** 16.407(0)
**Space Group No.:**	14	**(Å,°)**	α 90.00	β 97.98(0)	γ 90.00

R-Factor (%): 3.88 **Temperature(K):** 100 **Density(g/cm^3):** 1.609

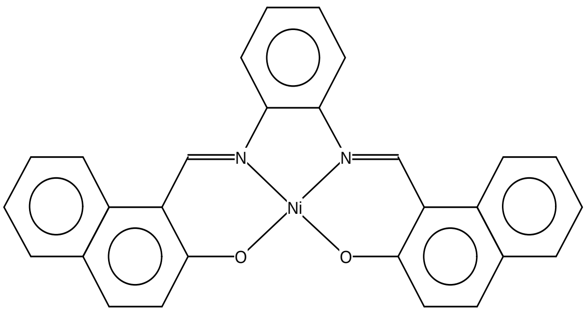

DADXIR

Reference: E.C.Escudero-Adan, M.M.Belmonte, E.Martin, G.Salassa, J.Benet-Buchholz, A.W.Kleij (2011) *J.Org.Chem.* ,**76,**5404

Formula: $C_{42}\ H_{42}\ N_8\ O_{16}\ S_4\ Zn_2,4(C_2\ H_6\ O_1\ S_1)$

Compound Name: (μ_2-1,2,4,5-tetrakis(N-(6-nitrosalicylidenealdiminato))benzene)-tetrakis(dimethyl sulfoxide)-di-zinc(ii) dimethyl sulfoxide solvate

**Space Group:**	P-1	**Cell:**	***a*** 17.278(1)	***b*** 20.330(1)	***c*** 21.131(1)
**Space Group No.:**	2	**(Å,°)**	α 63.51(0)	β 76.35(0)	γ 73.52(0)

R-Factor (%): 6.17 **Temperature(K):** 100 **Density(g/cm^3):** 1.562

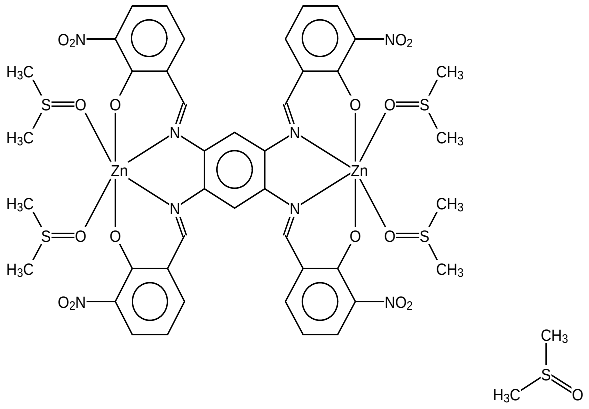

DADXOX

Reference: E.C.Escudero-Adan, M.M.Belmonte, E.Martin, G.Salassa, J.Benet-Buchholz, A.W.Kleij (2011) *J.Org.Chem.* ,**76,**5404

Formula: $C_{40}\ H_{32}\ Br_4\ N_6\ O_6\ Zn_2,2(C_3\ H_7\ N_1\ O_1)$

Compound Name: (μ_2-1,2,4,5-tetrakis(N-(6-bromosalicylidenealdiminato))benzene)-bis(N,N-dimethylformamide)-di-zinc(ii) N,N-dimethylformamide solvate

**Space Group:**	P21/c	**Cell:**	***a*** 8.792(0)	***b*** 20.225(2)	***c*** 14.387(1)
**Space Group No.:**	14	**(Å,°)**	α 90.00	β 106.64(0)	γ 90.00

R-Factor (%): 4.39 **Temperature(K):** 100 **Density(g/cm^3):** 1.747

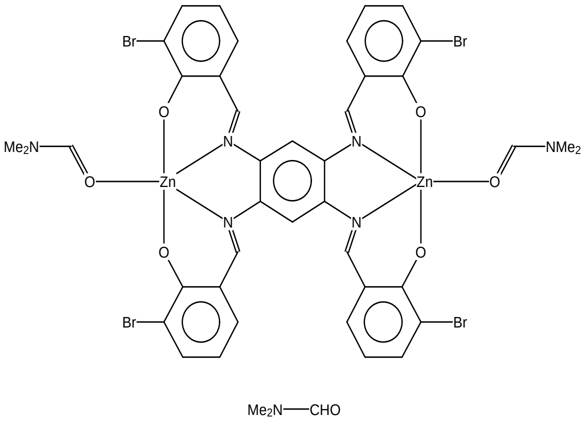

DADXUD

Reference: E.C.Escudero-Adan, M.M.Belmonte, E.Martin, G.Salassa, J.Benet-Buchholz, A.W.Kleij (2011) *J.Org.Chem.* ,**76,**5404

Formula: $C_{40}\ H_{32}\ Br_2\ N_8\ O_{10}\ Zn_2,2(C_3\ H_7\ N_1\ O_1)$

Compound Name: (μ_2-2-Bromo-6-(((5-((3-bromo-2-oxidobenzylidene)amino)-4-((2-oxido-3-nitrobenzylidene)amino)-2-((2-oxido-3-nitrobenzylidene)amino)phenyl) imino)methyl)phenolato)-bis(N,N-dimethylformamide)-di-zinc(ii) N,N-dimethylformamide solvate

**Space Group:**	P21	**Cell:**	***a*** 8.728(0)	***b*** 20.321(1)	***c*** 14.646(1)
**Space Group No.:**	4	**(Å,°)**	α 90.00	β 107.05(0)	γ 90.00

R-Factor (%): 4.82 **Temperature(K):** 100 **Density(g/cm^3):** 1.633

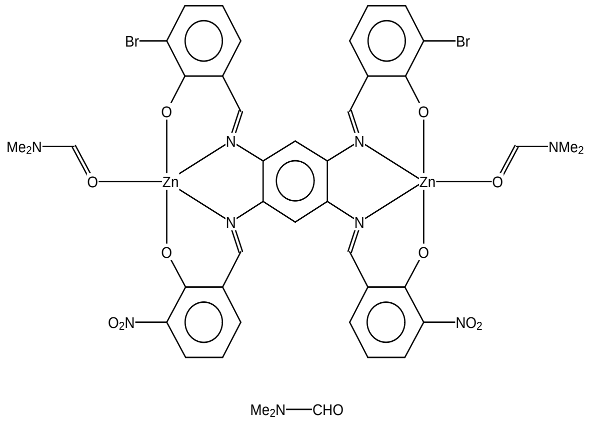

Search: search26 (Tue Dec 2 15:48:50 2014): Hits 109-112

DADYAK

Reference: E.C.Escudero-Adan, M.M.Belmonte, E.Martin, G.Salassa, J.Benet-Buchholz, A.W.Kleij (2011) *J.Org.Chem.* ,**76,**5404

Formula: $C_{46} H_{42} N_{16} O_{24} Zn_2,7(C_3 H_7 N_1 O_1)$

Compound Name: (μ_2-1,2,4,5-tetrakis(N-(4,6-dinitrosalicylidenealdiminato))benzene)-tetrakis(N,N-dimethylformamide)-di-zinc(ii) N,N-dimethylformamide solvate

Space Group:	C2/c	***Cell:***	***a*** 29.066(1)	***b*** 13.386(0)	***c*** 22.137(1)
Space Group No.:	15	***(Å,°)***	α 90.00	β 106.56(0)	γ 90.00

R-Factor (%): 5.04 ***Temperature(K):*** 100 ***Density(g/cm³):*** 1.485

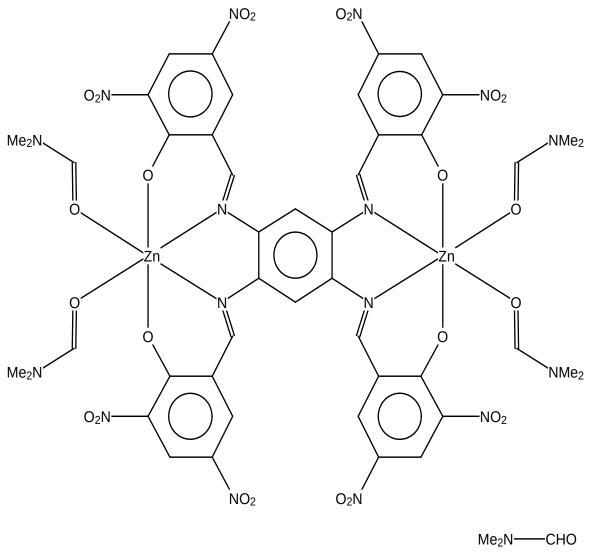

DAFJUR

Reference: M.Mastalerz, I.M.Oppel (2011) *Eur.J.Org.Chem.* ,5971

Formula: $C_{94} H_{88} N_4 O_4 Zn_2$

Compound Name: bis(μ_2-2,4-di-t-butyl-6-[((2-[(2-oxido-5-tritylbenzylidene)amino]phenyl)imino)methyl]phenolato)-di-zinc(ii)

Space Group:	P-1	***Cell:***	***a*** 9.399(0)	***b*** 11.426(0)	***c*** 19.040(0)
Space Group No.:	2	***(Å,°)***	α 97.23(0)	β 98.22(0)	γ 101.55(0)

R-Factor (%): 3.79 ***Temperature(K):*** 110 ***Density(g/cm³):*** 1.246

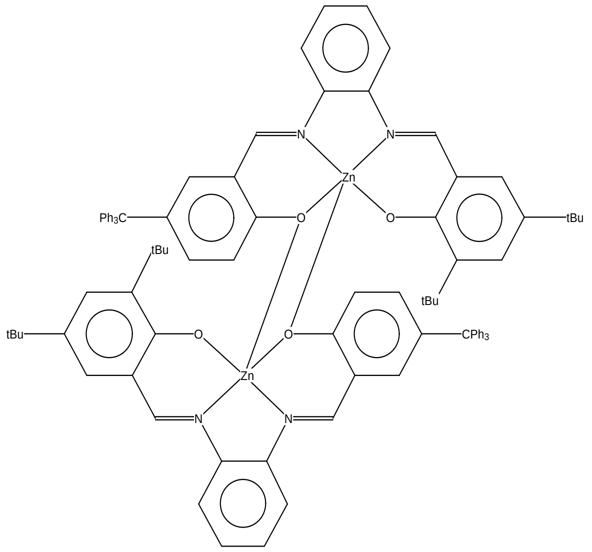

DAJLOP

Reference: D.St.C.Black, D.C.Craig, N.Kumar, L.C.H.Wong (1985) *Chem.Commun.* ,1172

Formula: $C_{28} H_{24} N_4 Ni_1 O_4,C_1 H_1 Cl_3$

Compound Name: (N,N'-bis(4,6-Dimethoxyindol-7-ylidene)-1,2-diaminobenzene-N,N',N'',N''')-nickel(ii) chloroform solvate

Synonym: (1,2-Phenylene-bis(nitrilomethylidyne)-7,7'-di(4,6-dimethoxyindolato))-nickel(ii) chloroform solvate

Space Group:	P-1	***Cell:***	***a*** 10.202(5)	***b*** 11.997(6)	***c*** 12.540(8)
Space Group No.:	2	***(Å,°)***	α 89.34(3)	β 84.88(4)	γ 66.56(5)

R-Factor (%): 5.70 ***Temperature(K):*** 295 ***Density(g/cm³):*** 1.560

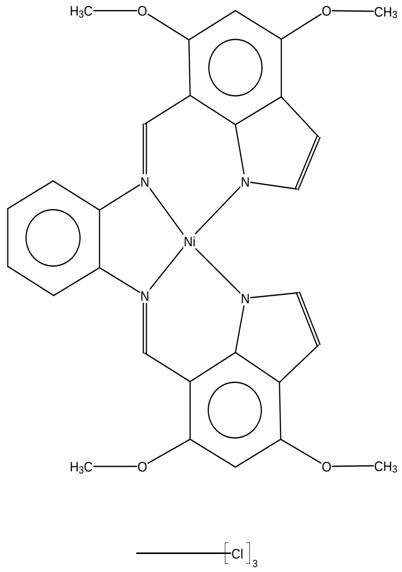

DARBEF

Reference: A.Tzubery, E.Y.Tshuva (2012) *Inorg.Chem.* ,**51,**1796

Formula: $C_{36} H_{32} N_2 O_4 Ti_1$

Compound Name: bis(2,6-dimethylphenolato)-(2,2'-(1,2-phenylenebis((nitrilo)methylylidene))diphenolato)-titanium

Space Group:	P21/n	***Cell:***	***a*** 22.886(4)	***b*** 9.457(1)	***c*** 29.041(6)
Space Group No.:	14	***(Å,°)***	α 90.00	β 100.42(0)	γ 90.00

R-Factor (%): 13.97 ***Temperature(K):*** 173 ***Density(g/cm³):*** 1.299

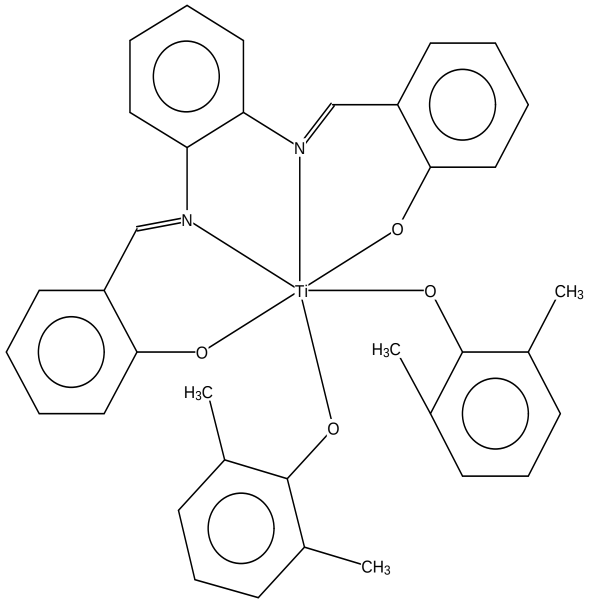

DARBIJ

Reference: A.Tzubery, E.Y.Tshuva (2012) *Inorg.Chem.* ,**51,**1796

Formula: C_{38} H_{36} N_2 O_4 Ti_1

Compound Name: bis(2,6-dimethylphenolato)-(2,2'-(1,2-phenylenebis((nitrilo)methylylidene))bis(4-methylphenolato))-titanium

Space Group:	P21/n	***Cell:***	***a*** 13.152(0)	***b*** 14.281(1)	***c*** 16.989(1)		
Space Group No.:	14	***(Å,°)***	α 90.00	β 100.44(0)	γ 90.00		
R-Factor (%):	7.07	***Temperature(K):***	173	***Density(g/cm^3):***	1.339		

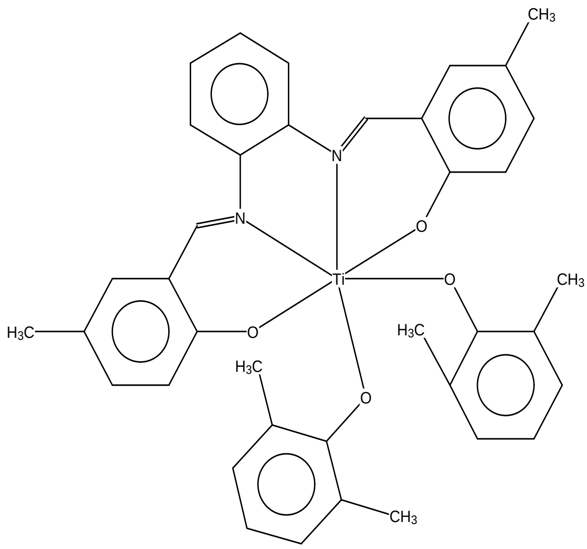

DARBOP

Reference: A.Tzubery, E.Y.Tshuva (2012) *Inorg.Chem.* ,**51,**1796

Formula: C_{44} H_{48} N_2 O_4 Ti_1

Compound Name: bis(2,6-dimethylphenolato)-(2,2'-(1,2-phenylenebis((nitrilo)methylylidene))bis(4-t-butylphenolato))-titanium

Space Group:	P21/c	***Cell:***	***a*** 9.101(4)	***b*** 26.330(10)	***c*** 17.051(7)		
Space Group No.:	14	***(Å,°)***	α 90.00	β 101.48(0)	γ 90.00		
R-Factor (%):	8.37	***Temperature(K):***	123	***Density(g/cm^3):***	1.189		

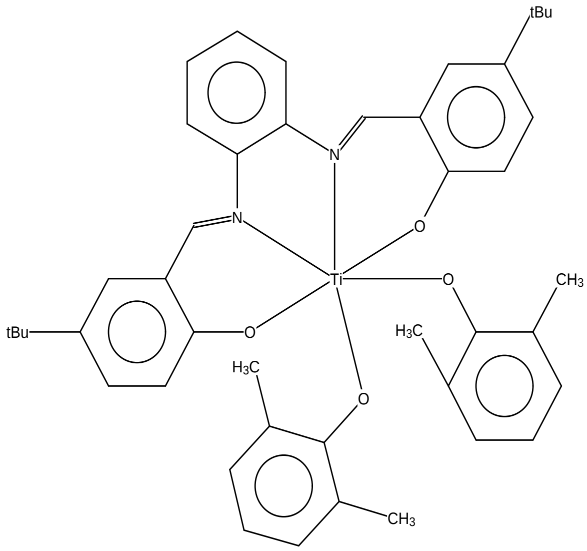

DARBUV

Reference: A.Tzubery, E.Y.Tshuva (2012) *Inorg.Chem.* ,**51,**1796

Formula: C_{52} H_{64} N_2 O_4 Ti_1

Compound Name: bis(2,6-dimethylphenolato)-(2,2'-(1,2-phenylenebis((nitrilo)methylylidene))bis(4,6-di-t-butylphenolato))-titanium

Space Group:	P21/c	***Cell:***	***a*** 13.656(1)	***b*** 18.749(1)	***c*** 18.542(1)		
Space Group No.:	14	***(Å,°)***	α 90.00	β 99.33(0)	γ 90.00		
R-Factor (%):	10.28	***Temperature(K):***	173	***Density(g/cm^3):***	1.175		

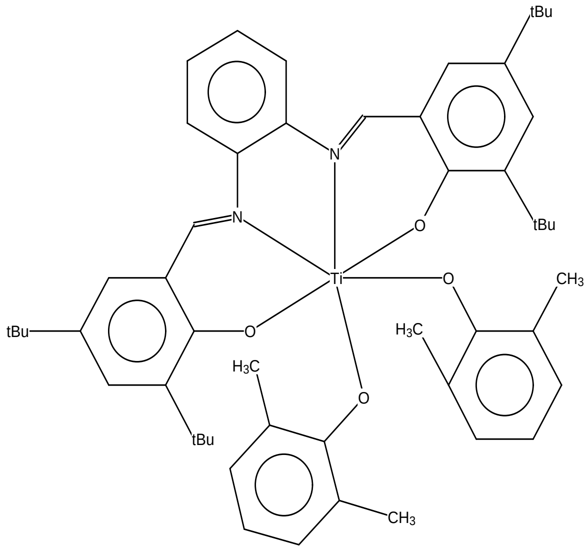

DARCAC

Reference: A.Tzubery, E.Y.Tshuva (2012) *Inorg.Chem.* ,**51,**1796

Formula: C_{38} H_{36} N_2 O_6 Ti_1

Compound Name: bis(2,6-dimethylphenolato)-(2,2'-(1,2-phenylenebis((nitrilo)methylylidene))bis(6-methoxyphenolato))-titanium

Space Group:	Cc	***Cell:***	***a*** 13.372(1)	***b*** 23.313(4)	***c*** 12.053(2)		
Space Group No.:	9	***(Å,°)***	α 90.00	β 117.45(0)	γ 90.00		
R-Factor (%):	6.25	***Temperature(K):***	173	***Density(g/cm^3):***	1.324		

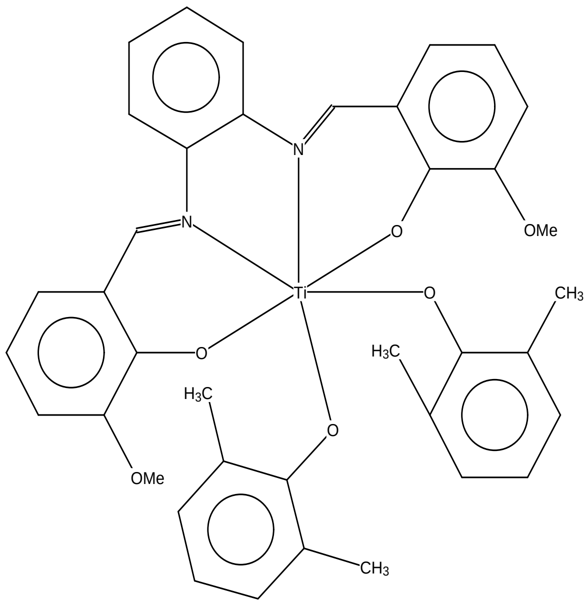

Search: search26 (Tue Dec 2 15:48:50 2014): Hits 117-120

DARCEG

Reference: A.Tzubery, E.Y.Tshuva (2012) *Inorg.Chem.* ,**51,**1796

Formula: $C_{36}\ H_{30}\ Br_2\ N_2\ O_4\ Ti_1,C_8\ H_{10}\ O_1$

Compound Name: bis(2,6-dimethylphenolato)-(2,2'-(1,2-phenylenebis((nitrilo)methylylidene))bis(6-bromophenolato))-titanium 2,6-dimethylphenol solvate

Space Group:	P21/c	***Cell:***	***a***	11.673(1)	***b***	19.951(1)	***c***	17.293(1)
Space Group No.:	14	***(Å,°)***	α	90.00	β	105.85(0)	γ	90.00

R-Factor (%): 3.75 ***Temperature(K):*** 173 ***Density(g/cm^3):*** 1.516

DARCIK

Reference: A.Tzubery, E.Y.Tshuva (2012) *Inorg.Chem.* ,**51,**1796

Formula: $C_{28}\ H_{22}\ N_2\ O_4\ Ti_1,C_1\ H_2\ Cl_2$

Compound Name: (Benzene-1,2-diolato)-(2,2'-(1,2-phenylenebis((nitrilo)methylylidene))bis(4-methylphenolato))-titanium dichloromethane solvate

Space Group:	P-1	***Cell:***	***a***	8.877(3)	***b***	9.769(3)	***c***	16.796(5)
Space Group No.:	2	***(Å,°)***	α	93.97(0)	β	104.30(0)	γ	109.98(0)

R-Factor (%): 4.83 ***Temperature(K):*** 173 ***Density(g/cm^3):*** 1.482

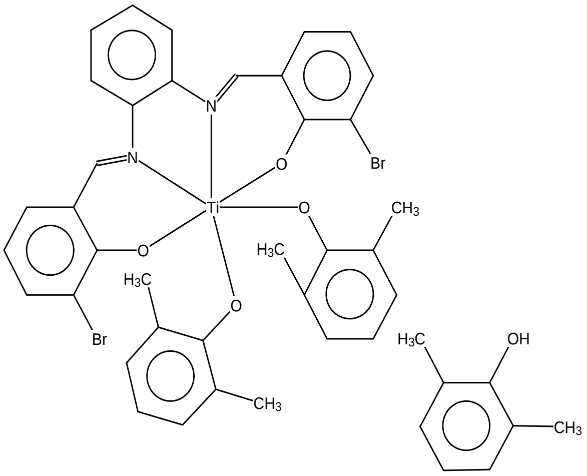

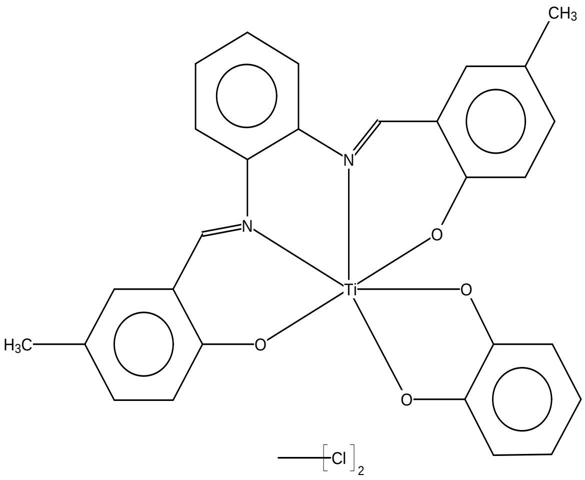

DARKEO

Reference: V.Bereau, C.Duhayon, A.Sournia-Saquet, J.-P.Sutter (2012) *Inorg.Chem.* ,**51,**1309

Formula: $C_{34}\ H_{26}\ Al_1\ N_2\ O_8\ ^{1+},C_1\ H_4\ O_1,N_1\ O_3\ ^{1-},2(H_2\ O_1)$

Compound Name: (diphenyl 3,3'-(1,2-phenylenebis((nitrilo)methylylidene))bis(4-(hydroxy)benzoatato))-diaqua-aluminium nitrate methanol solvate dihydrate

Space Group:	P21/c	***Cell:***	***a***	16.272(0)	***b***	15.363(0)	***c***	15.261(0)
Space Group No.:	14	***(Å,°)***	α	90.00	β	114.58(0)	γ	90.00

R-Factor (%): 6.49 ***Temperature(K):*** 180 ***Density(g/cm^3):*** 1.431

DARKIS

Reference: V.Bereau, C.Duhayon, A.Sournia-Saquet, J.-P.Sutter (2012) *Inorg.Chem.* ,**51,**1309

Formula: $C_{34}\ H_{24}\ Al_1\ N_4\ O_{12}\ ^{1+},N_1\ O_3\ ^{1-},1.5(C_2\ H_6\ O_1)$

Compound Name: (2,2'-(benzene-1,2-diylbis[nitrilomethylylidene])bis(4-[(4-nitrophenoxy)carbonyl]phenolate))-di-aqua-aluminium nitrate ethanol solvate

Space Group:	P-1	***Cell:***	***a***	11.791(0)	***b***	12.557(0)	***c***	14.029(0)
Space Group No.:	2	***(Å,°)***	α	87.45(0)	β	84.79(0)	γ	62.54(0)

R-Factor (%): 4.13 ***Temperature(K):*** 100 ***Density(g/cm^3):*** 1.518

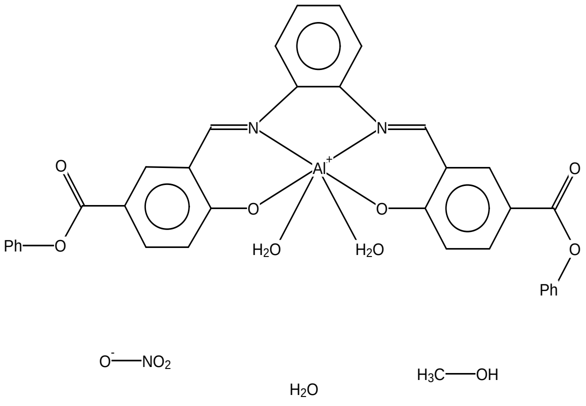

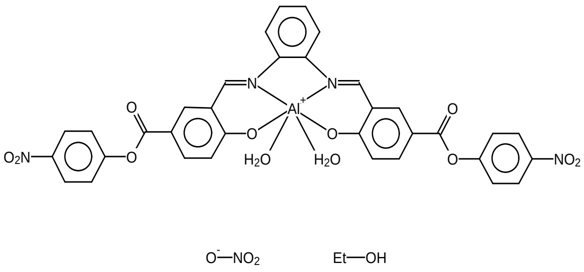

Search: search26 (Tue Dec 2 15:48:50 2014): Hits 121-124

DASSAS

Reference:	D.J.Darensbourg, D.R.Billodeaux (2005) *Inorg.Chem.* ,**44,** 1433
Formula:	C_{22} H_{19} Al_1 N_2 O_2,C_1 H_2 Cl_2
Compound Name:	Ethyl-(N,N'-bis(salicylidene)-1,2-phenylenediamine)-aluminium(iii) dichloromethane solvate

Space Group:	P21/c	***Cell:***	***a***	15.977(4)	***b***	28.208(7)	***c***	9.176(2)
Space Group No.:	14	***(Å,°)***	α	90.00	β	106.07(0)	γ	90.00
R-Factor (%):	8.65	***Temperature(K):***	110		***Density(g/cm³):***	1.522		

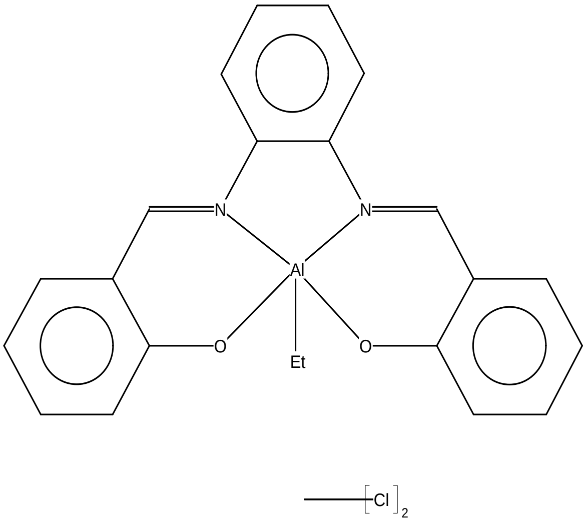

DAVGOX

Reference:	S.Biswas, K.Mitra, B.Adhikary, C.R.Lucas (2005) *Transition Met.Chem.* ,**30,**586
Formula:	C_{34} H_{27} Mn_1 N_4 O_4,C_1 H_4 O_1
Compound Name:	(N,N'-bis(Salicylidene)benzene-1,2-diamine-N,N',O,O')-(methanol)-(2-(benzimidazol-2-yl)phenolato-O)-manganese(iii) methanol solvate

Space Group:	P21/n	***Cell:***	***a***	9.804(1)	***b***	16.265(1)	***c***	18.729(2)
Space Group No.:	14	***(Å,°)***	α	90.00	β	91.26(0)	γ	90.00
R-Factor (%):	4.79	***Temperature(K):***	193		***Density(g/cm³):***	1.429		

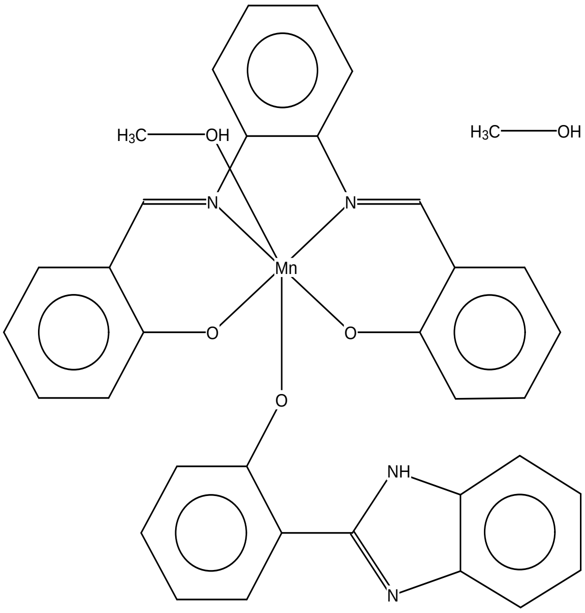

DAVGUD

Reference:	S.Biswas, K.Mitra, B.Adhikary, C.R.Lucas (2005) *Transition Met.Chem.* ,**30,**586
Formula:	C_{40} H_{29} Mn_2 N_4 O_5 $^{1+}$,Cl_1 O_4 $^{1-}$
Compound Name:	(μ_2-Hydroxo)-bis(N,N'-bis(salicylidene)benzene-1,2-diamine-N,N',O,O')-di-manganese(iii) perchlorate

Space Group:	Pcca	***Cell:***	***a***	14.469(7)	***b***	13.670(10)	***c***	18.659(4)
Space Group No.:	54	***(Å,°)***	α	90.00	β	90.00	γ	90.00
R-Factor (%):	9.10	***Temperature(K):***	299		***Density(g/cm³):***	1.539		

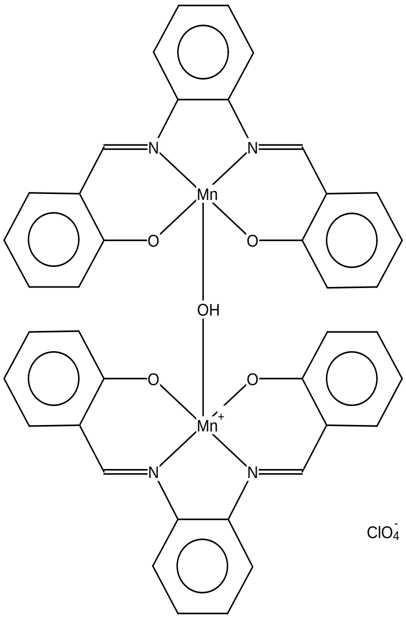

DAWJER

Reference:	Hongjun Zhu, Mei Wang, Chengbing Ma, Bo Li, Changneng Chen, Licheng Sun (2005) *J.Organomet.Chem.* ,**690,**3929
Formula:	C_{39} H_{52} Cl_2 N_2 O_3 Zr_1,2(C_3 H_6 O_1)
Compound Name:	(Acetone-O)-dichloro-(N,N'-o-phenylenebis(3,5-di-t-butylsalicylideneiminato)-N,N',O,O')-zirconium acetone solvate

Space Group:	P21/n	***Cell:***	***a***	15.953(0)	***b***	11.494(0)	***c***	25.519(1)
Space Group No.:	14	***(Å,°)***	α	90.00	β	100.69(0)	γ	90.00
R-Factor (%):	6.78	***Temperature(K):***	293		***Density(g/cm³):***	1.264		

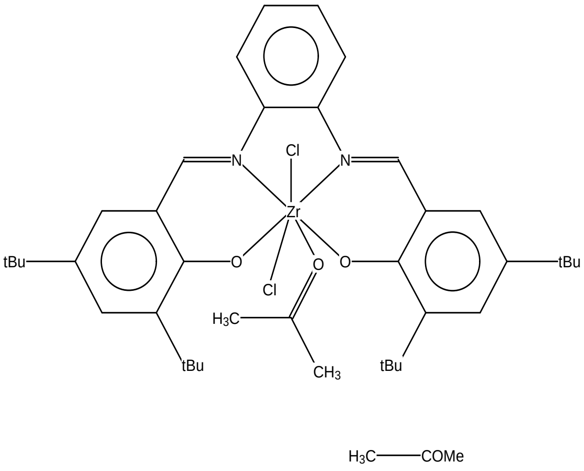

DAWJIV

Reference:	Hongjun Zhu, Mei Wang, Chengbing Ma, Bo Li, Changneng Chen, Licheng Sun (2005) *J.Organomet.Chem.* ,**690,**3929
Formula:	$C_{36}\ H_{46}\ Cl_2\ N_2\ O_2\ Zr_1,C_4\ H_8\ O_1$
Compound Name:	Dichloro-(N,N'-o-phenylenebis(3,5-di-t-butylsalicylideneiminato)-N,N',O,O')-zirconium tetrahydrofuran solvate

Space Group:	C2/c	***Cell:***	***a***	19.097(0)	***b***	19.503(0)	***c***	11.773(0)
Space Group No.:	15	***(Å,°)***	α	90.00	β	109.78(0)	γ	90.00
R-Factor (%):	6.97	***Temperature(K):***	293		***Density(g/cm³):***	1.244		

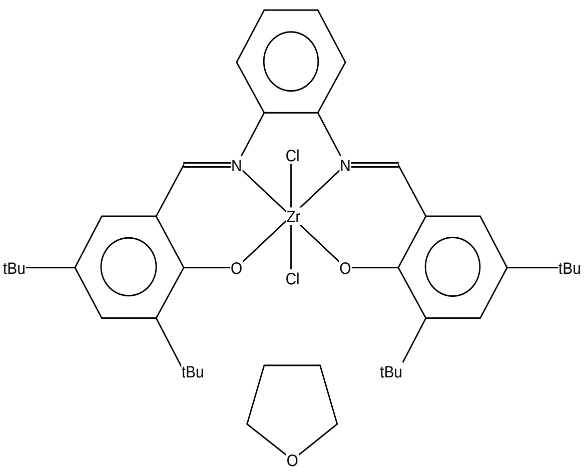

DEDPIM

Reference:	Xiaoping Yang, R.A.Jones, Qiaoyin Wu, M.M.Oye, Wing-Kit Lo, Wai-Kwok Wong, A.L.Holmes (2006) *Polyhedron* ,**25,**271
Formula:	$C_{23}\ H_{20}\ Br_2\ Cl_2\ N_2\ O_5\ Zn_2,C_1\ H_4\ O_1,0.5(C_4\ H_{10}\ O_1)$
Compound Name:	(N,N'-o-Phenylene-bis(5-bromo-3-methoxysalicylidineamine))-dichloro-methanol-di-zinc methanol diethyl ether solvate

Space Group:	P-1	***Cell:***	***a***	11.504(2)	***b***	11.857(2)	***c***	13.937(3)
Space Group No.:	2	***(Å,°)***	α	96.69(3)	β	113.13(3)	γ	111.37(3)
R-Factor (%):	5.80	***Temperature(K):***	153		***Density(g/cm³):***	1.787		

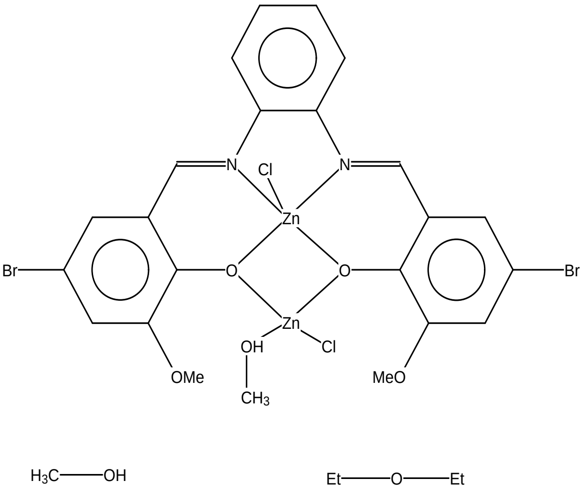

DEDPOS

Reference:	Xiaoping Yang, R.A.Jones, Qiaoyin Wu, M.M.Oye, Wing-Kit Lo, Wai-Kwok Wong, A.L.Holmes (2006) *Polyhedron* ,**25,**271
Formula:	$C_{24}\ H_{19}\ Br_2\ Eu_1\ N_4\ O_{12}\ Zn_1,C_2\ H_6\ O_1,H_2\ O_1$
Compound Name:	(μ_2-Acetato-O,O')-(μ_2-N,N'-o-phenylene-bis(5-bromo-3-methoxysalicylidineamine))-bis(nitrato-O,O')-europium-zinc ethanol solvate monohydrate

Space Group:	C2/c	***Cell:***	***a***	21.630(4)	***b***	10.911(2)	***c***	27.591(6)
Space Group No.:	15	***(Å,°)***	α	90.00	β	91.16(3)	γ	90.00
R-Factor (%):	9.45	***Temperature(K):***	153		***Density(g/cm³):***	2.034		

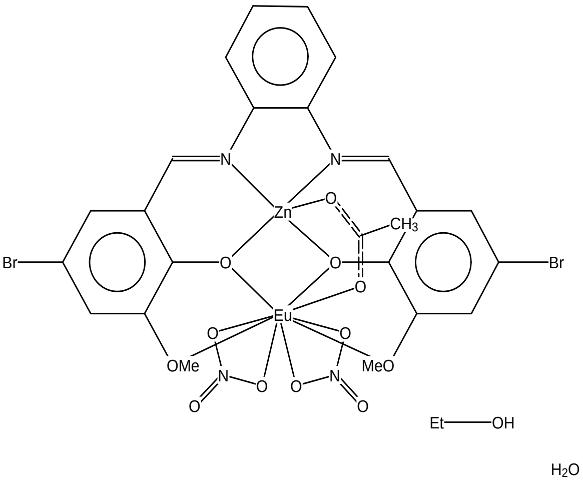

DEDPUY

Reference:	Xiaoping Yang, R.A.Jones, Qiaoyin Wu, M.M.Oye, Wing-Kit Lo, Wai-Kwok Wong, A.L.Holmes (2006) *Polyhedron* ,**25,**271
Formula:	$C_{24}\ H_{21}\ Br_2\ N_4\ Nd_1\ O_{13}\ Zn_1,C_2\ H_6\ O_1$
Compound Name:	(μ_2-Acetato-O,O')-(μ_2-N,N'-o-phenylene-bis(5-bromo-3-methoxysalicylidineamine))-aqua-bis(nitrato-O,O')-neodymium-zinc ethanol solvate

Space Group:	P-1	***Cell:***	***a***	11.002(2)	***b***	11.655(2)	***c***	14.447(3)
Space Group No.:	2	***(Å,°)***	α	84.23(3)	β	74.96(3)	γ	66.08(3)
R-Factor (%):	4.82	***Temperature(K):***	153		***Density(g/cm³):***	2.008		

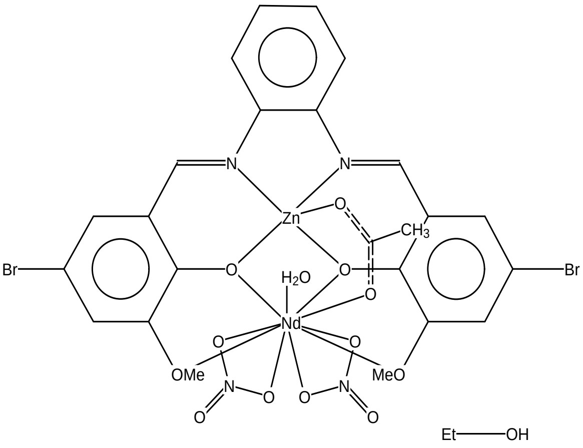

Search: search26 (Tue Dec 2 15:48:50 2014): Hits 129-132

DEJKUZ

Reference: Wai-Kwok Wong, Xiaoping Yang, R.A.Jones, J.H.Rivers, V.Lynch, Wing-Kit Lo, Dang Xiao, M.M.Oye, A.L.Holmes (2006) *Inorg.Chem.* ,**45,**4340

Formula: $C_{44} H_{42} Cl_2 N_4 Nd_1 O_{11} Zn_2{}^{1+}, Cl_1{}^{1-}, 2(C_1 H_4 O_1), 5(H_2 O_1)$

Compound Name: (μ_2-N,N'-bis(3-Methoxysalicylidene)-1,2-phenylenediamine-N,N',O,O,O',O',O'',O''')-(μ_2-N,N'-bis(3-methoxysalicylidene)-1,2-phenylenediamine-N,N',O,O,O',O')-triaqua-dichloro-neodymium(iii)-di-zinc(ii) chloride methanol solvate pentahydrate

Space Group:	P212121	***Cell:***	***a*** 13.271(3)	***b*** 13.470(3)	***c*** 31.170(6)		
Space Group No.:	19	***(Å,°)***	α 90.00	β 90.00	γ 90.00		
R-Factor (%):	7.29	***Temperature(K):***	153	***Density(g/cm³):***	1.595		

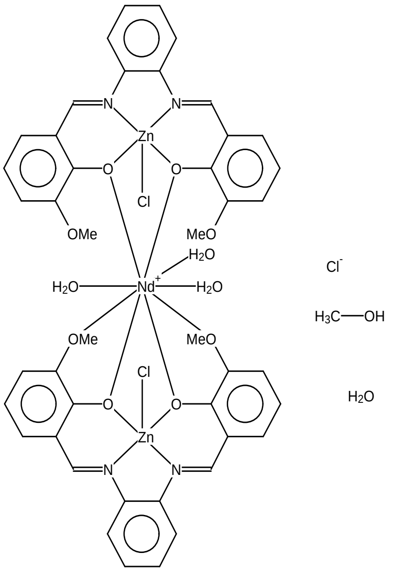

DEJLAG

Reference: Wai-Kwok Wong, Xiaoping Yang, R.A.Jones, J.H.Rivers, V.Lynch, Wing-Kit Lo, Dang Xiao, M.M.Oye, A.L.Holmes (2006) *Inorg.Chem.* ,**45,**4340

Formula: $C_{46} H_{44} Cl_6 N_4 Nd_2 O_{10} Zn_2, C_1 H_4 O_1$

Compound Name: bis(μ_2-N,N'-bis(3-Methoxysalicylidene)-1,2-phenylenediamine-N,N',O,O,O',O',O'',O''')-bis(μ_2-chloro)-tetrachloro-bis(methanol)-di-neodymium(iii)-di-zinc(ii) methanol solvate

Space Group:	P-1	***Cell:***	***a*** 13.516(3)	***b*** 14.284(3)	***c*** 14.358(3)		
Space Group No.:	2	***(Å,°)***	α 82.55(3)	β 76.20(3)	γ 72.59(3)		
R-Factor (%):	5.08	***Temperature(K):***	153	***Density(g/cm³):***	1.913		

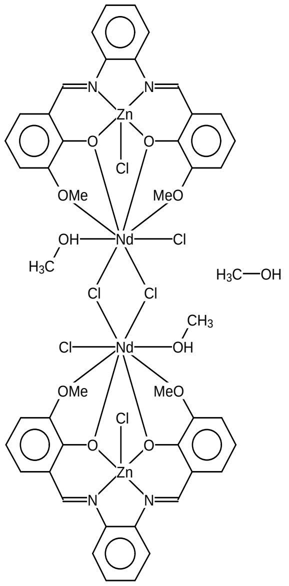

DEJLEK

Reference: Wai-Kwok Wong, Xiaoping Yang, R.A.Jones, J.H.Rivers, V.Lynch, Wing-Kit Lo, Dang Xiao, M.M.Oye, A.L.Holmes (2006) *Inorg.Chem.* ,**45,**4340

Formula: $C_{24} H_{21} N_6 Nd_1 O_{13} Zn_1, C_2 H_3 N_1$

Compound Name: (μ_2-N,N'-bis(3-Methoxysalicylidene)-1,2-phenylenediamine-N,N',O,O,O',O',O'',O''')-(acetonitrile)-tris(nitrato-O,O')-neodymium(iii)-zinc(ii) acetonitrile solvate

Space Group:	Pna21	***Cell:***	***a*** 9.512(1)	***b*** 16.439(3)	***c*** 19.317(4)		
Space Group No.:	33	***(Å,°)***	α 90.00	β 90.00	γ 90.00		
R-Factor (%):	3.41	***Temperature(K):***	153	***Density(g/cm³):***	1.874		

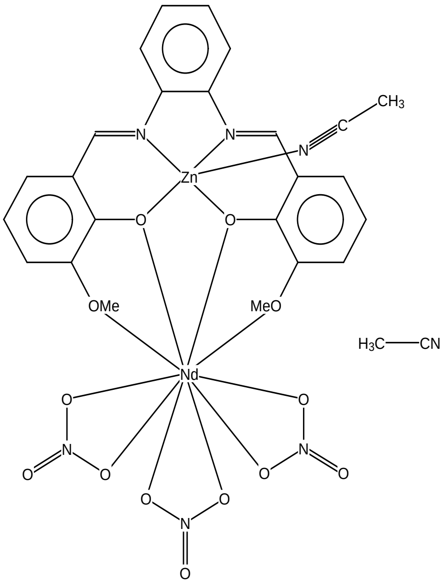

DEJLIO

Reference: Wai-Kwok Wong, Xiaoping Yang, R.A.Jones, J.H.Rivers, V.Lynch, Wing-Kit Lo, Dang Xiao, M.M.Oye, A.L.Holmes (2006) *Inorg.Chem.* ,**45,**4340

Formula: $C_{44} H_{40} N_6 Nd_1 O_{16} Zn_2{}^{1+}, N_1 O_3{}^{1-}, C_2 H_6 O_1, H_2 O_1$

Compound Name: bis(μ_2-N,N'-bis(3-Methoxysalicylidene)-1,2-phenylenediamine-N,N',O,O,O',O',O'',O''')-diaqua-(nitrato-O,O')-(nitrato-O)-neodymium(iii)-di-zinc(ii) nitrate ethanol solvate monohydrate

Space Group:	P21/c	***Cell:***	***a*** 14.081(0)	***b*** 13.999(0)	***c*** 26.215(1)		
Space Group No.:	14	***(Å,°)***	α 90.00	β 100.28(0)	γ 90.00		
R-Factor (%):	3.67	***Temperature(K):***	293	***Density(g/cm³):***	1.711		

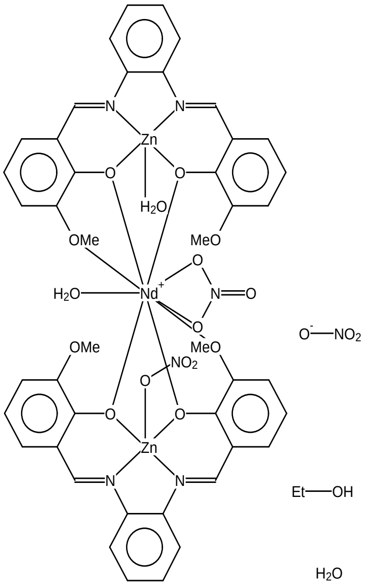

Search: search26 (Tue Dec 2 15:48:50 2014): Hits 133-136

DELDIJ

Reference: Quan Li, Pengfei Yan, Peng Chen, Guangfeng Hou, Guangming Li (2012) *J.Inorg.Organomet.Polym.Mater.* ,**22,**1174

Formula: $C_{62}\ H_{50}\ N_9\ O_{17}\ Tb_3,C_1\ H_2\ Cl_2,C_1\ H_4\ O_1,2(H_2\ O_1)$

Compound Name: bis(μ_2-2,2'-(1,2-Phenylenebis(nitrilomethylylidene))diphenolato)-bis(methanol)-tris(nitrato-O,O')-(2,2'-(1,2-phenylenebis(nitrilomethylylidene))diphenolato)-tri-terbium(iii) dichloromethane methanol solvate dihydrate

Space Group:	P-1	***Cell:***	***a*** 13.831(3)	***b*** 15.266(3)	***c*** 19.322(4)		
Space Group No.:	2	***(Å,°)***	α 85.79(3)	β 71.61(3)	γ 73.49(3)		
R-Factor (%):	5.27	***Temperature(K):***	293	***Density(g/cm³):***	1.631		

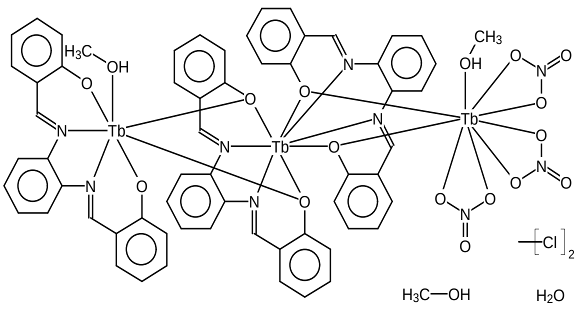

DELDOP

Reference: Quan Li, Pengfei Yan, Peng Chen, Guangfeng Hou, Guangming Li (2012) *J.Inorg.Organomet.Polym.Mater.* ,**22,**1174

Formula: $C_{62}\ H_{50}\ Ho_3\ N_9\ O_{17},C_1\ H_2\ Cl_2,C_1\ H_4\ O_1,2(H_2\ O_1)$

Compound Name: bis(μ_2-2,2'-(1,2-Phenylenebis(nitrilomethylylidene))diphenolato)-bis(methanol)-tris(nitrato-O,O')-(2,2'-(1,2-phenylenebis(nitrilomethylylidene))diphenolato)-tri-holmium(iii) dichloromethane methanol solvate dihydrate

Space Group:	P-1	***Cell:***	***a*** 13.797(3)	***b*** 15.123(3)	***c*** 19.267(4)		
Space Group No.:	2	***(Å,°)***	α 85.74(3)	β 71.66(3)	γ 73.42(3)		
R-Factor (%):	4.81	***Temperature(K):***	293	***Density(g/cm³):***	1.672		

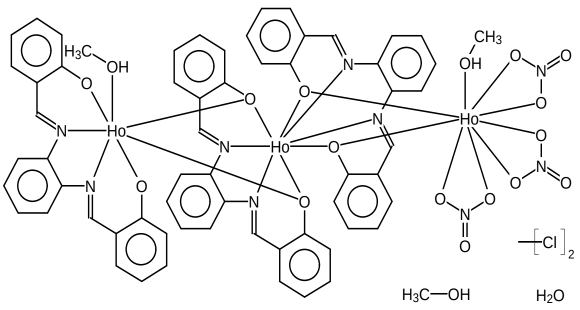

DELDUV

Reference: Quan Li, Pengfei Yan, Peng Chen, Guangfeng Hou, Guangming Li (2012) *J.Inorg.Organomet.Polym.Mater.* ,**22,**1174

Formula: $C_{62}\ H_{50}\ Er_3\ N_9\ O_{17},C_1\ H_2\ Cl_2,C_1\ H_4\ O_1,2(H_2\ O_1)$

Compound Name: bis(μ_2-2,2'-(1,2-Phenylenebis(nitrilomethylylidene))diphenolato)-bis(methanol)-tris(nitrato-O,O')-(2,2'-(1,2-phenylenebis(nitrilomethylylidene))diphenolato)-tri-erbium(iii) dichloromethane methanol solvate dihydrate

Space Group:	P-1	***Cell:***	***a*** 13.779(3)	***b*** 15.118(3)	***c*** 19.220(4)		
Space Group No.:	2	***(Å,°)***	α 85.94(3)	β 71.70(3)	γ 73.42(3)		
R-Factor (%):	4.68	***Temperature(K):***	293	***Density(g/cm³):***	1.685		

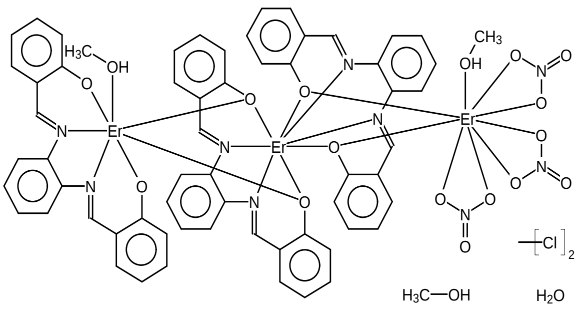

DELFAD

Reference: Quan Li, Pengfei Yan, Peng Chen, Guangfeng Hou, Guangming Li (2012) *J.Inorg.Organomet.Polym.Mater.* ,**22,**1174

Formula: $C_{62}\ H_{50}\ Lu_3\ N_9\ O_{17},C_1\ H_2\ Cl_2,C_1\ H_4\ O_1,2(H_2\ O_1)$

Compound Name: bis(μ_2-2,2'-(1,2-Phenylenebis(nitrilomethylylidene))diphenolato)-bis(methanol)-tris(nitrato-O,O')-(2,2'-(1,2-phenylenebis(nitrilomethylylidene))diphenolato)-tri-lutetium(iii) dichloromethane methanol solvate dihydrate

Space Group:	P-1	***Cell:***	***a*** 13.698(3)	***b*** 14.958(3)	***c*** 19.034(4)		
Space Group No.:	2	***(Å,°)***	α 85.66(3)	β 71.66(3)	γ 73.23(3)		
R-Factor (%):	4.13	***Temperature(K):***	293	***Density(g/cm³):***	1.753		

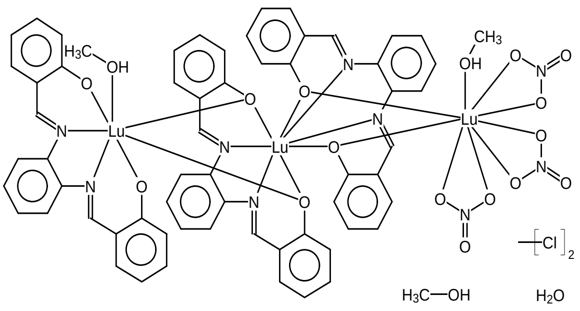

Search: search26 (Tue Dec 2 15:48:50 2014): Hits 137-140

DELFEH

Reference: Quan Li, Pengfei Yan, Peng Chen, Guangfeng Hou, Guangming Li (2012) *J.Inorg.Organomet.Polym.Mater.* ,**22,**1174

Formula: $C_{61}\ H_{46}\ N_6\ Nd_2\ O_7$

Compound Name: (μ_2-2,2'-(1,2-Phenylenebis(nitrilomethylylidene))diphenolato)-methanol-bis(2,2'-(1,2-phenylenebis(nitrilomethylylidene))diphenolato)-di-neodymium(iii)

Space Group:	P-1	***Cell:***	***a*** 14.254(3)	***b*** 16.233(3)	***c*** 24.374(5)
Space Group No.:	2	***(Å,°)***	α 89.94(3)	β 81.21(3)	γ 76.88(3)

R-Factor (%): 8.75 ***Temperature(K):*** 293 ***Density(g/cm³):*** 1.547

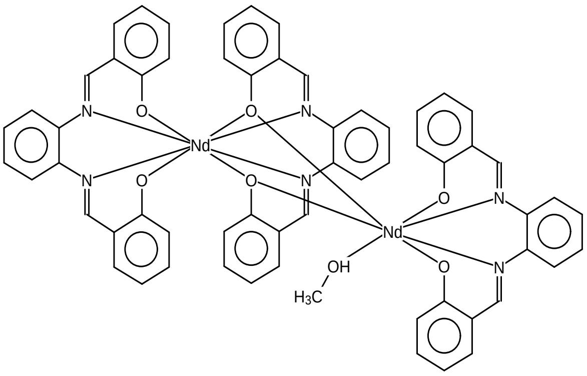

DEYGUK

Reference: K.Takao, Y.Ikeda (2007) *Inorg.Chem.* ,**46,**1550

Formula: $C_{40}\ H_{28}\ N_4\ O_8\ U_2$

Compound Name: bis(μ_2-N,N'-Disalicylidene-o-phenylenediamine-N,N',O,O,O')-tetraoxo-di-uranium(vi)

Space Group:	P-1	***Cell:***	***a*** 15.689(7)	***b*** 16.044(5)	***c*** 17.642(7)
Space Group No.:	2	***(Å,°)***	α 67.00(3)	β 78.25(3)	γ 81.72(3)

R-Factor (%): 5.78 ***Temperature(K):*** 93 ***Density(g/cm³):*** 1.945

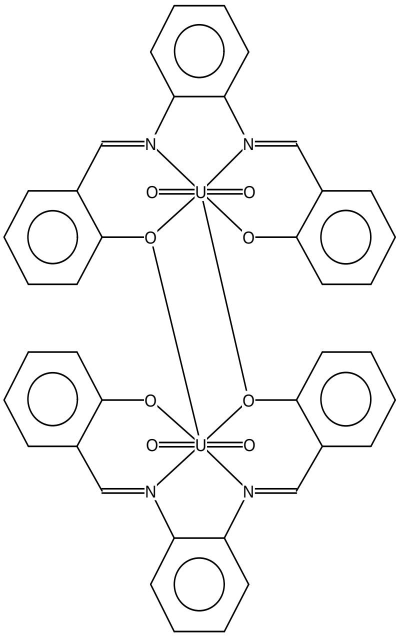

DIDKIK

Reference: L.G.Marzilli, M.F.Summers, N.Bresciani-Pahor, E.Zangrando, J.-P.Charland, L.Randaccio (1985) *J.Am.Chem.Soc.* , **107,**6880

Formula: $C_{23}\ H_{21}\ Co_1\ N_2\ O_2,1.5(H_2\ O_1)$

Compound Name: (Disalicylidene-o-phenylenediamine)-isopropyl-cobalt sesquihydrate

Space Group:	Pbcn	***Cell:***	***a*** 26.075(6)	***b*** 9.617(3)	***c*** 16.109(3)
Space Group No.:	60	***(Å,°)***	α 90.00	β 90.00	γ 90.00

R-Factor (%): 5.70 ***Temperature(K):*** 295 ***Density(g/cm³):*** 1.458

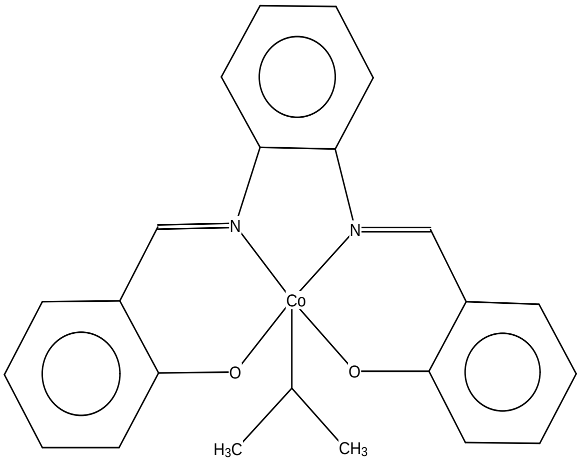

H_2O

DIDKOQ

Reference: L.G.Marzilli, M.F.Summers, N.Bresciani-Pahor, E.Zangrando, J.-P.Charland, L.Randaccio (1985) *J.Am.Chem.Soc.* , **107,**6880

Formula: $C_{21}\ H_{17}\ Co_1\ N_2\ O_2,0.5(H_2\ O_1)$

Compound Name: (Disalicylidene-o-phenylenediamine)-methyl-cobalt hemihydrate

Space Group:	P-1	***Cell:***	***a*** 12.100(8)	***b*** 20.392(9)	***c*** 11.606(8)
Space Group No.:	2	***(Å,°)***	α 53.30(10)	β 127.60(10)	γ 121.70(10)

R-Factor (%): 4.10 ***Temperature(K):*** 295 ***Density(g/cm³):*** 1.500

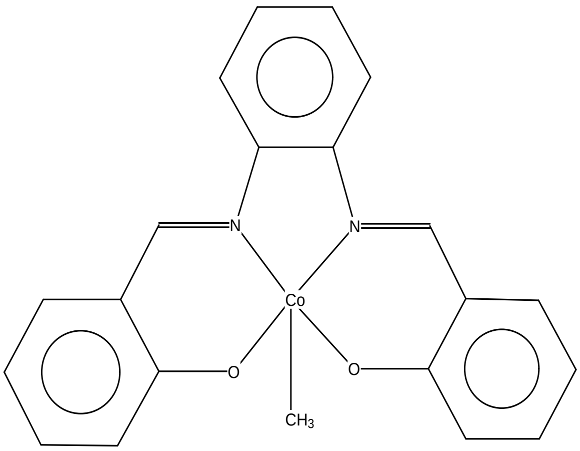

H_2O

Search: search26 (Tue Dec 2 15:48:50 2014): Hits 141-144

DIDKUW

Reference: L.G.Marzilli, M.F.Summers, N.Bresciani-Pahor, E.Zangrando, J.-P.Charland, L.Randaccio (1985) *J.Am.Chem.Soc.* , **107,**6880

Formula: C_{27} H_{21} Co_1 F_3 N_3 O_2,0.5(H_2 O_1),0.5(C_5 H_5 N_1)

Compound Name: (Disalicylidene-o-phenylenediamine)-pyridine-(2,2,2-trifluoroethyl)-cobalt hemihydrate pyridine solvate

Space Group:	P21/c	***Cell:***	***a*** 14.255(8)	***b*** 19.318(8)	***c*** 19.434(8)		
Space Group No.:	14	***(Å,°)***	α 90.00	β 93.70(10)	γ 90.00		
R-Factor (%):	5.50	***Temperature(K):***	295	***Density(g/cm^3):***	1.453		

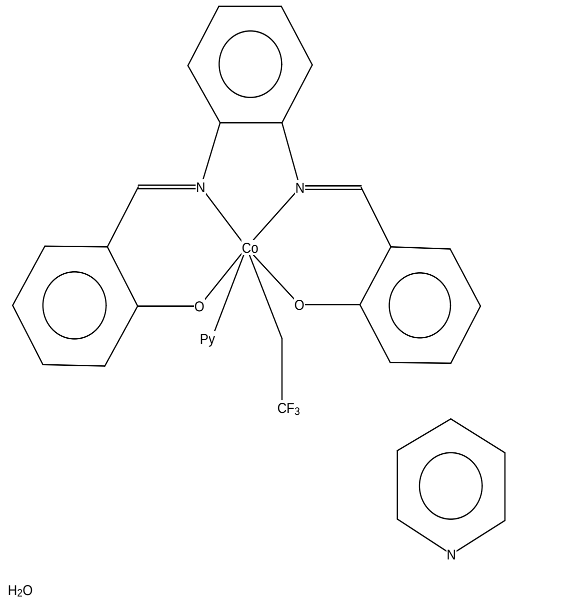

DOXCUO

Reference: M.Mazzanti, S.Gambarotta, C.Floriani, A.Chiesi-Villa, C.Guastini (1986) *Inorg.Chem.* ,**25,**2308

Formula: C_{24} H_{22} Cl_1 N_2 O_3 V_1,C_4 H_8 O_1

Compound Name: Chloro-(phenylene-bis(salicyladiminato)-N,N')-(tetrahydrofuran-O)-vanadium(iii) tetrahydrofuran solvate

Space Group:	P21/c	***Cell:***	***a*** 16.179(4)	***b*** 12.038(3)	***c*** 14.506(4)		
Space Group No.:	14	***(Å,°)***	α 90.00	β 111.56(3)	γ 90.00		
R-Factor (%):	6.10	***Temperature(K):***	295	***Density(g/cm^3):***	1.378		

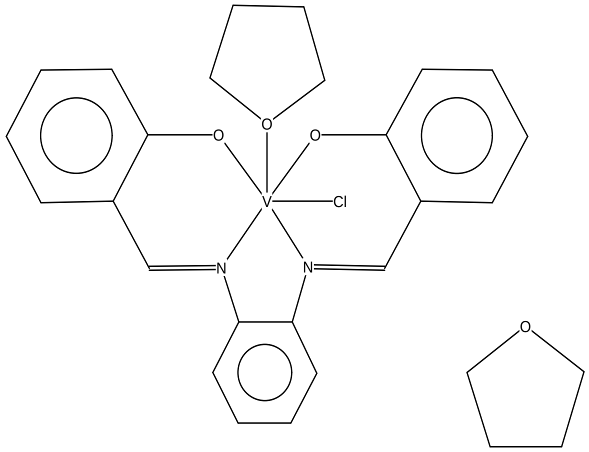

DOYSUF

Reference: T.Panther, U.Behrens (2000) *Z.Anorg.Allg.Chem.* ,**626,** 1934

Formula: C_{39} H_{50} Co_1 N_6 Ni_1 O_4 $^{2+}$,2(Cl_1 O_4 $^{1-}$),C_3 H_7 N_1 O_1

Compound Name: (3,7,15,22-Tetra-aza-11,26-di-t-butyl-28,29-dioxatetracyclo(22.3.1^{1,24}.1^{9,13}.0^{16,21})nonacosa-1(28),2,7,9,11,13(29),14,16,18,20,22,24,26-tridecene)-bis(dimethylformamide)-cobalt-nickel diperchlorate dimethylformamide solvate

Space Group:	P-1	***Cell:***	***a*** 9.770(1)	***b*** 21.574(11)	***c*** 22.656(5)		
Space Group No.:	2	***(Å,°)***	α 89.68(3)	β 88.92(2)	γ 85.04(2)		
R-Factor (%):	4.80	***Temperature(K):***	153	***Density(g/cm^3):***	1.475		

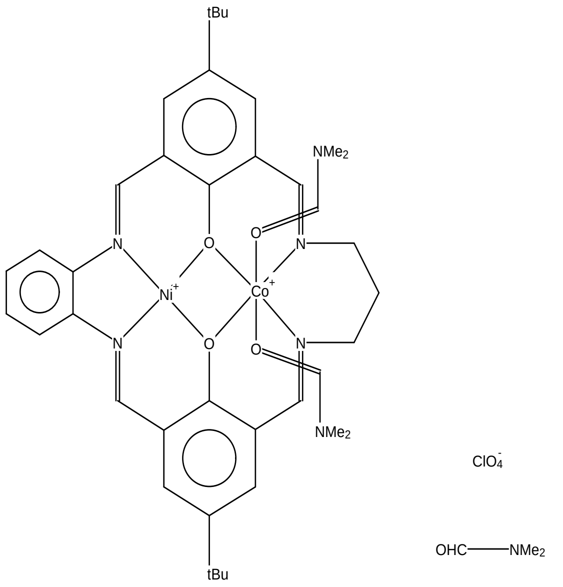

DOYTAM

Reference: T.Panther, U.Behrens (2000) *Z.Anorg.Allg.Chem.* ,**626,** 1934

Formula: C_{33} H_{36} Cl_2 Cu_1 N_4 Ni_1 O_{10},C_3 H_7 N_1 O_1

Compound Name: (3,7,15,22-Tetra-aza-11,26-di-t-butyl-28,29-dioxatetracyclo(22.3.1^{1,24}.1^{9,13}.0^{16,21})nonacosa-1(28),2,7,9,11,13(29),14,16,18,20,22,24,26-tridecene)-bis(perchlorato-O)-copper-nickel dimethylformamide solvate

Space Group:	P21/n	***Cell:***	***a*** 15.448(2)	***b*** 16.670(2)	***c*** 15.626(2)		
Space Group No.:	14	***(Å,°)***	α 90.00	β 100.05(1)	γ 90.00		
R-Factor (%):	4.25	***Temperature(K):***	295	***Density(g/cm^3):***	1.534		

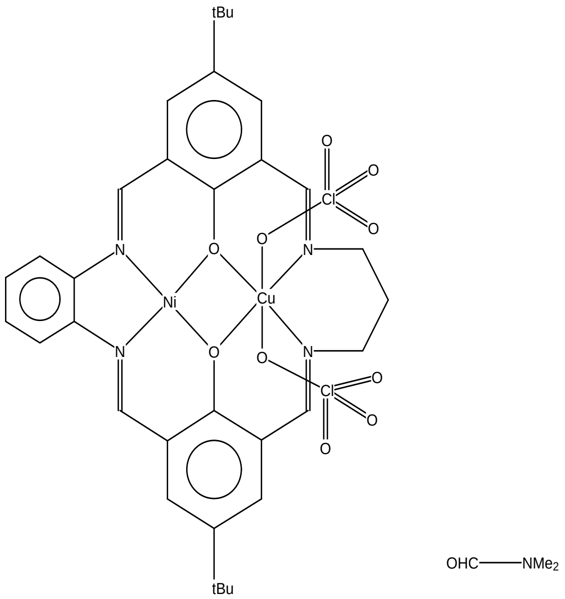

Search: search26 (Tue Dec 2 15:48:50 2014): Hits 145-148

DOYTEQ

Reference: T.Panther, U.Behrens (2000) *Z.Anorg.Allg.Chem.* ,**626,** 1934

Formula: $C_{33}\,H_{36}\,Cl_2\,Cu_2\,N_4\,O_{10}$,$C_3\,H_7\,N_1\,O_1$

Compound Name: (μ_2-3,7,15,22-Tetra-aza-11,26-di-t-butyl-28,29-dioxatetracyclo(22.3.1^{1,24}.1^{9,13}.0^{16,21})nonacosa-1(28),2,7,9,11,13(29),14,16,18,20,22,24,26-tridecene)-(μ_2-perchlorato-O,O')-(perchlorato-O)-di-copper dimethylformamide solvate

Space Group: P21/n ***Cell:*** ***a*** 15.462(4) ***b*** 16.445(5) ***c*** 15.439(4)
Space Group No.: 14 ***(Å,°)*** α 90.00 β 101.85(2) γ 90.00

R-Factor (%): 5.41 ***Temperature(K):*** 173 ***Density(g/cm^3):*** 1.590

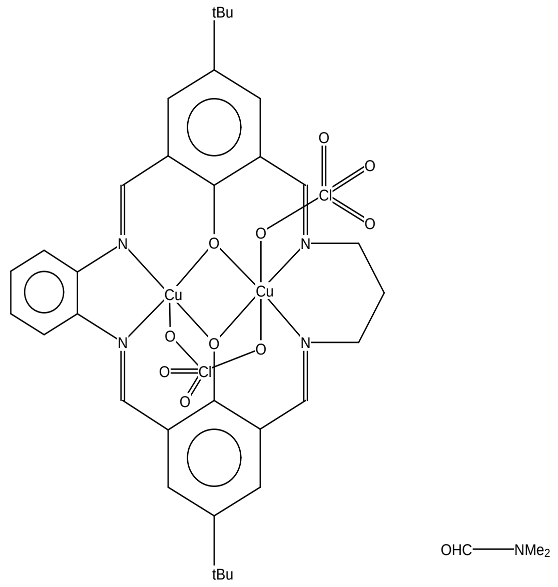

DOYTIU

Reference: T.Panther, U.Behrens (2000) *Z.Anorg.Allg.Chem.* ,**626,** 1934

Formula: $C_{33}\,H_{36}\,Cl_2\,Cu_1\,N_4\,O_{10}\,Zn_1$,$C_1\,H_2\,Cl_2$

Compound Name: (μ_2-Perchlorato-O,O')-(μ_2-3,7,15,22-tetra-aza-11,26-di-t-butyl-28,29-dioxatetracyclo(22.3.1^{1,24}.1^{9,13}.0^{16,21})nonacosa-1(28),2,7,9,11,13(29),14,16,18,20,22,24,26-tridecene)-(perchlorato-O)-copper-zinc dichloromethane solvate

Space Group: P21/n ***Cell:*** ***a*** 15.233(2) ***b*** 16.324(4) ***c*** 15.444(3)
Space Group No.: 14 ***(Å,°)*** α 90.00 β 100.74(1) γ 90.00

R-Factor (%): 6.54 ***Temperature(K):*** 173 ***Density(g/cm^3):*** 1.643

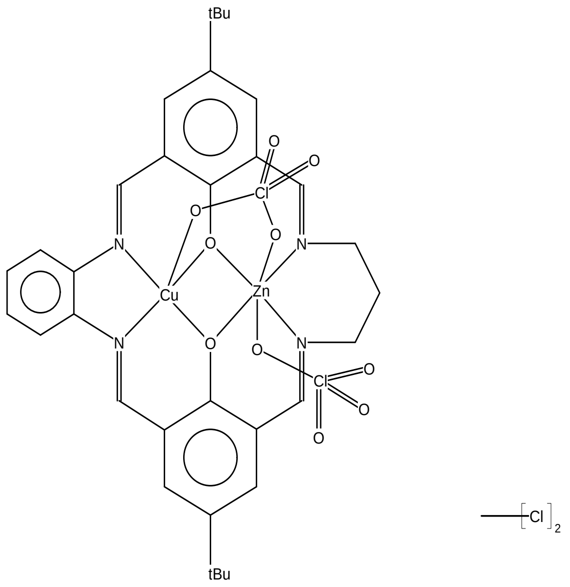

DOYTOA

Reference: T.Panther, U.Behrens (2000) *Z.Anorg.Allg.Chem.* ,**626,** 1934

Formula: $C_{33}\,H_{36}\,Cl_2\,Cu_1\,N_4\,O_{10}\,Pd_1$,$C_4\,H_{10}\,O_1$

Compound Name: (μ_2-3,7,15,22-Tetra-aza-11,26-di-t-butyl-28,29-dioxatetracyclo(22.3.1^{1,24}.1^{9,13}.0^{16,21})nonacosa-1(28),2,7,9,11,13(29),14,16,18,20,22,24,26-tridecene)-bis(perchlorato-O)-copper-palladium diethyl ether solvate

Space Group: P21/n ***Cell:*** ***a*** 15.118(4) ***b*** 16.538(3) ***c*** 15.866(9)
Space Group No.: 14 ***(Å,°)*** α 90.00 β 98.06(3) γ 90.00

R-Factor (%): 5.49 ***Temperature(K):*** 173 ***Density(g/cm^3):*** 1.630

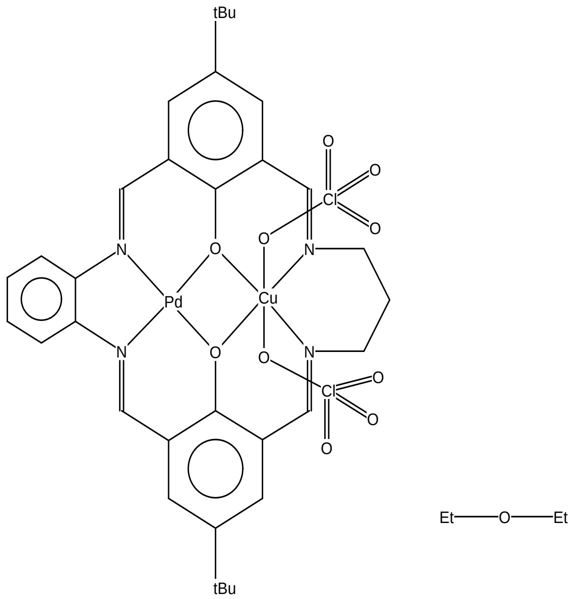

DOZPAK

Reference: Xianghong Wu, M.S.Bharara, T.H.Bray, B.K.Tate, A.E.V.Gorden (2009) *Inorg.Chim.Acta* ,**362,**1847

Formula: $C_{32}\,H_{27}\,N_5\,O_6\,U_1$

Compound Name: (2-Benzyl-3-hydroxy-6,7-bis(2-oxybenzylideneamino)quinoxaline)-dioxo-(dimethylformamide)-uranium

Space Group: C2/c ***Cell:*** ***a*** 33.449(1) ***b*** 7.404(0) ***c*** 28.564(1)
Space Group No.: 15 ***(Å,°)*** α 90.00 β 124.20(0) γ 90.00

R-Factor (%): 4.35 ***Temperature(K):*** 193 ***Density(g/cm^3):*** 1.852

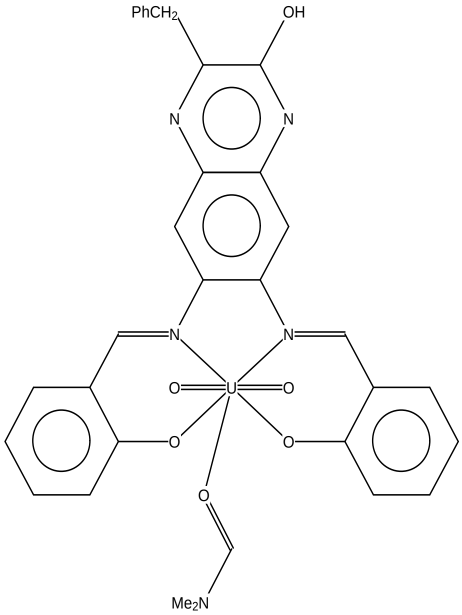

DOZPEO

Reference: Xianghong Wu, M.S.Bharara, T.H.Bray, B.K.Tate, A.E.V.Gorden (2009) *Inorg.Chim.Acta* ,**362,**1847

Formula: $C_{45} H_{54} N_4 O_6 U_1$,3($C_3 H_6 O_1$),0.5($H_2 O_1$)

Compound Name: (2-Benzyl-3-hydroxy-6,7-bis(3,5-di-t-butyl-2-oxybenzylideneamino) quinoxaline)-aqua-dioxo-uranium acetone solvate hemihydrate

Space Group:	P21/n	***Cell:***	***a***	21.084(1)	***b***	23.115(1)	***c***	23.334(1)
Space Group No.:	14	***(Å,°)***	α	90.00	β	107.46(0)	γ	90.00

R-Factor (%): 3.33 ***Temperature(K):*** 293 ***Density(g/cm³):*** 1.431

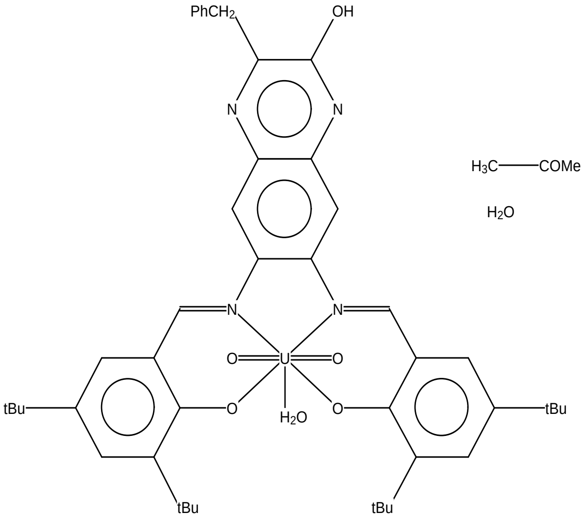

DOZPOY

Reference: Xianghong Wu, M.S.Bharara, T.H.Bray, B.K.Tate, A.E.V.Gorden (2009) *Inorg.Chim.Acta* ,**362,**1847

Formula: $C_{28} H_{27} N_5 O_6 U_1$

Compound Name: (2-Isopropyl-3-hydroxy-6,7-bis(2-oxybenzylideneamino)quinoxaline)-dioxo-(dimethylformamide)-uranium

Space Group:	C2/c	***Cell:***	***a***	26.074(1)	***b***	7.291(0)	***c***	28.867(1)
Space Group No.:	15	***(Å,°)***	α	90.00	β	99.57(0)	γ	90.00

R-Factor (%): 3.90 ***Temperature(K):*** 293 ***Density(g/cm³):*** 1.884

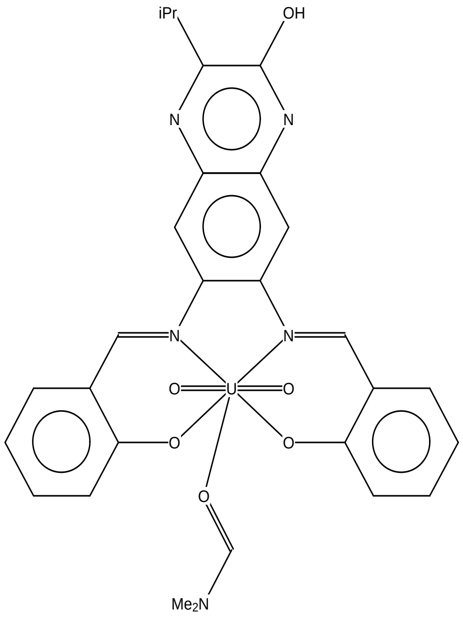

DOZPUE

Reference: Xianghong Wu, M.S.Bharara, T.H.Bray, B.K.Tate, A.E.V.Gorden (2009) *Inorg.Chim.Acta* ,**362,**1847

Formula: $C_{32} H_{27} N_5 O_8 U_1$,0.25($C_3 H_7 N_1 O_1$)

Compound Name: (2-Benzyl-3-hydroxy-6,7-bis(3-hydroxy-2-oxybenzylideneamino) quinoxaline)-dioxo-(dimethylformamide)-uranium dimethylformamide solvate

Space Group:	P21/n	***Cell:***	***a***	16.403(1)	***b***	7.196(0)	***c***	28.974(3)
Space Group No.:	14	***(Å,°)***	α	90.00	β	93.97(0)	γ	90.00

R-Factor (%): 7.46 ***Temperature(K):*** 293 ***Density(g/cm³):*** 1.686

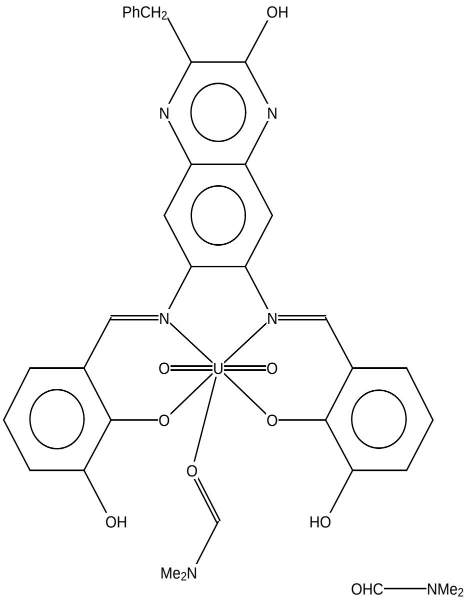

DUCJER

Reference: Hailong Wang, Daopeng Zhang, Zhong-Hai Ni, Xiyou Li, Laijin Tian, Jianzhuang Jiang (2009) *Inorg.Chem.* ,**48,**5946

Formula: $C_{44} H_{36} Cd_1 Cl_2 N_4 O_8 Zn_2$,$H_2 O_1$

Compound Name: bis(μ_2-N,N'-bis(3-Methoxysalicylidene)benzene-1,2-diamine)-dichloro-cadmium(ii)-di-zinc(ii) monohydrate

Space Group:	P21/c	***Cell:***	***a***	13.148(3)	***b***	25.385(6)	***c***	16.826(4)
Space Group No.:	14	***(Å,°)***	α	90.00	β	110.16(0)	γ	90.00

R-Factor (%): 10.01 ***Temperature(K):*** 293 ***Density(g/cm³):*** 1.362

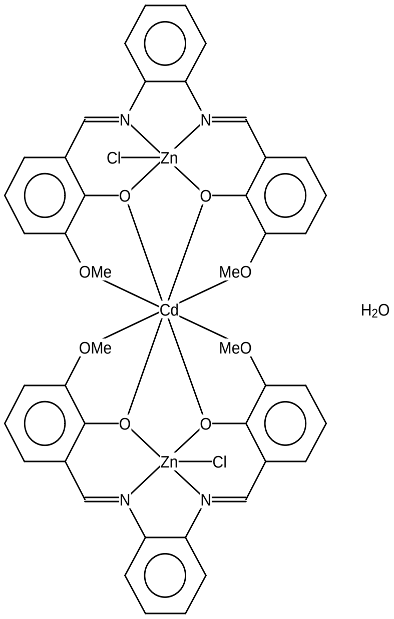

DUCJOB

Reference: Hailong Wang, Daopeng Zhang, Zhong-Hai Ni, Xiyou Li, Laijin Tian, Jianzhuang Jiang (2009) *Inorg.Chem.* ,**48,**5946

Formula: $C_{44}\ H_{36}\ Cl_2\ N_4\ O_8\ Pb_1\ Zn_2,2(H_2\ O_1)$

Compound Name: bis(μ_2-N,N'-bis(3-Methoxysalicylidene)benzene-1,2-diamine)-dichloro-lead(ii)-di-zinc(ii) dihydrate

Space Group:	P21/c	***Cell:***	***a***	13.265(15)	***b***	25.830(30)	***c***	17.310(20)
Space Group No.:	14	***(Å,°)***	α	90.00	β	112.04(2)	γ	90.00

R-Factor (%): 9.90 ***Temperature(K):*** 293 ***Density(g/cm^3):*** 1.442

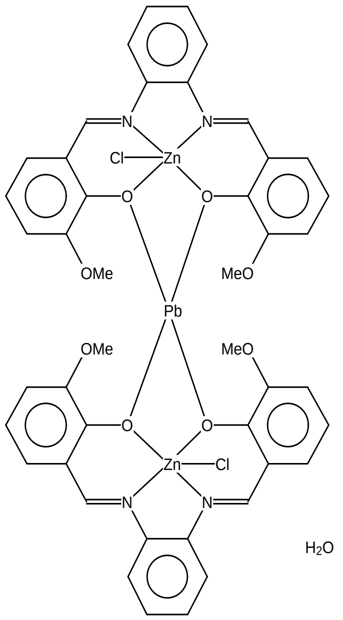

DUCJOB01

Reference: Hailong Wang, Daopeng Zhang, Zhong-Hai Ni, Xiyou Li, Laijin Tian, Jianzhuang Jiang (2009) *Inorg.Chem.* ,**48,**5946

Formula: $C_{44}\ H_{36}\ Cl_2\ N_4\ O_8\ Pb_1\ Zn_2,2(H_2\ O_1)$

Compound Name: bis(μ_2-N,N'-bis(3-Methoxysalicylidene)benzene-1,2-diamine)-dichloro-lead(ii)-di-zinc(ii) dihydrate

Space Group:	C2/c	***Cell:***	***a***	22.417(3)	***b***	14.161(1)	***c***	13.383(1)
Space Group No.:	15	***(Å,°)***	α	90.00	β	99.94(0)	γ	90.00

R-Factor (%): 1.71 ***Temperature(K):*** 293 ***Density(g/cm^3):*** 1.895

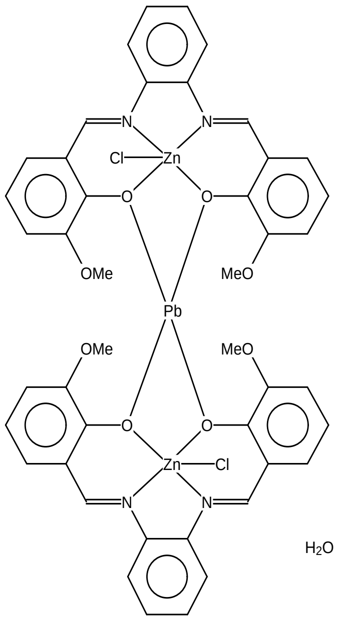

DUCKAO

Reference: Hailong Wang, Daopeng Zhang, Zhong-Hai Ni, Xiyou Li, Laijin Tian, Jianzhuang Jiang (2009) *Inorg.Chem.* ,**48,**5946

Formula: $C_{50}\ H_{50}\ Cl_2\ Gd_1\ N_6\ O_{10}\ Zn_2\ ^{1+},Cl_1\ ^{1-},2(H_2\ O_1)$

Compound Name: bis(μ_2-N,N'-bis(3-Methoxysalicylidene)benzene-1,2-diamine)-dichloro-bis(N,N-dimethylformamide)-gadolinium(iii)-di-zinc(ii) chloride dihydrate

Space Group:	P21/n	***Cell:***	***a***	11.409(2)	***b***	33.103(1)	***c***	15.811(1)
Space Group No.:	14	***(Å,°)***	α	90.00	β	106.99(0)	γ	90.00

R-Factor (%): 8.21 ***Temperature(K):*** 295 ***Density(g/cm^3):*** 1.541

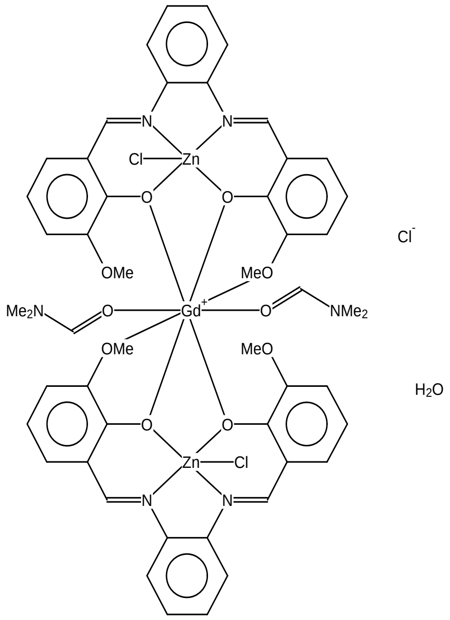

DUCKOC

Reference: Hailong Wang, Daopeng Zhang, Zhong-Hai Ni, Xiyou Li, Laijin Tian, Jianzhuang Jiang (2009) *Inorg.Chem.* ,**48,**5946

Formula: $C_{49}\ H_{46}\ Cl_2\ Eu_1\ N_5\ O_{11}\ Zn_2,C_2\ H_3\ N_1$

Compound Name: bis(μ_2-N,N'-bis(3-Methoxysalicylidene)benzene-1,2-diamine)-dichloro-(acetato)-(N,N-dimethylformamide)-europium(iii)-di-zinc(ii) acetonitrile solvate

Space Group:	P-1	***Cell:***	***a***	11.251(0)	***b***	12.603(0)	***c***	19.424(1)
Space Group No.:	2	***(Å,°)***	α	81.65(0)	β	80.53(0)	γ	68.83(0)

R-Factor (%): 2.98 ***Temperature(K):*** 298 ***Density(g/cm^3):*** 1.680

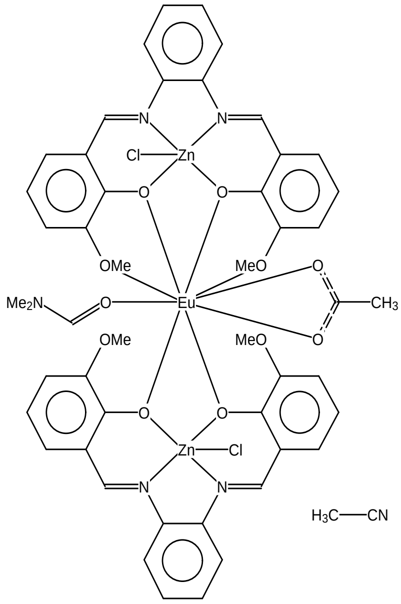

Search: search26 (Tue Dec 2 15:48:50 2014): Hits 157-160

DUCKUI

Reference: Hailong Wang, Daopeng Zhang, Zhong-Hai Ni, Xiyou Li, Laijin Tian, Jianzhuang Jiang (2009) *Inorg.Chem.* ,**48,**5946

Formula: $C_{49} H_{46} Cl_2 N_5 Nd_1 O_{11} Zn_2,C_2 H_3 N_1$

Compound Name: bis(μ_2-N,N'-bis(3-Methoxysalicylidene)benzene-1,2-diamine)-dichloro-(acetato)-(N,N-dimethylformamide)-neodymium(iii)-di-zinc(ii) acetonitrile solvate

Space Group:	P-1	***Cell:***	***a*** 11.352(0)	***b*** 12.560(0)	***c*** 19.365(1)		
Space Group No.:	2	***(Å,°)***	α 81.74(0)	β 80.50(0)	γ 69.14(0)		
R-Factor (%):	2.49	***Temperature(K):***	298	***Density(g/cm³):***	1.662		

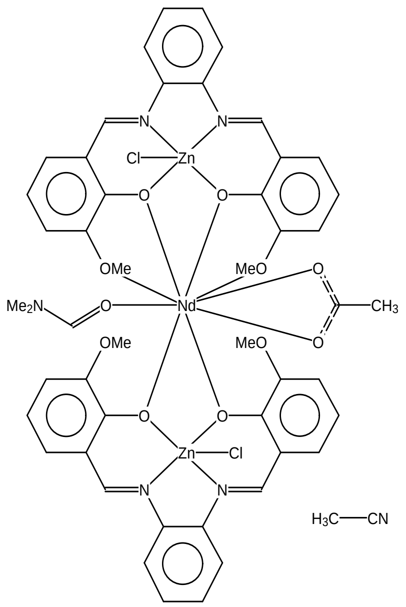

DUCLAP

Reference: Hailong Wang, Daopeng Zhang, Zhong-Hai Ni, Xiyou Li, Laijin Tian, Jianzhuang Jiang (2009) *Inorg.Chem.* ,**48,**5946

Formula: $C_{50} H_{50} Cl_2 N_6 O_{10} Tb_1 Zn_2{}^{1+},Cl_1{}^{1-},2(H_2 O_1)$

Compound Name: bis(μ_2-N,N'-bis(3-Methoxysalicylidene)benzene-1,2-diamine)-dichloro-bis(N,N-dimethylformamide)-terbium(iii)-di-zinc(ii) chloride dihydrate

Space Group:	P21/n	***Cell:***	***a*** 11.418(2)	***b*** 32.990(6)	***c*** 15.814(3)		
Space Group No.:	14	***(Å,°)***	α 90.00	β 108.08(0)	γ 90.00		
R-Factor (%):	6.41	***Temperature(K):***	298	***Density(g/cm³):***	1.556		

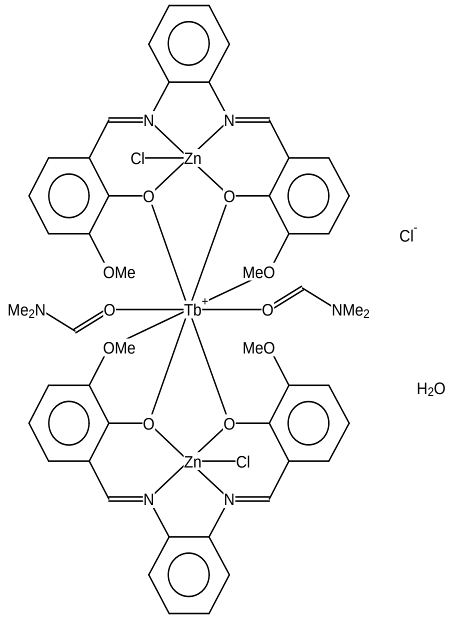

DUCLET

Reference: Hailong Wang, Daopeng Zhang, Zhong-Hai Ni, Xiyou Li, Laijin Tian, Jianzhuang Jiang (2009) *Inorg.Chem.* ,**48,**5946

Formula: $C_{50} H_{50} Cl_2 Dy_1 N_6 O_{10} Zn_2{}^{1+},Cl_1{}^{1-},2(H_2 O_1)$

Compound Name: bis(μ_2-N,N'-bis(3-Methoxysalicylidene)benzene-1,2-diamine)-dichloro-bis(N,N-dimethylformamide)-dysprosium(iii)-di-zinc(ii) chloride dihydrate

Space Group:	P21/n	***Cell:***	***a*** 11.353(2)	***b*** 33.003(5)	***c*** 15.775(3)		
Space Group No.:	14	***(Å,°)***	α 90.00	β 107.94(0)	γ 90.00		
R-Factor (%):	7.40	***Temperature(K):***	298	***Density(g/cm³):***	1.572		

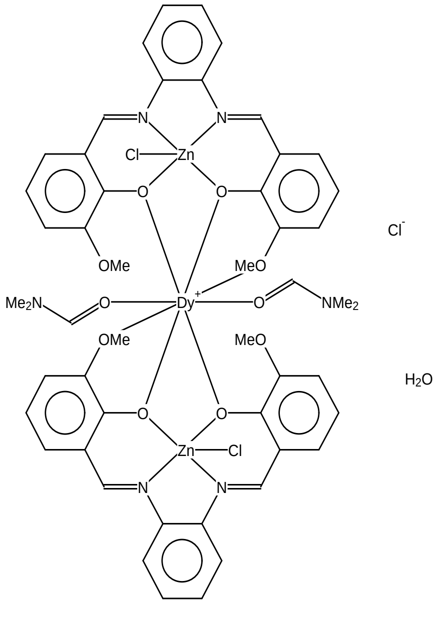

DUFKUL

Reference: A.Nabei, T.Kuroda-Sowa, T.Shimizu, T.Okubo, M.Maekawa, M.Munakata (2009) *Polyhedron* ,**28,**1734

Formula: $C_{66} H_{44} Fe_2 N_6 O_4,2(C_1 H_4 O_1)$

Compound Name: (μ_2-4,4'-Bipyridine-N,N')-bis(N,N'-bis(2-oxy-1-naphthaldehyde)-1,2-phenylenedi-imine-N,N',O,O')-di-iron(ii) methanol solvate

Space Group:	C2/c	***Cell:***	***a*** 25.393(4)	***b*** 6.038(0)	***c*** 35.301(6)		
Space Group No.:	15	***(Å,°)***	α 90.00	β 108.64(0)	γ 90.00		
R-Factor (%):	9.73	***Temperature(K):***	110	***Density(g/cm³):***	1.504		

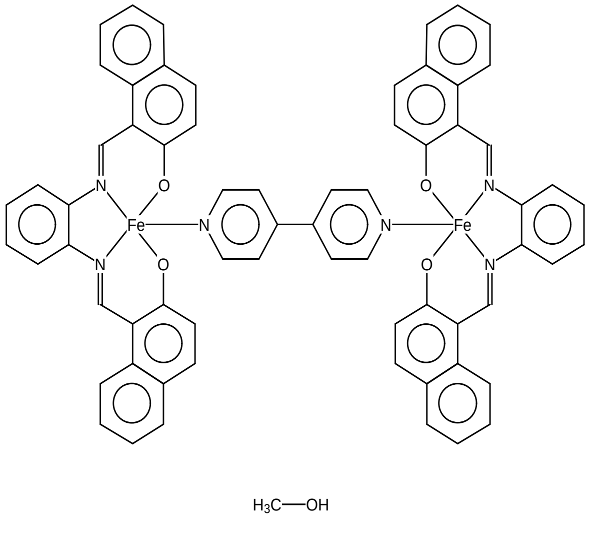

DUFLAS

Reference: A.Nabei, T.Kuroda-Sowa, T.Shimizu, T.Okubo, M.Maekawa, M.Munakata (2009) *Polyhedron* ,**28,**1734

Formula: $C_{22}\ H_{20}\ Fe_1\ N_2\ O_3$

Compound Name: Ethanol-(N,N'-bis(salicylidene)-1,2-phenylenediamine-N,N',O,O')-iron(ii)

Space Group: Pnma ***Cell:*** ***a*** 19.562(4) ***b*** 17.775(3) ***c*** 5.168(0)
Space Group No.: 62 ***(Å,°)*** α 90.00 β 90.00 γ 90.00

R-Factor (%): 7.69 ***Temperature(K):*** 150 ***Density(g/cm³):*** 1.539

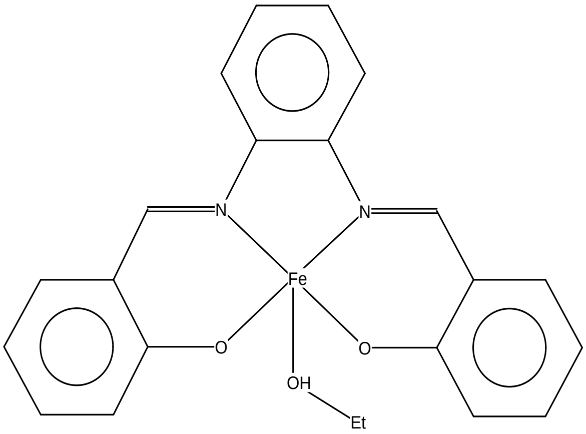

DUFLEW

Reference: A.Nabei, T.Kuroda-Sowa, T.Shimizu, T.Okubo, M.Maekawa, M.Munakata (2009) *Polyhedron* ,**28,**1734

Formula: $C_{21}\ H_{16}\ Cl_2\ Fe_1\ N_2\ O_3$

Compound Name: Methanol-(N,N'-bis(5-chlorosalicylidene)-1,2-phenylenediamine-N,N',O,O')-iron(ii)

Space Group: P-1 ***Cell:*** ***a*** 8.184(4) ***b*** 10.218(5) ***c*** 11.371(6)
Space Group No.: 2 ***(Å,°)*** α 93.35(0) β 96.64(0) γ 98.84(0)

R-Factor (%): 3.28 ***Temperature(K):*** 106 ***Density(g/cm³):*** 1.682

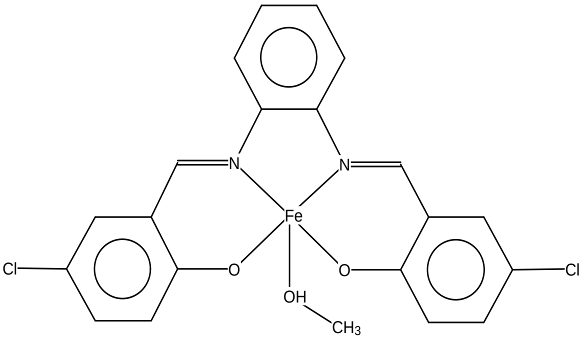

DUMKEC

Reference: Kwang Ha (2010) *Acta Crystallogr.,Sect.E:Struct.Rep.Online* ,**66,**m233

Formula: $C_{40}\ H_{24}\ Br_4\ Cl_2\ Mn_2\ N_4\ O_4,2(C_3\ H_7\ N_1\ O_1)$

Compound Name: bis(μ_2-4,4'-Dibromo-2,2'-[o-phenylenebis(nitrilomethylidyne)]diphenolato) -dichloro-di-manganese(iii) N,N-dimethylformamide solvate

Space Group: P21/c ***Cell:*** ***a*** 9.780(0) ***b*** 20.134(1) ***c*** 11.859(0)
Space Group No.: 14 ***(Å,°)*** α 90.00 β 90.94(0) γ 90.00

R-Factor (%): 4.47 ***Temperature(K):*** 200 ***Density(g/cm³):*** 1.808

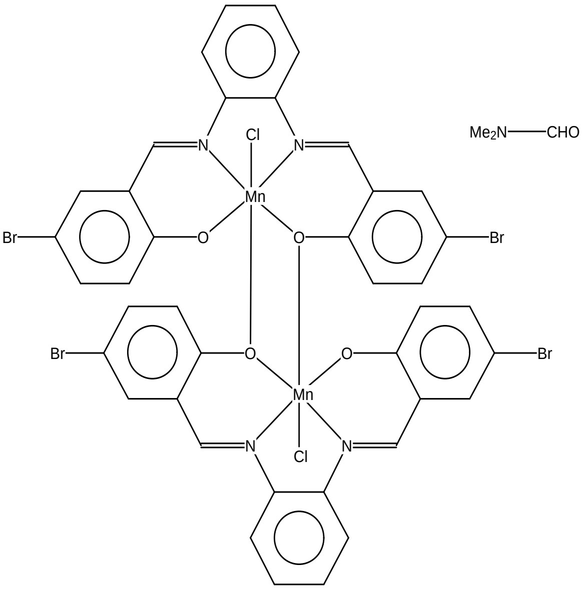

DUPVIU

Reference: Xiao-Jian Ma (2010) *Acta Crystallogr.,Sect.E:Struct.Rep.Online* ,**66,**m45

Formula: $C_{24}\ H_{24}\ Cl_1\ Cu_1\ N_2\ Na_1\ O_5,C_3\ H_7\ N_1\ O_1$

Compound Name: (μ_2-2,2'-(1,2-phenylenebis((nitrilo)methylylidene))bis(6-(ethoxy) phenolato))-aqua-chloro-sodium-copper N,N-dimethylformamide solvate

Synonym: (μ_2-6,6'-diethoxy-2,2'-[1,2-phenylenebis(nitrilomethylidyne)]diphenolato)-aqua-chlorido-copper(ii)-sodium(i) N,N-dimethylformamide solvate

Space Group: P21/n ***Cell:*** ***a*** 12.253(1) ***b*** 19.566(3) ***c*** 12.490(1)
Space Group No.: 14 ***(Å,°)*** α 90.00 β 111.65(0) γ 90.00

R-Factor (%): 3.23 ***Temperature(K):*** 298 ***Density(g/cm³):*** 1.469

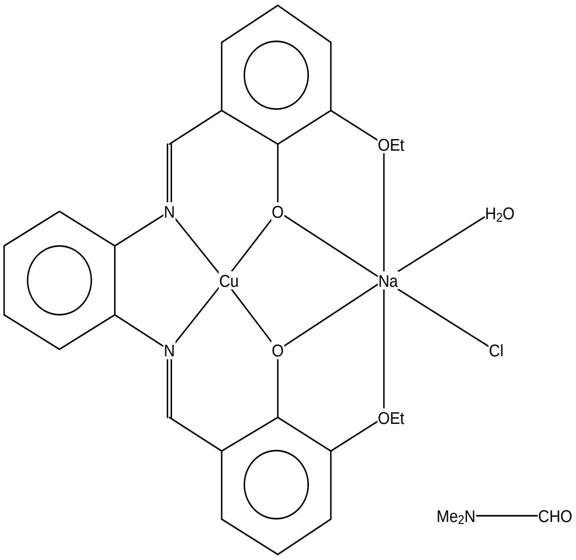

DUPWER

Reference: Cui-Juan Wang, Xian-Li Zhou, Bing-Jun Zhang (2010) *Acta Crystallogr.,Sect.E:Struct.Rep.Online* ,**66,**m50

Formula: C_{28} H_{20} Mn_1 N_5 O_3

Compound Name: Aqua-azido-(1,1'-(1,2-phenylenebis((nitrilo)methylylidene))bis(2-naphtholato))-manganese(iii)

Synonym: Aqua-azido-(3,3'-[o-phenylenebis(nitrilomethylidyne)]di-2-naphtholato)-manganese(iii)

Space Group:	P-1	***Cell:***	***a*** 6.683(0)	***b*** 11.880(0)	***c*** 15.378(0)
Space Group No.:	2	***(Å,°)***	α 99.45(0)	β 97.69(0)	γ 98.18(0)

R-Factor (%): 3.48 ***Temperature(K):*** 298 ***Density(g/cm³):*** 1.495

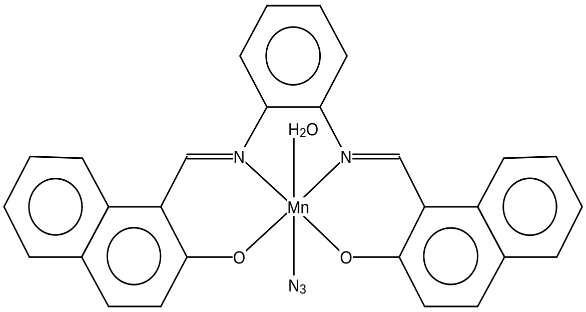

DUQBEX

Reference: M.Niu, S.Fan, K.Liu, Z.Cao, D.Wang (2010) *Acta Crystallogr.,Sect.E:Struct.Rep.Online* ,**66,**m77

Formula: C_{20} H_{14} Cu_1 N_2 O_4,2(C_1 H_4 O_1)

Compound Name: (4,4'-(1,2-Phenylenebis((nitrilo-κN)methylylidene))dibenzene-1,3-diolato-κO3)-copper(ii) methanol solvate

Synonym: (5,5'-Dihydroxy-2,2'-(o-phenylene)bis(nitrilomethylidyne)diphenolato)-copper(ii) methanol solvate disolvate

Space Group:	P-1	***Cell:***	***a*** 7.952(1)	***b*** 11.066(2)	***c*** 11.870(2)
Space Group No.:	2	***(Å,°)***	α 91.80(0)	β 94.60(0)	γ 94.24(0)

R-Factor (%): 3.66 ***Temperature(K):*** 293 ***Density(g/cm³):*** 1.517

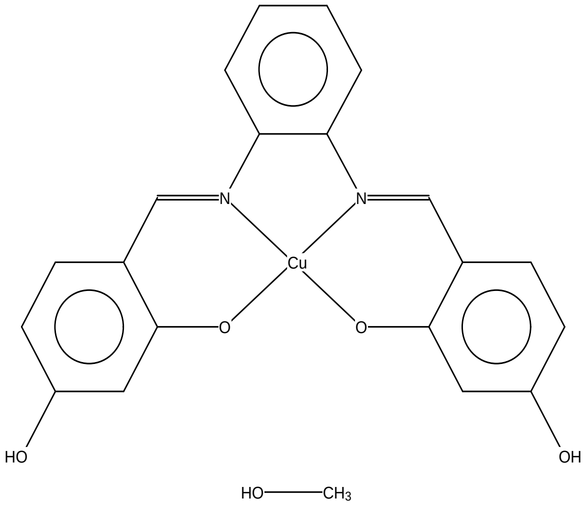

EBIZUM

Reference: Peng Chen, Han Chen, Pengfei Yan, Yan Wang, Guangming Li (2011) *CrystEngComm* ,**13,**6237

Formula: C_{48} H_{52} Cl_6 N_4 O_{12} Pr_2 Zn_2

Compound Name: bis(μ_2-chloro)-bis(μ_2-2,2'-(1,2-phenylenebis(nitrilomethylylidene))bis(6-methoxyphenolato))-tetrachloro-tetramethanol-di-praseodymium-di-zinc

Space Group:	P-1	***Cell:***	***a*** 10.393(2)	***b*** 11.270(2)	***c*** 12.065(2)
Space Group No.:	2	***(Å,°)***	α 105.91(3)	β 95.95(3)	γ 96.73(3)

R-Factor (%): 2.48 ***Temperature(K):*** 293 ***Density(g/cm³):*** 1.867

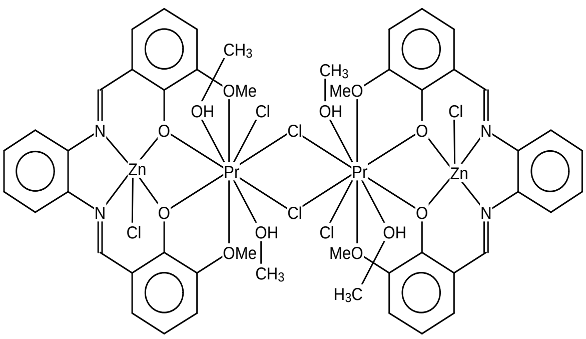

EBOBAA

Reference: Peng Chen, Han Chen, Pengfei Yan, Yan Wang, Guangming Li (2011) *CrystEngComm* ,**13,**6237

Formula: C_{25} H_{25} Cl_2 N_2 O_7 Tb_1 Zn_1

Compound Name: dichloro-acetato-methanol-(2,2'-(1,2-phenylenebis(nitrilomethylylidene))bis(6-methoxyphenolato))-zinc-terbium

Space Group:	P-1	***Cell:***	***a*** 8.011(1)	***b*** 13.379(3)	***c*** 13.674(3)
Space Group No.:	2	***(Å,°)***	α 74.93(3)	β 83.71(3)	γ 86.61(3)

R-Factor (%): 4.55 ***Temperature(K):*** 293 ***Density(g/cm³):*** 1.797

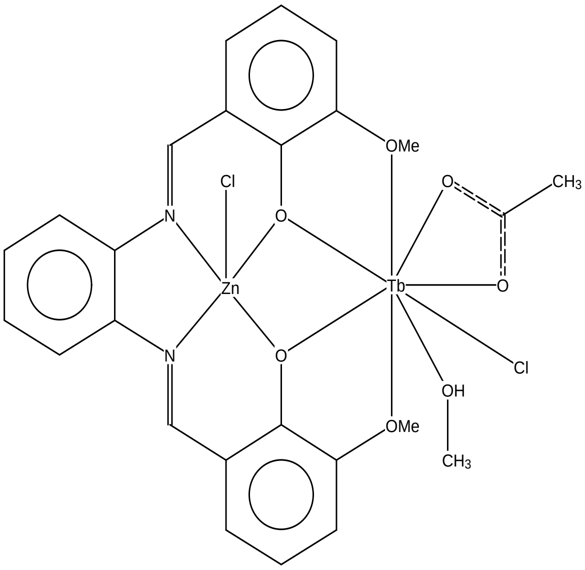

EBOBEE

Reference: Peng Chen, Han Chen, Pengfei Yan, Yan Wang, Guangming Li (2011) *CrystEngComm* ,**13,**6237

Formula: $C_{25}\ H_{25}\ Cl_2\ Dy_1\ N_2\ O_7\ Zn_1$

Compound Name: dichloro-acetato-methanol-(2,2'-(1,2-phenylenebis(nitrilomethylylidene)) bis(6-methoxyphenolato))-zinc-dysprosium

Space Group:	P-1	***Cell:***	***a***	7.990(3)	***b***	13.377(5)	***c***	13.685(5)
Space Group No.:	2	***(Å,°)***	α	74.41(0)	β	83.64(0)	γ	86.54(0)

R-Factor (%): 7.74 ***Temperature(K):*** 296 ***Density(g/cm^3):*** 1.814

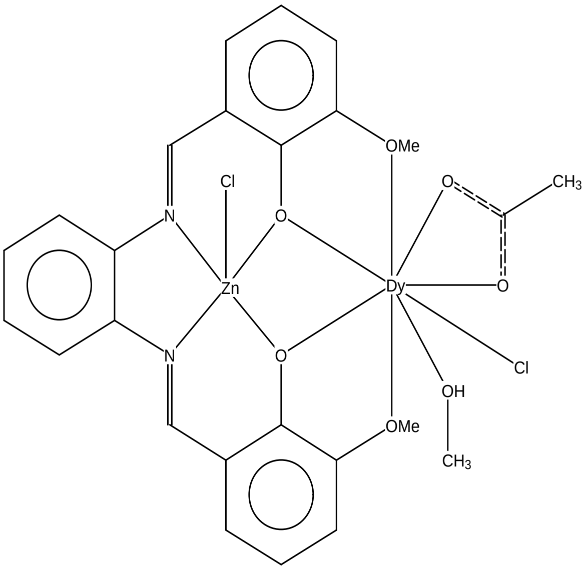

EBOBII

Reference: Peng Chen, Han Chen, Pengfei Yan, Yan Wang, Guangming Li (2011) *CrystEngComm* ,**13,**6237

Formula: $C_{52}\ H_{54}\ Lu_4\ N_4\ O_{20}\ {}^{2+},Cl_4\ Zn_1\ {}^{2-},2(H_2\ O_1)$

Compound Name: bis(μ_3-2,2'-(1,2-phenylenebis(nitrilomethylylidene))bis(6-methoxyphenol))-bis(μ_3-hydroxo)-tetrakis(μ_2-acetato)-diaqua-tetra-lutetium tetrachloro-zinc dihydrate

Space Group:	P-1	***Cell:***	***a***	11.979(6)	***b***	13.200(7)	***c***	13.260(7)
Space Group No.:	2	***(Å,°)***	α	116.24(0)	β	91.78(0)	γ	104.45(0)

R-Factor (%): 4.54 ***Temperature(K):*** 293 ***Density(g/cm^3):*** 1.846

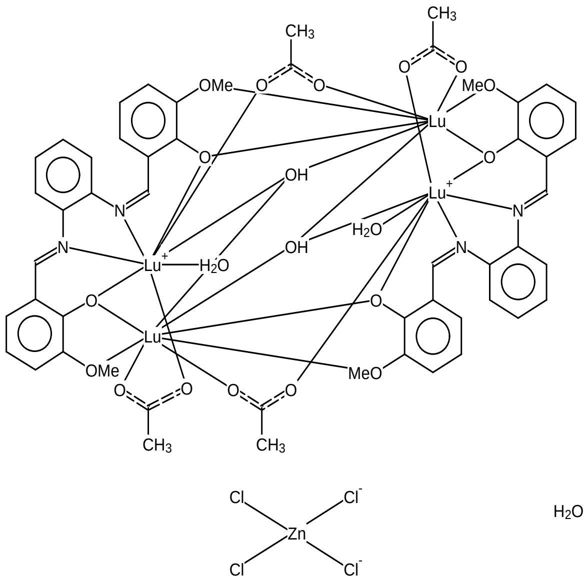

ECAGOG

Reference: Po-Heng Lin, Wen-Bin Sun, Mang-Fei Yu, Guang-Ming Li, Peng-Fei Yan, M.Murugesu (2011) *Chem.Commun.* ,**47,**10993

Formula: $C_{50}\ H_{46}\ Dy_2\ N_4\ O_9,2(C_1\ H_2\ Cl_2)$

Compound Name: Aqua-bis(pentane-2,4-dionato)-bis(μ_2-2,2'-(1,2-phenylenebis((nitrilo) methylylidene))diphenolato)-di-dysprosium dichloromethane solvate

Space Group:	P21/n	***Cell:***	***a***	15.448(4)	***b***	19.370(4)	***c***	17.834(5)
Space Group No.:	14	***(Å,°)***	α	90.00	β	91.45(1)	γ	90.00

R-Factor (%): 2.72 ***Temperature(K):*** 293 ***Density(g/cm^3):*** 1.671

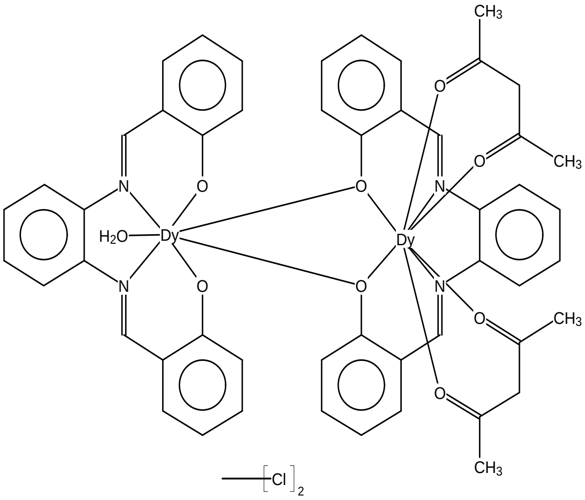

EJAGIG

Reference: A.Liao, X.Yang, J.M.Stanley, R.A.Jones, B.J.Holliday (2010) *J.Chem.Cryst.* ,**40,**1060

Formula: $C_{48}\ H_{42}\ Gd_1\ N_4\ O_{12}\ Zn_2\ {}^{1+},C_1\ F_3\ O_3\ S_1\ {}^{1-},C_4\ H_{10}\ O_1$

Compound Name: bis(μ_2-2,2'-(1,2-phenylenebis(nitrilomethylylidene))bis(6-methoxyphenolato))-bis(μ_2-acetato)-gadolinium-di-zinc trifluoromethanesulfonate diethyl ether solvate

Space Group:	P-1	***Cell:***	***a***	9.474(1)	***b***	16.333(3)	***c***	19.291(4)
Space Group No.:	2	***(Å,°)***	α	88.89(3)	β	78.16(2)	γ	76.69(3)

R-Factor (%): 4.87 ***Temperature(K):*** 223 ***Density(g/cm^3):*** 1.610

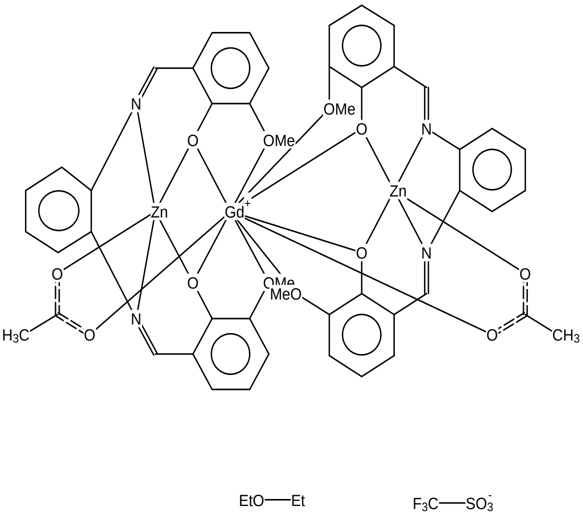

EJINAN

Reference: Peng-Fei Yan, Shuo Chen, Peng Chen, Ju-Wen Zhang, Guang-Ming Li (2011) *CrystEngComm* ,**13,**36

Formula: $C_{62} H_{50} N_9 O_{17} Yb_3, C_1 H_4 O_1, 5(H_2 O_1)$

Compound Name: bis(μ_2-2,2'-(1,2-Phenylenebis((nitrilo)methylylidene))diphenolato-N,N',O,O,O',O')-bis(methanol)-tris(nitrato-O,O')-(2,2'-(1,2-phenylenebis((nitrilo)methylylidene))diphenolato-N,N',O,O')-tri-ytterbium(iii) methanol solvate pentahydrate

Synonym: bis(μ_2-N,N'-o-Phenylene-bis(salicylideneiminato)-N,N',O,O,O',O')-bis(methanol)-tris(nitrato-O,O')-(N,N'-o-phenylene-bis(salicylideneiminato)-N,N',O,O')-tri-ytterbium(iii) methanol solvate pentahydrate

Space Group:	P-1	***Cell:***	***a*** 13.785(3)	***b*** 15.175(3)	***c*** 19.161(4)
Space Group No.:	2	***(Å,°)***	α 86.08(3)	β 71.73(3)	γ 73.39(3)

R-Factor (%): 8.97 ***Temperature(K):*** 293 ***Density(g/cm³):*** 1.671

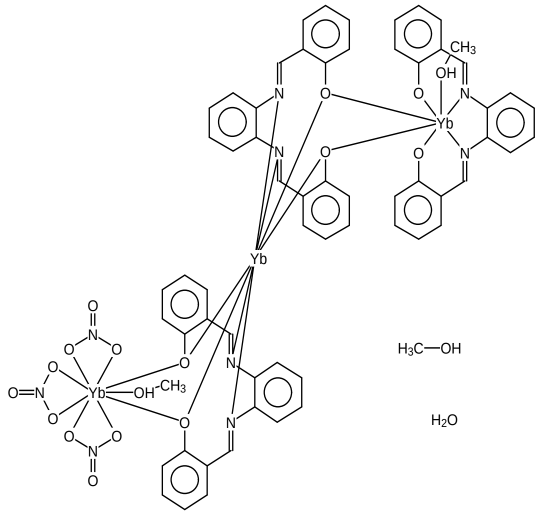

EJINER

Reference: Peng-Fei Yan, Shuo Chen, Peng Chen, Ju-Wen Zhang, Guang-Ming Li (2011) *CrystEngComm* ,**13,**36

Formula: $C_{61} H_{46} N_6 O_7 Yb_2, 0.5(H_2 O_1)$

Compound Name: (μ_2-2,2'-(1,2-Phenylenebis((nitrilo)methylylidene))diphenolato-N,N',O,O,O',O')-methanol-bis(2,2'-(1,2-phenylenebis((nitrilo)methylylidene))diphenolato-N,N',O,O')-di-ytterbium(iii) hemihydrate

Synonym: (μ_2-N,N'-o-Phenylene-bis(salicylideneiminato)-N,N',O,O,O',O')-methanol-bis(N,N'-o-phenylene-bis(salicylideneiminato)-N,N',O,O')-di-ytterbium(iii) hemihydrate

Space Group:	P21/c	***Cell:***	***a*** 14.504(3)	***b*** 23.595(7)	***c*** 18.676(7)
Space Group No.:	14	***(Å,°)***	α 90.00	β 124.20(4)	γ 90.00

R-Factor (%): 5.19 ***Temperature(K):*** 293 ***Density(g/cm³):*** 1.671

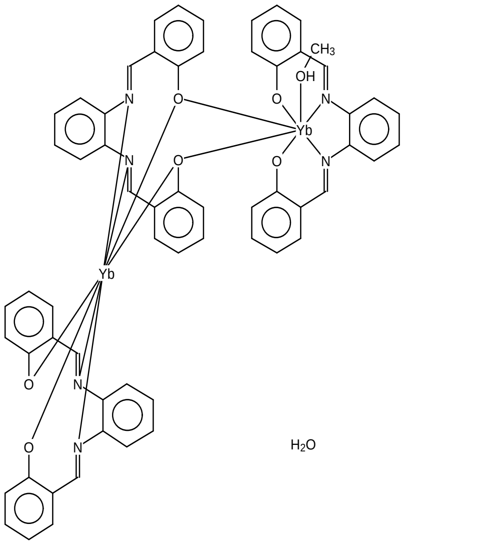

EKIWEB

Reference: D.Anselmo, E.C.Escudero-Adan, J.Benet-Bouchholz, A.W.Kleij (2010) *Dalton Trans.* ,**39,**8733

Formula: $C_{40} H_{57} N_3 O_2 Zn_1$

Compound Name: (tri-n-butylamine)-(2,2'-(1,2-phenylenebis((nitrilo)methylylidene))bis(6-t-butylphenolato))-zinc(ii)

Space Group:	Pca21	***Cell:***	***a*** 17.186(0)	***b*** 19.056(1)	***c*** 22.154(1)
Space Group No.:	29	***(Å,°)***	α 90.00	β 90.00	γ 90.00

R-Factor (%): 3.81 ***Temperature(K):*** 100 ***Density(g/cm³):*** 1.240

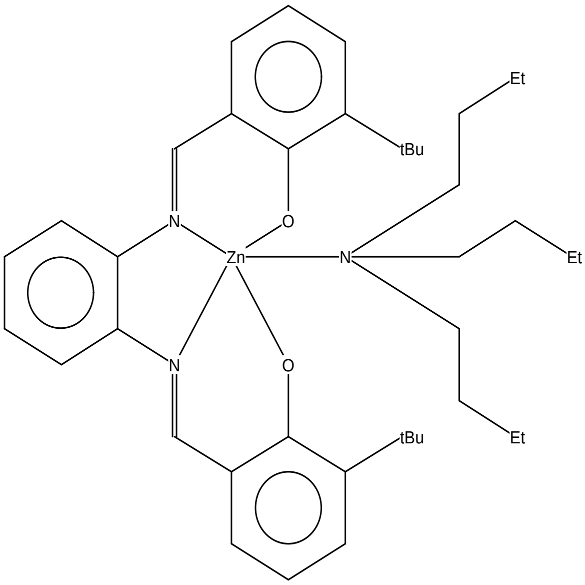

EKIWIF

Reference: D.Anselmo, E.C.Escudero-Adan, J.Benet-Bouchholz, A.W.Kleij (2010) *Dalton Trans.* ,**39,**8733

Formula: $C_{56} H_{61} N_4 O_5 Zn_2{}^{1-}, C_1 H_2 Cl_2, C_{16} H_{36} N_1{}^{1+}$

Compound Name: (μ_2-hydroxo)-bis(2,2'-(1,2-phenylenebis((nitrilo)methylylidene))bis(6-t-butylphenolato))-di-zinc tetra-n-butylammonium dichloromethane solvate

Space Group:	Pbca	***Cell:***	***a*** 23.195(5)	***b*** 24.208(5)	***c*** 24.371(5)
Space Group No.:	61	***(Å,°)***	α 90.00	β 90.00	γ 90.00

R-Factor (%): 5.41 ***Temperature(K):*** 100 ***Density(g/cm³):*** 1.289

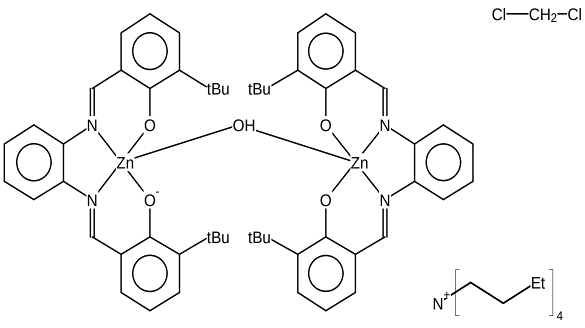

Search: search26 (Tue Dec 2 15:48:50 2014): Hits 177-180

ELUFUM

Reference: M.Cametti, M.Nissinen, A.D.Cort, L.Mandolini, K.Rissanen (2003) *Chem.Commun.* ,2420

Formula: $C_4 H_{12} N_1{}^{1+}, C_{34} H_{26} Cl_1 N_2 O_6 U_1{}^{1-}$

Compound Name: Tetramethylammonium chloro-(3,3'-bis(benzyloxy)-N,N'-phenylene-bis(salicylideneaminato)-N,N',O,O')-dioxo-uranium

Space Group: I-4 ***Space Group No.:*** 82

Cell: (Å,°) ***a*** 19.293(3) ***b*** 19.293(3) ***c*** 19.421(4) α 90.00 β 90.00 γ 90.00

R-Factor (%): 4.82 ***Temperature(K):*** 173 ***Density(g/cm³):*** 1.665

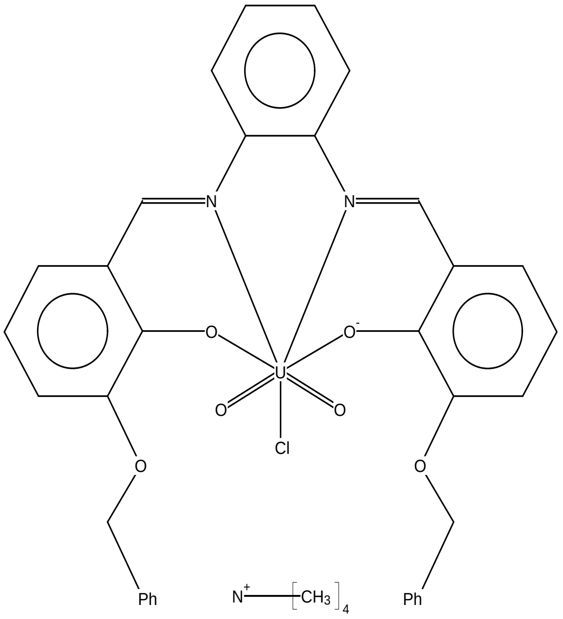

ELUGAT

Reference: M.Cametti, M.Nissinen, A.D.Cort, L.Mandolini, K.Rissanen (2003) *Chem.Commun.* ,2420

Formula: $C_{16} H_{36} N_1{}^{1+}, C_{34} H_{26} Cl_1 N_2 O_6 U_1{}^{1-}$

Compound Name: Tetrabutylammonium chloro-(3,3'-bis(benzyloxy)-N,N'-phenylene-bis(salicylideneaminato)-N,N',O,O')-dioxo-uranium

Space Group: P-1 ***Space Group No.:*** 2

Cell: (Å,°) ***a*** 13.323(0) ***b*** 18.294(0) ***c*** 20.369(0) α 94.68(0) β 104.46(0) γ 91.97(0)

R-Factor (%): 3.51 ***Temperature(K):*** 173 ***Density(g/cm³):*** 1.492

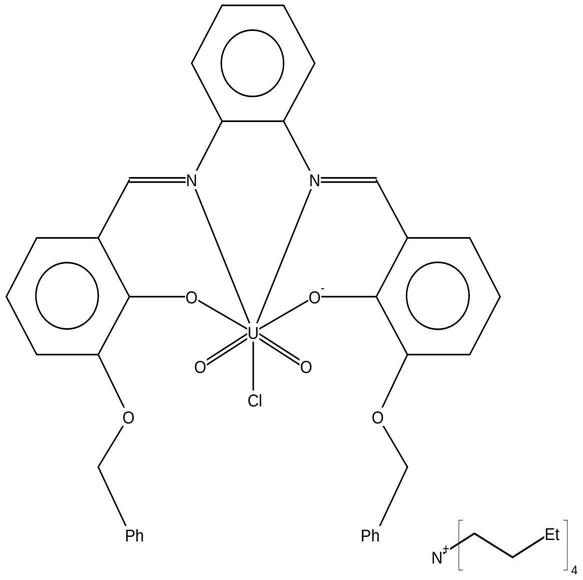

ESUMIP

Reference: N.E.Eltayeb, S.G.Teoh, E.Kusrini, R.Adnan, H.K.Fun (2010) *Spectrochim.Acta,Part A* ,**75,**453

Formula: $C_{22} H_{20} Cl_1 Mn_1 N_2 O_5, C_2 H_6 O_1, H_2 O_1$

Compound Name: Aqua(chloro)(2,2'-(1,2-phenylenebis((nitrilo)methylylidene))bis(6-methoxyphenolato))manganese(ii) ethanol solvate monohydrate

Space Group: Pbca ***Space Group No.:*** 61

Cell: (Å,°) ***a*** 17.102(3) ***b*** 16.765(3) ***c*** 16.026(3) α 90.00 β 90.00 γ 90.00

R-Factor (%): 12.75 ***Temperature(K):*** 100 ***Density(g/cm³):*** 1.581

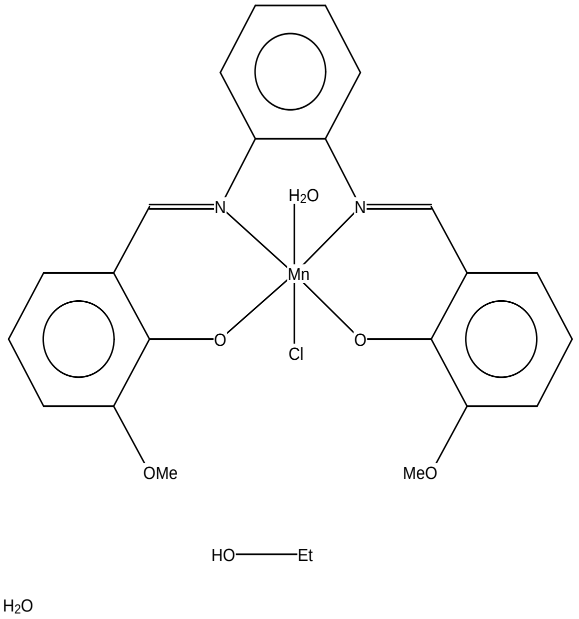

EWOLAE

Reference: M.Cametti, L.Ilander, A.Valkonen, M.Nieger, M.Nissinen, E.Nauha, K.Rissanen (2010) *Inorg.Chem.* ,**49,**11473

Formula: $C_4 H_{12} N_1{}^{1+}, C_{34} H_{24} Br_2 Cl_1 N_2 O_6 U_1{}^{1-}, 0.13(H_2 O_1)$

Compound Name: Tetramethylammonium chloro-dioxo-(2,2'-(1,2-phenylenebis((nitrilo) methylylidene))bis(6-((4-bromobenzyl)oxy)phenolato))-uranium hydrate

Space Group: I-4 ***Space Group No.:*** 82

Cell: (Å,°) ***a*** 19.663(0) ***b*** 19.663(0) ***c*** 19.580(0) α 90.00 β 90.00 γ 90.00

R-Factor (%): 3.01 ***Temperature(K):*** 173 ***Density(g/cm³):*** 1.871

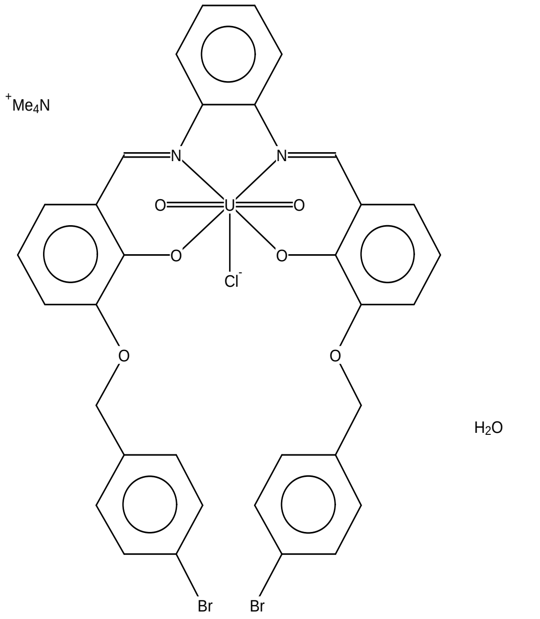

EWOLEI

Reference: M.Cametti, L.Ilander, A.Valkonen, M.Nieger, M.Nissinen, E.Nauha, K.Rissanen (2010) *Inorg.Chem.* ,**49,**11473

Formula: $4(C_4 H_{12} N_1{}^{1+}),3(C_{36} H_{30} Cl_1 N_2 O_8 U_1{}^{1-}),Cl_1{}^{1-},3(C_1 H_1 Cl_3)$

Compound Name: tetrakis(Tetramethylammonium) tris(chloro-dioxo-(2,2'-(1,2-phenylenebis((nitrilo)methylylidene))bis(6-((4-methoxybenzyl)oxy) phenolato))-uranium) chloride chloroform solvate

Space Group: I-43d ***Cell:*** ***a*** 37.759(4) ***b*** 37.759(4) ***c*** 37.759(4)
Space Group No.: 220 ***(Å,°)*** α 90.00 β 90.00 γ 90.00

R-Factor (%): 5.26 ***Temperature(K):*** 173 ***Density(g/cm³):*** 1.661

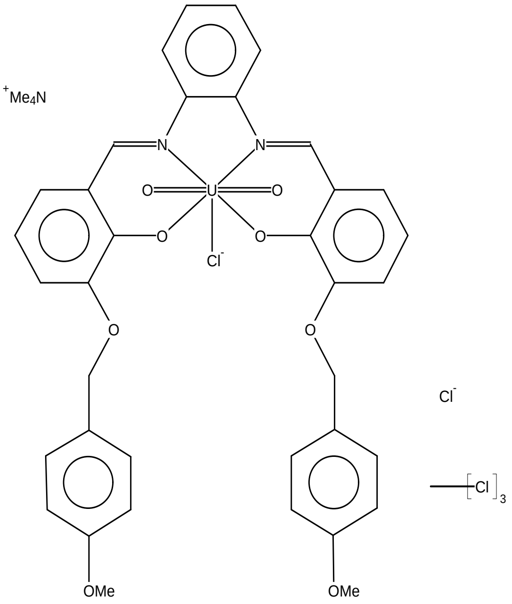

EWOLIM

Reference: M.Cametti, L.Ilander, A.Valkonen, M.Nieger, M.Nissinen, E.Nauha, K.Rissanen (2010) *Inorg.Chem.* ,**49,**11473

Formula: $C_{37} H_{30} N_4 O_{11} U_1,C_3 H_6 O_1$

Compound Name: Acetone-dioxo-(2,2'-(1,2-phenylenebis((nitrilo)methylylidene))bis(6-((4-nitrobenzyl)oxy)phenolato))-uranium acetone solvate

Space Group: Pnma ***Cell:*** ***a*** 9.487(0) ***b*** 16.943(0) ***c*** 23.859(0)
Space Group No.: 62 ***(Å,°)*** α 90.00 β 90.00 γ 90.00

R-Factor (%): 4.37 ***Temperature(K):*** 123 ***Density(g/cm³):*** 1.737

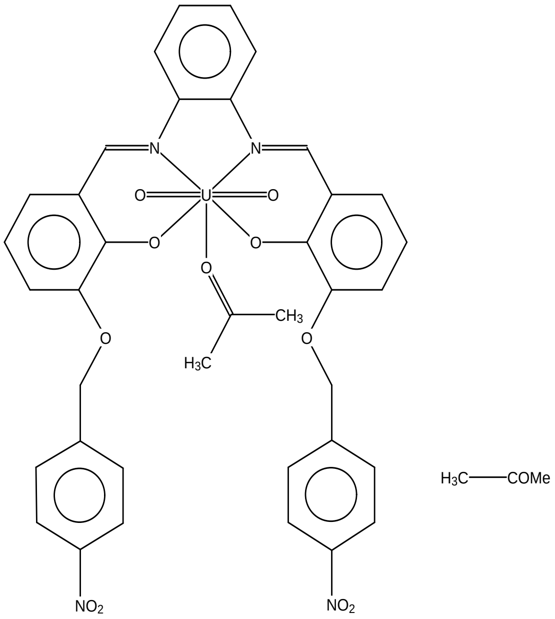

EWOMAF

Reference: M.Cametti, L.Ilander, A.Valkonen, M.Nieger, M.Nissinen, E.Nauha, K.Rissanen (2010) *Inorg.Chem.* ,**49,**11473

Formula: $C_{34} H_{40} N_2 O_7 U_1,1.13(C_2 H_3 N_1),H_2 O_1$

Compound Name: Aqua-dioxo-(2,2'-(1,2-phenylenebis((nitrilo)methylylidene))bis(6-(cyclohexylmethoxy)phenolato))-uranium acetonitrile solvate monohydrate

Space Group: P21/c ***Cell:*** ***a*** 26.143(0) ***b*** 14.674(0) ***c*** 18.773(0)
Space Group No.: 14 ***(Å,°)*** α 90.00 β 90.30(0) γ 90.00

R-Factor (%): 5.37 ***Temperature(K):*** 123 ***Density(g/cm³):*** 1.644

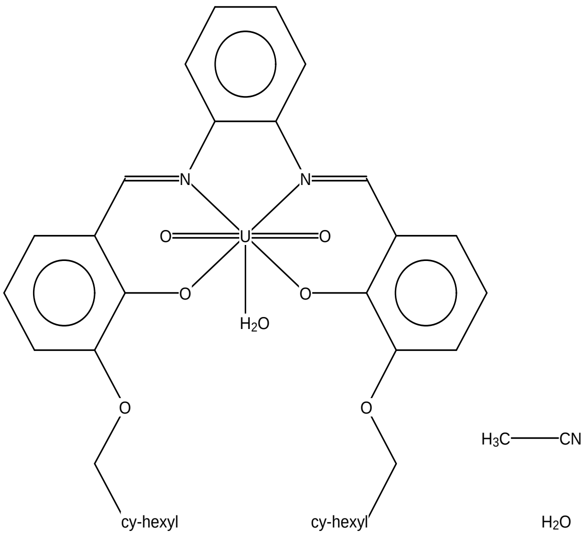

EXOJOQ

Reference: D.J.Price, S.R.Batten, B.Moubaraki, K.S.Murray (2003) *Indian J.Chem.,Sect.A:Inorg.,Bio-inorg.,Phys.,Theor.Anal.Chem.* ,**42,** 2256

Formula: $(C_{22} H_{14} Mn_1 N_5 O_2)n$

Compound Name: catena-[(μ_2-Dicyanamide)-(N,N'-o-phenylenebis(salicylaldiminato))-manganese(iii)]

Space Group: P21/n ***Cell:*** ***a*** 7.542(0) ***b*** 16.796(0) ***c*** 15.301(0)
Space Group No.: 14 ***(Å,°)*** α 90.00 β 103.85(0) γ 90.00

R-Factor (%): 3.41 ***Temperature(K):*** 123 ***Density(g/cm³):*** 1.536

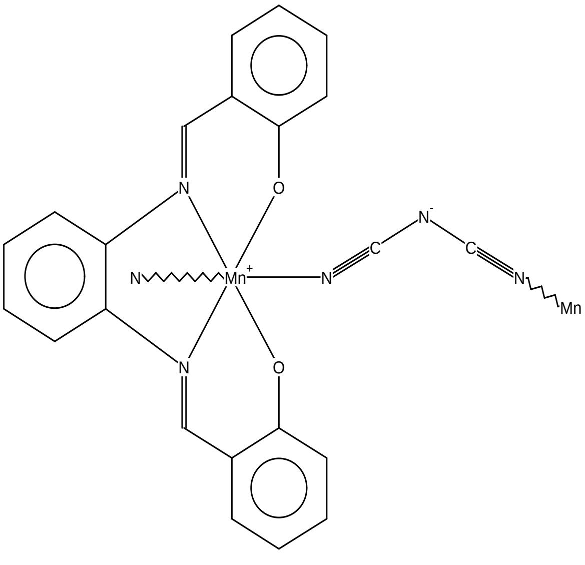

Search: search26 (Tue Dec 2 15:48:50 2014): Hits 185-188

EXOJUW

Reference: D.J.Price, S.R.Batten, B.Moubaraki, K.S.Murray (2003) *Indian J.Chem.,Sect.A:Inorg.,Bio-inorg.,Phys.,Theor.Anal.Chem.* ,**42,** 2256

Formula: $(C_{22} H_{14} Fe_1 N_5 O_2)n$

Compound Name: catena-[(μ_2-Dicyanamide)-(N,N'-o-phenylenebis(salicylaldiminato))-iron(iii)]

Space Group:	P21/n	***Cell:***	***a*** 7.702(0)	***b*** 16.987(0)	***c*** 14.986(0)
Space Group No.:	14	***(Å,°)***	α 90.00	β 104.20(0)	γ 90.00

R-Factor (%): 3.62 ***Temperature(K):*** 123 ***Density(g/cm³):*** 1.524

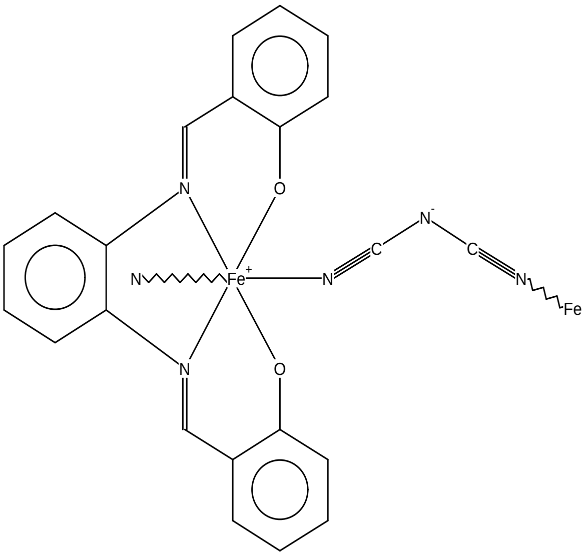

EYEGAR

Reference: J.Rahchamani, M.Behzad, A.Bezaatpour, V.Jahed, G.Dutkiewicz, M.Kubicki, M.Salehi (2011) *Polyhedron* ,**30,**2611

Formula: $C_{22} H_{16} N_4 O_9 V_1,C_3 H_7 N_1 O_1$

Compound Name: (2,2'-((4,5-Dinitro-1,2-phenylene)bis((nitrilo)methylylidene))bis(5-methoxyphenolato))-oxo-vanadium dimethylformamide solvate

Space Group:	P21/n	***Cell:***	***a*** 13.447(2)	***b*** 16.376(2)	***c*** 24.025(3)
Space Group No.:	14	***(Å,°)***	α 90.00	β 92.56(2)	γ 90.00

R-Factor (%): 8.11 ***Temperature(K):*** 295 ***Density(g/cm³):*** 1.519

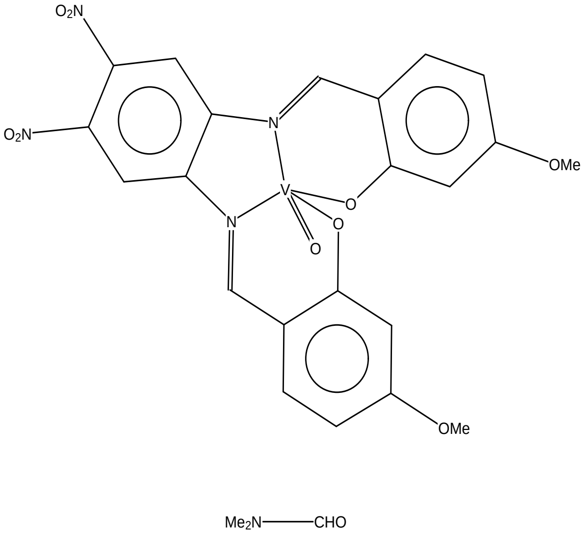

FASLES

Reference: S.Majumder, S.Dutta, L.M.Carrella, E.Rentschler, S.Mohanta (2011) *J.Mol.Struct.* ,**1006,**216

Formula: $C_{25} H_{28} Fe_1 N_2 O_6{}^{1+},Cl_1 O_4{}^{1-}$

Compound Name: Aqua-methanol-(2,2'-(1,2-phenylenebis((nitrilo)methylylidene))bis(6-ethoxyphenolato)-N,N',O,O')-iron(iii) perchlorate

Space Group:	P-1	***Cell:***	***a*** 9.790(0)	***b*** 11.854(0)	***c*** 13.025(1)
Space Group No.:	2	***(Å,°)***	α 111.58(0)	β 102.74(0)	γ 103.25(0)

R-Factor (%): 5.63 ***Temperature(K):*** 293 ***Density(g/cm³):*** 1.567

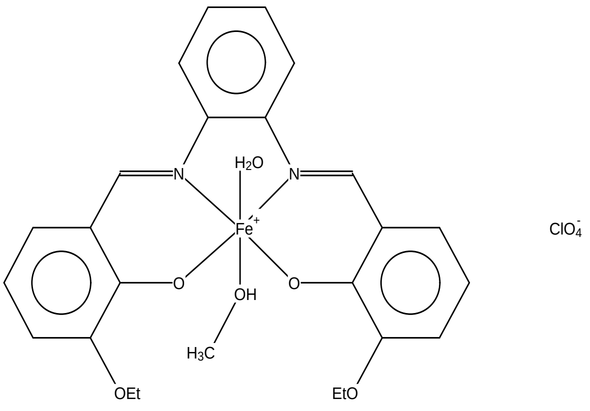

FASLIW

Reference: S.Majumder, S.Dutta, L.M.Carrella, E.Rentschler, S.Mohanta (2011) *J.Mol.Struct.* ,**1006,**216

Formula: $C_{24} H_{26} Fe_1 N_2 O_6{}^{1+},N_1 O_3{}^{1-},H_2 O_1$

Compound Name: Diaqua-(2,2'-(1,2-phenylenebis((nitrilo)methylylidene))bis(6-ethoxyphenolato)-N,N',O,O')-iron(iii) nitrate monohydrate

Space Group:	P-1	***Cell:***	***a*** 9.779(3)	***b*** 11.442(4)	***c*** 13.014(5)
Space Group No.:	2	***(Å,°)***	α 111.01(1)	β 103.52(1)	γ 99.58(1)

R-Factor (%): 9.26 ***Temperature(K):*** 293 ***Density(g/cm³):*** 1.502

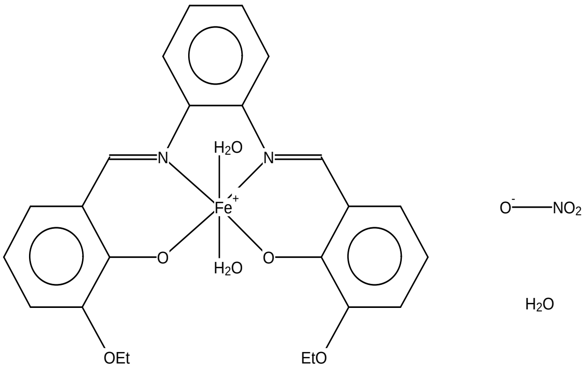

Search: search26 (Tue Dec 2 15:48:50 2014): Hits 189-192

FAZHIY

Reference: I.Yoon, M.Narita, T.Shimizu, M.Asakawa (2004) *J.Am.Chem.Soc.* ,**126,**16740

Formula: $C_{34}H_{32}N_2O_8Pd_1^{1+},C_{22}H_{32}N_1^{1+},Cl_1O_4^{1-}$

Compound Name: (2)-(bis(4-t-Butylbenzyl)ammonium)-((6,7,9,10,31,32,34,35-octahydro-16,12:25,29-di(mentheno)dibenzo(h,x)(1,4,7,10,13,16,23,26) hexaoxadiazacyclodotriacontine-37,38-diolato)-palladium)-rotaxane perchlorate

Space Group:	P-1	***Cell:***	***a*** 12.556(0)	***b*** 14.390(0)	***c*** 15.660(0)
Space Group No.:	2	***(Å,°)***	α 104.29(0)	β 96.23(0)	γ 102.11(0)

R-Factor (%): 3.57 ***Temperature(K):*** 203 ***Density(g/cm^3):*** 1.399

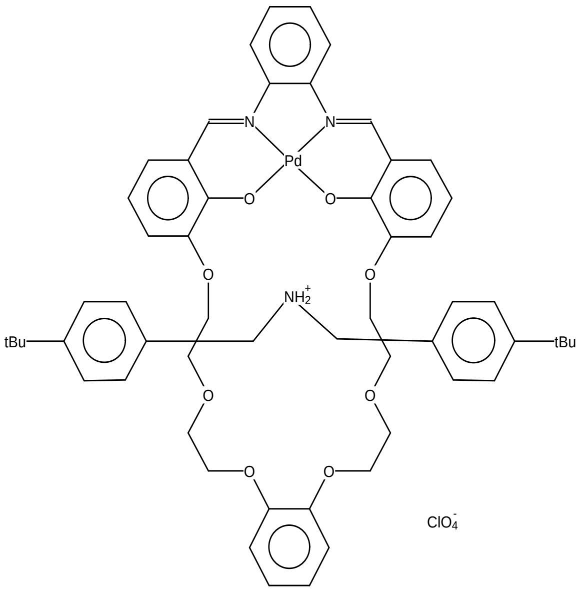

FEBSUC

Reference: C.Camp, V.Guidal, B.Biswas, J.Pecaut, L.Dubois, M.Mazzanti (2012) *Chemical Science* ,**3,**2433

Formula: $C_{25}H_{29}K_1N_1O_6^{1+},C_{44}H_{36}N_4Nd_1O_4^{1-},2(C_5H_5N_1)$

Compound Name: (Dibenzo-18-crown-6)-pyridine-potassium bis(2,2'-(1,2-phenylenebis((nitrilo)methylylidene))bis(6-methylphenolato))-neodymium(iii) pyridine solvate

Space Group:	P21/c	***Cell:***	***a*** 14.798(0)	***b*** 23.260(0)	***c*** 20.814(0)
Space Group No.:	14	***(Å,°)***	α 90.00	β 92.14(0)	γ 90.00

R-Factor (%): 4.69 ***Temperature(K):*** 150 ***Density(g/cm^3):*** 1.360

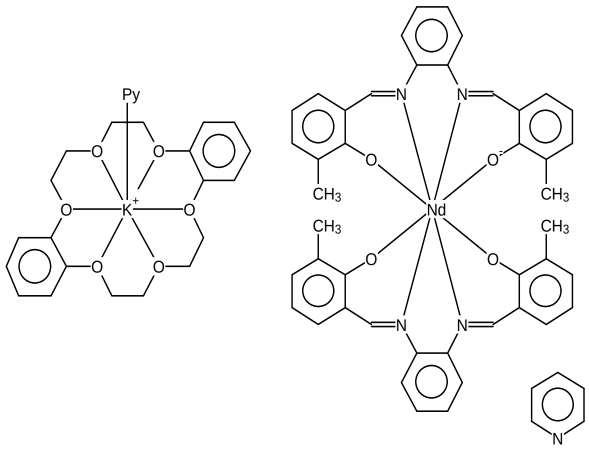

FEBTAJ

Reference: C.Camp, V.Guidal, B.Biswas, J.Pecaut, L.Dubois, M.Mazzanti (2012) *Chemical Science* ,**3,**2433

Formula: $C_{28}H_{40}K_1O_8^{1+},C_{72}H_{92}N_4Nd_1O_4^{1-},1.35(C_6H_{14})$

Compound Name: (Dibenzo-18-crown-6)-bis(tetrahydrofuran)-potassium bis(2,2'-(1,2-phenylenebis((nitrilo)methylylidene))bis(4,6-di-t-butylphenolato))-neodymium(iii) n-hexane solvate

Space Group:	P-1	***Cell:***	***a*** 16.171(1)	***b*** 17.540(1)	***c*** 19.536(1)
Space Group No.:	2	***(Å,°)***	α 90.67(0)	β 113.70(0)	γ 96.87(0)

R-Factor (%): 7.06 ***Temperature(K):*** 150 ***Density(g/cm^3):*** 1.243

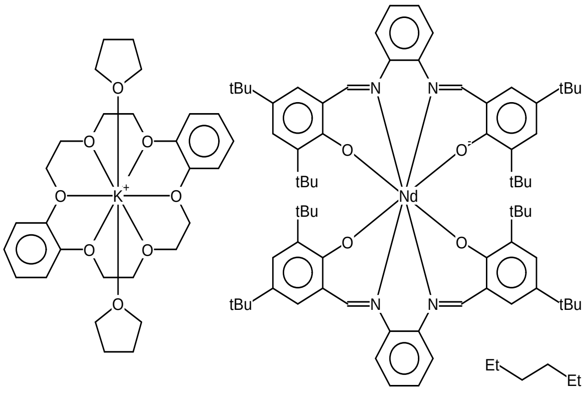

FEWVUY

Reference: Y.Nishida, K.Kino, S.Kida (1987) *J.Chem.Soc.,Dalton Trans.* ,1157

Formula: $C_{26}H_{20}Fe_1N_6O_2^{1+},C_{24}H_{20}B_1^{1-}$

Compound Name: (o-Phenylene-bis(salicylideneiminato-N,N',O,O'))-bis(imidazole)-iron(iii) tetraphenylborate

Space Group:	Pna21	***Cell:***	***a*** 26.966(8)	***b*** 16.969(7)	***c*** 8.977(3)
Space Group No.:	33	***(Å,°)***	α 90.00	β 90.00	γ 90.00

R-Factor (%): 5.80 ***Temperature(K):*** 295 ***Density(g/cm^3):*** 1.332

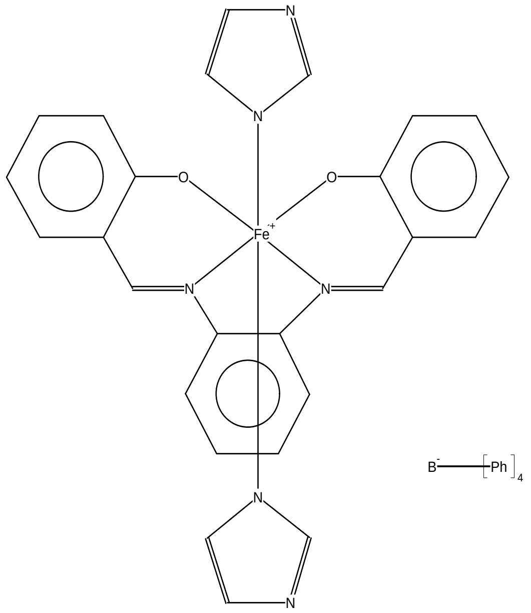

FEZCAO

Reference: C.Floriani, M.Fiallo, A.Chiesi-Villa, C.Guastini (1987) *J.Chem.Soc.,Dalton Trans.* ,1367

Formula: $C_{48} H_{44} Co_2 N_4 Na_1 O_6 {}^{1+}, C_{24} H_{20} B_1 {}^{1-}$

Compound Name: bis(N,N'-o-Phenylene-bis(salicylideneiminato-N,N',O,O')-cobalt(iii))-O,O',O'',O'''-bis(tetrahydrofuran)-sodium tetraphenylborate

Space Group:	Pnna	***Cell:***	***a*** 20.223(4)	***b*** 29.197(8)	***c*** 20.204(4)
Space Group No.:	52	***(Å,°)***	α 90.00	β 90.00	γ 90.00
R-Factor (%):	6.50	***Temperature(K):*** 295	***Density(g/cm³):*** 1.373		

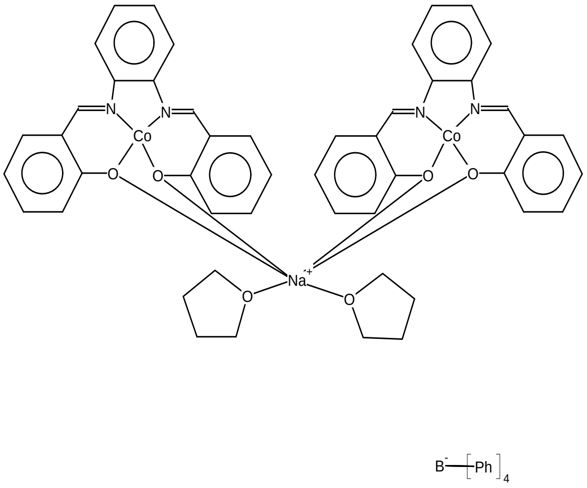

FITKIC

Reference: C.J.van Staveren, D.E.Fenton, D.N.Reinhoudt, J.van Eerden, S.Harkema (1987) *J.Am.Chem.Soc.* ,**109,**3456

Formula: $C_{35} H_{44} N_4 O_{13} U_1$

Compound Name: (27,30-Diaza-23,24,25:28,29:32,33,34-tribenzo-1,4,7,10,13,16,19,22-octa-oxatetratriaconta-26,30-diene-24,33-diolato-N,N',O,O')-dioxo-(urea-O)-uranium

Space Group:	P21/c	***Cell:***	***a*** 16.280(5)	***b*** 11.734(3)	***c*** 20.531(5)
Space Group No.:	14	***(Å,°)***	α 90.00	β 109.11(3)	γ 90.00
R-Factor (%):	2.80	***Temperature(K):*** 180	***Density(g/cm³):*** 1.733		

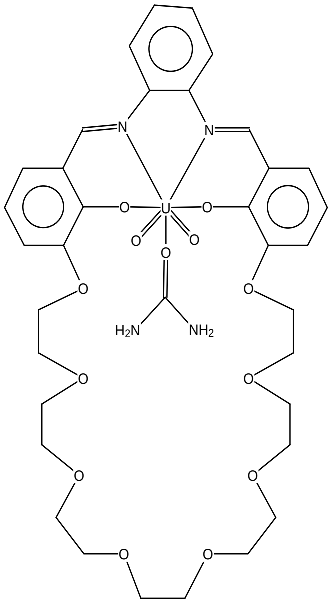

FITKIC10

Reference: C.J.van Staveren, J.van Eerden, F.C.J.M.van Veggel, S.Harkema, D.N.Reinhoudt (1988) *J.Am.Chem.Soc.* ,**110,**4994

Formula: $C_{35} H_{44} N_4 O_{13} U_1$

Compound Name: (9,10,12,13,15,16,18,19,21,22,24,25,27,28-Tetradecahydro-3,7:30,34-dimetheno-37,38-benzo-8,11,14,17,20,23,26,29,1,36-octaoxadiazacyclo-octatriacontine-39,40-diolato-N,N',O,O')-dioxo-(urea-O)-uranium

Space Group:	P21/c	***Cell:***	***a*** 16.280(5)	***b*** 11.734(3)	***c*** 20.531(5)
Space Group No.:	14	***(Å,°)***	α 90.00	β 109.11(3)	γ 90.00
R-Factor (%):	2.80	***Temperature(K):*** 180	***Density(g/cm³):*** 1.733		

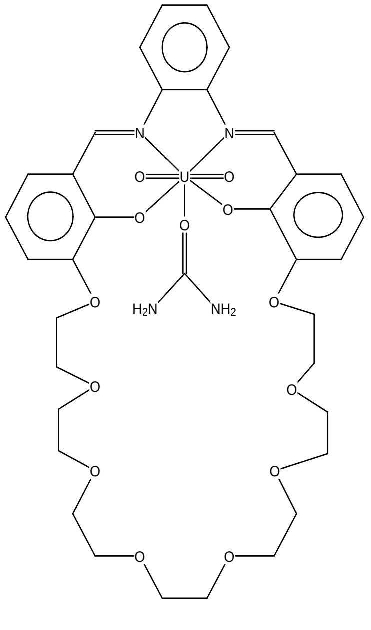

FIZZAP

Reference: A.Mederos, A.Medina, P.Gili, F.G.Manrique, P.Nunez (1986) *Anales de Quimica,Ser.B* ,**82,**338

Formula: $C_{20} H_{13} Cl_1 Cu_1 N_2 O_2$

Compound Name: N,N'-3,4-Chlorophenylene-bis(salicylaldiminato)-copper(ii)

Space Group:		***Cell:***	***a*** 12.260(20)	***b*** 11.650(20)	***c*** 13.470(10)
Space Group No.:	0	***(Å,°)***	α 68.57(7)	β 75.28(1)	γ 68.59(1)
R-Factor (%):	0.00	***Temperature(K):*** 295	***Density(g/cm³):*** 1.659		

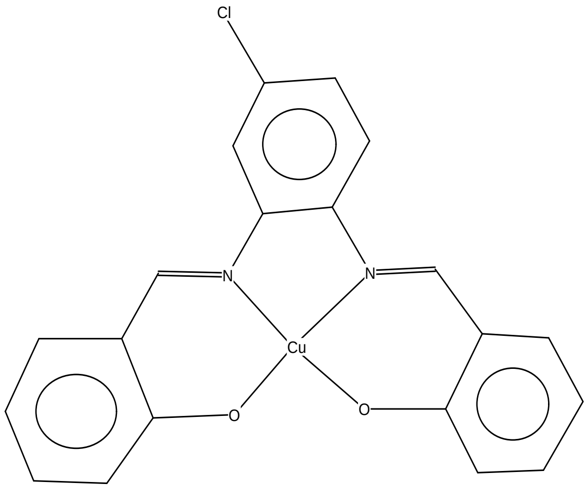

Search: search26 (Tue Dec 2 15:48:50 2014): Hits 197-200

FIZZET

Reference: A.Mederos, A.Medina, P.Gili, F.G.Manrique, P.Nunez (1986) *Anales de Quimica,Ser.B* ,**82,**338

Formula: $C_{21} H_{16} Cu_1 N_2 O_2$

Compound Name: N,N'-3,4-Toluene-bis(salicylaldiminato)-copper(ii)

Space Group:		***Cell:***	***a*** 5.358(5)	***b*** 25.000(20)	***c*** 13.770(10)
Space Group No.:	0	***(Å,°)***	α 90.00	β 100.38(8)	γ 90.00

R-Factor (%): 0.00 ***Temperature(K):*** 295 ***Density(g/cm³):*** 1.435

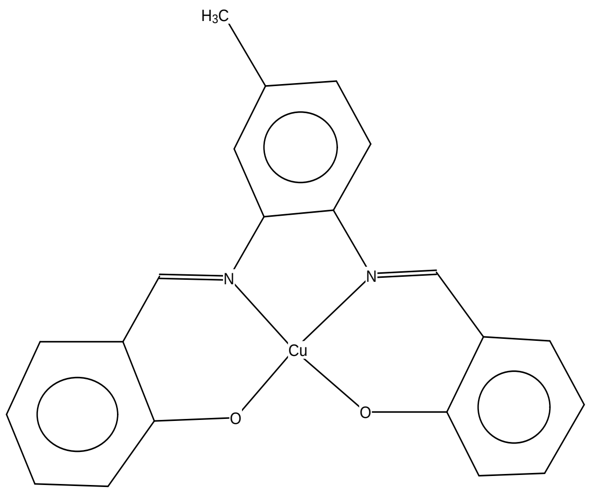

FONJEY

Reference: Gang-Chun Sun, Yi-Zhi Li, Nai-Sheng Chen, Liu-Fang Wang (2005) *Acta Crystallogr.,Sect.E:Struct.Rep.Online* ,**61,** m1065

Formula: $C_{36} H_{36} Cl_1 Cu_1 N_4 O_8$ $^{1+}$,$Cl_1 O_4$ $^{1-}$,$C_3 H_7 N_1 O_1$

Compound Name: (3,6,14,21-Tetra-aza-3,6-difuryl-10,25-dimethyl-27,28-dioxotetracyclo(18.4.3.3^{8,12}.0^{15,20})octacosa-8,10,12(28),13,17,19,21,23,25,27(1)-decaene-N,N',O,O')-(perchlorato-O)-copper(ii) perchlorate dimethylformamide solvate

Space Group:	P21/n	***Cell:***	***a*** 9.717(2)	***b*** 27.863(3)	***c*** 14.902(5)
Space Group No.:	14	***(Å,°)***	α 90.00	β 93.06(2)	γ 90.00

R-Factor (%): 6.08 ***Temperature(K):*** 293 ***Density(g/cm³):*** 1.524

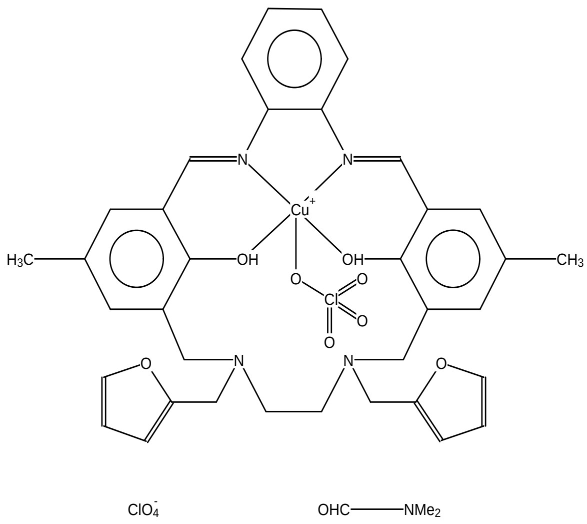

FONQII

Reference: F.Sakamoto, T.Sumiya, M.Fujita, T.Tada, Xiang Shi Tan, E.Suzuki, I.Okura, Y.Fujii (1998) *Chem.Lett.* ,1127

Formula: $C_{28} H_{38} Mn_1 N_4 O_4$ $^{3+}$,3(Br_1 $^{1-}$),2($H_2 O_1$)

Compound Name: Diaqua-(N,N'-bis(5-trimethylammoniomethylsalicylidene)benzene-1.2-diamine)manganese(iii) tribromide dihydrate

Space Group:	C2/c	***Cell:***	***a*** 15.280(10)	***b*** 17.546(9)	***c*** 15.720(10)
Space Group No.:	15	***(Å,°)***	α 90.00	β 119.11(5)	γ 90.00

R-Factor (%): 8.70 ***Temperature(K):*** 295 ***Density(g/cm³):*** 1.489

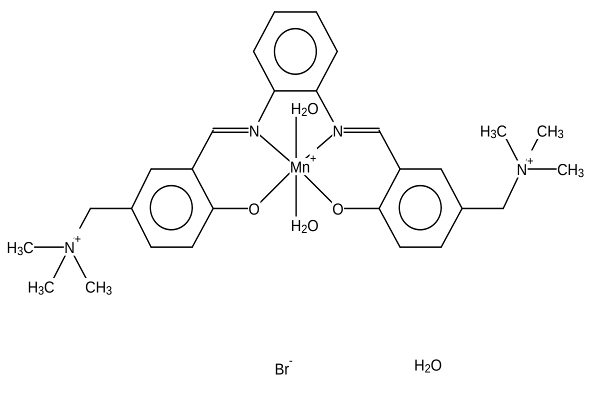

FOWKAE

Reference: M.Salehi, S.Meghdadi, M.Amirnasr, K.Mereiter (2009) *Acta Crystallogr.,Sect.E:Struct.Rep.Online* ,**65,**m942

Formula: $C_{30} H_{24} Co_1 N_4 O_2$ $^{1+}$,$Cl_1 O_4$ $^{1-}$

Compound Name: (2,2'-(1,2-Phenylenebis(nitrilomethylylidene))diphenolato)-bis(pyridine)-cobalt(iii) perchlorate

Space Group:	C2/c	***Cell:***	***a*** 33.403(1)	***b*** 10.659(0)	***c*** 16.350(0)
Space Group No.:	15	***(Å,°)***	α 90.00	β 112.18(0)	γ 90.00

R-Factor (%): 3.09 ***Temperature(K):*** 200 ***Density(g/cm³):*** 1.555

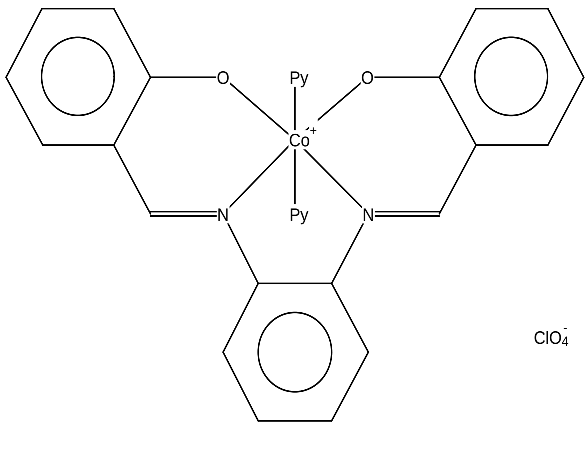

FUGCUG

Reference: M.Niu, G.Liu, D.Wang, J.Dou (2009)
Acta Crystallogr.,Sect.E:Struct.Rep.Online ,**65,**m1357

Formula: $C_{20}\ H_{14}\ N_2\ Ni_1\ O_4,2(C_1\ H_4\ O_1)$

Compound Name: (5,5'-Dihydroxy-2,2'-(o-phenylenebis(nitrilomethylidyne))diphenolato-N,N',O,O')-nickel(ii) methanol solvate

Synonym: (1,1'-Dihydroxy-4,4'-(1,2-Phenylenebis(nitrilomethylylidene))dibenzene-3-olate-N,N',O,O')-nickel(ii) methanol solvate

Space Group:	C2/c	***Cell:***	***a***	15.673(3)	***b*** 15.090(2)	***c***	8.868(0)
Space Group No.:	15	***(Å,°)***	α	90.00	β 104.59(0)	γ	90.00
R-Factor (%):	3.97	***Temperature(K):***	298	***Density(g/cm³):***	1.535		

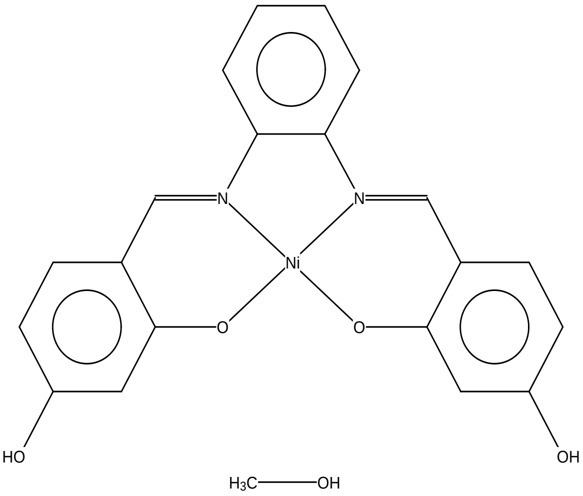

FUGHUL

Reference: Ting-Ting Wang, Ji-Min Xie (2009)
Acta Crystallogr.,Sect.E:Struct.Rep.Online ,**65,**m1387

Formula: $C_8\ H_{20}\ N_1\ ^{1+},2(C_{22}\ H_{22}\ Mn_1\ N_2\ O_6\ ^{1+}),C_6\ Fe_1\ N_6\ ^{3-},2(C_2\ H_6\ O_1),2(C_1\ H_4\ O_1)$

Compound Name: Tetraethylammonium bis(diaqua(6,6'-dimethoxy-2,2'-[o-phenylenebis(nitrilomethylidyne)]diphenolato)-manganese(iii)) hexacyano-iron(iii) methanol ethanol solvate

Space Group:	C2/c	***Cell:***	***a***	24.830(20)	***b*** 12.467(11)	***c***	22.915(19)
Space Group No.:	15	***(Å,°)***	α	90.00	β 98.08(1)	γ	90.00
R-Factor (%):	4.83	***Temperature(K):***	296	***Density(g/cm³):***	1.352		

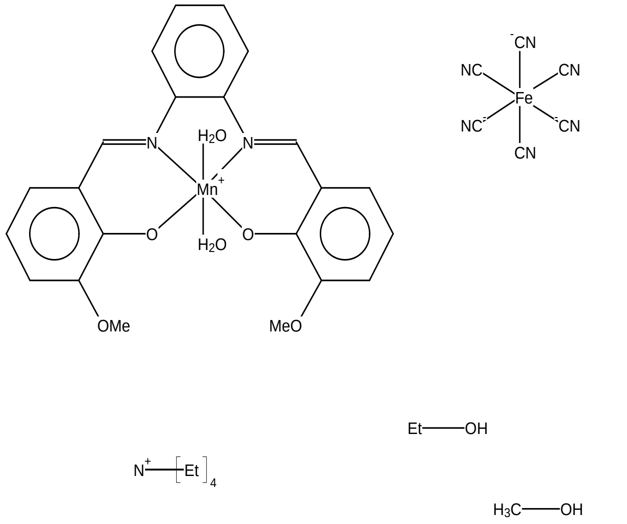

FUQHUV

Reference: A.W.Kleij, M.Lutz, A.L.Spek, J.N.H.Reek (2009)
Private Communication ,

Formula: $C_{45}\ H_{42}\ Cl_2\ N_3\ O_2\ P_1\ Zn_1$

Compound Name: (2,2'-((4,5-Dichloro-1,2-phenylene)bis((nitrilo-κN)methylylidene))bis(6-t-butylphenolato-κO))-(4-(diphenylphosphino)pyridine-κN)-zinc

Space Group:	P21/c	***Cell:***	***a***	12.458(0)	***b*** 19.835(0)	***c***	19.854(0)
Space Group No.:	14	***(Å,°)***	α	90.00	β 124.23(0)	γ	90.00
R-Factor (%):	3.01	***Temperature(K):***	150	***Density(g/cm³):***	1.349		

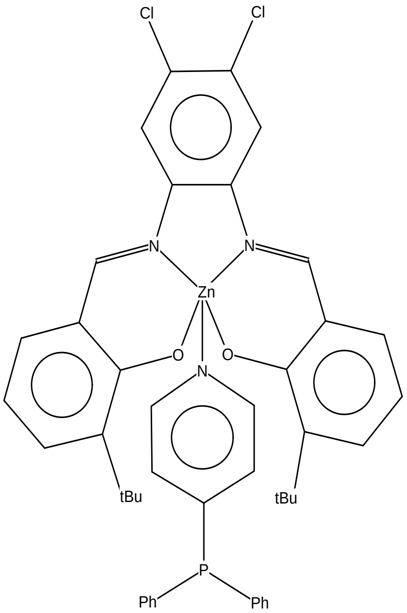

GAQPIX

Reference: N.Komatsuzaki, Y.Himeda, M.Goto, K.Kasuga, H.Sugihara, H.Arakawa (1999) *Chem.Lett.* ,327

Formula: $C_{46}\ H_{32}\ N_8\ Ni_1\ O_2\ Ru_1\ ^{2+},2(Cl_1\ O_4\ ^{1-}),H_2\ O_1$

Compound Name: (μ_2-5,6-bis(Salicylideneamino)-1,10-phenanthroline-N,N',N'',N''',O,O')-bis(2,2'-bipyridyl-N,N')-ruthenium-nickel diperchlorate monohydrate

Space Group:	P-1	***Cell:***	***a***	9.525(2)	***b*** 12.208(3)	***c***	20.436(5)
Space Group No.:	2	***(Å,°)***	α	96.69(2)	β 90.55(2)	γ	99.94(2)
R-Factor (%):	6.80	***Temperature(K):***	298	***Density(g/cm³):***	1.580		

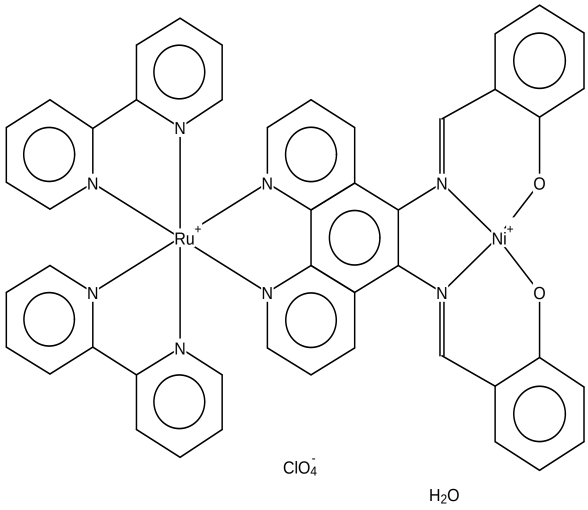

Search: search26 (Tue Dec 2 15:48:50 2014): Hits 205-208

GATHUF

Reference: A.W.Kleij, M.Lutz, A.L.Spek, P.W.N.M.van Leeuwen, J.N.H.Reek (2005) *Chem.Commun.* ,3661

Formula: $C_{99} H_{96} Cl_6 N_9 O_6 P_1 Zn_3,5(C_2 H_3 N_1)$

Compound Name: (μ_3-tris(4-Pyridyl)phosphine)-tris(N,N'-(4,5-dichloro-o-phenylenediamine) bis(3-t-butylsalicylidenealdiminaoto)-zinc(ii)) acetonitrile solvate

Space Group:	P-1	***Cell:***	***a***	14.482(0)	***b***	17.058(0)	***c***	23.233(0)
Space Group No.:	2	***(Å,°)***	α	78.24(0)	β	87.65(0)	γ	71.21(0)

R-Factor (%): 4.21 ***Temperature(K):*** 150 ***Density(g/cm^3):*** 1.345

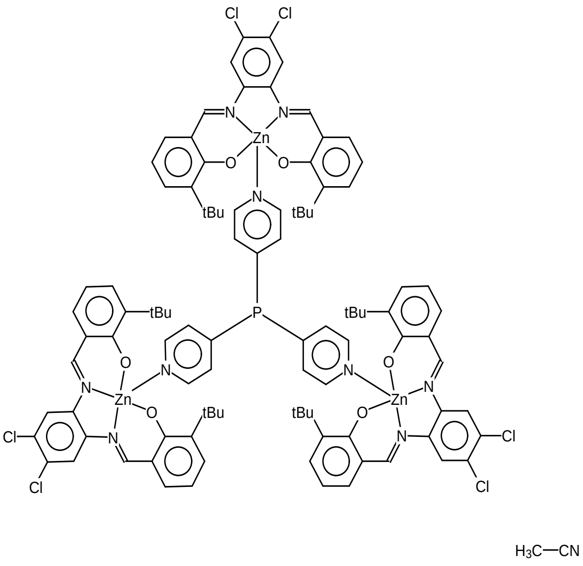

GEKXAW

Reference: L.Salmon, P.Thuery, E.Riviere, S.Miyamoto, T.Yamato, M.Ephritikhine (2006) *New J.Chem.* ,**30,**1220

Formula: $C_{56} H_{56} Cl_4 N_4 O_4 U_2,4(C_4 H_8 O_1)$

Compound Name: bis(μ_2-Chloro)-(N,N:N',N'-bis(2,2'-dioxy-3,3'-dimethylidene-5,5'-di-t-butylbiphenyl)bis(benzene-1,2-diamine))-dichloro-di-uranium(iv) tetrahydrofuran solvate

Space Group:	P-1	***Cell:***	***a***	11.817(0)	***b***	12.694(0)	***c***	12.975(0)
Space Group No.:	2	***(Å,°)***	α	86.74(0)	β	65.50(0)	γ	79.66(0)

R-Factor (%): 3.30 ***Temperature(K):*** 100 ***Density(g/cm^3):*** 1.673

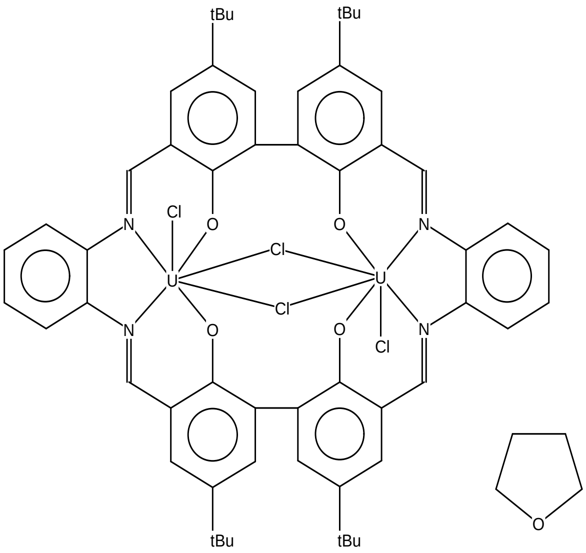

GEKXEA

Reference: L.Salmon, P.Thuery, E.Riviere, S.Miyamoto, T.Yamato, M.Ephritikhine (2006) *New J.Chem.* ,**30,**1220

Formula: $C_{66} H_{66} Cl_4 N_6 O_4 U_2,3(C_5 H_5 N_1)$

Compound Name: bis(μ_2-Chloro)-(N,N:N',N'-bis(2,2'-dioxy-3,3'-dimethylidene-5,5'-di-t-butylbiphenyl)bis(benzene-1,2-diamine))-dichloro-dipyridyl-di-uranium(iv) pyridine solvate

Space Group:	P21/n	***Cell:***	***a***	14.282(0)	***b***	12.093(0)	***c***	21.821(1)
Space Group No.:	14	***(Å,°)***	α	90.00	β	91.60(0)	γ	90.00

R-Factor (%): 4.09 ***Temperature(K):*** 100 ***Density(g/cm^3):*** 1.642

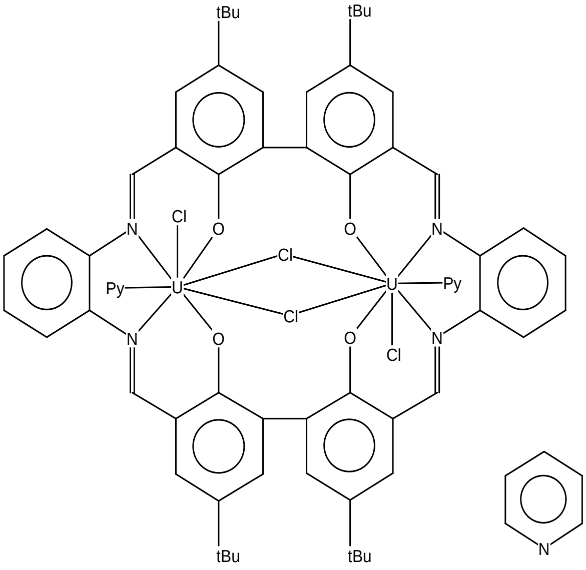

GEKXIE

Reference: L.Salmon, P.Thuery, E.Riviere, S.Miyamoto, T.Yamato, M.Ephritikhine (2006) *New J.Chem.* ,**30,**1220

Formula: $C_{76} H_{84} N_4 O_{12} U_2,5(C_4 H_8 O_1)$

Compound Name: (μ_2-N,N:N',N'-bis(2,2'-dioxy-3,3'-dimethylidene-5,5'-di-t-butylbiphenyl) bis(benzene-1,2-diamine))-tetrakis(acetylacetonato)-di-uranium(iv) tetrahydrofuran solvate

Space Group:	P21/c	***Cell:***	***a***	16.273(0)	***b***	29.795(1)	***c***	20.313(0)
Space Group No.:	14	***(Å,°)***	α	90.00	β	111.16(0)	γ	90.00

R-Factor (%): 4.86 ***Temperature(K):*** 100 ***Density(g/cm^3):*** 1.506

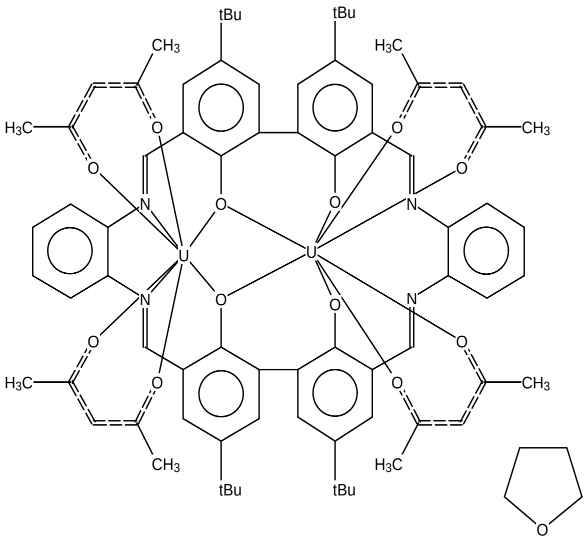

Search: search26 (Tue Dec 2 15:48:50 2014): Hits 209-212

GEKXOK

Reference:	L.Salmon, P.Thuery, E.Riviere, S.Miyamoto, T.Yamato, M.Ephritikhine (2006) *New J.Chem.* ,**30,**1220
Formula:	$C_{81} H_{92} N_4 O_{14} U_2$,2($C_5 H_5 N_1$)
Compound Name:	(μ_2-14,19,34,39-tetrakis(t-Butyl)-3,10,23,30-tetraazaoctacyclo[35.3.1.1^{12,16}.1^{17,21}.1^{32,36}.0^{2,10}.0^{4,9}.0^{24,29}]tetratetraconta-1(41),2,4,6,8,12(44),13,15,17(43),18,20,22,24,26,28,30,32(42),33,35,37,39-henicosaene-44-ol-41,42,43-triolato)-pentakis(acetylacetonato)-di-uranium(iv) pyridine solvate

Space Group:	P-1	***Cell:***	***a***	15.520(0)	***b***	16.775(0)	***c***	18.193(0)
Space Group No.:	2	***(Å,°)***	α	77.59(0)	β	81.49(0)	γ	68.00(0)
R-Factor (%):	3.09	***Temperature(K):***	100		***Density(g/cm^3):***	1.537		

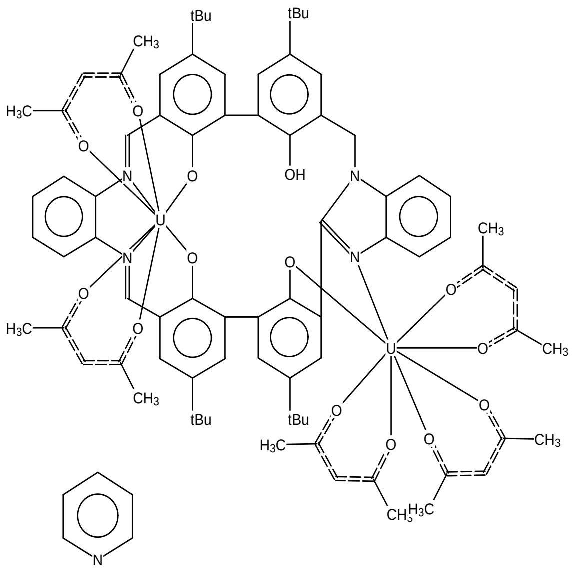

GIBZEW

Reference:	C.J.van Staveren, J.van Eerden, F.C.J.M.van Veggel, S.Harkema, D.N.Reinhoudt (1988) *J.Am.Chem.Soc.* ,**110,**4994
Formula:	$C_{34} H_{40} N_2 Ni_1 O_{10}$,$C_1 H_4 N_2 O_1$
Compound Name:	(9,10,12,13,15,16,18,19,21,22,24,25,27,28-Tetradecahydro-3,7:30,34-dimetheno-37,38-benzo-8,11,14,17,20,23,26,29,1,36-octaoxadiazacyclo-octatriacontine-39,40-diolato-N,N',O,O')-nickel urea clathrate

Space Group:	P21/n	***Cell:***	***a***	8.584(4)	***b***	22.435(8)	***c***	18.156(5)
Space Group No.:	14	***(Å,°)***	α	90.00	β	90.41(4)	γ	90.00
R-Factor (%):	5.20	***Temperature(K):***	295		***Density(g/cm^3):***	1.435		

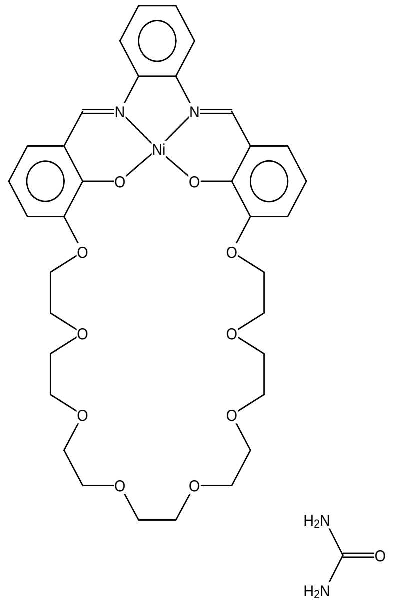

GOLMAW

Reference:	Zhong Yu, T.Kuroda-Sowa, A.Nabei, M.Maekawa, T.Okubo (2009) *Acta Crystallogr.,Sect.E:Struct.Rep.Online* ,**65,**m257
Formula:	$C_{27} H_{20} Co_1 N_3 O_4 S_1$,$C_4 H_{10} O_1$,$C_1 H_2 Cl_2$
Compound Name:	(6,6'-Dimethoxy-2,2'-(naphthalene-2,3-diylbis(nitrilomethylidyne)) diphenolato)-isothiocyanato-cobalt(iii) diethyl ether dichloromethane solvate

Space Group:	P21/n	***Cell:***	***a***	9.194(0)	***b***	13.364(1)	***c***	25.910(3)
Space Group No.:	14	***(Å,°)***	α	90.00	β	92.46(0)	γ	90.00
R-Factor (%):	7.49	***Temperature(K):***	120		***Density(g/cm^3):***	1.463		

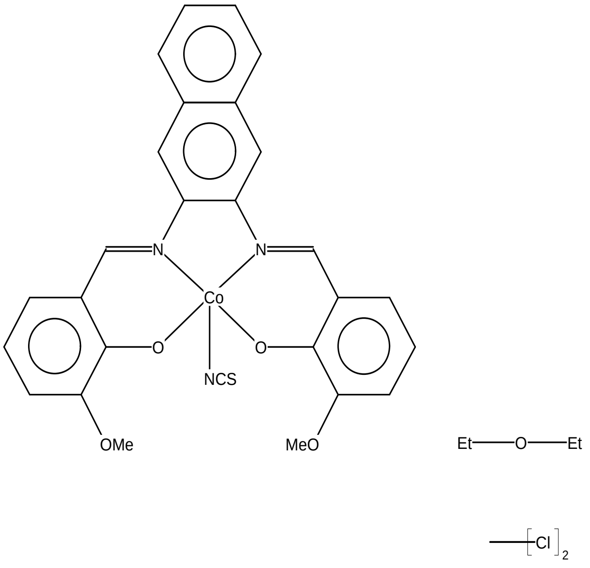

GUDYOU

Reference:	Pei-Pei Yang, Zong-Wei Li, Xiao-Ling Wang, Li-Cun Li, Dai-Zheng Liao (2009) *Inorg.Chim.Acta* ,**362,**3333
Formula:	($C_{33} H_{21} Mn_1 N_6 O_3$)n,n($C_2 H_3 N_1$)
Compound Name:	catena-(μ_2-((4-((Cyano-N)-(cyano-N)methylene)cyclohexa-2,5-dien-1-yl)(methoxy)malononitrilato)-(2,2'-(1,2-phenylene-bis((nitrilo-N)methylylidene))diphenolato-O)-di-manganese acetonitrile solvate)

Space Group:	P21/n	***Cell:***	***a***	15.381(4)	***b***	13.967(3)	***c***	15.629(4)
Space Group No.:	14	***(Å,°)***	α	90.00	β	116.22(0)	γ	90.00
R-Factor (%):	5.30	***Temperature(K):***	113		***Density(g/cm^3):***	1.424		

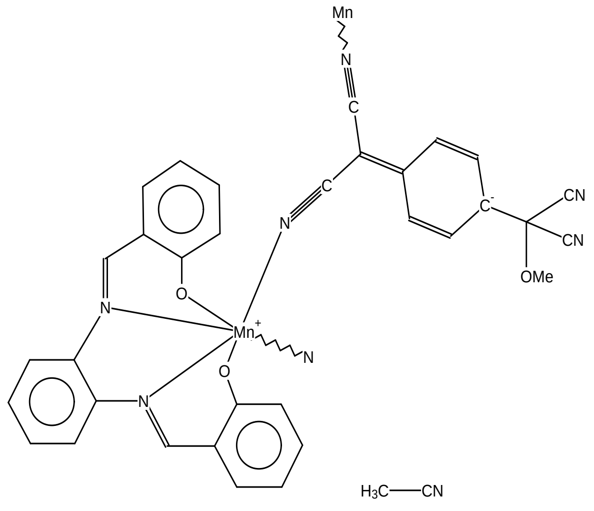

Search: search26 (Tue Dec 2 15:48:50 2014): Hits 213-216

GUNRAI

Reference: T.Panther, U.Baumann, U.Behrens (2001)
Z.Anorg.Allg.Chem. ,**627,**238

Formula: $C_{36} H_{40} Cu_2 N_4 O_2{}^{2+}$,2($Cl_1 O_4{}^{1-}$),2($C_3 H_7 N_1 O_1$)

Compound Name: (μ_2-bis(4-t-Butyl-2,6-bis(iminomethyl)phenolato)-N,N''-(o-phenylene)-N',N'''-(cyclohexane-1,2-diyl))-di-copper(ii) diperchlorate dimethylformamide solvate

Space Group: P1 ***Cell:*** ***a*** 9.598(3) ***b*** 16.568(3) ***c*** 16.593(4)
Space Group No.: 1 ***(Å,°)*** α 61.85(2) β 79.05(2) γ 73.50(2)

R-Factor (%): 5.58 ***Temperature(K):*** 173 ***Density(g/cm³):*** 1.541

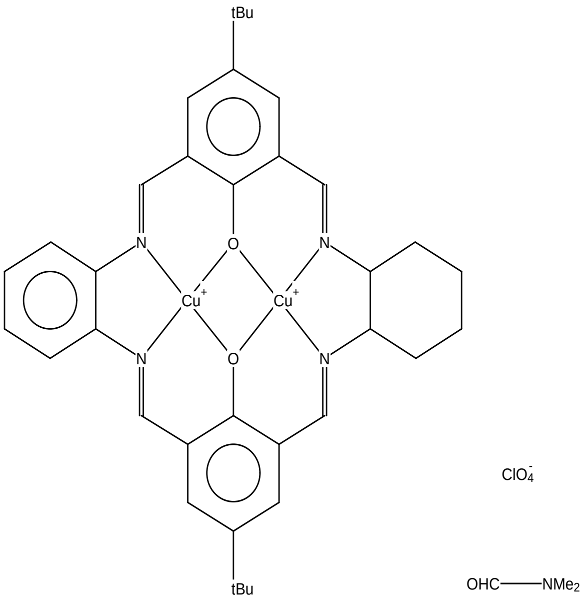

GUNREM

Reference: T.Panther, U.Baumann, U.Behrens (2001)
Z.Anorg.Allg.Chem. ,**627,**238

Formula: $C_{50} H_{42} Cu_2 N_4 O_2{}^{2+}$,2($Cl_1 O_4{}^{1-}$),2($C_3 H_7 N_1 O_1$)

Compound Name: (μ_2-bis(4-t-Butyl-2,6-bis(iminomethyl)phenolato)-N,N''-(o-phenylene)-N',N'''-(1,1'-binaphthyl-2,2'-diyl))-di-copper(ii) diperchlorate dimethylformamide solvate

Space Group: P21 ***Cell:*** ***a*** 12.530(8) ***b*** 15.466(9) ***c*** 14.551(8)
Space Group No.: 4 ***(Å,°)*** α 90.00 β 104.17(4) γ 90.00

R-Factor (%): 3.47 ***Temperature(K):*** 173 ***Density(g/cm³):*** 1.461

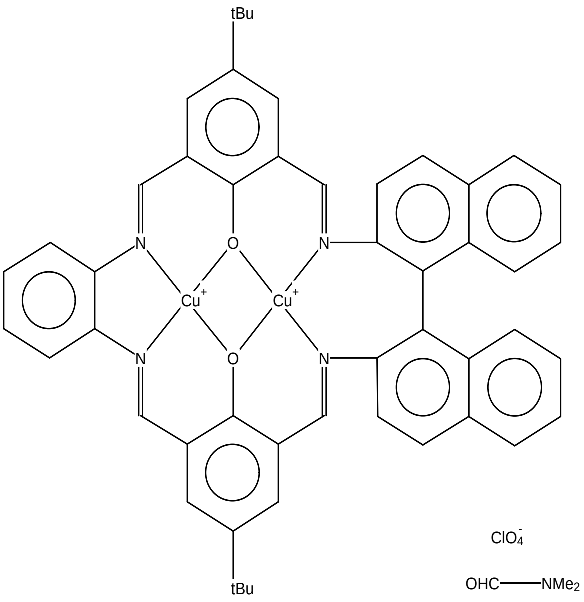

GURKUZ

Reference: K.Oyaizu, E.L.Dewi, E.Tsuchida (2001) *Inorg.Chim.Acta* ,
321,205

Formula: $C_{40} H_{28} Fe_2 N_4 O_5$,$C_1 H_2 Cl_2$,$C_4 H_{10} O_1$

Compound Name: (μ_2-Oxo)-bis(N,N'-o-phenylenebis(salicylideneiminato))-di-iron(iii) dichloromethane diethyl ether solvate

Space Group: P-1 ***Cell:*** ***a*** 13.525(7) ***b*** 14.874(10) ***c*** 10.886(4)
Space Group No.: 2 ***(Å,°)*** α 103.24(4) β 103.37(4) γ 66.38(5)

R-Factor (%): 6.60 ***Temperature(K):*** 295 ***Density(g/cm³):*** 1.576

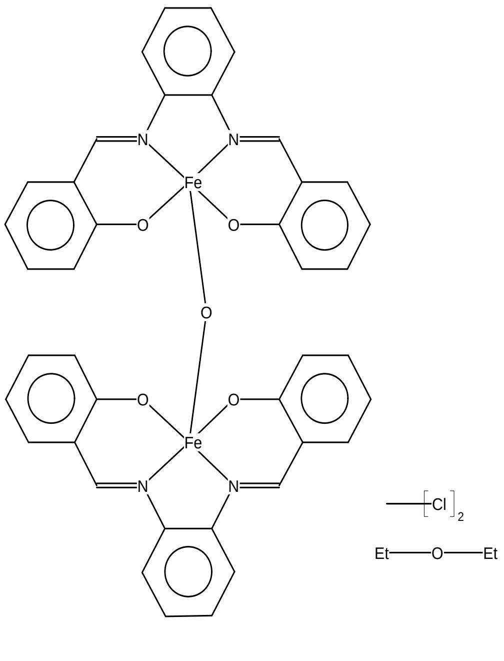

GUTGAE

Reference: Chun-Hua Ge, Ai-Li Cui, Hui-Zhong Kou (2009)
Inorg.Chem.Commun. ,**12,**926

Formula: $C_{40} H_{32} Cu_2 Mn_2 N_{22} O_6$,4($H_2 O_1$)

Compound Name: tetrakis(μ_2-Azido-N,N)-diaqua-diazido-bis(o-phenylenebis(salicylideneaminato))-di-copper(ii)-di-manganese(iii) tetrahydrate

Space Group: P21/n ***Cell:*** ***a*** 13.847(3) ***b*** 10.880(2) ***c*** 17.039(3)
Space Group No.: 14 ***(Å,°)*** α 90.00 β 109.88(3) γ 90.00

R-Factor (%): 6.17 ***Temperature(K):*** 293 ***Density(g/cm³):*** 1.686

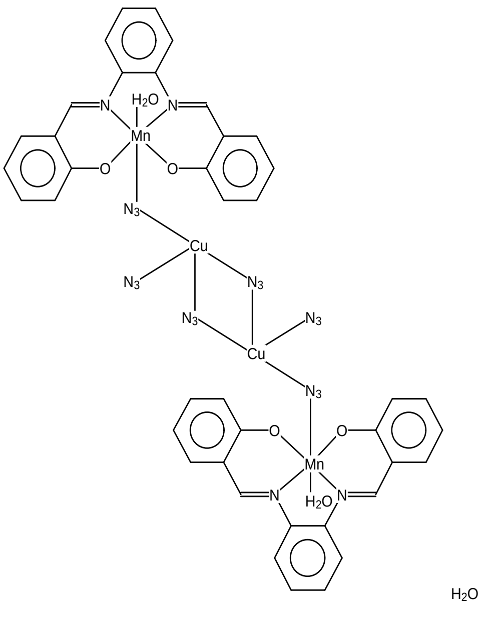

Search: search26 (Tue Dec 2 15:48:50 2014): Hits 217-220

GUWQEV

Reference:	Liqun Sun (2010) *Z.Kristallogr.-New Cryst.Struct.* ,**225,**149
Formula:	$C_{20} H_{14} Co_1 N_2 O_4$,2($C_1 H_4 O_1$)
Compound Name:	(5,5'-Dihydroxy-2,2'-[o-phenylenebis-(nitrilomethylidyne)]diphenolato)-cobalt(ii) methanol solvate
Synonym:	(4,4'-(1,2-Phenylenebis((nitrilo)methylidene))dibenzene-1,3-diolato)-cobalt methanol solvate

Space Group:	P-1	***Cell:***	***a***	8.048(1)	***b***	10.964(1)	***c***	11.689(1)
Space Group No.:	2	***(Å,°)***	α	91.84(0)	β	94.56(0)	γ	94.34(0)
R-Factor (%):	3.56	***Temperature(K):***	293	***Density(g/cm³):***	1.522			

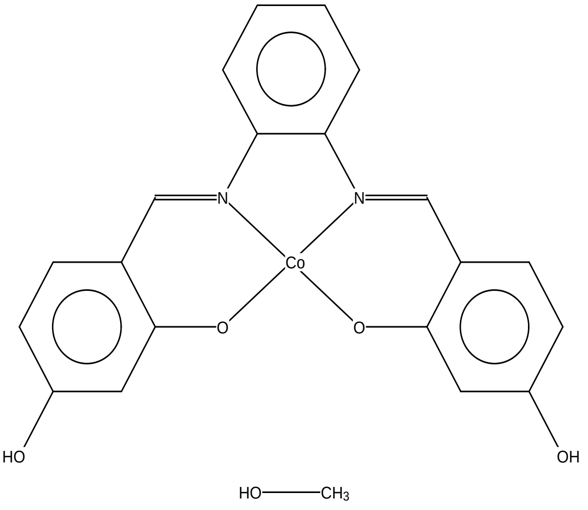

GUYQIA

Reference:	M.Sanchez, M.J.Harvey, F.Nordstrom, S.Parkin, D.A.Atwood (2002) *Inorg.Chem.* ,**41,**5397
Formula:	$C_{46} H_{68} N_2 O_5 Sr_1$
Compound Name:	(Ethanol)-bis(tetrahydrofuran)-(N,N'-o-phenylene-bis(3,5-di-t-butylsalicylideneimine))-strontium

Space Group:	P212121	***Cell:***	***a***	10.559(0)	***b***	16.207(0)	***c***	26.762(0)
Space Group No.:	19	***(Å,°)***	α	90.00	β	90.00	γ	90.00
R-Factor (%):	5.39	***Temperature(K):***	173	***Density(g/cm³):***	1.184			

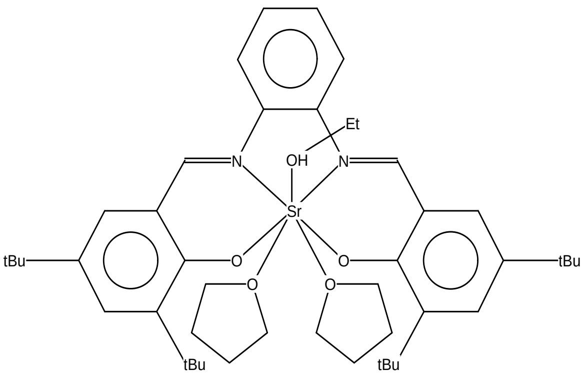

HABXUF

Reference:	Tu-Lin Che, Quan-Chang Gao, Jian-She Zhao, Gai Zhang (2008) *Chin.J.Chem.* ,**26,**1079
Formula:	$C_{22} H_{20} Cl_1 Mn_1 N_2 O_5$,$C_1 H_4 O_1$
Compound Name:	Aqua-chloro-(2,2'-(1,2-phenylenebis((nitrilo)methylylidene))bis(6-methoxyphenolato))-manganese(iii) methanol solvate

Space Group:	P-1	***Cell:***	***a***	9.144(3)	***b***	11.581(4)	***c***	12.068(7)
Space Group No.:	2	***(Å,°)***	α	107.38(0)	β	99.04(0)	γ	107.29(0)
R-Factor (%):	5.65	***Temperature(K):***	296	***Density(g/cm³):***	1.525			

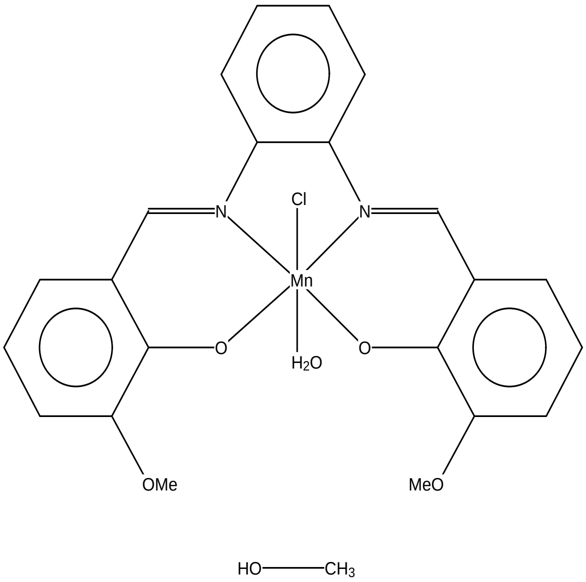

HAGKAD

Reference:	M.Das, S.Chattopadhyay (2011) *Inorg.Chim.Acta* ,**378,**303
Formula:	$H_4 N_1$ $^{1+}$,2($C_{22} H_{18} N_2 Ni_1 O_4$),$C_1 N_1 S_1$ $^{1-}$,$H_2 O_1$
Compound Name:	Ammonium bis((2,2'-(1,2-phenylenebis((nitrilo)methylylidene))bis(6-methoxyphenolato)-N,N',O,O')-nickel(ii)) thiocyanate monohydrate

Space Group:	P21/n	***Cell:***	***a***	13.864(0)	***b***	14.027(0)	***c***	22.271(1)
Space Group No.:	14	***(Å,°)***	α	90.00	β	94.30(0)	γ	90.00
R-Factor (%):	6.14	***Temperature(K):***	293	***Density(g/cm³):***	1.477			

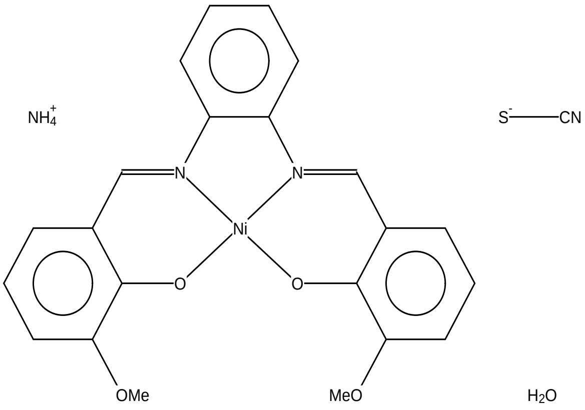

Search: search26 (Tue Dec 2 15:48:50 2014): Hits 221-224

HAJNOV

Reference: A.R.van Doorn, W.Verboom, S.Harkema, D.N.Reinhoudt (1992) *Synthesis* ,119

Formula: $C_{39} H_{45} N_3 O_{13} U_1,C_2 H_3 N_1,3(H_2 O_1)$

Compound Name: Aqua-(9,10,12,13,15,16,26,27,29,30,32,33-dodecahydro-3,7:35,39-(24H) dimetheno-19,23-nitrilo-18H-8,11,14,17,25,28,31,34,1,41-benzo-octaoxadiazacyclotritetracontine-46,48-diolato)-dioxo-uranium acetonitrile trihydrate clathrate

Space Group: P21/c ***Cell:*** ***a*** 12.846(3) ***b*** 15.120(3) ***c*** 23.494(5)
Space Group No.: 14 ***(Å,°)*** α 90.00 β 98.63(2) γ 90.00

R-Factor (%): 5.80 ***Temperature(K):*** 295 ***Density(g/cm3):*** 1.615

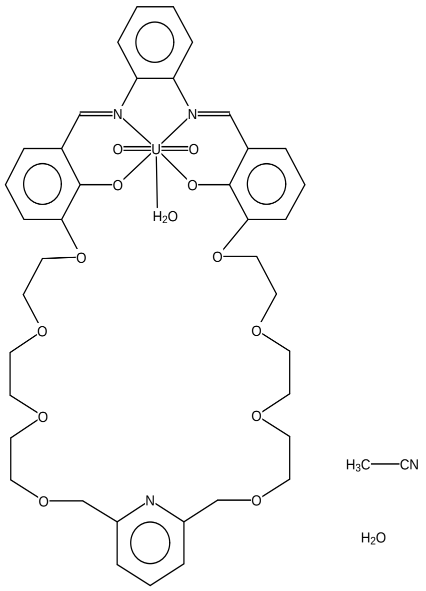

HAJZIB

Reference: S.G.Jonasdottir, C.G.Kim, D.Coucouvanis (1993) *Inorg.Chem.* ,**32**,3591

Formula: $C_{24} H_{20} Mo_2 N_4 Ni_1 O_4 S_4{}^{2-},C_8 H_{20} N_1{}^{1+},K_1{}^{1+},3(C_3 H_7 N_1 O_1)$

Compound Name: Potassium tetraethylammonium (μ_2-2,3-ethylene-5,6:13,14-bis(5'-methylbenzoyl)-9,10-(4',5'-dioxophenylene)-1,4,8,11-tetrazacyclotetradeca-7,12-diene-N,N',N'',N''',O,O')-bis(μ_2-sulfido)-disulfido-dioxo-di-molybdenum-nickel dimethylformamide solvate

Space Group: P21/a ***Cell:*** ***a*** 9.350(7) ***b*** 32.194(20) ***c*** 17.603(8)
Space Group No.: 14 ***(Å,°)*** α 90.00 β 104.29(5) γ 90.00

R-Factor (%): 8.00 ***Temperature(K):*** 295 ***Density(g/cm3):*** 1.547

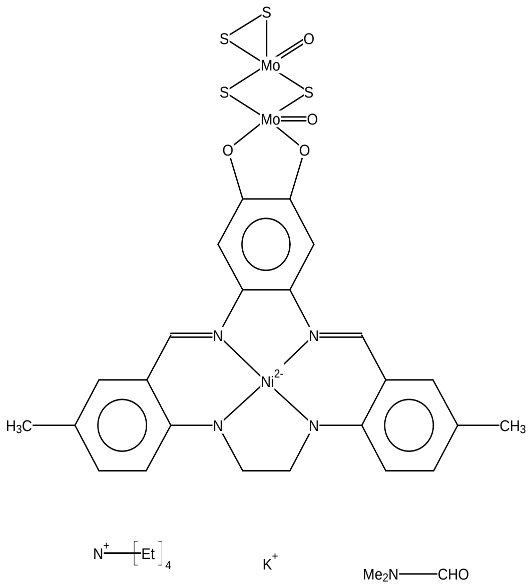

HAMSOF

Reference: S.Mondal, S.Hazra, S.Sarkar, S.Sasmal, S.Mohanta (2011) *J.Mol.Struct.* ,**1004**,204

Formula: $C_{24} H_{24} Cu_1 N_3 Na_1 O_8,2(C_2 H_3 N_1)$

Compound Name: (μ_2-2,2'-(1,2-Phenylenebis((nitrilo)methylylidene))bis(6-ethoxyphenolato) -N,N',O,O,O',O',O'',O''')-aqua-(nitrato-O)-copper(ii)-sodium(i) acetonitrile solvate

Space Group: P21/n ***Cell:*** ***a*** 10.791(6) ***b*** 24.059(13) ***c*** 11.829(6)
Space Group No.: 14 ***(Å,°)*** α 90.00 β 105.93(1) γ 90.00

R-Factor (%): 4.48 ***Temperature(K):*** 296 ***Density(g/cm3):*** 1.464

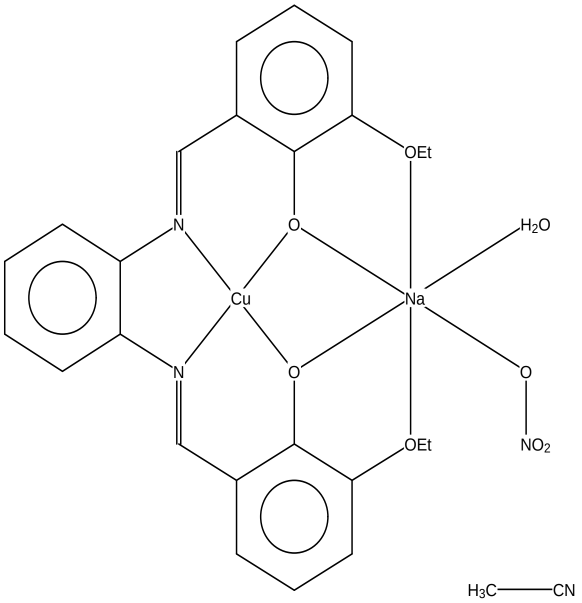

HAMSUL

Reference: S.Mondal, S.Hazra, S.Sarkar, S.Sasmal, S.Mohanta (2011) *J.Mol.Struct.* ,**1004**,204

Formula: $C_{25} H_{26} Cu_1 N_5 Na_1 O_5,C_1 H_4 O_1$

Compound Name: (μ_2-2,2'-(1,2-Phenylenebis((nitrilo)methylylidene))bis(6-ethoxyphenolato) -N,N',O,O,O',O',O'',O''')-azido-(methanol-O)-copper(ii)-sodium(i) methanol solvate

Space Group: P-1 ***Cell:*** ***a*** 9.389(12) ***b*** 10.710(14) ***c*** 14.644(19)
Space Group No.: 2 ***(Å,°)*** α 69.75(1) β 88.35(1) γ 82.64(1)

R-Factor (%): 7.70 ***Temperature(K):*** 296 ***Density(g/cm3):*** 1.443

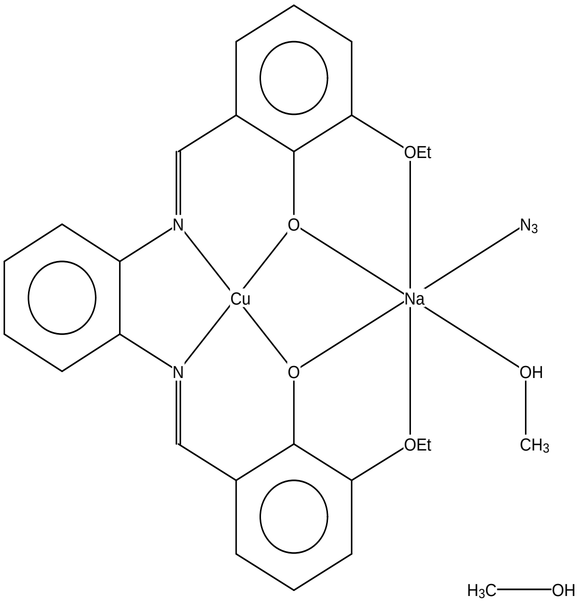

Search: search26 (Tue Dec 2 15:48:50 2014): Hits 225-228

HAMTAS

Reference: S.Mondal, S.Hazra, S.Sarkar, S.Sasmal, S.Mohanta (2011) *J.Mol.Struct.* ,**1004,**204

Formula: $C_{26} H_{25} Cl_1 Cu_1 N_3 Na_1 O_8$,0.5($C_2 H_3 N_1$)

Compound Name: (μ_2-2,2'-(1,2-Phenylenebis((nitrilo)methylylidene))bis(6-ethoxyphenolato)-N,N',O,O,O',O',O'',O''')-acetonitrile-(perchlorato-O)-copper(ii)-sodium(i) acetonitrile solvate

**Space Group:**	P-1	**Cell:**	**a** 8.605(0)	**b** 12.407(1)	**c** 14.442(1)
**Space Group No.:**	2	**(Å,°)**	α 73.45(0)	β 73.77(0)	γ 82.33(0)
**R-Factor (%):**	6.06	**Temperature(K):** 296	**Density(g/cm³):** 1.524		

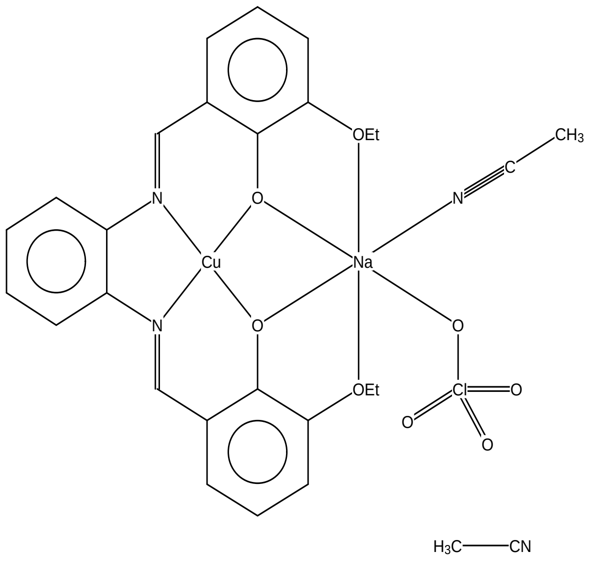

HAMTEW

Reference: S.Mondal, S.Hazra, S.Sarkar, S.Sasmal, S.Mohanta (2011) *J.Mol.Struct.* ,**1004,**204

Formula: $C_{25} H_{26} B_1 Cu_1 F_4 N_2 Na_1 O_5$,$H_2 O_1$

Compound Name: (μ_2-2,2'-(1,2-Phenylenebis((nitrilo)methylylidene))bis(6-ethoxyphenolato)-N,N',O,O,O',O',O'',O''')-(tetrafluoroborato-F)-methanol-copper(ii)-sodium(i) monohydrate

**Space Group:**	P-1	**Cell:**	**a** 9.885(1)	**b** 10.342(1)	**c** 14.882(2)
**Space Group No.:**	2	**(Å,°)**	α 69.76(0)	β 88.53(0)	γ 74.73(0)
**R-Factor (%):**	6.60	**Temperature(K):** 296	**Density(g/cm³):** 1.513		

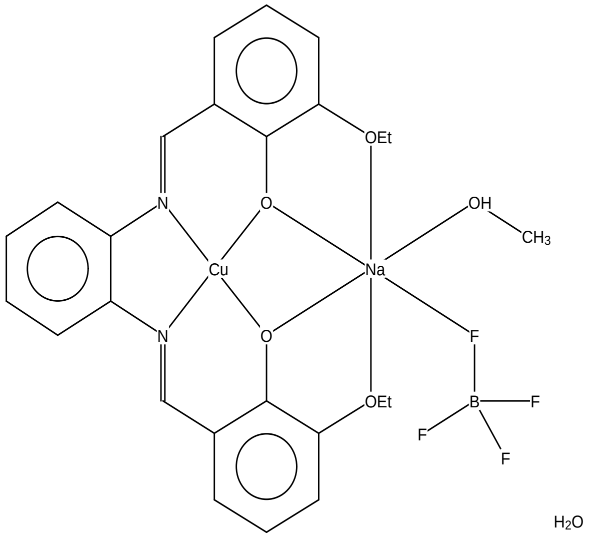

HAMTIA

Reference: S.Mondal, S.Hazra, S.Sarkar, S.Sasmal, S.Mohanta (2011) *J.Mol.Struct.* ,**1004,**204

Formula: 2($C_{24} H_{24} Cu_1 N_2 Na_1 O_5{}^{1+}$),$C_{24} H_{22} Cu_1 N_2 O_4$,2($C_{24} H_{20} B_1{}^{1-}$)

Compound Name: bis((μ_2-2,2'-(1,2-Phenylenebis((nitrilo)methylylidene))bis(6-ethoxyphenolato)-N,N',O,O,O',O',O'',O''')-aqua-copper(ii)-sodium(i)) (2,2'-(1,2-phenylenebis((nitrilo)methylylidene))bis(6-ethoxyphenolato)-N,N',O,O')-copper(ii) bis(tetraphenylborate)

**Space Group:**	Pbcn	**Cell:**	**a** 27.066(3)	**b** 17.953(1)	**c** 20.758(1)
**Space Group No.:**	60	**(Å,°)**	α 90.00	β 90.00	γ 90.00
**R-Factor (%):**	6.62	**Temperature(K):** 296	**Density(g/cm³):** 1.395		

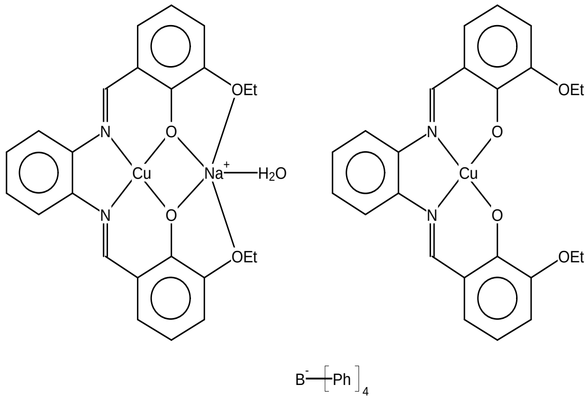

HAMTOG

Reference: S.Mondal, S.Hazra, S.Sarkar, S.Sasmal, S.Mohanta (2011) *J.Mol.Struct.* ,**1004,**204

Formula: $C_{27} H_{28} Cl_1 Cu_1 K_1 N_2 O_9$

Compound Name: (μ_2-2,2'-(1,2-Phenylenebis((nitrilo)methylylidene))bis(6-ethoxyphenolato)-N,N',O,O,O',O',O'',O''')-acetone-(perchlorato-O)-copper(ii)-potassium(i)

**Space Group:**	P-1	**Cell:**	**a** 10.240(0)	**b** 10.575(0)	**c** 14.720(0)
**Space Group No.:**	2	**(Å,°)**	α 70.99(0)	β 81.69(0)	γ 71.32(0)
**R-Factor (%):**	5.32	**Temperature(K):** 296	**Density(g/cm³):** 1.543		

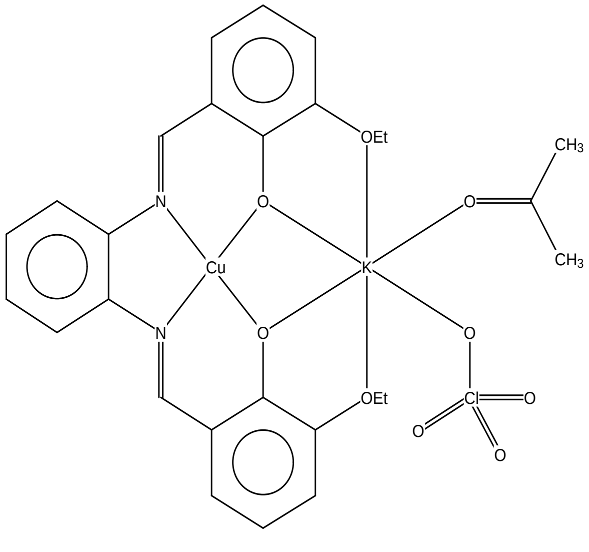

Search: search26 (Tue Dec 2 15:48:50 2014): Hits 229-232

HAMTUM

Reference: S.Mondal, S.Hazra, S.Sarkar, S.Sasmal, S.Mohanta (2011) *J.Mol.Struct.* ,**1004,**204

Formula: C_{24} H_{24} Ca_1 Cl_2 Cu_1 N_2 O_{13}

Compound Name: (μ_2-2,2'-(1,2-Phenylenebis((nitrilo)methylylidene))bis(6-ethoxyphenolato)-N,N',O,O,O',O',O'',O''')-aqua-bis(perchlorato-O)-calcium(ii)-copper(ii)

Space Group:	P-1	***Cell:***	***a*** 9.494(5)	***b*** 11.759(6)	***c*** 13.524(6)
Space Group No.:	2	***(Å,°)***	α 98.66(0)	β 102.25(0)	γ 105.14(0)

R-Factor (%): 7.84 ***Temperature(K):*** 296 ***Density(g/cm^3):*** 1.728

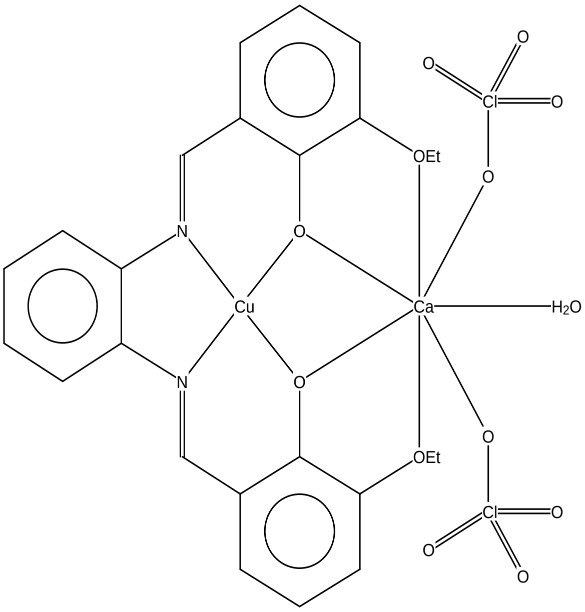

HAMVAU

Reference: S.Mondal, S.Hazra, S.Sarkar, S.Sasmal, S.Mohanta (2011) *J.Mol.Struct.* ,**1004,**204

Formula: C_{24} H_{24} Ca_1 Cu_1 N_4 O_{11},C_{24} H_{22} Cu_1 N_2 O_4,C_3 H_6 O_1,H_2 O_1

Compound Name: (μ_2-2,2'-(1,2-Phenylenebis((nitrilo)methylylidene))bis(6-ethoxyphenolato)-N,N',O,O,O',O',O'',O''')-aqua-(nitrato-O)-(nitrato-O,O')-calcium(ii)-copper(ii) (2,2'-(1,2-phenylenebis((nitrilo)methylylidene))bis(6-ethoxyphenolato)-N,N',O,O')-copper(ii) acetone solvate monohydrate

Space Group:	P21/n	***Cell:***	***a*** 18.316(1)	***b*** 15.053(1)	***c*** 18.885(1)
Space Group No.:	14	***(Å,°)***	α 90.00	β 96.81(0)	γ 90.00

R-Factor (%): 4.56 ***Temperature(K):*** 296 ***Density(g/cm^3):*** 1.529

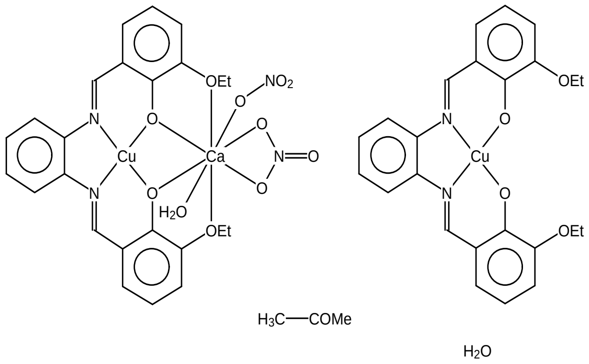

HAMVEY

Reference: S.Mondal, S.Hazra, S.Sarkar, S.Sasmal, S.Mohanta (2011) *J.Mol.Struct.* ,**1004,**204

Formula: C_{48} H_{44} Ba_1 Cu_2 N_6 O_{14},C_1 H_4 O_1

Compound Name: bis(μ_2-2,2'-(1,2-Phenylenebis((nitrilo)methylylidene))bis(6-ethoxyphenolato)-N,N',O,O,O',O',O'',O''')-bis(nitrato-O,O')-barium(ii)-di-copper(ii) methanol solvate

Space Group:	P-1	***Cell:***	***a*** 11.630(2)	***b*** 12.766(2)	***c*** 18.656(3)
Space Group No.:	2	***(Å,°)***	α 81.97(0)	β 72.42(0)	γ 66.78(0)

R-Factor (%): 5.90 ***Temperature(K):*** 296 ***Density(g/cm^3):*** 1.678

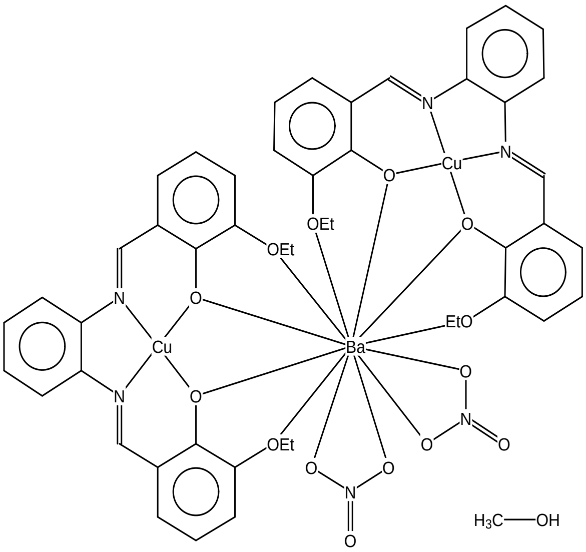

HAMVIC

Reference: S.Mondal, S.Hazra, S.Sarkar, S.Sasmal, S.Mohanta (2011) *J.Mol.Struct.* ,**1004,**204

Formula: C_{24} H_{22} Cu_1 N_4 O_{10} Pb_1,C_1 H_4 O_1

Compound Name: (μ_2-2,2'-(1,2-Phenylenebis((nitrilo)methylylidene))bis(6-ethoxyphenolato)-N,N',O,O,O',O',O'',O''')-bis(nitrato-O,O')-copper(ii)-lead(ii) methanol solvate

Space Group:	P-1	***Cell:***	***a*** 7.203(1)	***b*** 14.092(2)	***c*** 14.301(2)
Space Group No.:	2	***(Å,°)***	α 102.13(0)	β 101.37(0)	γ 103.65(0)

R-Factor (%): 5.41 ***Temperature(K):*** 296 ***Density(g/cm^3):*** 2.068

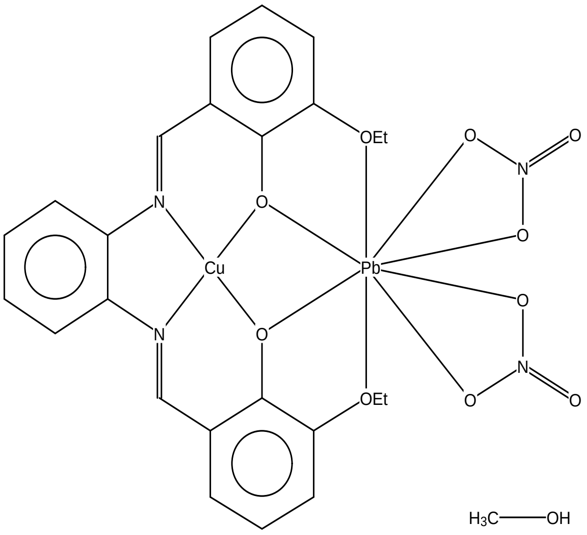

Search: search26 (Tue Dec 2 15:48:50 2014): Hits 233-236

HAMVOI

Reference: S.Mondal, S.Hazra, S.Sarkar, S.Sasmal, S.Mohanta (2011) *J.Mol.Struct.* ,**1004,**204

Formula: $C_{24}\,H_{22}\,Bi_1\,Cu_1\,N_5\,O_{13},C_3\,H_6\,O_1$

Compound Name: (μ_2-2,2'-(1,2-Phenylenebis((nitrilo)methylylidene))bis(6-ethoxyphenolato)-N,N',O,O,O',O',O'',O''')-tris(nitrato-O,O')-bismuth(iii)-copper(ii) acetone solvate

Space Group:	Pna21	***Cell:***	***a*** 9.287(0)	***b*** 16.682(1)	***c*** 20.283(1)
Space Group No.:	33	***(Å,°)***	α 90.00	β 90.00	γ 90.00

R-Factor (%): 2.80 ***Temperature(K):*** 296 ***Density(g/cm^3):*** 1.943

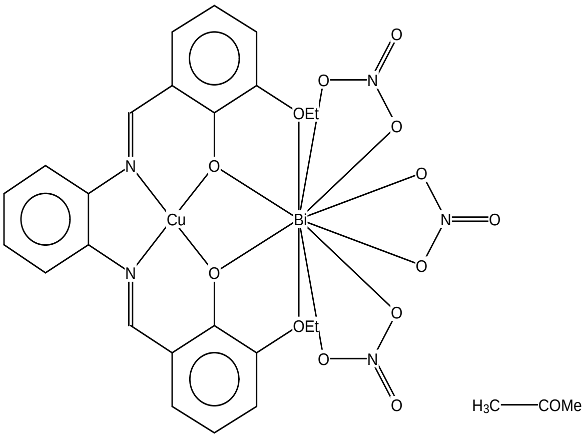

HATLAQ

Reference: M.J.Rodriguez, M.I.Fernandez, A.M.Gonzalez-Noya, M.Maneiro, R.Pedrido, M.Vazquez, B.Donnadieu, M.R.Bermejo (2005) *Z.Anorg.Allg.Chem.* ,**631,**2161

Formula: $C_{20}\,H_{16}\,Co_1\,N_4\,O_8,2(C_3\,H_7\,N_1\,O_1)$

Compound Name: Diaqua-(N,N'-bis(5-nitrosalicylidene)benzene-1,2-diamine)-cobalt(ii) dimethylformamide solvate

Space Group:	P-1	***Cell:***	***a*** 11.179(4)	***b*** 11.558(4)	***c*** 11.982(4)
Space Group No.:	2	***(Å,°)***	α 74.08(3)	β 71.76(3)	γ 87.55(3)

R-Factor (%): 10.97 ***Temperature(K):*** 180 ***Density(g/cm^3):*** 1.518

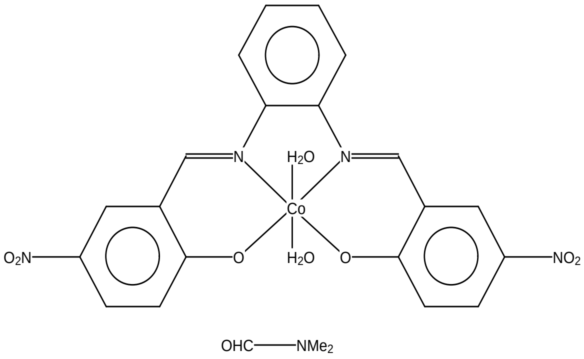

HBACOV

Reference: A.J.Greenwood, K.Henrick, P.G.Owston, P.A.Tasker (1980) *Chem.Commun.* ,88

Formula: $C_{22}\,H_{18}\,N_4\,O_1\,V_1$

Compound Name: (17,18,19,20-Tetrahydro-tribenzo(e,i,m)(1,4,8,11)-tetra-azacyclotetradecinato)-oxo-vanadium(iv)

Space Group:	P21/c	***Cell:***	***a*** 15.387	***b*** 13.918	***c*** 8.253
Space Group No.:	14	***(Å,°)***	α 90.00	β 94.06	γ 90.00

R-Factor (%): 8.50 ***Temperature(K):*** 295 ***Density(g/cm^3):*** 1.527

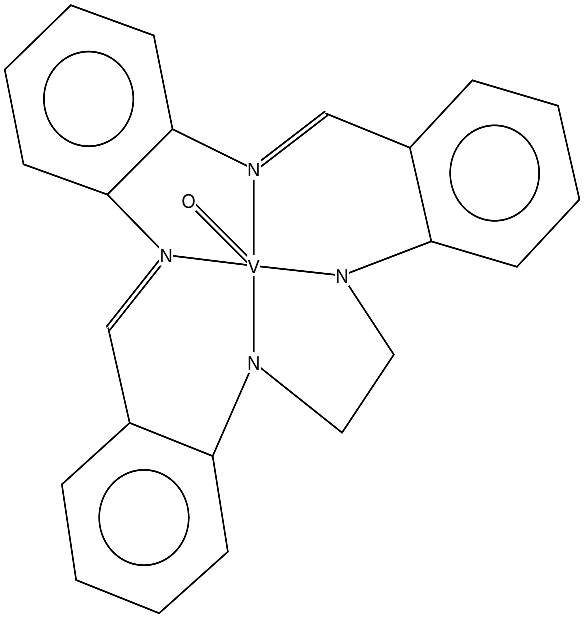

HEHSAO

Reference: F.Azevedo, M.A.A.F.de C.T.Carrondo, B.de Castro, M.Convery, D.Domingues, C.Freire, M.T.Duarte, K.Nielsen, I.C.Santos (1994) *Inorg.Chim.Acta* ,**219,**43

Formula: $C_{20}\,H_{10}\,Cl_4\,N_2\,Ni_1\,O_2$

Compound Name: N,N'-1,2-Benzene-1,2-diyl-bis(3,5-dichlorosalicylideneiminato)-nickel(ii)

Space Group:	P21/n	***Cell:***	***a*** 13.372(5)	***b*** 8.785(2)	***c*** 16.534(5)
Space Group No.:	14	***(Å,°)***	α 90.00	β 101.60(3)	γ 90.00

R-Factor (%): 4.40 ***Temperature(K):*** 295 ***Density(g/cm^3):*** 1.783

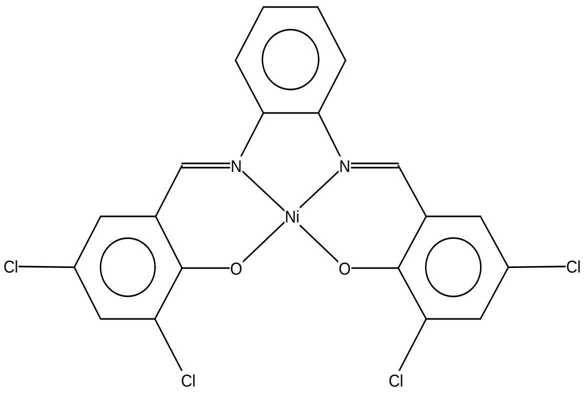

HENWIH

Reference: A.E.Vaughn, D.B.Bassil, C.L.Barnes, S.A.Tucker, P.B.Duval (2006) *J.Am.Chem.Soc.* ,**128,**10656

Formula: $C_{10}\ H_{10}\ Co_1\ ^{1+}, C_{36}\ H_{47}\ N_2\ O_5\ U_1\ ^{1-}$

Compound Name: Cobaltocenium (N,N'-bis(3,5-di-t-butylsalicylidene)benzene-1,2-diamine-N,N',O,O')-hydroxy-dioxo-uranium(vi) unknown solvate

Space Group: P21/c ***Cell:*** ***a*** 17.896(2) ***b*** 19.084(3) ***c*** 17.980(2)
Space Group No.: 14 ***(Å,°)*** α 90.00 β 113.34(0) γ 90.00

R-Factor (%): 4.60 ***Temperature(K):*** 173 ***Density(g/cm³):*** 1.196

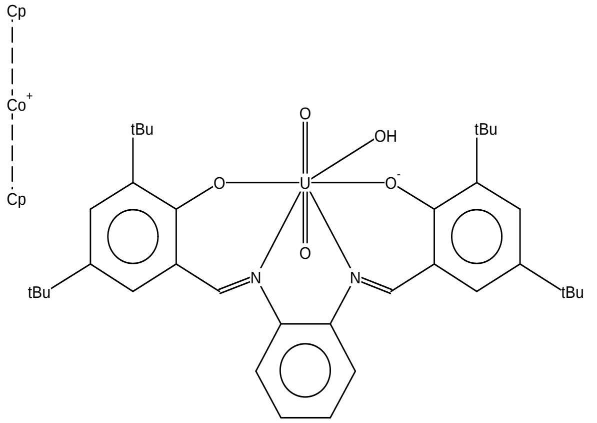

HEQPOI

Reference: D.T.Rosa, V.G.Young, D.Coucouvanis (1998) *Inorg.Chem.* ,**37,**5042

Formula: $C_{48}\ H_{72}\ Mn_1\ N_2\ O_{10}\ ^{1+}, Cl_1\ ^{1-}, C_2\ H_6\ O_1$

Compound Name: Aqua-(4,5-bis(3,5-di-t-butylsalicylideneimine)benzo-18-crown-6)-ethanol-manganese chloride ethanol solvate

Space Group: P-1 ***Cell:*** ***a*** 10.939(0) ***b*** 14.379(0) ***c*** 18.448(0)
Space Group No.: 2 ***(Å,°)*** α 104.96(0) β 104.25(0) γ 99.14(0)

R-Factor (%): 5.88 ***Temperature(K):*** 173 ***Density(g/cm³):*** 1.225

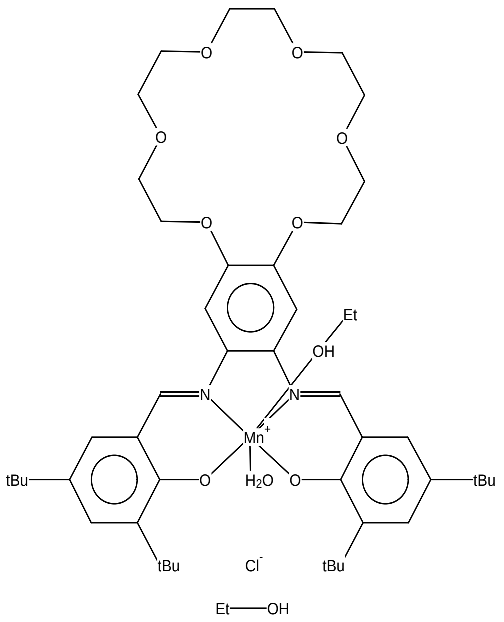

HEQPOI01

Reference: J.D.Pike, D.T.Rosa, D.Coucouvanis (2001) *Eur.J.Inorg.Chem.* ,761

Formula: $C_{48}\ H_{72}\ Mn_1\ N_2\ O_{10}\ ^{1+}, Cl_1\ ^{1-}, C_2\ H_6\ O_1$

Compound Name: Aqua-(4,5-bis(3,5-di-t-butylsalicylideneamino)benzo-18-crown-6)-ethanol-manganese(iii) chloride ethanol solvate

Space Group: P-1 ***Cell:*** ***a*** 10.939(0) ***b*** 14.379(0) ***c*** 18.448(0)
Space Group No.: 2 ***(Å,°)*** α 104.96(0) β 104.25(0) γ 99.14(0)

R-Factor (%): 5.88 ***Temperature(K):*** 173 ***Density(g/cm³):*** 1.225

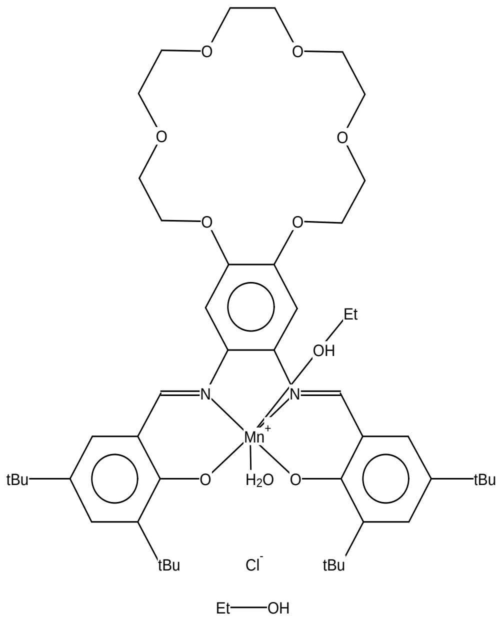

HEQSAX

Reference: R.Hernandez-Molina, A.Mederos, S.Dominguez, P.Gili, C.Ruiz-Perez, A.Castineiras, X.Solans, F.Lloret, J.A.Real (1998) *Inorg.Chem.* ,**37,**5102

Formula: $C_{31}\ H_{32}\ Fe_1\ N_6\ O_4\ ^{1+}, Cl_1\ O_4\ ^{1-}$

Compound Name: (N,N'-3,4-Toluene-bis(3-ethoxysalicylideneiminato))-bis(imidazolyl)-iron(iii) perchlorate

Space Group: P-1 ***Cell:*** ***a*** 10.609(3) ***b*** 10.762(3) ***c*** 15.043(3)
Space Group No.: 2 ***(Å,°)*** α 105.13(2) β 90.67(2) γ 102.09(2)

R-Factor (%): 6.36 ***Temperature(K):*** 295 ***Density(g/cm³):*** 1.454

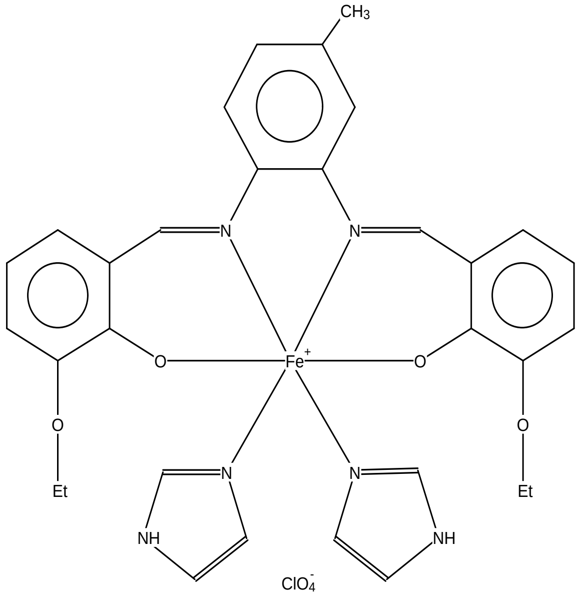

Search: search26 (Tue Dec 2 15:48:50 2014): Hits 241-244

HEQSIF

Reference: R.Hernandez-Molina, A.Mederos, S.Dominguez, P.Gili, C.Ruiz-Perez, A.Castineiras, X.Solans, F.Lloret, J.A.Real (1998) *Inorg.Chem.* ,**37,**5102

Formula: C_{28} H_{25} Cl_3 Fe_1 N_6 Na_1 O_{12}

Compound Name: (μ_2-N,N'-4-Chloro-o-phenylene-bis(3-ethoxysalicylideneiminato))-bis(imidazolyl)-diperchlorato-iron(iii)-sodium

Space Group: C2/c ***Cell:*** ***a*** 14.969(6) ***b*** 17.324(6) ***c*** 13.050(10)
Space Group No.: 15 ***(Å,°)*** α 90.00 β 100.85(6) γ 90.00

R-Factor (%): 5.56 ***Temperature(K):*** 295 ***Density(g/cm³):*** 1.644

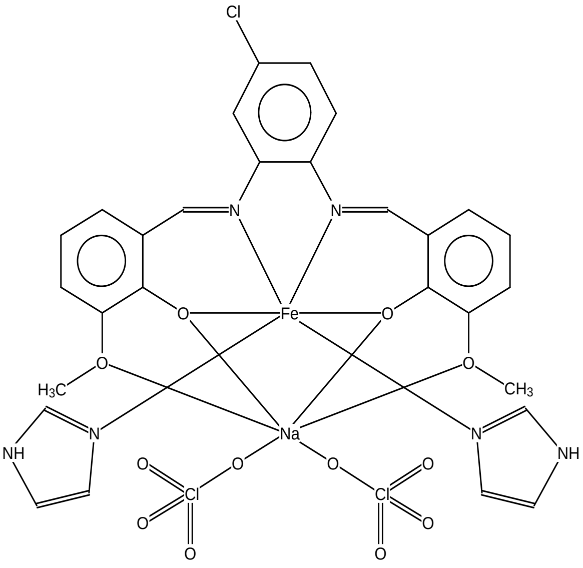

HEQSIF01

Reference: R.Hernandez-Molina, A.Mederos, S.Dominguez, P.Gili, C.Ruiz-Perez, A.Castineiras, X.Solans, F.Lloret, J.A.Real (1998) *Inorg.Chem.* ,**37,**5102

Formula: C_{28} H_{25} Cl_3 Fe_1 N_6 Na_1 O_{12}

Compound Name: (μ_2-N,N'-4-Chloro-o-phenylene-bis(3-ethoxysalicylideneiminato))-bis(imidazolyl)-diperchlorato-iron(iii)-sodium

Space Group: C2/c ***Cell:*** ***a*** 14.914(8) ***b*** 17.493(2) ***c*** 12.780(10)
Space Group No.: 15 ***(Å,°)*** α 90.00 β 102.65(5) γ 90.00

R-Factor (%): 3.56 ***Temperature(K):*** 118 ***Density(g/cm³):*** 1.680

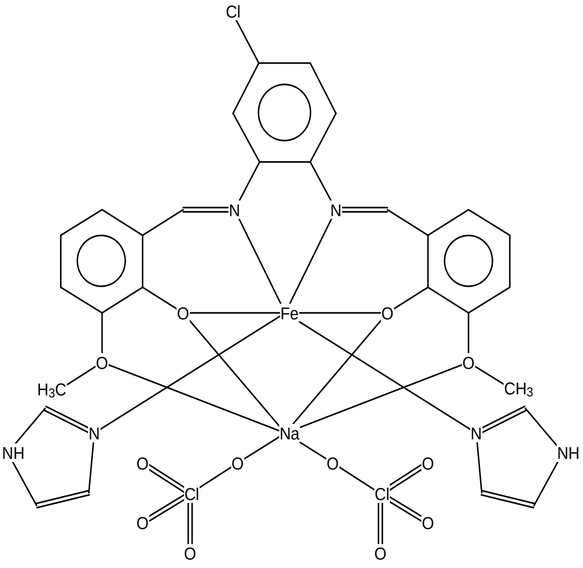

HEVBUF

Reference: C.A.McAuliffe, A.Nabhan, R.G.Pritchard, M.Watkinson, M.Bermejo, A.Sousa (1994) *Acta Crystallogr.,Sect.C:Cryst.Struct.Commun.* ,**50,**1676

Formula: C_{24} H_{26} Mn_1 N_2 O_4 $^{1+}$,C_{24} H_{20} B_1 $^{1-}$

Compound Name: bis(Ethanol)-(N,N'-o-phenylene-bis(salicylideneaminato))-manganese(iii) tetraphenylborate

Space Group: P21/n ***Cell:*** ***a*** 11.967(9) ***b*** 18.125(9) ***c*** 19.502(7)
Space Group No.: 14 ***(Å,°)*** α 90.00 β 106.62(3) γ 90.00

R-Factor (%): 6.69 ***Temperature(K):*** 295 ***Density(g/cm³):*** 1.279

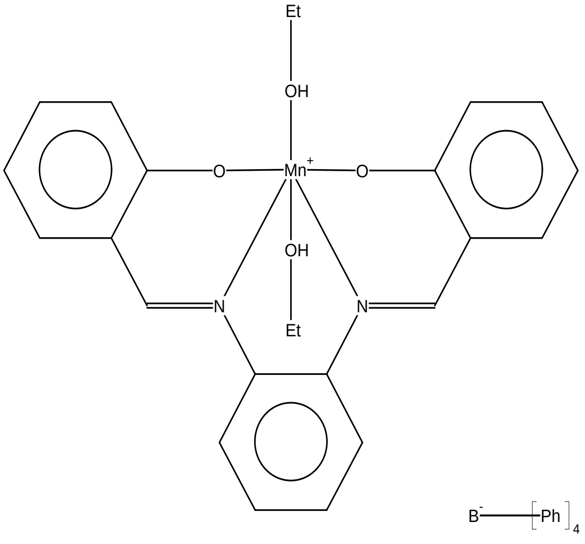

HEWBER

Reference: L.Benisvy, R.Kannappan, Yu-Fei Song, S.Milikisyants, M.Huber, I.Mutikainen, U.Turpeinen, P.Gamez, L.Bernasconi, E.J.Baerends, F.Hartl, J.Reedijk (2007) *Eur.J.Inorg.Chem.* ,637

Formula: C_{38} H_{50} N_2 Ni_1 O_2

Compound Name: (N,N'-bis(3,5-Di-t-butylsalicylidene)-4,5-dimethyl-1,2-phenylenediamine-N,N',O,O')-nickel(ii)

Space Group: P-1 ***Cell:*** ***a*** 9.250(1) ***b*** 14.865(2) ***c*** 14.951(2)
Space Group No.: 2 ***(Å,°)*** α 119.22(1) β 101.65(1) γ 92.31(1)

R-Factor (%): 6.57 ***Temperature(K):*** 173 ***Density(g/cm³):*** 1.198

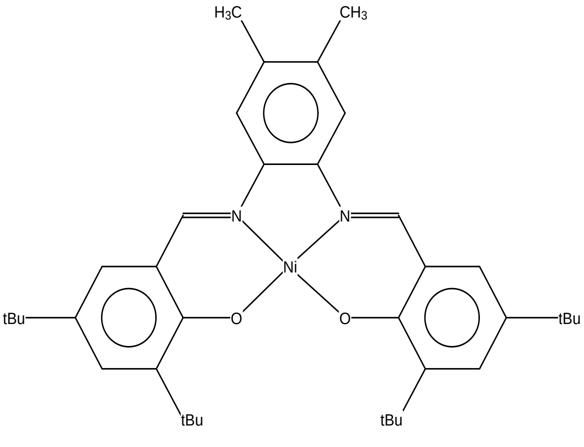

Search: search26 (Tue Dec 2 15:48:50 2014): Hits 245-248

HEYXEP

Reference: M.Cametti, M.Nissinen, A.D.Cort, L.Mandolini, K.Rissanen (2007) *J.Am.Chem.Soc.* ,**129,**3641

Formula: $C_6 H_8 N_1{}^{1+}, C_{34} H_{26} Br_1 N_2 O_6 U_1{}^{1-}, C_2 H_3 N_1$

Compound Name: N-Methylpyridinium bromo-(3,3'-bis(benzyloxy)-N,N'-phenylene-bis(salicylideneaminato))-dioxo-uranium acetonitrile solvate

Space Group:	C2/c	***Cell:***	***a***	35.560(1)	***b***	9.655(0)	***c***	24.101(0)
Space Group No.:	15	***(Å,°)***	α	90.00	β	112.40(0)	γ	90.00

R-Factor (%): 7.29 ***Temperature(K):*** 173 ***Density(g/cm³):*** 1.757

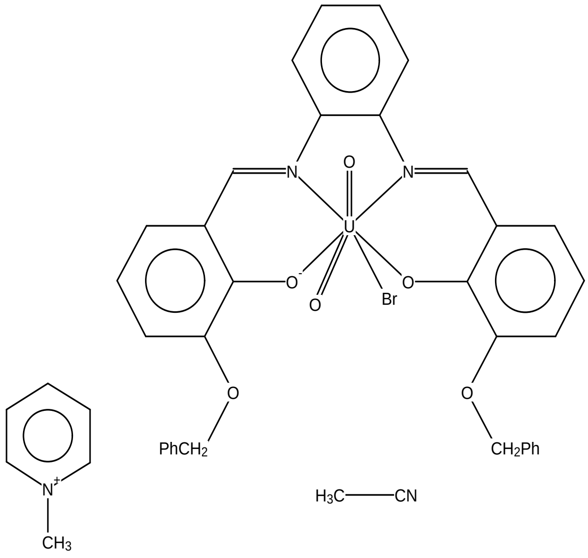

HEYXIT

Reference: M.Cametti, M.Nissinen, A.D.Cort, L.Mandolini, K.Rissanen (2007) *J.Am.Chem.Soc.* ,**129,**3641

Formula: $C_7 H_{16} N_1 O_2{}^{1+}, C_{42} H_{30} Cl_1 N_2 O_6 U_1{}^{1-}, 2(C_2 H_3 N_1)$

Compound Name: Acetylcholinium chloro-(3,3'-bis(2-naphththylmethoxy)-N,N'-phenylene-bis(salicylideneaminato))-dioxo-uranium acetonitrile solvate

Space Group:	P-1	***Cell:***	***a***	12.302(0)	***b***	16.878(0)	***c***	26.486(0)
Space Group No.:	2	***(Å,°)***	α	72.56(0)	β	84.51(0)	γ	75.44(0)

R-Factor (%): 4.67 ***Temperature(K):*** 173 ***Density(g/cm³):*** 1.518

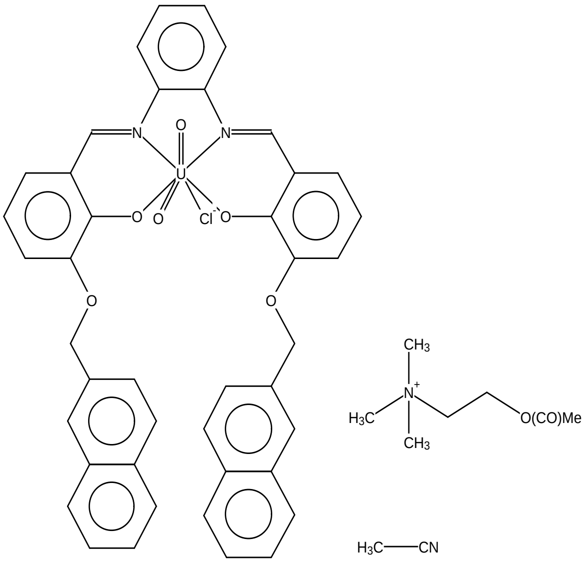

HEYXOZ

Reference: M.Cametti, M.Nissinen, A.D.Cort, L.Mandolini, K.Rissanen (2007) *J.Am.Chem.Soc.* ,**129,**3641

Formula: $4(C_{42} H_{30} Cl_1 N_2 O_6 U_1), 5.33(C_4 H_{12} N_1{}^{1+}), 5.33(Cl_1{}^{1-}), C_2 H_3 N_1$

Compound Name: Tetramethylammonium tetrakis(chloro-(3,3'-bis(2-naphththylmethoxy)-N, N'-phenylene-bis(salicylideneaminato))-dioxo-uranium) chloride acetonitrile solvate

Space Group:	R3c	***Cell:***	***a***	54.160(0)	***b***	54.160(0)	***c***	33.133(0)
Space Group No.:	161	***(Å,°)***	α	90.00	β	90.00	γ	120.00

R-Factor (%): 4.51 ***Temperature(K):*** 173 ***Density(g/cm³):*** 1.546

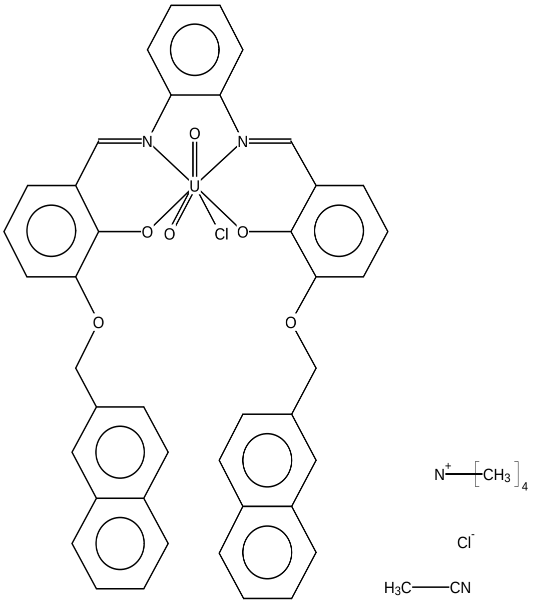

HEYXUF

Reference: M.Cametti, M.Nissinen, A.D.Cort, L.Mandolini, K.Rissanen (2007) *J.Am.Chem.Soc.* ,**129,**3641

Formula: $C_4 H_{12} N_1{}^{1+}, C_{20} H_{14} Cl_1 N_2 O_4 U_1{}^{1-}, 0.33(C_2 H_3 N_1)$

Compound Name: Tetramethylammonium (chloro-(N,N'-phenylene-bis(salicylideneaminato))-dioxo-uranium) acetonitrile solvate

Space Group:	R-3	***Cell:***	***a***	22.598(0)	***b***	22.598(0)	***c***	25.731(0)
Space Group No.:	148	***(Å,°)***	α	90.00	β	90.00	γ	120.00

R-Factor (%): 4.99 ***Temperature(K):*** 173 ***Density(g/cm³):*** 1.858

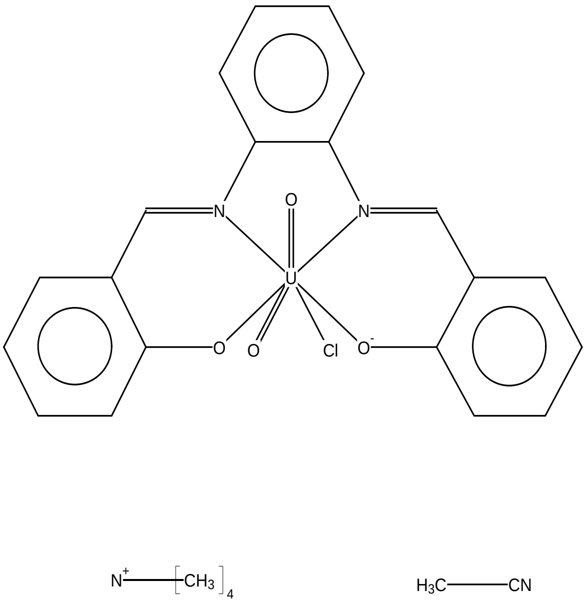

Search: search26 (Tue Dec 2 15:48:50 2014): Hits 249-252

HEYYAM

Reference: M.Cametti, M.Nissinen, A.D.Cort, L.Mandolini, K.Rissanen (2007) *J.Am.Chem.Soc.* ,**129,**3641

Formula: $C_{44}\ H_{33}\ N_3\ O_6\ U_1,C_2\ H_3\ N_1$

Compound Name: Acetonitrile-(3,3'-bis(2-naphththylmethoxy)-N,N'-phenylene-bis(salicylideneaminato))-dioxo-uranium acetonitrile solvate

Space Group:	P-1	***Cell:***	***a***	11.821(0)	***b***	12.861(0)	***c***	14.846(0)
Space Group No.:	2	***(Å,°)***	α	73.70(0)	β	88.33(0)	γ	64.80(0)

R-Factor (%): 3.56 ***Temperature(K):*** 173 ***Density(g/cm^3):*** 1.667

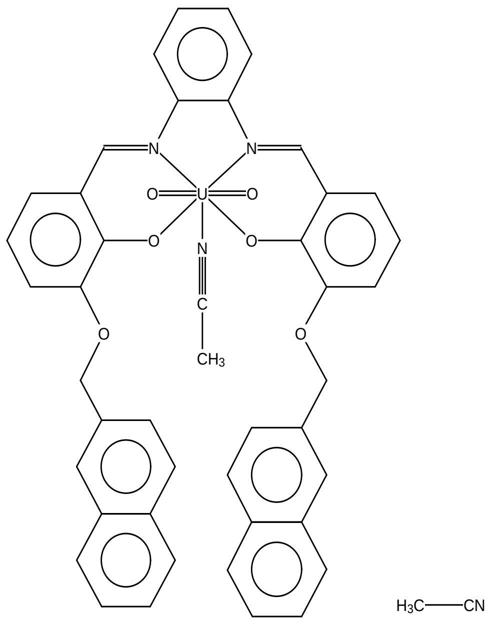

HIHQUK

Reference: D.K.Dey, M.K.Das, H.Noth (1999) *Z.Naturforsch.,B:Chem.Sci.* ,**54,**145

Formula: $C_{34}\ H_{28}\ N_2\ O_4\ Sn_1,0.5(C_1\ H_1\ Cl_3)$

Compound Name: (N,N'-1,2-Phenylene-bis(3-methoxysalicylideneiminato))-diphenyl-tin(iv) chloroform solvate

Space Group:	C2/c	***Cell:***	***a***	21.458(0)	***b***	16.547(0)	***c***	19.089(0)
Space Group No.:	15	***(Å,°)***	α	90.00	β	113.55(0)	γ	90.00

R-Factor (%): 9.60 ***Temperature(K):*** 193 ***Density(g/cm^3):*** 1.511

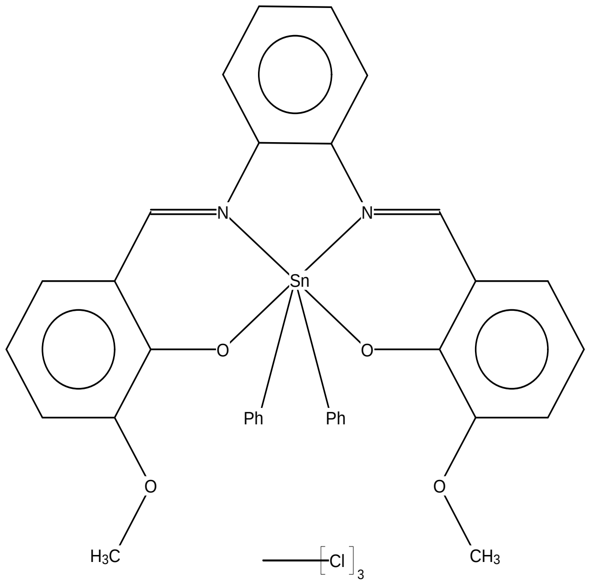

HIHWOK

Reference: D.K.Dey, M.K.Das, H.Noth (1999) *Z.Naturforsch.,B:Chem.Sci.* ,**54,**145

Formula: $C_{24}\ H_{24}\ N_2\ O_4\ Sn_1$

Compound Name: (N,N'-1,2-Phenylene-bis(3-methoxysalicylideneiminato))-dimethyl-tin(iv)

Space Group:	P21/n	***Cell:***	***a***	7.801(0)	***b***	16.280(2)	***c***	16.928(2)
Space Group No.:	14	***(Å,°)***	α	90.00	β	92.14(0)	γ	90.00

R-Factor (%): 1.95 ***Temperature(K):*** 200 ***Density(g/cm^3):*** 1.618

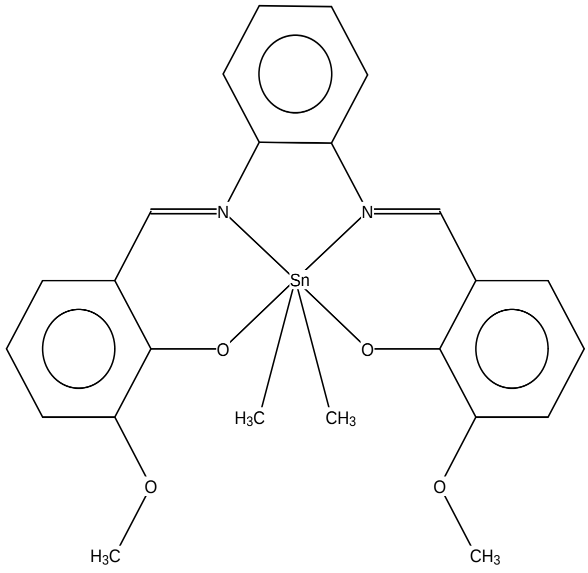

HIQXOV

Reference: N.E.Eltayeb, S.G.Teoh, S.Chantrapromma, Hoong-Kun Fun, K.Ibrahim (2007) *Acta Crystallogr.,Sect.E:Struct.Rep.Online* ,**63,**m3193

Formula: $C_{23}\ H_{22}\ Cl_1\ Mn_1\ N_2\ O_5$

Compound Name: Chloro-(4,4'-dimethoxy-2,2'-(1,2-phenylenebis(nitrilomethylidyne)) diphenolato)-methanol-manganese(iii)

Space Group:	P21/c	***Cell:***	***a***	12.211(0)	***b***	7.550(0)	***c***	22.420(1)
Space Group No.:	14	***(Å,°)***	α	90.00	β	90.40(0)	γ	90.00

R-Factor (%): 4.42 ***Temperature(K):*** 100 ***Density(g/cm^3):*** 1.597

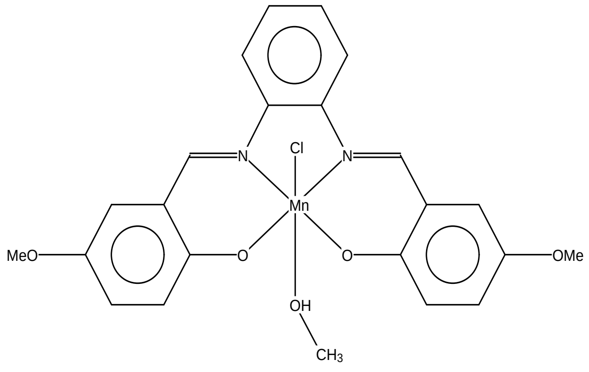

HISPII

Reference: T.Dube, S.Gambarotta, G.Yap (1998) *Organometallics* ,**17,** 3967

Formula: $C_{144} H_{188} N_8 O_{12} Sm_4,4(C_7 H_8)$

Compound Name: tetrakis((μ_3-Hydroxo)-(phenylene-bis(3,5-di-t-butylsalicylidene) dimethyldiamidato)-samarium) toluene solvate

Space Group:	I41/a	***Cell:***	***a*** 31.491(0)	***b*** 31.491(0)	***c*** 16.203(3)
Space Group No.:	88	***(Å,°)***	α 90.00	β 90.00	γ 90.00

R-Factor (%): 5.01 ***Temperature(K):*** 173 ***Density(g/cm³):*** 1.320

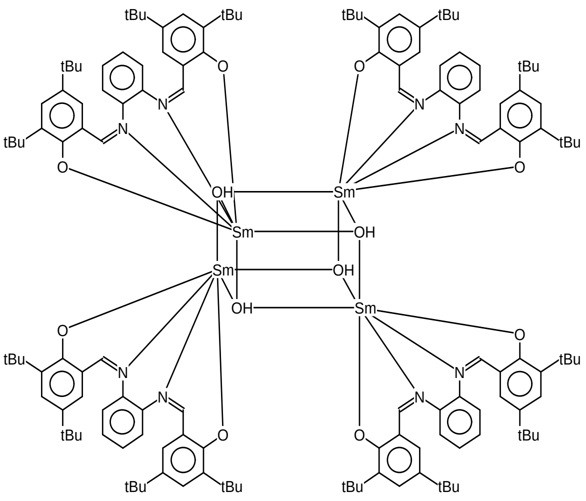

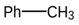

HITWAI

Reference: A.Hori, T.Ozawa, H.Yoshida, Y.Imori, Y.Kuribayashi, E.Nakano, N.Azuma (1998) *Inorg.Chim.Acta* ,**281,**207

Formula: $C_{20} H_{14} Cr_1 N_3 O_2,C_2 H_3 N_1$

Compound Name: (N,N'-bis(Salicylidene)-o-phenylenediamine)-nitrido-chromium(v) acetonitrile solvate

Space Group:	C2/c	***Cell:***	***a*** 26.840(10)	***b*** 6.671(2)	***c*** 24.026(5)
Space Group No.:	15	***(Å,°)***	α 90.00	β 112.83(2)	γ 90.00

R-Factor (%): 7.47 ***Temperature(K):*** 295 ***Density(g/cm³):*** 1.412

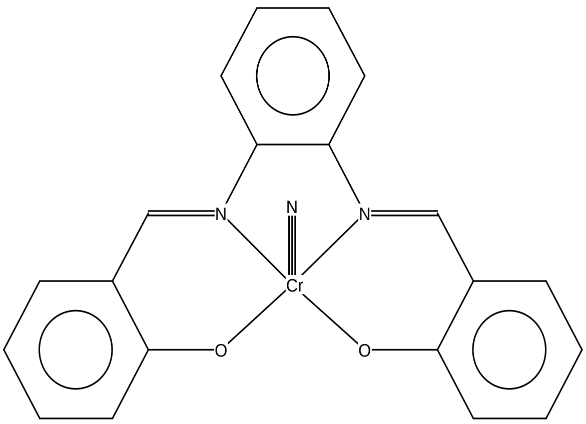

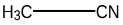

HUHVAI

Reference: Ping Liu, Xiu-Juan Feng, Ren He (2010) *Tetrahedron* ,**66,** 631

Formula: $C_{36} H_{46} N_2 O_2 Pd_1$

Compound Name: (2,2'-(1,2-Phenylenebis((nitrilo)methylylidene))bis(4,6-di-t-butylphenolato))-palladium

Space Group:	P-1	***Cell:***	***a*** 9.278(1)	***b*** 13.059(1)	***c*** 14.572(1)
Space Group No.:	2	***(Å,°)***	α 105.95(0)	β 94.24(0)	γ 96.84(0)

R-Factor (%): 3.87 ***Temperature(K):*** 273 ***Density(g/cm³):*** 1.279

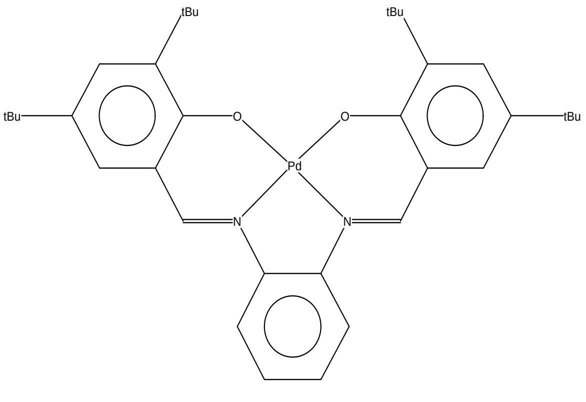

HUPWOF

Reference: M.M.Belmonte, E.C.Escudero-Adan, J.Benet-Buchholz, A.W.Kleij (2009) *Eur.J.Inorg.Chem.* ,5307

Formula: $3(C_{86} H_{108} N_6 O_6 Zn_3),6(C_2 H_3 N_1)$

Compound Name: bis(μ_2-6-Methylpyridin-2-ylmethyloxy)-bis(N,N'-bis(3,5-di-t-butylsalicylidene)-1,2-phenylenediamine)-tri-zinc(ii) acetonitrile solvate

Space Group:	P21/c	***Cell:***	***a*** 23.205(0)	***b*** 60.695(1)	***c*** 18.418(0)
Space Group No.:	14	***(Å,°)***	α 90.00	β 101.71(0)	γ 90.00

R-Factor (%): 5.50 ***Temperature(K):*** 100 ***Density(g/cm³):*** 1.255

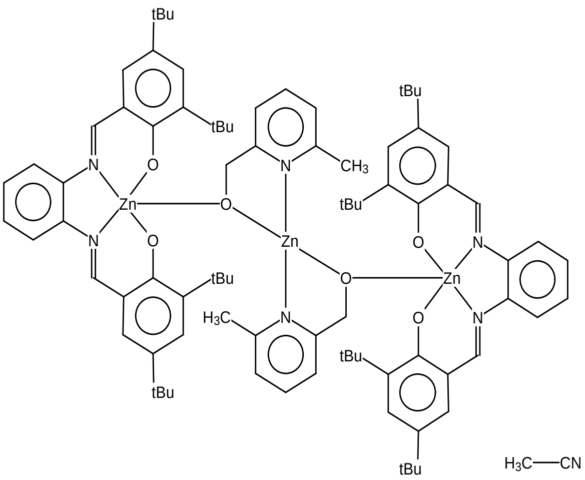

HUPWUL

Reference: M.M.Belmonte, E.C.Escudero-Adan, J.Benet-Buchholz, A.W.Kleij (2009) *Eur.J.Inorg.Chem.* ,5307

Formula: $C_{70} H_{76} N_6 O_8 Zn_3,2(C_6 H_6)$

Compound Name: bis(μ_2-6-(Hydroxymethyl)pyridin-2-ylmethyloxy)-bis(N,N'-bis(3-t-butylsalicylidene)-1,2-phenylenediamine)-tri-zinc(ii) benzene solvate

Space Group:	P21/n	***Cell:***	***a*** 20.009(1)	***b*** 17.028(2)	***c*** 22.617(2)		
Space Group No.:	14	***(Å,°)***	α 90.00	β 109.01	γ 90.00		
R-Factor (%):	6.80	***Temperature(K):***	100	***Density(g/cm³):***	1.351		

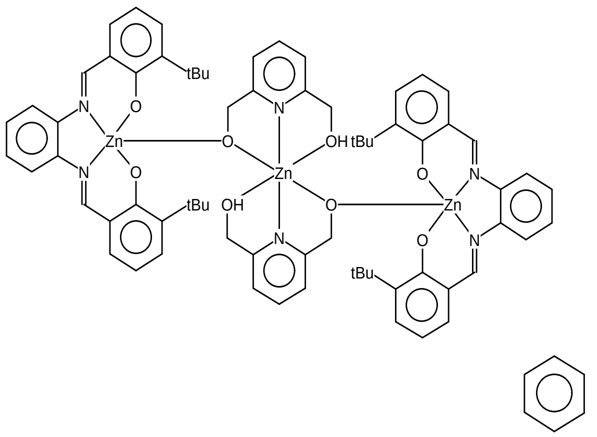

HUPXAS

Reference: M.M.Belmonte, E.C.Escudero-Adan, J.Benet-Buchholz, A.W.Kleij (2009) *Eur.J.Inorg.Chem.* ,5307

Formula: $C_{94} H_{112} N_6 O_8 Zn_3,C_2 H_3 N_1$

Compound Name: bis(μ_2-6-Methylpyridin-2-ylmethyloxy)-bis(N,N'-bis(3,5-di-t-butylsalicylidene)-2,3-naphthylenediamine)-tri-zinc(ii) acetonitrile solvate

Space Group:	P-1	***Cell:***	***a*** 15.388(1)	***b*** 16.730(1)	***c*** 18.435(1)		
Space Group No.:	2	***(Å,°)***	α 79.70(0)	β 88.98(0)	γ 68.22(0)		
R-Factor (%):	7.38	***Temperature(K):***	100	***Density(g/cm³):***	1.297		

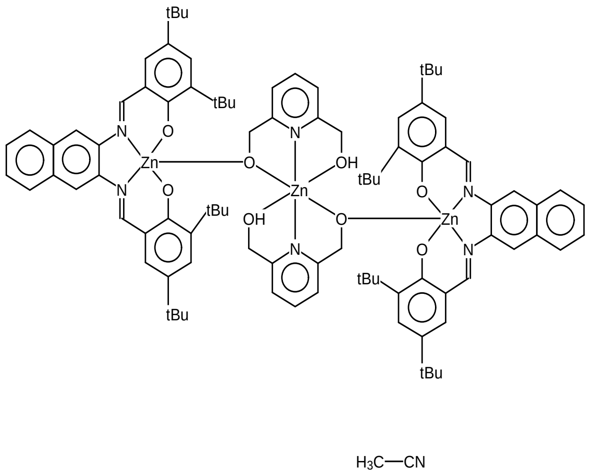

HUPXEW

Reference: M.M.Belmonte, E.C.Escudero-Adan, J.Benet-Buchholz, A.W.Kleij (2009) *Eur.J.Inorg.Chem.* ,5307

Formula: $C_{78} H_{92} N_6 O_8 Zn_3,3.75(C_2 H_3 N_1)$

Compound Name: bis(μ_2-6-Methylpyridin-2-ylmethyloxy)-bis(N-(3,5-di-t-butylsalicylidene)-N'-(3-t-butylsalicylidene)-1,2-phenylenediamine)-tri-zinc(ii) acetonitrile solvate

Space Group:	C2/c	***Cell:***	***a*** 37.807(9)	***b*** 22.190(5)	***c*** 22.298(5)		
Space Group No.:	15	***(Å,°)***	α 90.00	β 90.64(0)	γ 90.00		
R-Factor (%):	7.12	***Temperature(K):***	100	***Density(g/cm³):***	1.130		

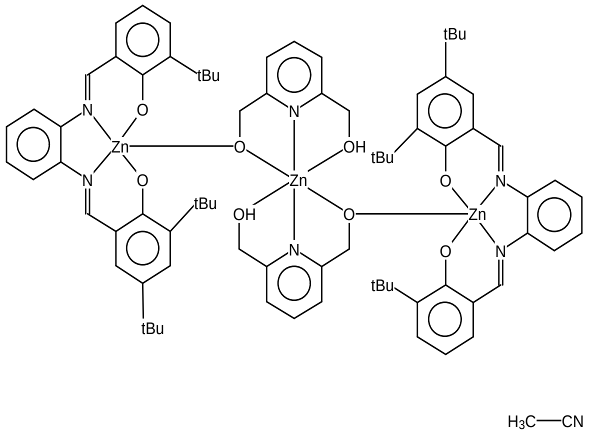

HUPXIA

Reference: M.M.Belmonte, E.C.Escudero-Adan, J.Benet-Buchholz, A.W.Kleij (2009) *Eur.J.Inorg.Chem.* ,5307

Formula: $C_{42} H_{53} N_3 O_3 Zn_1$

Compound Name: (N,N'-bis(3,5-di-t-butylsalicylidene)-1,2-phenylenediamine)-(pyridin-2-ylmethanol)-zinc

Space Group:	P21/c	***Cell:***	***a*** 14.444(0)	***b*** 10.417(0)	***c*** 25.729(0)		
Space Group No.:	14	***(Å,°)***	α 90.00	β 95.23(0)	γ 90.00		
R-Factor (%):	3.49	***Temperature(K):***	100	***Density(g/cm³):***	1.229		

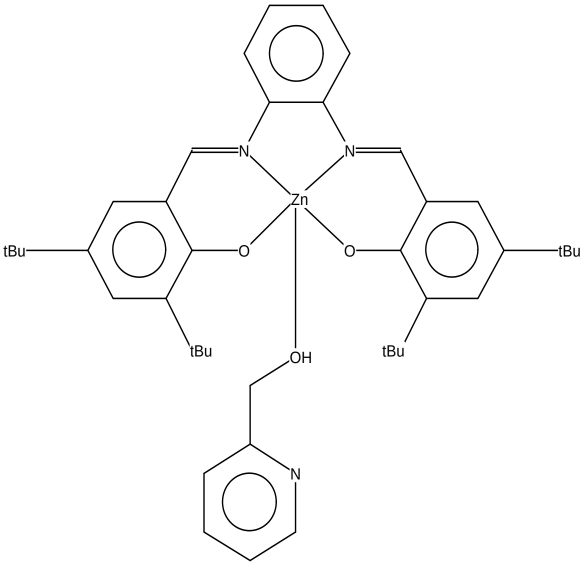

HUPXOG

Reference: M.M.Belmonte, E.C.Escudero-Adan, J.Benet-Buchholz, A.W.Kleij (2009) *Eur.J.Inorg.Chem.* ,5307

Formula: $C_{43} H_{55} N_3 O_3 Zn_1$

Compound Name: (N,N'-bis(3,5-di-t-butylsalicylidene)-1,2-phenylenediamine)-(pyridin-2-ylethanol)-zinc

**Space Group:** P21/c	**Cell:**	**a** 12.985(0)	**b** 25.135(0)	**c** 11.941(0)
**Space Group No.:** 14	**(Å,°)**	α 90.00	β 90.29(0)	γ 90.00

R-Factor (%): 4.04 **Temperature(K):** 100 **Density(g/cm³):** 1.240

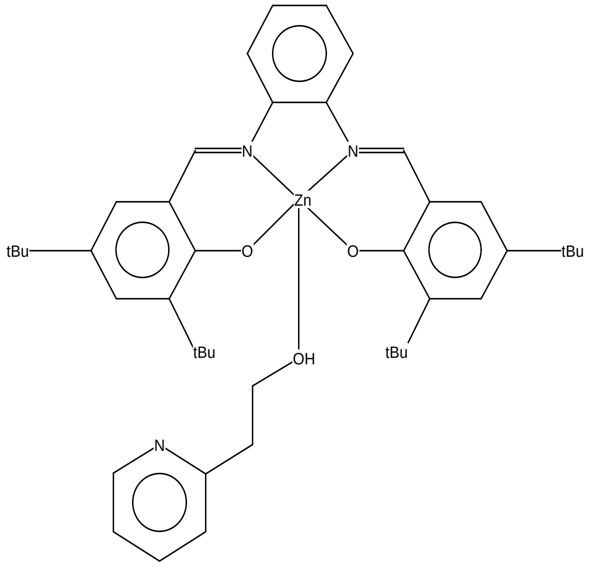

HUPXUM

Reference: M.M.Belmonte, E.C.Escudero-Adan, J.Benet-Buchholz, A.W.Kleij (2009) *Eur.J.Inorg.Chem.* ,5307

Formula: $C_{42} H_{52} Br_1 N_3 O_3 Zn_1,C_{36} H_{48} N_2 O_3 Zn_1,C_2 H_3 N_1$

Compound Name: (N,N'-bis(3,5-di-t-butylsalicylidene)-1,2-phenylenediamine)-(6-bromopyridin-2-ylmethanol)-zinc aqua-(N,N'-bis(3,5-di-t-butylsalicylidene)-1,2-phenylenediamine)-zinc acetonitrile solvate

**Space Group:** P21/c	**Cell:**	**a** 13.982(0)	**b** 19.742(0)	**c** 27.942(1)
**Space Group No.:** 14	**(Å,°)**	α 90.00	β 102.51(0)	γ 90.00

R-Factor (%): 5.77 **Temperature(K):** 100 **Density(g/cm³):** 1.284

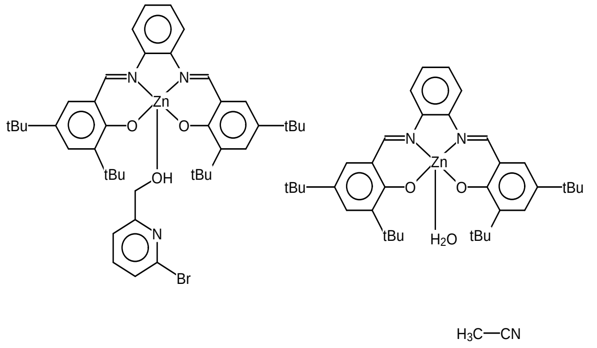

HUPYAT

Reference: M.M.Belmonte, E.C.Escudero-Adan, J.Benet-Buchholz, A.W.Kleij (2009) *Eur.J.Inorg.Chem.* ,5307

Formula: $C_{43} H_{55} N_3 O_2 Zn_1$

Compound Name: (2-Ethylpyridine)-(N,N'-bis(3,5-di-t-butylsalicylidene)-1,2-phenylenediamine)-zinc

**Space Group:** P-1	**Cell:**	**a** 11.998(1)	**b** 12.362(0)	**c** 13.259(0)
**Space Group No.:** 2	**(Å,°)**	α 87.31(0)	β 83.15(0)	γ 76.46(0)

R-Factor (%): 2.98 **Temperature(K):** 100 **Density(g/cm³):** 1.245

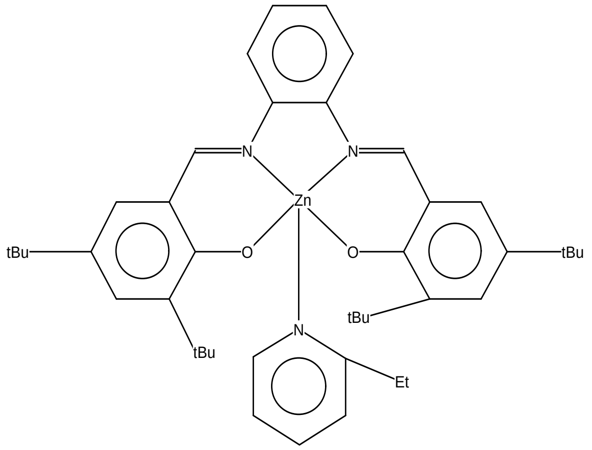

HURWOH

Reference: M.Schley, S.Fritzsche, P.Lonnecke, E.Hey-Hawkins (2010) *Dalton Trans.* ,**39,**4090

Formula: $C_{37} H_{46} N_2 Ni_1 O_4,2(C_4 H_8 O_2)$

Compound Name: (3,4-bis((3,5-di-t-butylsalicylidene)amino)benzoic acid)-nickel(ii) 1,4-dioxane solvate

**Space Group:** P21/c	**Cell:**	**a** 17.476(1)	**b** 14.909(1)	**c** 19.130(1)
**Space Group No.:** 14	**(Å,°)**	α 90.00	β 115.32(0)	γ 90.00

R-Factor (%): 3.62 **Temperature(K):** 208 **Density(g/cm³):** 1.206

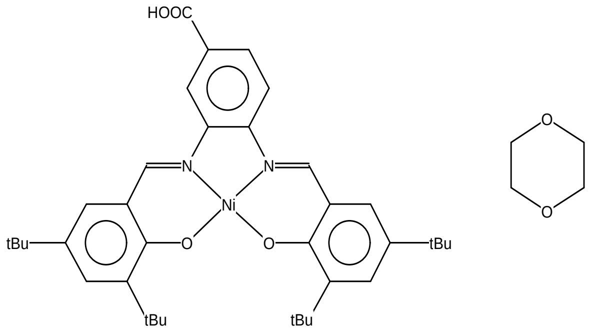

HURWUN

Reference: M.Schley, S.Fritzsche, P.Lonnecke, E.Hey-Hawkins (2010) *Dalton Trans.* ,**39,**4090

Formula: $C_{39} H_{49} Cl_1 Cr_1 N_3 O_4,6(C_2 H_3 N_1)$

Compound Name: (acetonitrile)-(3,4-bis((3,5-di-t-butylsalicylidene)amino)benzoic acid)-chloro-chromium(iii) acetonitrile solvate

Space Group: P21/c ***Cell:*** ***a*** 15.512(1) ***b*** 16.751(1) ***c*** 21.455(1)
Space Group No.: 14 ***(Å,°)*** α 90.00 β 101.41(0) γ 90.00

R-Factor (%): 5.72 ***Temperature(K):*** 213 ***Density(g/cm³):*** 1.164

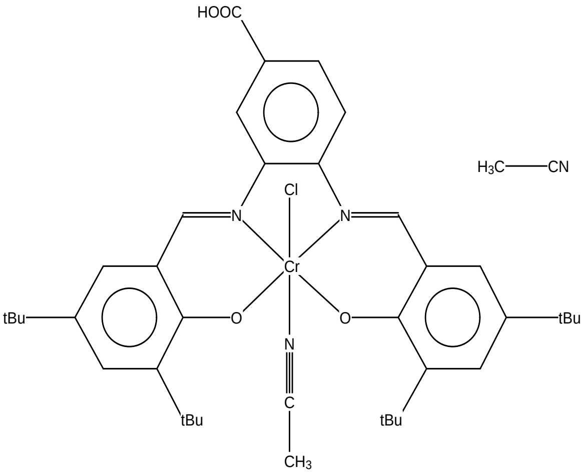

HURXAU

Reference: M.Schley, S.Fritzsche, P.Lonnecke, E.Hey-Hawkins (2010) *Dalton Trans.* ,**39,**4090

Formula: $C_{37} H_{46} Cl_2 Mo_1 N_2 O_4,4(C_2 H_3 N_1)$

Compound Name: (3,4-bis((3,5-di-t-butylsalicylidene)amino)benzoic acid)-dichloro-molybdenum(iv) acetonitrile solvate

Space Group: P21/c ***Cell:*** ***a*** 15.129(2) ***b*** 16.508(3) ***c*** 20.585(3)
Space Group No.: 14 ***(Å,°)*** α 90.00 β 106.59(0) γ 90.00

R-Factor (%): 7.97 ***Temperature(K):*** 218 ***Density(g/cm³):*** 1.232

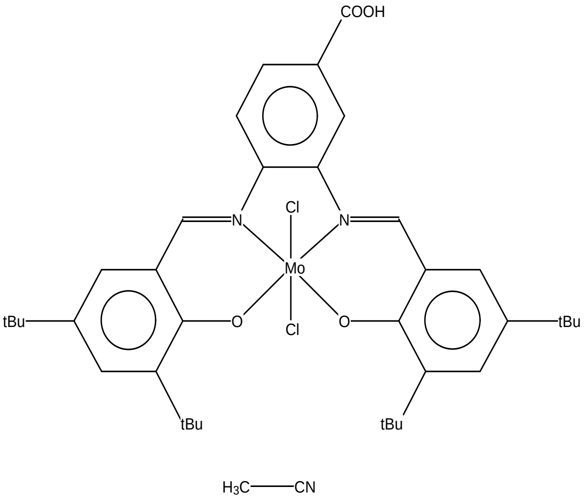

HURXIC

Reference: M.Schley, S.Fritzsche, P.Lonnecke, E.Hey-Hawkins (2010) *Dalton Trans.* ,**39,**4090

Formula: $C_{134} H_{178} B_4 Fe_2 La_2 N_{28} O_9,3.5(C_7 H_8)$

Compound Name: bis(μ_2-3,4-bis((3,5-di-t-butylsalicylidene)amino)benzoato)-(μ_2-oxo)-tetrakis(hydrogen tris(3,5-dimethylpyrazol-1-yl)borate)-di-iron-di-lanthanum toluene solvate

Space Group: P21/c ***Cell:*** ***a*** 19.289(1) ***b*** 24.006(1) ***c*** 36.938(2)
Space Group No.: 14 ***(Å,°)*** α 90.00 β 101.82(0) γ 90.00

R-Factor (%): 6.53 ***Temperature(K):*** 213 ***Density(g/cm³):*** 1.222

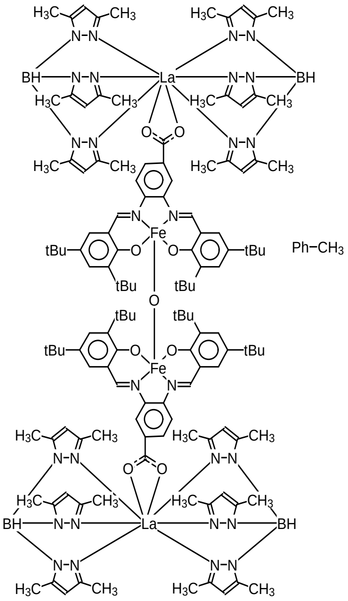

HURXOI

Reference: M.Schley, S.Fritzsche, P.Lonnecke, E.Hey-Hawkins (2010) *Dalton Trans.* ,**39,**4090

Formula: $C_{67} H_{89} B_2 La_1 N_{14} Ni_1 O_4,0.5(C_6 H_{14}),C_4 H_8 O_2$

Compound Name: (μ_2-3,4-bis((3,5-di-t-butylsalicylidene)amino)benzoato)-bis(hydrogen tris(3,5-dimethylpyrazol-1-yl)borate)-lanthanum-nickel 1,4-dioxane hexane solvate

Space Group: C2/c ***Cell:*** ***a*** 23.428(1) ***b*** 36.338(1) ***c*** 21.033(0)
Space Group No.: 15 ***(Å,°)*** α 90.00 β 112.11(0) γ 90.00

R-Factor (%): 9.55 ***Temperature(K):*** 180 ***Density(g/cm³):*** 1.205

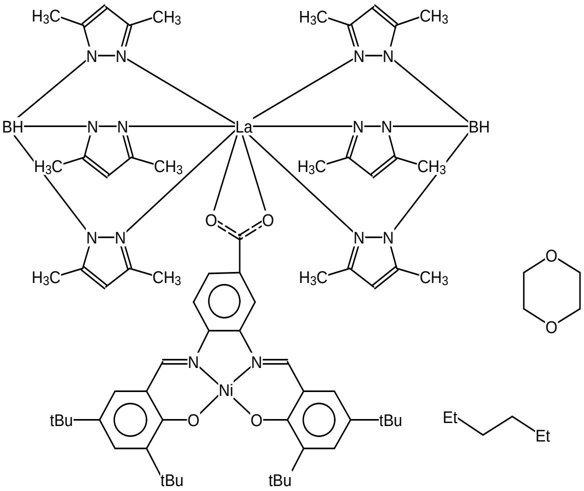

HURXUO

Reference: M.Schley, S.Fritzsche, P.Lonnecke, E.Hey-Hawkins (2010) *Dalton Trans.* ,**39,**4090

Formula: C_{67} H_{89} B_2 Cu_1 La_1 N_{14} O_4,C_6 H_{14}

Compound Name: (μ_2-3,4-bis((3,5-di-t-butylsalicylidene)amino)benzoato)-bis(hydrogen tris(3,5-dimethylpyrazol-1-yl)borate)-copper-lanthanum hexane solvate

Space Group:	P21/c	***Cell:***	***a***	16.560(1)	***b***	22.667(1)	***c***	21.813(1)
Space Group No.:	14	***(Å,°)***	α	90.00	β	107.79(0)	γ	90.00

R-Factor (%): 3.66 ***Temperature(K):*** 213 ***Density(g/cm^3):*** 1.248

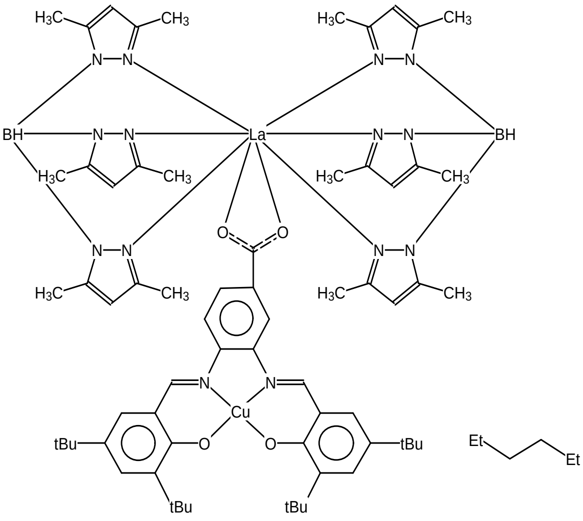

HURYOJ

Reference: M.Schley, S.Fritzsche, P.Lonnecke, E.Hey-Hawkins (2010) *Dalton Trans.* ,**39,**4090

Formula: C_{42} H_{54} Cl_1 Cr_1 N_4 O_4,C_4 H_8 O_2

Compound Name: (3,4-bis((3,5-di-t-butylsalicylidene)amino)benzoic acid)-chloro-(3,5-dimethyl-1H-pyrazole)-chromium(iii) 1,4-dioxane solvate

Space Group:	P-1	***Cell:***	***a***	9.525(1)	***b***	15.812(3)	***c***	17.539(3)
Space Group No.:	2	***(Å,°)***	α	66.03(1)	β	79.98(1)	γ	76.19(1)

R-Factor (%): 4.75 ***Temperature(K):*** 150 ***Density(g/cm^3):*** 1.215

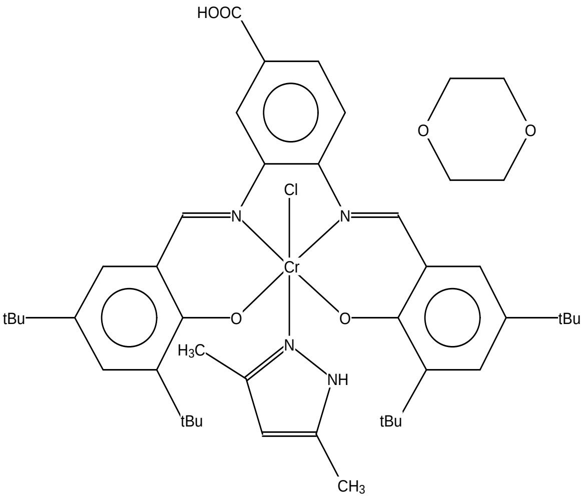

IBIWAT

Reference: Xue Gong, Ying-Ying Ge, Mao Fang, Zhi-Gang Gu, Sheng-Run Zheng, Wei-Shan Li, She-Jun Hu, Shu-Bin Li, Yue-Peng Cai (2011) *CrystEngComm* ,**13,**6911

Formula: C_{25} H_{25} Cd_1 N_5 O_{11} Zn_1

Compound Name: (μ_2-2,2'-(1,2-Phenylenebis(nitrilomethylylidene))-bis(6-methoxyphenolato))-(dimethylformamide)-bis(nitrato-O,O')-cadmium-zinc

Space Group:	P21/c	***Cell:***	***a***	9.931(0)	***b***	18.862(1)	***c***	15.025(1)
Space Group No.:	14	***(Å,°)***	α	90.00	β	97.40(0)	γ	90.00

R-Factor (%): 2.81 ***Temperature(K):*** 298 ***Density(g/cm^3):*** 1.783

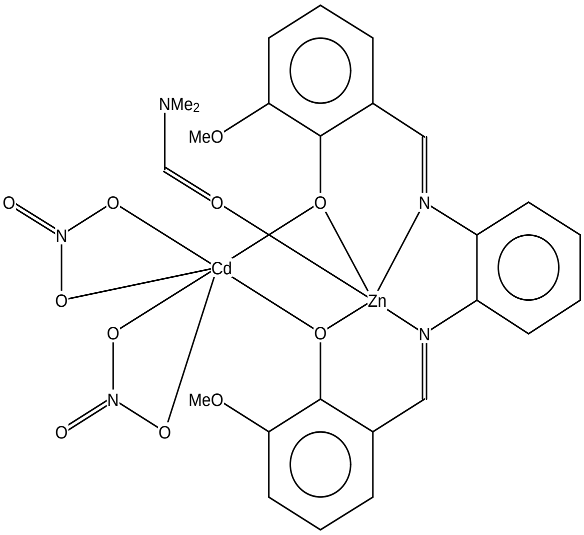

ICATAI

Reference: D.J.Darensbourg, R.M.Mackiewicz, J.L.Rodgers, C.C.Fang, D.R.Billodeaux, J.H.Reibenspies (2004) *Inorg.Chem.* ,**43,** 6024

Formula: C_{40} H_{54} Cl_1 Cr_1 N_2 O_3,3(C_4 H_8 O_1)

Compound Name: (N,N'-bis(3,5-di-t-butylsalicylidenato)-1,2-phenylenediamine)-chloro-(tetrahydrofuran)-chromium(iii) tetrahydrofuran solvate

Space Group:	P21/c	***Cell:***	***a***	16.253(16)	***b***	26.260(30)	***c***	11.705(12)
Space Group No.:	14	***(Å,°)***	α	90.00	β	98.16(1)	γ	90.00

R-Factor (%): 6.44 ***Temperature(K):*** 110 ***Density(g/cm^3):*** 1.229

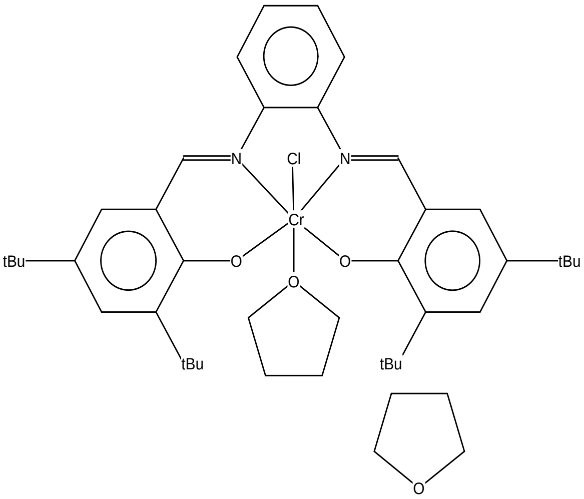

ICUXOV

Reference: M.A.Vazquez-Fernandez, M.R.Bermejo, M.I.Fernandez-Garcia, G.Gonzalez-Riopedre, M.J.Rodriguez-Douton, M.Maneiro (2011) *J.Inorg.Biochem.* ,**105,**1538

Formula: $C_{23} H_{24} Mn_1 N_2 O_6{}^{1+}, C_{22} H_{22} Mn_1 N_2 O_6{}^{1+}, C_1 H_4 O_1, 2(N_1 O_3{}^{1-})$

Compound Name: (2,2'-(1,2-phenylenebis((nitrilo)methylylidene))bis(6-methoxyphenolato))-aqua-methanol-manganese(iii) (2,2'-(1,2-phenylenebis((nitrilo) methylylidene))bis (6-methoxyphenolato))-diaqua-manganese(iii) dinitrate methanol solvate

Space Group:	P-1	***Cell:***	***a*** 11.589(5)	***b*** 13.820(5)	***c*** 16.115(5)
Space Group No.:	2	***(Å,°)***	α 87.90(0)	β 81.87(0)	γ 67.38(0)

R-Factor (%): 5.27 ***Temperature(K):*** 120 ***Density(g/cm^3):*** 1.550

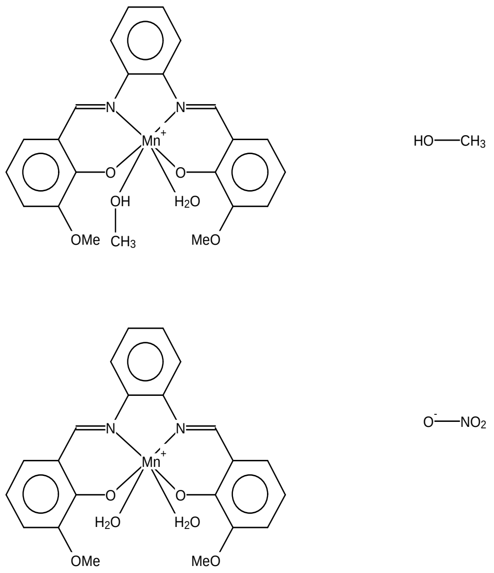

ICUXUB

Reference: M.A.Vazquez-Fernandez, M.R.Bermejo, M.I.Fernandez-Garcia, G.Gonzalez-Riopedre, M.J.Rodriguez-Douton, M.Maneiro (2011) *J.Inorg.Biochem.* ,**105,**1538

Formula: $C_{24} H_{26} Mn_1 N_2 O_6{}^{1+}, 2(C_1 H_4 O_1), N_1 O_3{}^{1-}$

Compound Name: Diaqua-(2,2'-(1,2-phenylenebis((nitrilo)methylylidene))bis(6-ethoxyphenolato))-manganese(iii) nitrate methanol solvate

Space Group:	P-1	***Cell:***	***a*** 10.791(5)	***b*** 12.638(5)	***c*** 12.788(5)
Space Group No.:	2	***(Å,°)***	α 111.18(0)	β 101.70(0)	γ 109.99(0)

R-Factor (%): 5.44 ***Temperature(K):*** 180 ***Density(g/cm^3):*** 1.451

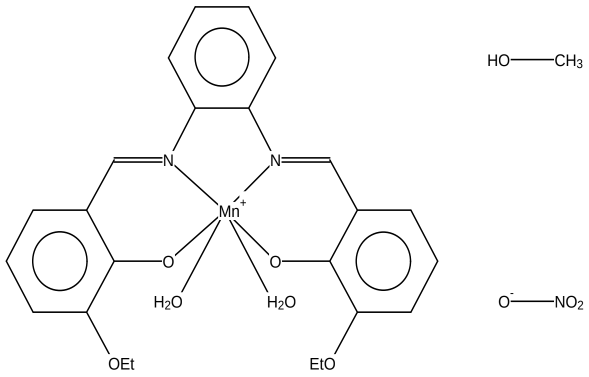

ICUYIQ

Reference: M.A.Vazquez-Fernandez, M.R.Bermejo, M.I.Fernandez-Garcia, G.Gonzalez-Riopedre, M.J.Rodriguez-Douton, M.Maneiro (2011) *J.Inorg.Biochem.* ,**105,**1538

Formula: $C_{24} H_{23} Mn_1 N_2 O_7, H_2 O_1$

Compound Name: (2,2'-(1,2-phenylenebis((nitrilo)methylylidene))bis(6-methoxyphenolato))-acetato-aqua-manganese(iii) monohydrate

Space Group:	P-1	***Cell:***	***a*** 10.518(3)	***b*** 11.023(3)	***c*** 11.067(3)
Space Group No.:	2	***(Å,°)***	α 101.95(0)	β 97.69(0)	γ 114.25(0)

R-Factor (%): 4.17 ***Temperature(K):*** 110 ***Density(g/cm^3):*** 1.570

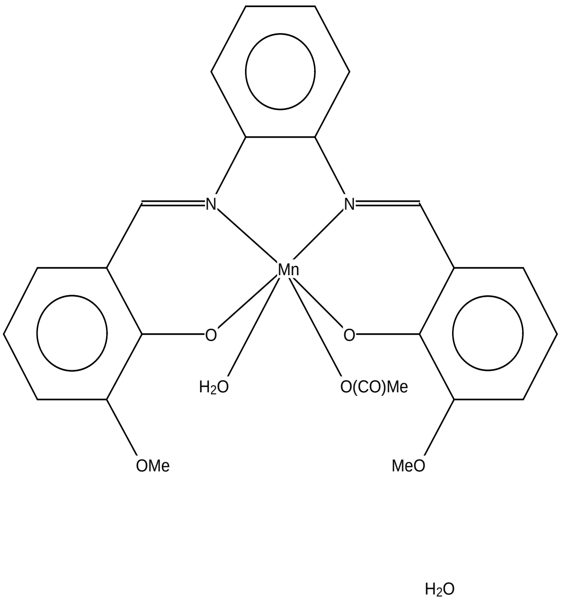

IDELOT

Reference: L.Salmon, P.Thuery, M.Ephritikhine (2006) *Private Communication ,*

Formula: $C_{22} H_{18} Cu_1 N_2 O_4, C_5 H_5 N_1$

Compound Name: (N,N'-(1,2-Phenylene)-bis(3-methoxysalicylaldiminato))-copper pyridine solvate

Space Group:	P21/c	***Cell:***	***a*** 11.099(0)	***b*** 18.786(0)	***c*** 11.264(0)
Space Group No.:	14	***(Å,°)***	α 90.00	β 104.69(0)	γ 90.00

R-Factor (%): 4.39 ***Temperature(K):*** 100 ***Density(g/cm^3):*** 1.512

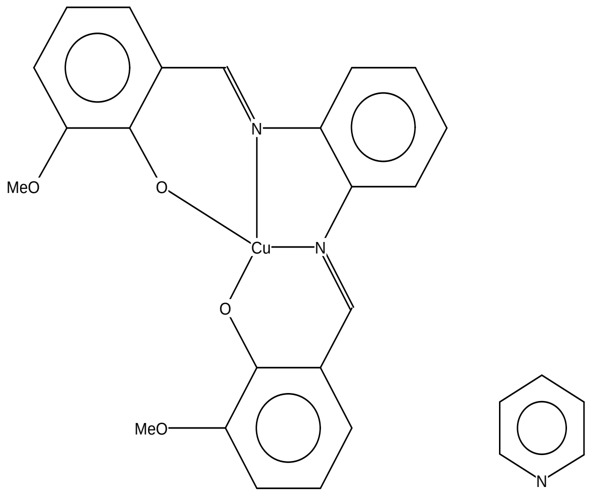

Search: search26 (Tue Dec 2 15:48:50 2014): Hits 277-280

IDEMUA

Reference: L.Salmon, P.Thuery, M.Ephritikhine (2006) *Private Communication ,*

Formula: $C_{62} H_{62} Li_2 N_4 O_{14} U_1 Zn_2,2(C_4 H_8 O_1)$

Compound Name: bis((μ_3-N,N'-(4,5-Dimethyl-1,2-phenylene)-bis(3-oxysalicylaldiminato)-(μ_2-acetylacetonato-O,O')-tetrahydrofuran))-di-lithium-di-zinc-uranium tetrahydrofuran solvate

Space Group:	P-1	***Cell:***	***a*** 14.917(3)	***b*** 15.562(2)	***c*** 15.995(3)
Space Group No.:	2	***(Å,°)***	α 107.25(1)	β 97.58(0)	γ 102.81(0)

R-Factor (%): 6.79 ***Temperature(K):*** 100 ***Density(g/cm^3):*** 1.586

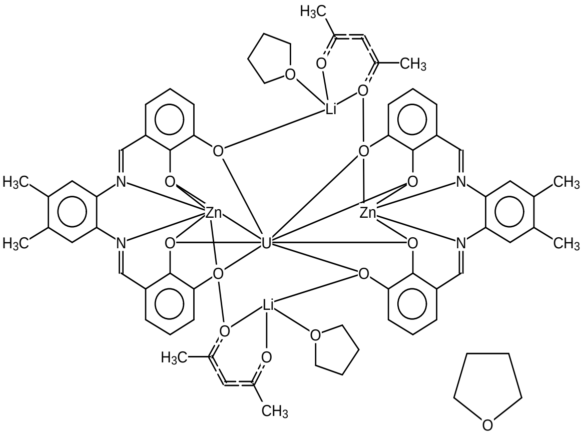

IFERIU

Reference: Shu-Ni Li, Jun Li, Zong-Xun Tang, Zhong-Yuan Zhou (2001) *Huaxue Xuebao(Chin.)(Acta Chim.Sinica)* ,**59,**78

Formula: $C_8 H_{20} N_1{}^{1+},2(C_{20} H_{18} Mn_1 N_2 O_4{}^{1+}),C_6 Fe_1 N_6{}^{3-},C_1 H_4 O_1,H_2 O_1$

Compound Name: Tetraethylammonium (1,2-bis(1,2-salicylideneamino)benzene)-manganese hexacyano-iron methanol solvate monohydrate

Space Group:	P-1	***Cell:***	***a*** 12.150(4)	***b*** 14.834(6)	***c*** 16.625(6)
Space Group No.:	2	***(Å,°)***	α 81.90(0)	β 76.98(0)	γ 83.11(0)

R-Factor (%): 7.40 ***Temperature(K):*** 295 ***Density(g/cm^3):*** 1.388

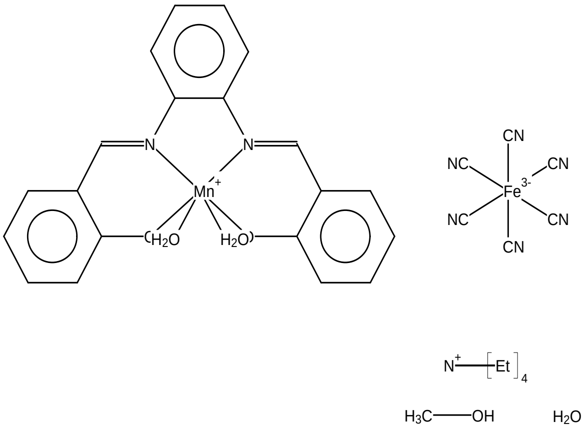

IFIYEC

Reference: S.Curreli, E.C.Escudero-Adan, J.Benet-Buchholz, A.W.Kleij (2008) *Eur.J.Inorg.Chem.* ,2863

Formula: $C_{60} H_{66} N_8 O_{14} S_2 Zn_2,6.5(C_2 H_6 O_1 S_1),H_2 O_1$

Compound Name: (μ_2-3,3'-bis((3,5-Dinitro-2-oxyphenylmethylene)amino)-4,4'-bis((3,5-di-t-butyl-2-oxyphenylmethylene)amino)biphenyl-N,N',N'',N''',O,O',O'',O''')-bis(dimethylsulfoxide-O)-di-zinc(ii) dimethylsulfoxide solvate monohydrate

Space Group:	P-1	***Cell:***	***a*** 14.750(0)	***b*** 16.577(0)	***c*** 19.912(1)
Space Group No.:	2	***(Å,°)***	α 79.46(0)	β 74.02(0)	γ 73.35(0)

R-Factor (%): 8.55 ***Temperature(K):*** 100 ***Density(g/cm^3):*** 1.375

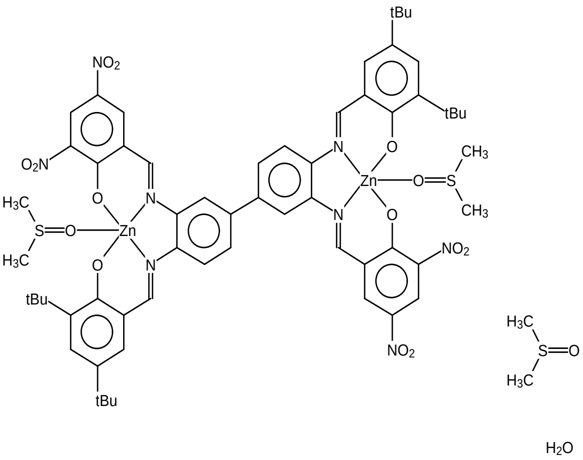

IFIYIG

Reference: S.Curreli, E.C.Escudero-Adan, J.Benet-Buchholz, A.W.Kleij (2008) *Eur.J.Inorg.Chem.* ,2863

Formula: $C_{52} H_{50} N_8 O_{14} S_2 Zn_2,3.5(C_2 H_6 O_1 S_1)$

Compound Name: (μ_2-3,3'-bis((3,5-Dinitro-2-oxyphenylmethylene)amino)-4,4'-bis((3-t-butyl-2-oxyphenylmethylene)amino)biphenyl-N,N',N'',N''',O,O',O'',O''')-bis(dimethylsulfoxide-O)-di-zinc(ii) dimethylsulfoxide solvate

Space Group:	C2/c	***Cell:***	***a*** 36.439(1)	***b*** 9.588(0)	***c*** 38.452(1)
Space Group No.:	15	***(Å,°)***	α 90.00	β 99.92(0)	γ 90.00

R-Factor (%): 5.88 ***Temperature(K):*** 100 ***Density(g/cm^3):*** 1.485

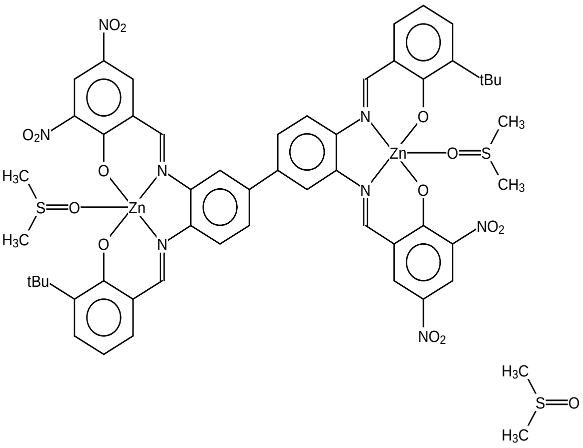

Search: search26 (Tue Dec 2 15:48:50 2014): Hits 281-284

IFIYOM

Reference: S.Curreli, E.C.Escudero-Adan, J.Benet-Buchholz, A.W.Kleij (2008) *Eur.J.Inorg.Chem.* ,2863

Formula: $(C_{66} H_{62} Cl_4 N_6 O_4 Zn_2)n,10n(C_4 H_8 O_1)$

Compound Name: catena-[(μ2-4,4'-Bipyridine-N,N')-(μ2-3,3'-bis((3,5-dichloro-2-oxyphenylmethylene)amino)-4,4'-bis((3,5-di-t-butyl-2-oxyphenylmethylene)amino)biphenyl-N,N',N'',N''',O,O',O'',O''')-di-zinc(ii) tetrahydrofuran solvate]

Space Group:	P-1	***Cell:***	***a*** 9.648(0)	***b*** 16.163(0)	***c*** 17.576(0)		
Space Group No.:	2	***(Å,°)***	α 87.57(0)	β 74.28(0)	γ 74.18(0)		
R-Factor (%):	6.96	***Temperature(K):***	100	***Density(g/cm³):***	1.307		

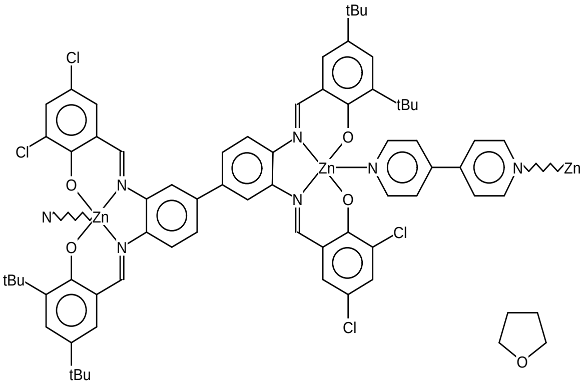

IFUROQ

Reference: M.Vazquez, M.R.Bermejo, M.Fondo, A.M.Garcia-Deibe, A.M.Gonzalez, R.Pedrido, J.Sanmartin (2002) *Z.Anorg.Allg.Chem.* , **628,**1068

Formula: $C_{34} H_{28} N_4 Ni_1 O_4 S_2,C_2 H_3 N_1$

Compound Name: (N,N'-bis(2-Tosylaminobenzylidene)-1,2-diaminobenzene)-nickel acetonitrile solvate

Space Group:	P212121	***Cell:***	***a*** 12.119(0)	***b*** 13.301(0)	***c*** 21.254(1)		
Space Group No.:	19	***(Å,°)***	α 90.00	β 90.00	γ 90.00		
R-Factor (%):	4.99	***Temperature(K):***	298	***Density(g/cm³):***	1.397		

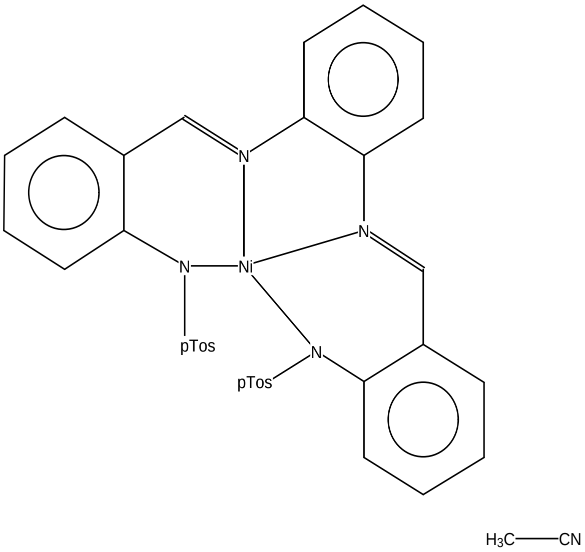

IGAFOL

Reference: C.F.Works, C.J.Jocher, G.D.Bart, Xianhui Bu, P.C.Ford (2002) *Inorg.Chem.* ,**41,**3728

Formula: $C_{28} H_{30} Cl_1 N_3 O_3 Ru_1$

Compound Name: Chloro-(N,N'-o-phenylene-bis(3,5-t-butylsalicylideneiminato))-nitrosyl-ruthenium

Space Group:	P-1	***Cell:***	***a*** 6.782(0)	***b*** 14.141(0)	***c*** 14.612(0)		
Space Group No.:	2	***(Å,°)***	α 81.24(0)	β 79.59(0)	γ 89.71(0)		
R-Factor (%):	2.73	***Temperature(K):***	293	***Density(g/cm³):***	1.446		

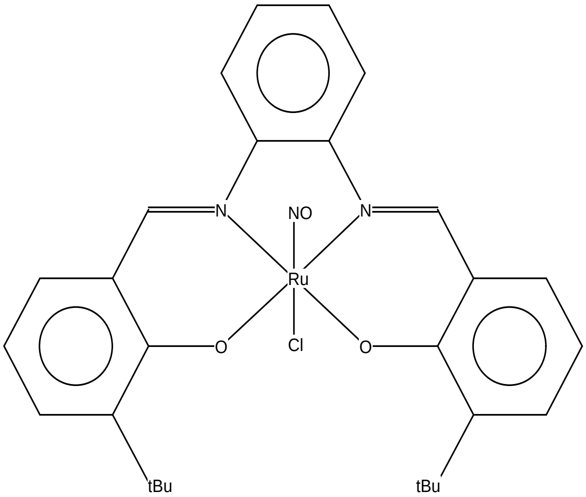

IHINUJ

Reference: M.Schley, P.Lonnecke, E.Hey-Hawkins (2009) *J.Organomet.Chem.* ,**694,**2480

Formula: $C_{38} H_{54} Mn_1 N_2 O_6$ $^{1+}$,Cl_1 $^{1-}$,$C_1 H_4 O_1$

Compound Name: (2,2'-((1,2-Dihydroxybenzene-4,5-diyl)bis(nitrilomethylylidene))bis(4,6-di-t-butylphenolato)-N,N',O,O')-bis(methanol)-manganese(iii) chloride methanol solvate

Space Group:	P-1	***Cell:***	***a*** 7.810(0)	***b*** 15.265(3)	***c*** 18.278(5)		
Space Group No.:	2	***(Å,°)***	α 74.88(1)	β 88.64(1)	γ 87.32(1)		
R-Factor (%):	3.99	***Temperature(K):***	180	***Density(g/cm³):***	1.197		

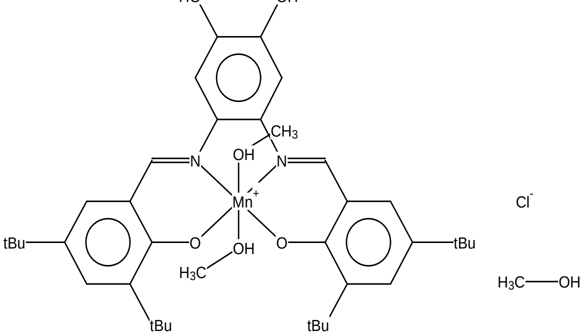

IHIPAR

Reference: M.Schley, P.Lonnecke, E.Hey-Hawkins (2009) *J.Organomet.Chem.* ,**694,**2480

Formula: $C_{56} H_{74} N_2 Ni_1 O_4 Zr_1,3(C_6 H_6)$

Compound Name: (μ_2-4,5-bis(((3,5-Di-t-butyl-2-oxidophenyl)methylidene)amino)benzene-1,2-diolato-N,N',O,O',O'',O''')-bis(η^5-pentamethylcyclopentadienyl)-nickel(ii)-zirconium benzene solvate

Space Group:	P21/c	***Cell:***	***a*** 14.722(3)	***b*** 18.722(4)	***c*** 25.725(5)
Space Group No.:	14	***(Å,°)***	α 90.00	β 105.88(3)	γ 90.00
R-Factor (%):	3.96	***Temperature(K):*** 213	***Density(g/cm^3):*** 1.192		

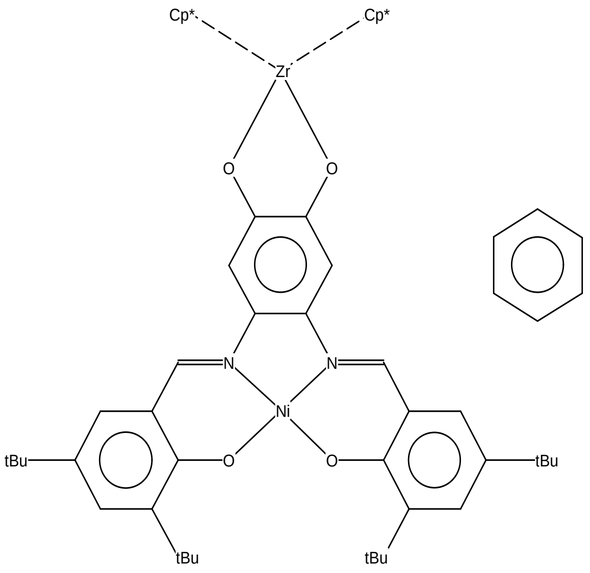

IHIPEV

Reference: M.Schley, P.Lonnecke, E.Hey-Hawkins (2009) *J.Organomet.Chem.* ,**694,**2480

Formula: $C_{56} H_{74} Cu_1 N_2 O_4 Zr_1,1.5(C_7 H_8)$

Compound Name: (μ_2-4,5-bis(((3,5-Di-t-butyl-2-oxidophenyl)methylidene)amino)benzene-1,2-diolato-N,N',O,O',O'',O''')-bis(η^5-pentamethylcyclopentadienyl)-copper(ii)-zirconium toluene solvate

Space Group:	P-1	***Cell:***	***a*** 13.241(0)	***b*** 13.496(0)	***c*** 18.989(0)
Space Group No.:	2	***(Å,°)***	α 99.59(0)	β 110.37(0)	γ 98.38(0)
R-Factor (%):	3.62	***Temperature(K):*** 213	***Density(g/cm^3):*** 1.229		

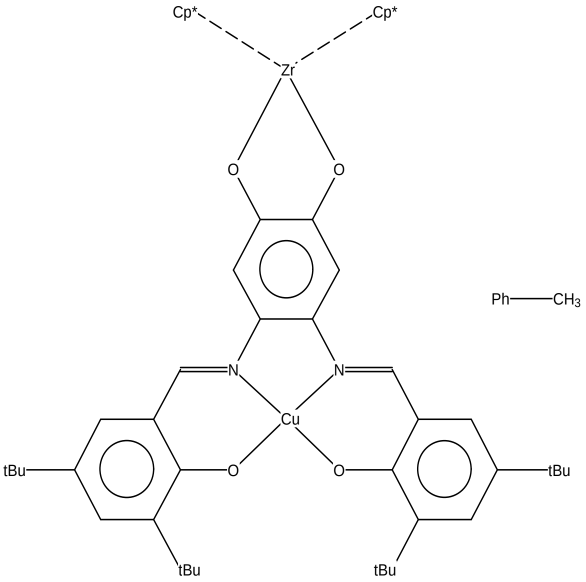

IHIPIZ

Reference: M.Schley, P.Lonnecke, E.Hey-Hawkins (2009) *J.Organomet.Chem.* ,**694,**2480

Formula: $C_{61} H_{79} Cl_1 Cr_1 N_3 O_4 Zr_1,2(C_7 H_8)$

Compound Name: (μ_2-4,5-bis(((3,5-Di-t-butyl-2-oxidophenyl)methylidene)amino)benzene-1,2-diolato-N,N',O,O',O'',O''')-bis(η^5-pentamethylcyclopentadienyl)-chloro-pyridine-chromium(iii)-zirconium toluene solvate

Space Group:	P212121	***Cell:***	***a*** 15.907(3)	***b*** 17.995(4)	***c*** 25.179(5)
Space Group No.:	19	***(Å,°)***	α 90.00	β 90.00	γ 90.00
R-Factor (%):	4.69	***Temperature(K):*** 213	***Density(g/cm^3):*** 1.181		

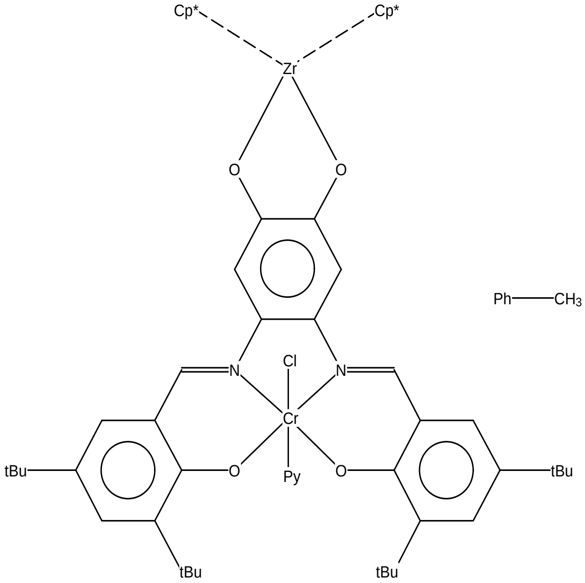

IHITOJ

Reference: E.Escudero-Adan, J.Benet-Buchholz, A.W.Kleij (2009) *Eur.J.Inorg.Chem.* ,3562

Formula: $C_{43} H_{55} N_3 O_2 Zn_1$

Compound Name: (2,5-Dimethylpyridine-N)-(2,2'-(1,2-phenylenebis((nitrilo)methylylidene))bis(4,6-di-t-butylphenolato)-N,N',O,O')-zinc(ii)

Space Group:	P-1	***Cell:***	***a*** 10.841(0)	***b*** 12.570(0)	***c*** 15.914(0)
Space Group No.:	2	***(Å,°)***	α 82.27(0)	β 79.10(0)	γ 64.88(0)
R-Factor (%):	3.18	***Temperature(K):*** 100	***Density(g/cm^3):*** 1.228		

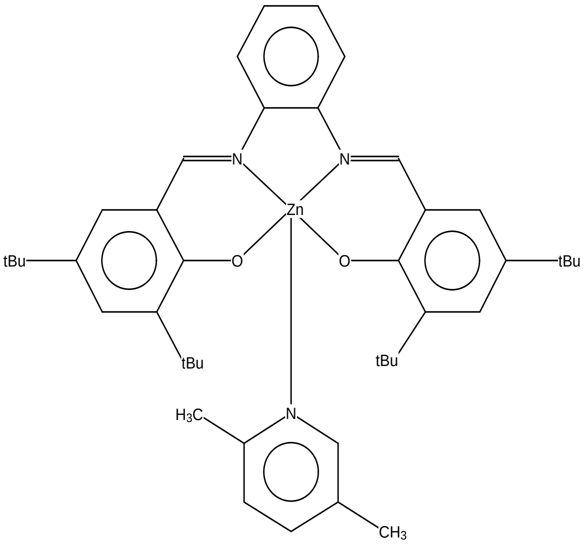

Search: search26 (Tue Dec 2 15:48:50 2014): Hits 289-292

IHITUP

Reference: E.Escudero-Adan, J.Benet-Buchholz, A.W.Kleij (2009) *Eur.J.Inorg.Chem.* ,3562

Formula: $C_{36} H_{48} N_2 O_3 Zn_1,2(C_7 H_9 N_1)$

Compound Name: Aqua-(2,2'-(1,2-phenylenebis((nitrilo)methylylidene))bis(4,6-di-t-butylphenolato)-N,N',O,O')-zinc(ii) 2,6-dimethylpyridine solvate

Space Group:	P21/c	***Cell:***	***a***	11.686(0)	***b***	28.296(1)	***c***	14.764(0)
Space Group No.:	14	***(Å,°)***	α	90.00	β	105.60(0)	γ	90.00

R-Factor (%): 6.02 ***Temperature(K):*** 100 ***Density(g/cm³):*** 1.182

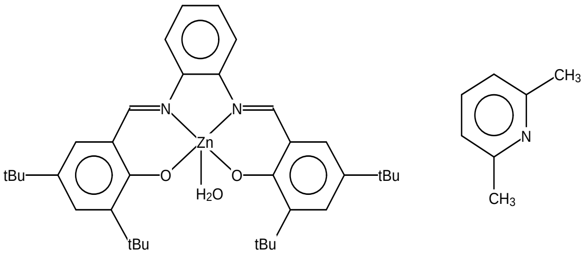

IHIVAX

Reference: E.Escudero-Adan, J.Benet-Buchholz, A.W.Kleij (2009) *Eur.J.Inorg.Chem.* ,3562

Formula: $C_{28} H_{30} N_4 O_7 Zn_1,C_2 H_3 N_1,2(C_7 H_9 N_1)$

Compound Name: Aqua-(1-(((3,5-di-t-butyl-2-oxidophenyl)methylene)amino)-3-(((3,5-dinitro-2-oxidophenyl)methylene)amino)benzene-N,N',O,O')-zinc(ii) 2,6-dimethylpyridine acetonitrile solvate

Space Group:	C2/c	***Cell:***	***a***	42.112(1)	***b***	10.356(0)	***c***	24.526(0)
Space Group No.:	15	***(Å,°)***	α	90.00	β	122.20(0)	γ	90.00

R-Factor (%): 5.87 ***Temperature(K):*** 100 ***Density(g/cm³):*** 1.255

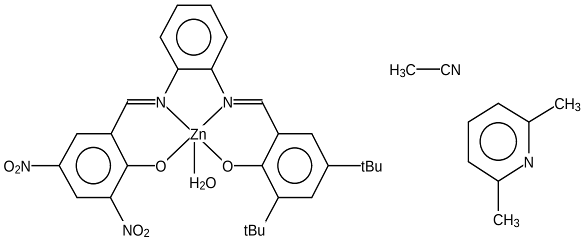

IKEXAY

Reference: S.Bhattacharya, S.Mondal, S.Sasmal, H.A.Sparkes, J.A.K.Howard, M.Nayak, S.Mohanta (2011) *CrystEngComm* ,**13,**1029

Formula: $2(C_{24} H_{22} Cu_1 N_2 O_4),H_4 N_2 O_{10} U_1,0.68(H_2 O_1)$

Compound Name: bis[(2,2'-(1,2-Phenylenebis((nitrilo)methylylidene))bis(6-ethoxyphenolato))-copper] diaqua-bis(nitrato)-dioxo-uranium hydrate

Space Group:	P-1	***Cell:***	***a***	9.368(0)	***b***	11.849(0)	***c***	11.872(0)
Space Group No.:	2	***(Å,°)***	α	70.66(0)	β	88.79(0)	γ	78.81(0)

R-Factor (%): 1.89 ***Temperature(K):*** 120 ***Density(g/cm³):*** 1.873

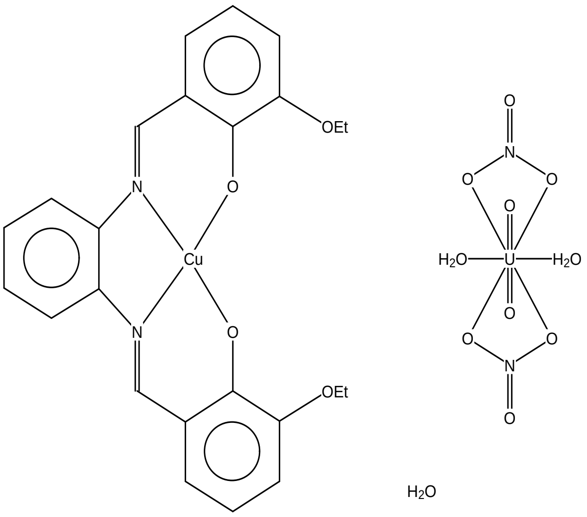

IKEXEC

Reference: S.Bhattacharya, S.Mondal, S.Sasmal, H.A.Sparkes, J.A.K.Howard, M.Nayak, S.Mohanta (2011) *CrystEngComm* ,**13,**1029

Formula: $2(C_{24} H_{22} N_2 Ni_1 O_4),H_4 N_2 O_{10} U_1$

Compound Name: bis[(2,2'-(1,2-Phenylenebis((nitrilo)methylylidene))bis(6-ethoxyphenolato))-nickel] diaqua-bis(nitrato)-dioxo-uranium

Space Group:	P-1	***Cell:***	***a***	9.529(0)	***b***	11.649(0)	***c***	11.817(0)
Space Group No.:	2	***(Å,°)***	α	70.48(0)	β	88.43(0)	γ	77.80(0)

R-Factor (%): 2.16 ***Temperature(K):*** 120 ***Density(g/cm³):*** 1.860

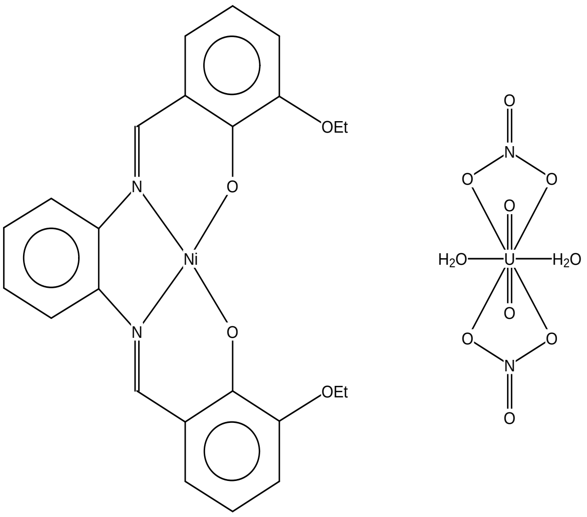

Search: search26 (Tue Dec 2 15:48:50 2014): Hits 293-296

INAPIX

Reference: M.L.Mejia, J.H.Rivers, S.F.Swingle, Zheng Lu, Xiaoping Yang, M.Findlater, G.Reeske, B.J.Holliday (2010) *Main Group Chem.* ,**9,**167

Formula: C_{44} H_{36} Br_4 Ga_2 N_4 O_{11},2(C_1 H_4 O_1),H_2 O_1

Compound Name: (μ_2-oxo)-diaqua-bis(2,2'-(1,2-phenylenebis(nitrilomethylylidene))-bis(4-bromo-6-methoxyphenolato))-di-gallium methanol solvate monohydrate

Space Group:	P-1	***Cell:***	***a*** 15.021(3)	***b*** 15.060(4)	***c*** 15.761(3)		
Space Group No.:	2	***(Å,°)***	α 102.89(3)	β 109.32(2)	γ 101.55(3)		
R-Factor (%):	10.99	***Temperature(K):***	153	***Density(g/cm³):***	1.419		

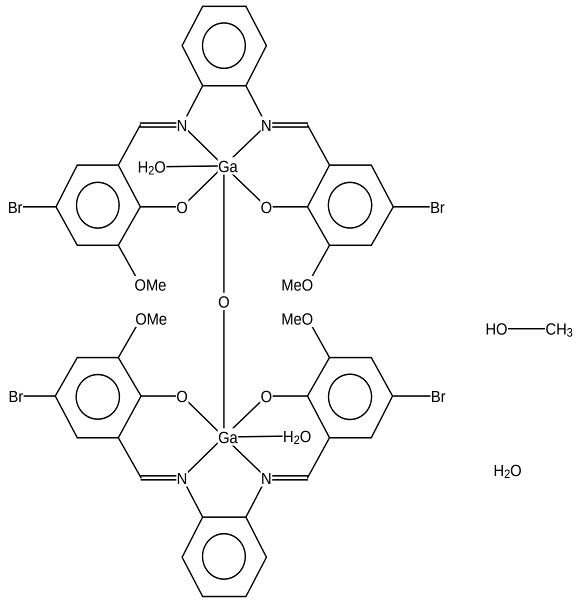

INAPOD

Reference: M.L.Mejia, J.H.Rivers, S.F.Swingle, Zheng Lu, Xiaoping Yang, M.Findlater, G.Reeske, B.J.Holliday (2010) *Main Group Chem.* ,**9,**167

Formula: C_{23} H_{22} Br_2 Ga_1 N_2 O_6 $^{1+}$,C_1 H_4 O_1,N_1 O_3 $^{1-}$

Compound Name: Aqua-(methanol)-(2,2'-(1,2-phenylenebis((nitrilo)methylylidene))-bis(4-bromo-6-methoxyphenolato))-gallium nitrate methanol solvate

Space Group:	P21/n	***Cell:***	***a*** 11.566(2)	***b*** 16.341(3)	***c*** 14.426(3)		
Space Group No.:	14	***(Å,°)***	α 90.00	β 90.69(3)	γ 90.00		
R-Factor (%):	3.45	***Temperature(K):***	153	***Density(g/cm³):***	1.818		

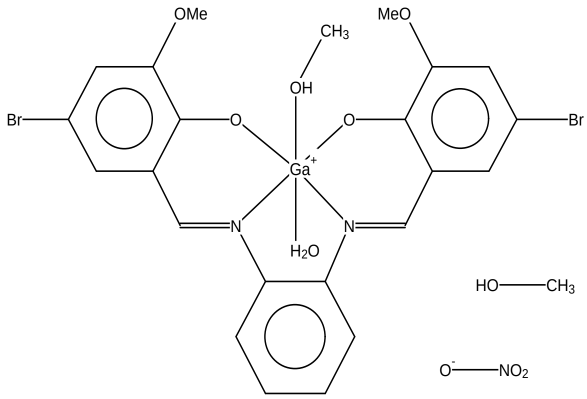

INOJOL

Reference: Weixu Feng, Yani Hui, Tao Wei, Xingqiang Lu, Jirong Song, Zhongning Chen, Shunsheng Zhao, Wai-Kwok Wong, R.A.Jones (2011) *Inorg.Chem.Commun.* ,**14,**75

Formula: C_{44} H_{34} N_9 Nd_1 O_{13} Zn_2

Compound Name: bis(μ_2-2,2'-(o-Phenylenebis(nitrilomethylidyne))diphenolato-N,N',O,O':O,O')-bis(acetonitrile)-bis(nitrato-O,O')-(nitrato-O)-neodymium(iii)-di-zinc(ii)

Synonym: bis(μ_2-N,N'-bis(Salicylidene)benzene-1,2-diamine-N,N',O,O':O,O')-bis(acetonitrile)-bis(nitrato-O,O')-(nitrato-O)-neodymium(iii)-di-zinc(ii)

Space Group:	Pn	***Cell:***	***a*** 13.602(3)	***b*** 12.227(2)	***c*** 13.623(3)		
Space Group No.:	7	***(Å,°)***	α 90.00	β 100.98(3)	γ 90.00		
R-Factor (%):	4.51	***Temperature(K):***	293	***Density(g/cm³):***	1.750		

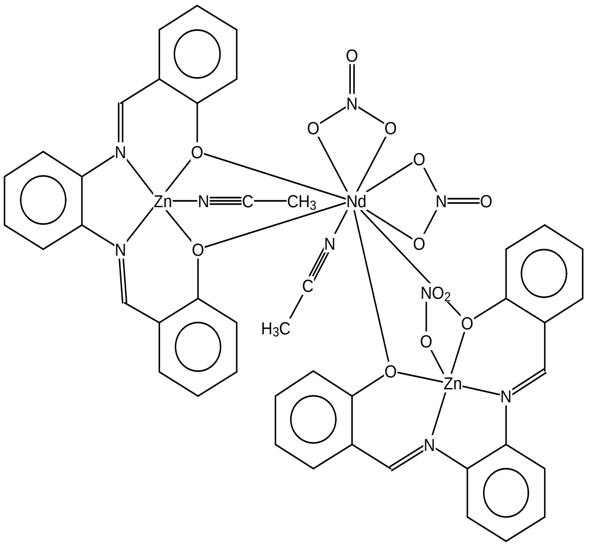

INOJUR

Reference: Weixu Feng, Yani Hui, Tao Wei, Xingqiang Lu, Jirong Song, Zhongning Chen, Shunsheng Zhao, Wai-Kwok Wong, R.A.Jones (2011) *Inorg.Chem.Commun.* ,**14,**75

Formula: C_{43} H_{35} Cl_1 N_5 Nd_1 O_5 Zn_1

Compound Name: (μ_2-2,2'-(o-Phenylenebis(nitrilomethylidyne))diphenolato-N,N',O,O':O,O')-(2,2'-(o-phenylenebis(nitrilomethylidyne))diphenolato-N,N',O,O')-chloro-(dimethylformamide-O)-neodymium(iii)-zinc(ii)

Synonym: (μ_2-N,N'-bis(Salicylidene)benzene-1,2-diamine-N,N',O,O':O,O')-(N,N'-bis(salicylidene)benzene-1,2-diamine-N,N',O,O')-chloro-(dimethylformamide-O)-neodymium(iii)-zinc(ii)

Space Group:	P21/n	***Cell:***	***a*** 13.938(3)	***b*** 9.908(2)	***c*** 28.035(6)		
Space Group No.:	14	***(Å,°)***	α 90.00	β 97.23(3)	γ 90.00		
R-Factor (%):	5.13	***Temperature(K):***	293	***Density(g/cm³):***	1.637		

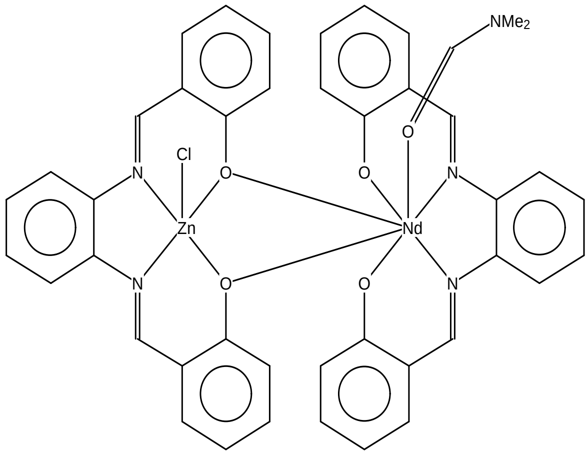

Search: search26 (Tue Dec 2 15:48:50 2014): Hits 297-300

IPADEJ

Reference: Yan Li, Qing Ma, Hua-Tian Shi, Qun Chen, Qian-Feng Zhang (2011) *Z.Naturforsch.,B:Chem.Sci.* ,**66,**324

Formula: $C_{38}\ H_{29}\ Cl_1\ N_2\ O_2\ P_1\ Ru_1,2(C_1\ H_2\ Cl_2)$

Compound Name: Chloro-(N,N'-bis(salicylidene)-o-phenylenediaminato)-triphenylphosphine-ruthenium(iii) dichloromethane solvate

Space Group:	P21/n	***Cell:***	***a*** 13.113(0) ***b*** 18.326(0) ***c*** 16.988(0)
Space Group No.:	14	***(Å,°)***	α 90.00 β 105.25(0) γ 90.00

R-Factor (%): 3.43 ***Temperature(K):*** 296 ***Density(g/cm3):*** 1.489

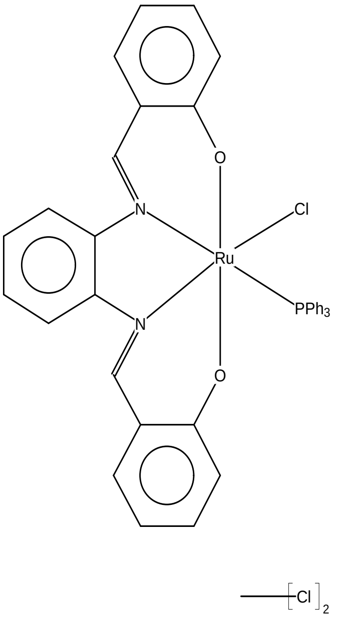

IQEFIU

Reference: A.Cucos, A.Ursu, A.M.Madalan, C.Duhayon, J.-P.Sutter, M.Andruh (2011) *CrystEngComm* ,**13,**3756

Formula: $C_{22}\ H_{18}\ N_2\ Ni_1\ O_4,0.5(H_4\ N_1\ ^{1+}),0.5(Cl_1\ O_4\ ^{1-}),0.5(C_1\ H_4\ O_1)$

Compound Name: hemiammonium hemiperchlorate (N,N'-(1,2-phenylene)-bis(3-methoxysalicylideneamine))-nickel(ii) methanol solvate

Space Group:	P21/n	***Cell:***	***a*** 13.745(0) ***b*** 14.063(0) ***c*** 22.834(0)
Space Group No.:	14	***(Å,°)***	α 90.00 β 95.61(0) γ 90.00

R-Factor (%): 4.76 ***Temperature(K):*** 293 ***Density(g/cm3):*** 1.536

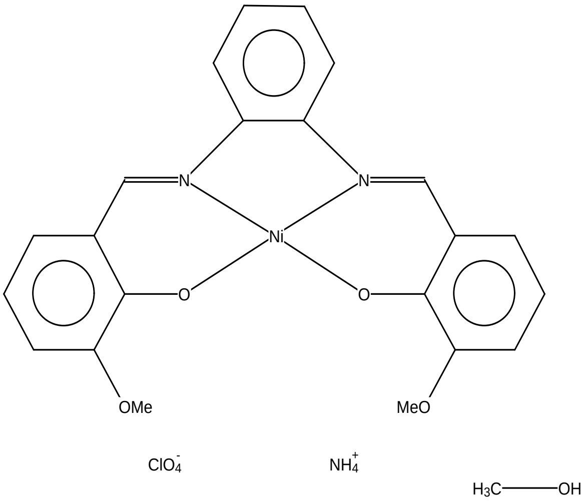

IQUJOU

Reference: Qibao Wang, Ruifang Ding, Ning Wang, Daodong Zhang, Jikun Li (2010) *Z.Anorg.Allg.Chem.* ,**636,**861

Formula: $C_{38}\ H_{52}\ N_2\ O_2\ Sn_1$

Compound Name: Dibutyl-(2,2'-(1,2-phenylenebis((nitrilo)methylylidene))bis(6-t-butyl-4-methylphenolato))-tin

Space Group:	P21/c	***Cell:***	***a*** 23.167(3) ***b*** 7.821(1) ***c*** 20.415(3)
Space Group No.:	14	***(Å,°)***	α 90.00 β 105.72(0) γ 90.00

R-Factor (%): 3.58 ***Temperature(K):*** 273 ***Density(g/cm3):*** 1.282

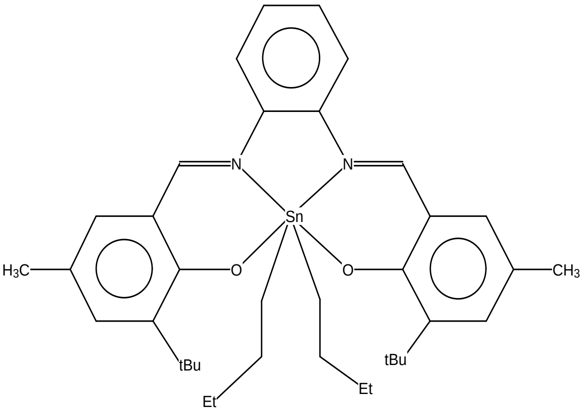

ISIWIR

Reference: H.C.Hardwick, D.S.Royal, M.Helliwell, S.J.A.Pope, L.Ashton, R.Goodacre, C.A.Sharrad (2011) *Dalton Trans.* ,**40,**5939

Formula: $C_{25}\ H_{19}\ N_3\ O_4\ U_1$

Compound Name: Dioxo-(2,2'-(1,2-phenylenebis((nitrilo)methylylidene))diphenolato)-(pyridine)-uranium

Space Group:	P21/c	***Cell:***	***a*** 17.382(1) ***b*** 9.622(0) ***c*** 14.651(1)
Space Group No.:	14	***(Å,°)***	α 90.00 β 113.86(0) γ 90.00

R-Factor (%): 2.87 ***Temperature(K):*** 100 ***Density(g/cm3):*** 1.966

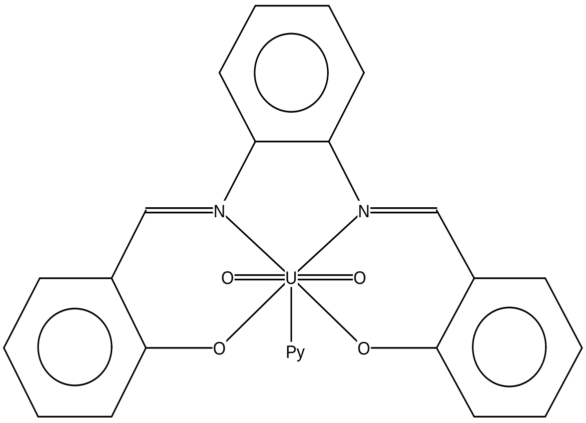

Search: search26 (Tue Dec 2 15:48:50 2014): Hits 301-304

ISOZEV

Reference: Y.Wang, S.Bhandari, S.Parkin, D.A.Atwood (2004) *J.Organomet.Chem.* ,**689,**759

Formula: $C_{72}\,H_{92}\,Al_2\,N_4\,O_5,C_7\,H_8$

Compound Name: (μ_2-Oxo)-bis(o-phenylenebis(3,5-di-t-butylsalicylaldiminato))-di-aluminium toluene solvate

Space Group:	C2/c	***Cell:***	***a*** 24.489(2)	***b*** 17.021(1)	***c*** 18.995(1)
Space Group No.:	15	***(Å,°)***	α 90.00	β 106.26(1)	γ 90.00

R-Factor (%): 4.93 ***Temperature(K):*** 173 ***Density(g/cm^3):*** 1.083

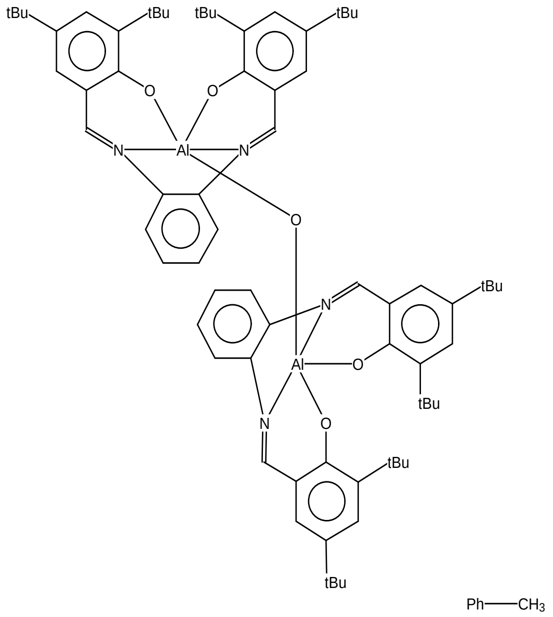

IVOVUL

Reference: A.Jana, R.Koner, T.Weyhermueller, P.Lemoine, M.Ghosh, S.Mohanta (2011) *Inorg.Chim.Acta* ,**375,**263

Formula: $C_{24}\,H_{28}\,Cu_1\,Fe_1\,N_2\,O_7\,^{2+},2(C_{24}\,H_{22}\,Cu_1\,N_2\,O_4),2(Cl_1\,O_4\,^{1-}),C_3\,H_6\,O_1,H_2\,O_1$

Compound Name: (μ_2-2,2'-(1,2-Phenylenebis((nitrilo)methylylidene))bis(6-ethoxyphenolato))-triaqua-copper-iron (2,2'-(1,2-phenylenebis((nitrilo)methylylidene))bis(6-ethoxyphenolato))-copper diperchlorate acetone solvate monohydrate

Space Group:	P21/n	***Cell:***	***a*** 14.219(1)	***b*** 27.030(3)	***c*** 20.334(2)
Space Group No.:	14	***(Å,°)***	α 90.00	β 107.23(1)	γ 90.00

R-Factor (%): 4.61 ***Temperature(K):*** 293 ***Density(g/cm^3):*** 1.586

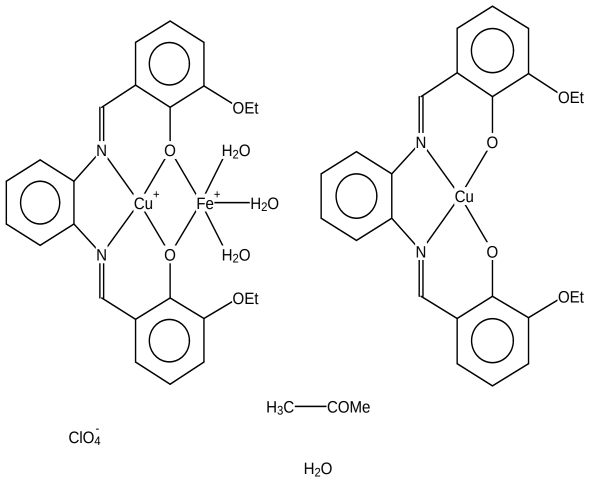

IYUHUF

Reference: L.Salmon, P.Thuery, M.Ephritikhine (2004) *Dalton Trans.* , 1635

Formula: $C_{44}\,H_{36}\,N_4\,O_8\,U_1,C_4\,H_8\,O_1$

Compound Name: mer-bis(N,N'-bis(3-Methoxysalicylidene)-1,2-phenylenediamine)-uranium(iv) tetrahydrofuran solvate

Space Group:	C2/c	***Cell:***	***a*** 21.878(1)	***b*** 13.397(0)	***c*** 14.914(0)
Space Group No.:	15	***(Å,°)***	α 90.00	β 110.33(0)	γ 90.00

R-Factor (%): 3.92 ***Temperature(K):*** 100 ***Density(g/cm^3):*** 1.716

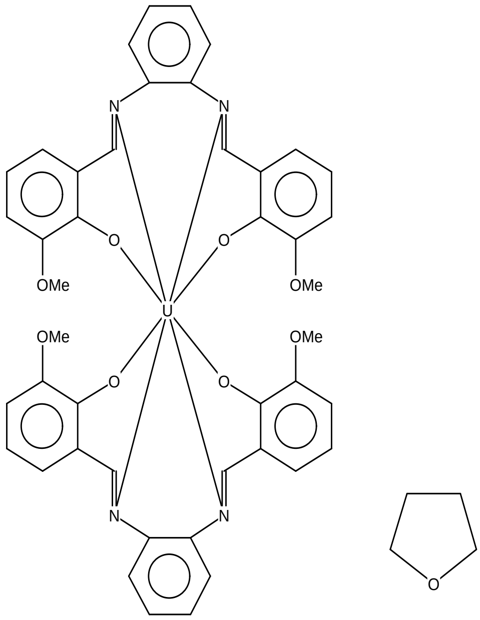

IYUJIV

Reference: L.Salmon, P.Thuery, M.Ephritikhine (2004) *Dalton Trans.* , 1635

Formula: $C_{70}\,H_{52}\,N_6\,O_{16}\,U_3,3(C_4\,H_8\,O_1)$

Compound Name: (μ_3-N,N'-bis(3-Hydroxysalicylidene)-1,2-phenylenediamine)-(μ_2-N,N'-bis(3-hydroxysalicylidene)-1,2-phenylenediamine)-bis(acetylacetonato)-tri-uranium(iv) tetrahydrofuran solvate

Space Group:	P-1	***Cell:***	***a*** 12.727(0)	***b*** 15.432(1)	***c*** 19.649(1)
Space Group No.:	2	***(Å,°)***	α 75.85(0)	β 87.79(0)	γ 78.11(0)

R-Factor (%): 4.93 ***Temperature(K):*** 100 ***Density(g/cm^3):*** 1.962

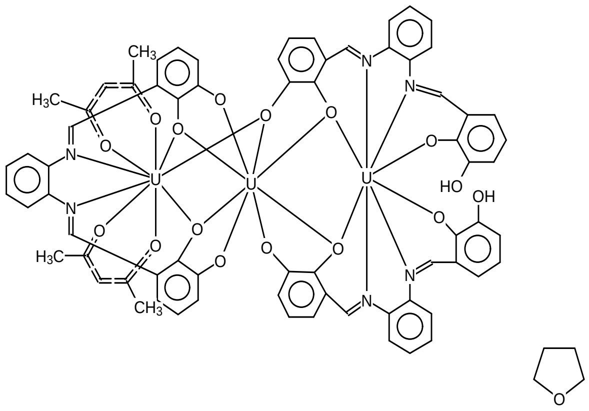

IZEHEB

Reference: M.Das, S.Chatterjee, S.Chattopadhyay (2011) *Inorg.Chem.Commun.* ,**14,**1337

Formula: C_{44} H_{36} N_4 Na_1 Ni_2 O_8 $^{1+}$,Cl_1 O_4 $^{1-}$

Compound Name: bis(μ_2-2,2'-(1,2-Phenylenebis(nitrilomethylylidene))bis(6-methoxyphenolato))-di-nickel-sodium perchlorate

Space Group:	P21/c	***Cell:***	***a***	11.971(4)	***b***	18.218(5)	***c***	19.054(6)
Space Group No.:	14	***(Å,°)***	α	90.00	β	91.89(0)	γ	90.00

R-Factor (%): 5.02 ***Temperature(K):*** 150 ***Density(g/cm^3):*** 1.581

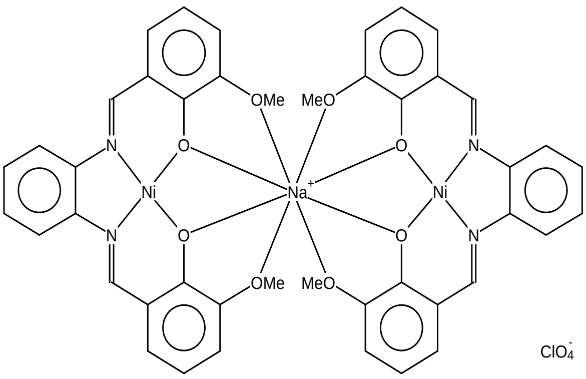

IZULOF

Reference: M.Yamamura, H.Miyazaki, M.Iida, S.Akine, T.Nabeshima (2011) *Inorg.Chem.* ,**50,**5315

Formula: C_{162} H_{200} N_{12} O_{48} Zn_{12},2(C_1 H_2 Cl_2),2(C_1 H_4 O_1),C_6 H_{14},2(H_2 O_1)

Compound Name: bis(μ_6-1^4,1^5,7^4,7^5,13^4,13^5-hexabutoxy-2,6,8,12,14,18-hexaaza-1,7,13(1,2),4,10,16(1,4)-hexabenzenacyclooctadecaphane-2,5,8,11,14,17-hexaene-4^2,4^3,10^2,10^3,16^2,16^3-hexaolato)-bis(μ_3-hydroxo)-octakis(μ_2-propanoato)-bis(propanoato)-diaqua-dodeca-zinc dichloromethane hexane methanol solvate dihydrate

Space Group:	P-1	***Cell:***	***a***	15.288(6)	***b***	17.956(6)	***c***	18.488(5)
Space Group No.:	2	***(Å,°)***	α	68.24(1)	β	76.24(1)	γ	80.30(1)

R-Factor (%): 9.21 ***Temperature(K):*** 120 ***Density(g/cm^3):*** 1.538

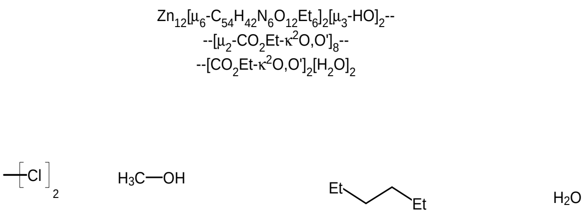

JEKSIC

Reference: Lian-Bin Wu, Zi-Qiang Hu, Guo-Qiao Lai (2006) *Jiegou Huaxue(Chin.J.Struct.Chem.)* ,**25,**567

Formula: C_{28} H_{26} Co_1 N_6 O_4,5(H_2 O_1)

Compound Name: bis(imidazole-N^3)-(N,N'-o-phenylenebis(3-methoxysalicylideneiminato)-N,N',O,O')-cobalt(ii) pentahydrate

Space Group:	P21/n	***Cell:***	***a***	9.681(0)	***b***	26.720(0)	***c***	12.799(0)
Space Group No.:	14	***(Å,°)***	α	90.00	β	100.93(0)	γ	90.00

R-Factor (%): 6.89 ***Temperature(K):*** 295 ***Density(g/cm^3):*** 1.348

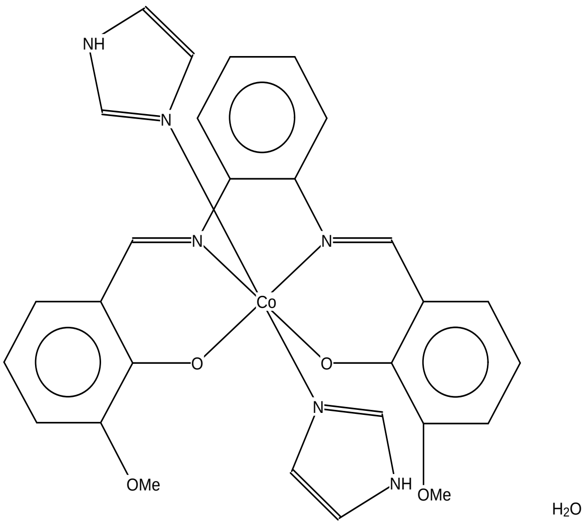

JEXKAY

Reference: A.M.van den Bergen, J.D.Cashion, G.D.Fallon, B.O.West (1990) *Aust.J.Chem.* ,**43,**1559

Formula: C_{22} H_{18} N_2 O_2 Sn_1

Compound Name: N,N'-(4,5-Dimethyl-1,2-phenylene)-bis(salicyclideneiminato-N,O)-tin(ii)

Space Group:	Pmn21	***Cell:***	***a***	20.082(10)	***b***	4.871(3)	***c***	9.418(5)
Space Group No.:	31	***(Å,°)***	α	90.00	β	90.00	γ	90.00

R-Factor (%): 4.20 ***Temperature(K):*** 295 ***Density(g/cm^3):*** 1.662

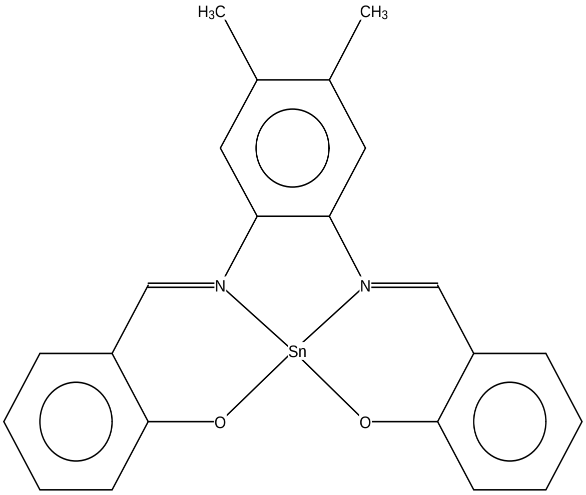

Search: search26 (Tue Dec 2 15:48:50 2014): Hits 309-312

JIMXOT

Reference: M.Narita, Il Yoon, M.Aoyagi, M.Goto, T.Shimizu, M.Asakawa (2007) *Eur.J.Inorg.Chem.* ,4229

Formula: $C_{34} H_{32} N_2 Ni_1 O_8, C_{22} H_{32} N_1{}^{1+}, F_6 P_1{}^{1-}$

Compound Name: (2)-(bis(4-t-Butylbenzyl)ammonium)-((28,35-diaza-2,5,8,15,18,21-hexaoxapentacyclo(35.3.1.1^{22,26}.0^{9,14}.0^{29,34})dotetraconta-1(41),9(14),10,12,22(42),23,25,27,29(34),30,32,35,37,39-tetradecaene-41,42-diolato)-nickel(ii))-rotaxane hexafluorophosphate

Space Group:	P-1	***Cell:***	***a*** 11.897(1)	***b*** 15.361(1)	***c*** 15.454(1)		
Space Group No.:	2	***(Å,°)***	α 104.46(0)	β 97.95(0)	γ 96.02(0)		
R-Factor (%):	5.38	***Temperature(K):***	203	***Density(g/cm³):***	1.377		

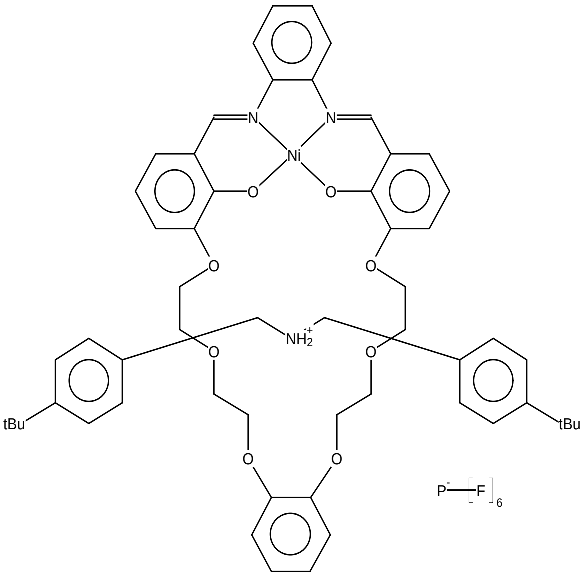

JIYHEF

Reference: S.J.Wezenberg, E.C.Escudero-Adan, J.Benet-Buchholz, A.W.Kleij (2008) *Inorg.Chem.* ,**47,**2925

Formula: $C_{136} H_{176} N_{10} O_8 Pd_2 Zn_2, 4(C_3 H_6 O_1)$

Compound Name: (μ_2-N,N',N'',N'''-tetrakis(3,5-Di-t-butylsalicylidenato)-1,2,4,5-phenylenetetraamine)-(2,2'-(pyridine-3,4-diyl-bis((nitrilo)methylylidene))-bis(4,6-di-t-butylphenolato-O))-di-palladium-di-zinc acetone solvate

Space Group:	P-1	***Cell:***	***a*** 12.906(1)	***b*** 16.703(1)	***c*** 18.811(1)		
Space Group No.:	2	***(Å,°)***	α 98.90(0)	β 108.75(0)	γ 106.40(0)		
R-Factor (%):	8.20	***Temperature(K):***	100	***Density(g/cm³):***	1.243		

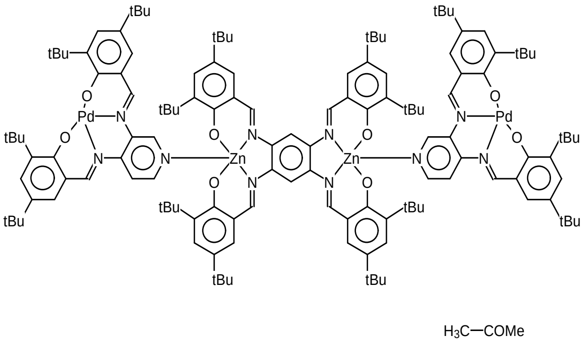

JUJDUN

Reference: F.M.Ashmawy, A.R.Ujaimi, C.A.McAuliffe, R.V.Parish, R.G.Pritchard (1991) *Inorg.Chim.Acta* ,**187,**155

Formula: $C_{40} H_{28} Fe_2 N_4 O_5, C_2 H_6 O_1 S_1$

Compound Name: (μ_2-Oxo)-bis(N,N'-o-phenylene-bis(salicylaldiminato))-di-iron(iii) dimethylsulfoxide solvate

Space Group:	P21/c	***Cell:***	***a*** 11.958(2)	***b*** 13.861(4)	***c*** 23.660(8)		
Space Group No.:	14	***(Å,°)***	α 90.00	β 101.86(4)	γ 90.00		
R-Factor (%):	5.40	***Temperature(K):***	295	***Density(g/cm³):***	1.444		

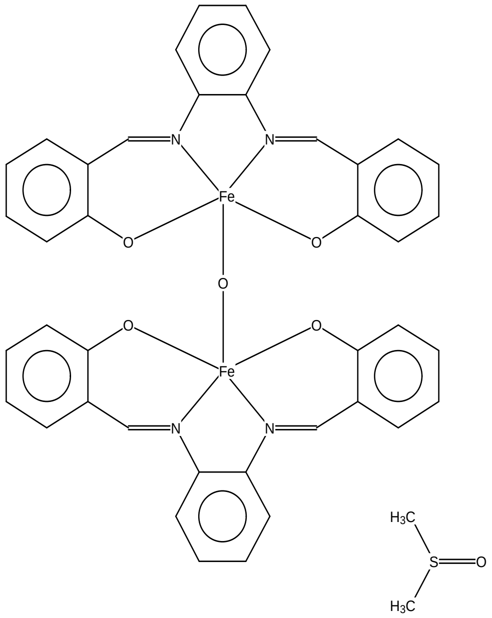

JUJDUN01

Reference: J.Kamenicek, Z.Travnicek, Z.Sindelar, J.Walla (1996) *Pol.J.Chem.* ,**70,**854

Formula: $C_{40} H_{28} Fe_2 N_4 O_5, C_2 H_6 O_1 S_1$

Compound Name: (μ_2-Oxo)-bis(N,N'-o-phenylene-bis(salicylaldiminato))-di-iron(iii) dimethylsulfoxide solvate

Space Group:	P21/c	***Cell:***	***a*** 11.693(2)	***b*** 13.869(3)	***c*** 23.662(5)		
Space Group No.:	14	***(Å,°)***	α 90.00	β 102.03(3)	γ 90.00		
R-Factor (%):	4.01	***Temperature(K):***	295	***Density(g/cm³):***	1.477		

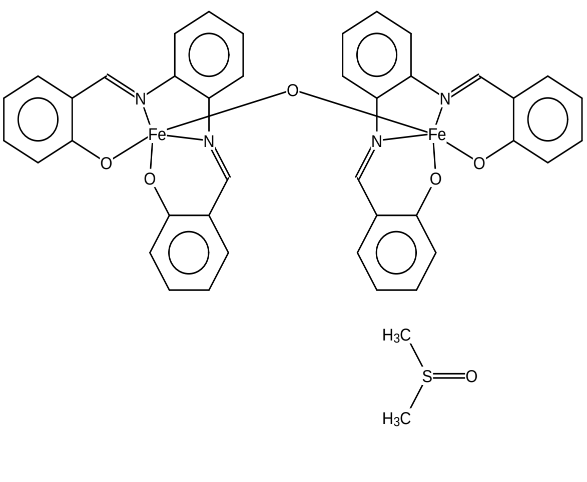

Search: search26 (Tue Dec 2 15:48:50 2014): Hits 313-316

JUKQIP

Reference:	F.Franceschi, E.Solari, C.Floriani, M.Rosi, A.Chiesi-Villa, C.Rizzoli (1999) *Chem.-Eur.J.* ,**5,**708
Formula:	C_{48} H_{48} N_4 Na_2 O_{10} V_2
Compound Name:	bis(μ_3-N,N'-Phenylene-bis(salicylideneiminato))-bis(dimethoxyethane)-di-sodium-di-vanadium

Space Group:	P-1	***Cell:***	***a*** 12.026(2)	***b*** 20.888(3)	***c*** 9.641(1)
Space Group No.:	2	***(Å,°)***	α 92.56(2)	β 106.98(2)	γ 92.71(2)
R-Factor (%):	5.38	***Temperature(K):*** 138	***Density(g/cm³):*** 1.422		

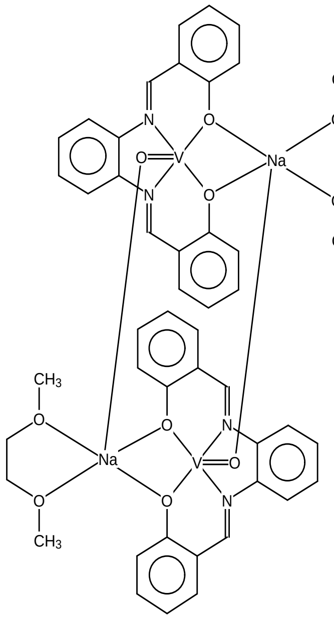

JUKRIQ

Reference:	F.Franceschi, E.Solari, C.Floriani, M.Rosi, A.Chiesi-Villa, C.Rizzoli (1999) *Chem.-Eur.J.* ,**5,**708
Formula:	C_{39} H_{29} N_3 O_2 Ti_1
Compound Name:	N,N'-Phenylene-bis(salicylideneiminato)-triphenylmethylimido-titanium

Space Group:	P212121	***Cell:***	***a*** 9.528(1)	***b*** 17.573(3)	***c*** 18.534(3)
Space Group No.:	19	***(Å,°)***	α 90.00	β 90.00	γ 90.00
R-Factor (%):	5.99	***Temperature(K):*** 295	***Density(g/cm³):*** 1.326		

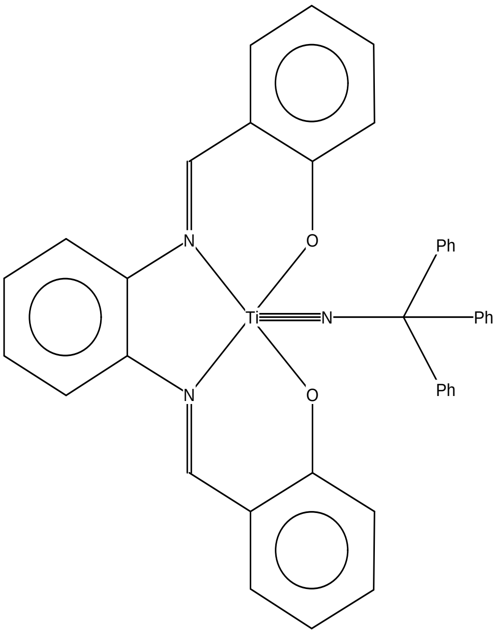

KAFHUW

Reference:	Xiaojing Wen, Jin-Yong Dong (2010) *Appl.Organomet.Chem.* ,**24,**503
Formula:	C_{36} H_{46} Cl_2 N_2 O_2 Ti_1,C_4 H_8 O_1
Compound Name:	Dichloro-(2,2'-(1,2-phenylenebis((nitrilo)methylylidene))bis(4,6-di-t-butylphenolato)-N,N',O,O')-titanium(iv) tetrahydrofuran solvate

Space Group:	C2/c	***Cell:***	***a*** 18.941(4)	***b*** 18.022(4)	***c*** 12.544(3)
Space Group No.:	15	***(Å,°)***	α 90.00	β 114.49(3)	γ 90.00
R-Factor (%):	7.50	***Temperature(K):*** 173	***Density(g/cm³):*** 1.244		

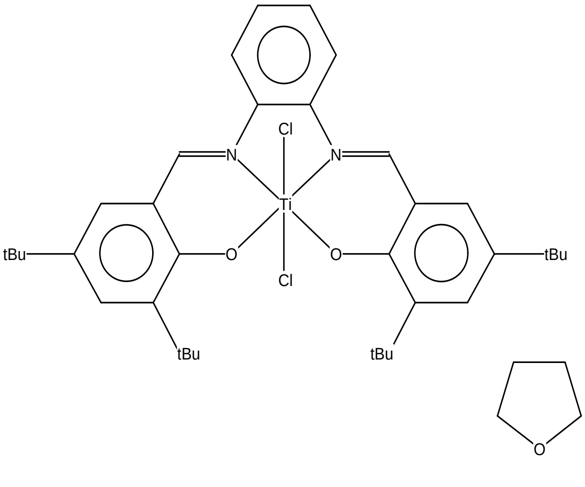

KAKZOM

Reference:	S.Biswas, K.Mitra, C.H.Schwalbe, C.R.Lucas, S.K.Chattopadhyay, B.Adhikary (2005) *Inorg.Chim.Acta* ,**358,**2473
Formula:	C_{21} H_{14} Mn_1 N_3 O_2 S_1
Compound Name:	Isothiocyanato-(N,N'-o-phenylenebis(salicylideneimine))-manganese(iii)

Space Group:	P21/n	***Cell:***	***a*** 12.847(0)	***b*** 10.059(0)	***c*** 14.187(0)
Space Group No.:	14	***(Å,°)***	α 90.00	β 100.01(0)	γ 90.00
R-Factor (%):	2.86	***Temperature(K):*** 193	***Density(g/cm³):*** 1.572		

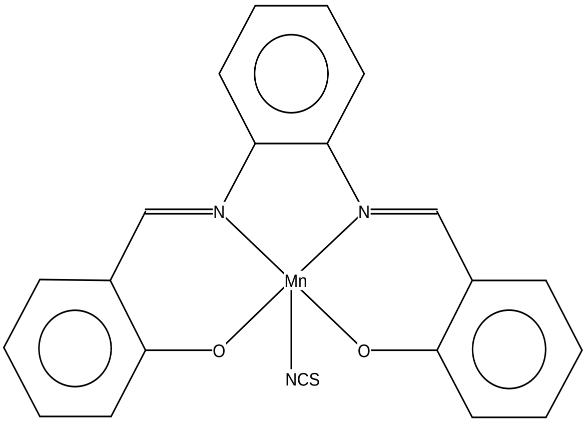

Search: search26 (Tue Dec 2 15:48:50 2014): Hits 317-320

KAKZUS

Reference:	S.Biswas, K.Mitra, C.H.Schwalbe, C.R.Lucas, S.K.Chattopadhyay, B.Adhikary (2005) *Inorg.Chim.Acta* ,**358,**2473
Formula:	C_{20} H_{16} Cl_1 Mn_1 N_2 O_7
Compound Name:	Aqua-(perchlorato-O)-(N,N'-o-phenylenebis(salicylideneimine))-manganese(iii)

Space Group:	P-1	***Cell:***	***a***	8.048(1)	***b***	11.206(1)	***c***	11.844(2)
Space Group No.:	2	***(Å,°)***	α	109.33(1)	β	98.56(1)	γ	93.36(1)
R-Factor (%):	3.84	***Temperature(K):***	293	***Density(g/cm³):***	1.633			

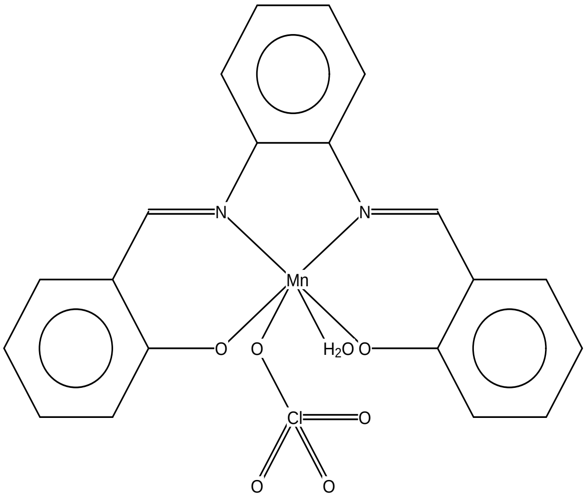

KENSOM

Reference:	J.W.Kramer, E.B.Lobkovsky, G.W.Coates (2006) *Org.Lett.* ,**8,**3709
Formula:	C_{44} H_{62} Cr_1 N_2 O_4 $^{1+}$,C_4 Co_1 O_4 $^{1-}$
Compound Name:	(N,N'-bis(3,5-di-t-butylsalicylidene)-1,2-phenylenediamino)-bis(tetrahydrofuran)-chromium tetracarbonylcobalt(-1)

Space Group:	P-1	***Cell:***	***a***	11.972(1)	***b***	13.290(1)	***c***	15.310(1)
Space Group No.:	2	***(Å,°)***	α	101.63(0)	β	91.25(0)	γ	90.41(0)
R-Factor (%):	4.18	***Temperature(K):***	173	***Density(g/cm³):***	1.261			

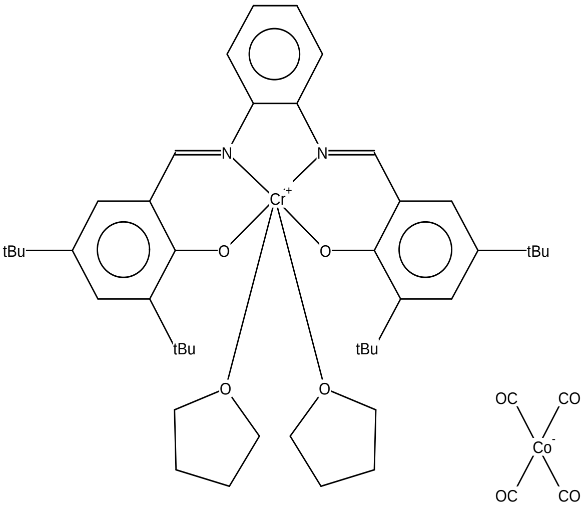

KESPAA

Reference:	L.Salmon, P.Thuery, T.Yamato, M.Ephritikhine (2006) *Private Communication ,*
Formula:	C_{60} H_{64} Cl_4 N_4 O_4 U_2,2(C_4 H_8 O_1)
Compound Name:	bis(μ_2-Chloro)-(μ_2-N,N:N',N'-bis(2,2'-dioxy-3,3'-dimethylidene-5,5'-di-t-butylbiphenyl)bis(4,5-dimethylbenzene-1,2-diamine))-dichloro-di-uranium tetrahydrofuran solvate

Space Group:	R-3	***Cell:***	***a***	40.486(2)	***b***	40.486(2)	***c***	10.755(0)
Space Group No.:	148	***(Å,°)***	α	90.00	β	90.00	γ	120.00
R-Factor (%):	3.20	***Temperature(K):***	100	***Density(g/cm³):***	1.632			

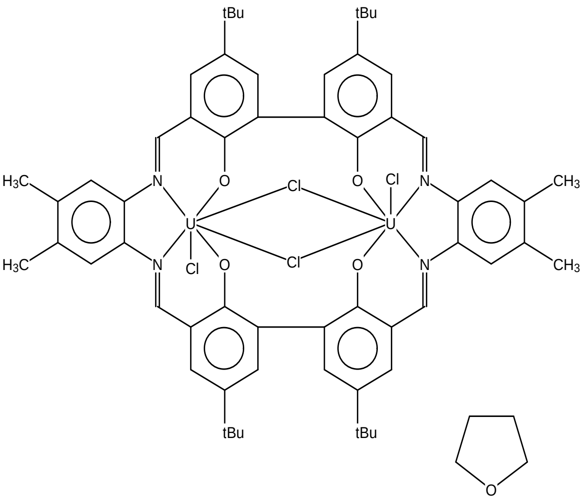

KESPEE

Reference:	L.Salmon, P.Thuery, M.Ephritikhine (2006) *Private Communication ,*
Formula:	C_{72} H_{80} F_{12} N_4 O_{18} S_4 U_2,2(C_4 H_8 O_1)
Compound Name:	bis(μ_2-Trifluoromethanesulfonato-O,O')-(μ_2-N,N:N',N'-bis(2,2'-dioxy-3,3'-dimethylidene-5,5'-di-t-butylbiphenyl)bis(4,5-dimethylbenzene-1,2-diamine))-bis(tetrahydrofuran)-bis(trifluoromethanesulfonato-O)-di-uranium tetrahydrofuran solvate

Space Group:	P-1	***Cell:***	***a***	12.136(1)	***b***	14.212(1)	***c***	15.371(1)
Space Group No.:	2	***(Å,°)***	α	106.07(0)	β	111.24(0)	γ	105.30(0)
R-Factor (%):	4.70	***Temperature(K):***	100	***Density(g/cm³):***	1.734			

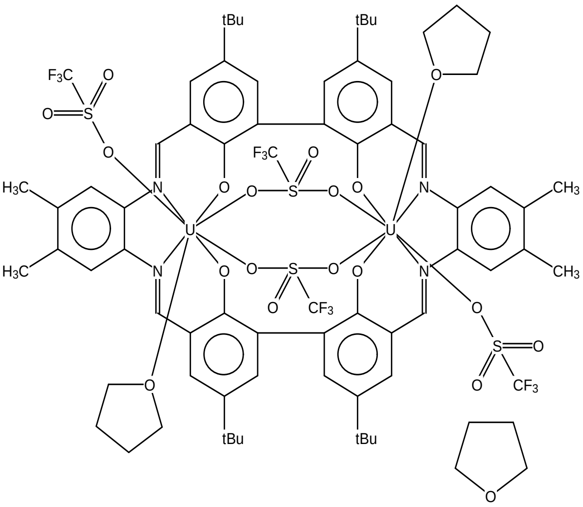

Search: search26 (Tue Dec 2 15:48:50 2014): Hits 321-324

KESWIP

Reference: Zhenzhen Dong, R.J.Karpowicz Junior, Shi Bai, G.P.A.Yap, J.M.Fox (2006) *J.Am.Chem.Soc.* ,**128,**14242

Formula: $C_{78} H_{90} N_6 Ni_1 O_8$

Compound Name: (N,N'-(Benzene-1,2-diyl-bis((nitrilo)methylylidene(2-(hydroxy)benzene-1,3-diyl)carbonylimino(3,4-dihexylbenzene-1,6-diyl)))-bis(3-methyl-2,3-dihydro-1-benzofuran-7-carboxamidato))-nickel(ii)

Space Group:	P43212	***Cell:***	***a***	14.646(2)	***b***	14.646(2)	***c***	32.517(7)
Space Group No.:	96	***(Å,°)***	α	90.00	β	90.00	γ	90.00

R-Factor (%): 8.34 ***Temperature(K):*** 120 ***Density(g/cm^3):*** 1.236

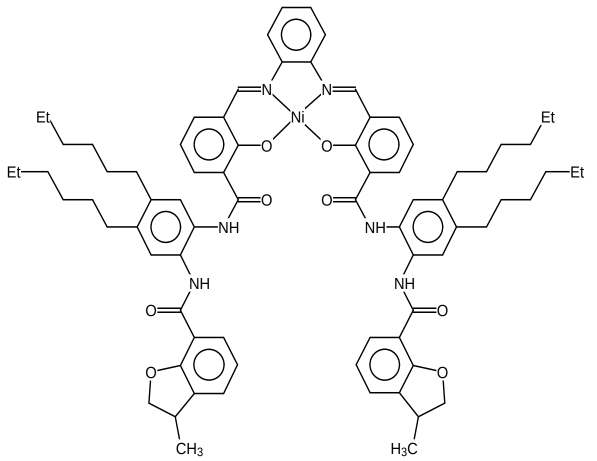

KESWUB

Reference: Zhenzhen Dong, R.J.Karpowicz Junior, Shi Bai, G.P.A.Yap, J.M.Fox (2006) *J.Am.Chem.Soc.* ,**128,**14242

Formula: $C_{80} H_{90} N_6 Ni_1 O_{12}$

Compound Name: (N,N'-(Benzene-1,2-diyl-bis((nitrilo)methylylidene(2-(hydroxy)benzene-1,3-diyl)carbonylimino(5-dodecyloxycarbonylbenzene-1,6-diyl)))-bis(3-methyl-2,3-dihydro-1-benzofuran-7-carboxamidato))-nickel(ii)

Space Group:	P43212	***Cell:***	***a***	14.755(2)	***b***	14.755(2)	***c***	32.076(6)
Space Group No.:	96	***(Å,°)***	α	90.00	β	90.00	γ	90.00

R-Factor (%): 5.64 ***Temperature(K):*** 120 ***Density(g/cm^3):*** 1.319

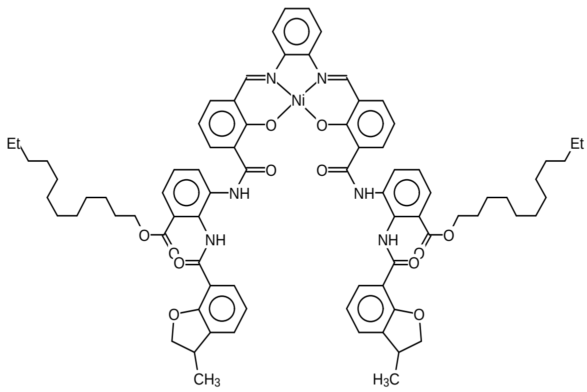

KESYOY

Reference: A.Biswas, S.Mondal, S.Mohanta (2013) *J.Coord.Chem.* , **66,**152

Formula: $C_{24} H_{21} Cl_1 Cu_1 N_3 Na_1 O_8$

Compound Name: (μ_2-2,2'-(1,2-Phenylenebis(nitrilomethylylidene))bis(6-methoxyphenolato))-perchlorato-acetonitrile-copper-sodium

Space Group:	P21/c	***Cell:***	***a***	12.001(1)	***b***	16.872(2)	***c***	16.576(1)
Space Group No.:	14	***(Å,°)***	α	90.00	β	132.05(0)	γ	90.00

R-Factor (%): 2.91 ***Temperature(K):*** 296 ***Density(g/cm^3):*** 1.603

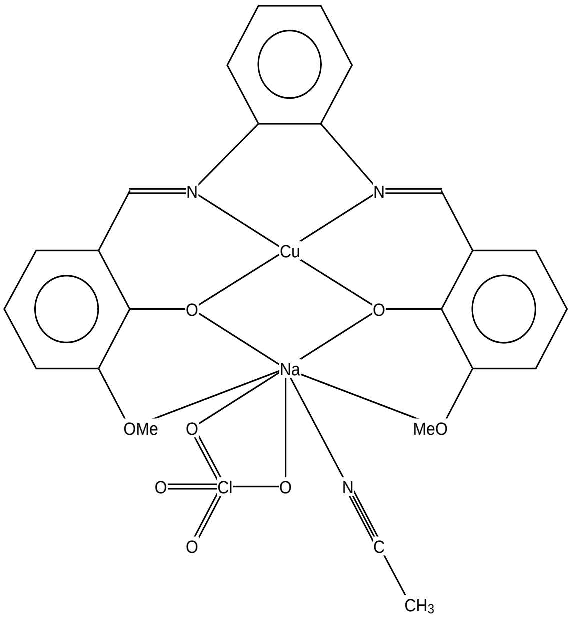

KESZAL

Reference: A.Biswas, S.Mondal, S.Mohanta (2013) *J.Coord.Chem.* , **66,**152

Formula: $C_{25} H_{24} Cl_1 Cu_1 K_1 N_2 O_9$

Compound Name: (μ_2-2,2'-(1,2-phenylenebis((nitrilo)methylylidene))bis(6-(methoxy)phenolato))-acetone-perchlorato-potassium-copper

Space Group:	P21/c	***Cell:***	***a***	7.740(0)	***b***	12.813(0)	***c***	26.646(1)
Space Group No.:	14	***(Å,°)***	α	90.00	β	94.28(0)	γ	90.00

R-Factor (%): 3.89 ***Temperature(K):*** 296 ***Density(g/cm^3):*** 1.599

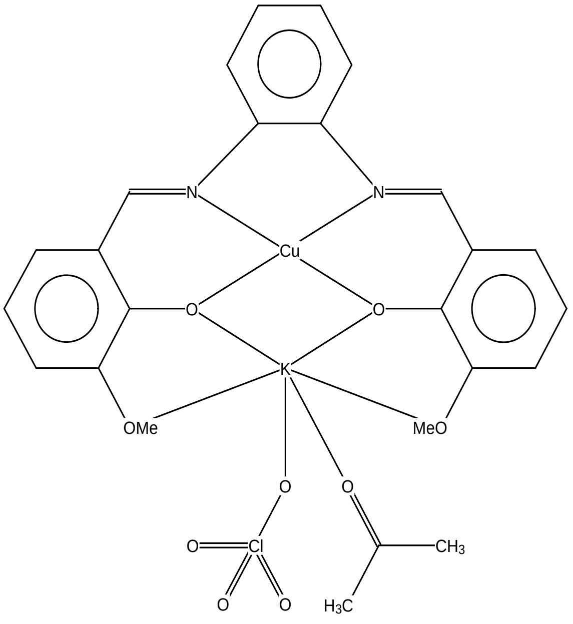

Search: search26 (Tue Dec 2 15:48:50 2014): Hits 325-328

KESZEP

Reference: A.Biswas, S.Mondal, S.Mohanta (2013) *J.Coord.Chem.* , **66,**152

Formula: $C_{44}\ H_{36}\ K_1\ N_4\ Ni_2\ O_8\ ^{1+}$,$Cl_1\ O_4\ ^{1-}$

Compound Name: bis(μ_2-2,2'-(1,2-Phenylenebis(nitrilomethylylidene))bis(6-methoxyphenolato))-di-nickel-potassium perchlorate

Space Group:	Pca21	***Cell:***	***a*** 19.508(0)	***b*** 12.141(0)	***c*** 17.709(0)
Space Group No.:	29	***(Å,°)***	α 90.00	β 90.00	γ 90.00

R-Factor (%): 4.68 ***Temperature(K):*** 296 ***Density(g/cm^3):*** 1.591

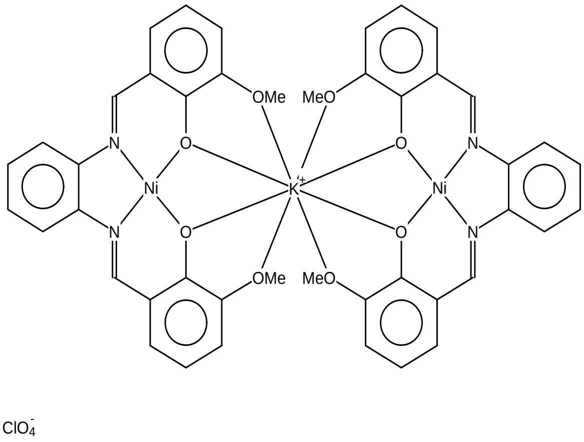

KESZIT

Reference: A.Biswas, S.Mondal, S.Mohanta (2013) *J.Coord.Chem.* , **66,**152

Formula: $C_{44}\ H_{36}\ Cl_1\ Cs_1\ Cu_2\ N_4\ O_{12}$,$2(C_2\ H_3\ N_1)$

Compound Name: bis(μ_2-2,2'-(1,2-phenylenebis(nitrilomethylylidene))bis(6-methoxyphenolato))-perchlorate-di-copper-cesium acetonitrile solvate

Space Group:	P21/c	***Cell:***	***a*** 13.620(0)	***b*** 24.278(0)	***c*** 14.405(0)
Space Group No.:	14	***(Å,°)***	α 90.00	β 92.88(0)	γ 90.00

R-Factor (%): 3.66 ***Temperature(K):*** 296 ***Density(g/cm^3):*** 1.662

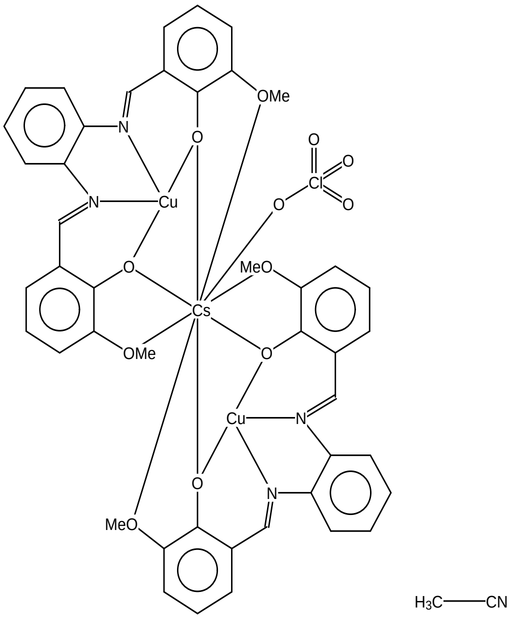

KESZOZ

Reference: A.Biswas, S.Mondal, S.Mohanta (2013) *J.Coord.Chem.* , **66,**152

Formula: $C_{44}\ H_{36}\ Cl_1\ Cs_1\ N_4\ Ni_2\ O_{12}$

Compound Name: bis(μ_2-2,2'-(1,2-phenylenebis(nitrilomethylylidene))bis(6-methoxyphenolato))-perchlorate-di-nickel-cesium unknown solvate

Space Group:	P21/c	***Cell:***	***a*** 14.033(3)	***b*** 14.122(3)	***c*** 25.468(4)
Space Group No.:	14	***(Å,°)***	α 90.00	β 118.58(0)	γ 90.00

R-Factor (%): 4.39 ***Temperature(K):*** 296 ***Density(g/cm^3):*** 1.646

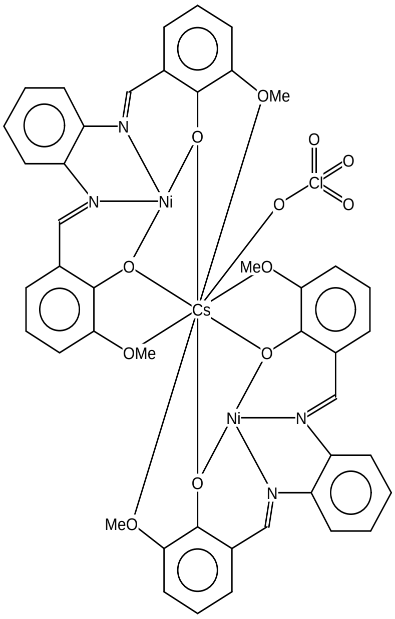

KIGJAM

Reference: A.A.Khandar, B.Shaabani, F.Belaj, A.Bakhtiari (2007) *Inorg.Chim.Acta* ,**360,**3255

Formula: $C_{32}\ H_{28}\ Co_1\ N_4\ O_2\ ^{1+}$,$Cl_1\ O_4\ ^{1-}$,$C_1\ H_2\ Cl_2$

Compound Name: bis(4-Methylpyridine-N)-((2,2'-(1,2-phenylenebis(nitrilomethylidyne)) bis(phenolato))-N,N',O,O')-cobalt(iii) perchlorate dichloromethane solvate

Space Group:	Pbca	***Cell:***	***a*** 19.868(4)	***b*** 16.403(3)	***c*** 20.474(5)
Space Group No.:	61	***(Å,°)***	α 90.00	β 90.00	γ 90.00

R-Factor (%): 6.49 ***Temperature(K):*** 95 ***Density(g/cm^3):*** 1.481

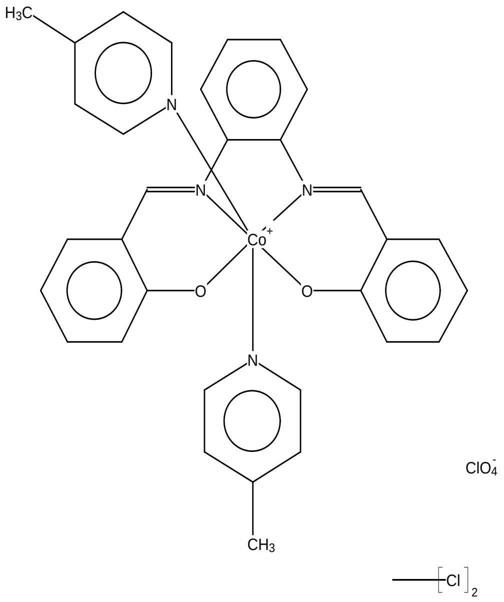

Search: search26 (Tue Dec 2 15:48:50 2014): Hits 329-332

KIRHEY

Reference: A.R.van Doorn, M.Bos, S.Harkema, J.van Eerden, W.Verboom, D.N.Reinhoudt (1991) *J.Org.Chem.* ,**56,**2371

Formula: $C_{43}\ H_{39}\ N_3\ O_6\ U_1$

Compound Name: (4-t-Butylpyridine)-(3,3'-bis(2-methoxyphenyl)-N,N'-phenylenebis(salicylideneaminato)-N,N',O,O')-dioxo-uranium

Space Group:	P-1	***Cell:***	***a*** 9.970(3)	***b*** 15.175(3)	***c*** 12.623(5)
Space Group No.:	2	***(Å,°)***	α 92.13(1)	β 100.46(1)	γ 92.60(1)

R-Factor (%): 13.40 ***Temperature(K):*** 295 ***Density(g/cm³):*** 1.651

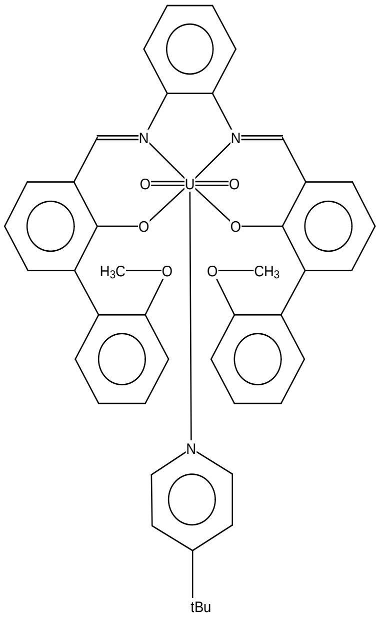

KIWNEJ

Reference: A.Elmali, Y.Elerman, I.Svoboda (2000) *Acta Crystallogr.,Sect.C:Cryst.Struct.Commun.* ,**56,**423

Formula: $C_{20}\ H_{12}\ Br_2\ Cu_1\ N_2\ O_2$

Compound Name: (N,N'-bis(5-Bromosalicylidene)-o-phenylenediaminato)-copper(ii)

Space Group:	P21/n	***Cell:***	***a*** 12.114(1)	***b*** 8.095(2)	***c*** 18.538(6)
Space Group No.:	14	***(Å,°)***	α 90.00	β 106.30(1)	γ 90.00

R-Factor (%): 4.00 ***Temperature(K):*** 295 ***Density(g/cm³):*** 2.039

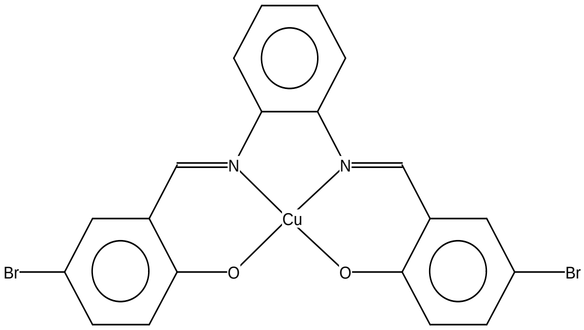

KOCMUK

Reference: E.Solari, S.De Angelis, C.Floriani, A.Chiesi-Villa, C.Rizzoli (1991) *J.Chem.Soc.,Dalton Trans.* ,2471

Formula: $C_{48}\ H_{48}\ N_4\ Na_4\ O_8$

Compound Name: bis(μ_4-N,N'-o-Phenylene-bis(salicylideneiminato))-bis(dimethoxyethane)-tetra-sodium

Space Group:	P21/c	***Cell:***	***a*** 12.045(1)	***b*** 25.851(2)	***c*** 16.467(1)
Space Group No.:	14	***(Å,°)***	α 90.00	β 107.61(1)	γ 90.00

R-Factor (%): 7.90 ***Temperature(K):*** 295 ***Density(g/cm³):*** 1.224

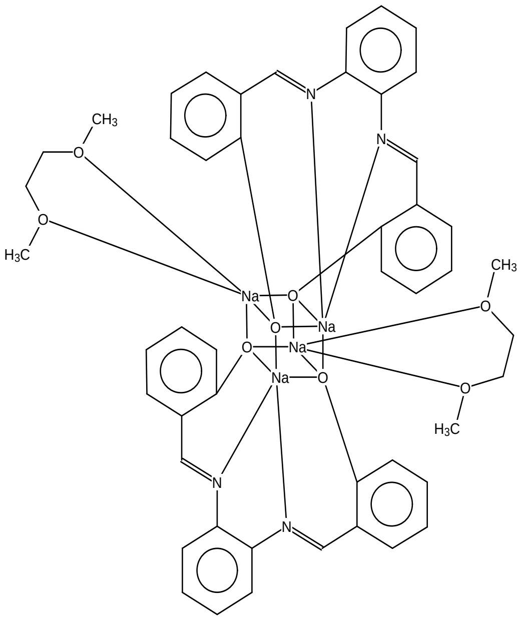

KOHWAF

Reference: E.Solari, C.Floriani, D.Cunningham, T.Higgins, P.McArdle (1991) *J.Chem.Soc.,Dalton Trans.* ,3139

Formula: $C_{60}\ H_{46}\ Co_3\ N_6\ O_6,0.66(H_2\ O_1)$

Compound Name: (μ_3-N,N'-o-Phenylene-bis(salicylideneaminato))-bis(μ_2-N,N'-o-phenylene-bis(salicylideneiminato))-tri-cobalt(iii) hydrate

Space Group:	C2/c	***Cell:***	***a*** 37.070(5)	***b*** 14.036(2)	***c*** 32.754(4)
Space Group No.:	15	***(Å,°)***	α 90.00	β 102.07(2)	γ 90.00

R-Factor (%): 8.40 ***Temperature(K):*** 295 ***Density(g/cm³):*** 1.358

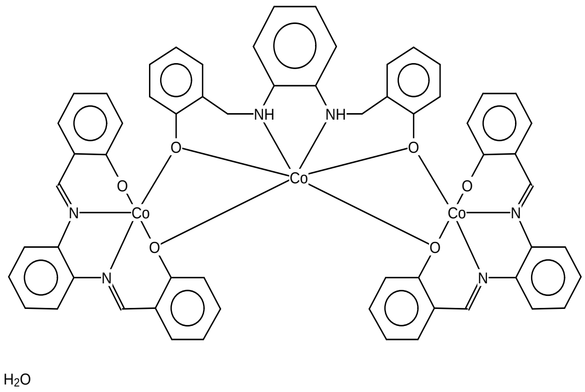

Search: search26 (Tue Dec 2 15:48:50 2014): Hits 333-336

KUBNAX

Reference: A.C.W.Leung, J.K.-H.Hui, J.H.Chong, M.J.MacLachlan (2009) *Dalton Trans.* ,5199

Formula: $C_{60}\ H_{78}\ N_4\ O_{10}\ Zn_2, C_1\ H_4\ O_1$

Compound Name: (μ_2-4,6-bis(4,5-bis(n-Hexyloxy)-2-(salicylideneamino) phenyliminomethyl)benzene-1,3-diolato)-bis(methanol)-di-zinc methanol solvate

Space Group:	P-1	***Cell:***	***a*** 10.363(1)	***b*** 17.347(3)	***c*** 17.738(3)		
Space Group No.:	2	***(Å,°)***	α 113.27(0)	β 91.31(0)	γ 91.65(0)		
R-Factor (%):	7.22	***Temperature(K):***	173	***Density(g/cm^3):***	1.337		

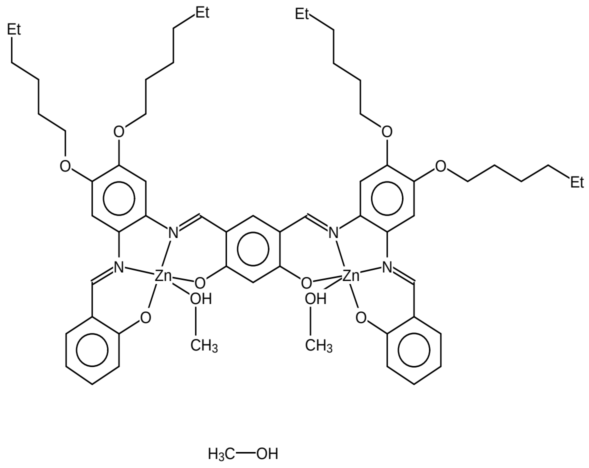

KUHYAO

Reference: S.Majumder, S.Hazra, S.Dutta, P.Biswas, S.Mohanta (2009) *Polyhedron* ,**28,**2473

Formula: $C_{25}\ H_{28}\ Mn_1\ N_2\ O_6\ ^{1+}, Cl_1\ O_4\ ^{1-}$

Compound Name: Aqua-(methanol)-(2,2'-(1,2-phenylenebis((nitrilo-N)methylylidene))-bis(6-ethoxyphenolato-O))-manganese(iii) perchlorate

Space Group:	P-1	***Cell:***	***a*** 9.714(3)	***b*** 11.971(4)	***c*** 13.107(7)
Space Group No.:	2	***(Å,°)***	α 112.86(1)	β 102.59(1)	γ 102.57(0)
R-Factor (%):	4.58	***Temperature(K):***	293	***Density(g/cm^3):***	1.561

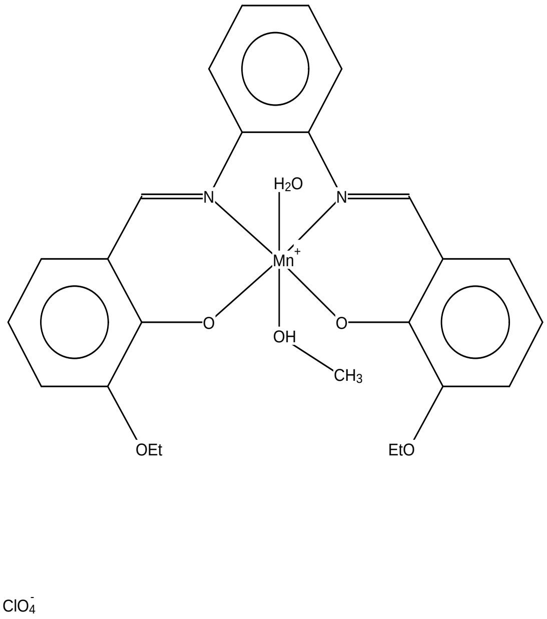

KUHYES

Reference: S.Majumder, S.Hazra, S.Dutta, P.Biswas, S.Mohanta (2009) *Polyhedron* ,**28,**2473

Formula: $C_{24}\ H_{24}\ Mn_1\ N_5\ O_5, C_3\ H_7\ N_1\ O_1$

Compound Name: Aqua-(azido)-(2,2'-(1,2-phenylenebis((nitrilo-N)methylylidene))-bis(6-ethoxyphenolato-O))-manganese(iii) dimethylformamide solvate

Space Group:	P21/c	***Cell:***	***a*** 12.428(1)	***b*** 15.503(1)	***c*** 15.147(1)
Space Group No.:	14	***(Å,°)***	α 90.00	β 105.19(0)	γ 90.00
R-Factor (%):	3.39	***Temperature(K):***	296	***Density(g/cm^3):***	1.393

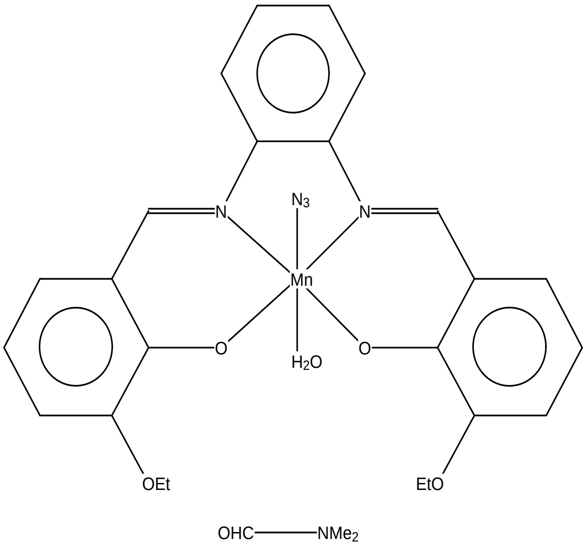

KUSCIL

Reference: A.Ali, N.Abdullah, M.J.Maah, K.M.Lo (2010) *Acta Crystallogr.,Sect.E:Struct.Rep.Online* ,**66,**m458

Formula: $C_{20}\ H_{10}\ Br_2\ Cl_2\ N_2\ Ni_1\ O_2$

Compound Name: (6,6'-Dibromo-4,4'-dichloro-2,2'-[o-phenylenebis(nitrilomethylidyne)]diphenolato)-nickel(ii)

Space Group:	P21/c	***Cell:***	***a*** 10.429(0)	***b*** 9.271(0)	***c*** 20.673(0)
Space Group No.:	14	***(Å,°)***	α 90.00	β 102.10(0)	γ 90.00
R-Factor (%):	3.77	***Temperature(K):***	296	***Density(g/cm^3):***	2.038

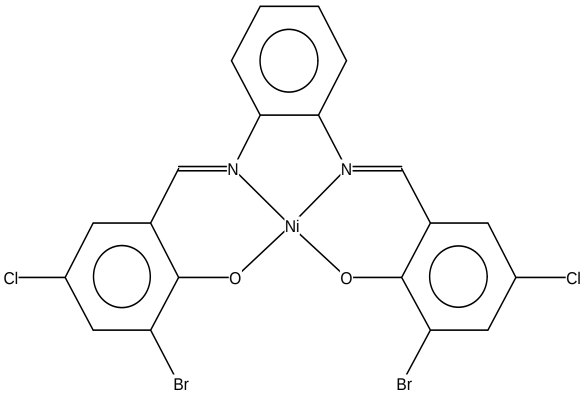

Search: search26 (Tue Dec 2 15:48:50 2014): Hits 337-340

LADCUO

Reference: D.M.Rudkevich, W.P.R.V.Stauthamer, W.Verboom, J.F.J.Engbersen, S.Harkema, D.N.Reinhoudt (1992) *J.Am.Chem.Soc.* , **114,**9671

Formula: $C_{20} H_{14} Cl_1 N_2 O_4 U_1{}^{1-}, C_8 H_{20} N_1{}^{1+}$

Compound Name: Tetraethylammonium chloro-dioxo-(o-phenylene-bis(salicylideneaminato))-uranium chloride

Space Group:	P21/n	***Cell:***	***a***	12.130(3)	***b***	17.196(5)	***c***	13.579(3)
Space Group No.:	14	***(Å,°)***	α	90.00	β	95.10(3)	γ	90.00

R-Factor (%): 7.70 ***Temperature(K):*** 295 ***Density(g/cm³):*** 1.766

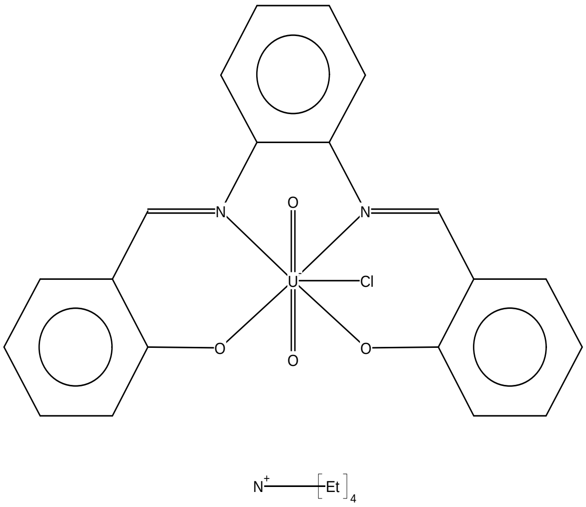

LALTOH

Reference: A.Elmali, Y.Elerman, I.Svoboda, H.Fuess (1993) *Acta Crystallogr.,Sect.C:Cryst.Struct.Commun.* ,**49,**1365

Formula: $C_{20} H_{14} Cl_1 Fe_1 N_2 O_2$

Compound Name: Chloro-(N,N'-(o-phenylene)-bis(salicylideneaminato))-iron(iii)

Space Group:	P1	***Cell:***	***a***	15.106(7)	***b***	13.570(5)	***c***	7.386(3)
Space Group No.:	1	***(Å,°)***	α	101.55(2)	β	94.18(2)	γ	92.49(2)

R-Factor (%): 4.66 ***Temperature(K):*** 295 ***Density(g/cm³):*** 1.368

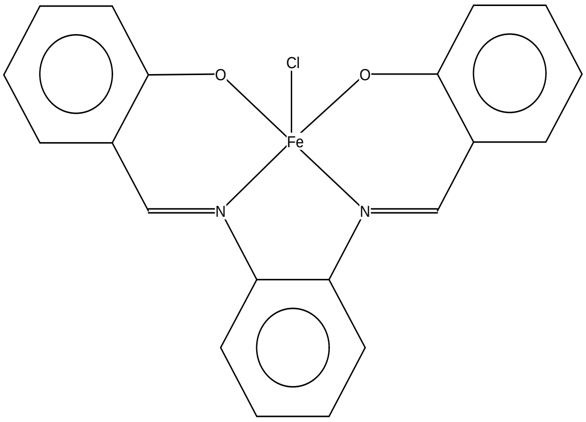

LAMBOR

Reference: R.Koner, Gene-Hsiang Lee, Yu Wang, Ho-Hsiang Wei, S.Mohanta (2005) *Eur.J.Inorg.Chem.* ,1500

Formula: $C_{24} H_{22} Cu_1 Gd_1 N_5 O_{13}$

Compound Name: (μ_2-N,N'-o-Phenylenebis(3-ethoxysalicylideneaminato))-trinitrato-copper(ii)-gadolinium(iii)

Space Group:	Pna21	***Cell:***	***a***	9.221(0)	***b***	16.541(0)	***c***	19.925(0)
Space Group No.:	33	***(Å,°)***	α	90.00	β	90.00	γ	90.00

R-Factor (%): 4.25 ***Temperature(K):*** 295 ***Density(g/cm³):*** 1.769

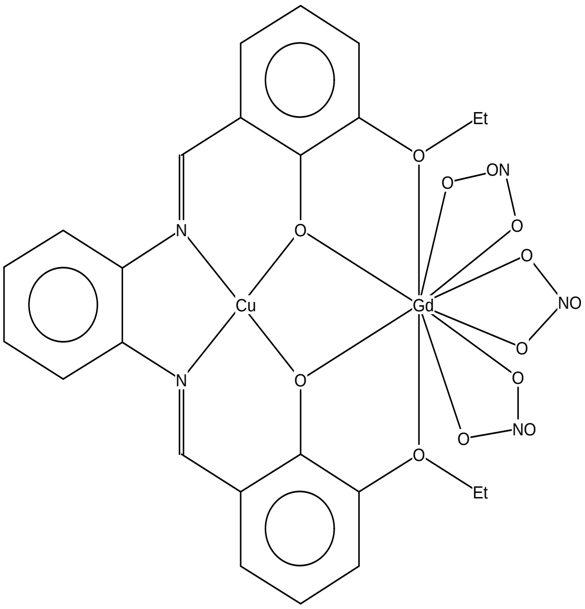

LAMROI

Reference: Yongling Sun (2012) *Acta Crystallogr.,Sect.E:Struct.Rep.Online* ,**68,**m282

Formula: $C_{22} H_{16} Br_2 N_2 Ni_1 O_4$

Compound Name: (2,2'-(1,2-Phenylenebis((nitrilo)methylylidene))bis(4-bromo-6-methoxyphenolato))-nickel(ii)

Space Group:	P21/n	***Cell:***	***a***	15.288(7)	***b***	8.213(3)	***c***	16.473(7)
Space Group No.:	14	***(Å,°)***	α	90.00	β	90.17(0)	γ	90.00

R-Factor (%): 6.47 ***Temperature(K):*** 293 ***Density(g/cm³):*** 1.898

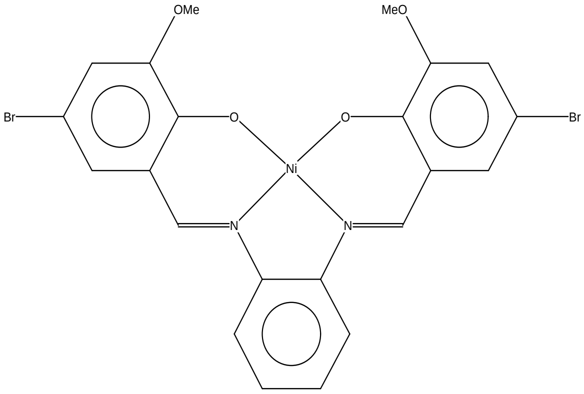

Search: search26 (Tue Dec 2 15:48:50 2014): Hits 341-344

LANWAY

Reference: P.A.Gosling, R.P.Sijbesma, A.L.Spek, R.J.M.Nolte (1993) *Rec.Trav.Chim.Pays-Bas(Fr.)(Rec.J.R.Neth.Chem.Soc.)* ,**112,**404

Formula: $C_{64} H_{50} N_8 Ni_2 O_{10}$

Compound Name: (μ_2-1,6:3,4-bis(N,N'-Disalicylidene-2,3-dimethylene-1,4-dimethoxy-5,6-phenylenediaminato)-tetrahydro-3a,6a-diphenylimidazo(4,5-d)imidazole-2,5(1H,3H)-dione)-di-nickel(ii)

Space Group: P-1 ***Cell:*** ***a*** 12.588(3) ***b*** 16.855(4) ***c*** 18.415(4)
Space Group No.: 2 ***(Å,°)*** α 73.10(2) β 76.96(2) γ 75.89(2)

R-Factor (%): 13.00 ***Temperature(K):*** 295 ***Density(g/cm3):*** 1.123

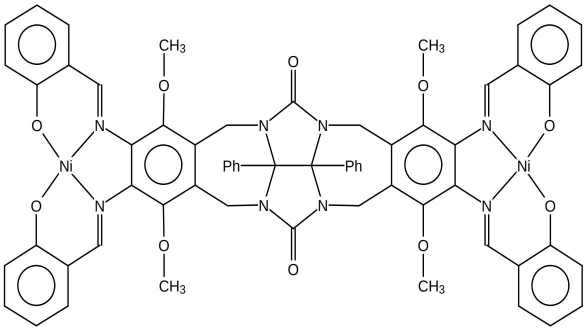

LAYDIA

Reference: A.Gutierrez, M.F.Perpinan, A.E.Sanchez, M.C.Torralba, M.R.Torres (2012) *Polyhedron* ,**44,**165

Formula: $C_{44} H_{34} Co_2 Mn_1 N_4 O_8$

Compound Name: bis(μ_2-Acetato-O,O')-bis(μ_2-2,2'-(1,2-phenylenebis(nitrilomethylylidene))diphenolato)-di-cobalt(ii)-manganese(ii)

Space Group: P-1 ***Cell:*** ***a*** 9.409(0) ***b*** 9.585(0) ***c*** 10.963(0)
Space Group No.: 2 ***(Å,°)*** α 94.59(0) β 104.23(0) γ 95.19(0)

R-Factor (%): 3.96 ***Temperature(K):*** 293 ***Density(g/cm3):*** 1.609

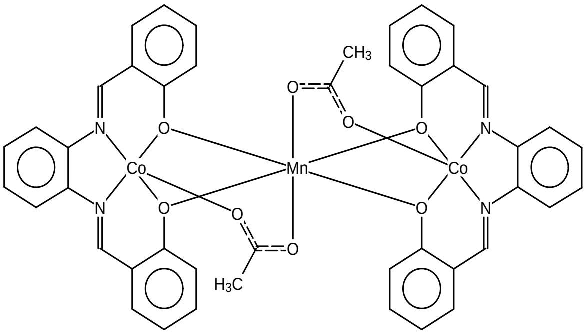

LAYDOG

Reference: A.Gutierrez, M.F.Perpinan, A.E.Sanchez, M.C.Torralba, M.R.Torres (2012) *Polyhedron* ,**44,**165

Formula: $C_{40} H_{28} Cl_2 Co_2 Mn_1 N_4 O_4$

Compound Name: bis(μ_2-Chloro)-bis(μ_2-2,2'-(1,2-phenylenebis(nitrilomethylylidene)) diphenolato)-di-cobalt(ii)-manganese(ii)

Space Group: P21/n ***Cell:*** ***a*** 15.205(1) ***b*** 13.977(0) ***c*** 16.228(1)
Space Group No.: 14 ***(Å,°)*** α 90.00 β 94.45(0) γ 90.00

R-Factor (%): 4.60 ***Temperature(K):*** 293 ***Density(g/cm3):*** 1.685

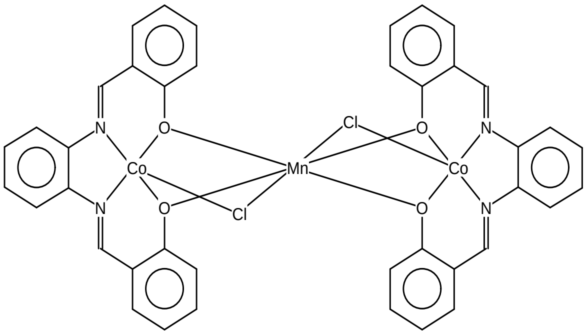

LAYDUM

Reference: A.Gutierrez, M.F.Perpinan, A.E.Sanchez, M.C.Torralba, M.R.Torres (2012) *Polyhedron* ,**44,**165

Formula: $C_{44} H_{34} Co_3 N_4 O_8$

Compound Name: bis(μ_2-Acetato-O,O')-bis(μ_2-2,2'-(1,2-phenylenebis(nitrilomethylylidene))diphenolato)-tri-cobalt(ii)

Space Group: P-1 ***Cell:*** ***a*** 9.384(1) ***b*** 9.546(1) ***c*** 10.869(1)
Space Group No.: 2 ***(Å,°)*** α 94.71(0) β 104.54(0) γ 94.79(0)

R-Factor (%): 3.97 ***Temperature(K):*** 293 ***Density(g/cm3):*** 1.642

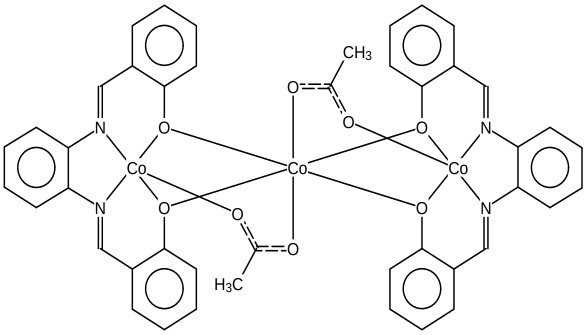

Search: search26 (Tue Dec 2 15:48:50 2014): Hits 345-348

LAYFAU

Reference: A.Gutierrez, M.F.Perpinan, A.E.Sanchez, M.C.Torralba, M.R.Torres (2012) *Polyhedron* ,**44,**165

Formula: $C_{22}\ H_{20}\ Co_1\ N_2\ O_3$

Compound Name: Ethanol-(2,2'-(1,2-phenylenebis(nitrilomethylylidene))diphenolato)-cobalt(ii)

Space Group:	Pnma	***Cell:***	***a***	19.913(3)	***b***	18.288(3)	***c***	5.174(0)
Space Group No.:	62	***(Å,°)***	α	90.00	β	90.00	γ	90.00

R-Factor (%): 5.55 ***Temperature(K):*** 293 ***Density(g/cm³):*** 1.478

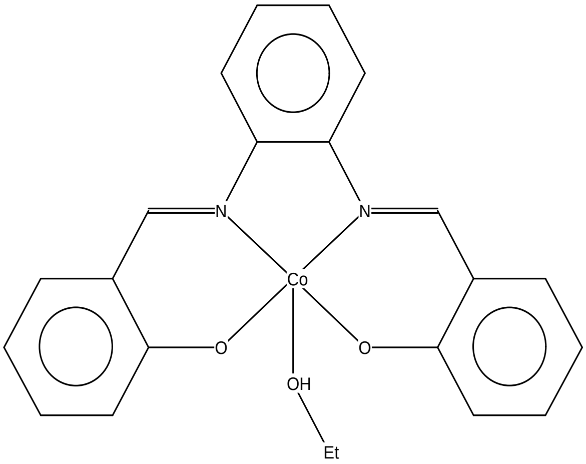

LESVAH

Reference: C.Ramirez de Arellano, T.Cuenca, M.Sanz (2006) *Private Communication ,*

Formula: $C_{36}\ H_{46}\ Cl_2\ N_2\ O_2\ Zr_1,0.5(C_1\ H_2\ Cl_2)$

Compound Name: Dichloro-(1,2-phenylene-bis(3,5-di-t-butylsalicylaldiminato))-zirconium dichloromethane solvate

Space Group:	P21	***Cell:***	***a***	11.455(2)	***b***	25.723(5)	***c***	13.447(3)
Space Group No.:	4	***(Å,°)***	α	90.00	β	104.05(3)	γ	90.00

R-Factor (%): 6.91 ***Temperature(K):*** 150 ***Density(g/cm³):*** 1.285

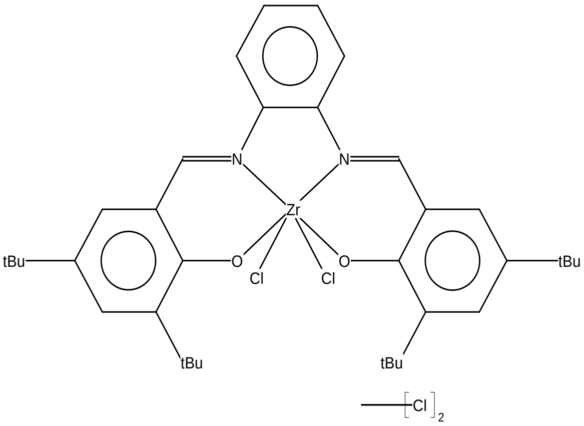

LEVCIA

Reference: Yanwei Ren, Yanchao Shi, Junxian Chen, Shaorong Yang, Chaorong Qi, Huanfeng Jiang (2013) *RSC Advances* , **3,**2167

Formula: $(C_{68}\ H_{72}\ Cd_2\ N_4\ Ni_2\ O_{16})n,3n(C_3\ H_7\ N_1\ O_1)$

Compound Name: catena-[(μ_5-3,3'-(1,2-Phenylenebis(nitrilomethylylidene(5-t-butyl-4-hydroxy-3,1-phenylene)))bisacrylato)-(μ_3-3,3'-(1,2-phenylenebis(nitrilomethylylidene(5-t-butyl-4-hydroxy-3,1-phenylene))) bisacrylato)-tetraaqua-di-cadmium-di-nickel dimethylformamide solvate]

Space Group:	P-1	***Cell:***	***a***	16.348(3)	***b***	16.635(3)	***c***	20.293(4)
Space Group No.:	2	***(Å,°)***	α	89.29(3)	β	87.12(3)	γ	68.48(3)

R-Factor (%): 8.10 ***Temperature(K):*** 293 ***Density(g/cm³):*** 1.142

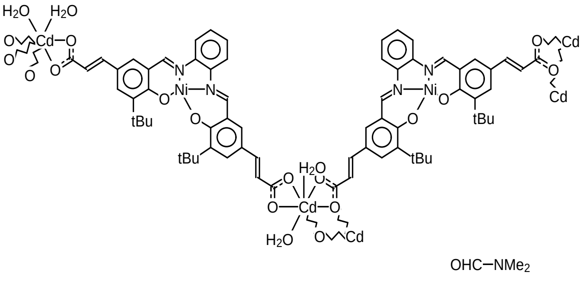

LICMEQ

Reference: N.E.Eltayeb, S.G.Teoh, Shea-Lin Ng, H.-K.Fun, K.Ibrahim (2007) *Acta Crystallogr.,Sect.E:Struct.Rep.Online* ,**63,**m1284

Formula: $C_{25}\ H_{19}\ N_3\ O_2\ Zn_1$

Compound Name: (2,2'-(1,2-Phenylenebis(nitrilomethylidyne))-diphenolato-κ⁴O,N,N',O')-(pyridine-κN)-zinc(ii)

Space Group:	Cc	***Cell:***	***a***	17.861(0)	***b***	11.993(0)	***c***	9.495(0)
Space Group No.:	9	***(Å,°)***	α	90.00	β	95.54(0)	γ	90.00

R-Factor (%): 4.52 ***Temperature(K):*** 100 ***Density(g/cm³):*** 1.505

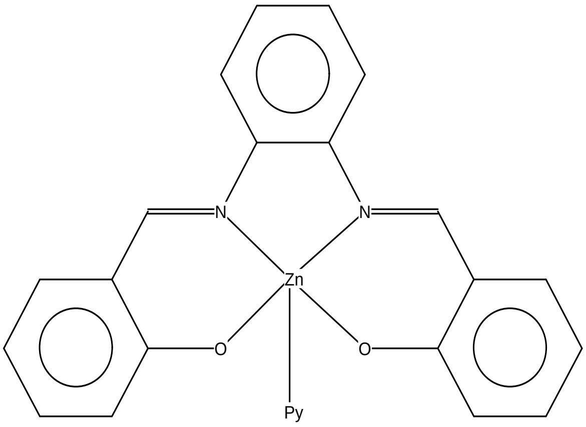

LINWOV

Reference: E.C.Escudero-Adan, J.Benet-Buchholz, A.W.Kleij (2007) *Inorg.Chem.* ,**46,**7265

Formula: $C_{39}\ H_{39}\ N_5\ O_2\ Zn_1$

Compound Name: Pyridyl-(N-(3,5-di-t-butyl-2-oxybenzylidene)-N'-(5-(phenylazo)-2-oxybenzylidene)phenylene-1,2-diamine)-zinc(ii)

Space Group:	P-1	***Cell:***	***a*** 7.334(0)	***b*** 10.842(0)	***c*** 22.876(0)
Space Group No.:	2	***(Å,°)***	α 100.66(0)	β 96.05(0)	γ 104.07(0)

R-Factor (%): 4.82 ***Temperature(K):*** 100 ***Density(g/cm^3):*** 1.310

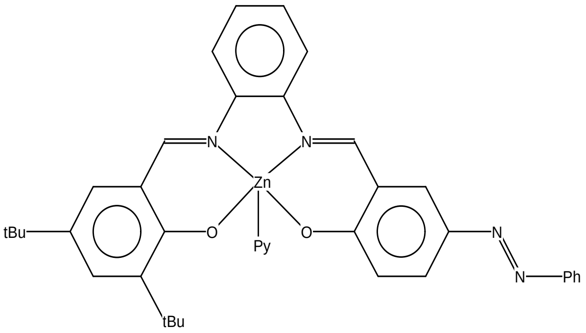

LOBQID

Reference: N.E.Eltayeb, S.G.Teoh, S.Chantrapromma, H.-K.Fun, R.Adnan (2008) *Acta Crystallogr.,Sect.E:Struct.Rep.Online* ,**64,**m738

Formula: $C_{31}\ H_{36}\ N_2\ O_3\ Zn_1$

Compound Name: (Acetone-κO)-(6,6'-di-t-butyl-2,2'-(1,2-phenylenebis(nitrilomethylidyne)) diphenolato-κ^{4}O,N,N',O')-zinc(ii)

Space Group:	C2/m	***Cell:***	***a*** 10.580(1)	***b*** 16.360(1)	***c*** 15.729(2)
Space Group No.:	12	***(Å,°)***	α 90.00	β 94.45(1)	γ 90.00

R-Factor (%): 7.32 ***Temperature(K):*** 100 ***Density(g/cm^3):*** 1.346

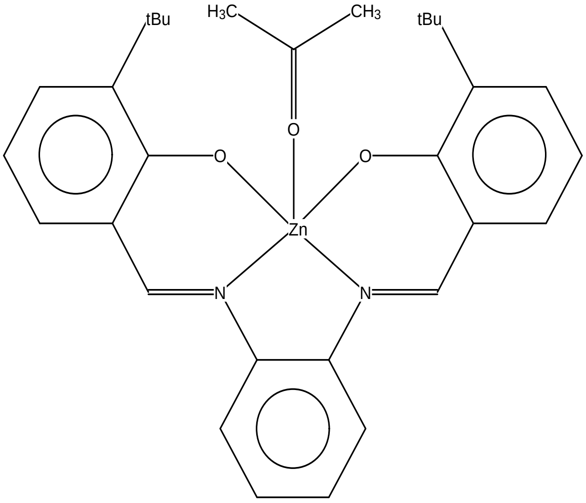

LOQKUY

Reference: A.Arola-Arnal, J.Benet-Buchholz, S.Neidle, R.Vilar (2008) *Inorg.Chem.* ,**47,**11910

Formula: $C_{34}\ H_{39}\ F_1\ N_4\ Ni_1\ O_4,1.25(C_7\ H_8)$

Compound Name: (N,N'-bis(4-(2-(Piperidin-1-yl)ethoxy)salicylidene)-4-fluorobenzene-1,2-diamine-N,N',O,O')-nickel(ii) toluene solvate

Space Group:	Iba2	***Cell:***	***a*** 26.765(4)	***b*** 31.067(4)	***c*** 9.584(1)
Space Group No.:	45	***(Å,°)***	α 90.00	β 90.00	γ 90.00

R-Factor (%): 8.21 ***Temperature(K):*** 100 ***Density(g/cm^3):*** 1.268

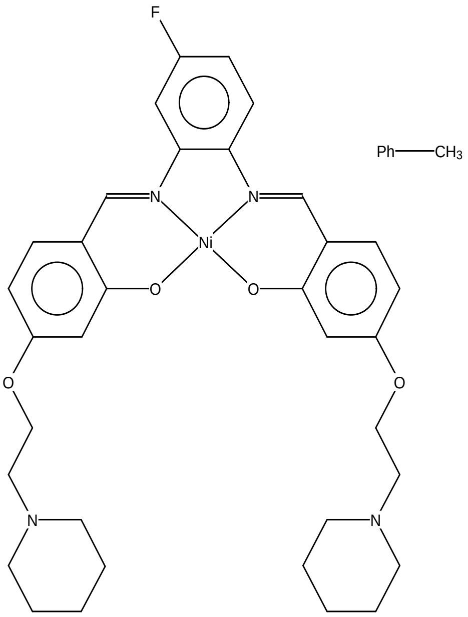

LOQLAF

Reference: A.Arola-Arnal, J.Benet-Buchholz, S.Neidle, R.Vilar (2008) *Inorg.Chem.* ,**47,**11910

Formula: $C_{20}\ H_{16}\ N_2\ O_5\ Zn_1,2.5(H_2\ O_1)$

Compound Name: Aqua-(N,N'-bis(4-hydroxysalicylidene)benzene-1,2-diamine-N,N',O,O')-zinc(ii) hydrate

Space Group:	P31	***Cell:***	***a*** 19.058(1)	***b*** 19.058(1)	***c*** 4.956(0)
Space Group No.:	144	***(Å,°)***	α 90.00	β 90.00	γ 120.00

R-Factor (%): 4.70 ***Temperature(K):*** 100 ***Density(g/cm^3):*** 1.517

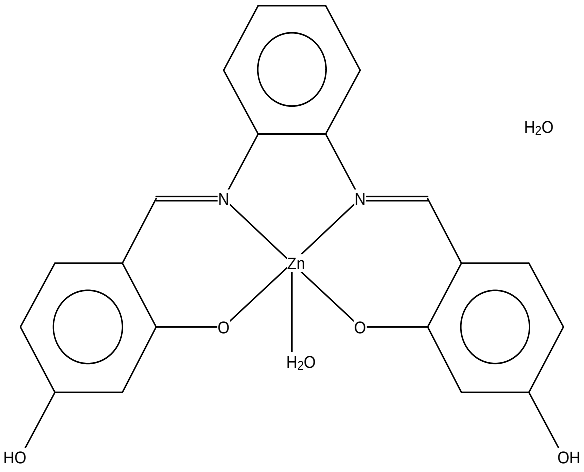

LUSLAN

Reference: P.D.Frischmann, G.A.Facey, P.Y.Ghi, A.J.Gallant, D.L.Bryce, F.Lelj, M.J.MacLachlan (2010) *J.Am.Chem.Soc.* ,**132,**3893

Formula: $C_{92} H_{120} Cd_7 N_8 O_{29}$,$C_{84} H_{108} Cd_7 N_6 O_{28}$,6.4($C_3 H_7 N_1 O_1$)

Compound Name: (μ_6-1^{4,5},7^{4,5},13^{4,5}-hexaethoxy-2,6,8,12,14,18-hexaaza-1,7,13(1,2)-tribenzena-4,10,16(1,4)-tribenzenacyclooctadecaphane-2,5,8,11,14,17-hexaene-4^{2,3},10^{2,3},16^{2,3}-hexaolato)-tris(μ_3-acetato)-(μ_3-hydroxo)-tris(μ_2-acetato)-hydroxy-diaqua-hepta-cadmium (μ_6-1^{4,5},7^{4,5},13^{4,5}-hexaethoxy-2,6,8,12,14,18-hexaaza-1,7,13(1,2)-tribenzena-4,10,16(1,4)-tribenzenacyclooctadecaphane-2,5,8,11,14,17-hexaene-4^{2,3},10^{2,3},16^{2,3}-hexaolato)-tris(μ_3-acetato)-(μ_3-hydroxo)-tris(μ_2-acetato)-bis(N,N-dimethylformamide)-acetato-hepta-cadmium N,N-dimethylformamide solvate

**Space Group:**	P-1	**Cell:**	***a*** 20.083(3)	***b*** 22.576(4)	***c*** 34.101(6)
**Space Group No.:**	2	**(Å,°)**	α 81.28(0)	β 88.26(0)	γ 82.51(0)

R-Factor (%): 11.58 **Temperature(K):** 173 **Density(g/cm³):** 1.204

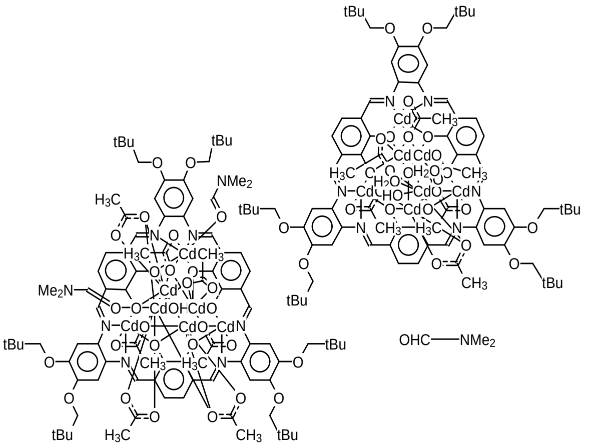

LUZPAX

Reference: J.D.Pike, D.T.Rosa, D.Coucouvanis (2001) *Eur.J.Inorg.Chem.* ,761

Formula: $C_{96} H_{144} Cs_1 Mn_2 N_4 O_{20}$ $^{3+}$,Cl_1 $^{1-}$,2(I_1 $^{1-}$)

Compound Name: bis((μ_2-4,5-bis(3,5-di-t-butylsalicylideneamino)benzo-18-crown-6)-aqua-ethanol-manganese(iii))-cesium chloride diiodide

**Space Group:**	C2/c	**Cell:**	***a*** 24.096(0)	***b*** 30.278(0)	***c*** 18.994(0)
**Space Group No.:**	15	**(Å,°)**	α 90.00	β 114.10(0)	γ 90.00

R-Factor (%): 17.25 **Temperature(K):** 158 **Density(g/cm³):** 1.158

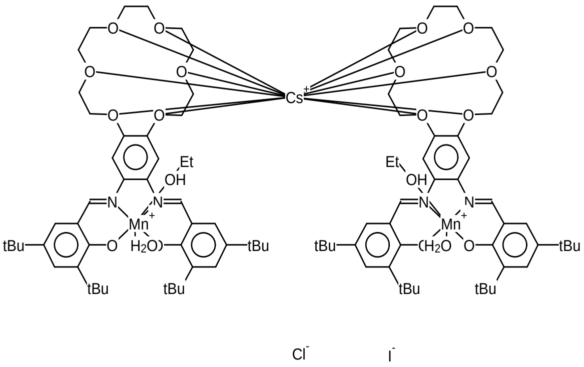

MAGSIX

Reference: F.Thomas, O.Jarjayes, C.Duboc, C.Philouze, E.Saint-Aman, J.-L.Pierre (2004) *Dalton Trans.* ,2662

Formula: $C_{36} H_{46} Cu_1 N_2 O_2$

Compound Name: (N,N'-o-Phenylene-bis(3,5-di-t-butylsalicylideneimine)-N,N',O,O')-copper(ii)

**Space Group:**	P-1	**Cell:**	***a*** 10.044(3)	***b*** 12.110(3)	***c*** 13.844(1)
**Space Group No.:**	2	**(Å,°)**	α 84.85(1)	β 88.64(1)	γ 70.95(1)

R-Factor (%): 3.73 **Temperature(K):** 150 **Density(g/cm³):** 1.262

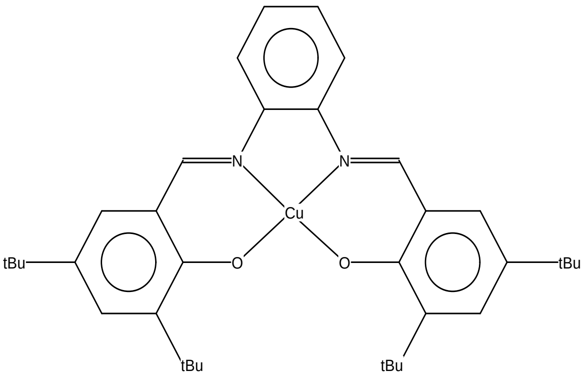

MAGSIX01

Reference: M.P.Weberski Junior, C.C.McLauchlan, C.G.Hamaker (2006) *Polyhedron* ,**25,**119

Formula: $C_{36} H_{46} Cu_1 N_2 O_2$

Compound Name: (N,N'-o-Phenylene-bis(3,5-di-t-butylsalicylideneaminato))-copper(ii)

**Space Group:**	P-1	**Cell:**	***a*** 10.047(7)	***b*** 12.069(8)	***c*** 13.847(6)
**Space Group No.:**	2	**(Å,°)**	α 85.11(4)	β 88.78(5)	γ 71.22(6)

R-Factor (%): 4.77 **Temperature(K):** 100 **Density(g/cm³):** 1.263

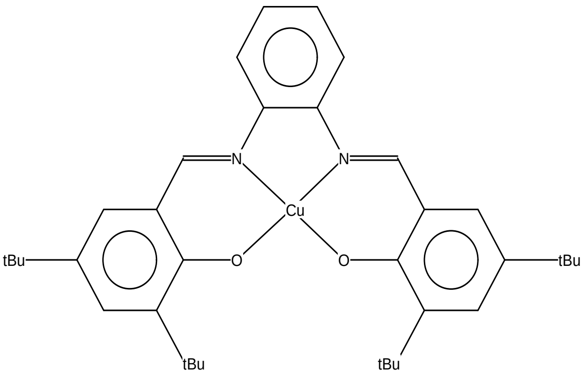

Search: search26 (Tue Dec 2 15:48:50 2014): Hits 357-360

MAHVIB

Reference: Ming-Jin Xie, Shi-Ping Yan, Dai-Zheng Liao, Zhong-Hui Jian, Peng Chen (2004) *Acta Crystallogr.,Sect.E:Struct.Rep.Online* ,**60,**m1530

Formula: $C_{20} H_{14} N_2 O_5 V_1,2(C_2 H_6 O_1 S_1),2(H_2 O_1)$

Compound Name: (N,N'-bis(4-Hydroxysalicylidene)-o-phenylenediamine)-oxo-vanadium(iv) bis(dimethyl sulfoxide) dihydrate

Space Group:	P-1	***Cell:***	***a*** 9.325(3)	***b*** 12.312(4)	***c*** 12.829(4)		
Space Group No.:	2	***(Å,°)***	α 78.46(0)	β 72.07(0)	γ 85.07(0)		
R-Factor (%):	6.08	***Temperature(K):***	293	***Density(g/cm³):***	1.465		

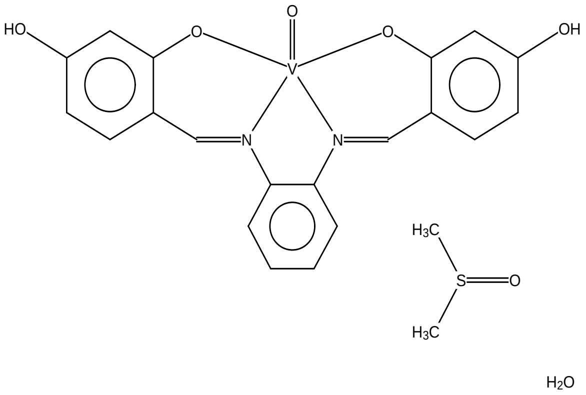

MAHVIB01

Reference: Ming-jin Xie, Ling Li, Xiao-Da Yang, Wei-ping Yan, Shi-ping Yan, Yan-fen Niu, Zhao-hui Meng (2010) *Eur.J.Med.Chem.* , **45,**2327

Formula: $C_{20} H_{14} N_2 O_5 V_1,2(C_2 H_6 O_1 S_1),2(H_2 O_1)$

Compound Name: (N,N'-bis(4-Hydroxysalicylidene)-o-phenylenediamine)-oxo-vanadium(iv) dimethyl sulfoxide solvate dihydrate

Space Group:	P-1	***Cell:***	***a*** 9.325(3)	***b*** 12.312(4)	***c*** 12.829(4)		
Space Group No.:	2	***(Å,°)***	α 78.46(0)	β 72.07(0)	γ 85.07(0)		
R-Factor (%):	6.08	***Temperature(K):***	293	***Density(g/cm³):***	1.465		

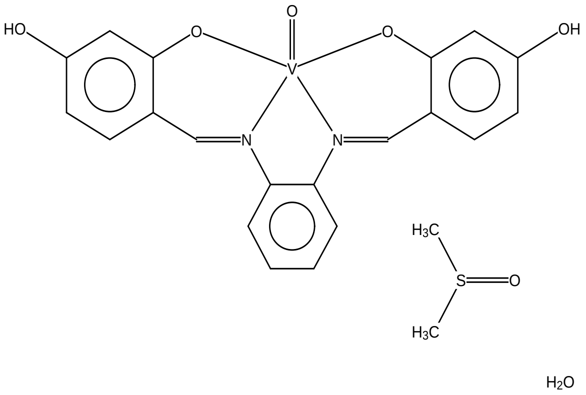

MAMMET

Reference: P.Plitt, H.Pritzkow, T.Oeser, R.Kraemer (2005) *J.Inorg.Biochem.* ,**99,**1230

Formula: $C_{32} H_{22} N_2 O_3 V_1$

Compound Name: (N,N'-bis(3-Phenyl-2-oxybenzylidene)o-phenylenediamine)-oxo-vanadium(iv) unknown solvate

Space Group:	P-1	***Cell:***	***a*** 9.630(3)	***b*** 12.328(5)	***c*** 12.788(4)		
Space Group No.:	2	***(Å,°)***	α 107.28(3)	β 95.17(2)	γ 106.55(1)		
R-Factor (%):	3.78	***Temperature(K):***	103	***Density(g/cm³):***	1.299		

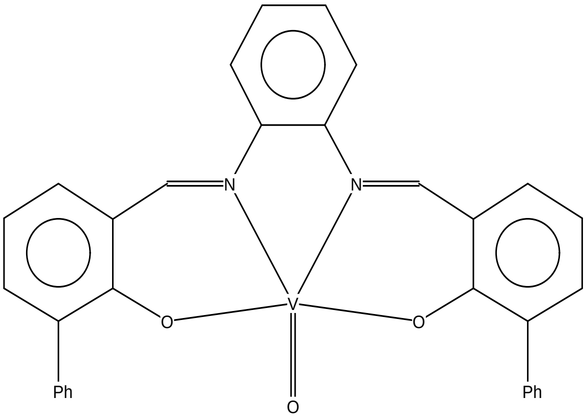

MAMMIX

Reference: P.Plitt, H.Pritzkow, T.Oeser, R.Kraemer (2005) *J.Inorg.Biochem.* ,**99,**1230

Formula: $C_{32} H_{22} Cu_1 N_2 O_4,2.5(C_2 H_6 O_1 S_1)$

Compound Name: (N,N'-bis(3-(2-Hydroxyphenyl)-2-oxybenzylidene)o-phenylenediamine)-copper(ii) dimethylsulfoxide solvate

Space Group:	C2/c	***Cell:***	***a*** 26.326(2)	***b*** 8.618(0)	***c*** 30.389(2)		
Space Group No.:	15	***(Å,°)***	α 90.00	β 99.81(0)	γ 90.00		
R-Factor (%):	7.13	***Temperature(K):***	100	***Density(g/cm³):***	1.481		

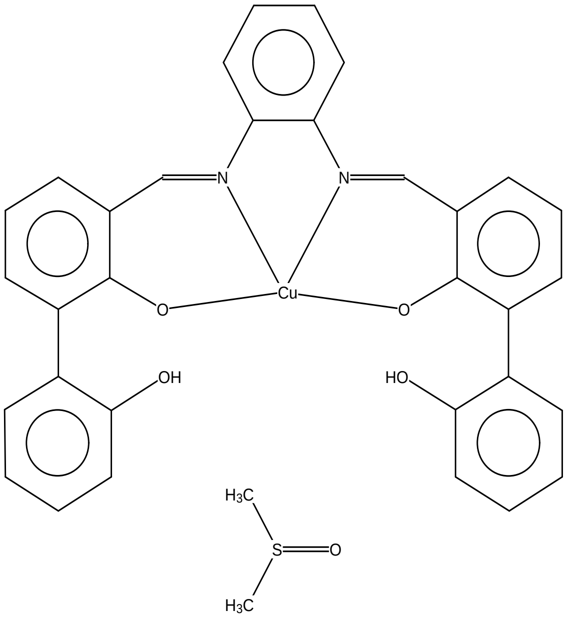

MAMMOD

Reference: P.Plitt, H.Pritzkow, T.Oeser, R.Kraemer (2005)
J.Inorg.Biochem. ,**99,**1230

Formula: $C_{32} H_{22} Cu_1 N_2 O_2$

Compound Name: (N,N'-bis(3-Phenyl-2-oxybenzylidene)o-phenylenediamine)-copper(ii)

Space Group: Pbca ***Cell:*** ***a*** 19.206(1) ***b*** 7.950(0) ***c*** 30.575(2)
Space Group No.: 61 ***(Å,°)*** α 90.00 β 90.00 γ 90.00

R-Factor (%): 4.08 ***Temperature(K):*** 103 ***Density(g/cm³):*** 1.508

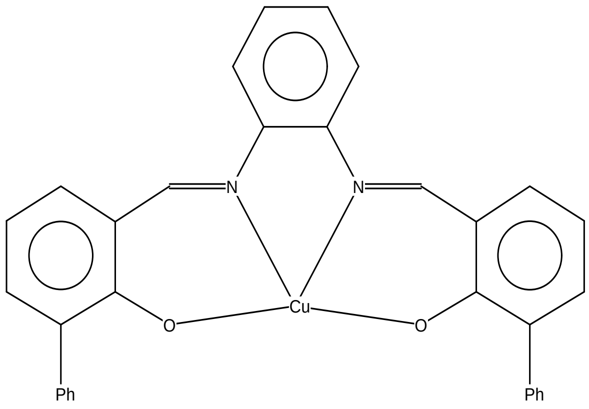

MAPDUD

Reference: Yu Wu, Zongqiu Hu, Mingtian Li, Xucheng Fu (2005)
Acta Crystallogr.,Sect.E:Struct.Rep.Online ,**61,**m1352

Formula: $C_{25} H_{15} Br_4 N_3 O_2 Zn_1, C_3 H_7 N_1 O_1$

Compound Name: Pyridine-(4,4',6,6'-tetrabromo-2,2'-(1,2-phenylene-bis(nitrilomethylidyne)) diphenolato)-zinc(ii) dimethylformamide solvate

Space Group: P21/c ***Cell:*** ***a*** 8.229(0) ***b*** 15.281(1) ***c*** 23.793(3)
Space Group No.: 14 ***(Å,°)*** α 90.00 β 95.95(0) γ 90.00

R-Factor (%): 4.45 ***Temperature(K):*** 292 ***Density(g/cm³):*** 1.892

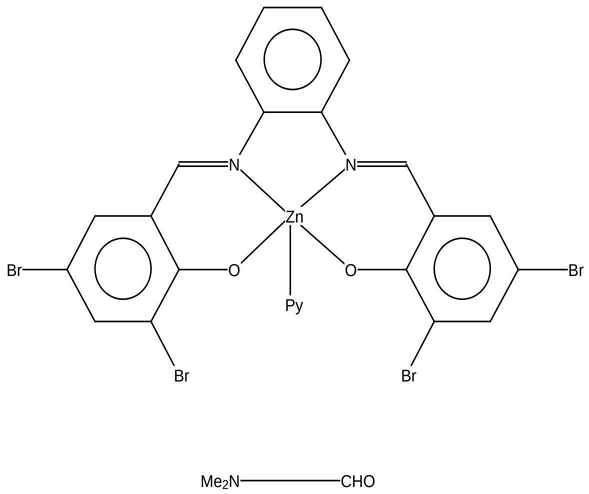

MARCAK

Reference: D.J.Darensbourg, P.Ganguly, D.Billodeaux (2005)
Macromolecules ,**38,**5406

Formula: $C_{20} H_{10} Cl_6 N_2 O_2 Sn_1, 2(C_4 H_8 O_1)$

Compound Name: Dichloro-(N,N-bis(3,5-dichlorosalicylidene)-1,2-phenylenediamine)-tin(iv) tetrahydrofuran solvate

Space Group: P21/n ***Cell:*** ***a*** 13.713(3) ***b*** 14.784(3) ***c*** 15.649(3)
Space Group No.: 14 ***(Å,°)*** α 90.00 β 107.85(0) γ 90.00

R-Factor (%): 6.86 ***Temperature(K):*** 293 ***Density(g/cm³):*** 1.729

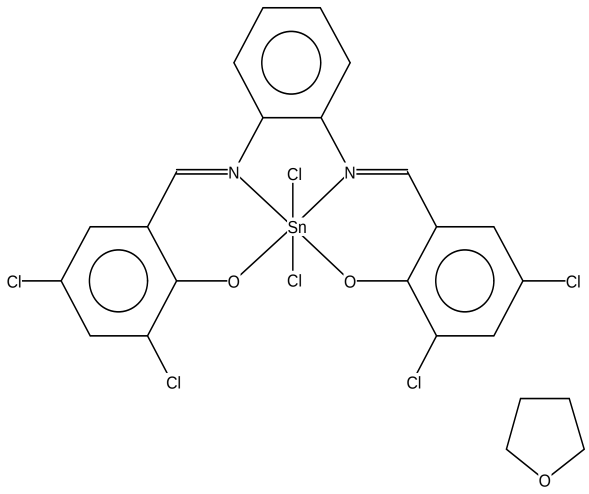

MARCEO

Reference: D.J.Darensbourg, P.Ganguly, D.Billodeaux (2005)
Macromolecules ,**38,**5406

Formula: $C_{24} H_{23} Cl_1 N_2 O_2 Sn_1$

Compound Name: n-Butyl-chloro-(N,N-bis(salicylidene)-1,2-phenylenediamine)-tin(iv)

Space Group: P21/c ***Cell:*** ***a*** 14.276(5) ***b*** 13.930(4) ***c*** 10.985(4)
Space Group No.: 14 ***(Å,°)*** α 90.00 β 94.39(0) γ 90.00

R-Factor (%): 4.18 ***Temperature(K):*** 295 ***Density(g/cm³):*** 1.603

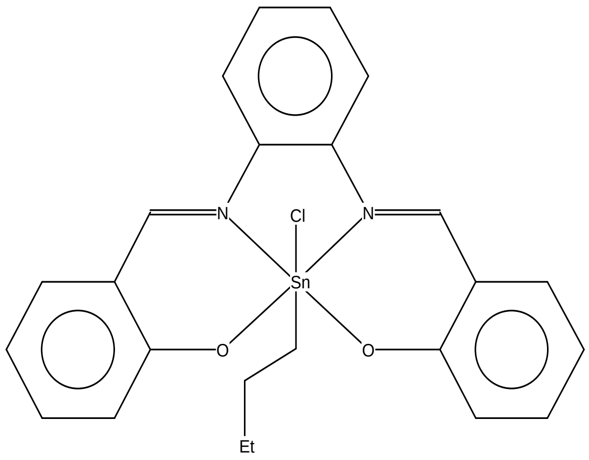

MATFIX

Reference: Fan Zhang, Shi Bai, G.P.A.Yap, V.Tarwade, J.M.Fox (2005) *J.Am.Chem.Soc.* ,**127,**10590

Formula: $C_{62}\,H_{62}\,N_6\,Ni_1\,O_8,C_1\,H_1\,Cl_3,C_2\,H_3\,N_1$

Compound Name: (4,5-Dihexyl-1,2-bis(2'-hydroxy-3'-(N-(2-(2-methoxybenzoyl) aminophenyl)carbamoyl)benzylideneamino)benzene)-nickel(ii) acetonitrile chloroform solvate

Space Group:	P212121	***Cell:***	***a***	15.742(2)	***b***	16.419(2)	***c***	23.119(3)
Space Group No.:	19	***(Å,°)***	α	90.00	β	90.00	γ	90.00

R-Factor (%): 6.70 ***Temperature(K):*** 120 ***Density(g/cm³):*** 1.377

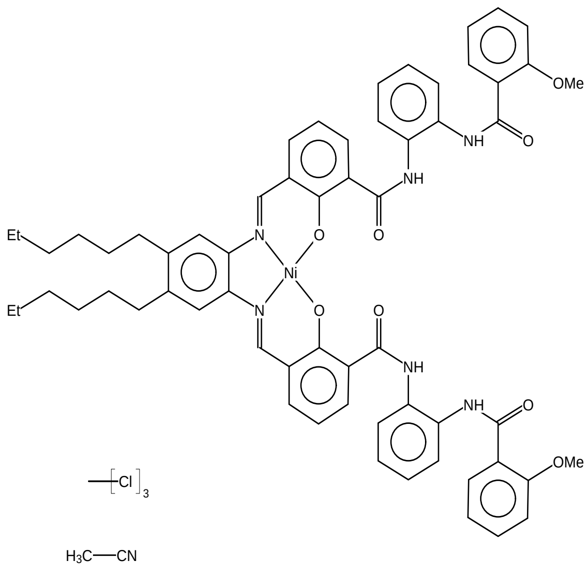

MATFOD

Reference: Fan Zhang, Shi Bai, G.P.A.Yap, V.Tarwade, J.M.Fox (2005) *J.Am.Chem.Soc.* ,**127,**10590

Formula: $C_{62}\,H_{62}\,Cu_1\,N_6\,O_8,2(C_1\,H_1\,Cl_3)$

Compound Name: (4,5-Dihexyl-1,2-bis(2'-hydroxy-3'-(N-(2-(2-methoxybenzoyl) aminophenyl)carbamoyl)benzylideneamino)benzene)-copper(ii) chloroform solvate

Space Group:	P212121	***Cell:***	***a***	15.745(3)	***b***	15.865(3)	***c***	24.245(5)
Space Group No.:	19	***(Å,°)***	α	90.00	β	90.00	γ	90.00

R-Factor (%): 5.95 ***Temperature(K):*** 120 ***Density(g/cm³):*** 1.449

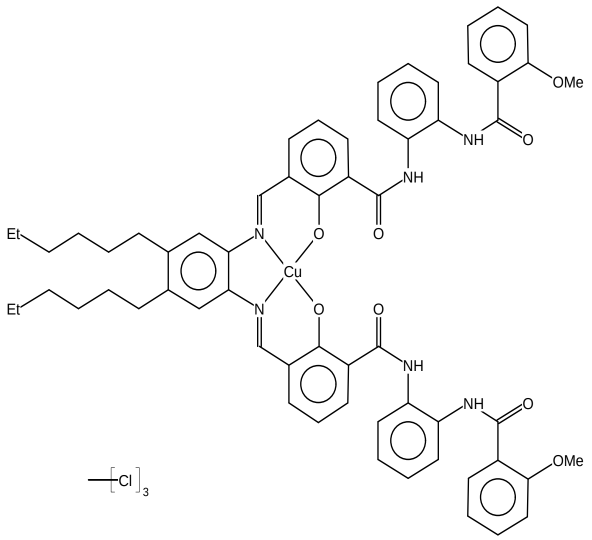

MAYDAR

Reference: F.Franceschi, E.Solari, R.Scopelliti, C.Floriani (2000) *Angew.Chem.,Int.Ed.* ,**39,**1685

Formula: $C_{66}\,H_{51}\,N_9\,Na_2\,Ni_3\,O_6,C_{36}\,H_{48}\,N_4\,Ni_1,3(C_2\,H_3\,N_1)$

Compound Name: (μ_3-N,N'-(o-Phenylene)-bis(salicylideneiminato))-bis(μ_2-N,N'-(o-phenylene)-bis(salicylideneiminato))-(μ_2-acetonitrile-N,N)-bis(acetonitrile-N)-tri-nickel-di-sodium (5,5,10,10,15,15,20,20-octaethylporphyrinogenato-nickel) acetonitrile solvate

Space Group:	Pna21	***Cell:***	***a***	20.868(3)	***b***	21.738(3)	***c***	20.969(1)
Space Group No.:	33	***(Å,°)***	α	90.00	β	90.00	γ	90.00

R-Factor (%): 9.26 ***Temperature(K):*** 143 ***Density(g/cm³):*** 1.401

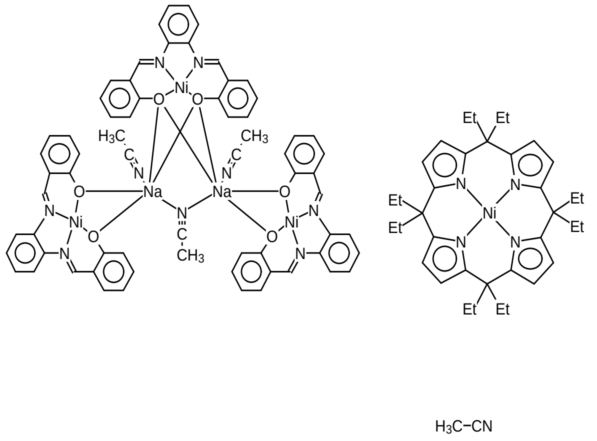

MAYMUW

Reference: G.Cosquer, F.Pointillart, B.Le Guennic, Y.Le Gal, S.Golhen, O.Cador, L.Ouahab (2012) *Inorg.Chem.* ,**51,**8488

Formula: $C_{45}\,H_{29}\,Cu_1\,F_{18}\,N_2\,O_8\,S_6\,Yb_1$

Compound Name: (μ_2-2,2'-((2-(4,5-bis(n-Propylsulfanyl)-1,3-dithiol-2-ylidene)-1,3-benzodithiole-5,6-diyl)bis(nitrilomethylylidene))diphenolato)-tris(1,1,1,5,5,5-hexafluoroacetylacetonato-O,O')-copper(ii)-ytterbium(iii)

Space Group:	P-1	***Cell:***	***a***	12.587(0)	***b***	13.728(0)	***c***	17.466(0)
Space Group No.:	2	***(Å,°)***	α	87.32(0)	β	88.10(0)	γ	66.92(0)

R-Factor (%): 4.56 ***Temperature(K):*** 293 ***Density(g/cm³):*** 1.792

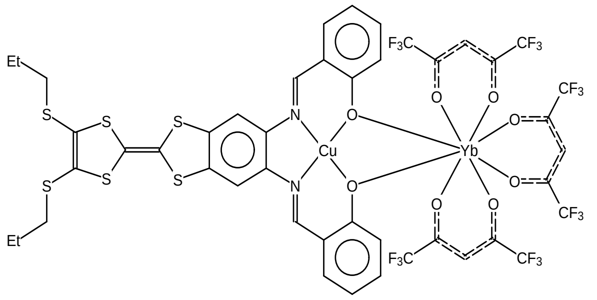

MAYNAD

Reference: G.Cosquer, F.Pointillart, B.Le Guennic, Y.Le Gal, S.Golhen, O.Cador, L.Ouahab (2012) *Inorg.Chem.* ,**51,**8488

Formula: $C_{45}\ H_{29}\ F_{18}\ N_2\ Ni_1\ O_8\ S_6\ Y_1$

Compound Name: (μ_2-2,2'-((2-(4,5-bis(n-Propylsulfanyl)-1,3-dithiol-2-ylidene)-1,3-benzodithiole-5,6-diyl)bis(nitrilomethylylidene))diphenolato)-tris(1,1,1,5,5,5-hexafluoroacetylacetonato-O,O')-nickel(ii)-yttrium(iii)

Space Group:	P-1	***Cell:***	***a***	13.277(0)	***b***	13.313(1)	***c***	16.510(1)
Space Group No.:	2	***(Å,°)***	α	85.51(0)	β	77.72(0)	γ	84.33(0)

R-Factor (%): 5.17 ***Temperature(K):*** 293 ***Density(g/cm³):*** 1.651

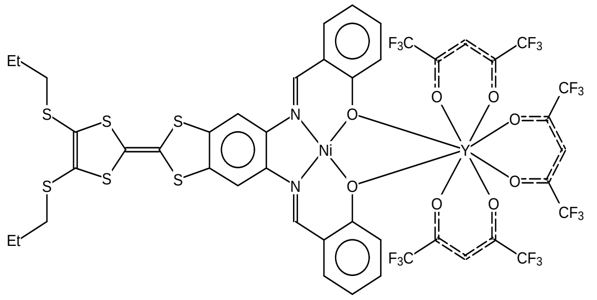

MEHQEV

Reference: M.-A.Munoz-Hernandez, B.Sannigrahi, D.A.Atwood (1999) *J.Am.Chem.Soc.* ,**121,**6747

Formula: $C_{68}\ H_{84}\ Al_2\ N_4\ O_4\ ^{2+}$,2($Cl_4\ Ga_1\ ^{1-}$),2($C_1\ H_2\ Cl_2$)

Compound Name: bis(μ_2-N-(3,5-Di-t-butyl-2-oxysalicylidene)-N'-(3-t-butyl-2-oxysalicylidene)-1,2-diamino-4,5-dimethylbenzene)-di-aluminium bis(tetrachlorogallate) dichloromethane solvate

Space Group:	P-1	***Cell:***	***a***	10.963(1)	***b***	11.589(1)	***c***	17.237(2)
Space Group No.:	2	***(Å,°)***	α	95.72(0)	β	103.28(0)	γ	97.42(0)

R-Factor (%): 8.21 ***Temperature(K):*** 295 ***Density(g/cm³):*** 1.323

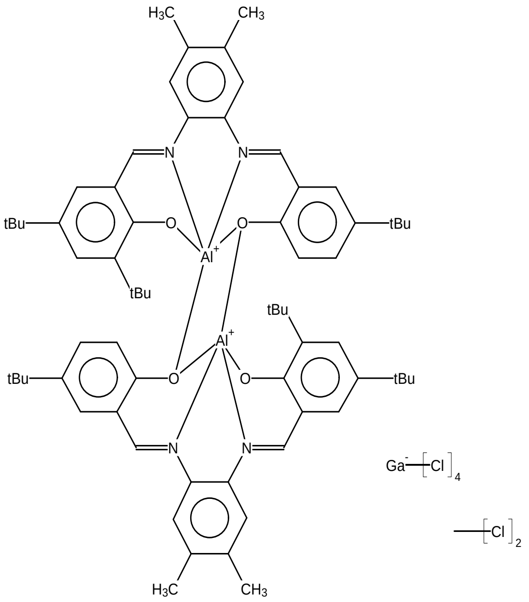

MEPXEL

Reference: Wing-Kit Lo, Wai-Kwok Wong, Wai-Yeung Wong, Jianping Guo, Kai-Tai Yeung, Yuen-Kit Cheng, Xiaoping Yang, R.A.Jones (2006) *Inorg.Chem.* ,**45,**9315

Formula: $C_{22}\ H_{22}\ La_1\ N_5\ O_{15}\ Zn_1$

Compound Name: (μ_2-N,N'-bis(3-Methoxysalicylidene)phenylene-1,2-diamine)-diaqua-tris(nitrato-O,O')-lanthanum(iii)-zinc(ii)

Space Group:	P212121	***Cell:***	***a***	13.799(0)	***b***	14.124(0)	***c***	14.339(0)
Space Group No.:	19	***(Å,°)***	α	90.00	β	90.00	γ	90.00

R-Factor (%): 2.01 ***Temperature(K):*** 293 ***Density(g/cm³):*** 1.903

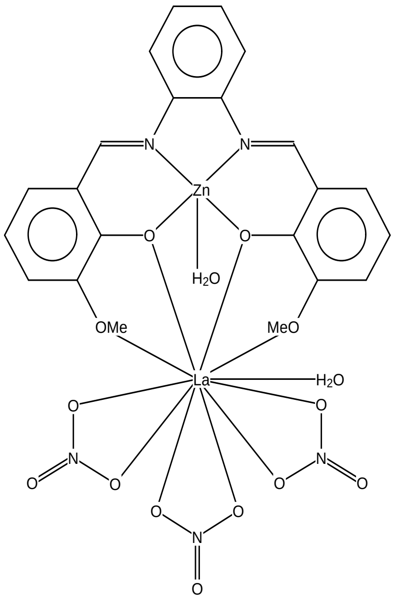

MEPXIP

Reference: Wing-Kit Lo, Wai-Kwok Wong, Wai-Yeung Wong, Jianping Guo, Kai-Tai Yeung, Yuen-Kit Cheng, Xiaoping Yang, R.A.Jones (2006) *Inorg.Chem.* ,**45,**9315

Formula: $C_{24}\ H_{24}\ N_5\ Nd_1\ O_{14}\ Zn_1$

Compound Name: (μ_2-N,N'-bis(3-Methoxysalicylidene)phenylene-1,2-diamine)-(μ_2-nitrato-O,O')-(ethanol)-bis(nitrato-O,O')-neodymium(iii)-zinc(ii)

Space Group:	P-1	***Cell:***	***a***	9.573(1)	***b***	12.557(1)	***c***	13.714(1)
Space Group No.:	2	***(Å,°)***	α	86.35(0)	β	73.59(0)	γ	67.87(0)

R-Factor (%): 5.57 ***Temperature(K):*** 293 ***Density(g/cm³):*** 1.852

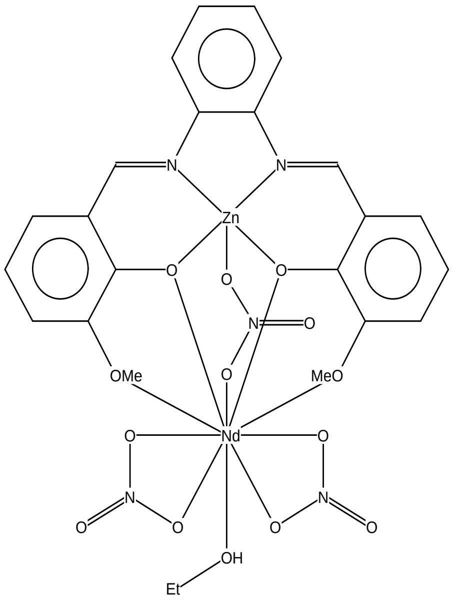

MEPXOV

Reference: Wing-Kit Lo, Wai-Kwok Wong, Wai-Yeung Wong, Jianping Guo, Kai-Tai Yeung, Yuen-Kit Cheng, Xiaoping Yang, R.A.Jones (2006) *Inorg.Chem.* ,**45,**9315

Formula: $C_{24}\ H_{24}\ Er_1\ N_5\ O_{14}\ Zn_1$

Compound Name: (μ_2-N,N'-bis(3-Methoxysalicylidene)phenylene-1,2-diamine)-(ethanol)-bis(nitrato-O,O')-(nitrato-O)-erbium(iii)-zinc(ii)

Space Group:	P21/c	***Cell:***	***a*** 15.906(1)	***b*** 9.163(0)	***c*** 20.528(1)		
Space Group No.:	14	***(Å,°)***	α 90.00	β 106.69(0)	γ 90.00		
R-Factor (%):	4.46	***Temperature(K):***	293	***Density(g/cm³):***	1.945		

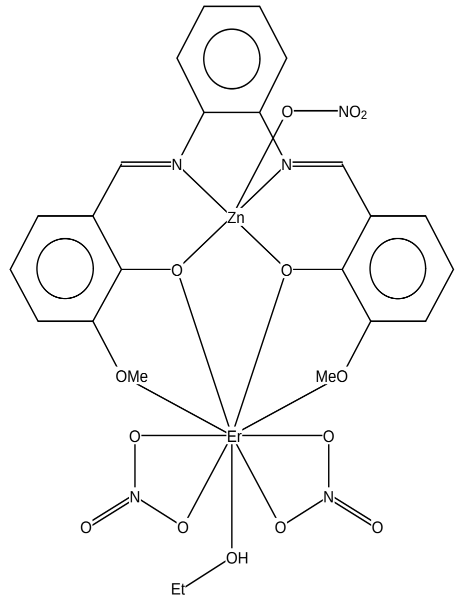

MEPXUB

Reference: Wing-Kit Lo, Wai-Kwok Wong, Wai-Yeung Wong, Jianping Guo, Kai-Tai Yeung, Yuen-Kit Cheng, Xiaoping Yang, R.A.Jones (2006) *Inorg.Chem.* ,**45,**9315

Formula: $C_{36}\ H_{32}\ N_5\ Nd_1\ O_{14}\ Zn_1,C_2\ H_3\ N_1$

Compound Name: (μ_2-N,N'-bis(3-Methoxy-5-(p-tolyl)salicylidene)phenylene-1,2-diamine)-aqua-tris(nitrato-O,O')-neodymium(iii)-zinc(ii) acetonitrile solvate

Space Group:	Fdd2	***Cell:***	***a*** 50.377(3)	***b*** 16.419(0)	***c*** 19.360(1)		
Space Group No.:	43	***(Å,°)***	α 90.00	β 90.00	γ 90.00		
R-Factor (%):	3.83	***Temperature(K):***	293	***Density(g/cm³):***	1.675		

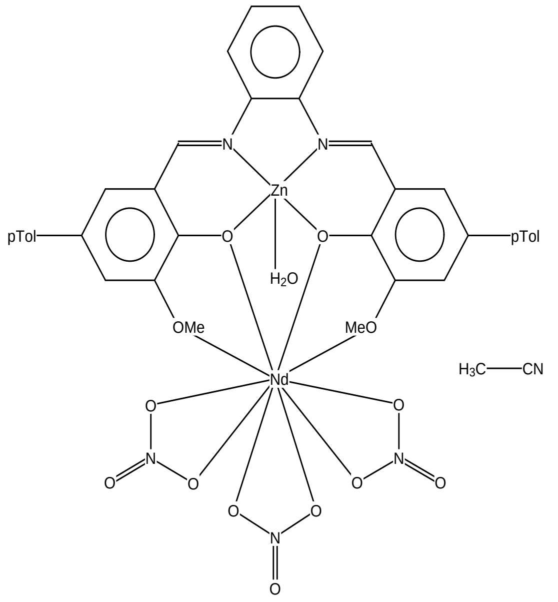

MEZWET

Reference: K.Chichak, U.Jacquemard, N.R.Branda (2002) *Eur.J.Inorg.Chem.* ,357

Formula: $C_{66}\ H_{86}\ N_4\ Ni_2\ O_4,1.4(C_3\ H_6\ O_1),0.3(C_1\ H_2\ Cl_2)$

Compound Name: (μ_2-N,N',N'',N'''-tetrakis(3,5-Di-t-butylsalicylidenato)-1,2,4,5-phenylenetetraamine)-di-nickel(ii) acetone dichloromethane solvate

Space Group:	Pbcn	***Cell:***	***a*** 27.449(1)	***b*** 21.800(1)	***c*** 23.354(1)		
Space Group No.:	60	***(Å,°)***	α 90.00	β 90.00	γ 90.00		
R-Factor (%):	6.67	***Temperature(K):***	193	***Density(g/cm³):***	1.163		

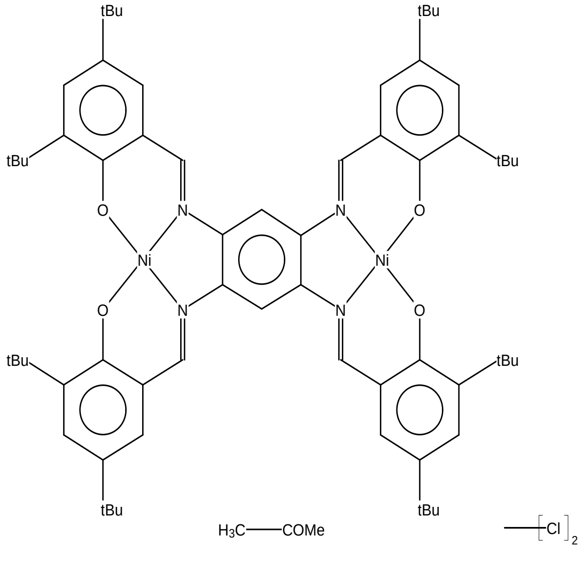

MEZWIX

Reference: K.Chichak, U.Jacquemard, N.R.Branda (2002) *Eur.J.Inorg.Chem.* ,357

Formula: $C_{72}\ H_{88}\ N_4\ Ni_2\ O_6,2(C_4\ H_8\ O_1)$

Compound Name: (μ_2-N,N',N'',N'''-tetrakis(3,5-Di-t-butylsalicylidenato)dibenzo[1,4]dioxin-2,3,7,8-tetraamine)-di-nickel(ii) tetrahydrofuran solvate

Space Group:	P21/c	***Cell:***	***a*** 10.696(1)	***b*** 14.073(2)	***c*** 25.424(4)		
Space Group No.:	14	***(Å,°)***	α 90.00	β 95.73(0)	γ 90.00		
R-Factor (%):	5.19	***Temperature(K):***	193	***Density(g/cm³):***	1.192		

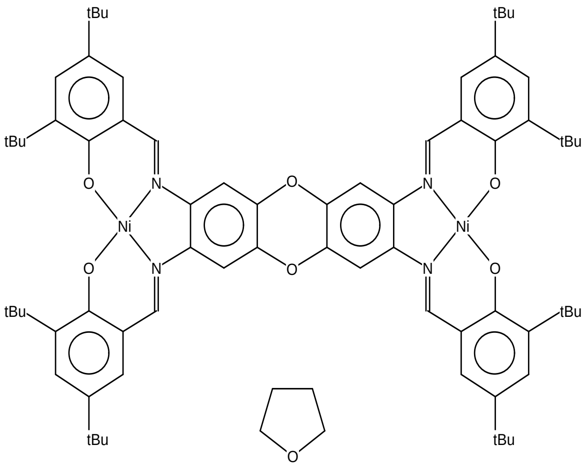

MIGDAI

Reference: Yong-Hao Ye, Yue Han, Ting-Ting Chen, Chang-Hong Liu (2007) *Acta Crystallogr.,Sect.E:Struct.Rep.Online* ,**63,**m1963

Formula: $C_{20}\ H_{14}\ Fe_1\ N_2\ O_2$

Compound Name: (N,N'-bis(Salicylidene)benzene-1,2-diaminato)-iron(ii)

Synonym: (2,2'-(o-Phenylenebis(nitrilomethylidyne))diphenolato)-iron(ii)

Space Group: P212121 ***Cell:*** ***a*** 5.468(1) ***b*** 16.616(4) ***c*** 17.310(3)
Space Group No.: 19 ***(Å,°)*** α 90.00 β 90.00 γ 90.00

R-Factor (%): 5.44 ***Temperature(K):*** 298 ***Density(g/cm³):*** 1.564

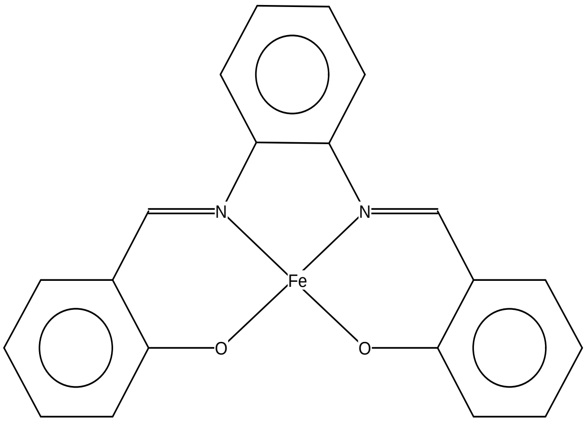

MILFUJ

Reference: A.D.Cort, L.Mandolini, C.Pasquini, K.Rissanen, L.Russo, L.Schiaffino (2007) *New J.Chem.* ,**31,**1633

Formula: $C_{60}\ H_{56}\ N_4\ O_4\ Zn_2,C_1\ H_4\ O_1$

Compound Name: bis(μ_2-2-(((3-(((2-hydroxy-3-(1-methylethyl)phenyl)methylidene)amino)naphthalen-2-yl)imino)methyl)-6-(1-methylethyl)phenolato)-di-zinc(ii) methanol solvate

Space Group: P-1 ***Cell:*** ***a*** 13.409(3) ***b*** 14.202(3) ***c*** 15.345(3)
Space Group No.: 2 ***(Å,°)*** α 109.09(3) β 99.73(3) γ 99.82(3)

R-Factor (%): 6.51 ***Temperature(K):*** 173 ***Density(g/cm³):*** 1.333

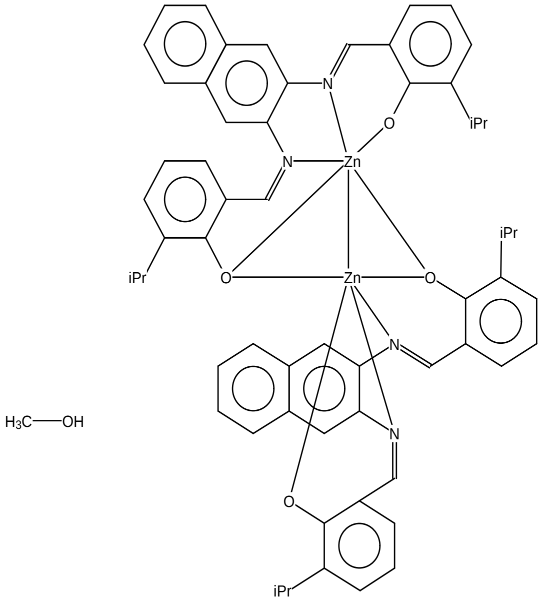

MILGAQ

Reference: A.D.Cort, L.Mandolini, C.Pasquini, K.Rissanen, L.Russo, L.Schiaffino (2007) *New J.Chem.* ,**31,**1633

Formula: $C_{37}\ H_{41}\ N_3\ O_2\ Zn_1,0.78(C_1\ H_1\ Cl_3)$

Compound Name: (1-azabicyclo[2.2.2]octane)-(2,2'-(naphthalene-2,3-diylbis[(nitrilo)methylylidene])-bis[6-(1-methylethyl)phenolato])-zinc chloroform solvate

Space Group: P21/c ***Cell:*** ***a*** 21.550(4) ***b*** 11.749(2) ***c*** 30.535(0)
Space Group No.: 14 ***(Å,°)*** α 90.00 β 109.83(3) γ 90.00

R-Factor (%): 19.53 ***Temperature(K):*** 173 ***Density(g/cm³):*** 1.312

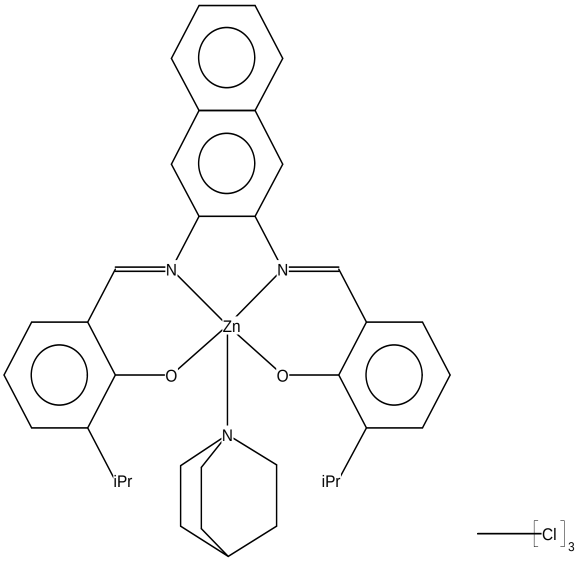

MIRTAJ

Reference: Shaomin Shi, Xuegang Song, Zongqiu Hu, Xieqing Yu (2008) *Acta Crystallogr.,Sect.E:Struct.Rep.Online* ,**64,**m36

Formula: $C_{46}\ H_{34}\ Br_8\ Cd_2\ N_6\ O_6$

Compound Name: bis(μ_2-4,4',6,6'-Tetrabromo-2,2'-(o-phenylenebis(nitrilomethylidyne))diphenolato)-bis(dimethylformamide)-di-cadmium(ii)

Space Group: P-1 ***Cell:*** ***a*** 9.683(0) ***b*** 12.043(1) ***c*** 12.488(1)
Space Group No.: 2 ***(Å,°)*** α 95.94(0) β 108.82(0) γ 90.31(0)

R-Factor (%): 5.93 ***Temperature(K):*** 296 ***Density(g/cm³):*** 1.977

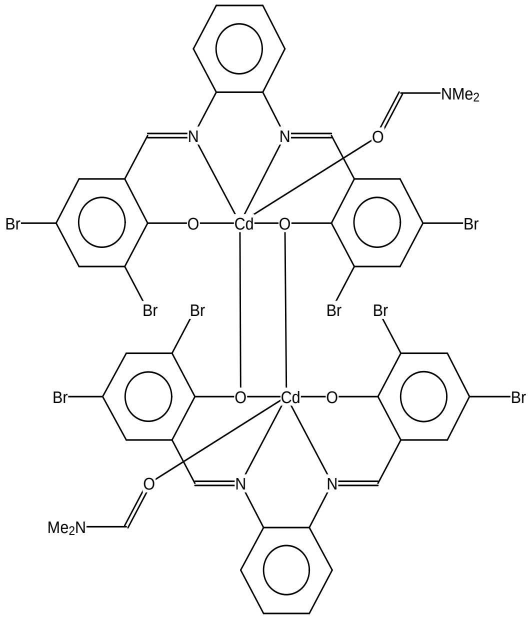

MIRTEM

Reference: R.Alvarez, A.Cabrera, G.Espinosa-Perez, S.Hernandez-Ortega, L.Velasco, B.Esquivel (2002) *Transition Met.Chem.* ,**27,**213

Formula: $C_{24}\,H_{21}\,Co_1\,N_2\,O_3$

Compound Name: Butanoyl-N,N'-bis(disalicylidene-1,3-diaminobenzene)-cobalt(iii)

Synonym: Butanoyl-salophen-cobalt(iii)

Space Group:	P-1	***Cell:***	***a*** 10.893(2)	***b*** 12.243(2)	***c*** 15.685(2)
Space Group No.:	2	***(Å,°)***	α 91.16(2)	β 96.60(2)	γ 101.08(2)

R-Factor (%): 7.74 ***Temperature(K):*** 293 ***Density(g/cm³):*** 1.449

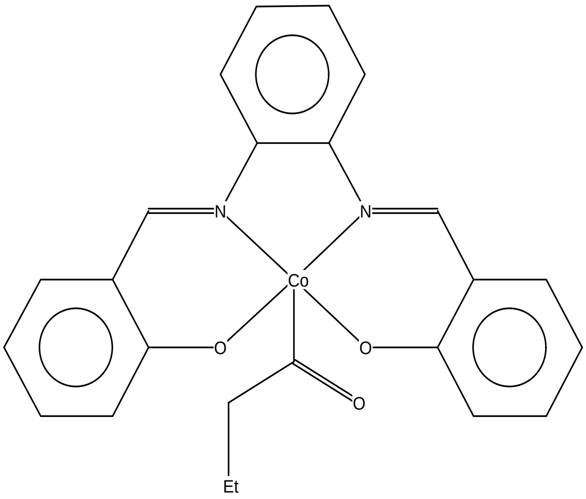

MIXLAH

Reference: M.Nayak, S.Hazra, P.Lemoine, R.Koner, C.R.Lucas, S.Mohanta (2008) *Polyhedron* ,**27,**1201

Formula: $C_{24}\,H_{28}\,Cu_2\,N_2\,O_7\,^{2+}$,1.5($C_{24}\,H_{22}\,Cu_1\,N_2\,O_4$),2($Cl_1\,O_4\,^{1-}$),$C_3\,H_6\,O_1$

Compound Name: (μ_2-N,N'-o-Phenylenebis(3-ethoxysalicylaldimine)-N,N',O,O',O'')-triaqua-di-copper(ii) sesquikis((N,N'-o-phenylenebis(3-ethoxysalicylaldimine)-N, N',O,O')-copper(ii)) diperchlorate acetone solvate

Space Group:	C2/c	***Cell:***	***a*** 32.846(8)	***b*** 14.816(9)	***c*** 29.213(9)
Space Group No.:	15	***(Å,°)***	α 90.00	β 113.35(5)	γ 90.00

R-Factor (%): 5.76 ***Temperature(K):*** 293 ***Density(g/cm³):*** 1.567

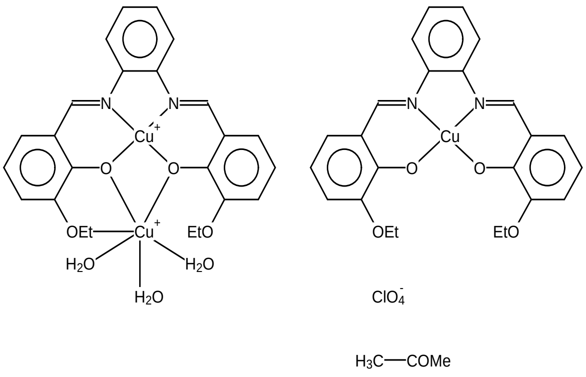

MIXLEL

Reference: M.Nayak, S.Hazra, P.Lemoine, R.Koner, C.R.Lucas, S.Mohanta (2008) *Polyhedron* ,**27,**1201

Formula: $C_{24}\,H_{28}\,Cu_1\,Mn_1\,N_2\,O_7\,^{2+}$,2($C_{24}\,H_{22}\,Cu_1\,N_2\,O_4$),2($Cl_1\,O_4\,^{1-}$),$C_3\,H_6\,O_1$,$H_2\,O_1$

Compound Name: (μ_2-N,N'-o-Phenylenebis(3-ethoxysalicylaldimine))-triaqua-copper(ii)-manganese(ii) bis((N,N'-o-phenylenebis(3-ethoxysalicylaldimine))-copper(ii)) diperchlorate acetone solvate monohydrate

Space Group:	P21/n	***Cell:***	***a*** 14.205(6)	***b*** 27.060(9)	***c*** 20.451(6)
Space Group No.:	14	***(Å,°)***	α 90.00	β 107.17(4)	γ 90.00

R-Factor (%): 5.51 ***Temperature(K):*** 293 ***Density(g/cm³):*** 1.576

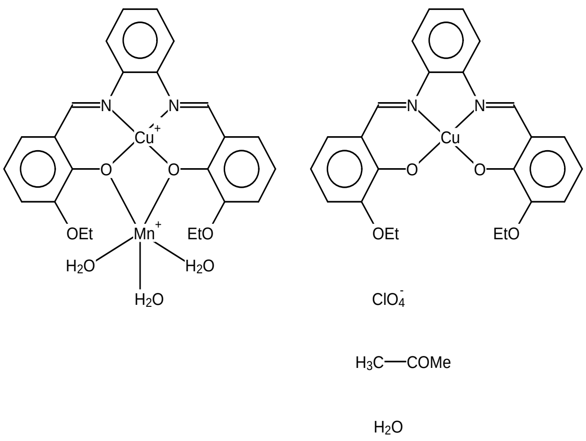

MOLSAH

Reference: B.Yearwood, S.Parkin, D.A.Atwood (2002) *Inorg.Chim.Acta* ,**333,**124

Formula: $C_{36}\,H_{46}\,Cl_2\,N_2\,O_2\,Sn_1$

Compound Name: Dichloro-(N,N'-phenylene-bis(3,5-di-t-butylsalicylideneiminato))-tin

Space Group:	P-1	***Cell:***	***a*** 11.369(2)	***b*** 13.087(2)	***c*** 13.101(2)
Space Group No.:	2	***(Å,°)***	α 74.72(1)	β 75.83(1)	γ 87.22(1)

R-Factor (%): 3.32 ***Temperature(K):*** 144 ***Density(g/cm³):*** 1.327

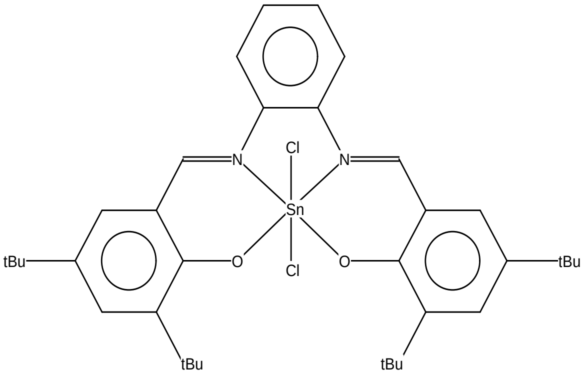

MOLSIP

Reference: B.Yearwood, S.Parkin, D.A.Atwood (2002) *Inorg.Chim.Acta* ,**333,**124

Formula: C_{38} H_{50} Cl_2 N_2 O_2 Sn_1,C_7 H_8

Compound Name: Dichloro-(N,N'-(4,5-dimethyl)phenylene-bis(3,5-di-t-butylsalicylideneiminato))-tin toluene solvate

Space Group:	P21/c	***Cell:***	***a***	25.429(1)	***b***	28.232(2)	***c***	12.086(1)
Space Group No.:	14	***(Å,°)***	α	90.00	β	97.16(1)	γ	90.00

R-Factor (%): 3.37 ***Temperature(K):*** 173 ***Density(g/cm³):*** 1.309

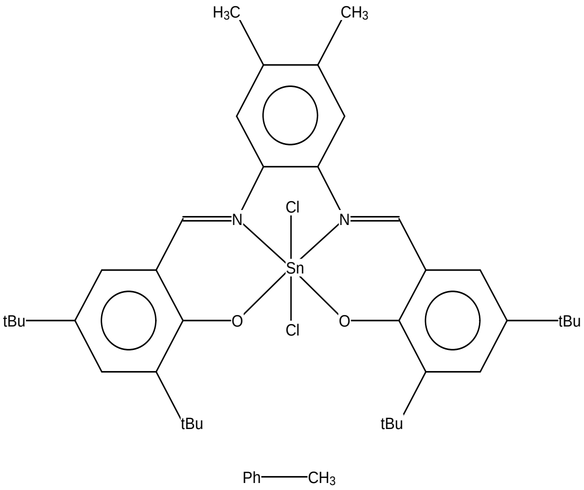

MOWNAN

Reference: Kou-Lin Zhang, Yan Xu, Chang-Ge Zheng, Yong Zhang, Zheng Wang, Xiao-Zeng You (2001) *Inorg.Chim.Acta* ,**318,**61

Formula: (C_{22} H_{17} Mn_1 N_2 O_4)n

Compound Name: catena-((μ_2-Acetato)-(phenylenebis(salicylideneiminato))-manganese(iii))

Space Group:	P21/n	***Cell:***	***a***	11.447(2)	***b***	14.155(3)	***c***	12.465(3)
Space Group No.:	14	***(Å,°)***	α	90.00	β	115.15(3)	γ	90.00

R-Factor (%): 3.76 ***Temperature(K):*** 298 ***Density(g/cm³):*** 1.556

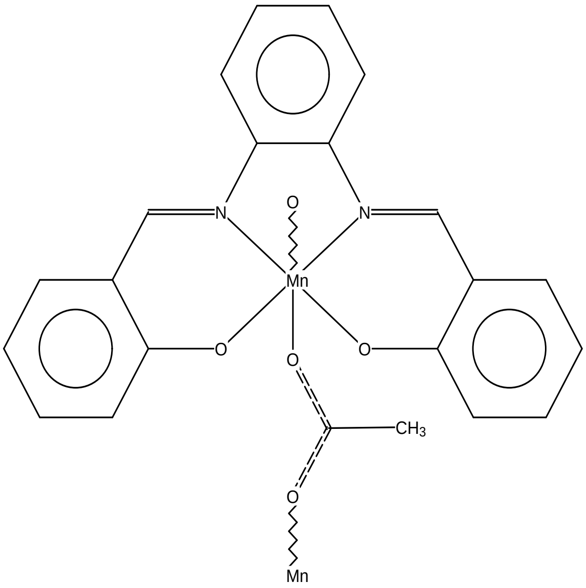

MOWNAN01

Reference: S.Biswas, K.Mitra, C.H.Schwalbe, C.R.Lucas, S.K.Chattopadhyay, B.Adhikary (2005) *Inorg.Chim.Acta* ,**358,**2473

Formula: (C_{22} H_{17} Mn_1 N_2 O_4)n

Compound Name: catena-((μ_2-Acetato)-(N,N'-o-phenylenebis(salicylideneimine))-manganese(iii))

Space Group:	P21/n	***Cell:***	***a***	11.405(0)	***b***	14.124(0)	***c***	12.467(0)
Space Group No.:	14	***(Å,°)***	α	90.00	β	115.22(0)	γ	90.00

R-Factor (%): 2.93 ***Temperature(K):*** 193 ***Density(g/cm³):*** 1.566

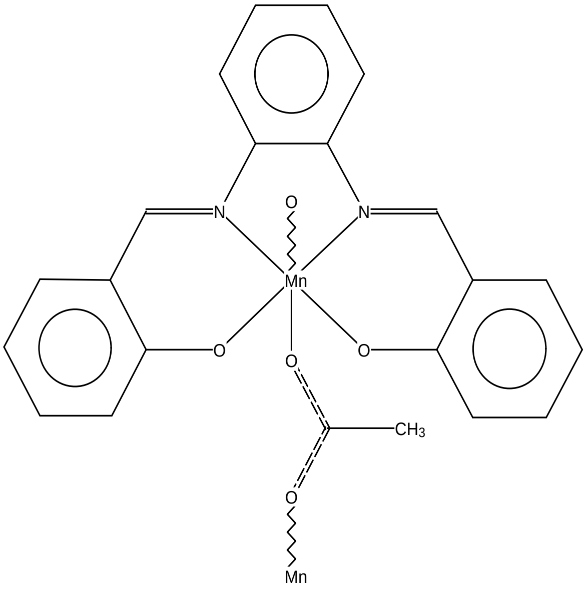

MUJDOL

Reference: Weiyu Bi, Tao Wei, Xingqiang Lu, Yani Hui, Jirong Song, Shunsheng Zhao, Wai-Kwok Wong, R.A.Jones (2009) *New J.Chem.* , **33,**2326

Formula: C_{50} H_{38} N_9 Nd_1 O_{13} Zn_2,C_4 H_{10} O_1

Compound Name: bis(μ_2-2,2'-(Phenylene-1,2-diylbis(nitrilomethylylidene))diphenolato)-tris(nitrato-O,O')-dipyridyl-neodymium(iii)-di-zinc(ii) diethyl ether solvate

Space Group:	Cc	***Cell:***	***a***	14.654(4)	***b***	19.038(5)	***c***	19.859(7)
Space Group No.:	9	***(Å,°)***	α	90.00	β	97.99(0)	γ	90.00

R-Factor (%): 3.83 ***Temperature(K):*** 273 ***Density(g/cm³):*** 1.600

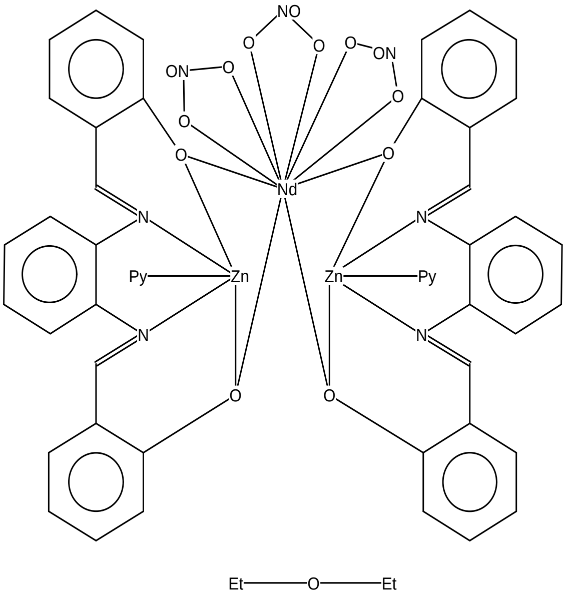

MURQIA

Reference: Wei You, Cheng Yao, Wei Huang (2010) *Wuji Huaxue Xuebao(Chin.)(Chin.J.Inorg.Chem.)* ,**26,**867

Formula: C_{20} H_{12} Br_4 N_2 O_3 Zn_1

Compound Name: Aqua-(2,2'-(1,2-phenylenebis((nitrilo)methylylidene))bis(4,6-dibromophenolato))-zinc(ii)

Space Group:	Pnma	***Cell:***	***a***	18.647(5)	***b***	24.454(6)	***c***	4.657(1)
Space Group No.:	62	***(Å,°)***	α	90.00	β	90.00	γ	90.00

R-Factor (%): 5.26 ***Temperature(K):*** 291 ***Density(g/cm³):*** 2.231

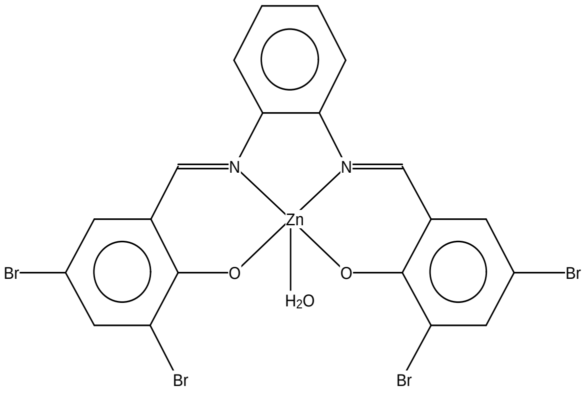

MURQOG

Reference: Wei You, Cheng Yao, Wei Huang (2010) *Wuji Huaxue Xuebao(Chin.)(Chin.J.Inorg.Chem.)* ,**26,**867

Formula: C_{20} H_{10} Br_4 N_2 Ni_1 O_2

Compound Name: (2,2'-(1,2-Phenylenebis((nitrilo)methylylidene))bis(4,6-dibromophenolato))-nickel(ii)

Space Group:	P21/n	***Cell:***	***a***	13.459(1)	***b***	8.253(1)	***c***	18.337(2)
Space Group No.:	14	***(Å,°)***	α	90.00	β	96.52(0)	γ	90.00

R-Factor (%): 5.04 ***Temperature(K):*** 291 ***Density(g/cm³):*** 2.260

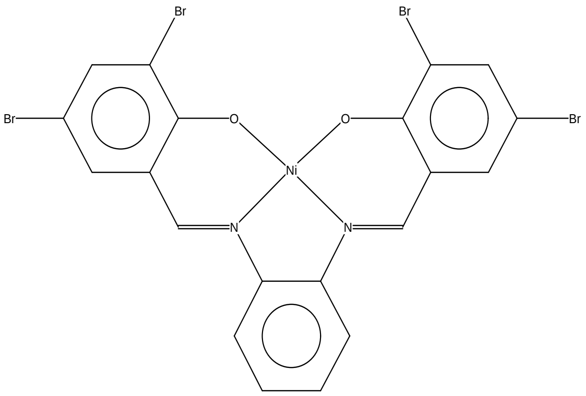

NALJAN

Reference: A.Jamshidvand, H.Kargar, R.Kia, M.N.Tahir (2010) *Acta Crystallogr.,Sect.E:Struct.Rep.Online* ,**66,**m1473

Formula: C_{26} H_{26} Cu_1 N_2 O_4

Compound Name: (6,6'-Diethoxy-2,2'-[4,5-dimethyl-o-phenylenebis(nitrilomethylidyne)]diphenolato)-copper(ii)

Space Group:	C2/c	***Cell:***	***a***	14.976(0)	***b***	15.880(0)	***c***	12.226(0)
Space Group No.:	15	***(Å,°)***	α	90.00	β	119.28(0)	γ	90.00

R-Factor (%): 6.63 ***Temperature(K):*** 296 ***Density(g/cm³):*** 1.294

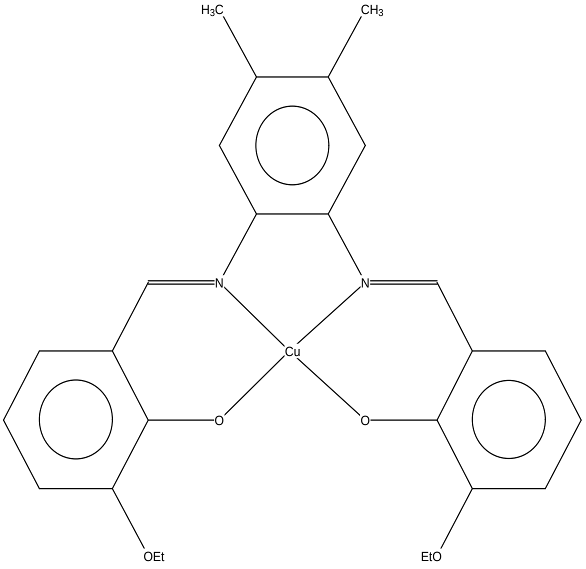

NANMIY

Reference: E.Gallo, E.Solari, N.Re, C.Floriani, A.Chiesi-Villa, C.Rizzoli (1997) *J.Am.Chem.Soc.* ,**119,**5144

Formula: C_{50} H_{38} Mn_2 N_6 O_4

Compound Name: bis((μ_2-N,N'-bis(Salicylidene)benzene-1,2-diamine)-pyridyl-manganese(ii))

Space Group:	P212121	***Cell:***	***a***	24.632(4)	***b***	16.821(3)	***c***	10.098(3)
Space Group No.:	19	***(Å,°)***	α	90.00	β	90.00	γ	90.00

R-Factor (%): 4.60 ***Temperature(K):*** 295 ***Density(g/cm³):*** 1.424

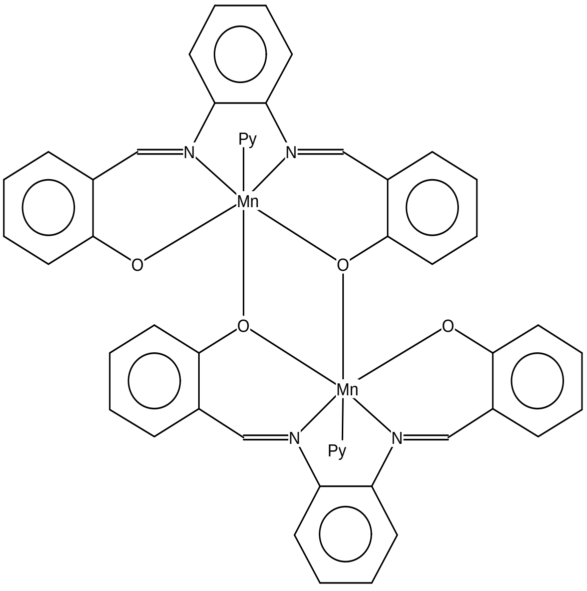

Search: search26 (Tue Dec 2 15:48:50 2014): Hits 393-396

NANNEV

Reference: E.Gallo, E.Solari, N.Re, C.Floriani, A.Chiesi-Villa, C.Rizzoli (1997) *J.Am.Chem.Soc.* ,**119,**5144

Formula: $C_{24} H_{22} Mn_1 N_2 O_3{}^{1+}, I_3{}^{1-}, C_4 H_8 O_1$

Compound Name: (N,N'-bis(Salicylidene)benzene-1,2-diamine)-tetrahydrofuran-manganese tri-iodide tetrahydrofuran solvate

Space Group:	P21/n	***Cell:***	***a***	9.886(8)	***b***	19.377(3)	***c***	15.883(4)
Space Group No.:	14	***(Å,°)***	α	90.00	β	94.47(3)	γ	90.00

R-Factor (%): 4.50 ***Temperature(K):*** 295 ***Density(g/cm^3):*** 1.958

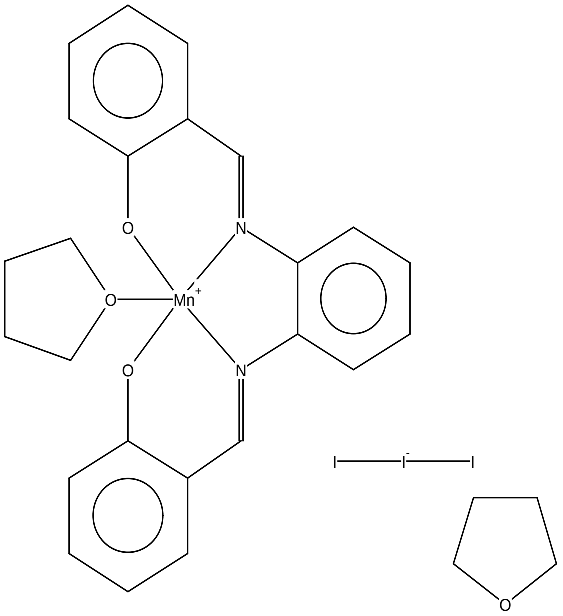

NANNIZ

Reference: E.Gallo, E.Solari, N.Re, C.Floriani, A.Chiesi-Villa, C.Rizzoli (1997) *J.Am.Chem.Soc.* ,**119,**5144

Formula: $C_{72} H_{92} Mn_2 N_4 O_6$

Compound Name: bis((μ_2-Oxo)-(μ_2-N,N'-bis(3,5-di-t-butyl-salicylidene)benzene-1,2-diamine)-manganese(iv))

Space Group:	P-1	***Cell:***	***a***	12.668(2)	***b***	13.830(3)	***c***	10.469(1)
Space Group No.:	2	***(Å,°)***	α	97.07(1)	β	113.74(1)	γ	80.87(1)

R-Factor (%): 5.50 ***Temperature(K):*** 295 ***Density(g/cm^3):*** 1.224

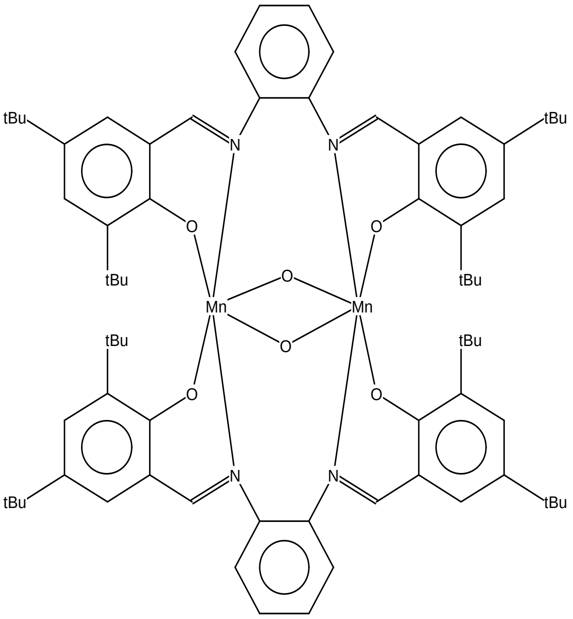

NASGEU

Reference: Xiaoping Yang, R.A.Jones (2005) *J.Am.Chem.Soc.* ,**127,** 7686

Formula: $C_{88} H_{68} Br_8 N_8 O_{18} Tb_3{}^{1+}, Cl_1{}^{1-}, 9(C_1 H_4 O_1), 4(H_2 O_1)$

Compound Name: bis(μ_2-N,N'-bis(5-Bromo-3-methoxysalicylidene)phenylene-1,2-diamine)-bis(N,N'-bis(5-bromo-3-methoxysalicylidene)phenylene-1,2-diamine)-diaqua-tri-terbium(iii) chloride methanol solvate tetrahydrate

Space Group:	P21/c	***Cell:***	***a***	22.764(5)	***b***	19.015(4)	***c***	29.285(6)
Space Group No.:	14	***(Å,°)***	α	90.00	β	104.78(3)	γ	90.00

R-Factor (%): 8.45 ***Temperature(K):*** 153 ***Density(g/cm^3):*** 1.646

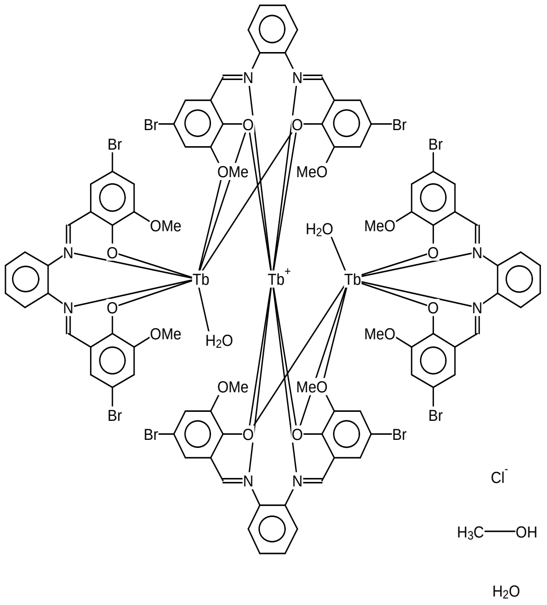

NASGIY

Reference: Xiaoping Yang, R.A.Jones (2005) *J.Am.Chem.Soc.* ,**127,** 7686

Formula: $C_{70} H_{54} Br_6 Cl_1 N_6 O_{16} Tb_3, 2(C_2 H_6 O_1), C_4 H_{10} O_1, H_2 O_1$

Compound Name: bis(μ_2-N,N'-bis(5-Bromo-3-methoxysalicylidene)phenylene-1,2-diamine)-(μ_2-acetato-O,O')-(acetato-O,O')-(N,N'-bis(5-bromo-3-methoxysalicylidene)phenylene-1,2-diamine)-chloro-tri-terbium(iii) diethyl ether ethanol solvate monohydrate

Space Group:	P21/n	***Cell:***	***a***	20.211(4)	***b***	22.089(4)	***c***	20.519(4)
Space Group No.:	14	***(Å,°)***	α	90.00	β	114.69(3)	γ	90.00

R-Factor (%): 8.14 ***Temperature(K):*** 153 ***Density(g/cm^3):*** 1.924

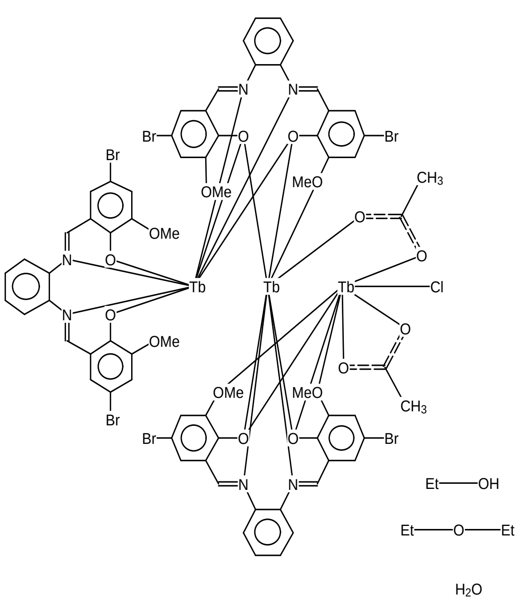

Search: search26 (Tue Dec 2 15:48:50 2014): Hits 397-400

NASZOX

Reference: T.Mallah, S.Parsons, D.Messenger (2005)
Private Communication ,

Formula: $2(C_{24}H_{20}P_1{}^{1+}),C_{24}H_{12}N_4Ni_2O_8{}^{2-},1.7(C_2H_3N_1),2.3(H_2O_1)$

Compound Name: bis(Tetraphenylphosphonium) (μ_2-1,2-bis(N-salicylidenealdiminato) benzene-4,5-bis(oximato))-di-nickel acetonitrile solvate hydrate

Space Group:	P21/n	***Cell:***	***a***	16.339(1)	***b***	18.876(1)	***c***	21.488(2)
Space Group No.:	14	***(Å,°)***	α	90.00	β	95.66(0)	γ	90.00

R-Factor (%): 4.65 ***Temperature(K):*** 220 ***Density(g/cm^3):*** 1.402

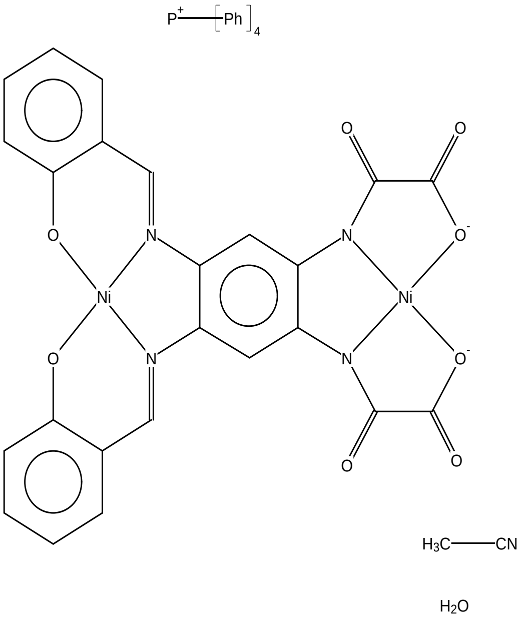

NATBIU

Reference: T.Mallah, S.Parsons, D.Messenger (2005)
Private Communication ,

Formula: $2(C_{24}H_{20}P_1{}^{1+}),C_{24}H_{14}N_6Ni_2O_6{}^{2-},2(C_2H_3N_1),2(H_2O_1)$

Compound Name: bis(Tetraphenylphosphonium) (μ_2-1,2-bis(N-salicylidenealdiminato) benzene-4,5-bis(oxamido))-di-nickel acetonitrile solvate dihydrate

Space Group:	P21/n	***Cell:***	***a***	16.299(4)	***b***	18.891(5)	***c***	21.462(5)
Space Group No.:	14	***(Å,°)***	α	90.00	β	95.77(1)	γ	90.00

R-Factor (%): 5.18 ***Temperature(K):*** 220 ***Density(g/cm^3):*** 1.411

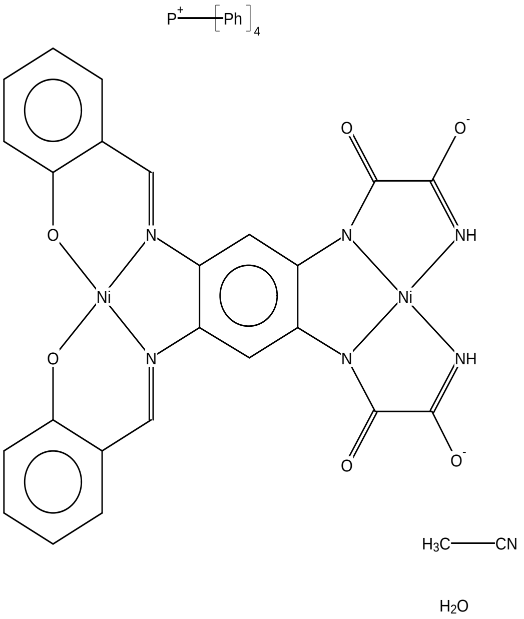

NATCAN

Reference: T.Mallah, S.Parsons, D.Messenger (2005)
Private Communication ,

Formula: $C_{70}H_{102}Mn_2N_4O_8,5.16(C_1H_4O_1)$

Compound Name: (μ_2-1,2,4,5-tetrakis(N-(4,6-di-t-butylsalicylidenealdiminato))benzene)-tetrakis(methanol)-di-manganese methanol solvate

Space Group:	C2/m	***Cell:***	***a***	11.975(2)	***b***	32.119(6)	***c***	13.061(3)
Space Group No.:	12	***(Å,°)***	α	90.00	β	96.28(2)	γ	90.00

R-Factor (%): 8.30 ***Temperature(K):*** 220 ***Density(g/cm^3):*** 0.933

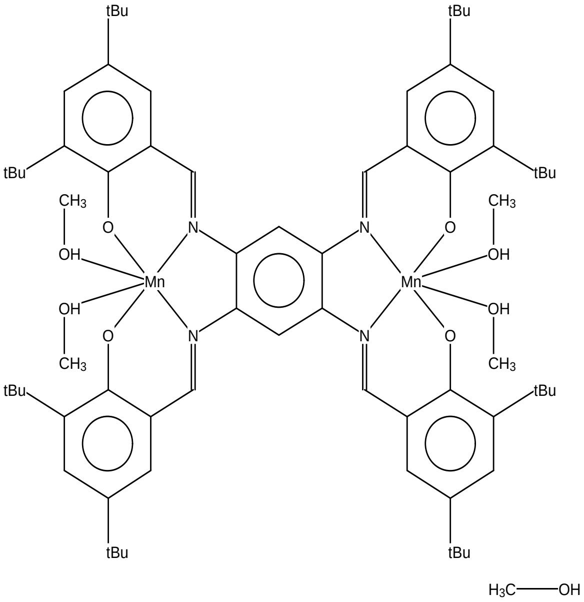

NECWOI

Reference: L.Salmon, P.Thuery, E.Riviere, M.Ephritikhine (2006)
Inorg.Chem. ,**45,**83

Formula: $C_{50}H_{34}N_6O_8U_1Zn_2,3(C_5H_5N_1)$

Compound Name: bis(μ_2-N,N'-bis(3-Oxysalicylidene)-1,2-phenylenediamine)-bis(pyridine-N) -uranium(iv)-di-zinc(ii) pyridine solvate

Space Group:	P21/n	***Cell:***	***a***	15.735(1)	***b***	21.006(3)	***c***	17.375(2)
Space Group No.:	14	***(Å,°)***	α	90.00	β	105.07(0)	γ	90.00

R-Factor (%): 8.28 ***Temperature(K):*** 100 ***Density(g/cm^3):*** 1.740

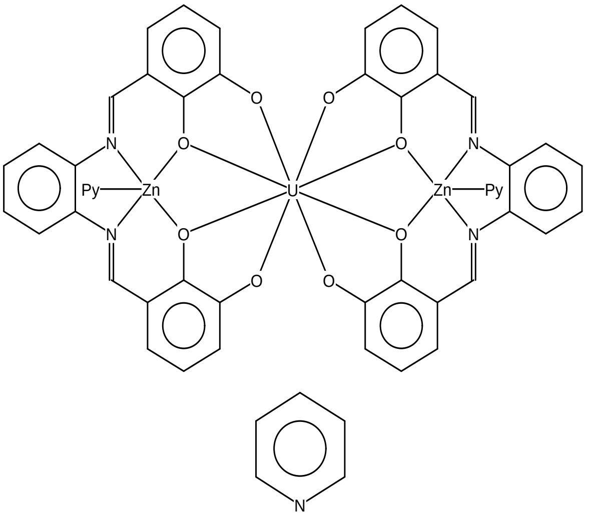

NECWUO

Reference: L.Salmon, P.Thuery, E.Riviere, M.Ephritikhine (2006) *Inorg.Chem.* ,**45,**83

Formula: $C_{54} H_{42} N_6 O_8 U_1 Zn_2, C_5 H_5 N_1$

Compound Name: bis(μ_2-N,N'-bis(3-Oxysalicylidene)-4,5-dimethyl-1,2-phenylenediamine)-bis(pyridine-N)-uranium(iv)-di-zinc(ii) pyridine solvate

Space Group: P21/c **Space Group No.:** 14

Cell: (Å,°) *a* 13.513(0) *b* 24.797(1) *c* 16.054(0) α 90.00 β 109.33(0) γ 90.00

R-Factor (%): 4.51 **Temperature(K):** 100 **Density(g/cm^3):** 1.768

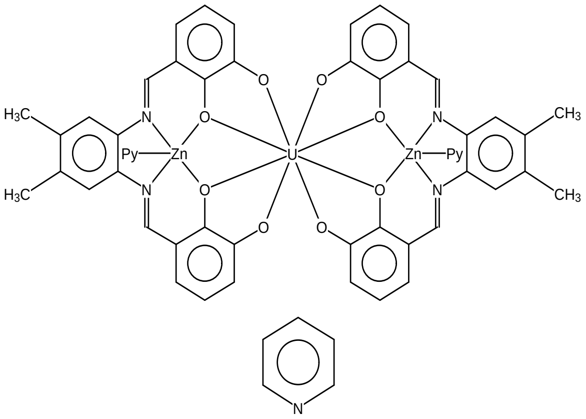

NELVIK

Reference: A.J.Gallant, J.H.Chong, M.J.MacLachlan (2006) *Inorg.Chem.* ,**45,**5248

Formula: $C_{84} H_{102} N_6 O_{25} Zn_7, C_6 H_6$

Compound Name: (μ_6-6,7,20,21,34,35-Hexapentoxy-41,42,45,46,47,48-hexahydroxy-3,10,17,24,31,38-hexa-azaheptacyclo(38.2.2.2^{12,15}.2^{26,29}.0^{4,9}.0^{18,23}.0^{32,37})octatetraconta-1(41),2,4,6,8,10,12(45),13,15(46),16,18,20,22,24,26(47),27,29(48),30,32,34,36,38,40(42),43-tetracosaene)-(μ_4-oxo)-pentakis(μ_2-acetato)-(acetato)-hepta-zinc(ii) benzene solvate

Space Group: C2/c **Space Group No.:** 15

Cell: (Å,°) *a* 27.342(5) *b* 38.204(5) *c* 20.526(5) α 90.00 β 113.34(0) γ 90.00

R-Factor (%): 5.51 **Temperature(K):** 173 **Density(g/cm^3):** 1.438

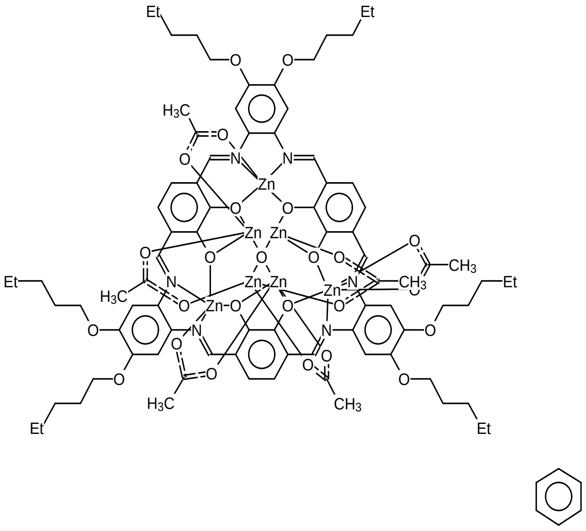

NELVOQ

Reference: A.J.Gallant, J.H.Chong, M.J.MacLachlan (2006) *Inorg.Chem.* ,**45,**5248

Formula: $C_{68} H_{72} N_6 O_{26} S_1 Zn_7, 5(C_2 H_6 O_1 S_1)$

Compound Name: (μ_6-6,7,20,21,34,35-Hexaethoxy-41,42,45,46,47,48-hexahydroxy-3,10,17,24,31,38-hexa-azaheptacyclo(38.2.2.2^{12,15}.2^{26,29}.0^{4,9}.0^{18,23}.0^{32,37})octatetraconta-1(41),2,4,6,8,10,12(45),13,15(46),16,18,20,22,24,26(47),27,29(48),30,32,34,36,38,40(42),43-tetracosaene)-(μ_4-oxo)-tris(μ_3-acetato-O,O,O')-tris(μ_2-acetato-O,O')-(dimethylsulfoxide)-hepta-zinc(ii) dimethylsulfoxide solvate

Space Group: P-1 **Space Group No.:** 2

Cell: (Å,°) *a* 12.036(1) *b* 26.181(3) *c* 26.547(3) α 118.82(0) β 93.44(0) γ 90.15(0)

R-Factor (%): 7.31 **Temperature(K):** 173 **Density(g/cm^3):** 1.031

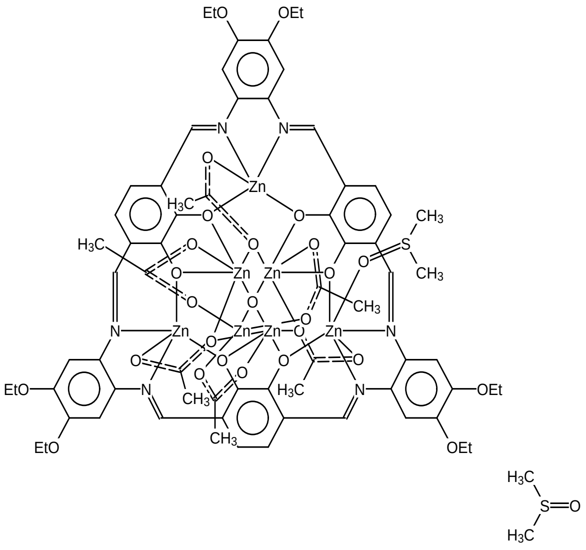

NENHOE

Reference: T.Nabeshima, H.Miyazaki, A.Iwasaki, S.Akine, T.Saiki, C.Ikeda, S.Sato (2006) *Chem.Lett.* ,**35,**1070

Formula: $C_{78} H_{90} N_6 O_{25} Zn_7, 2(C_4 H_8 O_2), C_4 H_8 O_1$

Compound Name: (μ_6-1^4,1^5,7^4,7^5,13^4,13^5-Hexa-n-butoxy-4^2,4^3,10^2,10^3,16^2,16^3-hexahydroxy-2,6,8,12,14,18-hexa-aza-1,7,13(1,2)-tribenzena-4,10,16(1,4)tribenzena-octadecacyclophane-2,5,8,11,14,17-hexaene)-(μ_4-oxo)-tris(μ_2-acetato)-tris(acetato)-hepta-zinc ethyl acetate tetrahydrofuran solvate

Space Group: P21/n **Space Group No.:** 14

Cell: (Å,°) *a* 15.130(16) *b* 36.380(30) *c* 18.320(20) α 90.00 β 107.12(6) γ 90.00

R-Factor (%): 6.98 **Temperature(K):** 120 **Density(g/cm^3):** 1.528

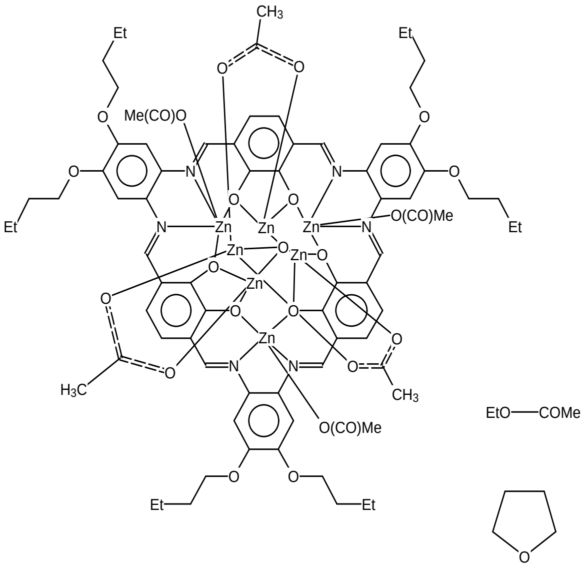

Search: search26 (Tue Dec 2 15:48:50 2014): Hits 405-408

NENHOE01

Reference: T.Nabeshima, H.Miyazaki, A.Iwasaki, S.Akine, T.Saiki, C.Ikeda (2007) *Tetrahedron* ,**63,**3328

Formula: $C_{78} H_{90} N_6 O_{25} Zn_7,2(C_4 H_8 O_2),C_4 H_8 O_1$

Compound Name: (μ_6-$1^4,1^5,7^4,7^5,13^4,13^5$-Hexa-n-butoxy-$4^2,4^3,10^2,10^3,16^2,16^3$-hexahydroxy-2,6,8,12,14,18-hexa-aza-1,7,13(1,2)-tribenzena-4,10,16(1,4)tribenzena-octadecacyclophane-2,5,8,11,14,17-hexaene)-(μ_4-oxo)-tris(μ_2-acetato)-tris(acetato)-hepta-zinc ethyl acetate tetrahydrofuran solvate

Space Group: P21/n ***Cell:*** ***a*** 15.130(16) ***b*** 36.380(30) ***c*** 18.320(20)
Space Group No.: 14 ***(Å,°)*** α 90.00 β 107.12(6) γ 90.00

R-Factor (%): 6.98 ***Temperature(K):*** 120 ***Density(g/cm^3):*** 1.528

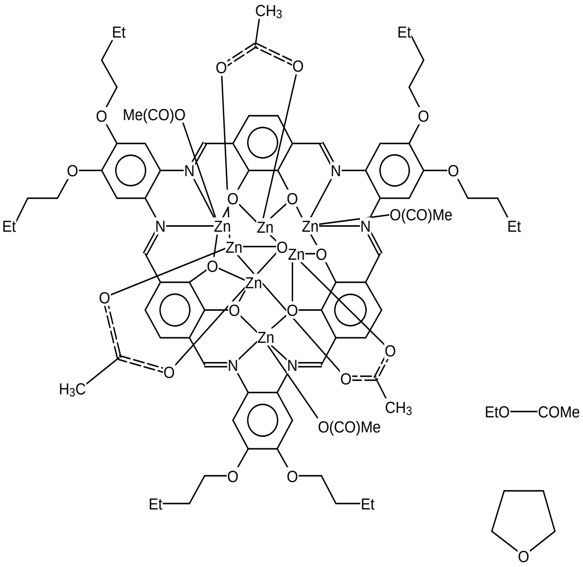

NEQHAS

Reference: K.Kubono, N.Hirayama, H.Kokusen, K.Yokoi (2001) *Anal.Sci.* ,**17,**193

Formula: $C_{40} H_{20} Br_8 Ce_1 N_4 O_4$

Compound Name: mer-bis(N,N'-bis(3,5-Dibromosalicylidene)benzene-1,2-diaminato-N,N',O,O')-cerium(iv)

Space Group: P-1 ***Cell:*** ***a*** 12.890(10) ***b*** 15.740(20) ***c*** 12.140(40)
Space Group No.: 2 ***(Å,°)*** α 100.20(10) β 99.40(10) γ 82.99(8)

R-Factor (%): 6.70 ***Temperature(K):*** 293 ***Density(g/cm^3):*** 1.953

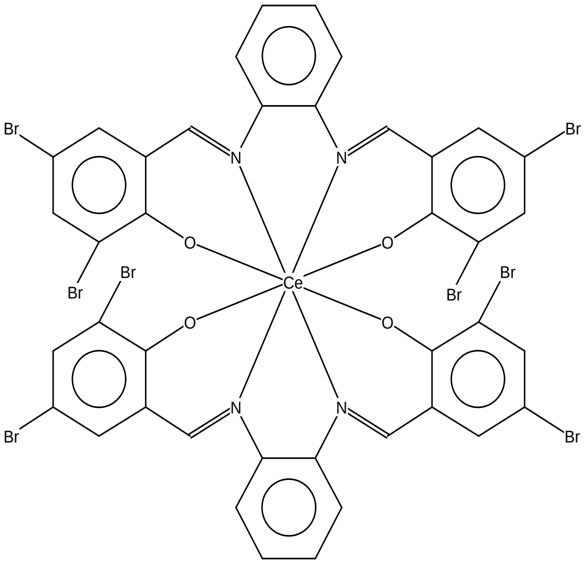

NICQAS

Reference: K.Takao, Y.Ikeda (2007) *Inorg.Chem.* ,**46,**1550

Formula: $C_{22} H_{20} N_2 O_5 S_1 U_1$

Compound Name: Dimethylsulfoxide-(N,N'-disalicylidene-o-phenylenediamine-N,N',O,O')-dioxo-uranium(vi)

Space Group: P21 ***Cell:*** ***a*** 13.303(7) ***b*** 9.422(4) ***c*** 17.205(8)
Space Group No.: 4 ***(Å,°)*** α 90.00 β 94.45(5) γ 90.00

R-Factor (%): 2.67 ***Temperature(K):*** 113 ***Density(g/cm^3):*** 2.047

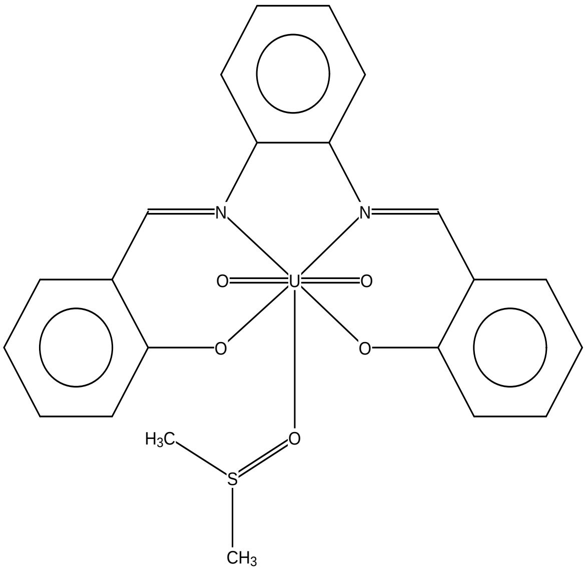

NICQEW

Reference: K.Takao, Y.Ikeda (2007) *Inorg.Chem.* ,**46,**1550

Formula: $C_{40} H_{28} N_4 O_8 U_2,0.5(C_1 H_2 Cl_2)$

Compound Name: bis(μ_2-N,N'-Disalicylidene-o-phenylenediamine-N,N',O,O,O')-tetraoxo-di-uranium(vi) dichloromethane solvate

Space Group: P-1 ***Cell:*** ***a*** 15.717(5) ***b*** 15.993(7) ***c*** 17.619(5)
Space Group No.: 2 ***(Å,°)*** α 67.45(3) β 77.99(3) γ 81.66(3)

R-Factor (%): 5.94 ***Temperature(K):*** 93 ***Density(g/cm^3):*** 2.016

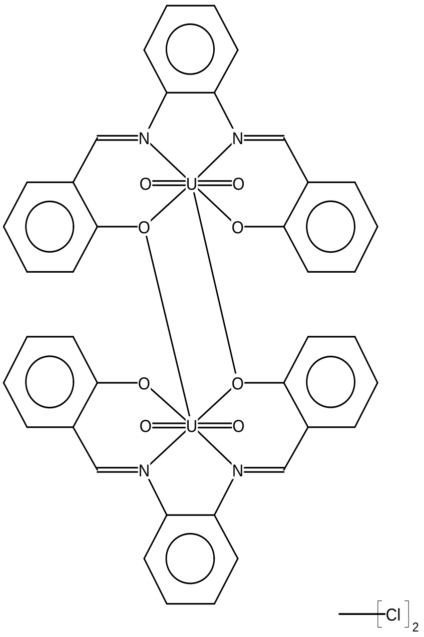

NICQIA

Reference: K.Takao, Y.Ikeda (2007) *Inorg.Chem.* ,**46,**1550

Formula: C_{23} H_{21} N_3 O_5 U_1,C_1 H_2 Cl_2

Compound Name: Dimethylformamide-(N,N'-disalicylidene-o-phenylenediamine-N,N',O,O')-dioxo-uranium(vi) dichloromethane solvate

Space Group: P21/n ***Cell:*** ***a*** 10.667(4) ***b*** 9.608(3) ***c*** 24.859(10)
Space Group No.: 14 ***(Å,°)*** α 90.00 β 100.65(3) γ 90.00

R-Factor (%): 2.58 ***Temperature(K):*** 123 ***Density(g/cm³):*** 1.969

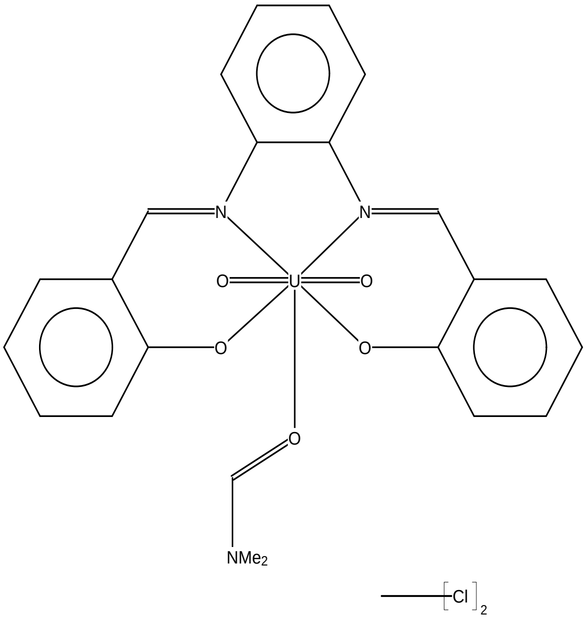

NIQVOY

Reference: E.Solari, C.Maltese, F.Franceschi, C.Floriani, A.Chiesi-Villa, C.Rizzoli (1997) *J.Chem.Soc.,Dalton Trans.* ,2903

Formula: C_{40} H_{28} N_4 O_4 Zr_1,2(C_5 H_5 N_1)

Compound Name: bis(N,N'-bis(Salicylidene)-o-phenylenediaminato)-zirconium pyridine solvate

Space Group: P21/c ***Cell:*** ***a*** 14.387(4) ***b*** 20.410(5) ***c*** 14.455(3)
Space Group No.: 14 ***(Å,°)*** α 90.00 β 104.88(2) γ 90.00

R-Factor (%): 5.50 ***Temperature(K):*** 295 ***Density(g/cm³):*** 1.422

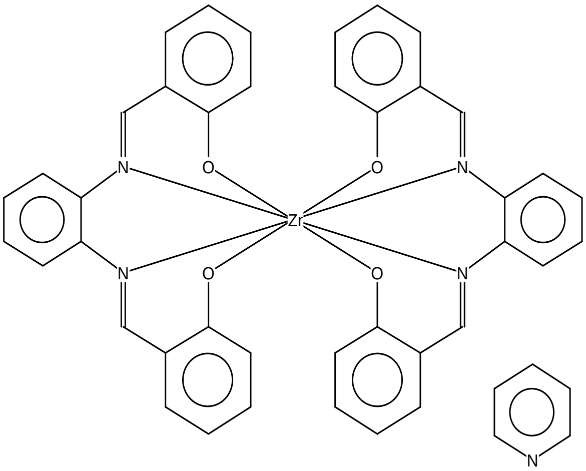

NIQWEP

Reference: E.Solari, C.Maltese, F.Franceschi, C.Floriani, A.Chiesi-Villa, C.Rizzoli (1997) *J.Chem.Soc.,Dalton Trans.* ,2903

Formula: C_{40} H_{28} N_4 O_4 Zr_1,1.5(C_7 H_8)

Compound Name: bis(N,N'-Disalicylidene-1,2-phenylenediamine)-zirconium(iv) toluene solvate

Space Group: P-1 ***Cell:*** ***a*** 14.215(1) ***b*** 14.203(1) ***c*** 10.900(1)
Space Group No.: 2 ***(Å,°)*** α 103.52(1) β 99.16(1) γ 83.11(1)

R-Factor (%): 4.10 ***Temperature(K):*** 295 ***Density(g/cm³):*** 1.354

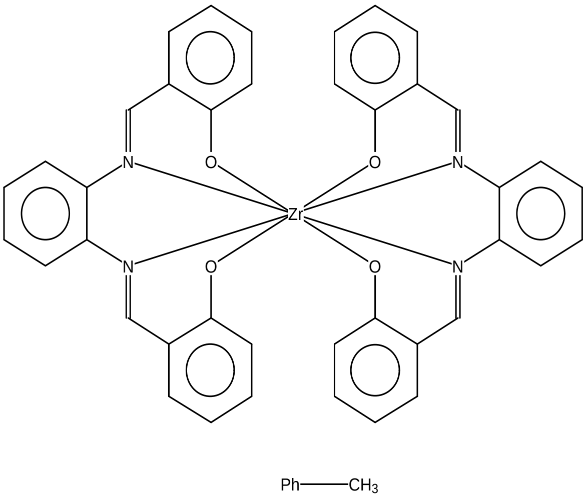

NIXTUJ

Reference: M.S.Hill, D.A.Atwood (1998) *Eur.J.Inorg.Chem.* ,67

Formula: C_{36} H_{46} Cl_1 Ga_1 N_2 O_2

Compound Name: (N,N'-Phenylenebis(3,5-di-t-butylsalicylidenealdiminato))-chloro-gallium

Space Group: P-1 ***Cell:*** ***a*** 10.876(0) ***b*** 17.029(1) ***c*** 20.584(1)
Space Group No.: 2 ***(Å,°)*** α 107.24(0) β 90.02(0) γ 107.55(0)

R-Factor (%): 5.91 ***Temperature(K):*** 295 ***Density(g/cm³):*** 1.238

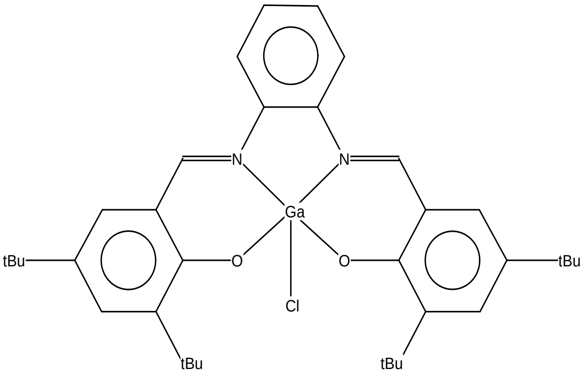

NIXVAR

Reference: M.S.Hill, D.A.Atwood (1998) *Eur.J.Inorg.Chem.* ,67

Formula: $C_{39}\ H_{53}\ Ga_1\ N_2\ O_2,2(H_2\ O_1)$

Compound Name: Methyl-(N,N'-(4,5-dimethyl)phenylenebis(3,5-di-t-butylsalicylidenealdiminato))-gallium dihydrate

Space Group: P21/n ***Cell:*** ***a*** 15.674(0) ***b*** 10.441(0) ***c*** 23.784(1)
Space Group No.: 14 ***(Å,°)*** α 90.00 β 90.50(0) γ 90.00

R-Factor (%): 5.58 ***Temperature(K):*** 295 ***Density(g/cm^3):*** 1.173

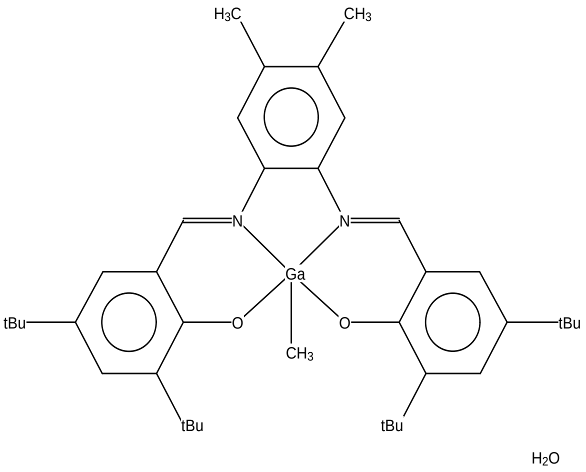

NIXVEV

Reference: M.S.Hill, D.A.Atwood (1998) *Eur.J.Inorg.Chem.* ,67

Formula: $C_{38}\ H_{51}\ Ga_1\ N_2\ O_2$

Compound Name: Ethyl-(N,N'-phenylenebis(3,5-di-t-butylsalicylidenealdiminato))-gallium

Space Group: P-1 ***Cell:*** ***a*** 9.977(0) ***b*** 12.959(0) ***c*** 14.751(0)
Space Group No.: 2 ***(Å,°)*** α 85.87(0) β 79.49(0) γ 71.46(0)

R-Factor (%): 4.93 ***Temperature(K):*** 295 ***Density(g/cm^3):*** 1.191

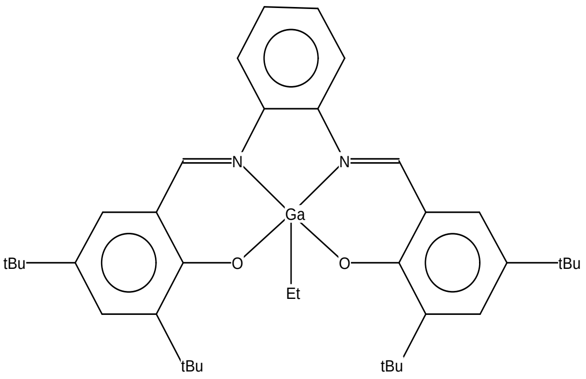

NIXVIZ

Reference: M.S.Hill, D.A.Atwood (1998) *Eur.J.Inorg.Chem.* ,67

Formula: $C_{40}\ H_{55}\ Ga_1\ N_2\ O_2$

Compound Name: Ethyl (N,N'-(4,5-dimethyl)phenylenebis(3,5-di-t-butylsalicylidenealdiminato))-gallium

Space Group: P21/c ***Cell:*** ***a*** 14.461(0) ***b*** 14.663(0) ***c*** 19.124(1)
Space Group No.: 14 ***(Å,°)*** α 90.00 β 106.44(0) γ 90.00

R-Factor (%): 6.13 ***Temperature(K):*** 295 ***Density(g/cm^3):*** 1.137

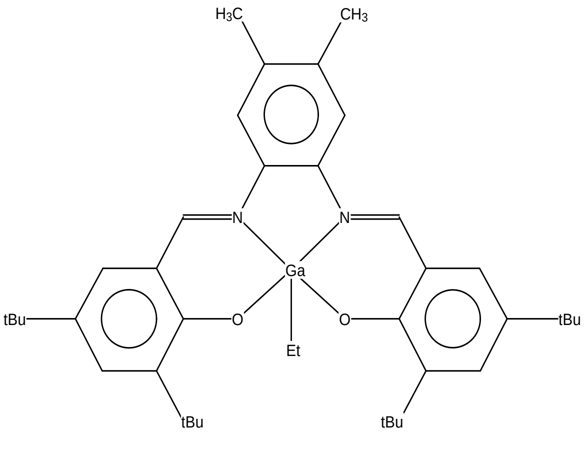

NIZGOS

Reference: Yun Qi Tian, Jian Tong (1997) *Chin.Chem.Lett.* ,**8,**107

Formula: $C_{30}\ H_{22}\ N_4\ Ni_2\ O_2\ ^{2+},2(Cl_1\ O_4\ ^{1-})$

Compound Name: 14,29-Dimethyl-3,10,18,25-tetra-aza-31,32-dioxypentacyclo-(25.3.1.1^{12,16}.0^{4,9}.0^{19,24})dotriaconta-1(31),2,4,6,8,10,12,14,16(32),17,19,21,23,25,27,29-hexadecaene-di-nickel(ii) diperchlorate

Space Group: P-1 ***Cell:*** ***a*** 8.589(3) ***b*** 9.046(4) ***c*** 10.865(4)
Space Group No.: 2 ***(Å,°)*** α 69.98(4) β 87.86(3) γ 64.86(3)

R-Factor (%): 9.00 ***Temperature(K):*** 295 ***Density(g/cm^3):*** 1.835

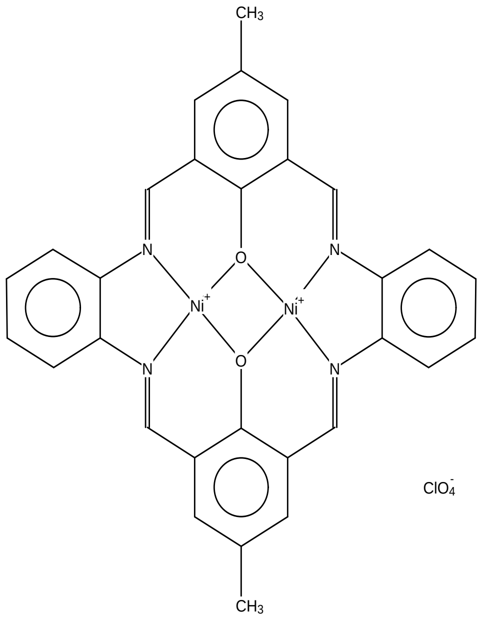

NOCBIR

Reference: Xiaoping Yang, R.A.Jones, Wai-Kwok Wong (2008) *Dalton Trans.* ,1676

Formula: $C_{88} H_{70} Br_8 Eu_5 N_{12} O_{32}{}^{1+}, N_1 O_3{}^{1-}, 3(H_2 O_1)$

Compound Name: bis(μ_2-N,N'-bis(5-Bromo-3-methoxy-2-oxobenzylidene)-1,2-phenylenediamine-N,N',O,O,O',O',O'',O''')-bis(μ_2-N,N'-bis(5-bromo-3-methoxy-2-oxobenzylidene)-1,2-phenylenediamine-N,N',O,O,O',O',O'')-bis(μ_2-nitrato-O,O,O')-dihydroxy-dinitrato-dihydroxy-diaqua-penta-europium(iii) nitrate trihydrate

Space Group:	P-1	***Cell:***	***a*** 17.300(3)	***b*** 18.267(4)	***c*** 20.124(4)		
Space Group No.:	2	***(Å,°)***	α 75.52(3)	β 87.75(4)	γ 74.48(3)		
R-Factor (%):	7.75	***Temperature(K):***	153	***Density(g/cm³):***	1.861		

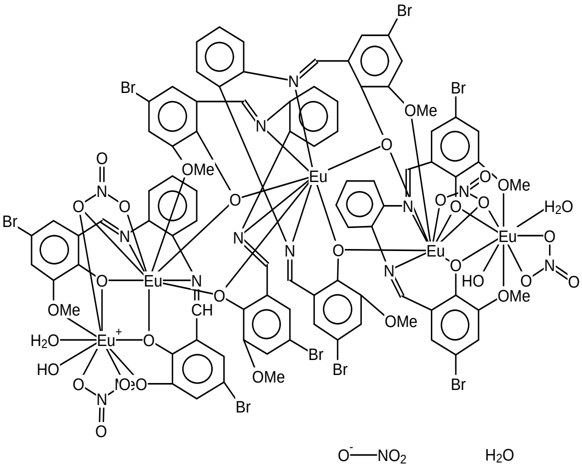

NOCBOX

Reference: Xiaoping Yang, R.A.Jones, Wai-Kwok Wong (2008) *Dalton Trans.* ,1676

Formula: $C_{89} H_{70} Br_8 N_{13} Nd_5 O_{34}, 2(H_2 O_1), 3(C_1 H_4 O_1)$

Compound Name: bis(μ_2-N,N'-bis(5-Bromo-3-methoxy-2-oxobenzylidene)-1,2-phenylenediamine-N,N',O,O,O',O',O'',O''')-bis(μ_2-N,N'-bis(5-bromo-3-methoxy-2-oxobenzylidene)-1,2-phenylenediamine-N,N',O,O,O',O',O'')-bis(μ_2-nitrato-O,O,O')-trinitrato-dihydroxy-methanol-penta-neodymium(iii) methanol solvate dihydrate

Space Group:	P-1	***Cell:***	***a*** 16.878(3)	***b*** 19.282(4)	***c*** 22.601(5)		
Space Group No.:	2	***(Å,°)***	α 96.53(3)	β 102.92(4)	γ 104.75(3)		
R-Factor (%):	7.09	***Temperature(K):***	153	***Density(g/cm³):***	1.636		

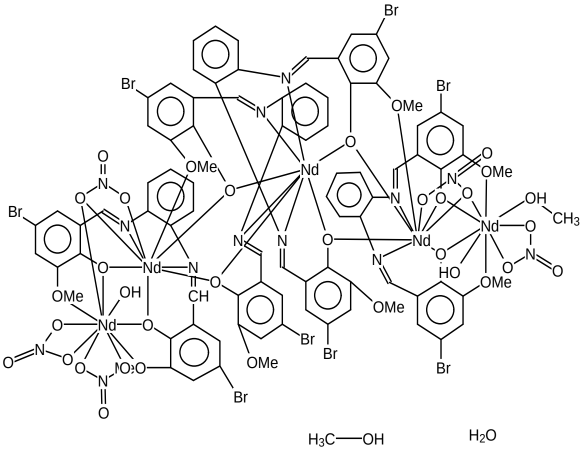

NOCBUD

Reference: Xiaoping Yang, R.A.Jones, Wai-Kwok Wong (2008) *Dalton Trans.* ,1676

Formula: $C_{94} H_{78} Br_8 Eu_5 F_{12} N_8 O_{34} S_4{}^{1+}, C_1 F_3 O_3 S_1{}^{1-}, H_2 O_1$

Compound Name: bis(μ_2-N,N'-bis(5-Bromo-3-methoxy-2-oxobenzylidene)-1,2-phenylenediamine-N,N',O,O,O',O',O'',O''')-bis(μ_2-N,N'-bis(5-bromo-3-methoxy-2-oxobenzylidene)-1,2-phenylenediamine-N,N',O,O,O',O',O'')-bis(μ_2-methoxy)-tetrakis(trifluoromethanesulfonato)-tetraaqua-penta-europium(iii) trifluoromethanesulfonate monohydrate

Space Group:	C2/c	***Cell:***	***a*** 24.973(5)	***b*** 23.003(6)	***c*** 23.628(5)		
Space Group No.:	15	***(Å,°)***	α 90.00	β 105.21(3)	γ 90.00		
R-Factor (%):	4.88	***Temperature(K):***	153	***Density(g/cm³):***	1.920		

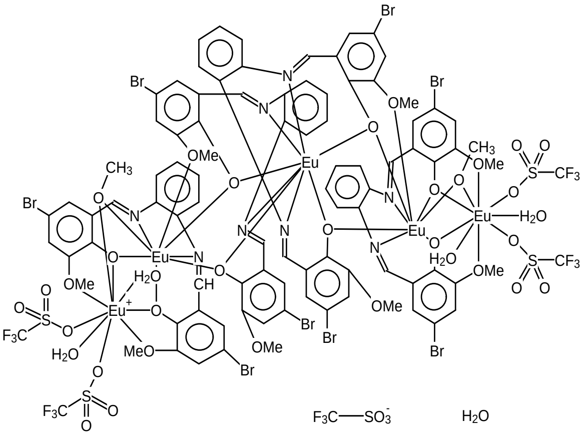

NOGPIJ

Reference: Wei-Yu Bi, Xing-Qiang Lu, Wen-Li Cai, Ji-Rong Song, Wai-Kwok Wong, Xiao-Ping Yang, R.A.Jones (2008) *Z.Anorg.Allg.Chem.* ,**634,**1795

Formula: $C_{27} H_{23} N_6 Nd_1 O_{13} Zn_1$

Compound Name: (μ_2-N,N'-bis(3-methoxysalicylidene)phenylene-1,2-diamine)-tris(nitrato-O,O')-pyridine-neodymium(iii)-zinc(ii)

Space Group:	P21/c	***Cell:***	***a*** 9.460(1)	***b*** 15.648(3)	***c*** 21.552(4)		
Space Group No.:	14	***(Å,°)***	α 90.00	β 100.73(3)	γ 90.00		
R-Factor (%):	6.35	***Temperature(K):***	293	***Density(g/cm³):***	1.799		

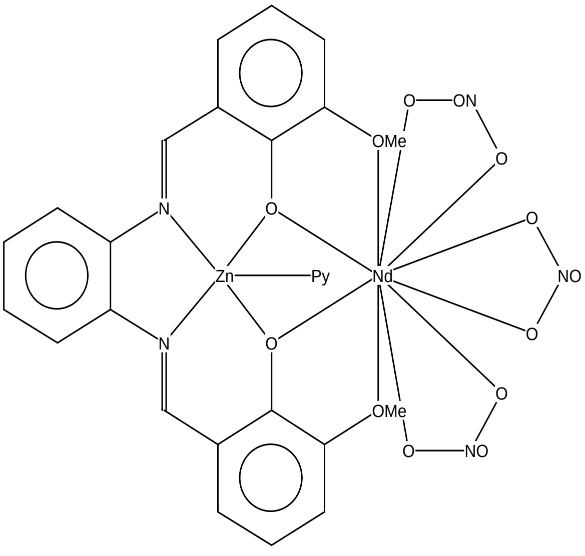

NOGPOP

Reference: Wei-Yu Bi, Xing-Qiang Lu, Wen-Li Cai, Ji-Rong Song, Wai-Kwok Wong, Xiao-Ping Yang, R.A.Jones (2008) *Z.Anorg.Allg.Chem.* ,**634,**1795

Formula: $C_{27}\ H_{21}\ Br_2\ N_6\ Nd_1\ O_{13}\ Zn_1, C_2\ H_3\ N_1$

Compound Name: (μ_2-N,N'-bis(5-bromo-3-methoxysalicylidene)phenylene-1,2-diamine)-tris(nitrato-O,O')-pyridine-neodymium(iii)-zinc(ii) acetonitrile solvate

Space Group:	P21/n	***Cell:***	***a***	12.575(2)	***b***	18.356(3)	***c***	16.086(3)
Space Group No.:	14	***(Å,°)***	α	90.00	β	97.37(0)	γ	90.00

R-Factor (%): 4.77 ***Temperature(K):*** 273 ***Density(g/cm^3):*** 1.890

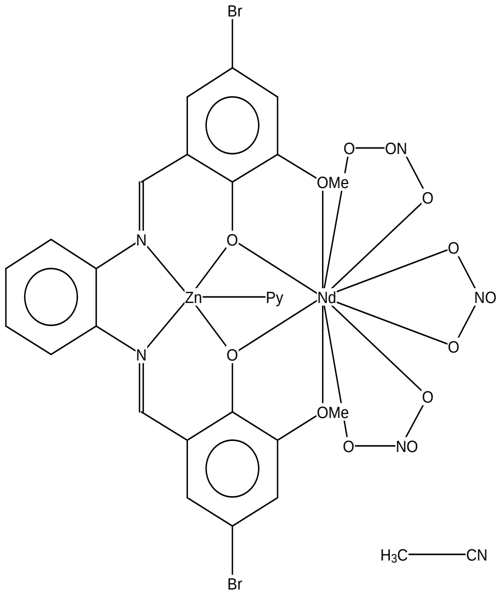

NOMQIQ

Reference: Wei-Yu Bi, Xing-Qiang Lu, Wen-Li Chai, Wen-Juan Jin, Ji-Rong Song, Wai-Kwok Wong (2008) *Inorg.Chem.Commun.* ,**11,**1316

Formula: $C_{27}\ H_{28}\ N_5\ Nd_1\ O_{13}\ Zn_1, C_2\ H_3\ N_1$

Compound Name: (μ_2-acetato)-(μ_2-N,N'-bis(3-methoxysalicylidene)phenylene-1,2-diamine)-bis(nitrato-O,O')-(N,N-dimethylformamide)-neodymium(iii)-zinc(ii) acetonitrile solvate

Space Group:	P-1	***Cell:***	***a***	9.846(2)	***b***	13.450(3)	***c***	13.915(3)
Space Group No.:	2	***(Å,°)***	α	104.10(3)	β	98.40(3)	γ	102.64(3)

R-Factor (%): 6.42 ***Temperature(K):*** 293 ***Density(g/cm^3):*** 1.717

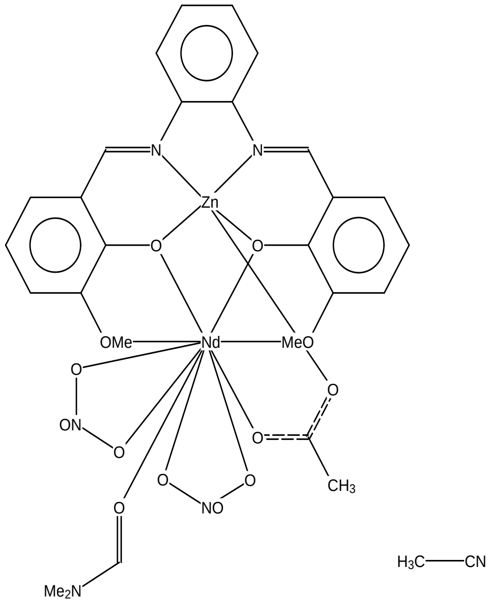

NOMQOW

Reference: Wei-Yu Bi, Xing-Qiang Lu, Wen-Li Chai, Wen-Juan Jin, Ji-Rong Song, Wai-Kwok Wong (2008) *Inorg.Chem.Commun.* ,**11,**1316

Formula: $C_{27}\ H_{28}\ N_5\ Nd_1\ O_{13}\ Zn_1, C_4\ H_8\ O_1$

Compound Name: (μ_2-acetato)-(μ_2-N,N'-bis(3-methoxysalicylidene)phenylene-1,2-diamine)-bis(nitrato-O,O')-(N,N-dimethylformamide)-neodymium(iii)-zinc(ii) tetrahydrofuran solvate

Space Group:	P-1	***Cell:***	***a***	11.433(2)	***b***	12.612(3)	***c***	13.004(3)
Space Group No.:	2	***(Å,°)***	α	102.33(3)	β	103.12(3)	γ	93.18(3)

R-Factor (%): 6.20 ***Temperature(K):*** 293 ***Density(g/cm^3):*** 1.708

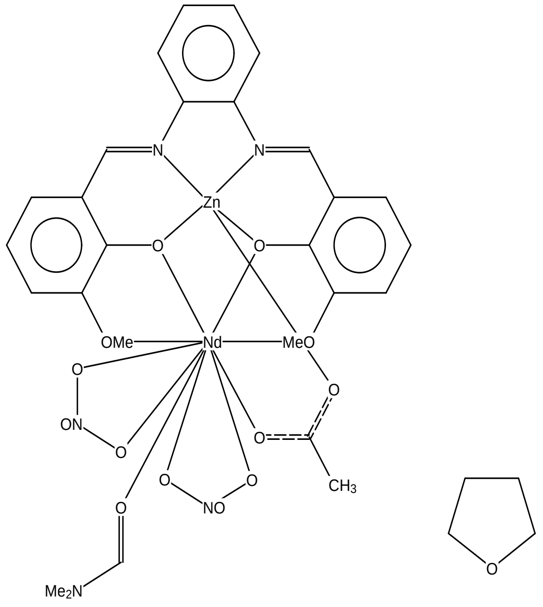

NOMQUC

Reference: Wei-Yu Bi, Xing-Qiang Lu, Wen-Li Chai, Wen-Juan Jin, Ji-Rong Song, Wai-Kwok Wong (2008) *Inorg.Chem.Commun.* ,**11,**1316

Formula: $C_{27}\ H_{28}\ N_5\ Nd_1\ O_{13}\ Zn_1, C_2\ H_6\ O_1$

Compound Name: (μ_2-acetato)-(μ_2-N,N'-bis(3-methoxysalicylidene)phenylene-1,2-diamine)-bis(nitrato-O,O')-(N,N-dimethylformamide)-neodymium(iii)-zinc(ii) ethanol solvate

Space Group:	P-1	***Cell:***	***a***	9.434(1)	***b***	12.536(3)	***c***	16.855(3)
Space Group No.:	2	***(Å,°)***	α	77.68(3)	β	85.72(3)	γ	69.28(3)

R-Factor (%): 8.48 ***Temperature(K):*** 293 ***Density(g/cm^3):*** 1.616

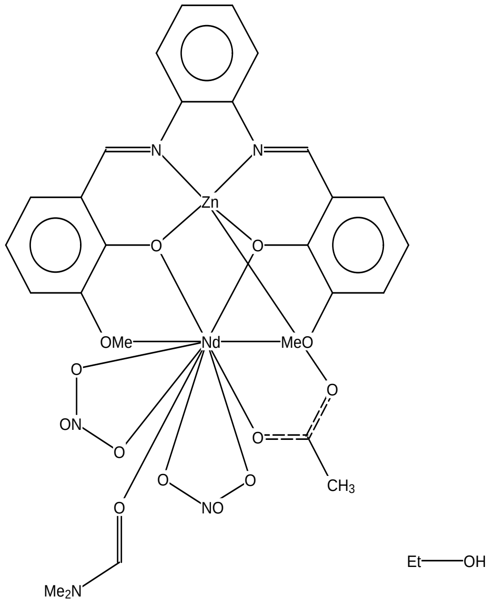

NOQQUG

Reference: Jianxin Xing (2009) *Acta Crystallogr.,Sect.E:Struct.Rep.Online* ,**65,**m469

Formula: C_{21} H_{16} Br_2 Cu_1 N_2 O_3

Compound Name: (2,2'-[4,5-Dibromo-o-phenylenebis(nitrilodimethylidyne)]diphenolato-κ[4]O, N,N',O')-(methanol-κO)-copper(ii)

Space Group: Pnma	***Cell:***	***a*** 19.164(4)	***b*** 19.416(4)	***c*** 5.329(1)
Space Group No.: 62	***(Å,°)***	α 90.00	β 90.00	γ 90.00

R-Factor (%): 4.25 ***Temperature(K):*** 273 ***Density(g/cm³):*** 1.902

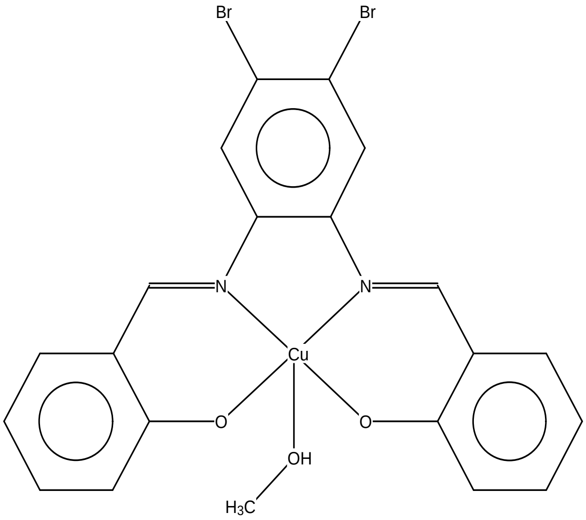

NOQWAR

Reference: Y.Ciringh, S.W.Gordon-Wylie, R.E.Norman, G.R.Clark, S.T.Weintraub, C.P.Horwitz (1997) *Inorg.Chem.* ,**36,**4968

Formula: C_{24} H_{24} Mn_1 N_2 O_3 $^{1+}$,F_6 P_1 $^{1-}$

Compound Name: Aqua-1,3-bis(3,6-dimethylsalicylideneamino)-benzene-manganese(iii) hexafluorophosphate

Space Group: P21/n	***Cell:***	***a*** 12.465(1)	***b*** 13.366(2)	***c*** 15.240(1)
Space Group No.: 14	***(Å,°)***	α 90.00	β 103.05(0)	γ 90.00

R-Factor (%): 5.20 ***Temperature(K):*** 295 ***Density(g/cm³):*** 1.580

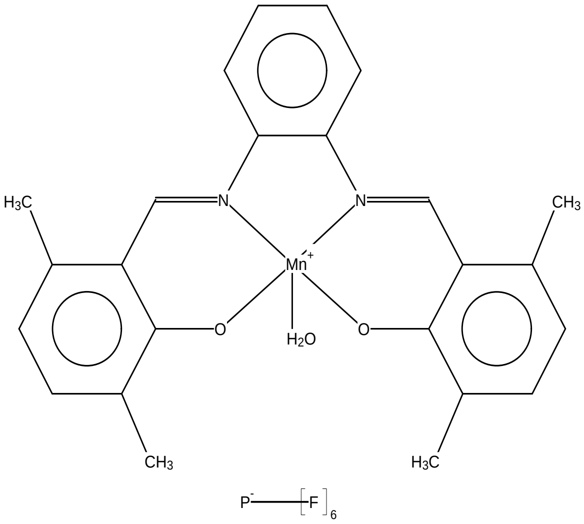

NOQWEV

Reference: Y.Ciringh, S.W.Gordon-Wylie, R.E.Norman, G.R.Clark, S.T.Weintraub, C.P.Horwitz (1997) *Inorg.Chem.* ,**36,**4968

Formula: C_{30} H_{31} Ba_1 F_6 Mn_1 N_2 O_{16} S_2

Compound Name: (μ_2-Acetato)-(μ_2-1,4-diaza-2,3:6,8:19,21-tribenzo-7,9,12,15,18,20-hexaoxacyclodocosa-4,22-diene)-diaqua-bis(trifluoromethylsulfonato)-barium-manganese(iii)

Space Group: P-1	***Cell:***	***a*** 10.747(2)	***b*** 12.271(8)	***c*** 16.206(6)
Space Group No.: 2	***(Å,°)***	α 106.10(4)	β 103.07(2)	γ 106.06(3)

R-Factor (%): 3.52 ***Temperature(K):*** 193 ***Density(g/cm³):*** 1.864

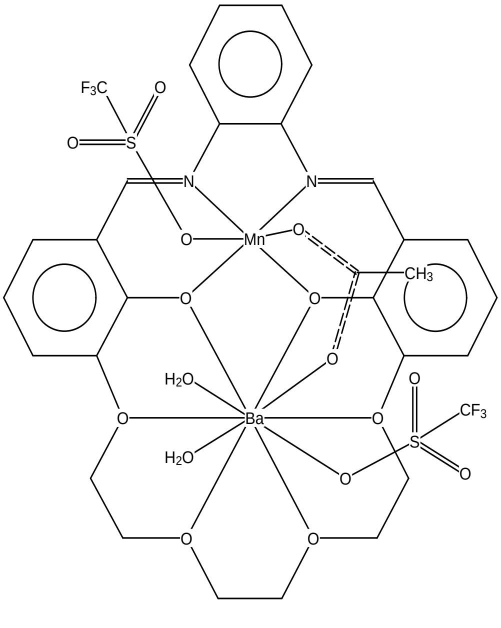

NUSQAU

Reference: Peng Wu, Dik-Lung Ma, Chung-Hang Leung, Siu-Cheong Yan, Nianyong Zhu, R.Abagyan, Chi-Ming Che (2009) *Chem.-Eur.J.* ,**15,**13008

Formula: C_{28} H_{30} Cl_2 N_4 O_2 Pt_1,2(C_1 H_1 Cl_3)

Compound Name: (N,N'-bis(4-(diethylamino)salicylidene)-4,5-dichlorobenzene-1,2-diamine) -platinum chloroform solvate

Space Group: P-1	***Cell:***	***a*** 8.759(2)	***b*** 14.285(3)	***c*** 14.983(3)
Space Group No.: 2	***(Å,°)***	α 103.73(3)	β 90.56(3)	γ 91.05(3)

R-Factor (%): 3.52 ***Temperature(K):*** 263 ***Density(g/cm³):*** 1.750

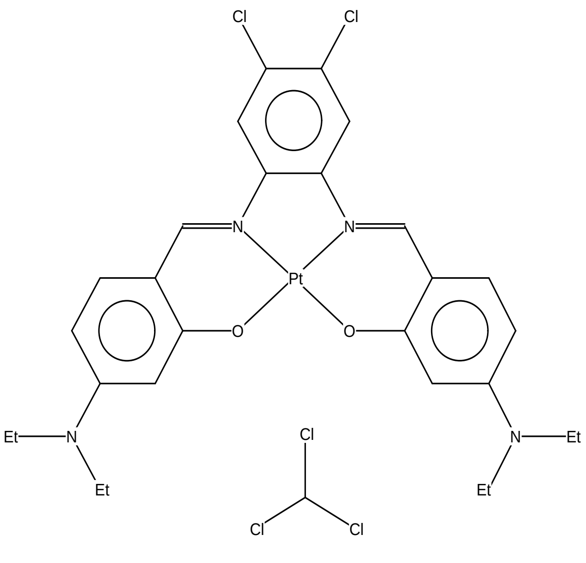

Search: search26 (Tue Dec 2 15:48:50 2014): Hits 429-432

OBAPAI

Reference: F.Averseng, P.G.Lacroix, I.Malfant, G.Lenoble, P.Cassoux, K.Nakatani, I.Maltey-Fanton, J.A.Delaire, A.Aukauloo (1999) *Chem.Mater.* ,**11,**995

Formula: $C_{28}H_{30}N_6Ni_1O_6$,1.5($C_4H_8O_2$)

Compound Name: (N,N'-(4,5-Dinitro-1,2-phenylene)bis(4-(diethylamino)salicylideneaminato))-nickel(ii) 1,4-dioxane solvate

Space Group:	P-1	***Cell:***	***a*** 8.611(1)	***b*** 14.154(2)	***c*** 15.812(2)
Space Group No.:	2	***(Å,°)***	α 113.83(2)	β 98.50(2)	γ 100.27(2)

R-Factor (%): 5.59 ***Temperature(K):*** 180 ***Density(g/cm^3):*** 1.456

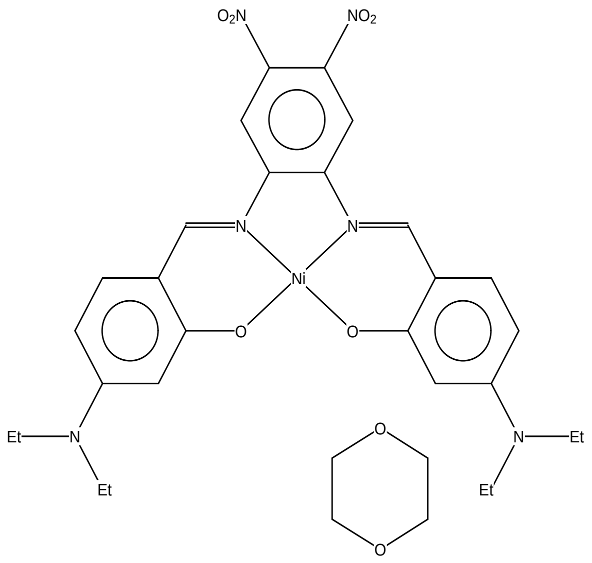

OCEZIG

Reference: O.Rotthaus, O.Jarjayes, F.Thomas, C.Philouze, C.P.Del Valle, E.Saint-Aman, J.-L.Pierre (2006) *Chem.-Eur.J.* ,**12,**2293

Formula: $C_{36}H_{46}N_2Ni_1O_2$

Compound Name: (1,2-bis(3,5-di-t-butyl-2-hydroxybenzimino)benzene)-nickel(ii)

Space Group:	P-1	***Cell:***	***a*** 9.190(2)	***b*** 12.981(3)	***c*** 14.386(4)
Space Group No.:	2	***(Å,°)***	α 107.98(2)	β 93.46(2)	γ 95.56(2)

R-Factor (%): 5.57 ***Temperature(K):*** 150 ***Density(g/cm^3):*** 1.227

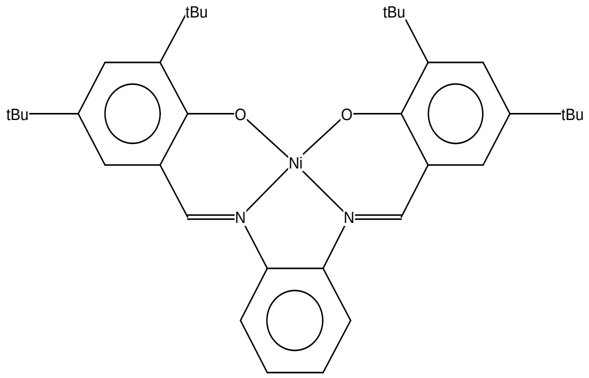

OCOXAH

Reference: R.Herchel, L.Pavelek, Z.Travnicek (2011) *Dalton Trans.* , **40,**11896

Formula: ($C_{29}H_{21}Fe_1N_6O_3$)n,n($C_1H_4O_1$)

Compound Name: catena-((μ_2-2-(((4H-1,2,4-triazol-4-yl)imino)methyl)phenolato)-(amino(2,2'-(1,2-phenylenebis((nitrilo)methylylidene))diphenolato))-di-iron methanol solvate)

Space Group:	P21/c	***Cell:***	***a*** 11.167(0)	***b*** 15.187(0)	***c*** 16.408(0)
Space Group No.:	14	***(Å,°)***	α 90.00	β 105.39(0)	γ 90.00

R-Factor (%): 3.56 ***Temperature(K):*** 100 ***Density(g/cm^3):*** 1.459

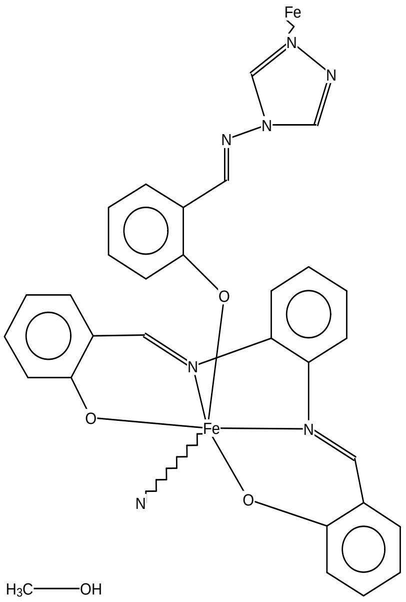

OGOKIF

Reference: M.Cametti, L.Ilander, K.Rissanen (2009) *Inorg.Chem.* ,**48,** 8632

Formula: 2($C_6H_{16}N_1{}^{1+}$),$C_{40}H_{26}N_4O_{12}U_2{}^{2-}$,2($C_3H_6O_1$)

Compound Name: bis(Triethylammonium) bis(μ_2-N-(2,3-dioxidobenzylidene)-N'-(3-hydroxy-2-oxidobenzylidene)-1,2-phenylenediamine-N,N',O,O',O'')-tetra-oxo-di-uranium acetone solvate

Space Group:	Pbca	***Cell:***	***a*** 11.482(0)	***b*** 17.212(0)	***c*** 29.856(0)
Space Group No.:	61	***(Å,°)***	α 90.00	β 90.00	γ 90.00

R-Factor (%): 7.32 ***Temperature(K):*** 173 ***Density(g/cm^3):*** 1.746

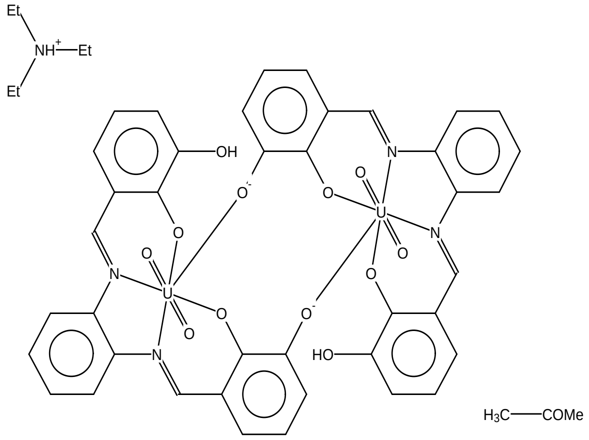

Search: search26 (Tue Dec 2 15:48:50 2014): Hits 433-436

OHIRAZ

Reference: Zhong Yu, T.Shimizu, T.Tominaga, T.Okubo, M.Maekawa, T.Kuroda-Sowa (2009) *Bull.Chem.Soc.Jpn.* ,**82,**1274

Formula: $C_{42}\ H_{32}\ Cl_4\ Fe_2\ N_4\ O_6$

Compound Name: bis(μ_2-2,2'-(1,2-phenylenebis((nitrilo)methylylidene))bis(4-chlorophenolato))-bis(methanol)-di-iron(ii)

Space Group:	C2/c	***Cell:***	***a*** 13.345(11)	***b*** 22.916(17)	***c*** 12.746(10)		
Space Group No.:	15	***(Å,°)***	α 90.00	β 91.73(1)	γ 90.00		
R-Factor (%):	7.32	***Temperature(K):***	117	***Density(g/cm³):***	1.606		

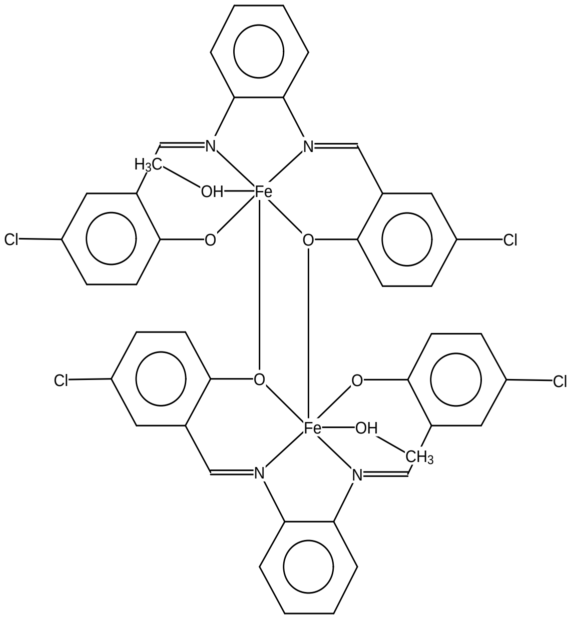

OHORIN

Reference: T.M.Ross, S.M.Neville, D.S.Innes, D.R.Turner, B.Moubaraki, K.S.Murray (2010) *Dalton Trans.* ,**39,**149

Formula: $(C_{34}\ H_{28}\ Fe_1\ N_6\ O_2\ ^{1+})n,n(C_{24}\ H_{20}\ B_1\ ^{1-}),2n(C_2\ H_6\ O_1)$

Compound Name: catena-((μ_2-2,5-bis(4-pyridyl)-3,4-diazahexa-2,4-diene)-(N,N'-bis(salicylidene)benzene-1,2-diamine)-iron(iii) tetraphenylborate ethanol solvate)

Space Group:	Cc	***Cell:***	***a*** 24.299(1)	***b*** 11.894(0)	***c*** 18.189(0)		
Space Group No.:	9	***(Å,°)***	α 90.00	β 93.56(0)	γ 90.00		
R-Factor (%):	8.14	***Temperature(K):***	123	***Density(g/cm³):***	1.291		

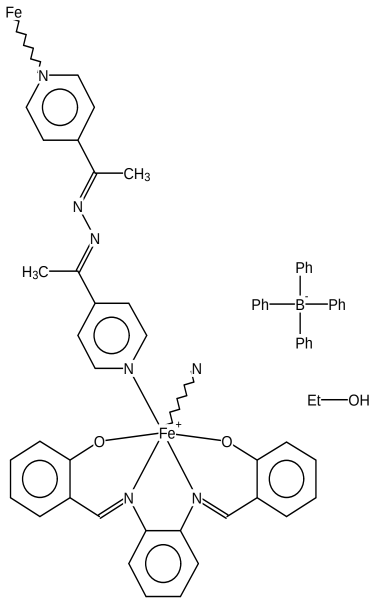

OKOYIW

Reference: Changneng Chen, Deguang Huang, Xiaofeng Zhang, Feng Chen, Hongping Zhu, Qiutian Liu, Cunxi Zhang, Daizheng Liao, Licun Li, Licheng Sun (2003) *Inorg.Chem.* ,**42,**3540

Formula: $C_{21}\ H_{18}\ Mn_1\ N_3\ O_6,C_2\ H_3\ N_1$

Compound Name: (N,N'-1,2-Phenylene-bis(salicylideneiminato-N,N',O,O'))-(methanol)-(nitrato-O)-manganese(iii) acetonitrile solvate

Space Group:	P21/c	***Cell:***	***a*** 9.957(2)	***b*** 7.868(1)	***c*** 25.339(5)		
Space Group No.:	14	***(Å,°)***	α 90.00	β 95.53(3)	γ 90.00		
R-Factor (%):	4.42	***Temperature(K):***	293	***Density(g/cm³):***	1.696		

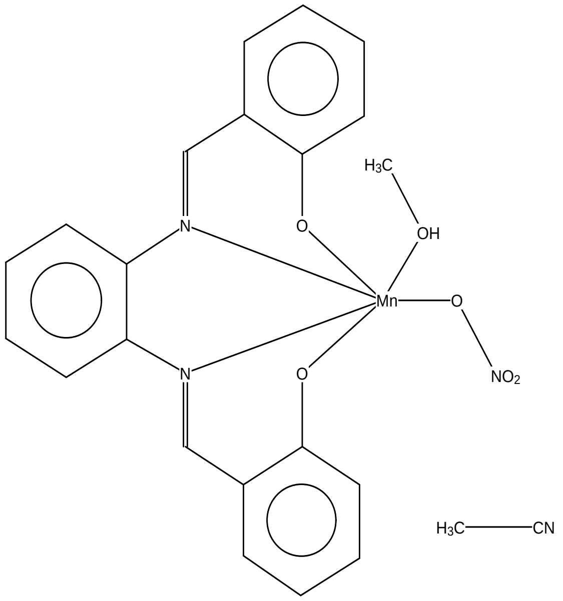

OLASAW

Reference: V.Mougel, P.Horeglad, G.Nocton, J.Pecaut, M.Mazzanti (2010) *Chem.-Eur.J.* ,**16,**14365

Formula: $C_{104}\ H_{104}\ I_2\ K_6\ N_8\ O_{28}\ U_4,2(C_{20}\ H_{40}\ K_1\ O_8\ ^{1+}),2(I_1\ ^{1-}),C_4\ H_8\ O_1$

Compound Name: bis(μ_4-2,2'-(1,2-Phenylenebis(nitrilomethylylidene))diphenolato)-tetrakis(μ_4-oxo)-bis(μ_3-oxo)-bis(μ_3-2,2'-(1,2-phenylenebis(nitrilomethylylidene))diphenolato)-bis(μ_2-18-crown-6)-dioxo-diiodo-tetra-uranium-hexa-potassium bis((18-crown-6)-bis(tetrahydrofuran)-potassium) bis(iodide) tetrahydrofuran solvate

Space Group:	P21/n	***Cell:***	***a*** 20.560(0)	***b*** 18.748(0)	***c*** 22.300(0)		
Space Group No.:	14	***(Å,°)***	α 90.00	β 96.72(0)	γ 90.00		
R-Factor (%):	4.66	***Temperature(K):***	150	***Density(g/cm³):***	1.780		

$K_6U_4[\mu_4\text{-}C_{20}H_{14}N_2O_2\text{-}\kappa^2N,N',\kappa^5O,O']_2[\mu_4\text{-}O]_4$--
--$[\mu_3\text{-}C_{20}H_{14}N_2O_2\text{-}\kappa^2N,N',\kappa^4O,O']_2$--
--$[\mu_3\text{-}O]_2$--
--$[\mu_2\text{-}C_{12}H_{24}O_6\text{-}\kappa^7O,O',O'',O''',O'''',O''''']_2$--
--$[O]_2[I]_2$

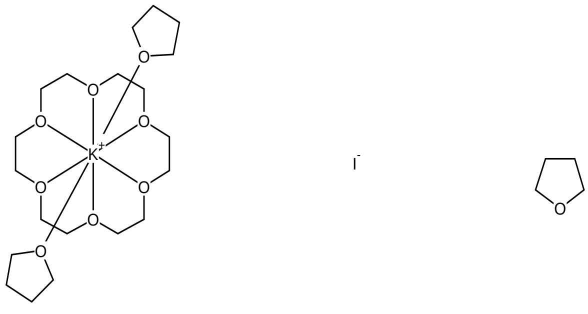

Search: search26 (Tue Dec 2 15:48:50 2014): Hits 437-440

OQAPAY

Reference: C.D.Ene, S.Nastase, C.Maxim, A.M.Madalan, F.Tuna, M.Andruh (2010) *Inorg.Chim.Acta* ,**363,**4247

Formula: $C_{27}\ H_{20}\ Fe_1\ Mn_1\ N_{10}\ O_5\ ^{2-}$,2($C_{22}\ H_{22}\ Mn_1\ N_2\ O_6\ ^{1+}$),8($H_2\ O_1$)

Compound Name: bis(diaqua-(2,2'-(1,2-phenylenebis((nitrilo)methylylidene))bis(6-methoxyphenolato))-manganese(iii)) (μ_2-cyanido)-aqua-(azido)-tetrakis(cyanido)-(2,2'-(1,2-phenylenebis((nitrilo)methylylidene))bis(6-methoxyphenolato))-manganese(iii)-iron(iii) octahydrate

Space Group:	Cc	***Cell:***	***a*** 19.915(1)	***b*** 19.860(1)	***c*** 20.195(2)		
Space Group No.:	9	***(Å,°)***	α 90.00	β 101.81(0)	γ 90.00		
R-Factor (%):	7.96	***Temperature(K):***	293	***Density(g/cm³):***	1.487		

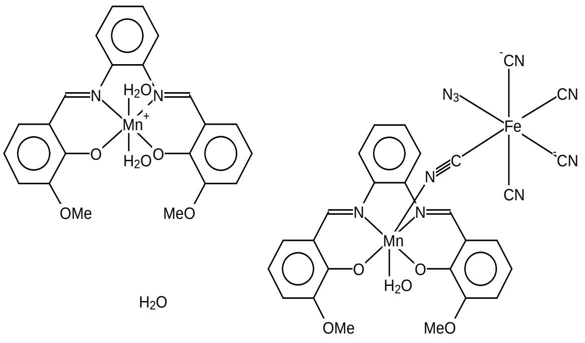

OVONUJ

Reference: P.Bhowmik, H.P.Nayek, M.Corbella, N.Aliaga-Alcalde, S.Chattopadhyay (2011) *Dalton Trans.* ,**40,**7916

Formula: $C_{22}\ H_{20}\ Mn_1\ N_5\ O_5$,2($H_2\ O_1$)

Compound Name: Aqua-(azido)-(2,2'-(1,2-phenylenebis((nitrilo)methylylidene))bis(6-methoxyphenolato))-manganese dihydrate

Space Group:	Pbca	***Cell:***	***a*** 16.069(3)	***b*** 15.616(3)	***c*** 18.099(4)		
Space Group No.:	61	***(Å,°)***	α 90.00	β 90.00	γ 90.00		
R-Factor (%):	6.55	***Temperature(K):***	150	***Density(g/cm³):***	1.537		

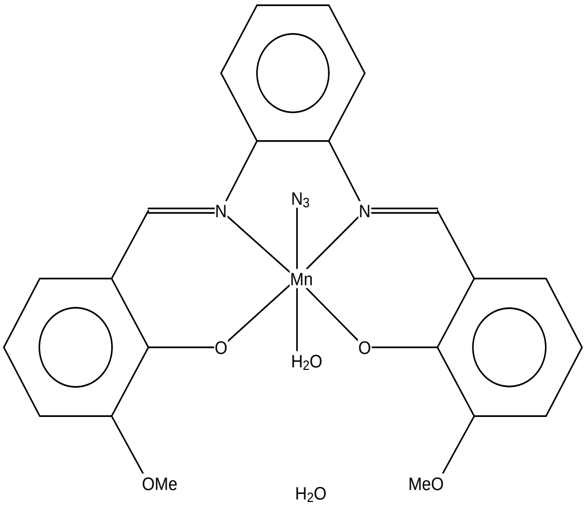

OVOPAR

Reference: P.Bhowmik, H.P.Nayek, M.Corbella, N.Aliaga-Alcalde, S.Chattopadhyay (2011) *Dalton Trans.* ,**40,**7916

Formula: ($C_{20}\ H_{14}\ Mn_1\ N_5\ O_2$)n

Compound Name: catena-((μ_2-azido-N,N'')-(2,2'-(1,2-phenylenebis(nitrilomethylylidene)) diphenolato)-manganese)

Space Group:	Pna21	***Cell:***	***a*** 18.760	***b*** 13.356	***c*** 6.616		
Space Group No.:	33	***(Å,°)***	α 90.00	β 90.00	γ 90.00		
R-Factor (%):	7.26	***Temperature(K):***	293	***Density(g/cm³):***	1.648		

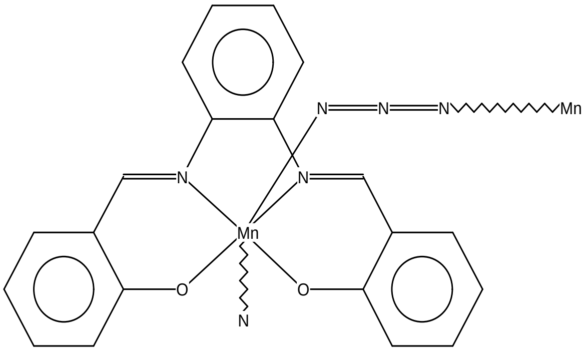

OVOPEV

Reference: P.Bhowmik, H.P.Nayek, M.Corbella, N.Aliaga-Alcalde, S.Chattopadhyay (2011) *Dalton Trans.* ,**40,**7916

Formula: $C_{25}\ H_{27}\ Mn_1\ N_3\ O_6\ ^{1+}$,$Cl_1\ O_4\ ^{1-}$

Compound Name: Aqua-(N,N-dimethylformamide)-(2,2'-(1,2-phenylenebis((nitrilo) methylylidene))bis(6-methoxyphenolato))-manganese perchlorate

Space Group:	P21/n	***Cell:***	***a*** 11.143(0)	***b*** 16.359(0)	***c*** 15.400(0)		
Space Group No.:	14	***(Å,°)***	α 90.00	β 108.42(0)	γ 90.00		
R-Factor (%):	5.09	***Temperature(K):***	293	***Density(g/cm³):***	1.546		

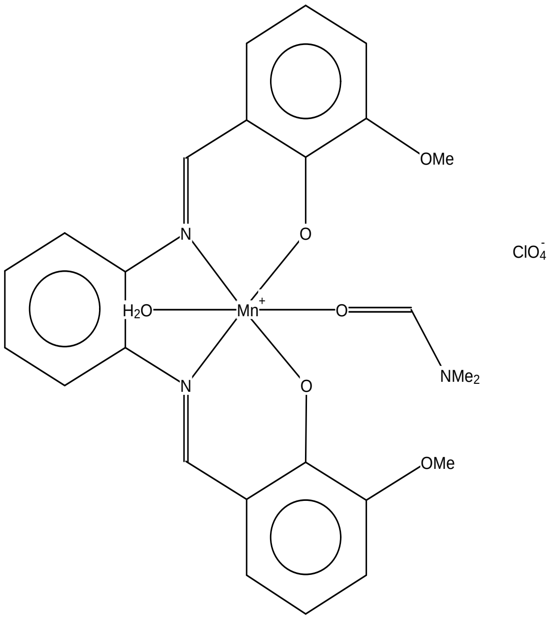

Search: search26 (Tue Dec 2 15:48:50 2014): Hits 441-444

OWEGAZ

Reference: H.Fukumoto, K.Yamane, Y.Kase, T.Yamamoto (2010) *Macromolecules* ,**43,**10366

Formula: $C_{20}\ H_{12}\ Br_2\ N_2\ Ni_1\ O_2$

Compound Name: (2,2'-((3,6-Dibromo-1,2-phenylene)bis((nitrilo)methylylidene))diphenolato) -nickel

Space Group:	Pbca	***Cell:***	***a***	10.084(2)	***b***	14.552(4)	***c***	24.360(6)
Space Group No.:	61	***(Å,°)***	α	90.00	β	90.00	γ	90.00

R-Factor (%): 5.18 ***Temperature(K):*** 293 ***Density(g/cm^3):*** 1.973

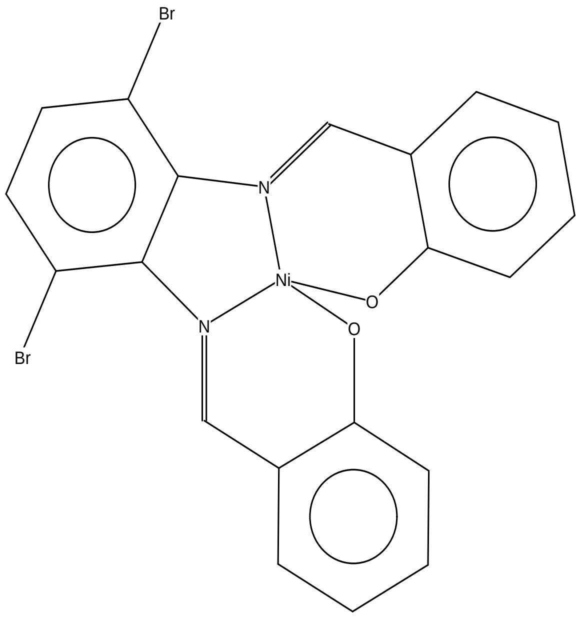

OXDCFE

Reference: S.Gozen, R.Peters, P.G.Owston, P.A.Tasker (1980) *Chem.Commun.* ,1199

Formula: $C_{44}\ H_{28}\ Fe_2\ N_8\ O_5$,0.67($C_3\ H_7\ N_1\ O_1$)

Compound Name: (μ_2-Oxo)-bis(17,18,19,20-tetrahydro-18,19-dioxotribenzo(e,i,m) (1,4,8, 11)tetra-azacyclotetradecinato-iron(iii)) dimethylformamide solvate

Space Group:	P21/c	***Cell:***	***a***	10.819(3)	***b***	26.738(7)	***c***	14.910(4)
Space Group No.:	14	***(Å,°)***	α	90.00	β	101.82(8)	γ	90.00

R-Factor (%): 7.00 ***Temperature(K):*** 295 ***Density(g/cm^3):*** 1.431

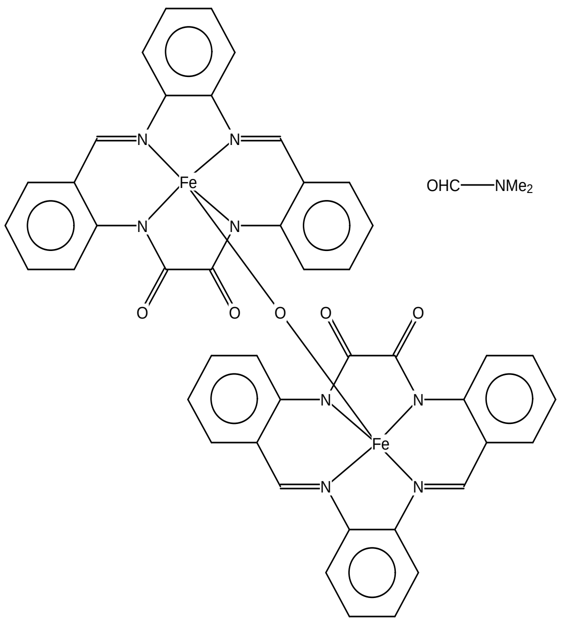

PALHIU

Reference: P.Przychodzen, M.Rams, C.Guyard-Duhayon, B.Sieklucka (2005) *Inorg.Chem.Commun.* ,**8,**350

Formula: 2($C_{22}\ H_{22}\ Mn_1\ N_2\ O_6\ ^{1+}$),$C_{30}\ H_{20}\ Mn_1\ N_{10}\ O_5\ W_1\ ^{2-}$,2($H_2\ O_1$)

Compound Name: bis(Diaqua-(N,N'-(o-phenylene)-bis(3-methoxysalicylideneaminato)-N,N', O,O')-manganese(iii)) (μ_2-cyano)-aqua-heptacyano-(N,N'-(o-phenylene) -bis(3-methoxysalicylideneaminato)-N,N',O,O')-manganese(iii)- tungsten(v) dihydrate

Space Group:	P-1	***Cell:***	***a***	13.494(2)	***b***	17.080(4)	***c***	18.739(5)
Space Group No.:	2	***(Å,°)***	α	86.10(2)	β	82.16(2)	γ	67.60(2)

R-Factor (%): 5.44 ***Temperature(K):*** 295 ***Density(g/cm^3):*** 1.517

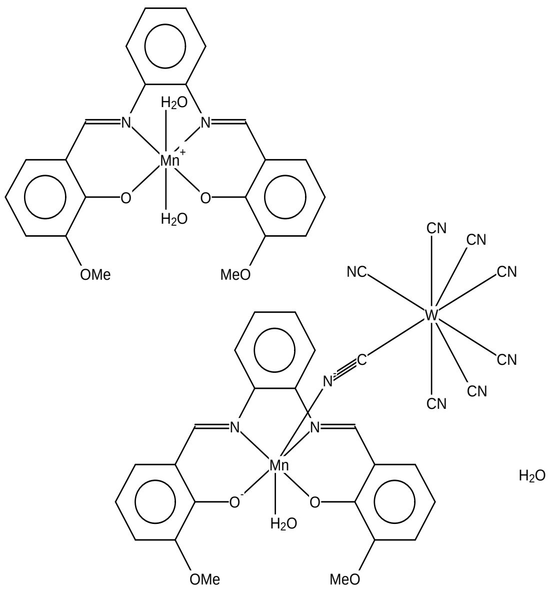

PAWJUS

Reference: E.Solari, C.Maltese, M.Latronico, C.Floriani, A.Chiesi-Villa, C.Rizzoli (1998) *J.Chem.Soc.,Dalton Trans.* ,2395

Formula: $C_{36}\ H_{46}\ Cl_2\ Mo_1\ N_2\ O_2$,$C_4\ H_8\ O_1$

Compound Name: trans-Dichloro-(N,N'-o-phenylene-bis(3,5-di-t-butylsalicylideneiminato))-molybdenum(iv) tetrahydrofuran solvate

Space Group:	P-1	***Cell:***	***a***	13.648(3)	***b***	15.105(5)	***c***	11.149(3)
Space Group No.:	2	***(Å,°)***	α	108.48(3)	β	105.37(2)	γ	63.91(2)

R-Factor (%): 5.07 ***Temperature(K):*** 133 ***Density(g/cm^3):*** 1.334

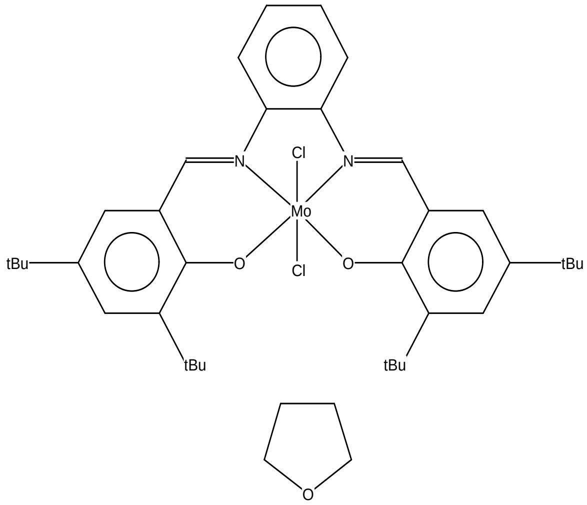

PAYDIC

Reference: H.H.Yao, W.T.Huang, J.M.Lo, F.L.Liao, S.L.Wang (1997) *Eur.J.Solid State Inorg.Chem.* ,**34,**355

Formula: $C_{20}\ H_{14}\ Cu_1\ N_2\ O_2,H_2\ O_1$

Compound Name: (N,N'-bis(Salicyclidene)-trans-1,2-di-iminobenzene)-copper(ii) monohydrate

Space Group: R-3 ***Cell:*** ***a*** 31.678(0) ***b*** 31.678(0) ***c*** 9.083(0)
Space Group No.: 148 ***(Å,°)*** α 90.00 β 90.00 γ 120.00

R-Factor (%): 5.37 ***Temperature(K):*** 295 ***Density(g/cm^3):*** 1.499

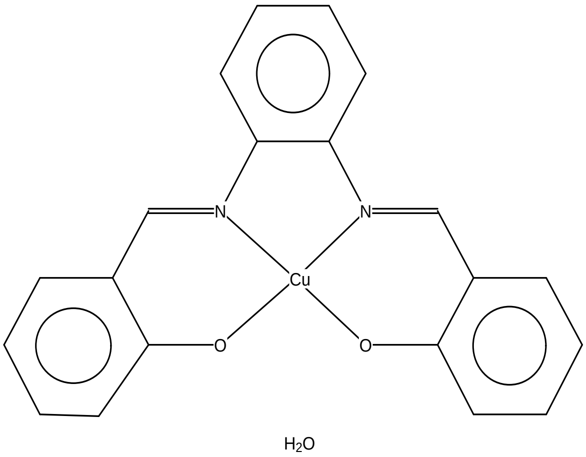

PAYDIC01

Reference: Shu-Sheng Zhang, Bo Yang, Yan-Fang Wang, Xue-Mei Li, Kui Jiao (2005) *Asian J.Chem.* ,**17,**929

Formula: $C_{20}\ H_{14}\ Cu_1\ N_2\ O_2,H_2\ O_1$

Compound Name: (N,N'-1,2-phenylenebis(salicylideneiminato)-N,N',O,O')-copper(ii) monohydrate

Space Group: R-3 ***Cell:*** ***a*** 31.677(5) ***b*** 31.677(5) ***c*** 9.041(1)
Space Group No.: 148 ***(Å,°)*** α 90.00 β 90.00 γ 120.00

R-Factor (%): 6.79 ***Temperature(K):*** 293 ***Density(g/cm^3):*** 1.506

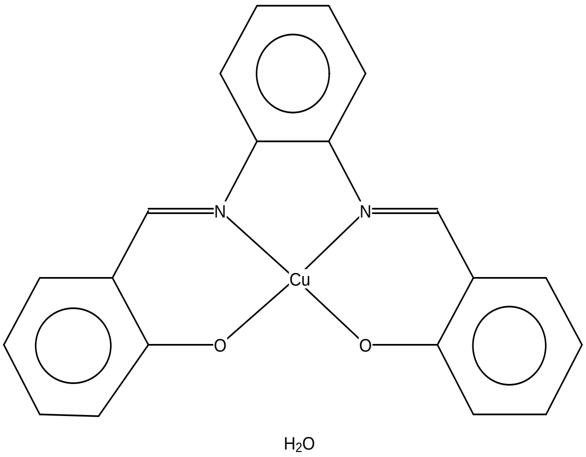

PECPEU

Reference: Dan Wang, Xiaojian Kong, Xiaozeng Li (2012) *Z.Kristallogr.-New Cryst.Struct.* ,**227,**191

Formula: $C_{44}\ H_{28}\ Cd_1\ Cl_2\ N_8\ Ni_2\ O_4$

Compound Name: Dichloro-bis(μ_2-tribenzo-[d,h,l][3,7,10,14]-tetraaza[14]annulene-1,2-diolato)-cadmium(ii)-di-nickel(ii)

Space Group: P2/n ***Cell:*** ***a*** 12.660(6) ***b*** 10.090(5) ***c*** 14.706(7)
Space Group No.: 13 ***(Å,°)*** α 90.00 β 95.53(0) γ 90.00

R-Factor (%): 5.46 ***Temperature(K):*** 113 ***Density(g/cm^3):*** 1.836

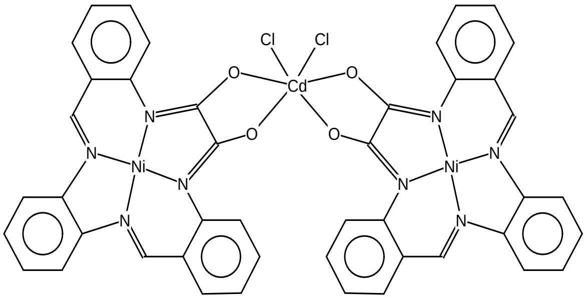

PEGWUV

Reference: Cong-Ming Zhang, Xi-Feng Gao, Mei Zhu, Yun-Gai Li, Qing-Lun Wang, Li-Cun Li (2013) *J.Mol.Struct.* ,**1033,**8

Formula: $(C_{34}\ H_{28}\ Mn_1\ N_6\ O_2\ ^{1+})n,n(Cl_1\ O_4\ ^{1-}),2n(H_2\ O_1)$

Compound Name: catena-((μ_2-1,1'-(1,4-Phenylenebis(methylene))-bis(1H-imidazole))-(2,2'-(1,2-phenylenebis((nitrilo-N)methylylidene))-diphenolato)-manganese perchlorate dihydrate)

Space Group: C2/c ***Cell:*** ***a*** 11.455(3) ***b*** 15.070(3) ***c*** 20.728(4)
Space Group No.: 15 ***(Å,°)*** α 90.00 β 103.70(0) γ 90.00

R-Factor (%): 7.28 ***Temperature(K):*** 293 ***Density(g/cm^3):*** 1.420

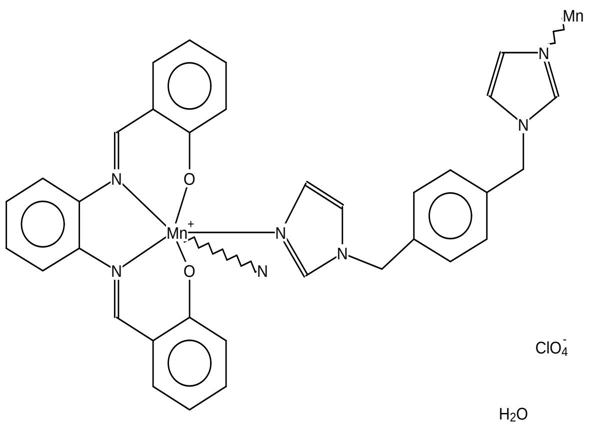

Search: search26 (Tue Dec 2 15:48:50 2014): Hits 449-452

PEJXAE

Reference: R.Prabhakaran, Rui Huang, K.Natarajan (2006) *Inorg.Chim.Acta* ,**359,**3359

Formula: $C_{46}\ H_{33}\ As_1\ Cl_1\ N_2\ O_2\ Ru_1,0.5(C_1\ H_2\ Cl_2)$

Compound Name: (N,N'-bis(2-oxy-1-naphthaldehyde)-o-phenylenediamine-N,N',O,O')-chloro-(triphenylarsine)-ruthenium(iii) dichloromethane solvate

Space Group: P-1 ***Cell:*** ***a*** 11.986(2) ***b*** 14.176(3) ***c*** 23.032(5)
Space Group No.: 2 ***(Å,°)*** α 82.19(3) β 89.87(3) γ 86.91(3)

R-Factor (%): 6.14 ***Temperature(K):*** 173 ***Density(g/cm3):*** 1.544

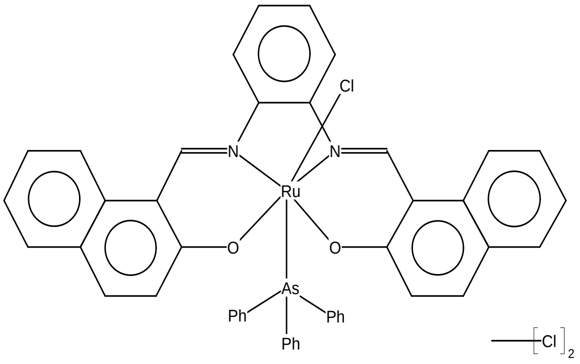

PEJXEI

Reference: R.Prabhakaran, Rui Huang, K.Natarajan (2006) *Inorg.Chim.Acta* ,**359,**3359

Formula: $C_{46}\ H_{33}\ Br_1\ N_2\ O_2\ P_1\ Ru_1,2(C_1\ H_2\ Cl_2)$

Compound Name: (N,N'-bis(2-oxy-1-naphthaldehyde)-o-phenylenediamine-N,N',O,O')-bromo-(triphenylphosphine)-ruthenium(iii) dichloromethane solvate

Space Group: P21/c ***Cell:*** ***a*** 16.838(3) ***b*** 11.071(2) ***c*** 24.201(5)
Space Group No.: 14 ***(Å,°)*** α 90.00 β 100.46(3) γ 90.00

R-Factor (%): 3.42 ***Temperature(K):*** 173 ***Density(g/cm3):*** 1.538

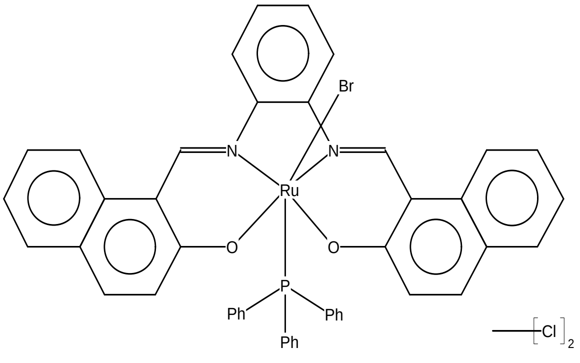

PENYOY

Reference: G.Cosquer, F.Pointillart, B.Le Guennic, Y.Le Gal, S.Golhen, O.Cador, L.Ouahab (2012) *Inorg.Chem.* ,**51,**8488

Formula: $C_{45}\ H_{29}\ Cu_1\ F_{18}\ N_2\ O_8\ S_6\ Y_1$

Compound Name: (μ_2-2,2'-((2-(4,5-bis(n-Propylsulfanyl)-1,3-dithiol-2-ylidene)-1,3-benzodithiole-5,6-diyl)bis(nitrilomethylylidene))diphenolato)-tris(1,1,1,5,5,5-hexafluoroacetylacetonato-O,O')-copper(ii)-yttrium(iii)

Space Group: P-1 ***Cell:*** ***a*** 12.660(50) ***b*** 13.750(50) ***c*** 17.510(70)
Space Group No.: 2 ***(Å,°)*** α 86.55(4) β 87.99(4) γ 67.17(2)

R-Factor (%): 0.00 ***Temperature(K):*** 293 ***Density(g/cm3):*** 1.673

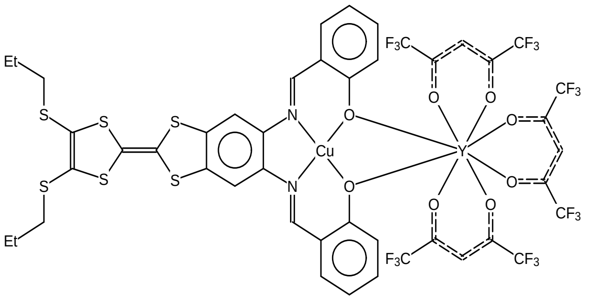

PENYUE

Reference: G.Cosquer, F.Pointillart, B.Le Guennic, Y.Le Gal, S.Golhen, O.Cador, L.Ouahab (2012) *Inorg.Chem.* ,**51,**8488

Formula: $C_{45}\ H_{29}\ Cu_1\ Er_1\ F_{18}\ N_2\ O_8\ S_6$

Compound Name: (μ_2-2,2'-((2-(4,5-bis(n-Propylsulfanyl)-1,3-dithiol-2-ylidene)-1,3-benzodithiole-5,6-diyl)bis(nitrilomethylylidene))diphenolato)-tris(1,1,1,5,5,5-hexafluoroacetylacetonato-O,O')-copper(ii)-erbium(iii)

Space Group: P-1 ***Cell:*** ***a*** 12.636(50) ***b*** 13.828(50) ***c*** 17.495(50)
Space Group No.: 2 ***(Å,°)*** α 86.99(4) β 87.30(4) γ 66.18(2)

R-Factor (%): 0.00 ***Temperature(K):*** 293 ***Density(g/cm3):*** 1.774

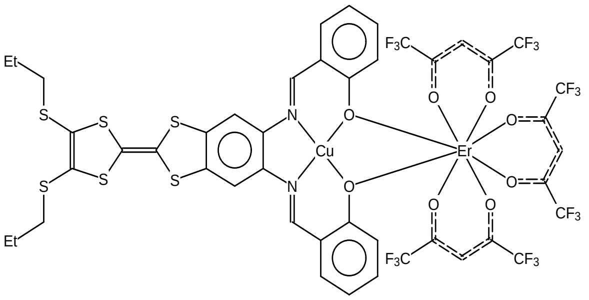

Search: search26 (Tue Dec 2 15:48:50 2014): Hits 453-456

PENZAL

Reference:	G.Cosquer, F.Pointillart, B.Le Guennic, Y.Le Gal, S.Golhen, O.Cador, L.Ouahab (2012) *Inorg.Chem.* ,**51,**8488
Formula:	C_{45} H_{29} Er_1 F_{18} N_2 Ni_1 O_8 S_6
Compound Name:	(μ_2-2,2'-((2-(4,5-bis(n-Propylsulfanyl)-1,3-dithiol-2-ylidene)-1,3-benzodithiole-5,6-diyl)bis(nitrilomethylylidene))diphenolato)-tris(1,1,1,5,5,5-hexafluoroacetylacetonato-O,O')-erbium(iii)-nickel(ii)

Space Group:	P-1	***Cell:***	***a*** 13.000(50)	***b*** 13.520(50)	***c*** 16.500(60)		
Space Group No.:	2	***(Å,°)***	α 77.32(0)	β 87.24(0)	γ 86.02(0)		
R-Factor (%):	0.00	***Temperature(K):***	293	***Density(g/cm³):***	1.750		

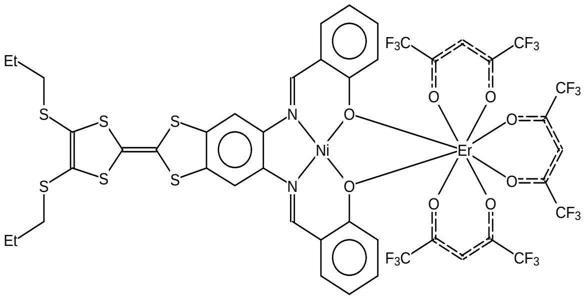

PENZEP

Reference:	G.Cosquer, F.Pointillart, B.Le Guennic, Y.Le Gal, S.Golhen, O.Cador, L.Ouahab (2012) *Inorg.Chem.* ,**51,**8488
Formula:	C_{45} H_{29} F_{18} N_2 Ni_1 O_8 S_6 Yb_1
Compound Name:	(μ_2-2,2'-((2-(4,5-bis(n-Propylsulfanyl)-1,3-dithiol-2-ylidene)-1,3-benzodithiole-5,6-diyl)bis(nitrilomethylylidene))diphenolato)-tris(1,1,1,5,5,5-hexafluoroacetylacetonato-O,O')-nickel(ii)-ytterbium(iii)

Space Group:	P-1	***Cell:***	***a*** 13.110(40)	***b*** 13.370(20)	***c*** 16.510(60)		
Space Group No.:	2	***(Å,°)***	α 77.23(4)	β 87.40(5)	γ 86.00(5)		
R-Factor (%):	0.00	***Temperature(K):***	293	***Density(g/cm³):***	1.761		

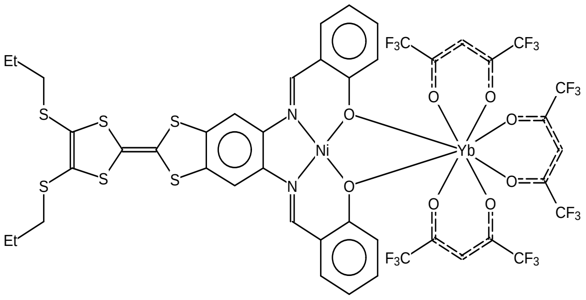

PEQFEX

Reference:	Xiao-Ping Yang, R.A.Jones, M.M.Oye, A.L.Holmes, Wai-Kwok Wong (2006) *Cryst.Growth Des.* ,**6,**2122
Formula:	C_{70} H_{54} Br_6 Cl_1 N_6 O_{16} Yb_3,0.5(C_2 H_3 N_1),1.5(C_1 H_4 O_1),3(H_2 O_1)
Compound Name:	bis(μ_2-N,N'-bis(5-Bromo-3-methoxysalicylidene)phenylene-1,2-diamine)-(μ_2-acetato-O,O')-(acetato-O,O')-(N,N'-bis(5-bromo-3-methoxysalicylidene)phenylene-1,2-diamine)-chloro-tri-ytterbium(iii) acetonitrile methanol solvate trihydrate

Space Group:	P-1	***Cell:***	***a*** 21.653(4)	***b*** 21.919(4)	***c*** 22.130(4)		
Space Group No.:	2	***(Å,°)***	α 86.26(3)	β 77.68(3)	γ 67.69(3)		
R-Factor (%):	5.98	***Temperature(K):***	153	***Density(g/cm³):***	1.674		

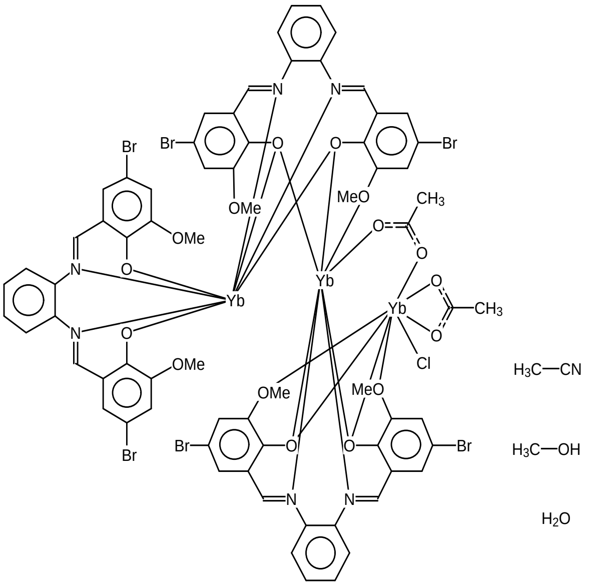

PIFFEQ

Reference:	N.E.Eltayeb, S.G.Teoh, S.Chantrapromma, Hoong-Kun Fun, K.Ibrahim (2007) *Acta Crystallogr.,Sect.E:Struct.Rep.Online* ,**63,**m1633
Formula:	C_{22} H_{20} N_2 O_5 Zn_1
Compound Name:	Aqua-(4,4'-dimethoxy-2,2'-(1,2-phenylenebis(nitrilomethylidyne))diphenolato-κ⁴O,O',N,N')-zinc(ii)

Space Group:	Pmn21	***Cell:***	***a*** 23.362(0)	***b*** 4.912(0)	***c*** 7.943(0)		
Space Group No.:	31	***(Å,°)***	α 90.00	β 90.00	γ 90.00		
R-Factor (%):	2.71	***Temperature(K):***	100	***Density(g/cm³):***	1.668		

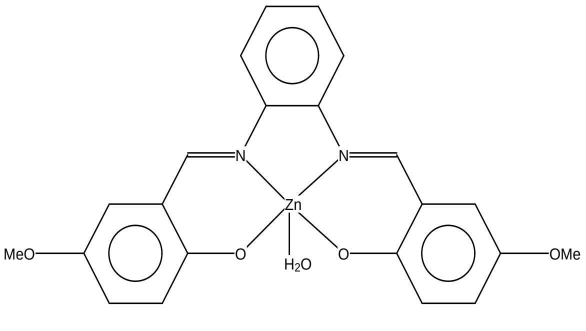

Search: search26 (Tue Dec 2 15:48:50 2014): Hits 457-460

PIPKOO

Reference: K.Brychcy, K.Drager, K.-J.Jens, M.Tilset, U.Behrens (1994) *Chem.Ber.* ,**127**,465

Formula: $C_{42}\ H_{48}\ Cu_2\ N_6\ O_4\ ^{2+}$,2($Cl_1\ O_4\ ^{1-}$)

Compound Name: (μ_2-14,29-Di-t-butyl-3,10,18,25-tetra-azapentacyclo(25.3.1.1^{12,16}0^{4,9}0^{19,24})-dotriaconta-1(31),2,4,6,8,10,12,14,16(32),17,19,21,23,25,27,29-hexadeca-en-31,32-diolato-N,N',N'',N''',O,O')-bis(dimethylformamide)-di-copper(ii) diperchlorate

Space Group:	P21/c	***Cell:***	***a***	9.332(2)	***b***	21.721(8)	***c***	11.174(3)
Space Group No.:	14	***(Å,°)***	α	90.00	β	102.61(2)	γ	90.00

R-Factor (%): 5.30 ***Temperature(K):*** 295 ***Density(g/cm^3):*** 1.543

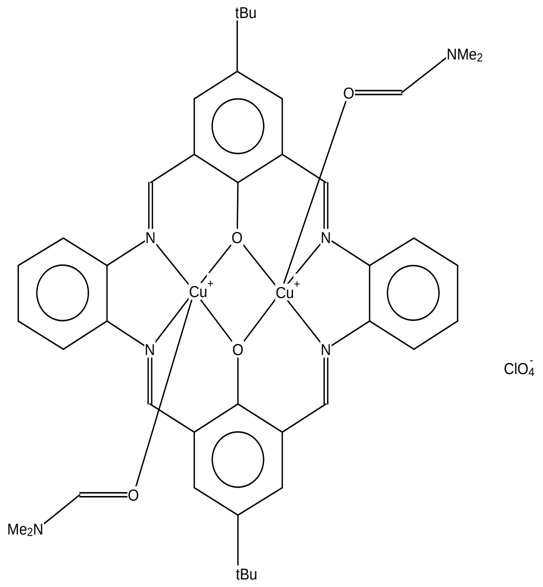

PIPKUU

Reference: K.Brychcy, K.Drager, K.-J.Jens, M.Tilset, U.Behrens (1994) *Chem.Ber.* ,**127**,465

Formula: $C_{39}\ H_{41}\ Cl_1\ Cu_2\ N_5\ O_7\ ^{1+}$,$Cl_1\ O_4\ ^{1-}$,2($C_3\ H_7\ N_1\ O_1$)

Compound Name: (μ_2-14,29-Di-t-butyl-3,10,18,25-tetra-azapentacyclo(25.3.1.1^{12,16}.0^{4,} $_9$.0^{19,24})-dotriaconta-1(31),2,4,6,8,10,12,14,16(32),17,19,21,23,25,27,29-hexadecaen-31,32-diolato-N,N',N'',N''',O,O')-dimethylformamide-(perchlorato-O)-di-copper(ii) perchlorate dimethylformamide solvate

Space Group:	P21/n	***Cell:***	***a***	10.276(2)	***b***	22.397(5)	***c***	20.685(5)
Space Group No.:	14	***(Å,°)***	α	90.00	β	94.00(7)	γ	90.00

R-Factor (%): 6.49 ***Temperature(K):*** 173 ***Density(g/cm^3):*** 1.538

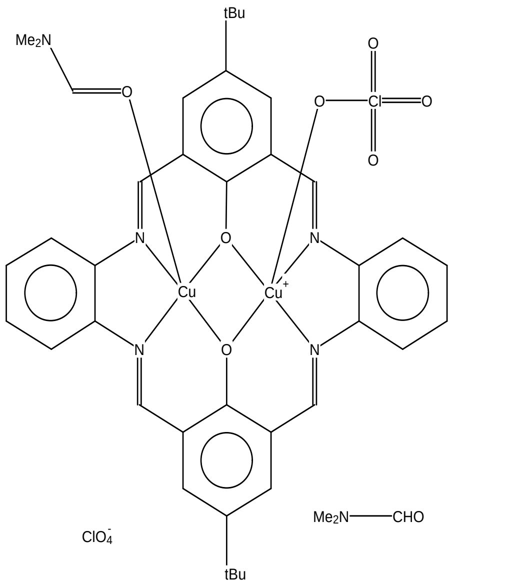

PIPLAB

Reference: K.Brychcy, K.Drager, K.-J.Jens, M.Tilset, U.Behrens (1994) *Chem.Ber.* ,**127**,465

Formula: $C_{36}\ H_{26}\ Cl_2\ Cu_2\ F_8\ N_4\ O_{10}$

Compound Name: (μ_2-14,29-Di-t-butyl-5,6,7,8,20,21,22,23-octafluoro-3,10,18,25-tetra-azapentacyclo(25.3.1.1^{12,16}0^{4,9}0^{19,24})-dotriaconta-1(31),2,4,6,8,10,12,14,16(32),17,19,21,23,25,27,29-hexadeca-en-31,32-diolato-N,N',N'',N''',O,O')-bis(μ_2-perchlorato-O,O')-di-copper(ii)

Space Group:	P21/c	***Cell:***	***a***	8.082(4)	***b***	13.605(2)	***c***	18.419(9)
Space Group No.:	14	***(Å,°)***	α	90.00	β	97.91(2)	γ	90.00

R-Factor (%): 5.83 ***Temperature(K):*** 173 ***Density(g/cm^3):*** 1.696

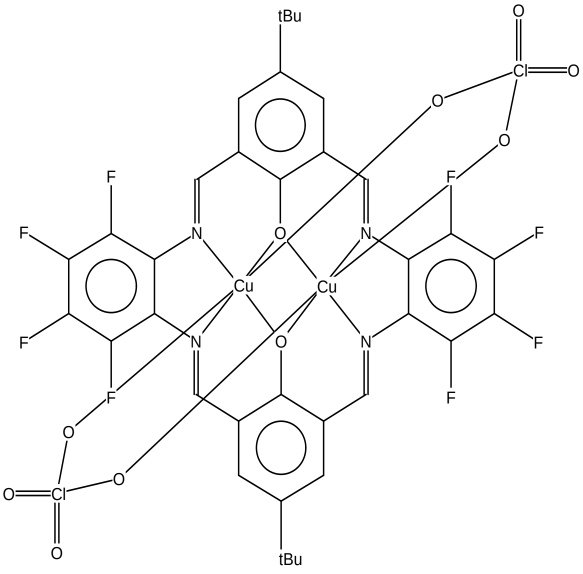

PIPLEF

Reference: K.Brychcy, K.Drager, K.-J.Jens, M.Tilset, U.Behrens (1994) *Chem.Ber.* ,**127**,465

Formula: $C_{36}\ H_{16}\ Cl_2\ Cu_2\ F_{22}\ N_4\ O_{12}$,$C_4\ H_{10}\ O_1$,$C_1\ H_4\ O_1$

Compound Name: (μ_2-14,29-bis(Heptafluoro-2-propyl)-5,6,7,8,20,21,22,23-octafluoro-3,10,18,25-tetra-azapentacyclo(25.3.1.1^{12,16}.0^{4,9}.0^{19,24})-dotriaconta-hexadecaen-31,32-diolato)-bis((methanolato-O)-(perchlorato-O))-di-copper(ii) methanol diethyl ether solvate

Space Group:	P21/c	***Cell:***	***a***	15.785(2)	***b***	8.127(2)	***c***	19.638(3)
Space Group No.:	14	***(Å,°)***	α	90.00	β	92.24(2)	γ	90.00

R-Factor (%): 5.53 ***Temperature(K):*** 173 ***Density(g/cm^3):*** 1.872

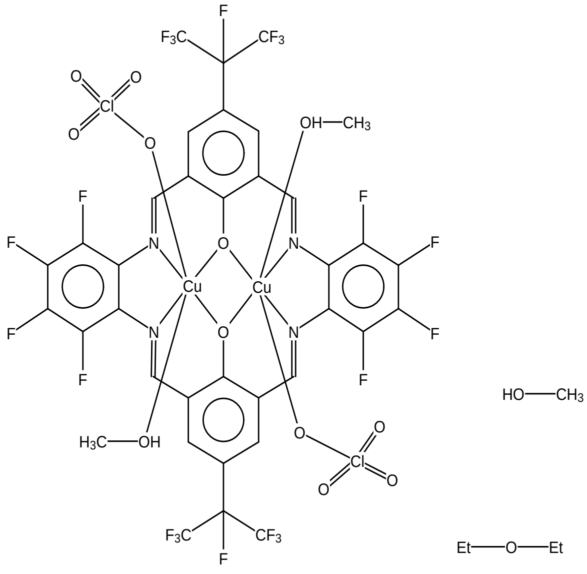

Search: search26 (Tue Dec 2 15:48:50 2014): Hits 461-464

PIYCEF

Reference: D.M.Rudkevich, W.Verboom, Z.Brzozka, M.J.Palys, W.P.R.V.Stauthamer, G.J.van.Hummel, S.M.Franken, S.Harkema, J.F.J.Engbersen, D.N.Reinhoudt (1994) *J.Am.Chem.Soc.* ,**116,**4341

Formula: $C_{16} H_{36} N_1{}^{1+}, C_{22} H_{20} N_2 O_{10} P_1 U_1{}^{1-}, C_2 H_3 N_1$

Compound Name: Tetra-n-butylammonium ((2,2'-(1,2-phenylene-bis(nitrilomethylidyne))-bis(6-methoxyphenolato))-N,N',O,O')-dioxo-(dihydrogenphosphato-O)-uranium acetonitrile solvate

Space Group: P-1 ***Cell:*** ***a*** 9.569(2) ***b*** 13.190(3) ***c*** 18.423(2)
Space Group No.: 2 ***(Å,°)*** α 90.22(1) β 102.48(1) γ 106.18

R-Factor (%): 5.10 ***Temperature(K):*** 170 ***Density(g/cm³):*** 1.565

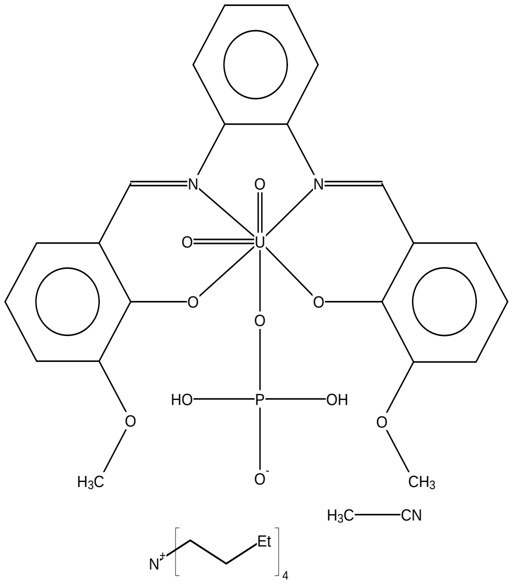

PIYCOP

Reference: D.M.Rudkevich, W.Verboom, Z.Brzozka, M.J.Palys, W.P.R.V.Stauthamer, G.J.van.Hummel, S.M.Franken, S.Harkema, J.F.J.Engbersen, D.N.Reinhoudt (1994) *J.Am.Chem.Soc.* ,**116,**4341

Formula: $2(C_{16} H_{36} N_1{}^{1+}), C_{38} H_{34} N_4 O_{12} P_1 U_1{}^{1-}, H_2 O_4 P_1{}^{1-}, 3(C_2 H_3 N_1)$

Compound Name: bis(Tetra-n-butylammonium) ((2,2'-(1,2-phenylene-bis(nitrilomethylidyne(2-hydroxy-3,1-phenylene)oxy))-bis(n-(4-methylphenyl)acetamidato))-N,N',O,O')-dioxo-(dihydrogenphosphato-O)-uranium dihydrogenphosphate acetonitrile solvate

Space Group: P-1 ***Cell:*** ***a*** 12.461(2) ***b*** 15.532(1) ***c*** 22.440(3)
Space Group No.: 2 ***(Å,°)*** α 82.40(1) β 76.77(1) γ 84.76(2)

R-Factor (%): 8.40 ***Temperature(K):*** 170 ***Density(g/cm³):*** 1.360

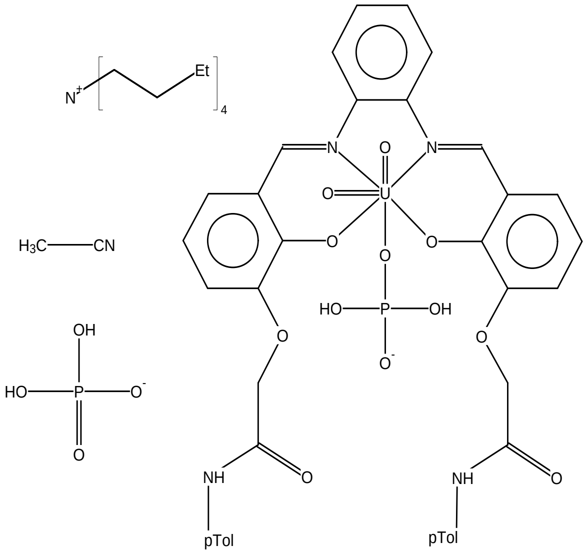

PIYCUW

Reference: E.C.Escudero-Adan, J.Benet-Buchholz, A.W.Kleij (2008) *Dalton Trans.* ,734

Formula: $C_{40} H_{52} N_4 O_2 Zn_1$

Compound Name: (N,N'-bis(3,5-Di-t-butylsalicylidene)benzene-1,2-diamine)-(N-methylimidazole)-zinc

Space Group: Ibam ***Cell:*** ***a*** 32.987(1) ***b*** 9.826(0) ***c*** 23.020(1)
Space Group No.: 72 ***(Å,°)*** α 90.00 β 90.00 γ 90.00

R-Factor (%): 3.07 ***Temperature(K):*** 100 ***Density(g/cm³):*** 1.222

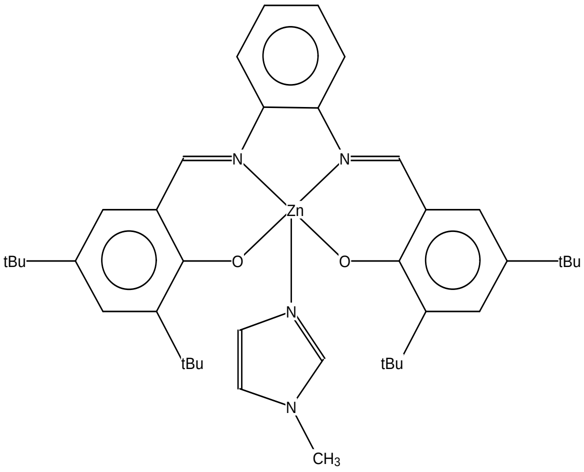

PIYDAD

Reference: E.C.Escudero-Adan, J.Benet-Buchholz, A.W.Kleij (2008) *Dalton Trans.* ,734

Formula: $C_{44} H_{54} N_4 O_2 Zn_1$

Compound Name: (N,N'-bis(3,5-Di-t-butylsalicylidene)benzene-1,2-diamine)-(N-methylbenzimidazole)-zinc

Space Group: P-1 ***Cell:*** ***a*** 9.479(0) ***b*** 14.673(1) ***c*** 14.975(1)
Space Group No.: 2 ***(Å,°)*** α 103.50(0) β 107.77(0) γ 91.80(0)

R-Factor (%): 3.32 ***Temperature(K):*** 100 ***Density(g/cm³):*** 1.276

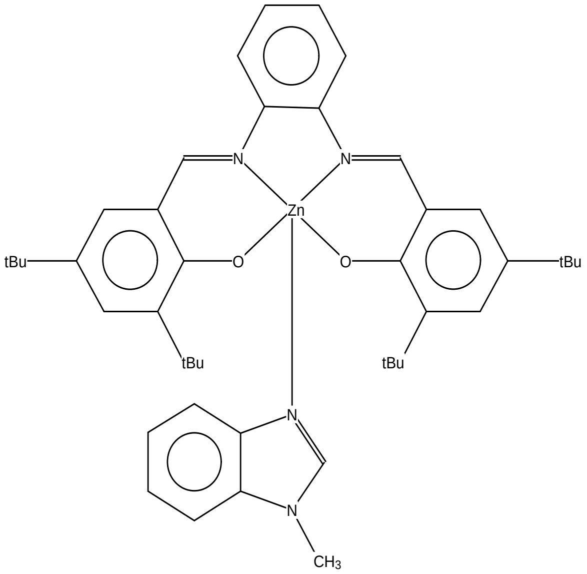

PODGOE

Reference: K.Brychcy, K.-J.Jens, M.Tilset, U.Behrens (1994)
Chem.Ber. ,**127,**991

Formula: $C_{72} H_{68} Cu_2 Mn_1 N_8 O_4{}^{2+}$,2($Cl_1 O_4{}^{1-}$),2($C_2 H_3 N_1$)

Compound Name: (bis(μ_2-14,29-Di-t-butyl-3,10,18,25-tetra-azapentacyclo(25.3.1.1^{12,16}.0^{4,9}.0^{19,24})dotriaconta-1(31),2,4,6,8,10,12,14,16(32),17,19,21,23,25,27,29-hexadecaen-31,32-diolato-N,N',O,O'-copper)-N'',N''',O,O')-manganese diperchlorate acetonitrile solvate

Space Group:	P21/n	***Cell:***	***a*** 14.333(2)	***b*** 17.338(2)	***c*** 28.533(4)
Space Group No.:	14	***(Å,°)***	α 90.00	β 103.74(2)	γ 90.00

R-Factor (%): 4.20 ***Temperature(K):*** 153 ***Density(g/cm^3):*** 1.516

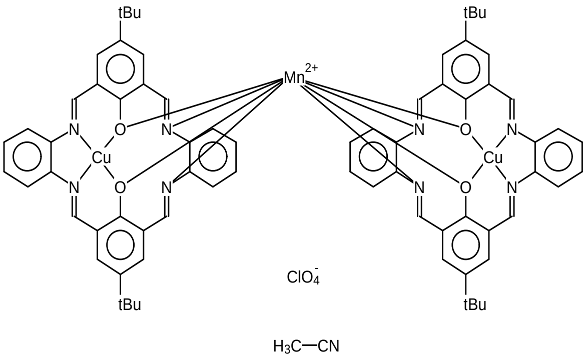

POFFOG

Reference: H.-K.Fun, R.Kia, V.Mirkhani, H.Zargoshi (2008)
Acta Crystallogr.,Sect.E:Struct.Rep.Online ,**64,**m1181

Formula: $C_{20} H_{14} N_2 Ni_1 O_4$,2($H_2 O_1$)

Compound Name: (5,5'-Dihydroxy-2,2'-(o-phenylenebis(nitrilomethylidyne))diphenolato)-nickel(ii) dihydrate

Space Group:	C2/c	***Cell:***	***a*** 10.905(0)	***b*** 17.660(0)	***c*** 9.037(0)
Space Group No.:	15	***(Å,°)***	α 90.00	β 101.15(0)	γ 90.00

R-Factor (%): 4.84 ***Temperature(K):*** 100 ***Density(g/cm^3):*** 1.716

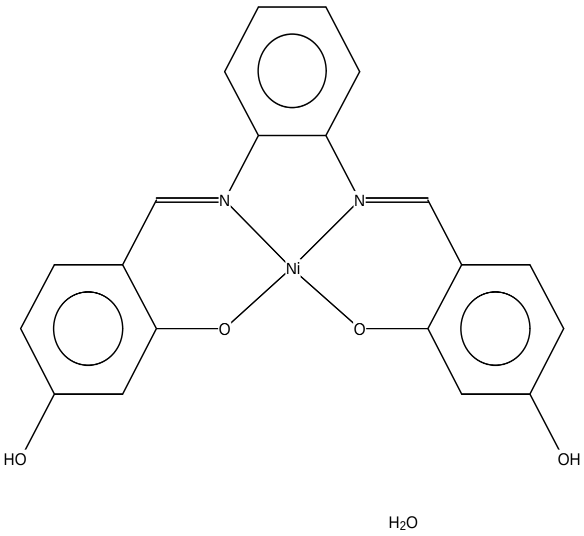

POFFOG01

Reference: A.Arola-Arnal, J.Benet-Buchholz, S.Neidle, R.Vilar (2008)
Inorg.Chem. ,**47,**11910

Formula: $C_{20} H_{14} N_2 Ni_1 O_4$,2($H_2 O_1$)

Compound Name: (N,N'-bis(4-Hydroxysalicylidene)benzene-1,2-diamine-N,N',O,O')-nickel(ii) dihydrate

Space Group:	C2/c	***Cell:***	***a*** 10.894(0)	***b*** 17.652(0)	***c*** 9.052(0)
Space Group No.:	15	***(Å,°)***	α 90.00	β 101.29(0)	γ 90.00

R-Factor (%): 2.83 ***Temperature(K):*** 100 ***Density(g/cm^3):*** 1.716

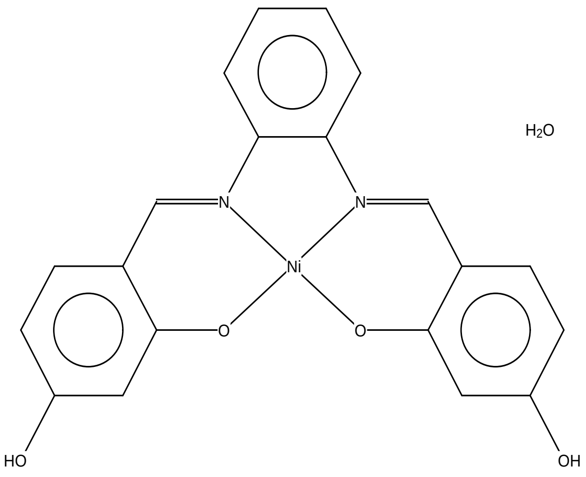

POWHIT

Reference: Yu Wu, Bin Xie, Li-Ke Zou, Wei-Ping Wu, Lu Lu (2009)
Acta Crystallogr.,Sect.E:Struct.Rep.Online ,**65,**m758

Formula: $C_{23} H_{17} Br_4 Cu_1 N_3 O_3$,$C_3 H_7 N_1 O_1$

Compound Name: (N,N-Dimethylformamide-O)-(4,4',6,6'-tetrabromo-2,2'-(o-phenylenebis(nitrilomethylidyne))diphenolato-N,N',O,O')-copper(ii) N,N-dimethylformamide solvate

Space Group:	P-1	***Cell:***	***a*** 7.374(1)	***b*** 11.954(1)	***c*** 17.212(2)
Space Group No.:	2	***(Å,°)***	α 94.21(0)	β 100.31(0)	γ 104.12(0)

R-Factor (%): 3.50 ***Temperature(K):*** 93 ***Density(g/cm^3):*** 1.941

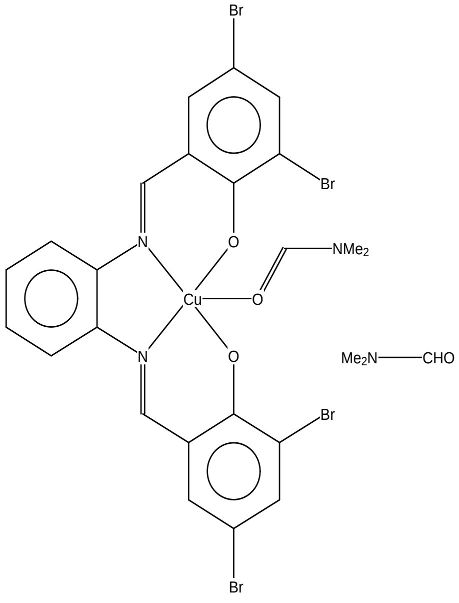

Search: search26 (Tue Dec 2 15:48:50 2014): Hits 469-472

POXMIZ

Reference: Wei-Yu Bi, Xing-Qiang Lu, Wen-Li Chai, Tao Wei, Ji-Rong Song, Shun-Sheng Zhao, Wai-Kwok Wong (2009) *Inorg.Chem.Commun.* ,**12,**267

Formula: $C_{31} H_{29} N_6 Nd_1 O_{13} Zn_1$

Compound Name: (μ_2-N,N'-bis(3-methoxysalicylidene)ethylene-1,2-diamine)-(μ_2-isonicotinato)-(dimethylformamide)-bis(nitrato-O,O')-neodymium(iii)-zinc(ii)

Space Group:	P21/n	***Cell:***	***a***	9.017(1)	***b***	17.063(3)	***c***	22.300(3)
Space Group No.:	14	***(Å,°)***	α	90.00	β	100.54(0)	γ	90.00

R-Factor (%): 3.55 ***Temperature(K):*** 293 ***Density(g/cm^3):*** 1.779

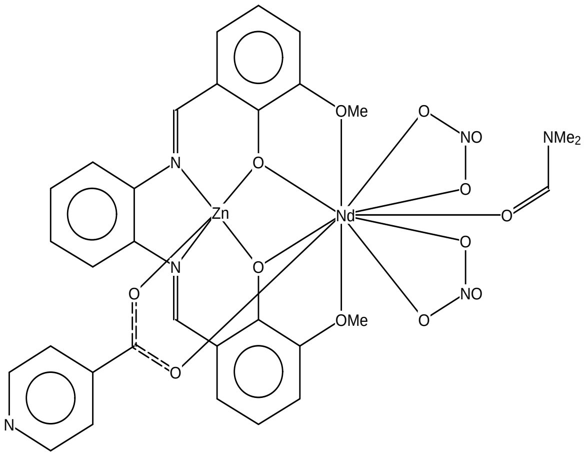

PSALCT

Reference: M.B.Ferrari, G.G.Fava, C.Pelizzi (1976) *Acta Crystallogr.,Sect.B:Struct.Crystallogr.Cryst.Chem.* ,**32,**901

Formula: $C_{20} H_{14} Cu_1 N_2 O_2,C_1 H_4 N_2 S_1$

Compound Name: (N,N'-o-Phenylenebis(salicylaldiminato))-copper(ii) thiourea

Space Group:	P-1	***Cell:***	***a***	11.460(10)	***b***	16.870(20)	***c***	10.940(10)
Space Group No.:	2	***(Å,°)***	α	74.30(10)	β	85.70(10)	γ	80.60(10)

R-Factor (%): 6.50 ***Temperature(K):*** 295 ***Density(g/cm^3):*** 1.502

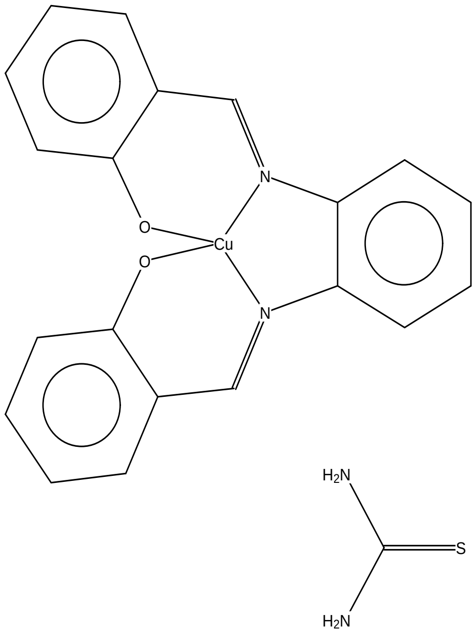

PSALTH

Reference: R.J.Hill, C.E.F.Rickard (1978) *J.Inorg.Nucl.Chem.* ,**40,**2029

Formula: $C_{40} H_{28} N_4 O_4 Th_1$

Compound Name: bis(N,N'-o-Phenylene-bis(salicylaldiminato))-thorium(iv)

Space Group:	C2/c	***Cell:***	***a***	15.587(1)	***b***	12.726(1)	***c***	16.204(2)
Space Group No.:	15	***(Å,°)***	α	90.00	β	91.72(0)	γ	90.00

R-Factor (%): 5.50 ***Temperature(K):*** 295 ***Density(g/cm^3):*** 1.779

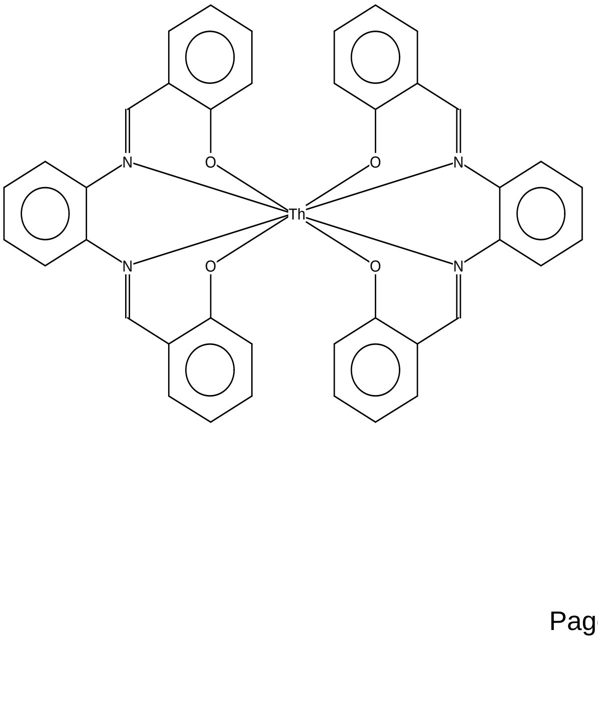

PSALTQ

Reference: P.Cassoux, A.Gleizes (1980) *Inorg.Chem.* ,**19,**665

Formula: $2(C_{20} H_{14} Cu_1 N_2 O_2),C_{12} H_4 N_4$

Compound Name: N,N'-(1,2-Phenylene)-bis(salicylaldiminato)-copper(ii)-7,7,8,8-tetracyanoquinodimethane complex

Space Group:	P-1	***Cell:***	***a***	11.970(4)	***b***	12.567(4)	***c***	7.020(2)
Space Group No.:	2	***(Å,°)***	α	103.44(2)	β	92.35(2)	γ	92.48(2)

R-Factor (%): 3.90 ***Temperature(K):*** 295 ***Density(g/cm^3):*** 1.556

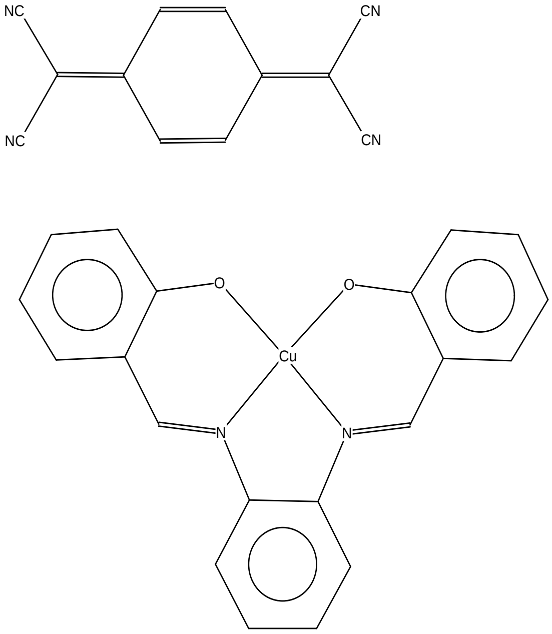

Search: search26 (Tue Dec 2 15:48:50 2014): Hits 473-476

PUHKUY

Reference: A.Gleizes, M.Julve, N.Kuzmina, A.Alikhanyan, F.Lloret, I.Malkerova, J.L.Sanz, F.Senocq (1998) *Eur.J.Inorg.Chem.* ,1169

Formula: $C_{35}\ H_{17}\ Cu_1\ F_{18}\ N_2\ O_8\ Y_1$

Compound Name: (N,N'-o-Phenylenebis(salicylidenealdiminato))-tris(hexafluoroacetylacetonato)-copper(ii)-yttrium(iii)

Space Group:	P21/n	***Cell:***	***a***	10.295(4)	***b***	20.815(6)	***c***	19.349(6)
Space Group No.:	14	***(Å,°)***	α	90.00	β	101.53(4)	γ	90.00

R-Factor (%): 6.50 ***Temperature(K):*** 295 ***Density(g/cm^3):*** 1.779

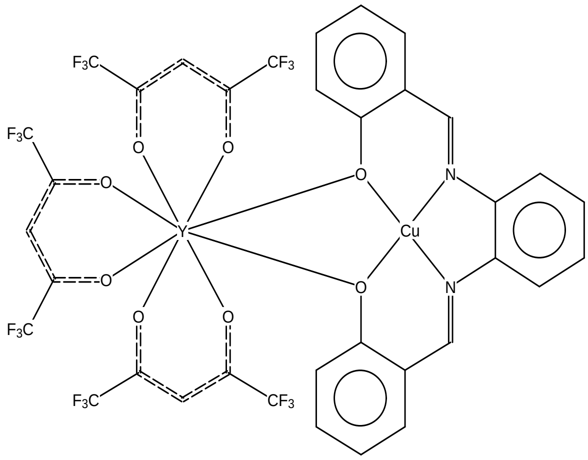

PUHLEJ

Reference: A.Gleizes, M.Julve, N.Kuzmina, A.Alikhanyan, F.Lloret, I.Malkerova, J.L.Sanz, F.Senocq (1998) *Eur.J.Inorg.Chem.* ,1169

Formula: $C_{35}\ H_{17}\ F_{18}\ La_1\ N_2\ Ni_1\ O_8$

Compound Name: (N,N'-o-Phenylenebis(salicylidenealdiminato))-tris(hexafluoroacetylacetonato)-lanthanum(iii)-nickel(ii)

Space Group:	P21/n	***Cell:***	***a***	17.200(100)	***b***	22.410(60)	***c***	19.700(300)
Space Group No.:	14	***(Å,°)***	α	90.00	β	90.40(90)	γ	90.00

R-Factor (%): 0.00 ***Temperature(K):*** 295 ***Density(g/cm^3):*** 1.982

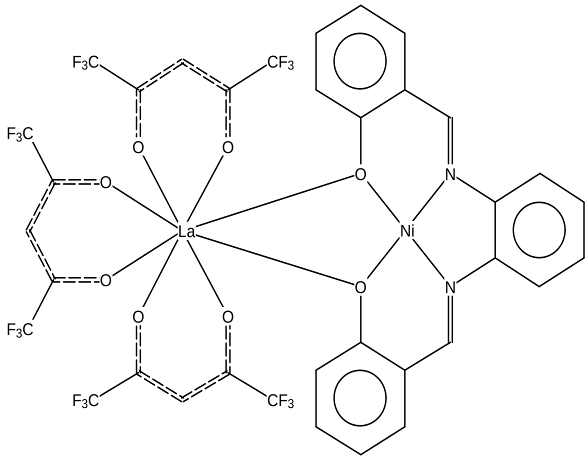

PUHLIN

Reference: A.Gleizes, M.Julve, N.Kuzmina, A.Alikhanyan, F.Lloret, I.Malkerova, J.L.Sanz, F.Senocq (1998) *Eur.J.Inorg.Chem.* ,1169

Formula: $C_{35}\ H_{17}\ F_{18}\ N_2\ Ni_1\ O_8\ Y_1$

Compound Name: (N,N'-o-Phenylenebis(salicylidenealdiminato))-tris(hexafluoroacetylacetonato)-nickel(ii)-yttrium(iii)

Space Group:	P21/n	***Cell:***	***a***	17.290(10)	***b***	22.349(6)	***c***	19.590(10)
Space Group No.:	14	***(Å,°)***	α	90.00	β	90.12(6)	γ	90.00

R-Factor (%): 0.00 ***Temperature(K):*** 295 ***Density(g/cm^3):*** 1.901

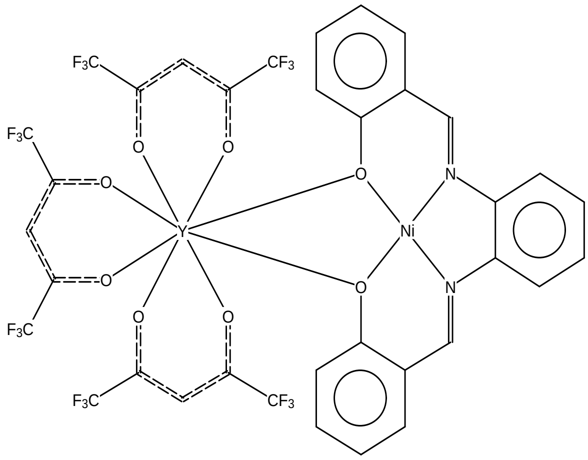

PUHLOT

Reference: A.Gleizes, M.Julve, N.Kuzmina, A.Alikhanyan, F.Lloret, I.Malkerova, J.L.Sanz, F.Senocq (1998) *Eur.J.Inorg.Chem.* ,1169

Formula: $C_{35}\ H_{17}\ F_{18}\ N_2\ Ni_1\ O_8\ Yb_1$

Compound Name: (N,N'-o-Phenylenebis(salicylidenealdiminato))-tris(hexafluoroacetylacetonato)-nickel(ii)-ytterbium(iii)

Space Group:	P21/n	***Cell:***	***a***	17.191(3)	***b***	22.056(6)	***c***	19.563(4)
Space Group No.:	14	***(Å,°)***	α	90.00	β	90.18(2)	γ	90.00

R-Factor (%): 0.00 ***Temperature(K):*** 295 ***Density(g/cm^3):*** 2.090

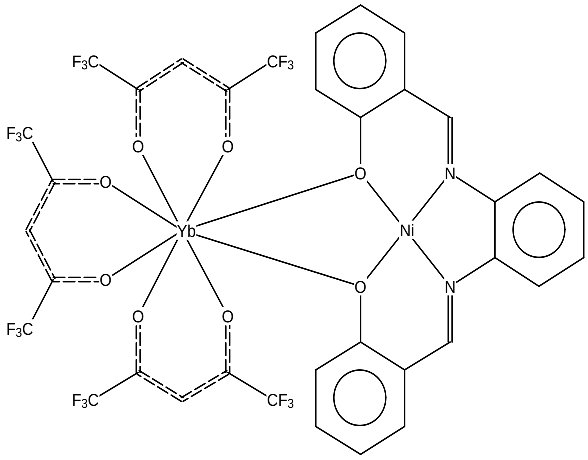

PUKTOE

Reference: R.Hernandez-Molina, A.Mederos, P.Gili, S.Dominguez, P.Nunez, G.Germain, T.Debaerdemaeker (1997) *Inorg.Chim.Acta* ,**256,** 319

Formula: $C_{21} H_{16} N_2 Ni_1 O_2$,1.5($C_1 H_1 Cl_3$)

Compound Name: (N,N'-4-Methyl-o-phenylene-bis(salicylideneiminato))-nickel chloroform solvate

Space Group:	P-1	***Cell:***	***a*** 13.855(3)		***b*** 14.591(3)		***c*** 15.247(4)
Space Group No.:	2	***(Å,°)***	α 116.24(2)		β 115.97(2)		γ 91.30(2)

R-Factor (%): 6.41 ***Temperature(K):*** 295 ***Density(g/cm³):*** 1.570

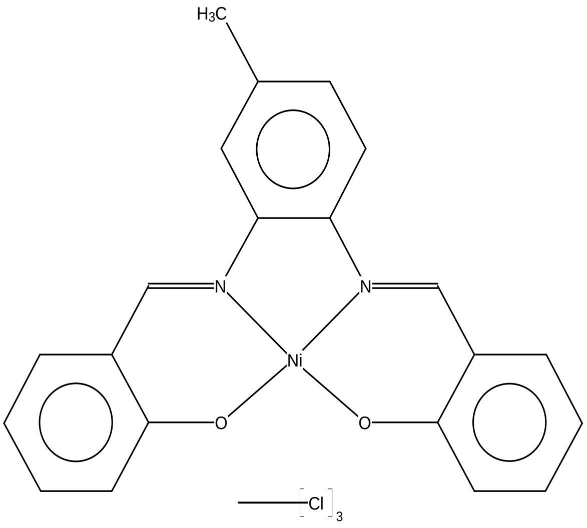

PULJEL

Reference: Liling He, S.R.Wagner, M.L.Illingsworth, A.J.Jensen, G.P.Yap, A.L.Rheingold (1997) *Chem.Mater.* ,**9,**3005

Formula: $C_{40} H_{29} N_5 O_4 Zr_1$,$C_1 H_2 Cl_2$

Compound Name: (4-Amino-N,N'-disalicylidene-1,2-phenylenediaminato)-(N,N'-disalicylidene-1,2-phenylenediaminato)-zirconium(iv) dichloromethane solvate

Space Group:	P21/n	***Cell:***	***a*** 12.227(2)		***b*** 11.934(2)		***c*** 25.646(9)
Space Group No.:	14	***(Å,°)***	α 90.00		β 90.12(4)		γ 90.00

R-Factor (%): 6.53 ***Temperature(K):*** 295 ***Density(g/cm³):*** 1.455

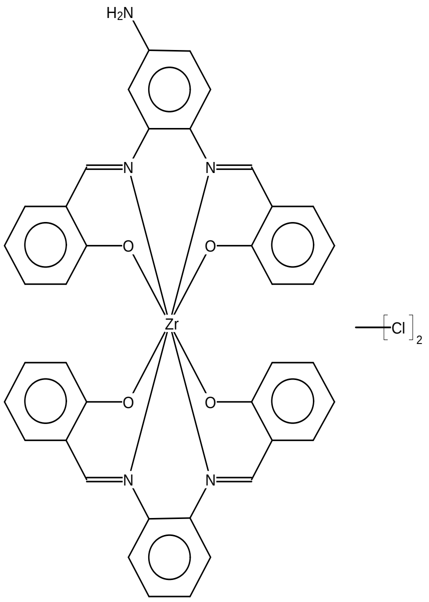

PUSPIC

Reference: Siang-Guan Teoh, Guan-Yeow Yeap, Ching-Ching Loh, Lai-Wan Foong, Soon-Beng Teo, Hoong-Kun Fun (1997) *Polyhedron* , **16,**2213

Formula: $C_{40} H_{28} N_2 O_2 Sn_1$

Compound Name: Diphenyl-(N,N'-bis(2-hydroxy-1-naphthaldehyde)-1,2-phenylenediiminato)-tin(iv)

Space Group:	I41cd	***Cell:***	***a*** 33.051(3)		***b*** 33.051(3)		***c*** 12.305(3)
Space Group No.:	110	***(Å,°)***	α 90.00		β 90.00		γ 90.00

R-Factor (%): 5.79 ***Temperature(K):*** 295 ***Density(g/cm³):*** 1.359

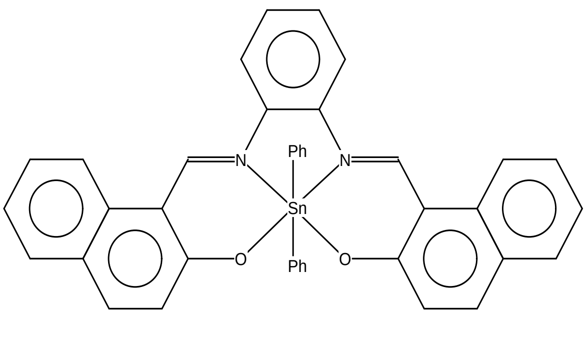

PUSWIK

Reference: S.Akine, F.Utsuno, T.Nabeshima (2010) *Chem.Commun.* , **46,**1029

Formula: $C_{40} H_{24} N_4 Ni_2 O_6$,4($C_2 H_6 O_1 S_1$)

Compound Name: (μ_2-2,23-Dioxa-9,16,30,37-tetraazaheptacyclo[37.3.1.$1^{3,7}$.$1^{18,22}$.$1^{24,28}$.$0^{10,15}$.$0^{31,36}$]hexatetraconta-1(43),3(46),4,6,8,10,12,14,16,18(45),19,21,24(44),25,27,29,31,33,35,37,39,41-docosaene-43,44,45,46-tetrolato)-di-nickel dimethyl sulfoxide solvate

Space Group:	P-1	***Cell:***	***a*** 11.244(0)		***b*** 11.738(0)		***c*** 11.749(0)
Space Group No.:	2	***(Å,°)***	α 63.32(0)		β 66.06(0)		γ 60.30(0)

R-Factor (%): 4.75 ***Temperature(K):*** 120 ***Density(g/cm³):*** 1.543

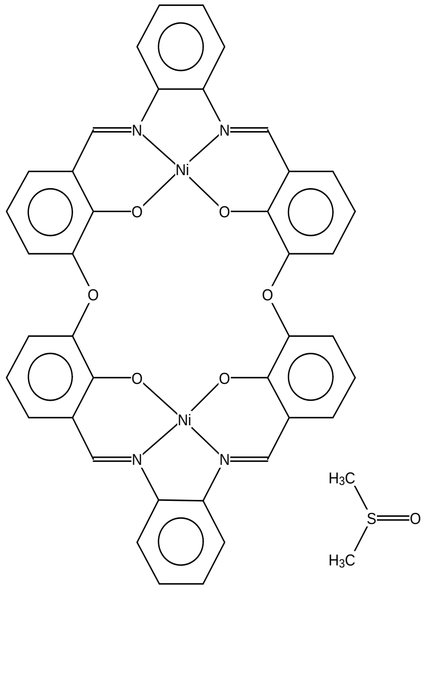

Search: search26 (Tue Dec 2 15:48:50 2014): Hits 481-484

PYSALP

Reference: G.D.Fallon, B.M.Gatehouse (1976) *Acta Crystallogr.,Sect.B:Struct.Crystallogr.Cryst.Chem.* ,**32,**2591

Formula: $C_{20}\ H_{14}\ N_2\ O_2\ Pd_1$

Compound Name: (N,N'-o-Phenylenebis(salicylaldiminato))-palladium(ii)

Space Group:	P212121	***Cell:***	***a*** 16.786(16)	***b*** 17.677(18)	***c*** 5.336(6)		
Space Group No.:	19	***(Å,°)***	α 90.00	β 90.00	γ 90.00		
R-Factor (%):	7.80	***Temperature(K):***	295	***Density(g/cm^3):***	1.765		

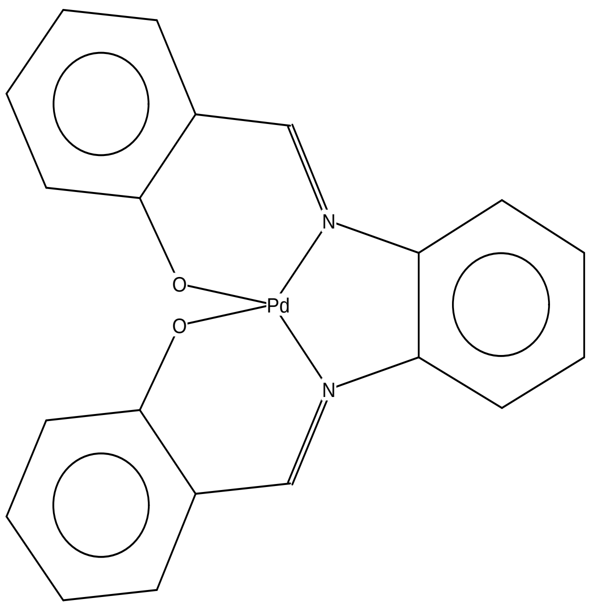

QAJGUF

Reference: A.Sahraei, H.Kargar, R.Kia, M.N.Tahir (2011) *Acta Crystallogr.,Sect.E:Struct.Rep.Online* ,**67,**m82

Formula: $C_{24}\ H_{22}\ N_2\ Ni_1\ O_4$

Compound Name: (5,5'-Dimethoxy-2,2'-[4,5-dimethyl-o-phenylenebis(nitrilomethylidyne)]diphenolato)nickel(II)

Synonym: (2,2'-((4,5-Dimethyl-1,2-phenylene)-bis((nitrilo)methylylidene))-bis(5-methoxyphenolato))-nickel

Space Group:	P21/c	***Cell:***	***a*** 11.324(1)	***b*** 16.553(1)	***c*** 12.162(1)		
Space Group No.:	14	***(Å,°)***	α 90.00	β 113.26(0)	γ 90.00		
R-Factor (%):	4.77	***Temperature(K):***	296	***Density(g/cm^3):***	1.462		

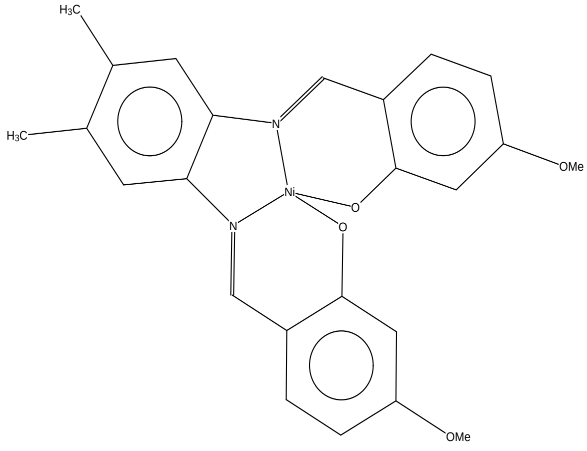

QAVZAP

Reference: D.J.Darensbourg, R.M.Mackiewicz, J.D.Rodgers (2005) *J.Am.Chem.Soc.* ,**127,**14026

Formula: $C_{54}\ H_{79}\ Cl_1\ Cr_1\ N_2\ O_3\ P_1,C_4\ H_{10}\ O_1$

Compound Name: chloro-(N,N'-bis(3,5-di-t-butylsalicylidenato)-1,2-cyclohexanediamine)-(tricyclohexylphosphine oxide)-chromium(iii) diethyl ether solvate

Space Group:	P21/n	***Cell:***	***a*** 10.636(1)	***b*** 39.908(6)	***c*** 13.825(2)		
Space Group No.:	14	***(Å,°)***	α 90.00	β 94.51(0)	γ 90.00		
R-Factor (%):	9.98	***Temperature(K):***	293	***Density(g/cm^3):***	1.132		

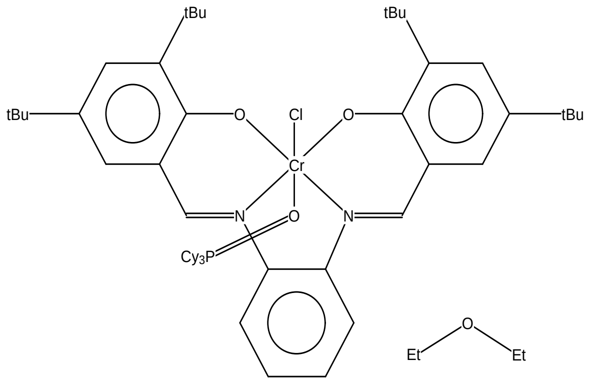

QAYYEW

Reference: M.Mariappan, M.Suenaga, A.Mukhopadhyay, B.G.Maiya (2012) *Inorg.Chim.Acta* ,**390,**95

Formula: $C_{33}\ H_{21}\ N_3\ Ni_1\ O_2,C_3\ H_7\ N_1\ O_1$

Compound Name: (2,2'-((4-(Acridin-9-yl)-1,2-phenylene)bis((nitrilo)methylylidene)) diphenolato)-nickel N,N-dimethylformamide solvate

Space Group:	P21/c	***Cell:***	***a*** 17.947(1)	***b*** 13.439(1)	***c*** 12.867(1)		
Space Group No.:	14	***(Å,°)***	α 90.00	β 108.17(0)	γ 90.00		
R-Factor (%):	5.25	***Temperature(K):***	298	***Density(g/cm^3):***	1.404		

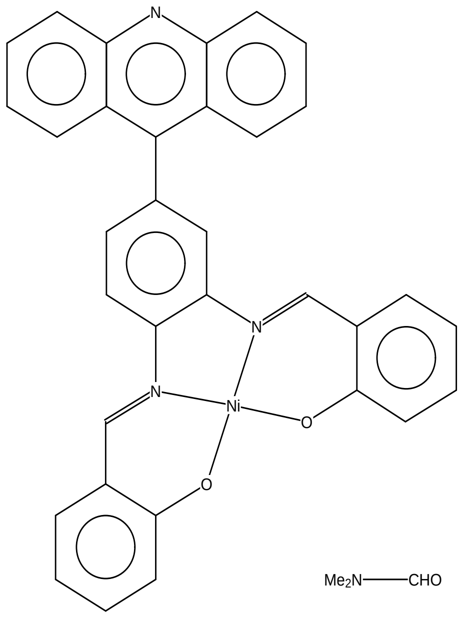

Search: search26 (Tue Dec 2 15:48:50 2014): Hits 485-488

QEJTOO

Reference: Tsz-Wing Wong, Tai-Chu Lau, Wing-Tak Wong (1999)
Inorg.Chem. ,**38,**6181

Formula: $C_{21}\ H_{16}\ Cl_2\ N_3\ O_3\ Os_1\ ^{1+},Cl_1\ O_4\ ^{1-}$

Compound Name: Nitrido-(N,N'-bis(5-chlorosalicylidene)-o-phenylenediamine)-methanol-osmium(vi) perchlorate

Space Group:	P21/c	***Cell:***	***a*** 10.738(2)	***b*** 11.019(2)	***c*** 19.645(2)
Space Group No.:	14	***(Å,°)***	α 90.00	β 99.86(1)	γ 90.00

R-Factor (%): 4.60 ***Temperature(K):*** 295 ***Density(g/cm^3):*** 2.085

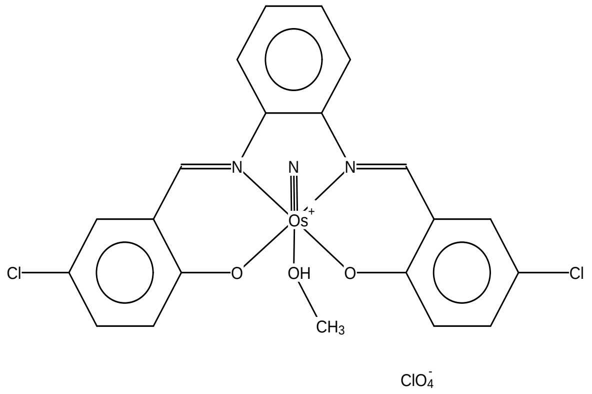

QEJTUU

Reference: Tsz-Wing Wong, Tai-Chu Lau, Wing-Tak Wong (1999)
Inorg.Chem. ,**38,**6181

Formula: $C_{21}\ H_{18}\ N_3\ O_3\ Os_1\ ^{1+},Cl_1\ O_4\ ^{1-}$

Compound Name: Nitrido-(N,N'-bis(salicylidene)-o-phenylenediamine)-methanol-osmium(vi) perchlorate

Space Group:	P21/n	***Cell:***	***a*** 11.552(1)	***b*** 14.856(1)	***c*** 13.554(2)
Space Group No.:	14	***(Å,°)***	α 90.00	β 110.91(2)	γ 90.00

R-Factor (%): 4.60 ***Temperature(K):*** 295 ***Density(g/cm^3):*** 1.987

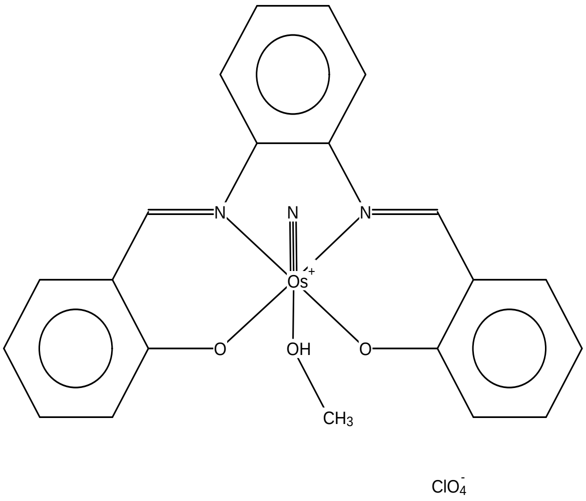

QEJVAC

Reference: Tsz-Wing Wong, Tai-Chu Lau, Wing-Tak Wong (1999)
Inorg.Chem. ,**38,**6181

Formula: $C_{38}\ H_{29}\ Cl_1\ N_3\ O_2\ Os_1\ P_1,H_2\ O_1$

Compound Name: Chloro-(N,N'-bis(salicylidene)-o-phenylenediamine)-triphenylphosphoraniminato-osmium(vi) monohydrate

Space Group:	P21/n	***Cell:***	***a*** 14.722(1)	***b*** 14.241(2)	***c*** 16.038(2)
Space Group No.:	14	***(Å,°)***	α 90.00	β 98.39(2)	γ 90.00

R-Factor (%): 6.70 ***Temperature(K):*** 295 ***Density(g/cm^3):*** 1.666

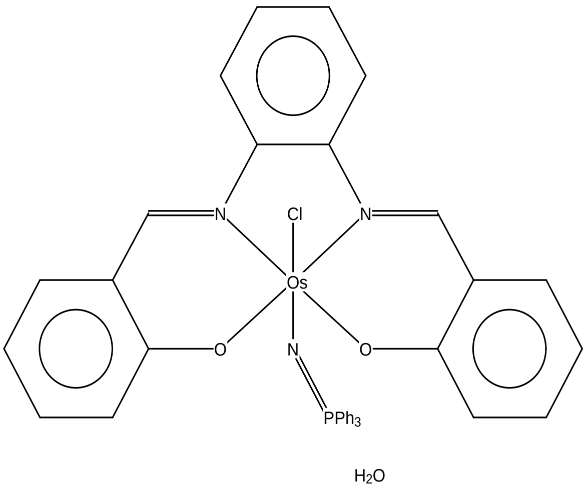

QIXJEN

Reference: N.E.Eltayeb, S.G.Teoh, S.Chantrapromma,
Hoong-Kun Fun, R.Adnan (2008)
Acta Crystallogr.,Sect.E:Struct.Rep.Online ,**64,**m570

Formula: $C_{22}\ H_{18}\ Cl_1\ Mn_1\ N_2\ O_2$

Compound Name: Chlorido-(5,5'-dimethyl-2,2'-(1,2-phenylenebis(nitrilomethylidyne)) diphenolato-κ^{4}O,N,N',O')-manganese(iii)

Space Group:	C2/c	***Cell:***	***a*** 20.959(0)	***b*** 13.590(0)	***c*** 14.932(0)
Space Group No.:	15	***(Å,°)***	α 90.00	β 119.64(0)	γ 90.00

R-Factor (%): 4.46 ***Temperature(K):*** 100 ***Density(g/cm^3):*** 1.555

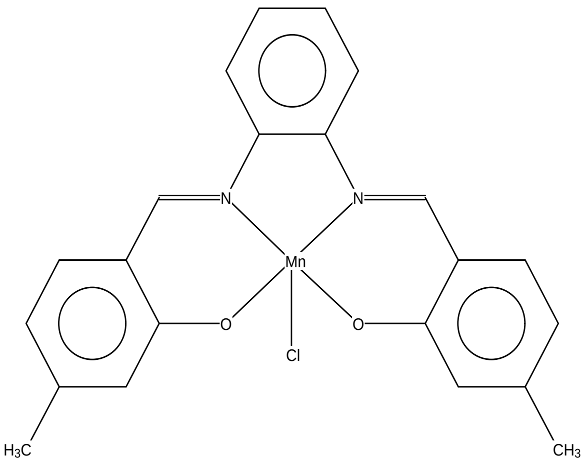

QONKIO

Reference: M.Kuil, I.M.Puijk, A.W.Kleij, D.M.Tooke, A.L.Spek, J.N.H.Reek (2009) *Chem.Asian J.* ,**4,**50

Formula: $(C_{70} H_{90} N_6 O_4 Zn_2)n,n(C_1 H_2 Cl_2)$

Compound Name: catena-((μ_2-Benzene-1,2,4,5-tetrayltetrakis(3,5-di-t-butylsalicylideneiminato))-(μ_2-pyrazine)-di-zinc(ii) dichloromethane solvate)

Space Group: P-1
Space Group No.: 2

Cell: ***(Å,°)*** ***a*** 11.314(2) ***b*** 11.393(3) ***c*** 14.964(3) α 103.94(1) β 94.95(1) γ 106.66(1)

R-Factor (%): 4.67 ***Temperature(K):*** 150 ***Density(g/cm³):*** 1.216

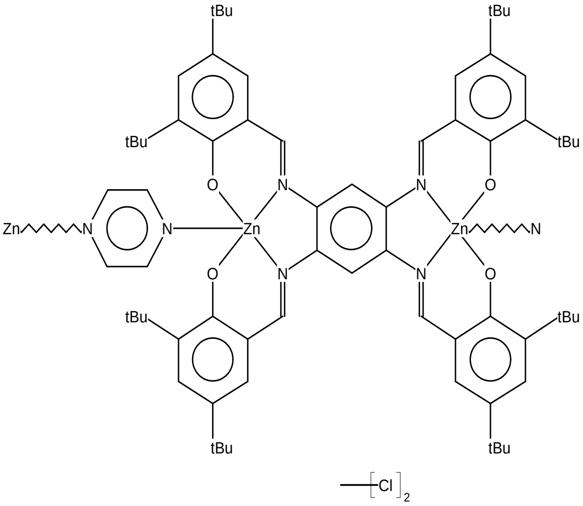

QONKOU

Reference: M.Kuil, I.M.Puijk, A.W.Kleij, D.M.Tooke, A.L.Spek, J.N.H.Reek (2009) *Chem.Asian J.* ,**4,**50

Formula: $(C_{160} H_{204} N_{12} O_8 Zn_4)n,12n(C_1 H_2 Cl_2)$

Compound Name: catena-(bis(μ_2-Benzene-1,2,4,5-tetrayltetrakis(3,5-di-t-butylsalicylideneiminato))-bis(μ_2-1,2-diphenylethylenediamine)-tetra-zinc(ii) dichloromethane solvate)

Space Group: P21
Space Group No.: 4

Cell: ***(Å,°)*** ***a*** 13.835(0) ***b*** 39.310(2) ***c*** 15.352(0) α 90.00 β 93.33(0) γ 90.00

R-Factor (%): 6.09 ***Temperature(K):*** 150 ***Density(g/cm³):*** 1.476

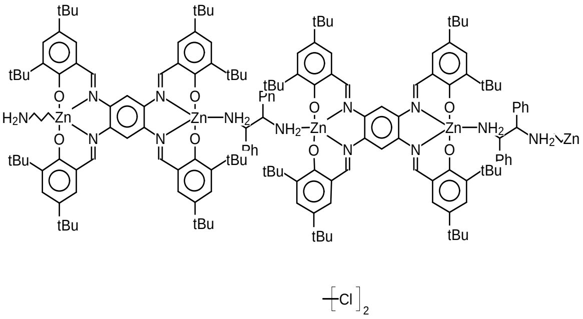

QOTLAN

Reference: H.Kargar, R.Kia, A.Jamshidvand, H.-K.Fun (2009) *Acta Crystallogr.,Sect.E:Struct.Rep.Online* ,**65,**m498

Formula: $C_{26} H_{26} N_2 Ni_1 O_4,2(H_2 O_1)$

Compound Name: (6,6'-Diethoxy-2,2'-(4,5-dimethyl-o-phenylenebis(nitrilomethylidyne)) diphenolato)-nickel(ii) dihydrate

Space Group: Pbcn
Space Group No.: 60

Cell: ***(Å,°)*** ***a*** 12.871(0) ***b*** 16.113(0) ***c*** 11.855(0) α 90.00 β 90.00 γ 90.00

R-Factor (%): 4.61 ***Temperature(K):*** 294 ***Density(g/cm³):*** 1.419

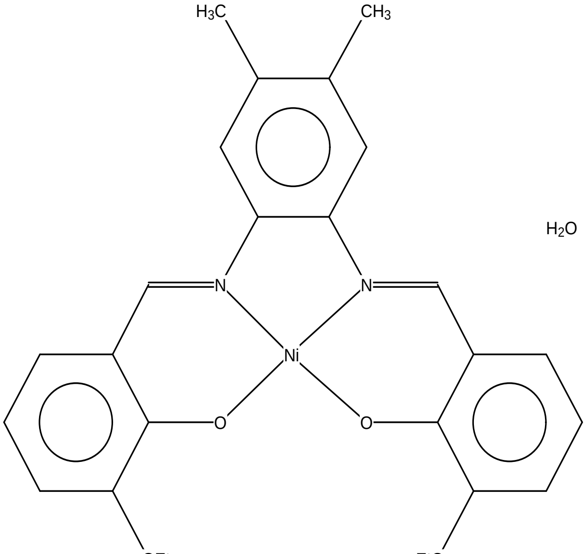

QOXRAX

Reference: E.C.Escudero-Adan, J.Benet-Buchholz, A.W.Kleij (2009) *Chem.-Eur.J.* ,**15,**4233

Formula: $C_{36} H_{46} N_2 O_2 Zn_1,C_7 H_9 N_1,C_{37} H_{50} N_2 O_3 Zn_1$

Compound Name: (N,N'-1,2-Phenylene-bis(3,5-di-t-butylsalicylaldiminato))-zinc (N,N'-1,2-phenylene-bis(3,5-di-t-butylsalicylaldiminato))-(methanol)-zinc 2,6-dimethylpyridine

Space Group: P21/c
Space Group No.: 14

Cell: ***(Å,°)*** ***a*** 13.122(0) ***b*** 24.207(1) ***c*** 11.614(0) α 90.00 β 101.92(0) γ 90.00

R-Factor (%): 5.03 ***Temperature(K):*** 100 ***Density(g/cm³):*** 1.240

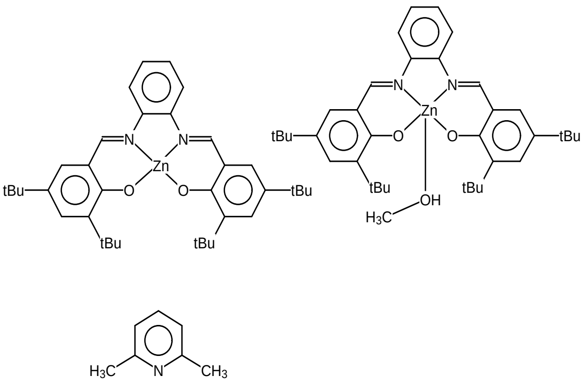

Search: search26 (Tue Dec 2 15:48:50 2014): Hits 493-496

QOXREB

Reference: E.C.Escudero-Adan, J.Benet-Buchholz, A.W.Kleij (2009)
Chem.-Eur.J. ,**15,**4233

Formula: $C_{37} H_{50} N_2 O_3 Zn_1, C_2 H_3 N_1$

Compound Name: (N,N'-1,2-Phenylene-bis(3,5-di-t-butylsalicylaldiminato))-(methanol)-zinc acetonitrile solvate

Space Group:	P21/c	***Cell:***	***a*** 13.172(0)	***b*** 24.553(0)	***c*** 11.519(0)		
Space Group No.:	14	***(Å,°)***	α 90.00	β 103.80(0)	γ 90.00		
R-Factor (%):	5.34	***Temperature(K):***	100	***Density(g/cm³):***	1.243		

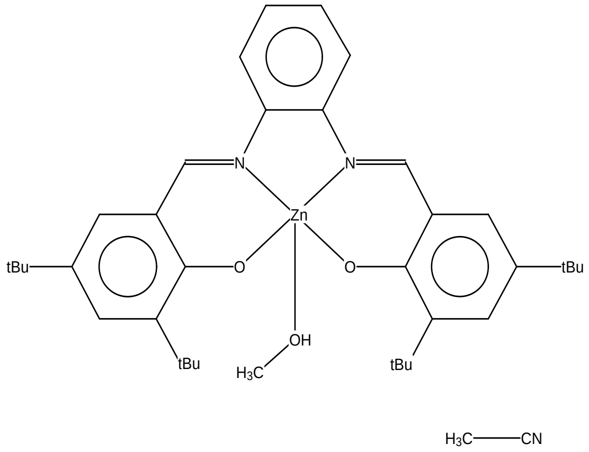

QOXRIF

Reference: E.C.Escudero-Adan, J.Benet-Buchholz, A.W.Kleij (2009)
Chem.-Eur.J. ,**15,**4233

Formula: $C_{36} H_{46} N_2 O_2 Zn_1, C_{37} H_{50} N_2 O_3 Zn_1, C_{10} H_9 N_1$

Compound Name: (N,N'-1,2-Phenylene-bis(3,5-di-t-butylsalicylaldiminato))-zinc (N,N'-1,2-phenylene-bis(3,5-di-t-butylsalicylaldiminato))-(methanol)-zinc 2-methylquinoline

Space Group:	P21/c	***Cell:***	***a*** 13.226(0)	***b*** 24.530(0)	***c*** 11.530(0)		
Space Group No.:	14	***(Å,°)***	α 90.00	β 104.13(0)	γ 90.00		
R-Factor (%):	7.60	***Temperature(K):***	100	***Density(g/cm³):***	1.267		

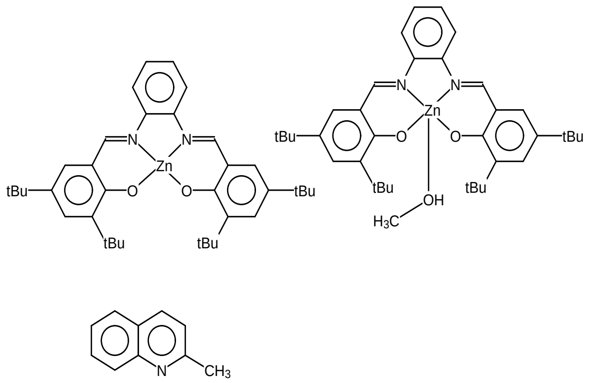

QOXROL

Reference: E.C.Escudero-Adan, J.Benet-Buchholz, A.W.Kleij (2009)
Chem.-Eur.J. ,**15,**4233

Formula: $C_{36} H_{46} N_2 O_2 Zn_1, C_{37} H_{50} N_2 O_3 Zn_1, C_{13} H_9 N_1$

Compound Name: (N,N'-1,2-Phenylene-bis(3,5-di-t-butylsalicylaldiminato))-zinc (N,N'-1,2-phenylene-bis(3,5-di-t-butylsalicylaldiminato))-(methanol)-zinc acridine

Space Group:	P21212	***Cell:***	***a*** 24.308(1)	***b*** 26.131(1)	***c*** 11.678(0)		
Space Group No.:	18	***(Å,°)***	α 90.00	β 90.00	γ 90.00		
R-Factor (%):	3.46	***Temperature(K):***	100	***Density(g/cm³):***	1.271		

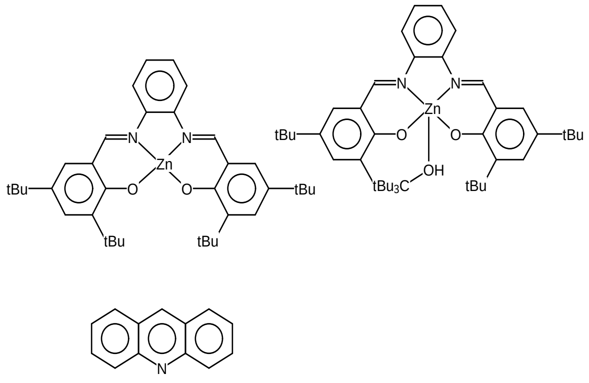

QUBJAY

Reference: Ying-Zhong Shen, Yi Pan, Le-Yong Wang, Gang Dong, Xiao-Ping Jin, Xiao-Ying Huang, Hongwen Hu (1999)
J.Organomet.Chem. ,**590,**242

Formula: $C_{21} H_{17} In_1 N_2 O_2$

Compound Name: Methyl-(N,N'-bis(salicylidene)-1,2-phenylenediamino)-indium

Space Group:	P212121	***Cell:***	***a*** 9.718(2)	***b*** 10.572(3)	***c*** 17.604(3)		
Space Group No.:	19	***(Å,°)***	α 90.00	β 90.00	γ 90.00		
R-Factor (%):	3.80	***Temperature(K):***	295	***Density(g/cm³):***	1.631		

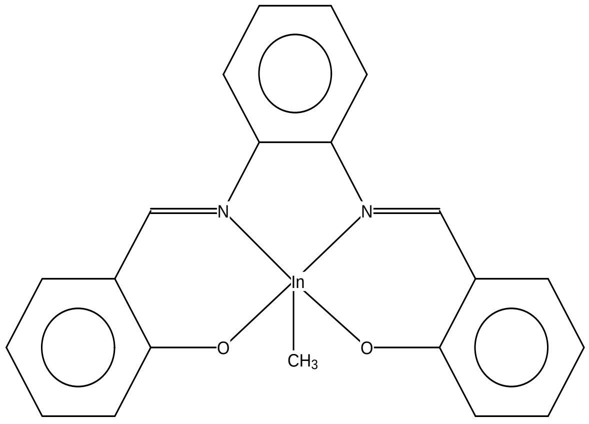

Search: search26 (Tue Dec 2 15:48:50 2014): Hits 497-500

QUJGEI

Reference:	M.Fleck, S.Hazra, S.Majumder, S.Mohanta (2008) *Cryst.Res.Technol.* ,**43,**1220
Formula:	$C_{24}\ H_{24}\ N_2\ O_7\ U_1,0.5(H_2\ O_1)$
Compound Name:	Aqua-dioxo-(2,2'-(1,2-phenylenebis((nitrilo)methylylidene))bis(6-ethoxyphenolato)-N,N',O,O')-uranium(vi) hemihydrate

Space Group:	P-1	***Cell:***	***a*** 11.865(2)	***b*** 13.043(3)	***c*** 16.781(3)		
Space Group No.:	2	***(Å,°)***	α 109.72(3)	β 92.08(3)	γ 94.08(3)		
R-Factor (%):	5.84	***Temperature(K):***	293	***Density(g/cm³):***	1.909		

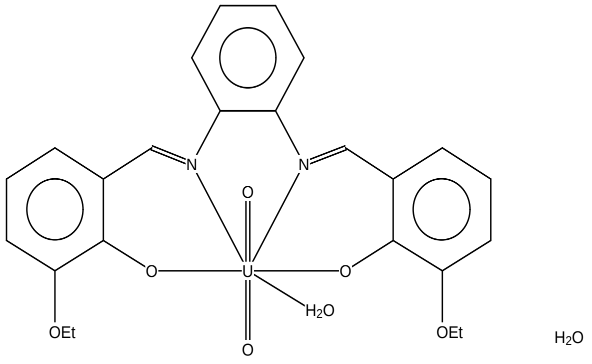

RADFEJ

Reference:	M.M.Belmonte, S.J.Wezenberg, R.M.Haak, D.Anselmo, E.C.Escudero-Adan, J.Benet-Buchholz, A.W.Kleij (2010) *Dalton Trans.* ,**39,**4541
Formula:	$C_{56}\ H_{60}\ N_4\ O_4\ Zn_2,C_2\ H_3\ N_1$
Compound Name:	bis(μ_2-N,N'-bis(3-t-butylsalicylidene)phenylene-1,2-diamine)-di-zinc acetonitrile solvate

Space Group:	P-1	***Cell:***	***a*** 11.518(0)	***b*** 13.598(0)	***c*** 16.090(1)
Space Group No.:	2	***(Å,°)***	α 94.28(0)	β 91.42(0)	γ 97.66(0)
R-Factor (%):	4.00	***Temperature(K):***	100	***Density(g/cm³):***	1.368

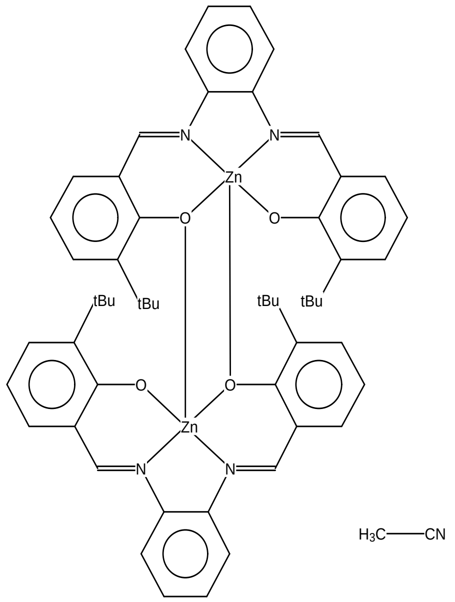

RADFIN

Reference:	M.M.Belmonte, S.J.Wezenberg, R.M.Haak, D.Anselmo, E.C.Escudero-Adan, J.Benet-Buchholz, A.W.Kleij (2010) *Dalton Trans.* ,**39,**4541
Formula:	$C_{52}\ H_{44}\ N_4\ O_4\ Zn_2$
Compound Name:	bis(μ_2-N,N'-bis(3-allylsalicylidene)phenylene-1,2-diamine)-di-zinc

Space Group:	Pca21	***Cell:***	***a*** 14.664(0)	***b*** 12.665(0)	***c*** 22.417(0)
Space Group No.:	29	***(Å,°)***	α 90.00	β 90.00	γ 90.00
R-Factor (%):	3.22	***Temperature(K):***	100	***Density(g/cm³):***	1.467

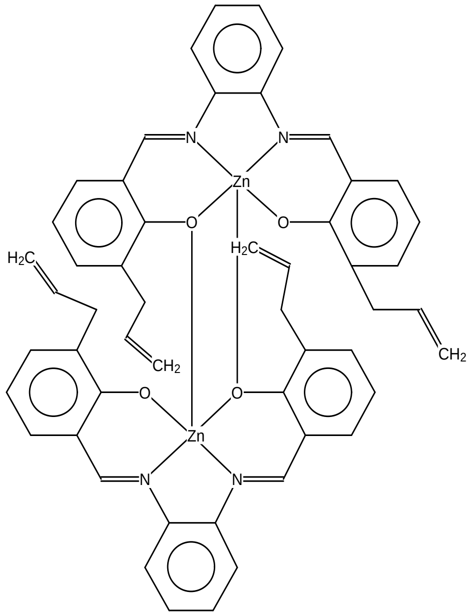

RADFOT

Reference:	M.M.Belmonte, S.J.Wezenberg, R.M.Haak, D.Anselmo, E.C.Escudero-Adan, J.Benet-Buchholz, A.W.Kleij (2010) *Dalton Trans.* ,**39,**4541
Formula:	$C_{56}\ H_{60}\ N_4\ O_4\ Zn_2,2(C_2\ H_3\ N_1)$
Compound Name:	bis(μ_2-N-(3,5-di-t-butylsalicylidene)-N'-(salicylidene)phenylene-1,2-diamine)-di-zinc acetonitrile solvate

Space Group:	P-1	***Cell:***	***a*** 10.215(1)	***b*** 12.272(0)	***c*** 12.755(0)
Space Group No.:	2	***(Å,°)***	α 114.88(0)	β 97.48(0)	γ 105.22(0)
R-Factor (%):	2.67	***Temperature(K):***	100	***Density(g/cm³):***	1.315

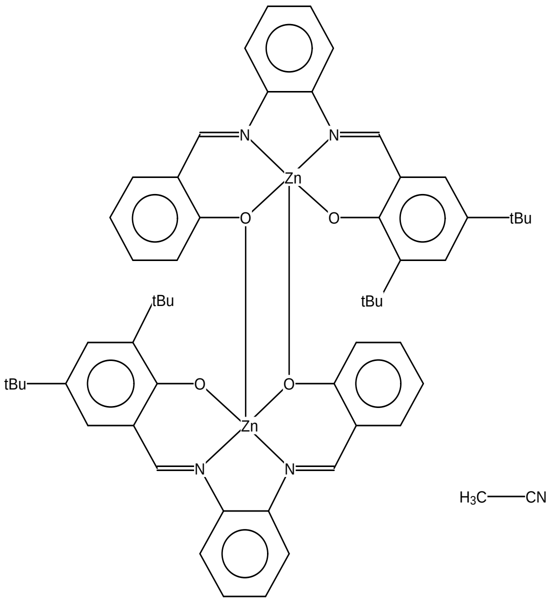

Search: search26 (Tue Dec 2 15:48:50 2014): Hits 501-504

RADFUZ

Reference: M.M.Belmonte, S.J.Wezenberg, R.M.Haak, D.Anselmo, E.C.Escudero-Adan, J.Benet-Buchholz, A.W.Kleij (2010) *Dalton Trans.* ,**39,**4541

Formula: C_{64} H_{64} N_4 O_4 Zn_2,2(C_2 H_3 N_1)

Compound Name: bis(μ_2-N-(3,5-di-t-butylsalicylidene)-N'-(2-oxynaphthyl-1-methylene) phenylene-1,2-diamine)-di-zinc acetonitrile solvate

Space Group:	P-1	***Cell:***	***a*** 10.030(4)	***b*** 10.664(4)	***c*** 15.255(7)		
Space Group No.:	2	***(Å,°)***	α 79.51(0)	β 75.80(0)	γ 75.14(1)		
R-Factor (%):	5.70	***Temperature(K):***	100	***Density(g/cm³):***	1.277		

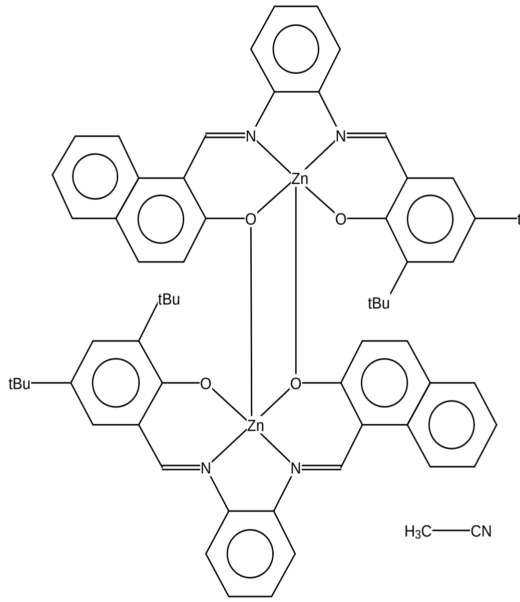

RADGAG

Reference: M.M.Belmonte, S.J.Wezenberg, R.M.Haak, D.Anselmo, E.C.Escudero-Adan, J.Benet-Buchholz, A.W.Kleij (2010) *Dalton Trans.* ,**39,**4541

Formula: C_{56} H_{56} N_8 O_{12} Zn_2,C_7 H_9 N_1

Compound Name: bis(μ_2-N-(3,5-dinitrosalicylidene)-N'-(3,5-di-t-butylsalicylidene)phenylene-1,2-diamine)-di-zinc 2,6-dimethylpyridine solvate

Space Group:	C2/c	***Cell:***	***a*** 9.902(0)	***b*** 22.125(1)	***c*** 28.000(1)
Space Group No.:	15	***(Å,°)***	α 90.00	β 98.87(0)	γ 90.00
R-Factor (%):	4.76	***Temperature(K):***	100	***Density(g/cm³):***	1.393

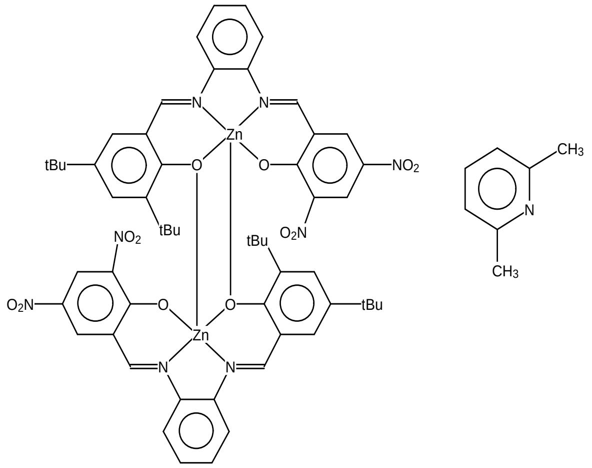

RADGEK

Reference: M.M.Belmonte, S.J.Wezenberg, R.M.Haak, D.Anselmo, E.C.Escudero-Adan, J.Benet-Buchholz, A.W.Kleij (2010) *Dalton Trans.* ,**39,**4541

Formula: C_{62} H_{68} N_4 O_4 Zn_2,3(C_2 H_3 N_1)

Compound Name: bis(μ_2-N-(3-allylsalicylidene)-N-(3,5-di-t-butylsalicylidene)phenylene-1,2-diamine)-di-zinc acetonitrile solvate

Space Group:	P21/n	***Cell:***	***a*** 16.310(0)	***b*** 13.011(0)	***c*** 29.462(1)
Space Group No.:	14	***(Å,°)***	α 90.00	β 102.98(0)	γ 90.00
R-Factor (%):	8.28	***Temperature(K):***	100	***Density(g/cm³):***	1.294

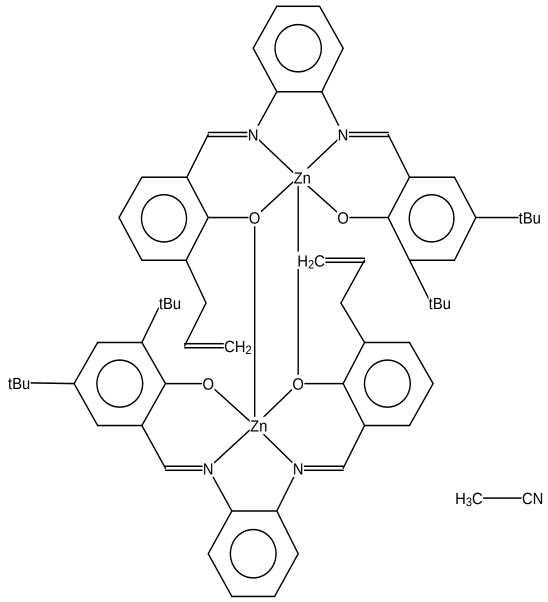

RADGIO

Reference: M.M.Belmonte, S.J.Wezenberg, R.M.Haak, D.Anselmo, E.C.Escudero-Adan, J.Benet-Buchholz, A.W.Kleij (2010) *Dalton Trans.* ,**39,**4541

Formula: C_{64} H_{76} N_4 O_4 Zn_2,3(C_2 H_3 N_1)

Compound Name: bis(μ_2-N-(3,5-di-t-butylsalicylidene)-N'-(5-t-butylsalicylidene)phenylene-1,2-diamine)-di-zinc acetonitrile solvate

Space Group:	P-1	***Cell:***	***a*** 13.432(0)	***b*** 15.373(0)	***c*** 17.826(0)
Space Group No.:	2	***(Å,°)***	α 108.67(0)	β 91.98(0)	γ 101.82(0)
R-Factor (%):	4.42	***Temperature(K):***	100	***Density(g/cm³):***	1.193

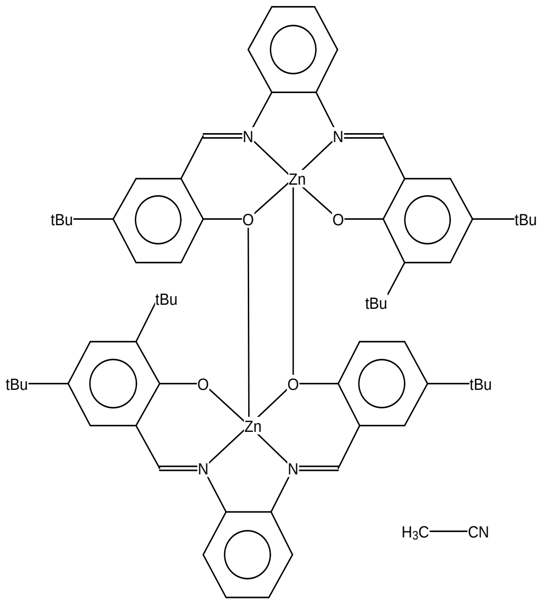

RATXAM

Reference: A.W.Kleij, M.Kuil, D.M.Tooke, M.Lutz, A.L.Spek, J.N.H.Reek (2005) *Chem.-Eur.J.* ,**11,**4743

Formula: $C_{156} H_{196} N_{12} O_8 Zn_4, C_2 H_3 N_1$

Compound Name: bis(μ_2-Benzene-1,2,4,5-tetrayltetrakis(3,5-di-t-butylsalicylideneiminato))-bis(μ_2-1,2-bis(4-pyridyl)ethane)-tetra-zinc(ii) acetonitrile solvate

Space Group:	P21/c	***Cell:***	***a*** 17.760(0)	***b*** 12.825(0)	***c*** 41.889(2)		
Space Group No.:	14	***(Å,°)***	α 90.00	β 101.18(0)	γ 90.00		
R-Factor (%):	5.19	***Temperature(K):***	150	***Density(g/cm³):***	0.947		

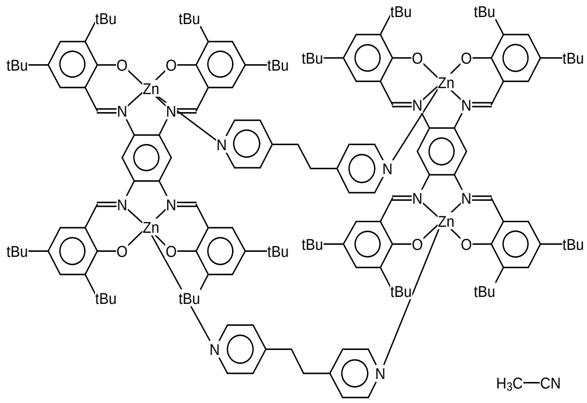

RATXEQ

Reference: A.W.Kleij, M.Kuil, D.M.Tooke, M.Lutz, A.L.Spek, J.N.H.Reek (2005) *Chem.-Eur.J.* ,**11,**4743

Formula: $C_{66} H_{64} Cl_4 N_6 O_4 Zn_2, C_2 H_3 N_1$

Compound Name: bis(μ_2-4,4'-Bipyridyl)-bis(4,5-dichloro-1,2-phenylenebis(3-t-butylsalicylideneiminato))-di-zinc(ii) acetonitrile solvate

Space Group:	C2/c	***Cell:***	***a*** 49.284(0)	***b*** 13.312(0)	***c*** 19.976(0)		
Space Group No.:	15	***(Å,°)***	α 90.00	β 106.79(0)	γ 90.00		
R-Factor (%):	3.57	***Temperature(K):***	150	***Density(g/cm³):***	1.396		

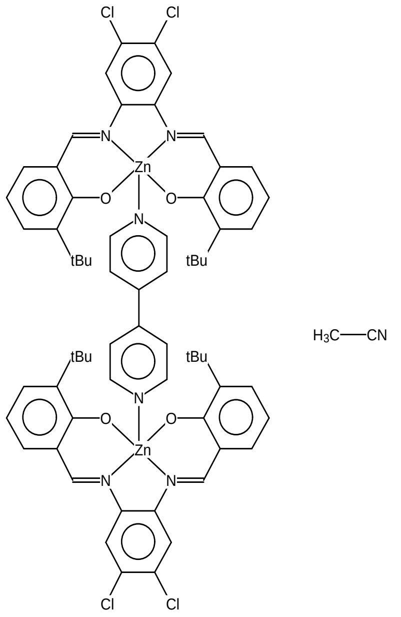

RATXIU

Reference: A.W.Kleij, M.Kuil, D.M.Tooke, M.Lutz, A.L.Spek, J.N.H.Reek (2005) *Chem.-Eur.J.* ,**11,**4743

Formula: $C_{152} H_{188} N_{12} O_8 Zn_4, 3(C_2 H_3 N_1)$

Compound Name: bis(μ_2-Benzene-1,2,4,5-tetrayltetrakis(3,5-di-t-butylsalicylideneiminato))-bis(μ_2-4,4'-bipyridyl)-tetra-zinc(ii) acetonitrile solvate

Space Group:	P21/c	***Cell:***	***a*** 17.599(0)	***b*** 13.076(0)	***c*** 40.238(0)		
Space Group No.:	14	***(Å,°)***	α 90.00	β 97.43(0)	γ 90.00		
R-Factor (%):	5.19	***Temperature(K):***	150	***Density(g/cm³):***	0.975		

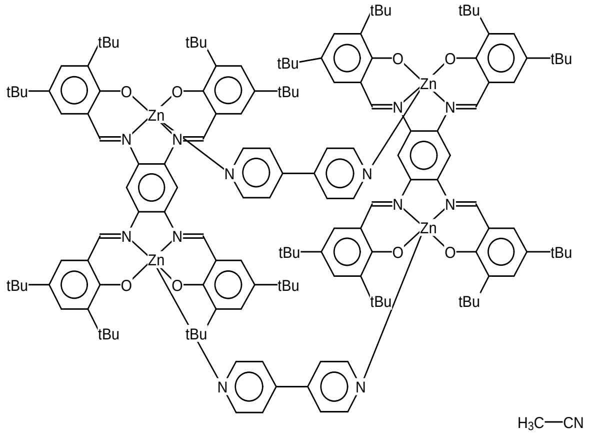

RAVKEE

Reference: Y.Elerman, M.Kabak, D.Ulku (1997) *Acta Crystallogr.,Sect.C:Cryst.Struct.Commun.* ,**53,**712

Formula: $C_{28} H_{18} Cl_1 Fe_1 N_2 O_2, C_4 H_8 O_2$

Compound Name: Chloro-(N,N'-bis(2-hydroxy-1-naphthylidene)-1,2-diaminobenzene)-iron(iii) 1,4-dioxane solvate

Space Group:	P-1	***Cell:***	***a*** 9.322(0)	***b*** 9.584(0)	***c*** 15.538(0)		
Space Group No.:	2	***(Å,°)***	α 94.43(1)	β 101.18(1)	γ 99.73(1)		
R-Factor (%):	6.70	***Temperature(K):***	295	***Density(g/cm³):***	1.479		

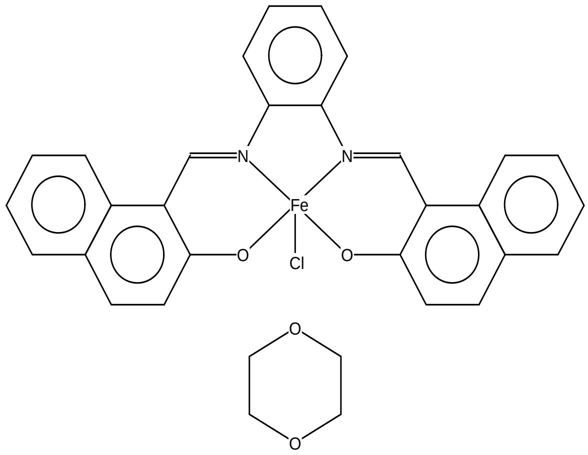

Search: search26 (Tue Dec 2 15:48:50 2014): Hits 509-512

REYPIV

Reference: O.Rotthaus, O.Jarjayes, F.Thomas, C.Philouze, E.Saint-Aman, J.-L.Pierre (2007) *Dalton Trans.* ,889

Formula: $C_{38}\ H_{52}\ N_2\ O_3\ Zn_1,C_2\ H_6\ O_1$

Compound Name: (2,4-Di-t-butyl-6-((2-((3,5-di-t-butyl-2-oxybenzylidene)amino)phenylimino) methyl)phenolato)-ethanol-zinc(ii) ethanol solvate

Space Group:	P21/a	***Cell:***	***a***	11.882(0)	***b***	25.396(2)	***c***	13.130(1)
Space Group No.:	14	***(Å,°)***	α	90.00	β	92.20(30)	γ	90.00

R-Factor (%): 6.24 ***Temperature(K):*** 293 ***Density(g/cm³):*** 1.168

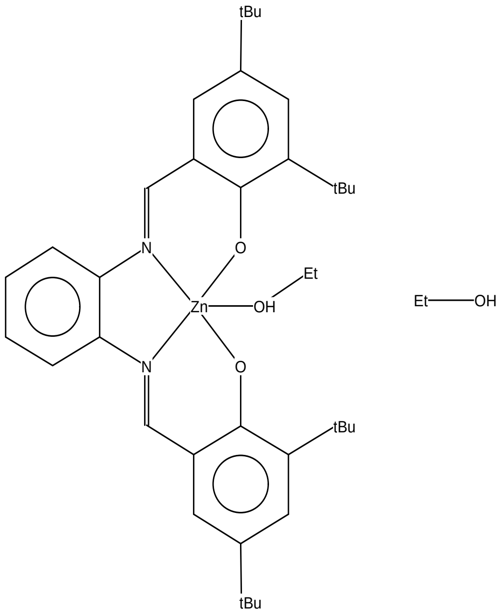

REYPIV01

Reference: N.E.Eltayeb, S.G.Teoh, S.Chantrapromma, H.-K.Fun, K.Ibrahim (2007) *Acta Crystallogr.,Sect.E:Struct.Rep.Online* ,**63,**m2024

Formula: $C_{38}\ H_{52}\ N_2\ O_3\ Zn_1,C_2\ H_6\ O_1$

Compound Name: (Ethanol-O)-(4,4',6,6'-tetra-t-butyl-2,2'-(1,2-phenylenebis(nitrilomethylidyne))diphenolato-N,N',O,O')-zinc(ii) ethanol solvate

Space Group:	P21/c	***Cell:***	***a***	13.091(0)	***b***	24.893(0)	***c***	11.629(0)
Space Group No.:	14	***(Å,°)***	α	90.00	β	92.30(0)	γ	90.00

R-Factor (%): 4.51 ***Temperature(K):*** 100 ***Density(g/cm³):*** 1.221

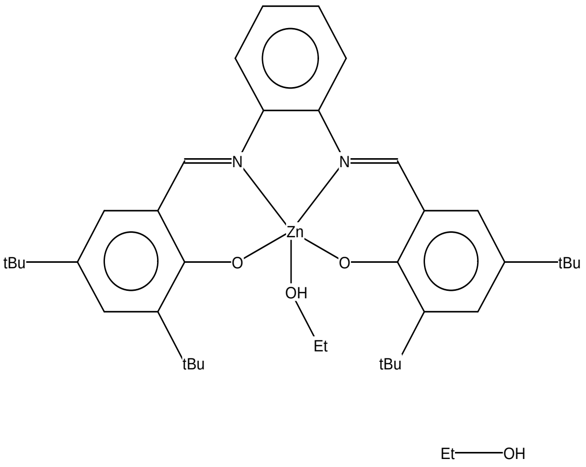

REYPIV02

Reference: M.E.Germain, T.R.Vargo, P.G.Khalifah, M.J.Knapp (2007) *Inorg.Chem.* ,**46,**4422

Formula: $C_{38}\ H_{52}\ N_2\ O_3\ Zn_1,C_2\ H_6\ O_1$

Compound Name: (N,N'-bis(3,5-Di-t-butylsalicylideneimine)benzene)-ethanol-zinc(ii) ethanol solvate

Space Group:	P21/c	***Cell:***	***a***	13.129(0)	***b***	24.929(0)	***c***	11.635(0)
Space Group No.:	14	***(Å,°)***	α	90.00	β	91.48(0)	γ	90.00

R-Factor (%): 4.77 ***Temperature(K):*** 126 ***Density(g/cm³):*** 1.215

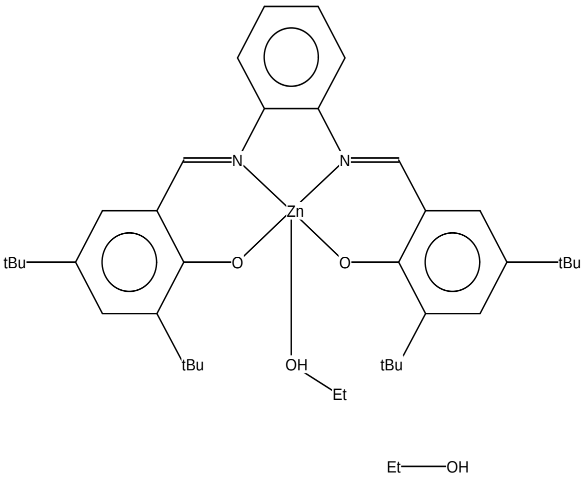

RIPZIA

Reference: O.Rotthaus, O.Jarjayes, C.P.Del Valle, C.Philouze, F.Thomas (2007) *Chem.Commun.* ,4462

Formula: $C_{32}\ H_{42}\ N_4\ Ni_1\ O_2\ ^{2+},2(Cl_1\ ^{1-}),1.81(C_1\ H_1\ Cl_3),H_2\ O_1$

Compound Name: (2,2'-(benzene-1,2-diylbis(nitrilomethylidene))bis(6-t-butyl-4-(dimethylammonio)phenolato))-nickel(ii) dichloride chloroform solvate monohydrate

Space Group:	P21/a	***Cell:***	***a***	10.934(1)	***b***	20.635(2)	***c***	18.842(2)
Space Group No.:	14	***(Å,°)***	α	90.00	β	100.24(2)	γ	90.00

R-Factor (%): 6.19 ***Temperature(K):*** 293 ***Density(g/cm³):*** 1.395

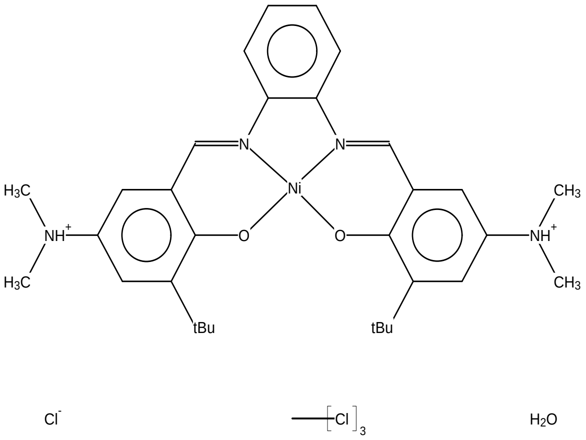

RIPZUM

Reference: P.D.Frischmann, M.J.MacLachlan (2007) *Chem.Commun.* ,4480

Formula: C_{66} H_{72} Cd_7 N_6 O_{28},7(C_3 H_7 N_1 O_1)

Compound Name: (μ_6-4^4,4^5,10^4,10^5,16^4,16^5-Hexaethoxy-1^2,1^3,7^2,7^3,13^2,13^3-hexaoxy-3,5,9,11,15,17-hexa-aza-1,7,13(1,4),4,10,16(1,2)-hexabenzenacyclo-octadecaphane-2,5,8,11,14,17-hexaene)-tris(μ_3-acetato)-(μ_3-oxo)-tris(μ_2-acetato)-triaqua-hepta-cadmium(ii) dimethylformamide solvate

Space Group:	R-3m	***Cell:***	***a*** 28.712(3)	***b*** 28.712(3)	***c*** 26.128(3)
Space Group No.:	166	***(Å,°)***	α 90.00	β 90.00	γ 120.00

R-Factor (%): 6.65 ***Temperature(K):*** 173 ***Density(g/cm^3):*** 1.440

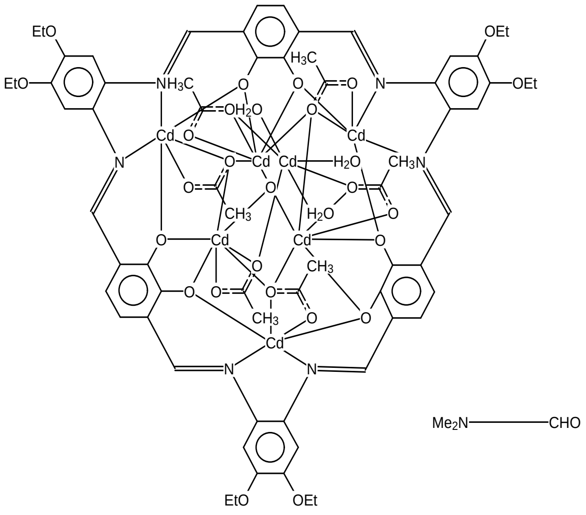

RIPZUM01

Reference: P.D.Frischmann, G.A.Facey, P.Y.Ghi, A.J.Gallant, D.L.Bryce, F.Lelj, M.J.MacLachlan (2010) *J.Am.Chem.Soc.* ,**132,**3893

Formula: C_{66} H_{72} Cd_7 N_6 O_{28},7(C_3 H_7 N_1 O_1)

Compound Name: (μ_6-$1^{4,5}$,$7^{4,5}$,$13^{4,5}$-hexaethoxy-2,6,8,12,14,18-hexaaza-1,7,13(1,2)-tribenzena-4,10,16(1,4)-tribenzenacyclooctadecaphane-2,5,8,11,14,17-hexaene-$4^{2,3}$,$10^{2,3}$,$16^{2,3}$-hexaolato)-tris(μ_3-acetato)-(μ_3-hydroxo)-tris(μ_2-acetato)-hydroxy-diaqua-hepta-cadmium N,N-dimethylformamide solvate

Space Group:	R-3m	***Cell:***	***a*** 28.712(3)	***b*** 28.712(3)	***c*** 26.128(3)
Space Group No.:	166	***(Å,°)***	α 90.00	β 90.00	γ 120.00

R-Factor (%): 6.65 ***Temperature(K):*** 173 ***Density(g/cm^3):*** 1.440

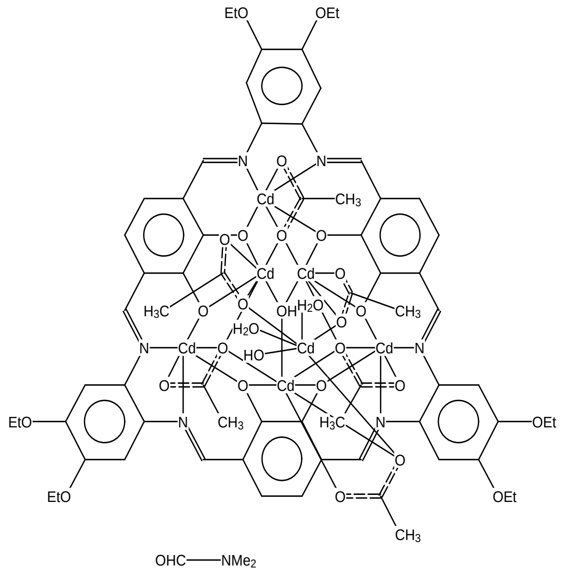

RIVCEF

Reference: Ying-Xia Zhou, Xian-Fu Zheng, Dan Han, Hong-Yun Zhang, Xiao-Qing Shen, Cao-Yuan Niu, Pei-Kun Chen, Hong-Wei Hou, Yu Zhu (2006) *Synth.React.Inorg.,Met.-Org.,Nano-Met.Chem.* ,**36,**693

Formula: C_{20} H_{14} Cl_1 Fe_1 N_2 O_2,C_2 H_3 N_1

Compound Name: chloro-(N,N'-o-phenylenebis(salicylidenealdiminato))-iron(iii) acetonitrile solvate

Space Group:	P-1	***Cell:***	***a*** 7.261(1)	***b*** 11.817(2)	***c*** 12.609(3)
Space Group No.:	2	***(Å,°)***	α 104.75(3)	β 105.80(3)	γ 98.20(3)

R-Factor (%): 4.68 ***Temperature(K):*** 291 ***Density(g/cm^3):*** 1.513

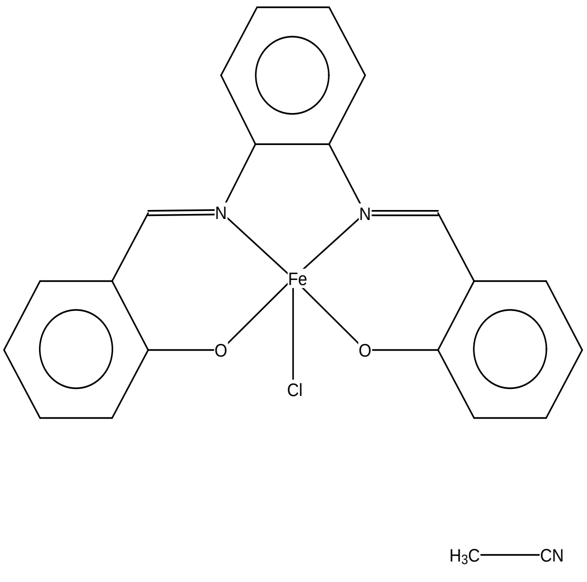

RIVCIJ

Reference: Ying-Xia Zhou, Xian-Fu Zheng, Dan Han, Hong-Yun Zhang, Xiao-Qing Shen, Cao-Yuan Niu, Pei-Kun Chen, Hong-Wei Hou, Yu Zhu (2006) *Synth.React.Inorg.,Met.-Org.,Nano-Met.Chem.* ,**36,**693

Formula: C_{22} H_{18} Mn_1 N_3 O_3 S_1,C_2 H_3 N_1

Compound Name: isothiocyanato-methanol-(N,N'-o-phenylenebis(salicylidenealdiminato))-manganese(iii) acetonitrile solvate

Space Group:	P21/c	***Cell:***	***a*** 8.776(1)	***b*** 29.402(6)	***c*** 9.941(2)
Space Group No.:	14	***(Å,°)***	α 90.00	β 111.29(3)	γ 90.00

R-Factor (%): 4.64 ***Temperature(K):*** 291 ***Density(g/cm^3):*** 1.391

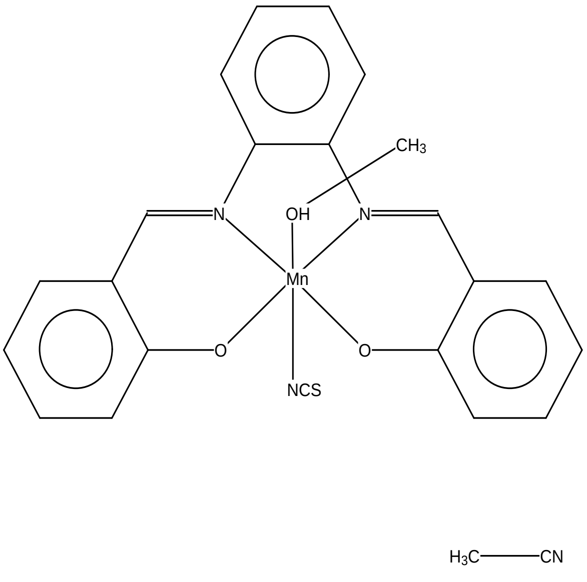

Search: search26 (Tue Dec 2 15:48:50 2014): Hits 517-520

RIZPUM

Reference: E.C.Escudero-Adan, J.Benet-Buchholz, A.W.Kleij (2008) *Inorg.Chem.* ,**47,**4256

Formula: $C_{46} H_{60} N_4 O_2 Zn_1,0.5(C_2 H_3 N_1)$

Compound Name: (1,2-Phenylene-bis(3,5-di-t-butylsalicylideneiminato))-(3-(N-methyl-2-tetrahydropyrrolyl)pyridine)-zinc(ii) acetonitrile solvate

Synonym: (1,2-Phenylene-bis(3,5-di-t-butylsalicylideneiminato))-(nicotine-N)-zinc(ii) acetonitrile solvate

Space Group:	P-1	***Cell:***	***a*** 16.743(0)	***b*** 17.340(0)	***c*** 17.503(0)		
Space Group No.:	2	***(Å,°)***	α 74.72(0)	β 65.09(0)	γ 85.27(0)		

R-Factor (%): 7.07 ***Temperature(K):*** 100 ***Density(g/cm³):*** 1.176

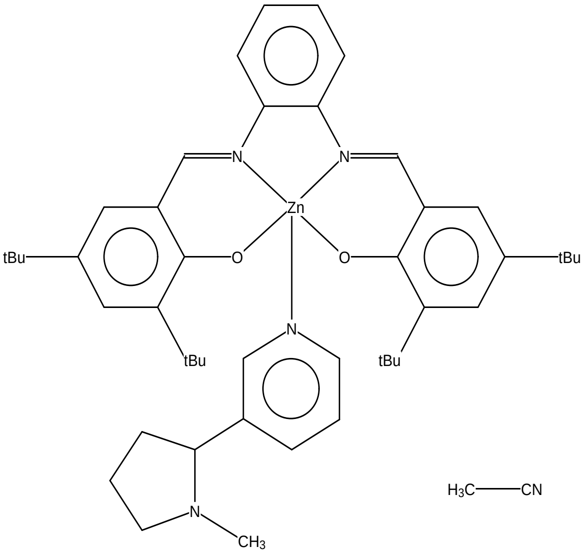

RIZQAT

Reference: E.C.Escudero-Adan, J.Benet-Buchholz, A.W.Kleij (2008) *Inorg.Chem.* ,**47,**4256

Formula: $C_{70} H_{82} N_8 O_4 Zn_2,C_1 H_2 Cl_2,C_2 H_3 N_1$

Compound Name: (μ_2-Benzene-1,2,4,5-tetrayl-tetrakis(3-t-butylsalicylideneiminato))-bis(3-(N-methyl-2-tetrahydropyrrolyl)pyridine)-di-zinc(ii) acetonitrile dichloromethane solvate

Space Group:	P-1	***Cell:***	***a*** 11.616(1)	***b*** 11.716(1)	***c*** 14.708(1)		
Space Group No.:	2	***(Å,°)***	α 78.04(0)	β 81.87(0)	γ 70.69(0)		

R-Factor (%): 4.76 ***Temperature(K):*** 100 ***Density(g/cm³):*** 1.222

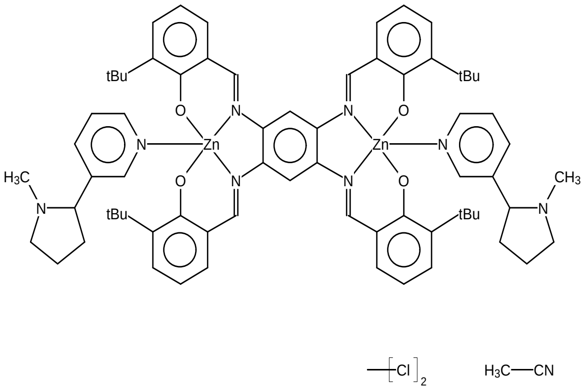

ROLSIV

Reference: A.Nabei, T.Kuroda-Sowa, T.Okubo, M.Maekawa, M.Munakata (2009) *Acta Crystallogr.,Sect.E:Struct.Rep.Online* ,**65,** m188

Formula: $C_{22} H_{18} Co_1 N_2 O_4,2(C_1 H_2 Cl_2)$

Compound Name: (6,6'-Dimethoxy-2,2'-(o-phenylenebis(nitrilomethylidyne))diphenolato)-cobalt(ii) dichloromethane solvate

Space Group:	P21/a	***Cell:***	***a*** 13.309(6)	***b*** 14.088(6)	***c*** 14.101(6)		
Space Group No.:	14	***(Å,°)***	α 90.00	β 109.68(0)	γ 90.00		

R-Factor (%): 4.63 ***Temperature(K):*** 150 ***Density(g/cm³):*** 1.609

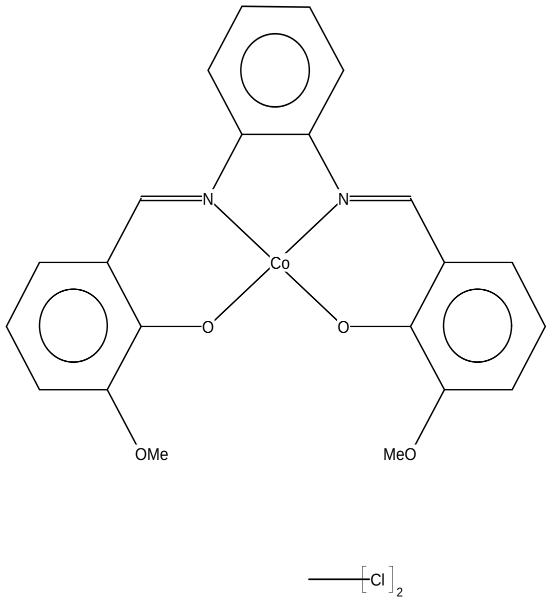

ROLXAS

Reference: Ming-Ming Yu, Hong Xu, Qiu-Zhi Shi, Ying-Nai Wei, Zhan-Xian Li (2009) *Acta Crystallogr.,Sect.E:Struct.Rep.Online* ,**65,** m225

Formula: $C_{22} H_{17} Br_1 N_2 Ni_1 O_4,C_1 H_4 O_1$

Compound Name: (6,6'-Dimethoxy-2,2'-(4-bromo-o-phenylenebis(nitrilomethylidyne)) diphenolato)-nickel(ii) methanol solvate

Space Group:	P-1	***Cell:***	***a*** 7.499(1)	***b*** 11.837(1)	***c*** 12.543(1)		
Space Group No.:	2	***(Å,°)***	α 105.04(0)	β 96.97(0)	γ 95.93(0)		

R-Factor (%): 4.38 ***Temperature(K):*** 295 ***Density(g/cm³):*** 1.710

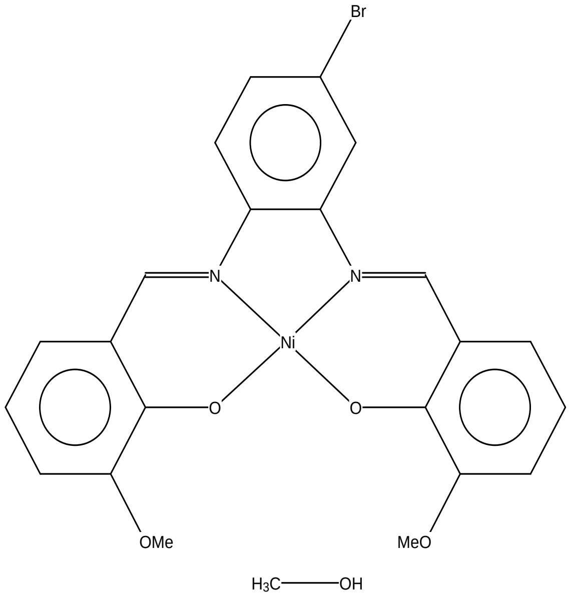

RUDXOD

Reference: S.Jonasdottir, Chang-Gyoun Kim, J.Kampf, D.Coucouvanis (1996) *Inorg.Chim.Acta* ,**243,**255

Formula: $C_{24} H_{22} Co_1 N_4 O_2, C_3 H_7 N_1 O_1$

Compound Name: (2,3-Ethylene-5,6:13,14-bis(5'-methylbenzo)-9,10-(4',5'-diolbenzo)-1,4,8,11-tetrazacyclotetradeca-7,11-diene)-cobalt(ii) dimethylformamide solvate

Space Group:	P21/c	***Cell:***	***a*** 9.087(2)	***b*** 7.962(2)	***c*** 34.012(7)		
Space Group No.:	14	***(Å,°)***	α 90.00	β 94.55(2)	γ 90.00		
R-Factor (%):	6.20	***Temperature(K):***	295	***Density(g/cm³):***	1.436		

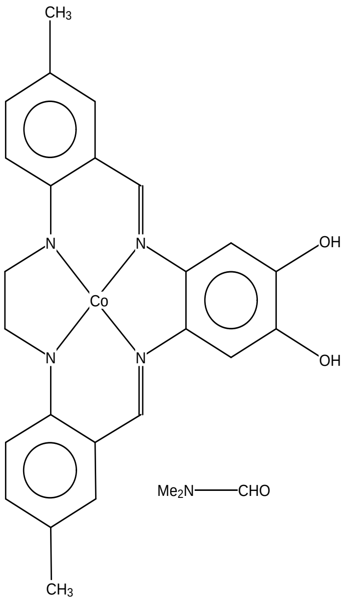

RUDXUJ

Reference: S.Jonasdottir, Chang-Gyoun Kim, J.Kampf, D.Coucouvanis (1996) *Inorg.Chim.Acta* ,**243,**255

Formula: $C_{24} H_{22} Co_1 N_4 O_2, C_4 H_8 O_1$

Compound Name: (2,3-Ethylene-5,6:13,14-bis(5'-methylbenzo)-9,10-(4',5'-diolbenzo)-1,4,8,11-tetrazacyclotetradeca-7,11-diene)-cobalt(ii) tetrahydrofuran solvate

Space Group:	P-1	***Cell:***	***a*** 11.134(4)	***b*** 13.383(6)	***c*** 17.286(6)
Space Group No.:	2	***(Å,°)***	α 77.42(3)	β 76.18(3)	γ 88.41(3)
R-Factor (%):	4.40	***Temperature(K):***	295	***Density(g/cm³):***	1.441

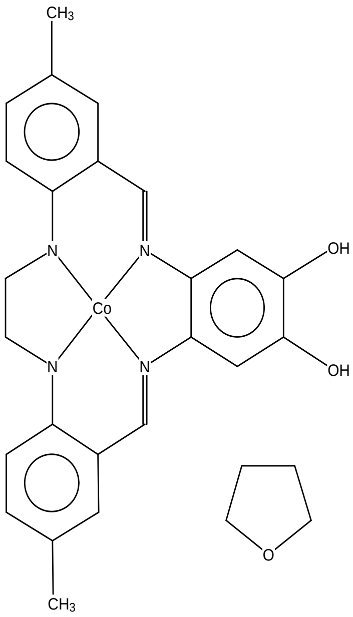

RUDYAQ

Reference: S.Jonasdottir, Chang-Gyoun Kim, J.Kampf, D.Coucouvanis (1996) *Inorg.Chim.Acta* ,**243,**255

Formula: $C_{48} H_{44} Cu_1 N_8 Ni_2 O_4{}^{2-}, 2(C_{16} H_{36} N_1{}^{1+}), 4(C_3 H_7 N_1 O_1)$

Compound Name: bis(Tetrabutylammonium) bis(μ_2-(2,3-ethylene-5,6:13,14-bis(5'-methylbenzo)-9,10-(4',5'-diolbenzo)-1,4,8,11-tetrazacyclotetradeca-7,11-diene)-nickel(ii))-copper(ii) dimethylformamide solvate

Space Group:	P21/c	***Cell:***	***a*** 10.260(20)	***b*** 35.210(40)	***c*** 12.541(13)
Space Group No.:	14	***(Å,°)***	α 90.00	β 95.54(11)	γ 90.00
R-Factor (%):	6.22	***Temperature(K):***	178	***Density(g/cm³):***	1.293

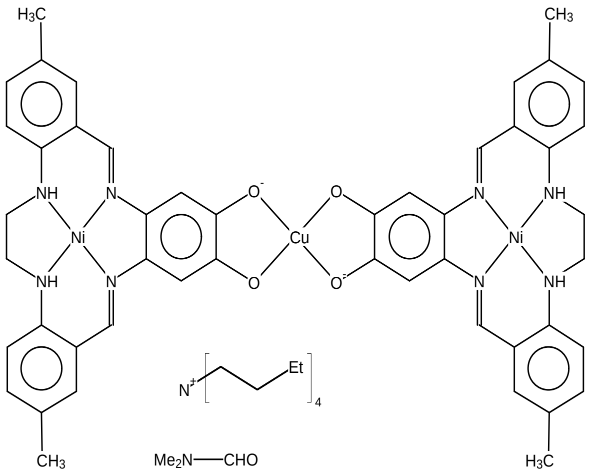

RUPWAB

Reference: N.E.Eltayeb, S.G.Teoh, C.S.Yeap, H.-K.Fun, R.Adnan (2009) *Acta Crystallogr.,Sect.E:Struct.Rep.Online* ,**65,**m1692

Formula: $C_{36} H_{46} Cl_1 Mn_1 N_2 O_2$

Compound Name: Chlorido-(4,4',6,6'-tetra-t-butyl-2,2'-[o-phenylenebis(nitrilomethylidyne)]diphenolato-κ⁴O,N,N',O')-manganese(iii)

Synonym: Chloro-(2,2'-(1,2-phenylenebis((nitrilo-κN)methylylidene))bis(4,6-di-t-butylphenolato-κO))-manganese(iii)

Space Group:	P-1	***Cell:***	***a*** 10.765(0)	***b*** 16.900(0)	***c*** 20.232(1)
Space Group No.:	2	***(Å,°)***	α 107.36(0)	β 90.01(0)	γ 107.29(0)
R-Factor (%):	4.90	***Temperature(K):***	100	***Density(g/cm³):***	1.252

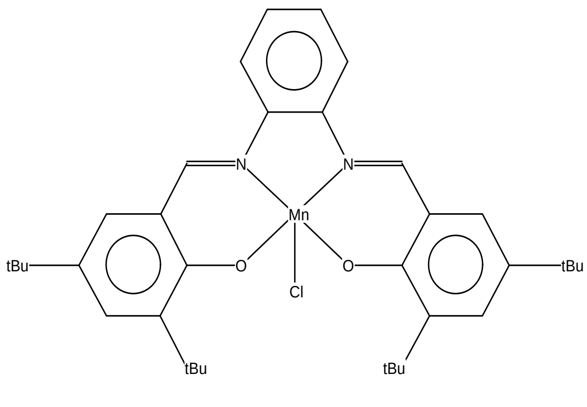

Search: search26 (Tue Dec 2 15:48:50 2014): Hits 525-528

RUQBUB

Reference: R.Mayilmurugan, H.Stoeckli-Evans, E.Suresh, M.Palaniandavar (2009) *Dalton Trans.* ,5101

Formula: $C_{72} H_{92} Fe_2 N_4 O_5,2(H_2 O_1)$

Compound Name: (μ_2-Oxo)-bis(N,N'-bis(3,5-di-t-butylsalicylidene)-1,2-phenylenediamine-N, N',O,O')-di-iron(iii) dihydrate

Space Group: P-1 ***Cell:*** ***a*** 14.198(1) ***b*** 15.141(1) ***c*** 17.959(1)
Space Group No.: 2 ***(Å,°)*** α 101.53(0) β 102.96(0) γ 100.44(0)

R-Factor (%): 6.45 ***Temperature(K):*** 173 ***Density(g/cm³):*** 1.151

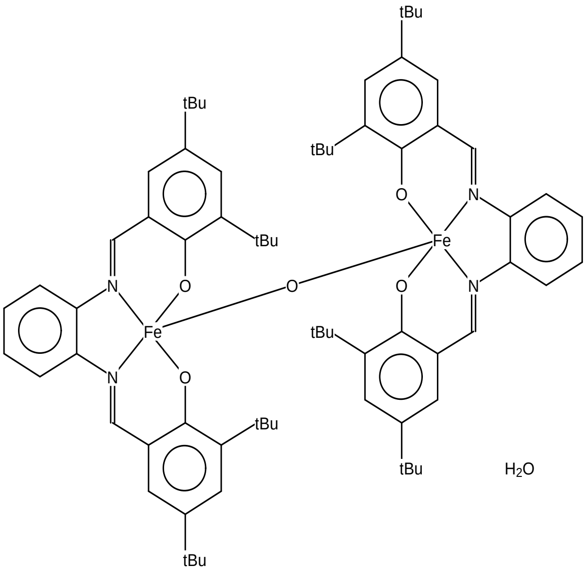

RURPEZ

Reference: D.A.Atwood, M.S.Hill, J.A.Jegier, D.Rutherford (1997) *Organometallics* ,**16,**2659

Formula: $C_{42} H_{59} Al_1 N_2 O_2,C_7 H_8$

Compound Name: Isobutyl-(N,N'-(4,5-dimethyl-o-phenylene)-bis(3,5-di-t-butylsalicylideniminato))-aluminium toluene solvate

Space Group: P21/c ***Cell:*** ***a*** 14.156(4) ***b*** 13.298(1) ***c*** 24.794(2)
Space Group No.: 14 ***(Å,°)*** α 90.00 β 97.44(2) γ 90.00

R-Factor (%): 6.12 ***Temperature(K):*** 295 ***Density(g/cm³):*** 1.066

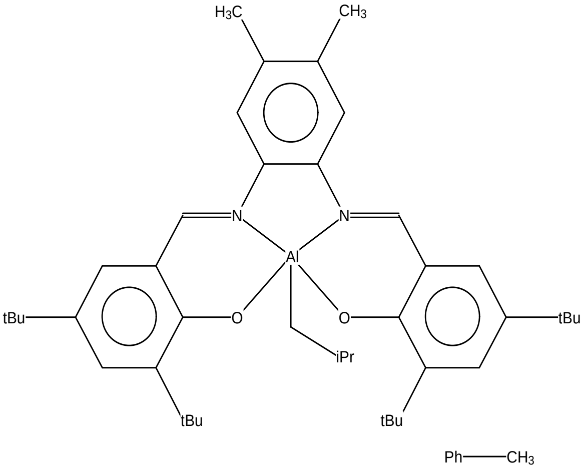

RURPUP

Reference: D.A.Atwood, M.S.Hill, J.A.Jegier, D.Rutherford (1997) *Organometallics* ,**16,**2659

Formula: $C_{54} H_{61} Al_1 N_2 O_3 Si_1$

Compound Name: (N,N'-Phenylene-bis(3,5-di-t-butylsalicylideniminato))-triphenylsiloxy-aluminium

Space Group: P21/c ***Cell:*** ***a*** 18.333(7) ***b*** 14.261(6) ***c*** 19.127(7)
Space Group No.: 14 ***(Å,°)*** α 90.00 β 108.16(2) γ 90.00

R-Factor (%): 6.95 ***Temperature(K):*** 295 ***Density(g/cm³):*** 1.176

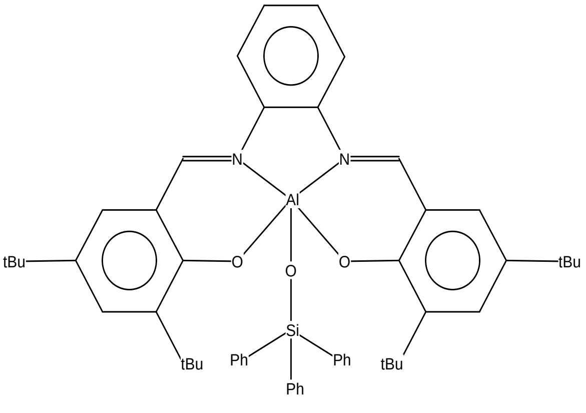

RUSXIN

Reference: M.Salehi, M.Amirnasr, K.Mereiter (2010) *J.Iran.Chem.Soc.* ,**7,**740

Formula: $C_{24} H_{24} Co_1 N_4 O_2 S_2{}^{1+},Cl_1 O_4{}^{1-},C_1 H_1 Cl_3$

Compound Name: bis(ethanethioamide)-(2,2'-(1,2-phenylenebis((nitrilo)methylylidene)) diphenolato)-cobalt(iii) perchlorate chloroform solvate

Space Group: Pca21 ***Cell:*** ***a*** 17.348(1) ***b*** 18.752(1) ***c*** 18.813(1)
Space Group No.: 29 ***(Å,°)*** α 90.00 β 90.00 γ 90.00

R-Factor (%): 4.70 ***Temperature(K):*** 100 ***Density(g/cm³):*** 1.611

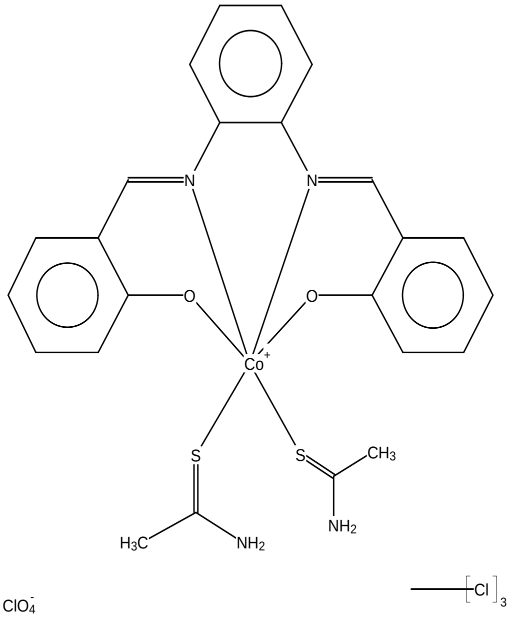

Search: search26 (Tue Dec 2 15:48:50 2014): Hits 529-532

RUSXOT

Reference: M.Salehi, M.Amirnasr, K.Mereiter (2010) *J.Iran.Chem.Soc.* ,**7,**740

Formula: $C_{34} H_{28} Co_1 N_4 O_2 S_2{}^{1+}, Cl_1 O_4{}^{1-}, C_1 H_1 Cl_3$

Compound Name: bis(benzenecarbothioamide)-(2,2'-(1,2-phenylenebis((nitrilo) methylylidene))diphenolato)-cobalt(iii) perchlorate chloroform solvate

Space Group:	P21/n	***Cell:***	***a*** 14.710(3)	***b*** 13.506(3)	***c*** 18.595(4)
Space Group No.:	14	***(Å,°)***	α 90.00	β 100.30(0)	γ 90.00

R-Factor (%): 7.69 ***Temperature(K):*** 100 ***Density(g/cm^3):*** 1.583

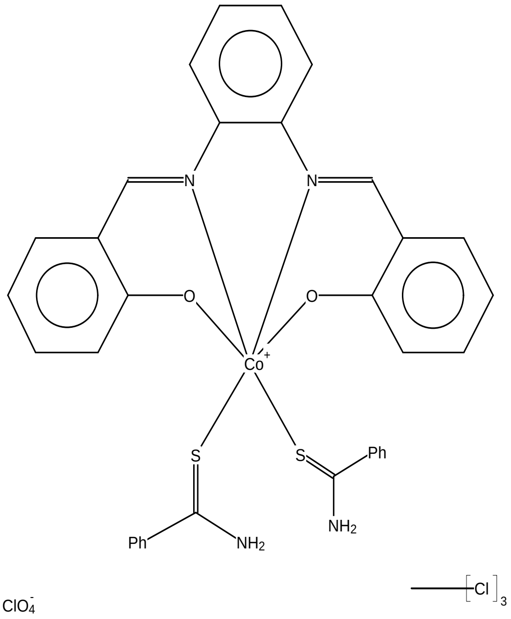

RUVYEN

Reference: Feng Luo, Ming-biao Luo (2010) *Inorg.Chem.Commun.* , **13,**319

Formula: $C_{20} H_{16} Cl_2 Co_2 N_2 O_3$

Compound Name: (μ_2-2,2'-(1,2-Phenylenebis((nitrilo)methylylidene))diphenolato)-aqua-dichloro-di-cobalt(ii)

Synonym: (μ_2-N,N'-o-Phenylenebis(salicylideneiminato))-aqua-dichloro-di-cobalt(ii)

Space Group:	P21/c	***Cell:***	***a*** 10.014(2)	***b*** 14.717(3)	***c*** 16.240(5)
Space Group No.:	14	***(Å,°)***	α 90.00	β 125.39(2)	γ 90.00

R-Factor (%): 2.72 ***Temperature(K):*** 298 ***Density(g/cm^3):*** 1.774

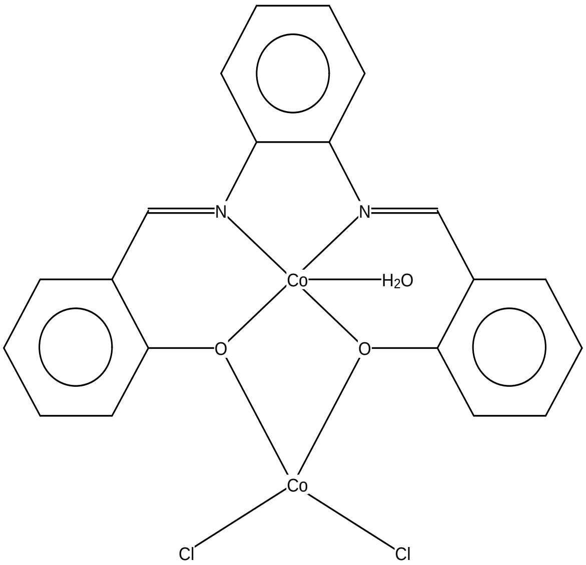

RUZGIC

Reference: A.Hori, M.Yonemura, M.Ohba, H.Okawa (2001) *Bull.Chem.Soc.Jpn.* ,**74,**495

Formula: $C_{29} H_{32} Cu_1 N_4 O_2$

Compound Name: (1^5,7^5,9,13-Tetramethyl-3,5,9,13-tetraaza-1,7(1,3),4(1,2)-tribenzenacyclotetradecaphane-2,5-diene-1^2,7^2-diolato)-copper(ii)

Space Group:	P21/c	***Cell:***	***a*** 8.371(3)	***b*** 15.026(3)	***c*** 19.854(2)
Space Group No.:	14	***(Å,°)***	α 90.00	β 93.26(2)	γ 90.00

R-Factor (%): 4.30 ***Temperature(K):*** 295 ***Density(g/cm^3):*** 1.418

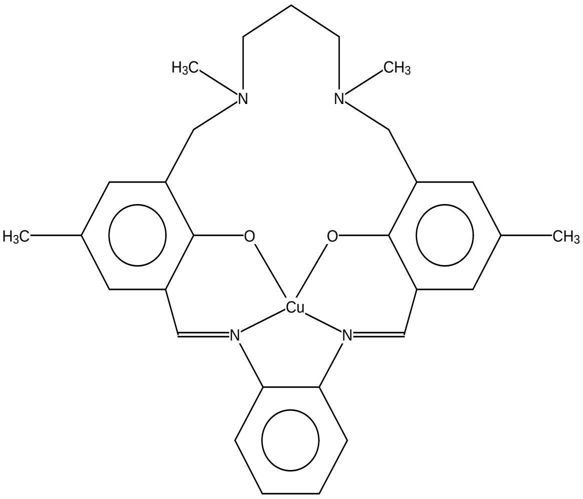

SABSOE

Reference: Huanwang Jing, S.K.Edulji, J.M.Gibbs, C.L.Stern, Hongying Zhou, S.T.Nguyen (2004) *Inorg.Chem.* ,**43,**4315

Formula: $C_{36} H_{46} N_2 O_2 Sn_1$

Compound Name: (N,N'-bis(3,5-Di-t-butylsalicylidene)-1,2-benzenediamine)-tin(ii)

Space Group:	P-1	***Cell:***	***a*** 9.692(2)	***b*** 12.177(2)	***c*** 15.156(3)
Space Group No.:	2	***(Å,°)***	α 88.41(0)	β 84.68(0)	γ 69.28(0)

R-Factor (%): 4.90 ***Temperature(K):*** 153 ***Density(g/cm^3):*** 1.311

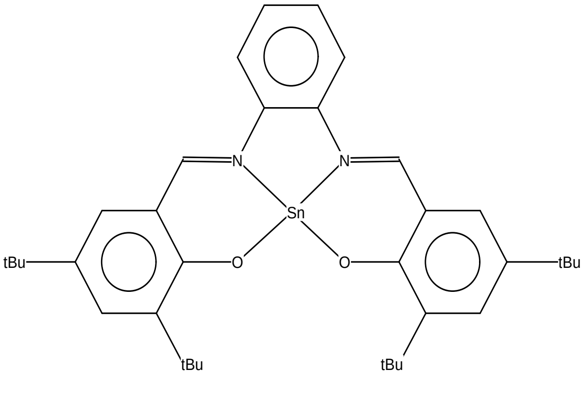

SABTEV

Reference: Huanwang Jing, S.K.Edulji, J.M.Gibbs, C.L.Stern, Hongying Zhou, S.T.Nguyen (2004) *Inorg.Chem.* ,**43,**4315

Formula: $C_{36}\ H_{46}\ Br_2\ N_2\ O_2\ Sn_1,C_1\ H_2\ Cl_2$

Compound Name: Dibromo-(N,N'-bis(3,5-di-t-butylsalicylidene)-1,2-benzenediamine)-tin(iv) dichloromethane solvate

Space Group:	P-1	***Cell:***	***a*** 11.419(2)	***b*** 13.057(2)	***c*** 13.845(3)
Space Group No.:	2	***(Å,°)***	α 75.09(0)	β 76.77(0)	γ 89.06(0)
R-Factor (%):	3.90	***Temperature(K):*** 153	***Density(g/cm^3):*** 1.545		

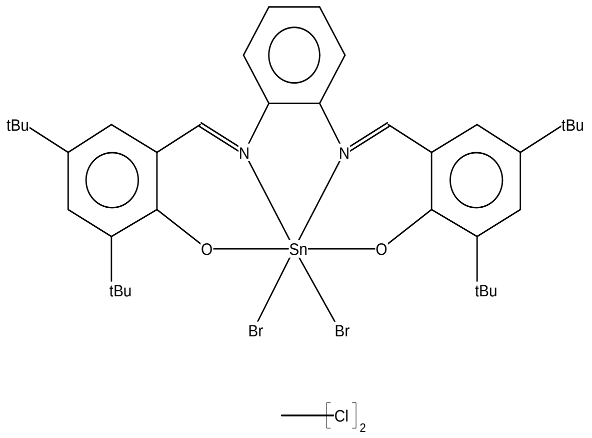

SAFDUA

Reference: Yang Lin, Guang-Feng Hou, Guang-Ming Li, Peng-Fei Yan (2012) *Acta Crystallogr.,Sect.E:Struct.Rep.Online* ,**68,** m61

Formula: $C_{23}\ H_{20}\ Co_1\ N_3\ O_5,0.5(C_2\ H_3\ N_1)$

Compound Name: Aqua-(cyano)-(2,2'-(1,2-phenylenebis((nitrilo)methylylidene))-bis(6-methoxyphenolato))-cobalt acetonitrile solvate

Space Group:	P-1	***Cell:***	***a*** 8.649(1)	***b*** 11.689(2)	***c*** 12.229(2)
Space Group No.:	2	***(Å,°)***	α 112.10(3)	β 102.30(3)	γ 97.85(3)
R-Factor (%):	4.94	***Temperature(K):*** 293	***Density(g/cm^3):*** 1.522		

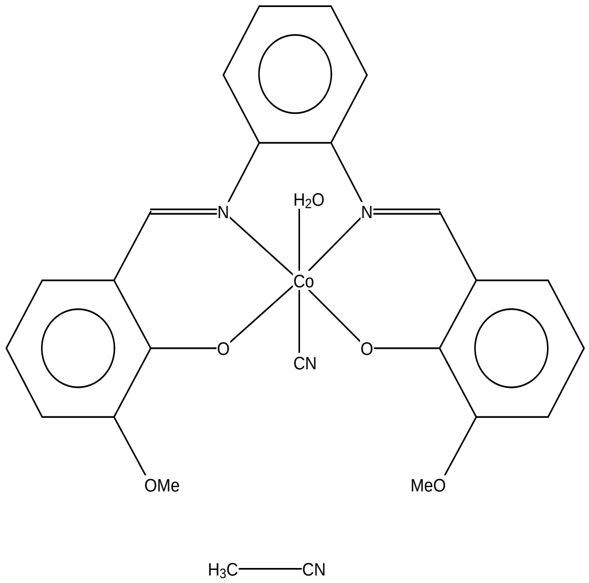

SAHSAV

Reference: D.T.Rosa, D.Coucouvanis (1998) *Inorg.Chem.* ,**37,**2328

Formula: $C_{92}\ H_{128}\ Cs_2\ N_4\ Ni_2\ O_{16}\ ^{2+},2(I_1\ ^{1-}),6(C_1\ H_2\ Cl_2)$

Compound Name: bis(μ_3-4,5-bis(3,5-Di-t-butylsalicylideneamino)-benzo-18-crown-6)-di-cesium-di-nickel di-iodide dichloromethane solvate

Space Group:	P-1	***Cell:***	***a*** 10.069(0)	***b*** 16.627(0)	***c*** 19.230(0)
Space Group No.:	2	***(Å,°)***	α 114.67(0)	β 102.10(0)	γ 93.29(0)
R-Factor (%):	5.55	***Temperature(K):*** 173	***Density(g/cm^3):*** 1.584		

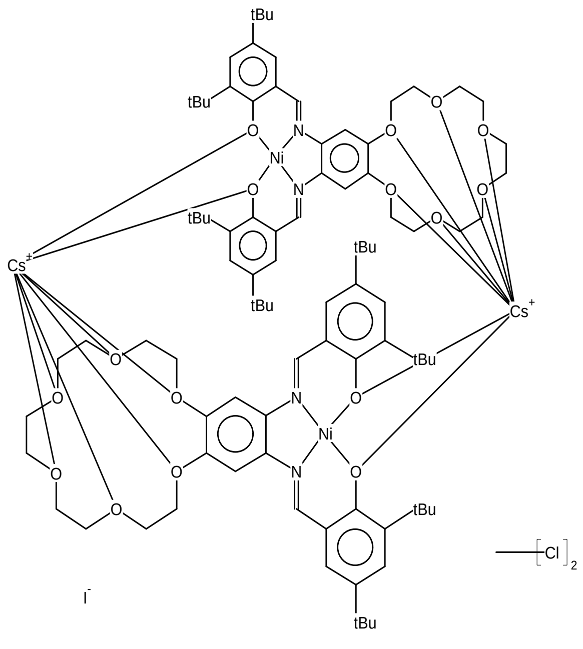

SAHSAV01

Reference: J.D.Pike, D.T.Rosa, D.Coucouvanis (2001) *Eur.J.Inorg.Chem.* ,761

Formula: $C_{92}\ H_{128}\ Cs_2\ N_4\ Ni_2\ O_{16}\ ^{2+},2(I_1\ ^{1-}),6(C_1\ H_2\ Cl_2)$

Compound Name: bis(μ_3-4,5-bis(3,5-Di-t-butylsalicylideneamino)benzo-18-crown-6)-di-cesium-di-nickel(ii) di-iodide dichloromethane solvate

Space Group:	P-1	***Cell:***	***a*** 10.069(0)	***b*** 16.627(0)	***c*** 19.230(0)
Space Group No.:	2	***(Å,°)***	α 114.67(0)	β 102.10(0)	γ 93.29(0)
R-Factor (%):	5.55	***Temperature(K):*** 173	***Density(g/cm^3):*** 1.584		

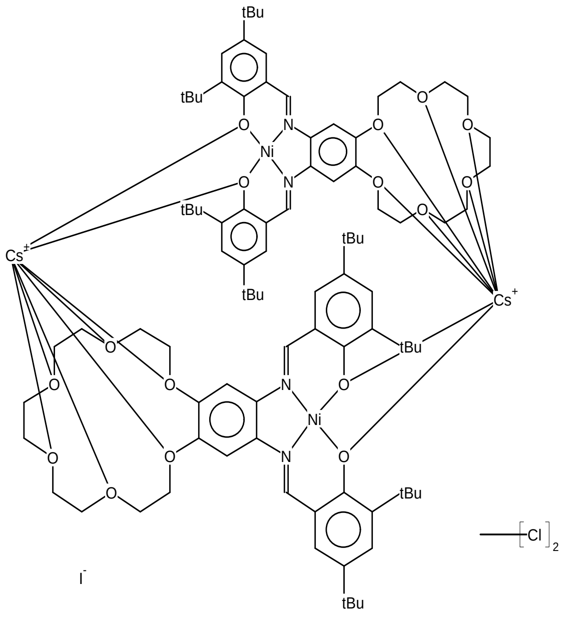

Search: search26 (Tue Dec 2 15:48:50 2014): Hits 537-540

SAHTAW

Reference: D.T.Rosa, D.Coucouvanis (1998) *Inorg.Chem.* ,**37**,2328

Formula: $C_{46} H_{66} K_1 N_2 Ni_1 O_9{}^{1+}, Cl_1{}^{1-}, C_1 H_1 Cl_3, C_1 H_4 O_1$

Compound Name: (μ_2-4,5-bis(3,5-Di-t-butylsalicylideneamino)-benzo-18-crown-6)-aqua-potassium-nickel chloride chloroform methanol solvate

Space Group:	P-1	***Cell:***	***a*** 10.379(0)		***b*** 15.955(0)		***c*** 18.449(0)
Space Group No.:	2	***(Å,°)***	α 112.16(1)		β 102.97(1)		γ 96.43(1)
R-Factor (%):	6.02	***Temperature(K):***	158		***Density(g/cm3):***		1.328

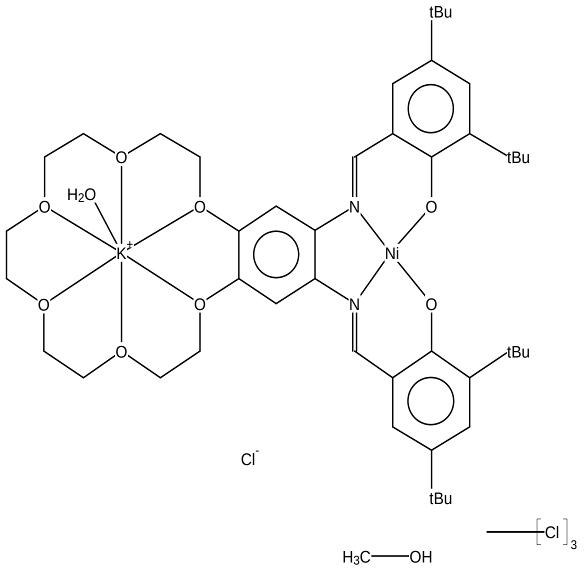

SAHTAW01

Reference: J.D.Pike, D.T.Rosa, D.Coucouvanis (2001) *Eur.J.Inorg.Chem.* ,761

Formula: $C_{46} H_{66} K_1 N_2 Ni_1 O_9{}^{1+}, Cl_1{}^{1-}, C_1 H_1 Cl_3, C_1 H_4 O_1$

Compound Name: (μ_2-4,5-bis(3,5-Di-t-butylsalicylideneamino)benzo-18-crown-6)-aqua-potassium-nickel(ii) chloride chloroform methanol solvate

Space Group:	P-1	***Cell:***	***a*** 10.379(0)		***b*** 15.955(0)		***c*** 18.449(0)
Space Group No.:	2	***(Å,°)***	α 112.16(1)		β 102.97(1)		γ 96.43(1)
R-Factor (%):	6.02	***Temperature(K):***	158		***Density(g/cm3):***		1.328

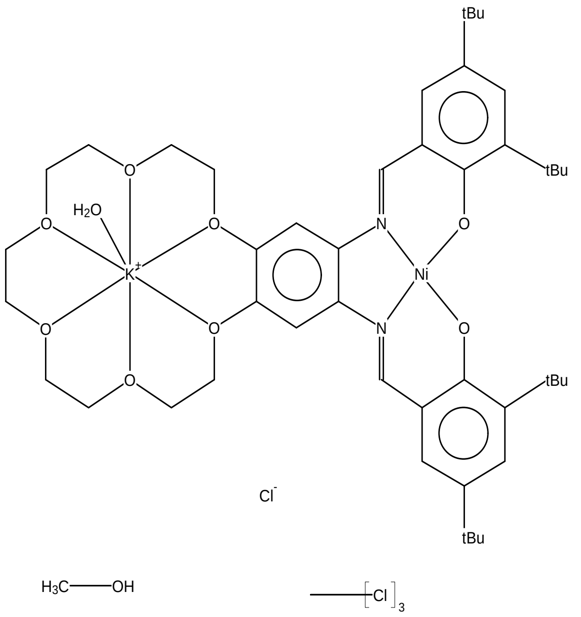

SAHTEA

Reference: D.T.Rosa, D.Coucouvanis (1998) *Inorg.Chem.* ,**37**,2328

Formula: $C_{46} H_{64} N_2 Ni_1 O_8, Cl_1{}^{1-}, C_6 H_{16} N_1{}^{1+}, C_1 H_4 O_1, C_3 H_6 O_1$

Compound Name: (4,5-bis(3,5-di-t-butylsalicylideneamino)benzo-18-crown-6)-nickel(ii) hexylammonium chloride acetone methanol solvate

Space Group:	P21/n	***Cell:***	***a*** 10.589(0)		***b*** 19.151(0)		***c*** 30.982(0)
Space Group No.:	14	***(Å,°)***	α 90.00		β 93.71(0)		γ 90.00
R-Factor (%):	5.55	***Temperature(K):***	158		***Density(g/cm3):***		1.123

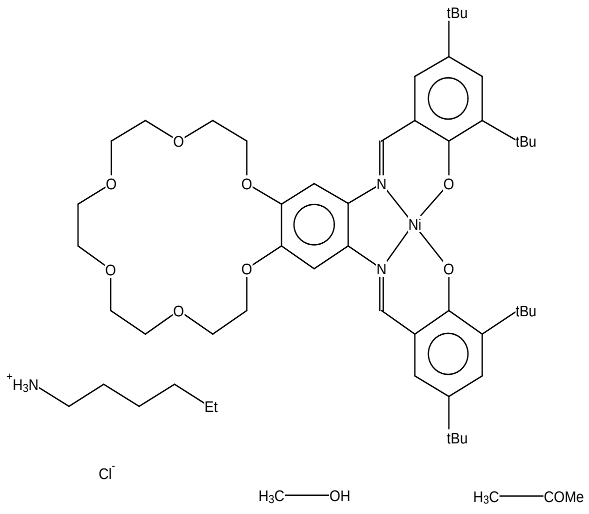

SAHTEA01

Reference: J.D.Pike, D.T.Rosa, D.Coucouvanis (2001) *Eur.J.Inorg.Chem.* ,761

Formula: $C_{46} H_{64} N_2 Ni_1 O_8, Cl_1{}^{1-}, C_6 H_{16} N_1{}^{1+}, C_1 H_4 O_1, C_3 H_6 O_1$

Compound Name: (4,5-bis(3,5-di-t-butylsalicylideneamino)benzo-18-crown-6)-nickel(ii) hexylammonium chloride acetone methanol solvate

Space Group:	P21/n	***Cell:***	***a*** 10.589(0)		***b*** 19.151(0)		***c*** 30.982(0)
Space Group No.:	14	***(Å,°)***	α 90.00		β 93.71(0)		γ 90.00
R-Factor (%):	5.55	***Temperature(K):***	158		***Density(g/cm3):***		1.123

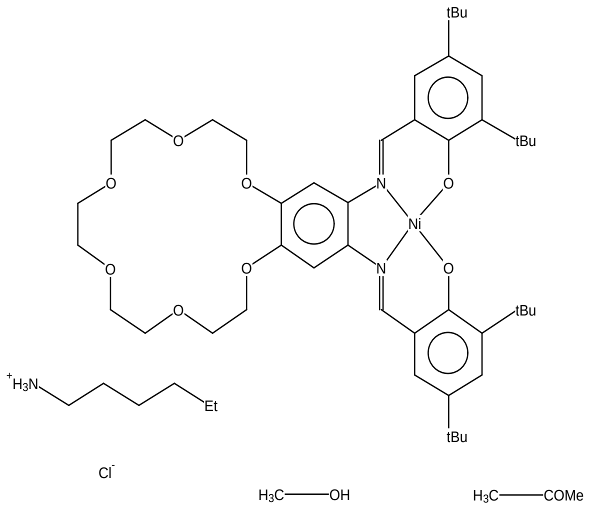

Search: search26 (Tue Dec 2 15:48:50 2014): Hits 541-544

SAHTUQ

Reference: D.T.Rosa, D.Coucouvanis (1998) *Inorg.Chem.* ,**37,**2328

Formula: $C_{92} H_{128} Cs_1 N_4 Ni_2 O_{16}$ $^{1+}$,I_1 $^{1-}$,2.5($C_1 H_4 O_1$),0.5($H_2 O_1$)

Compound Name: bis(μ_2-4,5-bis(3,5-di-t-butylsalicylideneamino)benzo-18-crown-6)-cesium-di-nickel(ii) iodide methanol solvate hemihydrate

Space Group:	P-1	***Cell:***	***a*** 18.818(4)	***b*** 19.544(4)	***c*** 20.335(4)
Space Group No.:	2	***(Å,°)***	α 106.57(3)	β 102.75(3)	γ 114.16(3)
R-Factor (%):	12.20	***Temperature(K):*** 295	***Density(g/cm³):*** 1.108		

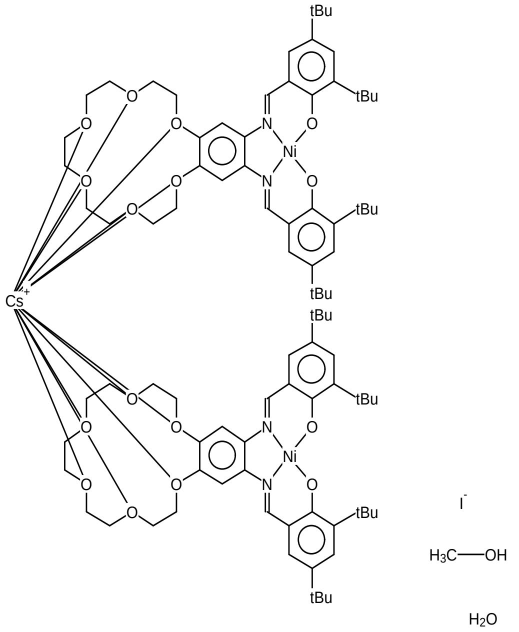

SAHTUQ01

Reference: J.D.Pike, D.T.Rosa, D.Coucouvanis (2001) *Eur.J.Inorg.Chem.* ,761

Formula: $C_{92} H_{128} Cs_1 N_4 Ni_2 O_{16}$ $^{1+}$,I_1 $^{1-}$,2.5($C_1 H_4 O_1$),0.5($H_2 O_1$)

Compound Name: bis(μ_2-4,5-bis(3,5-di-t-butylsalicylideneamino)benzo-18-crown-6)-cesium-di-nickel(ii) iodide methanol solvate hemihydrate

Space Group:	P-1	***Cell:***	***a*** 18.818(4)	***b*** 19.544(4)	***c*** 20.335(4)
Space Group No.:	2	***(Å,°)***	α 106.57(3)	β 102.75(3)	γ 114.16(3)
R-Factor (%):	12.20	***Temperature(K):*** 293	***Density(g/cm³):*** 1.108		

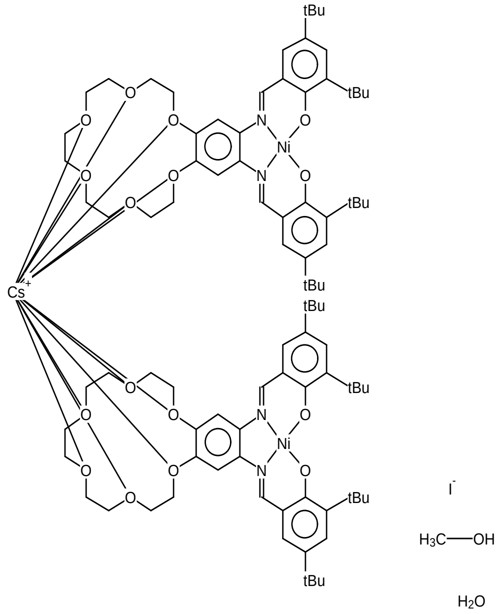

SAJMOG

Reference: R.Kannappan, S.Tanase, D.M.Tooke, A.L.Spek, I.Mutikainen, U.Turpeinen, J.Reedijk (2004) *Polyhedron* ,**23,**2285

Formula: $C_{39} H_{54} N_2 O_5 U_1$

Compound Name: (N,N'-bis(3,5-Di-t-butylsalicylidene)-4,5-dimethyl-1,2-phenylenediamine)-(methanol)-dioxo-uranium(vi)

Space Group:	P21/c	***Cell:***	***a*** 13.637(3)	***b*** 29.118(6)	***c*** 10.476(2)
Space Group No.:	14	***(Å,°)***	α 90.00	β 112.26(3)	γ 90.00
R-Factor (%):	4.42	***Temperature(K):*** 173	***Density(g/cm³):*** 1.499		

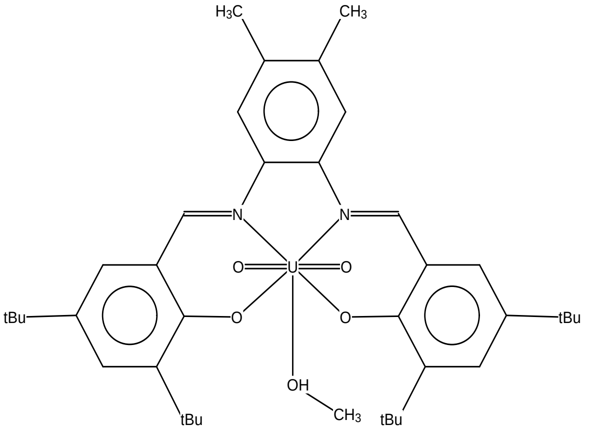

SALPUO

Reference: G.Bandoli, D.A.Clemente, U.Croatto, M.Vidali, P.A.Vigato (1971) *J.Chem.Soc.D* ,1330

Formula: $C_{22} H_{20} N_2 O_5 U_1$

Compound Name: Ethanol-(N,N'-o-phenylene-bis(salicylideneiminato))-dioxo-uranium

Space Group:	P21/c	***Cell:***	***a*** 10.382	***b*** 9.564	***c*** 23.026
Space Group No.:	14	***(Å,°)***	α 90.00	β 107.78	γ 90.00
R-Factor (%):	6.40	***Temperature(K):*** 295	***Density(g/cm³):*** 1.923		

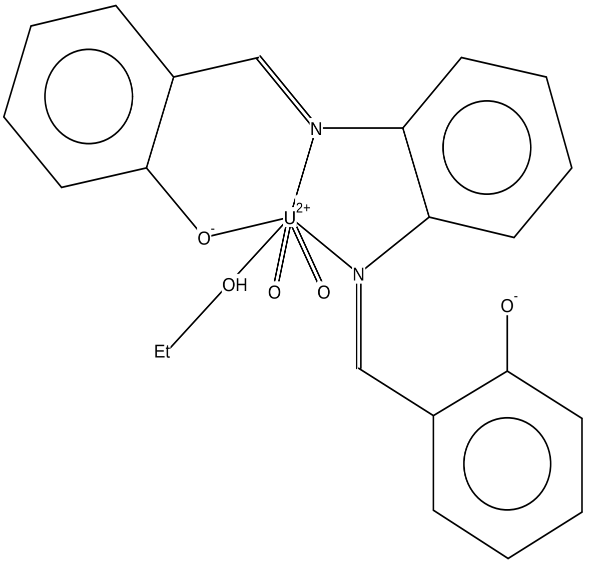

SAXRUE

Reference: F.C.J.M.van Veggel, M.Bos, S.Harkema, W.Verboom, D.N.Reinhoudt (1989) *Angew.Chem.,Int.Ed.* ,**28,**746

Formula: $C_{49}\ H_{42}\ Ba_1\ Cu_2\ F_3\ N_4\ O_{14}\ S_1\ ^{1+},C_1\ F_3\ O_3\ S_1\ ^{1-},H_2\ O_1$

Compound Name: (μ_3-bis(3,3'-(1,4,7-Trioxaheptane-1,7-diyl)-N,N'-o-phenylene-bis(salicylideneaminato)))-aqua-(trifluoromethanesulfonato-O)-barium-di-copper trifluoromethanesulfonate monohydrate

Space Group:	Pbca	***Cell:***	***a*** 28.589(9)	***b*** 27.786(4)	***c*** 13.383(3)
Space Group No.:	61	***(Å,°)***	α 90.00	β 90.00	γ 90.00

R-Factor (%): 7.80 ***Temperature(K):*** 295 ***Density(g/cm³):*** 1.789

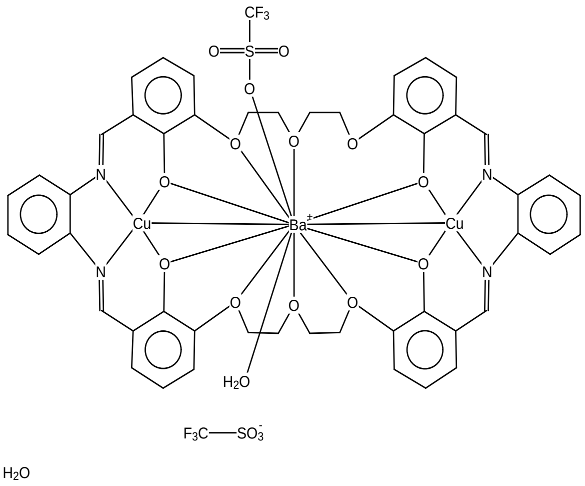

SAXRUE10

Reference: F.C.J.M.van Veggel, M.Bos, S.Harkema, H.van de Bovenkamp, W.Verboom, J.Reedijk, D.N.Reinhoudt (1991) *J.Org.Chem.* ,**56,**225

Formula: $C_{49}\ H_{42}\ Ba_1\ Cu_2\ F_3\ N_4\ O_{14}\ S_1\ ^{1+},C_1\ F_3\ O_3\ S_1\ ^{1-},H_2\ O_1$

Compound Name: (μ_3-13,14,16,17,38,39,41,42-Octahydro-7,11:19,23:32,36:44,48-tetramethenodibenzo(o,l_1)-(1,4,7,24,27,30,14,17,37,40)hexaoxatetra-azacyclohexatetracontine-51,52,53,54-tetraolato)-di-copper-aqua-(trifluromethanesulfonato-O)-barium trifluoromethanesulfonate monohydrate

Space Group:	Pbca	***Cell:***	***a*** 28.589(9)	***b*** 27.786(4)	***c*** 13.383(3)
Space Group No.:	61	***(Å,°)***	α 90.00	β 90.00	γ 90.00

R-Factor (%): 7.80 ***Temperature(K):*** 295 ***Density(g/cm³):*** 1.789

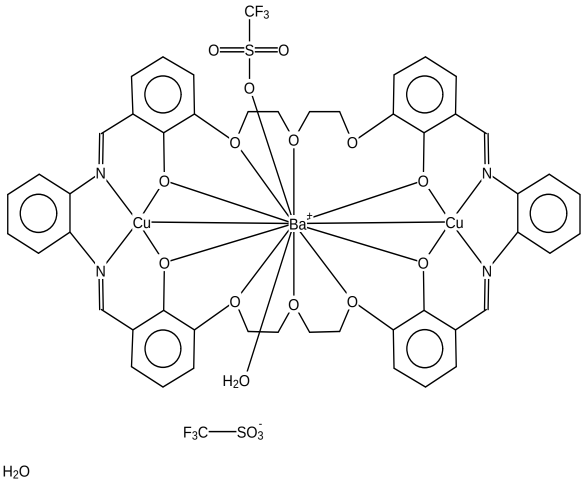

SAZPZR

Reference: R.D.Archer, R.O.Day, M.L.Illingsworth (1979) *Inorg.Chem.* ,**18,**2908

Formula: $C_{40}\ H_{28}\ N_4\ O_4\ Zr_1,2.5(C_6\ H_6)$

Compound Name: bis(N,N'-Disalicylidene-1,2-phenylenediamino)-zirconium(iv) benzene solvate

Space Group:	P-1	***Cell:***	***a*** 13.037(4)	***b*** 13.669(6)	***c*** 14.500(2)
Space Group No.:	2	***(Å,°)***	α 98.81(2)	β 116.47(2)	γ 92.78(3)

R-Factor (%): 4.10 ***Temperature(K):*** 295 ***Density(g/cm³):*** 1.341

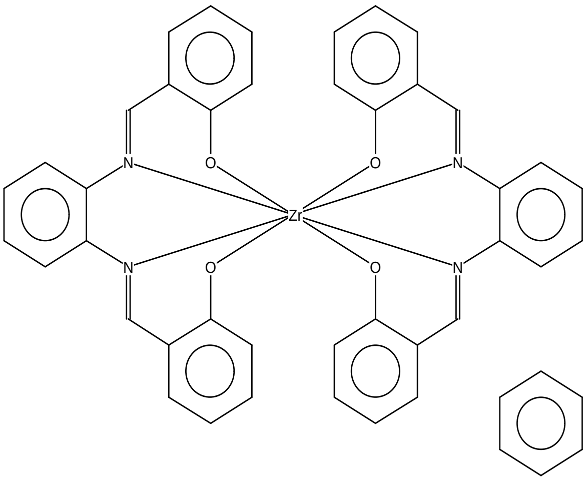

SCLIRB

Reference: R.Bonnaire, C.Potvin, J.M.Manoli (1980) *Inorg.Chim.Acta* , **45,**L255

Formula: $C_{36}\ H_{38}\ N_2\ O_2\ Rh_2$

Compound Name: (μ_2-N,N'-(1,2-Phenylene)-bis(salicylideneiminato))-bis(cyclo-octa-1,5-diene)-di-rhodium(i)

Space Group:	Pccn	***Cell:***	***a*** 12.647(2)	***b*** 15.964(2)	***c*** 14.507(3)
Space Group No.:	56	***(Å,°)***	α 90.00	β 90.00	γ 90.00

R-Factor (%): 3.80 ***Temperature(K):*** 295 ***Density(g/cm³):*** 1.670

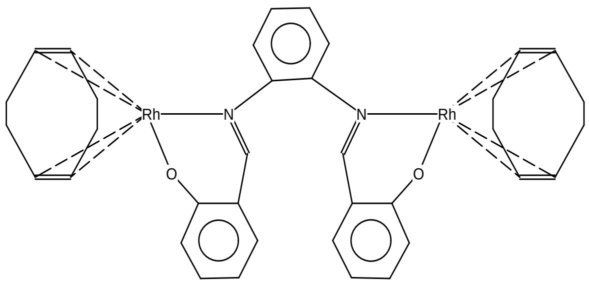

Search: search26 (Tue Dec 2 15:48:50 2014): Hits 549-552

SCLIRB10

Reference: R.Bonnaire, J.M.Manoli, C.Potvin, N.Platzer, N.Goasdoue, D.Davoust (1982) *Inorg.Chem.* ,**21,**2032

Formula: C_{36} H_{38} N_2 O_2 Rh_2

Compound Name: (μ_2-N,N'-o-Phenylene-bis(salicylaldiminato))-bis(η^4-cyclo-octa-1,5-diene) -di-rhodium(i)

Space Group: Pccn ***Cell:*** ***a*** 12.647(2) ***b*** 15.964(2) ***c*** 14.507(3)
Space Group No.: 56 ***(Å,°)*** α 90.00 β 90.00 γ 90.00

R-Factor (%): 3.82 ***Temperature(K):*** 295 ***Density(g/cm³):*** 1.670

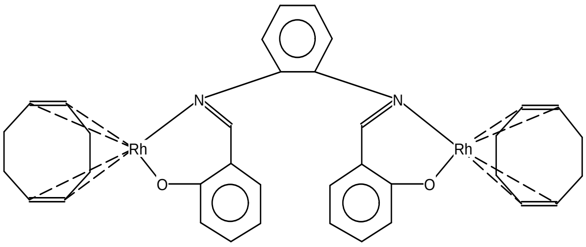

SCLIRB11

Reference: C.Janiak, A.-C.Chamayou, A.K.M.R.Uddin, M.Uddin, K.S.Haggen, M.Enamullah (2009) *Dalton Trans.* ,3698

Formula: C_{36} H_{38} N_2 O_2 Rh_2

Compound Name: (μ_2-N,N'-(1,2-Phenylene)bis(salicylaldiminato)-N,N',O,O')-bis(η^2,η^2-cyclo-octa-1,5-diene)-di-rhodium(i)

Space Group: P-1 ***Cell:*** ***a*** 10.652(0) ***b*** 11.988(0) ***c*** 12.089(0)
Space Group No.: 2 ***(Å,°)*** α 77.50(0) β 89.99(0) γ 76.81(0)

R-Factor (%): 2.99 ***Temperature(K):*** 173 ***Density(g/cm³):*** 1.669

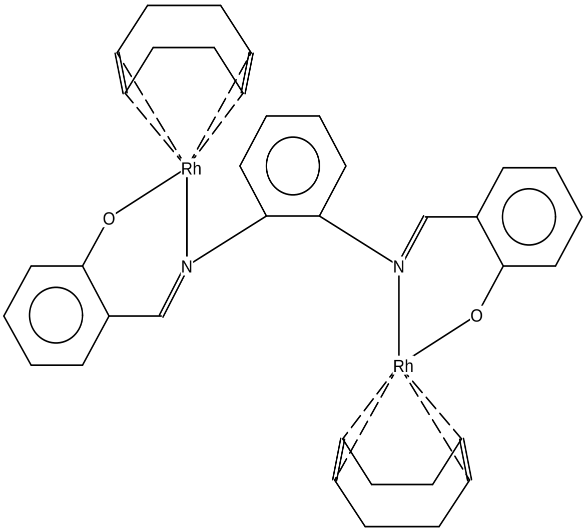

SEHXIO

Reference: A.S.Ogunlaja, W.Chidawanyika, E.Antunes, M.A.Fernandes, T.Nyokong, N.Torto, Z.R.Tshentu (2012) *Dalton Trans.* ,**41,**13908

Formula: C_{20} H_{14} N_2 O_3 V_1,1.5(H_2 O_1)

Compound Name: Oxo-(2,2'-(1,2-phenylenebis((nitrilo)methylylidene))diphenolato)-vanadium sesquihydrate

Space Group: P21/c ***Cell:*** ***a*** 19.892(0) ***b*** 6.881(0) ***c*** 27.651(0)
Space Group No.: 14 ***(Å,°)*** α 90.00 β 108.49(0) γ 90.00

R-Factor (%): 4.48 ***Temperature(K):*** 293 ***Density(g/cm³):*** 1.511

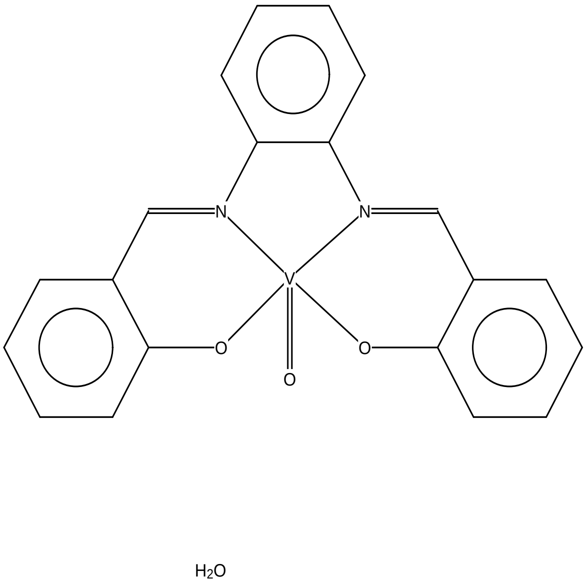

SELTIM

Reference: D.Christodoulou, M.G.Kanatzidis, D.Coucouvanis (1990) *Inorg.Chem.* ,**29,**191

Formula: C_{24} H_{18} Co_1 N_4 O_2

Compound Name: (2,3-Dioxo-5,6:13,14-dibenzo-9,10-(4',5'-dimethylbenzo)-1,4,8,11-tetra-azacyclotetradeca-7,11-diene)-cobalt(ii)

Space Group: P212121 ***Cell:*** ***a*** 6.877(1) ***b*** 11.945(10) ***c*** 23.062(9)
Space Group No.: 19 ***(Å,°)*** α 90.00 β 90.00 γ 90.00

R-Factor (%): 1.20 ***Temperature(K):*** 295 ***Density(g/cm³):*** 1.590

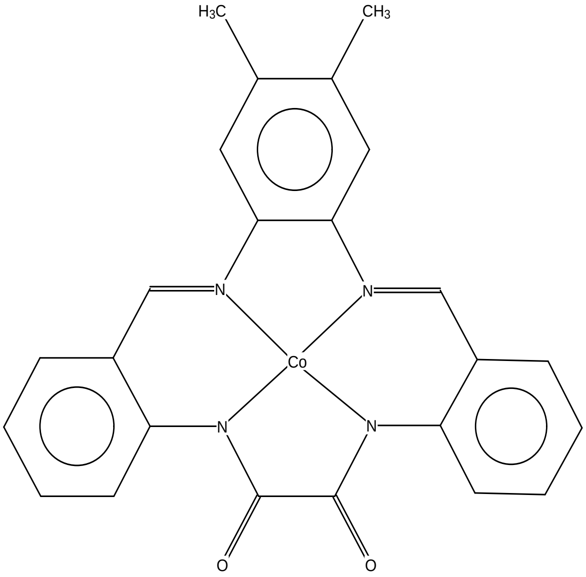

SERPUA

Reference: E.Solari, F.Corazza, C.Floriani, A.Chiesi-Villa, C.Guastini (1990) *J.Chem.Soc.,Dalton Trans.* ,1345

Formula: $C_{28} H_{30} Cl_2 Fe_2 N_2 O_4, C_4 H_8 O_1$

Compound Name: (μ_2-N,N'-o-Phenylene-bis(salicylideneiminato)-N,N',O,O,O',O')-di-chloro-bis(tetrahydrofuran)-di-iron tetrahydrofuran solvate

Space Group: P21/c ***Cell:*** ***a*** 12.882(2) ***b*** 11.940(2) ***c*** 21.701(3)
Space Group No.: 14 ***(Å,°)*** α 90.00 β 95.40(1) γ 90.00

R-Factor (%): 5.40 ***Temperature(K):*** 295 ***Density(g/cm3):*** 1.426

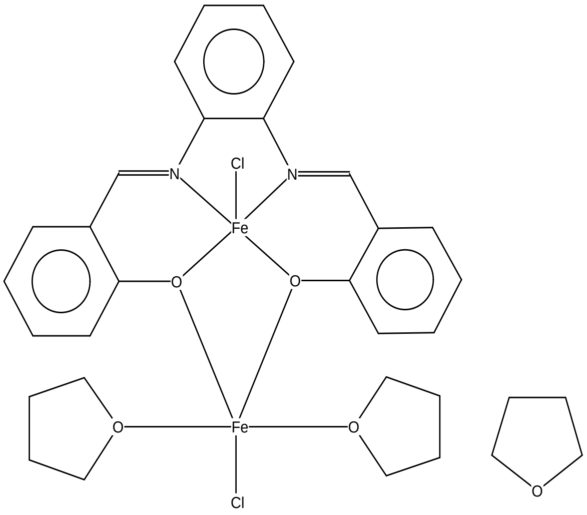

SERRUC

Reference: F.Corazza, E.Solari, C.Floriani, A.Chiesi-Villa, C.Guastini (1990) *J.Chem.Soc.,Dalton Trans.* ,1335

Formula: $C_{24} H_{22} Cl_2 N_2 O_3 Zr_1, 0.5(C_4 H_8 O_1)$

Compound Name: (N,N'-o-Phenylene-bis(salicylideneiminato)-N,N',O,O')-dichloro-tetrahydrofuran-zirconium tetrahydrofuran solvate

Space Group: P-1 ***Cell:*** ***a*** 12.759(2) ***b*** 13.332(2) ***c*** 7.587(1)
Space Group No.: 2 ***(Å,°)*** α 91.60(2) β 98.45(1) γ 85.30(1)

R-Factor (%): 3.30 ***Temperature(K):*** 295 ***Density(g/cm3):*** 1.526

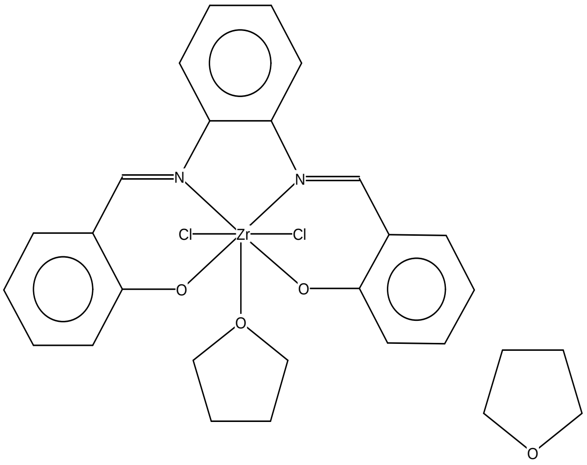

SERSAJ

Reference: F.Corazza, E.Solari, C.Floriani, A.Chiesi-Villa, C.Guastini (1990) *J.Chem.Soc.,Dalton Trans.* ,1335

Formula: $C_{24} H_{22} Cl_2 Hf_1 N_2 O_3, 0.5(C_4 H_8 O_1)$

Compound Name: (N,N'-o-Phenylene-bis(salicylideneiminato)-N,N',O,O')-dichloro-tetrahydrofuran-hafnium tetrahydrofuran solvate

Space Group: P-1 ***Cell:*** ***a*** 12.737(7) ***b*** 13.269(7) ***c*** 7.564(4)
Space Group No.: 2 ***(Å,°)*** α 91.48(1) β 98.56(1) γ 85.26(1)

R-Factor (%): 2.70 ***Temperature(K):*** 295 ***Density(g/cm3):*** 1.771

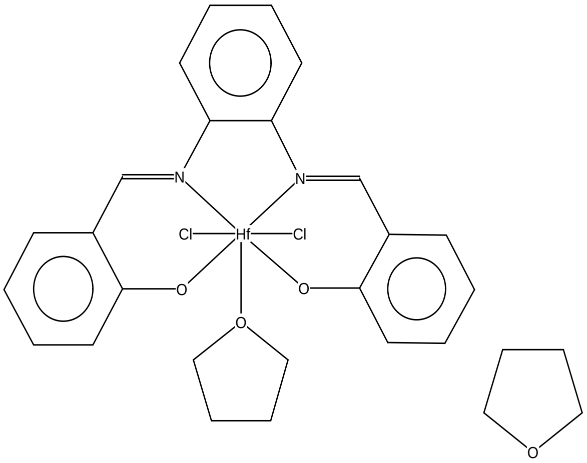

SISMAI

Reference: F.C.J.M.van Veggel, M.Bos, S.Harkema, H.van de Bovenkamp, W.Verboom, J.Reedijk, D.N.Reinhoudt (1991) *J.Org.Chem.* ,**56,**225

Formula: $C_{48} H_{42} Ba_1 N_4 Ni_2 O_{11}{}^{2+}, 2(C_1 F_3 O_3 S_1{}^{1-}), C_2 H_6 O_1 S_1$

Compound Name: (μ_3-13,14,16,17,38,39,41,42-Octahydro-7,11:19,23:32,36:44,48-tetramethenodibenzo(o,l$_1$)-(1,4,7,24,27,30,14,17,37,40)hexaoxatetra-azacyclohexatetracontine-51,52,53,54-tetraolato)-di-nickel-aqua-barium bis(trifluoromethanesulfonate) dimethyl sulfoxide solvate

Space Group: P-1 ***Cell:*** ***a*** 14.280(6) ***b*** 15.209(7) ***c*** 15.823(2)
Space Group No.: 2 ***(Å,°)*** α 67.86(2) β 71.16(2) γ 60.62(3)

R-Factor (%): 6.10 ***Temperature(K):*** 295 ***Density(g/cm3):*** 1.801

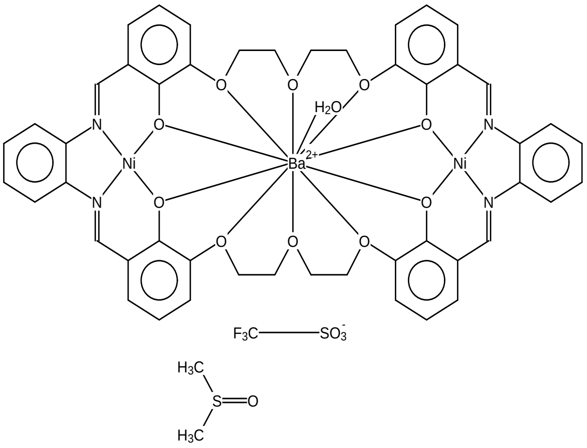

Search: search26 (Tue Dec 2 15:48:50 2014): Hits 557-560

SIYPOG

Reference: P.D.Frischmann, A.J.Gallant, J.H.Chong, M.J.MacLachlan (2008) *Inorg.Chem.* ,**47,**101

Formula: $C_{84} H_{102} N_6 O_{25} Zn_7,7.5(C_3 H_7 N_1 O_1)$

Compound Name: (μ_6-1^4,1^5,7^4,7^5,13^4,13^5-Hexa-neopentyloxy-4^2,4^3,10^2,10^3,16^2,16^3-hexaoxy-2,6,8,12,14,18-hexa-aza-1,7,13(1,2)tribenzena-4,10,16(1,4)tribenzena-cyclo-octadecaphane-2,5,8,11,14,17-hexaene)-(μ_4-oxo)-tris(μ_3-acetato-O,O,O')-tris(μ_2-acetato-O,O')-hepta-zinc(ii) dimethylformamide solvate

Space Group: P-1	***Cell:***	***a*** 16.144(1)	***b*** 19.997(1)	***c*** 20.212(1)
Space Group No.: 2	***(Å,°)***	α 108.28(0)	β 93.60(0)	γ 99.51(0)

R-Factor (%): 5.18 ***Temperature(K):*** 173 ***Density(g/cm³):*** 1.425

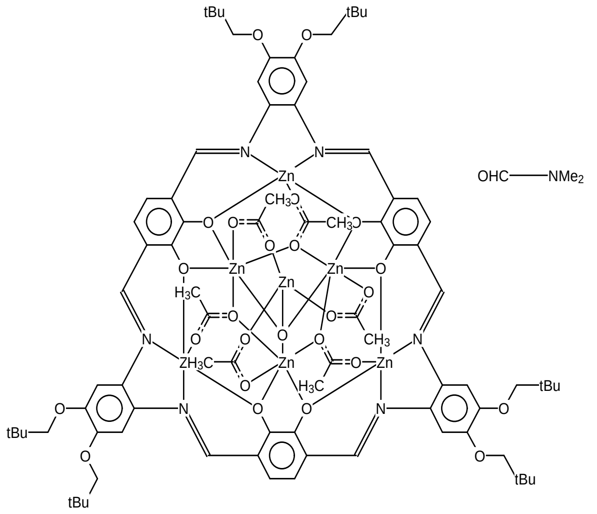

SIYPUM

Reference: P.D.Frischmann, A.J.Gallant, J.H.Chong, M.J.MacLachlan (2008) *Inorg.Chem.* ,**47,**101

Formula: $C_{76} H_{94} N_6 O_{18} Zn_4,2.5(C_3 H_7 N_1 O_1)$

Compound Name: (μ_4-1^4,1^5,7^4,7^5,13^4,13^5-Hexa-neopentyloxy-4^2,4^3,10^2,10^3,16^2,16^3-hexaoxy-2,6,8,12,14,18-hexa-aza-1,7,13(1,2)tribenzena-4,10,16(1,4)tribenzena-cyclo-octadecaphane-2,5,8,11,14,17-hexaene)-bis(μ_2-acetato-O,O')-diaqua-tetra-zinc(ii) dimethylformamide solvate

Space Group: P-1	***Cell:***	***a*** 14.910(1)	***b*** 20.137(1)	***c*** 21.238(1)
Space Group No.: 2	***(Å,°)***	α 62.14(0)	β 81.53(0)	γ 84.55(0)

R-Factor (%): 7.55 ***Temperature(K):*** 173 ***Density(g/cm³):*** 1.087

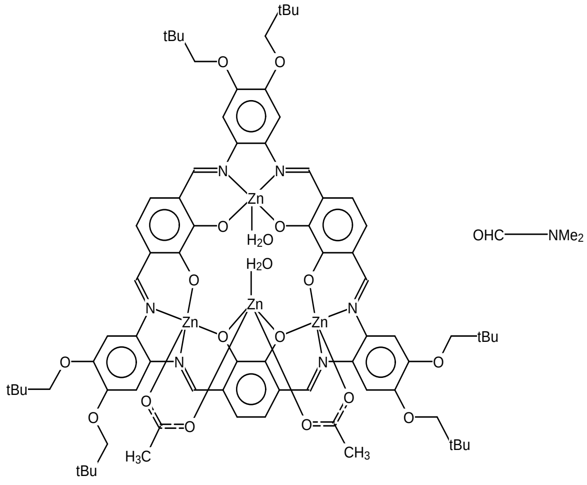

SIYQAT

Reference: P.D.Frischmann, A.J.Gallant, J.H.Chong, M.J.MacLachlan (2008) *Inorg.Chem.* ,**47,**101

Formula: $C_{64} H_{66} N_6 O_{18} S_1 Zn_4,x(C_2 H_6 O_1 S_1)$

Compound Name: (μ_4-1^4,1^5,7^4,7^5,13^4,13^5-Hexa-ethoxy-4^2,4^3,10^2,10^3,16^2,16^3-hexaoxy-2,6,8,12,14,18-hexa-aza-1,7,13(1,2)tribenzena-4,10,16(1,4)tribenzena-cyclo-octadecaphane-2,5,8,11,14,17-hexaene)-bis(μ_2-methacrylato-O,O')-aqua-dimethylsulfoxide-tetra-zinc(ii) dimethylsulfoxide solvate

Space Group: P-1	***Cell:***	***a*** 13.086(2)	***b*** 17.604(3)	***c*** 21.722(4)
Space Group No.: 2	***(Å,°)***	α 70.37(0)	β 81.47(0)	γ 70.96(0)

R-Factor (%): 6.42 ***Temperature(K):*** 173 ***Density(g/cm³):*** 1.178

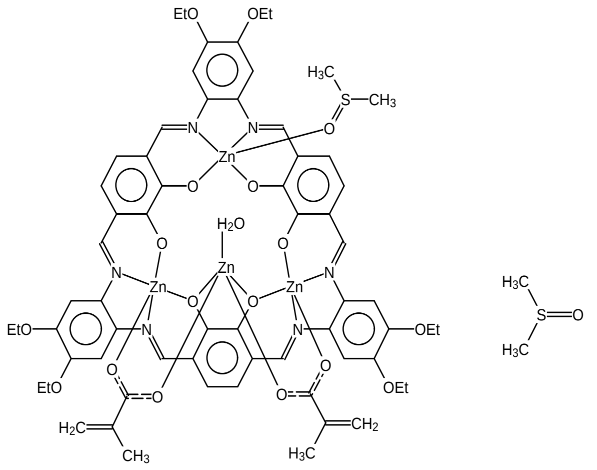

SIZNEV

Reference: Jin-Cai Wu, Shi-Xia Liu, T.D.Keene, A.Neels, V.Mereacre, A.K.Powell, S.Decurtins (2008) *Inorg.Chem.* ,**47,**3452

Formula: $C_{60} H_{52} Fe_2 N_4 O_5 S_{12},2(C_3 H_7 N_1 O_1)$

Compound Name: (μ_2-Oxo)-bis(2,2'-((2-(4,5-bis(n-propylsulfanyl)-1,3-dithiol-2-ylidene)-1,3-benzodithiole-5,6-diyl)bis(nitrilomethylidyne))diphenolato-N,N',O,O')-di-iron(iii) dimethylformamide solvate

Space Group: P-1	***Cell:***	***a*** 13.601(2)	***b*** 13.671(3)	***c*** 19.739(3)
Space Group No.: 2	***(Å,°)***	α 83.32(1)	β 71.90(1)	γ 79.77(1)

R-Factor (%): 7.10 ***Temperature(K):*** 173 ***Density(g/cm³):*** 1.504

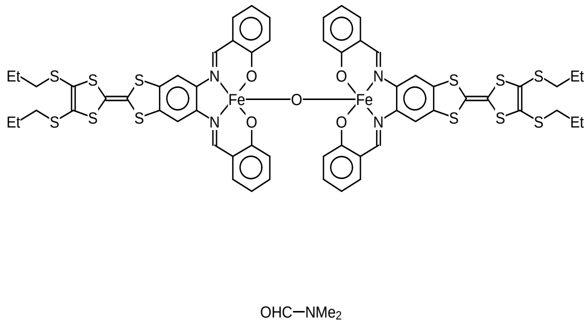

Search: search26 (Tue Dec 2 15:48:50 2014): Hits 561-564

SIZNIZ

Reference: Jin-Cai Wu, Shi-Xia Liu, T.D.Keene, A.Neels, V.Mereacre, A.K.Powell, S.Decurtins (2008) *Inorg.Chem.* ,**47,**3452

Formula: $C_{30} H_{26} N_2 Ni_1 O_2 S_6, C_3 H_7 N_1 O_1$

Compound Name: (2,2'-((2-(4,5-bis(n-Propylsulfanyl)-1,3-dithiol-2-ylidene)-1,3-benzodithiole-5,6-diyl)bis(nitrilomethylidyne))diphenolato-N,N',O,O')-nickel(ii) dimethylformamide solvate

Space Group:	P21/c	***Cell:***	***a*** 16.322(1)	***b*** 18.031(3)	***c*** 11.882(1)
Space Group No.:	14	***(Å,°)***	α 90.00	β 96.02(0)	γ 90.00

R-Factor (%): 6.39 ***Temperature(K):*** 173 ***Density(g/cm^3):*** 1.472

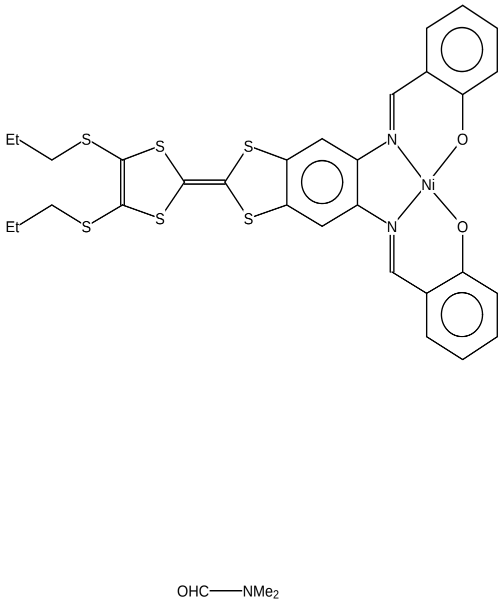

SIZNOF

Reference: Jin-Cai Wu, Shi-Xia Liu, T.D.Keene, A.Neels, V.Mereacre, A.K.Powell, S.Decurtins (2008) *Inorg.Chem.* ,**47,**3452

Formula: $C_{30} H_{26} Cu_1 N_2 O_2 S_6, 2(C_1 H_1 Cl_3)$

Compound Name: (2,2'-((2-(4,5-bis(n-Propylsulfanyl)-1,3-dithiol-2-ylidene)-1,3-benzodithiole-5,6-diyl)bis(nitrilomethylidyne))diphenolato-N,N',O,O')-copper(ii) chloroform solvate

Space Group:	Cc	***Cell:***	***a*** 39.121(6)	***b*** 5.337(0)	***c*** 20.584(3)
Space Group No.:	9	***(Å,°)***	α 90.00	β 118.43(1)	γ 90.00

R-Factor (%): 9.30 ***Temperature(K):*** 173 ***Density(g/cm^3):*** 1.654

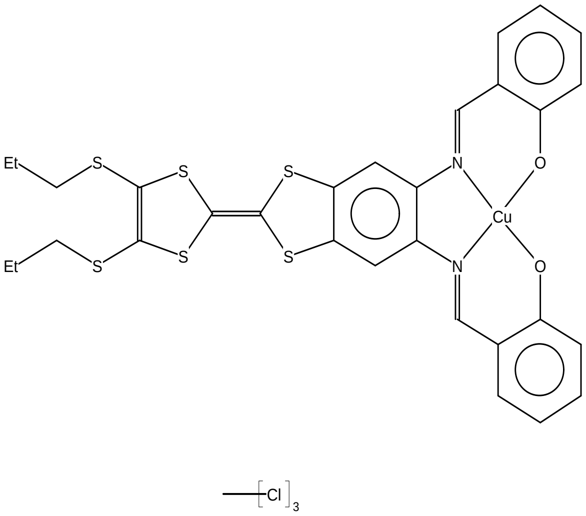

SOFQAG

Reference: A.Pietrangelo, B.C.Sih, B.N.Boden, Zhenwei Wang, Qifeng Li, K.C.Chou, M.J.MacLachlan, M.O.Wolf (2008) *Adv.Mater.* ,**20,** 2280

Formula: $C_{52} H_{66} N_2 Ni_1 O_4 S_2, 2(C_2 H_6 O_1)$

Compound Name: (2,2'-((4,5-bis(dodecyloxy)-1,2-phenylene)bis((nitrilo)methylylidene))-bis(4-(2-thienyl)phenolato))-nickel(ii) ethanol solvate

Space Group:	P-1	***Cell:***	***a*** 12.244(1)	***b*** 12.373(1)	***c*** 18.990(2)
Space Group No.:	2	***(Å,°)***	α 78.38(0)	β 81.87(0)	γ 73.95(0)

R-Factor (%): 5.40 ***Temperature(K):*** 173 ***Density(g/cm^3):*** 1.229

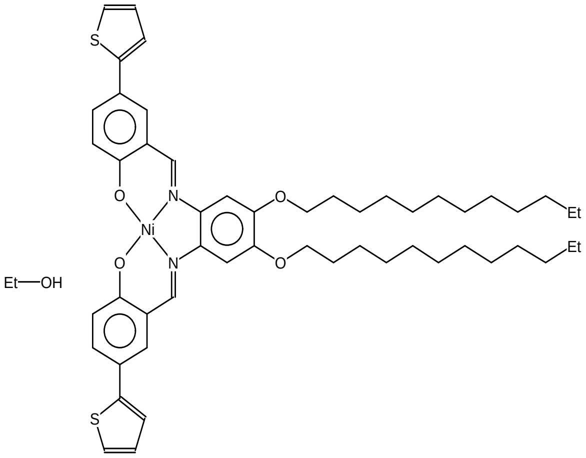

SOFQEK

Reference: A.Pietrangelo, B.C.Sih, B.N.Boden, Zhenwei Wang, Qifeng Li, K.C.Chou, M.J.MacLachlan, M.O.Wolf (2008) *Adv.Mater.* ,**20,** 2280

Formula: $C_{52} H_{66} Cu_1 N_2 O_4 S_2, C_1 H_4 O_1$

Compound Name: (2,2'-((4,5-bis(dodecyloxy)-1,2-phenylene)bis((nitrilo)methylylidene))-bis(4-(2-thienyl)phenolato))-copper(ii) methanol solvate

Space Group:	P21/c	***Cell:***	***a*** 24.632(3)	***b*** 21.587(2)	***c*** 9.652(1)
Space Group No.:	14	***(Å,°)***	α 90.00	β 98.85(0)	γ 90.00

R-Factor (%): 4.01 ***Temperature(K):*** 173 ***Density(g/cm^3):*** 1.235

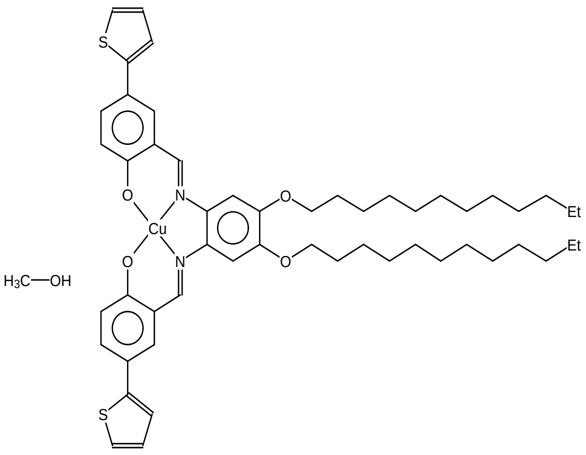

Search: search26 (Tue Dec 2 15:48:50 2014): Hits 565-568

SOFZAP

Reference: N.E.Eltayeb, S.G.Teoh, S.Chantrapromma, H.-K.Fun, R.Adnan (2008) *Acta Crystallogr.,Sect.E:Struct.Rep.Online* ,**64,**m912

Formula: $C_{80} H_{56} N_8 O_{20} Zn_8$,4($C_2 H_6 O_1 S_1$),2($H_2 O_1$)

Compound Name: tetrakis(μ_3-6,6'-Dioxido-2,2'-(1,2-phenylenebis(nitrilomethylidyne)) diphenolato)-tetra-aqua-octa-zinc(ii) dimethylsulfoxide solvate dihydrate

Space Group:	P42/n	***Cell:***	***a*** 18.132(0)	***b*** 18.132(0)	***c*** 13.381(0)
Space Group No.:	86	***(Å,°)***	α 90.00	β 90.00	γ 90.00

R-Factor (%): 4.98 ***Temperature(K):*** 100 ***Density(g/cm^3):*** 1.752

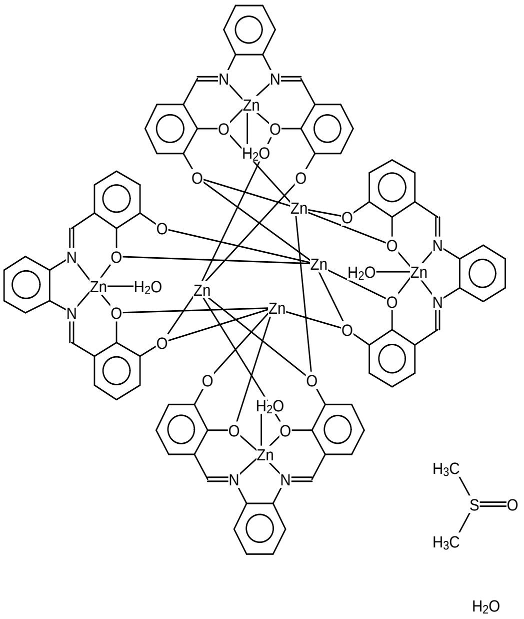

SOKQUE

Reference: T.Fukuda, F.Sakamoto, M.Sato, Y.Nakano, Xiang Shi Tan, Y.Fujii (1998) *Chem.Commun.* ,1391

Formula: $C_{21} H_{20} Mn_1 N_2 O_4$ $^{1+}$,$Cl_1 O_4$ $^{1-}$

Compound Name: Aqua-methanol-(N,N'-o-phenylene-bis(salicylidene-aminato))-manganese(iii) perchlorate

Synonym: Aqua-(benzene-1,2-diamine-N,N'-bis(salicylaldehydealdiminato))-(methanol)-manganese(iii) perchlorate

Space Group:	P21/c	***Cell:***	***a*** 11.804(2)	***b*** 13.435(2)	***c*** 14.358(2)
Space Group No.:	14	***(Å,°)***	α 90.00	β 109.11(0)	γ 90.00

R-Factor (%): 3.20 ***Temperature(K):*** 295 ***Density(g/cm^3):*** 1.602

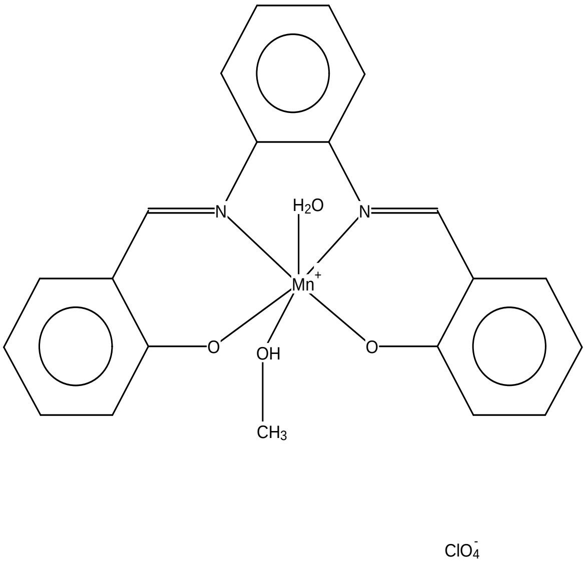

SOKQUE01

Reference: Li Jun, Yang Shu-Ming, Zhang Feng-Xiang, Tang Zong-Xun, Shi Qi-Zhen, Wu Qiang-Jin, Huang Zi-Xiang (2000) *Wuji Huaxue Xuebao(Chin.)(Chin.J.Inorg.Chem.)* ,**16,**84

Formula: $C_{21} H_{20} Mn_1 N_2 O_4$ $^{1+}$,$Cl_1 O_4$ $^{1-}$

Compound Name: Aqua-methanol-(N,N'-o-phenylene-bis(salicylidene-aminato))-manganese(iii) perchlorate

Synonym: Aqua-(benzene-1,2-diamine-N,N'-bis(salicylaldehydealdiminato))-(methanol)-manganese(iii) perchlorate

Space Group:	P21/n	***Cell:***	***a*** 11.748(7)	***b*** 13.985(7)	***c*** 13.538(4)
Space Group No.:	14	***(Å,°)***	α 90.00	β 92.63	γ 90.00

R-Factor (%): 5.50 ***Temperature(K):*** 295 ***Density(g/cm^3):*** 1.551

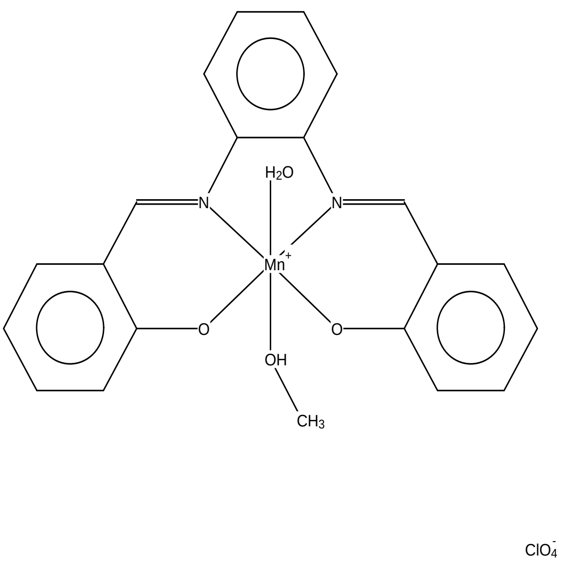

SONTEU

Reference: A.R.van Doorn, R.Schaafstra, M.Bos, S.Harkema, J.van Eerden, W.Verboom, D.N.Reinhoudt (1991) *J.Org.Chem.* ,**56,** 6083

Formula: $C_{35} H_{44} N_2 O_{13} U_1$,3($C_1 H_4 O_1$)

Compound Name: (9,10,12,13,15,16,18,19,21,22,24,25,27,28-Tetradecahydro-3,7:30,34-dimetheno-8,11,14,17,20,23,26,29,1,36-benzo-octaoxadiazacyclo-hexatriacontine-41,42-diolato)-(methanol-O)-dioxo-uranium methanol clathrate methanol solvate

Space Group:	P21/n	***Cell:***	***a*** 16.478(8)	***b*** 13.748(5)	***c*** 18.154(8)
Space Group No.:	14	***(Å,°)***	α 90.00	β 92.82(3)	γ 90.00

R-Factor (%): 5.80 ***Temperature(K):*** 148 ***Density(g/cm^3):*** 1.673

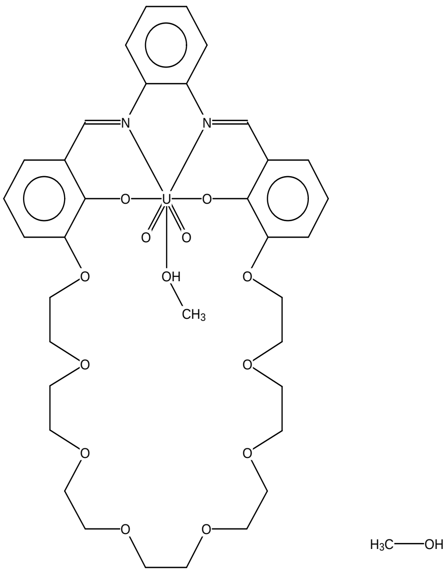

SONXOJ

Reference: L.San Felices, E.C.Escudero-Adan, J.Benet-Buchholz, A.W.Kleij (2009) *Inorg.Chem.* ,**48,**846

Formula: $C_{29} H_{32} N_2 O_9 Zn_2$

Compound Name: (μ_2-Acetato-1κO:2κO')-(μ_2-2-ethoxy-6-(((2-((3-(ethyloxy-1κO)-2-(hydroxy-2κO)benzylidene)amino-2κN)phenyl)imino-2κN)methyl) phenolato-2κO)-(acetato-1κ^{2}O,O')-methanol-di-zinc(ii)

Space Group:	P-1	***Cell:***	***a***	7.719(0)	***b***	13.374(0)	***c***	14.478(0)
Space Group No.:	2	***(Å,°)***	α	87.33(0)	β	77.49(0)	γ	78.70(0)

R-Factor (%): 5.32 ***Temperature(K):*** 100 ***Density(g/cm^3):*** 1.586

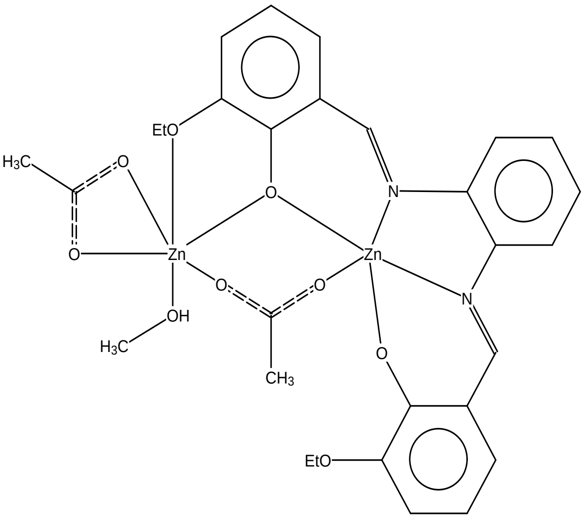

SONXUP

Reference: L.San Felices, E.C.Escudero-Adan, J.Benet-Buchholz, A.W.Kleij (2009) *Inorg.Chem.* ,**48,**846

Formula: $C_{24} H_{22} N_2 Ni_1 O_4$,2($C_1 H_1 Cl_3$),$H_2 O_1$

Compound Name: (2,2'-(1,2-Phenylenebis((nitrilo-κN)methylylidene))bis(6-ethoxyphenolato-κO))-nickel(ii) chloroform solvate monohydrate

Space Group:	P-1	***Cell:***	***a***	9.232(0)	***b***	11.610(0)	***c***	14.486(0)
Space Group No.:	2	***(Å,°)***	α	90.75(0)	β	95.53(0)	γ	107.26(0)

R-Factor (%): 3.06 ***Temperature(K):*** 100 ***Density(g/cm^3):*** 1.617

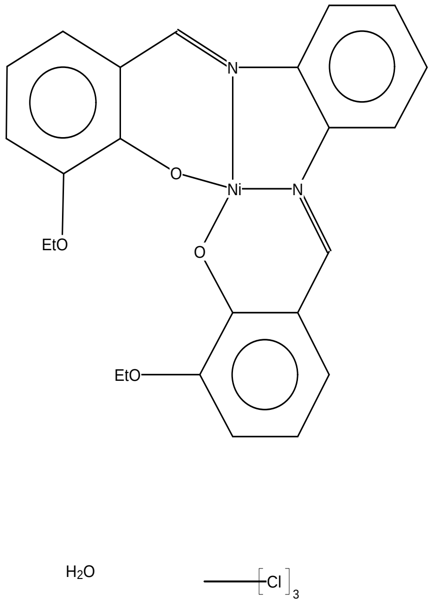

SOTLOD

Reference: S.J.Wezenberg, G.A.Metselaar, E.C.Escudero-Adan, J.Benet-Buchholz, A.W.Kleij (2009) *Inorg.Chim.Acta* ,**362,**1053

Formula: ($C_{41} H_{47} N_3 O_3 S_1 Zn_1$)n,2.5n($C_2 H_6 O_1 S_1$)

Compound Name: catena-[(μ_2-N-(3,5-Di-t-butylsalicylidene)-N'-(3-t-butyl-5-(2-(pyridin-4-yl) ethynyl)salicylidene)benzene-1,2-diamine-N,N',N'',O,O')-(dimethylsulfoxide-O)-zinc dimethylsulfoxide solvate]

Space Group:	P21/c	***Cell:***	***a***	18.313(1)	***b***	14.219(0)	***c***	20.303(1)
Space Group No.:	14	***(Å,°)***	α	90.00	β	112.67(0)	γ	90.00

R-Factor (%): 7.12 ***Temperature(K):*** 100 ***Density(g/cm^3):*** 1.256

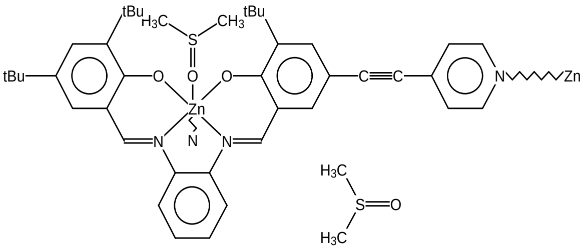

SUDJUW

Reference: A.Elmali, O.Atakol, I.Svoboda, H.Fuess (1993) *Z.Kristallogr.* ,**203,**275

Formula: $C_{40} H_{28} Fe_2 N_4 O_5$,0.5($C_4 H_8 O_2$)

Compound Name: (μ_2-Oxo)-bis(N,N'-o-phenylene-bis(salicylaldiminato))-iron(iii) dioxane solvate

Space Group:	P21/c	***Cell:***	***a***	14.650(2)	***b***	18.990(1)	***c***	13.296(1)
Space Group No.:	14	***(Å,°)***	α	90.00	β	76.08(0)	γ	90.00

R-Factor (%): 5.00 ***Temperature(K):*** 295 ***Density(g/cm^3):*** 1.481

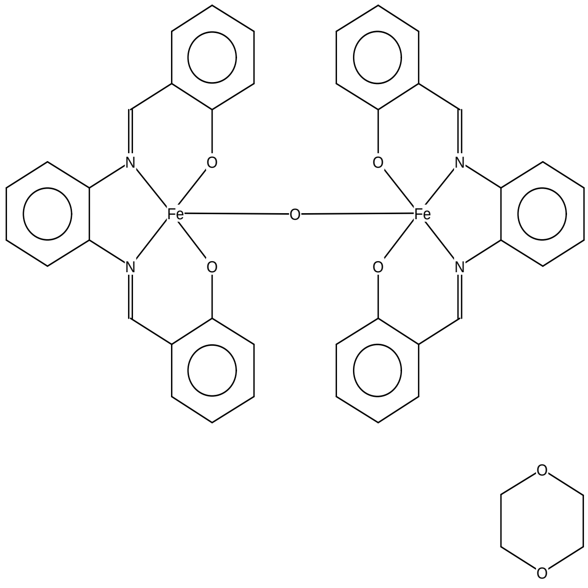

SUDJUW10

Reference: A.Elmali, Y.Elerman, I.Svoboda, H.Fuess, K.Griesar, W.Haase (1993) *Z.Naturforsch.,B:Chem.Sci.* ,**48,**313

Formula: $C_{40} H_{28} Fe_2 N_4 O_5,0.5(C_4 H_8 O_2)$

Compound Name: (μ_2-Oxo)-bis((N,N'-o-phenylene-bis(salicylaldiminato))-iron(iii)) 1,4-dioxane solvate

Space Group:	P21/c	***Cell:***	***a*** 14.650(2)	***b*** 18.990(1)	***c*** 13.296(1)		
Space Group No.:	14	***(Å,°)***	α 90.00	β 76.08(0)	γ 90.00		
R-Factor (%):	4.35	***Temperature(K):***	295	***Density(g/cm³):***	1.481		

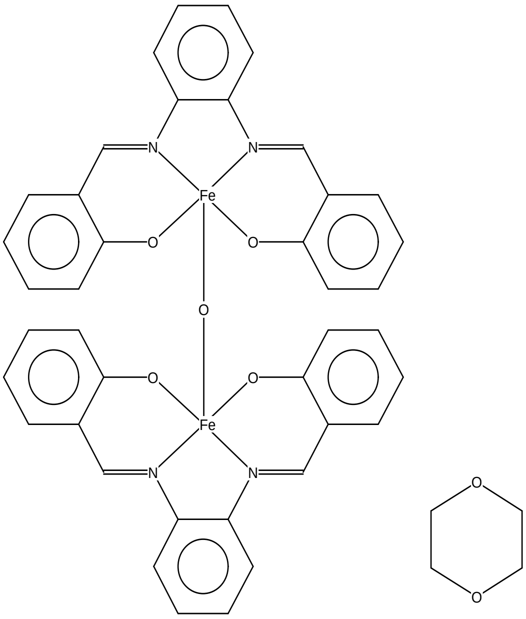

SUHHEJ

Reference: Hyounsoo Uh, P.D.Badger, S.J.Geib, S.Petoud (2009) *Helv.Chim.Acta* ,**92,**2313

Formula: $C_6 H_{16} N_1{}^{1+},C_{44} H_{28} Br_8 N_4 Nd_1 O_4{}^{1-}$

Compound Name: Triethylammonium bis(2,2'-((4,5-dimethylbenzene-1,2-diyl) bis(nitrilomethylylidene))bis(4,6-dibromophenolato)-N,N',O,O')-neodymium(iii)

Synonym: Triethylammonium bis(N,N'-(4,5-dimethyl-1,2-phenylene)-bis(4,6-dibromosalicyclideneiminato)-N,N',O,O')-neodymium(iii)

Space Group:	P21/n	***Cell:***	***a*** 8.261(0)	***b*** 14.164(0)	***c*** 46.845(1)		
Space Group No.:	14	***(Å,°)***	α 90.00	β 93.21(0)	γ 90.00		
R-Factor (%):	6.34	***Temperature(K):***	273	***Density(g/cm³):***	1.896		

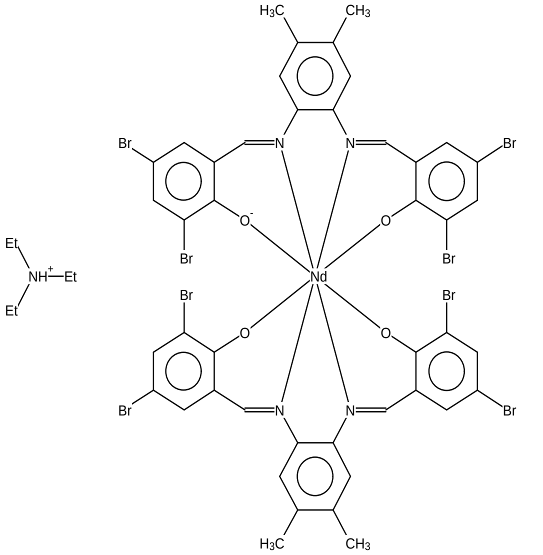

SUHHIN

Reference: Hyounsoo Uh, P.D.Badger, S.J.Geib, S.Petoud (2009) *Helv.Chim.Acta* ,**92,**2313

Formula: $C_{67} H_{58} N_6 Nd_2 O_7$

Compound Name: (μ_2-2,2'-((4,5-Dimethylbenzene-1,2-diyl)bis(nitrilomethylylidene)) diphenolato-N,N'O,O':O,O')-bis(2,2'-((4,5-dimethylbenzene-1,2-diyl) bis(nitrilomethylylidene))diphenolato-N,N',O,O')-(methanol-O)-di-neodymium(iii)

Synonym: (μ_2-N,N'-(4,5-Dimethyl-1,2-phenylene)-bis(salicyclideneiminato)-N,N'O, O':O,O')-bis(N,N'-(4,5-dimethyl-1,2-phenylene)-bis(salicyclideneiminato)-N,N',O,O')-(methanol-O)-di-neodymium(iii)

Space Group:	P21	***Cell:***	***a*** 14.297(1)	***b*** 25.028(3)	***c*** 17.742(1)		
Space Group No.:	4	***(Å,°)***	α 90.00	β 106.39(0)	γ 90.00		
R-Factor (%):	6.76	***Temperature(K):***	150	***Density(g/cm³):***	1.470		

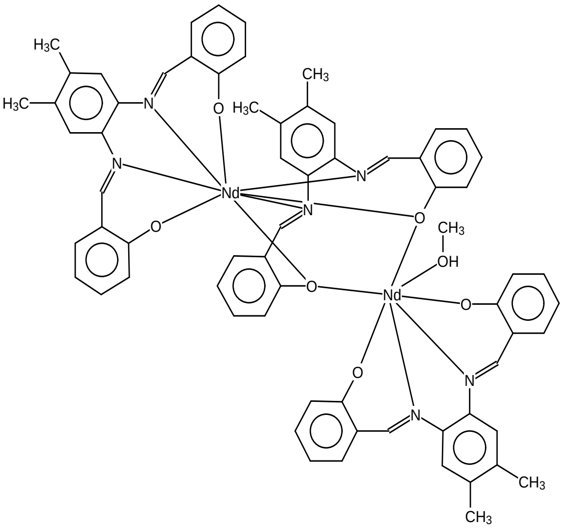

SUPDOX

Reference: J.Nishijo, S.Numao, K.Judai, N.Nishi (2009) *Polyhedron* , **28,**1664

Formula: $C_{40} H_{29} Mn_2 N_4 O_5{}^{1+},C_{12} H_8 Ni_1 S_4{}^{1-},2(C_2 H_3 N_1)$

Compound Name: (μ_2-Hydroxo)-bis(N,N'-bis(salicylidene)benzene-1,2-diamine-N,N',O,O')-di-manganese(iii) bis(benzene-1,2-dithiolato)-nickel(iii) acetonitrile solvate

Space Group:	C2/c	***Cell:***	***a*** 30.101(6)	***b*** 12.566(2)	***c*** 14.443(3)		
Space Group No.:	15	***(Å,°)***	α 90.00	β 105.67(0)	γ 90.00		
R-Factor (%):	7.40	***Temperature(K):***	296	***Density(g/cm³):***	1.486		

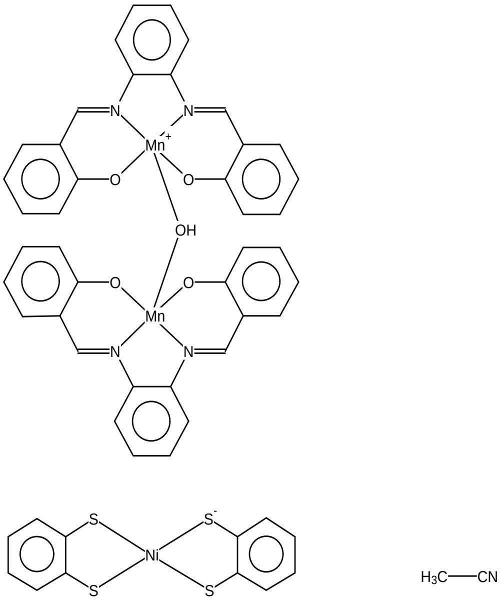

SUTKIB

Reference: A.L.Singer, D.A.Atwood (1998) *Inorg.Chim.Acta* ,**277,**157

Formula: C_{40} H_{54} N_2 O_3 Zn_1

Compound Name: (N,N'-Phenylene-bis(3,5-di-t-butylsalicylideneiminato))-tetrahydrofuran-zinc

Space Group:	P-1	***Cell:***	***a*** 11.399(2)	***b*** 13.128(2)	***c*** 13.184(2)		
Space Group No.:	2	***(Å,°)***	α 90.83(1)	β 104.60(10)	γ 90.60(1)		
R-Factor (%):	6.79	***Temperature(K):***	295	***Density(g/cm³):***	1.177		

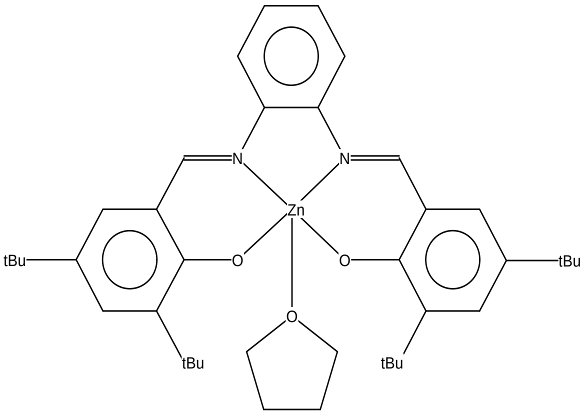

SUTKIB01

Reference: M.E.Germain, T.R.Vargo, P.G.Khalifah, M.J.Knapp (2007) *Inorg.Chem.* ,**46,**4422

Formula: C_{40} H_{54} N_2 O_3 Zn_1

Compound Name: (N,N'-bis(3,5-Di-t-butylsalicylideneimine)benzene)-tetrahydrofuran-zinc(ii)

Space Group:	P-1	***Cell:***	***a*** 10.960(0)	***b*** 13.067(0)	***c*** 13.154(0)		
Space Group No.:	2	***(Å,°)***	α 91.94(0)	β 104.09(0)	γ 92.05(0)		
R-Factor (%):	4.25	***Temperature(K):***	120	***Density(g/cm³):***	1.231		

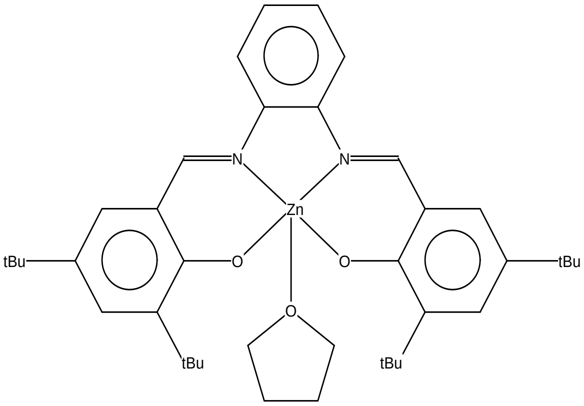

SUTKOH

Reference: A.L.Singer, D.A.Atwood (1998) *Inorg.Chim.Acta* ,**277,**157

Formula: C_{41} H_{51} N_3 O_2 Zn_1

Compound Name: (N,N'-Phenylene-bis(3,5-di-t-butylsalicylideneiminato))-pyridyl-zinc

Space Group:	Pbcm	***Cell:***	***a*** 15.612(0)	***b*** 10.551(0)	***c*** 23.248(1)		
Space Group No.:	57	***(Å,°)***	α 90.00	β 90.00	γ 90.00		
R-Factor (%):	6.57	***Temperature(K):***	295	***Density(g/cm³):***	1.185		

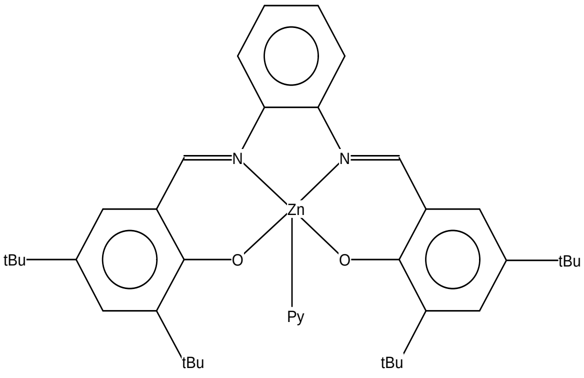

SUTKOH01

Reference: M.E.Germain, T.R.Vargo, P.G.Khalifah, M.J.Knapp (2007) *Inorg.Chem.* ,**46,**4422

Formula: C_{41} H_{51} N_3 O_2 Zn_1

Compound Name: (N,N'-bis(3,5-Di-t-butylsalicylideneimine)benzene)-pyridine-zinc(ii)

Space Group:	Pbcm	***Cell:***	***a*** 15.402(0)	***b*** 10.456(0)	***c*** 23.111(0)		
Space Group No.:	57	***(Å,°)***	α 90.00	β 90.00	γ 90.00		
R-Factor (%):	3.27	***Temperature(K):***	120	***Density(g/cm³):***	1.219		

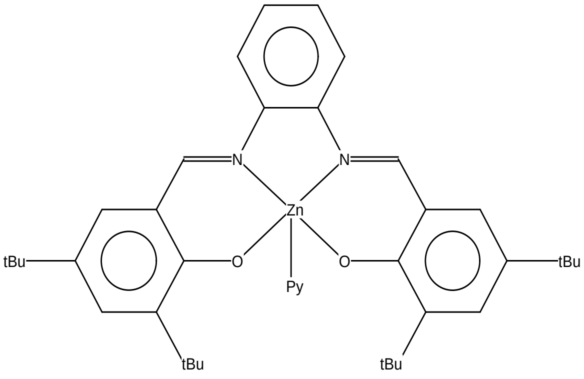

Search: search26 (Tue Dec 2 15:48:50 2014): Hits 581-584

SUWSOT

Reference: A.Decortes, M.M.Belmonte, J.Benet-Buchholz, A.W.Kleij (2010) *Chem.Commun.* ,**46,**4580

Formula: $C_{42}\ H_{58}\ N_2\ O_3\ Zn_1$

Compound Name: (Epoxyhexane-O)-(N,N'-bis(3,5-di-t-butylsalicylidene)-1,2-phenylenediamine)-zinc

Space Group: P21/c ***Cell:*** ***a*** 12.986(0) ***b*** 25.182(0) ***c*** 11.907(0)
Space Group No.: 14 ***(Å,°)*** α 90.00 β 94.09(0) γ 90.00

R-Factor (%): 5.27 ***Temperature(K):*** 100 ***Density(g/cm³):*** 1.205

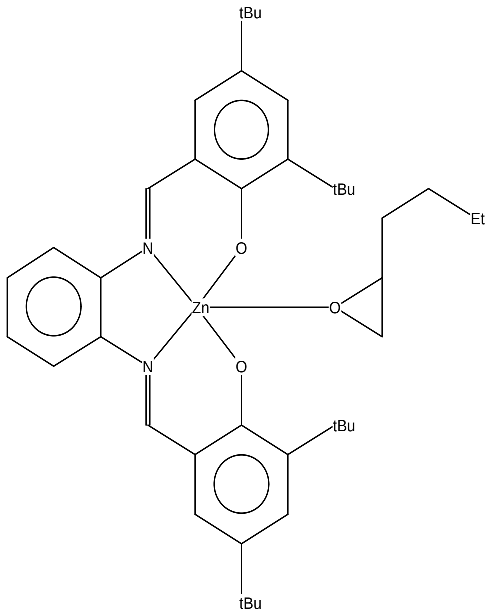

SUWSUZ

Reference: A.Decortes, M.M.Belmonte, J.Benet-Buchholz, A.W.Kleij (2010) *Chem.Commun.* ,**46,**4580

Formula: $C_{42}\ H_{56}\ N_2\ O_3\ Zn_1$

Compound Name: (7-Oxabicyclo[4.1.0]heptane)-(N,N'-bis(3,5-di-t-butylsalicylidene)-1,2-phenylenediamine)-zinc

Synonym: (Cyclohexane-oxide)-(N,N'-bis(3,5-di-t-butylsalicylidene)-1,2-phenylenediamine)-zinc

Space Group: P-1 ***Cell:*** ***a*** 12.020(1) ***b*** 12.974(1) ***c*** 13.129(1)
Space Group No.: 2 ***(Å,°)*** α 91.60(0) β 98.65(0) γ 103.73(0)

R-Factor (%): 6.01 ***Temperature(K):*** 296 ***Density(g/cm³):*** 1.189

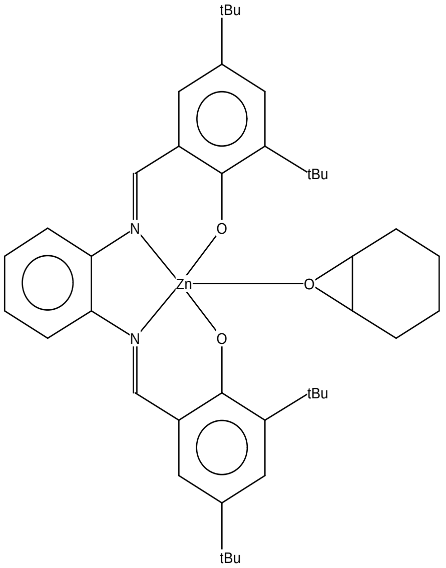

SUXWEO

Reference: Jia-Hao Yan, Xiao-Ping Shen, Hu Zhou (2010) *Acta Crystallogr.,Sect.E:Struct.Rep.Online* ,**66,**m1090

Formula: $C_{40}\ H_{28}\ Fe_2\ N_4\ O_5,C_1\ H_4\ O_1,2(H_2\ O_1)$

Compound Name: (μ_2-Oxido)-bis(2,2'-(o-phenylenebis(nitrilomethylidyne))diphenolato)-di-iron(iii) methanol solvate dihydrate

Synonym: (μ_2-Oxo)-bis(N,N'-bis(salicylidene)benzene-1,2-diamine-N,N',O,O')-di-iron(iii) methanol solvate dihydrate

Space Group: P-1 ***Cell:*** ***a*** 13.042(3) ***b*** 13.249(3) ***c*** 13.724(3)
Space Group No.: 2 ***(Å,°)*** α 116.60(3) β 110.50(3) γ 93.80(3)

R-Factor (%): 5.10 ***Temperature(K):*** 298 ***Density(g/cm³):*** 1.430

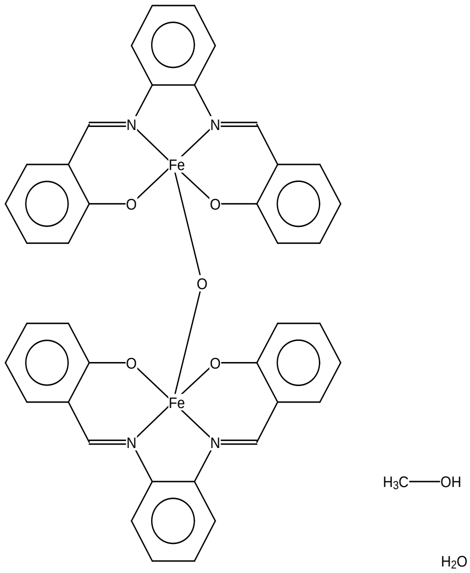

TAFGOY

Reference: S.J.Wezenberg, D.Anselmo, E.C.Escudero-Adan, J.Benet-Buchholz, A.W.Kleij (2010) *Eur.J.Inorg.Chem.* ,4611

Formula: $C_{54}\ H_{60}\ N_4\ O_8\ Zn_2\ ^{2-},2(C_{16}\ H_{36}\ N_1\ ^{1+}),1.5(C_2\ H_3\ N_1),H_2\ O_1$

Compound Name: bis(Tetra-n-butylammonium) (μ_2-1,2,4,5-tetrakis(3-t-butylsalicylideneamino)benzene)-bis(acetoxy)-di-zinc(ii) acetonitrile solvate monohydrate

Space Group: C2/c ***Cell:*** ***a*** 39.695(1) ***b*** 10.865(0) ***c*** 20.307(0)
Space Group No.: 15 ***(Å,°)*** α 90.00 β 91.97(0) γ 90.00

R-Factor (%): 5.81 ***Temperature(K):*** 100 ***Density(g/cm³):*** 1.205

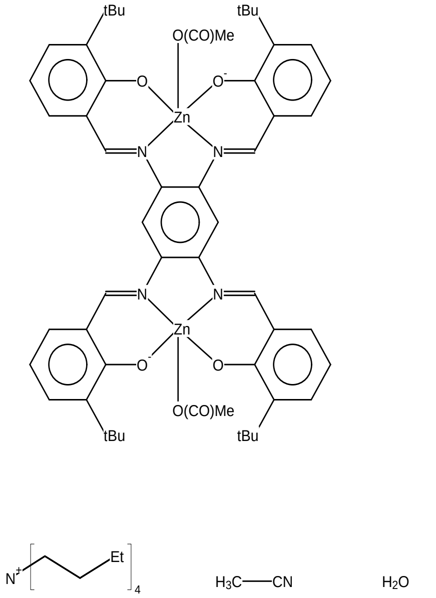

TATYOE

Reference: A.Kochem, O.Jarjayes, B.Baptiste, C.Philouze, H.Vezin, K.Tsukidate, F.Tani, M.Orio, Y.Shimatzaki, F.Thomas (2012) *Chem.-Eur.J.* ,**18,**1068

Formula: C_{38} H_{50} Cu_1 N_2 O_4 $^{1+}$,F_6 Sb_1 $^{1-}$,C_1 H_2 Cl_2

Compound Name: (2,2'-((4,5-Dimethoxy-1,2-phenylene)-bis((nitrilo-N)methylylidene))-bis(4,6-di-t-butylphenolato-O))-copper hexafluoroantimonate dichloromethane solvate

Space Group:	P-1	***Cell:***	***a*** 12.272(1)	***b*** 18.830(4)	***c*** 20.890(3)
Space Group No.:	2	***(Å,°)***	α 107.66(1)	β 101.35(0)	γ 101.70(1)

R-Factor (%): 4.07 ***Temperature(K):*** 200 ***Density(g/cm³):*** 1.509

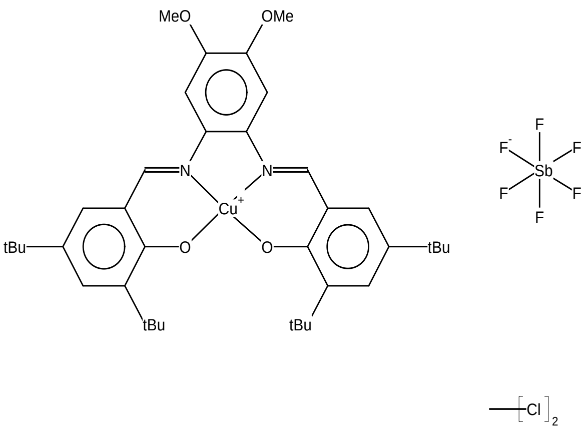

TATYUK

Reference: A.Kochem, O.Jarjayes, B.Baptiste, C.Philouze, H.Vezin, K.Tsukidate, F.Tani, M.Orio, Y.Shimatzaki, F.Thomas (2012) *Chem.-Eur.J.* ,**18,**1068

Formula: C_{38} H_{50} Cu_1 N_2 O_4

Compound Name: (2,2'-((4,5-Dimethoxy-1,2-phenylene)bis((nitrilo)methylylidene))-bis(4,6-di-t-butylphenolato))-copper

Space Group:	C2/c	***Cell:***	***a*** 29.993(4)	***b*** 19.415(5)	***c*** 13.786(4)
Space Group No.:	15	***(Å,°)***	α 90.00	β 117.21(1)	γ 90.00

R-Factor (%): 5.17 ***Temperature(K):*** 200 ***Density(g/cm³):*** 1.232

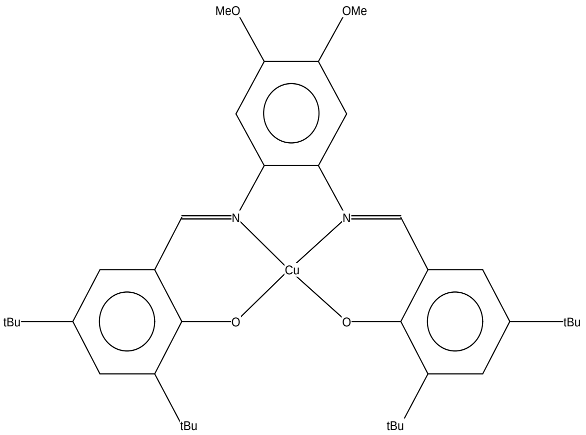

TATZAR

Reference: A.Kochem, O.Jarjayes, B.Baptiste, C.Philouze, H.Vezin, K.Tsukidate, F.Tani, M.Orio, Y.Shimatzaki, F.Thomas (2012) *Chem.-Eur.J.* ,**18,**1068

Formula: C_{30} H_{34} Cu_1 N_2 O_4

Compound Name: (2,2'-((1,2-phenylene)bis((nitrilo)methylylidene))-bis(4-methoxy-6-t-butylphenolato))-copper

Space Group:	P21/m	***Cell:***	***a*** 13.312(2)	***b*** 6.750(1)	***c*** 14.016(4)
Space Group No.:	11	***(Å,°)***	α 90.00	β 91.86(1)	γ 90.00

R-Factor (%): 4.16 ***Temperature(K):*** 200 ***Density(g/cm³):*** 1.451

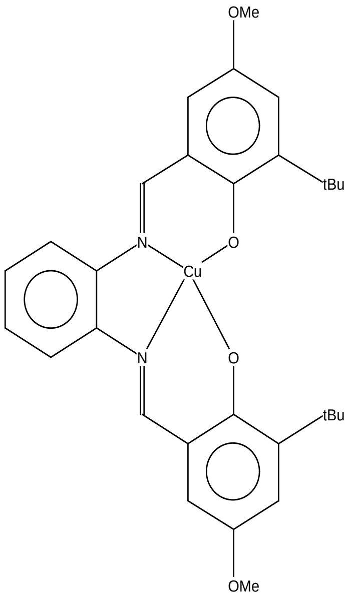

TEFKEV

Reference: A.W.Kleij, M.Kuil, M.Lutz, D.M.Tooke, A.L.Spek, P.C.J.Kamer, P.W.N.M.van Leeuwen, J.N.H.Reek (2006) *Inorg.Chim.Acta* ,**359,**1807

Formula: C_{37} H_{48} Cl_2 N_2 O_3 Zn_1,C_1 H_4 O_1

Compound Name: (N,N'-bis(3,5-Di-t-butylsalicylidene)-4,5-dichloro-1,2-phenylenediamine-N,N',O,O')-(methanol)-zinc(ii) methanol solvate

Space Group:	P-1	***Cell:***	***a*** 6.878(0)	***b*** 14.438(2)	***c*** 20.834(3)
Space Group No.:	2	***(Å,°)***	α 107.17(1)	β 99.27(1)	γ 94.07(0)

R-Factor (%): 4.95 ***Temperature(K):*** 150 ***Density(g/cm³):*** 1.265

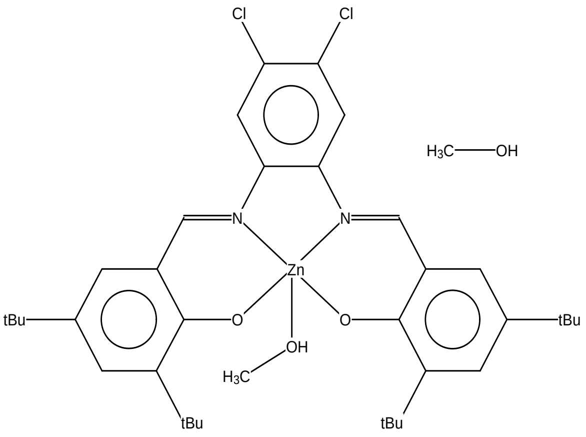

Search: search26 (Tue Dec 2 15:48:50 2014): Hits 589-592

TEFKIZ

Reference: A.W.Kleij, M.Kuil, M.Lutz, D.M.Tooke, A.L.Spek, P.C.J.Kamer, P.W.N.M.van Leeuwen, J.N.H.Reek (2006) *Inorg.Chim.Acta* ,**359,**1807

Formula: $C_{25} H_{15} Cl_4 N_3 O_2 Zn_1,C_2 H_3 N_1$

Compound Name: (N,N'-bis(3,5-Dichlorosalicylidene)-1,2-phenylenediamine-N,N',O,O')-(pyridine)-zinc(ii) acetonitrile solvate

Space Group: P-1 ***Cell:*** ***a*** 7.780(0) ***b*** 11.392(0) ***c*** 15.773(0)
Space Group No.: 2 ***(Å,°)*** α 108.22(0) β 96.29(0) γ 101.52(0)

R-Factor (%): 2.21 ***Temperature(K):*** 150 ***Density(g/cm^3):*** 1.656

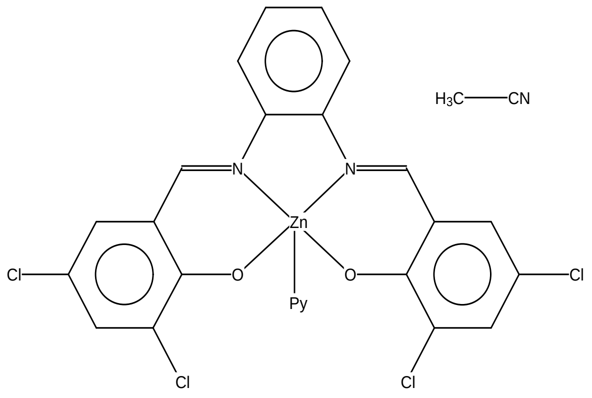

TEFKOF

Reference: A.W.Kleij, M.Kuil, M.Lutz, D.M.Tooke, A.L.Spek, P.C.J.Kamer, P.W.N.M.van Leeuwen, J.N.H.Reek (2006) *Inorg.Chim.Acta* ,**359,**1807

Formula: $C_{56} H_{56} Cl_4 N_4 O_4 Zn_2,3(C_2 H_3 N_1)$

Compound Name: bis(μ_2-N-(3,5-Di-t-butylsalicylidene)-N'-(3,5-dichlorosalicylidene)-1,2-phenylenediamine-N,N',O,O,O')-di-zinc(ii) acetonitrile solvate

Space Group: Pc ***Cell:*** ***a*** 12.311(0) ***b*** 24.448(0) ***c*** 11.167(0)
Space Group No.: 7 ***(Å,°)*** α 90.00 β 113.32(0) γ 90.00

R-Factor (%): 2.74 ***Temperature(K):*** 150 ***Density(g/cm^3):*** 1.339

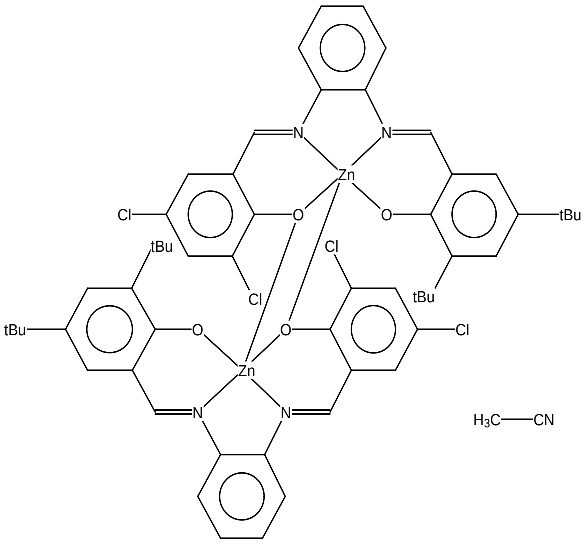

TEFKUL

Reference: A.W.Kleij, M.Kuil, M.Lutz, D.M.Tooke, A.L.Spek, P.C.J.Kamer, P.W.N.M.van Leeuwen, J.N.H.Reek (2006) *Inorg.Chim.Acta* ,**359,**1807

Formula: $C_{78} H_{100} Cl_4 N_6 O_4 Zn_2,C_2 H_3 N_1$

Compound Name: (μ_2-1,4-Diazabicyclo(2.2.2)octane-N,N')-bis(N,N'-bis(3,5-di-t-butylsalicylidene)-4,5-dichloro-1,2-phenylenediamine-N,N',O,O')-di-zinc(ii) acetonitrile solvate

Space Group: P-1 ***Cell:*** ***a*** 13.254(0) ***b*** 15.745(1) ***c*** 21.662(1)
Space Group No.: 2 ***(Å,°)*** α 83.80(0) β 87.32(0) γ 75.38(0)

R-Factor (%): 5.01 ***Temperature(K):*** 150 ***Density(g/cm^3):*** 1.145

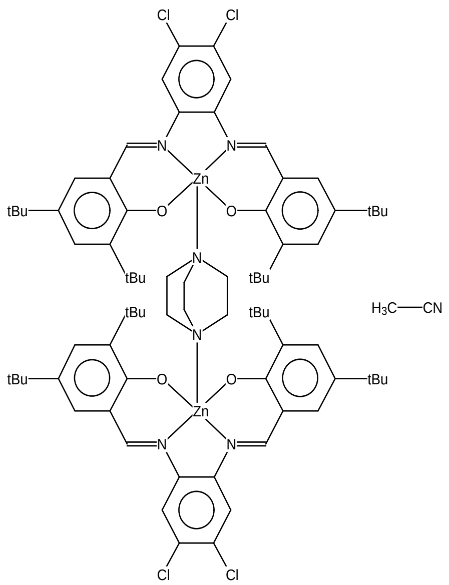

TEFLIA

Reference: A.W.Kleij, M.Kuil, M.Lutz, D.M.Tooke, A.L.Spek, P.C.J.Kamer, P.W.N.M.van Leeuwen, J.N.H.Reek (2006) *Private Communication* ,

Formula: $C_{30} H_{31} Cl_2 N_3 O_2 Zn_1$

Compound Name: (N,N'-bis(3-t-Butylsalicylidene)-4,5-dichloro-1,2-phenylenediamine-N,N',O,O')-(acetonitrile)-zinc(ii)

Space Group: P-1 ***Cell:*** ***a*** 6.873(0) ***b*** 13.729(0) ***c*** 15.542(0)
Space Group No.: 2 ***(Å,°)*** α 94.12(0) β 97.95(0) γ 102.18(0)

R-Factor (%): 3.24 ***Temperature(K):*** 150 ***Density(g/cm^3):*** 1.416

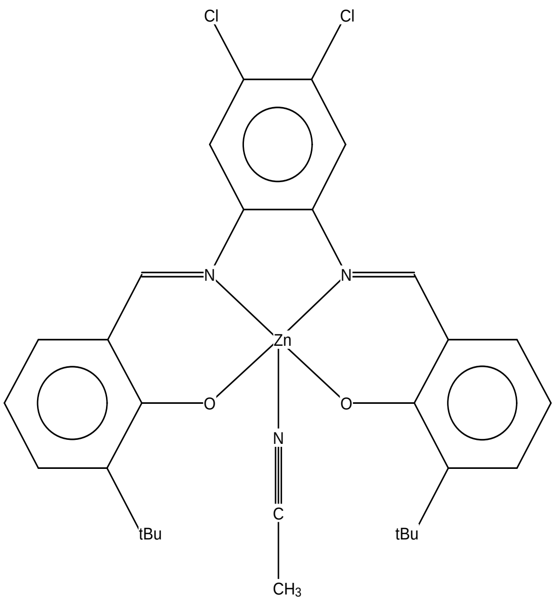

Search: search26 (Tue Dec 2 15:48:50 2014): Hits 593-596

TESPEO

Reference: Fang-Fang He, Hong-Qing Wang, Xiao-Feng Wang, Yu-Yuan Wang (2012) *Z.Kristallogr.-New Cryst.Struct.* ,**227,**521

Formula: C_{52} H_{44} N_4 O_4 Th_1

Compound Name: bis(2,2'-(1,2-Phenylenebis((nitrilo)methylylidene))bis(6-allylphenolato))-thorium(iv)

Space Group:	P-1	***Cell:***	***a*** 16.334(0)	***b*** 17.166(0)	***c*** 18.577(0)
Space Group No.:	2	***(Å,°)***	α 101.14(0)	β 95.20(0)	γ 117.05(0)

R-Factor (%): 3.14 ***Temperature(K):*** 296 ***Density(g/cm³):*** 1.522

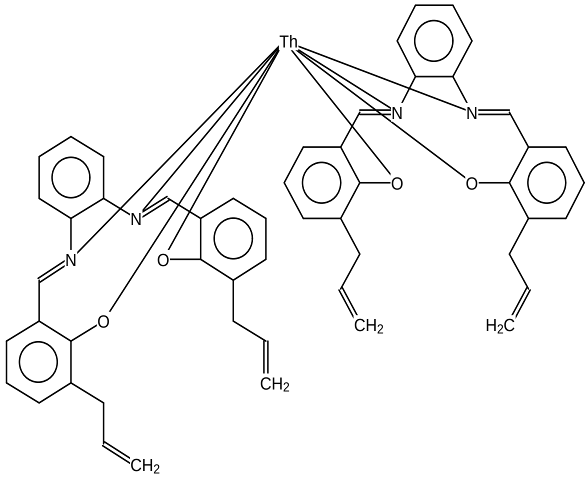

TEXTUM

Reference: L.Salmon, P.Thuery, M.Ephritikhine (2007) *Polyhedron* ,**26,** 645

Formula: C_{35} H_{27} Cl_2 Cu_1 N_5 O_4 U_1,2(C_5 H_5 N_1)

Compound Name: (μ_2-N,N'-bis(3-Hydroxysalicylidene)-1,2-benzenediamine)-dichloro-tris(pyridine)-copper(ii)-uranium(iv) pyridine solvate

Space Group:	P21/c	***Cell:***	***a*** 15.692(0)	***b*** 23.216(1)	***c*** 11.459(0)
Space Group No.:	14	***(Å,°)***	α 90.00	β 93.03(0)	γ 90.00

R-Factor (%): 3.89 ***Temperature(K):*** 100 ***Density(g/cm³):*** 1.772

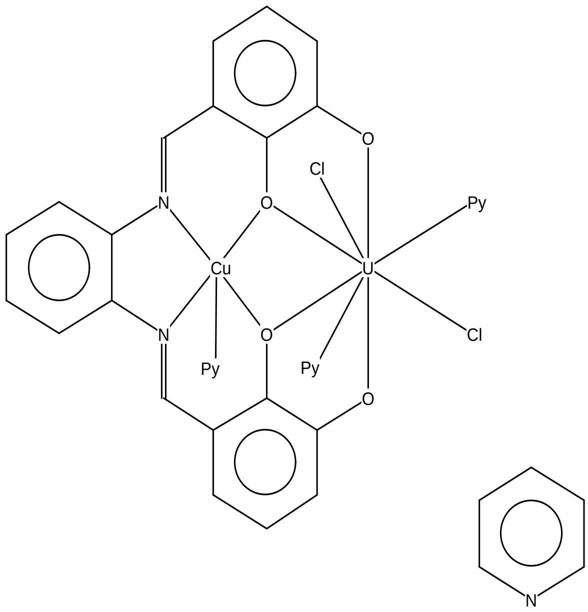

TINFEC

Reference: Xiaoping Yang, B.P.Hahn, R.A.Jones, Wai-Kwok Wong, K.J.Stevenson (2007) *Inorg.Chem.* ,**46,**7050

Formula: C_{46} H_{52} Br_4 Cl_2 Eu_4 N_4 O_{18} $^{2+}$,2(Cl_1 $^{1-}$),6(C_1 H_4 O_1)

Compound Name: bis(μ_3-Hydroxo)-bis(μ_2-N,N'-bis(5-bromo-3-methoxysalicylidene) phenylene-1,2-diamine-N,N',O,O,O',O',O'',O''')-bis(μ_2-chloro)-bis(methanol)-dihydroxy-tetra-aqua-tetra-europium(iii) dichloride methanol solvate

Space Group:	P21/c	***Cell:***	***a*** 15.491(3)	***b*** 19.933(4)	***c*** 15.115(3)
Space Group No.:	14	***(Å,°)***	α 90.00	β 114.09(3)	γ 90.00

R-Factor (%): 9.08 ***Temperature(K):*** 153 ***Density(g/cm³):*** 1.723

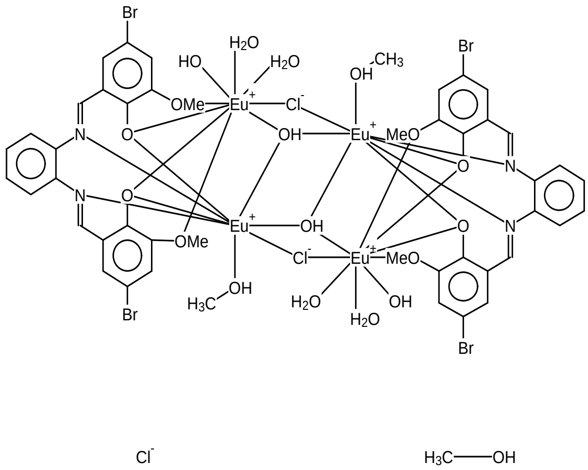

TINFIG

Reference: Xiaoping Yang, B.P.Hahn, R.A.Jones, Wai-Kwok Wong, K.J.Stevenson (2007) *Inorg.Chem.* ,**46,**7050

Formula: C_{116} H_{120} Br_8 Cl_8 Eu_8 N_8 O_{36} $^{4+}$,4(Cl_1 $^{1-}$),2(C_1 H_4 O_1),14(H_2 O_1)

Compound Name: (bis(μ_4-1,4-Benzenedicarboxylato-O,O,O',O'',O'',O''')-tetrakis(μ_2-N,N'-bis(5-bromo-3-methoxysalicylidene)phenylene-1,2-diamine-N,N',O,O,O',O',O'',O''')-tetrakis(μ_2-chloro)-dodecakis(methanol)-tetrachloro-octa-europium(iii)) tetrachloride methanol solvate tetradecahydrate

Space Group:	C2/c	***Cell:***	***a*** 21.184(4)	***b*** 32.694(7)	***c*** 32.822(7)
Space Group No.:	15	***(Å,°)***	α 90.00	β 95.79(3)	γ 90.00

R-Factor (%): 8.76 ***Temperature(K):*** 153 ***Density(g/cm³):*** 1.409

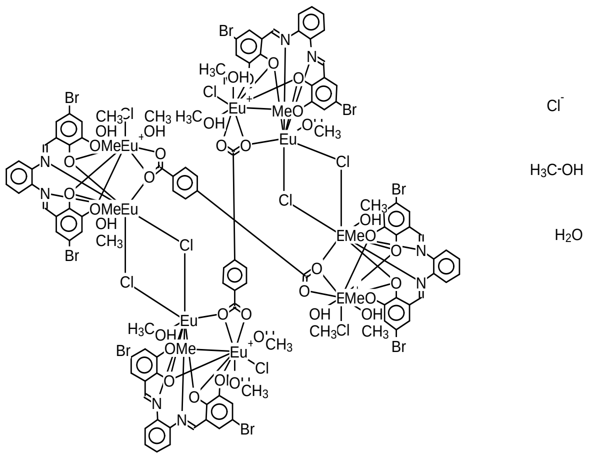

TIPHEG

Reference: B.Lassalle-Kaiser, R.Guillot, A.Aukauloo (2007) *Tetrahedron Lett.* ,**48,**7004

Formula: $2(C_{24}H_{20}P_1{}^{1+}),C_{24}H_{12}N_4Ni_2O_8{}^{2-},7(H_2O_1)$

Compound Name: bis(Tetraphenylphosphonium) (μ_2-N,N'-(4,5-bis(salicylideneamino)-1,2-phenylene)-bis(oxamato))-di-nickel(ii) heptahydrate

Space Group:	P21/n	***Cell:***	***a*** 16.345(2)	***b*** 18.780(1)	***c*** 21.253(3)
Space Group No.:	14	***(Å,°)***	α 90.00	β 95.75(0)	γ 90.00

R-Factor (%): 7.94 ***Temperature(K):*** 100 ***Density(g/cm^3):*** 1.439

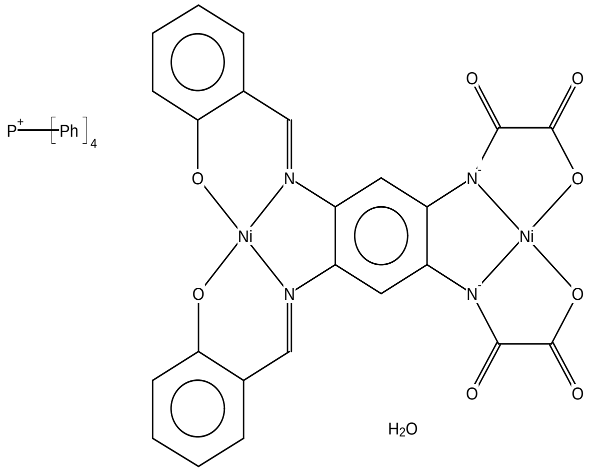

TIYPOG

Reference: S.M.Malinak, D.Coucouvanis (1996) *Inorg.Chem.* ,**35,**4810

Formula: $2(C_{16}H_{36}N_1{}^{1+}),C_{48}H_{40}Mo_2N_8Ni_2O_9{}^{2-}$

Compound Name: bis(Tetra-n-butylammonium) bis(μ_3-1,5,8,12-tetra-aza-(3,4-c) (9,10-c)di-p-tolyl-(13,14-d)catecholato-cyclotetradeca-1,11-diene)-(μ_2-oxo)-tetraoxo-di-molybdenum-di-nickel

Space Group:	P-1	***Cell:***	***a*** 12.324(3)	***b*** 17.740(4)	***c*** 20.920(4)
Space Group No.:	2	***(Å,°)***	α 108.79(3)	β 98.20(3)	γ 103.12(3)

R-Factor (%): 9.67 ***Temperature(K):*** 295 ***Density(g/cm^3):*** 1.350

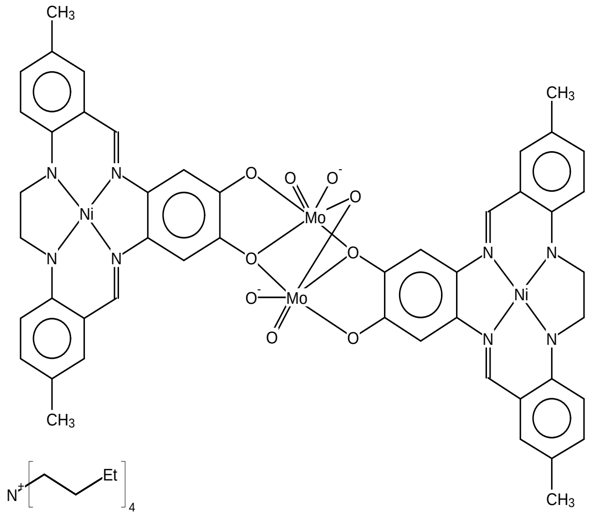

TIYPOG01

Reference: S.M.Malinak, D.T.Rosa, D.Coucouvanis (1998) *Inorg.Chem.* ,**37,**1175

Formula: $C_{48}H_{40}Mo_2N_8Ni_2O_9{}^{2-},2(C_{16}H_{36}N_1{}^{1+})$

Compound Name: bis(Tetra-n-butylammonium) bis(μ_3-1,5,8,12-tetra-aza-(3,4-c) (9,10-c)di-p-tolyl-(13,14-d)catecholato-cyclotetradeca-1,11-diene)-(μ_2-oxo)-tetraoxo-di-molybdenum-di-nickel

Space Group:	P-1	***Cell:***	***a*** 12.324(3)	***b*** 17.740(4)	***c*** 20.920(4)
Space Group No.:	2	***(Å,°)***	α 108.79(3)	β 98.20(3)	γ 103.12(3)

R-Factor (%): 9.67 ***Temperature(K):*** 295 ***Density(g/cm^3):*** 1.350

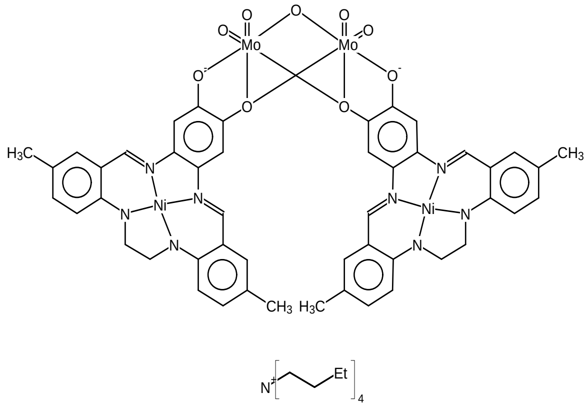

TIYPUM

Reference: S.M.Malinak, D.Coucouvanis (1996) *Inorg.Chem.* ,**35,**4810

Formula: $2(C_{16}H_{36}N_1{}^{1+}),C_{48}H_{40}Cu_2Mo_2N_4O_{17}{}^{2-}$

Compound Name: bis(Tetra-n-butylammonium) bis(μ_3-(4,5-bis(3-ethoxy-salicylideneamino) catecholato))-(μ_2-oxo)-tetraoxo-di-molybdenum-di-copper(ii)

Space Group:	P21/c	***Cell:***	***a*** 20.821(4)	***b*** 23.133(5)	***c*** 20.056(4)
Space Group No.:	14	***(Å,°)***	α 90.00	β 117.71(3)	γ 90.00

R-Factor (%): 9.95 ***Temperature(K):*** 295 ***Density(g/cm^3):*** 1.358

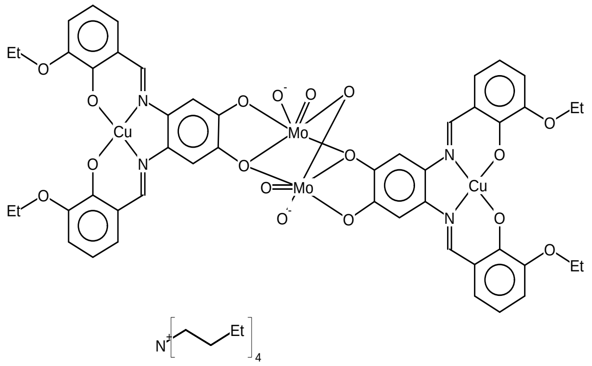

Search: search26 (Tue Dec 2 15:48:50 2014): Hits 601-604

TIYPUM01

Reference: S.M.Malinak, D.T.Rosa, D.Coucouvanis (1998) *Inorg.Chem.* ,**37,**1175

Formula: $C_{48}H_{40}Cu_2Mo_2N_4O_{17}{}^{2-}$,2($C_{16}H_{36}N_1{}^{1+}$)

Compound Name: bis(Tetra-n-butylammonium) bis(μ_3-(4,5-bis(3-ethoxy-salicylideneamino) catecholato))-(μ_2-oxo)-tetraoxo-di-molybdenum-di-copper(ii)

Space Group:	P21/c	***Cell:***	***a***	20.821(4)	***b***	23.133(5)	***c***	20.056(4)
Space Group No.:	14	***(Å,°)***	α	90.00	β	117.71(3)	γ	90.00

R-Factor (%): 9.50 ***Temperature(K):*** 295 ***Density(g/cm^3):*** 1.358

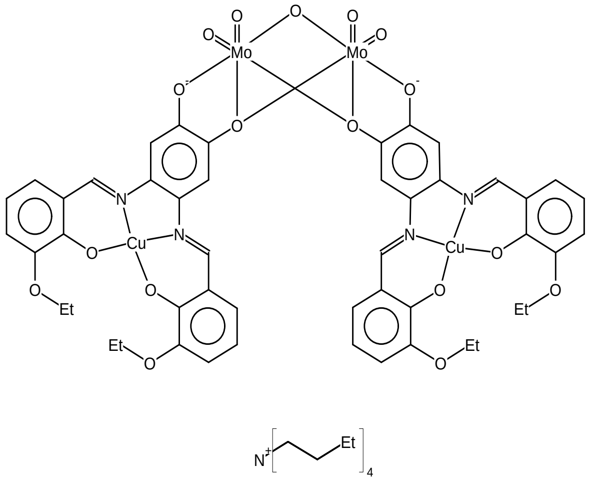

TOPREV

Reference: E.Gallo, E.Solari, N.Re, C.Floriani, A.Chiesi-Villa, C.Rizzoli (1996) *Angew.Chem.,Int.Ed.* ,**35,**1981

Formula: ($C_{72}H_{92}Mn_2N_4O_6$)n,2n(C_6H_{14})

Compound Name: catena-((μ_2-Hydroxo)-(μ_2-N,N'-o-phenylene-(3,5-di-t-butylsalicylidenimine)-(4,6-di-t-butylphenolato-2-formamido))-(N,N'-o-phenylene-bis(3,5-di-t-butylsalicylidenimine))-di-manganese(iii) n-hexane solvate)

Space Group:	P21/c	***Cell:***	***a***	17.395(4)	***b***	13.083(3)	***c***	34.904(6)
Space Group No.:	14	***(Å,°)***	α	90.00	β	103.61(2)	γ	90.00

R-Factor (%): 7.30 ***Temperature(K):*** 173 ***Density(g/cm^3):*** 1.197

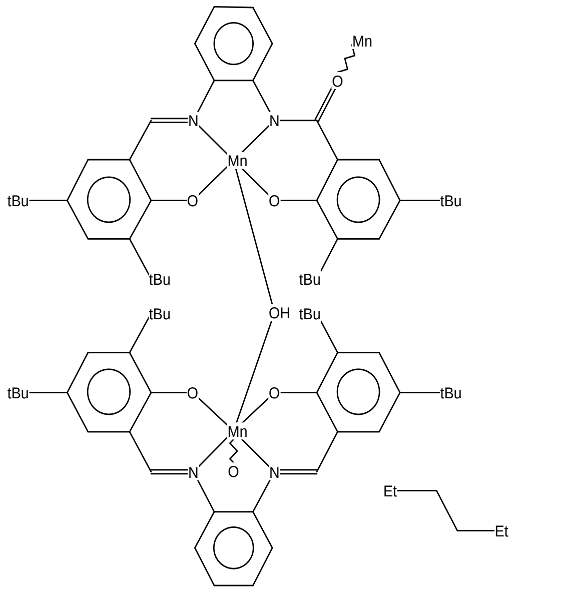

TORSOI

Reference: H.Torayama, T.Nishide, H.Asada, M.Fujiwara, T.Matsushita (1996) *Chem.Lett.* ,387

Formula: $C_{40}H_{28}Mn_2N_4O_6$,2($C_2H_3N_1$)

Compound Name: bis((μ_2-Oxo)-(N,N'-disalicylidene-1,2-diaminobenzene))-di-manganese(iv) acetonitrile solvate

Space Group:	P21/n	***Cell:***	***a***	8.937(2)	***b***	12.382(2)	***c***	17.736(2)
Space Group No.:	14	***(Å,°)***	α	90.00	β	95.98(1)	γ	90.00

R-Factor (%): 4.40 ***Temperature(K):*** 295 ***Density(g/cm^3):*** 1.451

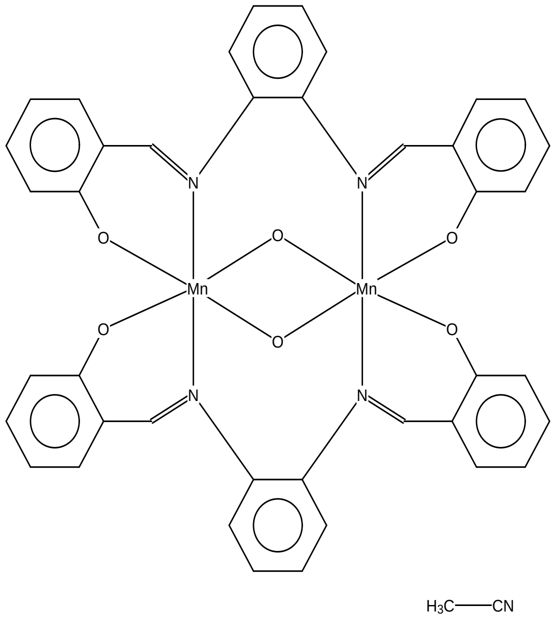

TORSOI01

Reference: H.Torayama, T.Nishide, H.Asada, M.Fujiwara, T.Matsushita (1998) *Polyhedron* ,**17,**105

Formula: $C_{40}H_{28}Mn_2N_4O_6$,2($C_2H_3N_1$)

Compound Name: bis((μ_2-N,N'-Disalicylidene-1,2-diaminobenzene-N,N',O,O')-(μ_2-oxo)-manganese(iv)) acetonitrile solvate

Space Group:	P21/n	***Cell:***	***a***	8.937(2)	***b***	12.382(2)	***c***	17.736(2)
Space Group No.:	14	***(Å,°)***	α	90.00	β	95.98(1)	γ	90.00

R-Factor (%): 4.80 ***Temperature(K):*** 295 ***Density(g/cm^3):*** 1.451

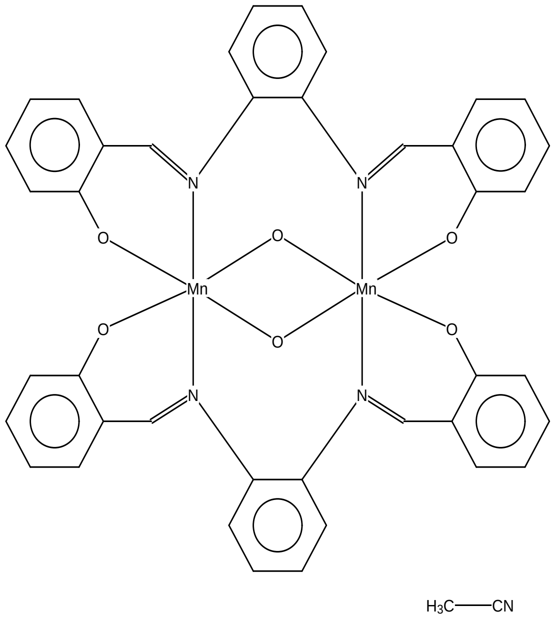

TUKLUG

Reference: E.Suresh, M.M.Bhadbhade, D.Srinivas (1996) *Polyhedron* , **15,**4133

Formula: C_{20} H_{12} Cl_2 Cu_1 N_2 O_2

Compound Name: (o-Phenylenebis(5-chlorosalicylidenaminato))-copper(ii)

Space Group: P21/c ***Cell:*** ***a*** 11.960(20) ***b*** 8.030(10) ***c*** 18.280(10)
Space Group No.: 14 ***(Å,°)*** α 90.00 β 105.70(2) γ 90.00

R-Factor (%): 0.00 ***Temperature(K):*** 295 ***Density(g/cm^3):*** 1.756

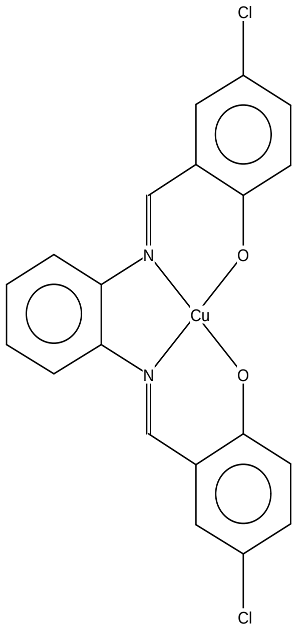

TUVFAS

Reference: Sun Hwa Lee, Li Xu, Byeong Kwon Park, Y.V.Mironov, Soo Hyun Kim, Young Ju Song, Cheal Kim, Youngmee Kim, Sung-Jin Kim (2010) *Chem.-Eur.J.* ,**16,**4678

Formula: C_{92} H_{64} Mn_4 N_{20} O_{12} Re_4 Te_4,8(H_2 O_1),4(C_1 H_4 O_1)

Compound Name: tetrakis(μ_3-tellurido)-tetrakis(μ_2-cyano)-tetrakis(2,2'-(1,2-phenylenebis(nitrilomethylylidene))diphenolato)-tetraaqua-octacyano-tetra-manganese-tetra-rhenium methanol solvate octahydrate

Space Group: P42/n ***Cell:*** ***a*** 19.220(9) ***b*** 19.220(9) ***c*** 14.420(14)
Space Group No.: 86 ***(Å,°)*** α 90.00 β 90.00 γ 90.00

R-Factor (%): 6.37 ***Temperature(K):*** 273 ***Density(g/cm^3):*** 2.113

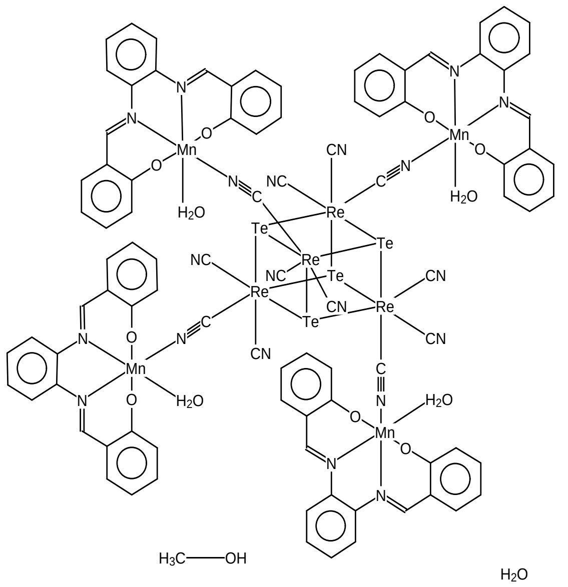

TUZJOO

Reference: J.K.-H.Hui, M.J.MacLachlan (2010) *Dalton Trans.* ,**39,**7310

Formula: C_{54} H_{58} N_4 O_{10} S_2 Zn_2,4(C_2 H_6 O_1 S_1),2(H_2 O_1)

Compound Name: (μ_2-1,8-bis(4,5-bis((2-oxybenzylidene)amino)-2-methoxyphenoxy) octane)-bis(dimethylsulfoxide)-di-zinc dimethylsulfoxide solvate dihydrate

Space Group: P21/n ***Cell:*** ***a*** 9.395(5) ***b*** 17.839(5) ***c*** 20.303(5)
Space Group No.: 14 ***(Å,°)*** α 90.00 β 95.38(0) γ 90.00

R-Factor (%): 6.67 ***Temperature(K):*** 173 ***Density(g/cm^3):*** 1.438

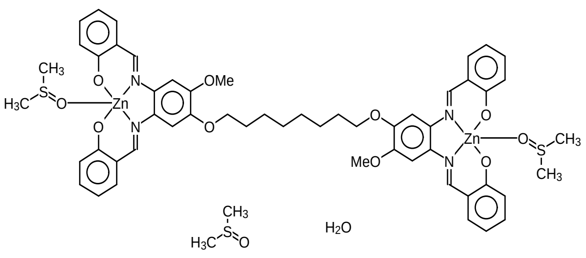

UCICUG

Reference: G.Cosquer, F.Pointillart, Y.Le Gal, S.Golhen, O.Cador, L.Ouahab (2011) *Chem.-Eur.J.* ,**17,**12502

Formula: C_{45} H_{29} Cu_1 Dy_1 F_{18} N_2 O_8 S_6

Compound Name: (μ_2-,2'-((2-(4,5-Bis(propylsulfanyl)-1,3-dithiol-2-ylidene)-1,3-benzodithiole-5,6-diyl)bis(nitrilomethylylidene))diphenolato)-tris(1,1,1,5,5,5-hexafluoro-2,4-pentanedionato-O,O')-copper-dysprosium

Space Group: P-1 ***Cell:*** ***a*** 12.637(5) ***b*** 13.692(5) ***c*** 17.564(5)
Space Group No.: 2 ***(Å,°)*** α 87.16(0) β 88.22(0) γ 66.89(0)

R-Factor (%): 6.15 ***Temperature(K):*** 293 ***Density(g/cm^3):*** 1.768

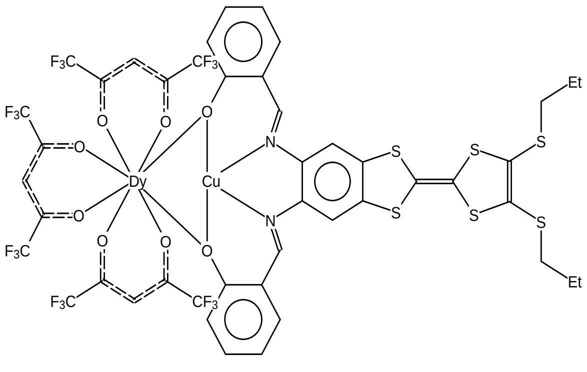

UCIDAN

Reference: G.Cosquer, F.Pointillart, Y.Le Gal, S.Golhen, O.Cador, L.Ouahab (2011) *Chem.-Eur.J.* ,**17,**12502

Formula: C_{45} H_{29} Dy_1 F_{18} N_2 Ni_1 O_8 S_6

Compound Name: (μ_2-,2'-((2-(4,5-Bis(propylsulfanyl)-1,3-dithiol-2-ylidene)-1,3-benzodithiole-5,6-diyl)bis(nitrilomethylylidene))diphenolato)-tris(1,1,1,5,5,5-hexafluoro-2,4-pentanedionato-O,O')-nickel-dysprosium

Space Group:	P-1	***Cell:***	***a***	13.295(0)	***b***	13.306(0)	***c***	16.506(1)
Space Group No.:	2	***(Å,°)***	α	77.62(0)	β	85.38(0)	γ	84.33(0)

R-Factor (%): 4.69 ***Temperature(K):*** 293 ***Density(g/cm³):*** 1.737

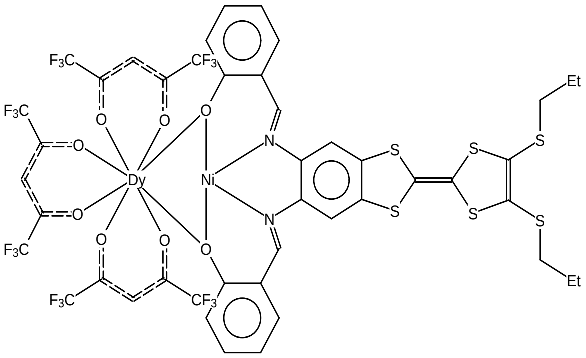

UCIDER

Reference: G.Cosquer, F.Pointillart, Y.Le Gal, S.Golhen, O.Cador, L.Ouahab (2011) *Chem.-Eur.J.* ,**17,**12502

Formula: C_{45} H_{29} Cu_1 F_{18} Gd_1 N_2 O_8 S_6

Compound Name: (μ_2-,2'-((2-(4,5-Bis(propylsulfanyl)-1,3-dithiol-2-ylidene)-1,3-benzodithiole-5,6-diyl)bis(nitrilomethylylidene))diphenolato)-tris(1,1,1,5,5,5-hexafluoro-2,4-pentanedionato-O,O')-copper-gadolinium

Space Group:	P-1	***Cell:***	***a***	12.678(1)	***b***	13.734(2)	***c***	17.559(2)
Space Group No.:	2	***(Å,°)***	α	86.81(0)	β	88.04(0)	γ	66.69(0)

R-Factor (%): 6.05 ***Temperature(K):*** 293 ***Density(g/cm³):*** 1.754

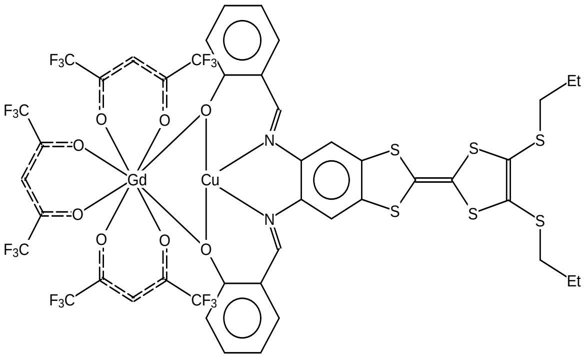

UCIDIV

Reference: G.Cosquer, F.Pointillart, Y.Le Gal, S.Golhen, O.Cador, L.Ouahab (2011) *Chem.-Eur.J.* ,**17,**12502

Formula: C_{45} H_{29} Cu_1 F_{18} N_2 O_8 S_6 Tb_1

Compound Name: (μ_2-,2'-((2-(4,5-Bis(propylsulfanyl)-1,3-dithiol-2-ylidene)-1,3-benzodithiole-5,6-diyl)bis(nitrilomethylylidene))diphenolato)-tris(1,1,1,5,5,5-hexafluoro-2,4-pentanedionato-O,O')-copper-terbium

Space Group:	P-1	***Cell:***	***a***	12.651(5)	***b***	13.715(5)	***c***	17.563(5)
Space Group No.:	2	***(Å,°)***	α	87.13(0)	β	88.23(0)	γ	66.81(0)

R-Factor (%): 5.35 ***Temperature(K):*** 293 ***Density(g/cm³):*** 1.760

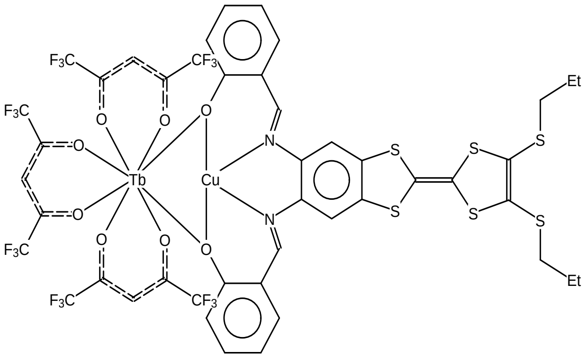

UCIDOB

Reference: G.Cosquer, F.Pointillart, Y.Le Gal, S.Golhen, O.Cador, L.Ouahab (2011) *Chem.-Eur.J.* ,**17,**12502

Formula: C_{45} H_{29} F_{18} N_2 Ni_1 O_8 S_6 Tb_1

Compound Name: (μ_2-,2'-((2-(4,5-Bis(propylsulfanyl)-1,3-dithiol-2-ylidene)-1,3-benzodithiole-5,6-diyl)bis(nitrilomethylylidene))diphenolato)-tris(1,1,1,5,5,5-hexafluoro-2,4-pentanedionato-O,O')-nickel-terbium

Space Group:	P-1	***Cell:***	***a***	13.277(0)	***b***	13.331(0)	***c***	16.513(0)
Space Group No.:	2	***(Å,°)***	α	77.51(0)	β	85.31(0)	γ	84.33(0)

R-Factor (%): 5.43 ***Temperature(K):*** 293 ***Density(g/cm³):*** 1.731

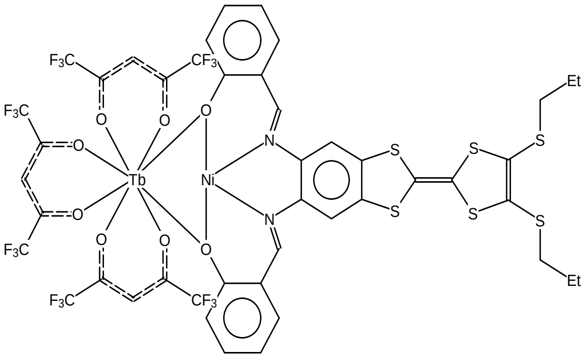

UCUDAY

Reference: A.C.W.Leung, J.H.Chong, B.O.Patrick, M.J.MacLachlan (2003) *Macromolecules* ,**36,**5051

Formula: C_{44} H_{60} I_2 N_2 Ni_1 O_4,C_1 H_4 O_1

Compound Name: (N,N'-(4,5-bis(dodecyloxy)-1,2-phenylene)-bis(5-iodosalicylaldiminato)-N,N',O,O')-nickel(ii) methanol solvate

Space Group: P21/a ***Cell:*** ***a*** 21.248(1) ***b*** 8.943(0) ***c*** 25.480(1)
Space Group No.: 14 ***(Å,°)*** α 90.00 β 111.59(0) γ 90.00

R-Factor (%): 3.77 ***Temperature(K):*** 173 ***Density(g/cm³):*** 1.513

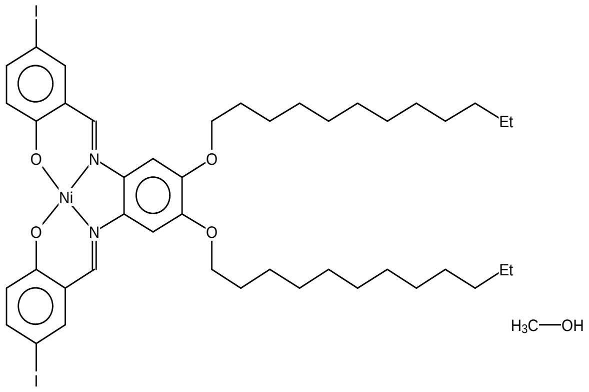

UDUGEG

Reference: M.Kuil, P.E.Goudriaan, A.W.Kleij, D.M.Tooke, A.L.Spek, P.W.N.M.van Leeuwen, J.N.H.Reek (2007) *Dalton Trans.* ,2311

Formula: C_{101} H_{117} Cl_1 N_6 O_4 P_2 Pd_1 Zn_2

Compound Name: (μ_2-1,2,4,5-tetrakis(N-(4,6-Di-t-butylsalicylidenealdiminato))benzene)-bis(μ_2-diphenyl(4-pyridyl)phosphine-N,P)-chloro-methyl-palladium-di-zinc(ii) unknown solvate

Space Group: C2/c ***Cell:*** ***a*** 32.664(1) ***b*** 12.443(1) ***c*** 25.374(1)
Space Group No.: 15 ***(Å,°)*** α 90.00 β 97.31(0) γ 90.00

R-Factor (%): 8.59 ***Temperature(K):*** 150 ***Density(g/cm³):*** 1.178

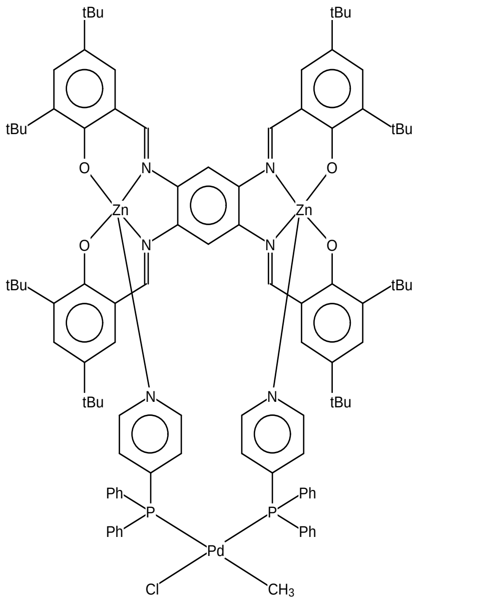

UDUGIK

Reference: M.Kuil, P.E.Goudriaan, A.W.Kleij, D.M.Tooke, A.L.Spek, P.W.N.M.van Leeuwen, J.N.H.Reek (2007) *Dalton Trans.* ,2311

Formula: C_{100} H_{114} Cl_2 N_6 O_4 P_2 Pt_1 Zn_2

Compound Name: (μ_2-1,2,4,5-tetrakis(N-(4,6-Di-t-butylsalicylidenealdiminato))benzene)-bis(μ_2-diphenyl(3-pyridyl)phosphine-N,P)-dichloro-platinum-di-zinc(ii) unknown solvate

Space Group: P-1 ***Cell:*** ***a*** 17.390(2) ***b*** 18.134(1) ***c*** 19.658(1)
Space Group No.: 2 ***(Å,°)*** α 115.30(0) β 96.58(1) γ 92.18(0)

R-Factor (%): 3.47 ***Temperature(K):*** 150 ***Density(g/cm³):*** 1.152

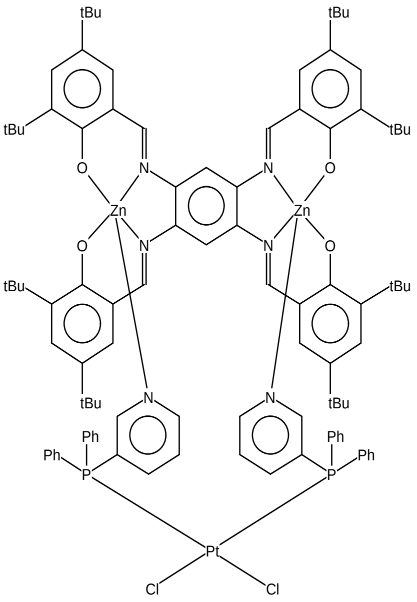

UDUGOQ

Reference: M.Kuil, P.E.Goudriaan, A.W.Kleij, D.M.Tooke, A.L.Spek, P.W.N.M.van Leeuwen, J.N.H.Reek (2007) *Dalton Trans.* ,2311

Formula: C_{100} H_{114} N_6 O_4 P_2 Zn_2,0.8(C_1 H_2 Cl_2)

Compound Name: (μ_2-1,2,4,5-tetrakis(N-(4,6-Di-t-butylsalicylidenealdiminato))benzene)-bis(3-(diphenylphosphino)pyridine-N)-di-zinc(ii) dichloromethane unknown solvate

Space Group: P-1 ***Cell:*** ***a*** 16.799(2) ***b*** 18.411(3) ***c*** 20.429(1)
Space Group No.: 2 ***(Å,°)*** α 110.62(0) β 107.51(1) γ 96.56(1)

R-Factor (%): 5.15 ***Temperature(K):*** 150 ***Density(g/cm³):*** 1.048

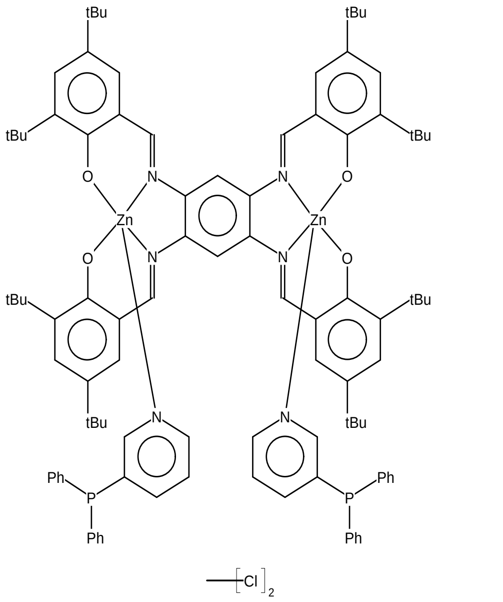

UFADEL

Reference: Rui-Ting Xue, Mei-Ju Niu, Jian-Min Dou, Da-Qi Wang (2007) *Acta Crystallogr.,Sect.E:Struct.Rep.Online* ,**63,**m2111

Formula: $C_{28}\ H_{18}\ Cu_1\ N_2\ O_2$

Compound Name: (1,1'-(o-Phenylenebis(nitrilomethylidyne))di-2-naphtholato)-copper(ii)

Space Group:	P21/n	***Cell:***	***a***	16.019(13)	***b***	7.764(6)	***c***	16.334(14)
Space Group No.:	14	***(Å,°)***	α	90.00	β	97.04(1)	γ	90.00

R-Factor (%): 5.73 ***Temperature(K):*** 298 ***Density(g/cm^3):*** 1.575

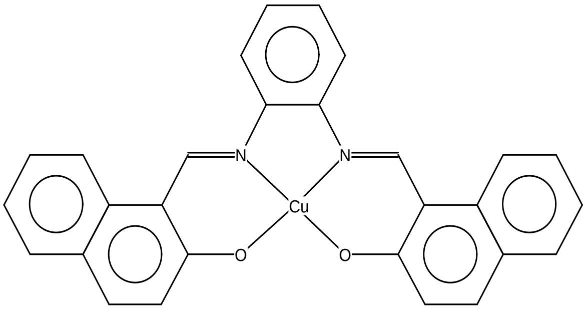

UFEKAS

Reference: Wai-Lun Man, Hoi-Ki Kwong, W.W.Y.Lam, Jing Xiang, Tsz-Wing Wong, Wing-Hong Lam, Wing-Tak Wong, Shie-Ming Peng, Tai-Chu Lau (2008) *Inorg.Chem.* ,**47,**5936

Formula: $C_{24}\ H_{20}\ N_4\ O_2\ Ru_1\ ^{1+}, C_2\ Au_1\ N_2\ ^{1-}$

Compound Name: bis(acetonitrile-N)-(N,N'-bis(salicylidene)-o-phenylenediamine)-ruthenium(iii) dicyano-gold(i)

Space Group:	Pbca	***Cell:***	***a***	13.219(0)	***b***	17.309(0)	***c***	21.553(0)
Space Group No.:	61	***(Å,°)***	α	90.00	β	90.00	γ	90.00

R-Factor (%): 8.81 ***Temperature(K):*** 295 ***Density(g/cm^3):*** 2.011

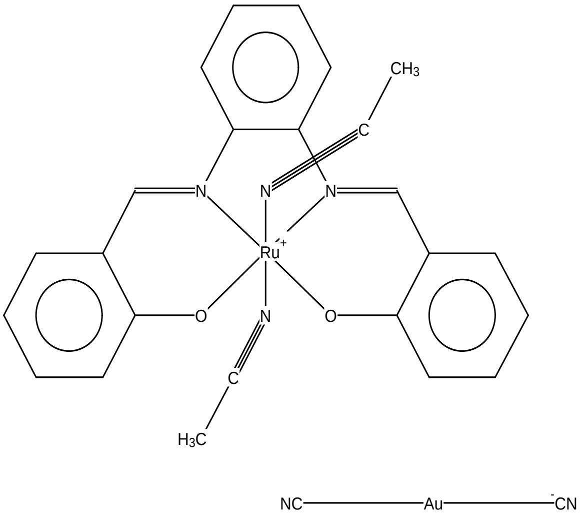

UGAMOF

Reference: Hailong Wang, Daopeng Zhang, Laijin Tian, Li-Fang Zhang (2008) *Acta Crystallogr.,Sect.E:Struct.Rep.Online* ,**64,** m1460

Formula: $C_{24}\ H_{24}\ Cl_2\ N_2\ O_5\ Pb_1\ S_1\ Zn_1, C_3\ H_7\ N_1\ O_1$

Compound Name: (μ_2-6,6'-dimethoxy-2,2'-(o-phenylenebis(nitrilomethylidyne))diphenolato) -dichlorido-(dimethyl sulfoxide)-lead(ii)-zinc(ii) N,N-dimethylformamide solvate

Space Group:	P21/c	***Cell:***	***a***	15.285(0)	***b***	18.843(0)	***c***	10.734(0)
Space Group No.:	14	***(Å,°)***	α	90.00	β	94.77(0)	γ	90.00

R-Factor (%): 3.58 ***Temperature(K):*** 295 ***Density(g/cm^3):*** 1.874

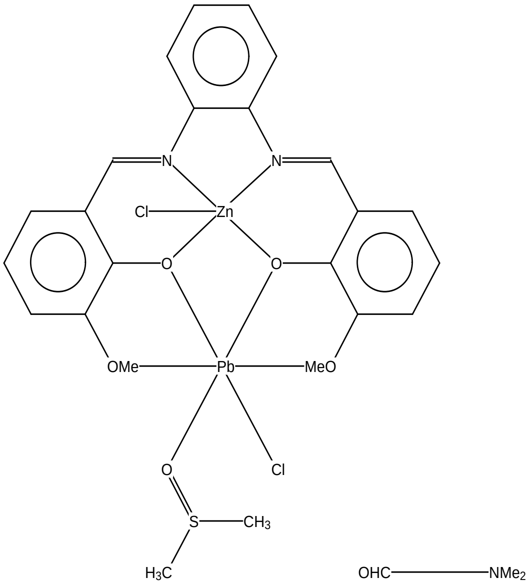

ULATOR

Reference: Z.Lim, C.M.Forsyth, B.Graham (2011) *Acta Crystallogr.,Sect.E:Struct.Rep.Online* ,**67,**m368

Formula: $C_{58}\ H_{42}\ N_8\ O_6\ Zn_4\ ^{2+}, 2(Cl_1\ O_4\ ^{1-}), 2(C_3\ H_7\ N_1\ O_1)$

Compound Name: bis(μ_2-methoxo)-bis(μ_2-3,10,18,25-tetraazapentacyclo[17.4.4.3.1.1]triconta-1(31),2,4(9),5,7,10,12,14,16(32) ,17,19(24),20,22,25,27,29-hexadecaene-31,32-diolato)-di-zinc(ii) diperchlorate N,N-dimethylformamide solvate

Space Group:	C2/c	***Cell:***	***a***	30.945(0)	***b***	10.451(0)	***c***	20.577(0)
Space Group No.:	15	***(Å,°)***	α	90.00	β	112.02(0)	γ	90.00

R-Factor (%): 4.48 ***Temperature(K):*** 123 ***Density(g/cm^3):*** 1.673

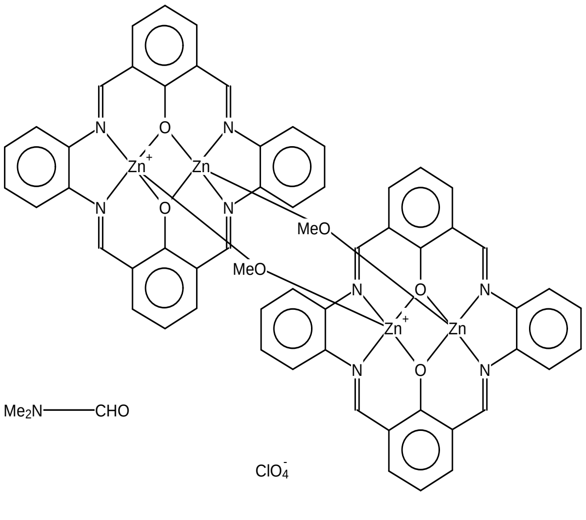

UMICID

Reference: S.R.Lane, N.Sisay, B.Carney, S.Dannoon, S.Williams, H.P.Engelbrecht, C.L.Barnes, S.S.Jurisson (2011) *Dalton Trans.* ,**40,** 269

Formula: $C_{20}\ H_{14}\ Cl_1\ N_2\ O_3\ Re_1,C_1\ H_1\ Cl_3$

Compound Name: Chloro-oxo-(2,2'-(1,2-phenylenebis((nitrilo)methylylidene))diphenolato)-rhenium chloroform solvate

Space Group:	Pbca	***Cell:***	***a***	19.439(1)	***b***	10.926(0)	***c***	21.487(1)
Space Group No.:	61	***(Å,°)***	α	90.00	β	90.00	γ	90.00

R-Factor (%): 3.50 ***Temperature(K):*** 173 ***Density(g/cm^3):*** 1.954

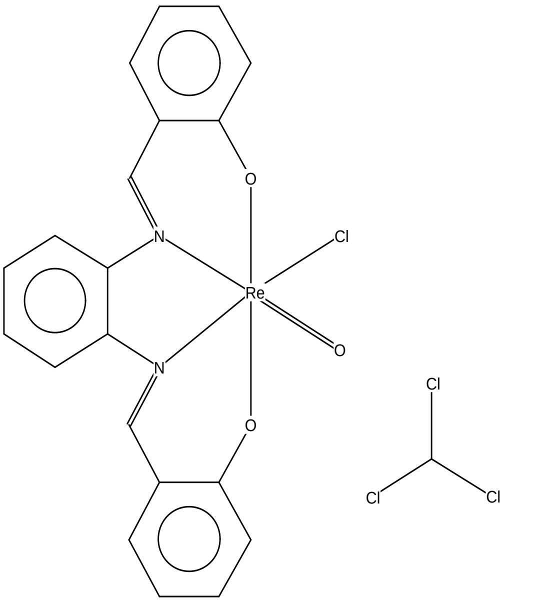

UMICOJ

Reference: S.R.Lane, N.Sisay, B.Carney, S.Dannoon, S.Williams, H.P.Engelbrecht, C.L.Barnes, S.S.Jurisson (2011) *Dalton Trans.* ,**40,** 269

Formula: $C_{38}\ H_{29}\ Cl_1\ N_2\ O_2\ P_1\ Re_1,2(C_1\ H_2\ Cl_2)$

Compound Name: Chloro-(2,2'-(1,2-phenylenebis((nitrilo)methylylidene))diphenolato)-(triphenylphosphine)-rhenium dichloromethane solvate

Space Group:	P21/c	***Cell:***	***a***	23.081(0)	***b***	13.195(0)	***c***	25.987(0)
Space Group No.:	14	***(Å,°)***	α	90.00	β	104.45(0)	γ	90.00

R-Factor (%): 3.98 ***Temperature(K):*** 173 ***Density(g/cm^3):*** 1.678

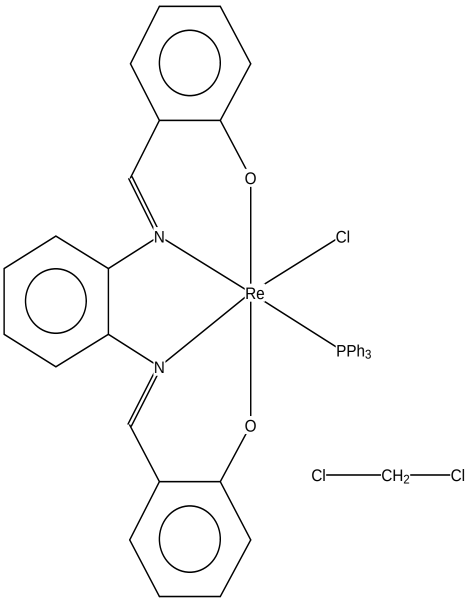

UPEZEV

Reference: Zhenzhen Dong, J.N.Plampin III, G.P.A.Yap, J.M.Fox (2010) *Org.Lett.* ,**12,**4002

Formula: $C_{42}\ H_{36}\ N_4\ Ni_1\ O_4$

Compound Name: (1,2-bis((2-oxy-3-((3-methyl-2,3-dihydro-1H-inden-4-yl)carbamoyl) benzylidene)amino)benzene)-nickel

Space Group:	P212121	***Cell:***	***a***	11.937(4)	***b***	14.935(4)	***c***	19.399(7)
Space Group No.:	19	***(Å,°)***	α	90.00	β	90.00	γ	90.00

R-Factor (%): 4.03 ***Temperature(K):*** 120 ***Density(g/cm^3):*** 1.382

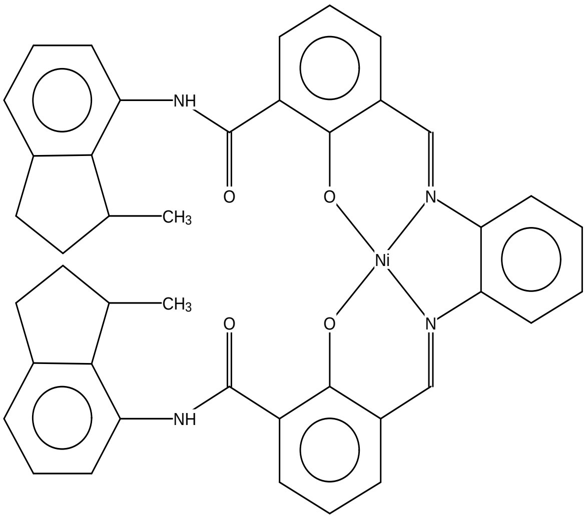

UTIBIJ

Reference: M.T.Raisanen, M.Nieger, A.M.Z.Slawin, M.Leskela, T.Repo (2011) *CrystEngComm* ,**13,**4701

Formula: $C_{32}\ H_{38}\ Cu_1\ N_2\ O_4,C_1\ H_2\ Cl_2$

Compound Name: (2,2'-(1,2-Phenylenebis((nitrilo)methylylidene))bis(5-(n-hexyloxy) phenolato)-N,N',O,O')-copper(ii) dichloromethane solvate

Synonym: N,N'-(o-Phenylene)bis(4-n-hexyloxysalicylideneiminato-N,N',O,O')-copper(ii) dichloromethane solvate

Space Group:	P21/c	***Cell:***	***a***	19.521(2)	***b***	14.028(2)	***c***	12.066(3)
Space Group No.:	14	***(Å,°)***	α	90.00	β	99.20(2)	γ	90.00

R-Factor (%): 8.62 ***Temperature(K):*** 123 ***Density(g/cm^3):*** 1.350

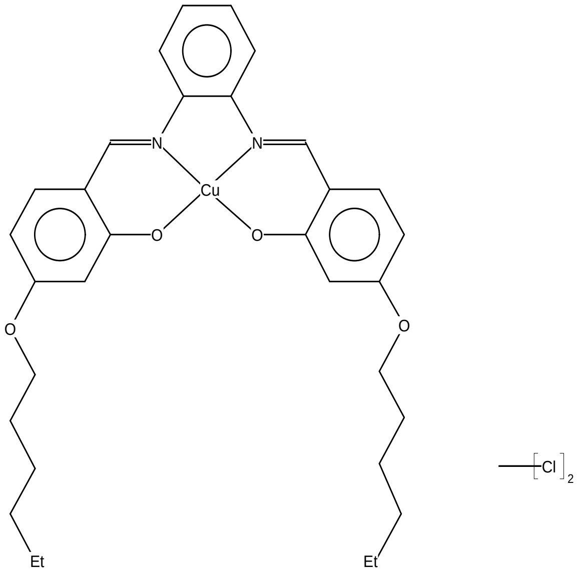

UTIBOP

Reference: M.T.Raisanen, M.Nieger, A.M.Z.Slawin, M.Leskela, T.Repo (2011) *CrystEngComm* ,**13,**4701

Formula: $C_{40} H_{54} N_2 Ni_1 O_4$

Compound Name: (2,2'-(1,2-Phenylenebis((nitrilo)methylylidene))bis(5-(n-decyloxy) phenolato)-N,N',O,O')-nickel(ii)

Synonym: N,N'-(o-Phenylene)bis(4-n-decyloxysalicylideneiminato-N,N',O,O')-nickel(ii)

**Space Group:**	P21/n	**Cell:**	**a** 14.940(6)	**b** 5.532(2)	**c** 42.014(2)		
**Space Group No.:**	14	**(Å,°)**	α 90.00	β 90.75(0)	γ 90.00		
**R-Factor (%):**	11.34	**Temperature(K):**	93	**Density(g/cm^3):**	1.312		

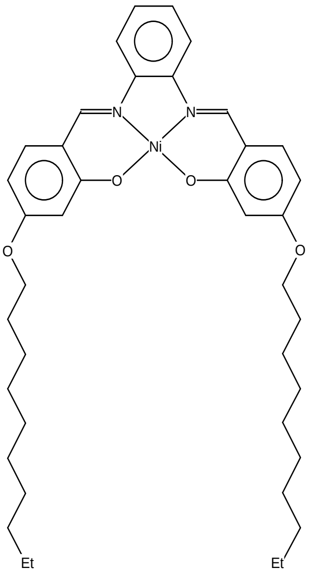

VAHSIG

Reference: F.C.J.M.van Veggel, S.Harkema, M.Bos, W.Verboom, C.J.van Staveren, G.J.Gerritsma, D.N.Reinhoudt (1989) *Inorg.Chem.* , **28,**1133

Formula: $C_{34} H_{40} Ba_1 Cl_1 N_2 Ni_1 O_{14}{}^{1+}, C_1 H_4 O_1, Cl_1 O_4{}^{1-}$

Compound Name: (μ_2-(9,10,12,13,15,16,18,19,21,22,24,25,27,28-Tetradecahydro-3,7:30,34-dimetheno-8,11,14,17,20,23,26,29,1,36-benzo-octaoxadiazacyclo-octatriacontanine-41,42-diolato))-perchlorato-barium-nickel perchlorate methanol clathrate

**Space Group:**	P21/n	**Cell:**	**a** 15.096(4)	**b** 20.278(4)	**c** 13.818(4)		
**Space Group No.:**	14	**(Å,°)**	α 90.00	β 93.61(1)	γ 90.00		
**R-Factor (%):**	8.10	**Temperature(K):**	295	**Density(g/cm^3):**	1.674		

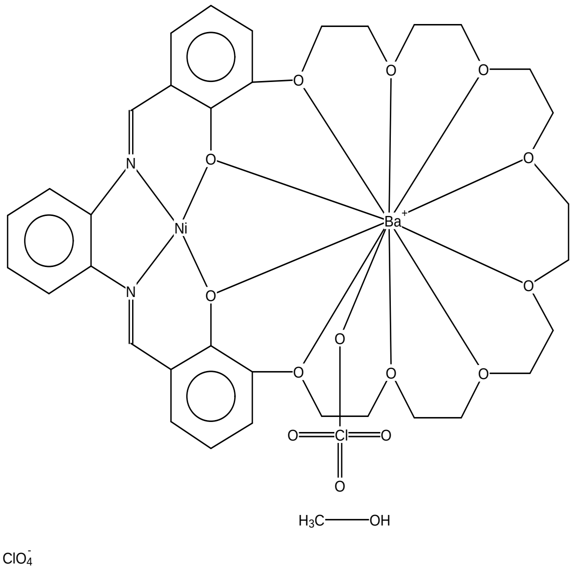

VAHTAZ

Reference: F.C.J.M.van Veggel, S.Harkema, M.Bos, W.Verboom, C.J.van Staveren, G.J.Gerritsma, D.N.Reinhoudt (1989) *Inorg.Chem.* , **28,**1133

Formula: $C_{56} H_{61} Ba_1 F_3 N_5 O_{13} S_1 Zn_1{}^{1+}, C_1 F_3 O_3 S_1{}^{1-}$

Compound Name: (μ_2-(39,40,41-Trimethoxy-12,17,22-trimethyl-25H-3,7:10,14:15,19; 20,24:27,31-pentametheno-9H-8,26,1,33-benzodioxadiazacyclopentatriacontine-38,42-diolato))-(trifluoromethanesulfonato-O)-tris(N,N-dimethylformamido-O)-barium-zinc trifluoromethanesulfonate

**Space Group:**	C2/c	**Cell:**	**a** 24.301(5)	**b** 26.232(4)	**c** 22.468(7)		
**Space Group No.:**	15	**(Å,°)**	α 90.00	β 119.06(2)	γ 90.00		
**R-Factor (%):**	8.50	**Temperature(K):**	173	**Density(g/cm^3):**	1.542		

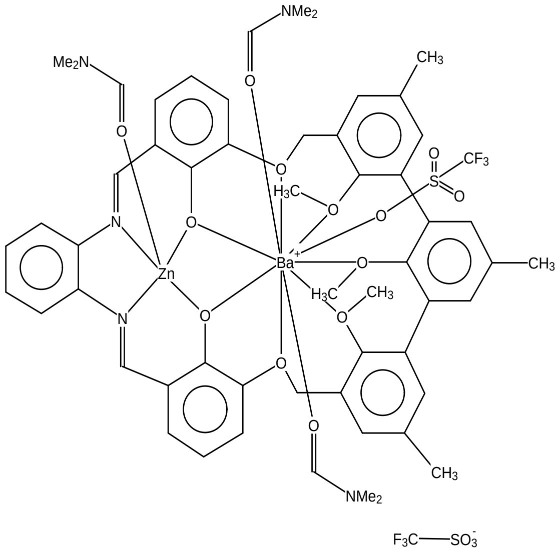

VEBFOY

Reference: A.Mitra, L.J.DePue, S.Parkin, D.A.Atwood (2006) *J.Am.Chem.Soc.* ,**128,**1147

Formula: $C_{36} H_{46} Al_1 Br_1 N_2 O_2$

Compound Name: Bromo-(N,N'-o-phenylenebis(3,5-di-t-butylsalicylideneiminato))-aluminium

**Space Group:**	P-1	**Cell:**	**a** 10.769(0)	**b** 12.457(0)	**c** 13.614(0)		
**Space Group No.:**	2	**(Å,°)**	α 92.43(0)	β 95.63(0)	γ 110.94(0)		
**R-Factor (%):**	5.82	**Temperature(K):**	90	**Density(g/cm^3):**	1.268		

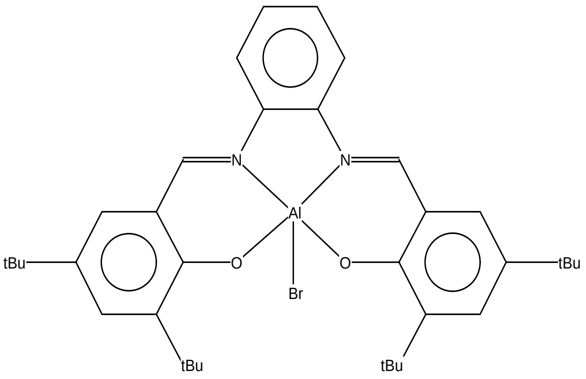

VEBGEP

Reference: A.Mitra, L.J.DePue, S.Parkin, D.A.Atwood (2006) *J.Am.Chem.Soc.* ,**128,**1147

Formula: $C_{72}\ H_{76}\ Al_1\ N_2\ O_4\ P_2\ ^{1+}$,$Br_1\ ^{1-}$,0.71($C_7\ H_8$)

Compound Name: (N,N'-o-Phenylenebis(3,5-di-t-butylsalicylideneiminato))-bis(triphenylphosphine oxide)-aluminium bromide toluene solvate

Space Group: Pbca ***Cell:*** ***a*** 22.612(0) ***b*** 22.858(0) ***c*** 26.785(0)
Space Group No.: 61 ***(Å,°)*** α 90.00 β 90.00 γ 90.00

R-Factor (%): 8.09 ***Temperature(K):*** 90 ***Density(g/cm³):*** 1.216

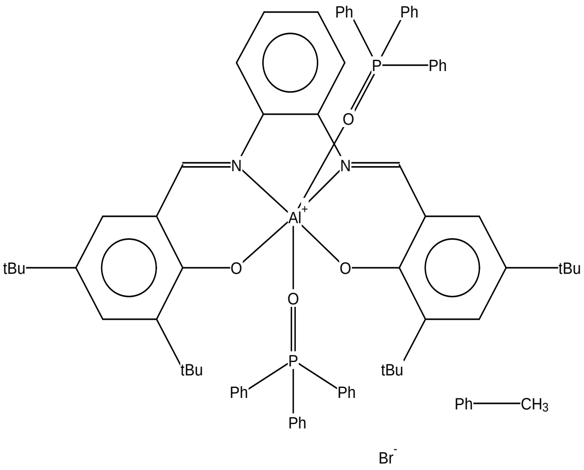

VEBGIT

Reference: A.Mitra, L.J.DePue, S.Parkin, D.A.Atwood (2006) *J.Am.Chem.Soc.* ,**128,**1147

Formula: $C_{72}\ H_{76}\ Al_1\ N_2\ O_{10}\ P_2\ ^{1+}$,$Br_1\ ^{1-}$,0.5($C_7\ H_8$)

Compound Name: (N,N'-Ethylenebis(3,5-di-t-butylsalicylideneiminato))-bis(triphenoxyphosphine oxide)-aluminium bromide toluene solvate

Space Group: P21/n ***Cell:*** ***a*** 19.338(0) ***b*** 15.386(0) ***c*** 23.759(0)
Space Group No.: 14 ***(Å,°)*** α 90.00 β 103.84(0) γ 90.00

R-Factor (%): 8.23 ***Temperature(K):*** 90 ***Density(g/cm³):*** 1.301

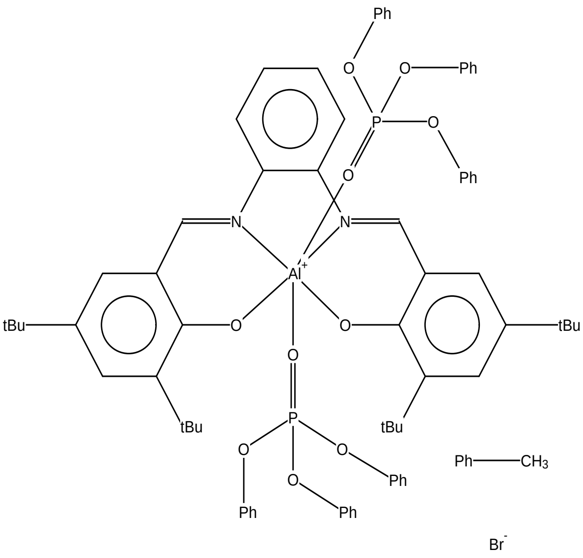

VEFWIO

Reference: M.V.Escarcega-Bobadilla, D.Anselmo, S.J.Wezenberg, E.C.Escudero-Adan, M.M.Belmonte, E.Martin, A.W.Kleij (2012) *Dalton Trans.* ,**41,**9766

Formula: $C_{78}\ H_{109}\ N_7\ O_8\ Zn_3$,$C_2\ H_3\ N_1$

Compound Name: (μ_2-3,3'-bis(((2-((3,5-di-t-butyl-2-hydroxybenzylidene)amino)-phenyl)imino)methyl)biphenyl-2,2'-diolato)-(μ_2-acetato)-tris(hexylamine)-(acetato-O)-tri-zinc acetonitrile solvate

Space Group: P-1 ***Cell:*** ***a*** 15.215(1) ***b*** 17.628(1) ***c*** 17.826(1)
Space Group No.: 2 ***(Å,°)*** α 112.37(0) β 113.18(0) γ 90.04(0)

R-Factor (%): 7.23 ***Temperature(K):*** 100 ***Density(g/cm³):*** 1.254

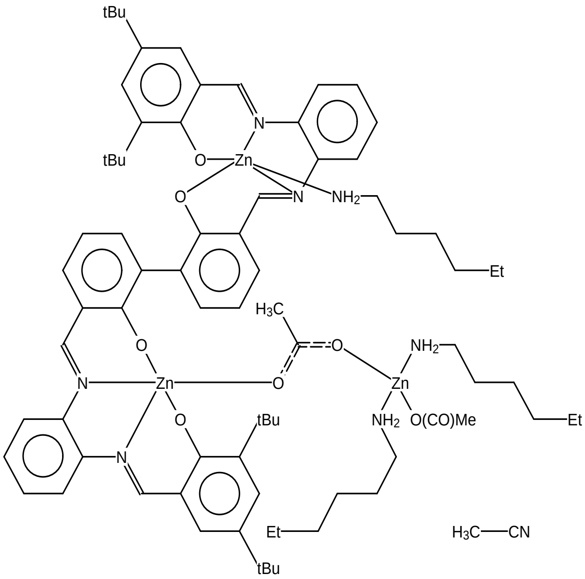

VENHON

Reference: D.d.Bellefeuille, M.S.Askari, B.Lasalle-Kaiser, Y.Journaux, A.Aukauloo, M.Orio, F.Thomas, X.Ottenwaelder (2012) *Inorg.Chem.* , **51,**12796

Formula: $C_{40}\ H_{56}\ N_4\ Ni_1\ O_2$

Compound Name: (2,2'-((4,5-bis(dimethylamino)-1,2-phenylene)bis((nitrilo)methylylidene))bis(4,6-di-t-butylphenolato))-nickel(ii)

Space Group: C2/c ***Cell:*** ***a*** 23.952(0) ***b*** 15.171(0) ***c*** 10.391(0)
Space Group No.: 15 ***(Å,°)*** α 90.00 β 101.88(0) γ 90.00

R-Factor (%): 4.14 ***Temperature(K):*** 110 ***Density(g/cm³):*** 1.229

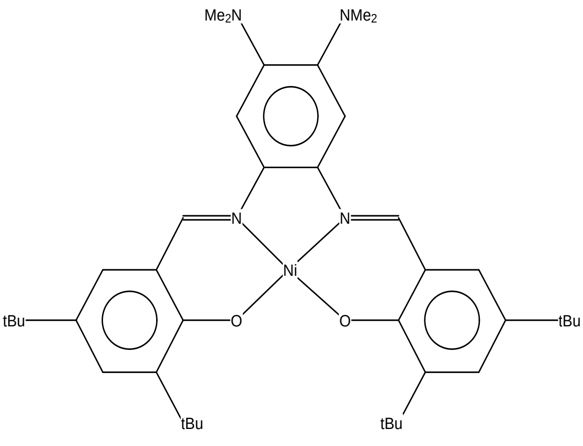

VENHUT

Reference: D.d.Bellefeuille, M.S.Askari, B.Lasalle-Kaiser, Y.Journaux, A.Aukauloo, M.Orio, F.Thomas, X.Ottenwaelder (2012) *Inorg.Chem.* , **51,**12796

Formula: $C_{40} H_{57} N_4 Ni_1 O_2{}^{1+}, F_6 Sb_1{}^{1-}$

Compound Name: (2,2'-((4-(dimethylamino)-5-(dimethylammonio)-1,2-phenylene)bis((nitrilo) methylylidene))bis(4,6-di-t-butylphenolato))-nickel(ii) hexafluoroantimonate

Space Group:	P21/c	***Cell:***	***a*** 15.515(0)	***b*** 9.074(0)	***c*** 32.074(0)		
Space Group No.:	14	***(Å,°)***	α 90.00	β 111.38(0)	γ 90.00		
R-Factor (%):	3.82	***Temperature(K):***	110	***Density(g/cm³):***	1.454		

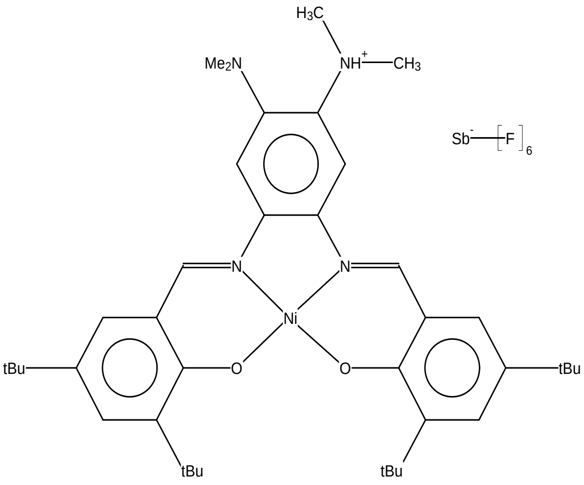

VIKJEE

Reference: Li Jun, Yang Shu-Ming, Zhang Feng-Xiang, Tang Zong-Xun, Shi Qi-Zhen, Wu Qiang-Jin, Huang Zi-Xiang (2000) *Wuji Huaxue Xuebao(Chin.)(Chin.J.Inorg.Chem.)* ,**16,**84

Formula: $C_{27} H_{19} Mn_1 N_2 O_4, C_2 H_3 N_1$

Compound Name: (Benzene-1,2-diamine-N,N'-bis(salicylaldehydealdiminato))-(2-formylphenolato)-manganese(iii) acetonitrile solvate

Space Group:	P21/n	***Cell:***	***a*** 10.252(2)	***b*** 20.146(3)	***c*** 12.494(4)
Space Group No.:	14	***(Å,°)***	α 90.00	β 111.12(2)	γ 90.00
R-Factor (%):	3.90	***Temperature(K):***	295	***Density(g/cm³):***	1.466

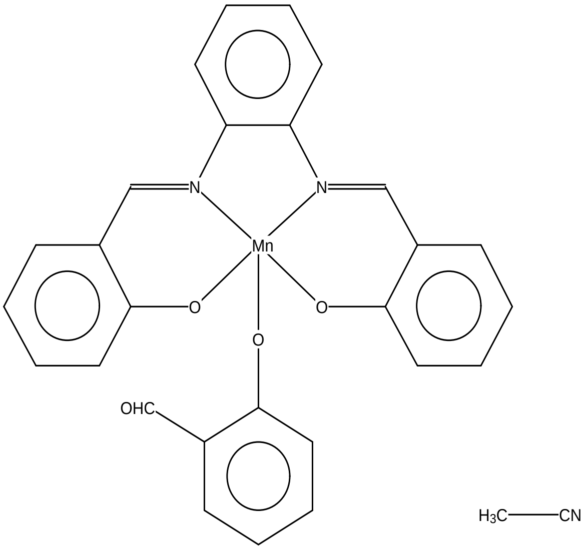

VIQMEO

Reference: N.E.Eltayeb, S.G.Teoh, S.Chantrapromma, Hoong-Kun Fun, K.Ibrahim (2007) *Acta Crystallogr.,Sect.E:Struct.Rep.Online* ,**63,**m2838

Formula: $C_{20} H_{16} N_2 O_5 Zn_1, 3(H_2 O_1)$

Compound Name: Aqua-(5,5'-dihydroxy-2,2'-(1,2-phenylene-bis(nitrilomethylidyne))-diphenolato-κ⁴O,N,N',O')-zinc(ii) trihydrate

Space Group:	P-1	***Cell:***	***a*** 4.746(0)	***b*** 13.929(0)	***c*** 15.113(0)
Space Group No.:	2	***(Å,°)***	α 82.15(0)	β 82.37(0)	γ 85.64(0)
R-Factor (%):	3.89	***Temperature(K):***	100	***Density(g/cm³):***	1.641

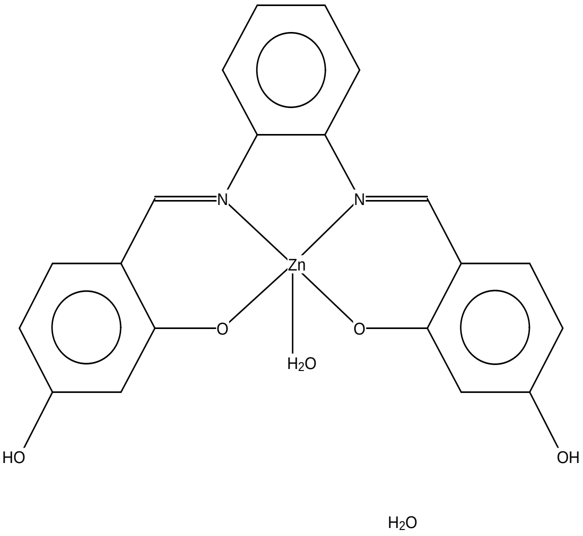

VOMLIS

Reference: P.A.Chetcuti, A.Liegard, G.Rihs, G.Rist (1991) *Helv.Chim.Acta* ,**74,**1591

Formula: $C_7 H_8 Fe_1 N_1 O_2{}^{1+}, C_{26} H_{24} Cu_1 N_2 O_6, F_6 P_1{}^{1-}$

Compound Name: (η^5-Cyclopentadienyl)-ammine-dicarbonyl-iron (9,10,12,13,15,16-hexahydro-3,7:18,22-dimetheno-benzo-8,11,14,17-tetraoxa-1,24-diazacyclohexacosane-29,30-diolato-O,O',N,N')-copper(ii) hexafluorophosphate clathrate

Space Group:	P-1	***Cell:***	***a*** 9.850(1)	***b*** 9.866(1)	***c*** 18.176(2)
Space Group No.:	2	***(Å,°)***	α 103.97(1)	β 99.16(1)	γ 89.66(1)
R-Factor (%):	4.49	***Temperature(K):***	190	***Density(g/cm³):***	1.695

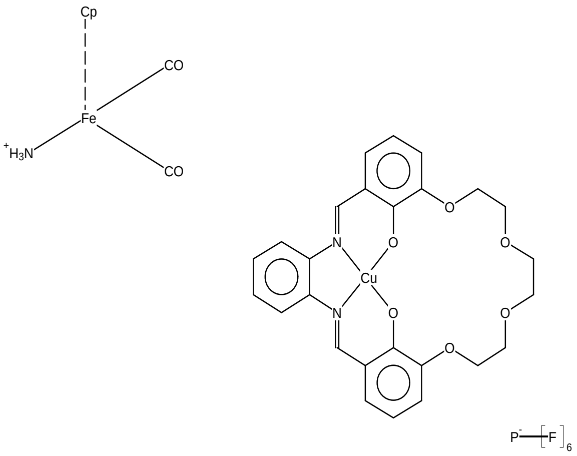

VOPKIV

Reference: M.Koikawa, Y.Matsuda, T.Tokii (2008)
Anal.Sci.:X-Ray Struct.Anal.Online ,**24,**x283

Formula: $C_{20} H_{14} Cu_1 N_2 O_6, C_1 H_4 O_1$

Compound Name: (2,2'-(o-Phenylenebis(iminomethyl))bis(5,6-dihydroxyphenolato-N,N',O,O'))-copper(ii) methanol solvate

Space Group: P21/n ***Cell:*** ***a*** 14.601(14) ***b*** 8.574(3) ***c*** 15.953(9)
Space Group No.: 14 ***(Å,°)*** α 90.00 β 109.49(8) γ 90.00

R-Factor (%): 6.39 ***Temperature(K):*** 296 ***Density(g/cm³):*** 1.672

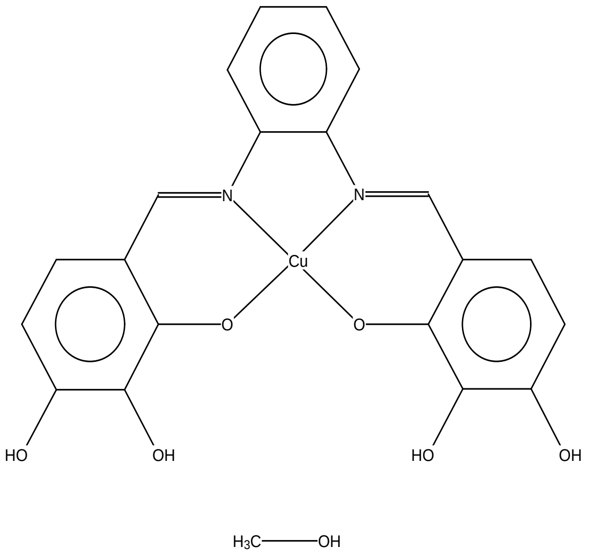

WABDEK

Reference: Haixia Wang (2010)
Acta Crystallogr.,Sect.E:Struct.Rep.Online ,**66,**m1571

Formula: $C_{21} H_{15} Cl_1 N_2 Ni_1 O_2$

Compound Name: (2,2'-[4-Chloro-5-methyl-o-phenylenebis(nitrilomethylidyne)]diphenolato)-nickel(ii)

Space Group: P21/c ***Cell:*** ***a*** 11.045(1) ***b*** 8.020(0) ***c*** 19.596(1)
Space Group No.: 14 ***(Å,°)*** α 90.00 β 106.37 γ 90.00

R-Factor (%): 5.16 ***Temperature(K):*** 293 ***Density(g/cm³):*** 1.681

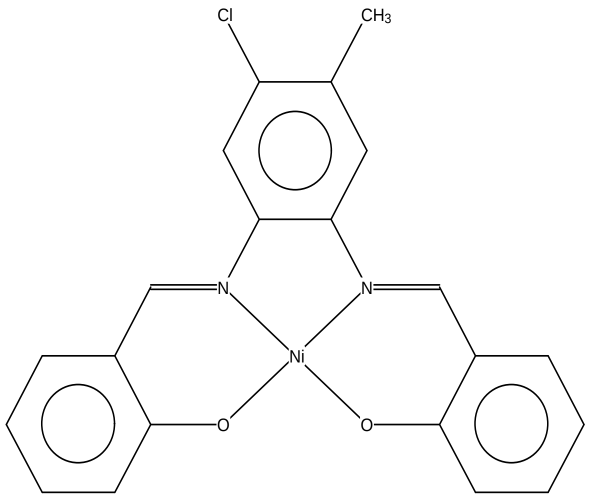

WAKQIJ

Reference: V.V.Roznyatovsky, N.E.Borisova, M.D.Reshetova, Yu.A.Ustynyuk, G.G.Aleksandrov, I.L.Eremenko, I.I.Moiseev (2004)
Izv.Akad.Nauk SSSR,Ser.Khim.(Russ.)(Russ.Chem.Bull.) ,1161

Formula: $C_{46} H_{52} Cu_2 N_4 O_6, 2(C_1 H_1 Cl_3), 2(C_2 H_6 O_1)$

Compound Name: (μ_2-14,29-Di-t-butyl-3,10,18,25-tetraazapentacyclo(25.3.1.1^{12,16}.0^{4,9}.0^{19,24})dotriaconta-1(31),2,4(9),5,7,12(32),13,15,17,19,21,23,27,29-tetradecaene-31,32-diolato)-bis(pivalato)-di-copper(ii) ethanol chloroform solvate

Space Group: P21/n ***Cell:*** ***a*** 16.220(40) ***b*** 9.980(30) ***c*** 18.270(40)
Space Group No.: 14 ***(Å,°)*** α 90.00 β 108.46(13) γ 90.00

R-Factor (%): 7.84 ***Temperature(K):*** 110 ***Density(g/cm³):*** 1.438

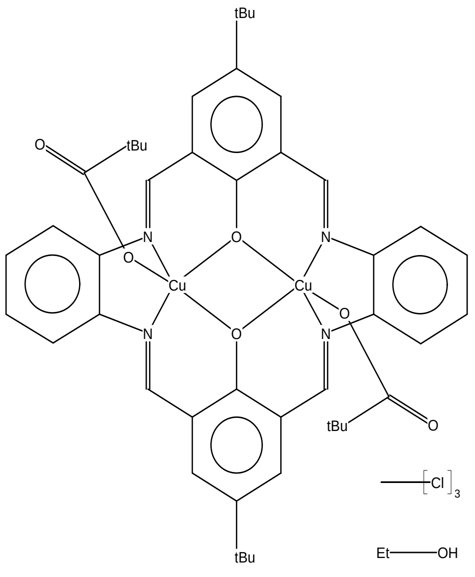

WATMOT

Reference: D.Coucouvanis, S.G.Jonasdottir, D.Christodoulou, C.G.Kim, J.W.Kampf (1993) *Inorg.Chem.* ,**32,**2987

Formula: $C_{24} H_{22} N_4 Ni_1 O_2, 2(C_4 H_8 O_1)$

Compound Name: (16,17-Dihydroxy-9,24-dimethyl-2,5,13,20-tetra-azacyclohexacosa-1(26),6,8,10,12,14,16,18,20,22,24-undecaene-N,N',N'',N''')-nickel(ii) tetrahydrofuran solvate

Synonym: (2,3-Ethylene-5,6:13,14-bis(5'-methylbenzo)-9,10-(4',5'-diolbenzo)-1,4,8,11-tetrazacyclotetradeca-7,11-diene)-nickel(ii) tetrahydrofuran solvate

Space Group: P-1 ***Cell:*** ***a*** 10.908(1) ***b*** 11.391(3) ***c*** 12.727(3)
Space Group No.: 2 ***(Å,°)*** α 84.56(2) β 80.28(2) γ 73.39(2)

R-Factor (%): 6.15 ***Temperature(K):*** 295 ***Density(g/cm³):*** 1.339

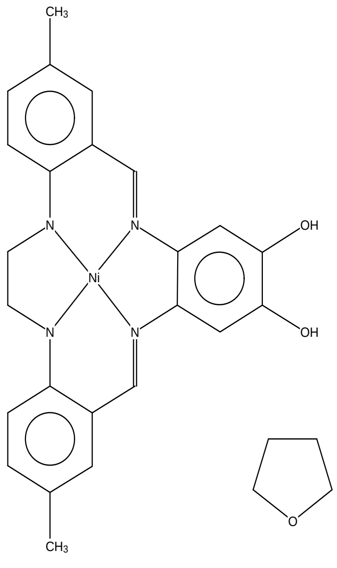

WATMOT01

Reference: S.Jonasdottir, Chang-Gyoun Kim, J.Kampf, D.Coucouvanis (1996) *Inorg.Chim.Acta* ,**243,**255

Formula: $C_{24}\ H_{22}\ N_4\ Ni_1\ O_2,2(C_4\ H_8\ O_1)$

Compound Name: (16,17-Dihydroxy-9,24-dimethyl-2,5,13,20-tetra-azacyclohexacosa-1(26), 6,8,10,12,14,16,18,20,22,24-undecaene-N,N',N'',N''')-nickel(ii) tetrahydrofuran solvate

Synonym: (2,3-Ethylene-5,6:13,14-bis(5'-methylbenzo)-9,10-(4',5'-diolbenzo)-1,4,8, 11-tetrazacyclotetradeca-7,11-diene)-nickel(ii) tetrahydrofuran solvate

Space Group:	P-1	***Cell:***	***a***	10.908(1)	***b***	11.391(3)	***c***	12.727(3)
Space Group No.:	2	***(Å,°)***	α	84.56(2)	β	80.28(2)	γ	73.39(2)

R-Factor (%): 6.15 ***Temperature(K):*** 295 ***Density(g/cm³):*** 1.339

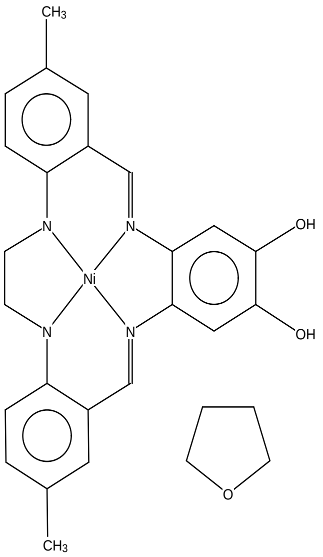

WEBPOK

Reference: G.Magadur, F.Bouanis, E.Norman, R.Guillot, J.-S.Lauret, V.Huc, C.-S.Cojocaru, T.Mallah (2012) *Chem.Commun.* ,**48,**9071

Formula: $C_{62}\ H_{48}\ N_6\ O_7\ Tb_2,0.5(H_2\ O_1)$

Compound Name: (μ_2-2,2'-(1,2-phenylenebis((nitrilo)methylylidene))diphenolato)-bis(2,2'-(1,2-phenylenebis((nitrilo)methylylidene))diphenolato)-(ethanol)-di-terbium hemihydrate

Space Group:	P21/c	***Cell:***	***a***	22.027(3)	***b***	23.021(3)	***c***	22.557(2)
Space Group No.:	14	***(Å,°)***	α	90.00	β	113.13(0)	γ	90.00

R-Factor (%): 6.90 ***Temperature(K):*** 100 ***Density(g/cm³):*** 1.662

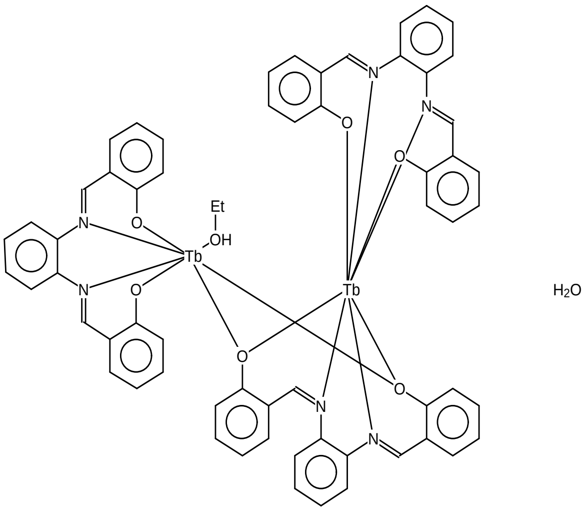

WEHGAT

Reference: K.Bhattacharya, M.Maity, D.Mondal, A.Endo, M.Chaudhury (2012) *Inorg.Chem.* ,**51,**7454

Formula: $C_{31}\ H_{26}\ Br_1\ Fe_1\ N_4\ O_8\ S_2\ V_1$

Compound Name: (μ_2-oxo)-(4-bromo-2-[([(methylsulfanyl)(sulfido) methylidene]hydrazinylidene)methyl]phenolato)-(2,2'-(1,2-phenylenebis(nitrilomethylylidene))bis(6-methoxyphenolato))-hydroxo-oxo-iron-vanadium

Space Group:	P-1	***Cell:***	***a***	11.337(1)	***b***	12.189(1)	***c***	12.912(1)
Space Group No.:	2	***(Å,°)***	α	70.42(0)	β	83.22(0)	γ	85.28(0)

R-Factor (%): 6.43 ***Temperature(K):*** 150 ***Density(g/cm³):*** 1.660

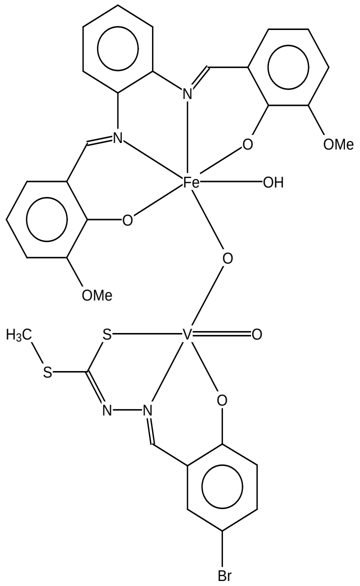

WEHGEX

Reference: K.Bhattacharya, M.Maity, D.Mondal, A.Endo, M.Chaudhury (2012) *Inorg.Chem.* ,**51,**7454

Formula: $C_{32}\ H_{30}\ Fe_1\ N_4\ O_9\ S_2\ V_1,C_2\ H_3\ N_1$

Compound Name: (μ_2-oxo)-(4-methoxo-2-[([(methylsulfanyl)(sulfido) methylidene]hydrazinylidene)methyl]phenolato)-(2,2'-(1,2-phenylenebis(nitrilomethylylidene))bis(6-methoxyphenolato))-aqua-oxo-iron-vanadium acetonitrile solvate

Space Group:	P-1	***Cell:***	***a***	11.531(1)	***b***	12.604(1)	***c***	13.428(1)
Space Group No.:	2	***(Å,°)***	α	106.12(0)	β	91.68(0)	γ	98.38(0)

R-Factor (%): 6.26 ***Temperature(K):*** 293 ***Density(g/cm³):*** 1.484

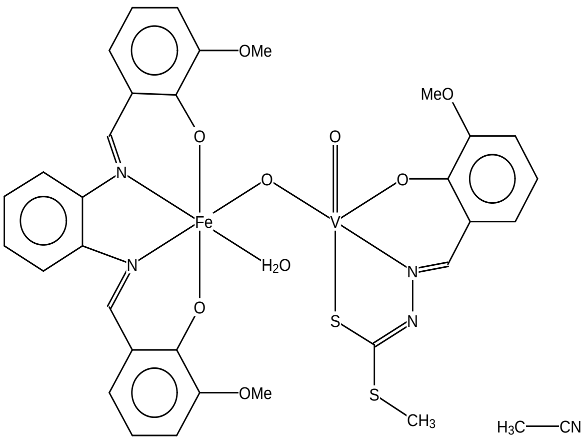

Search: search26 (Tue Dec 2 15:48:50 2014): Hits 645-648

WIBTIK

Reference: A.R.van Doorn, W.Verboom, D.N.Reinhoudt, S.Harkema (1994) *Acta Crystallogr.,Sect.C:Cryst.Struct.Commun.* ,**50,**1449

Formula: $C_{22}\ H_{20}\ N_2\ O_7\ U_1,0.5(H_2\ O_1)$

Compound Name: Aqua-(N,N'-(o-phenylene)-bis(6-methoxysalicylidenealdiminato)-O,O',N,N')-dioxo-uranium hemihydrate

Synonym: Aqua-dioxo-(2,2'-(1,2-phenylene-bis(nitrilomethylidyne)) bis(6-methoxyphenolato)-O,O',N,N')-uranium hemihydrate, Aqua-dioxo-(salophene)-uranium

Space Group:	P-1	***Cell:***	***a*** 12.056(6)	***b*** 12.293(5)	***c*** 15.843(6)
Space Group No.:	2	***(Å,°)***	α 84.01(4)	β 74.91(6)	γ 86.29(5)

R-Factor (%): 4.00 ***Temperature(K):*** 295 ***Density(g/cm³):*** 1.980

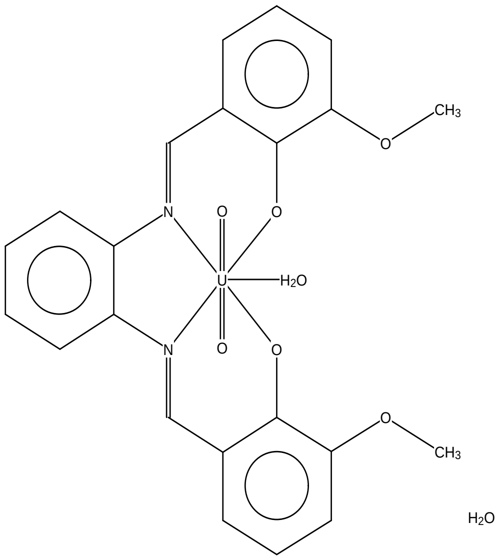

WIWKOD

Reference: E.C.Escudero-Adan, J.Benet-Buchholz, A.W.Kleij (2008) *Inorg.Chem.* ,**47,**410

Formula: $C_{86}\ H_{108}\ N_6\ O_8\ Zn_3,4.5(C_2\ H_3\ N_1)$

Compound Name: bis(μ_2-2-methanolpyridine-6-methanolato)-bis(N,N'-phenylenebis(3,5-di-t-butylsalicylaldiminato))-tri-zinc(ii) acetonitrile solvate

Space Group:	C2/c	***Cell:***	***a*** 28.687(1)	***b*** 21.255(1)	***c*** 31.098(1)
Space Group No.:	15	***(Å,°)***	α 90.00	β 102.18(0)	γ 90.00

R-Factor (%): 4.42 ***Temperature(K):*** 100 ***Density(g/cm³):*** 1.243

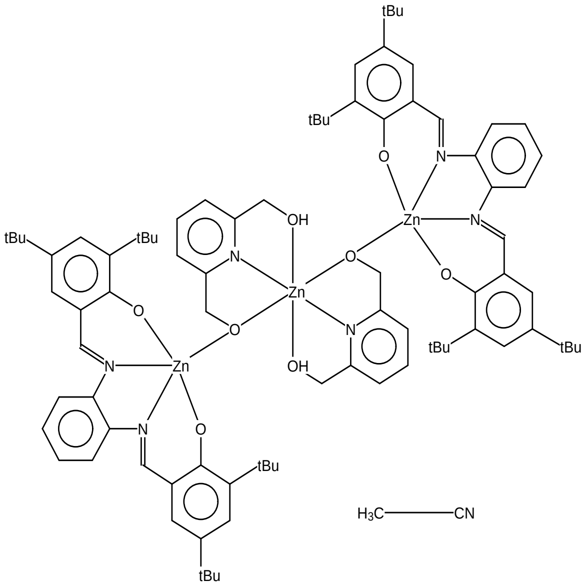

WOCNOR

Reference: A.Elmali, E.Kavlakoglu, Y.Elerman, I.Svoboda (2000) *Acta Crystallogr.,Sect.C:Cryst.Struct.Commun.* ,**56,**1097

Formula: $C_{20}\ H_{12}\ Br_2\ Cl_1\ Fe_1\ N_2\ O_2,C_{20}\ H_{14}\ Br_2\ Cl_1\ Fe_1\ N_2\ O_3,C_3\ H_7\ N_1\ O_1$

Compound Name: aquachloro(4,4'-dibromo-2,2'-[o-phenylenebis(nitrilomethylidyne)]diphenolato-O,N,N',O')iron(iii) chloro(4,4'-dibromo-2,2'-[o-phenylenebis(nitrilomethylidyne)]diphenolato-O,N,N',O')iron(iii) dimethylformamide solvate

Space Group:	P-1	***Cell:***	***a*** 11.623(2)	***b*** 13.426(3)	***c*** 15.156(2)
Space Group No.:	2	***(Å,°)***	α 76.44(1)	β 71.30(1)	γ 75.16(2)

R-Factor (%): 3.70 ***Temperature(K):*** 295 ***Density(g/cm³):*** 1.894

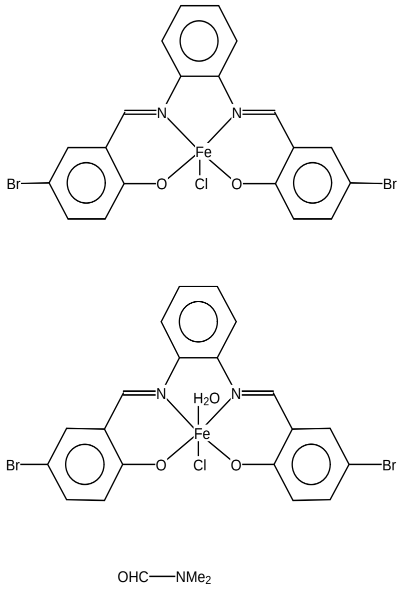

WOFDAX

Reference: D.Homden, C.Redshaw, J.A.Wright, D.L.Hughes, M.R.J.Elsegood (2008) *Inorg.Chem.* ,**47,**5799

Formula: $C_{86}\ H_{114}\ N_2\ O_7\ V_1,2(C_2\ H_3\ N_1)$

Compound Name: (1,2-bis(3-(2,2'-methylenebis(4,6-di-t-butylphenol))-5-t-butylsalicylidene)benzyldiimine)-oxo-vanadium acetonitrile solvate

Space Group:	P21/c	***Cell:***	***a*** 13.586(0)	***b*** 16.964(0)	***c*** 37.557(1)
Space Group No.:	14	***(Å,°)***	α 90.00	β 97.96(0)	γ 90.00

R-Factor (%): 6.40 ***Temperature(K):*** 140 ***Density(g/cm³):*** 1.101

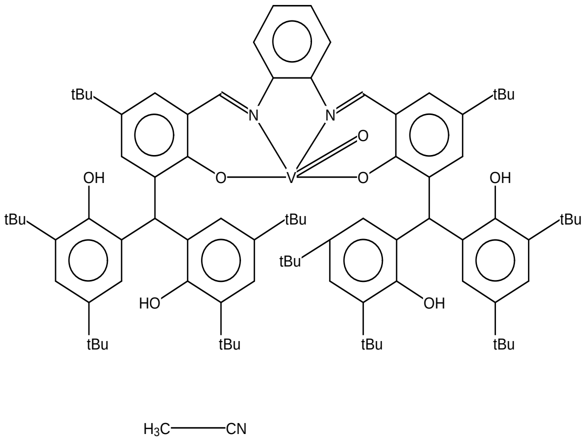

WOFDEB

Reference: D.Homden, C.Redshaw, J.A.Wright, D.L.Hughes, M.R.J.Elsegood (2008) *Inorg.Chem.* ,**47**,5799

Formula: $C_{86} H_{111} N_2 O_8 V_2,5.65(C_2 H_3 N_1)$

Compound Name: (1,2-bis(3-(2,2'-methylenebis(4,6-di-t-butylphenol))-5-t-butylsalicylidene) benzyldiimine)-oxo-vanadium acetonitrile solvate

Space Group: P-1 ***Cell:*** ***a*** 15.975(5) ***b*** 16.346(5) ***c*** 18.858(5)
Space Group No.: 2 ***(Å,°)*** α 82.47(0) β 80.97(0) γ 78.49(0)

R-Factor (%): 11.49 ***Temperature(K):*** 120 ***Density(g/cm³):*** 1.145

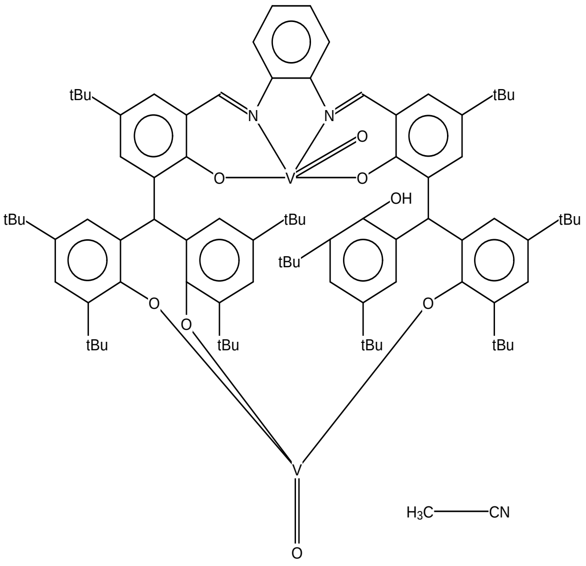

WOGQAL

Reference: Hailong Wang, Daopeng Zhang, Li-Fang Zhang (2008) *Acta Crystallogr.,Sect.E:Struct.Rep.Online* ,**64,**m1408

Formula: $C_{44} H_{36} Cl_4 N_4 O_8 Pb_2 Zn_2,2(C_3 H_7 N_1 O_1)$

Compound Name: bis(μ_2-Chlorido)-bis(μ_2-6,6'-dimethoxy-2,2'-(o-phenylenebis(nitrilomethylidyne))diphenolato)-dichlorido-di-lead(ii)-di-zinc(ii) N,N'-dimethylformamide solvate

Space Group: P21/c ***Cell:*** ***a*** 7.496(0) ***b*** 32.119(3) ***c*** 11.237(0)
Space Group No.: 14 ***(Å,°)*** α 90.00 β 95.73(0) γ 90.00

R-Factor (%): 3.78 ***Temperature(K):*** 295 ***Density(g/cm³):*** 1.952

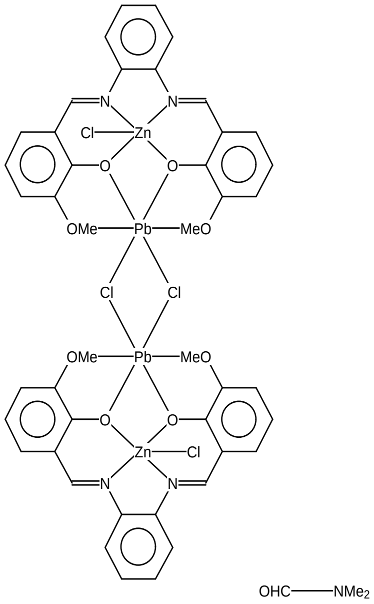

WUQJIC

Reference: J.A.A.W.Elemans, S.J.Wezenberg, M.J.J.Coenen, E.C.Escudero-Adan, J.Benet-Buchholz, D.den Boer, S.Speller, A.W.Kleij, S.De Feyter (2010) *Chem.Commun.* ,**46,**2548

Formula: $C_{51} H_{71} N_3 O_2 Zn_1$

Compound Name: (2,2'-(1,2-Phenylenebis((nitrilo)methylylidene))bis(4-dodecyl-6-methylphenolato))-pyridine-zinc(ii)

Space Group: P-1 ***Cell:*** ***a*** 8.261(8) ***b*** 12.037(12) ***c*** 23.260(20)
Space Group No.: 2 ***(Å,°)*** α 86.70(1) β 82.33(2) γ 88.33(2)

R-Factor (%): 8.14 ***Temperature(K):*** 100 ***Density(g/cm³):*** 1.195

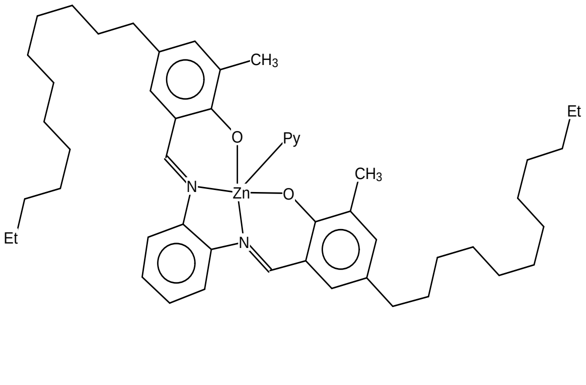

XABPOH

Reference: D.Maity, M.G.B.Drew, J.F.Godsell, S.Roy, G.Mukhopadhyay (2010) *Transition Met.Chem.* ,**35,**197

Formula: $C_{20} H_{16} Cl_2 Cu_2 N_2 O_3$

Compound Name: (μ_2-2,2'-(1,2-Phenylene-bis((nitrilo)methylylidene))-diphenolato)-aqua-(dichloro)-di-copper

Space Group: P21/n ***Cell:*** ***a*** 11.886(6) ***b*** 7.746(1) ***c*** 20.684(4)
Space Group No.: 14 ***(Å,°)*** α 90.00 β 93.68(3) γ 90.00

R-Factor (%): 7.21 ***Temperature(K):*** 150 ***Density(g/cm³):*** 1.854

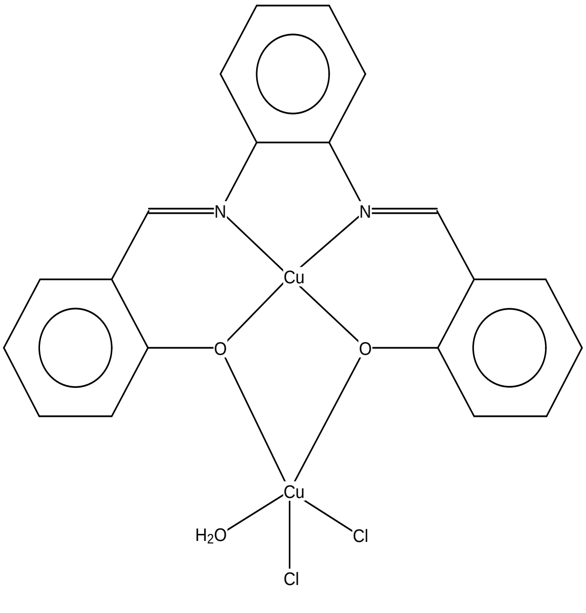

Search: search26 (Tue Dec 2 15:48:50 2014): Hits 653-656

XAKLEC

Reference: H.Kargar, R.Kia, M.N.Tahir, A.Sahraei (2010) *Acta Crystallogr.,Sect.E:Struct.Rep.Online* ,**66,**m1246

Formula: $C_{24}\ H_{22}\ N_2\ Ni_1\ O_4$

Compound Name: (6,6'-Dimethoxy-2,2'-[4,5-dimethyl-o-phenylenebis(nitrilomethylidyne)]diphenolato)-nickel(ii)

Space Group:	P21/n	***Cell:***	***a*** 12.806(0)	***b*** 12.651(0)	***c*** 13.026(0)
Space Group No.:	14	***(Å,°)***	α 90.00	β 101.73(0)	γ 90.00

R-Factor (%): 4.68 ***Temperature(K):*** 296 ***Density(g/cm^3):*** 1.482

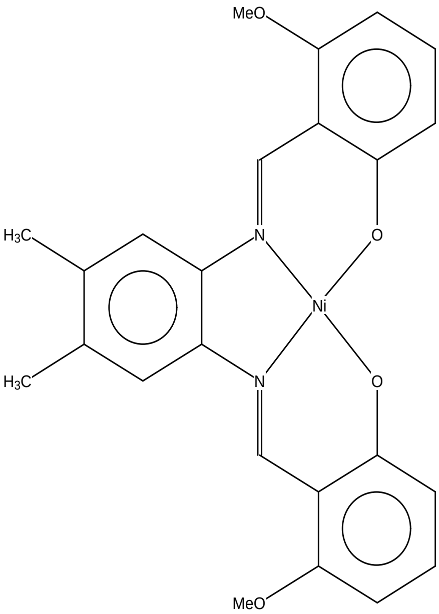

XALMAA

Reference: V.Mougel, J.Pecaut, M.Mazzanti (2012) *Chem.Commun.* , **48,**868

Formula: $C_{120}\ H_{134}\ N_{10}\ O_{16}\ U_5,4(C_2\ H_3\ N_1)$

Compound Name: (μ_3-oxo)-bis(μ_2-2,2'-(ethane-1,2-diylbis(nitrilomethylylidene)) diphenolato)-pentakis(μ_2-oxo)-bis(2,2'-(1,2-phenylenebis(nitrilomethylylidene))bis(4,6-di-t-butylphenolato))-(2,2'-(ethane-1,2-diylbis(nitrilomethylylidene))diphenolato)-penta-uranium acetonitrile solvate

Space Group:	P21/n	***Cell:***	***a*** 16.848(0)	***b*** 31.306(1)	***c*** 24.995(1)
Space Group No.:	14	***(Å,°)***	α 90.00	β 104.44(0)	γ 90.00

R-Factor (%): 10.38 ***Temperature(K):*** 150 ***Density(g/cm^3):*** 1.731

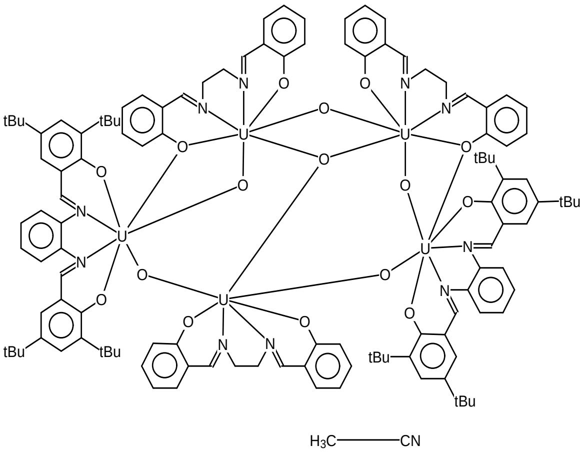

XARZUN

Reference: A.L.Spek, D.M.Tooke, M.Kuil, P.W.N.M.van Leeuwen (2011) *Private Communication* ,

Formula: $C_{59}\ H_{68}\ La_1\ N_7\ O_{15}\ Zn_2,5(C_3\ H_6\ O_1)$

Compound Name: bis(μ_2-2,2'-(1,2-Phenylenebis(nitrilomethylylidene))bis(4-t-butylphenolato))-bis(nitrato-O,O')-(nitrato-O)-aqua-(acetone)-lanthanum-di-zinc acetone solvate

Space Group:	P-1	***Cell:***	***a*** 12.228(1)	***b*** 14.547(0)	***c*** 25.133(0)
Space Group No.:	2	***(Å,°)***	α 95.56(0)	β 103.66(0)	γ 111.06(0)

R-Factor (%): 2.71 ***Temperature(K):*** 150 ***Density(g/cm^3):*** 1.401

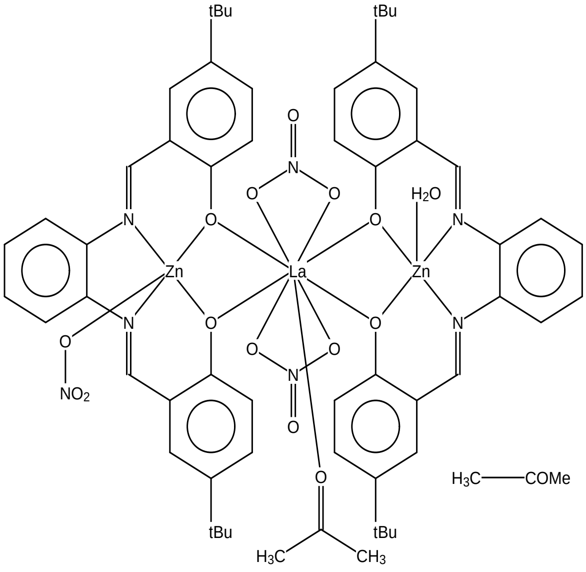

XAVWOG

Reference: R.Baggio, M.T.Garland, A.M.Atria, O.Hidalgo, S.Solezi (2001) *Acta Crystallogr.,Sect.E:Struct.Rep.Online* ,**57,**m19

Formula: $C_{20}\ H_{14}\ Cu_1\ N_2\ O_2,C_1\ H_2\ Cl_2$

Compound Name: (2,2'-(1,2-Phenylenebis(nitrilomethylidyne))diphenolato)copper(ii) dichloromethane solvate

Space Group:	Cc	***Cell:***	***a*** 15.787(3)	***b*** 14.359(3)	***c*** 9.163(1)
Space Group No.:	9	***(Å,°)***	α 90.00	β 111.59(2)	γ 90.00

R-Factor (%): 4.80 ***Temperature(K):*** 295 ***Density(g/cm^3):*** 1.592

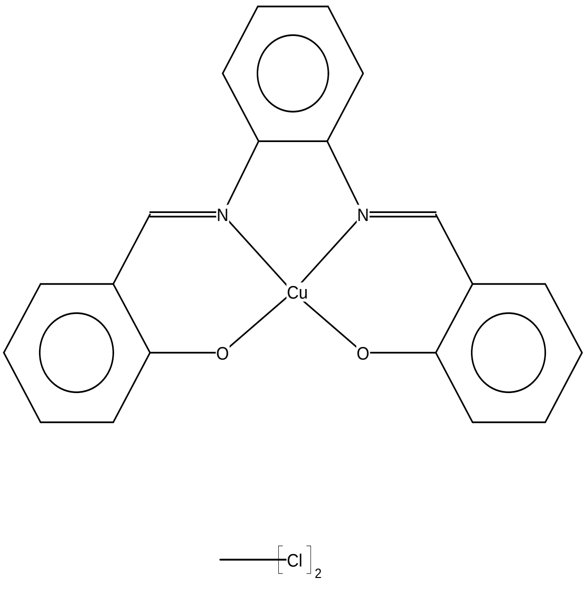

XAVYOK

Reference: N.E.Eltayeb, Siang Guan Teoh, R.Adnan, J.B.-J.Teh, H.-K.Fun (2011) *J.Fluorescence* ,**21,**1393

Formula: $C_{27} H_{23} N_3 O_2 Zn_1,0.25(C_5 H_5 N_1)$

Compound Name: (2,2'-(1,2-Phenylenebis((nitrilo)methylylidene))bis(4-methylphenolato))-(pyridine)-zinc(ii) pyridine solvate

Space Group:	P-1	***Cell:***	***a*** 9.544(0)	***b*** 11.941(0)	***c*** 21.173(0)
Space Group No.:	2	***(Å,°)***	α 89.78(0)	β 82.46(0)	γ 87.69(0)

R-Factor (%): 5.02 ***Temperature(K):*** 297 ***Density(g/cm3):*** 1.408

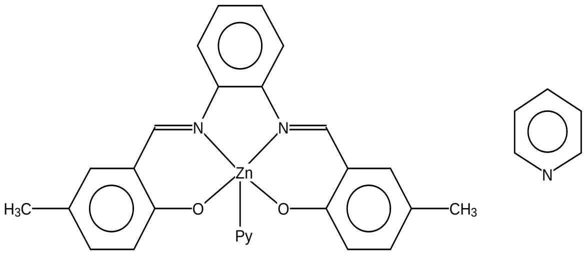

XELKUV

Reference: M.R.Bermejo, M.I.Fernandez, A.M.Gonzalez-Noya, M.Maneiro, R.Pedrido, M.J.Rodriguez, J.C.Garcia-Monteagudo, B.Donnadieu (2006) *J.Inorg.Biochem.* ,**100,**1470

Formula: $C_{25} H_{25} Mn_1 N_2 O_7,0.5(C_1 H_4 O_1)$

Compound Name: Aqua-(N,N'-bis(2-hydroxy-3-methoxybenzylidene)benzene-1,2-diamine)-(propanoato)-manganese(iii) methanol solvate

Space Group:	P-1	***Cell:***	***a*** 11.028(5)	***b*** 14.238(5)	***c*** 15.824(5)
Space Group No.:	2	***(Å,°)***	α 97.90(0)	β 92.81(0)	γ 101.86(0)

R-Factor (%): 6.14 ***Temperature(K):*** 150 ***Density(g/cm3):*** 1.484

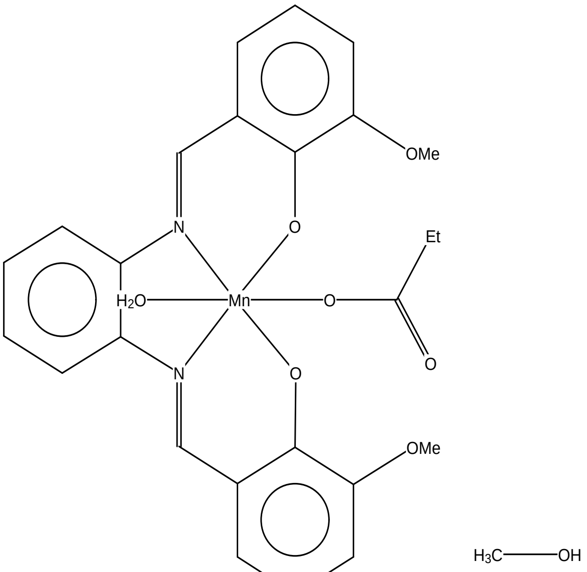

XELLAC

Reference: M.R.Bermejo, M.I.Fernandez, A.M.Gonzalez-Noya, M.Maneiro, R.Pedrido, M.J.Rodriguez, J.C.Garcia-Monteagudo, B.Donnadieu (2006) *J.Inorg.Biochem.* ,**100,**1470

Formula: $C_{28} H_{31} Mn_1 N_2 O_7$

Compound Name: Aqua-(N,N'-bis(2-hydroxy-3-methoxybenzylidene)benzene-1,2-diamine)-(hexanoato)-manganese(iii)

Space Group:	R-3	***Cell:***	***a*** 35.590(5)	***b*** 35.590(5)	***c*** 11.025(2)
Space Group No.:	148	***(Å,°)***	α 90.00	β 90.00	γ 120.00

R-Factor (%): 4.32 ***Temperature(K):*** 180 ***Density(g/cm3):*** 1.390

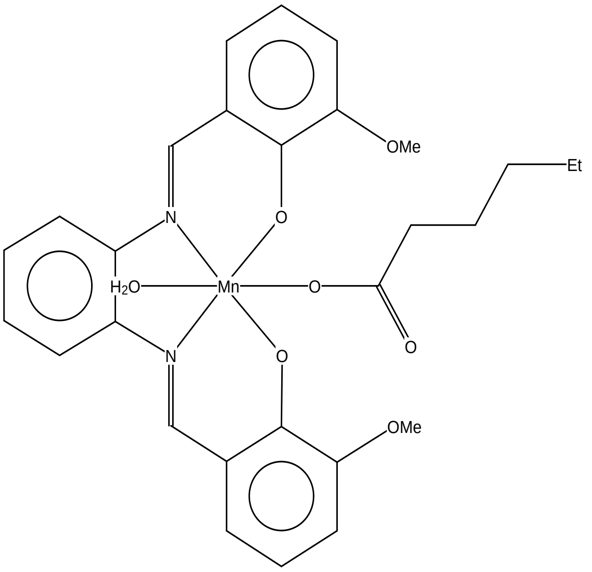

XEZQOI

Reference: M.Amirnasr, K.J.Schenk, A.Gorji, R.Vafazadeh (2001) *Polyhedron* ,**20,**695

Formula: $C_{28} H_{32} Co_1 N_4 O_4{}^{1+},Cl_1 O_4{}^{1-}$

Compound Name: bis(Morpholine-N)-(N,N'-disalicylidene-1,2-phenylenediamine-N,N',O,O')-cobalt(iii) perchlorate

Space Group:	P-1	***Cell:***	***a*** 9.447(0)	***b*** 10.280(0)	***c*** 15.002(1)
Space Group No.:	2	***(Å,°)***	α 106.49(0)	β 91.19(0)	γ 98.73(0)

R-Factor (%): 4.57 ***Temperature(K):*** 293 ***Density(g/cm3):*** 1.560

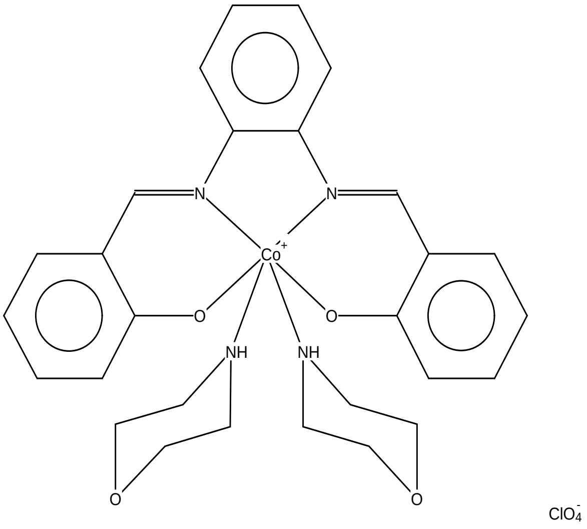

Search: search26 (Tue Dec 2 15:48:50 2014): Hits 661-664

XEZQUO

Reference: M.Amirnasr, K.J.Schenk, A.Gorji, R.Vafazadeh (2001) *Polyhedron* ,**20,**695

Formula: $C_{28}\ H_{32}\ Co_1\ N_4\ O_2\ ^{1+}, Cl_1\ O_4\ ^{1-}$

Compound Name: bis(Pyrrolidine-N)-(N,N'-disalicylidene-1,2-phenylenediamine-N,N',O,O')-cobalt(iii) perchlorate

Space Group:	I41/a	***Cell:***	***a*** 31.713(2)	***b*** 31.713(2)	***c*** 10.750(0)
Space Group No.:	88	***(Å,°)***	α 90.00	β 90.00	γ 90.00
R-Factor (%):	4.98	***Temperature(K):*** 293	***Density(g/cm³):*** 1.511		

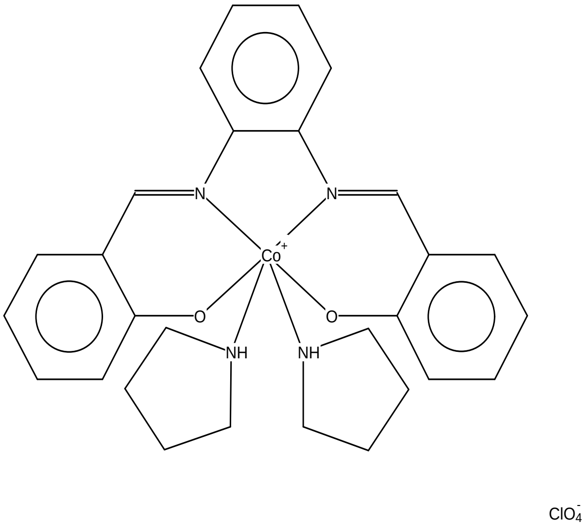

XIGGIE

Reference: Wen-Juan Ruan, Guo-Hang Hu, Shu-Jun Wang, Jian-Hua Tian, Qing-Lun Wang, Zhi-Ang Zhu (2005) *Chin.J.Chem.* ,**23,** 709

Formula: $C_{72}\ H_{92}\ Fe_2\ N_4\ O_5, C_1\ H_4\ O_1$

Compound Name: (μ_2-Oxo)-bis(N,N'-o-phenylene-bis(3,5-di-t-butylsalicylideneiminato))-di-iron(iii) methanol solvate

Space Group:	P-1	***Cell:***	***a*** 14.278(4)	***b*** 15.303(4)	***c*** 18.027(5)
Space Group No.:	2	***(Å,°)***	α 102.38(0)	β 103.04(0)	γ 100.53(0)
R-Factor (%):	6.12	***Temperature(K):*** 293	***Density(g/cm³):*** 1.130		

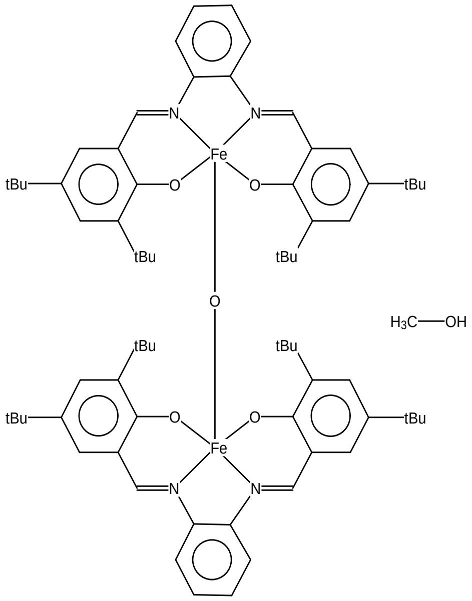

XISBEH

Reference: N.E.Eltayeb, Siang Guan Teoh, S.Chantrapromma, Hoong-Kun Fun, R.Adnan (2008) *Acta Crystallogr.,Sect.E:Struct.Rep.Online* ,**64,**m124

Formula: $C_{21}\ H_{16}\ Cl_3\ Mn_1\ N_2\ O_3$

Compound Name: Chloro-(4,4'-dichloro-2,2'-(1,2-phenylenebis(nitrilomethylidyne)) diphenolato-κ⁴O,N,N',O')-(methanol-κO)-manganese(iii)

Space Group:	P21/c	***Cell:***	***a*** 15.918(0)	***b*** 6.631(0)	***c*** 23.340(0)
Space Group No.:	14	***(Å,°)***	α 90.00	β 124.67(0)	γ 90.00
R-Factor (%):	4.18	***Temperature(K):*** 100	***Density(g/cm³):*** 1.658		

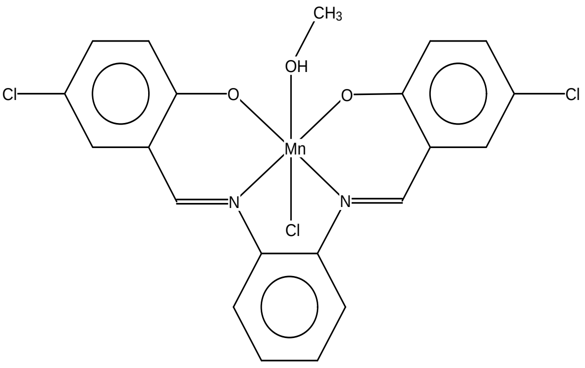

XIYJUL

Reference: N.E.Eltayeb, S.G.Teoh, S.Chantrapromma, H.-K.Fun, R.Adnan (2008) *Acta Crystallogr.,Sect.E:Struct.Rep.Online* ,**64,**m535

Formula: $C_{22}\ H_{18}\ Cl_1\ Mn_1\ N_2\ O_2, H_2\ O_1$

Compound Name: Chlorido-(6,6'-dimethyl-2,2'-(1,2-phenylenebis(nitrilomethylidyne)) diphenolato-κ⁴O,N,N',O')-manganese(iii) monohydrate

Space Group:	C2/c	***Cell:***	***a*** 27.184(0)	***b*** 6.803(0)	***c*** 21.890(0)
Space Group No.:	15	***(Å,°)***	α 90.00	β 108.98(0)	γ 90.00
R-Factor (%):	3.70	***Temperature(K):*** 100	***Density(g/cm³):*** 1.564		

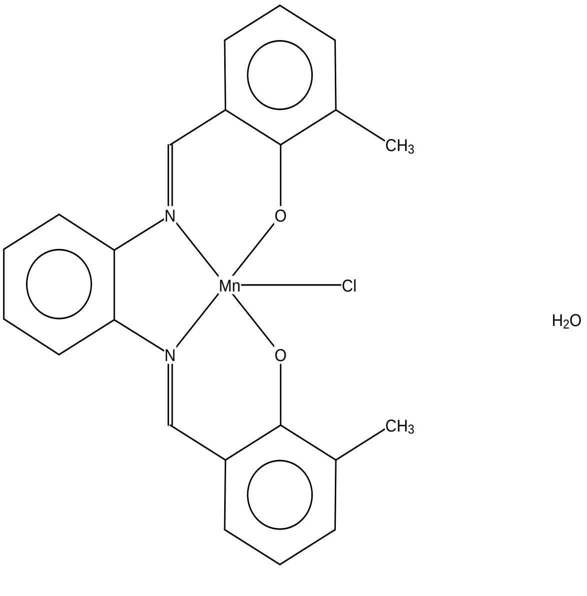

Search: search26 (Tue Dec 2 15:48:50 2014): Hits 665-668

XIYQOM

Reference: N.E.Eltayeb, S.G.Teoh, S.Chantrapromma, H.-K.Fun, R.Adnan (2008) *Acta Crystallogr.,Sect.E:Struct.Rep.Online* ,**64,**m670

Formula: $C_{22}\,H_{18}\,Cl_1\,Mn_1\,N_2\,O_4$

Compound Name: Chloro-(5,5'-dimethoxy-2,2'-(1,2-phenylenebis(nitrilomethylidyne))diphenolato-κ4O,N,N',O')-manganese(iii)

Space Group: Pbca ***Cell:*** ***a*** 13.728(0) ***b*** 15.025(0) ***c*** 19.209(0)
Space Group No.: 61 ***(Å,°)*** α 90.00 β 90.00 γ 90.00

R-Factor (%): 3.83 ***Temperature(K):*** 296 ***Density(g/cm3):*** 1.558

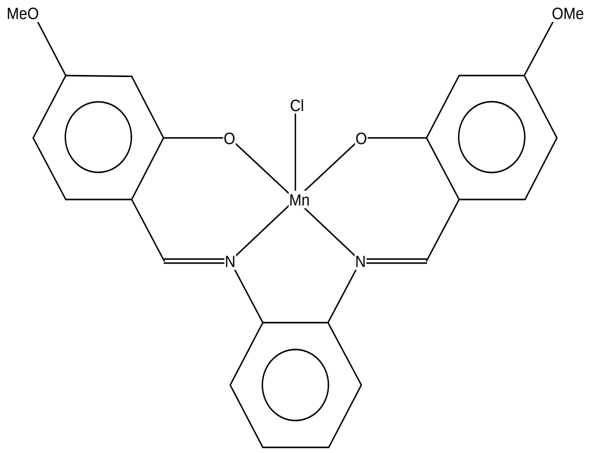

XOKSAS

Reference: Yuhua Feng, Jinxia Xu, Daizheng Liao, Shiping Yan, Zonghui Jiang (2008) *J.Coord.Chem.* ,**61,**3568

Formula: $C_{70}\,H_{52}\,Mn_2\,N_{10}\,O_4\,^{2+}$,$2(C_{24}\,H_{20}\,B_1\,^{1-})$

Compound Name: (μ2-4,4'-Bipyridine-N,N')-bis(4,4'-bipyridine-N)-bis(N,N'-o-phenylenebis(salicylideneaminato)-N,N',O,O')-di-manganese(iii) bis(tetraphenylborate)

Space Group: P-1 ***Cell:*** ***a*** 13.431(4) ***b*** 13.791(4) ***c*** 13.886(4)
Space Group No.: 2 ***(Å,°)*** α 73.60(0) β 80.41(0) γ 71.24(0)

R-Factor (%): 4.61 ***Temperature(K):*** 294 ***Density(g/cm3):*** 1.316

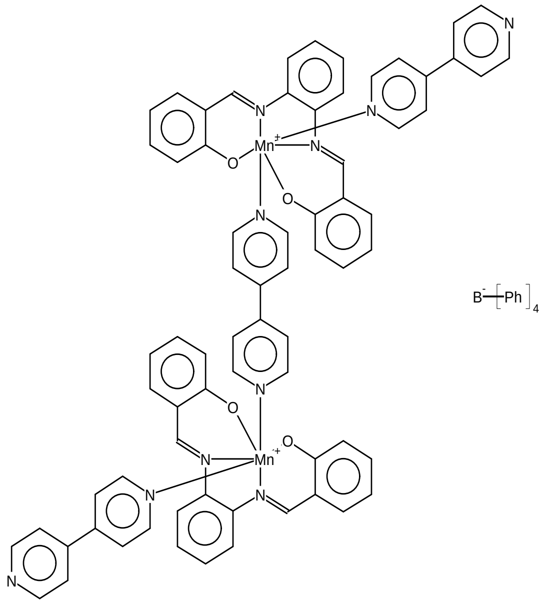

XUJRUQ

Reference: K.Agapiou, M.L.Mejia, Xiaoping Yang, B.J.Holliday (2009) *Dalton Trans.* ,4154

Formula: $C_{46}\,H_{40}\,Br_4\,Cd_2\,N_4\,O_{10}$,$2(H_2\,O_1)$

Compound Name: bis(μ2-N,N'-(1,2-Phenylenyl)-bis(5-bromo-2-oxy-3-methoxybenzaldimine)-N,N',O,O,O',O'')-bis(methanol)-di-cadmium(ii) dihydrate

Space Group: P-1 ***Cell:*** ***a*** 9.662(1) ***b*** 11.470(2) ***c*** 12.405(3)
Space Group No.: 2 ***(Å,°)*** α 70.93(3) β 71.84(2) γ 71.28(3)

R-Factor (%): 4.68 ***Temperature(K):*** 153 ***Density(g/cm3):*** 1.927

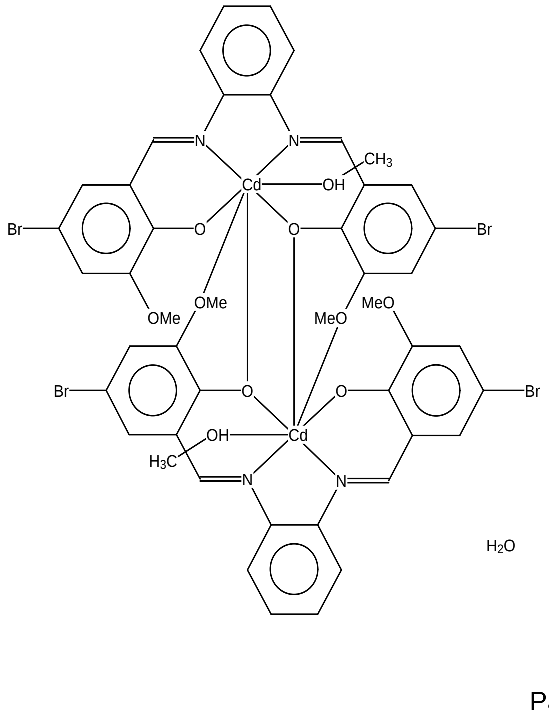

XUWXIW

Reference: M.S.Hill, D.A.Atwood (1998) *Main Group Chem.* ,**2,**191

Formula: $C_{38}\,H_{51}\,In_1\,N_2\,O_2$

Compound Name: ethyl-(N,N'-phenylenebis(3,5-di-t-butylsalicylideneiminato))-indium

Space Group: Pbcm ***Cell:*** ***a*** 15.847(0) ***b*** 9.831(0) ***c*** 23.636(1)
Space Group No.: 57 ***(Å,°)*** α 90.00 β 90.00 γ 90.00

R-Factor (%): 3.71 ***Temperature(K):*** 298 ***Density(g/cm3):*** 1.231

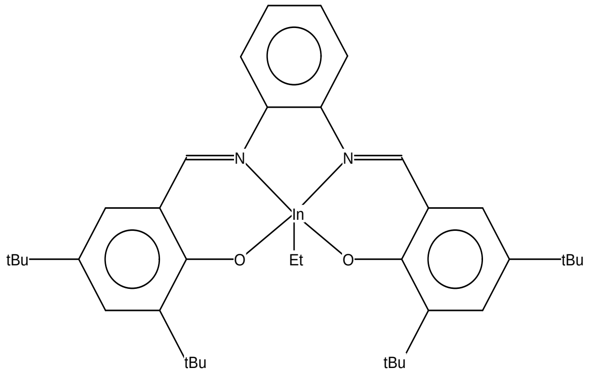

Search: search26 (Tue Dec 2 15:48:50 2014): Hits 669-672

XUXQUD

Reference: V.Bereau, V.Jubera, P.Arnaud, A.Kaiba, P.Guionneau, J.-P.Sutter (2010) *Dalton Trans.* ,**39,**2070

Formula: $C_{40} H_{40} Al_1 N_4 O_8$ $^{1+}$,$N_1 O_3$ $^{1-}$,3($H_2 O_1$)

Compound Name: (N,N'-bis(3-t-Butoxycarbonylsalicylidene)-1,2-phenylenediamine)-bis(pyridine N-oxide-O)-aluminium nitrate trihydrate

Space Group:	P-1	***Cell:***	***a*** 12.256(3)	***b*** 12.790(3)	***c*** 14.959(4)
Space Group No.:	2	***(Å,°)***	α 68.74(1)	β 70.91(1)	γ 81.62(1)

R-Factor (%): 7.35 ***Temperature(K):*** 150 ***Density(g/cm^3):*** 1.364

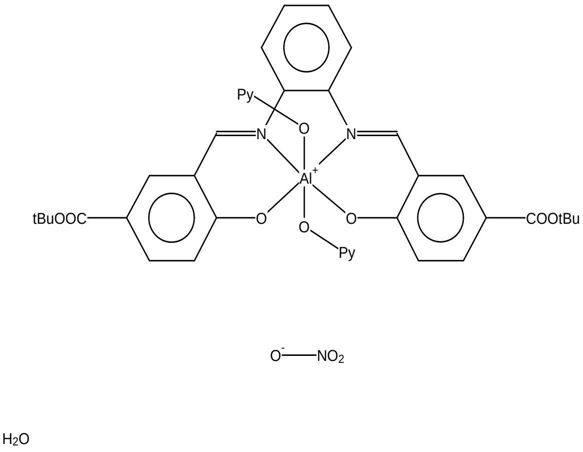

YACSEC

Reference: R.M.Haak, A.Decortes, E.C.Escudero-Adan, M.M.Belmonte, E.Martin, J.Benet-Buchholz, A.W.Kleij (2011) *Inorg.Chem.* ,**50,**7934

Formula: $C_{84} H_{56} Cl_8 N_8 O_{20} Zn_8$,$C_3 H_7 N_1 O_1$,3($C_1 H_4 O_1$),$C_{83} H_{54} Cl_8 N_8 O_{20} Zn_8$

Compound Name: tetrakis(μ_4-3,3'-((4,5-dichloro-1,2-phenylene)bis(nitrilomethylylidene))-bis(benzene-1,2-diolato))-aqua-tris(methanol)-octa-zinc tetrakis(μ_4-3,3'-((4,5-dichloro-1,2-phenylene)bis(nitrilomethylylidene))-bis(benzene-1,2-diolato))-tetrakis(methanol)-octa-zinc N,N-dimethylformamide methanol solvate

Space Group:	P21/c	***Cell:***	***a*** 18.098(0)	***b*** 31.825(0)	***c*** 35.489(1)
Space Group No.:	14	***(Å,°)***	α 90.00	β 96.92(0)	γ 90.00

R-Factor (%): 4.98 ***Temperature(K):*** 100 ***Density(g/cm^3):*** 1.559

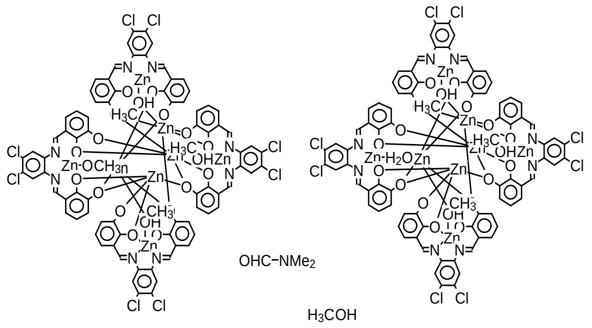

YACSOM

Reference: R.M.Haak, A.Decortes, E.C.Escudero-Adan, M.M.Belmonte, E.Martin, J.Benet-Buchholz, A.W.Kleij (2011) *Inorg.Chem.* ,**50,**7934

Formula: $C_{80} H_{56} N_8 O_{20} Zn_8$,4($C_3 H_7 N_1 O_1$)

Compound Name: tetrakis(μ_4-3,3'-((1,2-phenylene)bis(nitrilomethylylidene))-bis(benzene-1,2-diolato))-tetraaqua-octa-zinc N,N-dimethylformamide solvate

Space Group:	P42/n	***Cell:***	***a*** 18.219(1)	***b*** 18.219(1)	***c*** 13.450(1)
Space Group No.:	86	***(Å,°)***	α 90.00	β 90.00	γ 90.00

R-Factor (%): 3.88 ***Temperature(K):*** 100 ***Density(g/cm^3):*** 1.685

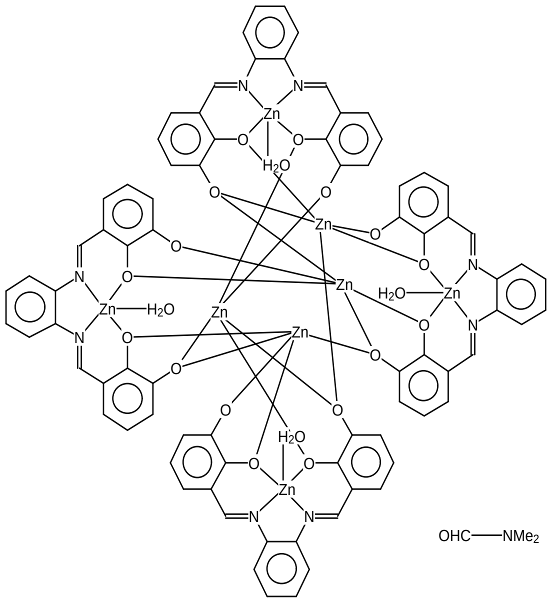

YACTAZ

Reference: A.Tzubery, E.Y.Tshuva (2011) *Inorg.Chem.* ,**50,**7946

Formula: $C_{36} H_{30} Cl_2 N_2 O_4 Ti_1$

Compound Name: bis(2,6-dimethylphenolato)-(2,2'-(1,2-phenylenebis((nitrilo)methylylidene))bis(4-chlorophenolato))-titanium

Space Group:	P21/n	***Cell:***	***a*** 13.102(1)	***b*** 14.176(1)	***c*** 17.061(1)
Space Group No.:	14	***(Å,°)***	α 90.00	β 101.42(0)	γ 90.00

R-Factor (%): 5.14 ***Temperature(K):*** 173 ***Density(g/cm^3):*** 1.440

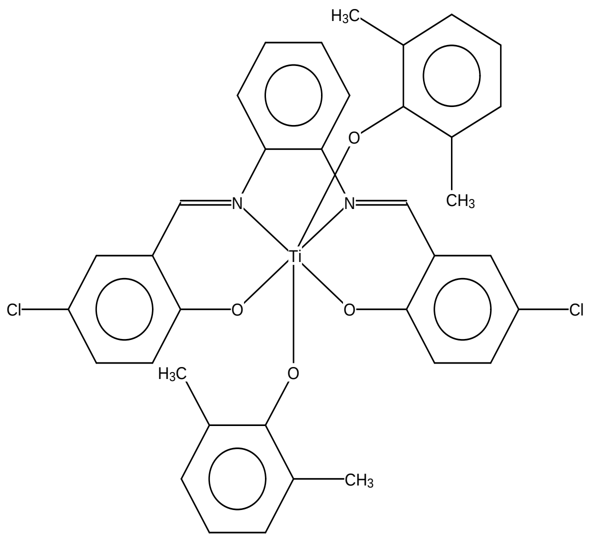

YACTED

Reference:	A.Tzubery, E.Y.Tshuva (2011) *Inorg.Chem.* ,**50,**7946
Formula:	$C_{36} H_{30} Cl_2 N_2 O_4 Ti_1,C_8 H_{10} O_1$
Compound Name:	bis(2,6-dimethylphenolato)-(2,2'-(1,2-phenylenebis((nitrilo)methylylidene))bis(6-chlorophenolato))-titanium 2,6-dimethylphenol solvate

Space Group:	P21/c	***Cell:***	***a*** 11.522(1)	***b*** 19.975(3)	***c*** 17.306(3)
Space Group No.:	14	***(Å,°)***	α 90.00	β 106.10(0)	γ 90.00
R-Factor (%):	6.80	***Temperature(K):*** 173	***Density(g/cm³):*** 1.381		

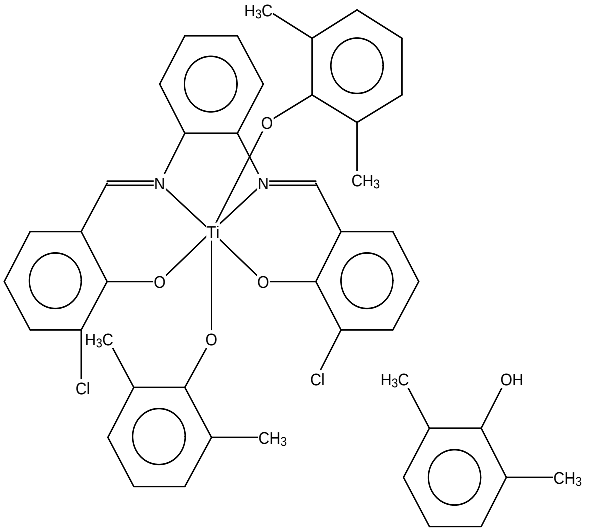

YADMOH

Reference:	A.Ghaemi, K.Fayyazi, B.Keyvani, S.W.Ng, E.R.T.Tiekink (2011) *Acta Crystallogr.,Sect.E:Struct.Rep.Online* ,**67,**m1481
Formula:	$C_{20} H_{14} N_2 Ni_1 O_2,H_2 O_1$
Compound Name:	(2,2'-(o-Phenylenebis(nitrilomethanylylidene))diphenolato-O,N,N',O')-nickel(ii) monohydrate

Space Group:	R-3	***Cell:***	***a*** 31.552(1)	***b*** 31.552(1)	***c*** 9.026(0)
Space Group No.:	148	***(Å,°)***	α 90.00	β 90.00	γ 120.00
R-Factor (%):	4.06	***Temperature(K):*** 294	***Density(g/cm³):*** 1.502		

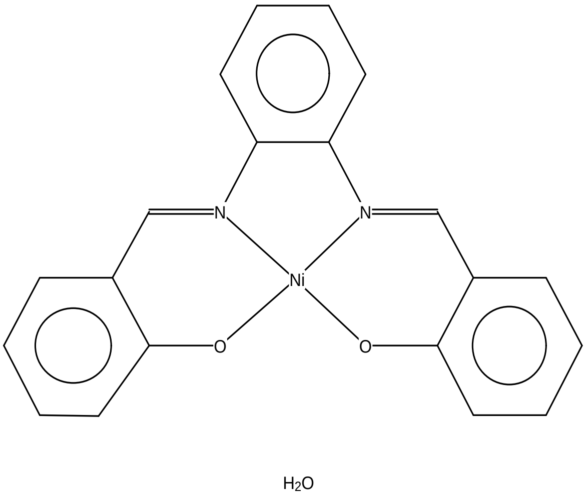

YAJVAI

Reference:	Xiao-song Zhang, Guo-hua Ding, Run-mei Fan (2010) *Hecheng Huaxue(Chin.)(Chin.J.Synth.Chem.)* ,**18,**67
Formula:	$C_{28} H_{18} Cu_1 N_2 O_2,C_3 H_7 N_1 O_1$
Compound Name:	(1,1'-(1,2-Phenylenebis((nitrilo)methylylidene))bis(2-naphtholato))-copper(ii) N,N-dimethylformamide solvate

Space Group:	P21/n	***Cell:***	***a*** 11.046(1)	***b*** 13.672(1)	***c*** 16.684(1)
Space Group No.:	14	***(Å,°)***	α 90.00	β 103.74(0)	γ 90.00
R-Factor (%):	7.90	***Temperature(K):*** 298	***Density(g/cm³):*** 1.496		

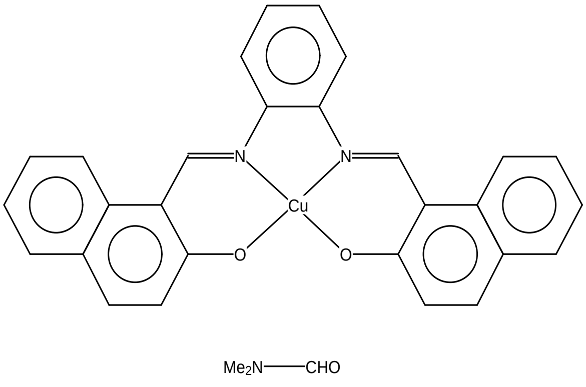

YALRAF

Reference:	M.Cametti, M.Nissinen, A.D.Cort, L.Mandolini, K.Rissanen (2005) *J.Am.Chem.Soc.* ,**127,**3831
Formula:	$C_{68} H_{52} Cs_2 F_2 N_4 O_{12} U_2,4(C_2 H_3 N_1)$
Compound Name:	bis((μ_3-N,N'-o-Phenylenebis(3-(benzyloxy)salicylideneaminato))-(μ_3-fluoro)-(μ_3-oxo)-oxo-cesium-uranium) acetonitrile solvate

Space Group:	P21/n	***Cell:***	***a*** 16.378(0)	***b*** 13.965(0)	***c*** 16.693(0)
Space Group No.:	14	***(Å,°)***	α 90.00	β 107.57(0)	γ 90.00
R-Factor (%):	2.68	***Temperature(K):*** 173	***Density(g/cm³):*** 1.881		

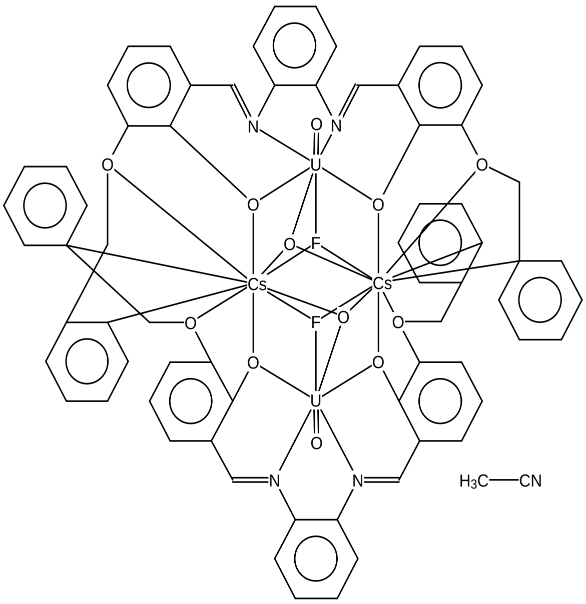

Search: search26 (Tue Dec 2 15:48:50 2014): Hits 677-680

YALREJ

Reference: M.Cametti, M.Nissinen, A.D.Cort, L.Mandolini, K.Rissanen (2005) *J.Am.Chem.Soc.* ,**127,**3831

Formula: $C_{68}\ H_{52}\ Cl_2\ Cs_2\ N_4\ O_{12}\ U_2,4(C_2\ H_3\ N_1)$

Compound Name: bis((μ_3-N,N'-o-Phenylenebis(3-(benzyloxy)salicylideneaminato))-(μ_3-chloro)-(μ_3-oxo)-oxo-cesium-uranium) acetonitrile solvate

Space Group:	P-1	***Cell:***	***a*** 12.034(0)	***b*** 12.362(0)	***c*** 14.584(0)
Space Group No.:	2	***(Å,°)***	α 108.14(0)	β 97.07(0)	γ 108.18(0)

R-Factor (%): 5.15 ***Temperature(K):*** 173 ***Density(g/cm³):*** 1.831

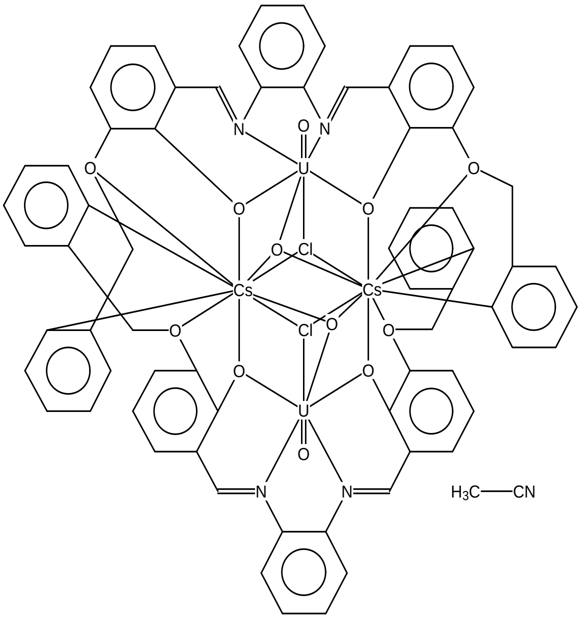

YALRIN

Reference: M.Cametti, M.Nissinen, A.D.Cort, L.Mandolini, K.Rissanen (2005) *J.Am.Chem.Soc.* ,**127,**3831

Formula: $C_{68}\ H_{52}\ Cl_2\ N_4\ O_{12}\ Rb_2\ U_2,4(C_2\ H_3\ N_1),0.5(H_2\ O_1)$

Compound Name: bis((μ_3-N,N'-o-Phenylenebis(3-(benzyloxy)salicylideneaminato))-(μ_3-chloro)-(μ_3-oxo)-oxo-rubidium-uranium) acetonitrile solvate hemihydrate

Space Group:	P-1	***Cell:***	***a*** 11.875(0)	***b*** 12.221(0)	***c*** 14.615(0)
Space Group No.:	2	***(Å,°)***	α 107.79(0)	β 96.73(0)	γ 108.30(0)

R-Factor (%): 5.82 ***Temperature(K):*** 173 ***Density(g/cm³):*** 1.790

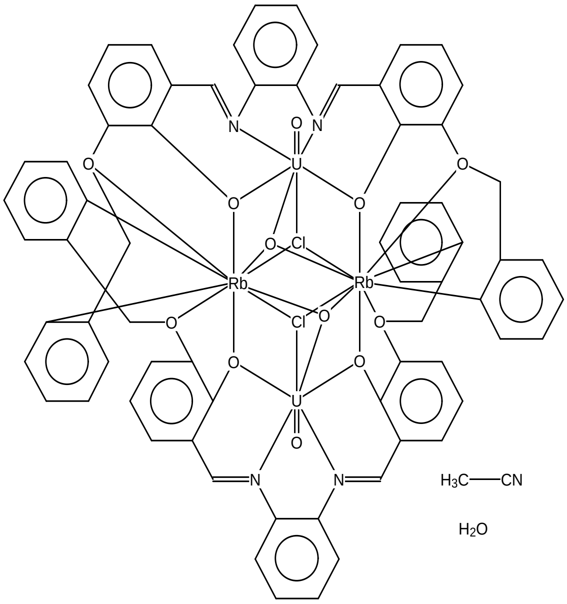

YALROT

Reference: M.Cametti, M.Nissinen, A.D.Cort, L.Mandolini, K.Rissanen (2005) *J.Am.Chem.Soc.* ,**127,**3831

Formula: $C_{68}\ H_{52}\ Cl_2\ K_2\ N_4\ O_{12}\ U_2,2(C_{35}\ H_{30}\ N_2\ O_7\ U_1),2(C_1\ H_4\ O_1)$

Compound Name: bis((μ_3-η^2-N,N'-o-Phenylenebis(3-(benzyloxy)salicylideneaminato))-(μ_3-chloro)-(μ_3-oxo)-oxo-potassium-uranium) bis((N,N'-o-phenylenebis(3-(benzyloxy)salicylideneaminato))-methanol-dioxo-uranium) methanol solvate

Space Group:	P-1	***Cell:***	***a*** 14.391(0)	***b*** 15.209(0)	***c*** 15.504(0)
Space Group No.:	2	***(Å,°)***	α 93.26(0)	β 107.14(0)	γ 100.84(0)

R-Factor (%): 3.84 ***Temperature(K):*** 173 ***Density(g/cm³):*** 1.819

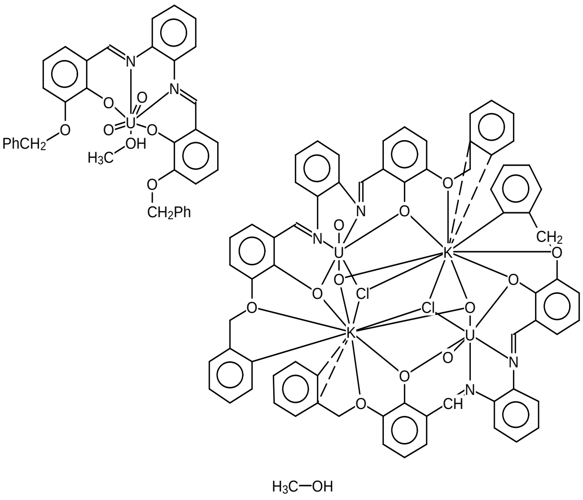

YALRUZ

Reference: M.Cametti, M.Nissinen, A.D.Cort, L.Mandolini, K.Rissanen (2005) *J.Am.Chem.Soc.* ,**127,**3831

Formula: $C_{110}\ H_{88}\ Cl_2\ Cs_2\ N_8\ O_{22}\ U_4,2(C_1\ H_4\ O_1),2(C_2\ H_3\ N_1),3(H_2\ O_1)$

Compound Name: bis((μ_3-η^2-N,N'-o-Phenylenebis(3-(benzyloxy)salicylideneaminato))-(μ_3-chloro)-(μ_3-oxo)-(μ_2-η^2-N,N'-o-phenylenebis(3-(benzyloxy) salicylideneaminato))-(μ_2-oxo)-methanol-dioxo-cesium-di-uranium) acetonitrile ethanol solvate trihydrate

Space Group:	C2/c	***Cell:***	***a*** 32.460(0)	***b*** 15.915(0)	***c*** 26.185(0)
Space Group No.:	15	***(Å,°)***	α 90.00	β 120.98(0)	γ 90.00

R-Factor (%): 3.43 ***Temperature(K):*** 173 ***Density(g/cm³):*** 1.926

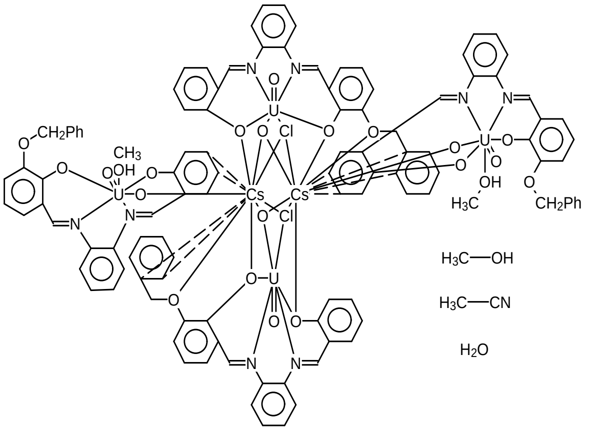

Search: search26 (Tue Dec 2 15:48:50 2014): Hits 681-684

YALSAG

Reference: M.Cametti, M.Nissinen, A.D.Cort, L.Mandolini, K.Rissanen (2005) *J.Am.Chem.Soc.* ,**127,**3831

Formula: $C_{36}\ H_{29}\ N_3\ O_6\ U_1$

Compound Name: (N,N'-o-Phenylenebis(3-(benzyloxy)salicylideneaminato))-acetonitrile-dioxo-uranium

Space Group:	Pnam	***Cell:***	***a*** 9.603(0)	***b*** 13.323(0)	***c*** 23.344(0)		
Space Group No.:	62	***(Å,°)***	α 90.00	β 90.00	γ 90.00		
R-Factor (%):	1.72	***Temperature(K):***	173	***Density(g/cm^3):***	1.863		

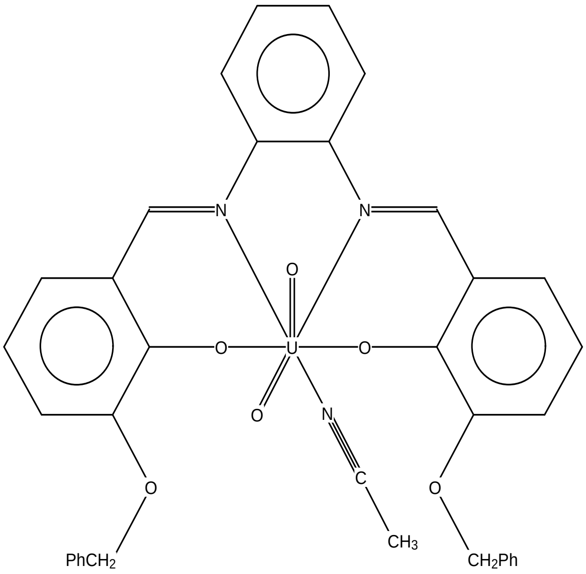

YALSEK

Reference: M.Cametti, M.Nissinen, A.D.Cort, L.Mandolini, K.Rissanen (2005) *J.Am.Chem.Soc.* ,**127,**3831

Formula: $C_{35}\ H_{30}\ N_2\ O_7\ U_1$

Compound Name: (N,N'-o-Phenylenebis(3-(benzyloxy)salicylideneaminato))-methanol-dioxo-uranium

Space Group:	Pnam	***Cell:***	***a*** 9.563(0)	***b*** 13.733(0)	***c*** 22.473(0)		
Space Group No.:	62	***(Å,°)***	α 90.00	β 90.00	γ 90.00		
R-Factor (%):	4.17	***Temperature(K):***	173	***Density(g/cm^3):***	1.865		

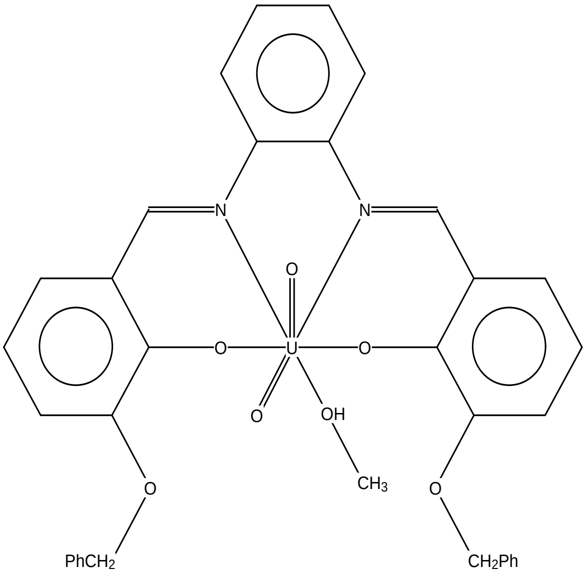

YALSIO

Reference: M.Cametti, M.Nissinen, A.D.Cort, L.Mandolini, K.Rissanen (2005) *J.Am.Chem.Soc.* ,**127,**3831

Formula: $C_{21}\ H_{18}\ N_2\ O_5\ U_1,C_{20}\ H_{16}\ N_2\ O_5\ U_1,C_1\ H_1\ Cl_3,C_1\ H_4\ O_1$

Compound Name: (N,N'-o-Phenylenebis(salicylideneaminato))-methanol-dioxo-uranium (N,N'-o-phenylenebis(salicylideneaminato))-aqua-dioxo-uranium chloroform methanol solvate

Space Group:	P-1	***Cell:***	***a*** 12.532(0)	***b*** 13.564(0)	***c*** 13.904(0)		
Space Group No.:	2	***(Å,°)***	α 99.61(0)	β 101.62(0)	γ 91.39(0)		
R-Factor (%):	3.99	***Temperature(K):***	173	***Density(g/cm^3):***	1.997		

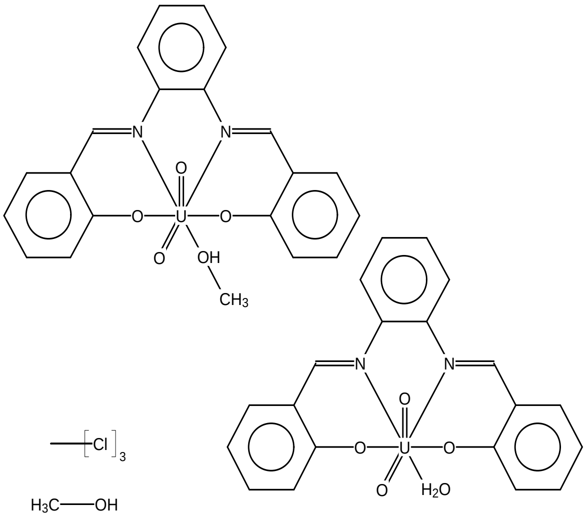

YALSOU

Reference: M.Cametti, M.Nissinen, A.D.Cort, L.Mandolini, K.Rissanen (2005) *J.Am.Chem.Soc.* ,**127,**3831

Formula: $C_{22}\ H_{17}\ N_3\ O_4\ U_1$

Compound Name: Acetonitrile-(N,N'-o-phenylenebis(salicylideneaminato)-dioxo-uranium)

Space Group:	Ccm21	***Cell:***	***a*** 12.573(0)	***b*** 16.883(0)	***c*** 9.479(0)		
Space Group No.:	36	***(Å,°)***	α 90.00	β 90.00	γ 90.00		
R-Factor (%):	1.96	***Temperature(K):***	173	***Density(g/cm^3):***	2.065		

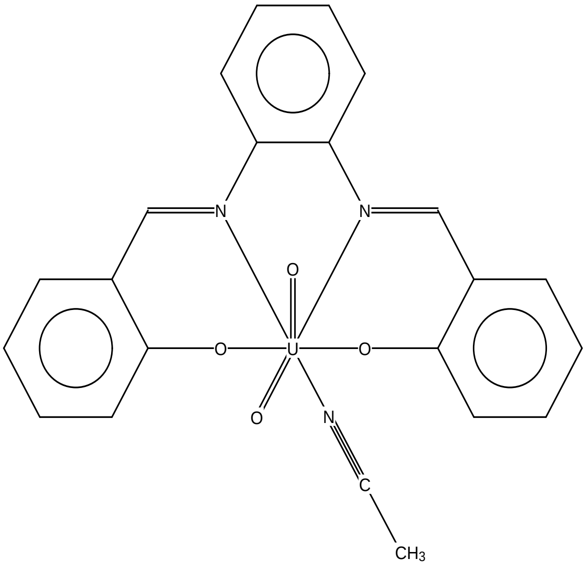

Search: search26 (Tue Dec 2 15:48:50 2014): Hits 685-688

YAZDUZ

Reference: H.Shimakoshi, T.Takemoto, I.Aritome, Y.Hisaeda (2005) *Inorg.Chem.* ,**44,**9134

Formula: $C_{70} H_{92} Co_2 N_8 O_8{}^{2+}$,$6(C_3 H_7 N_1 O_1)$,$2(Cl_1 O_4{}^{1-})$

Compound Name: bis(μ_2-2-(2-(2-aminoethoxy)ethoxy)ethamino-N,N')-(μ_2-5,20,26,41-tetrakis(t-butyl)-9,16,30,37-tetraazaheptacyclo(37.3.1.1^{3,7}.1^{18,22}.1^{24,28}.0^{10,15}.0^{31,36})hexatetraconta-1(43),3(46),4,6,8,10,12,14,16,18(45),19,21,24(44),25,27,29,31,33,35,37,39,41-docosaene-43,44,45,46-tetrolato)-di-cobalt(iii) bis(perchlorate) dimethylformamide solvate

Space Group:	P21/n	***Cell:***	***a*** 16.541(1)	***b*** 16.674(1)	***c*** 18.223(1)		
Space Group No.:	14	***(Å,°)***	α 90.00	β 105.62(0)	γ 90.00		
R-Factor (%):	6.78	***Temperature(K):***	100	***Density(g/cm³):***	1.323		

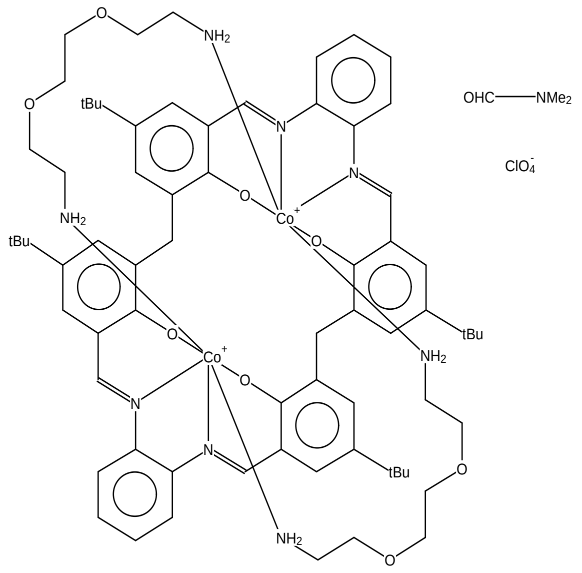

YAZFAH

Reference: H.Shimakoshi, T.Takemoto, I.Aritome, Y.Hisaeda (2005) *Inorg.Chem.* ,**44,**9134

Formula: $C_{76} H_{104} Co_2 N_8 O_4{}^{2+}$,$2(C_3 H_7 N_1 O_1)$,$2(C_1 F_3 O_3 S_1{}^{1-})$

Compound Name: bis(μ_2-9-aminononylamine-N,N')-(μ_2-5,20,26,41-tetrakis(t-butyl)-9,16,30,37-tetraazaheptacyclo(37.3.1.1^{3,7}.1^{18,22}.1^{24,28}.0^{10,15}.0^{31,36})hexatetraconta-1(43),3(46),4,6,8,10,12,14,16,18(45),19,21,24(44),25,27,29,31,33,35,37,39,41-docosaene-43,44,45,46-tetrolato)-di-cobalt(iii) bis(trifluoromethanesulfonate) dimethylformamide solvate

Space Group:	P-1	***Cell:***	***a*** 11.515(1)	***b*** 14.010(1)	***c*** 15.672(1)		
Space Group No.:	2	***(Å,°)***	α 65.82(0)	β 72.98(0)	γ 89.58(0)		
R-Factor (%):	7.13	***Temperature(K):***	100	***Density(g/cm³):***	1.333		

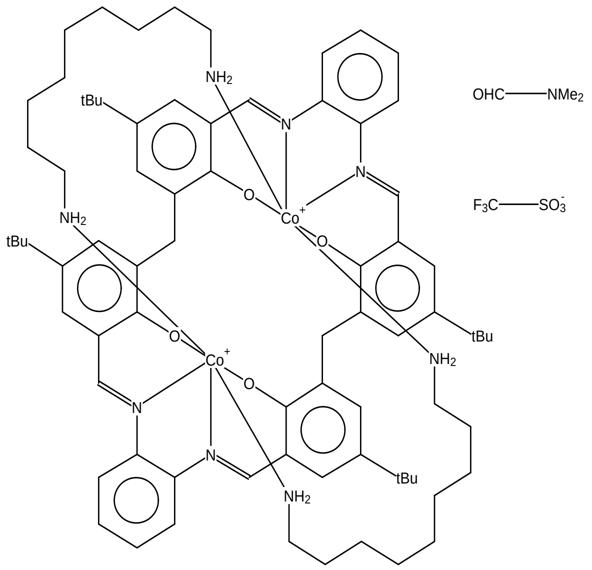

YECDEQ

Reference: M.P.Weberski Junior, C.C.McLauchlan, C.G.Hamaker (2006) *Polyhedron* ,**25,**119

Formula: $C_{36} H_{46} N_2 O_3 V_1$,$C_2 H_6 O_1$

Compound Name: (N,N'-Phenylene-bis(3,5-di-t-butylsalicylideneaminato))-oxo-vanadium(iv) ethanol solvate

Space Group:	P21/c	***Cell:***	***a*** 14.610(1)	***b*** 9.793(0)	***c*** 27.135(3)		
Space Group No.:	14	***(Å,°)***	α 90.00	β 116.13(0)	γ 90.00		
R-Factor (%):	7.73	***Temperature(K):***	100	***Density(g/cm³):***	1.242		

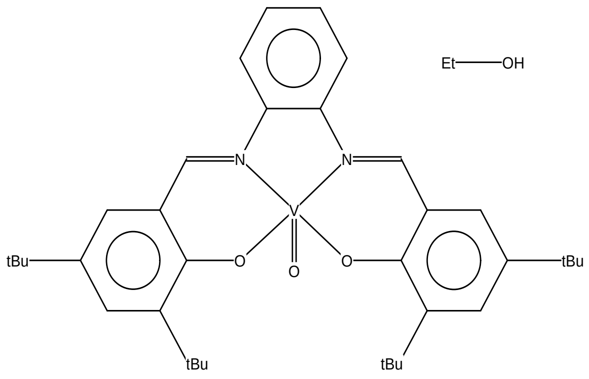

YEFLUQ

Reference: C.Floriani, E.Solari, F.Franceschi, R.Scopelliti, P.Belanzoni, M.Rosi (2001) *Chem.-Eur.J.* ,**7,**3052

Formula: $C_{72} H_{92} Mo_2 N_4 O_4$,$0.5(C_7 H_{16})$

Compound Name: bis(N,N'-o-Phenylene-bis(3,5-di-t-butylsalicylideneiminato))-di-molybdenum heptane solvate

Space Group:	P21/n	***Cell:***	***a*** 14.978(2)	***b*** 20.702(3)	***c*** 23.268(3)		
Space Group No.:	14	***(Å,°)***	α 90.00	β 104.66(1)	γ 90.00		
R-Factor (%):	7.25	***Temperature(K):***	173	***Density(g/cm³):***	1.256		

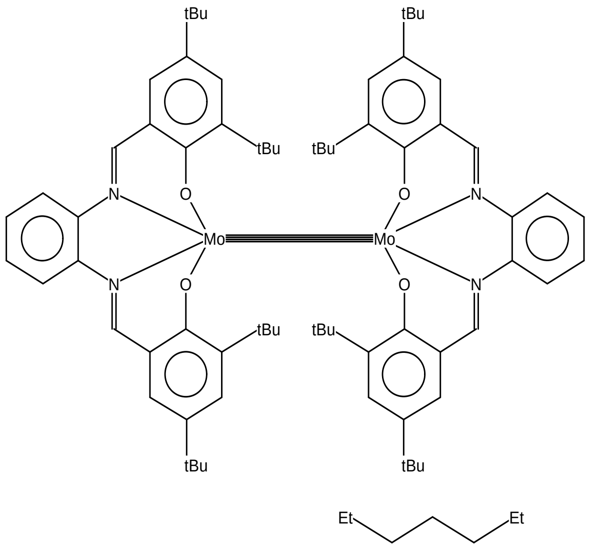

Search: search26 (Tue Dec 2 15:48:50 2014): Hits 689-692

YEGSUA

Reference: M.K.Koley, S.C.Sivasubramanian, B.Varghese, P.T.Manoharan, A.P.Koley (2012) *J.Coord.Chem.* ,**65,**3623

Formula: $C_{22} H_{14} Cr_1 N_4 O_2 S_2$

Compound Name: (2,2'-(1,2-Phenylenebis((nitrilo)methylylidene))diphenolato)-bis(thiocyanato)-chromium unknown solvate

Space Group: P-1 ***Cell:*** ***a*** 8.582(0) ***b*** 10.637(0) ***c*** 13.042(0)
Space Group No.: 2 ***(Å,°)*** α 81.62(0) β 87.06(0) γ 89.07(0)

R-Factor (%): 3.99 ***Temperature(K):*** 293 ***Density(g/cm^3):*** 1.362

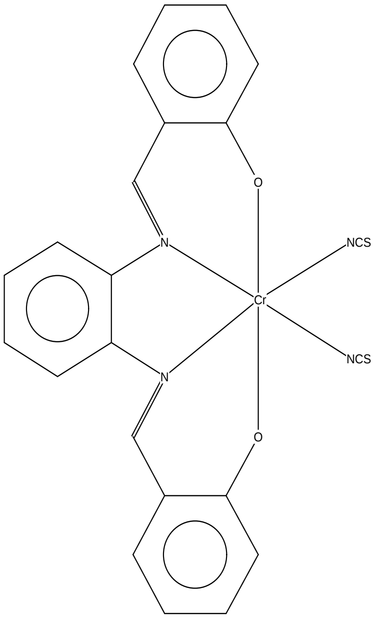

YELFEA

Reference: K.Brychcy, K.Drager, K.-J.Jens, M.Tilset, U.Behrens (1994) *Chem.Ber.* ,**127,**1817

Formula: $C_{36} H_{36} Co_1 N_4 O_2{}^{2+}$,2($Cl_1 O_4{}^{1-}$)

Compound Name: (14,29-Di-t-butyl-18H,25H-3,10,18,25-tetra-azapentacyclo(25.3.1.1^{12,16}.0^{4,9}.0^{19,24})dotriaconta-1(31),2,4,6,8,10,12,14,16(32),17,19,21,23,25,27,29-hexadecaene-31,32-diolato-N,N',O,O')-cobalt(ii) diperchlorate

Space Group: P21/c ***Cell:*** ***a*** 7.836(2) ***b*** 15.850(4) ***c*** 15.395(5)
Space Group No.: 14 ***(Å,°)*** α 90.00 β 103.61(2) γ 90.00

R-Factor (%): 5.90 ***Temperature(K):*** 295 ***Density(g/cm^3):*** 1.456

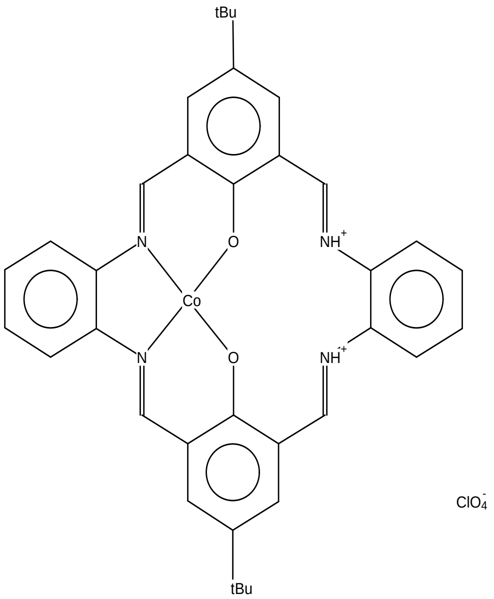

YENBEY

Reference: D.J.Anderson, R.Eisenberg (1994) *Inorg.Chem.* ,**33,**5378

Formula: $C_{40} H_{55} N_2 O_2 Rh_1$

Compound Name: n-Butyl-(benzene-1,2-bis(N-(4,6-di-t-butylsalicylaldiminato))-O,O',N,N')-rhodium(iii)

Space Group: P21/a ***Cell:*** ***a*** 12.031(8) ***b*** 25.540(6) ***c*** 12.780(10)
Space Group No.: 14 ***(Å,°)*** α 90.00 β 97.05(4) γ 90.00

R-Factor (%): 6.24 ***Temperature(K):*** 243 ***Density(g/cm^3):*** 1.191

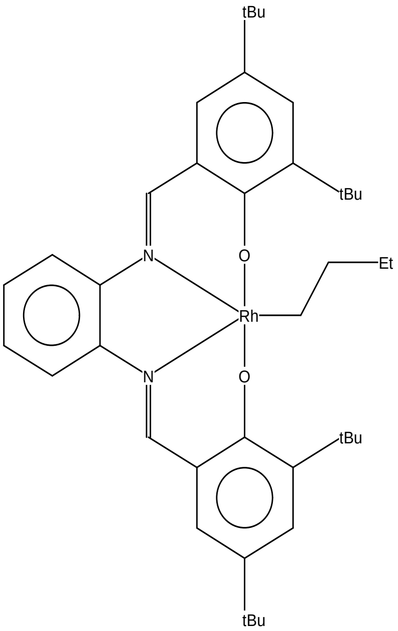

YIDROT

Reference: N.E.Eltayeb, S.G.Teoh, J.Bee-Jan Teh, Hoong-Kun Fun, K.Ibrahim (2007) *Acta Crystallogr.,Sect.E:Struct.Rep.Online* ,**63,**m1764

Formula: $C_{25} H_{17} Br_2 N_3 O_2 Zn_1$

Compound Name: (1,2-Phenylene-bis(5-bromosalicylaldiminato))-(pyridine-N)-zinc(ii)

Space Group: P-1 ***Cell:*** ***a*** 8.024(0) ***b*** 12.793(0) ***c*** 22.477(0)
Space Group No.: 2 ***(Å,°)*** α 91.99(0) β 94.23(0) γ 103.57(0)

R-Factor (%): 2.61 ***Temperature(K):*** 100 ***Density(g/cm^3):*** 1.834

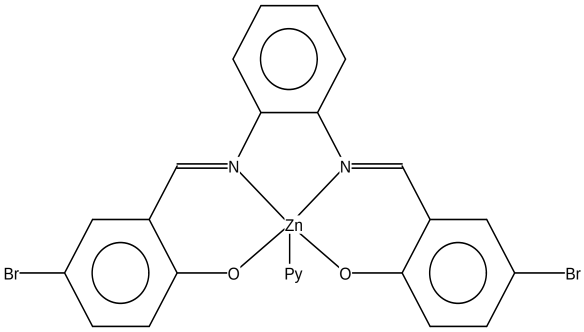

Search: search26 (Tue Dec 2 15:48:50 2014): Hits 693-696

YIDWEO

Reference: N.E.Eltayeb, S.G.Teoh, S.Chantrapromma, Hoong-Kun Fun, K.Ibrahim (2007) *Acta Crystallogr.,Sect.E:Struct.Rep.Online* ,**63,**m1672

Formula: $C_{25}\,H_{17}\,Cl_2\,N_3\,O_2\,Zn_1$

Compound Name: (4,4'-Dichloro-2,2'-(1,2-phenylenebis(nitrilomethylidyne))diphenolato-κ^{4}O, N,N',O')-(pyridine-κN)-zinc(ii)

Space Group:	P-1	***Cell:***	***a*** 7.984(0)	***b*** 12.663(0)	***c*** 22.316(1)
Space Group No.:	2	***(Å,°)***	α 91.57(0)	β 94.15(0)	γ 103.79(0)

R-Factor (%): 3.91 ***Temperature(K):*** 100 ***Density(g/cm^3):*** 1.606

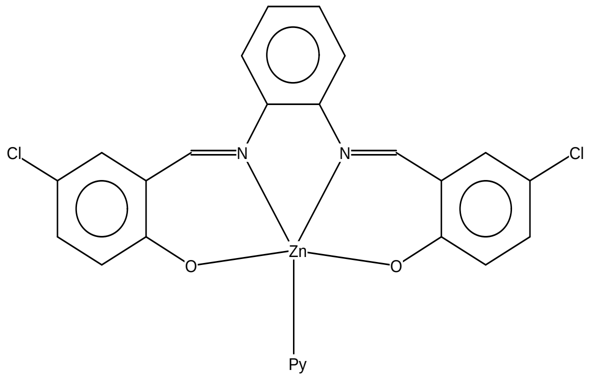

YIYLIC

Reference: Feng Luo, Ji-min Zheng, M.Kurmoo (2007) *Inorg.Chem.* , **46,**8448

Formula: $C_{80}\,H_{56}\,Cl_8\,N_8\,Ni_8\,O_8$,0.5($H_2\,O_1$)

Compound Name: hexakis(μ_2-Chloro)-tetrakis(μ_2-N,N'-o-phenylene-bis(salicylideneimino)) -dichloro-octa-nickel(ii) hemihydrate

Space Group:	P-1	***Cell:***	***a*** 12.543(3)	***b*** 12.896(3)	***c*** 13.782(3)
Space Group No.:	2	***(Å,°)***	α 97.06(3)	β 105.64(3)	γ 115.01(3)

R-Factor (%): 6.88 ***Temperature(K):*** 293 ***Density(g/cm^3):*** 1.791

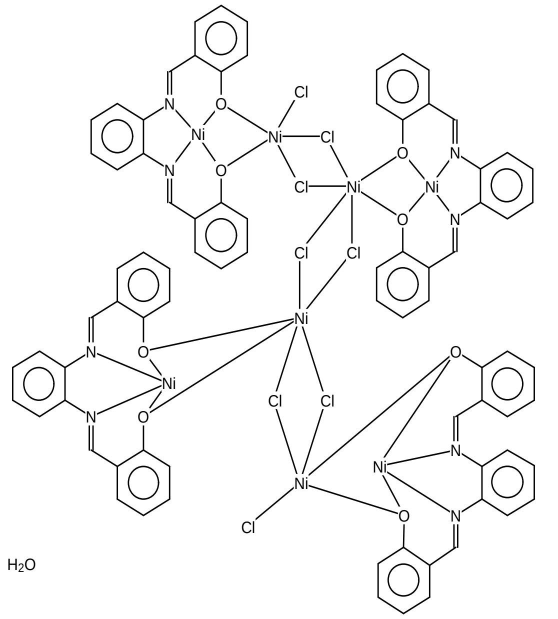

YOHZIF

Reference: S.J.Wezenberg, E.C.Escudero-Adan, J.Benet-Buchholz, A.W.Kleij (2008) *Org.Lett.* ,**10,**3311

Formula: $C_{45}\,H_{53}\,N_3\,O_2\,Zn_1$

Compound Name: (N,N'-bis(3,5-Di-t-butylsalicylidenaldiminato)benzene-1,2-diamine-N,N',O, O')-(quinoline-N)-zinc(ii)

Space Group:	P21/c	***Cell:***	***a*** 13.472(1)	***b*** 16.872(1)	***c*** 17.449(1)
Space Group No.:	14	***(Å,°)***	α 90.00	β 94.36(0)	γ 90.00

R-Factor (%): 3.86 ***Temperature(K):*** 100 ***Density(g/cm^3):*** 1.232

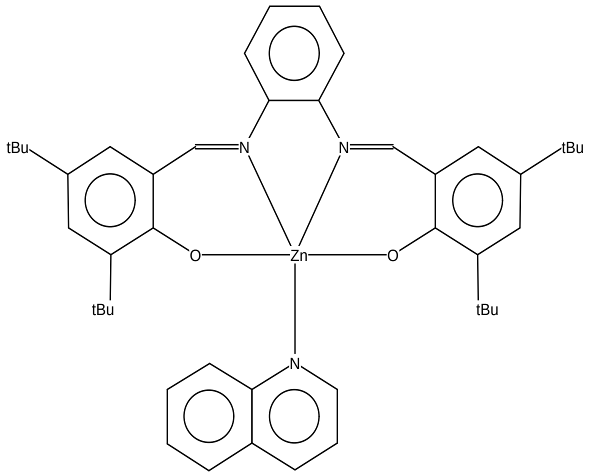

YOHZOL

Reference: S.J.Wezenberg, E.C.Escudero-Adan, J.Benet-Buchholz, A.W.Kleij (2008) *Org.Lett.* ,**10,**3311

Formula: 2($C_{36}\,H_{48}\,N_2\,O_3\,Zn_1$),$C_{13}\,H_9\,N_1$

Compound Name: bis(Aqua-(N,N'-bis(3,5-di-t-butylsalicylidenaldiminato)benzene-1,2-diamine-N,N',O,O')-zinc(ii)) acridine

Space Group:	P21/n	***Cell:***	***a*** 11.607(7)	***b*** 25.129(14)	***c*** 26.947(13)
Space Group No.:	14	***(Å,°)***	α 90.00	β 101.34(3)	γ 90.00

R-Factor (%): 5.06 ***Temperature(K):*** 100 ***Density(g/cm^3):*** 1.227

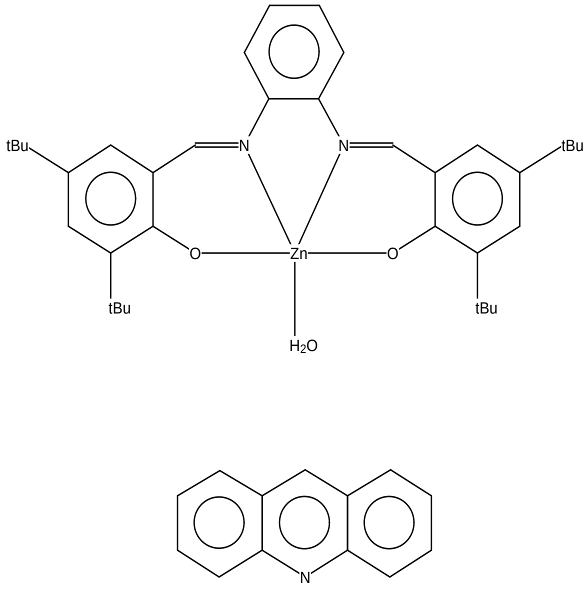

Search: search26 (Tue Dec 2 15:48:50 2014): Hits 697-700

YOHZUR

Reference: S.J.Wezenberg, E.C.Escudero-Adan, J.Benet-Buchholz, A.W.Kleij (2008) *Org.Lett.* ,**10,**3311

Formula: $C_{49}\ H_{55}\ N_3\ O_2\ Zn_1$

Compound Name: (N,N'-bis(3,5-Di-t-butylsalicylidenaldiminato)benzene-1,2-diamine-N,N',O,O')-(phenanthridine-N)-zinc(ii)

Space Group:	C2/m	***Cell:***	***a*** 23.038(1)	***b*** 10.062(0)	***c*** 19.419(1)
Space Group No.:	12	***(Å,°)***	α 90.00	β 110.73(0)	γ 90.00
R-Factor (%):	7.19	***Temperature(K):*** 100	***Density(g/cm^3):*** 1.236		

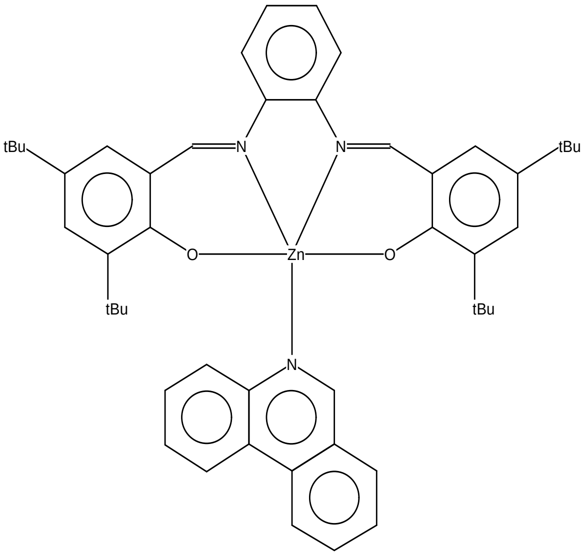

YOLJOY

Reference: Hong Wang, Shu-Lan Li, De-Xin Liu, Xue-Gui Cui, Xiao-Yan Li, Zhao-He Yang (1994) *Huaxue Xuebao(Chin.)(Acta Chim.Sinica)* ,**52,**676

Formula: $C_{22}\ H_{18}\ N_2\ Ni_1\ O_4,H_2\ O_1$

Compound Name: (1,2-Diaminobenzene-N,N'-bis(3-methoxysalicylideneiminato)-O,O',N,N')-nickel(ii) monohydrate

Space Group:	P21/n	***Cell:***	***a*** 8.854(2)	***b*** 19.067(5)	***c*** 11.807(4)
Space Group No.:	14	***(Å,°)***	α 90.00	β 99.43(2)	γ 90.00
R-Factor (%):	4.40	***Temperature(K):*** 295	***Density(g/cm^3):*** 1.524		

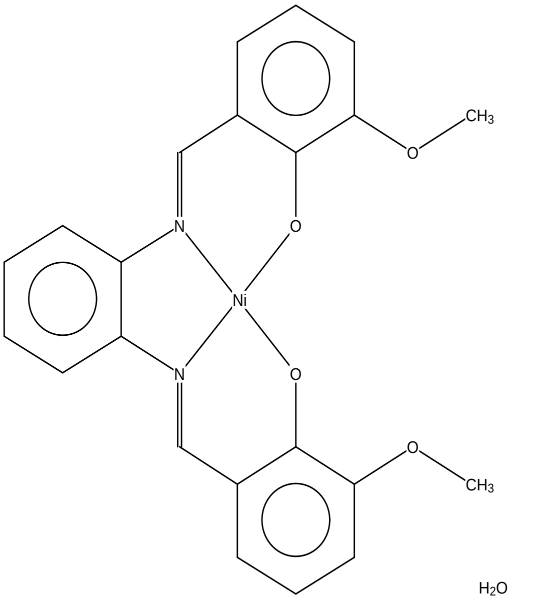

YUKHES

Reference: J.H.Chong, S.J.Ardakani, K.J.Smith, M.J.MacLachlan (2009) *Chem.-Eur.J.* ,**15,**11824

Formula: $C_{62}\ H_{38}\ N_6\ Ni_3\ O_6,5.5(C_2\ H_6\ O_1\ S_1),H_2\ O_1$

Compound Name: (μ_3-2,3,6,7,12,13-hexakis(Salicylideneamino)-9,10-dihydrotriptycene)-tri-nickel dimethylsulfoxide solvate monohydrate

Space Group:	P-1	***Cell:***	***a*** 13.236(1)	***b*** 18.408(3)	***c*** 20.908(4)
Space Group No.:	2	***(Å,°)***	α 82.03(0)	β 80.47(0)	γ 74.51(0)
R-Factor (%):	5.58	***Temperature(K):*** 173	***Density(g/cm^3):*** 1.094		

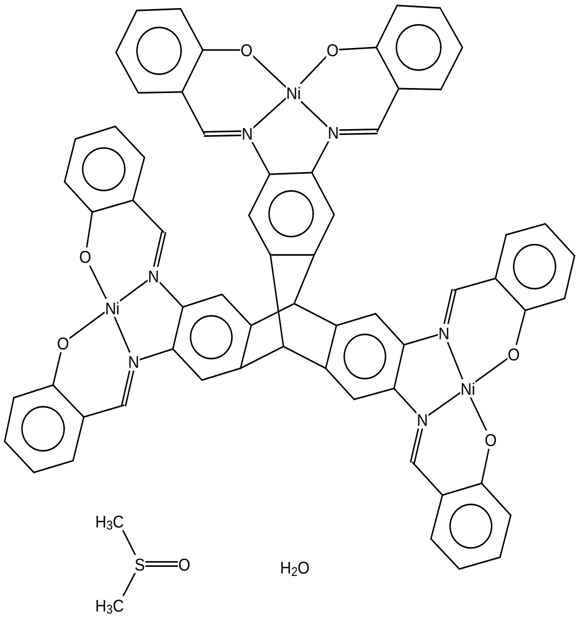

YULXOT

Reference: A.M.Castilla, S.Curreli, E.C.Escudero-Adan, M.M.Belmonte, J.Benet-Buchholz, A.W.Kleij (2009) *Org.Lett.* ,**11,**5218

Formula: $C_{51}\ H_{60}\ N_6\ O_8\ S_1\ Zn_1,3(C_2\ H_6\ O_1\ S_1)$

Compound Name: (2-(((3'-Amino-4-((3,5-di-t-butyl-2-hydroxybenzylidene)amino)-4'-((3,5-di-t-butyl-2-oxybenzylidene)amino)biphenyl-3-yl)imino)methyl)-4,6-dinitrophenolato)-(dimethylsulfoxide)-zinc dimethylsulfoxide solvate

Space Group:	P-1	***Cell:***	***a*** 6.939(2)	***b*** 18.930(6)	***c*** 24.814(8)
Space Group No.:	2	***(Å,°)***	α 98.58(0)	β 92.11(0)	γ 94.99(0)
R-Factor (%):	9.43	***Temperature(K):*** 100	***Density(g/cm^3):*** 1.260		

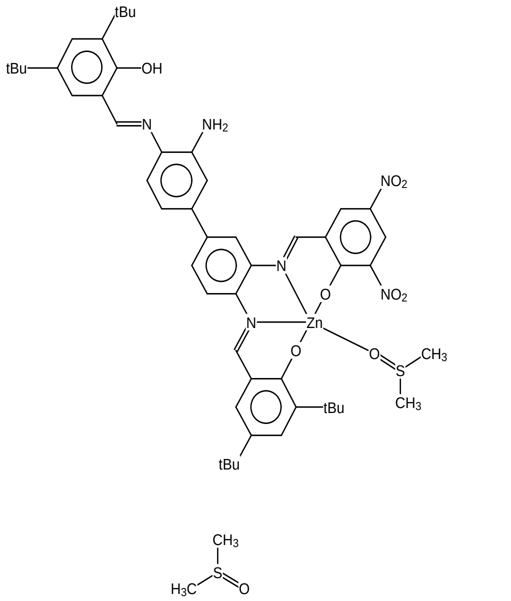

Search: search26 (Tue Dec 2 15:48:50 2014): Hits 701-704

YULXUZ

Reference: A.M.Castilla, S.Curreli, E.C.Escudero-Adan, M.M.Belmonte, J.Benet-Buchholz, A.W.Kleij (2009) *Org.Lett.* ,**11,**5218

Formula: C_{56} H_{54} Cl_2 N_6 Ni_1 O_8 Pd_1,4(C_2 H_6 O_1 S_1)

Compound Name: (μ_2-2,4-Di-t-butyl-6-(((4'-((3,5-di-t-butyl-2-oxybenzylidene)amino)-3'-((3,5-dichloro-2-oxybenzylidene)amino)-3-((2-oxy-3,5-dinitrobenzylidene)amino)biphenyl-4-yl)imino)methyl)phenolato)-palladium-nickel dimethylsulfoxide solvate

Space Group:	P-1	***Cell:***	***a*** 11.313(0)	***b*** 15.562(0)	***c*** 20.983(0)
Space Group No.:	2	***(Å,°)***	α 77.20(0)	β 88.03(0)	γ 69.34(0)

R-Factor (%): 5.93 ***Temperature(K):*** 100 ***Density(g/cm³):*** 1.467

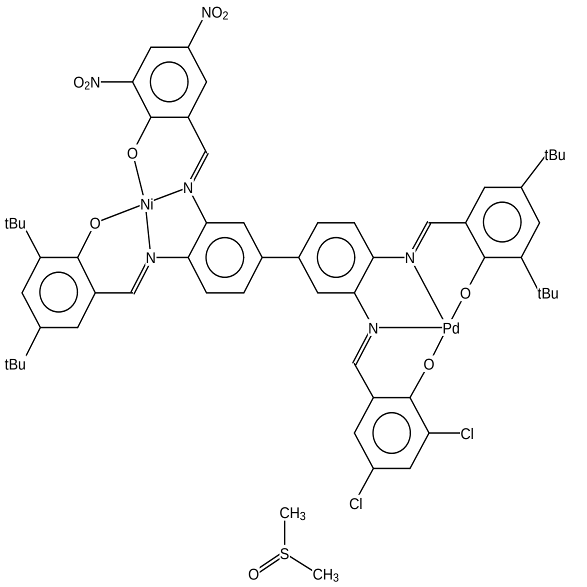

YUNPAY

Reference: Xin Wang, Xiao Min Zhang, Hai Xin Liu (1994) *Transition Met.Chem.* ,**19,**611

Formula: C_{20} H_{14} N_2 O_3 V_1,C_2 H_3 N_1

Compound Name: (Oxo-N,N'-disalicylidene-o-phenylenediamine-N,N',O,O')-vanadium(iv) acetonitrile solvate

Space Group:	P21/n	***Cell:***	***a*** 9.398	***b*** 19.161	***c*** 11.256
Space Group No.:	14	***(Å,°)***	α 90.00	β 109.92	γ 90.00

R-Factor (%): 5.20 ***Temperature(K):*** 295 ***Density(g/cm³):*** 1.472

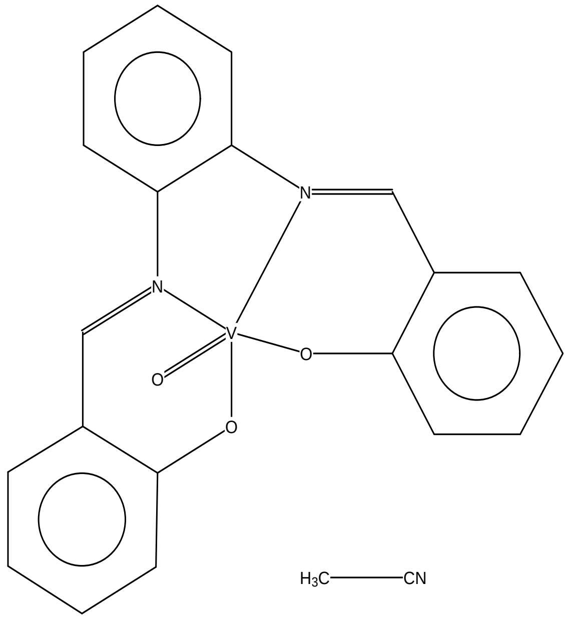

YUNPAY10

Reference: Xin Wang, Xiao Min Zhang, Hai Xin Liu (1995) *Polyhedron* ,**14,**293

Formula: C_{20} H_{14} N_2 O_3 V_1,C_2 H_3 N_1

Compound Name: (Oxo-N,N'-disalicylidene-o-phenylenediamine-N,N',O,O')-vanadium(iv) acetonitrile solvate

Space Group:	P21/n	***Cell:***	***a*** 9.398	***b*** 19.161	***c*** 11.256
Space Group No.:	14	***(Å,°)***	α 90.00	β 109.92	γ 90.00

R-Factor (%): 5.20 ***Temperature(K):*** 295 ***Density(g/cm³):*** 1.472

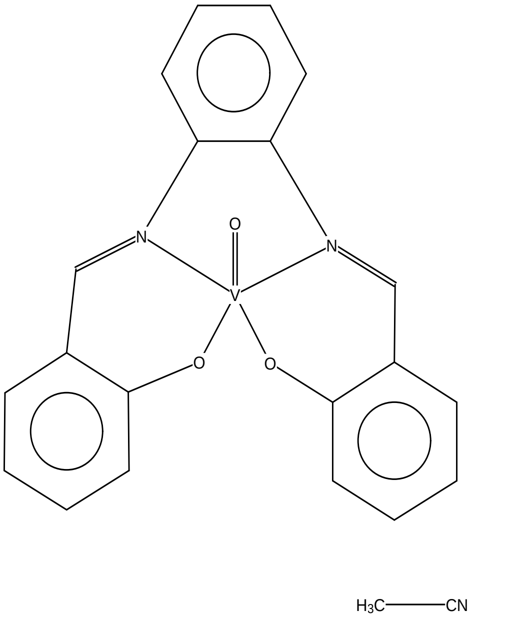

YUTCAS

Reference: Xing-Qiang Lu, Wei-Xu Feng, Ya-Ni Hui, Tao Wei, Ji-Rong Song, Shun-Sheng Zhao, Wai-Yeung Wong, Wai-Kwok Wong, R.A.Jones (2010) *Eur.J.Inorg.Chem.* ,2714

Formula: C_{56} H_{58} Br_4 N_{12} Nd_2 O_{26} Zn_2

Compound Name: bis(μ_3-2,2'-((3-Oxidobenzene-1,2-diyl)bis(nitrilomethylidene))bis(4-bromo-6-methoxyphenolato))-tetrakis(dimethylformamide)-tetrakis(nitrato-O,O')-di-neodymium-di-zinc

Space Group:	P21/c	***Cell:***	***a*** 12.034(2)	***b*** 17.983(3)	***c*** 16.910(3)
Space Group No.:	14	***(Å,°)***	α 90.00	β 108.52(0)	γ 90.00

R-Factor (%): 4.45 ***Temperature(K):*** 273 ***Density(g/cm³):*** 1.966

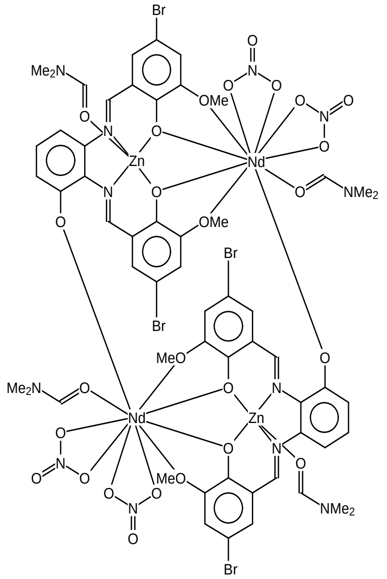

Search: search26 (Tue Dec 2 15:48:50 2014): Hits 705-708

YUTCEW

Reference: Xing-Qiang Lu, Wei-Xu Feng, Ya-Ni Hui, Tao Wei, Ji-Rong Song, Shun-Sheng Zhao, Wai-Yeung Wong, Wai-Kwok Wong, R.A.Jones (2010) *Eur.J.Inorg.Chem.* ,2714

Formula: $C_{60} H_{58} N_{12} Nd_2 O_{24} Zn_2,3(C_4 H_{10} O_1)$

Compound Name: bis(μ_3-2,2'-((3-Oxidobenzene-1,2-diyl)bis(nitrilomethylidene))bis(6-methoxyphenolato))-bis(dimethylformamide)-tetrakis(nitrato-O,O')-bis(pyridine)-di-neodymium-di-zinc diethyl ether solvate

Space Group:	P-1	***Cell:***	***a*** 12.947(0)	***b*** 13.862(0)	***c*** 13.925(0)
Space Group No.:	2	***(Å,°)***	α 101.01(0)	β 109.95(0)	γ 111.97(0)

R-Factor (%): 5.79 ***Temperature(K):*** 293 ***Density(g/cm³):*** 1.615

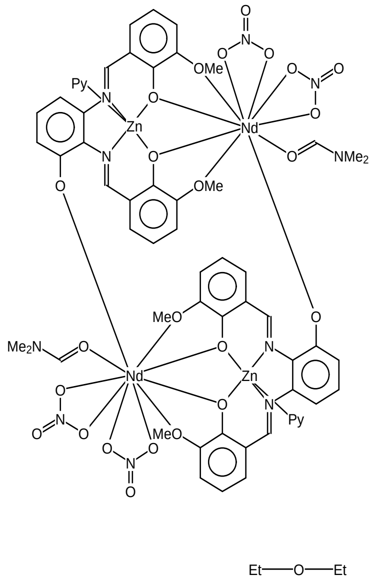

ZZZBSS

Reference: R.J.Hill, C.E.F.Rickard (1978) *J.Inorg.Nucl.Chem.* ,**40**,793

Formula: $C_{40} H_{28} N_4 O_4 Th_1$

Compound Name: bis(N,N'-o-Phenylenebis(salicylaldiminato))-thorium(iv)

Space Group:		***Cell:***	***a*** 15.587	***b*** 12.726	***c*** 16.204
Space Group No.:	0	***(Å,°)***	α 90.00	β 91.72	γ 90.00

R-Factor (%): 0.00 ***Temperature(K):*** 295 ***Density(g/cm³):*** 0.000

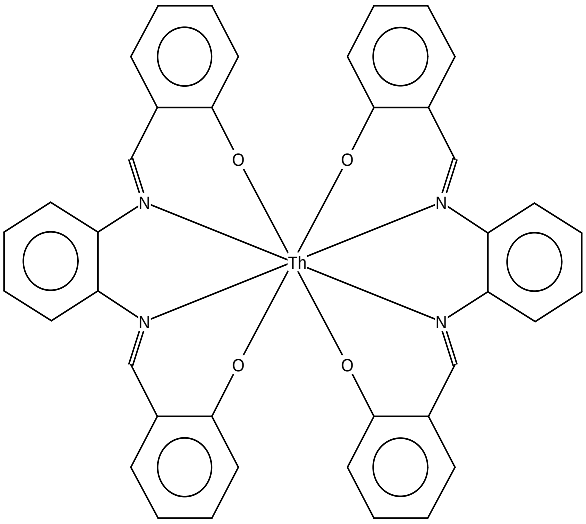

ZZZBSV

Reference: R.J.Hill, C.E.F.Rickard (1978) *J.Inorg.Nucl.Chem.* ,**40**,793

Formula: $C_{40} H_{28} N_4 O_4 U_1$

Compound Name: bis(N,N'-o-Phenylenebis(salicylaldiminato)) uranium(iv)

Space Group:		***Cell:***	***a*** 16.840	***b*** 22.890	***c*** 16.900
Space Group No.:	0	***(Å,°)***	α 90.00	β 93.50	γ 90.00

R-Factor (%): 0.00 ***Temperature(K):*** 295 ***Density(g/cm³):*** 0.000

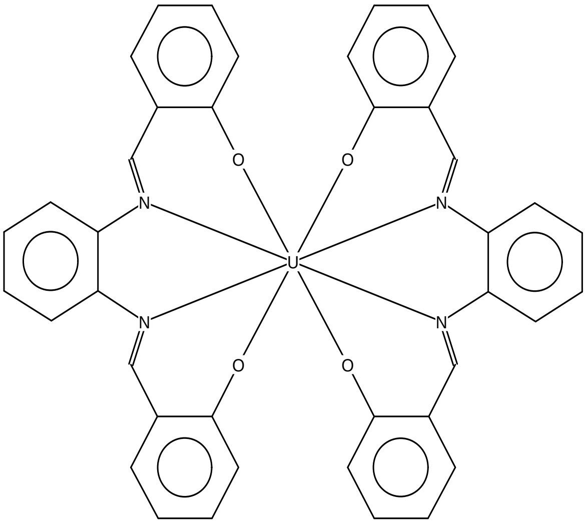

ZZZTWE

Reference: Montgomery, Morosin (1961) *Acta Crystallogr.* ,**14**,551

Formula: $C_{20} H_{14} Cu_1 N_2 O_2$

Compound Name: (N,N'-disalicylidene-o-phenylenediamine)-copper(ii)

Space Group:	P212121	***Cell:***	***a*** 16.870	***b*** 17.790	***c*** 5.341
Space Group No.:	19	***(Å,°)***	α 90.00	β 90.00	γ 90.00

R-Factor (%): 0.00 ***Temperature(K):*** 295 ***Density(g/cm³):*** 1.566

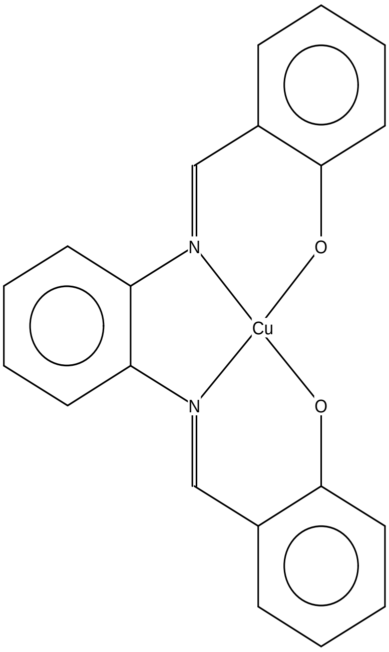

ZZZTWE01

Reference: Yu-Min Yang (2005) *Acta Crystallogr.,Sect.E:Struct.Rep.Online* ,**61,**m1425

Formula: $C_{20} H_{14} Cu_1 N_2 O_2$

Compound Name: (o-Phenylenebis(salicylideneaminato))-copper(ii)

Space Group: P212121 ***Cell:*** ***a*** 5.470(1) ***b*** 16.618(3) ***c*** 17.322(3)
Space Group No.: 19 ***(Å,°)*** α 90.00 β 90.00 γ 90.00

R-Factor (%): 7.56 ***Temperature(K):*** 298 ***Density(g/cm^3):*** 1.594

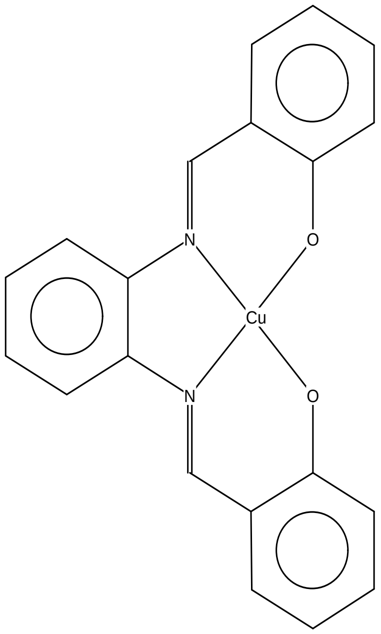

ZZZTWE02

Reference: E.Suresh, M.M.Bhadbhade, D.Srinivas (1996) *Polyhedron* , **15,**4133

Formula: $C_{20} H_{14} Cu_1 N_2 O_2$

Compound Name: (o-Phenylenebis(salicylidenaminato))-copper(ii)

Space Group: P212121 ***Cell:*** ***a*** 9.140(6) ***b*** 18.984(8) ***c*** 18.004(4)
Space Group No.: 19 ***(Å,°)*** α 90.00 β 90.00 γ 90.00

R-Factor (%): 5.50 ***Temperature(K):*** 295 ***Density(g/cm^3):*** 1.607

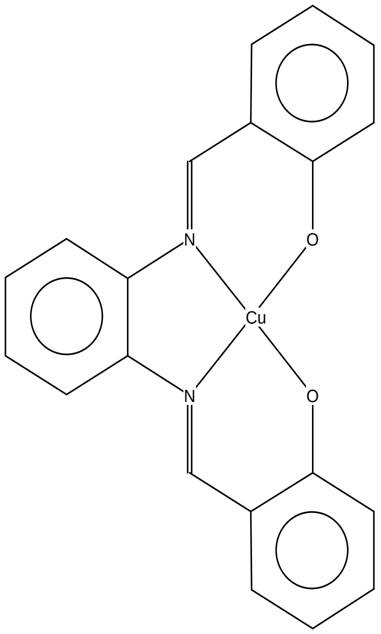

ZZZTZI

Reference: Montgomery, Morosin (1961) *Acta Crystallogr.* ,**14,**551

Formula: $C_{20} H_{14} N_2 Ni_1 O_2$

Compound Name: (N,N'-disalicylidene-o-phenylenediamine)-nickel(ii)

Space Group: P212121 ***Cell:*** ***a*** 16.726 ***b*** 17.411 ***c*** 5.455
Space Group No.: 19 ***(Å,°)*** α 90.00 β 90.00 γ 90.00

R-Factor (%): 0.00 ***Temperature(K):*** 295 ***Density(g/cm^3):*** 1.560

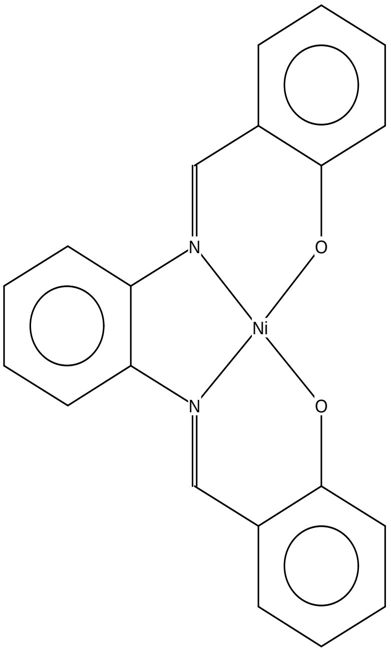

ZZZTZI01

Reference: A.Radha, M.Seshasayee, K.Ramalingam, G.Aravamudan (1985) *Acta Crystallogr.,Sect.C:Cryst.Struct.Commun.* ,**41,**1169

Formula: $C_{20} H_{14} N_2 Ni_1 O_2$

Compound Name: (N,N'-o-Phenylenedisalicylideneaminato-N,N',O,O')-nickel(ii)

Space Group: P212121 ***Cell:*** ***a*** 5.458(1) ***b*** 16.585(4) ***c*** 17.287(5)
Space Group No.: 19 ***(Å,°)*** α 90.00 β 90.00 γ 90.00

R-Factor (%): 5.20 ***Temperature(K):*** 295 ***Density(g/cm^3):*** 1.583

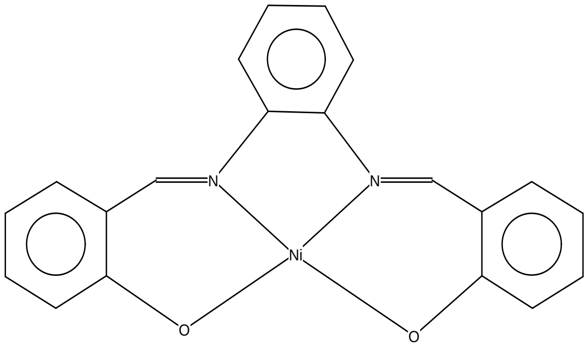

Search: search26 (Tue Dec 2 15:48:50 2014): Hits 713-716

ZZZTZI02

Reference: Jun Wang, Feng-Li Bei, Xing-You Xu, Xu-Jie Yang, Xin Wang (2003) *J.Chem.Cryst.* ,**33,**845

Formula: $C_{20}\ H_{14}\ N_2\ Ni_1\ O_2$

Compound Name: (N,N'-o-Phenylenedisalicylideneaminato-N,N',O,O')-nickel(ii)

Space Group: P212121 **Cell:** *a* 5.450(1) *b* 16.620(3) *c* 17.300(4)
Space Group No.: 19 **(Å,°)** α 90.00 β 90.00 γ 90.00

R-Factor (%): 4.68 **Temperature(K):** 293 **Density(g/cm^3):** 1.581

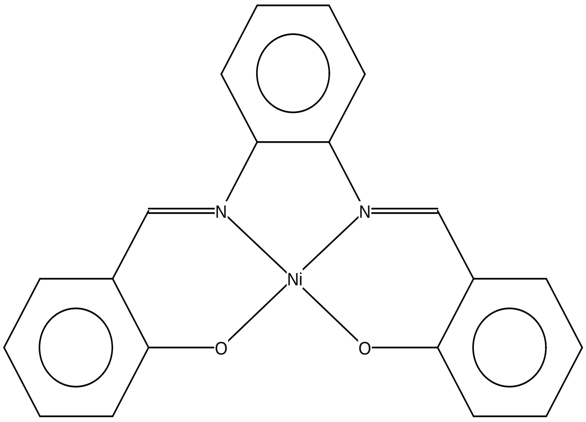

ZZZTZI03

Reference: Jun Wang, Feng-Li Bei, Wei-Xing Ma, Xu-Jie Yang, Lu-De Lu, Xin Wang (2003) *Wuji Huaxue Xuebao(Chin.)(Chin.J.Inorg.Chem.)* ,**19,**609

Formula: $C_{20}\ H_{14}\ N_2\ Ni_1\ O_2$

Compound Name: (N,N'-o-Phenylenedisalicylideneaminato-N,N',O,O')-nickel(ii)

Space Group: P212121 **Cell:** *a* 16.620(3) *b* 17.300(4) *c* 5.450(1)
Space Group No.: 19 **(Å,°)** α 90.00 β 90.00 γ 90.00

R-Factor (%): 4.68 **Temperature(K):** 293 **Density(g/cm^3):** 1.581

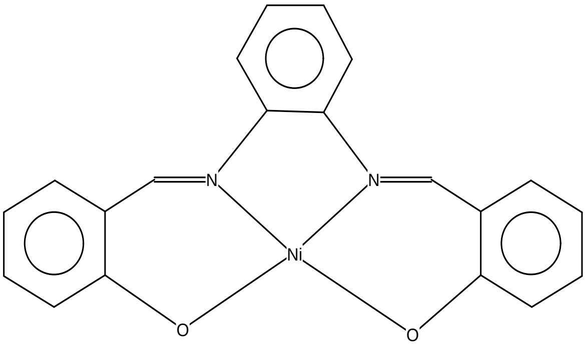

BICBEW

Reference: A.Biswas, L.Mandal, S.Mondal, C.Robert Lucas, S.Mohanta (2013) *CrystEngComm* ,**15,**5888

Formula: $C_{24}\ H_{28}\ Cu_1\ Fe_1\ N_2\ O_7\ ^{2+}$,2($C_{22}\ H_{18}\ Cu_1\ N_2\ O_4$),2($Cl_1\ O_4\ ^{1-}$),$C_1\ H_4\ O_1$

Compound Name: (μ_2-2,2'-(1,2-Phenylenebis(nitrilomethylylidene))bis(6-methoxyphenolato))-aqua-bis(methanol)-copper(ii)-iron(ii) bis((2,2'-(1,2-phenylenebis(nitrilomethylylidene))bis(6-methoxyphenolato))-copper(ii)) diperchlorate methanol solvate

Space Group: P21/c **Cell:** *a* 11.557(2) *b* 26.632(5) *c* 22.822(5)
Space Group No.: 14 **(Å,°)** α 90.00 β 95.72(0) γ 90.00

R-Factor (%): 3.76 **Temperature(K):** 296 **Density(g/cm^3):** 1.599

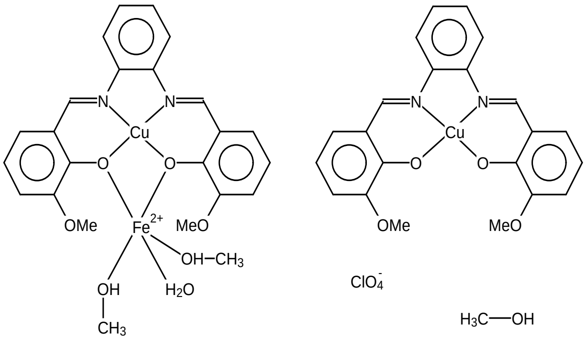

BICBIA

Reference: A.Biswas, L.Mandal, S.Mondal, C.Robert Lucas, S.Mohanta (2013) *CrystEngComm* ,**15,**5888

Formula: $C_{24}\ H_{28}\ Cu_1\ Mn_1\ N_2\ O_7\ ^{2+}$,2($C_{22}\ H_{18}\ Cu_1\ N_2\ O_4$),2($Cl_1\ O_4\ ^{1-}$),$C_1\ H_4\ O_1$

Compound Name: (μ_2-2,2'-(1,2-Phenylenebis(nitrilomethylylidene))bis(6-methoxyphenolato))-aqua-bis(methanol)-copper(ii)-manganese(ii) bis((2,2'-(1,2-phenylenebis(nitrilomethylylidene))bis(6-methoxyphenolato))-copper(ii)) diperchlorate methanol solvate

Space Group: P21/c **Cell:** *a* 11.565(1) *b* 26.500(3) *c* 22.771(3)
Space Group No.: 14 **(Å,°)** α 90.00 β 95.59(0) γ 90.00

R-Factor (%): 3.87 **Temperature(K):** 296 **Density(g/cm^3):** 1.608

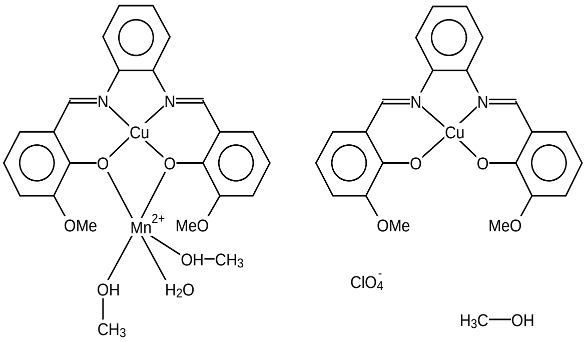

Search: search26 (Tue Dec 2 15:48:50 2014): Hits 717-720

BIDWAO

Reference: Yu-Ying Lai, M.Bornand, P.Chen (2012) *Organometallics* , **31**,7558

Formula: $C_{72}\ H_{92}\ Cl_2\ N_4\ Nb_2\ O_6, C_6\ H_6, 6(C_1\ H_2\ Cl_2)$

Compound Name: bis(μ_2-oxo)-dichloro-bis(2,2'-(1,2-phenylenebis(nitrilomethylylidene)) bis(4,6-di-t-butylphenolato))-di-niobium(v) benzene dichloromethane solvate

Space Group:	P21/c	***Cell:***	***a*** 12.471(0)	***b*** 24.025(1)	***c*** 17.095(0)
Space Group No.:	14	***(Å,°)***	α 90.00	β 107.78(0)	γ 90.00

R-Factor (%): 8.31 ***Temperature(K):*** 223 ***Density(g/cm³):*** 1.331

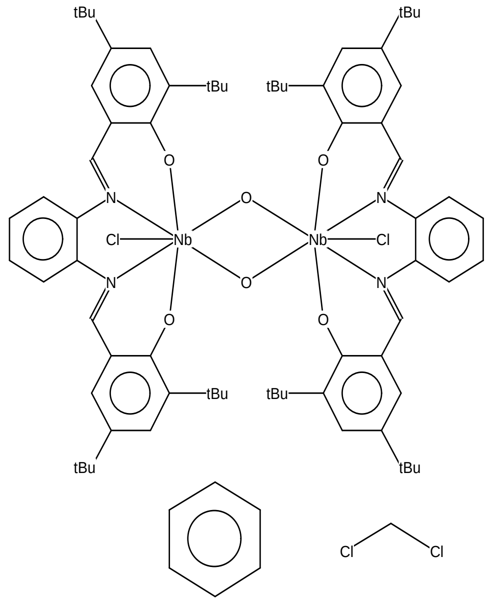

BIDWES

Reference: Yu-Ying Lai, M.Bornand, P.Chen (2012) *Organometallics* , **31**,7558

Formula: $C_{72}\ H_{92}\ N_4\ Nb_2\ O_6\ ^{2+}, 4(C_6\ H_6), 2(C_{32}\ H_{12}\ B_1\ F_{24}\ ^{1-})$

Compound Name: bis(μ_2-oxo)-bis(2,2'-(1,2-phenylenebis(nitrilomethylylidene))bis(4,6-di-t-butylphenolato))-di-niobium(v) bis(tetrakis(3,5-bis(trifluoromethyl)phenyl) borate) benzene solvate

Space Group:	P-1	***Cell:***	***a*** 13.223(0)	***b*** 17.527(0)	***c*** 17.937(0)
Space Group No.:	2	***(Å,°)***	α 81.66(0)	β 84.70(0)	γ 71.37(0)

R-Factor (%): 6.39 ***Temperature(K):*** 223 ***Density(g/cm³):*** 1.422

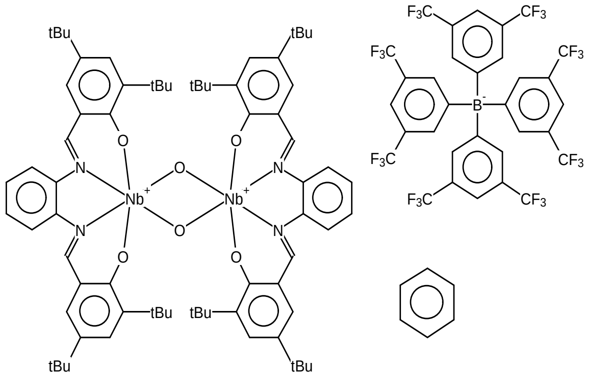

BIDWIW

Reference: Yu-Ying Lai, M.Bornand, P.Chen (2012) *Organometallics* , **31**,7558

Formula: $C_{72}\ H_{92}\ N_4\ Nb_2\ O_{14}\ Re_2, 4(C_2\ H_3\ N_1)$

Compound Name: bis(μ_2-oxo)-bis(perrhenato)-bis(2,2'-(1,2-phenylenebis(nitrilomethylylidene))bis(4,6-di-t-butylphenolato))-di-niobium(v) acetonitrile solvate

Space Group:	P21/c	***Cell:***	***a*** 14.267(0)	***b*** 29.566(0)	***c*** 20.311(0)
Space Group No.:	14	***(Å,°)***	α 90.00	β 91.76(0)	γ 90.00

R-Factor (%): 7.89 ***Temperature(K):*** 220 ***Density(g/cm³):*** 1.520

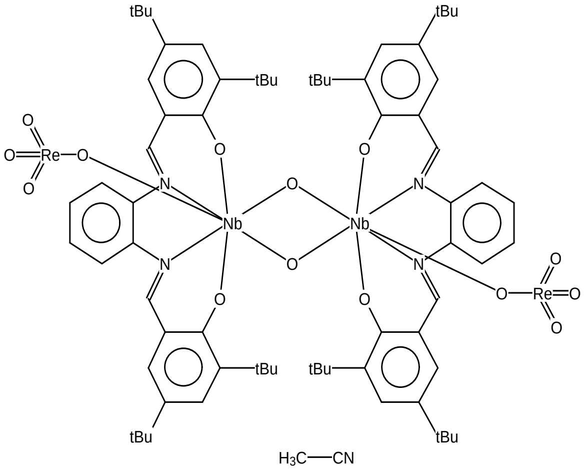

CIFKAF

Reference: Wan-Seok Kim, Kyung Yeon Lee, Eui-Hyun Ryu, Ja-Min Gu, Youngmee Kim, Suk Joong Lee, Seong Huh (2013) *Eur.J.Inorg.Chem.* ,4228

Formula: $(C_{70}\ H_{66}\ N_8\ O_4\ Zn_2)n, n(C_4\ H_{11}\ N_1)$

Compound Name: catena-((μ_4-2,2',2'',2'''-(Benzene-1,2,4,5-tetrayltetrakis(nitrilomethylylidene))tetrakis(6-t-butyl-4-(pyridin-4-yl) phenolato))-di-zinc diethylamine solvate)

Space Group:	P21/c	***Cell:***	***a*** 10.419(1)	***b*** 18.863(2)	***c*** 22.224(3)
Space Group No.:	14	***(Å,°)***	α 90.00	β 91.45(0)	γ 90.00

R-Factor (%): 12.47 ***Temperature(K):*** 273 ***Density(g/cm³):*** 0.979

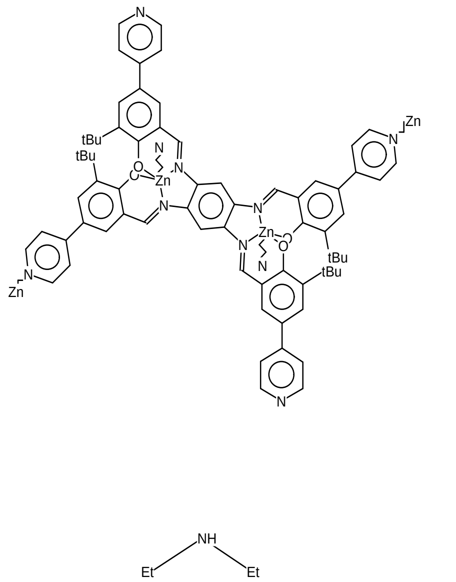

Search: search26 (Tue Dec 2 15:48:50 2014): Hits 721-724

DIHDUV

Reference: T.Silha, I.Nemec, R.Herchel, Z.Travnicek (2013) *CrystEngComm* ,**15,**5351

Formula: C_{56} H_{52} Mn_2 N_{10} O_{10} Pt_1 S_6

Compound Name: bis(μ_2-Thiocyanato)-diaqua-tetrakis(thiocyanato)-bis(2,2'-((3-methyl-1,2-phenylene)bis(nitrilomethylylidene))bis(6-ethoxyphenolato))-di-manganese-platinum

Space Group:	P21/c	***Cell:***	***a*** 14.055(0)	***b*** 14.444(0)	***c*** 17.531(0)
Space Group No.:	14	***(Å,°)***	α 90.00	β 122.72(0)	γ 90.00

R-Factor (%): 3.14 ***Temperature(K):*** 150 ***Density(g/cm^3):*** 1.689

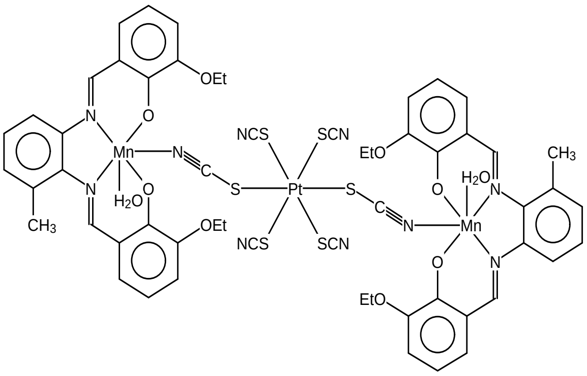

FIGKAJ

Reference: S.Bhattacharya, A.Jana, M.Fleck, S.Mohanta (2013) *Inorg.Chim.Acta* ,**405,**196

Formula: C_{22} H_{18} Cu_1 N_2 O_4,0.5(H_4 N_2 O_{10} U_1),H_2 O_1

Compound Name: (2,2'-(1,2-Phenylenebis((nitrilo)methylylidene))bis(6-methoxyphenolato))-copper diaqua-bis(nitrato)-dioxo-uranium monohydrate

Space Group:	P-1	***Cell:***	***a*** 9.148(1)	***b*** 11.578(1)	***c*** 13.165(2)
Space Group No.:	2	***(Å,°)***	α 100.32(1)	β 104.68(1)	γ 109.19(1)

R-Factor (%): 5.02 ***Temperature(K):*** 293 ***Density(g/cm^3):*** 1.826

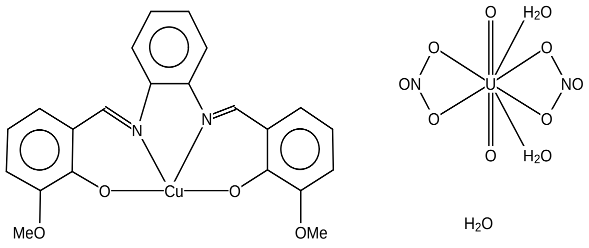

GICBUR

Reference: Xiaoping Yang, D.Schipper, A.Liao, J.M.Stanley, R.A.Jones, B.J.Holliday (2013) *Polyhedron* ,**52,**165

Formula: C_{26} H_{24} N_3 O_{11} Tb_1 Zn_1

Compound Name: (μ_2-Acetato)-(acetato)-(nitrato)-(μ_2-2,2'-(1,2-phenylenebis((nitrilo) methylylidene))bis(6-methoxyphenolato))-terbium-zinc

Space Group:	P21/c	***Cell:***	***a*** 14.045(3)	***b*** 13.408(3)	***c*** 15.376(4)
Space Group No.:	14	***(Å,°)***	α 90.00	β 102.68(3)	γ 90.00

R-Factor (%): 3.17 ***Temperature(K):*** 223 ***Density(g/cm^3):*** 1.831

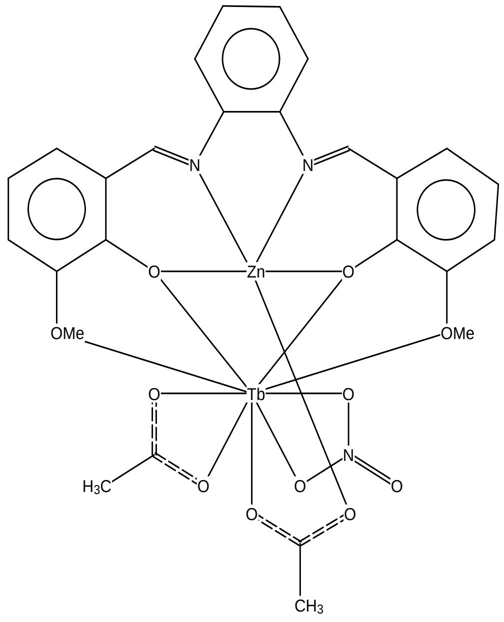

GICCAY

Reference: Xiaoping Yang, D.Schipper, A.Liao, J.M.Stanley, R.A.Jones, B.J.Holliday (2013) *Polyhedron* ,**52,**165

Formula: C_{25} H_{25} Eu_1 N_4 O_{13} Zn_1

Compound Name: (μ_2-Acetato)-bis(nitrato)-(methanol)-(μ_2-2,2'-(1,2-phenylenebis((nitrilo) methylylidene))bis(6-methoxyphenolato))-europium-zinc

Space Group:	P-1	***Cell:***	***a*** 9.742(1)	***b*** 12.729(3)	***c*** 14.172(3)
Space Group No.:	2	***(Å,°)***	α 84.04(3)	β 73.52(3)	γ 67.91(4)

R-Factor (%): 9.66 ***Temperature(K):*** 223 ***Density(g/cm^3):*** 1.716

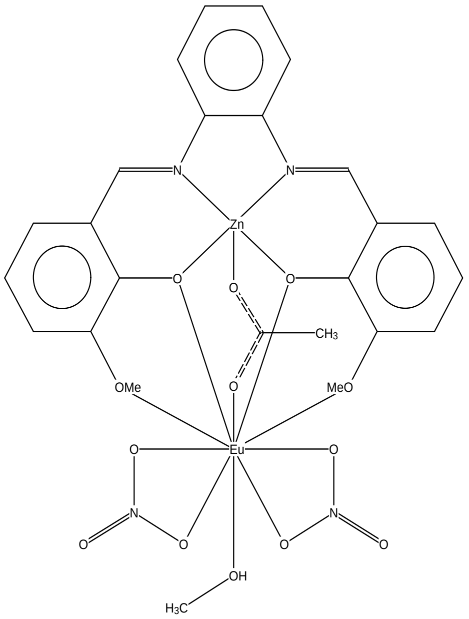

Search: search26 (Tue Dec 2 15:48:50 2014): Hits 725-728

GICCEC

Reference: Xiaoping Yang, D.Schipper, A.Liao, J.M.Stanley, R.A.Jones, B.J.Holliday (2013) *Polyhedron* ,**52,**165

Formula: $C_{48} H_{42} N_4 O_{12} Tb_1 Zn_2{}^{1+}, C_4 H_{10} O_1, C_1 F_3 O_3 S_1{}^{1-}$

Compound Name: bis(μ_2-Acetato)-bis(μ_2-2,2'-(1,2-phenylenebis((nitrilo)methylylidene)) bis(6-methoxyphenolato))-terbium-di-zinc trifluoromethanesulfonate diethyl ether solvate

Space Group:	P-1	***Cell:***	***a*** 9.444(1)	***b*** 16.108(3)	***c*** 19.170(4)		
Space Group No.:	2	***(Å,°)***	α 88.71(3)	β 78.14(4)	γ 76.87(3)		
R-Factor (%):	9.25	***Temperature(K):***	223	***Density(g/cm³):***	1.649		

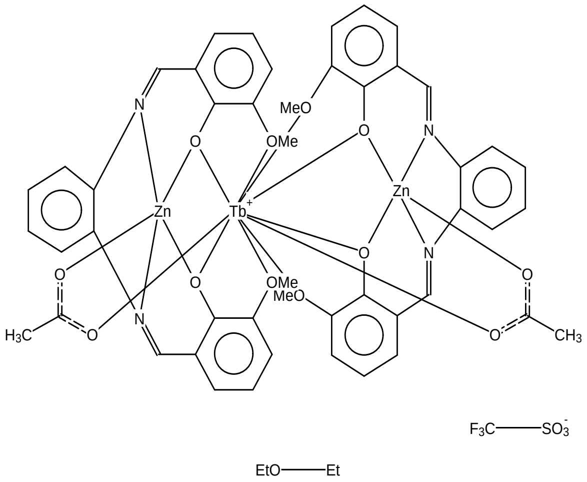

GICCIG

Reference: Xiaoping Yang, D.Schipper, A.Liao, J.M.Stanley, R.A.Jones, B.J.Holliday (2013) *Polyhedron* ,**52,**165

Formula: $C_{48} H_{42} Eu_1 N_4 O_{12} Zn_2{}^{1+}, C_4 H_{10} O_1, C_1 F_3 O_3 S_1{}^{1-}$

Compound Name: bis(μ_2-Acetato)-bis(μ_2-2,2'-(1,2-phenylenebis((nitrilo)methylylidene)) bis(6-methoxyphenolato))-europium-di-zinc trifluoromethanesulfonate diethyl ether solvate

Space Group:	P-1	***Cell:***	***a*** 9.460(5)	***b*** 16.153(5)	***c*** 19.244(4)		
Space Group No.:	2	***(Å,°)***	α 88.16(0)	β 77.84(0)	γ 77.04(0)		
R-Factor (%):	9.80	***Temperature(K):***	223	***Density(g/cm³):***	1.628		

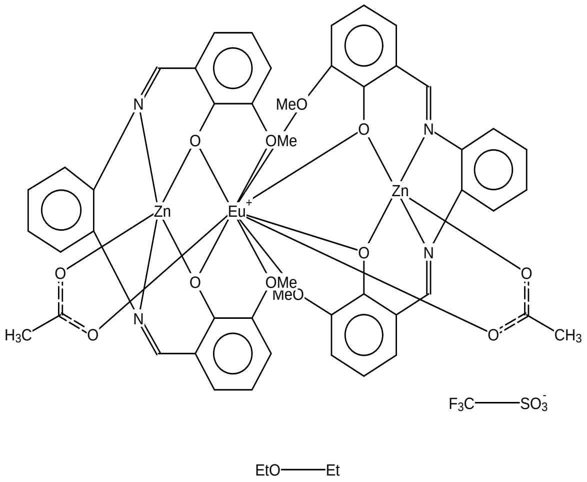

NIKSAD

Reference: S.Dharmalingam, S.Yoon (2013) *Acta Crystallogr.,Sect.E:Struct.Rep.Online* ,**69,**m495

Formula: $C_{30} H_{26} Co_1 N_6 O_2{}^{1+}, N_1 O_3{}^{1-}$

Compound Name: bis(4-aminopyridine)(2,2'-[1,2-pihenylenebis(nitrilomethanylylidene)]diphenolato)cobalt(iii) nitrate

Synonym: (2,2'-(1,2-Phenylenebis((nitrilo)methylylidene))diphenolato)(bis(pyridin-4-amine))cobalt nitrate

Space Group:	P21/c	***Cell:***	***a*** 13.072(3)	***b*** 15.136(3)	***c*** 17.192(6)		
Space Group No.:	14	***(Å,°)***	α 90.00	β 125.14(2)	γ 90.00		
R-Factor (%):	4.96	***Temperature(K):***	293	***Density(g/cm³):***	1.489		

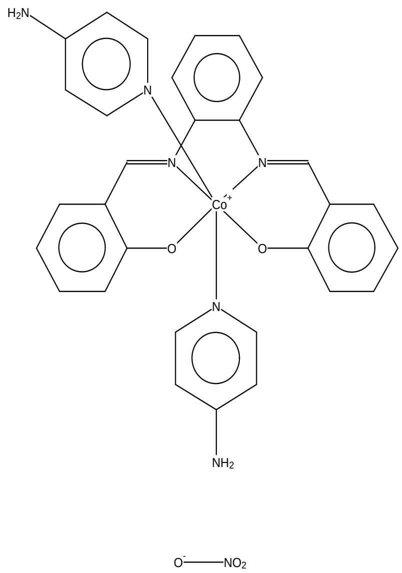

REWCON

Reference: D.Anselmo, G.Salassa, E.C.Escudero-Adan, E.Martin, A.W.Kleij (2013) *Dalton Trans.* ,**42,**7962

Formula: $C_{86} H_{86} N_6 Ni_3 O_6, 3.25(C_2 H_6 O_1 S_1)$

Compound Name: (μ_3-2,2',2'',2''',2'''',2'''''-(Pentacyclo[6.6.6.0^{2,7}.0^{9,14}.0^{15,20}]icosa-2,4,6,9, 11,13,15,17,19-nonaene-4,5,11,12,17,18-hexaylhexakis(nitrilomethylylidene))hexakis(6-t-butylphenolato))-tri-nickel(ii) dimethyl sulfoxide solvate

Space Group:	P-1	***Cell:***	***a*** 13.212(0)	***b*** 22.067(0)	***c*** 22.732(0)		
Space Group No.:	2	***(Å,°)***	α 100.08(0)	β 96.78(0)	γ 105.90(0)		
R-Factor (%):	5.84	***Temperature(K):***	100	***Density(g/cm³):***	0.930		

$Ni_3[\mu_3\text{-}C_{62}H_{32}N_6O_6(Bu^t)_6\text{-}\kappa^6N,N',N'',N''',N'''',N''''',\kappa^6O,O',O'',O''',O'''',O''''']$

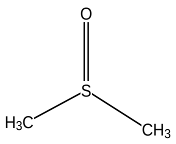

REWCUT

Reference: D.Anselmo, G.Salassa, E.C.Escudero-Adan, E.Martin, A.W.Kleij (2013) *Dalton Trans.* ,**42,**7962

Formula: C_{37} H_{27} N_3 O_2 Zn_1

Compound Name: (3,3'-(1,2-Phenylenebis((nitrilo)methylylidene))dibiphenyl-2-olato)-pyridine-zinc(ii)

Space Group: P21/n ***Cell:*** ***a*** 14.099(0) ***b*** 14.750(0) ***c*** 14.585(0)
Space Group No.: 14 ***(Å,°)*** α 90.00 β 112.86(0) γ 90.00

R-Factor (%): 5.02 ***Temperature(K):*** 100 ***Density(g/cm3):*** 1.452

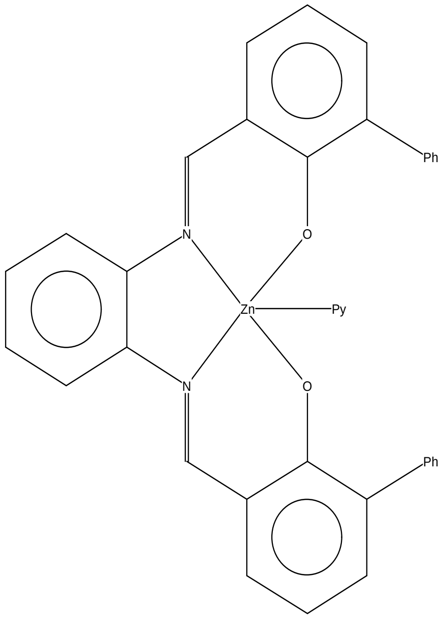

REWDAA

Reference: D.Anselmo, G.Salassa, E.C.Escudero-Adan, E.Martin, A.W.Kleij (2013) *Dalton Trans.* ,**42,**7962

Formula: C_{64} H_{44} N_4 O_4 Zn_2,1.8(C_2 H_3 N_1),0.2(C_1 H_1 Cl_3)

Compound Name: bis(μ$_2$-3,3'-(1,2-Phenylenebis(nitrilomethylylidene))dibiphenyl-2-olato)-di-zinc(ii) acetonitrile chloroform solvate

Space Group: C2/c ***Cell:*** ***a*** 24.932(1) ***b*** 11.900(0) ***c*** 21.784(2)
Space Group No.: 15 ***(Å,°)*** α 90.00 β 124.41(0) γ 90.00

R-Factor (%): 3.49 ***Temperature(K):*** 100 ***Density(g/cm3):*** 1.447

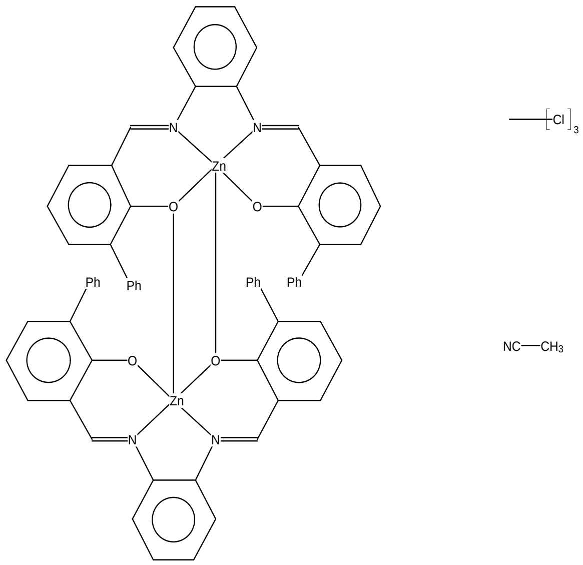

REWDEE

Reference: D.Anselmo, G.Salassa, E.C.Escudero-Adan, E.Martin, A.W.Kleij (2013) *Dalton Trans.* ,**42,**7962

Formula: C_{89} H_{98} N_6 O_9 Zn_3,1.5(C_1 H_4 O_1)

Compound Name: (μ$_3$-2,2',2'',2''',2'''',2'''''-(Pentacyclo[6.6.6.0^{2,7}.0^{9,14}.0^{15,20}]icosa-2,4,6,9,11,13,15,17,19-nonaene-4,5,11,12,17,18-hexaylhexakis(nitrilomethylylidene))hexakis(6-t-butylphenolato))-trimethanol-tri-zinc(ii) methanol solvate

Space Group: Pnna ***Cell:*** ***a*** 27.692(7) ***b*** 23.442(6) ***c*** 14.633(3)
Space Group No.: 52 ***(Å,°)*** α 90.00 β 90.00 γ 90.00

R-Factor (%): 9.93 ***Temperature(K):*** 100 ***Density(g/cm3):*** 1.147

Zn_3--
--[μ$_3$-$C_{62}H_{32}N_6O_6$(Bu^t)$_6$-κ^{6}N,N',N'',N''',N'''',N''''',κ^{6}O,O',O'',O''',O'''',O''''']$[HOMe]_3$

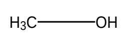

SIHTEK

Reference: Wen-Bin Sun, Bing-Lu Han, Po-Heng Lin, Hong-Feng Li, Peng Chen, Yong-Mei Tian, M.Murugesu, Peng-Fei Yan (2013) *Dalton Trans.* ,**42,**13397

Formula: C_{50} H_{44} Er_2 N_4 O_9,C_4 H_{10} O_1

Compound Name: (μ$_2$-2,2'-(1,2-Phenylenebis(nitrilomethylylidene))diphenolato)-bis(acetylacetonato-O,O')-aqua-(2,2'-(1,2-phenylenebis(nitrilomethylylidene))diphenolato)-di-erbium diethyl ether solvate

Space Group: P21/n ***Cell:*** ***a*** 16.020(1) ***b*** 19.639(1) ***c*** 16.227(1)
Space Group No.: 14 ***(Å,°)*** α 90.00 β 90.72(0) γ 90.00

R-Factor (%): 5.14 ***Temperature(K):*** 296 ***Density(g/cm3):*** 1.631

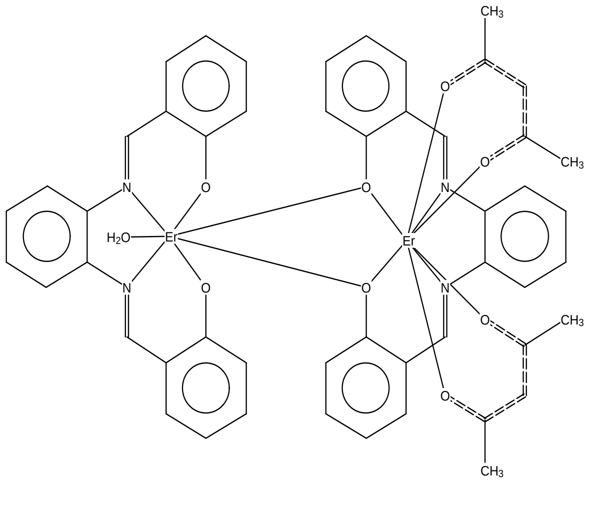

SIHTIO

Reference: Wen-Bin Sun, Bing-Lu Han, Po-Heng Lin, Hong-Feng Li, Peng Chen, Yong-Mei Tian, M.Murugesu, Peng-Fei Yan (2013) *Dalton Trans.* ,**42,**13397

Formula: $C_{50} H_{44} N_4 O_9 Tb_2$

Compound Name: (μ_2-2,2'-(1,2-Phenylenebis(nitrilomethylylidene))diphenolato)-bis(acetylacetonato-O,O')-aqua-(2,2'-(1,2-phenylenebis(nitrilomethylylidene))diphenolato)-di-terbium

Space Group: P21/n ***Cell:*** ***a*** 12.976(0) ***b*** 19.413(0) ***c*** 19.328(0)
Space Group No.: 14 ***(Å,°)*** α 90.00 β 106.25(0) γ 90.00

R-Factor (%): 3.44 ***Temperature(K):*** 296 ***Density(g/cm³):*** 1.652

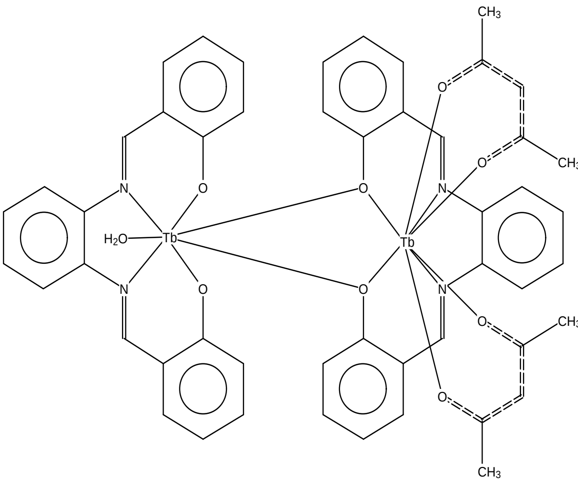

SIHTOU

Reference: Wen-Bin Sun, Bing-Lu Han, Po-Heng Lin, Hong-Feng Li, Peng Chen, Yong-Mei Tian, M.Murugesu, Peng-Fei Yan (2013) *Dalton Trans.* ,**42,**13397

Formula: $C_{50} H_{44} Gd_2 N_4 O_9,2(C_1 H_2 Cl_2)$

Compound Name: (μ_2-2,2'-(1,2-Phenylenebis(nitrilomethylylidene))diphenolato)-bis(acetylacetonato-O,O')-aqua-(2,2'-(1,2-phenylenebis(nitrilomethylylidene))diphenolato)-di-gadolinium dichloromethane solvate

Space Group: P21/n ***Cell:*** ***a*** 15.471(0) ***b*** 19.517(0) ***c*** 17.873(0)
Space Group No.: 14 ***(Å,°)*** α 90.00 β 91.07(0) γ 90.00

R-Factor (%): 3.52 ***Temperature(K):*** 296 ***Density(g/cm³):*** 1.636

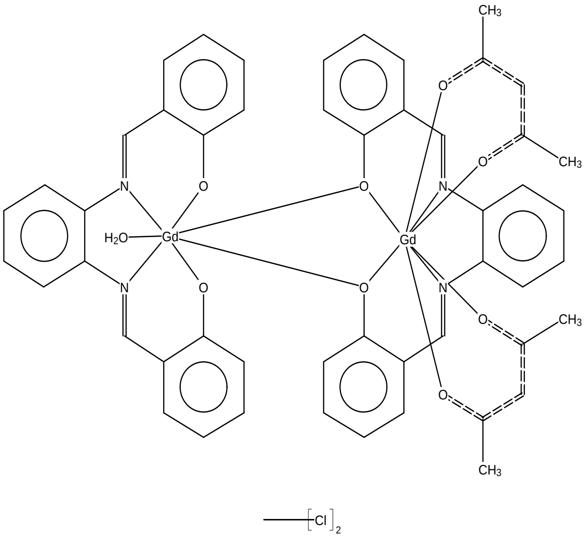

TEYXUS

Reference: Quan Li, Shuo Chen, Pengfei Yan, Peng Chen, Guangfeng Hou, Guangming Li (2013) *J.Coord.Chem.* ,**66,**1084

Formula: $C_{41} H_{32} Ho_1 N_7 O_{14} Zn_2,C_1 H_4 O_1,C_1 H_2 Cl_2$

Compound Name: bis(μ_2-2,2'-(1,2-Phenylenebis(nitrilomethylylidene))diphenolato)-trinitrato-methanol-holmium-di-zinc methanol dichloromethane solvate

Space Group: P-1 ***Cell:*** ***a*** 11.033(2) ***b*** 12.388(3) ***c*** 19.409(4)
Space Group No.: 2 ***(Å,°)*** α 73.77(3) β 79.45(3) γ 68.05(3)

R-Factor (%): 3.80 ***Temperature(K):*** 293 ***Density(g/cm³):*** 1.777

$HoZn_2[\mu_2\text{-}C_{20}H_{14}N_2O_2\text{-}\kappa^2N,N',\kappa^4O,O']_2[HOMe][NO_3\text{-}\kappa^2O,O']_3$

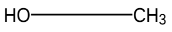

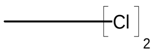

TEYYAZ

Reference: Quan Li, Shuo Chen, Pengfei Yan, Peng Chen, Guangfeng Hou, Guangming Li (2013) *J.Coord.Chem.* ,**66,**1084

Formula: $C_{41} H_{32} Eu_1 N_7 O_{14} Zn_2,C_1 H_4 O_1,C_1 H_2 Cl_2$

Compound Name: bis(μ_2-2,2'-(1,2-Phenylenebis(nitrilomethylylidene))diphenolato)-trinitrato-methanol-europium-di-zinc methanol dichloromethane solvate

Space Group: P-1 ***Cell:*** ***a*** 11.052(2) ***b*** 12.394(3) ***c*** 19.440(4)
Space Group No.: 2 ***(Å,°)*** α 73.57(3) β 79.36(3) γ 67.93(3)

R-Factor (%): 4.40 ***Temperature(K):*** 293 ***Density(g/cm³):*** 1.756

$EuZn_2[\mu_2\text{-}C_{20}H_{14}N_2O_2\text{-}\kappa^2N,N',\kappa^4O,O']_2[HOMe][NO_3\text{-}\kappa^2O,O']_3$

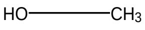

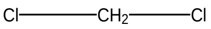

Search: search26 (Tue Dec 2 15:48:50 2014): Hits 737-740

TEYYED

Reference: Quan Li, Shuo Chen, Pengfei Yan, Peng Chen, Guangfeng Hou, Guangming Li (2013) *J.Coord.Chem.* ,**66,**1084

Formula: C_{42} H_{36} N_7 Nd_1 O_{15} Zn_2,C_1 H_2 Cl_2,C_1 H_4 O_1

Compound Name: bis(μ_2-2,2'-(1,2-Phenylenebis(nitrilomethylylidene))diphenolato)-trinitrato-dimethanol-neodymium-di-zinc methanol dichloromethane solvate

Space Group:	P21/c	***Cell:***	***a***	12.280(3)	***b***	29.186(6)	***c***	18.163(6)
Space Group No.:	14	***(Å,°)***	α	90.00	β	130.21(2)	γ	90.00
R-Factor (%):	8.12	***Temperature(K):***	293		***Density(g/cm3):***	1.698		

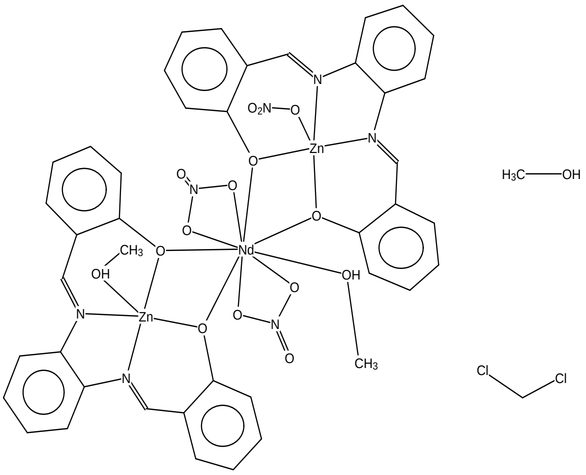

TEYYIH

Reference: Quan Li, Shuo Chen, Pengfei Yan, Peng Chen, Guangfeng Hou, Guangming Li (2013) *J.Coord.Chem.* ,**66,**1084

Formula: C_{42} H_{36} N_7 O_{15} Pr_1 Zn_2,C_1 H_4 O_1,C_1 H_2 Cl_2

Compound Name: bis(μ_2-2,2'-(1,2-Phenylenebis(nitrilomethylylidene))diphenolato)-trinitrato-dimethanol-praseodymium-di-zinc methanol dichloromethane solvate

Space Group:	P21/c	***Cell:***	***a***	12.297(3)	***b***	29.245(6)	***c***	18.129(6)
Space Group No.:	14	***(Å,°)***	α	90.00	β	129.90(2)	γ	90.00
R-Factor (%):	7.58	***Temperature(K):***	293		***Density(g/cm3):***	1.683		

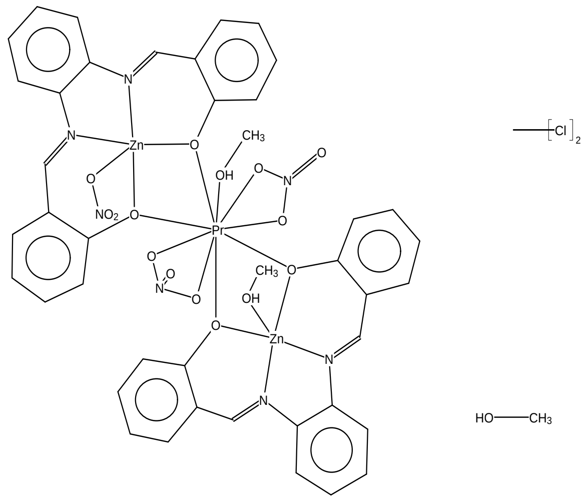

VILWIY

Reference: A.M.A.Dief, R.D.Torres, E.C.Sanudo, L.H.A.Rahman, N.A.Alcalde (2013) *Polyhedron* ,**64,**203

Formula: C_{60} H_{38} Br_6 N_6 Nd_2 O_7,2(C_1 H_1 Cl_3)

Compound Name: (μ_2-2,2'-(1,2-phenylenebis((nitrilo)methylylidene))bis(4-bromophenolato))-bis(2,2'-(1,2-phenylenebis((nitrilo)methylylidene))bis(4-bromophenolato))-aqua-di-neodymium chloroform solvate

Space Group:	P21/c	***Cell:***	***a***	15.233(0)	***b***	18.638(0)	***c***	23.477(1)
Space Group No.:	14	***(Å,°)***	α	90.00	β	96.03(0)	γ	90.00
R-Factor (%):	9.46	***Temperature(K):***	101		***Density(g/cm3):***	1.966		

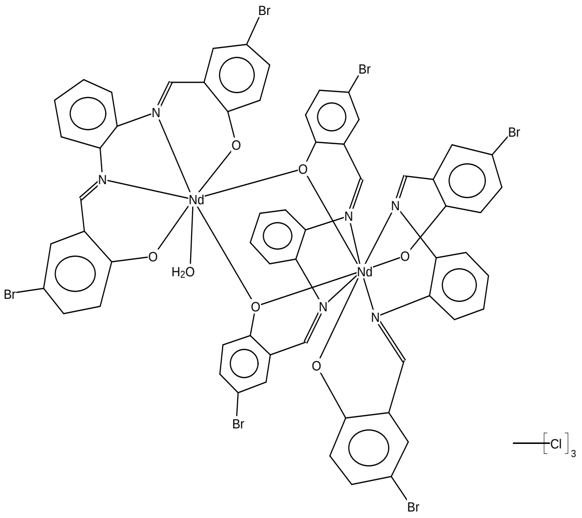

VINGAC

Reference: A.M.A.Dief, R.D.Torres, E.C.Sanudo, L.H.A.Rahman, N.A.Alcalde (2013) *Polyhedron* ,**64,**203

Formula: C_{60} H_{38} Br_6 N_6 O_7 Tb_2,2(C_1 H_1 Cl_3)

Compound Name: (μ_2-2,2'-(1,2-Phenylenebis(nitrilomethylylidene))bis(4-bromophenolato))-bis(2,2'-(1,2-Phenylenebis(nitrilomethylylidene))bis(4-bromophenoato))-aqua-di-terbium chloroform solvate

Space Group:	Pbcn	***Cell:***	***a***	29.096(0)	***b***	22.718(0)	***c***	19.652(0)
Space Group No.:	60	***(Å,°)***	α	90.00	β	90.00	γ	90.00
R-Factor (%):	7.05	***Temperature(K):***	101		***Density(g/cm3):***	2.036		

$Tb_2[\mu_2\text{-}C_{20}H_{12}Br_2N_2O_2\text{-}\kappa^2N,N',\kappa^4O,O']\text{--}$
$\text{--}[C_{20}H_{12}Br_2N_2O_2\text{-}\kappa^2N,N',\kappa^2O,O']_2[H_2O]$

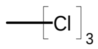

WEXHIS

Reference: Quan Li, Shuo Chen, Pengfei Yan, Peng Chen, Guangfeng Hou, Guangming Li (2013) *J.Coord.Chem.* ,**66,**1084

Formula: $C_{41}\ H_{32}\ N_7\ O_{14}\ Yb_1\ Zn_2,C_1\ H_4\ O_1,C_1\ H_2\ Cl_2$

Compound Name: bis(μ_2-2,2'-(1,2-Phenylenebis(nitrilomethylylidene))diphenolato)-tris(nitrato)-(methanol)-ytterbium-di-zinc dichloromethane methanol solvate

Space Group:	P-1	***Cell:***	***a*** 11.015(2)	***b*** 12.366(3)	***c*** 19.380(4)		
Space Group No.:	2	***(Å,°)***	α 73.79(3)	β 79.66(3)	γ 68.12(3)		

R-Factor (%): 4.04 ***Temperature(K):*** 293 ***Density(g/cm^3):*** 1.796

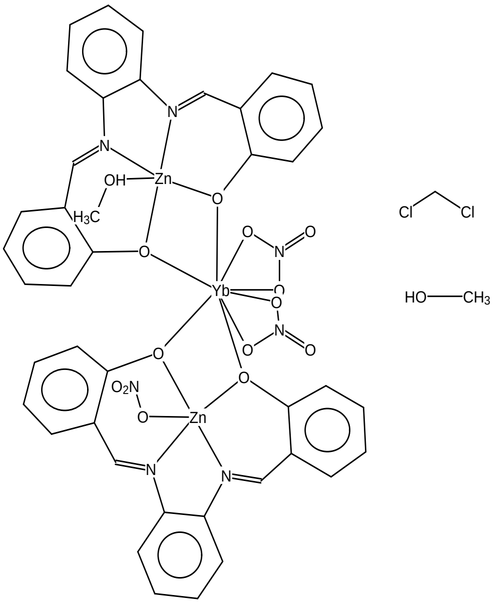

WEXQIB

Reference: Quan Li, Shuo Chen, Pengfei Yan, Peng Chen, Guangfeng Hou, Guangming Li (2013) *J.Coord.Chem.* ,**66,**1084

Formula: $C_{41}\ H_{32}\ Er_1\ N_7\ O_{14}\ Zn_2,C_1\ H_4\ O_1,C_1\ H_2\ Cl_2$

Compound Name: bis(μ_2-2,2'-(1,2-Phenylenebis(nitrilomethylylidene))diphenolato)-tris(nitrato)-(methanol)-erbium-di-zinc dichloromethane methanol solvate

Space Group:	P-1	***Cell:***	***a*** 11.045(2)	***b*** 12.386(3)	***c*** 19.394(4)
Space Group No.:	2	***(Å,°)***	α 73.88(3)	β 79.27(3)	γ 67.94(3)

R-Factor (%): 3.56 ***Temperature(K):*** 293 ***Density(g/cm^3):*** 1.781

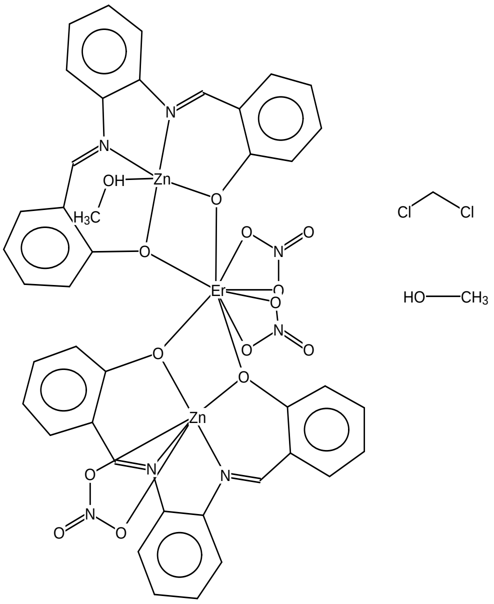

XEXTOL

Reference: D.Anselmo, R.Gramage-Doria, T.Besset, M.V.Escarcega-Bobadilla, G.Salassa, E.C.Escudero-Adan, M.M.Belmonte, E.Martin, J.N.H.Reek, A.W.Kleij (2013) *Dalton Trans.* , **42,**7595

Formula: $C_{70}\ H_{88}\ N_7\ O_4\ P_1\ Zn_2,0.5(C_1\ H_2\ Cl_2)$

Compound Name: bis(2,4-di-t-butyl-6-(((2-((3-t-butyl-2-(hydroxy)benzylidene)amino)phenyl)imino)methyl)phenolato)-(μ_2-1,3,5-triaza-7-phosphatricyclo[3.3.1.1^{3,7}]decane)-di-zinc dichloromethane solvate

Space Group:	P21/c	***Cell:***	***a*** 14.378(0)	***b*** 30.829(1)	***c*** 17.512(0)
Space Group No.:	14	***(Å,°)***	α 90.00	β 110.96(0)	γ 90.00

R-Factor (%): 5.88 ***Temperature(K):*** 100 ***Density(g/cm^3):*** 1.187

$Zn_2[\mu_2\text{-}C_6H_{12}N_3P\text{-}\kappa^2N,N'][C_{20}H_{11}N_2O_2(Bu^t)_3\text{-}\kappa^2N,N',\kappa^2O,O']_2$

—[Cl]$_2$

XEXTUR

Reference: D.Anselmo, R.Gramage-Doria, T.Besset, M.V.Escarcega-Bobadilla, G.Salassa, E.C.Escudero-Adan, M.M.Belmonte, E.Martin, J.N.H.Reek, A.W.Kleij (2013) *Dalton Trans.* , **42,**7595

Formula: $C_{70}\ H_{88}\ N_7\ O_4\ P_1\ Zn_2,2(C_3\ H_6\ O_1)$

Compound Name: bis(2,2'-((4-t-butyl-1,2-phenylene)bis((nitrilo)methylylidene))bis(6-t-butylphenolato))-(μ_2-1,3,5-triaza-7-phosphatricyclo[3.3.1.1^{3,7}]decane)-di-zinc acetone solvate

Space Group:	P21/c	***Cell:***	***a*** 16.350(1)	***b*** 15.101(1)	***c*** 29.107(2)
Space Group No.:	14	***(Å,°)***	α 90.00	β 93.35(0)	γ 90.00

R-Factor (%): 5.47 ***Temperature(K):*** 100 ***Density(g/cm^3):*** 1.268

$Zn_2[\mu_2\text{-}C_6H_{12}N_3P\text{-}\kappa^2N,N'][C_{20}H_{11}N_2O_2(Bu^t)_3\text{-}\kappa^2N,N',\kappa^2O,O']_2$

MeCO——CH_3

Search: search26 (Tue Dec 2 15:48:50 2014): Hits 745-748

XEXVAZ

Reference: D.Anselmo, R.Gramage-Doria, T.Besset, M.V.Escarcega-Bobadilla, G.Salassa, E.C.Escudero-Adan, M.M.Belmonte, E.Martin, J.N.H.Reek, A.W.Kleij (2013) *Dalton Trans.* , **42,**7595

Formula: $C_{90} H_{102} N_9 O_6 P_1 Zn_3,0.5(C_1 H_2 Cl_2)$

Compound Name: (μ_3- 1,3,5-Triaza-7-phosphatricyclo[3.3.1.1^{3,7}]decane)-tris(2,2'-(1,2-phenylenebis(nitrilomethylylidene))bis(6-t-butylphenolato))-tri-zinc(ii) dichloromethane solvate

Space Group:	Cc	***Cell:***	***a*** 29.253(3)	***b*** 16.982(1)	***c*** 33.515(3)		
Space Group No.:	9	***(Å,°)***	α 90.00	β 100.63(0)	γ 90.00		
R-Factor (%):	4.35	***Temperature(K):***	100	***Density(g/cm^3):***	1.360		

$Zn_3[\mu_3\text{-}C_6H_{12}N_3P\text{-}\kappa^3N,N',N''][C_{20}H_{12}N_2O_2(Bu^t)_2\text{-}\kappa^2N,N',\kappa^2O,O']_3$

Cl———CH$_2$———Cl

XEXVED

Reference: D.Anselmo, R.Gramage-Doria, T.Besset, M.V.Escarcega-Bobadilla, G.Salassa, E.C.Escudero-Adan, M.M.Belmonte, E.Martin, J.N.H.Reek, A.W.Kleij (2013) *Dalton Trans.* , **42,**7595

Formula: $C_{62} H_{68} Cl_4 N_7 O_4 P_1 Zn_2,C_2 H_3 N_1$

Compound Name: bis(2,2'-((4,5-dichloro-1,2-phenylene)bis((nitrilo)methylylidene))bis(6-t-butylphenolato))-(μ_2-1,3,5-triaza-7-phosphatricyclo[3.3.1.1^{3,7}]decane)-di-zinc acetonitrile solvate

Space Group:	C2/c	***Cell:***	***a*** 30.798(2)	***b*** 13.568(0)	***c*** 29.886(2)		
Space Group No.:	15	***(Å,°)***	α 90.00	β 101.81(0)	γ 90.00		
R-Factor (%):	3.10	***Temperature(K):***	100	***Density(g/cm^3):***	1.434		

$Zn_2[\mu_2\text{-}C_6H_{12}N_3P\text{-}\kappa^2N,N'][C_{20}H_{10}Cl_2N_2O_2(Bu^t)_2\text{-}\kappa^2N,N',\kappa^2O,O']_2$

NC———CH$_3$

XEXVIH

Reference: D.Anselmo, R.Gramage-Doria, T.Besset, M.V.Escarcega-Bobadilla, G.Salassa, E.C.Escudero-Adan, M.M.Belmonte, E.Martin, J.N.H.Reek, A.W.Kleij (2013) *Dalton Trans.* , **42,**7595

Formula: $C_{78} H_{72} F_6 N_9 O_6 P_1 Zn_3,0.25(H_2 O_1)$

Compound Name: (μ_3-1,3,5-Triaza-7-phosphatricyclo[3.3.1.1^{3,7}]decane)-tris(2-[((2-[(3-t-butyl-2-oxidobenzylidene)amino]phenyl)imino)methyl]-4,6-difluorophenolato)-tri-zinc hydrate

Space Group:	C2/c	***Cell:***	***a*** 18.155(0)	***b*** 31.546(0)	***c*** 25.243(0)		
Space Group No.:	15	***(Å,°)***	α 90.00	β 105.48(0)	γ 90.00		
R-Factor (%):	4.97	***Temperature(K):***	100	***Density(g/cm^3):***	1.504		

$Zn_3[\mu_3\text{-}C_6H_{12}N_3P\text{-}\kappa^3N,N',N''][C_{20}H_{11}F_2N_2O_2(Bu^t)\text{-}\kappa^2N,N',\kappa^2O,O']_3$

H_2O

XEXVON

Reference: D.Anselmo, R.Gramage-Doria, T.Besset, M.V.Escarcega-Bobadilla, G.Salassa, E.C.Escudero-Adan, M.M.Belmonte, E.Martin, J.N.H.Reek, A.W.Kleij (2013) *Dalton Trans.* , **42,**7595

Formula: $C_{78} H_{104} N_7 O_4 P_1 Zn_2,3(C_2 H_3 N_1)$

Compound Name: bis(2,2'-(1,2-phenylenebis((nitrilo)methylylidene))bis(4,6-di-t-butylphenolato))-(μ_2-1,3,5-triaza-7-phosphatricyclo[3.3.1.1^{3,7}]decane)-di-zinc acetonitrile solvate

Space Group:	P-1	***Cell:***	***a*** 15.432(0)	***b*** 18.224(1)	***c*** 31.756(2)		
Space Group No.:	2	***(Å,°)***	α 84.46(0)	β 83.05(0)	γ 74.35(0)		
R-Factor (%):	6.48	***Temperature(K):***	100	***Density(g/cm^3):***	1.161		

$Zn_2[\mu_2\text{-}C_6H_{12}N_3P\text{-}\kappa^2N,N'][C_{20}H_{10}N_2O_2(Bu^t)_4\text{-}\kappa^2N,N',\kappa^2O,O']_2$

NC———CH$_3$

Search: search26 (Tue Dec 2 15:48:50 2014): Hits 749-752

XIBSIM

Reference: Cheng-Feng Zhu, Feng-Jun Ban, En-Hong Sheng, Sai-Jing Zheng, Bai-Zhan Liu, Yong Cui (2013) *Jiegou Huaxue(Chin.J.Struct.Chem.)* ,**32,**205

Formula: $(C_{76} H_{84} N_4 O_{20} S_4 Zn_5)$n,6n$(C_2 H_6 O_1 S_1)$

Compound Name: catena-(bis(μ_5-3,3'-(1,2-Phenylenebis(nitrilomethylylidene))bis(5-t-butyl-4-oxybenzoato))-(μ_4-terephthalato)-tetrakis(dimethylsulfoxide)-penta-zinc dimethylsulfoxide solvate)

Space Group:	P-1	***Cell:***	***a*** 11.386(0)	***b*** 13.264(0)	***c*** 17.550(0)		
Space Group No.:	2	***(Å,°)***	α 92.22(0)	β 94.51(0)	γ 96.06(0)		
R-Factor (%):	3.80	***Temperature(K):***	173	***Density(g/cm³):***	1.454		

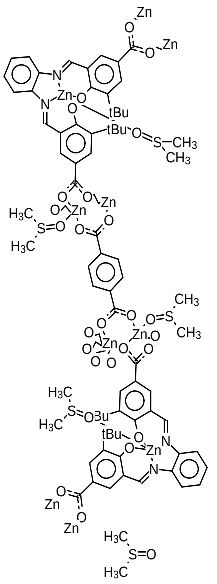

XILMIQ

Reference: Hailong Wang, Chenxi Liu, Tao Liu, Suyuan Zeng, Wei Cao, Qi Ma, Chunying Duan, Jianmin Dou, Jianzhuang Jiang (2013) *Dalton Trans.* ,**42,**15355

Formula: $C_{66} H_{56} Gd_2 N_6 O_{13}$,2$(H_2 O_1)$

Compound Name: (μ_2-2-(((2-((2-Oxy-3-methoxybenzylidene)amino)phenyl)imino)methyl)-6-methoxyphenolato)-bis(2,2'-(1,2-phenylenebis((nitrilo)methylylidene)) bis(6-methoxyphenolato))-aqua-di-gadolinium dihydrate

Space Group:	Pna21	***Cell:***	***a*** 21.873(1)	***b*** 12.402(0)	***c*** 23.739(1)		
Space Group No.:	33	***(Å,°)***	α 90.00	β 90.00	γ 90.00		
R-Factor (%):	4.36	***Temperature(K):***	293	***Density(g/cm³):***	1.539		

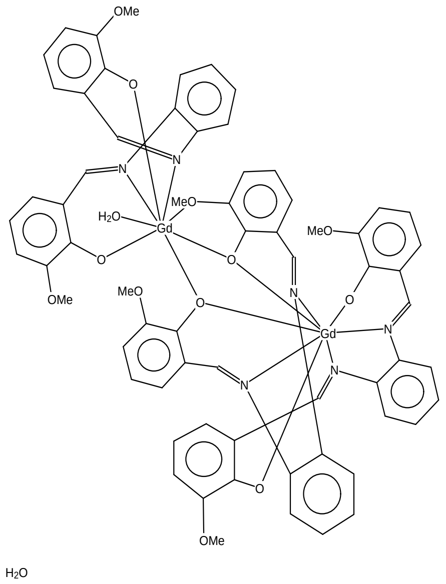

XILMOW

Reference: Hailong Wang, Chenxi Liu, Tao Liu, Suyuan Zeng, Wei Cao, Qi Ma, Chunying Duan, Jianmin Dou, Jianzhuang Jiang (2013) *Dalton Trans.* ,**42,**15355

Formula: $C_{66} H_{56} Dy_2 N_6 O_{13}$,2$(H_2 O_1)$

Compound Name: (μ_2-2-(((2-((2-Oxy-3-methoxybenzylidene)amino)phenyl)imino)methyl)-6-methoxyphenolato)-bis(2,2'-(1,2-phenylenebis((nitrilo)methylylidene)) bis(6-methoxyphenolato))-aqua-di-dysprosium dihydrate

Space Group:	Pna21	***Cell:***	***a*** 21.773(10)	***b*** 12.426(0)	***c*** 23.721(0)		
Space Group No.:	33	***(Å,°)***	α 90.00	β 90.00	γ 90.00		
R-Factor (%):	4.34	***Temperature(K):***	293	***Density(g/cm³):***	1.555		

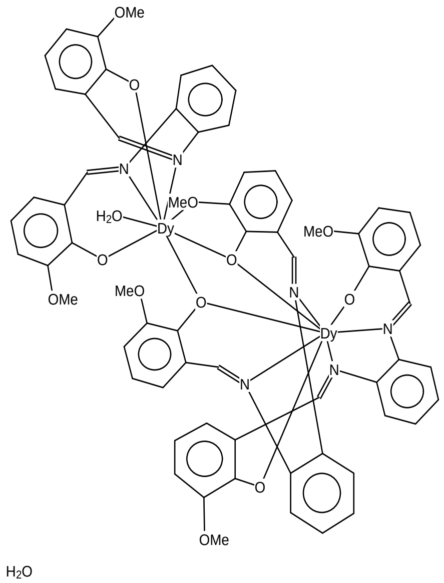

XILTOD

Reference: Hailong Wang, Chenxi Liu, Tao Liu, Suyuan Zeng, Wei Cao, Qi Ma, Chunying Duan, Jianmin Dou, Jianzhuang Jiang (2013) *Dalton Trans.* ,**42,**15355

Formula: $C_{86} H_{52} Dy_2 N_{18} O_5$,3$(C_1 H_1 Cl_3)$

Compound Name: Aqua-(μ_2-2,2'-(1,2-phenylenebis((nitrilo)methylylidene))bis(6-methoxyphenolato))-bis(phthalocyanine)-di-dysprosium chloroform solvate

Space Group:	P-1	***Cell:***	***a*** 12.282(0)	***b*** 12.696(0)	***c*** 14.856(0)		
Space Group No.:	2	***(Å,°)***	α 115.29(0)	β 107.33(0)	γ 91.93(0)		
R-Factor (%):	5.79	***Temperature(K):***	120	***Density(g/cm³):***	1.776		

$Dy_2[\mu_2\text{-}C_{20}H_{12}N_2O_2(OMe)_2\text{-}\kappa^2N,N',\kappa^4O,O']$--
--$[C_{32}H_{16}N_8\text{-}\kappa^4N,N',N'',N''']_2[H_2O]$

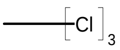

Search: search26 (Tue Dec 2 15:48:50 2014): Hits 753-756

XILWAS

Reference: Hailong Wang, Chenxi Liu, Tao Liu, Suyuan Zeng, Wei Cao, Qi Ma, Chunying Duan, Jianmin Dou, Jianzhuang Jiang (2013) *Dalton Trans.* ,**42,**15355

Formula: $C_{86}\ H_{52}\ Gd_2\ N_{18}\ O_5$,3($C_1\ H_1\ Cl_3$)

Compound Name: Aqua-(μ_2-2,2'-(1,2-phenylenebis((nitrilo-1κN)methylylidene))bis(6-methoxyphenolato))-bis(phthalocyanine)-di-gadolinium chloroform solvate

Space Group:	P-1	***Cell:***	***a*** 12.345(0)	***b*** 12.688(0)	***c*** 14.899(1)
Space Group No.:	2	***(Å,°)***	α 115.12(0)	β 107.45(0)	γ 91.66(0)

R-Factor (%): 6.42 ***Temperature(K):*** 120 ***Density(g/cm³):*** 1.751

$Gd_2[\mu_2\text{-}C_{20}H_{12}N_2O_2(OMe)_2\text{-}\kappa^2N,N',\kappa^4O,O']$--
--$[C_{32}H_{16}N_8\text{-}\kappa^4N,N',N'',N''']_2[H_2O]$

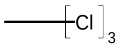

BOFTEX

Reference: P.Biswas, P.Bag, A.K.Dutta, U.Florke, K.Nag (2014) *Polyhedron* ,**75,**118

Formula: $C_{116}\ H_{120}\ Fe_4\ N_8\ O_{10}$,2($C_2\ H_3\ N_1$),$H_2\ O_1$

Compound Name: bis(μ_2-5,20,26,41-tetra-t-butyl-9,16,30,37-tetraazaheptacyclo[37.3.1.1^{3,7}.1^{18,22}.1^{24,28}.0^{10,15}.0^{31,36}]hexatetraconta-1(43),3(46),4,6,8,10,12,14,16,18(45),19,21,24(44),25,27,29,31,33,35,37,39,41-docosaene-43,44,45,46-tetrolato)-bis(μ_2-oxido)-tetra-iron(iii) acetonitrile solvate monohydrate

Space Group:	Ccca	***Cell:***	***a*** 22.656(5)	***b*** 24.799(5)	***c*** 18.338(4)
Space Group No.:	68	***(Å,°)***	α 90.00	β 90.00	γ 90.00

R-Factor (%): 25.50 ***Temperature(K):*** 120 ***Density(g/cm³):*** 1.360

Fe_4--
--$[\mu_2\text{-}C_{42}H_{24}N_4O_4(Bu^t)_4\text{-}\kappa^4N,N',N'',N''',\kappa^4O,O',O'',O''']_2[\mu_2\text{-}O]_2$

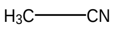

H_2O

BOFTIB

Reference: P.Biswas, P.Bag, A.K.Dutta, U.Florke, K.Nag (2014) *Polyhedron* ,**75,**118

Formula: $C_{58}\ H_{60}\ Cl_2\ Mn_2\ N_4\ O_4$,3($H_2\ O_1$)

Compound Name: dichloro-(μ_2-5,20,26,41-tetra-t-butyl-9,16,30,37-tetraazaheptacyclo[37.3.1.1^{3,7}.1^{18,22}.1^{24,28}.0^{10,15}.0^{31,36}]hexatetraconta-1(43),3(46),4,6,8,10,12,14,16,18(45),19,21,24(44),25,27,29,31,33,35,37,39,41-docosaene-43,44,45,46-tetrolato)-di-manganese trihydrate

Space Group:	P-1	***Cell:***	***a*** 9.002(4)	***b*** 12.405(5)	***c*** 12.675(5)
Space Group No.:	2	***(Å,°)***	α 82.09(0)	β 84.42(0)	γ 72.09(0)

R-Factor (%): 8.31 ***Temperature(K):*** 123 ***Density(g/cm³):*** 1.387

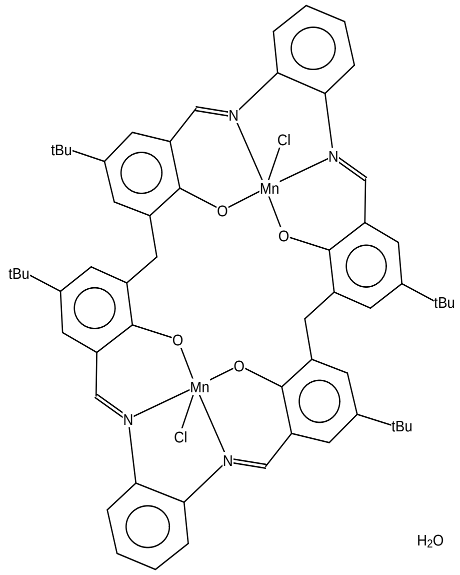

CITXIO

Reference: Feng Gao, Long Cui, Wei Liu, Liang Hu, Yu-Wu Zhong, Yi-Zhi Li, Jing-Lin Zuo (2013) *Inorg.Chem.* ,**52,**11164

Formula: $C_{43}\ H_{51}\ Cl_2\ Co_1\ Dy_1\ N_2\ O_{11}\ P_3\ S_6$,0.25($H_2\ O_1$)

Compound Name: tris(μ_2-bis(ethanolato)(dihydrido)oxophosphato)-(η^5-cyclopentadienyl)-(2,2'-((2-(4,5-bis(methylsulfanyl)-1,3-dithiol-2-ylidene)-1,3-benzodithiole-5,6-diyl)bis(nitrilomethylylidene))bis(4-chlorophenolato))-cobalt-dysprosium hydrate

Space Group:	P21/n	***Cell:***	***a*** 14.921(1)	***b*** 19.995(1)	***c*** 19.974(1)
Space Group No.:	14	***(Å,°)***	α 90.00	β 105.09(0)	γ 90.00

R-Factor (%): 6.07 ***Temperature(K):*** 123 ***Density(g/cm³):*** 1.563

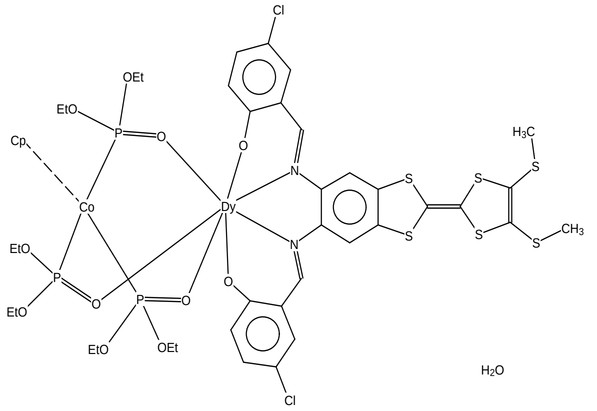

CITXOU

Reference: Feng Gao, Long Cui, Wei Liu, Liang Hu, Yu-Wu Zhong, Yi-Zhi Li, Jing-Lin Zuo (2013) *Inorg.Chem.* ,**52,**11164

Formula: $C_{43} H_{51} Cl_2 Co_1 N_2 O_{11} P_3 S_6 Tb_1$,0.25($H_2 O_1$)

Compound Name: tris(μ_2-bis(ethanolato)(dihydrido)oxophosphato)-(η^5-cyclopentadienyl)-(2,2'-((2-(4,5-bis(methylsulfanyl)-1,3-dithiol-2-ylidene)-1,3-benzodithiole-5,6-diyl)bis(nitrilomethylylidene))bis(4-chlorophenolato))-cobalt-terbium hydrate

Space Group: P21/n **Cell:** ***a*** 14.827(1) ***b*** 19.905(1) ***c*** 20.378(1)
Space Group No.: 14 ***(Å,°)*** α 90.00 β 105.30(0) γ 90.00

R-Factor (%): 4.87 **Temperature(K):** 123 **Density(g/cm^3):** 1.546

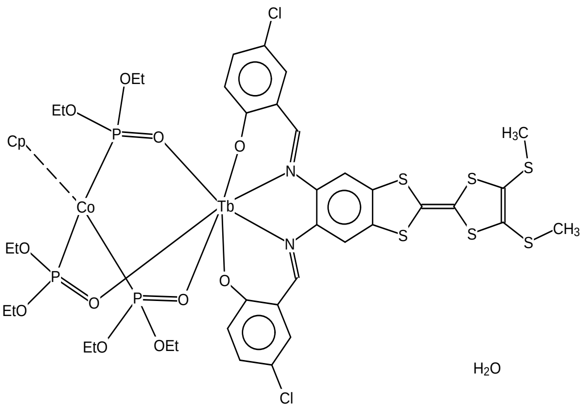

CITXUA

Reference: Feng Gao, Long Cui, Wei Liu, Liang Hu, Yu-Wu Zhong, Yi-Zhi Li, Jing-Lin Zuo (2013) *Inorg.Chem.* ,**52,**11164

Formula: $C_{43} H_{51} Cl_2 Co_1 Ho_1 N_2 O_{11} P_3 S_6$,0.25($H_2 O_1$)

Compound Name: tris(μ_2-bis(ethanolato)(dihydrido)oxophosphato)-(η^5-cyclopentadienyl)-(2,2'-((2-(4,5-bis(methylsulfanyl)-1,3-dithiol-2-ylidene)-1,3-benzodithiole-5,6-diyl)bis(nitrilomethylylidene))bis(4-chlorophenolato))-cobalt-holmium hydrate

Space Group: P21/n **Cell:** ***a*** 14.902(1) ***b*** 20.114(1) ***c*** 20.292(1)
Space Group No.: 14 ***(Å,°)*** α 90.00 β 105.57(0) γ 90.00

R-Factor (%): 5.24 **Temperature(K):** 123 **Density(g/cm^3):** 1.538

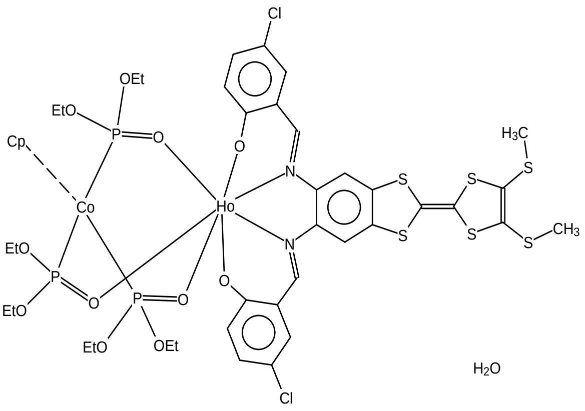

COBTIY

Reference: Qinghua Meng, J.K.Clegg, A.J.Brock, K.A.Jolliffe, L.F.Lindoy, Gang Wei (2014) *Polyhedron* ,**74,**113

Formula: $C_{112} H_{120} Fe_4 N_8 O_{10}$,3($C_4 H_8 O_1$),0.8($C_2 H_6 O_1$),1.2($H_2 O_1$)

Compound Name: bis(μ_2-2,2'-(1,2-Phenylenebis(nitrilomethylylidene))bis(4-t-butylphenolato))-bis(μ_2-oxo)-bis(2,2'-(1,2-phenylenebis(nitrilomethylylidene))bis(4-t-butylphenolato))-tetra-iron ethanol tetrahydrofuran solvate hydrate

Space Group: P-1 **Cell:** ***a*** 12.083(5) ***b*** 14.335(6) ***c*** 18.401(8)
Space Group No.: 2 ***(Å,°)*** α 72.46(0) β 86.68(0) γ 82.31(0)

R-Factor (%): 6.34 **Temperature(K):** 150 **Density(g/cm^3):** 1.233

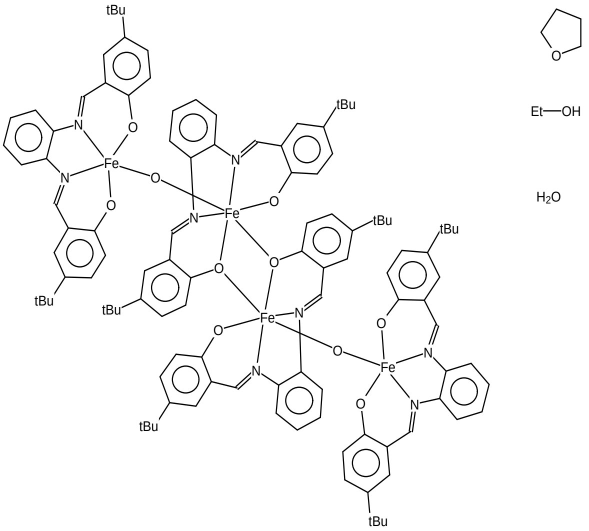

DIJMOA

Reference: S.Akine, S.Piao, M.Miyashita, T.Nabeshima (2013) *Tetrahedron Lett.* ,**54,**6541

Formula: $C_{68} H_{56} N_6 O_9 S_3 Zn_3$,5($C_1 H_2 Cl_2$)

Compound Name: (μ_3-8,15,29,36,49,56-hexaazaundecacyclo[20.20.20.1^{2,6}.1^{17,21}.1^{23,27}.1^{38,42}.1^{43,47}.1^{58,62}.0^{9,14}.0^{30,35}.0^{50,55}]octahexaconta-2(68),3,5,7,9,11,13,15,17(67),18,20,23(66),24,26,28,30,32,34,36,38(65),39,41,43(64),44,46,48,50,52,54,56,58,60,62-tritriacontaene-63,64,65,66,67,68-hexaoxo)-tris(dimethyl sulfoxide)-tri-zinc dichloromethane solvate

Space Group: P21/n **Cell:** ***a*** 16.989(0) ***b*** 25.023(0) ***c*** 18.631(0)
Space Group No.: 14 ***(Å,°)*** α 90.00 β 106.57(0) γ 90.00

R-Factor (%): 3.72 **Temperature(K):** 120 **Density(g/cm^3):** 1.591

Zn_3--
--[μ_3-$C_{62}H_{38}N_6O_6$-κ^6N,N',N'',N''',N'''',N''''',κ^6O,O',O'',O''',O'''',O'''''][$OSMe_2$]$_3$

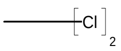

Search: search26 (Tue Dec 2 15:48:50 2014): Hits 761-764

DIJMUG

Reference: S.Akine, S.Piao, M.Miyashita, T.Nabeshima (2013) *Tetrahedron Lett.* ,**54,**6541

Formula: $C_{68} H_{56} N_6 O_9 S_3 Zn_3$,7.5($C_2 H_6 O_1 S_1$)

Compound Name: (μ_3-8,15,29,36,49,56-hexaazaundecacyclo[20.20.20.$1^{2,6}$.$1^{17,21}$.$1^{23,27}$.$1^{38,42}$.$1^{43,47}$.$1^{58,62}$.$0^{9,14}$.$0^{30,35}$.$0^{50,55}$]octahexaconta-2(68),3,5,7,9,11,13,15,17(67),18,20,23(66),24,26,28,30,32,34,36,38(65),39,41,43(64),44,46,48,50,52,54,56,58,60,62-tritriacontaene-63,64,65,66,67,68-hexaoxo)-tris(dimethyl sulfoxide)-tri-zinc dimethyl sulfoxide solvate

Space Group:	R-3	***Cell:***	***a*** 20.317(0)	***b*** 20.317(0)	***c*** 40.651(0)
Space Group No.:	148	***(Å,°)***	α 90.00	β 90.00	γ 120.00

R-Factor (%): 7.99 ***Temperature(K):*** 120 ***Density(g/cm³):*** 1.357

Zn_3--
--[μ_3-$C_{62}H_{38}N_6O_6$-κ^6N,N',N'',N''',N'''',N''''',κ^6O,O',O'',O''',O'''',O''''']$[OSMe_2]_3$

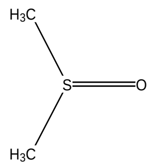

DOHFOX

Reference: E.A.Mikhalyova, A.V.Yakovenko, M.Zeller, K.S.Gavrilenko, S.E.Lofland, A.W.Addison, V.V.Pavlishchuk (2014) *Inorg.Chim.Acta* , **414,**97

Formula: $C_{60} H_{44} N_6 Nd_2 O_7$

Compound Name: (μ_2-2,2'-(1,2-Phenylenebis((nitrilo)methylylidene))diphenolato)-aqua-bis(2,2'-(1,2-phenylenebis((nitrilo)methylylidene))diphenolato)-di-neodymium

Space Group:	P-1	***Cell:***	***a*** 13.915(4)	***b*** 15.868(4)	***c*** 23.344(6)
Space Group No.:	2	***(Å,°)***	α 88.01(0)	β 84.82(0)	γ 79.63(0)

R-Factor (%): 7.87 ***Temperature(K):*** 100 ***Density(g/cm³):*** 1.644

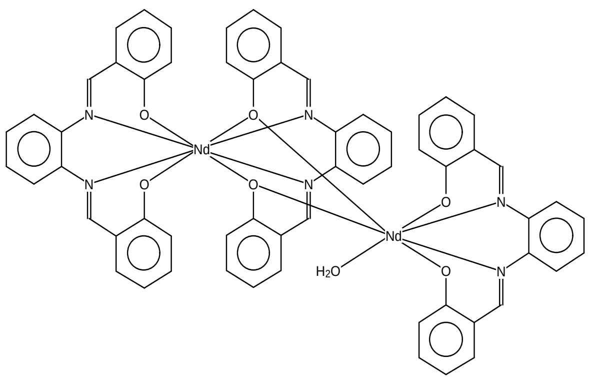

LISZEU

Reference: Guoqi Zhang (2014) *Inorg.Chem.Commun.* ,**40,**47

Formula: 2($C_{29} H_{34} N_2 O_3 Zn_1$),$C_{28} H_{32} N_2 O_3 Zn_1$,2($C_1 H_4 O_1$)

Compound Name: bis(methanol-(2,2'-(1,2-phenylenebis(nitrilomethylylidene))bis(6-t-butylphenolato))-zinc) aqua-(2,2'-(1,2-phenylenebis(nitrilomethylylidene))bis(6-t-butylphenolato))-zinc methanol solvate

Space Group:	P21	***Cell:***	***a*** 13.483(0)	***b*** 21.385(0)	***c*** 14.304(0)
Space Group No.:	4	***(Å,°)***	α 90.00	β 95.74(0)	γ 90.00

R-Factor (%): 3.09 ***Temperature(K):*** 173 ***Density(g/cm³):*** 1.313

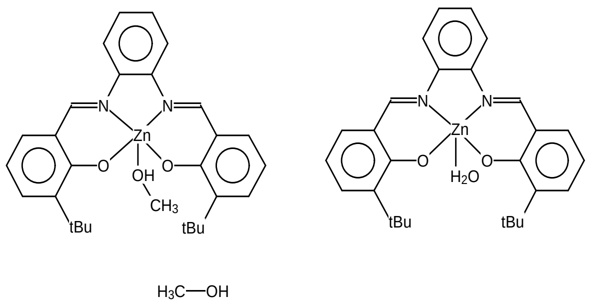

LOFKAU

Reference: H.Achira, M.Ito, T.Mutai, Isao Yoshikawa, K.Araki, H.Houjou (2014) *Dalton Trans.* ,**43,**5899

Formula: $C_{100} H_{132} N_4 Ni_2 O_8$,$C_2 H_6 O_1$

Compound Name: (μ_2-1-(((2-((3-(2-(3-(((4,5-bis(dodecyloxy)-2-(((2-(oxy)-1-naphthyl)methylene)amino)phenyl)imino)methyl)-2-(oxy)benzyl)prop-2-en-1-yl)-2-(oxy)benzylidene)amino)-4,5-bis(dodecyloxy)phenyl)imino)methyl)-2-naphtholato)-di-nickel ethanol solvate

Space Group:	P-1	***Cell:***	***a*** 13.544(7)	***b*** 19.397(7)	***c*** 20.246(10)
Space Group No.:	2	***(Å,°)***	α 64.07(2)	β 79.02(3)	γ 72.67(3)

R-Factor (%): 9.61 ***Temperature(K):*** 93 ***Density(g/cm³):*** 1.226

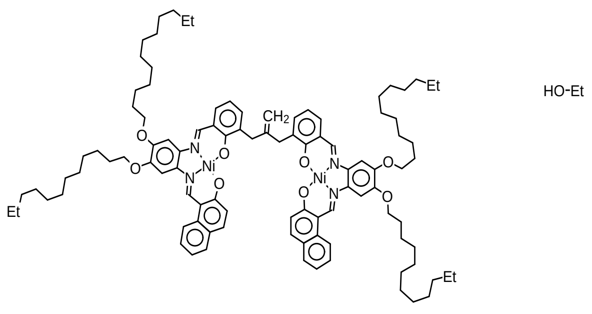

Search: search26 (Tue Dec 2 15:48:50 2014): Hits 765-768

POJWAO

Reference: A.Jana, P.Chakraborty, S.Mohanta (2014) *Polyhedron* ,**77**, 39

Formula: $C_{22}\,H_{18}\,Cu_1\,N_2\,O_4,C_2\,H_8\,N_1\,^{1+},Cl_1\,O_4\,^{1-}$

Compound Name: dimethylammonium (2,2'-(1,2-phenylenebis((nitrilo)methylylidene))bis(6-methoxyphenolato))-copper perchlorate

Space Group:	P-1	***Cell:***	***a*** 7.573(4)	***b*** 12.322(6)	***c*** 13.474(6)		
Space Group No.:	2	***(Å,°)***	α 78.51(0)	β 89.01(0)	γ 89.50(0)		
R-Factor (%):	4.95	***Temperature(K):***	296	***Density(g/cm³):***	1.573		

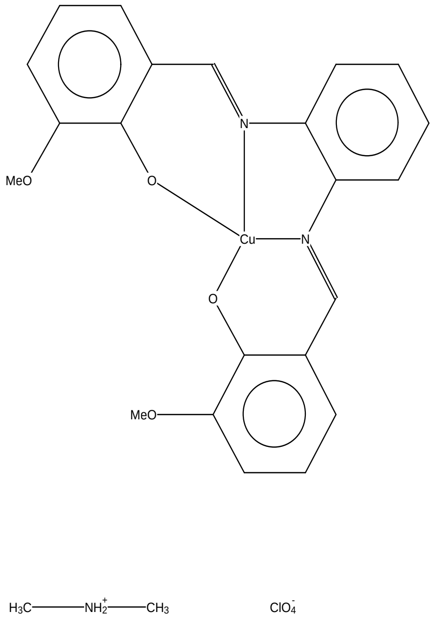

POJWOC

Reference: A.Jana, P.Chakraborty, S.Mohanta (2014) *Polyhedron* ,**77**, 39

Formula: $C_{22}\,H_{18}\,Cu_1\,N_2\,O_4,0.5(C_6\,H_{10}\,N_2\,^{2+}),Cl_1\,O_4\,^{1-},H_2\,O_1$

Compound Name: hemikis(benzene-1,2-diaminium) (2,2'-(1,2-phenylenebis(nitrilomethylylidene))bis(6-methoxyphenolato))-copper perchlorate monohydrate

Space Group:	P-1	***Cell:***	***a*** 13.630(0)	***b*** 14.339(0)	***c*** 15.540(1)		
Space Group No.:	2	***(Å,°)***	α 110.11(0)	β 95.04(0)	γ 113.27(0)		
R-Factor (%):	5.26	***Temperature(K):***	296	***Density(g/cm³):***	1.603		

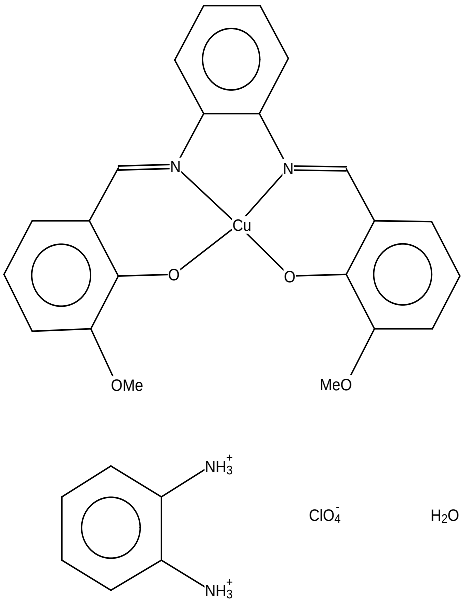

SOKBIF

Reference: Ting-Ting Li, Yong Chen, Fu-Min Li, Wei-Liang Zhao, Chuan-Jun Wang, Xiao-Jun Lv, Quan-Qing Xu, Wen-Fu Fu (2014) *Chem.-Eur.J.* ,

Formula: $C_{38}\,H_{28}\,N_4\,O_2\,Ru_1$

Compound Name: bis(isoquinoline)-(2,2'-(1,2-phenylenebis((nitrilo)methylylidene)) diphenolato)-ruthenium unknown solvate

Space Group:	P-1	***Cell:***	***a*** 14.093(1)	***b*** 15.911(1)	***c*** 16.576(2)		
Space Group No.:	2	***(Å,°)***	α 77.84(1)	β 76.41(1)	γ 77.80(1)		
R-Factor (%):	7.74	***Temperature(K):***	113	***Density(g/cm³):***	1.286		

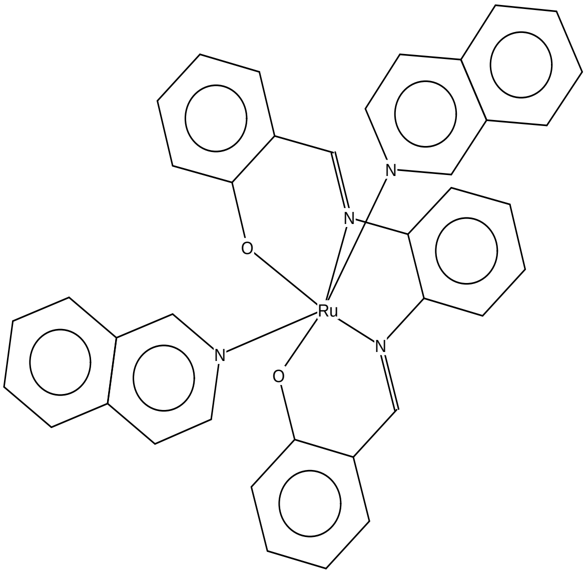

SOKFOP

Reference: Ting-Ting Li, Yong Chen, Fu-Min Li, Wei-Liang Zhao, Chuan-Jun Wang, Xiao-Jun Lv, Quan-Qing Xu, Wen-Fu Fu (2014) *Chem.-Eur.J.* ,

Formula: $C_{32}\,H_{26}\,F_2\,N_4\,O_2\,Ru_1$

Compound Name: bis(4-Methylpyridine)-(2,2'-(1,2-phenylenebis((nitrilo)methylylidene))bis(6-fluorophenolato))-ruthenium

Space Group:	Cc	***Cell:***	***a*** 13.034(3)	***b*** 15.878(3)	***c*** 13.536(3)		
Space Group No.:	9	***(Å,°)***	α 90.00	β 100.88(3)	γ 90.00		
R-Factor (%):	4.54	***Temperature(K):***	293	***Density(g/cm³):***	1.540		

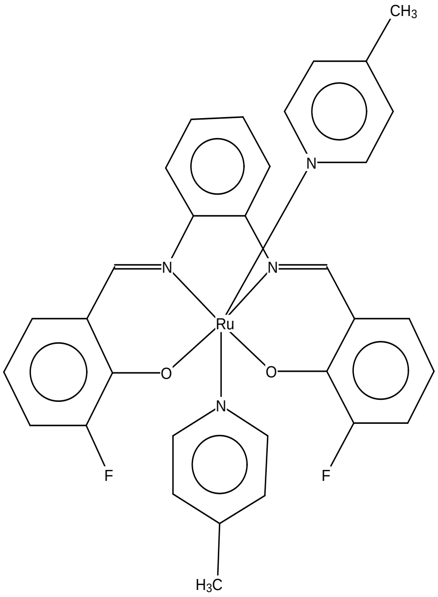

WISFOV

Reference: Zhen Zhang (2013) *Synth.React.Inorg.,Met.-Org.,Nano-Met.Chem.* ,**43,**1385

Formula: $C_{22} H_{20} Cu_1 N_2 O_5, C_{22} H_{18} Cu_1 N_2 O_4{}^{1+}, Cl_1 O_4{}^{1-}, 0.5(C_1 H_4 O_1)$

Compound Name: Aqua-(1,2-diaminobenzene-N,N'-bis(3-methoxysalicylideneiminato))-copper (1,2-diaminobenzene-N,N'-bis(3-methoxysalicylideneiminato))-copper perchlorate methanol solvate

Space Group:	Cmc21	***Cell:***	***a*** 17.981(8)	***b*** 17.776(8)	***c*** 14.047(8)
Space Group No.:	36	***(Å,°)***	α 90.00	β 90.00	γ 90.00
R-Factor (%):	4.84	***Temperature(K):***	298	***Density(g/cm^3):***	1.493

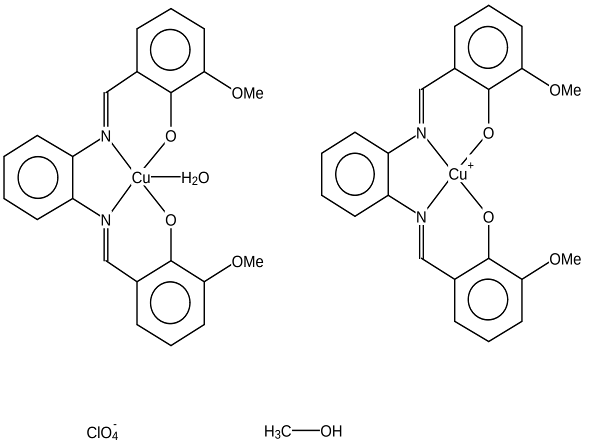

YIQGAI

Reference: A.Kochem, G.Gellon, N.Leconte, B.Baptiste, C.Philouze, O.Jarjayes, M.Orio, F.Thomas (2013) *Chem.-Eur.J.* ,**19,**16707

Formula: $C_{33} H_{41} N_3 Ni_1 O_2$

Compound Name: (2-(((2-((2-(amino)-3,5-di-t-butylbenzylidene)amino)phenyl)imino)methyl)-6-t-butyl-4-methoxyphenolato)-nickel(ii)

Space Group:	P-1	***Cell:***	***a*** 10.070(2)	***b*** 10.593(2)	***c*** 14.971(3)
Space Group No.:	2	***(Å,°)***	α 108.30(3)	β 92.35(3)	γ 102.84(3)
R-Factor (%):	4.92	***Temperature(K):***	200	***Density(g/cm^3):***	1.291

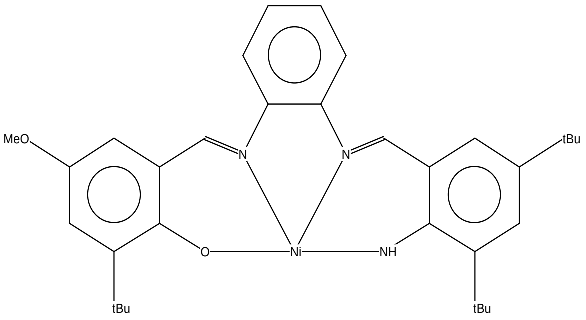

YIQGOW

Reference: A.Kochem, G.Gellon, N.Leconte, B.Baptiste, C.Philouze, O.Jarjayes, M.Orio, F.Thomas (2013) *Chem.-Eur.J.* ,**19,**16707

Formula: $C_{33} H_{41} N_3 Ni_1 O_2{}^{1+}, F_6 Sb_1{}^{1-}$

Compound Name: (2-((2-((2-amino-3,5-di-t-butylbenzylidene)amino)phenyl)carbonoimidoyl)-6-t-butyl-4-methoxyphenolato)-nickel(ii) hexafluoroantimonate

Space Group:	P21/c	***Cell:***	***a*** 16.946(3)	***b*** 18.575(4)	***c*** 10.963(2)
Space Group No.:	14	***(Å,°)***	α 90.00	β 102.19(3)	γ 90.00
R-Factor (%):	4.98	***Temperature(K):***	200	***Density(g/cm^3):***	1.587

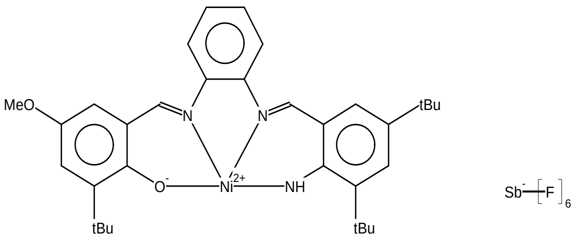

YIXFES

Reference: A.Biswas, S.Mondal, L.Mandal, A.Jana, P.Chakraborty, S.Mohanta (2014) *Inorg.Chim.Acta* ,**414,**199

Formula: $C_{50} H_{52} Ag_2 Cu_2 N_4 O_{10}{}^{2+}, 2(Cl_1 O_4{}^{1-})$

Compound Name: bis(μ_3-η^2-2,2'-(1,2-Phenylenebis(nitrilomethylylidene))bis(6-ethoxyphenolato))-bis(methanol)-di-copper-di-silver diperchlorate

Space Group:	P21/c	***Cell:***	***a*** 8.674(0)	***b*** 19.118(0)	***c*** 17.247(0)
Space Group No.:	14	***(Å,°)***	α 90.00	β 115.18(0)	γ 90.00
R-Factor (%):	4.39	***Temperature(K):***	296	***Density(g/cm^3):***	1.810

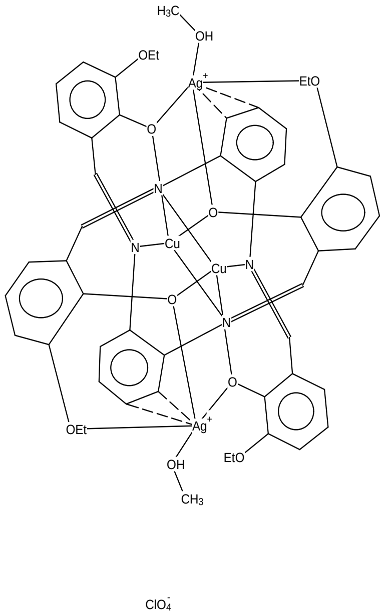

Supplement: Supplementary Information [file srep23499-s2.pdf]
